# Isothiourea catalysed enantioselective generation of point and axially chiral iminothia- and iminoselenazinanones†

**DOI:** 10.1039/d5sc02435h

**Published:** 2025-04-30

**Authors:** Alastair J. Nimmo, Alister S. Goodfellow, Jacob T. Guntley, Aidan P. McKay, David B. Cordes, Michael Bühl, Andrew D. Smith

**Affiliations:** a EaStCHEM, School of Chemistry, University of St Andrews Fife KY16 9ST UK buehl@st-andrews.ac.uk ads10@st-andrews.ac.uk

## Abstract

Symmetrical and unsymmetrical thioureas, as well as unsymmetrical selenoureas, are used in an isothiourea-catalysed Michael addition-lactamisation protocol using α,β-unsaturated pentafluorophenyl esters to generate iminothia- and iminoselenazinanone heterocycles with high enantioselectivity (up to 99 : 1 er). The scope and limitations of this process have been widely investigated (40 examples in total) with unsymmetrical thio- and selenoureas containing *ortho*-substituted *N*-aryl substituents giving atropisomeric products, leading to an effective process for iminothia- and iminoselenazinanones heterocyclic products containing both point and axially chiral stereogenic elements with excellent stereocontrol (up to >95 : 5 dr and 98 : 2 er). Mechanistic investigation showed that (i) the catalytically liberated aryloxide could deprotonate an electron-deficient thiourea; (ii) in the absence of an isothiourea catalyst, this leads to formation of racemic product; (iii) a crossover experiment indicates the reversibility of the thia-Michael addition. Computational analysis has identified the factors leading to enantioselectivity within this process, with stereocontrol arising from the lactamisation step within the catalytic cycle.

## Abstract 2

An isothiourea-catalysed addition of thio/selenoureas to α,β-unsaturated esters generates iminothia/iminoselenazinanones containing point and axially chiral stereogenic elements with excellent stereocontrol (up to >95 : 5 dr and 98 : 2 er).

## Graphical abstract

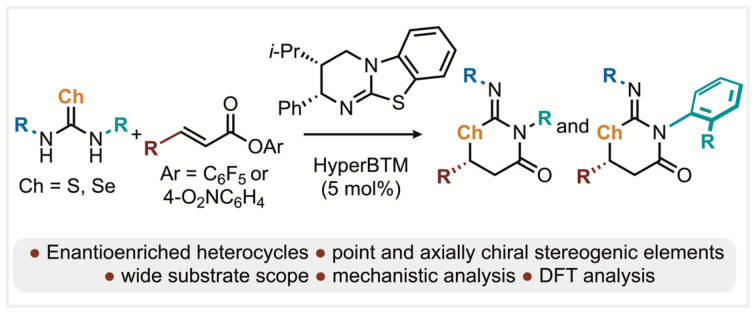

## 1. Introduction

**Scheme 1:**
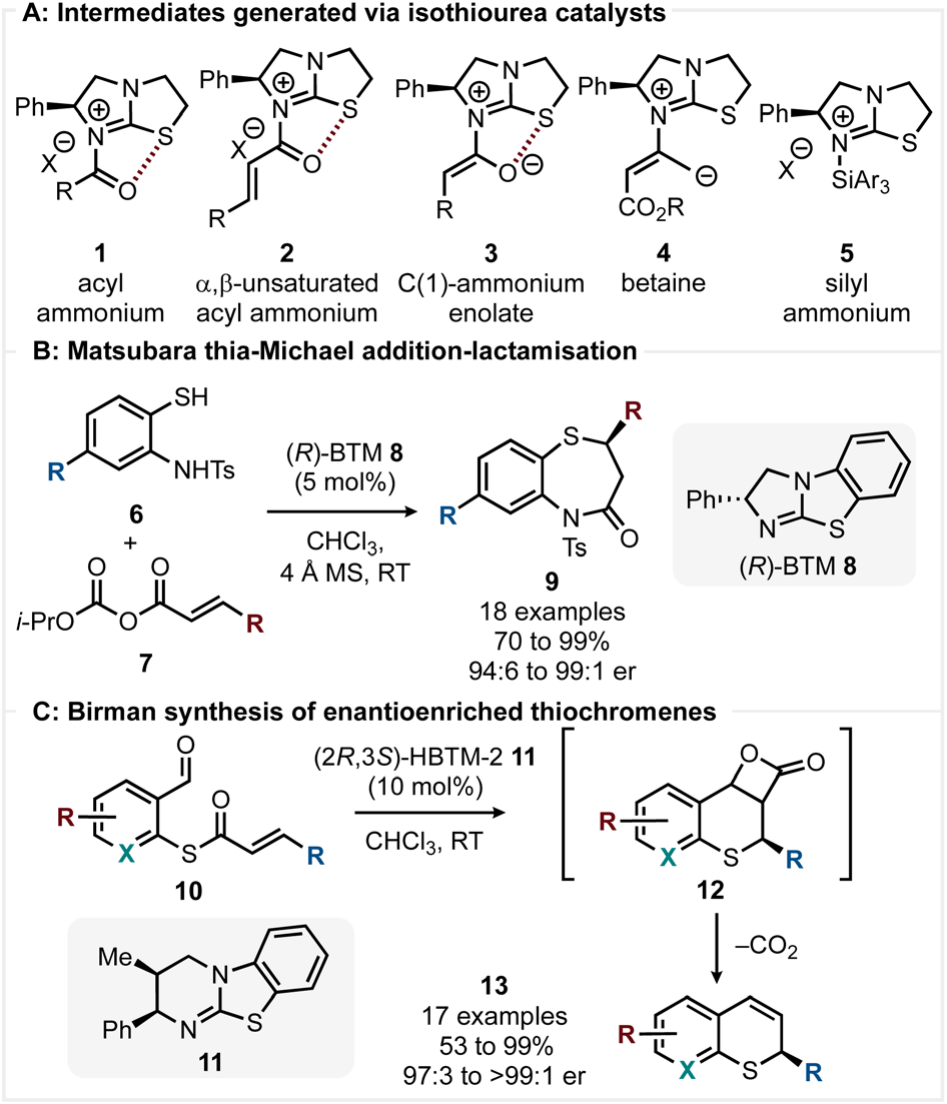
(A) Isothiourea derived catalytic intermediate. (B) & (C) Enantioselective C–S bond forming processes using α,β-unsaturated acyl ammonium species.

Since the introduction of isothioureas as catalysts for the acylative kinetic resolution of alcohols by Birman,^1^ these versatile Lewis bases have been widely developed and applied in enantioselective processes. Their simple and scalable synthesis, combined with their ability to generate multiple reactive intermediates 1–5 (acyl ammonium,^2^ α,β-unsaturated acyl ammonium,^3^ C(1)-ammonium enolate,^4^ betaine^5^ and silyl ammonium^6^ species) from simple starting materials has led to their widespread popularity (Scheme 1A). Isothiourea-catalysed Michael additions to *in situ* generated α,β-unsaturated acyl ammonium species have been used extensively to achieve enantioselective C–C bond formation.^7^ However, their use to selectively generate C-heteroatom bonds through conjugate addition of non-carbon centred nucleophiles is limited to relatively few C–S and C–N bond-forming processes. Within this area, Matsubara has reported a thia-Michael addition-lactamisation strategy for the synthesis of 1,5-benzothiazepines 9 using *N*-tosylated aminothiophenols 6 and α,β-unsaturated mixed anhydrides 7 in the presence of (*R*)-BTM 8 (Scheme 1B).^8^ The products were isolated in good to excellent yields (70 to 99%) and with excellent enantioselectivity (94 : 6 to 99 : 1 er). Mechanistic investigations revealed that the high enantioselectivity is a result of a dynamic kinetic process determined by the relative lactamisation rates of the diastereomeric thia-Michael addition products rather than the thia-Michael addition, which is readily reversible. This methodology was extended to 3-substituted and 2,3-disubstituted benzothiazepines with excellent stereoselectivity (79 : 21 to 94 : 6 er).^9^ Birman subsequently reported a route to enantioenriched thiochromenes 13 using thioesters 10 as α,β-unsaturated acyl ammonium precursors (Scheme 1C).^10^

Enantioselective C–S bond formation *via* thia-Michael addition is followed by an aldol-lactonisation cascade forming β-lactones 12, which readily decarboxylate, giving the corresponding thiochromene 13 in good to excellent yields (53 to 99%) and with excellent enantiocontrol (97 : 3 to >99 : 1 er). Alongside these advances, in previous work we developed the aza-Michael addition of 2-hydroxybenzophenone imines to α,β-unsaturated esters allowing access to β-amino amides in excellent yield (95%) and enantioselectivity (96 : 4 er).^11^

**Scheme 2:**
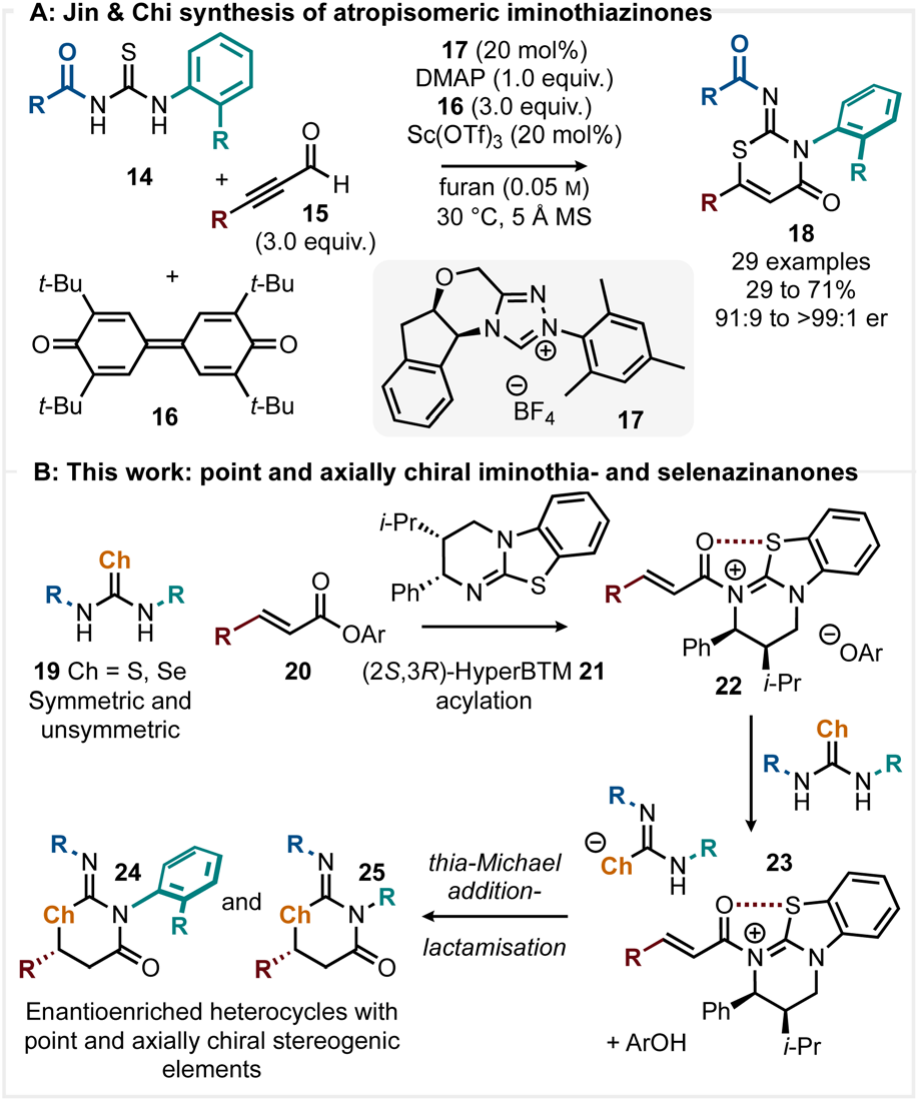
(A) NHC-catalysed synthesis of atropisomeric iminothiazinones. (B) This work: isothiourea catalysed preparation of point and axially chiral heterocycles.

In addition, in recent years there has been an explosion of interest concerning the development of selective synthetic methods for the preparation of atropisomeric species.^12^ While axially chiral biaryl (and heterobiaryl) species containing a C–C axis have been most widely studied, recent work has shown that the generation of axially chiral and configurationally stable C–N atropisomers is possible.^13^ Given these precedents, we considered alternative ways of developing enantioselective C–S and C–Se bond formation *via* an α,β-unsaturated acyl ammonium intermediate that could lead to heterocycles bearing both point and axially chiral stereogenic elements. In recent work Jin, Chi and coworkers used thioureas as dinucleophiles in an atropselective synthesis of iminothiazinones 18 using the NHC derived from precatalyst 17 (20 mol%, Scheme 2A).^14^ Thia-Michael addition-lactamisation of the thiourea 14 with an *in situ* generated acyl azolium intermediate gives iminothiazinones 18 in moderate to high yields (29 to 71%) with excellent enantioselectivity (91 : 9 to >99 : 1 er) with the formation of a C–N stereogenic axis. While effective, stoichiometric quantities (3 equiv.) of oxidant 16 was required to achieve oxidation of the Breslow intermediate in this protocol and furan was required as a solvent. This manuscript describes an alternative methodology to access related heterocyclic products that utilises α,β-unsaturated ester substrates and so avoids the need for *in situ* oxidation. Reaction *via* an *in situ* generated α,β-unsaturated acyl ammonium species derived from an α,β-unsaturated aryl ester and an isothiourea was postulated.^15^ In such processes, the aryloxide liberated upon isothiourea acylation can fulfil multiple roles, including that of Brønsted base.^10,15,16^ The hydrogen bond donor ability of electron-deficient thioureas, commonly exploited in catalytic applications, is concomitant with a p*K*_a_ that facilitates deprotonation by the catalytically liberated aryloxide,^17^ activating this towards thia-Michael addition with an α,β-unsaturated acyl ammonium intermediate 23 (Scheme 2B). This simple methodology would require no additional reagents and allow access to iminothiazinanone heterocycles 25 that possess a stereogenic centre. Furthermore, the incorporation of a 2-substituted aryl substituent on the thiourea would generate atropisomeric products 24 that also contain a stereogenic centre, while extension to selenoureas would give access to chiral iminoselenazinanones for the first time.

## 2. Results and discussion

### 2.1 Development of model process with symmetrical thioureas

Optimisation began with the reaction of α,β-unsaturated *para*-nitrophenyl (PNP) ester 27 with Schreiner's thiourea 26 and (2*S*,3*R*)-HyperBTM 21 (20 mol%) in MeCN (0.1 M) (Table 1, entry 1) giving product 33 in quantitative yield with 78 : 22 er. Variation of the reaction solvent (see ESI† for full range of solvents trialled) indicated that most common solvents gave >95% conversion to product. CH_2_Cl_2_ (entry 2) gave reduced enantioselectivity (66 : 34 er), while EtOAc, DMF, and THF (entries 3–5) all gave improved enantioselectivity. The use of 2-MeTHF and *i*-PrOAc also led to high product enantioselectivity (entries 6 and 7) with further optimisation using THF. Catalyst variation and loading were next tested. While HyperBTM 20 (5 mol%) gave 33 in quantitative yield and 91 : 9 er, the use of alternative isothioureas 8 and 34 led to only moderate selectivity (entries 8–10). Variation of the nucleofuge within the α,β-unsaturated ester or anhydride reaction component was next trialled. Among the range of aryl esters tested (entries 11–14) the observed product enantioselectivity correlated with the p*K*_a_ of the corresponding phenol.^18^ Pentafluorophenyl (PFP) ester 28 (entry 11) with the most acidic phenol (p*K*_a_ 5.53 in H_2_O) gave the greatest enantioselectivity (93 : 7 er) at 90% conversion, while 3,5-bis(trifluoromethyl)phenyl ester 31 (entry 14) with the least acidic phenol (p*K*_a_ 8.26 in H_2_O) gave the lowest observed product enantioselectivity (90 : 10 er). The use of *in situ* generated pivalic anhydride 32 did not lead to improved selectivity (entry 15, 83%, 90 : 10 er). The use of PFP ester 28 at 0 °C led to optimal reaction selectivity, giving product 33 in 95% isolated yield and 95 : 5 er (entry 16).

**Table 1:** Initial optimisation

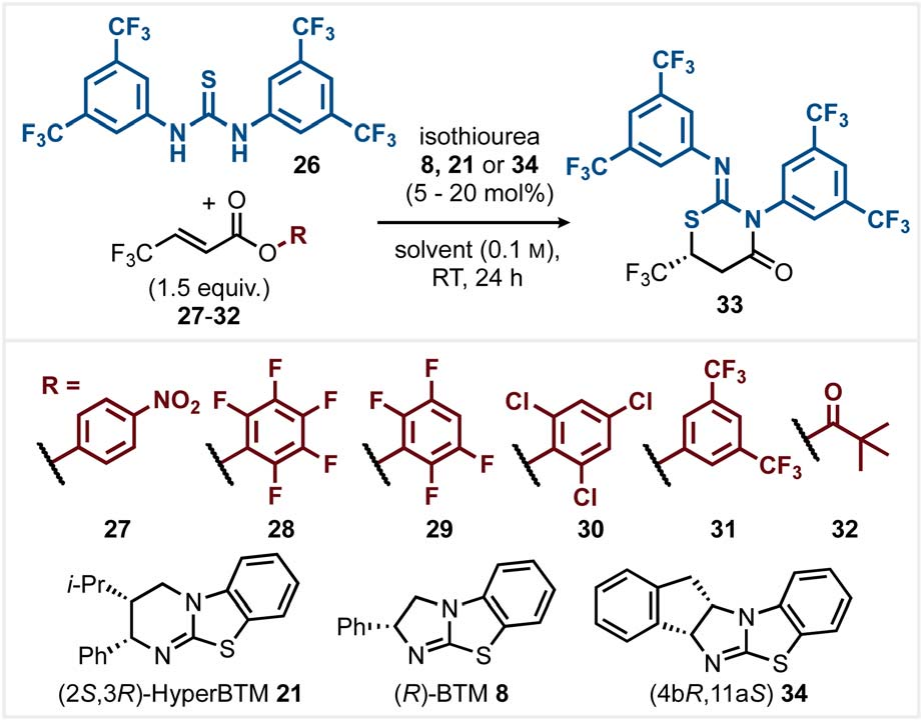					
Entry	Acyl donor	Catalyst (mol%)	Solvent	Yield^a^	er^b^
1	27	21 (20)	MeCN	Quant.	78 : 22
2	27	21 (20)	CH_2_Cl_2_	Quant.	66 : 34
3	27	21 (20)	EtOAc	Quant.	90 : 10
4	27	21 (20)	DMF	Quant.	84 : 16
5	27	21 (20)	THF	Quant.	91 : 9
6	27	21 (20)	2-MeTHF	Quant.	91 : 9
7	27	21 (20)	*i*-PrOAc	98	90 : 10
8	27	21 (5)	THF	Quant.	91 : 9
9	27	8 (5)	THF	96	84 : 16
10	27	34 (5)	THF	98	33 : 67
11	28	21 (5)	THF	90	93 : 7
12	29	21 (5)	THF	Quant.	92 : 8
13	30	21 (5)	THF	96	92 : 8
14	31	21 (5)	THF	95	90 : 10
15	32	21 (5)	THF	83	90 : 10
16^c^	28	21 (5)	THF	(95)	95 : 5

a Yield determined by ^1^H NMR analysis relative to internal standard 1,3,5-trimethoxybenzene (isolated yield in parentheses).

b er measured by HPLC analysis on a chiral stationary phase.

c Performed at 0 °C.

### 2.2 Scope of the developed process with symmetrical thioureas

**Scheme 3:**
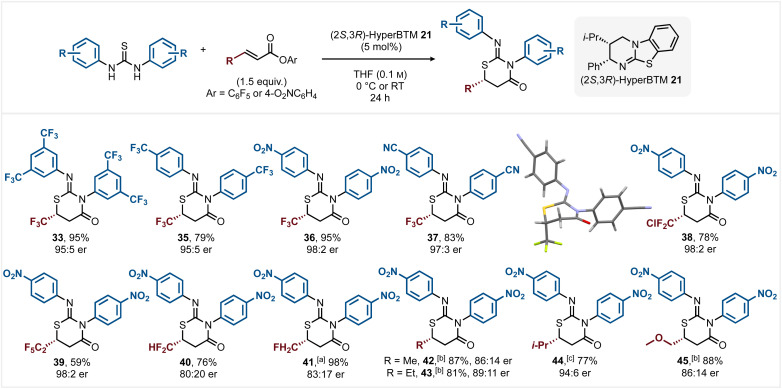
All yields are isolated; all er ratios determined by HPLC analysis on a chiral stationary phase; [a] 10 mol% 21 used at 0 °C; [b] 20 mol% 21 used at RT; [c] 30 mol% 21 used at RT.

With optimised conditions for the generation of model heterocycle 33 developed, the scope and limitations of this process were investigated through variation of the *N*-aryl substituents within the thiourea and the β-substituent within a range of α,β-unsaturated esters (Scheme 3). Using β-trifluoromethyl α,β-unsaturated PFP ester 28 as standard, the incorporation of 4-F_3_CC_6_H_4_ substituents within the bis-*N*-arylthiourea led to reduced product conversion at 0 °C, but at RT gave 35 in 79% yield with excellent enantioselectivity (95 : 5 er). The incorporation of strongly electron-withdrawing 4-O_2_NC_6_H_4_ and 4-NCC_6_H_4_*N*-aryl substituents within the thiourea showed similar reactivity to that of the model substrate, giving products 36 and 37 in excellent yields (95% and 83%) and enantioselectivity (98 : 2 er and 97 : 3 er respectively). The absolute configuration within (6*R*,*Z*)-37 was confirmed by single crystal X-ray analysis, with the configuration within all other products assigned by analogy.^19^ Further work probed the scope of the α,β-unsaturated ester Michael acceptors using bis-4-O_2_NC_6_H_4_ substituted thiourea. The incorporation of perhalogenated β-CF_2_Cl- and perfluoroethyl β-substituents gave products 38 and 39 in good yield (78% and 59%) with excellent enantioselectivity (both 98 : 2 er).^20^ While β-CF_2_H and β-CH_2_F substituents within the ester were tolerated giving products 40 and 41 respectively, reduced enantioselectivity was observed in both cases (80 : 20 er and 83 : 17 er respectively). To further test this methodology the use of β-alkyl substituted esters was probed as these generally show moderate reactivity in reactions involving the corresponding α,β-unsaturated acyl ammonium species. In this context, 42–45 were generated in excellent yields and promising enantioselectivity (86 : 14 er to 94 : 6 er) although these reactions were carried out at room temperature and required higher catalyst loadings (20–30 mol%). Unfortunately the use of β-aryl substituted PFP esters (β-aryl = Ph, 4-NO_2_C_6_H_4_) were unreactive in this process using bis-4-O_2_NC_6_H_4_ substituted thiourea and represent a limitation of this methodology.

### 2.3 Application to unsymmetrical thio- and selenoureas

**Scheme 4:**
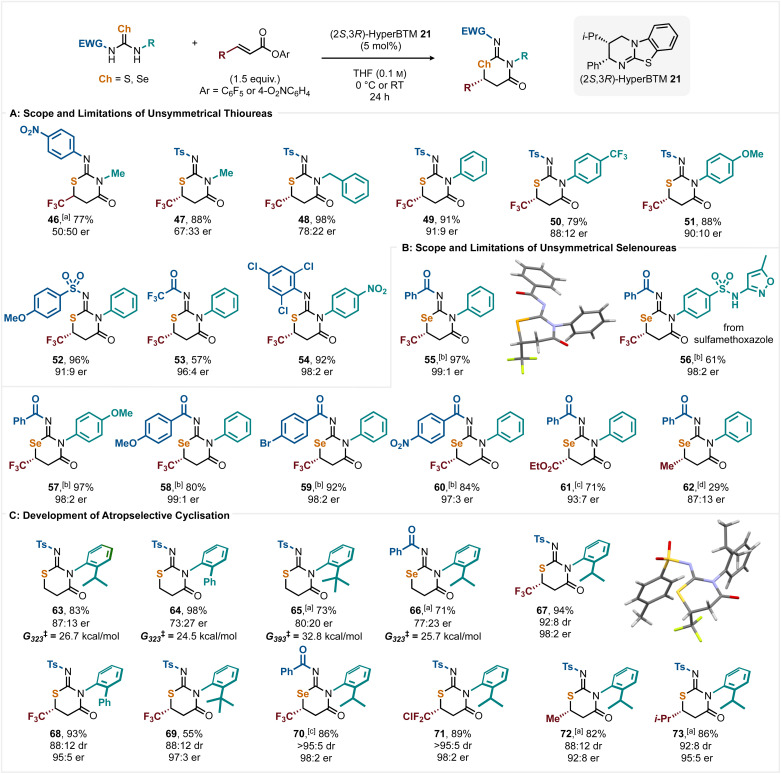
All yields are isolated; all er ratios determined by HPLC analysis on a chiral stationary phase; [a] 20 mol% 21 used at RT; [b] 10 mol% 21 used at 0 °C; [c] 10 mol% 21 used at RT; [d] 30 mol% 21 used at RT.

Further work extended this process to the use of unsymmetrical thio- and selenoureas (Scheme 4). In principle these starting materials could lead to the formation of regioisomeric products, and so to bias regioselectivity variation in the electronic properties of the nitrogen substituents was used to dictate reactivity. Initial studies considered the utility of a range of thioureas bearing electronically distinct *N*-substituents with PFP ester 28 (Scheme 4A). In each case a single regioisomeric product was observed but with varying levels of enantioselectivity. Reaction to generate 46 required 20 mol% catalyst and 48 h at RT to reach completion, giving 46 in 77% yield but in racemic form. Replacing the *N*-4-nitrophenyl substituent with an *N*-tosyl substituent led to improved reactivity, giving 47 in high yield (88%) using 5 mol% catalyst at 0 °C but with moderate enantioselectivity (67 : 33 er). Using an *N*-benzyl substituted derivative gave 48 in 98% yield with improved enantioselectivity (78 : 22 er), while *N*-aryl variants gave 49–51 in high yields (79 to 91%) and good enantioselectivity (88 : 12 to 91 : 9 er). Variation within the electron-withdrawing substituents was also tolerated, with *N*-4-methoxybenzenesulfonyl and *N*-trifluoroacetyl variants giving 52 and 53 with high enantioselectivity (91 : 9 er and 96 : 4 er). As a further test, it was postulated that regioselectivity could be achieved through a steric bias within two electron-withdrawing *N*-aryl substituents. Using the sterically demanding *N*-2,4,6-trichlorophenyl substituent to disfavour cyclisation led to a single regioisomer of 54 in excellent yield (92%) with excellent enantioselectivity (98 : 2 er). The constitution of 46 and 54 were confirmed by ^1^H–^15^N HMBC (see ESI† for further information).

Subsequent work extended this process to the use of unsymmetrical selenoureas to give iminoselenazinanones (Scheme 4B). Pleasingly, *N*-Bz,*N*-Ph-substituted selenourea led to 55 in excellent yield and enantioselectivity (97%, 99 : 1 er). In this series, alternative *N*-aryl substitution was investigated, with the incorporation of the antibiotic sulfamethoxazole within a selenourea giving 56 in good yield (61%) with excellent 98 : 2 er. 4-MeOC_6_H_4_ substitution gave 57 with excellent yield (97%) and enantioselectivity (98 : 2 er). Variation within the *N*-benzoyl substitution was also well tolerated, with 4-MeOC_6_H_4_, 4-BrC_6_H_4_, and 4-O_2_NC_6_H_4_ substituents giving products 58, 59, and 60 respectively in high yields (80 to 92%) and excellent enantioselectivity (97 : 3 to 99 : 1 er). To demonstrate that the selenoureas were reactive with alternative Michael acceptors, two representative examples were chosen. Reaction with ethyl ester substituted Michael acceptor at RT gave product 61 in 71% yield with high enantioselectivity (93 : 7 er). The reactivity of selenoureas was pushed to a limit with the β-methyl substituted Michael acceptor with the use of 30 mol% 21 at RT for 48 h giving 62 in low 29% yield (87 : 13 er) and so represents a limit of this methodology. Further work investigated the use of thioureas and a selenourea bearing *ortho*-substituted aryl substituents to give atropisomeric products (Scheme 4C). Initial studies showed that chalcogenoureas bearing an *ortho*-substituted aryl substituent give atropisomeric products 63–66 upon treatment with pentafluorophenyl acrylate and HyperBTM. Although the isolated yields of products 63–66 were good to excellent (71 to 98%), the enantioselectivities were only moderate (73 : 27 er to 87 : 13 er). The configurational stability of 63–66 was determined as described by Armstrong,^21^ with increasing barriers to rotation observed with increased steric hindrance of the 2-aryl substituent (Ph < *i*-Pr < *t*-Bu), and with Se-containing 66 possessing a lower barrier than its sulfur analogue. Intrigued by these observations, application to products containing both a stereogenic centre and a stereogenic axis were investigated. Using the previously developed conditions, treatment of a thiourea bearing an *ortho-iso*-propyl substituent with PFP ester 28 and HyperBTM gave 67 in excellent yield (94%) and stereoselectivity (92 : 8 dr, 98 : 2 er). Similarly, thioureas bearing *ortho*-phenyl and *ortho-tert*-butyl substituents gave the corresponding products 68 and 69 with excellent stereoselectivity. Using a selenourea bearing an *ortho-iso*-propyl substituent gave 70 in 86% yield and excellent stereoselectivity (>95 : 5 dr, 98 : 2 er). The generality of the enantio- and atropselective methodology was further investigated by reaction of an *ortho-iso*-propyl substituted thiourea with three alternative β-substituted α,β-unsaturated esters. CF_2_Cl substitution gave 71 in excellent yield (89%) and stereoselectivity (>95 : 5 dr, 98 : 2 er), while pleasingly β-alkyl substituted Michael acceptors were also tolerated, giving 72 and 73 with high yields (82% and 86%) and stereoselectivity (88 : 12 dr, 92 : 8 er and 92 : 8 dr, 95 : 5 er). The absolute configuration of (6*R*,*R*_a_,*Z*)-67 was confirmed by single crystal X-ray crystallography, with the relative configuration within 72 confirmed by ^1^H NOESY NMR analysis (see ESI Section 13† for further information). The configuration within all other products was assigned by analogy.

### 2.4 Mechanistic analysis and control studies

#### 2.4.1 Role of aryloxide

**Scheme 5:**
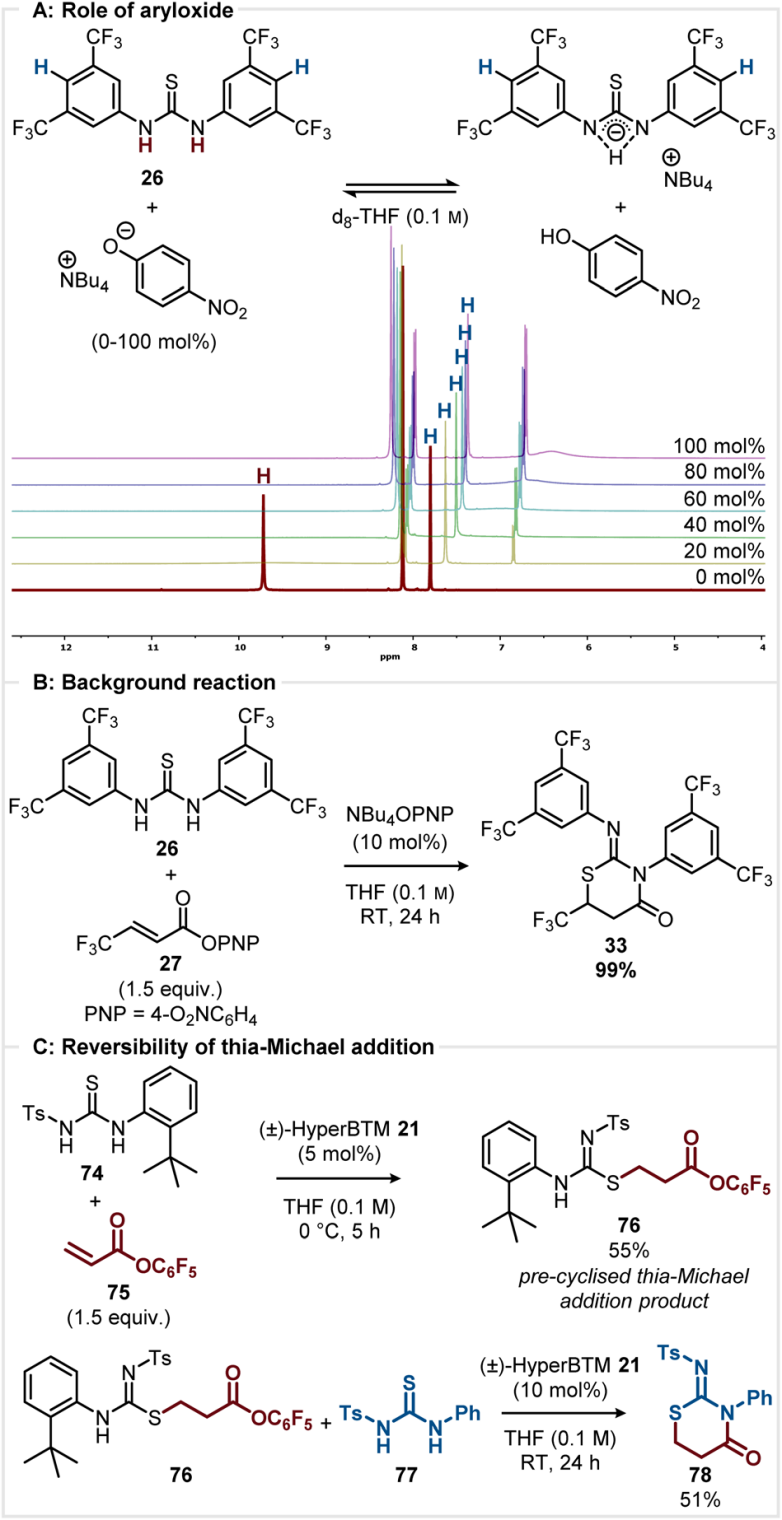
(A) Determining role of aryloxide by ^1^H NMR titration. (B) Aryloxide catalysed racemic background reaction. (C) Probing reversibility of the thia-Michael addition by crossover experiment.

Subsequent studies considered the role of aryloxide liberated upon catalyst acylation and its ability to promote thia-Michael addition through thiourea activation by deprotonation. Following the method described by Yatsimirsky, the feasibility of this deprotonation process was interrogated by NMR titration.^22^ Schreiner's thiourea 26 was titrated with NBu_4_OPNP in d_8_-THF (0.1 M) (Scheme 5A). Upon addition of only 20 mol% of NBu_4_OPNP the NH proton resonance (H, *δ*_H_ = 9.7 ppm) disappeared which is indicative of deprotonation.^22^ This is consistent with the reported p*K*_a_ of 26 (p*K*_a_ 8.5 in DMSO) and 4-nitrophenol (p*K*_a_ 10.8 in DMSO).^17^ As the concentration of NBu_4_OPNP was incrementally increased (to 100 mol%), the C(4)-*ortho* proton signal (H) experienced a significant upfield shift (from *δ*_H_ = 7.8 ppm to *δ*_H_ = 7.4 ppm) consistent with the equilibrium shifting towards the deprotonated thiourea. This titration suggests that under the reaction conditions the thiourea is likely to be deprotonated by a catalytically liberated aryloxide. Further work considered if this deprotonation could promote a racemic background reaction to generate 33. In the absence of any isothiourea catalyst or base, no conversion to 33 was observed, but on addition of 10 mol% NBu_4_OPNP the product 33 was formed in 99% yield by ^1^H NMR analysis (Scheme 5B). While this experiment does not give quantitative data regarding the rate of the background reaction compared to the isothiourea catalysed reaction, it highlights the benefit of the aryloxide base being generated catalytically and in proximity to the proposed α,β-unsaturated acyl ammonium intermediate. Further titration studies (see ESI Section 15† for further information) indicate that the thiourea does not hydrogen bond and activate the ester carbonyl group.

#### 2.4.2 Reversibility of thia-Michael addition

The potential reversibility of the proposed S- or Se-conjugate addition was next probed. The reaction of thiourea 74 and pentafluorophenyl acrylate 75 was stopped after 5 h, giving pre-cyclised thia-Michael addition product 76 in 55% yield. In this case, the *tert*-butyl substituent presumably hinders cyclisation, allowing aryloxide catalyst turnover (Scheme 5C). To investigate the potential reversibility of the thia-Michael addition, 76 was reacted with (±)-HyperBTM 21 and thiourea 77, giving crossover product 78 in 51% isolated yield. The formation of 78 indicates that thia-Michael addition is reversible under the reaction conditions, similar to the mechanism proposed by Matsubara (Scheme 1B).^9^

### 2.5 Computational analysis and proposed mechanism

**Scheme 6:**
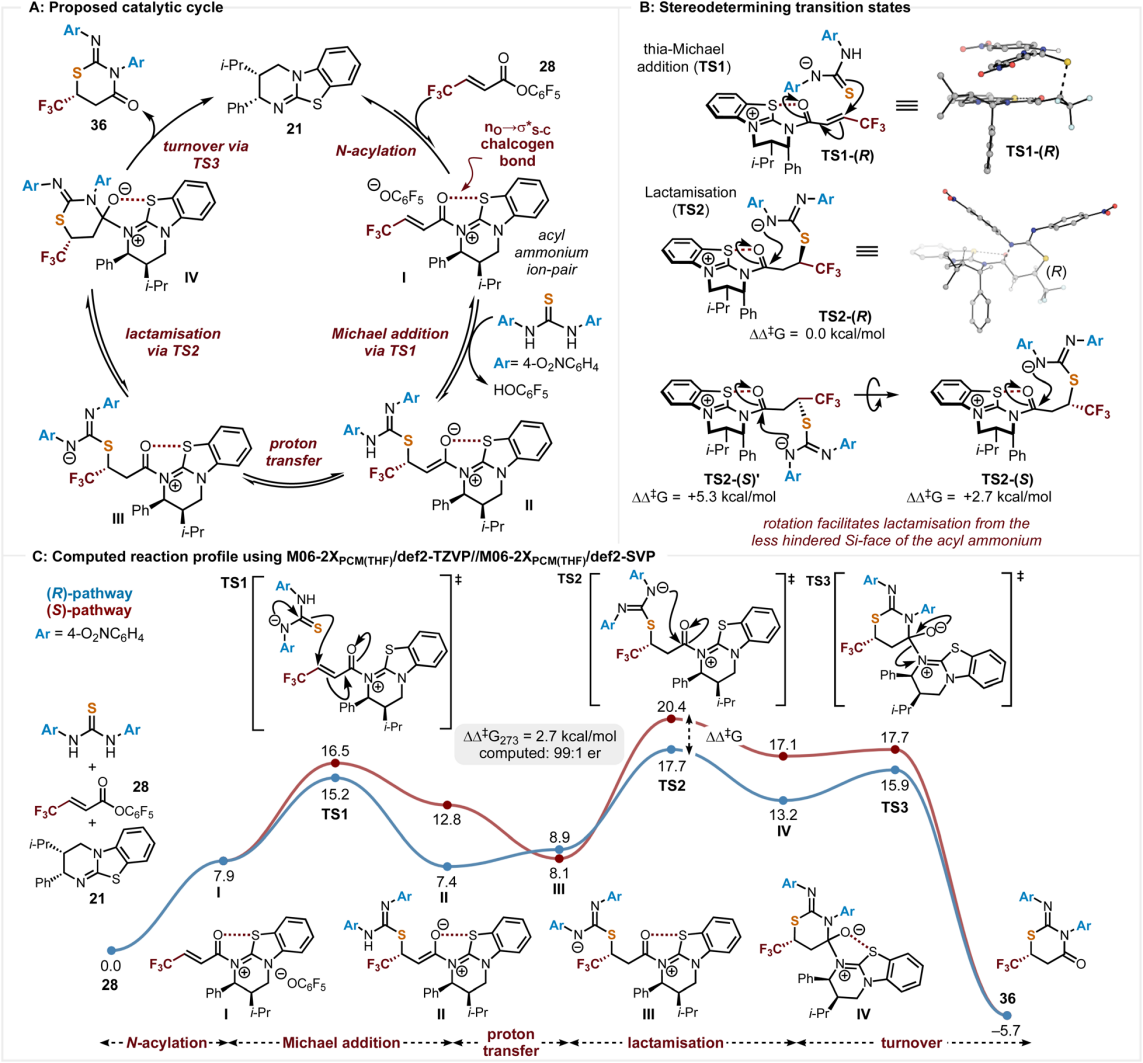
(A) Proposed catalytic cycle. (B) DFT analysis of the stereodetermining transition state. (C) Computed reaction profile leading to enantiomeric products. M06-2X_SMD(THF)_/def2-TZVP//M06-2X_SMD(THF)_/def2-SVP Gibbs free energies (Δ*G*_273_) shown in kcal mol^−1^.

Based upon these observations a catalytic cycle for this process can be outlined, with the origins of stereocontrol probed by DFT calculations performed at the M06- 2X_SMD(THF)_/def2-TZVP//M06-2X_SMD(THF)_/def2-SVP level of theory using Gaussian16 (Scheme 6). Using the symmetric bis-4-O_2_NC_6_H_4_ substituted thiourea and PFP ester 28 as a model, *N*-acylation of the PFP ester 28 by the Lewis base (2*R*,3*S*)-HyperBTM 21 generates the corresponding acyl ammonium ion pair I with a stabilising 1,5-O⋯S chalcogen bonding interaction (*n*_O_ → σ*_S–C,_*E*^(2)^ = 6.0 kcal mol^−1^, described by NBO second-order perturbation analysis).^23–32^ This ensures coplanarity between the 1,5-O- and S-atoms and provides a conformational bias.^26^ Deprotonation of the thiourea promotes thia-Michael addition *via*TS1 to give II. Subsequent proton transfer gives III, with intramolecular cyclisation *via*TS2 generating IV. Catalyst release is promoted by collapse of the tetrahedral intermediate IV*via*TS3 generating the heterocyclic product 36 in high enantioselectivity (Scheme 6A).

Stereoselectivity in the initial Michael addition considered the anionic thiourea nucleophile approaching either the *Re*- or *Si*-face of acyl ammonium intermediate I (Scheme 6B and C). Approach to the *Si*-face is hindered by the stereodirecting phenyl group within (2*S*,3*R*)-HyperBTM leading to favoured addition to the Re-face (TS1, ΔΔG_273_^‡^ = 1.3 kcal mol; 92 : 8 er (*R*)). After proton transfer, the pendant nitrogen nucleophile can initiate lactamisation (TS2) to generate the tetrahedral cyclised product adduct, before catalyst turnover (TS3) to generate the product. These diastereomeric lactamisation transition states (TS2) were computed to be higher in energy than the thia-Michael addition transition states, TS1 (2.5 kcal mol^−1^ higher for the (*R*)-pathway), due to the geometric constraints of ring-closure. While the stereogenic centre is formed in the Michael addition (TS1), computation suggested that the observed enantioselectivity arises from the energetic difference between the higher energy lactamisation transition states (TS2). Turnover of the catalyst (TS3) is strongly exergonic and proceeds through a lower energy transition state compared to TS2. Every step of the reaction (Scheme 6C) was computed to be reversible up until catalyst turnover (TS3), in agreement with the experimentally observed crossover and retro-Michael addition in Scheme 5C. Lactamisation (TS2) was computed to be enantiodetermining with the favoured (*R*)-pathway proceeding through nucleophilic addition from the less hindered Si-face of the acyl ammonium. The disfavoured (*S*)-pathway is initially positioned to lactamise from the hindered Re-face of the acyl ammonium (TS2-(S)′, ΔΔG_273_^‡^ = +5.3 kcal mol^−1^). However, rotation allows lactamisation from the less hindered Si-face (TS2-(*S*), ΔΔG_273_^‡^ = +2.7 kcal mol^−1^).

## 3. Conclusion

In conclusion, a range of symmetrical and unsymmetrical thioureas and selenoureas can be utilised in an isothiourea-catalysed protocol to generate iminothia- and iminoselenazinanone heterocycles with high enantioselectivity (up to 99 : 1 er). Notably, unsymmetrical thio- and selenoureas containing *ortho*-substituted *N*-aryl substituents generate atropisomeric products. This allows the generation of iminothia- and iminoselenazinanone heterocyclic products containing both point and axially chiral stereogenic elements with excellent stereocontrol (up to >95 : 5 dr and 98 : 2 er) for the first time. Mechanistic investigations indicate that catalytically liberated aryloxide can deprotonate an electron-deficient thiourea, while a crossover experiment indicates the reversibility of the thia-Michael addition. Extensive computational analysis has identified the factors leading to enantioselectivity within this process, with stereocontrol shown to arise from the lactamisation step within the catalytic cycle.

## Author contributions

Alastair J. Nimmo – conceptualization, investigation, writing. Alister S. Goodfellow – formal analysis, investigation writing-review and editing. Jacob T. Guntley – investigation. Aidan P. McKay, David. B. Cordes, investigation (X-ray analysis). Michael Bühl – investigation, funding acquisition, writing-review and editing. Andrew D. Smith – conceptualization, funding acquisition, project administration writing-review and editing.

## Conflicts of interest

The authors declare no conflict of interest.

## Supplementary Material

SC-016-D5SC02435H-s001

SC-016-D5SC02435H-s002

## Data Availability

All data (experimental procedures, characterisation data and cartesian coordinates for all DFT calculations) that support the findings of this study are available within the article and its ESI.† The research data supporting this publication can be accessed from “Isothiourea Catalysed Enantioselective Generation of Point and Axially Chiral Iminothia- and Iminoselenazinanones”. Pure ID: 314763684. University of St Andrews Research Portal “PURE” [https://doi.org/10.17630/3ffd33a9-50ae-441d-bd88-6a3343351c63].
