# Supplementary material for: Isothiourea catalysed enantioselective generation of point and axially chiral iminothia- and iminoselenazinanones
Source: Chem Sci. 2025 Apr 30;16(23):10494–502. doi: 10.1039/d5sc02435h (PMC12068085; doi:10.1039/d5sc02435h)
Supplement: SC-016-D5SC02435H-s001 [file SC-016-D5SC02435H-s001.pdf]

Supplementary Information (SI) for Chemical Science.
This journal is © The Royal Society of Chemistry 2025

Isothiourea Catalysed Enantioselective Generation of Point and Axially Chiral Iminothia- and Iminoselenazinanones

Alastair J. Nimmo, Alister S. Goodfellow, Jacob T. Guntley, Aidan P. McKay, David B. Cordes, Michael Bühl and Andrew D. Smith*

Supporting Information

1. General Information 2

2. General Procedures 5

3. Reaction Optimisation 9

4. Preparation of α,β-Unsaturated Esters 12

5. Preparation of Thioureas 21

6. Preparation of Selenoureas 30

7. Preparation of Ureas 35

8. Isothiourea Catalysis Products 36

9. Unsuccessful Substrates 73

10. Structure Elucidation of Minor Regioisomer 73

11. Conformation of Structure by ^{1}H–^{15}N HMBC 74

12. Calculation of Configurational Stability 76

13. Configuration of Major Diastereoisomer Determined by ^{1}H NOESY 82

14. Determining the Configuration of Minor Diastereoisomers 83

15. Mechanistic Studies 87

16. Computational Studies 91

17. Single Crystal X-ray Diffraction Data 97

18. References 101

Appendix I: ^{1}H, ^{19}F{^{1}H}, ^{13}C{^{1}H}, and ^{77}Se{^{1}H} NMR Spectra 103

Appendix II: HPLC Traces of Novel Compounds 321

Appendix III: Computational Data 363

1. General Information

All reagents and solvents were obtained from commercial suppliers and were used without further purification unless otherwise stated. Reactions involving moisture sensitive reagents were carried out in flame-dried glassware under an inert atmosphere (N_2 or Ar) using standard vacuum line techniques. Anhydrous solvents (Et_2O, CH_2Cl_2, THF, DMF and toluene) were obtained after passing through an alumina column (Mbraun SPS-800).

Reactions were carried out in flame-dried glassware or oven-dried vials under an inert atmosphere (N_2) using standard vacuum line techniques. Room temperature (RT) refers to 20–25 °C. Temperatures of 0 °C were obtained using an ice/saltwater bath in an insulated container. Reactions involving heating were performed using either DrySyn blocks or an oil bath and a contact thermocouple. Under reduced pressure refers to the use of either a Büchi Rotavapor R-200 with a Büchi V-491 heating bath and Büchi V-800 vacuum controller, a Büchi Rotavapor R-210 with a Büchi V-491 heating bath and Büchi V-850 vacuum controller, a Heidolph Laborota 4001 with vacuum controller, an IKA RV10 rotary evaporator with a IKA HB10 heating bath and ILMVAC vacuum controller, or an IKA RV10 rotary evaporator with a IKA HB10 heating bath and Vacuubrand CVC3000 vacuum controller. Rotary evaporator condensers are fitted to Julabo FL601 Recirculating Coolers filled with ethylene glycol and set to –5 °C.

Analytical thin layer chromatography was performed on pre-coated aluminium plates (Kieselgel 60 F254 silica) and visualisation was achieved using ultraviolet light (254 nm) and/or staining with aqueous $KMnO_4$ solution followed by heating. Manual column chromatography was performed in glass columns fitted with porosity 3 sintered discs over Kieselgel 60 silica using the solvent system stated. Automated chromatography was performed on a Biotage Selekt with a UV/Vis detector using the solvent system stated and cartridges filled with Kieselgel 60 silica.

Melting points were recorded on an Electrothermal 9100 melting point apparatus, (dec) refers to decomposition.

Optical rotations were measured on a Perkin Elmer Precisely/Model-341 polarimeter operating at the sodium D line with a 100 mm path cell at 20 °C. Concentrations (*c*) are stated in g/100 mL.

HPLC analyses were obtained on either a Shimadzu HPLC consisting of a DGU-20A5 degassing unit, LC-20AT liquid chromatography pump, SIL-20AHT autosampler, CMB-20A communications bus module, SPD-M20A diode array detector and a CTO-20A column oven or a Shimadzu HPLC consisting of a DGU-20A5R degassing unit, LC-20AD liquid chromatography pump, SIL-20AHT autosampler, SPD-20A UV/Vis detector and a CTO-20A column oven. Separation was achieved using either DAICEL CHIRALCEL OD-H and OJ-H columns or DAICEL CHIRALPAK AD-H, AS-H, IA, IB, IC and ID columns using the method stated. HPLC traces of enantiomerically enriched compounds were compared with authentic racemic spectra.

Infrared (IR) spectra were recorded on a Shimadzu IRAffinity-1 Fourier transform IR spectrophotometer fitted with a Specac Quest ATR accessory (diamond puck). Spectra were recorded of either thin films or solids, with characteristic absorption wavenumbers (ν_{max}) reported in cm^{-1}.

^{1}H, ^{13}C{^{1}H}, ^{19}F{^{1}H}, and ^{77}Se{^{1}H} NMR spectra were acquired on either a Bruker AV400 with a BBFO probe (^{1}H 400 MHz; ^{19}F{^{1}H} 377 MHz), a Bruker AVII 400 with a BBFO probe (^{1}H 400 MHz; ^{19}F{^{1}H} 376 MHz), a Bruker AVIII-HD 500 with a SmartProbe BBFO+ probe (^{1}H 500 MHz, ^{19}F{^{1}H} 470 MHz), a Bruker AVIII 500 with a CryoProbe Prodigy BBO probe (^{1}H 500 MHz, ^{13}C{^{1}H} 126 MHz, ^{77}Se{^{1}H} 95 MHz), or a Bruker AVIII-HD 700 with a CryoProbe Prodigy TCI probe (^{13}C{^{1}H} 176 MHz) in the deuterated solvent stated. All chemical shifts are quoted in parts per million (ppm) relative to the residual solvent peak. All coupling constants, J, are quoted in Hz. Multiplicities are indicated as s (singlet), d (doublet), t (triplet), q (quartet), m (multiplet), and multiples thereof. The abbreviation Ar denotes aromatic. NMR peak assignments were confirmed using 2D ^{1}H correlated spectroscopy (COSY), 2D ^{1}H–^{13}C or ^{1}H–^{15}N heteronuclear multiple-bond correlation spectroscopy (HMBC), and 2D ^{1}H–^{13}C heteronuclear single quantum coherence (HSQC) where necessary.

Mass spectrometry (HRMS) data were acquired by either electrospray ionisation (ESI), electron impact (EI), or matrix-assisted laser desorption/ionisation with no matrix (MALDI (no matrix)) at either the University of St Andrews Mass Spectrometry Facility or SIRCAMS at University of Edinburgh.

X-ray diffraction data were collected and processed by the University of St Andrews Single Crystal X-ray Diffraction Service.

2. General Procedures

General Procedure A: Preparation of isothiocyanates

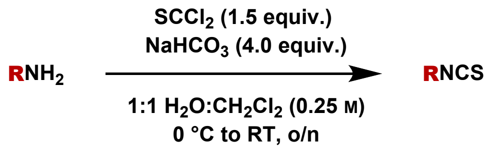

The appropriate amine (1.0 equiv.) and $NaHCO_3$ (4.0 equiv.) were dissolved in 1:1 CH_2Cl_2:H_2O (0.25 M) and the reaction mixture cooled to 0 °C. Thiophosgene (1.5 equiv.) was added dropwise then the reaction mixture warmed to RT to stir o/n. Once complete, the reaction mixture was diluted with H_2O and extracted with CH_2Cl_2 (× 2). The combined organics were dried over $MgSO_4$ then the solvent removed under reduced pressure to give the crude product which was used without further purification.

General Procedure B: Preparation of thioureas

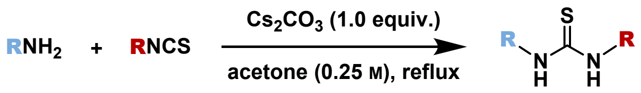

The appropriate amine (1.0 equiv.), isothiocyanate (1.0 equiv.), and Cs_2CO_3 (1.0 equiv.) were dissolved in acetone (0.25 M) and heated to reflux. Once complete, the mixture was cooled, diluted with H_2O, and acidified to pH 1 with 2 M HCl. The aqueous mixture was extracted with EtOAc (× 3), dried over $MgSO_4$ and the solvent removed under reduced pressure to give the crude product which was purified as specified.

General Procedure C: Preparation of selenoureas

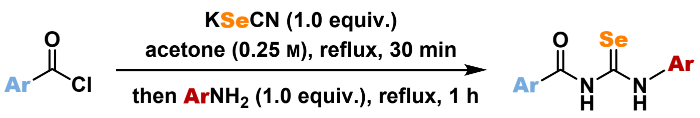

The appropriate acid chloride (1.0 equiv.) was added to a solution of KSeCN (1.0 equiv.) in acetone (0.25 M) and heated to reflux for 30 min. The requisite aniline (1.0 equiv.) was then added, and the reaction mixture heated at reflux for a further 1 h. Once complete, the reaction mixture was poured into ice water with stirring. The resulting precipitate was collected by vacuum filtration to give the crude product which was purified as specified.

General Procedure D: Preparation of α,β-unsaturated acids

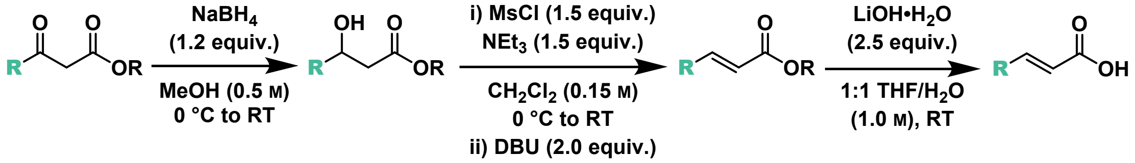

The appropriate β-ketoester (1.0 equiv.) was dissolved in MeOH (0.5 M) and cooled to 0 °C. $NaBH_4$ (1.2 equiv.) was added, and the reaction mixture warmed to RT. Once complete, the reaction mixture was quenched with 1 M HCl and extracted with EtOAc (× 2). The combined organics were then washed with sat. $NaHCO_3$ then brine, dried over $MgSO_4$ and the solvent removed under reduced pressure to afford the crude alcohol.

The crude alcohol (1.0 equiv.) and NEt_3 (1.5 equiv.) were dissolved in anhydrous CH_2Cl_2 (0.3 M), and the reaction mixture cooled to 0 °C. A solution of MsCl (1.5 equiv.) in CH_2Cl_2 (0.3 M) was then added slowly. The reaction mixture was then warmed to RT to stir for 2 h before DBU (2.0 equiv.) was added. Once complete, the reaction mixture was diluted with H_2O then washed with 1 M HCl then sat. $NaHCO_3$, dried over $MgSO_4$ and the solvent removed under reduced pressure. The resulting crude material was triturated with Et_2O, the filtrates combined, and the solvent removed under reduced pressure to give the crude α,β-unsaturated ester.

$LiOH{\cdot}H_2O$ (2.5 equiv.) was added to a solution of the crude α,β-unsaturated ester (1.0 equiv.) in 1:1 THF:H_2O (1.0 M) and the reaction mixture stirred at RT. Once complete, the majority of the THF was removed under reduced pressure then the reaction mixture acidified to pH 1 with 1 M HCl. The mixture was extracted with CH_2Cl_2 (× 3) then the combined organics dried over $MgSO_4$, and the solvent removed under reduced pressure to give the crude acid.

General Procedure E: Preparation of α,β-unsaturated esters *via* acid chloride

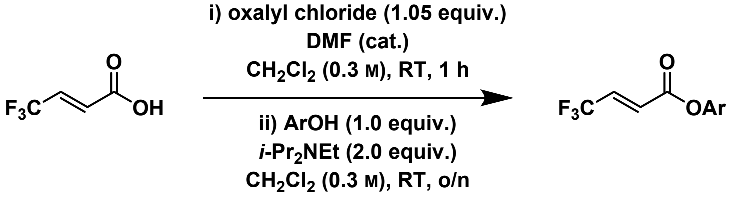

(*E*)-4,4,4-Trifluorobut-2-enoic acid (1.0 equiv.) and oxalyl chloride (1.05 equiv.) were dissolved in anhydrous CH_2Cl_2 (0.3 M). A few drops of DMF were added and the reaction mixture stirred at RT for 1 h. A solution of the requisite phenol (1.0 equiv.) and *i*-Pr_2NEt (2.0 equiv.) in anhydrous CH_2Cl_2 (0.3 M) was added slowly then the reaction mixture stirred at RT o/n. Once complete, the solvent was removed under reduced pressure and the solid triturated with Et_2O. The combined filtrates were then concentrated to give the crude product which was purified as specified.

General Procedure F: Preparation of α,β-unsaturated PFP esters

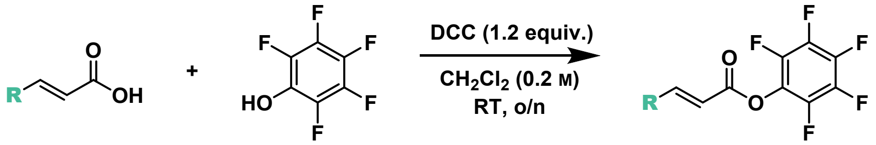

The requisite carboxylic acid (1.0 equiv.) and DCC (1.2 equiv.) were dissolved in anhydrous CH_2Cl_2 (0.2 M) with stirring at RT. After 30 min, perfluorophenol (1.0 equiv.) was added and the reaction mixture stirred o/n. Once complete, the solvent was removed under reduced pressure and the crude product purified as specified.

General Procedure G: Isothiourea-catalysed Michael addition of chalcogenoureas to α,β-unsaturated esters

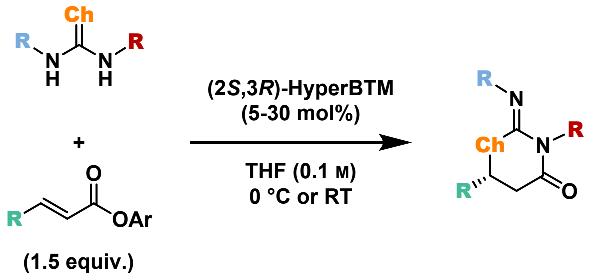

The requisite chalcogenourea (1.0 equiv.), α,β-unsaturated ester (1.5 equiv.) and the specified loading of (2*S*,3*R*)-HyperBTM were dissolved in anhydrous THF (0.1 M) then the reaction mixture stirred at the specified temperature and time. Once complete, the reaction mixture was diluted with EtOAc then washed with either sat. Na_2CO_3 (× 3) (when PNP ester used) or sat. $NaHCO_3$ (× 3) (when PFP ester used). The combined organics were dried over $MgSO_4$ then the solvent removed under reduced pressure to give the crude product which was purified as specified.

3. Reaction Optimisation

All optimisation reactions were performed on a 0.1 mmol scale.

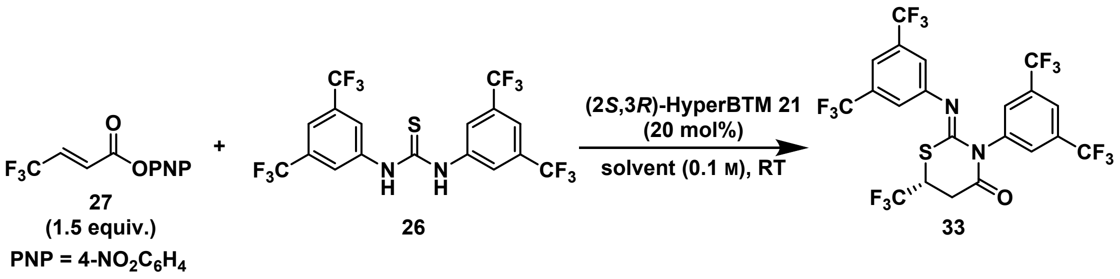

Entry	Solvent	er[a]	Yield[b] (%)
**1**	MeCN	78:22	quant.
**2**	CH_2Cl_2	66:34	quant.
**3**	EtOAc	90:10	quant.
**4**	DMF	84:16	quant.
**5**	THF	91:9	quant.
**6**	PhMe	73:27	quant.
**7**	MeOH	-	18
**8**	$CHCl_3$	69:31	quant.
**9**	Dioxane	84:16	98
**10**	Et_2O	85:15	98
**11**	Acetone	85:15	98
**12**	2-Me THF	91:9	quant.
**13**	*i*-PrOAc	90:10	98
**14**	Cyclohexanone	90:10	98
**15**	DMC	90:10	98
**16**	MTBE	86:14	95

Table S1 Solvent screen. [a] Determined by HPLC analysis on a chiral stationary phase. [b] Yield determined by ^{1}H NMR analysis relative to internal standard 1,3,5-trimethoxybenzene.

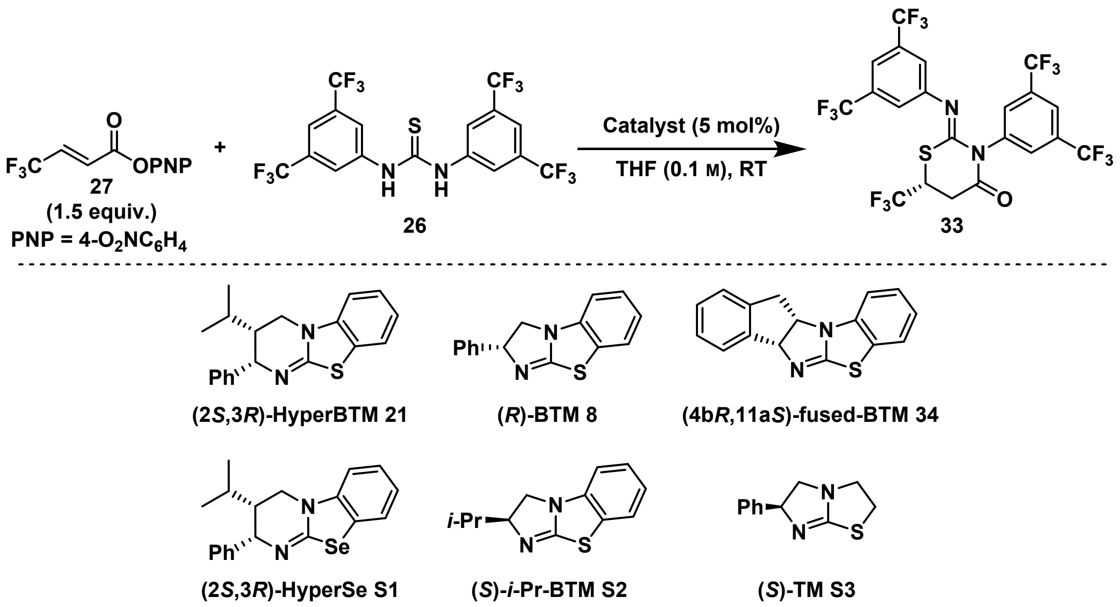

Entry	Catalyst	er[a]	Yield[b] (%)
**1**	**21**	91:9	quant.
**2**	**21**·HCl	91:9	93
**3**	**8**	84:16	96
**4**	**34**	33:67	98
**5**	**S1**	90:10	95
**6**	**S2**	27:73	90
**7**	**S3**	18:82	92

Table S2 Catalyst screen. [a] Determined by HPLC analysis on a chiral stationary phase. [b] Yield determined by ^1H NMR analysis relative to internal standard 1,3,5-trimethoxybenzene.

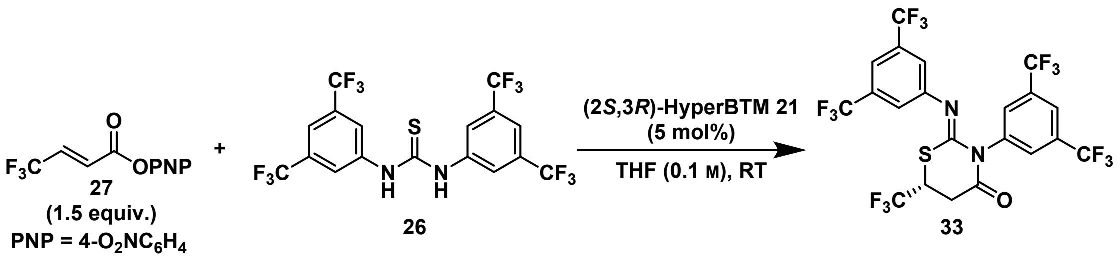

Entry	Conditions	er[a]	Yield[b] (%)
**1**	Concentration 0.5 M	87:13	92
**2**	Concentration 0.01 M	90:10	87
**3**	Stoichiometry reversed	91:9	quant.
**4**	**27** added over 1 h	90:10	98
**5**	**26** added over 1 h	91:9	84

Table S3 Optimisation of reaction conditions. [a] Determined by HPLC analysis on a chiral stationary phase. [b] Yield determined by ^{1}H NMR analysis relative to internal standard 1,3,5-trimethoxybenzene.

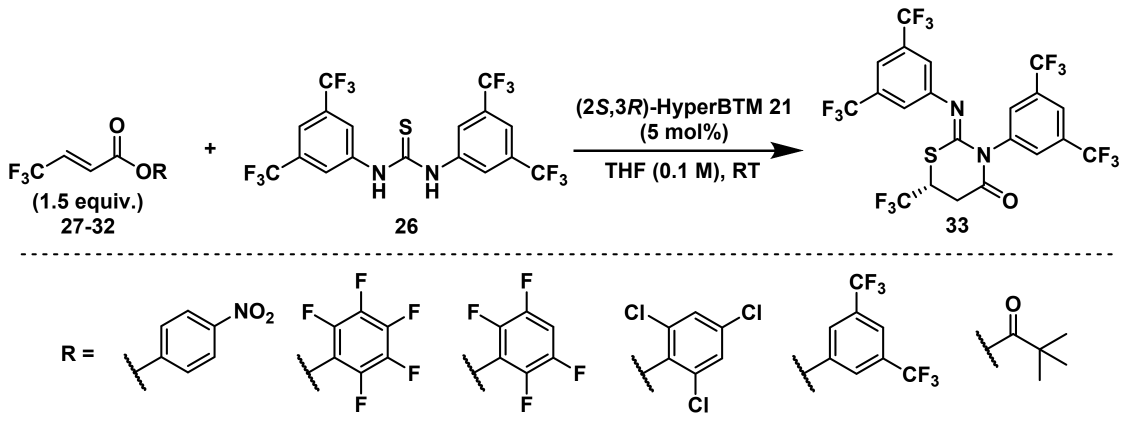

27 28 29 30 31 32

Entry	Precursor	Phenol pK_a[a]	er[b]	Yield[c] (%)
**1**	**27**	7.16	91:9	quant.
**2**	**28**	5.53	93:7	90
**3**	**29**	6.00	92:8	quant.
**4**	**30**	5.99	92:8	96
**5**	**31**	8.26	90:10	95
**6[d]**	**32**	-	90:10	83
**7[e]**	**28**	5.53	95:5	quant.

Table S4 Screen of α,β-unsaturated acyl ammonium precursors. [a] pK_a in H_2O[1-3] [b] Determined by HPLC analysis on a chiral stationary phase. [c] Yield determined by ^{1}H NMR analysis relative to internal standard 1,3,5-trimethoxybenzene. [d] Formed *in situ* from (*E*)-4,4,4-trifluorobut-2-enoic acid (1.5 equiv.), pivaloyl chloride (1.5 equiv.), and i-Pr_2NEt (1.5 equiv.). [e] Reaction temperature was 0 °C.

4. Preparation of α,β-Unsaturated Esters

The following esters were available in the laboratory, previously prepared in accordance with literature: **30**,[4] **31**,[4] **S4**,[5] **S5**,[6] and **S6**.[7]

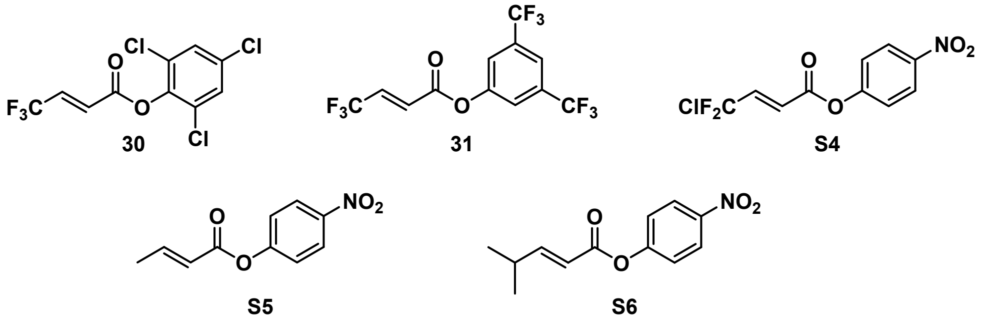

4-Nitrophenyl (*E*)-4,4,4-trifluorobut-2-enoate (27)

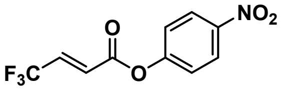

27

Following General Procedure E, (*E*)-4,4,4-trifluorobut-2-enoic acid (2.33 g, 16.6 mmol, 1.0 equiv.), oxalyl chloride (1.47 mL, 17.4 mmol, 1.05 equiv.), then 4-nitrophenol (2.31 g, 16.6 mmol, 1.0 equiv.) and *i*-Pr_2NEt (5.68 mL, 33.2 mmol, 2.0 equiv.) in CH_2Cl_2 (0.17 M) gave crude product that was purified by flash silica column chromatography (CH_2Cl_2, R_F 0.81), then recrystallised from hexane to give the title compound as colourless crystals (3.75 g, 86%). **mp** 70 – 72 °C {lit[4] 93 – 95 °C}; **^{1}H NMR** (400 MHz, $CDCl_3$) δ_H: 6.72 (1H, dq, *J* 15.8, 1.9, C(2)*H*), 7.02 (1H, dq, *J* 15.8, 6.4, C(3)*H*), 7.34 – 7.39 (2H, m, ArC(2,6)*H*), 8.28 – 8.35 (2H, m, ArC(3,5)*H*); **^{19}F{^{1}H} NMR** (377 MHz, $CDCl_3$) δ_F: –65.7 (s, C*F*$_3$). Spectroscopic data in accordance with the literature.[4]

Perfluorophenyl (*E*)-4,4,4-trifluorobut-2-enoate (28)

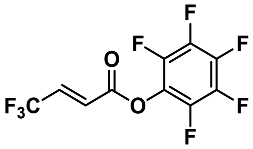

28

Following General Procedure F, (*E*)-4,4,4-trifluorobut-2-enoic acid (6.00 g, 42.8 mmol, 1.0 equiv.), pentafluorophenol (7.88 g, 42.8 mmol, 1.0 equiv.) and DCC (10.6 g, 51.4 mmol, 1.2 equiv.) in anhydrous CH_2Cl_2 (0.2 M) gave crude product that was purified by flash silica column chromatography (hexane:CH_2Cl_2 100:0 to 70:30, R_F 0.95 at 70:30) to give the title compound as a colourless oil (6.57 g, 50%). **^{1}H NMR** (400 MHz, $CDCl_3$) δ_H: 6.76 (1H, dq, *J* 15.8, 1.9, C(2)*H*), 7.06 (1H, dq, *J* 15.8, 6.3, C(3)*H*); **^{19}F{^{1}H} NMR** (377 MHz, $CDCl_3$) δ_F: –161.6 – –161.4 (m, ArC*F*), –156.6 – –156.4 (m, ArC*F*), –152.3 – –152.2 (m, ArC*F*), –66.0 (s, C*F*$_3$); **IR** ν_{max} (film) 1778, 1518, 1323, 1300, 1271, 1215, 1119, 993, 968, 937, 862. Spectroscopic data in accordance with the literature.[8]

2,3,5,6-Tetrafluorophenyl (*E*)-4,4,4-trifluorobut-2-enoate (29)

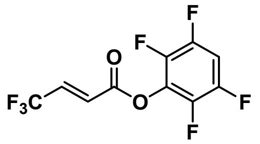

29

Following General Procedure E, (*E*)-4,4,4-trifluorobut-2-enoic acid (0.42 g, 3.0 mmol, 1.0 equiv.), oxalyl chloride (0.27 mL, 3.2 mmol, 1.05 equiv.), then 2,3,5,6-tetrafluorophenol (0.50 g, 3.0 mmol, 1.0 equiv.) and *i*-Pr_2NEt (1.03 mL, 6.0 mmol, 2.0 equiv.) in CH_2Cl_2 (0.17 M) gave crude product that was purified by flash silica column chromatography (hexane:CH_2Cl_2 80:20, R_F 0.57) to give the title compound as a colourless low-melting solid (0.62 g, 72%). **^{1}H NMR** (500 MHz, $CDCl_3$) δ_H: 6.76 (1H, dq, *J* 15.8, 1.9, C(2)*H*), 7.02 – 7.12 (2H, m, C(3)*H* and ArC(4)*H*); **^{19}F{^{1}H} NMR** (377 MHz, $CDCl_3$) δ_F: –152.5 – –152.4 (m, ArC*F*), –138.3 – –138.1 (m, ArC*F*), –65.9 (s, C*F*$_3$); **^{13}C{^{1}H} NMR** (126 MHz, $CDCl_3$) δ_C: 104.1 (t, *J* 22.8, Ar*C*(4)H), 121.6 (q, *J* 270.9, *C*F_3), 126.0 (q, *J* 6.2, *C*(2)H), 129.0 (tt, *J* 14.0, 4.0, Ar*C*(1)), 135.3 (q, *J* 36.3, *C*(3)H), 139.4 – 141.8 (m, ArCF), 145.1 – 147.4 (m, ArCF), 159.9 (*C*(1)); **IR** ν_{max} (film) 3094, 1776, 1524, 1487, 1308, 1275, 1213, 1121, 1063, 1051, 955, 843, 785; **HRMS** (ESI^-) $C_{10}H_2O_2F_7$ $[M-H]^-$ found 286.9952, requires 286.9949 (+1.0 ppm).

Ethyl (*E*)-4-fluorobut-2-enoate (S7)

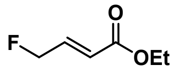

S7

Ethyl (*E*)-4-bromobut-2-eoate (3.86 g, 20.0 mmol, 1.0 equiv.) and silver(I) fluoride (7.61 g, 60.0 mmol, 30 equiv.) were dissolved in MeCN (0.5 M) and stirred at RT in the absence of light. Once complete, the reaction mixture was filtered through Celite® washing with THF then concentrated to give the crude product as a brown oil. Purification by flash silica column chromatography (hexane:EtOAc 98:2 to 90:10, R_F 0.22 at 90:10) gave the title compound as a colourless oil (1.56 g, 59%). **^{1}H NMR** (500 MHz, $CDCl_3$) δ_H: 1.30 (3H, t, *J* 7.1, CH_2CH_3), 4.22 (2H, q, *J* 7.1, CH_2CH_3), 5.05 (2H, ddd, *J* 46.1, 3.8, 2.0, CH_2F), 6.11 (1H, ddt, *J* 15.8, 2.0, 2.0, C(2)*H*), 6.96 (1H, ddt, *J* 23.1, 15.8, 3.8, C(3)*H*); **^{19}F{^{1}H} NMR** (377 MHz, $CDCl_3$) δ_F: –223.3 (s, CH_2F); **^{13}C{^{1}H} NMR** (126 MHz, $CDCl_3$) δ_C: 14.4 (CH_3), 60.8 (CH_2CH_3), 81.2 (d, *J* 171.3, CH_2F), 121.5 (d, *J* 11.0, *C*(2)H), 141.5 (d, *J*

16.0, C(3)H), 165.9 (C(1)); **IR** ν_{max} (film) 2959, 1724, 1308, 1275, 1179; **HRMS** (ESI$^+$) $C_6H_{10}O_2F$ $[M+H]^+$ found 133.0658, requires 133.0659 (–0.8 ppm).

(*E*)-4-Fluorobut-2-enoic acid (S8)

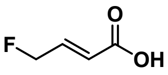

S8

Following General Procedure D, ethyl (*E*)-4-fluorobut-2-enoate **S7** (1.39 g, 10.5 mmol, 1.0 equiv.) and $LiOH{\cdot}H_2O$ (490 mg, 11.6 mmol, 1.1 equiv.) in H_2O:THF 1:1 (10 mL) after 5 h gave the title compound as a colourless solid (1.09 g, 62%), which was used without further purification. **mp** 100 – 102 °C; **^{1}H NMR** (500 MHz, $CDCl_3$) δ_H: 5.10 (2H, ddd, *J* 46.1, 3.5, 2.2, CH_2F), 6.14 (1H, dtd, *J* 15.8, 2.2, 1.4, C(2)*H*), 7.08 (1H, ddt, *J* 23.7, 15.8, 3.5, C(3)*H*); **^{19}F{^{1}H} NMR** (377 MHz, $CDCl_3$) δ_F: –224.1 (s, CH_2F); **^{13}C{^{1}H} NMR** (126 MHz, $CDCl_3$) δ_C: 81.0 (d, *J* 172.8, CH_2F), 120.5 (d, *J* 11.4, C(2)H), 144.5 (d, *J* 15.8, C(3)H), 171.4 (C(1)); **IR** ν_{max} (film) 2947, 1645, 1425, 1312, 1285, 1213, 1065, 1022, 964, 907; **HRMS** (ESI$^-$) $C_4H_4O_2F$ $[M-H]^-$ found 103.0196, requires 103.0201 (–4.9 ppm).

(*E*)-4,4-Difluorobut-2-enoic acid (S9)

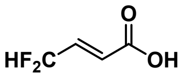

S9

Following General Procedure D, ethyl 4,4-difluoroacetoacetate (4.05 mL, 30.0 mmol, 1.0 equiv.) and $NaBH_4$ (1.36 g, 36.0 mmol, 1.2 equiv.) in MeOH (0.5 M) gave the crude alcohol. The crude alcohol (3.00 g, 17.8 mmol, 1.0 equiv.), NEt_3 (3.72 mL, 26.7 mmol, 1.5 equiv.), MsCl (2.07 mL, 26.7 mmol, 1.5 equiv.), then DBU (5.31 mL, 35.6 mmol, 2.0 equiv.) in CH_2Cl_2 (0.15 M) gave the crude α,β-unsaturated ethyl ester. The crude α,β-unsaturated ethyl ester (2.46 g, 16.4 mmol, 1.0 equiv.) and $LiOH{\cdot}H_2O$ (1.72 g, 41.0 mmol, 2.5 equiv.) in 1:1 THF:H_2O (1.0 M) gave the crude title compound (82:18 (*E*):(*Z*)) as a brown oil (1.42 g, 39%) which was used without further purification.

An analytical sample was obtained by sublimation to give the title compound as colourless crystals. **mp** 51 – 53 °C; **^{1}H NMR** (400 MHz, $CDCl_3$) δ_H: 6.27 (1H, tdd, *J* 54.6, 4.0, 1.0, CF_2*H*), 6.31 (1H, dtd, *J* 15.9, 3.0, 1.0, C(2)*H*), 6.92 (1H, dtd, *J* 15.9, 10.2, 4.0, C(3)*H*);

^{19}**F{^{1}H} NMR** (377 MHz, $CDCl_3$) δ_F: –116.8 (s, C*F*$_2$H); ^{13}**C{^{1}H} NMR** (126 MHz, $CDCl_3$) δ_C: 112.2 (t, *J* 237.8, *C*F$_2$H), 126.5 (t, *J* 10.4, *C*(2)H), 139.1 (t, *J* 24.2, *C*(3)H), 170.1 (*C*(1)); **IR** ν_{max} (film) 2880, 2664, 2575, 1697, 1657, 1423, 1385, 1346, 1279, 1250, 1225, 1047, 980, 876; **HRMS** (ESI$^-$) $C_4H_3O_2F_2$ [M-H]$^-$ found 121.0106, requires 121.0107 (–0.8 ppm).

(*E*)-4-Methoxybut-2-enoic acid (S10)

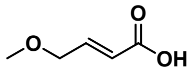

S10

Following General Procedure D, methyl 4-methoxyacetoacetate (3.88 mL, 30.0 mmol, 1.0 equiv.) and $NaBH_4$ (1.36 g, 36.0 mmol, 1.2 equiv.) in MeOH (0.5 M) gave the crude alcohol. The crude alcohol (2.03 g, 13.7 mmol, 1.0 equiv.), NEt_3 (2.09 mL, 20.6 mmol, 1.5 equiv.), MsCl (1.60 mL, 20.6 mmol, 1.5 equiv.), then DBU (4.10 mL, 27.4 mmol, 2.0 equiv.) in CH_2Cl_2 (0.15 M) gave the crude α,β-unsaturated methyl ester. The crude α,β-unsaturated ethyl ester (1.82 g, 14.0 mmol, 1.0 equiv.) and LiOH·H_2O (1.47 g, 35.0 mmol, 2.5 equiv.) in 1:1 THF:H_2O (1.0 M) gave the crude title compound (95:5 (*E*):(*Z*)) as a colourless solid (1.07 g, 31%) which was used without further purification. **mp** 48 – 50 °C; ^{1}**H NMR** (400 MHz, $CDCl_3$) δ_H: 3.40 (3H, s, OC*H*$_3$), 4.11 (2H, dd, *J* 4.1, 2.1, C(4)*H*$_2$), 6.07 (1H, dd, *J* 15.7, 2.1, C(2)*H*), 7.05 (1H, dd, *J* 15.7, 4.1, C(3)*H*); ^{13}**C{^{1}H} NMR** (126 MHz, $CDCl_3$) δ_C: 58.9 (OCH_3), 71.1 (*C*(4)H$_2$), 120.6 (*C*(2)H), 147.0 (*C*(3)H), 171.7 (*C*(1)); **IR** ν_{max} (film) 2934, 2596, 1686, 1655, 1643, 1449, 1425, 1387, 1308, 1217, 1186, 1125, 1022, 962, 920, 841.

Spectroscopic data in accordance with literature.[9]

Perfluorophenyl (*E*)-4,4,5,5,5-pentafluoropent-2-enoate (S11)

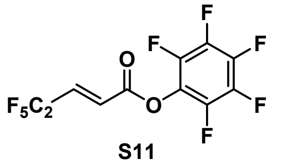

S11

Following General Procedure F, (*E*)-4,4,5,5,5-pentafluoropent-2-enoic acid (0.76 g, 4.0 mmol, 1.0 equiv.), pentafluorophenol (0.74 g, 4.0 mmol, 1.0 equiv.) and DCC (0.99 g, 4.8 mmol, 1.2 equiv.) in anhydrous CH_2Cl_2 (0.2 M) gave crude product that was

purified by flash silica column chromatography (hexane:Et_2O 100:0 to 95:5, R_F 0.82 at 95:5) to give the title compound as a colourless oil (0.26 g, 18%). **^{1}H NMR** (400 MHz, $CDCl_3$) δ_H: 6.81 (1H, dt, *J* 15.9, 2.1, C(2)*H*), 7.09 (1H, dt, *J* 15.9, 11.4, C(3)*H*); **^{19}F{^{1}H} NMR** (377 MHz, $CDCl_3$) δ_F: –162.0 – –161.8 (m, ArC*F*), –156.9 (t, *J* 21.4, ArC*F*), –152.6 – –152.5 (m, ArC*F*), –117.8 (q, *J* 2.2, C*F*$_2$CF$_3$), –84.6 (t, *J* 2.2, CF$_2$C*F*$_3$); **^{13}C{^{1}H} NMR** (126 MHz, $CDCl_3$) δ_C: 111.1 (tq, *J* 252.8, 39.7, *C*F$_2$CF$_3$), 118.3 (qt, *J* 285.6, 36.0, CF$_2$*C*F$_3$), 124.1 – 124.5 (m, Ar*C*(1)), 127.5 (t, *J* 8.5, *C*(2)H), 134.6 (t, *J* 24.3, *C*(3)H), 136.7 – 139.2 (m, ArCF), 138.7 – 141.3 (m, ArCF), 139.8 – 142.1 (m, ArCF), 159.5 (*C*(1)); **IR** ν_{max} (film) 1780, 1518, 1281, 1200, 1148, 1121, 1043, 995, 972, 939; **HRMS** (EI^+) $C_{11}H_2O_2F_{10}$ $[M]^+$ found 355.9897, requires 355.9890 (+2.0 ppm).

Perfluorophenyl (*E*)-4,4-difluorobut-2-enoate (S12)

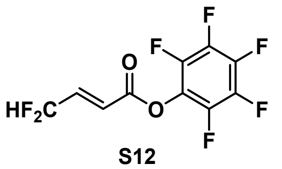

S12

Following General Procedure F, (*E*)-4,4-difluorobut-2-enoic acid **S9** (0.92 g, 7.5 mmol, 1.0 equiv.), pentafluorophenol (1.38 g, 7.5 mmol, 1.0 equiv.) and DCC (1.86 g, 9.0 mmol, 1.2 equiv.) in anhydrous CH_2Cl_2 (0.2 M) gave crude product that was purified by flash silica column chromatography (hexane:EtOAc 100:0 to 90:10, R_F 0.57 at 90:10) to give the title compound as a colourless oil (0.64 g, 30%). **^{1}H NMR** (400 MHz, $CDCl_3$) δ_H: 6.34 (1H, tdd, *J* 54.4, 3.7, 1.1, CF$_2$*H*), 6.55 (1H, dtd, *J* 15.9, 2.9, 1.1, C(2)*H*), 7.10 (1H, dtd, *J* 15.9, 10.3, 3.7, C(3)*H*); **^{19}F{^{1}H} NMR** (377 MHz, $CDCl_3$) δ_F: –162.0 – –161.9 (m, ArC*F*), –157.2 (t, *J* 21.7, ArC*F*), –152.5 – –152.4 (m, ArC*F*), –117.6 (s, C*F*$_2$H); **^{13}C{^{1}H} NMR** (126 MHz, $CDCl_3$) δ_C: 111.8 (t, *J* 238.6, *C*F$_2$H), 124.0 (t, *J* 10.4, *C*(2)H), 124.5 – 124.9 (m, Ar*C*(1)), 136.9 – 139.3 (m, ArCF), 138.8 – 141.2 (ArCF), 140.1 – 142.4 (m, ArCF), 140.9 (t, *J* 24.2, *C*(3)H), 160.6 (*C*(1)); **IR** ν_{max} (film) 2988, 1773, 1516, 1385, 1267, 1254, 1219, 1123, 1045, 993, 934, 864; **HRMS** (EI^+) $C_{10}H_3O_2F_7$ $[M]^+$ found 288.0013, requires 288.0016 (–1.0 ppm).

Perfluorophenyl (*E*)-4-fluorobut-2-enoate (S13)

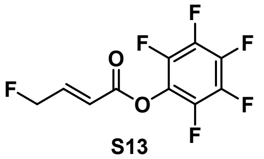

S13

Following General Procedure F, (*E*)-4-fluorobut-2-enoic acid **S8** (0.19 g, 1.8 mmol, 1.0 equiv.), pentafluorophenol (0.33 g, 1.8 mmol, 1.0 equiv.) and DCC (0.45 g, 2.2 mmol, 1.2 equiv.) in anhydrous CH_2Cl_2 (0.2 M) gave crude product that was purified by flash silica column chromatography (hexane:Et_2O 100:0 to 90:10, R_F 0.33 at 95:5) to give the title compound as a colourless solid (0.34 g, 70%). **mp** 44 – 46 °C; **^{1}H NMR** (400 MHz, $CDCl_3$) δ_H: 5.17 (2H, ddd, *J* 46.0, 3.2, 2.2, C(4)*H*$_2$F), 6.39 (1H, dtd, *J* 15.8, 2.2, 1.1, C(2)*H*), 7.27 (1H, ddt, *J* 24.2, 15.8, 3.2, C(3)*H*); **^{19}F{^{1}H} NMR** (377 MHz, $CDCl_3$) δ_F: –224.9 (s, C(4)H$_2$*F*),
–162.6 – –162.4 (m, ArC*F*), –158.1 (t, *J* 21.5, ArC*F*), –152.8 – –152.7 (m, ArC*F*); **^{13}C{^{1}H} NMR** (126 MHz, $CDCl_3$) δ_C: 80.9 (d, *J* 174.7, *C*(4)H$_2$F), 117.6 (d, *J* 12.0, *C*(2)H), 124.9 – 125.3 (m, Ar*C*(1)), 136.9 – 139.3 (m, ArCF), 138.5 – 140.9 (m, ArCF), 140.2 – 142.5 (m, ArCF), 147.1 (d, *J* 15.5, *C*(3)H), 161.5 (*C*(1)); **IR** ν_{max} (film) 2930, 1755, 1663, 1514, 1470, 1445, 1298, 1275, 1148, 1125, 1078, 991, 959, 937, 916, 824; **HRMS** (EI^+) $C_{10}H_4O_2F_6$ $[M]^+$ found 270.0118, requires 270.0110 (+3.0 ppm).

Perfluorophenyl (*E*)-4-methoxybut-2-enoate (S14)

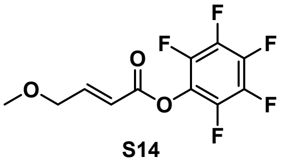

S14

Following General Procedure F, (*E*)-4-methoxybut-2-enoic acid **S10** (0.99 g, 8.5 mmol, 1.0 equiv.), pentafluorophenol (1.56 g, 8.5 mmol, 1.0 equiv.) and DCC (2.10 g, 10.2 mmol, 1.2 equiv.) in anhydrous CH_2Cl_2 (0.2 M) gave crude product that was purified by flash silica column chromatography (hexane:Et_2O 100:0 to 85:15, R_F 0.39 at 90:10) to give the title compound as a colourless oil (0.66 g, 28%). **^{1}H NMR** (400 MHz, $CDCl_3$) δ_H: 3.45 (3H, s, OC*H*$_3$), 4.19 (2H, dd, *J* 3.8, 2.2, C(4)*H*$_2$), 6.34 (1H, dt, *J* 15.7, 2.2, C(2)*H*), 7.26 (1H, dt, *J* 15.7, 3.8, C(3)*H*); **^{19}F{^{1}H} NMR** (376 MHz, $CDCl_3$) δ_F: –162.7 – –162.6 (m, ArC*F*),

–158.4 (t, *J* 21.8, ArC*F*), –152.8 – –152.7 (m, ArC*F*); **^{13}C{^{1}H} NMR** (126 MHz, $CDCl_3$) δ_C: 59.1 (OCH_3), 71.1 (*C*(4)H_2), 117.5 (*C*(2)H), 125.0 – 125.4 (m, ArC(1)), 136.8 – 139.2 (m, ArCF), 138.4 – 140.7 (m, ArCF), 140.2 – 142.6 (m, ArCF), 150.1 (*C*(3)H), 162.0 (*C*(1)); **IR** ν_{max} (film) 2934, 2830, 1759, 1659, 1516, 1472, 1281, 1192, 1111, 991, 928; **HRMS** (EI^+) $C_{11}H_7O_3F_5$ $[M]^+$ found 282.0308, requires 282.0310 (–0.7 ppm).

Perfluorophenyl (*E*)-pent-2-enoate (S15)

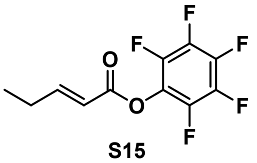

S15

(*E*)-Pent-2-enoic acid (0.51 mL, 5.0 mmol, 1.0 equiv.) and oxalyl chloride (0.44 mL, 5.25 mmol, 1.05 equiv.) were dissolved in anhydrous CH_2Cl_2 (0.3 M). A few drops of DMF were added and the reaction mixture stirred at RT for 1 h. A solution of pentafluorophenol (0.92 g, 5.0 mmol, 1.0 equiv.) and *i*-Pr_2NEt (1.71 mL, 10.0 mmol, 2.0 equiv.) in anhydrous CH_2Cl_2 (0.3 M) was then added dropwise and the reaction mixture stirred at RT o/n. Once complete, the solvent was removed under reduced pressure to give the crude product which was purified by flash silica column chromatography (hexane:CH_2Cl_2 80:20 to 50:50, R_F 0.84 at 50:50) to give the title compound as a colourless oil (0.84 g, 63%). **^{1}H NMR** (400 MHz, $CDCl_3$) δ_H: 1.15 (3H, t, *J* 7.4, C(5)H_3), 2.32 – 2.41 (2H, m, C(4)H_2), 6.06 (1H, dt, *J* 15.7, 1.8, C(2)*H*), 7.36 (1H, dt, *J* 15.7, 6.3, C(3)*H*); **^{19}F{^{1}H} NMR** (377 MHz, $CDCl_3$) δ_F: –162.8 – –162.6 (m, ArC*F*), –158.6 (t, *J* 21.7, ArC*F*), –152.9 – –152.7 (m, ArC*F*); **IR** ν_{max} (film) 2974, 1759, 1651, 1514, 1283, 1209, 1123, 1101, 1024, 991, 891, 854.
Spectroscopic data in accordance with the literature.[8]

Ethyl perfluorophenyl fumarate (S16)

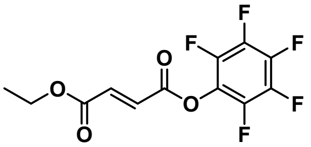

S16

(E)-4-Ethoxy-4-oxobut-2-enoic acid (2.88 g, 20.0 mmol, 2.0 equiv.) and EDCI·HCl (2.88 g, 15.0 mmol, 1.5 equiv.) were dissolved in anhydrous CH_2Cl_2 (0.3 M) and stirred at RT for 15 min. Pentafluorophenol (1.84 g, 10.0 mmol, 1.0 equiv.) was then added and the reaction mixture stirred at RT o/n. Once complete, the mixture was diluted with CH_2Cl_2 and washed with sat. $NaHCO_3$ (× 3), dried over $MgSO_4$, and the solvent removed under reduced pressure to give the crude product. Purification by flash silica column chromatography (hexane:CH_2Cl_2 80:20 to 0:100, R_F 0.44 at 50:50) gave the title compound as a colourless low-melting solid (1.83 g, 59%). **^{1}H NMR** (400 MHz, $CDCl_3$) δ_H: 1.35 (3H, t, J 7.1, CH_3), 4.32 (2H, q, J 7.1, CH_2), 7.06 (1H, d, 15.8, C*H*=CH), 7.12 (1H, d, 15.8, CH=C*H*); **^{19}F{^{1}H} NMR** (376 MHz, $CDCl_3$) δ_F: –162.0 – –161.8 (m, ArC*F*), –157.1 (t, J 21.6, ArC*F*), –152.4 – –152.3 (m, ArC*F*); **IR** ν_{max} (film) 2990, 1767, 1726, 1516, 1283, 1117, 993, 982, 926, 764.

Spectroscopic data in accordance with the literature.[8]

Perfluorophenyl acrylate (75)

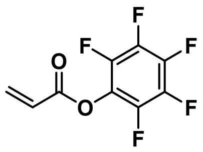

75

Following General Procedure F, acrylic acid (1.37 mL, 20.0 mmol, 1.0 equiv.), pentafluorophenol (3.68 g, 20.0 mmol, 1.0 equiv.) and DCC (4.95 g, 24.0 mmol, 1.2 equiv.) in anhydrous CH_2Cl_2 (0.2 M) gave crude product that was purified by flash silica column chromatography (hexane:Et_2O 100:0 to 90:10, R_F 0.66 at 90:10) to give the title compound as a colourless volatile oil (2.67 g, 56%). **^{1}H NMR** (400 MHz, $CDCl_3$) δ_H: 6.18 (1H, dd, J 10.5, 1.0, CHC$H^A$$H^B$), 6.37 (1H, dd, J 17.3, 10.5, C*H*CH$_2$), 6.72 (1H, dd, J 17.3, 1.0, CHCH^{A}CH^B); **^{19}F{^{1}H} NMR** (377 MHz, $CDCl_3$) δ_F: –162.5 – –162.3 (m, ArC*F*), –158.0 (t, J 21.7, ArC*F*), –152.7 – –152.6 (m, ArC*F*); **IR** ν_{max} (film) 1771, 1514, 1406, 1217, 1111, 991, 978, 870, 797.

Spectroscopic data in accordance with the literature.[10]

5. Preparation of Thioureas

1,3-Bis(3,5-bis(trifluoromethyl)phenyl)thiourea (26)

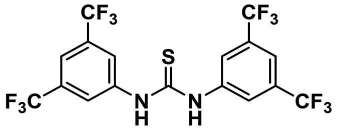

26

3,5-Bis(trifluoromethyl)phenyl isothiocyanate (1.83 mL, 10 mmol, 1.0 equiv.) and 3,5-bis(trifluoromethyl)aniline (1.56 mL, 10 mmol, 1.0 equiv.) were dissolved in anhydrous CH_2Cl_2 (0.2 M) and the reaction mixture stirred at RT for 5 h. Once complete the solvent was removed to give the crude product as a white solid. Recrystallisation from $CHCl_3$/EtOAc gave the title compound as colourless crystals (4.39 g, 88%). **mp** 143 – 145 °C {lit[11] 170 – 172}; **^{1}H NMR** (400 MHz, $CDCl_3$) δ_H: 7.78 (2H, s, ArC(4)*H*), 7.89 (4H, s, ArC(2,6)*H*), 7.92 (2H, s, N*H*); **^{19}F{^{1}H} NMR** (377 MHz, $CDCl_3$) δ_F: –63.0 (s, C*F*$_3$).

Spectroscopic data in accordance with literature.[11]

1,3-Bis(4-(trifluoromethyl)phenyl)thiourea (S17)

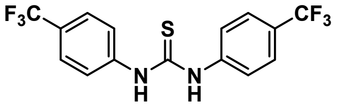

S17

4-Trifluoromethylphenyl isothiocyanate (2.03 g, 10 mmol, 1.0 equiv.) and 4-trifluoromethylaniline (1.26 mL, 10 mmol, 1.0 equiv.) were dissolved in anhydrous CH_2Cl_2 (0.2 M) and the reaction mixture stirred at RT for 6 h. Once complete the solvent was removed to give the crude product as a yellow solid. Recrystallisation from $CHCl_3$/EtOAc gave the title compound as colourless crystals (2.87 g, 79%). **mp** 131 – 133 °C {lit[11] 161 – 163 °C}; **^{1}H NMR** (400 MHz, $CDCl_3$) δ_H: 7.53 – 7.60 (4H, m, ArC(2,6)*H*), 7.68 – 7.75 (4H, m, ArC(3,5)*H*), 7.89 (2H, br s, N*H*); **^{19}F{^{1}H} NMR** (377 MHz, $CDCl_3$) δ_F: –62.5 (s, C*F*$_3$).

Spectroscopic data in accordance with literature.[11]

1,3-Bis(4-nitrophenyl)thiourea (S18)

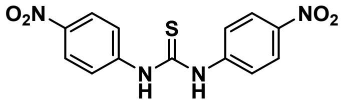

S18

4-Nitrophenylisothiocyanate (2.70 g, 15 mmol, 1.0 equiv.) and 4-nitroaniline (2.07 g, 15 mmol, 1.0 equiv.) were dissolved in anhydrous CH_2Cl_2 (0.2 M). Pyridine (1.21 mL, 15 mmol, 1.0 equiv.) was added and the reaction mixture stirred at RT. Once complete, the solvent was removed, and the crude product recrystallised from acetone to give the title compound as golden crystals (3.64 g, 76%). **mp** 163 – 165 °C; **^{1}H NMR** (400 MHz, $(CD_3)_2SO$) δ_H: 7.81 – 7.87 (4H, m, ArC(2,6)*H*), 8.21 – 8.27 (4H, m, ArC(3,5)*H*), 10.77 (2H, br s, N*H*); **^{13}C{^{1}H} NMR** (126 MHz, $(CD_3)_2SO$) δ_C: 122.5 (Ar*C*(2,6)H), 125.0 (Ar*C*(3,5)H), 143.2 (ArC(1)), 146.3(ArC(4)), 179.6 (*C*S); **IR** ν_{max} (film) 3318, 1589, 1580, 1539, 1493, 1317, 1292, 1242, 1233, 1173, 1105, 860, 843, 804; **HRMS** (ESI^-) $C_{13}H_9O_4N_4S$ $[M-H]^-$ found 317.0348, requires 317.0350 (–0.6 ppm).

1,3-Bis(4-cyanophenyl)thiourea (S19)

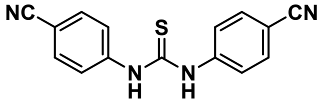

S19

4-Cyanophenylisothiocyanate (1.60 g, 10 mmol, 1.0 equiv.) and 4-aminobenzonitrile (1.18 g, 10 mmol, 1.0 equiv.) were dissolved in anhydrous Et_2O (0.2 M). Pyridine (0.81 mL, 10 mmol, 1.0 equiv.) was added and the reaction mixture stirred at RT. Once complete, the precipitate was filtered off washing with CH_2Cl_2 to afford the title compound as a white solid (2.09 g, 75%). **mp** 144 – 146 °C; **^{1}H NMR** (400 MHz, $(CD_3)_2SO$) δ_H: 7.74 – 7.83 (8H, m, ArC(2,6)*H* and ArC(3,5)*H*), 10.52 (2H, br s, N*H*); **^{13}C{^{1}H} NMR** (126 MHz, $(CD_3)_2SO$) δ_C: 106.3 (*C*N), 119.4 (Ar*C*(4)), 123.1 (Ar*C*(2,6)H), 133.3 (Ar*C*(3,5)H), 144.1 (Ar*C*(1)), 179.6 (*C*S); **IR** ν_{max} (film) 3291, 2218, 1589, 1526, 1506, 1327, 1290, 1254, 1229, 1198, 1173, 827; **HRMS** (ESI^+) $C_{15}H_{10}N_4NaS$ $[M+Na]^+$ found 301.0514, requires 301.0518 (–1.3 ppm).

1-(4-Nitrophenyl)-3-(2,4,6-trichlorophenyl)thiourea (S20)

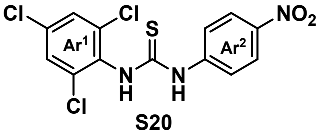

S20

2,4,6-Trichlorophenylisothiocyanate (1.19 g, 5.0 mmol, 1.0 equiv.) and 4-nitroaniline (0.69 g, 5.0 mmol, 1.0 equiv.) were dissolved in anhydrous CH_2Cl_2 (0.2 M). Pyridine (0.40 mL, 5.0 mmol, 1.0 equiv.) was added and the reaction mixture stirred at RT. Once complete, the solvent was removed under reduced pressure to afford the crude product which was recrystallised from acetone to give the title compound as yellow crystals (0.81 g, 43%). **mp** 140 – 142 °C; **^{1}H NMR** (500 MHz, $(CD_3)_2SO$) δ_H: 7.78 (2H, s, $Ar^1C(3,5)H$), 7.86 – 7.96 (2H, m, $Ar^2C(2,6)H$), 8.20 – 8.26 (2H, m, $Ar^2C(3,5)H$), 9.86 (1H, br s, N*H*), 10.79 (1H, br s, N*H*); **^{13}C{^{1}H} NMR** (126 MHz, $(CD_3)_2SO$) δ_C: 121.7 ($Ar^2C(2,6)H$), 124.4 ($Ar^2C(3,5)H$), 128.3 ($Ar^1C(3,5)H$), 132.8 ($Ar^1C(4)$), 133.8 ($Ar^1C(1)$), 135.6 ($Ar^1C(2,6)$), 142.7 ($Ar^2C(1)$), 145.8 ($Ar^1C(4)$), 180.4 (*C*S); **IR** ν_{max} (film) 3374, 3281, 3051, 1589, 1495, 1479, 1325, 1302, 1265, 1186, 1107, 853, 831, 818, 800; **HRMS** (ESI^-) $C_{13}H_7O_2N_3{}^{35}Cl_3S$ $[M-H]^-$ found 373.9337, requires 373.9330 (+1.9 ppm).

4-Methyl-*N*-(phenylcarbamothioyl)benzenesulfonamide (77)

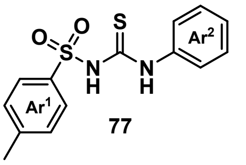

77

Following General Procedure B, *p*-toluenesulfonamide (0.86 g, 5.0 mmol, 1.0 equiv.), phenyl isothiocyanate (0.60 mL, 5.0 mmol, 1.0 equiv.) and Cs_2CO_3 (1.63 g, 5.0 mmol, 1.0 equiv.) in acetone (0.25 M) gave the title compound as a white solid (0.69 g, 45%). **mp** 105 – 107 °C {lit[12] 145 – 148 °C}; **^{1}H NMR** (400 MHz, $(CD_3)_2SO$) δ_H: 2.50 (3H, s, CH_3), 7.27 – 7.34 (1H, m, $Ar^2C(4)H$), 7.42 – 7.50 (2H, m, $Ar^2C(3,5)H$), 7.51 – 7.61 (4H, m, $Ar^1C(3,5)H$ and $Ar^2C(2,6)H$), 7.97 – 8.04 (2H, m, $Ar^1C(2,6)H$), 10.36 (1H, s, N*H*), 11.66 (1H, br s, N*H*).

Spectroscopic data in accordance with literature.[12]

N-((4-Methoxyphenyl)carbamothioyl)-4-methylbenzenesulfonamide (S21)

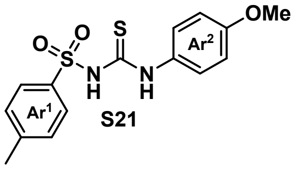

S21

Following General Procedures A and B, *p*-anisidine (0.62 g, 5.0 mmol, 1.0 equiv.), thiophosgene (0.57 mL, 7.5 mmol, 1.5 equiv.) and $NaHCO_3$ (1.68 g, 20.0 mmol, 4.0 equiv.) in 1:1 H_2O:CH_2Cl_2 (0.25 M) followed by *p*-toluenesulfonamide (0.86 g, 5.0 mmol, 1.0 equiv.) and Cs_2CO_3 (1.63 g, 5.0 mmol, 1.0 equiv.) in acetone (0.25 M) gave the crude product as a pale-brown solid. The crude product was recrystallised from EtOH to afford the title compound as pale-brown crystals (0.90 g, 54%). **mp** 108 – 110 °C {lit[13] 144 °C}; **^{1}H NMR** (400 MHz, ($CDCl_3$) δ_H: 2.45 (3H, s, CH_3), 3.79 (3H, s, OCH_3), 6.85 – 6.92 (2H, m, Ar^{2}C(3,5)*H*), 7.23 – 7.29 (2H, m, Ar^{1}C(3,5)*H*), 7.33 – 7.39 (2H, m, Ar^{2}C(2,6)*H*), 7.77 – 7.84 (2H, m, Ar^{1}C(2,6)*H*), 8.55 (1H, br s, N*H*), 9.60 (1H, br s, N*H*).

Spectroscopic data in accordance with the literature.[13]

4-Methyl-N-((4-(trifluoromethyl)phenyl)carbamothioyl)benzenesulfonamide (S22)

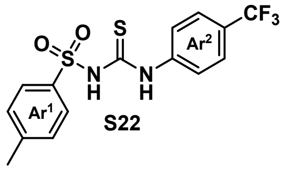

S22

Following General Procedure B, *p*-toluenesulfonamide (0.86 g, 5.0 mmol, 1.0 equiv.), 4-(trifluoromethyl)phenyl isothiocyanate (1.02 g, 5.0 mmol, 1.0 equiv.) and Cs_2CO_3 (1.63 g, 5.0 mmol, 1.0 equiv.) in acetone (0.25 M) gave the title compound as a white solid (1.73 g, 93%). **mp** 131 – 133 °C; **^{1}H NMR** (400 MHz, $CDCl_3$) δ_H: 2.47 (3H, s, CH_3), 7.35 – 7.41 (2H, m, Ar^{1}C(3,5)*H*), 7.61 – 7.71 (4H, m, Ar^{2}C(2,3,5,6)*H*), 7.78 – 7.84 (2H, m, Ar^{1}C(2,6)*H*), 8.24 (1H, br s, N*H*), 9.90 (1H, s, N*H*); **^{19}F{^{1}H} NMR** (377 MHz, $CDCl_3$) δ_F: –62.5 (s, CF_3); **^{13}C{^{1}H} NMR** (126 MHz, $CDCl_3$) δ_C: 21.9 (CH_3), 123.9 (q, *J* 272.1, CF_3), 124.0 (Ar^2*C*(2,6)H), 126.4 (q, *J* 3.8, Ar^2*C*(3,5)H), 127.2 (Ar^1*C*(2,6)H), 129.0 (q, *J* 33.0, Ar^2*C*(4)), 130.6 (Ar^{1}C(3,5)H), 135.3 (Ar^{1}C(1)), 140.5 (Ar^2*C*(1)), 146.3 (Ar^{1}C(4)), 176.8 (CS); **IR** ν_{max} (film) 3291, 3049, 2907, 1618, 1595, 1530, 1476, 1377, 1319, 1146, 1105, 1080, 1063, 1016, 920, 843, 812; **HRMS** (ESI^-) $C_{15}H_{12}O_2N_2F_3S_2$ $[M-H]^-$ found 373.0296, requires 373.0298 (–0.5 ppm).

4-Methoxy-*N*-(phenylcarbamothioyl)benzenesulfonamide (S23)

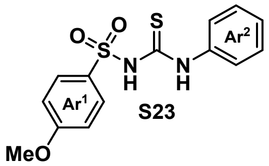

S23

Following General Procedures A and B, 4-methoxybenzenesulfonamide (0.56 g, 3.0 mmol, 1.0 equiv.), phenyl isothiocyanate (0.36 mL, 3.0 mmol, 1.0 equiv.) and Cs_2CO_3 (0.98 g, 3.0 mmol, 1.0 equiv.) in acetone (0.25 M) gave the title compound, after trituration with Et_2O, as a white solid (0.74 g, 76%). **mp** 102 – 104 °C; **^{1}H NMR** (500 MHz, $(CD_3)_2SO$) δ_H: 3.85 (3H, s, C*H*$_3$), 7.11 – 7.23 (3H, m, Ar^{1}C(3,5)*H* and Ar^{2}C(4)*H*), 7.31 – 7.38 (2H, m, Ar^{2}C(3,5)*H*), 7.40 – 7.46 (2H, m, Ar^{2}C(2,6)*H*), 7.89 – 7.94 (2H, m, Ar^{1}C(2,6)*H*), 10.18 (1H, s, N*H*), 11.45 (1H, br s, N*H*); **^{13}C{^{1}H} NMR** (126 MHz, $(CD_3)_2SO$) δ_C: 55.8 (*C*H$_3$), 114.2 (Ar^1*C*(3,5)H), 124.1 (Ar^2*C*(2,6)H), 125.8 (Ar^2*C*(4)H), 130.8 (Ar^1*C*(1)), 128.6 (Ar^2*C*(3,5)H), 130.1 (Ar^1*C*(2,6)H), 138.2 (Ar^2*C*(1)), 163.0 (Ar^1*C*(4)), 177.6 (*C*S); **IR** ν_{max} (film) 3292, 3007, 2901, 2841, 2747, 1591, 1508, 1495, 1379, 1265, 1173, 1140, 1082, 1022, 907, 845, 827, 800; **HRMS** (ESI^-) $C_{14}H_{13}O_3N_2S_2$ $[M-H]^-$ found 321.0369, requires 321.0373 (–1.2 ppm).

1-Methyl-3-(4-nitrophenyl)thiourea (S24)

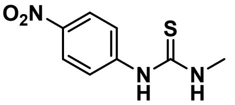

S24

Methylamine (2 M in THF) (1.1 mL, 2.2 mmol, 1.0 equiv.) was added to a solution of 4-nitrophenylisothiocyanate (0.40 g, 2.2 mmol, 1.0 equiv.) in anhydrous Et_2O (0.2 M). The reaction mixture was stirred at RT for 30 min. The resulting precipitate was filtered under vacuum, washing with Et_2O to afford the title compound as an off-white solid (0.29 g, 63%). **mp** 151 – 153 °C; **^{1}H NMR** (400 MHz, $(CD_3)_2SO$) δ_H: 2.93 (3H, d, *J* 4.4, C*H*$_3$), 7.74 – 7.83 (2H, m, ArC(2,6)*H*), 8.14 – 8.20 (2H, m, ArC(3,5)*H*), 8.25 (1H, br s, N*H*), 10.19 (1H, s, N*H*); **^{13}C{^{1}H} NMR** (126 MHz, $(CD_3)_2SO$) δ_C: 31.2 (CH$_3$), 120.3 (Ar*C*(2,6)H), 124.6 (Ar*C*(3,5)H), 141.7 (Ar*C*(1)), 146.4 (Ar*C*(4)), 180.8 (CS); **IR** ν_{max} (film) 3262, 3215, 3098, 3049, 1580, 1557, 1506, 1495, 1435, 1329, 1277, 1242, 1179, 1111, 1055, 849; **HRMS** (ESI^+) $C_8H_9O_2N_3NaS$ $[M+Na]^+$ found 234.0310, requires 234.0308 (+0.9 ppm).

4-Methyl-*N*-(methylcarbamothioyl)benzenesulfonamide (S25)

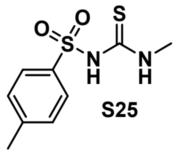

S25

Following General Procedure B, *p*-toluenesulfonamide (0.34 g, 2.0 mmol, 1.0 equiv.), methyl isothiocyanate (0.13 mL, 2.0 mmol, 1.0 equiv.) and Cs_2CO_3 (0.65 g, 2.0 mmol, 1.0 equiv.) in acetone (0.25 M) gave the crude product as a brown solid. Trituration with Et_2O gave the title compound as a colourless solid (0.20 g, 41%). **mp** 132 – 134 °C; **^{1}H NMR** (400 MHz, $(CD_3)_2SO$) δ_H: 2.39 (3H, s, C*H*$_3$), 2.88 (3H, d, *J* 4.5, NC*H*$_3$), 7.40 – 7.46 (2H, m, ArC(3,5)*H*), 7.78 – 7.83 (2H, m, ArC(2,6)*H*), 8.48 (1H, q, *J* 4.5, N*H*), 11.55 (1H, br s, N*H*); **^{13}C{^{1}H} NMR** (126 MHz, $(CD_3)_2SO$) δ_C: 21.1 (*C*H$_3$), 32.2 (N*C*H$_3$), 127.2 (Ar*C*(2,6)H), 129.6 (Ar*C*(3,5)H), 136.5 (Ar*C*(1)), 144.2 (Ar*C*(4)), 178.8 (*C*S); **IR** ν_{max} (film) 3337, 3294, 3211, 3038, 1570, 1543, 1483, 1464, 1416, 1364, 1159, 1152, 1123, 1084, 1036, 858, 810; **HRMS** (ESI^-) $C_9H_{11}O_2N_2S_2$ $[M-H]^-$ found 243.0268, requires 243.0267 (+0.4 ppm).

4-Methyl-*N*-(benzylcarbamothioyl)benzenesulfonamide (S26)

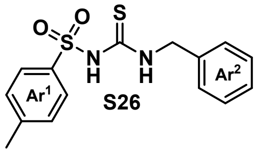

S26

Following General Procedures A and B, benzylamine (1.09 mL, 10.0 mmol, 1.0 equiv.), thiophosgene (1.15 mL, 15.0 mmol, 1.5 equiv.) and $NaHCO_3$ (3.36 g, 40.0 mmol, 4.0 equiv.) in 1:1 H_2O:CH_2Cl_2 (0.25 M) followed by *p*-toluenesulfonamide (1.71 g, 10.0 mmol, 1.0 equiv.) and Cs_2CO_3 (3.26 g, 10.0 mmol, 1.0 equiv.) in acetone (0.25 M) gave the crude product as a pale-brown solid. The crude product was triturated with Et_2O to afford the title compound as an off-white solid (1.67 g, 52%). **mp** 125 – 127 °C; **^{1}H NMR** (400 MHz, $(CD_3)_2SO$) δ_H: 2.41 (3H, s, C*H*$_3$), 4.68 (2H, d, *J* 5.7, C*H*$_2$), 7.09 – 7.16 (2H, m, Ar^{2}C(2,6)*H*), 7.19 – 7.31 (3H, m, Ar^{2}C(3,4,5)*H*), 7.38 – 7.45 (2H, m, Ar^{1}C(3,5)*H*), 7.77 – 7.82 (2H, m, Ar^{1}C(2,6)*H*), 8.89 (1H, t, *J* 5.7, N*H*), 11.62 (1H, s, N*H*); **^{13}C{^{1}H} NMR** (126 MHz, $(CD_3)_2SO$) δ_C: 21.1 (*C*H$_3$), 47.7 (*C*H$_2$), 127.0 (Ar^2*C*(4)H), 127.3 (Ar^1*C*(2,6)H), 127.3 (Ar^2*C*(2,6)H), 128.2 (Ar^2*C*(3,5(H), 129.7 (Ar^1*C*(3,5)H), 136.3 (Ar^1*C*(1)), 137.4 (Ar^2*C*(1)), 144.3 (Ar^1*C*(4)), 178.6 (*C*S); **IR** ν_{max} (film) 3321, 2980, 1543, 1489, 1435, 1383, 1344, 1256, 1171, 1134, 1084, 1065,

937, 856, 810; **HRMS** (ESI^-) $C_{15}H_{15}O_2N_2S_2$ $[M-H]^-$ found 319.0581, requires 319.0580 (+0.3 ppm).

2,2,2-Trifluoro-*N*-(phenylcarbamothioyl)acetamide (S27)

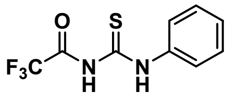

S27

Following General Procedure B, trifluoroacetamide (0.57 g, 5.0 mmol, 1.0 equiv.), phenyl isothiocyanate (0.60 mL, 5.0 mmol, 1.0 equiv.) and Cs_2CO_3 (1.63 g, 5.0 mmol, 1.0 equiv.) in acetone (0.25 M) gave the crude product as a yellow solid. Purification by flash silica column chromatography (hexane:CH_2Cl_2 25:75 to 0:100, R_F 0.29 at 0:100) gave the title compound as a white solid (0.48 g, 39%). **mp** 70 – 72 °C; **^{1}H NMR** (400 MHz, $(CD_3)_2SO$) δ_H: 7.26 – 7.33 (1H, m, ArC(4)*H*), 7.40 – 7.47 (2H, m, ArC(3,5)*H*), 7.63 – 7.71 (2H, m, ArC(2,6)*H*), 11.61 (1H, br s, N*H*), 12.47 (1H, br s, N*H*); **^{19}F{^{1}H} NMR** (377 MHz, $(CD_3)_2SO$) δ_F: –73.8 (s, C*F*$_3$); **^{13}C{^{1}H} NMR** (126 MHz, $(CD_3)_2SO$) δ_C: 115.3 (q, *J* 288.5, *C*F$_3$), 123.9 (Ar*C*(2,6)H), 126.7 (Ar*C*(4)H), 128.8 (Ar*C*(3,5)H), 137.9 (Ar*C*(1)), 154.6 (q, *J* 39.2, *C*OCF$_3$), 176.4 (CS); **IR** ν_{max} (film) 3262, 3211, 3044, 1715, 1595, 1533, 1452, 1302, 1207, 1152, 1107, 1070, 941, 858; **HRMS** (ESI^-) $C_9H_6ON_2F_3S$ $[M-H]^-$ found 247.0159, requires 247.0158 (+0.4 ppm).

***N*-(Phenylcarbamothioyl)benzamide (S28)**

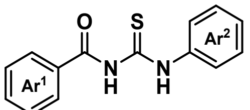

S28

Benzoyl chloride (1.16 mL, 10.0 mmol, 1.0 equiv.) was added slowly to a solution of NH_4SCN (0.84 g, 11.0 mmol, 1.1 equiv.) in acetone (0.25 M) then heated to reflux for 15 min. Aniline (0.91 mL, 10.0 mmol, 1.0 equiv.) was then added and the reaction mixture heated to reflux for a further 30 min. Once complete, the reaction mixture was poured into ice water with stirring. The resulting precipitate was filtered off under vacuum washing with H_2O to give the crude product as an off-white solid. Recrystallisation from EtOH (0.2 M) gave the title compound as colourless needles (2.01 g, 79%). **mp** 115 – 117

°C; **^{1}H NMR** (500 MHz, $(CD_3)_2SO$) δ_H: 7.24 – 7.31 (1H, m, Ar^{2}C(4)*H*), 7.40 – 7.46 (2H, m, Ar^{2}C(3,5)*H*), 7.51 – 7.57 (2H, m, Ar^{1}C(3,5)*H*), 7.63 – 7.68 (1H, m, Ar^{1}C(4)*H*), 7.69 – 7.73 (2H, m, Ar^{2}C(2,6)H), 7.96 – 8.02 (2H, m, Ar^{1}C(2,6)*H*), 11.57 (1H, s, N*H*), 12.64 (1H, s, N*H*); **^{13}C{^{1}H} NMR** (126 MHz, $(CD_3)_2SO$) δ_C: 124.3 (Ar^2*C*(2,6)H), 126.3 (Ar^2*C*(4)H), 128.4 (Ar^2*C*(3,5)H), 128.7 (Ar^1*C*(3,5)H), 128.7 (Ar^1*C*(2,6)H), 132.1 (Ar^1*C*(1)), 133.1 (Ar^1*C*(4)H), 138.0 (Ar^2*C*(1)), 168.3 (*C*S), 179.1 (*C*O); **IR** ν_{max} (film) 3258, 2986, 1670, 1599, 1558, 1526, 1483, 1447, 1356, 1254, 1142, 1070, 1026, 905, 793; **HRMS** (ESI^+) $C_{14}H_{12}ON_2NaS$ $[M + Na]^+$ found 279.0568, requires 279.0563 (+1.8 ppm).

***N*-((2-*iso*-Propylphenyl)carbamothioyl)-4-methylbenzenesulfonamide (S29)**

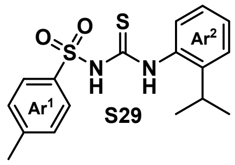

Following General Procedures A and B, 2-*iso*-propylaniline (1.42 mL, 10.0 mmol, 1.0 equiv.), thiophosgene (1.15 mL, 15.0 mmol, 1.5 equiv.) and $NaHCO_3$ (3.36 g, 40.0 mmol, 4.0 equiv.) in 1:1 H_2O:CH_2Cl_2 (0.25 M) followed by *p*-toluenesulfonamide (1.71 g, 10.0 mmol, 1.0 equiv.) and Cs_2CO_3 (3.26 g, 10.0 mmol, 1.0 equiv.) in acetone (0.25 M) gave the crude product as a pale-brown solid. The crude product was recrystallised from EtOH to afford the title compound as colourless crystals (1.85 g, 53%). **mp** 146 – 148 °C; **^{1}H NMR** (400 MHz, $(CD_3)_2SO$) δ_H: 1.03 (6H, d, *J* 6.8, CH(C*H*$_3$)$_2$), 2.42 (3H, s, C*H*$_3$), 2.71 (1H, hept, *J* 6.8, C*H*(CH$_3$)$_2$), 7.01 – 7.06 (1H, m, Ar^{2}C(6)*H*), 7.13 – 7.20 (1H, m, Ar^{2}C(5)*H*), 7.23 – 7.32 (2H, m, Ar^{2}C(3)*H* and Ar^{2}C(4)*H*), 7.43 – 7.49 (2H, m, Ar^{1}C(3,5)*H*), 7.87 – 7.93 (2H, m, Ar^{1}C(2,6)*H*), 9.76 (1H, br s, N*H*), 11.78 (1H, br s, N*H*); **^{13}C{^{1}H} NMR** (126 MHz, $(CD_3)_2SO$) δ_C: 21.1 (*C*H$_3$), 23.1 (CH(*C*H$_3$)$_2$), 27.5 (*C*H(CH$_3$)$_2$), 125.8 (Ar^2*C*(3)H), 125.9 (Ar^2*C*(6)H), 127.5 (Ar^1*C*(2,6)H), 127.9 (Ar^2*C*(5)H), 128.5 (Ar^2*C*(4)H), 129.6 (Ar^1*C*(3,5)H), 135.7 (Ar^2*C*(1)), 136.4 (Ar^1*C*(1)), 144.2 (Ar^1*C*(4)), 144.9 (Ar^2*C*(2)), 179.3 (*C*S); **IR** ν_{max} (film) 3281, 2961, 2911, 1516, 1474, 1456, 1373, 1244, 1177, 1134, 1080, 918, 849, 835, 812, 766; **HRMS** (ESI^+) $C_{17}H_{20}O_2N_2NaS_2$ $[M + Na]^+$ found 371.0867, requires 371.0858 (+2.4 ppm).

N-([1,1'-Biphenyl]-2-ylcarbamothioyl)-4-methylbenzenesulfonamide (S30)

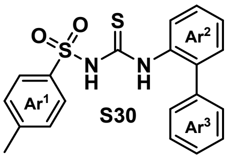

Following General Procedures A and B, 2-aminobiphenyl (1.69 g, 10.0 mmol, 1.0 equiv.), thiophosgene (1.15 mL, 15.0 mmol, 1.5 equiv.) and $NaHCO_3$ (3.36 g, 40.0 mmol, 4.0 equiv.) in 1:1 H_2O:CH_2Cl_2 (0.25 M) followed by *p*-toluenesulfonamide (1.71 g, 10.0 mmol, 1.0 equiv.) and Cs_2CO_3 (3.26 g, 10.0 mmol, 1.0 equiv.) in acetone (0.25 M) gave the crude product as a pale-brown solid. The crude product was triturated with Et_2O to afford the title compound as a white solid (2.20 g, 58%). **mp** 129 – 131 °C; **^{1}H NMR** (500 MHz, $(CD_3)_2SO$) δ_H: 2.43 (3H, s, CH_3), 7.27 – 7.39 (9H, m, $Ar^2C(3,4,5,6)H$ and $Ar^3C(2,3,4,5,6)H$), 7.40 – 7.44 (2H, m, $Ar^1C(3,5)H$), 7.69 – 7.74 (2H, m, $Ar^1C(2,6)H$), 9.66 (1H, br s, NH), 11.56 (1H, br s, NH); **^{13}C{^{1}H} NMR** (126 MHz, $(CD_3)_2SO$) δ_C: 21.1 (CH_3), 127.5 ($Ar^1C(2,6)H$), 127.5 (ArCH), 127.6 (ArCH), 127.7 (ArCH), 128.3 (ArCH), 128.6 (ArCH), 129.0 (ArCH), 129.5 ($Ar^1C(3,5)H$), 130.2 (ArCH), 135.2 (Ar*C*), 136.4 ($Ar^1C(1)$), 138.2 (Ar*C*), 138.3 (Ar*C*), 144.1 ($Ar^1C(4)$), 178.7 (CS); **IR** ν_{max} (film) 3283, 3028, 2901, 1593, 1524, 1476, 1389, 1173, 1136, 1080, 924, 831, 812, 762; **HRMS** (ESI^-) $C_{20}H_{17}O_2N_2S_2$ $[M-H]^-$ found 381.0731, requires 381.0737 (–1.6 ppm).

Note: the biphenyl carbon signals could not be unambiguously assigned, even with 2D NMR.

N-((2-(*tert*-Butyl)phenyl)carbamothioyl)-4-methylbenzenesulfonamide (74)

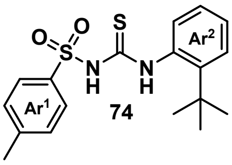

Following General Procedures A and B, 2-*tert*-butylaniline (1.56 mL, 10.0 mmol, 1.0 equiv.), thiophosgene (1.15 mL, 15.0 mmol, 1.5 equiv.) and $NaHCO_3$ (3.36 g, 40.0 mmol, 4.0 equiv.) in 1:1 H_2O:CH_2Cl_2 (0.25 M) followed by *p*-toluenesulfonamide (1.71 g, 10.0 mmol, 1.0 equiv.) and Cs_2CO_3 (3.26 g, 10.0 mmol, 1.0 equiv.) in acetone (0.25 M) gave the crude product as a pale-brown solid. The crude product was triturated with Et_2O to afford the title compound as a white solid (2.02 g, 56%). **mp** 150 – 152 °C; **^{1}H NMR** (400 MHz, $(CD_3)_2SO$) δ_H: 1.24 (9H, s, C(CH_3)$_3$), 2.41 (3H, s, CH_3), 6.91 – 6.96 (1H, m,

Ar^{2}C(6)*H*), 7.14 – 7.28 (2H, m, Ar^{2}C(4)*H* and Ar^{2}C(5)*H*), 7.37 – 7.42 (1H, m, Ar^{2}C(3)*H*), 7.42 – 7.48 (2H, m, Ar^{1}C(3,5)*H*), 7.83 – 7.90 (2H, m, Ar^{1}C(2,6)*H*), 9.56 (1H, s, N*H*), 11.86 (1H, br s, N*H*); **^{13}C{^{1}H} NMR** (126 MHz, $(CD_3)_2SO$) δ_C: 21.1 (*C*H$_3$), 30.5 (C(*C*H$_3$)$_3$), 34.6 (*C*(CH$_3$)$_3$), 126.3 (Ar^2*C*(3)H), 126.9 (Ar^2*C*(6)H), 127.6 (Ar^1*C*(2,6)H), 127.7 (Ar^2*C*(5)H), 129.6 (Ar^1*C*(3,5)H), 131.6 (Ar^2*C*(4)H), 136.2 (Ar^2*C*(1)), 136.5 (Ar^1*C*(1)), 144.2 (Ar^1*C*(4)), 145.8 (Ar^2*C*(2)), 178.9 (CS); **IR** ν_{max} (film) 3292, 2986, 2901, 2750, 1508, 1489, 1381, 1177, 1136, 1082, 918, 851, 835, 812, 760; **HRMS** (ESI^-) $C_{18}H_{21}O_2N_2S_2$ $[M-H]^-$ found 361.1047, requires 361.1050 (–0.8 ppm).

6. Preparation of Selenoureas

N-(Phenylcarbamoselenoyl)benzamide (S31)

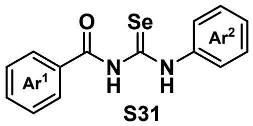

S31

Following General Procedure C, benzoyl chloride (0.58 mL, 5.0 mmol, 1.0 equiv.), potassium selenocyanate (0.72 g, 5.0 mmol, 1.0 equiv.) and aniline (0.46 mL, 5.0 mmol, 1.0 equiv.) in acetone (0.25 M) gave the crude product as an orange solid. Purification by flash silica column chromatography (hexane:EtOAc 90:10 to 50:50, R_F 0.30 at 80:20) gave the title compound as a yellow solid (0.49 g, 32%). **mp** 99 – 101 °C; **^{1}H NMR** (400 MHz, $(CD_3)_2SO$) δ_H: 7.29 – 7.36 (1H, m, Ar^{2}C(4)*H*), 7.39 – 7.49 (2H, m, Ar^{2}C(3,5)*H*), 7.51 – 7.58 (2H, m, Ar^{1}C(3,5)*H*), 7.63 – 7.72 (3H, m, Ar^{1}C(4)*H* and Ar^{2}C(2,6)*H*), 7.94 – 8.02 (2H, m, Ar^{1}C(2,6)*H*), 11.81 (1H, s, N*H*), 12.98 (1H, s, N*H*); **^{13}C{^{1}H} NMR** (126 MHz, $(CD_3)_2SO$) δ_C: 125.1 (Ar^2*C*(2,6)H), 126.9 (Ar^2*C*(4)H), 128.4 (Ar^1*C*(3,5)H), 128.7 (Ar^2*C*(3,5)H), 128.8 (Ar^1*C*(2,6)H), 131.9 (Ar^1*C*(1)), 133.2 (Ar^1*C*(4)H), 139.0 (Ar^2*C*(1)), 168.1 (*C*Se), 180.5 (*C*O); **^{77}Se{^{1}H} NMR** (95 MHz, $CDCl_3$) δ_{Se}: 415.6 (s, C*Se*); **IR** ν_{max} (film) 3237, 2957, 1668, 1597, 1558, 1520, 1497, 1483, 1447, 1356, 1260, 1130, 1076, 1061, 1024, 905, 835, 795; **HRMS** (ESI^+) $C_{14}H_{12}ON_2Na^{80}Se$ $[M+Na]^+$ found 327.0011, requires 327.0007 (+1.2 ppm).

4-Methoxy-*N*-(phenylcarbamoselenoyl)benzamide (S32)

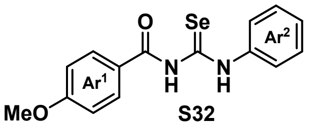

S32

Following General Procedure C, 4-methoxybenzoyl chloride (0.41 mL, 3.0 mmol, 1.0 equiv.), potassium selenocyanate (0.43 g, 3.0 mmol, 1.0 equiv.) and aniline (0.27 mL, 3.0 mmol, 1.0 equiv.) in acetone (0.25 M) gave the crude product as a brown solid. Purification by flash silica column chromatography (hexane:CH_2Cl_2 80:20 to 0:100, R_F 0.50 at 0:100) gave the title compound as a yellow solid (0.81 g, 81%). **mp** 118 – 120 °C; **^{1}H NMR** (400 MHz, $CDCl_3$) δ_H: 3.88 (3H, s, C*H*$_3$), 6.96 – 7.03 (2H, m, Ar^{1}C(3,5)*H*), 7.29 – 7.36 (1H, m, Ar^{2}C(4)*H*), 7.39 – 7.46 (2H, m, Ar^{2}C(3,5)*H*), 7.68 – 7.73 (2H, m, Ar^{2}C(2,6)*H*), 7.83 – 7.89 (2H, m, Ar^{1}C(2,6)*H*), 9.39 (1H, s, N*H*), 13.08 (1H, s, N*H*); **^{13}C{^{1}H} NMR** (126 MHz, $CDCl_3$) δ_C: 55.8 (CH_3), 114.6 (Ar^1*C*(3,5)H), 123.1 (Ar^1*C*(1)), 124.8 (Ar^2*C*(2,6)H), 127.6 (Ar^2*C*(4)H), 129.0 (Ar^2*C*(3,5)H), 129.9 (Ar^1*C*(2,6)H), 138.6 (Ar^2*C*(1)), 164.3 (Ar^1*C*(4)), 166.4 (*C*Se), 180.1 (*C*O); **^{77}Se{^{1}H} NMR** (95 MHz, $CDCl_3$) δ_{Se}: 394.3 (s, C*Se*); **IR** ν_{max} (film) 3264, 2940, 1657, 1589, 1522, 1491, 1447, 1312, 1256, 1177, 1157, 1136, 1115, 1026, 843, 804; **HRMS** (ESI^+) $C_{15}H_{14}O_2N_2Na^{80}Se$ $[M+Na]^+$ found 357.0113, requires 357.0113 (±0.0 ppm).

4-Bromo-*N*-(phenylcarbamoselenoyl)benzamide (S33)

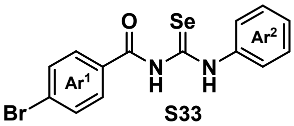

S33

Following General Procedure C, 4-bromobenzoyl chloride (0.66 g, 3.0 mmol, 1.0 equiv.), potassium selenocyanate (0.43 g, 3.0 mmol, 1.0 equiv.) and aniline (0.27 mL, 3.0 mmol, 1.0 equiv.) in acetone (0.25 M) gave the crude product as a beige solid. Purification by flash silica column chromatography (hexane:CH_2Cl_2 80:20 to 0:100, R_F 0.59 at 25:75) gave the title compound as a yellow solid (0.53 g, 46%). **mp** 115 – 117 °C; **^{1}H NMR** (400 MHz, $CDCl_3$) δ_H: 7.32 – 7.38 (1H, m, Ar^{2}C(4)*H*), 7.41 – 7.48 (2H, m, Ar^{2}C(3,5)*H*), 7.67 – 7.73 (4H, m, Ar^{1}C(3,5)*H* and Ar^{2}C(2,6)*H*), 7.75 – 7.80 (2H, m, Ar^{1}C(2,6)*H*), 9.38 (1H, s, N*H*), 12.92 (1H, s, N*H*); **^{13}C{^{1}H} NMR** (126 MHz, $CDCl_3$) δ_C: 124.8 (Ar^{2}C(2,6)H), 127.8 (Ar^{2}C(4)H), 129.2 (Ar^1*C*(3,5)H), 129.2 (Ar^2*C*(3,5)H), 129.4 (Ar^1*C*(4)), 132.8 (Ar^1*C*(2,6)H), 130.2 (Ar^1*C*(1)),

138.5 (Ar²C(1)), 166.1 (CSe), 180.0 (CO); **^{77}Se{^{1}H} NMR** (95 MHz, $CDCl_3$) δ_{Se}: 414.3 (s, C*Se*);

IR ν_{max} (film) 3358, 2972, 1672, 1589, 1508, 1394, 1327, 1256, 1146, 1130, 1109, 1072, 1009, 910, 837; **HRMS** (ESI^+) $C_{14}H_{11}ON_2{}^{79}BrNa^{80}Se$ $[M+Na]^+$ found 404.9107, requires 404.9112 (–1.2 ppm).

4-Nitro-*N*-(phenylcarbamoselenoyl)benzamide (S34)

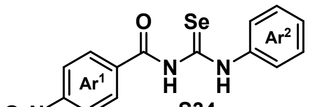

S34

Following General Procedure C, 4-nitrobenzoyl chloride (0.56 g, 3.0 mmol, 1.0 equiv.), potassium selenocyanate (0.43 g, 3.0 mmol, 1.0 equiv.) and aniline (0.27 mL, 3.0 mmol, 1.0 equiv.) in acetone (0.25 M) gave the crude product as an orange solid. Purification by flash silica column chromatography (CH_2Cl_2:Et_2O 100:0 to 90:10, R_F 0.48 at 100:0) gave the title compound as a yellow solid (0.57 g, 55%). **mp** 123 – 125 °C; **^{1}H NMR** (400 MHz, $CDCl_3$) δ_H: 7.34 – 7.40 (1H, m, Ar²C(4)*H*), 7.42 – 7.49 (2H, m, Ar²C(3,5)*H*), 7.67 – 7.73 (2H, m, Ar²C(2,6)*H*), 8.07 – 8.14 (2H, m, Ar¹C(2,6)*H*), 8.36 – 8.42 (2H, m, Ar¹C(3,5)*H*), 9.62 (1H, s, N*H*), 12.81 (1H, s, N*H*); **^{13}C{^{1}H} NMR** (126 MHz, $CDCl_3$) δ_C: 124.5 (Ar¹C(3,5)H), 124.8 (Ar²C(2,6)H), 128.0 (Ar²C(4)H), 129.1 (Ar²C(3,5)H), 129.3 (Ar¹C(2,6)H), 136.9 (Ar¹C(1)), 138.3 (Ar²C(1)), 150.9 (Ar¹C(4)), 165.1 (CSe), 179.8 (CO); **^{77}Se{^{1}H} NMR** (95 MHz, $CDCl_3$) δ_{Se}: 430.8 (s, C*Se*); **IR** ν_{max} (film) 3227, 2988, 1668, 1593, 1553, 1516, 1447, 1339, 1321, 1261, 1132, 1113, 1013, 868, 831, 795, 764; **HRMS** (ESI^+) $C_{14}H_{11}O_3N_3Na^{80}Se$ $[M+Na]^+$ found 371.9858, requires 371.9858 (±0.0 ppm).

***N*-((4-Methoxyphenyl)carbamoselenoyl)benzamide (S35)**

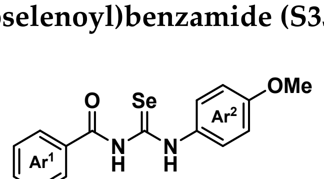

S35

Following General Procedure C, benzoyl chloride (0.35 mL, 3.0 mmol, 1.0 equiv.), potassium selenocyanate (0.43 g, 3.0 mmol, 1.0 equiv.) and *p*-anisidine (0.43 g, 3.0 mmol, 1.0 equiv.) in acetone (0.25 M) gave the crude product as an orange solid. Purification by

flash silica column chromatography (hexane:CH_2Cl_2 50:50 to 0:100, R_F 0.43 at 0:100) gave the title compound as a yellow solid (0.64 g, 64%). **mp** 110 – 112 °C; **^{1}H NMR** (500 MHz, $CDCl_3$) δ_H: 3.84 (3H, s, OC*H*$_3$), 6.94 – 6.98 (2H, m, Ar^{2}C(3,5)*H*), 7.53 – 7.60 (4H, m, Ar^{1}C(3,5)*H* and Ar^{2}C(2,6)*H*), 7.65 – 7.69 (1H, m, Ar^{1}C(4)*H*), 7.88 – 7.92 (2H, m, Ar^{1}C(2,6)*H*), 9.42 (1H, br s, N*H*), 12.84 (1H, br s, N*H*); **^{13}C{^{1}H} NMR** (126 MHz, $CDCl_3$) δ_C: 55.6 (O*C*H$_3$), 114.3 (Ar^2*C*(3,5)H), 126.4 (Ar^1*C*(3,5)H), 127.7 (Ar^1*C*(2,6)H), 129.4 (Ar^2*C*(2,6)H), 131.4 (Ar^1*C*(1)), 131.5 (Ar^2*C*(1)), 134.0 (Ar^1*C*(4)), 158.8 (Ar^2*C*(4)), 167.0 (*C*Se), 180.3 (*C*O); **^{77}Se{^{1}H} NMR** (95 MHz, $CDCl_3$) δ_{Se}: 391.1 (s, C*Se*); **IR** ν_{max} (film) 3136, 1668, 1599, 1506, 1487, 1460, 1445, 1331, 1298, 1246, 1161, 1138, 1103, 1024, 826; **HRMS** (ESI^+) $C_{15}H_{14}O_2N_2Na^{80}Se$ $[M + Na]^+$ found 357.0110, requires 357.0113 (–0.8 ppm).

N-((4-(N-(5-Methylisoxazol-3-yl)sulfamoyl)phenyl)carbamoselenoyl)benzamide (S36)

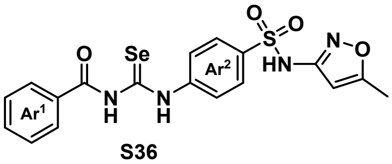

S36

Following General Procedure C, benzoyl chloride (0.35 mL, 3.0 mmol, 1.0 equiv.), potassium selenocyanate (0.43 g, 3.0 mmol, 1.0 equiv.) and sulfamethoxazole (0.76 g, 3.0 mmol, 1.0 equiv.) in acetone (0.25 M) gave the crude product as an orange solid. Recrystallisation from EtOH gave the title compound as a green solid (0.41 g, 29%). **mp** 146 – 148 °C; **^{1}H NMR** (400 MHz, $(CD_3)_2SO$) δ_H: 2.31 (3H, s, C*H*$_3$), 6.18 (1H, s, C*H*), 7.51 – 7.59 (2H, m, Ar^{1}C(3,5)*H*), 7.64 – 7.71 (1H, m, Ar^{1}C(4)*H*), 7.86 – 7.92 (2H, m, Ar^{2}C(3,5)*H*), 7.93 – 7.99 (4H, m, Ar^{1}C(2,6)*H* and Ar^{2}C(2,6)*H*), 11.51 (1H, br s, N*H*), 11.96 (1H, br s, N*H*), 13.02 (1H, br s, N*H*); **^{13}C{^{1}H} NMR** (126 MHz, $(CD_3)_2SO$) δ_C: 12.1 (*C*H$_3$), 95.4 (*C*H), 125.6 (Ar^2*C*(2,6)H), 127.4 (Ar^2*C*(3,5)H), 128.4 (Ar^1*C*(3,5)H), 128.9 (Ar^1*C*(2,6)H), 131.8 (Ar^1*C*(1)), 133.3 (Ar^1*C*(4)H), 137.0 (Ar^2*C*(4)), 143.3 (Ar^2*C*(1)), 157.5 (*C*=N), 167.9 (*C*Se), 170.4 (*C*-O), 181.0 (*C*O); **^{77}Se{^{1}H} NMR** (95 MHz, $CDCl_3$) δ_{Se}: 444.1 (s, C*Se*); **IR** ν_{max} (film) 3231, 3154, 2974, 1682, 1609, 1516, 1506, 1472, 1387, 1317, 1250, 1163, 1090, 1065, 930, 905, 858, 791; **HRMS** (ESI^+) $C_{18}H_{16}O_4N_4NaS^{80}Se$ $[M + Na]^+$ found 486.9949, requires 486.9950 (–0.2 ppm).

***N*-((2-*iso*-Propylphenyl)carbamoselenoyl)benzamide (S37)**

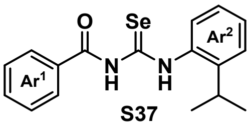

S37

Following General Procedure C, benzoyl chloride (0.35 mL, 3.0 mmol, 1.0 equiv.), potassium selenocyanate (0.43 g, 3.0 mmol, 1.0 equiv.) and 2-*iso*-propylaniline (0.42 mL, 3.0 mmol, 1.0 equiv.) in acetone (0.25 M) gave the crude product as a brown solid. Purification by flash silica column chromatography (hexane:CH_2Cl_2 80:20 to 0:100, R_F 0.53 at 25:75) gave the title compound as a yellow solid (0.61 g, 59%). **mp** 109 – 111 °C; **^{1}H NMR** (400 MHz, $CDCl_3$) δ_H: 1.29 (6H, d, *J* 6.9, CH(C*H*$_3$)$_2$), 3.14 (1H, h, *J* 6.9, C*H*(CH$_3$)$_2$), 7.27 – 7.32 (1H, m, Ar^{2}C(5)*H*), 7.36 – 7.42 (2H, m, Ar^{2}C(3,4)*H*), 7.54 – 7.60 (3H, m, Ar^{1}C(3,5)*H* and Ar^{2}C(6)*H*), 7.66 – 7.70 (1H, m, Ar^{1}C(4)*H*), 7.91 – 7.96 (2H, m, Ar^{1}C(2,6)*H*), 9.52 (1H, s, N*H*), 12.66 (1H, s, N*H*); **^{13}C{^{1}H} NMR** (126 MHz, $CDCl_3$) δ_C: 23.4 (CH(*C*H$_3$)$_2$), 28.7 (*C*H(CH$_3$)$_2$), 126.4 (Ar^2*C*(3)H), 126.5 (Ar^2*C*(5)H), 127.7 (Ar^2*C*(6)H), 127.8 (Ar^{1}C(2,6)H), 128.9 (Ar^2*C*(4)H), 129.4 (Ar^1*C*(3,5)H), 131.4 (Ar^1*C*(1)), 134.1 (Ar^{1}C(4)H), 136.5 (Ar^2*C*(1)), 144.0 (Ar^2*C*(2)), 167.1 (*C*Se), 182.2 (*C*O); **^{77}Se{^{1}H} NMR** (95 MHz, $CDCl_3$) δ_{Se}: 397.6 (s, C*Se*); **IR** ν_{max} (film) 3181, 2953, 1659, 1533, 1508, 1483, 1317, 1248, 1198, 1140, 1082, 1026, 756; **HRMS** (ESI^+) $C_{17}H_{18}ON_2Na^{80}Se$ $[M + Na]^+$ found 369.0475, requires 369.0477 (–0.5 ppm).

7. Preparation of Ureas

1,3-Bis(3,5-bis(trifluoromethyl)phenyl)urea (S38)

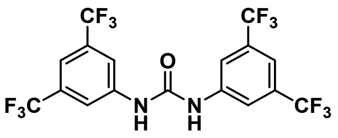

S38

3,5-Bis(trifluoromethyl)phenyl isocyanate (0.52 mL, 3.0 mmol, 1.0 equiv.), 3,5-bis(trifluoromethyl)aniline (0.56 mL, 3.6 mmol, 1.2 equiv.), and NEt_3 (0.84 mL, 6.0 mmol, 2.0 equiv.) were dissolved in CH_2Cl_2 (0.1 M) and stirred at RT for 4 h. Once complete, the precipitate was collected by vacuum filtration washing with CH_2Cl_2 to give the title compound as a white solid (0.63 g, 43%). **mp** 193 – 195 °C {lit[11] 236 – 238}; **^{1}H NMR** (400 MHz, CD_3CN) δ_H: 7.65 (2H, s, ArC(4)*H*), 7.99 (2H, br s, N*H*), 8.08 (4H, s, ArC(2,6)*H*); **^{19}F{^{1}H} NMR** (376 MHz, CD_3CN) δ_F: –63.6 (s, CF_3).

Spectroscopic data in accordance with literature.[11]

4-Methyl-*N*-(phenylcarbamoyl)benzenesulfonamide (S39)

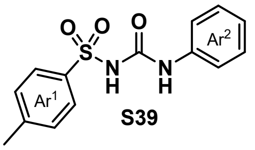

S39

p-Toluenesulfonamide (0.86 g, 5.0 mmol, 1.0 equiv.), phenyl isocyanate (0.54 mL, 5.0 mmol, 1.0 equiv.), and Cs_2CO_3 (1.63 g, 5.0 mmol, 1.0 equiv.) were dissolved in acetone (0.25 M) and heated to reflux for 4 h. Once complete, the reaction mixture was cooled to RT, diluted with H_2O and acidified to pH 1. The reaction mixture was extracted with EtOAc (× 3), the combined organics dried over $MgSO_4$, and the solvent removed under reduced pressure to give the crude product as an off-white solid. Recrystallisation from heptane/EtOAc gave the title compound as white crystals (0.20 g, 14%). **mp** 128 – 130 °C; **^{1}H NMR** (400 MHz, $CDCl_3$) δ_H: 2.43 (3H, s, CH_3), 7.10 – 7.17 (1H, m, Ar^{2}C(4)*H*), 7.29 – 7.41 (7H, m, Ar^{1}C(3,5)*H*, Ar^{2}C(2,3,5,6)*H* and N*H*), 7.80 – 7.85 (2H, m, Ar^{1}C(2,6)*H*), 8.44 (1H, br s, N*H*).

Spectroscopic data in accordance with literature.[14]

8. Isothiourea Catalysis Products

(*R*,*Z*)-3-(3,5-Bis(trifluoromethyl)phenyl)-2-((3,5-bis(trifluoromethyl)phenyl)imino)-6-(trifluoromethyl)-1,3-thiazinan-4-one (33)

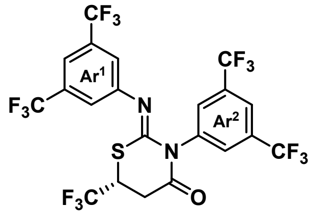

33

Following General Procedure G, perfluorophenyl (*E*)-4,4,4-trifluorobut-2-enoate **28** (138 mg, 0.45 mmol, 1.5 equiv.), 1,3-bis(3,5-bis(trifluoromethyl)phenyl)thiourea **26** (150 mg, 0.30 mmol, 1.0 equiv.) and (2*S*,3*R*)-HyperBTM **21** (4.6 mg, 0.015 mmol, 5 mol%) in anhydrous THF (0.1 M) for 24 h at 0 °C gave crude product that was purified by flash silica column chromatography (hexane:CH_2Cl_2 90:10 to 25:75) to give the title compound as a white solid (178 mg, 95%). **mp** 140 – 142 °C; $[\alpha]_D^{20}$ +2.8 (*c* 1.0 in $CHCl_3$); **Chiral HPLC analysis,** Chiralcel OD-H (90:10 *n*-hexane : IPA, flow rate 1.0 mLmin^{-1}, 254 nm, 30 °C) t_R (*S*) 4.7 min, t_R (*R*) 5.3 min, 95:5 er; **IR** ν_{max} (film) 2976, 1715, 1607, 1460, 1373, 1277, 1169, 1132, 1123, 1105, 1005, 908, 880; **^{1}H NMR** (400 MHz, $CDCl_3$) δ_H: 3.46 (1H, dd, *J* 17.2, 5.4, C(5)H^AH^B), 3.55 (1H, dd, *J* 17.2, 4.2, C(5)H^AH^B), 3.92 (1H, qdd, *J* 7.8, 5.4, 4.2, C(6)*H*), 7.23 (2H, s, Ar^1C(2,6)*H*), 7.66 (1H, s, Ar^1C(4)*H*), 7.69 (2H, s, Ar^2C(2,6)*H*), 7.97 (1H, s, Ar^2C(4)*H*); **^{19}F{^{1}H} NMR** (376 MHz, $CDCl_3$) δ_F: –71.4 (s, C*F*$_3$), –63.0 (s, C*F*$_3$), –62.8 (s, C*F*$_3$); **^{13}C{^{1}H} NMR** (126 MHz, $CDCl_3$) δ_C: 33.7 (*C*(5)H_2), 39.4 (q, *J* 32.8, *C*(6)H), 118.5 – 118.7 (m, Ar^1C(4)H), 120.8 – 121.0 (m, Ar^1C(2,6)H), 122.7 (q, *J* 273.0, CF_3), 122.9 (q, *J* 272.9, CF_3), 122.9 – 123.2 (m, Ar^2*C*(4)H), 124.5 (q, *J* 280.0, *C*F_3), 129.3 – 129.5 (m, Ar^2*C*(2,6)H), 132.3 – 133.5 (m, Ar^1*C*(3,5) and Ar^2*C*(3,5)), 138.2 (Ar^2C(1)), 147.4 (Ar^1C(1)), 150.2 (*C*(2)), 165.6 (*C*(4)); **HRMS** (ESI^+) $C_{21}H_{10}ON_2F_{15}S$ $[M+H]^+$ found 623.0274, requires 623.0269 (+0.8 ppm).

(*R*,*Z*)-6-(Trifluoromethyl)-3-(4-(trifluoromethyl)phenyl)-2-((4-(trifluoromethyl)phenyl)imino)-1,3-thiazinan-4-one (35)

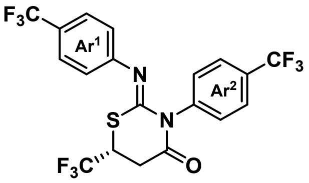

35

Following General Procedure G, perfluorophenyl (*E*)-4,4,4-trifluorobut-2-enoate **28** (138 mg, 0.45 mmol, 1.5 equiv.), 1,3-bis(4-(trifluoromethyl)phenyl)thiourea **S17** (109 mg, 0.30 mmol, 1.0 equiv.) and (2*S*,3*R*)-HyperBTM **21** (18.5 mg, 0.06 mmol, 20 mol%) in anhydrous THF (0.1 M) for 24 h at RT gave crude product that was purified by flash silica column chromatography (hexane:CH_2Cl_2 80:20 to 0:100, R_F 0.68 at 0:100) to give the title compound as white solid (115 mg, 79%). **mp** 99 – 101 °C; $[\alpha]_D^{20}$ +29.2 (*c* 1.0 in $CHCl_3$); **Chiral HPLC analysis,** Chiralpak AD-H (97:3 *n*-hexane : IPA, flow rate 2.0 mLmin^{-1}, 220 nm, 40 °C) t_R (*R*) 9.1 min, t_R (*S*) 14.5 min, 95:5 er; **IR** ν_{max} (film) 2359, 1705, 1603, 1364, 1323, 1163, 1119, 1105, 1065, 1015, 891, 841; **^{1}H NMR** (400 MHz, $CDCl_3$) δ_H: 3.41 (1H, dd, *J* 17.0, 5.4, C(5)H^AH^B), 3.49 (1H, dd, *J* 17.0, 4.3, C(5)H^AH^B), 3.92 (1H, qdd, *J* 7.9, 5.4, 4.3, C(6)*H*), 6.84 – 6.91 (2H, m, Ar^{1}C(2,6)*H*), 7.35 – 7.42 (2H, m, Ar^{2}C(2,6)*H*), 7.55 – 763 (2H, m, Ar^{1}C(3,5)*H*), 7.74 – 7.81 (2H, m, Ar^{2}C(3,5)*H*); **^{19}F{^{1}H} NMR** (376 MHz, $CDCl_3$) δ_F: –71.4 (s, C*F*$_3$), –62.7 (s, C*F*$_3$), –62.1 (s, C*F*$_3$); **^{13}C{^{1}H} NMR** (126 MHz, $CDCl_3$) δ_C: 33.9 (*C*(5)H_2), 39.4 (q, *J* 32.4, *C*(6)H), 120.5 (Ar^1*C*(2,6)H), 123.7 (q, *J* 272.4, *C*F$_3$), 124.1 (q, *J* 271.7, *C*F$_3$), 124.7 (q, *J* 279.9, *C*F$_3$), 126.5 (q, *J* 4.1 Ar^{1}C(3,5)H), 126.6 (q, *J* 4.1 Ar^{2}C(3,5)H), 126.9 (q, *J* 32.7, Ar^1*C*(4)), 129.2 (Ar^2*C*(2,6)H), 130.8 (q, *J* 32.8, Ar^{2}C(4)), 140.4 (Ar^2*C*(1)), 148.7 (*C*(2)), 149.7 (Ar^1*C*(1)), 165.8 (*C*(4)); **HRMS** (ESI^-) $C_{19}H_{10}ON_2F_9S$ $[M-H]^-$ found 485.0369, requires 485.0376 (–1.4 ppm).

(*R*,*Z*)-3-(4-Nitrophenyl)-2-((4-nitrophenyl)imino)-6-(trifluoromethyl)-1,3-thiazinan-4-one (36)

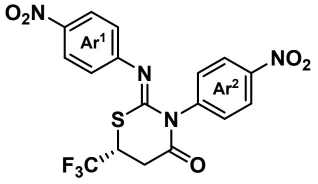

36

Following General Procedure G, perfluorophenyl (*E*)-4,4,4-trifluorobut-2-enoate **28** (138 mg, 0.45 mmol, 1.5 equiv.), 1,3-bis(4-nitrophenyl)thiourea **S18** (95.5 mg, 0.30 mmol, 1.0 equiv.) and (2*S*,3*R*)-HyperBTM **21** (4.6 mg, 0.015 mmol, 5 mol%) in anhydrous THF (0.1 M) for 24 h at 0 °C gave crude product that was purified by flash silica column chromatography (CH_2Cl_2:Et_2O 100:0 to 90:10, R_F 0.71 at 90:10) to give the title compound as a pale yellow solid (125 mg, 95%). **mp** 72 – 74 °C; $[\alpha]_D^{20}$ +66.4 (*c* 1.1 in $CHCl_3$); **Chiral HPLC analysis,** Chiralpak AD-H (70:30 *n*-hexane : IPA, flow rate 1.0 $mLmin^{-1}$, 211 nm, 30 °C) t_R (*R*) 13.1 min, t_R (*S*) 24.7 min, 98:2 er; **IR** ν_{max} (film) 3078, 2953, 1709, 1612, 1582, 1510, 1337, 1296, 1225, 1157, 1109, 893, 856, 843; **^{1}H NMR** (400 MHz, $CDCl_3$) δ_H: 3.47 (1H, dd, *J* 17.2, 5.3, C(5)H^AH^B), 3.53 (1H, dd, *J* 17.2, 4.4, C(5)H^AH^B), 4.00 (1H, qdd, *J* 7.9, 5.3, 4.4, C(6)*H*), 6.87 – 6.92 (2H, m, $Ar^1C(2,6)H$), 7.41 – 7.46 (2H, m, $Ar^2C(2,6)H$), 8.18 – 8.23 (2H, m, $Ar^1C(3,5)H$), 8.34 – 8.39 (2H, m, $Ar^2C(3,5)H$); **^{19}F{^{1}H} NMR** (377 MHz, $CDCl_3$) δ_F: –71.4 (s, CF_3); **^{13}C{^{1}H} NMR** (126 MHz, $CDCl_3$) δ_C: 33.7 (*C*(5)H_2), 39.5 (q, *J* 32.7, *C*(6)H), 120.9 ($Ar^1C(2,6)$H), 124.6 (q, *J* 280.1, CF_3), 124.9 ($Ar^2C(3,5)$H), 125.3 ($Ar^1C(3,5)$H), 129.9 ($Ar^2C(2,6)$H), 142.8 ($Ar^2C(1)$), 144.8 ($Ar^1C(4)$), 147.7 ($Ar^2C(4)$), 149.1 (*C*(2)), 152.2 ($Ar^1C(1)$), 165.6 (*C*(4)); **HRMS** (ESI^+) $C_{17}H_{11}O_5N_4F_3NaS$ $[M + Na]^+$ found 463.0290, requires 463.0294 (–0.9 ppm).

(*R*,*Z*)-4-((3-(4-Cyanophenyl)-4-oxo-6-(trifluoromethyl)-1,3-thiazinan-2-ylidene)amino)benzonitrile (37)

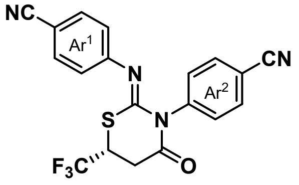

37

Following General Procedure G, perfluorophenyl (*E*)-4,4,4-trifluorobut-2-enoate **28** (138 mg, 0.45 mmol, 1.5 equiv.), 1,3-bis(4-cyanophenyl)thiourea **S19** (83.5 mg, 0.3 mmol, 1.0 equiv.) and (2*S*,3*R*)-HyperBTM **21** (4.6 mg, 0.015 mmol, 5 mol%) in anhydrous THF (0.1 M) for 24 h at 0 °C gave crude product that was purified by flash silica column chromatography (hexane:EtOAc 100:0 to 50:50, R_F 0.32 at 50:50) to give the title compound as a white solid (100 mg, 83%). **mp** 149 – 151 °C; $[\alpha]_D^{20}$ +64.7 (*c* 1.0 in $CHCl_3$); **Chiral HPLC analysis,** Chiralpak AD-H (70:30 *n*-hexane : IPA, flow rate 1.0 $mLmin^{-1}$, 211 nm, 30 °C) t_R (*R*) 9.8 min, t_R (*S*) 16.5 min, 97:3 er; **IR** ν_{max} (film) 2951, 2228, 1709, 1618, 1593, 1499, 1364, 1227, 1179, 1161, 1119, 889, 841; **^{1}H NMR** (400 MHz, $CDCl_3$) δ_H: 3.42 (1H, dd, *J* 17.1, 5.4, C(5)H^AH^B), 3.49 (1H, dd, *J* 17.1, 4.2, C(5)H^AH^B), 3.96 (1H, qdd, *J* 7.9, 5.4, 4.2, C(6)*H*), 6.82 – 6.88 (2H, m, $Ar^1C(2,6)H$), 7.34 – 7.38 (2H, m, $Ar^2C(2,6)H$), 7.58 – 763 (2H, m, $Ar^1C(3,5)H$), 7.76 – 7.81 (2H, m, $Ar^2C(3,5)H$); **^{19}F{^{1}H} NMR** (376 MHz, $CDCl_3$) δ_F: –71.4 (s, CF_3); **^{13}C{^{1}H} NMR** (126 MHz, $CDCl_3$) δ_C: 33.8 ($C(5)H_2$), 39.4 (q, *J* 32.6, *C*(6)H), 108.4 ($Ar^1C(4)$), 112.9 ($Ar^2C(4)$), 118.0 (Ar^2CN), 118.8 (Ar^1CN), 121.2 ($Ar^1C(2,6)H$), 124.6 (q, *J* 280.0, CF_3), 129.8 ($Ar^2C(2,6)H$), 133.3 ($Ar^2C(3,5)H$), 133.5 ($Ar^1C(3,5)H$), 141.2 ($Ar^2C(1)$), 148.9 (*C*(2)), 150.4 ($Ar^1C(1)$), 165.6 (*C*(4)); **HRMS** (ESI^+) $C_{19}H_{11}ON_4F_3NaS$ $[M+Na]^+$ found 423.0489, requires 423.0498 (–2.1 ppm).

(*R*,*Z*)-6-(Chlorodifluoromethyl)-3-(4-nitrophenyl)-2-((4-nitrophenyl)imino)-1,3-thiazinan-4-one (38)

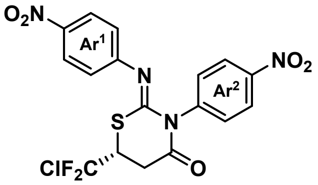

38

Following General Procedure G, 4-nitrophenyl (*E*)-4-chloro-4,4-difluorobut-2-enoate **S4** (125 mg, 0.45 mmol, 1.5 equiv.), 1,3-bis(4-nitrophenyl)thiourea **S18** (95.5 mg, 0.30 mmol, 1.0 equiv.) and (2*S*,3*R*)-HyperBTM **21** (4.6 mg, 0.015 mmol, 5 mol%) in anhydrous THF (0.1 M) at 0 °C for 48 h, without workup, gave crude product that was purified by flash silica column chromatography (CH_2Cl_2:hexane 50:50 to 100:0, R_F 0.43 at 100:0 then EtOAc:hexane 30:70 to 70:30, R_F 0.38 at 50:50) to give the title compound as a pale yellow solid (107 mg, 78%). *Note: a workup was not performed to avoid elimination of HCl.*

mp 55 – 57 °C; $[\alpha]_D^{20}$ +104.5 (*c* 1.0 in $CHCl_3$); **Chiral HPLC analysis,** Chiralpak AD-H (70:30 *n*-hexane : IPA, flow rate 1.0 mLmin^{-1}, 211 nm, 30 °C) t_R (*R*) 15.3 min, t_R (*S*) 23.5 min, 98:2 er; **IR** ν_{max} (film) 1711, 1616, 1584, 1516, 1342, 1300, 1157, 1109, 858; **^{1}H NMR** (500 MHz, $CDCl_3$) δ_H: 3.48 (1H, dd, *J* 17.2, 5.5, C(5)H^AH^B), 3.59 (1H, dd, *J* 17.2, 4.4, C(5)H^AH^B), 4.05 – 4.15 (1H, m, C(6)*H*), 6.83 – 6.92 (2H, m, Ar^{1}C(2,6)*H*), 7.36 – 7.46 (2H, m, Ar^{2}C(2,6)*H*), 8.16 – 8.22 (2H, m, Ar^{1}C(3,5)*H*), 8.30 – 8.39 (2H, m, Ar^{2}C(3,5)*H*); **^{19}F{^{1}H} NMR** (377 MHz, $CDCl_3$) δ_F: –58.1 (d, *J* 167.1, CF^AF^BCl), –56.2 (d, *J* 167.1, CF^AF^BCl); **^{13}C{^{1}H} NMR** (126 MHz, $CDCl_3$) δ_C: 34.8 (*C*(5)H_2), 39.5 (t, *J* 27.7, *C*(6)H), 121.0 (Ar^1*C*(2,6)H), 124.9 (Ar^2*C*(3,5)H), 125.4 (Ar^1*C*(3,5)H), 128.0 (t, *J* 294.7, *C*F_2Cl), 130.0 (Ar^2*C*(2,6)H), 142.9 (Ar^2*C*(1)), 144.8 (Ar^1*C*(4)), 147.8 (Ar^2*C*(4)), 149.3 (*C*(2)), 152.3 (Ar^1*C*(1)), 165.8 (*C*(4)); **HRMS** (ESI^+) $C_{17}H_{11}O_5N_4{}^{35}ClF_2NaS$ $[M + Na]^+$ found 478.9987, requires 478.9999 (–2.5 ppm).

(*R*,*Z*)-3-(4-Nitrophenyl)-2-((4-nitrophenyl)imino)-6-(perfluoroethyl)-1,3-thiazinan-4-one (39) and (*Z*)-3-(4-nitrophenyl)-2-((4-nitrophenyl)imino)-5-(2,2,3,3,3-pentafluoropropyl)thiazolidin-4-one (S40)

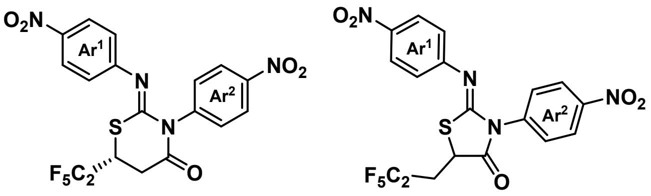

39 **S40**

Following General Procedure G, perfluorophenyl (*E*)-4,4,5,5,5-pentafluoropent-2-enoate **S11** (160 mg, 0.45 mmol, 1.5 equiv.), 1,3-bis(4-nitrophenyl)thiourea **S18** (95.5 mg, 0.30 mmol, 1.0 equiv.) and (2*S*,3*R*)-HyperBTM **21** (4.6 mg, 0.015 mmol, 5 mol%) in anhydrous THF (0.1 M) for 48 h at 0 °C gave crude product that was purified by flash silica column chromatography (CH_2Cl_2:hexane 30:70 to 100:0) to give:

39: (86 mg, 59%) as a white solid. **mp** 93 – 95 °C; $[\alpha]_D^{20}$ +79.4 (*c* 1.0 in $CHCl_3$); **Chiral HPLC analysis,** Chiralpak AD-H (80:20 *n*-hexane : IPA, flow rate 1.0 mLmin^{-1}, 211 nm, 30 °C) t_R (*R*) 16.9 min, t_R (*S*) 25.2 min, 98:2 er; **IR** ν_{max} (film) 3082, 2953, 1713, 1612, 1582, 1510, 1491, 1337, 1296, 1198, 1153, 1107, 1030, 856, 843; **^{1}H NMR** (500 MHz, $CDCl_3$) δ_H: 3.50 (1H, dd, *J* 17.2, 5.1, C(5)H^AH^B), 3.54 (1H, dd, *J* 17.2, 4.6, C(5)H^AH^B), 3.98 – 4.09 (1H, m, C(6)*H*), 6.84 – 6.90 (2H, m, Ar^{1}C(2,6)*H*), 7.39 – 7.46 (2H, m, Ar^{2}C(2,6)*H*), 8.16 – 8.21 (2H, m, Ar^{1}C(3,5)*H*), 8.32 – 8.37 (2H, m, Ar^{2}C(3,5)*H*); **^{19}F{^{1}H} NMR** (377 MHz, $CDCl_3$) δ_F: –118.2 (d, *J* 271.7, CF^AF^BCF$_3$), –115.6 (d, *J* 271.7, CF^AF^BCF$_3$), –81.0 (s, CF_3); **^{13}C{^{1}H} NMR** (126 MHz, $CDCl_3$) δ_C: 33.9 (*C*(5)H$_2$), 37.9 (t, *J* 24.4, *C*(6)H), 113.8 (tq, *J* 258.4, 37.7, *C*F$_2$CF$_3$), 118.5 (qt, *J* 287.3, 35.7, CF$_2$*C*F$_3$), 120.9 (Ar^1*C*(2,6)H), 124.9 (Ar^2*C*(3,5)H), 125.3 (Ar^1*C*(3,5)H), 129.9 (Ar^2*C*(2,6)H), 142.7 (Ar^2*C*(1)), 144.8 (Ar^1*C*(4)), 147.7 (Ar^2*C*(4)), 149.0 (*C*(2)), 152.2 (Ar^1*C*(1)), 165.6 (*C*(4)); **HRMS** (ESI^+) $C_{18}H_{12}O_5N_4F_5S$ $[M+H]^+$ found 491.0437, requires 491.0443 (–1.2 ppm).

S40: (15 mg, 10%) as a white solid. **mp** 88 – 90 °C; **Chiral HPLC analysis,** Chiralpak AD-H (70:30 *n*-hexane : IPA, flow rate 1.0 mLmin^{-1}, 211 nm, 30 °C) t_R 10.2 min, t_R 16.2 min, 50:50 er; **IR** ν_{max} (film) 2359, 1734, 1638, 1585, 1516, 1341, 1194, 1155, 1069, 858; **^{1}H NMR** (500 MHz, $CDCl_3$) δ_H: 2.61 – 2.76 (1H, m, CH^AH^B), 3.20 – 3.35 (1H, m, CH^AH^B), 4.61 (1H, dd, *J* 11.0, 2.2, C(5)*H*), 7.01 – 7.07 (2H, m, Ar^{1}C(2,6)*H*), 7.60 – 7.65 (2H, m, Ar^{2}C(2,6)*H*), 8.22 – 8.27 (2H, m, Ar^{1}C(3,5)*H*), 8.38 – 8.44 (2H, m, Ar^{2}C(3,5)*H*); **^{19}F{^{1}H} NMR** (376 MHz, $CDCl_3$)

δ_F: –118.0 (d, *J* 265.5, C*F*^{A}F^{B}CF$_3$), –116.1 (d, *J* 265.5, CF^A*F*^{B}CF$_3$), –85.4 (s, C*F*$_3$); **^{13}C{^{1}H} NMR** (126 MHz, CDCl$_3$) δ_C: 34.9 (t, *J* 20.7, *C*H$_2$C$_2$F$_5$), 40.7 (*C*(5)), 121.4 (Ar^1*C*(2,6)H), 124.8 (Ar^2*C*(3,5)H), 125.4 (Ar^{1}C(3,5)H), 129.0 (Ar^2*C*(2,6)H), 139.1 (Ar^2*C*(1)), 145.1 (Ar^1*C*(4)), 147.8 (Ar^2*C*(4)), 152.7 (*C*(2)), 153.7 (Ar^1*C*(1)), 171.2 (*C*(4)); **HRMS** (ESI^-) $C_{18}H_{10}O_5N_4F_5S$ $[M-H]^-$ found 489.0294, requires 489.0298 (–0.7 ppm).

Note: the C_2F_5 $^{13}C\{^1H\}$ NMR multiplets can be seen on the baseline but are not fully resolved.

(*R*,*Z*)-6-(Difluoromethyl)-3-(4-nitrophenyl)-2-((4-nitrophenyl)imino)-1,3-thiazinan-4-one (40)

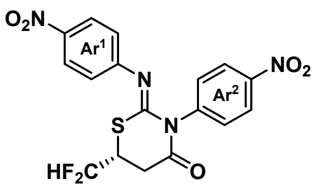

40

Following General Procedure G, perfluorophenyl (*E*)-4,4-difluorobut-2-enoate **S12** (130 mg, 0.45 mmol, 1.5 equiv.), 1,3-bis(4-nitrophenyl)thiourea **S18** (95.5 mg, 0.30 mmol, 1.0 equiv.) and (2*S*,3*R*)-HyperBTM **21** (4.6 mg, 0.015 mmol, 5 mol%) in anhydrous THF (0.1 M) for 24 h at 0 °C gave crude product that was purified twice by flash silica column chromatography (CH_2Cl_2:Et_2O 100:0 to 95:5, R$_F$ 0.49 at 95:5 then hexane:EtOAc 100:0 to 0:100, R$_F$ 0.28 at 50:50) to give the title compound as a pale yellow solid (96 mg, 76%). **mp** 133 – 135 °C; $[\alpha]_D^{20}$ +52.0 (*c* 1.0 in CHCl$_3$); **Chiral HPLC analysis,** Chiralpak AD-H (70:30 *n*-hexane : IPA, flow rate 1.0 mLmin^{-1}, 211 nm, 30 °C) t$_R$ (*R*) 24.4 min, t$_R$ (*S*) 34.1 min, 80:20 er; **IR** ν_{max} (film) 1707, 1614, 1584, 1342, 1219, 1159, 1109, 860; **^{1}H NMR** (500 MHz, CDCl$_3$) δ_H: 3.39 (1H, dd, *J* 17.0, 5.4, C(5)*H*^{A}H^B), 3.45 (1H, dd, *J* 17.0, 4.4, C(5)H^A*H*^{B}), 3.70 – 3.82 (1H, m, C(6)*H*), 6.10 (1H, td, *J* 55.2, 2.2, CF$_2$*H*), 6.86 – 6.92 (2H, m, Ar^{1}C(2,6)*H*), 7.41 – 7.47 (2H, m, Ar^{2}C(2,6)*H*), 8.17 – 8.22 (2H, m, Ar^{1}C(3,5)*H*), 8.33 – 8.39 (2H, m, Ar^{2}C(3,5)*H*); **^{19}F{^{1}H} NMR** (376 MHz, CDCl$_3$) δ_F: –123.0 (d, *J* 283.2, C*F*^{A}F^{B}H), –118.8 (d, *J* 283.2, CF^A*F*^{B}H); **^{13}C{^{1}H} NMR** (126 MHz, CDCl$_3$) δ_C: 33.1 (*C*(5)H$_2$), 39.2 (t, *J* 23.6, *C*(6)H), 114.7 (t, *J* 247.6, *C*F$_2$H), 121.0 (Ar^1*C*(2,6)H), 124.8 (Ar^2*C*(3,5)H), 125.2 (Ar^{1}C(3,5)H), 130.0 (Ar^2*C*(2,6)H), 143.2 (Ar^2*C*(1)), 144.6 (Ar^1*C*(4)), 147.6 (Ar^2*C*(4)), 150.2 (*C*(2)), 152.7 (Ar^1*C*(1)), 166.7 (*C*(4)); **HRMS** (ESI^+) $C_{17}H_{13}O_5N_4F_2S$ $[M+H]^+$ found 423.0560, requires 423.0569 (–2.2 ppm).

(*R*,*Z*)-6-(Fluoromethyl)-3-(4-nitrophenyl)-2-((4-nitrophenyl)imino)-1,3-thiazinan-4-one (41)

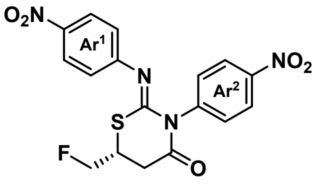

41

Following General Procedure G, perfluorophenyl (*E*)-4-fluorobut-2-enoate **S13** (122 mg, 0.45 mmol, 1.5 equiv.), 1,3-bis(4-nitrophenyl)thiourea **S18** (95.5 mg, 0.30 mmol, 1.0 equiv.) and (2*S*,3*R*)-HyperBTM **21** (9.3 mg, 0.03 mmol, 10 mol%) in anhydrous THF (0.1 M) for 48 h at 0 °C gave crude product that was purified by flash silica column chromatography (hexane:EtOAc 100:0 to 0:100, R_F 0.19 at 50:50) to give the title compound as a pale yellow solid (118 mg, 98%). **mp** 150 – 152 °C; $[\alpha]_D^{20}$ +39.0 (*c* 0.1 in MeCN); **Chiral HPLC analysis,** Chiralcel OJ-H (50:50 *n*-hexane : IPA, flow rate 0.7 mLmin^{-1}, 211 nm, 40 °C) t_R (*S*) 37.1 min, t_R (*R*) 44.5 min, 83:17 er; **IR** ν_{max} (film) 2920, 1705, 1612, 1578, 1526, 1508, 1491, 1337, 1285, 1213, 1153, 1103, 1016, 856, 839; **^{1}H NMR** (400 MHz, $(CD_3)_2CO$) δ_H: 3.24 (1H, dd, *J* 16.5, 6.9, C(5)H^AH^B), 3.41 (1H, dd, *J* 16.5, 4.4, C(5)H^AH^B), 4.03 – 4.16 (1H, m, C(6)*H*), 4.67 – 4.77 (1H, m, CH^AH^BF), 4.79 – 4.88 (1H, m, CH^AH^BF), 6.92 – 6.97 (2H, m, Ar^{1}C(2,6)*H*), 7.60 – 7.65 (2H, m, Ar^{2}C(2,6)*H*), 8.13 – 8.18 (2H, m, Ar^{1}C(3,5)*H*), 8.27 – 8.32 (2H, m, Ar^{2}C(3,5)*H*); **^{19}F{^{1}H} NMR** (376 MHz, $(CD_3)_2CO$) δ_F: –222.0 (s, CH_2F); **^{13}C{^{1}H} NMR** (126 MHz, $(CD_3)_2CO$) δ_C: 37.1 (d, *J* 3.6, *C*(5)H_2), 38.1 (d, *J* 20.1, *C*(6)H), 85.4 (d, *J* 173.3, CH_2F), 122.0 (Ar^1*C*(2,6)H), 124.8 (Ar^2*C*(3,5)H), 125.7 (Ar^1*C*(3,5)H), 131.4 (Ar^2*C*(2,6)H), 144.9 (Ar^2*C*(1)), 145.4 (Ar^{1}C(4)), 148.1 (Ar^2*C*(4)), 153.7 (*C*(2)), 154.8 (Ar^{1}C(1)), 168.9 (*C*(4)); **HRMS** (ESI^+) $C_{17}H_{14}O_5N_4FS$ $[M+H]^+$ found 405.0655, requires 405.0663 (–2.0 ppm).

(*S*,*Z*)-6-Methyl-3-(4-nitrophenyl)-2-((4-nitrophenyl)imino)-1,3-thiazinan-4-one (42)

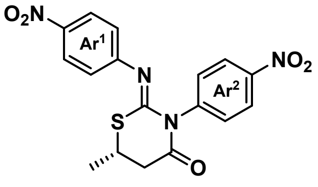

42

Following General Procedure G, 4-nitrophenyl (*E*)-but-2-enoate **S5** (93.2 mg, 0.45 mmol, 1.5 equiv.), 1,3-bis(4-nitrophenyl)thiourea **S18** (95.5 mg, 0.30 mmol, 1.0 equiv.) and (2*S*,3*R*)-HyperBTM **21** (18.5 mg, 0.06 mmol, 20 mol%) in anhydrous THF (0.1 M) for 48 h at RT gave crude product that was purified by flash silica column chromatography (hexane:CH_2Cl_2 30:70 to 0:100, R_F 0.34 at 0:100) to give the title compound as a pale yellow solid (101 mg, 87%). **mp** 186 – 188 °C; $[\alpha]_D^{20}$ –21.5 (*c* 0.2 in MeCN); **Chiral HPLC analysis,** Chiralpak IB (80:20 *n*-hexane : IPA, flow rate 1.5 mLmin^{-1}, 211 nm, 30 °C) t_R (*R*) 19.3 min, t_R (*S*) 21.7 min, 86:14 er; **IR** ν_{max} (film) 3107, 3080, 2978, 1699, 1612, 1582, 1528, 1508, 1491, 1327, 1283, 1217, 1152, 1105, 856, 839; **^{1}H NMR** (500 MHz, CD_3CN) δ_H: 1.40 (3H, d, *J* 6.8, C*H*$_3$), 2.95 (1H, dd, *J* 16.4, 10.1, C(5)H^AH^B), 3.18 (1H, dd, *J* 16.4, 3.3, C(5)H^AH^B), 3.78 (1H, dqd, *J* 10.1, 6.8, 3.3, C(6)*H*), 6.85 – 6.92 (2H, m, Ar^{1}C(2,6)*H*), 7.54 – 7.59 (2H, m, Ar^{2}C(2,6)*H*), 8.12 – 8.19 (2H, m, Ar^{1}C(3,5)*H*), 8.27 – 8.33 (2H, m, Ar^{2}C(3,5)*H*); **^{13}C{^{1}H} NMR** (126 MHz, CD_3CN) δ_C: 20.4 (CH_3), 33.6 (*C*(5)H_2), 43.4 (*C*(6)H), 122.0 (Ar^{1}C(2,6)H), 125.2 (Ar^2*C*(3,5)H), 126.0 (Ar^1*C*(3,5)H), 131.6 (Ar^2*C*(2,6)H), 143.9 (Ar^2*C*(1)), 145.1 (Ar^{1}C(4)), 145.7 (Ar^2*C*(4)), 148.3 (*C*(2)), 155.1 (Ar^1*C*(1)), 170.4 (*C*(4)); **HRMS** (ESI^+) $C_{17}H_{14}O_5N_4NaS$ $[M+Na]^+$ found 409.0573, requires 409.0577 (–1.0 ppm).

(*S*,*Z*)-6-Ethyl-3-(4-nitrophenyl)-2-((4-nitrophenyl)imino)-1,3-thiazinan-4-one (43)

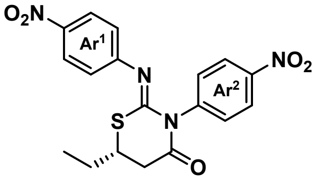

43

Following General Procedure G, perfluorophenyl (*E*)-pent-2-enoate **S15** (120 mg, 0.45 mmol, 1.5 equiv.), 1,3-bis(4-nitrophenyl)thiourea **S18** (95.5 mg, 0.30 mmol, 1.0 equiv.) and (2*S*,3*R*)-HyperBTM **21** (18.5 mg, 0.06 mmol, 20 mol%) in anhydrous THF (0.1 M) for

48 h at RT gave crude product that was purified by flash silica column chromatography (hexane:EtOAc 100:0 to 50:50, R_F 0.44 at 50:50) to give the title compound as a pale yellow solid (97 mg, 81%). **mp** 147 – 149 °C; $[\alpha]_D^{20}$ –38.9 (*c* 1.0 in $CHCl_3$); **Chiral HPLC analysis,** Chiralcel OD-H (70:30 *n*-hexane : IPA, flow rate 1.0 $mLmin^{-1}$, 211 nm, 30 °C) t_R (*R*) 18.1 min, t_R (*S*) 20.7 min, 89:11 er; **IR** ν_{max} (film) 2968, 1705, 1609, 1580, 1508, 1339, 1302, 1217, 1155, 1109, 858; **^{1}H NMR** (500 MHz, $CDCl_3$) δ_H: 1.08 (3H, t, *J* 7.4, CH_2CH_3), 1.73 – 1.91 (2H, m, $CH^AH^BCH_3$ and $CH^AH^BCH_3$), 3.04 (1H, dd, *J* 16.2, 10.0, C(5)H^AH^B), 3.30 (1H, dd, *J* 16.2, 3.2, C(5)H^AH^B), 3.46 – 3.54 (1H, m, C(6)*H*), 6.84 – 6.90 (2H, m, Ar^{1}C(2,6)*H*), 7.41 – 7.48 (2H, m, Ar^{2}C(2,6)*H*), 8.16 – 8.23 (2H, m, Ar^{1}C(3,5)*H*), 8.33 – 8.40 (2H, m, Ar^{2}C(3,5)*H*); **^{13}C{^{1}H} NMR** (126 MHz, $CDCl_3$) δ_C: 11.2 (CH_2CH_3), 27.9 (CH_2CH_3), 39.6 (*C*(5)H_2), 41.2 (*C*(6)H), 121.1 (Ar^1*C*(2,6)H), 124.7 (Ar^2*C*(3,5)H), 125.1 (Ar^1*C*(3,5)H), 130.1 (Ar^2*C*(2,6)H), 143.6 (Ar^2*C*(1)), 144.4 (Ar^1*C*(4)), 147.5 (Ar^2*C*(4)), 153.1 (*C*(2)), 153.3 (Ar^1*C*(1)), 169.0 (*C*(4)); **HRMS** (ESI^+) $C_{18}H_{16}O_5N_4NaS$ $[M + Na]^+$ found 423.0722, requires 423.0734 (–2.8 ppm).

(*R*,*Z*)-6-*iso*-Propyl-3-(4-nitrophenyl)-2-((4-nitrophenyl)imino)-1,3-thiazinan-4-one (44)

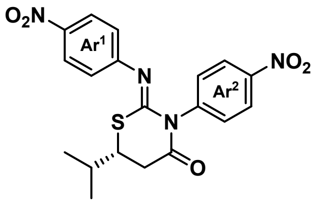

44

Following General Procedure G, 4-nitrophenyl (*E*)-4-methylpent-2-enoate **S6** (106 mg, 0.45 mmol, 1.5 equiv.), 1,3-bis(4-nitrophenyl)thiourea **S18** (95.5 mg, 0.30 mmol, 1.0 equiv.) and (2*S*,3*R*)-HyperBTM **21** (27.8 mg, 0.09 mmol, 30 mol%) in anhydrous THF (0.1 M) for 72 h at RT gave crude product that was purified twice by flash silica column chromatography (hexane:EtOAc 100:0 to 50:50, R_F 0.69 at 50:50 then hexane:CH_2Cl_2 50:50 to 0:100, R_F 0.49 at 0:100) to give the title compound as a pale yellow solid (95 mg, 77%). **mp** 54 – 56 °C; $[\alpha]_D^{20}$ –55.7 (*c* 1.0 in $CHCl_3$); **Chiral HPLC analysis,** Chiralpak AD-H (60:40 *n*-hexane : IPA, flow rate 1.0 $mLmin^{-1}$, 211 nm, 30 °C) t_R (*R*) 18.3 min, t_R (*S*) 22.5 min, 94:6 er; **IR** ν_{max} (film) 2967, 1705, 1612, 1582, 1510, 1339, 1281, 1219, 1155, 1109, 858; **^{1}H NMR** (500 MHz, $CDCl_3$) δ_H: 1.06 (6H, d, *J* 6.8, CH(CH_3)$_2$), 1.98 (1H, septd, *J* 6.8, 6.4, *CH*(CH_3)$_2$), 3.05 (1H, dd, *J* 16.2, 10.7, C(5)H^AH^B), 3.25 (1H, dd, *J* 16.2, 3.1, C(5)H^AH^B), 3.44

(1H, ddd, *J* 10.7, 6.4, 3.1, C(6)*H*), 6.82 – 6.87 (2H, m, $Ar^1C(2,6)H$), 7.40 – 7.45 (2H, m, $Ar^2C(2,6)H$), 8.14 – 8.19 (2H, m, $Ar^1C(3,5)H$), 8.31 – 8.36 (2H, m, $Ar^2C(3,5)H$); **$^{13}C\{^1H\}$ NMR** (126 MHz, $CDCl_3$) δ_C: 19.6 ($CH(CH_3)_2$), 32.2 ($CH(CH_3)_2$), 39.3 ($C(5)H_2$), 44.9 (*C*(6)H), 121.2 ($Ar^1C(2,6)H$), 124.8 ($Ar^2C(3,5)H$), 125.2 ($Ar^1C(3,5)H$), 130.2 ($Ar^2C(2,6)H$), 143.8 ($Ar^2C(1)$), 144.4 ($Ar^1C(4)$), 147.5 ($Ar^2C(4)$), 153.4 (*C*(2)), 153.5 ($Ar^1C(1)$), 169.5 (*C*(4)); **HRMS** (ESI^+) $C_{19}H_{19}O_5N_4S$ $[M + H]^+$ found 415.1061, requires 415.1071 (–2.3 ppm).

(*R*,*Z*)-6-(Methoxymethyl)-3-(4-nitrophenyl)-2-((4-nitrophenyl)imino)-1,3-thiazinan-4-one (45)

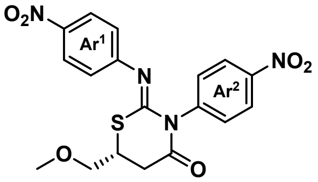

45

Following General Procedure G, perfluorophenyl (*E*)-4-methoxybut-2-enoate **S14** (127 mg, 0.45 mmol, 1.5 equiv.), 1,3-bis(4-nitrophenyl)thiourea **S18** (95.5 mg, 0.30 mmol, 1.0 equiv.) and (2*S*,3*R*)-HyperBTM **21** (18.5 mg, 0.06 mmol, 20 mol%) in anhydrous THF (0.1 M) for 24 h at RT gave crude product that was purified by flash silica column chromatography (CH_2Cl_2:Et_2O 100:0 to 95:5, R_F 0.58 at 95:5) to give the title compound as a pale yellow solid (110 mg, 88%). **mp** 67 – 69 °C; $[\alpha]_D^{20}$ +47.7 (*c* 1.0 in $CHCl_3$); **Chiral HPLC analysis,** Chiralpak AD-H (70:30 *n*-hexane : IPA, flow rate 1.0 mLmin^{-1}, 211 nm, 30 °C) t_R (*S*) 28.8 min, t_R (*R*) 35.2 min, 86:14 er; **IR** ν_{max} (film) 2930, 1705, 1612, 1582, 1516, 1508, 1341, 1298, 1219, 1157, 1111, 860; **^1H NMR** (500 MHz, $CDCl_3$) δ_H: 3.15 (1H, dd, *J* 16.4, 7.1, $C(5)H^AH^B$), 3.26 (1H, dd, *J* 16.4, 4.1, $C(5)H^AH^B$), 3.41 (3H, s, OCH_3), 3.61 – 3.73 (3H, m, C(6)*H* and CH_2O), 6.81 – 6.89 (2H, m, $Ar^1C(2,6)H$), 7.39 – 7.46 (2H, m, $Ar^2C(2,6)H$), 8.11 – 8.18 (2H, m, $Ar^1C(3,5)H$), 8.29 – 8.36 (2H, m, $Ar^2C(3,5)H$); **$^{13}C\{^1H\}$ NMR** (126 MHz, $CDCl_3$) δ_C: 37.8 ($C(5)H_2$), 38.0 (*C*(6)H), 59.6 (OCH_3), 74.6 (CH_2O), 121.2 ($Ar^1C(2,6)H$), 124.7 ($Ar^2C(3,5)H$), 125.1 ($Ar^1C(3,5)H$), 130.1 ($Ar^2C(2,6)H$), 144.0 ($Ar^2C(1)$), 144.3 ($Ar^1C(4)$), 147.4 ($Ar^2C(4)$), 153.1 (*C*(2)), 153.5 ($Ar^1C(1)$), 168.6 (*C*(4)); **HRMS** (ESI^+) $C_{18}H_{17}O_6N_4S$ $[M + H]^+$ found 417.0858, requires 417.0863 (–1.2 ppm).

(*Z*)-3-Methyl-2-((4-nitrophenyl)imino)-6-(trifluoromethyl)-1,3-thiazinan-4-one (46)

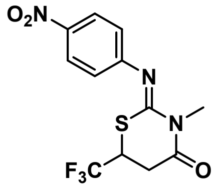

46

Following General Procedure G, perfluorophenyl (*E*)-4,4,4-trifluorobut-2-enoate **28** (138 mg, 0.45 mmol, 1.5 equiv.), 1-methyl-3-(4-nitrophenyl)thiourea **S24** (63.4 mg, 0.30 mmol, 1.0 equiv.) and (2*S*,3*R*)-HyperBTM **21** (18.5 mg, 0.06 mmol, 20 mol%) in anhydrous THF (0.1 M) for 48 h at RT gave crude product that was purified by flash silica column chromatography (hexane:CH_2Cl_2 90:10 to 0:100, R_F 0.34 at 0:100) to give the title compound as a white solid (77 mg, 77%). **mp** 97 – 99 °C; **Chiral HPLC analysis,** Chiralcel OD-H (90:10 *n*-hexane : IPA, flow rate 1.0 mLmin^{-1}, 211 nm, 30 °C) t_R 19.0 min, t_R 24.9 min, 50:50 er; **IR** ν_{max} (film) 2934, 1686, 1611, 1584, 1508, 1337, 1215, 1200, 1165, 1126, 1107, 1049, 962, 854; **^{1}H NMR** (400 MHz, $CDCl_3$) δ_H: 3.20 (1H, dd, *J* 17.0, 5.3, C(5)H^AH^B), 3.28 (1H, dd, *J* 17.0, 5.2, C(5)H^AH^B), 3.49 (3H, s, CH_3), 3.75 – 3.85 (1H, m, C(6)*H*), 6.93 – 7.00 (2H, m, ArC(2,6)*H*), 8.19 – 8.26 (2H, m, ArC(3,5)*H*); **^{19}F{^{1}H} NMR** (377 MHz, $CDCl_3$) δ_F: –71.7 (s, CF_3); **^{13}C{^{1}H} NMR** (126 MHz, $CDCl_3$) δ_C: 31.4 (CH_3), 33.4 (*C*(5)H_2), 39.2 (q, *J* 32.6, *C*(6)H), 121.3 (Ar*C*(3,5)H), 124.5 (q, *J* 279.8, CF_3), 125.4 (Ar*C*(2,6)H), 144.7 (Ar*C*(1)), 148.4 (Ar*C*(4)), 153.2 (*C*(2)), 166.1 (*C*(4)); **HRMS** (ESI^+) $C_{12}H_{10}O_3N_3F_3NaS$ $[M+Na]^+$ found 356.0289, requires 356.0287 (+0.6 ppm).

(*R*,*Z*)-4-Methyl-*N*-(3-methyl-4-oxo-6-(trifluoromethyl)-1,3-thiazinan-2-ylidene)benzenesulfonamide (47)

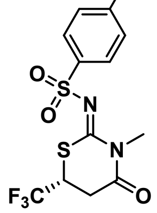

47

Following General Procedure G, perfluorophenyl (*E*)-4,4,4-trifluorobut-2-enoate **28** (138 mg, 0.45 mmol, 1.5 equiv.), 4-methyl-*N*-(methylcarbamothioyl)benzenesulfonamide

S25 (73.3 mg, 0.30 mmol, 1.0 equiv.), and (2*S*,3*R*)-HyperBTM **21** (4.6 mg, 0.015 mmol, 5 mol%) in anhydrous THF (0.1 M) for 24 h at 0 °C gave crude product that was purified by flash silica column chromatography (CH_2Cl_2:Et_2O 100:0 to 90:10, R_F 0.50 at 95:5) to give the title compound as a white solid (97 mg, 88%). **mp** 113 – 115 °C; $[\alpha]_D^{20}$ –2.5 (*c* 1.0 in $CHCl_3$); **Chiral HPLC analysis,** Chiralpak IB (80:20 *n*-hexane : IPA, flow rate 1.5 mLmin^{-1}, 254 nm, 30 °C) t_R (*S*) 11.6 min, t_R (*R*) 23.6 min, 67:33 er; **IR** ν_{max} (film) 2957, 1717, 1599, 1510, 1414, 1315, 1155, 1125, 1088, 1051, 962, 926, 820, 775; **^{1}H NMR** (400 MHz, $CDCl_3$) δ_H: 2.42 (3H, s, C*H*$_3$), 3.25 – 3.28 (2H, m, C(5)*H*$_2$), 3.30 (3H, s, NC*H*$_3$), 3.94 (1H, qt, *J* 7.7, 5.3, C(6)*H*), 7.28 – 7.34 (2H, m, ArC(3,5)*H*), 7.80 – 7.86 (2H, m, ArC(2,6)*H*); **^{19}F{^{1}H} NMR** (377 MHz, $CDCl_3$) δ_F: –71.3 (s, C*F*$_3$); **^{13}C{^{1}H} NMR** (126 MHz, $CDCl_3$) δ_C: 21.7 (CH_3), 32.2 (NCH_3), 32.8 (*C*(5)H_2), 39.6 (q, *J* 32.5, *C*(6)H), 124.3 (q, *J* 280.2, *C*F_3), 127.1 (Ar*C*(2,6)H), 129.7 (Ar*C*(3,5)H), 137.6 (Ar*C*(1)), 144.3 (Ar*C*(4)), 160.1 (*C*(2)), 166.0 (*C*(4)); **HRMS** (ESI^+) $C_{13}H_{13}O_3N_2F_3NaS_2$ $[M+Na]^+$ found 389.0214, requires 389.0212 (+0.5 ppm).

(*R*,*Z*)-*N*-(3-Benzyl-4-oxo-6-(trifluoromethyl)-1,3-thiazinan-2-ylidene)-4-methylbenzenesulfonamide (48)

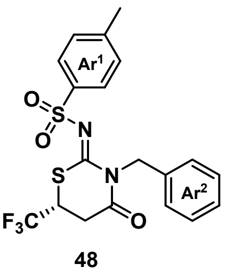

48

Following General Procedure G, perfluorophenyl (*E*)-4,4,4-trifluorobut-2-enoate **28** (138 mg, 0.45 mmol, 1.5 equiv.), 4-methyl-*N*-(benzylcarbamothioyl)benzenesulfonamide **S26** (96.1 mg, 0.30 mmol, 1.0 equiv.), and (2*S*,3*R*)-HyperBTM **21** (4.6 mg, 0.015 mmol, 5 mol%) in anhydrous THF (0.1 M) for 24 h at 0 °C gave crude product that was purified by flash silica column chromatography (hexane:CH_2Cl_2 100:0 to 0:100, R_F 0.17 at 0:100) to give the title compound as a colourless oil (130 mg, 98%). $[\alpha]_D^{20}$ +12.8 (*c* 0.5 in CH_2Cl_2); **Chiral HPLC analysis,** Chiralcel OD-H (70:30 *n*-hexane : IPA, flow rate 1.0 mLmin^{-1}, 211 nm, 30 °C) t_R (*S*) 13.4 min, t_R (*R*) 27.4 min, 78:22 er; **IR** ν_{max} (film) 2953, 1717, 1508, 1429, 1373, 1352, 1317, 1304, 1215, 1173, 1152, 1121, 1088, 1020, 961, 889, 816, 779; **^{1}H NMR** (400 MHz, $CDCl_3$) δ_H: 2.43 (3H, s, C*H*$_3$), 3.27 (1H, dd, *J* 17.0, 5.5, C(5)*H*^{A}H^B), 3.33 (1H, dd, *J* 17.0,

4.9, C(5)H^A*H*^{B}), 3.90 (1H, qdd, *J* 7.9, 5.5, 4.9, C(6)*H*), 5.09 (1H, d, *J* 14.1, NC*H*^{A}H^B), 5.22 (1H, d, *J* 14.1, NCH^A*H*^{B}), 7.05 – 7.10 (2H, m, Ar^{2}C(2,6)*H*), 7.11 – 7.22 (3H, m, Ar^{2}C(3,4,5)*H*), 7.23 – 7.28 (2H, m, Ar^{1}C(3,5)*H*), 7.64 – 7.69 (2H, m, Ar^{1}C(2,6)*H*); **^{19}F{^{1}H} NMR** (377 MHz, $CDCl_3$) δ_F: –71.3 (s, C*F*$_3$); **^{13}C{^{1}H} NMR** (126 MHz, $CDCl_3$) δ_C: 21.7 (CH_3), 32.8 (*C*(5)H_2), 39.5 (q, *J* 32.6, *C*(6)H), 47.7 (NCH_2), 124.3 (q, *J* 280.3, CF_3), 127.1 (Ar^1*C*(2,6)H), 127.7 (Ar^2*C*(4)H), 128.4 (Ar^{2}C(3,5)H), 128.8 (Ar^{2}C(2,6)H), 129.5 (Ar^{1}C(3,5)H), 135.6 (Ar^{2}C(1)), 137.3 (Ar^1*C*(1)), 144.1 (Ar^1*C*(4)), 159.2 (*C*(2)), 166.0 (*C*(4)); **HRMS** (ESI^+) $C_{19}H_{17}O_3N_2F_3NaS_2$ $[M+Na]^+$ found 465.0519, requires 465.00525 (–1.3 ppm).

(*R*,*Z*)-4-Methyl-*N*-(4-oxo-3-phenyl-6-(trifluoromethyl)-1,3-thiazinan-2-ylidene)benzenesulfonamide (49)

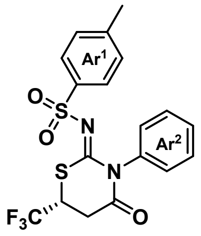

49

Following General Procedure G, perfluorophenyl (*E*)-4,4,4-trifluorobut-2-enoate **28** (138 mg, 0.45 mmol, 1.5 equiv.), 4-methyl-*N*-(phenylcarbamothioyl)benzenesulfonamide **77** (91.9 mg, 0.30 mmol, 1.0 equiv.), and (2*S*,3*R*)-HyperBTM **21** (4.6 mg, 0.015 mmol, 5 mol%) in anhydrous THF (0.1 M) for 24 h at 0 °C gave crude product that was purified by flash silica column chromatography (CH_2Cl_2:Et_2O 100:0 to 90:10, R_F 0.35 at 95:5) to give the title compound as a white solid (117 mg, 91%). **mp** 157 – 159 °C; $[\alpha]_D^{20}$ +66.6 (*c* 0.5 in CH_3CN); **Chiral HPLC analysis,** Chiralpak IA (80:20 *n*-hexane : IPA, flow rate 1.0 mLmin^{-1}, 211 nm, 30 °C) t_R (*S*) 13.7 min, t_R (*R*) 15.5 min, 91:9 er; **IR** ν_{max} (film) 2970, 1722, 1533, 1279, 1167, 1142, 1121, 1088, 1005, 959, 837, 816; **^{1}H NMR** (400 MHz, CD_3CN) δ_H: 2.38 (3H, s, C*H*$_3$), 3.33 (1H, dd, *J* 17.4, 4.2, C(5)*H*^{A}H^B), 3.58 (1H, dd, *J* 17.4, 5.5, C(5)H^A*H*^{B}), 4.37 (1H, qdd, *J* 8.4, 5.5, 4.2, C(6)*H*), 7.05 (2H, br s, Ar^{2}C(2,6)*H*), 7.25 – 7.32 (2H, m, Ar^{1}C(3,5)*H*), 7.38 – 7.53 (5H, m, Ar^{1}C(2,6)*H* and Ar^{2}C(3,4,5)*H*); **^{19}F{^{1}H} NMR** (377 MHz, CD_3CN)

δ_F: –71.9 (s, C*F*$_3$); **^{13}C{^{1}H} NMR** (126 MHz, CD_3CN) δ_C: 21.5 (CH_3), 33.6 (*C*(5)H_2), 40.3 (q, *J* 32.0, *C*(6)H), 126.1 (q, *J* 279.5, CF_3), 127.3 (Ar^{1}C(2,6)H), 129.0 (Ar^{2}C(2,6)H), 129.7

(Ar^2*C*(3,5)H), 130.3 (Ar^2*C*(4)H), 130.4 (Ar^1*C*(3,5)H), 138.3 (Ar^2*C*(1)), 138.6 (Ar^1*C*(1)), 145.1 (Ar^{1}C(4)), 163.5 (*C*(2)), 167.7 (*C*(4)); **HRMS** (ESI^+) $C_{18}H_{15}O_3N_2F_3NaS_2$ $[M+Na]^+$ found 451.0372, requires 451.0368 (+0.9 ppm).

(*R*,*Z*)-4-Methyl-*N*-(4-oxo-6-(trifluoromethyl)-3-(4-(trifluoromethyl)phenyl)-1,3-thiazinan-2-ylidene)benzenesulfonamide (50)

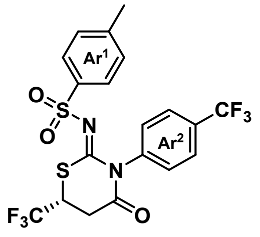

50

Following General Procedure G, perfluorophenyl (*E*)-4,4,4-trifluorobut-2-enoate **28** (138 mg, 0.45 mmol, 1.5 equiv.), 4-methyl-*N*-((4-(trifluoromethyl)phenyl)carbamothioyl) benzenesulfonamide **S22** (112 mg, 0.30 mmol, 1.0 equiv.), and (2*S*,3*R*)-HyperBTM **21** (4.6 mg, 0.015 mmol, 5 mol%) in anhydrous THF (0.1 M) for 24 h at 0 °C gave crude product that was purified by flash silica column chromatography (CH_2Cl_2:Et_2O 100:0 to 95:5, R_F 0.37 at 95:5) to give the title compound as a white solid (118 mg, 79%). **mp** 182 – 184 °C; $[\alpha]_D^{20}$ +56.8 (*c* 0.5 in CH_3CN); **Chiral HPLC analysis,** Chiralpak ID (93:7 *n*-hexane : IPA, flow rate 1.0 mLmin^{-1}, 254 nm, 40 °C) t_R (*S*) 22.9 min, t_R (*R*) 25.8 min, 88:12 er; **IR** ν_{max} (film) 2968, 2359, 1732, 1520, 1373, 1331, 1281, 1167, 1125, 1090, 1069, 1003, 961, 851, 818; **^{1}H NMR** (400 MHz, CD_3CN) δ_H: 2.38 (3H, s, C*H*$_3$), 3.35 (1H, dd, *J* 17.4, 4.3, C(5)H^AH^B), 3.59 (1H, dd, *J* 17.4, 5.5, C(5)H^AH^B), 4.41 (1H, qdd, *J* 8.4, 5.5, 4.3, C(6)*H*), 7.20 – 7.31 (4H, m, Ar^{1}C(3,5)*H* and Ar^{2}C(2,6)*H*), 7.46 – 7.52 (2H, m, Ar^{1}C(2,6)*H*), 7.73 – 7.78 (2H, m, Ar^{2}C(3,5)*H*); **^{19}F{^{1}H} NMR** (377 MHz, CD_3CN) δ_F: –71.9 (s, C*F*$_3$), -63.2 (s, C*F*$_3$); **^{13}C{^{1}H} NMR** (126 MHz, CD_3CN) δ_C: 21.5 (CH_3), 33.5 (*C*(5)H_2), 40.4 (q, *J* 31.9, *C*(6)H), 125.0 (q, *J* 271.6, CF_3), 126.0 (q, *J* 279.5, CF_3), 127.4 (Ar^1*C*(2,6)H), 127.4 – 127.5 (m, Ar^2*C*(3,5)H), 130.2 (Ar^2*C*(2,6)H), 130.4 (Ar^{1}C(3,5)H), 131.1 (q, *J* 32.5, Ar^2*C*(4)), 138.4 (Ar^1*C*(1)), 141.9 (Ar^2*C*(1)), 145.4 (Ar^1*C*(4)), 163.3 (*C*(2)), 167.7 (*C*(4)); **HRMS** (ESI^+) $C_{19}H_{14}O_3N_2F_6NaS_2$ $[M+Na]^+$ found 519.0244, requires 519.0242 (+0.4 ppm).

(*R*,*Z*)-*N*-(3-(4-Methoxyphenyl)-4-oxo-6-(trifluoromethyl)-1,3-thiazinan-2-ylidene)-4-methylbenzenesulfonamide (51)

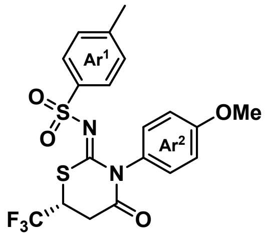

51

Following General Procedure G, perfluorophenyl (*E*)-4,4,4-trifluorobut-2-enoate **28** (138 mg, 0.45 mmol, 1.5 equiv.), *N*-((4-methoxyphenyl)carbamothioyl)-4-methylbenzenesulfonamide **S21** (101 mg, 0.30 mmol, 1.0 equiv.), and (2*S*,3*R*)-HyperBTM **21** (4.6 mg, 0.015 mmol, 5 mol%) in anhydrous THF (0.1 M) for 24 h at 0 °C gave crude product that was purified by flash silica column chromatography (CH_2Cl_2:Et_2O 100:0 to 95:5, R_F 0.33 at 95:5) to give the title compound as a mixture of regioisomers (90:10) as a white solid (122 mg, 88%). **mp** 131 – 133 °C; $[\alpha]_D^{20}$ +79.8 (*c* 0.5 in CH_3CN); **Chiral HPLC analysis,** Chiralpak AD-H (80:20 *n*-hexane : IPA, flow rate 1.0 mLmin^{-1}, 254 nm, 30 °C) t_R (*S*) 18.0 min, t_R (*R*) 22.1 min, 90:10 er; **IR** ν_{max} (film) 2959, 1724, 1607, 1518, 1508, 1371, 1279, 1248, 1163, 1142, 1123, 1086, 1028, 1001, 957, 885, 845, 816; **^{1}H NMR** (400 MHz, CD_3CN) δ_H: 2.39 (3H, s, CH_3), 3.32 (1H, dd, *J* 17.3, 4.2, C(5)H^AH^B), 3.56 (1H, dd, *J* 17.3, 5.5, C(5)H^AH^B), 3.81 (3H, s, OCH_3), 4.35 (1H, qdd, *J* 8.4, 5.5, 4.2, C(6)*H*), 6.93 – 6.99 (4H, m, Ar^{2}C(2,3,5,6)*H*), 7.26 – 7.32 (2H, m, Ar^{1}C(3,5)*H*), 7.50 – 7.55 (2H, m, Ar^{2}C(2,6)*H*); **^{19}F{^{1}H} NMR** (376 MHz, CD_3CN) δ_F: –71.9 (s, CF_3); **^{13}C{^{1}H} NMR** (126 MHz, CD_3CN) δ_C: 21.5 (CH_3), 33.7 (*C*(5)H_2), 40.2 (q, *J* 31.9, *C*(6)H), 56.2 (OCH_3),115.4 (Ar^2*C*(3,5)H), 126.1 (q, *J* 279.5, CF_3), 127.3 (Ar^1*C*(2,6)H), 130.0 (br, Ar^2*C*(2,6)H), 130.4 (Ar^{1}C(3,5)H), 130.5 (Ar^{2}C(1)), 138.7 (Ar^{1}C(1)), 145.1 (Ar^{1}C(4)), 160.6 (Ar^{1}C(4)), 163.8 (*C*(2)), 167.8 (*C*(4)); **HRMS** (ESI^+) $C_{19}H_{17}O_4N_2F_3NaS_2$ $[M + Na]^+$ found 481.0473, requires 481.0474 (–0.2 ppm).

(*R*,*Z*)-4-Methoxy-*N*-(4-oxo-3-phenyl-6-(trifluoromethyl)-1,3-thiazinan-2-ylidene) benzenesulfonamide (52)

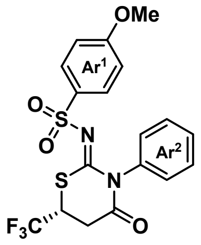

52

Following General Procedure G, perfluorophenyl (*E*)-4,4,4-trifluorobut-2-enoate **28** (138 mg, 0.45 mmol, 1.5 equiv.), 4-methoxy-*N*-(phenylcarbamothioyl)benzenesulfonamide **S23** (96.7 mg, 0.30 mmol, 1.0 equiv.), and (2*S*,3*R*)-HyperBTM **21** (4.6 mg, 0.015 mmol, 5 mol%) in anhydrous THF (0.1 M) for 24 h at 0 °C gave crude product that was purified by flash silica column chromatography (CH_2Cl_2:Et_2O 100:0 to 95:5, R_F 0.30 at 95:5) to give the title compound as a white solid (128 mg, 96%). **mp** 128 – 130 °C; $[\alpha]_D^{20}$ +77.0 (*c* 0.5 in CH_3CN); **Chiral HPLC analysis,** Chiralpak IA (80:20 *n*-hexane : IPA, flow rate 1.0 mLmin^{-1}, 211 nm, 30 °C) t_R (*S*) 17.2 min, t_R (*R*) 21.2 min, 91:9 er; **IR** ν_{max} (film) 2968, 1719, 1593, 1518, 1491, 1323, 1260, 1167, 1138, 1117, 1088, 1007, 959, 883, 833, 766; **^{1}H NMR** (400 MHz, CD_3CN) δ_H: 3.33 (1H, dd, *J* 17.4, 4.2, C(5)H^AH^B), 3.56 (1H, dd, *J* 17.4, 5.5, C(5)H^AH^B), 3.83 (3H, s, OCH_3), 4.37 (1H, qdd, *J* 8.4, 5.5, 4.2, C(6)*H*), 6.94 – 6.99 (2H, m, Ar^{1}C(3,5)*H*), 7.04 (2H, br s, Ar^{2}C(2,6)*H*), 7.39 – 7.48 (3H, m, Ar^{2}C(3,4,5)*H*), 7.54 – 7.59 (2H, m, Ar^{1}C(2,6)*H*); **^{19}F{^{1}H} NMR** (377 MHz, CD_3CN) δ_F: –71.8 (s, CF_3); **^{13}C{^{1}H} NMR** (126 MHz, CD_3CN) δ_C: 33.6 (*C*(5)H_2), 40.3 (q, *J* 31.9, *C*(6)H), 56.5 (OCH_3), 115.0 (Ar^1*C*(3,5)H), 126.1 (q, *J* 279.5, CF_3), 129.1 (Ar^{2}C(2,6)H), 129.6 (Ar^1*C*(2,6)H), 129.7 (Ar^2*C*(4)H), 130.3 (Ar^2*C*(3,5)H), 133.1 (Ar^1*C*(1)), 138.3 (Ar^2*C*(1)), 163.2 (Ar^1*C*(4)), 164.2 (*C*(2)), 167.7 (*C*(4)); **HRMS** (ESI^+) $C_{18}H_{15}O_4N_2F_3NaS_2$ $[M+Na]^+$ found 467.0315, requires 467.0318 (–0.6 ppm).

(*R*,*Z*)-2,2,2-Trifluoro-*N*-(4-oxo-3-phenyl-6-(trifluoromethyl)-1,3-thiazinan-2-ylidene)acetamide (53)

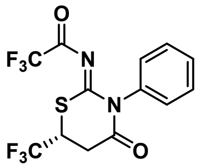

53

Following General Procedure G, perfluorophenyl (*E*)-4,4,4-trifluorobut-2-enoate **28** (138 mg, 0.45 mmol, 1.5 equiv.), 2,2,2-trifluoro-*N*-(phenylcarbamothioyl)acetamide **S27** (74.5 mg, 0.30 mmol, 1.0 equiv.), and (2*S*,3*R*)-HyperBTM **21** (4.6 mg, 0.015 mmol, 5 mol%) in anhydrous THF (0.1 M) for 24 h at 0 °C gave crude product that was purified by flash silica column chromatography (hexane:CH_2Cl_2 80:20 to 0:100, R_F 0.45 at 0:100) to give the title compound as a colourless glass (63 mg, 57%). $[\alpha]_D^{20}$ +11.0 (*c* 0.6 in $CHCl_3$); **Chiral HPLC analysis,** Chiralcel OD-H (90:10 *n*-hexane : IPA, flow rate 2.0 $mLmin^{-1}$, 211 nm, 40 °C) t_R (*S*) 7.0 min, t_R (*R*) 16.5 min, 96:4 er; **IR** ν_{max} (film) 2949, 1655, 1595, 1491, 1360, 1315, 1231, 1167, 1115, 1043, 978, 910, 835, 797, 756; **^{1}H NMR** (400 MHz, $CDCl_3$) δ_H: 3.45 (1H, dd, *J* 17.3, 5.4, C(5)H^AH^B), 3.52 (1H, dd, *J* 17.3, 4.4, C(5)H^AH^B), 3.97 (1H, qdd, *J* 7.9, 5.4, 4.4, C(6)*H*), 7.03 – 7.10 (2H, m, ArC(2,6)*H*), 7.41 – 7.51 (3H, m, ArC(3,4,5)*H*); **^{19}F{^{1}H} NMR** (377 MHz, $CDCl_3$) δ_F: –76.8 (s, C*F*$_3$), –71.1 (s, C*F*$_3$); **^{13}C{^{1}H} NMR** (126 MHz, $CDCl_3$) δ_C: 33.2 (*C*(5)H_2), 39.8 (q, *J* 32.5, *C*(6)H), 115.5 (q, *J* 287.0, CF_3), 124.6 (q, *J* 280.3, CF_3), 127.7 (Ar^2*C*(2,6)H), 129.4 (Ar^2*C*(4)H), 129.6 (Ar^{2}C(3,5)H), 136.5 (Ar*C*(1)), 165.9 (*C*(2)), 166.3

(q, *J* 39.1, *C*(O)CF_3), 167.5 (*C*(4)); **HRMS** (ESI^-) $C_{13}H_7O_2N_2F_6S$ $[M-H]^-$ found 369.0142, requires 369.0138 (+1.1 ppm).

(*R*,*Z*)-3-(4-Nitrophenyl)-2-((2,4,6-trichlorophenyl)imino)-6-(trifluoromethyl)-1,3-thiazinan-4-one (54)

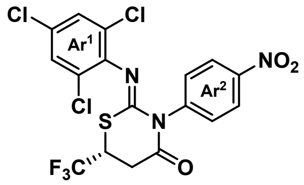

54

Following General Procedure G, perfluorophenyl (*E*)-4,4,4-trifluorobut-2-enoate **28** (138 mg, 0.45 mmol, 1.5 equiv.), 1-(4-nitrophenyl)-3-(2,4,6-trichlorophenyl)thiourea **S20** (113 mg, 0.30 mmol, 1.0 equiv.) and (2*S*,3*R*)-HyperBTM **21** (9.3 mg, 0.015 mmol, 5 mol%) in anhydrous THF (0.1 M) for 48 h at 0 °C gave crude product that was purified by flash silica column chromatography (hexane:EtOAc 95:5 to 70:30, R_F 0.23 at 75:25) to give the title compound as a yellow solid (138 mg, 92%). **mp** 157 – 159 °C; $[\alpha]_D^{20}$ +41.7 (*c* 1.0 in $CHCl_3$); **Chiral HPLC analysis,** Chiralpak AD-H (90:10 *n*-hexane : IPA, flow rate 1.0 mLmin^{-1}, 211 nm, 30 °C) t_R (*R*) 14.0 min, t_R (*S*) 17.9 min, 98:2 er; **IR** ν_{max} (film) 1713, 1614, 1524, 1348, 1298, 1227, 1177, 1121, 853, 841; **^{1}H NMR** (500 MHz, $CDCl_3$) δ_H: 3.46 (1H, dd, *J* 17.3, 5.4, C(5)H^AH^B), 3.51 (1H, dd, *J* 17.3, 4.0, C(5)H^AH^B), 3.86 – 3.95 (1H, m, C(6)*H*), 7.30 (1H, d, *J* 2.2, Ar^{1}C(3)*H*), 7.32 (1H, d, *J* 2.2, Ar^{1}C(5)*H*), 7.44 – 7.49 (2H, m, Ar^{2}C(2,6)*H*), 8.33 – 8.38 (2H, m, Ar^{2}C(3,5)*H*); **^{19}F{^{1}H} NMR** (376 MHz, $CDCl_3$) δ_F: –71.3 (s, CF_3); **^{13}C{^{1}H} NMR** (126 MHz, $CDCl_3$) δ_C: 33.7 (*C*(5)H_2), 39.5 (q, *J* 32.8, *C*(6)H), 124.7 (q, *J* 280.2, *C*F_3), 124.9 (Ar^2*C*(3,5)H), 126.6 (Ar^1*C*(2)), 127.0 (Ar^1*C*(6)), 128.3 (Ar^1*C*(5)H), 128.7 (Ar^1*C*(3)H), 130.1 (Ar^2*C*(2,6)H), 130.2 (Ar^1*C*(4)), 140.1 (Ar^1*C*(1)), 142.7 (Ar^2*C*(1)), 147.9 (Ar^2*C*(4)), 152.3 (*C*(2)), 165.6 (*C*(4)); **HRMS** (ESI^+) $C_{17}H_{10}O_3N_3F_3{}^{35}Cl_3S$ $[M+H]^+$ found 497.9442, requires 497.9455 (–2.6 ppm).

(*R*,*Z*)-*N*-(4-Oxo-3-phenyl-6-(trifluoromethyl)-1,3-selenazinan-2-ylidene)benzamide (55)

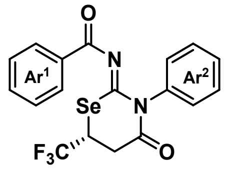

55

Following General Procedure G, perfluorophenyl (*E*)-4,4,4-trifluorobut-2-enoate **28** (138 mg, 0.45 mmol, 1.5 equiv.), *N*-(phenylcarbamoselenoyl)benzamide **S31** (91.0 mg, 0.30 mmol, 1.0 equiv.), and (2*S*,3*R*)-HyperBTM **21** (9.3 mg, 0.03 mmol, 10 mol%) in anhydrous THF (0.1 M) for 24 h at 0 °C gave crude product that was purified by flash silica column chromatography (hexane:CH_2Cl_2 60:40 to 0:100, R_F 0.47 at 0:100) to give the title compound as a colourless solid (124 mg, 97%). **mp** 117 – 119 °C; $[\alpha]_D^{20}$ +21.3 (*c* 0.8 in $CHCl_3$); **Chiral HPLC analysis,** Chiralpak IA (80:20 *n*-hexane : IPA, flow rate 1.0 mLmin^{-1}, 254 nm, 30 °C) t_R (*S*) 8.0 min, t_R (*R*) 10.2 min, 99:1 er; **IR** ν_{max} (film) 2965, 1717,

1634, 1497, 1476, 1362, 1325, 1233, 1169, 1153, 1103, 1086, 1024, 887; **^{1}H NMR** (500 MHz, $CDCl_3$) δ_H: 3.56 (1H, dd, *J* 16.7, 5.0, C(5)H^AH^B), 3.66 (1H, dd, *J* 16.7, 5.2, C(5)H^AH^B), 3.79 (1H, qdd, *J* 9.0, 5.2, 5.0, C(6)*H*), 7.16 – 7.20 (2H, m, Ar^{2}C(2,6)*H*), 7.28 – 7.34 (2H, m, Ar^{1}C(3,5)*H*), 7.45 – 7.50 (2H, m, Ar^{1}C(4)*H* and Ar^{2}C(4)*H*), 7.51 – 7.56 (2H, m, Ar^{2}C(3,5)*H*), 7.72 – 7.76 (2H, m, Ar^{1}C(2,6)*H*); **^{19}F{^{1}H} NMR** (470 MHz, $CDCl_3$) δ_F: –69.1 (s, C*F*$_3$); **^{13}C{^{1}H} NMR** (126 MHz, $CDCl_3$) δ_C: 32.8 (q, *J* 31.5, *C*(6)H), 35.5 (*C*(5)H_2), 126.0 (q, *J* 278.1, *C*F_3), 128.0 (Ar^2*C*(2,6)H), 128.5 (Ar^1*C*(3,5)H), 128.6 (Ar^2*C*(4)H), 129.4 (Ar^2*C*(3,5)H), 130.3 (Ar^1*C*(2,6)H), 133.6 (Ar^1*C*(4)), 134.6 (Ar^1*C*(1)), 139.6 (Ar^2*C*(1)), 164.8 (*C*(2)), 168.2 (*C*(4)), 177.1 (*C*O); **^{77}Se{^{1}H} NMR** (95 MHz, $CDCl_3$) δ_{Se}: 498.9 (s, C*Se*); **HRMS** (ESI^+) $C_{18}H_{13}O_2N_2F_3Na^{80}Se$ $[M+Na]^+$ found 448.9988, requires 448.9987 (+0.2 ppm).

(*R*,*Z*)-*N*-(3-(4-(*N*-(5-Methylisoxazol-3-yl)sulfamoyl)phenyl)-4-oxo-6-(trifluoromethyl)-1,3-selenazinan-2-ylidene)benzamide (56)

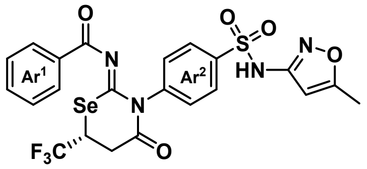

56

Following General Procedure G, perfluorophenyl (*E*)-4,4,4-trifluorobut-2-enoate **28** (138 mg, 0.45 mmol, 1.5 equiv.), *N*-((4-(*N*-(5-methylisoxazol-3-yl)sulfamoyl) phenyl)carbamoselenoyl)benzamide **S36** (139 mg, 0.30 mmol, 1.0 equiv.), and (2*S*,3*R*)-HyperBTM **21** (9.3 mg, 0.03 mmol, 10 mol%) in anhydrous THF (0.1 M) for 24 h at 0 °C gave crude product that was purified by flash silica column chromatography (hexane:EtOAc 90:10 to 0:100, R_F 0.42 at 50:50) to give the title compound as a white solid (108 mg, 61%). **mp** 140 °C (dec.); $[\alpha]_D^{20}$ +49.0 (*c* 0.2 in CH_3CN); **Chiral HPLC analysis,** Chiralpak AD-H (60:40 *n*-hexane : IPA, flow rate 1.0 mLmin^{-1}, 254 nm, 30 °C) t_R (*S*) 8.8 min, t_R (*R*) 12.0 min, 98:2 er; **IR** ν_{max} (film) 3273, 1718, 1616, 1458, 1387, 1323, 1248, 1219, 1161, 1090, 1067, 993, 926, 891; **^{1}H NMR** (500 MHz, CD_3CN) δ_H: 2.29 (3H, s, C*H*$_3$), 3.53 (1H, dd, *J* 17.0, 4.8, C(5)H^AH^B), 3.69 (1H, dd, *J* 17.0, 5.1, C(5)H^AH^B), 3.79 (1H, qdd, *J* 9.5, 5.1, 4.8, C(6)*H*), 6.21 (1H, s, C*H*), 7.30 – 7.36 (2H, m, Ar^{1}C(3,5)*H*), 7.39 – 7.45 (2H, m, Ar^{2}C(2,6)*H*), 7.52 – 7.58 (1H, m, Ar^{1}C(4)*H*), 7.58 – 7.63 (2H, m, Ar^{2}C(3,5)*H*), 8.01 – 8.06 (2H, m, Ar^{1}C(2,6)*H*); **^{19}F{^{1}H} NMR** (470 MHz, CD_3CN) δ_F: –69.8 (s, C*F*$_3$); **^{13}C{^{1}H} NMR** (126 MHz,

CD_3CN) δ_C: 12.6 (CH_3), 33.3 (q, *J* 31.0, *C*(6)H), 35.8 (*C*(5)H_2), 96.5 (CH), 127.5 (q, *J* 277.4, CF_3), 129.1 (Ar^2*C*(2,6)H), 129.5 (Ar^{1}C(3,5)H), 130.3 (Ar^2*C*(3,5)H), 130.6 (Ar^{1}C(2,6)H), 134.5 (Ar^1*C*(4)H), 135.3 (Ar^1*C*(1)), 140.0 (Ar^{2}C(4)), 145.7 (Ar^{2}C(1)), 158.4 (*C*=N), 166.8 (*C*(2)), 169.7 (*C*(4)), 172.2 (*C*-O), 177.5 (*C*O); **^{77}Se{^{1}H} NMR** (95 MHz, CD_3CN) δ_{Se}: 495.0 (s, C*Se*); **HRMS** (ESI^-) $C_{22}H_{16}O_5N_4F_3S^{80}Se$ $[M-H]^-$ found 584.9960, requires 584.9964 (–0.7 ppm).

(*R*,*Z*)-*N*-(3-(4-Methoxyphenyl)-4-oxo-6-(trifluoromethyl)-1,3-selenazinan-2-ylidene)benzamide (57)

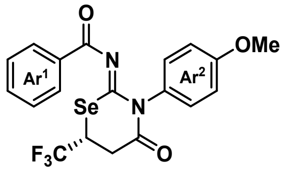

57

Following General Procedure G, perfluorophenyl (*E*)-4,4,4-trifluorobut-2-enoate **28** (138 mg, 0.45 mmol, 1.5 equiv.), *N*-((4-methoxyphenyl)carbamoselenoyl)benzamide **S35** (100 mg, 0.30 mmol, 1.0 equiv.), and (2*S*,3*R*)-HyperBTM **21** (9.3 mg, 0.03 mmol, 10 mol%) in anhydrous THF (0.1 M) for 24 h at 0 °C gave crude product that was purified by flash silica column chromatography (hexane:CH_2Cl_2 60:40 to 0:100, R_F 0.38 at 0:100) to give the title compound as a colourless solid (133 mg, 97%). **mp** 100 – 102 °C; $[\alpha]_D^{20}$ +27.5 (*c* 1.0 in $CHCl_3$); **Chiral HPLC analysis,** Chiralpak IA (80:20 *n*-hexane : IPA, flow rate 1.0 mLmin^{-1}, 220 nm, 30 °C) t_R (*S*) 10.8 min, t_R (*R*) 12.1 min, 98:2 er; **IR** ν_{max} (film) 2959, 1715, 1632, 1508, 1476, 1364, 1325, 1248, 1233, 1153, 1103, 1086, 1045, 1024, 889, 791; **^{1}H NMR** (500 MHz, $CDCl_3$) δ_H: 3.54 (1H, dd, *J* 16.7, 5.0, C(5)H^AH^B), 3.64 (1H, dd, *J* 16.7, 5.3, C(5)H^AH^B), 3.77 (1H, qdd, *J* 9.0, 5.3, 5.0, C(6)*H*), 3.88 (3H, s, OCH_3), 7.02 – 7.06 (2H, m, Ar^{2}C(3,5)*H*), 7.07 – 7.12 (2H, m, Ar^{2}C(2,6)*H*), 7.31 – 7.37 (2H, m, Ar^{1}C(3,5)*H*), 7.46 – 7.52 (1H, m, Ar^{1}C(4)*H*), 7.78 – 7.83 (2H, m, Ar^{1}C(2,6)*H*); **^{19}F{^{1}H} NMR** (376 MHz, $CDCl_3$) δ_F: –69.1 (s, CF_3); **^{13}C{^{1}H} NMR** (126 MHz, $CDCl_3$) δ_C: 32.7 (q, *J* 31.6, *C*(6)H), 35.5 (*C*(5)H_2), 55.6 (OCH_3), 114.6 (Ar^2*C*(3,5)H), 126.0 (q, *J* 278.1, CF_3), 128.5 (Ar^{1}C(3,5)H), 129.0 (Ar^{2}C(2,6)H), 130.3 (Ar^{1}C(2,6)H), 132.1 (Ar^{2}C(1)), 133.6 (Ar^1*C*(4)H), 134.6 (Ar^1*C*(1)), 159.4 (Ar^{2}C(4)), 165.0 (*C*(2)), 168.4 (*C*(4)), 177.2 (CO); **^{77}Se{^{1}H} NMR** (95 MHz, $CDCl_3$) δ_{Se}: 500.0

(s, C*Se*); **HRMS** (ESI^+) $C_{19}H_{15}O_3N_2F_3Na^{80}Se$ $[M+Na]^+$ found 479.0093, requires 479.0092 (+0.2 ppm).

(*R*,*Z*)-4-Methoxy-*N*-(4-oxo-3-phenyl-6-(trifluoromethyl)-1,3-selenazinan-2-ylidene)benzamide (58)

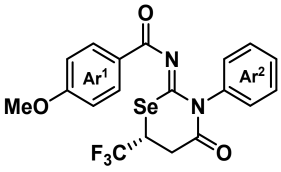

58

Following General Procedure G, perfluorophenyl (*E*)-4,4,4-trifluorobut-2-enoate **28** (138 mg, 0.45 mmol, 1.5 equiv.), 4-methoxy-*N*-(phenylcarbamoselenoyl)benzamide **S32** (100 mg, 0.30 mmol, 1.0 equiv.), and (2*S*,3*R*)-HyperBTM **21** (9.3 mg, 0.03 mmol, 10 mol%) in anhydrous THF (0.1 M) for 24 h at 0 °C gave crude product that was purified by flash silica column chromatography (hexane:CH_2Cl_2 80:20 to 0:100, R_F 0.34 at 0:100) to give the title compound as a colourless solid (110 mg, 80%). **mp** 156 – 158 °C; $[\alpha]_D^{20}$ +20.0 (*c* 0.5 in $CHCl_3$); **Chiral HPLC analysis,** Chiralpak IB (90:10 *n*-hexane : IPA, flow rate 1.0 mLmin^{-1}, 211 nm, 30 °C) t_R (*S*) 30.6 min, t_R (*R*) 37.3 min, 99:1 er; **IR** ν_{max} (film) 2359, 1717, 1597, 1510, 1489, 1312, 1260, 1236, 1152, 1101, 1084, 849; **^{1}H NMR** (400 MHz, $CDCl_3$)

δ_H: 3.55 (1H, dd, *J* 16.6, 5.0, C(5)H^AH^B), 3.65 (1H, dd, *J* 16.6, 5.3, C(5)H^AH^B), 3.71 – 3.84 (4H, m, C(6)*H* and OCH_3), 6.75 – 6.81 (2H, m, Ar^{1}C(3,5)*H*), 7.14 – 7.20 (2H, m, Ar^{2}C(2,6)*H*), 7.43 – 7.56 (3H, m, Ar^{2}C(3,5)*H* and Ar^{2}C(4)*H*), 7.66 – 7.72 (2H, m, Ar^{1}C(2,6)*H*); **^{19}F{^{1}H} NMR** (377 MHz, $CDCl_3$) δ_F: –69.0 (s, CF_3); **^{13}C{^{1}H} NMR** (126 MHz, $CDCl_3$) δ_C: 32.8 (q, *J* 31.5, *C*(6)H), 35.6 (*C*(5)H_2), 55.6 (O*C*H_3), 113.8 (Ar^1*C*(3,5)H), 126.1 (q, *J* 278.1, *C*F_3), 127.4 (Ar^1*C*(1)), 128.1 (Ar^2*C*(2,6)H), 128.5 (Ar^2*C*(4)H), 129.4 (Ar^2*C*(3,5)H), 132.6 (Ar^1*C*(2,6)H), 139.8 (Ar^2*C*(1)), 163.7 (*C*(2)), 164.1 (Ar^{1}C(4)), 168.2 (*C*(4)), 176.3 (*C*O); **^{77}Se{^{1}H} NMR** (95 MHz, $CDCl_3$) δ_{Se}: 496.9 (s, C*Se*); **HRMS** (ESI^+) $C_{19}H_{15}O_3N_2F_3Na^{80}Se$ $[M+Na]^+$ found 479.0091, requires 479.0092 (–0.2 ppm).

(*R*,*Z*)-4-Bromo-*N*-(4-oxo-3-phenyl-6-(trifluoromethyl)-1,3-selenazinan-2-ylidene)benzamide (59)

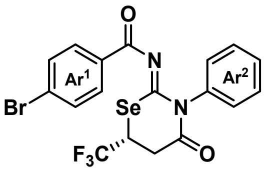

59

Following General Procedure G, perfluorophenyl (*E*)-4,4,4-trifluorobut-2-enoate **28** (138 mg, 0.45 mmol, 1.5 equiv.), 4-bromo-*N*-(phenylcarbamoselenoyl)benzamide **S33** (115 mg, 0.30 mmol, 1.0 equiv.), and (2*S*,3*R*)-HyperBTM **21** (9.3 mg, 0.03 mmol, 10 mol%) in anhydrous THF (0.1 M) for 24 h at 0 °C gave crude product that was purified by flash silica column chromatography (hexane:CH_2Cl_2 100:0 to 0:100, R_F 0.63 at 0:100) to give the title compound as a colourless glass (139 mg, 92%). $[\alpha]_D^{20}$ +17.9 (*c* 0.7 in $CHCl_3$); **Chiral HPLC analysis,** Chiralpak IA (80:20 *n*-hexane : IPA, flow rate 1.0 $mLmin^{-1}$, 254 nm, 30 °C) t_R (*S*) 11.1 min, t_R (*R*) 12.5 min, 98:2 er; **IR** ν_{max} (film) 1715, 1630, 1587, 1489, 1474, 1362, 1325, 1231, 1165, 1153, 1103, 1084, 1045, 1010, 762; **^{1}H NMR** (400 MHz, $CDCl_3$) δ_H: 3.55 (1H, dd, *J* 16.7, 5.1, C(5)H^AH^B), 3.66 (1H, dd, *J* 16.7, 5.1, C(5)H^AH^B), 3.80 (1H, qdd, *J* 8.8, 5.1, 5.1, C(6)*H*), 7.13 – 7.18 (2H, m, Ar^2C(2,6)*H*), 7.41 – 7.60 (7H, m, Ar^1C(2,6)*H*, Ar^1C(3,5)*H*, Ar^2C(3,5)*H* and Ar^2C(4)*H*); **^{19}F{^{1}H} NMR** (377 MHz, $CDCl_3$) δ_F: –69.1 (s, C*F*$_3$); **^{13}C{^{1}H} NMR** (126 MHz, $CDCl_3$) δ_C: 32.8 (q, *J* 31.5, *C*(6)H), 35.4 (*C*(5)H_2), 126.0 (q, *J* 278.3, CF_3), 127.9 (Ar^2*C*(2,6)H), 128.6 (Ar^2*C*(4)H), 128.8 (Ar^1C(4)), 129.4 (Ar^2*C*(3,5)H), 131.7 (Ar^1*C*(2,6)H), 131.8 (Ar^1C(3,5)H), 133.5 (Ar^1C(1)), 139.5 (Ar^2*C*(1)), 165.6 (*C*(2)), 168.1 (*C*(4)), 176.2 (*C*O); **^{77}Se{^{1}H} NMR** (95 MHz, $CDCl_3$) δ_{Se}: 500.7 (s, C*Se*); **HRMS** (ESI^+) $C_{18}H_{12}O_2N_2{}^{79}BrF_3Na^{80}Se$ $[M+Na]^+$ found 526.9088, requires 526.9092 (–0.8 ppm).

(*R*,*Z*)-4-Nitro-*N*-(4-oxo-3-phenyl-6-(trifluoromethyl)-1,3-selenazinan-2-ylidene)benzamide (60)

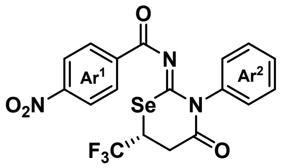

60

Following General Procedure G, perfluorophenyl (*E*)-4,4,4-trifluorobut-2-enoate **28** (138 mg, 0.45 mmol, 1.5 equiv.), 4-nitro-*N*-(phenylcarbamoselenoyl)benzamide **S34** (104 mg, 0.30 mmol, 1.0 equiv.), and (2*S*,3*R*)-HyperBTM **21** (9.3 mg, 0.03 mmol, 10 mol%) in anhydrous THF (0.1 M) for 24 h at 0 °C gave crude product that was purified by flash silica column chromatography (hexane:CH_2Cl_2 70:30 to 0:100, R_F 0.51 at 0:100) to give the title compound as a pale yellow solid (118 mg, 84%). **mp** 86 – 88 °C; $[\alpha]_D^{20}$ +23.4 (*c* 0.5 in $CHCl_3$); **Chiral HPLC analysis,** Chiralpak IA (80:20 *n*-hexane : IPA, flow rate 1.0 mLmin^{-1}, 211 nm, 30 °C) t_R (*S*) 13.8 min, t_R (*R*) 15.8 min, 97:3 er; **IR** ν_{max} (film) 2359, 1717, 1636, 1597, 1522, 1458, 1362, 1329, 1231, 1153, 1103, 1088, 1045, 854; **^{1}H NMR** (400 MHz, $CDCl_3$) δ_H: 3.58 (1H, dd, *J* 16.8, 5.1, C(5)H^AH^B), 3.69 (1H, dd, *J* 16.8, 5.1, C(5)H^AH^B), 3.85 (1H, qdd, *J* 8.8, 5.1, 5.1, C(6)*H*), 7.13 – 7.19 (2H, m, Ar^{2}C(2,6)*H*), 7.47 – 7.57 (3H, m, Ar^{2}C(3,5)*H* and Ar^{2}C(4)*H*), 7.80 – 7.86 (2H, m, Ar^{1}C(2,6)*H*), 8.09 – 8.15 (2H, m, Ar^{1}C(3,5)*H*); **^{19}F{^{1}H} NMR** (377 MHz, $CDCl_3$) δ_F: –69.1 (s, C*F*$_3$); **^{13}C{^{1}H} NMR** (126 MHz, $CDCl_3$) δ_C: 33.0 (q, *J* 31.8, *C*(6)H), 35.3 (*C*(5)H_2), 123.6 (Ar^1*C*(3,5)H), 125.9 (q, *J* 278.3, CF_3), 127.9 (Ar^2*C*(2,6)H), 128.9 (Ar^2*C*(4)H), 129.6 (Ar^2*C*(3,5)H), 131.1 (Ar^1*C*(2,6)H), 139.4 (Ar^1*C*(1)), 139.8 (Ar^2*C*(1)), 150.5 (Ar^1*C*(4)), 167.6 (*C*(2)), 167.9 (*C*(4)), 175.0 (*C*O); **^{77}Se{^{1}H} NMR** (95 MHz, $CDCl_3$) δ_{Se}: 504.4 (s, C*Se*); **HRMS** (ESI^+) $C_{18}H_{12}O_4N_3F_3Na^{80}Se$ $[M+Na]^+$ found 493.9837, requires 493.9837 (±0.0 ppm).

Ethyl (*R*,*Z*)-2-(benzoylimino)-4-oxo-3-phenyl-1,3-selenazinane-6-carboxylate (61)

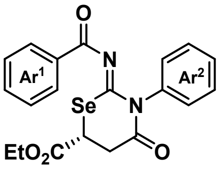

61

Following General Procedure G, ethyl perfluorophenyl fumarate **S16** (140 mg, 0.45 mmol, 1.5 equiv.), *N*-(phenylcarbamoselenoyl)benzamide **S31** (91.0 mg, 0.30 mmol, 1.0 equiv.), and (2*S*,3*R*)-HyperBTM **21** (9.3 mg, 0.03 mmol, 10 mol%) in anhydrous THF (0.1 M) for 24 h at RT gave crude product that was purified by flash silica column chromatography (hexane:EtOAc 100:0 to 50:50, R_F 0.48 at 60:40) to give the title compound as a colourless solid (92 mg, 71%). **mp** 88 – 90 °C; $[\alpha]_D^{20}$ –46.2 (*c* 0.5 in CH_2Cl_2); **Chiral HPLC analysis,** Chiralpak IA (80:20 *n*-hexane : IPA, flow rate 1.0 mLmin^{-1}, 211 nm, 30 °C) t_R (*S*) 12.1 min, t_R (*R*) 18.8 min, 93:7 er; **IR** ν_{max} (film) 2980, 1721, 1628, 1614, 1597, 1576, 1493, 1470, 1452, 1371, 1331, 1308, 1233, 1209, 1190, 1169, 1090, 1049, 1022, 991, 858, 806, 779; ^{1}**H NMR** (500 MHz, $CDCl_3$) δ_H: 1.30 (3H, t, *J* 7.1, C*H*$_3$), 3.48 (1H, dd, *J* 16.5, 4.1, C(5)H^AH^B), 3.61 (1H, dd, *J* 16.5, 5.6, C(5)H^AH^B), 3.95 (1H, dd, *J* 5.6, 4.1, C(6)*H*), 4.26 (2H, q, *J* 7.1, C*H*$_2$CH$_3$), 7.21 – 7.25 (2H, m, Ar^{2}C(2,6)*H*), 7.27 – 7.32 (2H, m, Ar^{1}C(3,5)*H*), 7.43 – 7.48 (2H, m, Ar^{1}C(4)*H* and Ar^{2}C(4)*H*), 7.50 – 7.55 (2H, m, Ar^{2}C(3,5)*H*), 7.71 – 7.75 (2H, m, Ar^{1}C(2,6)*H*); ^{13}**C{^{1}H} NMR** (126 MHz, $CDCl_3$) δ_C: 14.2 (*C*H$_3$), 31.2 (*C*(6)H), 37.9 (*C*(5)H$_2$), 62.4 (*C*H$_2$CH$_3$), 128.2 (Ar^2*C*(2,6)H), 128.3 (Ar^2*C*(4)H), 128.4 (Ar^1*C*(3,5)H), 129.2 (Ar^2*C*(3,5)H), 130.2 (Ar^1*C*(2,6)H), 133.3 (Ar^1*C*(4)), 134.9 (Ar^1*C*(1)), 140.0 (Ar^2*C*(1)), 166.0 (*C*(2)), 169.7 (*C*(4)), 171.4 (*C*(O)O), 177.0 (N*C*O); ^{77}**Se{^{1}H} NMR** (95 MHz, $CDCl_3$) δ_{Se}: 560.2 (s, C*Se*); **HRMS** (ESI^+) $C_{20}H_{18}O_4N_2Na^{80}Se$ $[M+Na]^+$ found 453.0320, requires 453.0324 (–0.9 ppm).

(*S*,*Z*)-*N*-(6-Methyl-4-oxo-3-phenyl-1,3-selenazinan-2-ylidene)benzamide (62)

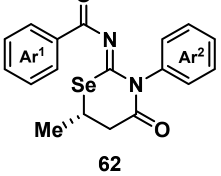

62

Following General Procedure G, 4-nitrophenyl (*E*)-but-2-enoate **S5** (93.2 mg, 0.45 mmol, 1.5 equiv.), *N*-(phenylcarbamoselenoyl)benzamide **S31** (91.0 mg, 0.30 mmol, 1.0 equiv.),

and (2*S*,3*R*)-HyperBTM **21** (27.8 mg, 0.09 mmol, 30 mol%) in anhydrous THF (0.1 M) for 48 h at RT gave crude product that was purified, directly without aqueous workup, by flash silica column chromatography (hexane:EtOAc 100:0 to 50:50, R_F 0.35 at 70:30) to give the title compound as a colourless solid (32 mg, 29%). **mp** 115 – 117 °C; $[\alpha]_D^{20}$ –70.0 (*c* 0.5 in $CHCl_3$); **Chiral HPLC analysis,** Chiralpak IA (90:10 *n*-hexane : IPA, flow rate 1.0 mLmin^{-1}, 211 nm, 30 °C) t_R (*R*) 16.0 min, t_R (*S*) 17.5 min, 87:13 er; **IR** ν_{max} (film) 3063, 2957, 2922, 1705, 1632, 1472, 1352, 1314, 1219, 1165, 1059, 1013, 716, 694; **^{1}H NMR** (500 MHz, $CDCl_3$) δ_H: 1.61 (3H, d, *J* 6.9, CH_3), 3.23 (1H, dd, *J* 16.0, 10.2, C(5)H^AH^B), 3.48 (1H, dd, *J* 16.0, 3.2, C(5)H^AH^B), 3.62 (1H, dqd, *J* 10.2, 6.9, 3.2, C(6)*H*), 7.17 – 7.22 (2H, m, Ar^{2}C(2,6)*H*), 7.26 – 7.32 (2H, m, Ar^{1}C(3,5)*H*), 7.42 – 7.48 (2H, m, Ar^{1}C(4)*H* and Ar^{2}C(4)*H*), 7.50 – 7.55 (2H, m, Ar^{2}C(3,5)*H*), 7.70 – 7.75 (2H, m, Ar^{1}C(2,6)*H*); **^{13}C{^{1}H} NMR** (126 MHz, $CDCl_3$) δ_C: 20.8 (CH_3), 26.4 (*C*(6)H), 44.8 (*C*(5)H_2), 128.3 (Ar^2*C*(4)H), 128.4 (Ar^1*C*(3,5)H and Ar^2*C*(2,6)H), 129.3 (Ar^2*C*(3,5)H), 130.1 (Ar^1*C*(2,6)H), 133.1 (Ar^1*C*(4)), 135.2 (Ar^1*C*(1)), 140.0 (Ar^2*C*(1)), 165.7 (*C*(2)), 171.2 (*C*(4)), 176.8 (*C*O); **^{77}Se{^{1}H} NMR** (95 MHz, $CDCl_3$) δ_{Se}: 543.3 (s, C*Se*); **HRMS** (ESI^+) $C_{18}H_{16}O_2N_2Na^{80}Se$ $[M+Na]^+$ found 395.0269, requires 395.0269 (±0.0 ppm).

(*R*$_a$,*Z*)-*N*-(3-(2-*iso*-Propylphenyl)-4-oxo-1,3-thiazinan-2-ylidene)-4-methylbenzenesulfonamide (63)

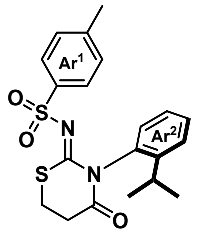

63

Following General Procedure G, perfluorophenyl acrylate **75** (107 mg, 0.45 mmol, 1.5 equiv.), *N*-((2-*iso*-propylphenyl)carbamothioyl)-4-methylbenzenesulfonamide **S29** (105 mg, 0.30 mmol, 1.0 equiv.), and (2*S*,3*R*)-HyperBTM **21** (4.6 mg, 0.015 mmol, 5 mol%) in anhydrous THF (0.1 M) for 24 h at 0 °C gave crude product that was purified by flash silica column chromatography (CH_2Cl_2:Et_2O 100:0 to 90:10, R_F 0.32 at 90:10) to give the title compound as a colourless solid (101 mg, 83%). **mp** 151 – 153 °C; $[\alpha]_D^{20}$ +51.2 (*c* 0.5 in $CHCl_3$); **Chiral HPLC analysis,** Chiralpak IB (80:20 *n*-hexane : IPA, flow rate 1.0

mLmin^{-1}, 211 nm, 30 °C) t_R (R_a) 26.0 min, t_R (S_a) 31.5 min, 87:13 er; **IR** ν_{max} (film) 2965, 1717, 1491, 1341, 1314, 1291, 1256, 1150, 1088, 993, 908, 835, 787; **^{1}H NMR** (400 MHz, $CDCl_3$) δ_H: 0.97 (3H, d, *J* 6.8, $CH(CH_3)^A(CH_3)^B$), 1.10 (3H, d, *J* 6.8, $CH(CH_3)^A(CH_3)^B$), 2.33 (3H, s, CH_3), 2.55 (1H, h, *J* 6.8, $CH(CH_3)_2$), 3.15 – 3.34 (4H, m, C(5)H_2 and C(6)H_2), 6.87 – 6.95 (1H, m, Ar^{2}C(6)*H*), 7.07 – 7.13 (2H, m, Ar^{1}C(3,5)*H*), 7.17 – 7.24 (1H, m, Ar^{2}C(5)*H*), 7.30 – 7.42 (4H, m, Ar^{2}C(3)*H*, Ar^{2}C(4)*H* and Ar^{1}C(2,6)*H*); **^{13}C{^{1}H} NMR** (126 MHz, $CDCl_3$) δ_C: 21.5 (CH_3), 23.1 ($CH(CH_3)^A(CH_3)^B$), 23.3 ($CH(CH_3)^A(CH_3)^B$), 23.8 (*C*(6)H_2), 28.6 ($CH(CH_3)_2$), 34.4 (*C*(5)H_2), 126.5 (Ar^1*C*(2,6)H), 126.7 (Ar^2*C*(3)H), 126.7 (Ar^2*C*(6)H), 128.1 (Ar^2*C*(5)H), 129.1 (Ar^1*C*(3,5)H), 129.4 (Ar^2*C*(4)H), 134.7 (Ar^2*C*(1)), 138.2 (Ar^1*C*(1)), 143.2 (Ar^1*C*(4)), 145.6 (Ar^2*C*(2)), 164.3 (*C*(2)), 169.1 (*C*(4)); **HRMS** (ESI^+) $C_{20}H_{22}O_3N_2NaS_2$ $[M+Na]^+$ found 425.0967, requires 425.0964 (+0.7 ppm).

(R_a,*Z*)-*N*-(3-([1,1'-Biphenyl]-2-yl)-4-oxo-1,3-thiazinan-2-ylidene)-4-methylbenzenesulfonamide (64)

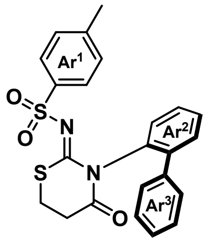

64

Following General Procedure G, perfluorophenyl acrylate **75** (107 mg, 0.45 mmol, 1.5 equiv.), *N*-([1,1'-biphenyl]-2-ylcarbamothioyl)-4-methylbenzenesulfonamide **S30** (115 mg, 0.30 mmol, 1.0 equiv.), and (2*S*,3*R*)-HyperBTM **21** (4.6 mg, 0.015 mmol, 5 mol%) in anhydrous THF (0.1 M) for 24 h at 0 °C gave crude product that was purified by flash silica column chromatography (hexane:EtOAc 100:0 to 0:100, R_F 0.23 at 50:50) to give the title compound as a colourless glass (128 mg, 98%). $[\alpha]_D^{20}$ +3.2 (*c* 0.5 in $CHCl_3$); **Chiral HPLC analysis,** Chiralpak IB (80:20 *n*-hexane : IPA, flow rate 1.0 mLmin^{-1}, 211 nm, 30 °C) t_R (R_a) 31.9 min, t_R (S_a) 41.2 min, 73:27 er; **IR** ν_{max} (film) 1717, 1597, 1497, 1435, 1342, 1315, 1288, 1150, 1088, 993, 907, 837, 787; **^{1}H NMR** (400 MHz, $CDCl_3$) δ_H: 2.41 (3H, s, CH_3), 2.49 – 2.61 (1H, m, C(6)H^AH^B), 2.81 – 3.02 (3H, m, C(5)H_2 and C(6)H^AH^B), 6.93 – 6.99 (2H, m, Ar^{3}C(2,6)*H*), 7.13 – 7.35 (7H, m, Ar^{1}C(3,5)*H*, Ar^{2}C(3,6)*H*, and Ar^{3}C(3,4,5)*H*), 7.39 – 7.49 (2H, m, Ar^{2}C(4,5)*H*), 7.49 – 7.55 (2H, m, Ar^{1}C(2,6)*H*); **^{13}C{^{1}H} NMR** (126 MHz, $CDCl_3$) δ_C: 21.6

(CH_3), 22.9 (C(6)H_2), 34.3 (C(5)H_2), 126.8 (Ar^1C(2,6)H), 127.8 (Ar^3C(4)H), 128.2 (Ar^3C(3,5)H), 128.2 (Ar^3C(2,6)H), 128.4 (Ar^2C(5)H), 129.0 (Ar^2C(4)H), 129.2 (Ar^1C(3,5)H), 129.4 (Ar^2C(6)H), 130.5 (Ar^2C(3)H), 135.1 (Ar^2C(1)), 138.1 (Ar^1C(1)), 138.4 (Ar^3C(1)), 139.9 (Ar^2C(2)), 143.4 (Ar^1C(4)), 165.1 (C(2)), 169.0 (C(4)); **HRMS** (ESI^+) $C_{23}H_{20}O_3N_2NaS_2$ $[M + Na]^+$ found 459.0807, requires 459.0808 (–0.2 ppm).

(R_a,Z)-N-(3-(2-(*tert*-Butyl)phenyl)-4-oxo-1,3-thiazinan-2-ylidene)-4-methylbenzenesulfonamide (65)

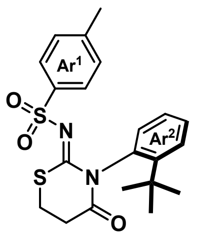

65

Following General Procedure G, perfluorophenyl acrylate **75** (107 mg, 0.45 mmol, 1.5 equiv.), N-((2-(*tert*-butyl)phenyl)carbamothioyl)-4-methylbenzenesulfonamide **74** (109 mg, 0.30 mmol, 1.0 equiv.), and (2S,3R)-HyperBTM **21** (18.5 mg, 0.06 mmol, 20 mol%) in anhydrous THF (0.1 M) for 48 h at RT gave crude product that was purified by flash silica column chromatography (hexane:EtOAc 100:0 to 0:100, R_F 0.23 at 60:40) to give the title compound as a colourless glass (91 mg, 73%). $[\alpha]_D^{20}$ –34.0 (c 0.5 in CH_2Cl_2); **Chiral HPLC analysis,** Chiralpak IB (60:40 n-hexane : IPA, flow rate 1.0 mLmin^{-1}, 211 nm, 30 °C) t_R (R_a) 12.1 min, t_R (S_a) 15.2 min, 80:20 er; **IR** ν_{max} (film) 2965, 1719, 1516, 1495, 1437, 1341, 1288, 1250, 1171, 1150, 1088, 991, 905, 827, 787; **^{1}H NMR** (400 MHz, $CDCl_3$) δ_H: 1.20 (9H, s, C(CH_3)$_3$), 2.34 (3H, s, CH_3), 3.11 – 3.25 (3H, m, C(5)H^AH^B and C(6)H_2), 3.27 – 3.38 (1H, m, C(5)H^AH^B), 6.80 (1H, dd, J 7.8, 1.5, Ar^2C(6)H), 7.07 – 7.13 (2H, m, Ar^1C(3,5)H), 7.21 (1H, ddd, J 7.8, 7.2, 1.5, Ar^2C(5)H), 7.33 (1H, ddd, J 8.1, 7.2, 1.5, Ar^2C(4)H), 7.36 – 7.40 (2H, m, Ar^1C(2,6)H), 7.49 (1H, dd, J 8.1, 1.5, Ar^2C(3)H); **^{13}C{^{1}H} NMR** (126 MHz, $CDCl_3$) δ_C: 21.6 (CH_3), 22.7 (C(6)H_2), 31.6 (C(CH_3)$_3$), 34.7 (C(5)H_2), 35.9 (C(CH_3)$_3$), 126.5 (Ar^1C(2,6)H), 127.1 (Ar^2C(5)H), 128.9 (Ar^2C(4)H), 129.1 (Ar^1C(3,5)H), 129.1 (Ar^2C(6)H), 131.3 (Ar^2C(3)H), 134.8 (Ar^2C(1)), 138.4 (Ar^1C(1)), 143.2 (Ar^1C(4)), 145.8 (Ar^2C(2)), 164.8 (C(2)), 169.7 (C(4)); **HRMS** (ESI^+) $C_{21}H_{24}O_3N_2NaS_2$ $[M + Na]^+$ found 439.1117, requires 439.1121 (–0.9 ppm).

(R_a,Z)-N-(3-(2-*iso*-Propylphenyl)-4-oxo-1,3-selenazinan-2-ylidene)benzamide (66)

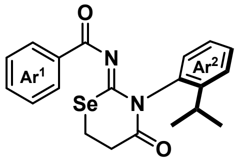

66

Following General Procedure G, perfluorophenyl acrylate **75** (107 mg, 0.45 mmol, 1.5 equiv.), *N*-((2-*iso*-propylphenyl)carbamoselenoyl)benzamide **S37** (104 mg, 0.30 mmol, 1.0 equiv.), and (2*S*,3*R*)-HyperBTM **21** (18.5 mg, 0.06 mmol, 20 mol%) in anhydrous THF (0.1 M) for 48 h at RT gave crude product that was purified by flash silica column chromatography (hexane:CH_2Cl_2 100:0 to 0:100, R_F 0.25 at 25:75) to give the title compound as a colourless solid (85 mg, 71%). **mp** 129 – 131 °C; $[\alpha]_D^{20}$ +35.0 (*c* 0.2 in $CHCl_3$); **Chiral HPLC analysis,** Chiralpak IB (80:20 *n*-hexane : IPA, flow rate 1.0 mLmin^{-1}, 254 nm, 30 °C) t_R (R_a) 8.3 min, t_R (S_a) 9.7 min, 77:23 er; **IR** ν_{max} (film) 2961, 1705, 1630, 1576, 1472, 1323, 1306, 1229, 1153, 1080, 1065, 1020, 762; **^{1}H NMR** (500 MHz, $CDCl_3$) δ_H: 1.11 (3H, d, *J* 6.9, $CH(CH_3)^A(CH_3)^B$), 1.18 (3H, d, *J* 6.9, $CH(CH_3)^A(CH_3)^B$), 2.73 (1H, h, *J* 6.9, $CH(CH_3)_2$), 3.02 – 3.17 (2H, m, C(6)H_2), 3.40 – 3.48 (2H, m, C(5)H_2), 7.03 – 7.08 (1H, m, Ar^{2}C(6)*H*), 7.24 – 7.34 (3H, m, Ar^{1}C(3,5)*H* and Ar^{2}C(5)*H*), 7.40 – 7.49 (3H, m, Ar^{1}C(4)*H* and Ar^{2}C(3,4)*H*), 7.67 – 7.72 (2H, m, Ar^{1}C(2,6)*H*); **^{13}C{^{1}H} NMR** (126 MHz, $CDCl_3$) δ_C: 15.1 (*C*(6)H_2), 23.5 ($CH(CH_3)^A(CH_3)^B$), 23.9 ($CH(CH_3)^A(CH_3)^B$), 28.8 ($CH(CH_3)_2$), 36.7 (*C*(5)H_2), 126.6 (Ar^2*C*(4)H), 126.7 (Ar^2*C*(5)H), 128.0 (Ar^2*C*(6)H), 128.3 (Ar^1*C*(3,5)H), 129.0 (Ar^2*C*(3)H), 130.1 (Ar^1*C*(2,6)H), 133.1 (Ar^1*C*(4)H), 135.2 (Ar^1*C*(1)), 137.8 (Ar^2*C*(1)), 145.3 (Ar^2*C*(2)), 166.3 (*C*(2)), 171.3 (*C*(4)), 176.7 (*C*O); **^{77}Se{^{1}H} NMR** (95 MHz, $CDCl_3$) δ_{Se}: 464.9 (s, C*Se*); **HRMS** (ESI^+) $C_{20}H_{20}O_2N_2Na^{80}Se$ $[M+Na]^+$ found 423.0581, requires 423.0582 (–0.2 ppm).

(R,R_a,Z)-N-(3-(2-*iso*-Propylphenyl)-4-oxo-6-(trifluoromethyl)-1,3-thiazinan-2-ylidene)-4-methylbenzenesulfonamide (67) and (S,R_a,Z)-N-(3-(2-*iso*-propylphenyl)-4-oxo-6-(trifluoromethyl)-1,3-thiazinan-2-ylidene)-4-methylbenzenesulfonamide (S41)

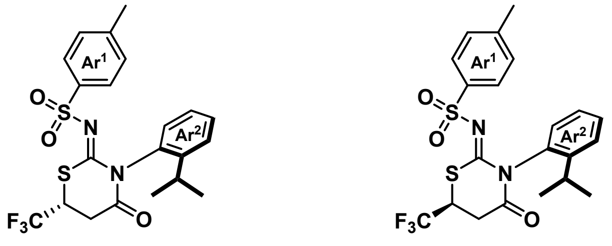

Following General Procedure G, perfluorophenyl (E)-4,4,4-trifluorobut-2-enoate **28** (138 mg, 0.45 mmol, 1.5 equiv.), N-((2-*iso*-propylphenyl)carbamothioyl)-4-methylbenzenesulfonamide **S29** (105 mg, 0.30 mmol, 1.0 equiv.), and (2S,3R)-HyperBTM **21** (4.6 mg, 0.015 mmol, 5 mol%) in anhydrous THF (0.1 M) for 24 h at 0 °C gave crude product that was purified by flash silica column chromatography (hexane:CH_2Cl_2 50:50 to 0:100, R_F 0.23 at 0:100) to give the title compound (92:8 dr) as a colourless solid (133 mg, 94%). **mp** 45 – 47 °C; $[\alpha]_D^{20}$ +94.5 (c 1.0 in $CHCl_3$); **IR** ν_{max} (film) 2967, 1719, 1518, 1508, 1362, 1319, 1277, 1229, 1152, 1119, 1088, 999, 826, 772; **HRMS** (ESI^+) $C_{21}H_{21}O_3N_2F_3NaS_2$ $[M+Na]^+$ found 493.0840, requires 493.0838 (+0.4 ppm).

Data for major diastereoisomer (R,R_a,Z)-**67**: **Chiral HPLC analysis,** Chiralpak AD-H (80:20 n-hexane : IPA, flow rate 1.0 mLmin^{-1}, 254 nm, 30 °C) t_R (R,R_a) 7.8 min, t_R (S,S_a) 17.5 min, 98:2 er; **^{1}H NMR** (400 MHz, $CDCl_3$) δ_H: 1.00 (3H, d, J 6.8, CH(CH_3)^A(CH$_3$)^B), 1.11 (3H, d, J 6.8, CH(CH$_3$)^A(CH_3)^B), 2.38 (3H, s, CH_3), 2.55 (1H, h, J 6.8, CH(CH$_3$)$_2$), 3.45 (2H, d, J 4.8, C(5)H_2), 4.05 (1H, qt, J 8.0, 4.8, C(6)H), 6.79 – 6.84 (1H, m, Ar^{2}C(6)H), 7.13 – 7.19 (2H, m, Ar^{1}C(3,5)H), 7.20 – 7.25 (1H, m, Ar^{2}C(5)H), 7.34 – 7.41 (2H, m, Ar^{2}C(3)H and Ar^{2}C(4)H), 7.42 – 7.46 (2H, m, Ar^{1}C(2,6)H); **^{19}F{^{1}H} NMR** (377 MHz, $CDCl_3$) δ_F: –71.0 (s, CF_3); **^{13}C{^{1}H} NMR** (126 MHz, $CDCl_3$) δ_C: 21.5 (CH$_3$), 23.2 (CH(CH$_3$)^A(CH$_3$)^B), 23.7 (CH(CH$_3$)^A(CH$_3$)^B), 28.7 (CH(CH$_3$)$_2$), 33.0 (C(5)H$_2$), 39.9 (q, J 32.5, C(6)H), 124.6 (q, J 280.4, CF$_3$), 126.6 (Ar^{1}C(2,6)H), 126.7 (Ar^{2}C(3)H), 126.9 (Ar^{2}C(6)H), 127.0 (Ar^{2}C(5)H), 129.2 (Ar^1C(3,5)H), 129.6 (Ar^2C(4)H), 134.3 (Ar^{2}C(1)), 137.5 (Ar^{1}C(1)), 143.7 (Ar^{1}C(4)), 146.1 (Ar^{2}C(2)), 159.7 (C(2)), 165.6 (C(4)).

Data for minor diastereoisomer (S,R_a,Z)-**S41**: **Chiral HPLC analysis,** er not determined; **^{1}H NMR** (400 MHz, $CDCl_3$) (*selected*) δ_H: 0.93 (3H, d, J 6.9, CH(CH_3)^A(CH$_3$)^B), 1.09 (3H, d,

J 6.9, CH(CH$_3$)^A(C*H*$_3$)^B), 4.10 – 4.17 (1H, m, C(6)*H*), 6.89 – 6.93 (1H, m, Ar^{2}C(5)*H*); **^{19}F{^{1}H} NMR** (377 MHz, CDCl$_3$) δ$_F$: –71.4 (s, C*F*$_3$); **^{13}C{^{1}H} NMR** (126 MHz, CDCl$_3$) (*selected*) δ$_C$: 22.6 (CH(*C*H$_3$)^A(CH$_3$)^B), 23.7 (CH(CH$_3$)^A(*C*H$_3$)^B), 28.1 (*C*H(CH$_3$)$_2$), 32.7 (*C*(5)H$_2$), 128.0 (Ar^2*C*(5)H), 134.3 (Ar^2*C*(1)), 137.6 (Ar^1*C*(1)), 145.8 (Ar^2*C*(2)), 159.5 (*C*(2)), 165.9 (*C*(4)).

(*R*,*R*$_a$,*Z*)-*N*-(3-([1,1'-Biphenyl]-2-yl)-4-oxo-6-(trifluoromethyl)-1,3-thiazinan-2-ylidene)-4-methylbenzenesulfonamide (68) and (*R*,*S*$_a$,*Z*)-*N*-(3-([1,1'-biphenyl]-2-yl)-4-oxo-6-(trifluoromethyl)-1,3-thiazinan-2-ylidene)-4-methylbenzenesulfonamide (S42)

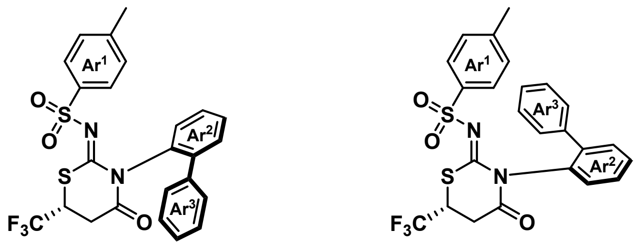

Following General Procedure G, perfluorophenyl (*E*)-4,4,4-trifluorobut-2-enoate **28** (138 mg, 0.45 mmol, 1.5 equiv.), *N*-([1,1'-biphenyl]-2-ylcarbamothioyl)-4-methylbenzenesulfonamide **S30** (115 mg, 0.30 mmol, 1.0 equiv.), and (2*S*,3*R*)-HyperBTM **21** (4.6 mg, 0.015 mmol, 5 mol%) in anhydrous THF (0.1 M) for 24 h at 0 °C gave crude product (90:10 dr) that was purified by flash silica column chromatography (hexane:CH$_2$Cl$_2$ 100:0 to 0:100, R$_F$ 0.30 at 0:100) to give the title compound as an inseparable mixture of diastereoisomers (88:12 dr) as a colourless glass (140 mg, 93%). $[\alpha]_D^{20}$ +57.8 (*c* 0.5 in CHCl$_3$); **IR** ν$_{max}$ (film) 2955, 1724, 1520, 1362, 1321, 1279, 1227, 1153, 1121, 1088, 1001, 957, 887, 826, 773; **HRMS** (ESI^+) C$_{24}$H$_{19}$O$_3$N$_2$F$_3$NaS$_2$ $[M+Na]^+$ found 527.0679, requires 527.0681 (–0.4 ppm).

Data for major diastereoisomer (*R*,*R*$_a$,*Z*)-**68**: **Chiral HPLC analysis,** Chiralcel OD-H (70:30 *n*-hexane : IPA, flow rate 1.0 mLmin^{-1}, 211 nm, 30 °C) t$_R$ (*S*,*S*$_a$) 7.8 min, t$_R$ (*R*,*R*$_a$) 18.5 min, 95:5 er; **^{1}H NMR** (400 MHz, CDCl$_3$) δ$_H$: 2.43 (3H, s, C*H*$_3$), 2.91 (1H, dd, *J* 16.7, 4.8, C(5)*H*^{A}H^B), 3.19 (1H, dd, *J* 16.7, 6.3, C(5)H^A*H*^{B}), 3.53 – 3.65 (1H, m, C(6)*H*), 6.96 – 7.01 (2H, m, Ar^{3}C(2,6)*H*), 7.20 – 7.37 (7H, m, Ar^{1}C(3,5)*H*, Ar^{2}C(3,6)*H*, and Ar^{3}C(3,4,5)*H*), 7.39 – 7.50 (2H, m, Ar^{2}C(4,5)*H*), 7.52 – 7.58 (2H, m, Ar^{1}C(2,6)*H*); **^{19}F{^{1}H} NMR** (377 MHz, CDCl$_3$) δ$_F$: –71.0 (s, C*F*$_3$); **^{13}C{^{1}H} NMR** (126 MHz, CDCl$_3$) δ$_C$: 21.6 (*C*H$_3$), 33.2 (*C*(5)H$_2$), 40.0 (q, *J* 32.4, *C*(6)H), 124.2 (q, *J* 280.1, *C*F$_3$), 126.9 (Ar^1*C*(2,6)H), 127.9 (Ar^3*C*(4)H), 128.1

(Ar^3*C*(3,5)H), 128.3 (Ar^3*C*(2,6)H), 128.4 (Ar^2*C*(5)H), 128.7 (Ar^2*C*(4)H), 129.4 (Ar^1*C*(3,5)H), 129.4 (Ar^2*C*(6)H), 130.6 (Ar^2*C*(3)H), 134.8 (Ar^2*C*(1)), 137.5 (Ar^1*C*(1)), 138.3 (Ar^3*C*(1)), 140.3 (Ar^2*C*(2)), 144.0 (Ar^1*C*(4)), 160.1 (*C*(2)), 165.8 (*C*(4)).

Data for minor diastereoisomer (*R*,*S*$_a$,*Z*)-**S42**: **Chiral HPLC analysis,** Chiralcel OD-H (70:30 *n*-hexane : IPA, flow rate 1.0 mLmin^{-1}, 211 nm, 30 °C) t_R (*S*,*R*$_a$) 14.0 min, t_R (*R*,*S*$_a$) 33.2 min, 63:37 er; **^{1}H NMR** (400 MHz, $CDCl_3$) (*selected*) δ_H: 3.02 (1H, dd, *J* 16.6, 6.4, C(5)H^AH^B), 3.93 – 4.05 (1H, m, C(6)*H*), 7.04 – 7.08 (2H, m, Ar^{3}C(2,6)*H*); **^{19}F{^{1}H} NMR** (377 MHz, $CDCl_3$) δ_F: –71.6 (s, C*F*$_3$); **^{13}C{^{1}H} NMR** (126 MHz, $CDCl_3$) (*selected*) δ_C: 32.6 (*C*(5)H_2), 127.8 (Ar^3*C*(4)H), 128.1 (Ar^3*C*(3,5)H), 128.6 (Ar^2*C*(4)H), 129.2 (Ar^2*C*(6)H), 131.2 (Ar^2*C*(3)H), 137.6 (Ar^1*C*(1)), 137.9 (Ar^3*C*(1)), 141.0 (Ar^2*C*(2)), 143.9 (Ar^1*C*(4)), 160.0 (*C*(2)), 166.0 (*C*(4)).

(*R*,*R*$_a$,*Z*)-*N*-(3-(2-(*tert*-Butyl)phenyl)-4-oxo-6-(trifluoromethyl)-1,3-thiazinan-2-ylidene)-4-methylbenzenesulfonamide (69) and (*S*,*R*$_a$,*Z*)-*N*-(3-(2-(*tert*-butyl)phenyl)-4-oxo-6-(trifluoromethyl)-1,3-thiazinan-2-ylidene)-4-methylbenzenesulfonamide (S43)

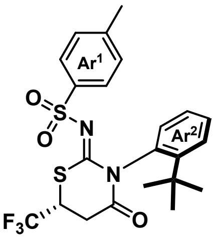

69

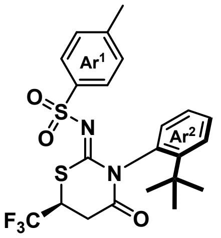

S43

Following General Procedure G, perfluorophenyl (*E*)-4,4,4-trifluorobut-2-enoate **28** (138 mg, 0.45 mmol, 1.5 equiv.), *N*-((2-(*tert*-butyl)phenyl)carbamothioyl)-4-methylbenzenesulfonamide **74** (109 mg, 0.30 mmol, 1.0 equiv.), and (2*S*,3*R*)-HyperBTM **21** (4.6 mg, 0.015 mmol, 5 mol%) in anhydrous THF (0.1 M) for 48 h at RT gave crude product that was purified by flash silica column chromatography (hexane:EtOAc 100:0 to 50:50, R_F 0.60 at 60:40) to give the title compound as an inseparable mixture of diastereoisomers (88:12 dr) as a colourless glass (80 mg, 55%). $[\alpha]_D^{20}$ +48.6 (*c* 0.5 in $CHCl_3$); **IR** ν_{max} (film) 2961, 1722, 1522, 1508, 1362, 1319, 1281, 1227, 1153, 1119, 1088, 999, 957, 887, 826, 770; **HRMS** (ESI^+) $C_{22}H_{23}O_3N_2F_3NaS_2$ $[M+Na]^+$ found 507.0993, requires 507.0994 (–0.2 ppm).

Data for major diastereoisomer (*R*,*R*$_a$,*Z*)-**69**: **Chiral HPLC analysis,** Chiralpak IB (90:10 *n*-hexane : IPA, flow rate 1.0 mLmin^{-1}, 211 nm, 30 °C) t_R (*R*,*R*$_a$) 15.8 min, t_R (*S*,*S*$_a$) 20.3 min,

97:3 er; **^{1}H NMR** (500 MHz, $CDCl_3$) δ_H: 1.19 (9H, s, C(CH_3)$_3$), 2.36 (3H, s, CH_3), 3.31 (1H, dd, *J* 16.9, 5.2, C(5)H^AH^B), 3.42 (1H, dd, *J* 16.9, 5.1, C(5)H^AH^B), 4.01 (1H, qdd, *J* 7.7, 5.2, 5.1, C(6)*H*), 6.67 (1H, dd, *J* 7.9, 1.4, Ar^{2}C(6)*H*), 7.12 – 7.16 (2H, m, Ar^{1}C(3,5)*H*), 7.19 (1H, ddd, *J* 7.9, 7.2, 1.5, Ar^{2}C(5)*H*), 7.33 (1H, ddd, *J* 8.3, 7.2, 1.5, Ar^{2}C(4)*H*), 7.40 – 7.44 (2H, m, Ar^{1}C(2,6)*H*), 7.50 (1H, dd, *J* 8.3, 1.5, Ar^{2}C(3)*H*); **^{19}F{^{1}H} NMR** (377 MHz, $CDCl_3$) δ_F: –71.1 (s, CF_3); **^{13}C{^{1}H} NMR** (126 MHz, $CDCl_3$) δ_C: 21.6 (CH_3), 31.6 (C(CH_3)$_3$), 33.6 (*C*(5)H_2), 36.2 (*C*(CH_3)$_3$), 40.0 (q, *J* 32.5, *C*(6)H), 124.5 (q, *J* 280.3, *C*F_3), 126.7 (Ar^1*C*(2,6)H), 127.4 (Ar^2*C*(5)H), 129.2 (Ar^2*C*(4)H), 129.3 (Ar^1*C*(3,5)H), 129.5 (Ar^2*C*(6)H), 129.9 (Ar^2*C*(3)H), 134.0 (Ar^2*C*(1)), 137.7 (Ar^1*C*(1)), 143.7 (Ar^1*C*(4)), 146.4 (Ar^2*C*(2)), 160.2 (*C*(2)), 166.5 (*C*(4)).

Data for minor diastereoisomer (*S*,*R*$_a$,*Z*)-**S43**: **Chiral HPLC analysis,** Chiralpak IB (90:10 *n*-hexane : IPA, flow rate 1.0 mLmin^{-1}, 211 nm, 30 °C) t_R (*S*,*R*$_a$) 26.1 min, t_R (*R*,*S*$_a$) 40.6 min, 53:47 er; **^{1}H NMR** (500 MHz, $CDCl_3$) (*selected*) δ_H: 1.15 (9H, s, C(CH_3)$_3$), 4.16 – 4.26 (1H, m, C(6)*H*), 7.36 – 7.39 (2H, m, Ar^{1}C(2,6)*H*); **^{19}F{^{1}H} NMR** (377 MHz, $CDCl_3$) δ_F: –70.7 (s, CF_3); **^{13}C{^{1}H} NMR** (126 MHz, $CDCl_3$) (*selected*) δ_C: 31.2 (C(CH_3)$_3$), 35.9 (*C*(CH_3)$_3$), 126.6 (Ar^1*C*(2,6)H), 129.2 (Ar^1*C*(3,5)H), 130.2 (Ar^2*C*(3)H), 134.8 (Ar^2*C*(1)), 137.8 (Ar^1*C*(1)), 147.0 (Ar^2*C*(2)), 159.9 (*C*(2)), 166.9 (*C*(4)).

(*R*,*R*$_a$,*Z*)-*N*-(3-(2-*iso*-Propylphenyl)-4-oxo-6-(trifluoromethyl)-1,3-selenazinan-2-ylidene)benzamide (70)

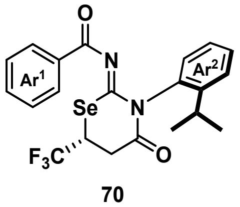

70

Following General Procedure G, perfluorophenyl (*E*)-4,4,4-trifluorobut-2-enoate **28** (138 mg, 0.45 mmol, 1.5 equiv.), *N*-((2-*iso*-propylphenyl)carbamoselenoyl)benzamide **S37** (104 mg, 0.30 mmol, 1.0 equiv.), and (2*S*,3*R*)-HyperBTM **21** (9.3 mg, 0.03 mmol, 10 mol%) in anhydrous THF (0.1 M) for 48 h at RT gave crude product that was purified by flash silica column chromatography (hexane:CH_2Cl_2 100:0 to 0:100, R_F 0.30 at 25:75) to give the title compound (>95:5 dr) as a colourless glass (120 mg, 86%). $[\alpha]_D^{20}$ +75.7 (*c* 1.0 in $CHCl_3$); **Chiral HPLC analysis,** Chiralpak IB (98:2 *n*-hexane : IPA, flow rate 2.0 mLmin^{-1}, 220 nm, 30 °C) t_R (*S*,*S*$_a$) 8.6 min, t_R (*R*,*R*$_a$) 12.4 min, 98:2 er; **IR** ν_{max} (film) 2967, 1713, 1632, 1495, 1477,

1362, 1323, 1312, 1263, 1233, 1155, 1103, 1045, 1024, 758; **^{1}H NMR** (400 MHz, $CDCl_3$) δ_H: 1.12 (3H, d, *J* 6.9, CH(C*H*$_3$)^A(CH$_3$)^B), 1.18 (3H, d, *J* 6.9, CH(CH$_3$)^A(C*H*$_3$)^B), 2.69 (1H, h, *J* 6.9, C*H*(CH$_3$)$_2$), 3.57 (1H, dd, *J* 16.7, 5.1, C(5)*H*^{A}H^B), 3.67 (1H, dd, *J* 16.7, 4.9, C(5)H^A*H*^{B}), 3.79 (1H, qdd, *J* 8.8, 5.1, 4.9, C(6)*H*), 6.98 – 7.03 (1H, m, Ar^{2}C(6)*H*), 7.27 – 7.35 (3H, m, Ar^{1}C(3,5)*H* and Ar^{2}C(5)*H*), 7.44 – 7.51 (3H, m, Ar^{1}C(4)*H* and Ar^{2}C(3,4)*H*), 7.69 – 7.74 (2H, m, Ar^{1}C(2,6)*H*); **^{19}F{^{1}H} NMR** (377 MHz, $CDCl_3$) δ_F: –69.0 (s, C*F*$_3$); **^{13}C{^{1}H} NMR** (126 MHz, $CDCl_3$) δ_C: 23.6 (CH(*C*H$_3$)^A(CH$_3$)^B), 23.9 (CH(CH$_3$)^A(*C*H$_3$)^B), 28.8 (*C*H(CH$_3$)$_2$), 32.7 (q, *J* 31.6, *C*(6)H), 35.4 (*C*(5)H$_2$), 126.1 (q, *J* 278.2, *C*F$_3$), 126.6 (Ar^2*C*(4)H), 127.0 (Ar^2*C*(5)H), 127.0 (Ar^2*C*(6)H), 128.5 (Ar^1*C*(3,5)H), 129.3 (Ar^2*C*(3)H), 130.3 (Ar^1*C*(2,6)H), 133.6 (Ar^1*C*(4)H), 134.5 (Ar^1*C*(1)), 137.5 (Ar^2*C*(1)), 145.7 (Ar^2*C*(2)), 164.6 (*C*(2)), 168.1 (*C*(4)), 177.1 (*C*O); **^{77}Se{^{1}H} NMR** (95 MHz, $CDCl_3$) δ_{Se}: 496.5 (s, C*Se*); **HRMS** (ESI^+) $C_{21}H_{19}O_2N_2F_3Na^{80}Se$ $[M + Na]^+$ found 491.0459, requires 491.0456 (+0.6 ppm).

(*R*,*R*$_a$,*Z*)-*N*-(6-(Chlorodifluoromethyl)-3-(2-*iso*-propylphenyl)-4-oxo-1,3-thiazinan-2-ylidene)-4-methylbenzenesulfonamide (71)

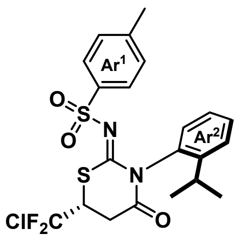

71

Following General Procedure G, 4-nitrophenyl (*E*)-4-chloro-4,4-difluorobut-2-enoate **S4** (125 mg, 0.45 mmol, 1.5 equiv.), *N*-((2-*iso*-propylphenyl)carbamothioyl)-4-methylbenzenesulfonamide **S29** (105 mg, 0.30 mmol, 1.0 equiv.), and (2*S*,3*R*)-HyperBTM **21** (4.6 mg, 0.015 mmol, 5 mol%) in anhydrous THF (0.1 M) for 24 h at 0 °C gave crude product (>95:5 dr) that was purified by flash silica column chromatography (hexane:Et_2O 80:20 until elution of PNPOH then hexane:CH_2Cl_2 50:50 to 0:100, R_F 0.30 at 0:100) to give the title compound (>95:5 dr) as a colourless solid (130 mg, 89%). *Note: a workup was not performed to avoid elimination of HCl.*

mp 53 – 55 °C; $[\alpha]_D^{20}$ +92.6 (*c* 0.5 in $CHCl_3$); **Chiral HPLC analysis,** Chiralpak AD-H (80:20 *n*-hexane : IPA, flow rate 1.0 mLmin^{-1}, 211 nm, 30 °C) t_R (*R*,*R*$_a$) 8.7 min, t_R (*S*,*S*$_a$) 20.3 min, 98:2 er; **IR** ν_{max} (film) 2967, 1722, 1520, 1506, 1354, 1319, 1283, 1206, 1152, 1088, 970, 928,

826, 812, 768; **^{1}H NMR** (400 MHz, $CDCl_3$) δ_H: 0.98 (3H, d, *J* 6.8, CH(CH_3)^A(CH_3)^B), 1.09 (3H, d, *J* 6.8, CH(CH_3)^A(CH_3)^B), 2.35 (3H, s, CH_3), 2.52 (1H, h, *J* 6.8, C*H*(CH_3)$_2$), 3.54 (1H, dd, *J* 17.1, 5.6, C(5)$H^A$$H^B$), 3.54 (1H, dd, *J* 17.1, 4.3, C(5)$H^A$$H^B$), 4.14 (1H, dddd, *J* 12.1, 9.5, 5.6, 4.3, C(6)*H*), 6.77 – 6.82 (1H, m, Ar^{2}C(6)*H*), 7.11 – 7.16 (2H, m, Ar^{1}C(3,5)*H*), 7.17 – 7.22 (1H, m, Ar^{2}C(5)*H*), 7.31 – 7.40 (2H, m, Ar^{2}C(3)*H* and Ar^{2}C(4)*H*), 7.40 – 7.45 (2H, m, Ar^{1}C(2,6)*H*); **^{19}F{^{1}H} NMR** (377 MHz, $CDCl_3$) δ_F: –57.9 (d, *J* 167.1, C$F^A$$F^B$Cl), –56.1 (d, *J* 167.1, C$F^A$$F^B$Cl); **^{13}C{^{1}H} NMR** (126 MHz, $CDCl_3$) δ_C: 21.6 (CH_3), 23.2 (CH(CH_3)^A(CH_3)^B), 23.8 (CH(CH_3)^A(CH_3)^B), 28.7 (*C*H(CH_3)$_2$), 34.0 (*C*(5)H_2), 46.5 (t, *J* 27.7, *C*(6)H), 126.6 (Ar^1*C*(2,6)H), 126.7 (Ar^2*C*(3)H), 127.0 (Ar^2*C*(6)H), 127.0 (Ar^2*C*(5)H), 127.9 (t, *J* 295.0, CF_2Cl), 129.3 (Ar^1*C*(3,5)H), 129.7 (Ar^2*C*(4)H), 134.3 (Ar^2*C*(1)), 137.6 (Ar^1*C*(1)), 143.7 (Ar^1*C*(4)), 146.1 (Ar^2*C*(2)), 160.0 (*C*(2)), 165.7 (*C*(4)); **HRMS** (ESI^+) $C_{21}H_{21}O_3N_2F_2{}^{35}ClNaS_2$ $[M+Na]^+$ found 509.0540, requires 509.0542 (–0.4 ppm).

(*S*,*R*$_a$,*Z*)-*N*-(3-(2-*iso*-Propylphenyl)-6-methyl-4-oxo-1,3-thiazinan-2-ylidene)-4-methylbenzenesulfonamide (72) and (*R*,*R*$_a$,*Z*)-*N*-(3-(2-*iso*-propylphenyl)-6-methyl-4-oxo-1,3-thiazinan-2-ylidene)-4-methylbenzenesulfonamide (S44)

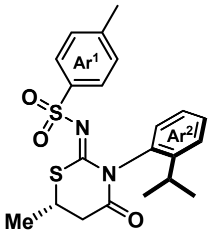

72

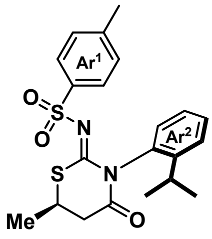

S44

Following General Procedure G, 4-nitrophenyl (*E*)-but-2-enoate **S5** (93.2 mg, 0.45 mmol, 1.5 equiv.), *N*-((2-*iso*-propylphenyl)carbamothioyl)-4-methylbenzenesulfonamide **S29** (105 mg, 0.30 mmol, 1.0 equiv.), and (2*S*,3*R*)-HyperBTM **21** (18.5 mg, 0.06 mmol, 20 mol%) in anhydrous THF (0.1 M) for 24 h at RT gave crude product (88:12 dr) that was purified by flash silica column chromatography (hexane:CH_2Cl_2 100:0 to 0:100, R_F 0.17 at 0:100) to give the title compound as an inseparable mixture of diastereoisomers (88:12 dr) as a colourless oil (102 mg, 82%). $[\alpha]_D^{20}$ –21.2 (*c* 0.5 in $CHCl_3$); **IR** ν_{max} (film) 2965, 1717, 1597, 1506, 1491, 1449, 1348, 1319, 1300, 1150, 1088, 1034, 974, 864, 826, 791; **HRMS** (ESI^+) $C_{21}H_{24}O_3N_2NaS_2$ $[M+Na]^+$ found 439.1118, requires 439.1121 (–0.7 ppm).

Data for major diastereoisomer (S,R_a,Z)-**72**: **Chiral HPLC analysis,** Chiralpak IA (95:5 *n*-hexane : IPA, flow rate 1.0 mLmin^{-1}, 211 nm, 40 °C) t_R (S,R_a) 47.0 min, t_R (R,S_a) 58.5 min, 92:8 er; **^{1}H NMR** (400 MHz, $CDCl_3$) δ_H: 0.98 (3H, d, *J* 6.8, CH(C*H*$_3$)^A(CH$_3$)^B), 1.10 (3H, d, *J* 6.8, CH(CH$_3$)^A(C*H*$_3$)^B), 1.54 (3H, d, *J* 6.8, C(6)HC*H*$_3$), 2.33 (3H, s, C*H*$_3$), 2.58 (1H, h, *J* 6.8, C*H*(CH$_3$)$_2$), 3.00 (1H, dd, *J* 16.3, 10.6, C(5)*H*^{A}H^B), 3.24 (1H, dd, *J* 16.3, 3.2, C(5)H^A*H*^{B}), 3.72 (1H, dqd, *J* 10.6, 6.8, 3.2, C(6)*H*), 6.90 – 6.95 (1H, m, Ar^{2}C(6)*H*), 7.07 – 7.12 (2H, m, Ar^{1}C(3,5)*H*), 7.19 – 7.24 (1H, m, Ar^{2}C(5)*H*), 7.31 – 7.41 (4H, m, Ar^{2}C(3,4)*H* and Ar^{1}C(2,6)*H*); **^{13}C{^{1}H} NMR** (126 MHz, $CDCl_3$) δ_C: 19.9 (C(6)HCH$_3$), 21.5 (*C*H$_3$), 23.0 (CH(*C*H$_3$)^A(CH$_3$)^B), 23.8 (CH(CH$_3$)^A(*C*H$_3$)^B), 28.6 (*C*H(CH$_3$)$_2$), 33.6 (*C*(5)H$_2$), 42.4 (*C*(6)H), 126.4 (Ar^1*C*(2,6)H), 126.6 (Ar^2*C*(3)H), 126.7 (Ar^2*C*(6)H), 128.6 (Ar^2*C*(5)H), 129.1 (Ar^1*C*(3,5)H), 129.4 (Ar^2*C*(4)H), 134.7 (Ar^2*C*(1)), 138.3 (Ar^1*C*(1)), 143.1 (Ar^1*C*(4)), 145.1 (Ar^2*C*(2)), 163.9 (*C*(2)), 168.9 (*C*(4)).

Data for minor diastereoisomer (R,R_a,Z)-**S44**: **Chiral HPLC analysis,** Chiralpak IA (95:5 *n*-hexane : IPA, flow rate 1.0 mLmin^{-1}, 211 nm, 40 °C) t_R (S,S_a) 53.4 min, t_R (R,R_a) 76.0 min, 88:12 er; **^{1}H NMR** (400 MHz, $CDCl_3$) (*selected*) δ_H: 3.00 (1H, dd, *J* 16.3, 10.9, C(5)*H*^{A}H^B), 3.22 (1H, dd, *J* 16.3, 3.2, C(5)H^A*H*^{B}), 6.85 – 6.89 (1H, m, Ar^{2}C(6)*H*); **^{13}C{^{1}H} NMR** (126 MHz, $CDCl_3$) (*selected*) δ_C: 23.1 (CH(*C*H$_3$)^A(CH$_3$)^B), 28.7 (*C*H(CH$_3$)$_2$), 42.3 (*C*(6)H), 126.5 (Ar^1*C*(2,6)H), 126.8 (Ar^2*C*(3)H), 127.6 (Ar^2*C*(6)H), 134.8 (Ar^2*C*(1)), 146.2 (Ar^2*C*(2)), 163.8 (*C*(2)).

(*R*,*R*$_a$,*Z*)-*N*-(6-*iso*-Propyl-3-(2-*iso*-propylphenyl)-4-oxo-1,3-thiazinan-2-ylidene)-4-methylbenzenesulfonamide (73) and (*S*,*R*$_a$,*Z*)-*N*-(6-*iso*-propyl-3-(2-*iso*-propylphenyl)-4-oxo-1,3-thiazinan-2-ylidene)-4-methylbenzenesulfonamide (S45)

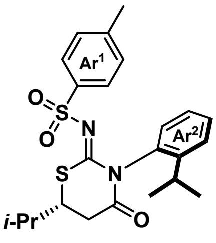

73

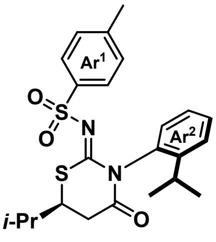

S45

Following General Procedure G, 4-nitrophenyl (*E*)-4-methylpent-2-enoate **S6** (106 mg, 0.45 mmol, 1.5 equiv.), *N*-((2-*iso*-propylphenyl)carbamothioyl)-4-methylbenzenesulfonamide **S29** (105 mg, 0.30 mmol, 1.0 equiv.), and (2*S*,3*R*)-HyperBTM **21** (18.5 mg, 0.06 mmol, 20 mol%) in anhydrous THF (0.1 M) for 24 h at RT gave crude

product (92:8 dr) that was purified by flash silica column chromatography (hexane:CH_2Cl_2 80:20 to 0:100, R_F 0.25 at 0:100) to give the title compound as an inseparable mixture of diastereoisomers (92:8 dr) as a colourless oil (115 mg, 86%). $[\alpha]_D^{20}$ –36.2 (*c* 0.5 in $CHCl_3$); **IR** ν_{max} (film) 2965, 1717, 1516, 1508, 1491, 1339, 1314, 1265, 1150, 1088, 876, 816, 791, 758; **HRMS** (ESI^+) $C_{23}H_{28}O_3N_2NaS_2$ $[M+Na]^+$ found 467.1431, requires 467.1434 (–0.6 ppm).

Data for major diastereoisomer (*R*,R_a,*Z*)-**73**: **Chiral HPLC analysis,** Chiralpak AD-H (90:10 *n*-hexane : IPA, flow rate 1.0 mLmin^{-1}, 211 nm, 30 °C) t_R (*R*,R_a) 33.1 min, t_R (*S*,S_a) 47.1 min, 95:5 er; **^{1}H NMR** (400 MHz, $CDCl_3$) δ_H: 1.00 (3H, d, *J* 6.8, $CH(CH_3)^A(CH_3)^B$), 1.11 (3H, d, *J* 6.8, $CH(CH_3)^A(CH_3)^B$), 1.12 (3H, d, *J* 6.8, $CH(CH_3)^A(CH_3)^B$), 1.14 (3H, d, *J* 6.8, $CH(CH_3)^A(CH_3)^B$), 2.05 (1H, h, *J* 6.8, $CH(CH_3)_2$), 2.33 (3H, s, CH_3), 2.59 (1H, h, *J* 6.8, $CH(CH_3)_2$), 3.06 (1H, dd, *J* 16.1, 10.8, $C(5)H^AH^B$), 3.24 (1H, dd, *J* 16.1, 3.2, $C(5)H^AH^B$), 3.44 (1H, ddd, *J* 10.8, 6.5, 3.2, C(6)*H*), 6.88 – 6.93 (1H, m, $Ar^2C(6)H$), 7.06 – 7.11 (2H, m, $Ar^1C(3,5)H$), 7.18 – 7.24 (1H, m, $Ar^2C(5)H$), 7.31 – 7.40 (4H, m, $Ar^2C(3,4)H$ and $Ar^1C(2,6)H$); **$^{13}C\{^1H\}$ NMR** (126 MHz, $CDCl_3$) δ_C: 19.4 ($CH(CH_3)^A(CH_3)^B$), 19.7 ($CH(CH_3)^A(CH_3)^B$), 21.5 (CH_3), 23.0 ($CH(CH_3)^A(CH_3)^B$), 23.9 ($CH(CH_3)^A(CH_3)^B$), 28.5 ($CH(CH_3)_2$), 31.9 ($CH(CH_3)_2$), 38.3 ($C(5)H_2$), 45.2 (*C*(6)H), 126.4 ($Ar^1C(2,6)H$), 126.6 ($Ar^2C(3)H$), 126.7 ($Ar^2C(6)H$), 128.4 ($Ar^2C(5)H$), 129.0 ($Ar^1C(3,5)H$), 129.3 ($Ar^2C(4)H$), 134.8 ($Ar^2C(1)$), 138.4 ($Ar^1C(1)$), 143.0 ($Ar^1C(4)$), 145.1 ($Ar^2C(2)$), 164.0 (*C*(2)), 169.4 (*C*(4)).

Data for minor diastereoisomer (*S*,R_a,*Z*)-**S45**: **Chiral HPLC analysis,** Chiralpak AD-H (90:10 *n*-hexane : IPA, flow rate 1.0 mLmin^{-1}, 211 nm, 30 °C) t_R (*R*,S_a) 29.1 min, t_R (*S*,R_a) 54.7 min, 68:32 er; **^{1}H NMR** (400 MHz, $CDCl_3$) (*selected*) δ_H: 0.96 (3H, d, *J* 6.8, $CH(CH_3)^A(CH_3)^B$), 3.52 (1H, ddd, *J* 12.0, 6.4, 2.9, C(6)*H*), 6.85 – 6.89 (1H, m, $Ar^2C(6)H$); **$^{13}C\{^1H\}$ NMR** (126 MHz, $CDCl_3$) (*selected*) δ_C: 19.3 ($CH(CH_3)^A(CH_3)^B$), 19.6 ($CH(CH_3)^A(CH_3)^B$), 23.2 ($CH(CH_3)^A(CH_3)^B$), 23.8 ($CH(CH_3)^A(CH_3)^B$), 28.7 ($CH(CH_3)_2$), 31.6 ($CH(CH_3)_2$), 45.3 (*C*(6)H), 126.5 ($Ar^1C(2,6)H$), 126.8 ($Ar^2C(3)H$), 127.4 ($Ar^2C(6)H$), 134.9 ($Ar^2C(1)$), 138.4 ($Ar^1C(1)$), 146.3 ($Ar^2C(2)$), 164.0 (*C*(2)), 169.5 (*C*(4)).

9. Unsuccessful Substrates

Substrates **S46 – S49** could not be synthesised using the methods detailed in Sections 5 and 6.

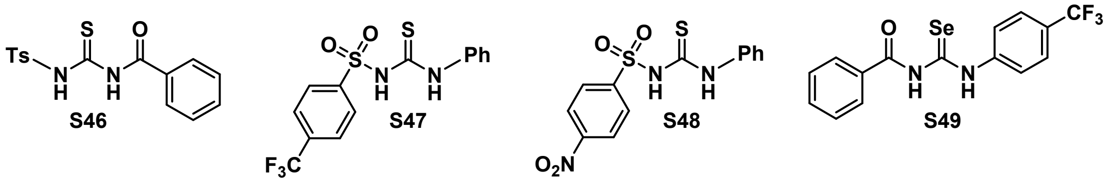

Substrates **S28, S38 and S39** were unreactive under the optimised reaction conditions, returning only starting materials.

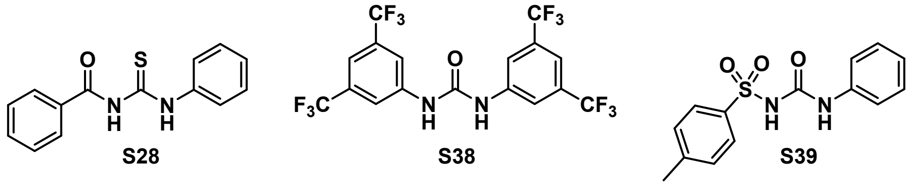

10. Structure Elucidation of Minor Regioisomer

The structure of regioisomeric product **S40** has been elucidated by NMR spectroscopy (Figure S1). Large coupling constants consistent with $^2J_{C\text{-}F}$ and $^3J_{H\text{-}F}$ coupling indicate that the carbon bearing diastereotopic CH_2 protons (•) is adjacent to the perfluoroethyl group. This can only be true for a 5-membered heterocycle with an exocyclic $CH_2C_2F_5$. The ^{13}C chemical shift of ring carbon (• 40.7 ppm) and quaternary carbon (• 152.7 ppm) of **S40** was compared to literature known compounds **S50** (• 42.8 ppm and • 154.2 ppm)[15] and **S51** (• 61.2 ppm and • 179.8 ppm).[16] There was no literature data available for the third possible class of regioisomers **S52**. Good agreement with the ^{13}C chemical shifts of **S50** led to unambiguous assignment of the structure of **S40**.

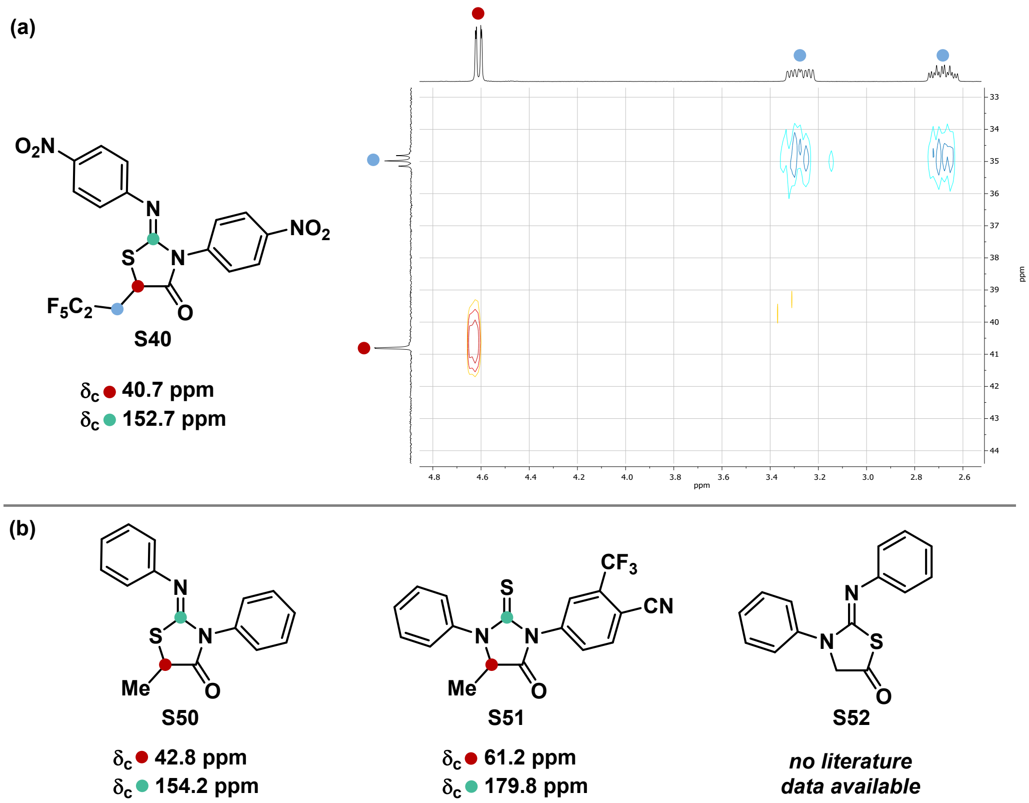

Figure S1 (a) Structural assignment of regioisomeric product **S40** by ^{1}H–^{13}C HSQC NMR and (b) comparison of ^{13}C chemical shifts to known compounds. All chemical shifts were measured in $CDCl_3$.

11. Conformation of Structure by ^{1}H–^{15}N HMBC

The structures of both **46** and **54** were confirmed by ^{1}H–^{15}N HMBC (Figure S2). In each case the diastereotopic protons (•) showed only one cross peak corresponding to the ring nitrogen (•). Any other proton signals that showed a cross peak with the ring nitrogen (•) would then be the substituent on this nitrogen. The methyl protons (•) in **46** showed a cross peak with the ring nitrogen (•), confirming the structure of **46**. Similarly, the *ortho* protons of the 4-nitrophenyl ring (•) in **54** showed a cross peak with the ring nitrogen (•), confirming the structure of **54**.

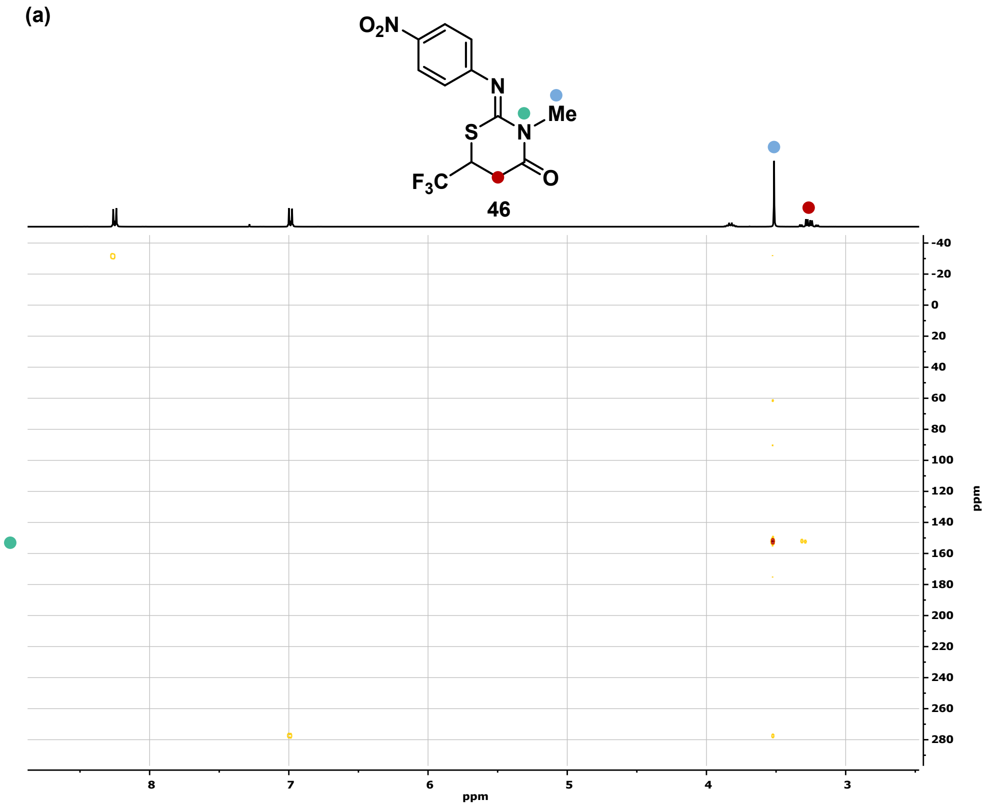

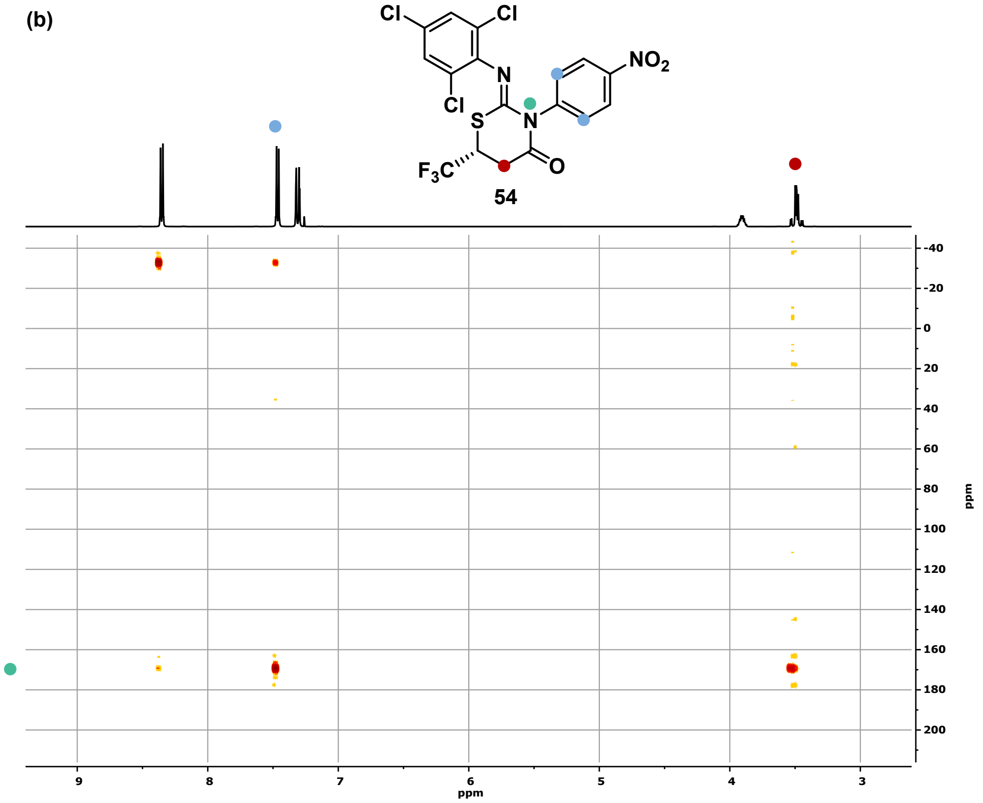

Figure S2 (a) ^{1}H–^{15}N HMBC spectrum of **46** and (b) ^{1}H–^{15}N HMBC spectrum of **54**.

12. Calculation of Configurational Stability

Following the procedure of Armstrong,[17] **63** (20 mg) was dissolved in MeCN (10 mL) and heated to 50 °C. Aliquots (0.3 mL) were taken at defined time points and the er determined by HPLC analysis on a chiral stationary phase (Table S5).

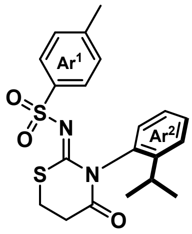

63

Time (s)	ee	ln(1/ee)
0	0.74724	0.291370
1800	0.73276	0.310937
3600	0.71728	0.332289
5400	0.69818	0.359278
7200	0.69006	0.370977
9000	0.67914	0.386928
12600	0.64948	0.431583
19800	0.59322	0.522190
27000	0.55182	0.594533
34200	0.50880	0.675700
86400	0.27864	1.277835

Table S5 Racemisation of **63** at 50 °C in MeCN.

According to Equation S1, a plot of ln(1/ee) vs time afforded a straight line with gradient equal to the rate constant for racemisation (k_{rac}) at this temperature.

$$\ln\left(\frac{1}{ee}\right) = k_{rac}t + \ln\left(\frac{1}{ee_{t\,=\,0}}\right)$$

Equation S1 Kinetic analysis for racemisation.

The graph plotted from the data in Table S5 for the racemisation of **63** is shown in Figure S3.

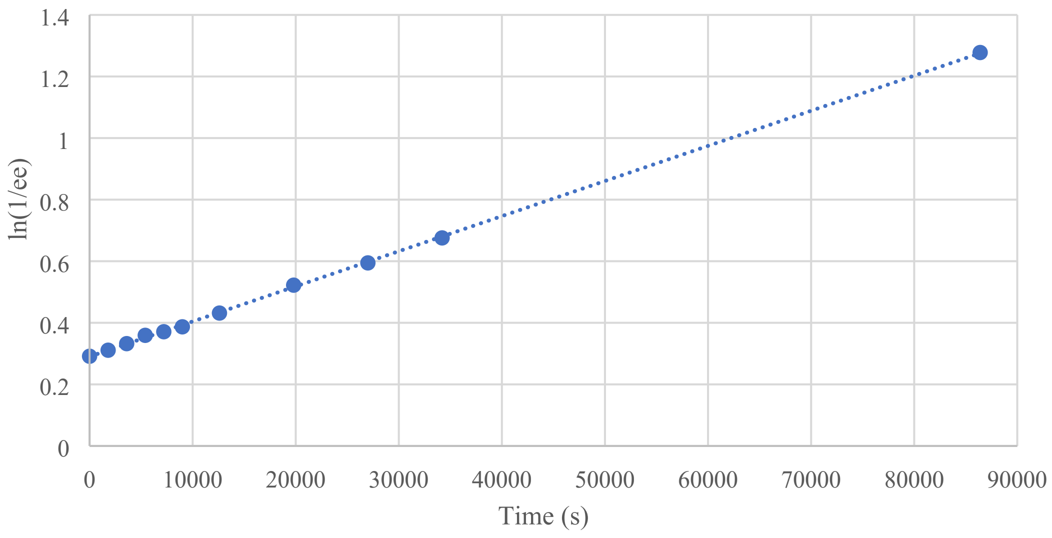

Figure S3 Plot of ln(1/ee) vs time for racemisation of **63**.

From Figure S3: $k_{rac} = gradient = 1.1406 \times 10^{-5}\, s^{-1}$

$$k_{ent} = \frac{k_{rac}}{2} = \frac{1.1406 \times 10^{-5}}{2} = 5.703 \times 10^{-6}\, s^{-1}$$

$$\Delta G^{\ddagger}_{323} = RT\ln\left(\frac{k_B T}{k_{ent} h}\right) = 8.314 \times 323.15 \times \ln\left(\frac{1.381 \times 10^{-23} \times 323.15}{5.703 \times 10^{-6} \times 6.626 \times 10^{-34}}\right)$$

$$\Delta G^{\ddagger}_{323} = 1.118 \times 10^{5}\, J\, mol^{-1} = 26.7 \pm 0.1\, kcal\, mol^{-1}$$

Following the procedure of Armstrong,[17] **64** (20 mg) was dissolved in MeCN (10 mL) and heated to 50 °C. Aliquots (0.3 mL) were taken at defined time points and the er determined by HPLC analysis on a chiral stationary phase (Table S6).

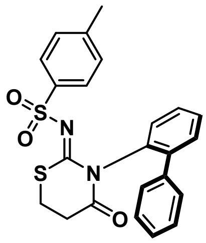

64

Time (s)	ee	ln(1/ee)
0	0.21488	1.537676
1800	0.10596	2.244694
3600	0.05192	2.958051
5400	0.02722	3.603803
7200	0.01340	4.312501
9000	0.00732	4.917145
10800	0.00400	5.521461

Table S6 Racemisation of **64** at 50 °C in MeCN.

The graph plotted from the data in Table S6 for the racemisation of **64** is shown in Figure S4.

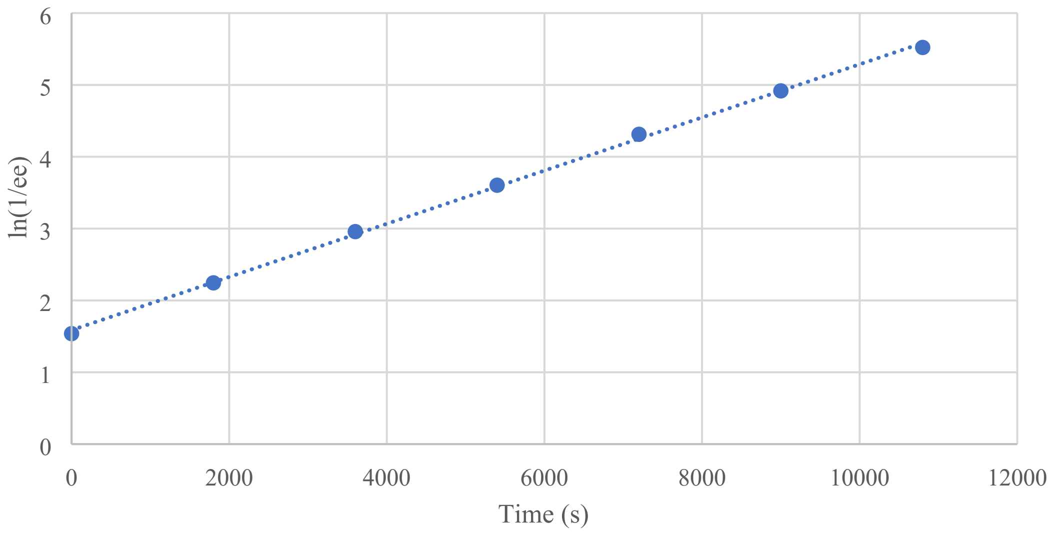

Figure S4 Plot of ln(1/ee) vs time for racemisation of **64**.

From Figure S4: $k_{rac} = gradient = 3.7005 \times 10^{-4}\, s^{-1}$

$$k_{ent} = \frac{k_{rac}}{2} = \frac{3.7005 \times 10^{-4}}{2} = 1.850 \times 10^{-4}\, s^{-1}$$

$$\Delta G^{\ddagger}_{323} = RT\ln\left(\frac{k_B T}{k_{ent} h}\right) = 8.314 \times 323.15 \times \ln\left(\frac{1.381 \times 10^{-23} \times 323.15}{1.850 \times 10^{-4} \times 6.626 \times 10^{-34}}\right)$$

$$\Delta G^{\ddagger}_{323} = 1.025 \times 10^{5}\, J\, mol^{-1} = 24.5 \pm 0.1\, kcal\, mol^{-1}$$

Following the procedure of Armstrong,[17] **65** (20 mg) was dissolved in mesitylene (10 mL) and heated to 120 °C. Aliquots (0.3 mL) were taken at defined time points and the er determined by HPLC analysis on a chiral stationary phase (Table S7).

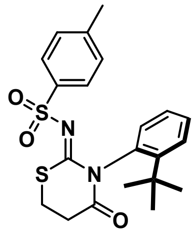

65

Time (s)	ee	ln(1/ee)
0	0.58250	0.540426
1800	0.57108	0.560226
3600	0.56300	0.574476
5400	0.55304	0.592325
9000	0.52972	0.635407
16200	0.50322	0.686728
23400	0.47508	0.744272
30600	0.44534	0.808917
86400	0.25960	1.348613

Table S7 Racemisation of **65** at 120 °C in mesitylene.

The graph plotted from the data in Table S7 for the racemisation of **65** is shown in Figure S5.

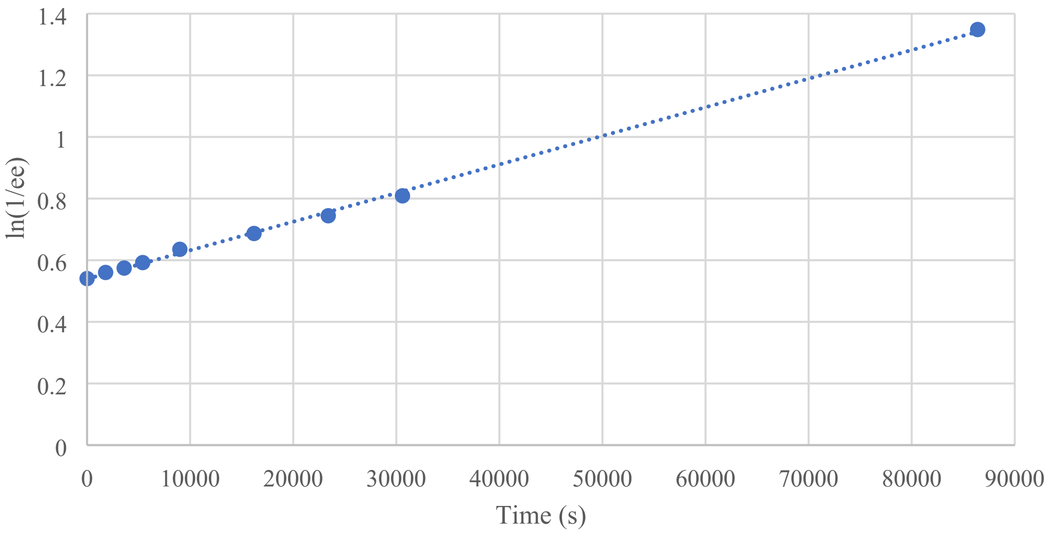

Figure S5 Plot of ln(1/ee) vs time for racemisation of **65**.

From Figure S5: $k_{rac} = gradient = 9.2813 \times 10^{-6}\, s^{-1}$

$$k_{ent} = \frac{k_{rac}}{2} = \frac{9.2813 \times 10^{-6}}{2} = 4.641 \times 10^{-6}\, s^{-1}$$

$$\Delta G^{\ddagger}_{393} = RT\ln\left(\frac{k_B T}{k_{ent} h}\right) = 8.314 \times 393.15 \times \ln\left(\frac{1.381 \times 10^{-23} \times 393.15}{4.641 \times 10^{-6} \times 6.626 \times 10^{-34}}\right)$$

$$\Delta G^{\ddagger}_{393} = 1.373 \times 10^{5}\, J\, mol^{-1} = 32.8 \pm 0.1\, kcal\, mol^{-1}$$

Following the procedure of Armstrong,[17] **66** (20 mg) was dissolved in MeCN (10 mL) and heated to 50 °C. Aliquots (0.3 mL) were taken at defined time points and the er determined by HPLC analysis on a chiral stationary phase (Table S8).

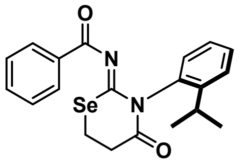

66

Time (s)	ee	ln(1/ee)
0	0.44606	0.807302
1800	0.40798	0.896537
3600	0.37000	0.994252
5400	0.34116	1.075404
7200	0.31204	1.164624
9000	0.28442	1.257303
12600	0.23702	1.439611
16200	0.19638	1.627704
19800	0.16160	1.822631
27000	0.10966	2.210371
34200	0.07418	2.601261

Table S8 Racemisation of **66** at 50 °C in MeCN.

The graph plotted from the data in Table S8 for the racemisation of **66** is shown in Figure S6.

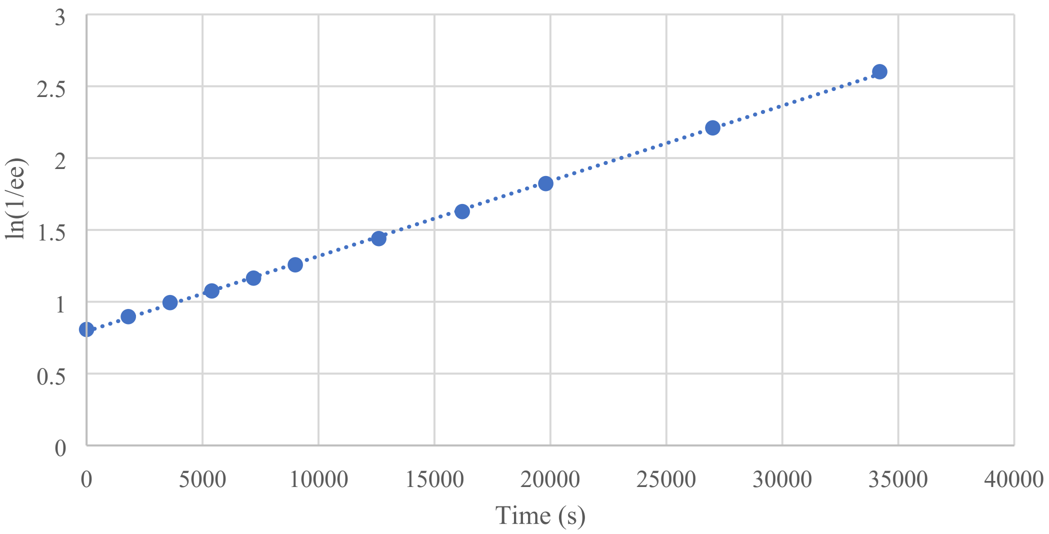

Figure S6 Plot of ln(1/ee) vs time for racemisation of **66**.

From Figure S6: $k_{rac} = gradient = 5.2356 \times 10^{-5}\ s^{-1}$

$$k_{ent} = \frac{k_{rac}}{2} = \frac{5.2356 \times 10^{-5}}{2} = 2.618 \times 10^{-5}\ s^{-1}$$

$$\Delta G^{\ddagger}_{323} = RT\ln\left(\frac{k_B T}{k_{ent} h}\right) = 8.314 \times 323.15 \times \ln\left(\frac{1.381 \times 10^{-23} \times 323.15}{2.618 \times 10^{-5} \times 6.626 \times 10^{-34}}\right)$$

$$\Delta G^{\ddagger}_{323} = 1.077 \times 10^{5}\ J\ mol^{-1} = 25.7 \pm 0.1\ kcal\ mol^{-1}$$

13. Configuration of Major Diastereoisomer Determined by ^{1}H NOESY

The absolute configuration of (6*R*,*R*$_a$,*Z*)-**67** was supported by single crystal X-ray crystallography. However, since the crystal used to obtain the X-ray structure was grown from a mixture of diastereoisomers, it was not known if the structure of the major or minor diastereoisomer was obtained. Unfortunately, HPLC analysis of the crystal from which the X-ray structure was obtained did not yield a UV trace and so was unsuccessful. The relative configuration of the major diastereoisomer of atropisomeric product **72** was determined by ^{1}H NOESY NMR.The coincident methyl peak for both diastereoisomers was irradiated and a response for the C(6) aromatic proton from only the major diastereoisomer was obtained. A reciprocal NOE was also obtained when the C(6) proton of the major diastereoisomer was irradiated. Assuming the stereoselectivity of the reaction does not change, the crystal structure obtained was of the major diastereoisomer of **67**.

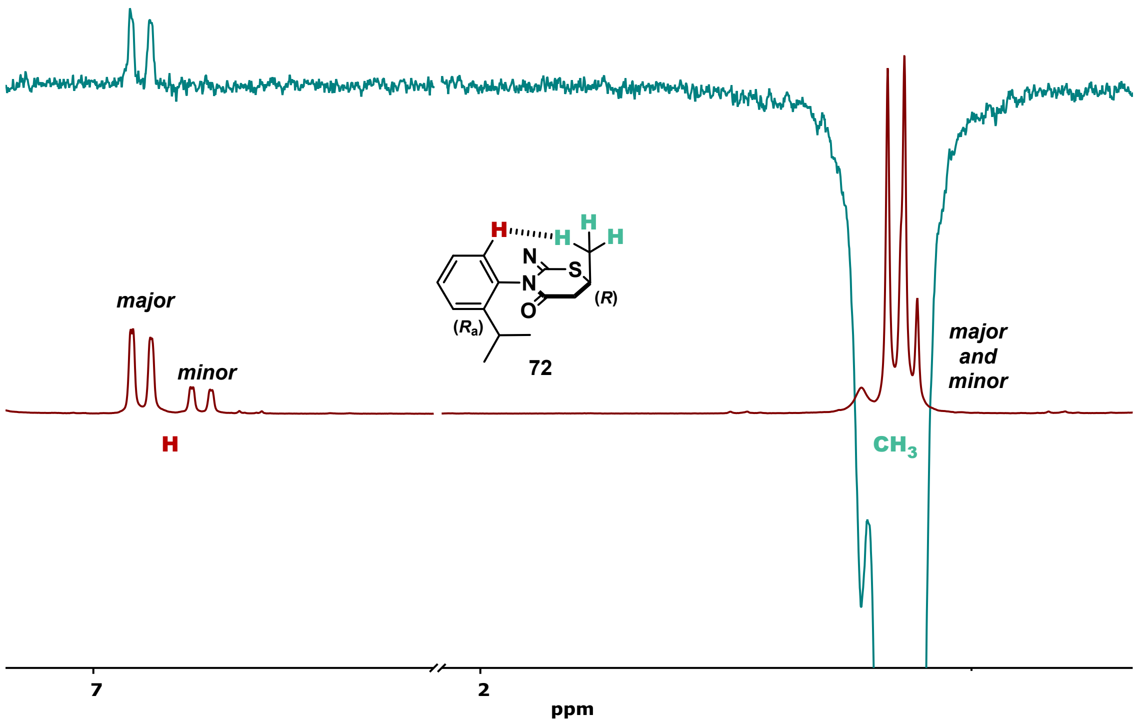

Figure S7 Top: ^{1}H NOESY NMR of **72** and bottom: ^{1}H NMR of **72**. Tosyl substituent omitted for clarity.

14. Determining the Configuration of Minor Diastereoisomers

The configuration of the major enantiomer within the minor diastereoisomer of the atropisomeric products was determined by HPLC analysis on a chiral stationary phase. A sample of known dr and er was heated to facilitate epimerisation via rotation around the hindered axis. It cannot be guaranteed that a diastereomeric ratio can be accurately determined by HPLC. However, for the following samples the initial dr determined by HPLC was consistent with that obtained by ^{1}H NMR analysis.

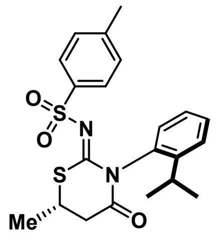

(*S*,*R*$_a$)-72

A sample of (S,R_a)-**72** with 87:13 dr, 92:8 er_{major}, and 88:12 er_{minor} (Figure S8) was heated at 70 °C for 1.5 h. Once complete, the sample now had 74:26 dr, 90:10 er_{major}, and 40:60 er_{minor} (Figure S9). The reduction in dr was expected due to epimerisation. The major enantiomer within the minor diastereoisomer was changed and thus assigned as (S,S_a), coming from the epimerisation of (S,R_a). The major enantiomer within the minor diastereoisomer in the initial mixture was therefore assigned (R,R_a).

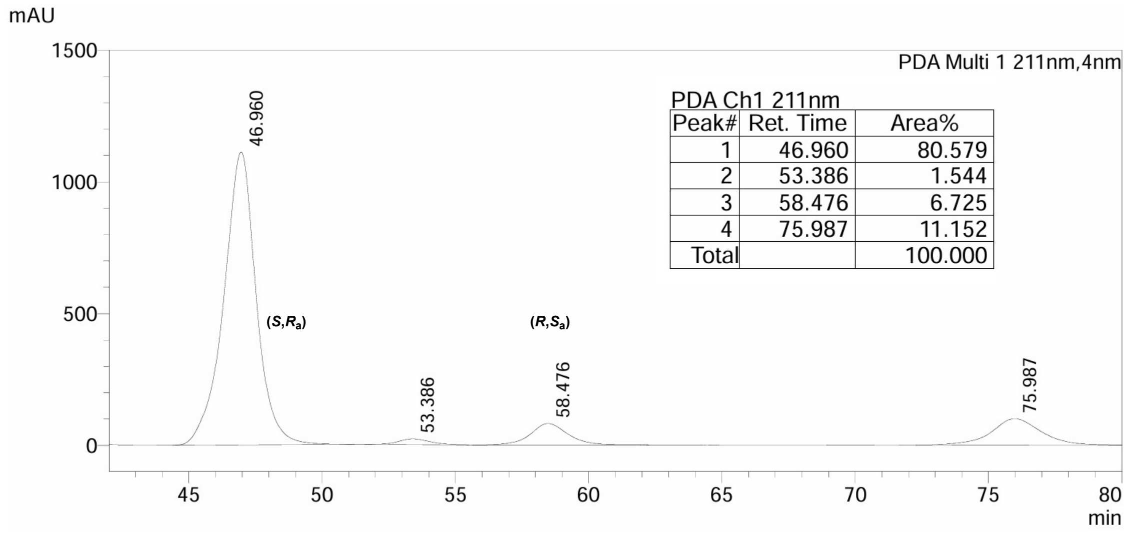

Figure S8 HPLC of **72** before heating.

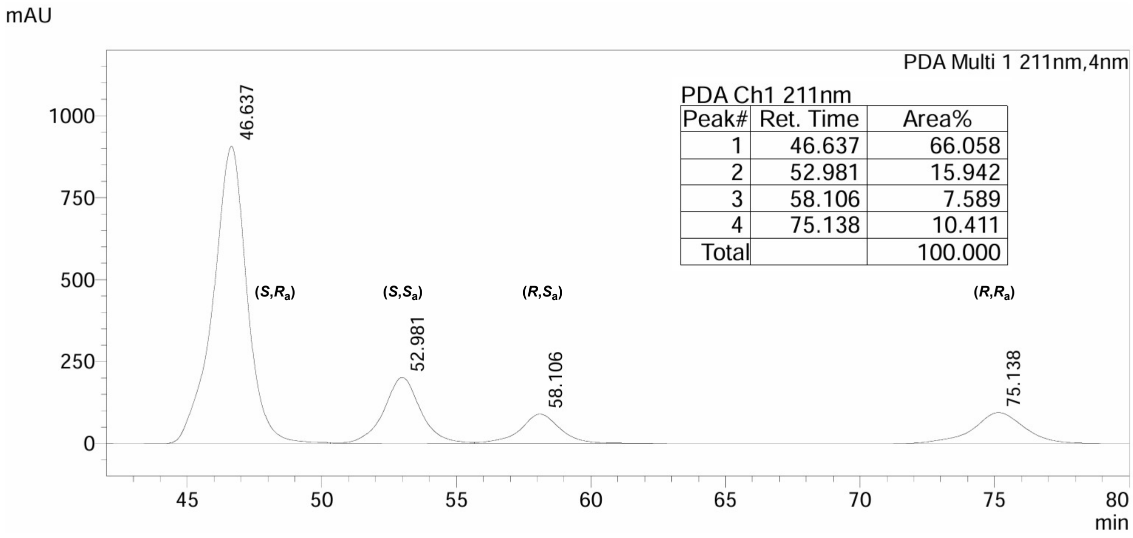

Figure S9 HPLC of **72** after heating.

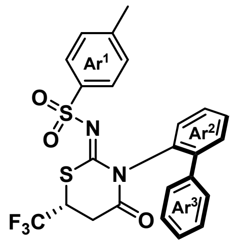

(R,R_a)-68

A sample of (R,R_a)-**68** with 87:13 dr, 95:5 er_{major}, and 63:37 er_{minor} (Figure S10) was heated at 50 °C for 30 min. Once complete, the sample now had 65:35 dr, 91:9 er_{major}, and 90:10 er_{minor} (Figure S11). Again, the reduction in dr was expected due to epimerisation. The er within the minor diastereoisomer had increased and the major enantiomer was assigned (R,S_a), coming from the epimerisation of (R,R_a). The minor enantiomer within the minor diastereoisomer was therefore assigned (S,R_a). In this case, epimerisation either during the reaction or during product isolation was the main source of the minor diastereoisomer, and hence the opposite enantioselectivity observed.

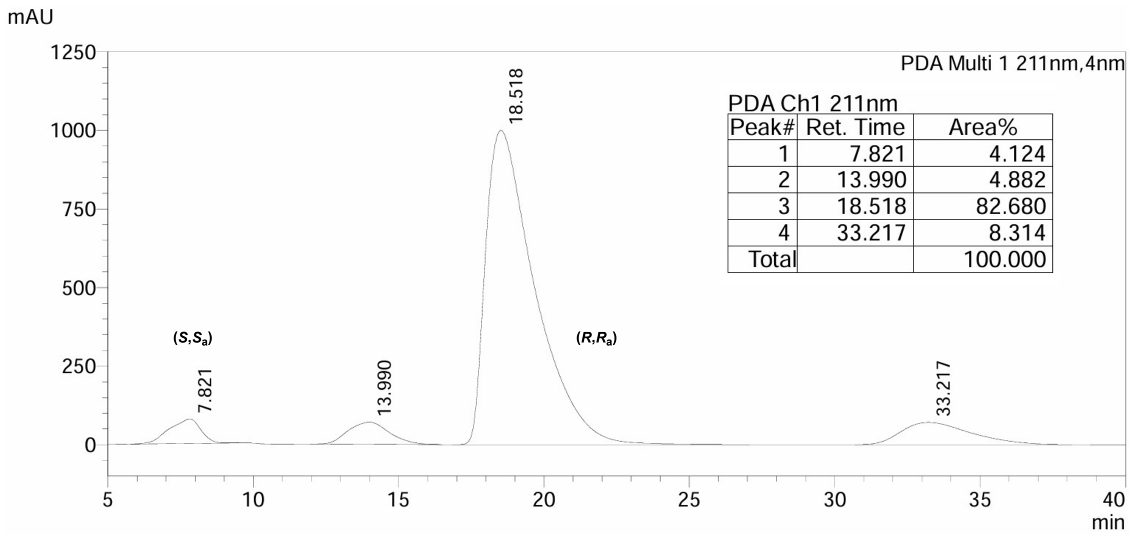

Figure S10 HPLC of **68** before heating.

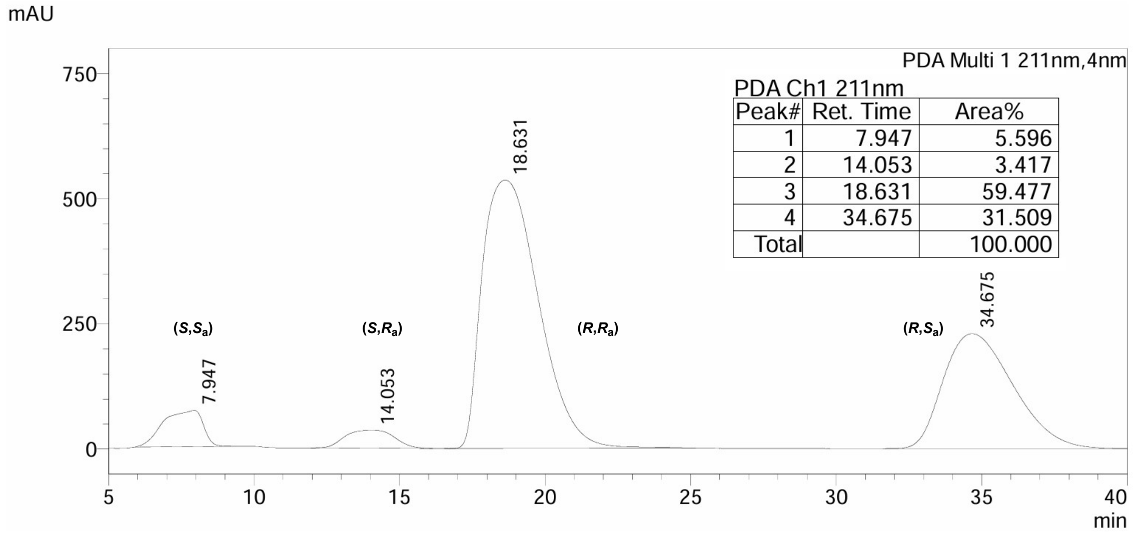

Figure S11 HPLC of **68** after heating.

15. Mechanistic Studies

Procedure for NMR titration of Schreiner's thiourea 26 with NBu_4OPNP (Scheme 5A)

Schreiner's thiourea **26** (37.5 mg, 0.075 mmol, 1.0 equiv.) was dissolved in d_8-THF (0.1 M) and a reference ^1H NMR taken. NBu_4OPNP (5.7 mg, 0.015 mmol, 20 mol%) was added to the solution and another ^1H NMR taken. This was repeated for 40 mol%, 60 mol%, 80 mol%, and 100 mol% of NBu_4OPNP.

Procedure for background reaction with NBu_4OPNP (Scheme 5B)

Schreiner's thiourea **26** (50.0 mg, 0.10 mmol, 1.0 equiv.), 4-nitrophenyl (*E*)-4,4,4-trifluorobut-2-enoate **27** (39.2 mg, 0.15 mmol, 1.5 equiv.), and NBu_4OPNP (3.8 mg, 0.01 mmol, 10 mol%) were dissolved in anhydrous THF (0.1 M) and stirred at RT for 24 h. Once complete, the solvent was removed under reduced pressure, 1,3,5-trimethoxybenzene (0.1 M in $CDCl_3$, 0.5 mL, 0.05 mmol) was then added and ^1H NMR obtained.

Procedure for NMR titration of PNP ester S5 with Schreiner's thiourea 26 (Figure S12)

4-Nitrophenyl crotonate **S5** (23.3 mg, 0.11 mmol, 1.5 equiv.) was dissolved in d_8-THF (0.1 M) and a reference ^1H NMR taken. Schreiner's thiourea **26** (7.5 mg, 0.015 mmol, 20 mol%) was added to the solution and another ^1H NMR taken. This was repeated for 40 mol%, 60 mol%, 80 mol%, 100 mol%, and 150 mol% of Schreiner's thiourea **26**.

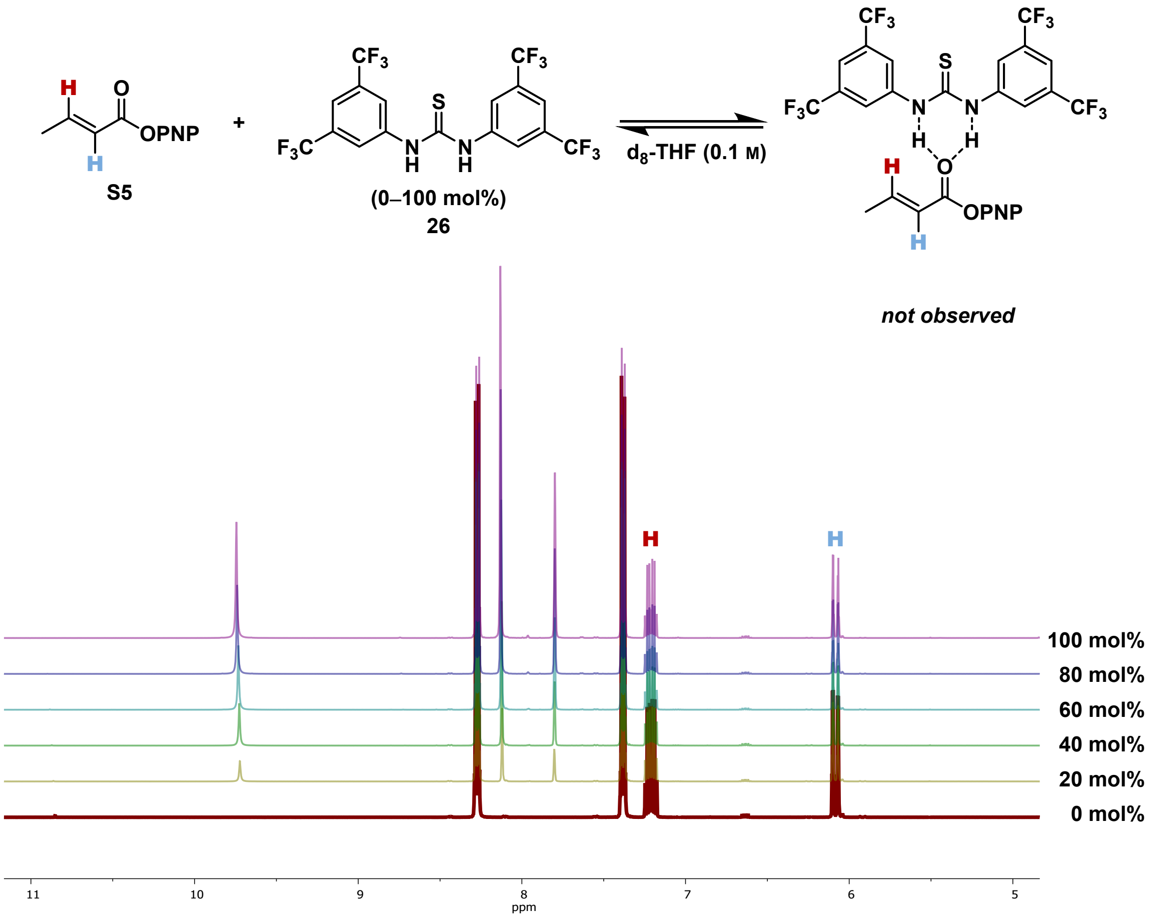

Figure S12 NMR titration of PNP ester **S5** with Schreiner's thiourea **26**.

Perfluorophenyl 3-((*N*-(2-(*tert*-butyl)phenyl)-*N*'-tosylcarbamimidoyl)thio)propanoate (76)

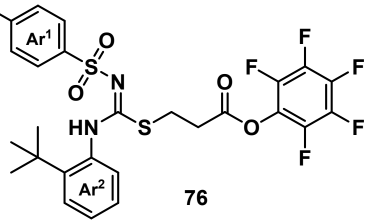

76

Perfluorophenyl acrylate **75** (360 mg, 1.5 mmol, 1.5 equiv.), *N*-((2-(*tert*-butyl)phenyl)carbamothioyl)-4-methylbenzenesulfonamide **74** (360 mg, 1.0 mmol, 1.0 equiv.) and (±)-HyperBTM **21** (15.4 mg, 0.05 mmol, 5 mol%) were dissolved in anhydrous THF (0.1 M) and stirred at 0 °C for 5 h. After removal of the solvent under reduced pressure, the crude reaction mixture was purified directly by flash silica column chromatography (hexane:EtOAc 100:0 to 0:100, R_F 0.23 at 50:50) to give the title compound as a white solid (330 mg, 55%). **mp** 99 – 101 °C; **^{1}H NMR** (500 MHz, $CDCl_3$) δ_H: 1.40 (9H, s, C(CH_3)$_3$), 2.44 (3H, s, CH_3), 2.94 (2H, t, *J* 6.9, C(3)H_2), 3.21 (2H, t, *J* 6.9, C(2)H_2), 7.15 (1H, dd, *J* 7.8, 1.5, Ar^{2}C(6)*H*), 7.20 – 7.25 (1H, m, Ar^{2}C(5)*H*), 7.30 – 7.37 (3H, m, Ar^{1}C(3,5)*H* and Ar^{2}C(4)*H*), 7.48 (1H, dd, *J* 8.0, 1.5, Ar^{2}C(3)*H*), 7.87 – 7.91 (2H, m, Ar^{1}C(2,6)*H*), 9.83 (1H, br s, N*H*); **^{19}F{^{1}H} NMR** (470 MHz, $CDCl_3$) δ_F: –162.2 – –162.0 (m, ArC*F*), –157.6 (t, *J* 21.7, ArC*F*), –152.6 – –152.5 (m, ArC*F*); **^{13}C{^{1}H} NMR** (126 MHz, $CDCl_3$) δ_C: 21.7 (CH_3), 26.3 (*C*(2)H_2), 30.6 (C(CH_3)$_3$), 33.6 (*C*(3)H_2), 35.3 (*C*(CH_3)$_3$), 124.7 – 125.0 (Ar*C*(1)), 126.5 (Ar^1*C*(2,6)H), 127.0 (Ar^2*C*(5)H), 127.8 (Ar^2*C*(3)H), 129.6 (Ar^2*C*(4)H), 129.7 (Ar^1*C*(3,5)H), 131.3 (Ar^2*C*(6)H), 134.0 (Ar^2*C*(1)), 136.7 – 139.2 (m, ArCF), 138.5 – 140.8 (Ar*C*F), 139.5 (Ar^1*C*(1)), 139.9 – 142.4 (m, ArCF), 143.4 (Ar^1*C*(4)), 147.2 (Ar^2*C*(2)), 167.6 (CN), 168.1 (CO); **IR** ν_{max} (film) 3269, 2972, 1790, 1562, 1516, 1402, 1321, 1269, 1138, 1092, 1084, 1038, 982, 856, 829; **HRMS** (ESI^+) $C_{27}H_{25}O_4N_2F_5S_2Na$ $[M+Na]^+$ found 623.1067, requires 623.1068 (–0.2 ppm).

(*Z*)-4-Methyl-*N*-(4-oxo-3-phenyl-1,3-thiazinan-2-ylidene)benzenesulfonamide (78)

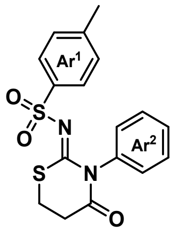

78

Perfluorophenyl 3-((*N*-(2-(*tert*-butyl)phenyl)-*N*'-tosylcarbamimidoyl)thio)propanoate **76** (120 mg, 0.20 mmol, 1.0 equiv.), 4-methyl-*N*-(phenylcarbamothioyl)benzenesulfonamide **77** (61.3 mg, 0.20 mmol, 1.0 equiv.), and (±)-HyperBTM **21** (6.2 mg, 0.020 mmol, 10 mol%) were dissolved in anhydrous THF (0.1 M) and stirred for 24 h at RT. Once complete, the reaction mixture was diluted with EtOAc and washed with sat. $NaHCO_3$ (× 3), dried over $MgSO_4$, then the solvent removed under reduced pressure to afford the crude product. The crude product was purified by flash silica column chromatography (CH_2Cl_2:Et_2O 100:0 to 90:10, R_F 0.32 at 95:5) to give the title compound as a white solid (37 mg, 51%). **mp** 164 – 166 °C; **IR** ν_{max} (film) 2920, 1713, 1514, 1344, 1279, 1140, 1088, 993, 841, 694, 671; **^{1}H NMR** (400 MHz, $CDCl_3$) δ_H: 2.36 (3H, s, C*H*$_3$), 3.17 – 3.23 (2H, m, C(5)*H*$_2$), 3.24 – 3.30 (2H, m, C(6)*H*$_2$), 7.01 – 7.06 (2H, m, Ar^{2}C(2,6)*H*), 7.10 – 7.15 (2H, m, Ar^{1}C(3,5)*H*), 7.35 – 7.45 (5H, m, Ar^{1}C(2,6)*H* and Ar^{2}C(3,4,5)*H*); **^{13}C{^{1}H} NMR** (126 MHz, $CDCl_3$) δ_C: 21.6 (*C*H$_3$), 23.4 (*C*(5)H$_2$), 34.5 (*C*(5)H$_2$), 126.5 (Ar^1*C*(2,6)H), 128.4 (Ar^2*C*(2,6)H), 128.7 (Ar^2*C*(4)H), 129.2 (Ar^2*C*(3,5)H), 129.4 (Ar^1*C*(3,5)H), 137.1 (Ar^2*C*(1)), 138.3 (Ar^1*C*(1)), 143.3 (Ar^1*C*(4)), 164.7 (*C*(2)), 169.1 (*C*(4)); **HRMS** (ESI^+) $C_{17}H_{16}O_3N_2NaS_2$ $[M+Na]^+$ found 383.0493, requires 383.0495 (–0.5 ppm).

16. Computational Studies

Geometry optimisations were performed with the *meta*-hybrid M06-2X functional[18] using the double-ζ, def2-SVP basis set from the redefinition of the Ahlrichs family of basis sets.[19-22] Implicit solvation was considered through the use of the SMD model employing the parameters of THF (ε = 7.4257).[23] An ultrafine integration grid (99 radial shells with 590 angular points per shell) was used for all calculations and all species were formally treated as closed-shell systems with restricted Kohn-Sham DFT used throughout. The nature of minima and transition states located were verified by the computation of harmonic frequencies at the same level of theory. Single-point energies (E_{sp}) were also evaluated using the M06-2X functional[18] with a larger, triple-ζ, def2-TZVP basis. Implicit solvation was also included at this level of theory using the same ultrafine integration grid (99,590). Additional empirical dispersion corrections were not included as the functional implicitly accounts for dispersion due to the nature of its construction. Thermochemistry was evaluated at 1 atm and 298.15 K using thermodynamic calculations at the level of geometry optimisation (thermal corrections to enthalpy, $\delta H_{298.15}$, and entropies $S_{298.15}$) in combination with energetics obtained from single-point calculations. Gibbs free energies were calculated at 273 K (and 323 K for atropisomeric rotational barriers to correspond with experiment) using Equation **S2**, with additional Martin Hay Pratt empirical entropic corrections included (S_{MHP} = 3.05 kcal/mol per particle, evaluated at 276 atm to mimic bulk THF, Equations **S3-S4**).[24] Conformational flexibility of minima species was evaluated using CREST,[25] retaining the lowest lying GFN2-xTB conformation of the minima. The key enantiodetermining transition state **TS1** was subject to a more rigorous conformational treatment, with DFT evaluation of different staggered conformations around the forming S-C bond with *cis-cis*, *trans-cis* and *trans-trans* conformations of the thiourea nucleophile (see Figures **S13-S14**). All computations were performed using the Gaussian16, C.01 programme[26] with visualisation of structures using PyMol.[27] This and similar levels of DFT have previously been used successfully to rationalise reactivities and selectivities of organocatalytic reactions with isothioureas.[5, 28]

$$G_{298.15} = E_{sp} + \delta H_{298.15} - TS_{298.15} + \mathrm{S}_{\mathrm{MHP}}$$

Equation S2 Calculation of Gibbs free energies.

$$p = \rho RT/M = 276.340 \text{ atm}$$

Equation S3 Evaluation of pressure for S_{MHP} correction.[24] Where p = pressure of THF, ρ = density of THF (0.889 g/cm^3), M = molar mass of THF (72.107 g/mol).

$$S_{MHP} = RT \ln(p/p^0) = 3.05 \text{ kcal/mol per particle,}$$

Equation S4 Evaluation of S_{MHP} correction.[24] Where R = ideal gas constant, T = temperature (273.15 K).

Conformational Screening of Michael addition TS

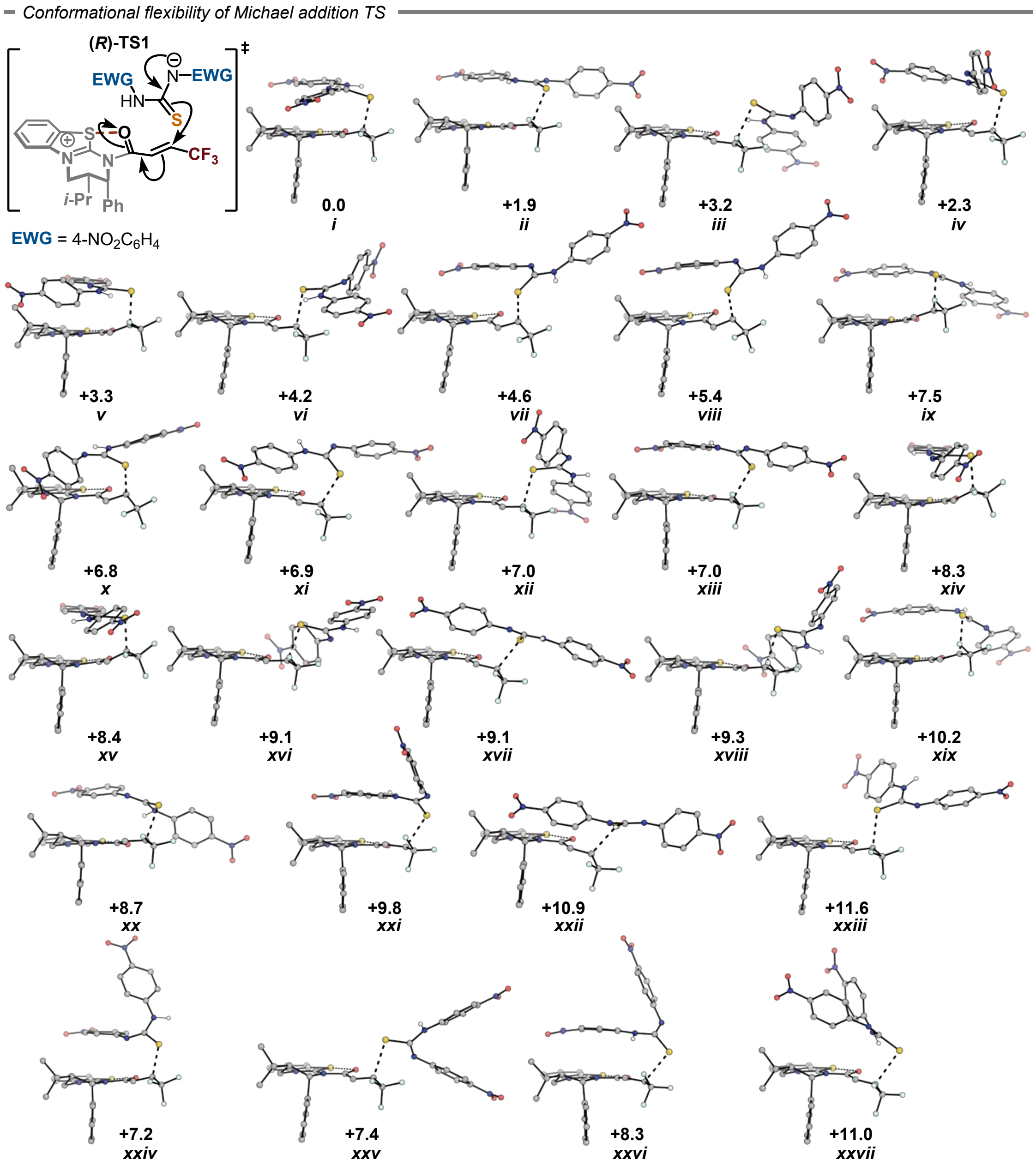

Figure S13 Conformational screening of (*R*)-TS1. Gibbs free energies are shown in kcal/mol.

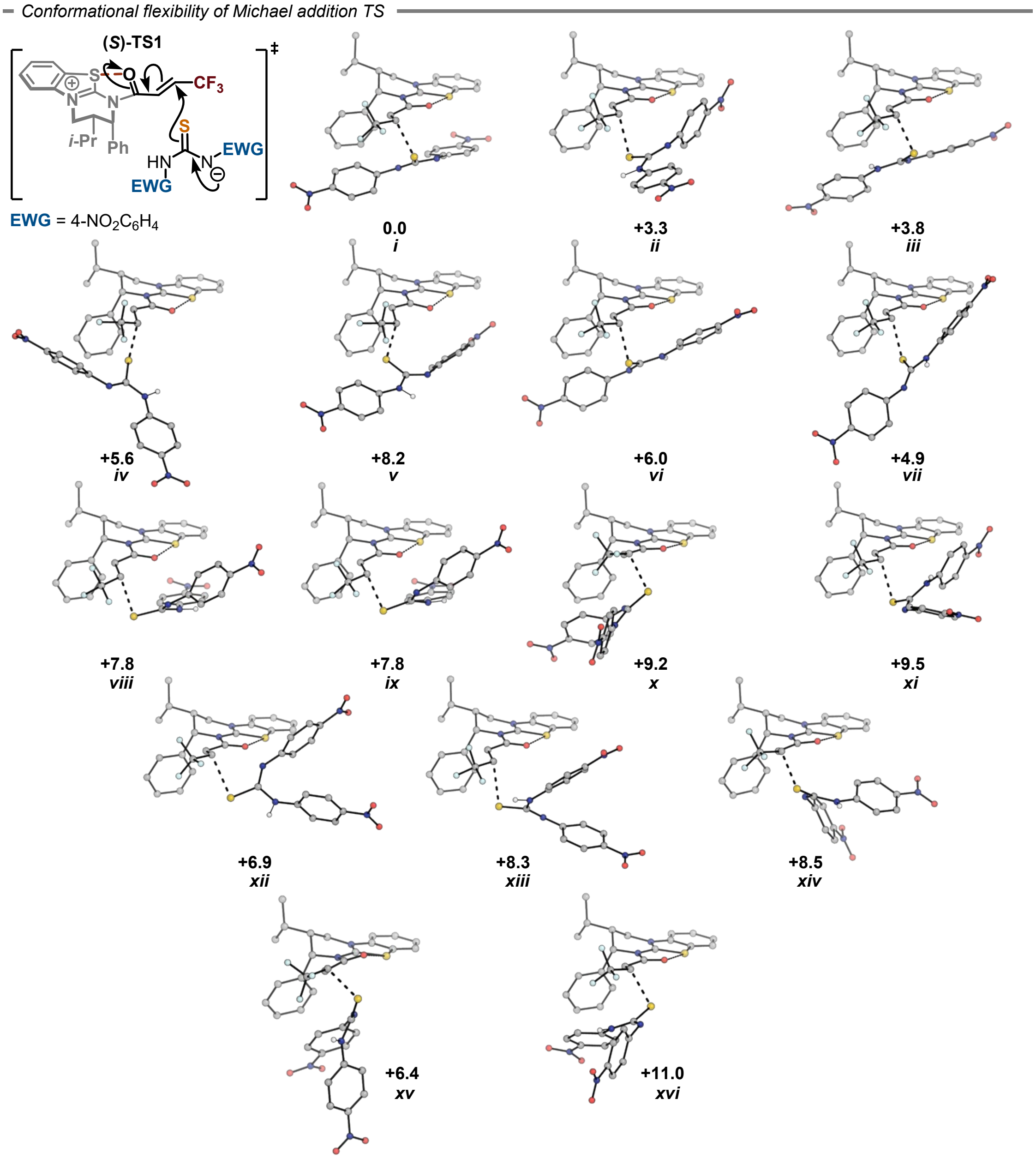

Figure S14 Conformational screening of (*S*)-TS1. Gibbs free energies are shown in kcal/mol.

Stability of Michael Adduct Species

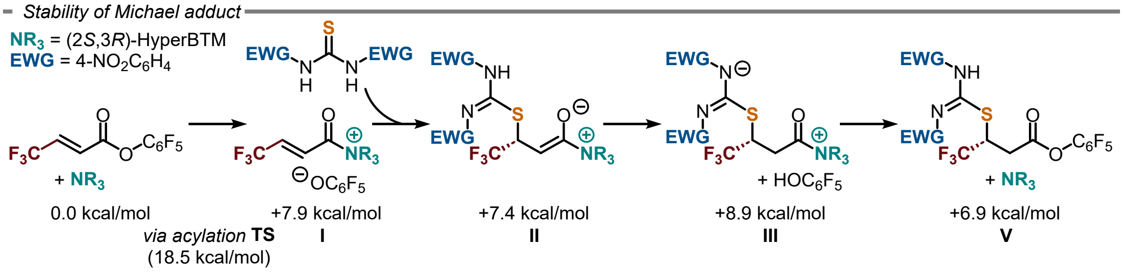

Figure S15 Computed stability of different Michael addition adducts. Gibbs free energies (ΔG_{273}) are shown in kcal/mol.

Variation of Michael Acceptor

By changing the CF_3 of the Michael acceptor to a methyl group, there is a reduction in driving force alongside an increase in barrier heights for **TS1-3**, indicating that the reaction may be slightly slower with the more donating methyl group. The barrier for catalyst acylation is also increased from 18.5 kcal/mol to 22.2 kcal/mol. The presence of an electron withdrawing group increases the electrophilicity of this component, facilitating the Michael addition and subsequent reactivity. The er of this substrate (**42**) is computed to remain high through the lactamisation (99:1 (*R*) er computed, 86:14 (*R*) er experimental), though the selectivity of the Michael addition step is significantly reduced, and we would predict an almost racemic mixture of the products based on the kinetic selectivity of the Michael addition alone.

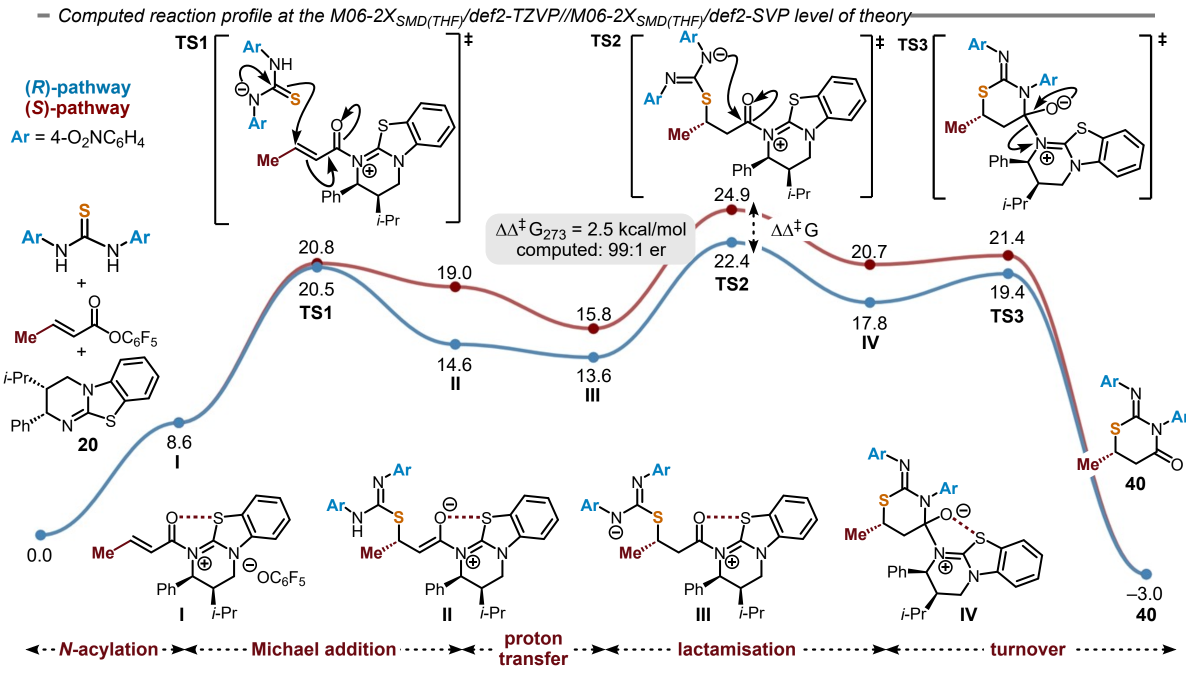

Figure S16 Computed reaction profile of methyl analogue **42** at the M06-2X$_{SMD(THF)}$/def2-TZVP//M06-2X$_{SMD(THF)}$/def2-SVP level of theory. Gibbs free energies (ΔG_{273}) are shown in kcal/mol. Lactamisation in the (*S*)-pathway is facilitated by rotation of the acyl component (same as **36**).

Atropisomerism Rotational Barriers

Rotational barriers were computed for rotation of the ortho-alkyl or -aryl moiety past the C=N-R group (see TS in Figure S17) for consistency as this was computed to be lower in energy than rotation of the *i*-Pr moiety past the C=O group for product **67** ($\Delta\Delta^{‡}G$ = 0.5 kcal/mol). Calculations appear to overestimate the barrier height compared to experiment but reflect the trends with the introduction of more bulk.

Atropisomerism rotational barriers

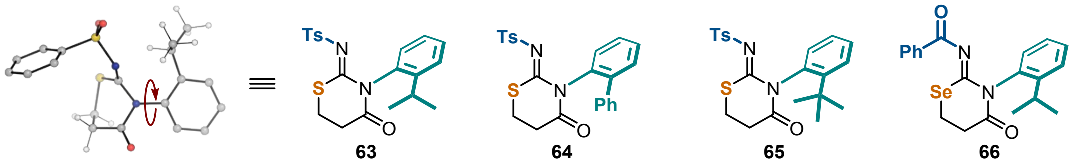

$\Delta^{‡}G$		63	64	65	66
	Experiment	26.7 kcal/mol	24.5 kcal/mol	32.8 kcal/mol[a]	25.7 kcal/mol
	Computed	28.7 kcal/mol	27.0 kcal/mol	37.4 kcal/mol[a]	26.7 kcal/mol

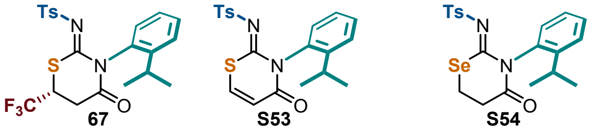

$\Delta^{‡}G$		67	S53	S54
	Computed	28.7 kcal/mol	33.6 kcal/mol	27.3 kcal/mol

overestimation compared to experiment

Figure S17 Computed and experimental rotational barrier heights of atropisomeric products evaluated at 323 K. Gibbs free energies (ΔG_{323}) are shown in kcal/mol. [a] evaluated at 393 K.

17. Single Crystal X-ray Diffraction Data

X-ray diffraction data for compounds **37**, **55**, and **67** were collected using a Rigaku MM-007HF High Brilliance RA generator/confocal optics with either XtaLAB P100 or P200 diffractometers [Cu Kα radiation (λ = 1.54187 Å)]. Data for all compounds analysed were collected (using a calculated strategy) and processed (including correction for Lorentz, polarization and absorption) using CrysAlisPro.[29] Structures were solved by dual-space methods (SHELXT)[30] and refined by full-matrix least-squares against F^2 (SHELXL-2019/3).[31] Non-hydrogen atoms were refined anisotropically, and hydrogen atoms were refined using a riding model. All calculations were performed using the Olex2[32] interface. Selected crystallographic data are presented in Tables S9 – S11.

CCDC 2410987-2410989 contains the supplementary crystallographic data for this paper. These data can be obtained free of charge from The Cambridge Crystallographic Data Centre via www.ccdc.cam.ac.uk/structures.

Table S9: X-ray data for 37

	**(6*R*,*Z*)-37**
CCDC	2410987
empirical formula	$C_{19.33}H_{11.33}ClF_3N_4OS$
fw	440.17
crystal description	colourless prism
crystal size [mm]	0.30×0.21×0.18
space group	R3
a [Å]	21.67271(16)
b [Å]	21.67271(16)
c [Å]	10.76754(10)
vol [Å]^3	4379.99(8)
Z	9
p (calc) [g/cm^3]	1.502
μ [mm^{-1}]	3.174
F(000)	2010
reflections collected	41954
independent reflections ($\boldsymbol{R}_{int}$)	3444(0.0239)
parameters/restraints	265/1
GOF on F^2	1.058
R_1 [I > 2σ(l)]	0.0215
wR_2 (all data)	0.0560
largest diff. peak/hole [e/Å^3]	0.17, -0.28
Flack parameter	0.005(4)

Table S10: X-ray data for 55

	**(6*R*,*Z*)-55**
CCDC	2410988
empirical formula	$C_{18}H_{13}F_3N_2O_2Se$
fw	425.26
crystal description	colourless prism
crystal size [mm]	0.09×0.07×0.01
space group	$P2_1$
a [Å]	9.51115(5)
b [Å]	16.17558(9)
c [Å]	10.71507(5)
vol [Å]^3	1647.968(15)
Z	4
p (calc) [g/cm^3]	1.714
μ [mm^{-1}]	3.538
F(000)	848.0
reflections collected	61612
independent reflections (R_{int})	6656(0.0709)
parameters/restraints	469/1
GOF on F^2	1.070
R_1 [I > 2σ(l)]	0.0280
wR_2 (all data)	0.0755
largest diff. peak/hole [e/Å^3]	0.44, -0.50
Flack parameter	-0.046(10)

Table S11: X-ray data for 67

	**(6*R*,*R*$_a$,*Z*)-67**
CCDC	2410989
empirical formula	$C_{21}H_{21}F_3N_2O_3S_2$
fw	470.52
crystal description	colourless prism
crystal size [mm]	0.500×0.025×0.007
space group	$P2_12_12_1$
a [Å]	8.07483(6)
b [Å]	15.56758(15)
c [Å]	17.59729(15)
vol [Å]^3	2212.08(3)
Z	4
p (calc) [g/cm^3]	1.413
μ [mm^{-1}]	2.639
F(000)	976.0
reflections collected	43774
independent reflections ($\boldsymbol{R}_{int}$)	4547(0.0938)
parameters/restraints	283/0
GOF on F^2	1.043
R_1 [I > 2σ(l)]	0.0386
wR_2 (all data)	0.1033
largest diff. peak/hole [e/Å^3]	0.69, -0.20
Flack parameter	-0.008(10)

18. References

[1] A. Kütt, V. Movchun, T. Rodima, T. Dansauer, E. B. Rusanov, I. Leito, I. Kaljurand, J. Koppel, V. Pihl, I. Koppel, G. Ovsjannikov, L. Toom, M. Mishima, M. Medebielle, E. Lork, G.-V. Röschenthaler, I. A. Koppel and A. A. Kolomeitsev, *J. Org. Chem.*, 2008, **73**, 2607-2620.

[2] S. Espinosa, E. Bosch and M. Roses, *J. Chromatogr. A*, 2002, **964**, 55-66.

[3] J. Han and F. M. Tao, *J. Phys. Chem. A*, 2006, **110**, 257-263.

[4] M. D. Greenhalgh, S. Qu, A. M. Z. Slawin and A. D. Smith, *Chem. Sci.*, 2018, **9**, 4909-4918.

[5] J. Wu, C. M. Young, A. A. Watts, A. M. Z. Slawin, G. R. Boyce, M. Bühl and A. D. Smith, *Org. Lett.*, 2022, **24**, 4040-4045.

[6] A. Matviitsuk, M. D. Greenhalgh, D.-J. B. Antúnez, A. M. Z. Slawin and A. D. Smith, *Angew. Chem. Int. Ed.*, 2017, **56**, 12282-12287.

[7] H. Liu, A. M. Z. Slawin and A. D. Smith, *Org. Lett.*, 2020, **22**, 1301-1305.

[8] B. Kim, Y. Song and S. Y. Lee, *Chem. Commun.*, 2021, **57**, 11052-11055.

[9] Z. Wang, L. Hu, N. Chekshin, Z. Zhuang, S. Qian, J. X. Qiao and J.-Q. Yu, *Science*, 2021, **374**, 1281-1285.

[10] J.-C. Blazejewski, J. W. Hofstraat, C. Lequesne, C. Wakselman and U. E. Wiersum, *J. Fluorine Chem.*, 1998, **91**, 175-177.

[11] N. Busschaert, I. L. Kirby, S. Young, S. J. Coles, P. N. Horton, M. E. Light and P. A. Gale, *Angew. Chem. Int. Ed.*, 2012, **51**, 4426-4430.

[12] M. C. Risi, G. C. Saunders and W. Henderson, *Inorg. Chim. Acta*, 2021, **526**, 120506.

[13] F. Lacan, M. Varache-Lembège, J. Vercauteren, J.-M. Léger, B. Masereel, J.-M. Dogné and A. Nuhrich, *Eur. J. Med. Chem.*, 1999, **34**, 311-328.

[14] D. Tan, V. Štrukil, C. Mottillo and T. Friščić, *Chem. Commun.*, 2014, **50**, 5248-5250.

[15] B. Haouas, N. Sbei, H. Ayari, M. L. Benkhoud and B. Batanero, *New J. Chem.*, 2018, **42**, 11776-11781.

[16] L. Ma, Y. Zhou, D. Yang, M.-W. Wang, W. Lu and J. Jin, *Molecules*, 2022, **27**, 5867.

[17] J.-P. Heeb, J. Clayden, M. D. Smith and R. J. Armstrong, *Nat. Protoc.*, 2023, **18**, 2745-2771.

[18] Y. Zhao and D. G. Truhlar, *Theor. Chem. Acc.*, 2008, **120**, 215-241.

[19] F. Weigend and R. Ahlrichs, *Phys. Chem. Chem. Phys.*, 2005, **7**, 3297-3305.

[20] F. Weigend, *Phys. Chem. Chem. Phys.*, 2006, **8**, 1057-1065.

[21] A. Schäfer, H. Horn and R. Ahlrichs, *J. Chem. Phys.*, 1992, **97**, 2571-2577.

[22] A. Schäfer, C. Huber and R. Ahlrichs, *J. Chem. Phys.*, 1994, **100**, 5829-5835.

[23] A. V. Marenich, C. J. Cramer and D. G. Truhlar, *J. Phys. Chem. B*, 2009, **113**, 6378-6396.

[24] R. L. Martin, P. J. Hay and L. R. Pratt, *J. Phys. Chem. A*, 1998, **102**, 3565-3573.

[25] P. Pracht, F. Bohle and S. Grimme, *Phys. Chem. Chem. Phys.*, 2020, **22**, 7169–7192.

[26] G. W. T. M. J. Frisch, H. B. Schlegel, G. E. Scuseria, M. A. Robb, J. R. Cheeseman, G. Scalmani, V. Barone, G. A. Petersson, H. Nakatsuji, X. Li, M. Caricato, A. V Marenich, J. Bloino, B. G. Janesko, R. Gomperts, B. Mennucci, H. P. Hratchian, J. V. Ortiz, A. F. Izmaylov, J. L. Sonnenberg, D. Williams-Young, F. Ding, F. Lipparini, F. Egidi, J. Goings, B. Peng, A. Petrone, T. Henderson, D. Ranasinghe, V. G. Zakrzewski, J. Gao, N. Rega, G. Zheng, W. Liang, M. Hada, M. Ehara, K. Toyota, R. Fukuda, J. Hasegawa, M. Ishida, T. Nakajima, Y. Honda, O. Kitao, H.

Nakai, T. Vreven, K. Throssell, J. A. Montgomery, Jr., J. E. Peralta, F. Ogliaro, M. J. Bearpark, J. J. Heyd, E. N. Brothers, K. N. Kudin, V. N. Staroverov, T. A. Keith, R. Kobayashi, J. Normand, K. Raghavachari, A. P. Rendell, J. C. Burant, S. S. Iyengar, J. Tomasi, M. Cossi, J. M. Millam, M. Klene, C. Adamo, R. Cammi, J. W. Ochterski, R. L. Martin, K. Morokuma, O. Farkas, J. B. Foresman and D. J. Fox, Gaussian 16, Revision C.01, Gaussian Inc., Wallingford CT, 2019.

[27] The PyMOL Molecular Graphics System, Version 2.4.1, Schrödinger, LLC.

[28] C. Wang, S.-J. Li, Q.-C. Zhang, D. Wei and L. Ding, *Catal. Sci. Technol.*, 2020, **10**, 3664-3669.

[29] *CrysAlisPro* v1.171.42.82a, 94a. and 43.109a Rigaku Oxford Diffraction, Rigaku Corporation, Tokyo, Japan, 2023.

[30] G. M. Sheldrick, *Acta Crystallogr., Sect. A: Found. Adv.*, 2015, **71**, 3-8.

[31] G. M. Sheldrick, *Acta Crystallogr., Sect. C: Struct. Chem.*, 2015, **71**, 3-8.

[32] O. V. Dolomanov, L. J. Bourhis, R. J. Gildea, J. A. K. Howard and H. Puschmann, *J. Appl. Crystallogr.*, 2009, **42**, 339-341.

Appendix I: ^{1}H, $^{19}F\{^{1}H\}$, $^{13}C\{^{1}H\}$, and $^{77}Se\{^{1}H\}$ NMR Spectra

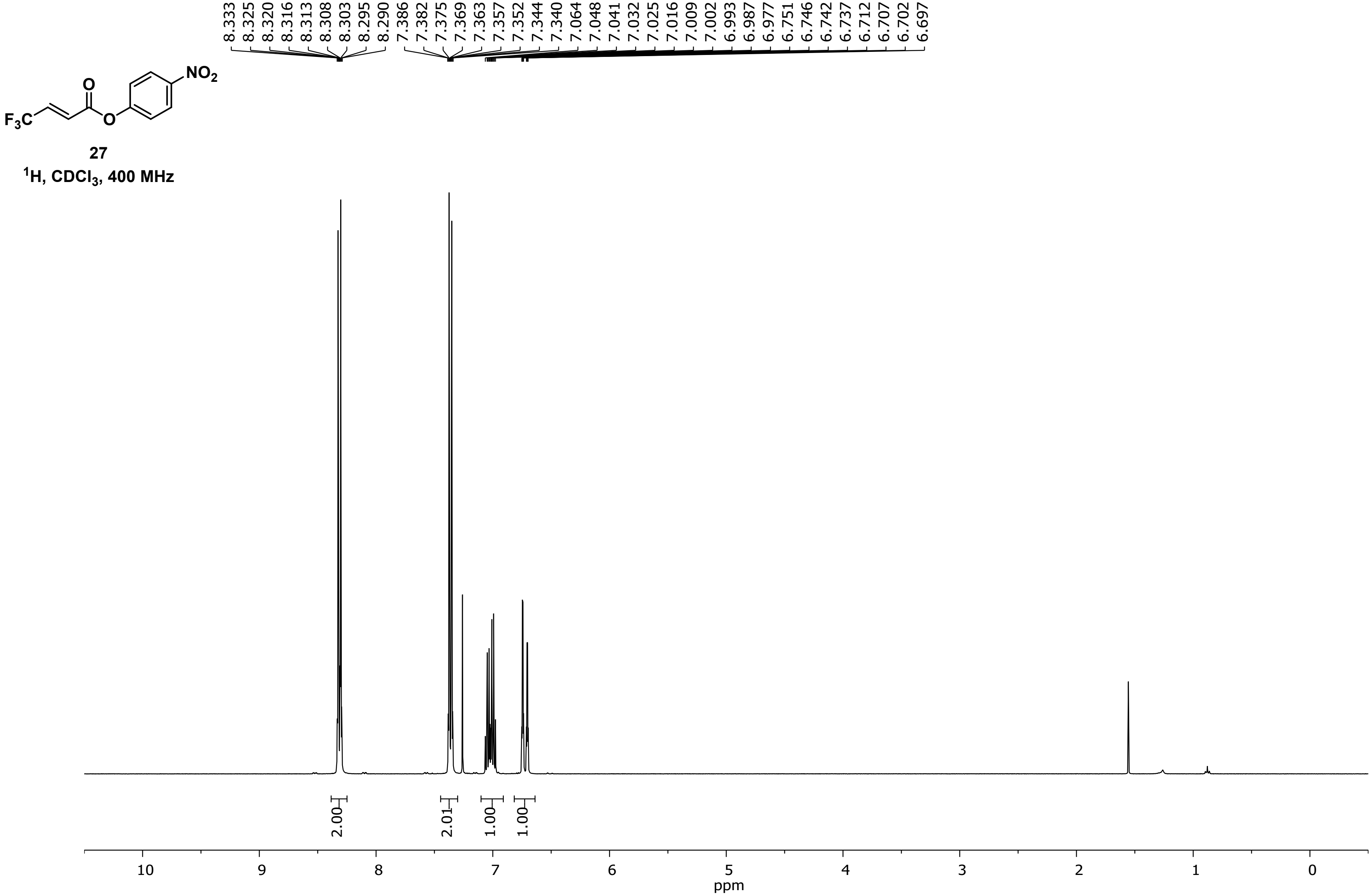

NO2
O
F3C
O
27
1H, CDCl3, 400 MHz
8.333
8.325
8.320
8.316
8.313
8.308
8.303
8.295
8.290
7.386
7.382
7.375
7.369
7.363
7.357
7.352
7.344
7.340
7.064
7.048
7.041
7.032
7.025
7.016
7.009
7.002
6.993
6.987
6.977
6.751
6.746
6.742
6.737
6.712
6.707
6.702
6.697
2.00
2.01
1.00
1.00
10
9
8
7
6
5
4
3
2
1
0
ppm

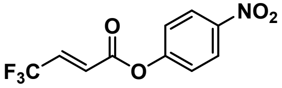

27

$^{19}F\{^{1}H\}$, $CDCl_3$, 377 MHz

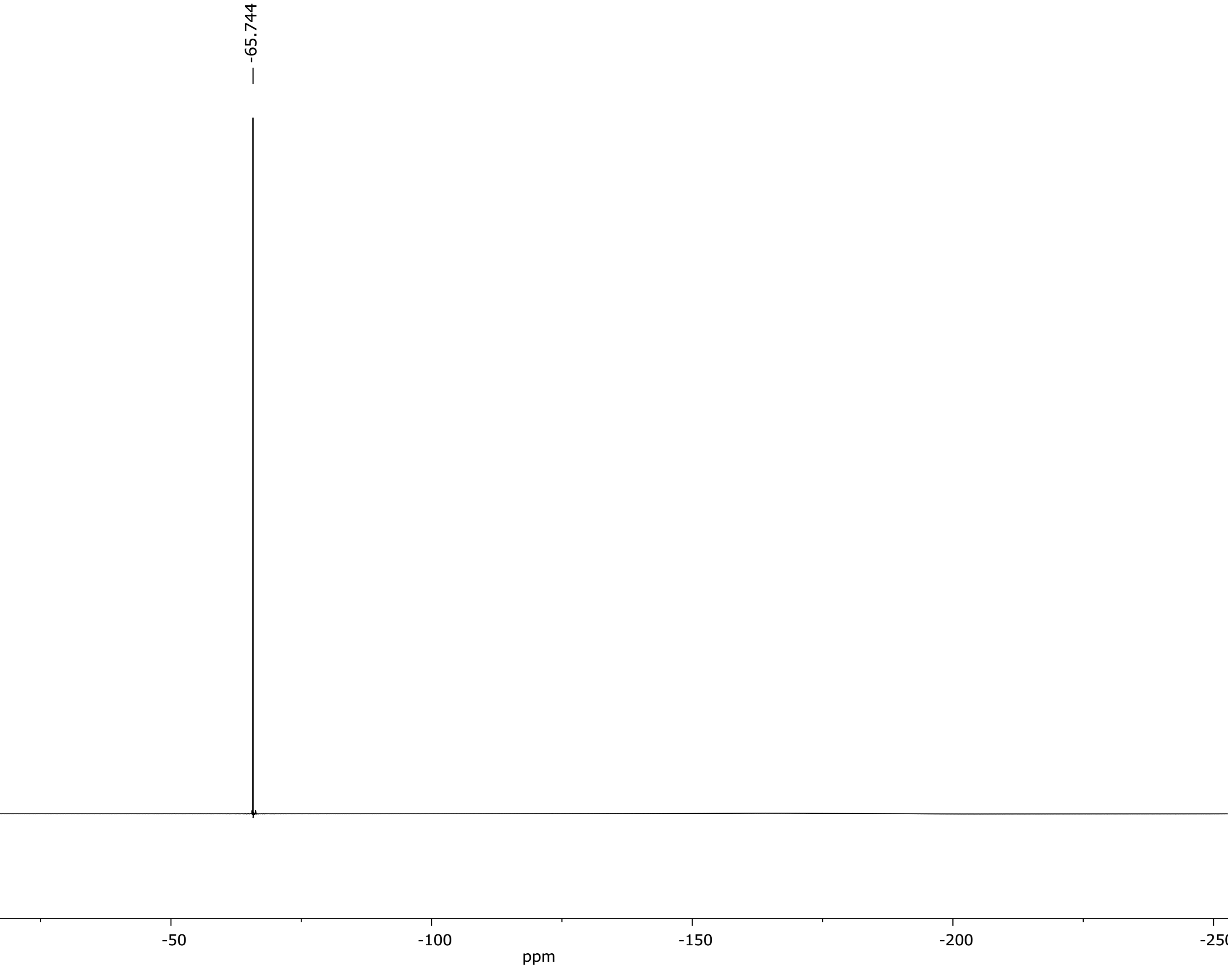

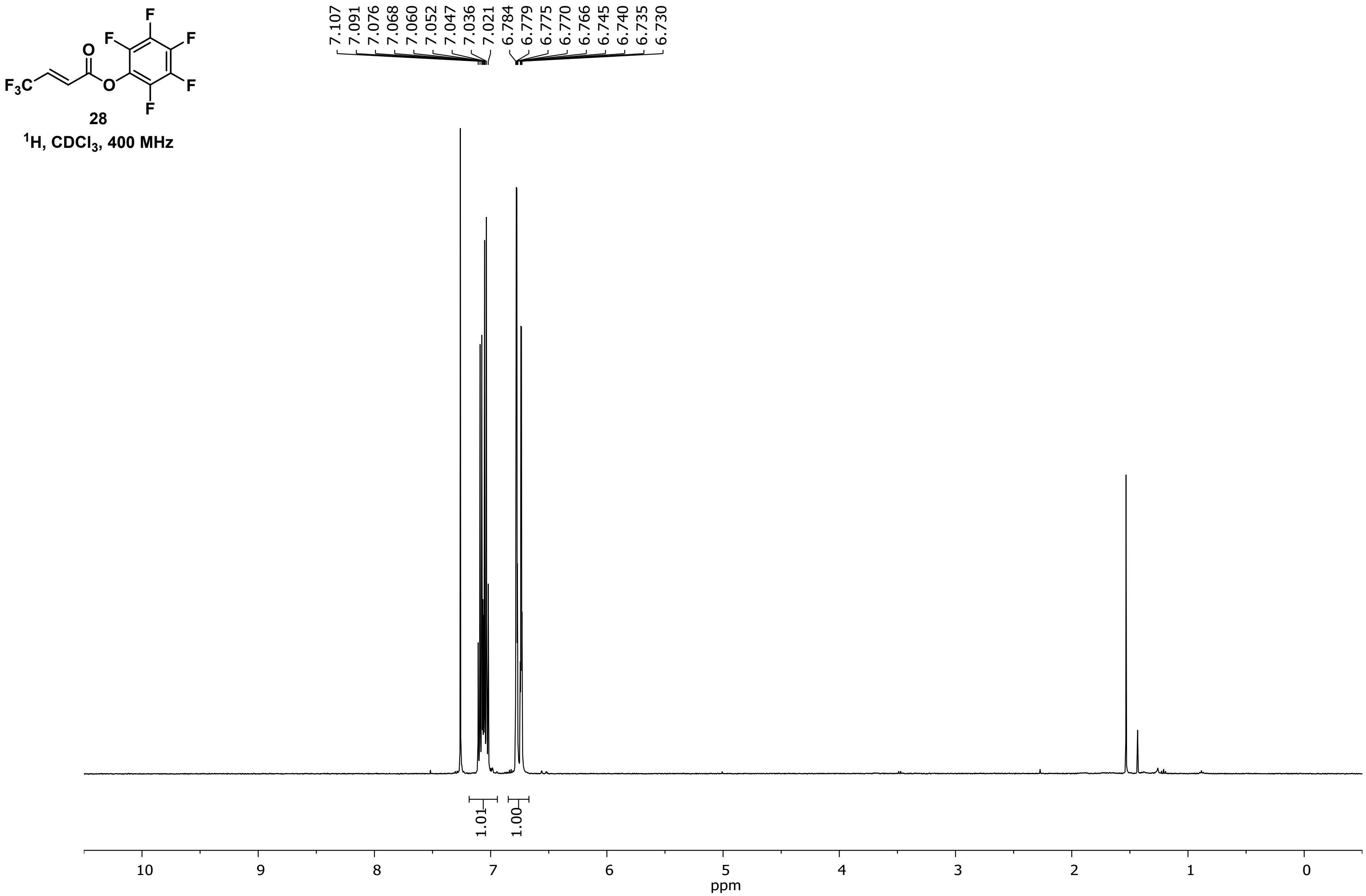

F
F
F
O
F
F
F_3C
O
F
28
^1H, $CDCl_3$, 400 MHz
7.107
7.091
7.076
7.068
7.060
7.052
7.047
7.036
7.021
6.784
6.779
6.775
6.770
6.766
6.745
6.740
6.735
6.730
1.01
1.00
10
9
8
7
6
5
4
3
2
1
0
ppm

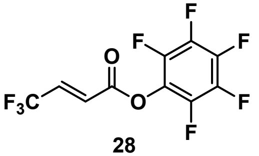

28

$^{19}F\{^{1}H\}$, $CDCl_3$, 377 MHz

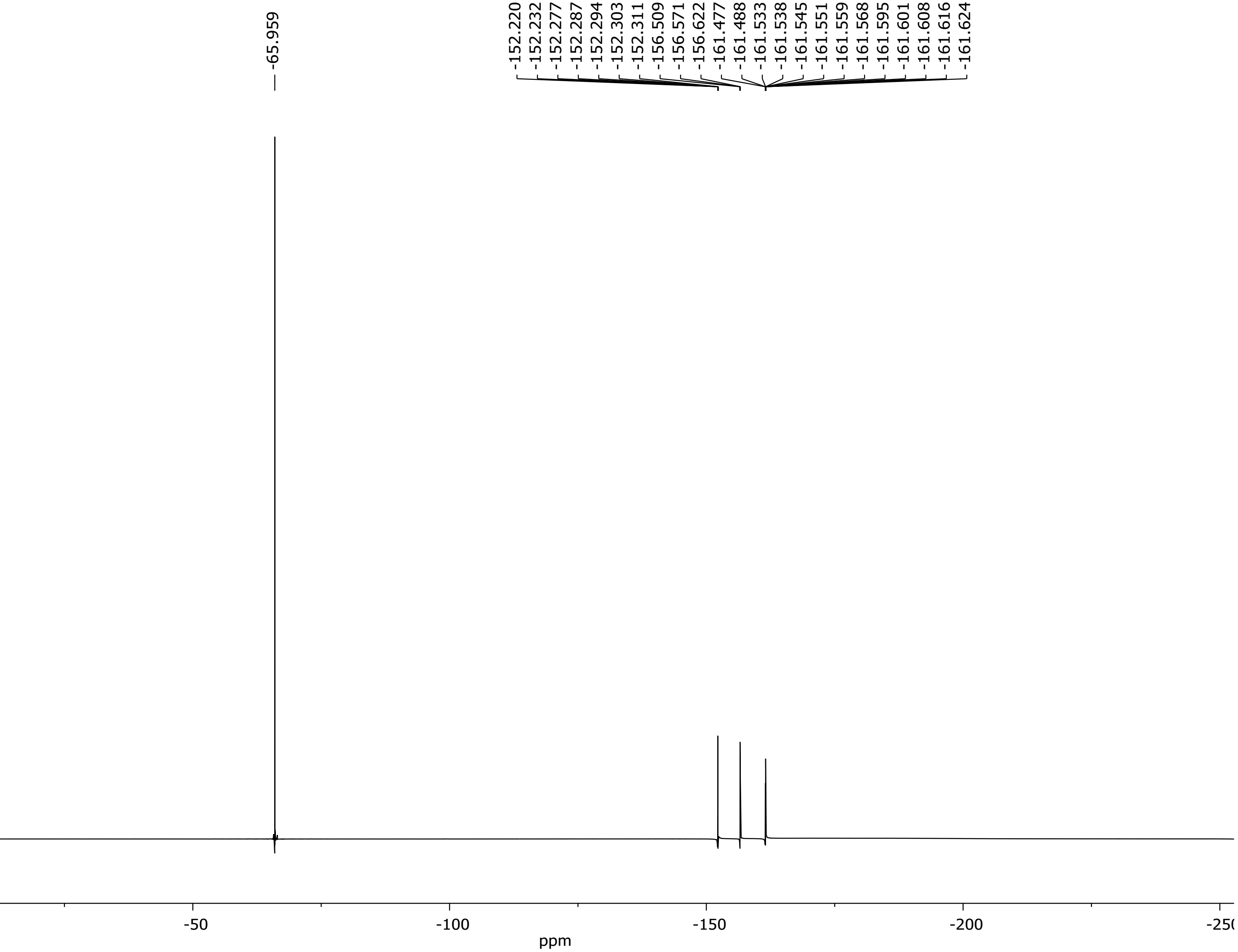

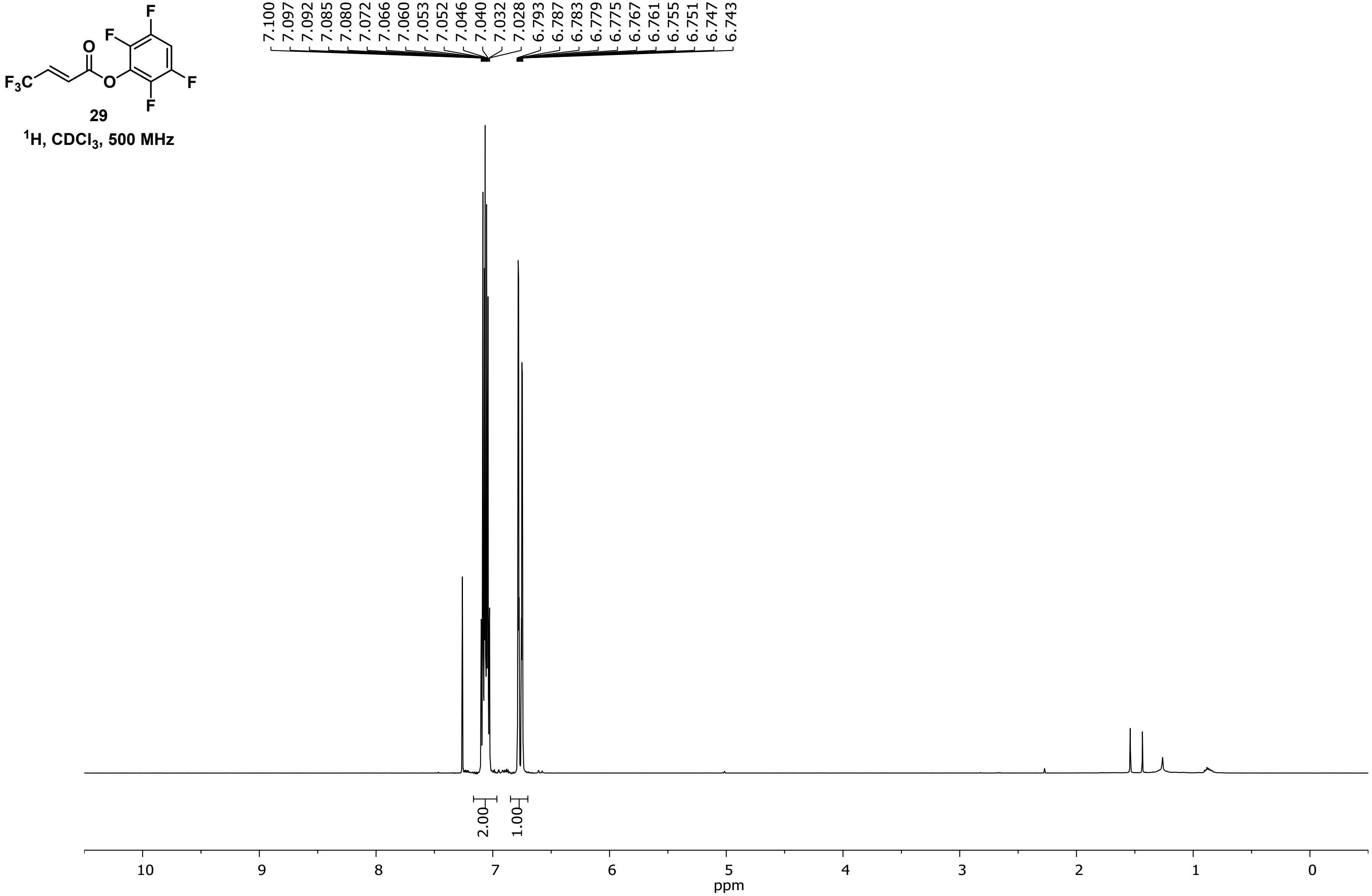

29

^{1}H, $CDCl_3$, 500 MHz

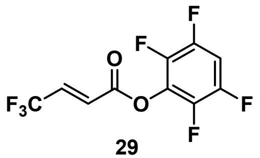

29

$^{19}F\{^1H\}$, $CDCl_3$, 377 MHz

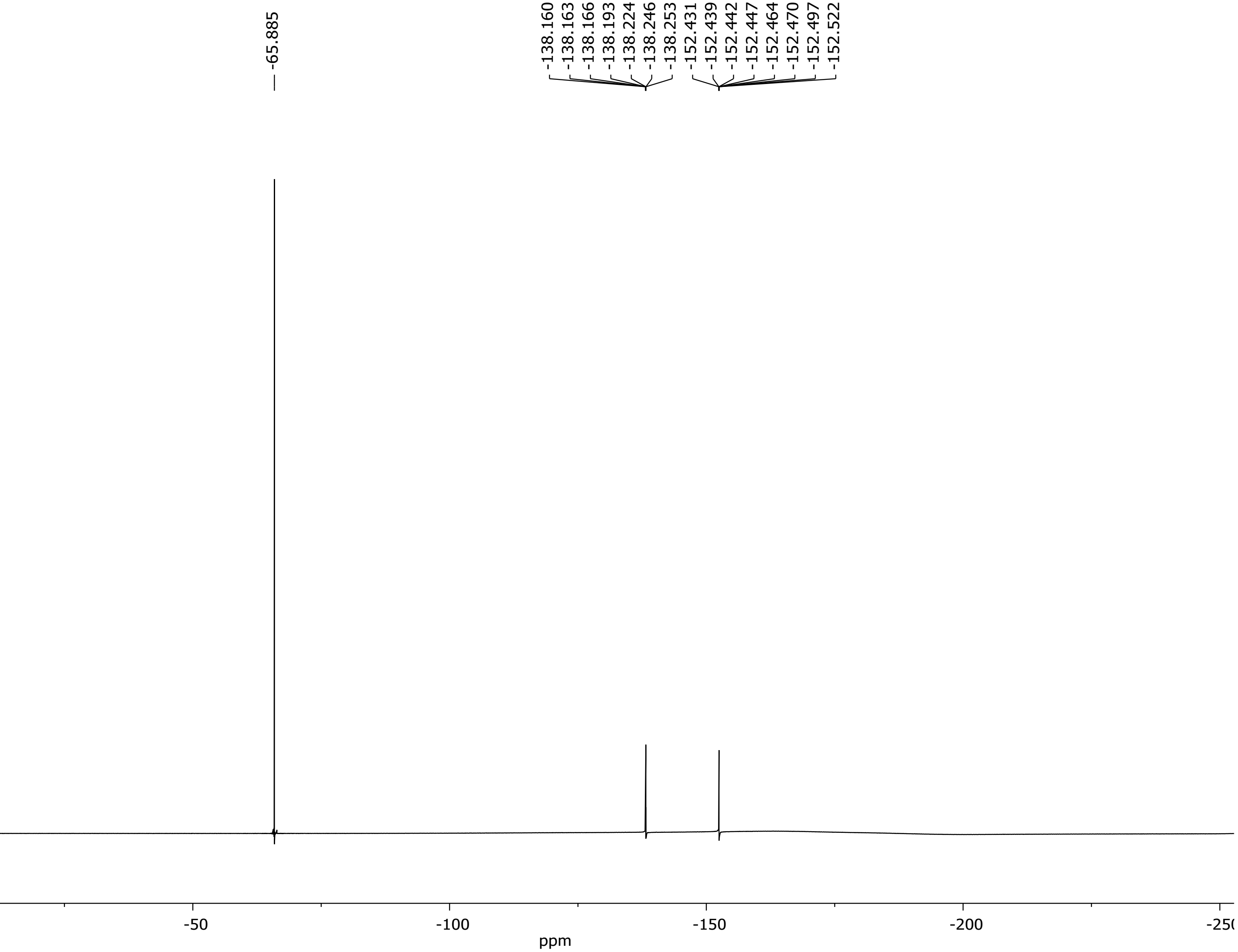

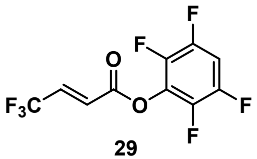

29

$^{13}C\{^1H\}$, $CDCl_3$, 126 MHz

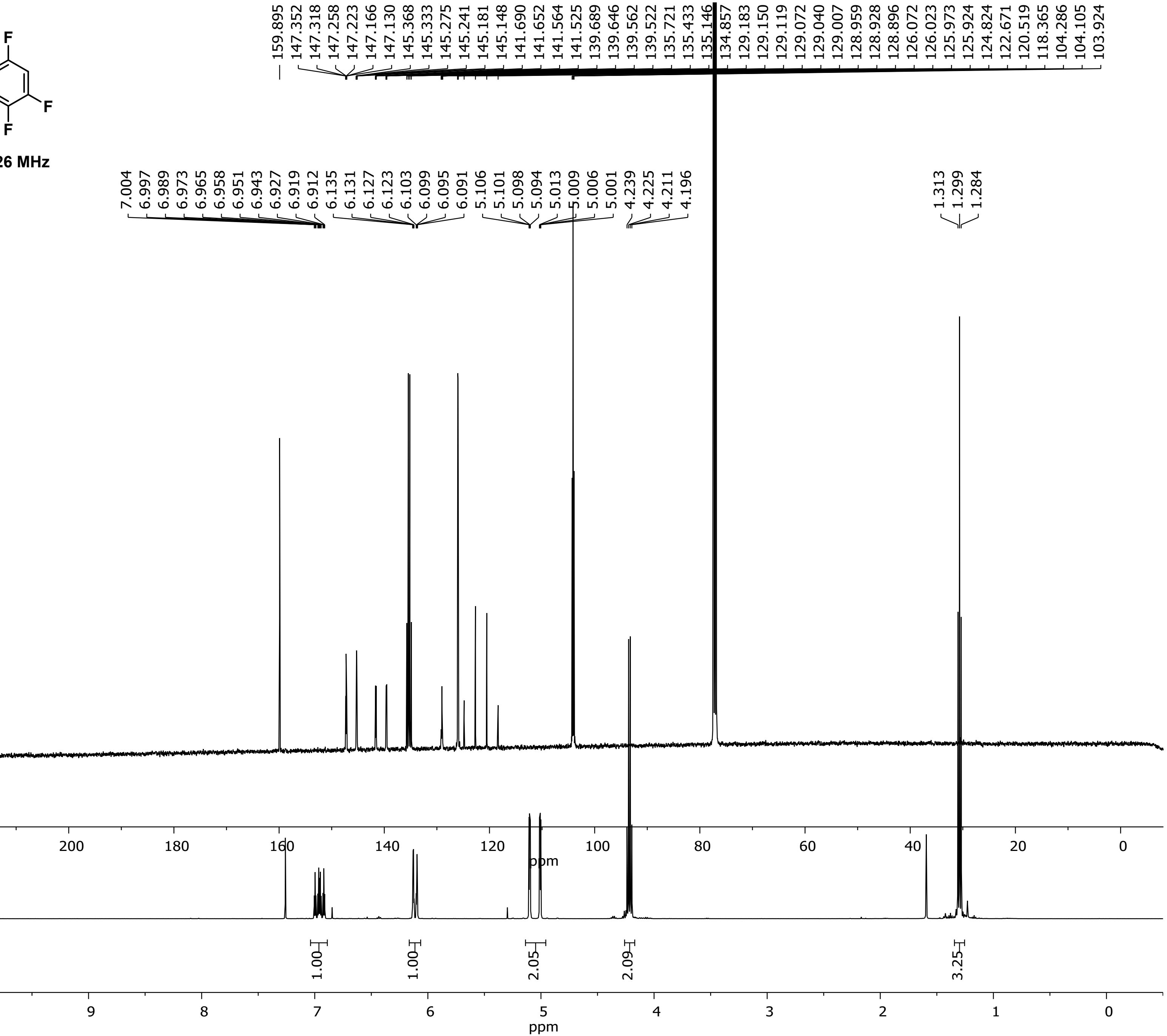

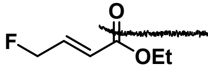

S7

^1H, $CDCl_3$, 500 MHz

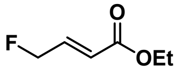

S7

$^{19}F\{^{1}H\}$, $CDCl_3$, 376 MHz

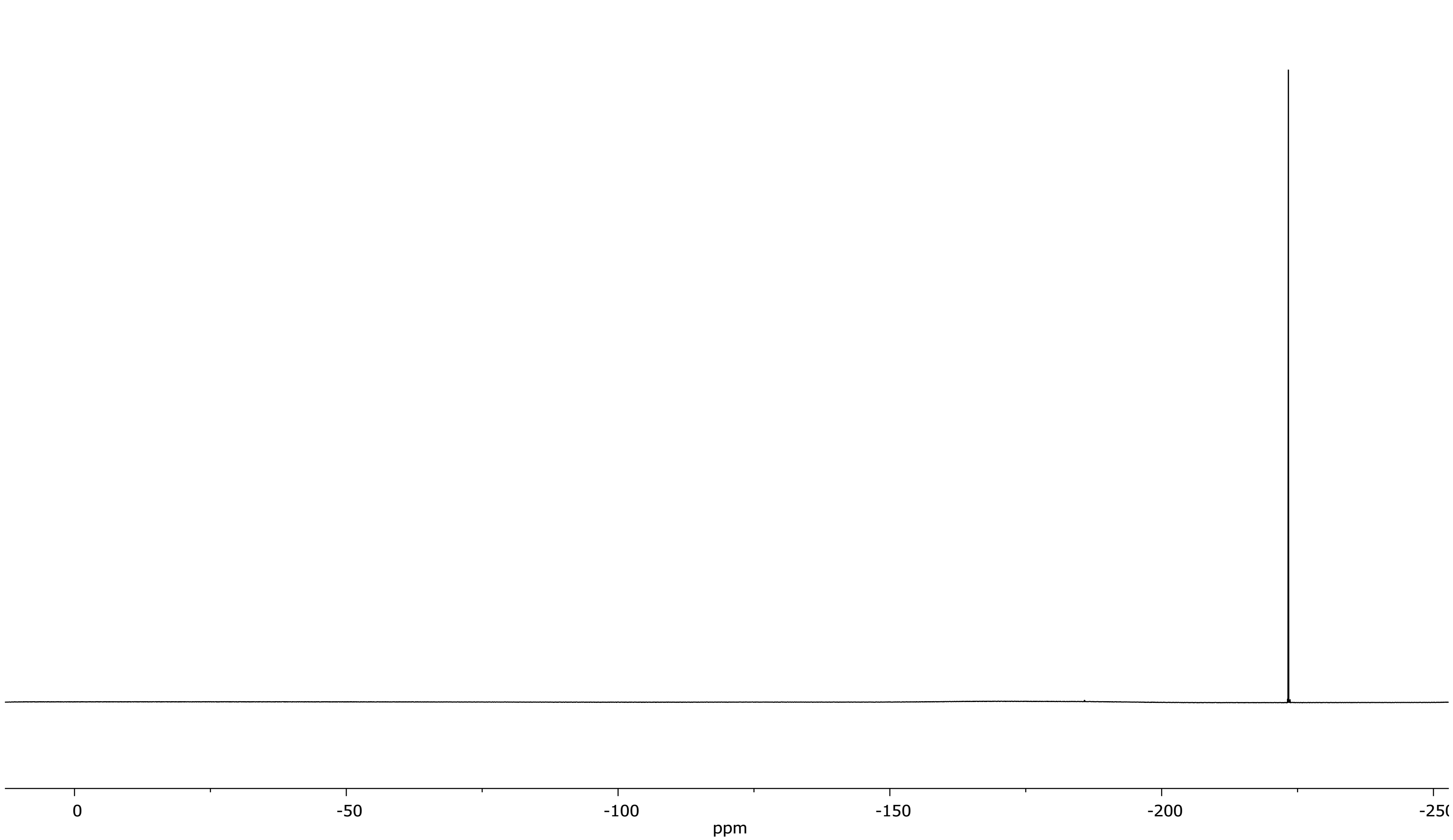

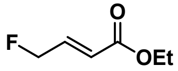

S7

$^{13}C\{^{1}H\}$, $CDCl_3$, 126 MHz

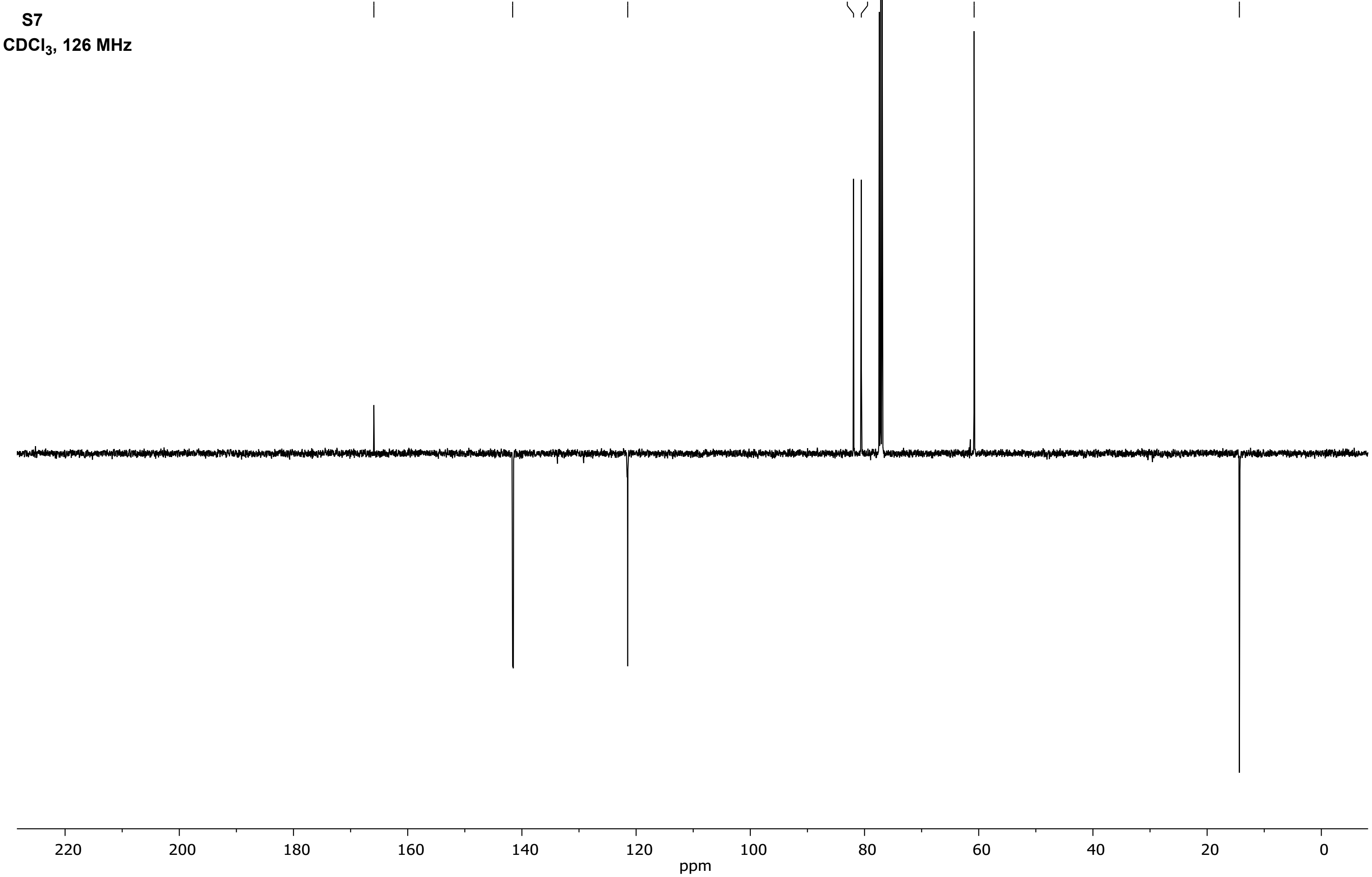

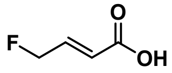

S8

^{1}H, $CDCl_3$, 500 MHz

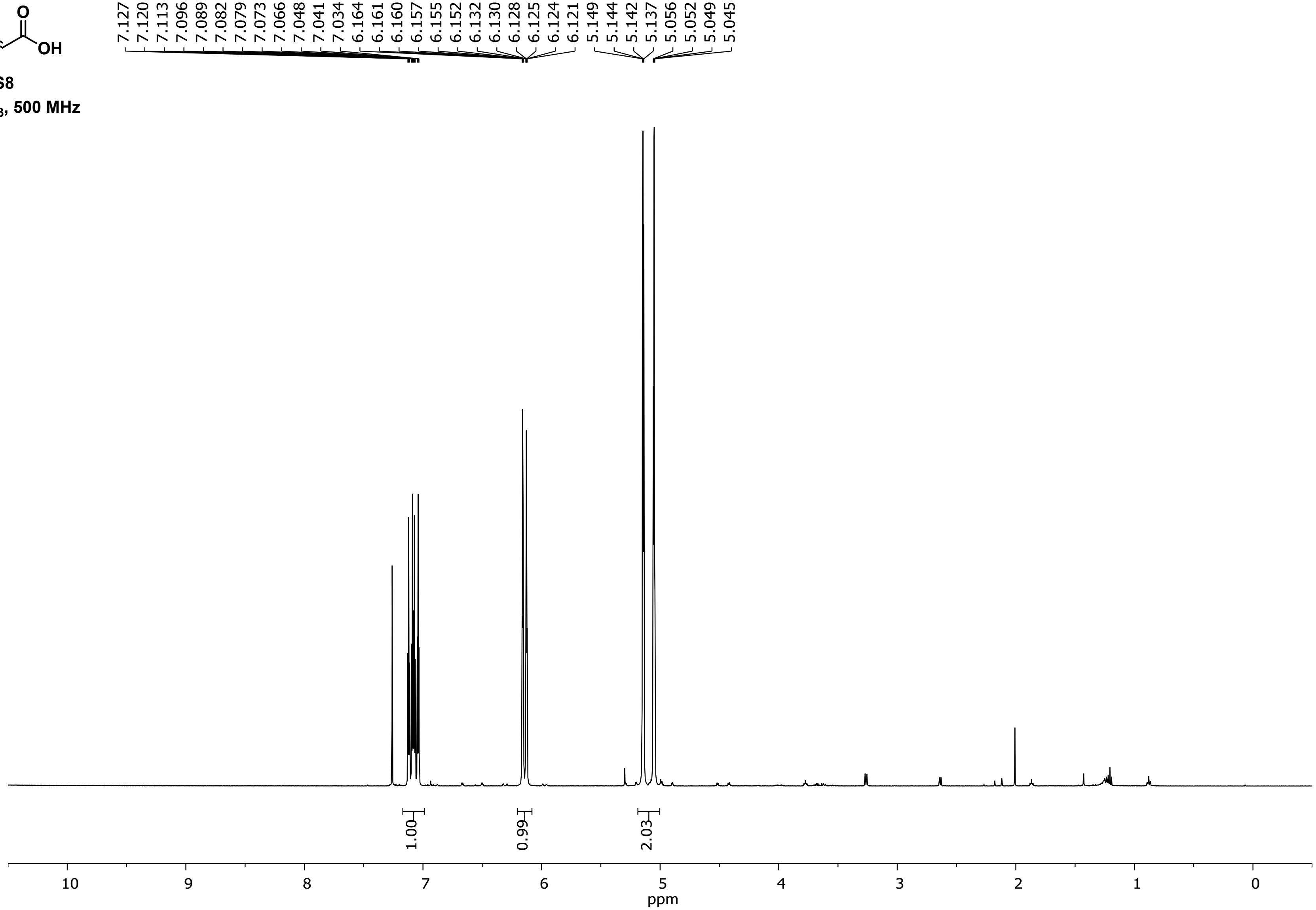

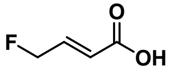

S8

$^{19}F\{^{1}H\}$, $CDCl_3$, 376 MHz

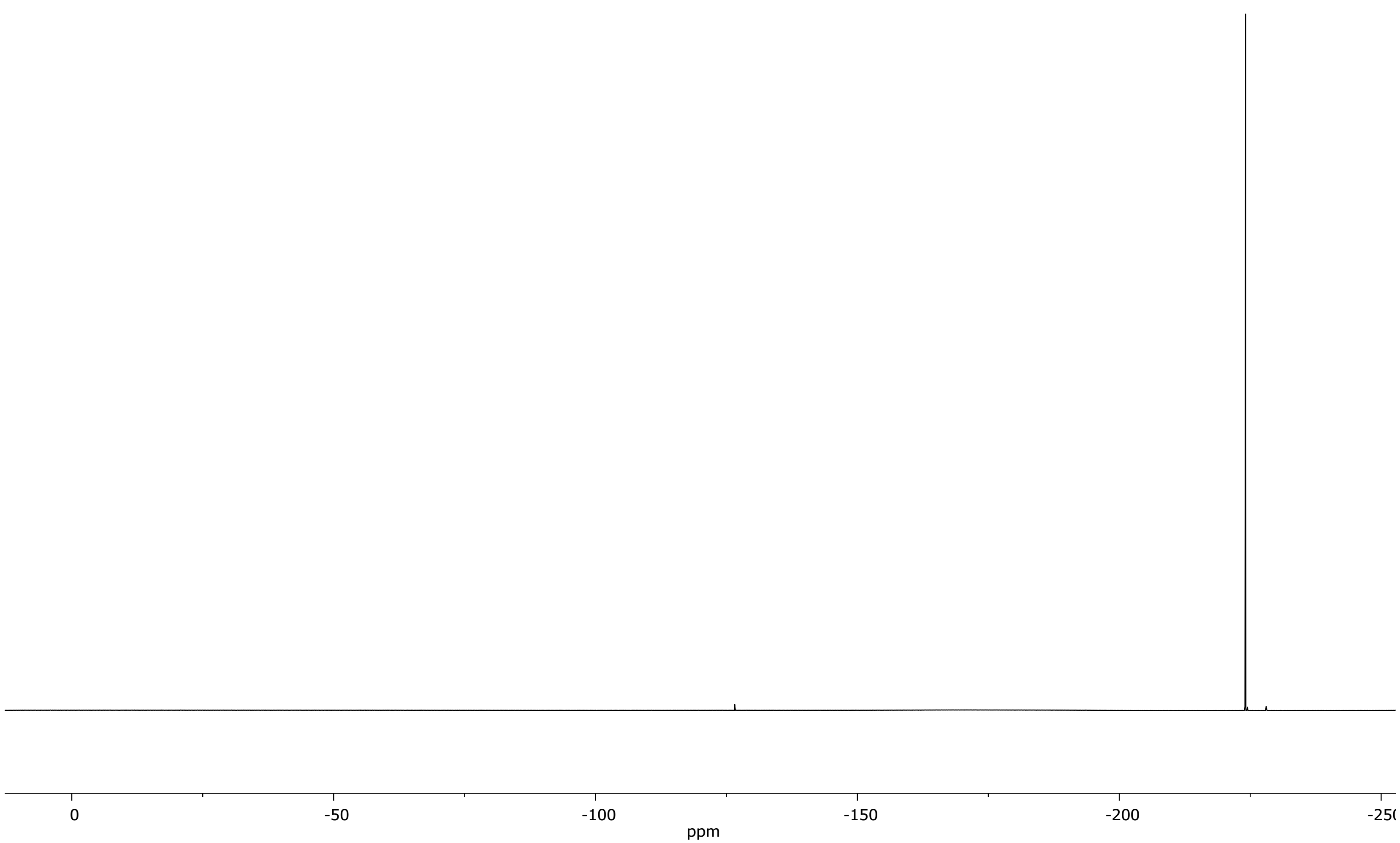

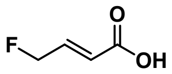

S8

$^{13}C\{^{1}H\}$, $CDCl_3$, 126 MHz

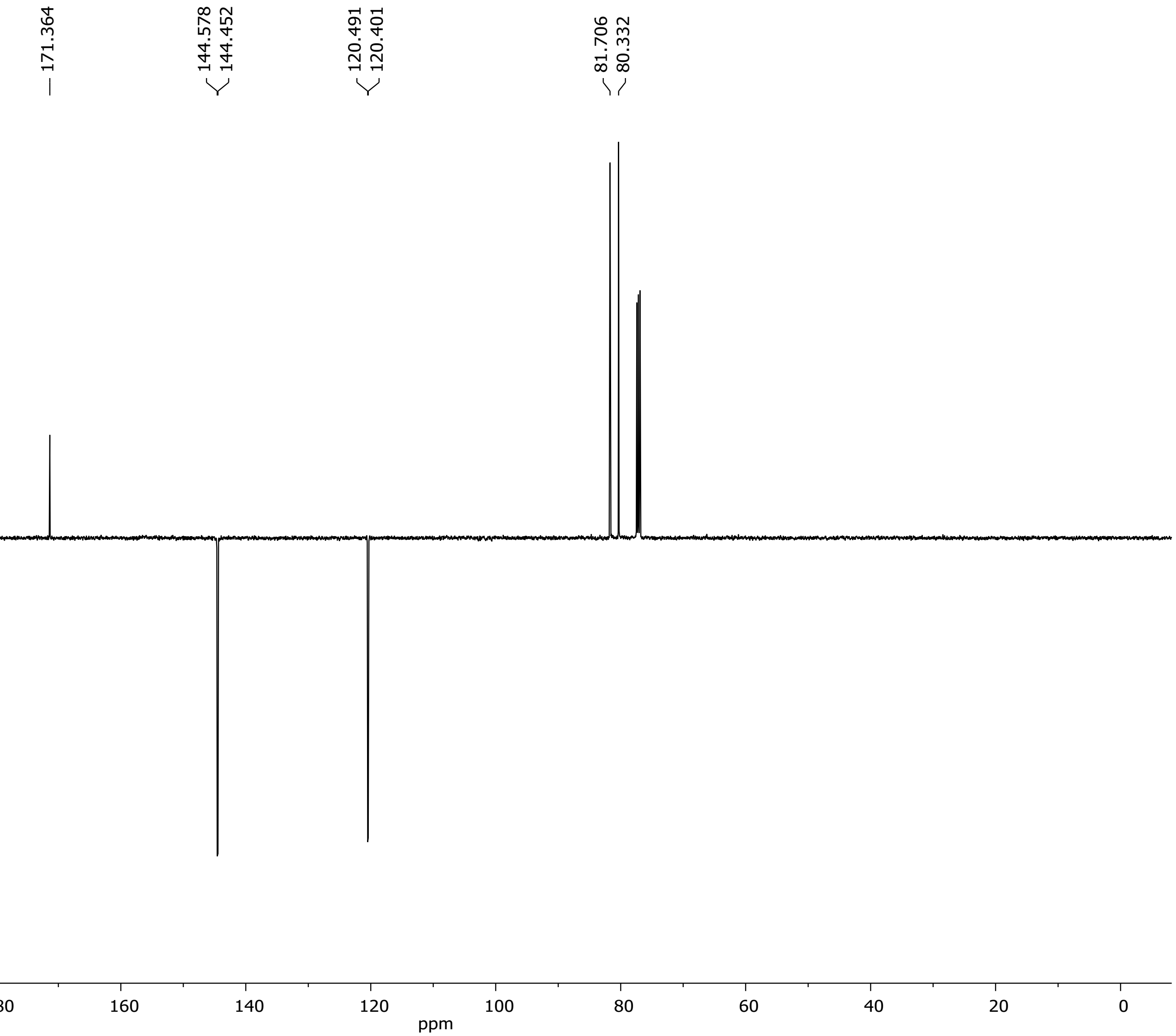

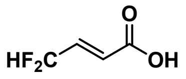

S9

^{1}H, $CDCl_3$, 400 MHz

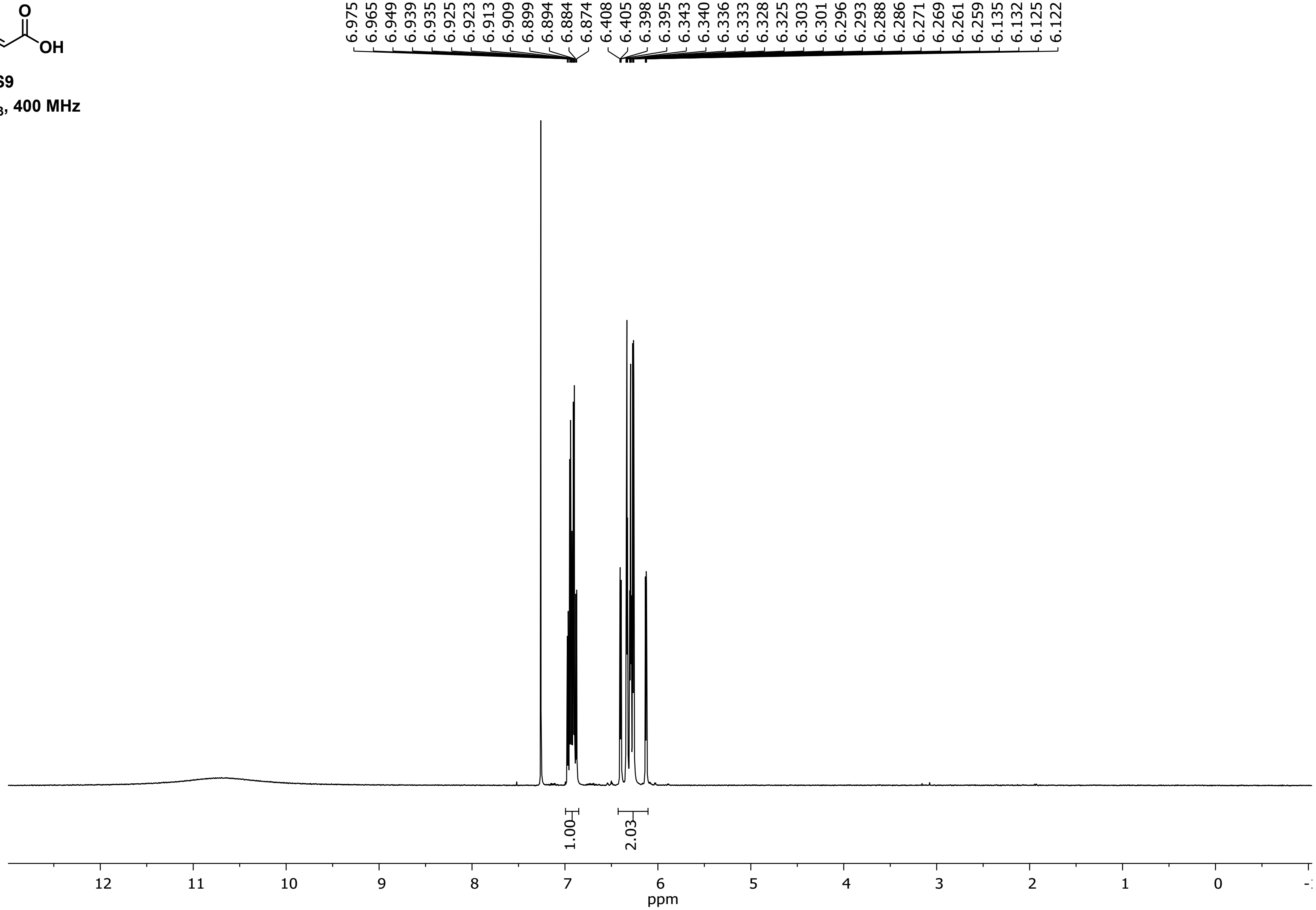

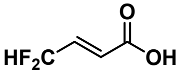

S9

$^{19}F\{^{1}H\}$, $CDCl_3$, 377 MHz

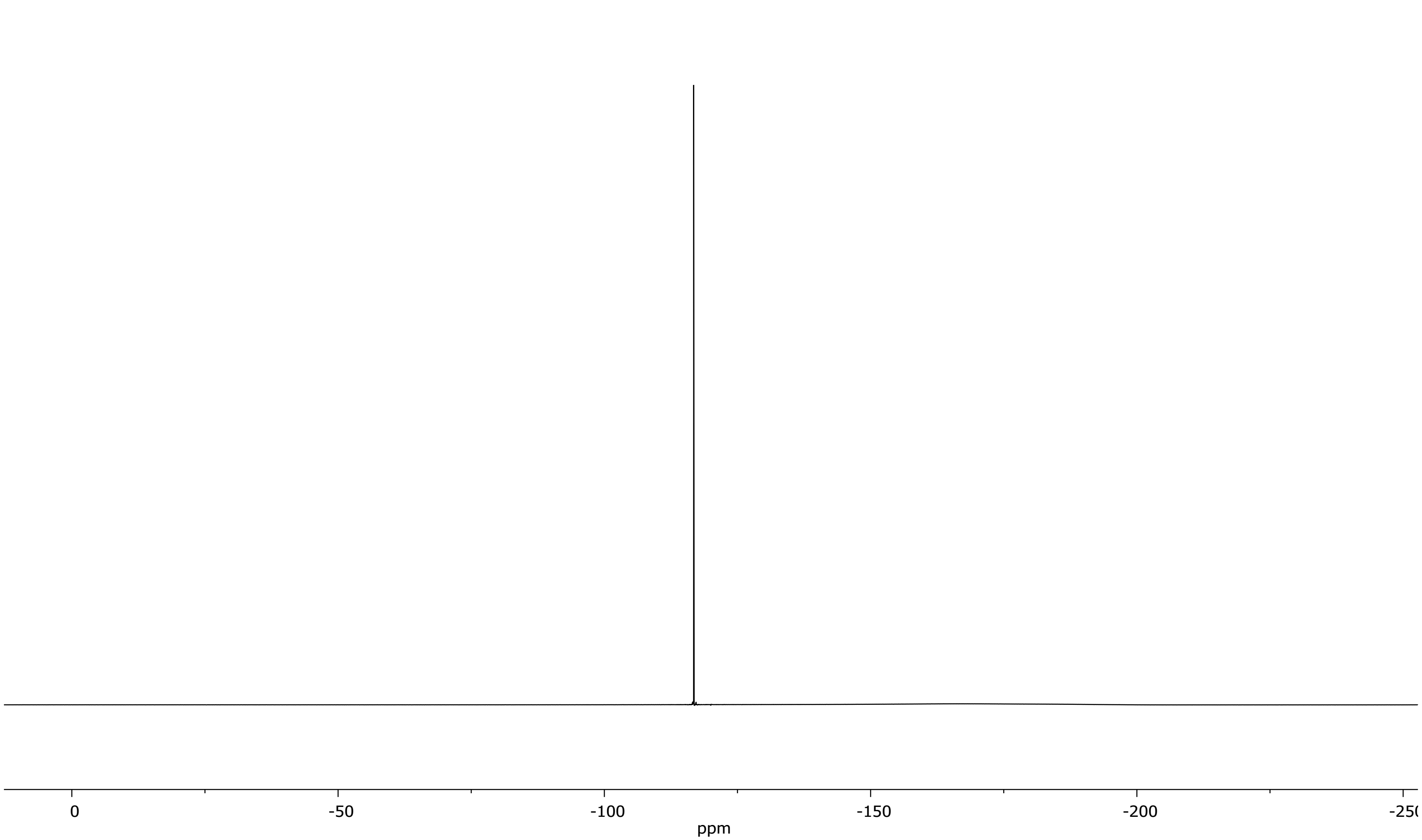

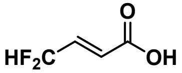

S9

$^{13}C\{^1H\}$, $CDCl_3$, 126 MHz

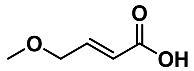

S10

^1H, $CDCl_3$, 400 MHz

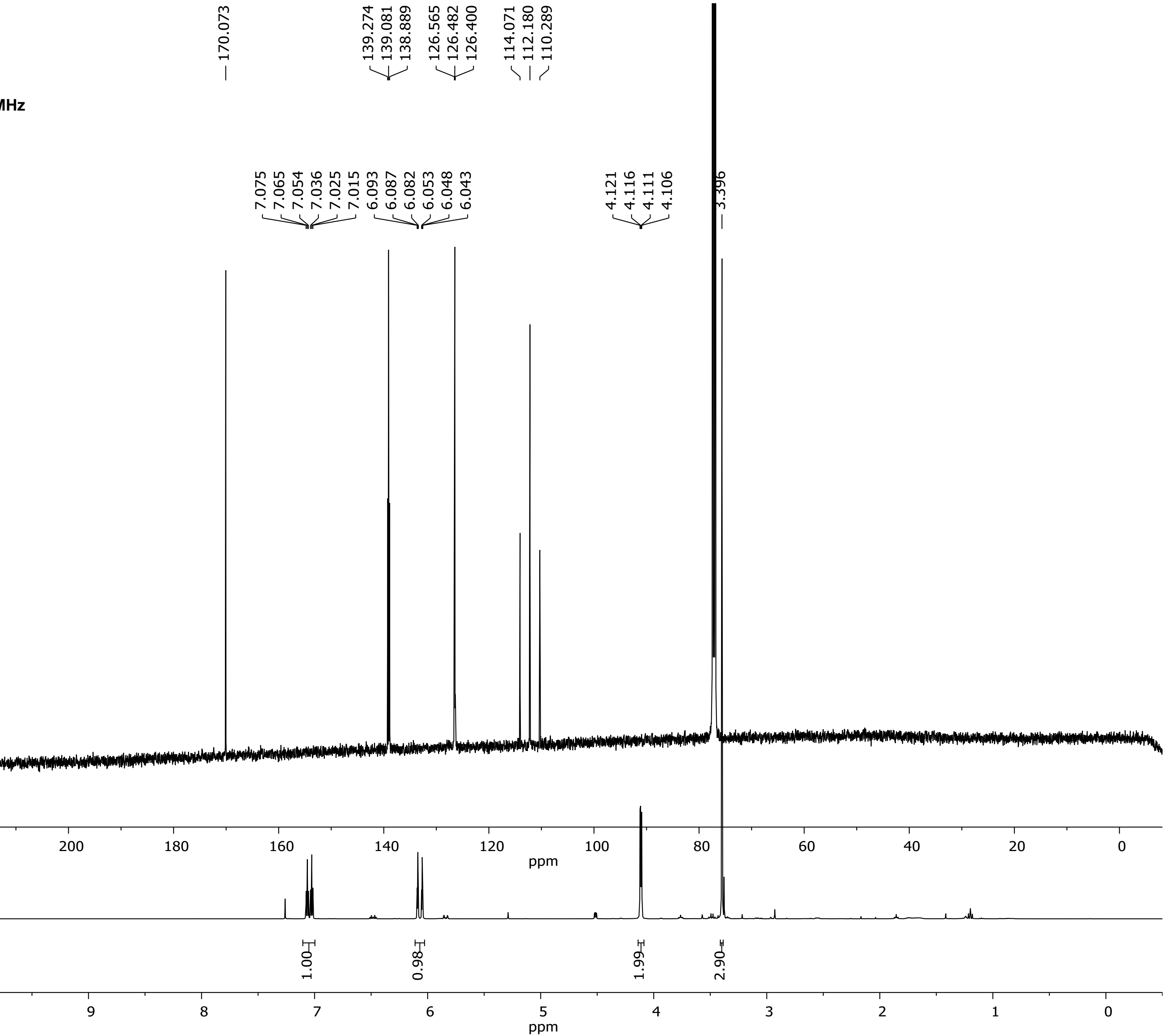

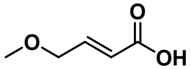

S10

$^{13}C\{^1H\}$, $CDCl_3$, 126 MHz

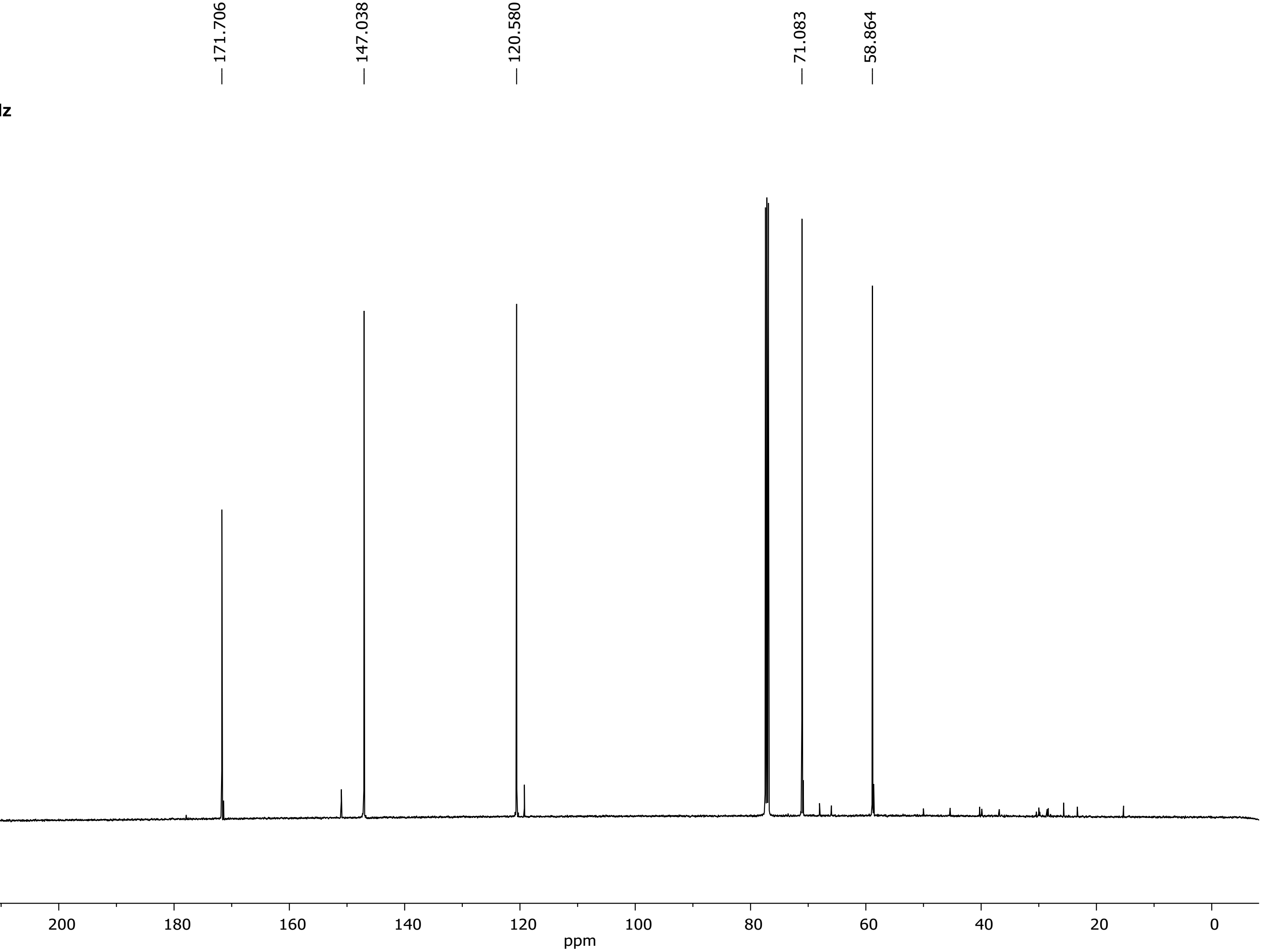

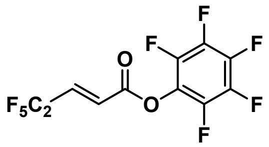

S11

^{1}H, $CDCl_3$, 400 MHz

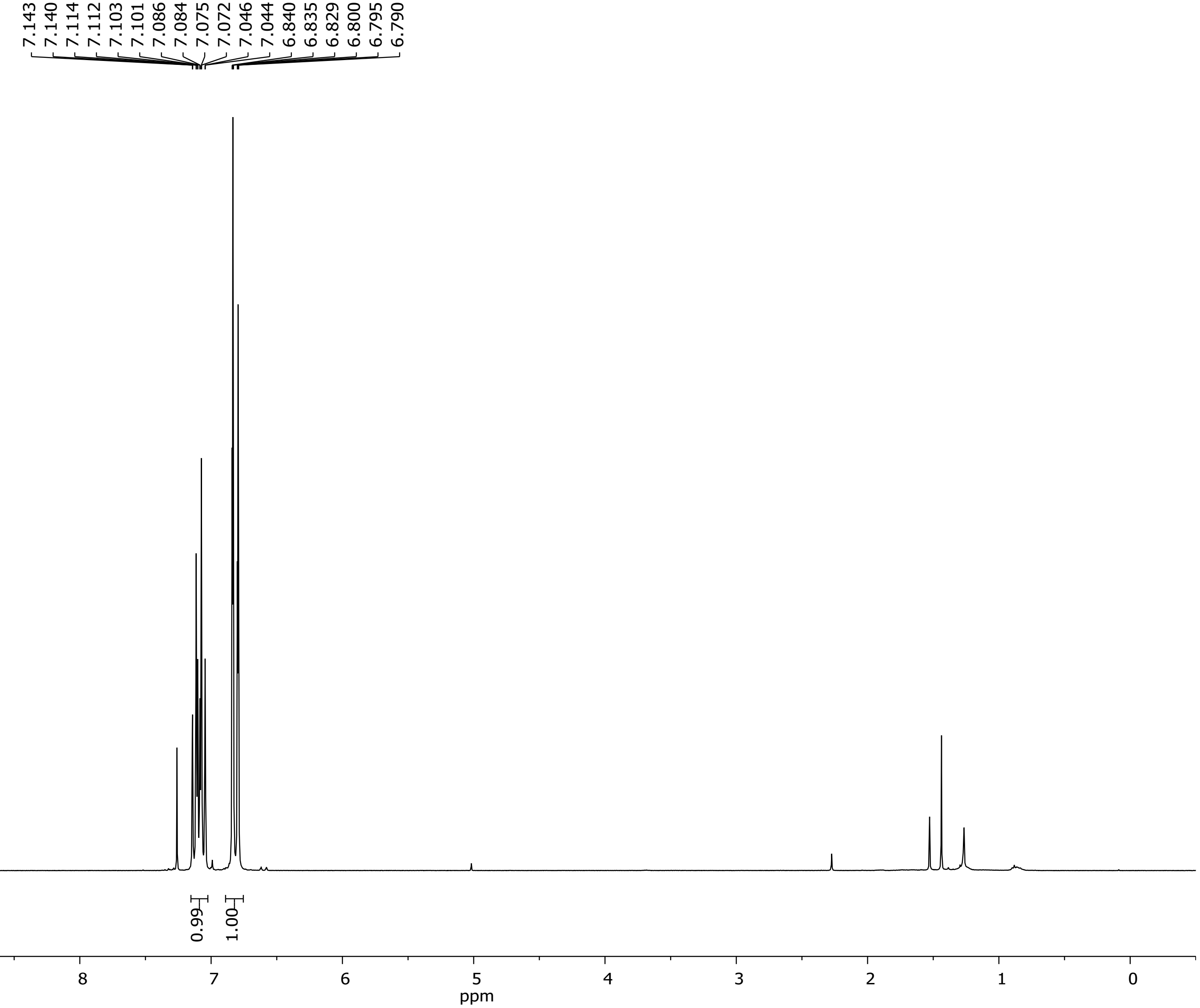

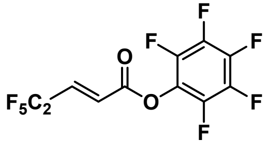

S11

$^{19}F\{^1H\}$, $CDCl_3$, 377 MHz

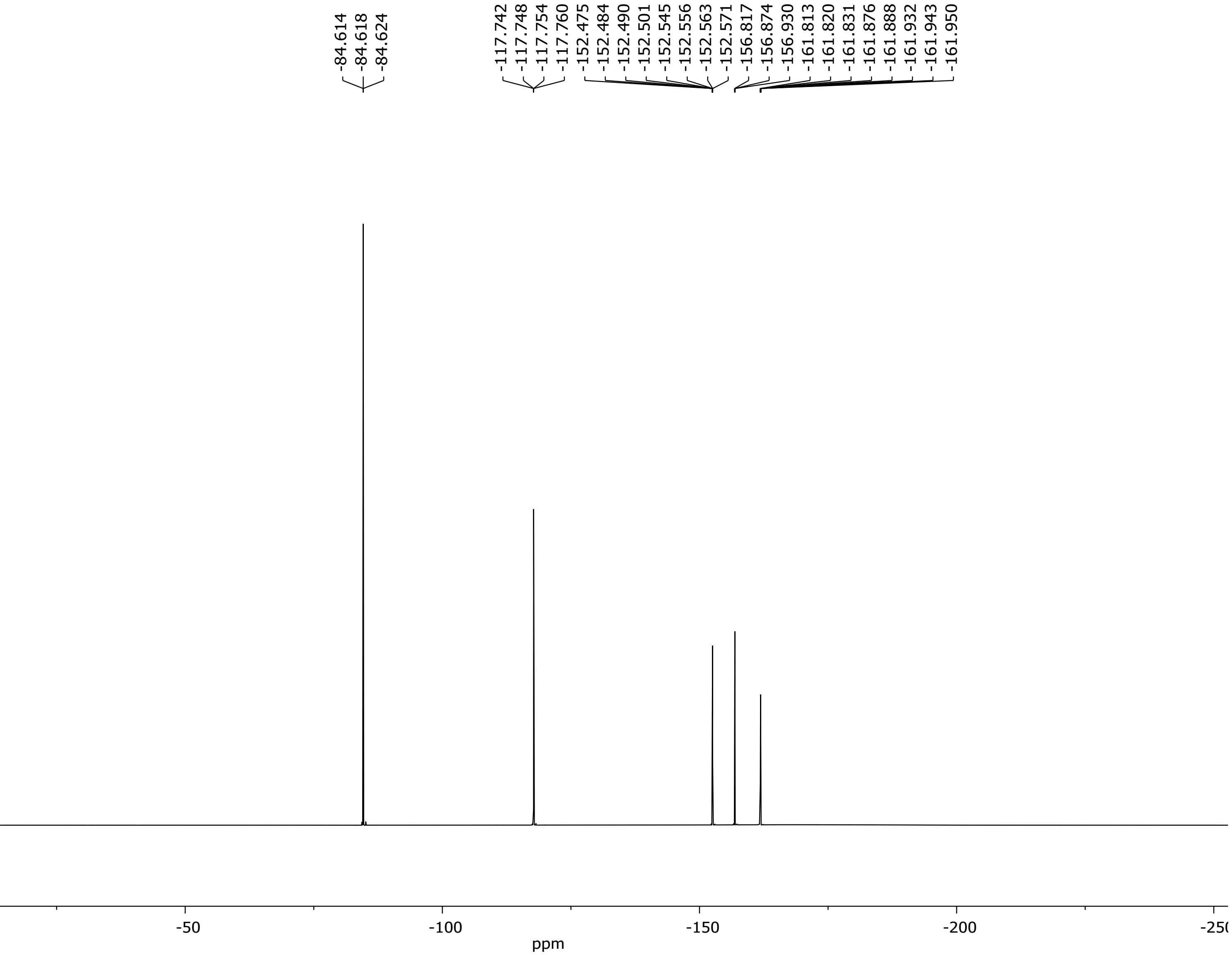

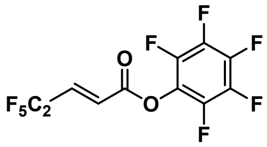

S11

$^{13}C\{^{1}H\}$, $CDCl_3$, 126 MHz

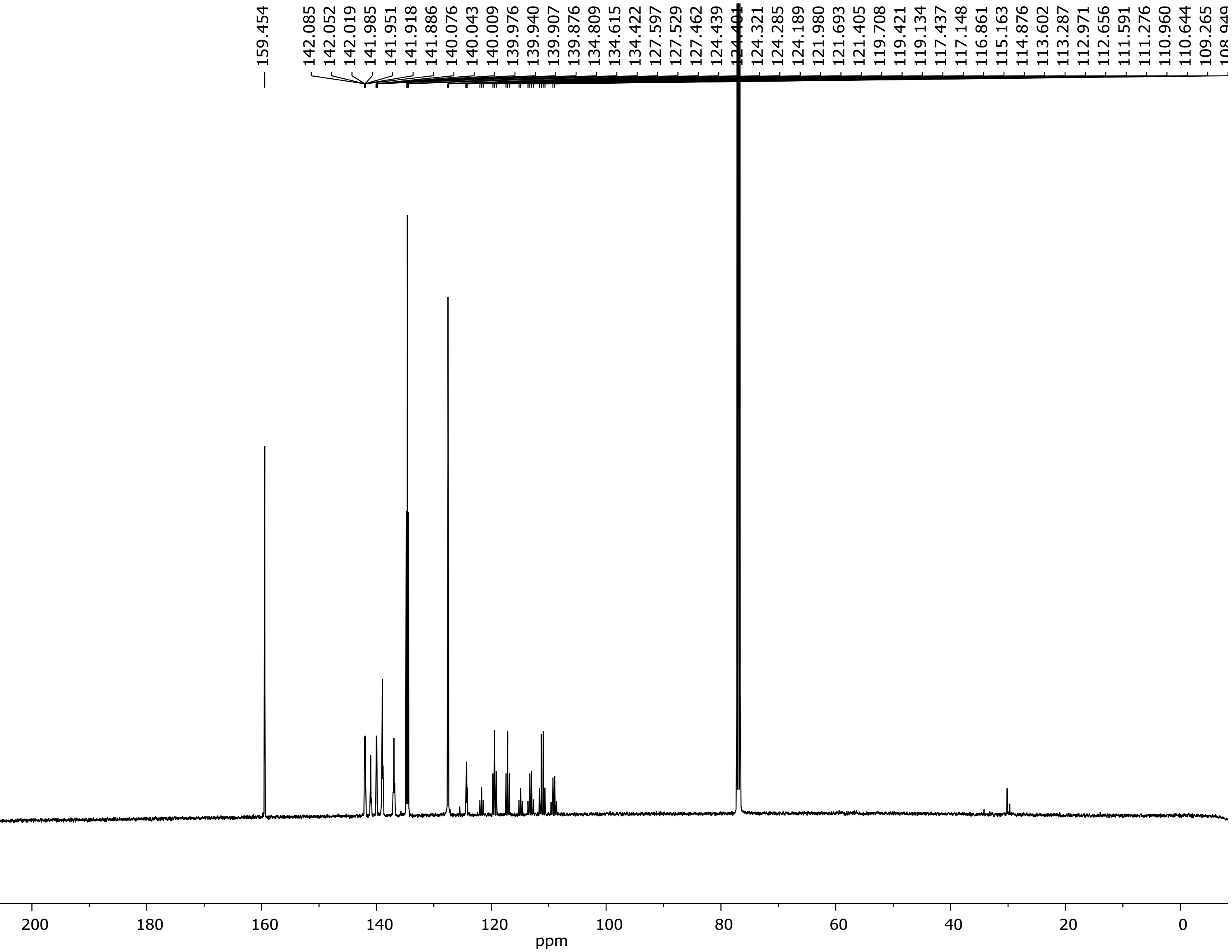

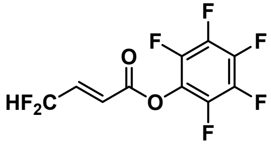

S12

^{1}H, $CDCl_3$, 400 MHz

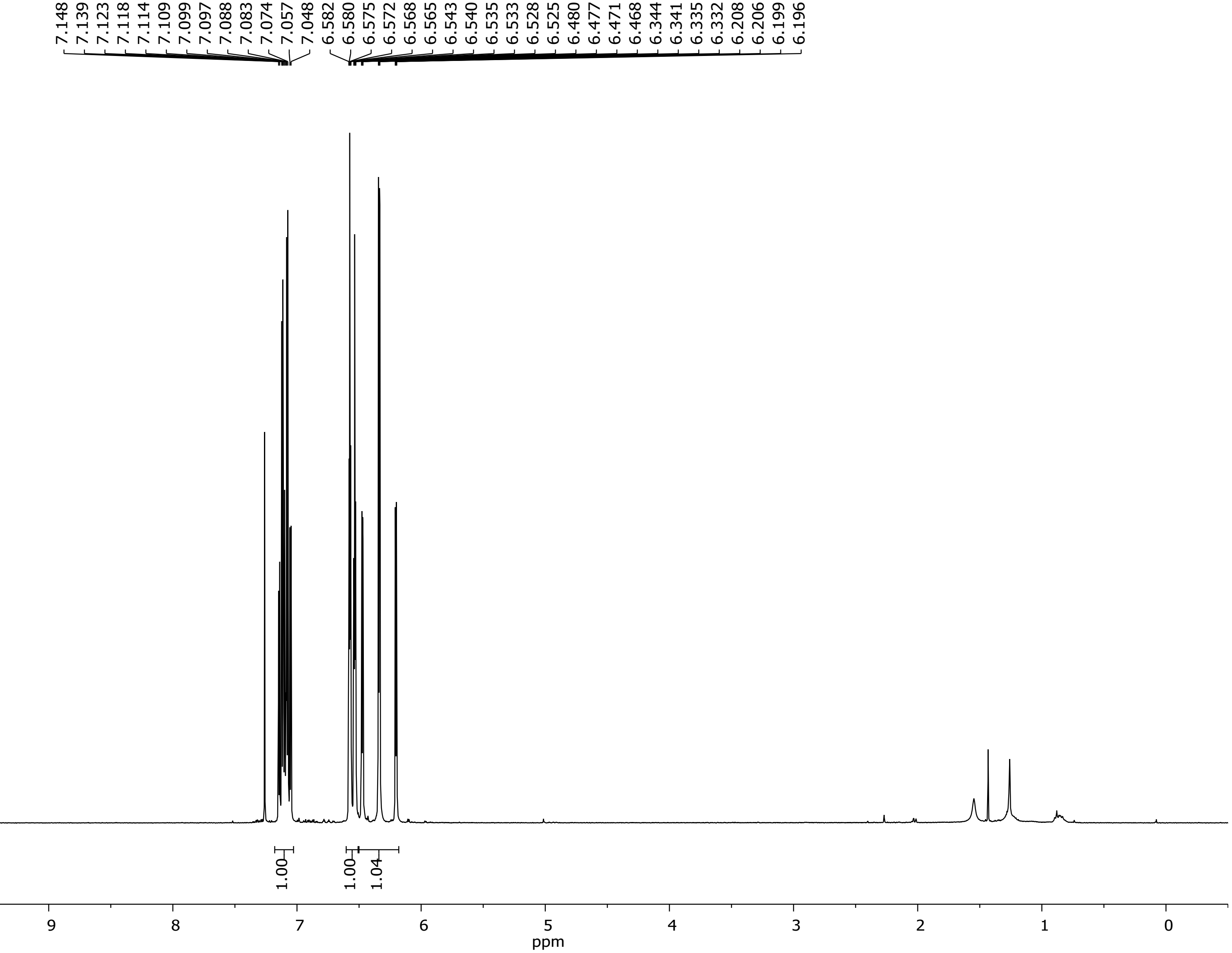

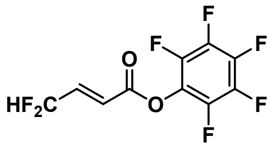

S12

$^{19}F\{^{1}H\}$, $CDCl_3$, 377 MHz

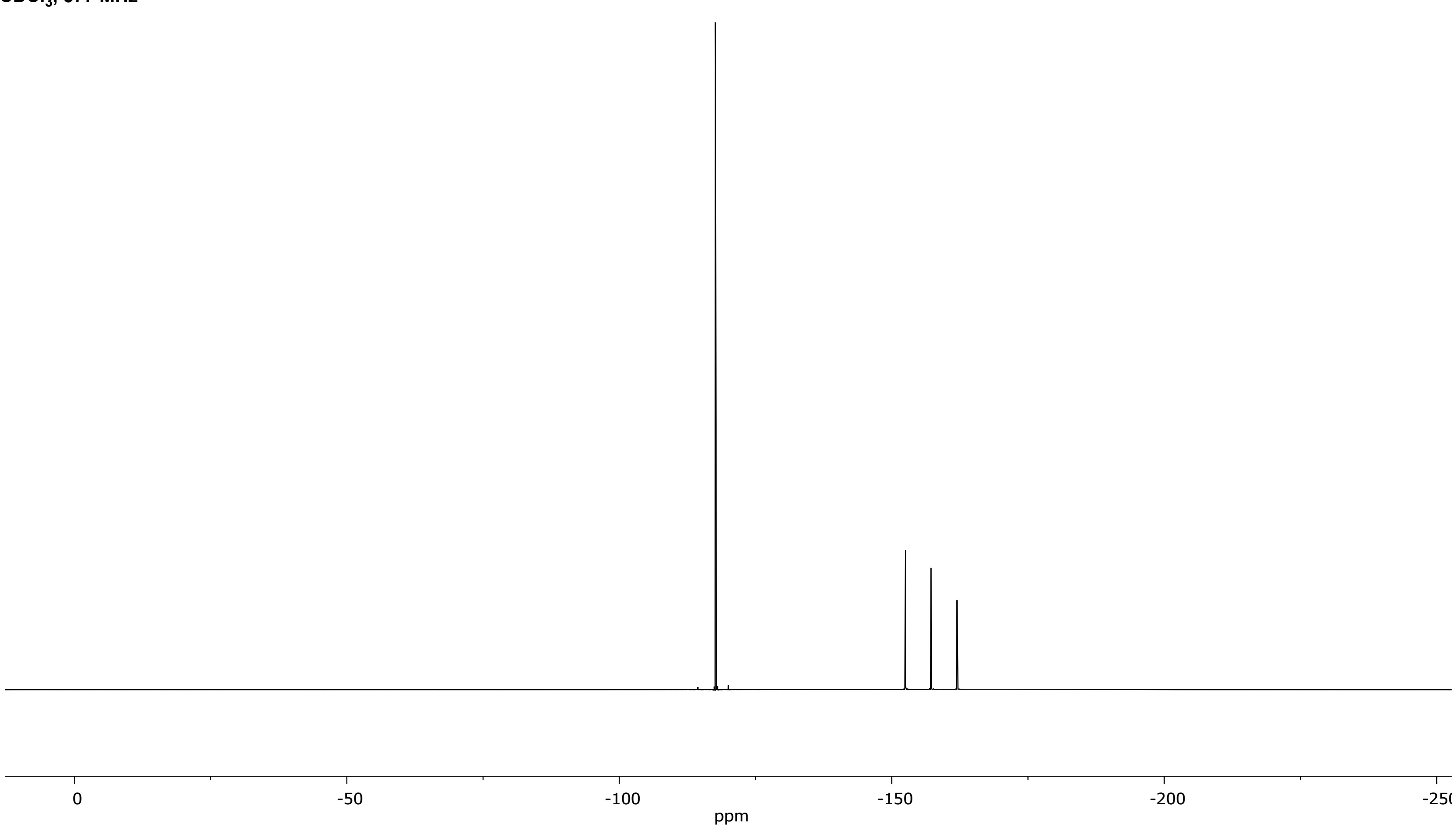

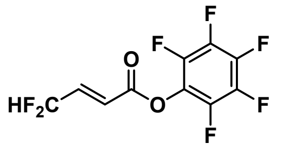

S12

$^{13}C\{^1H\}$, $CDCl_3$, 126 MHz

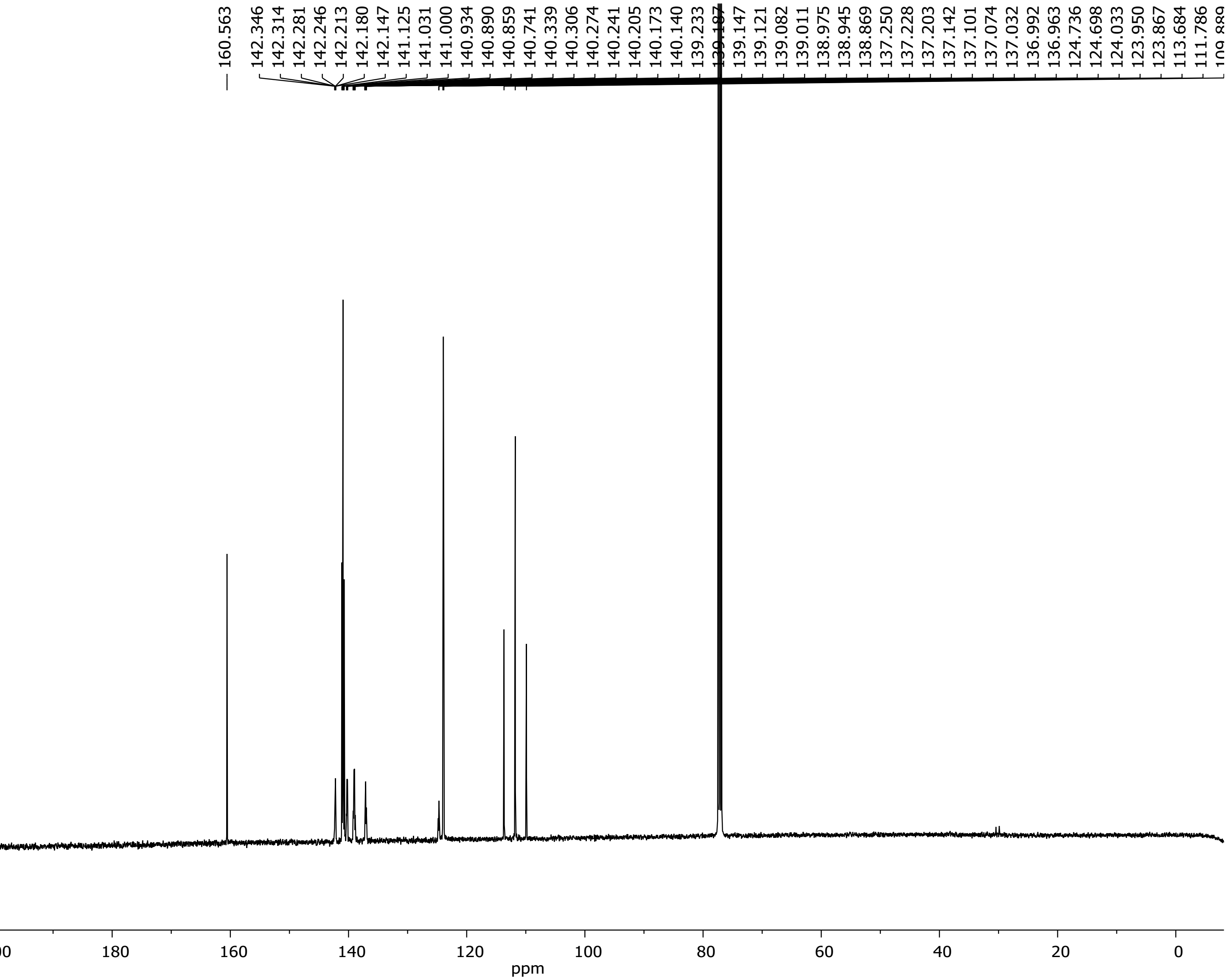

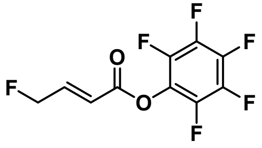

S13

^{1}H, $CDCl_3$, 400 MHz

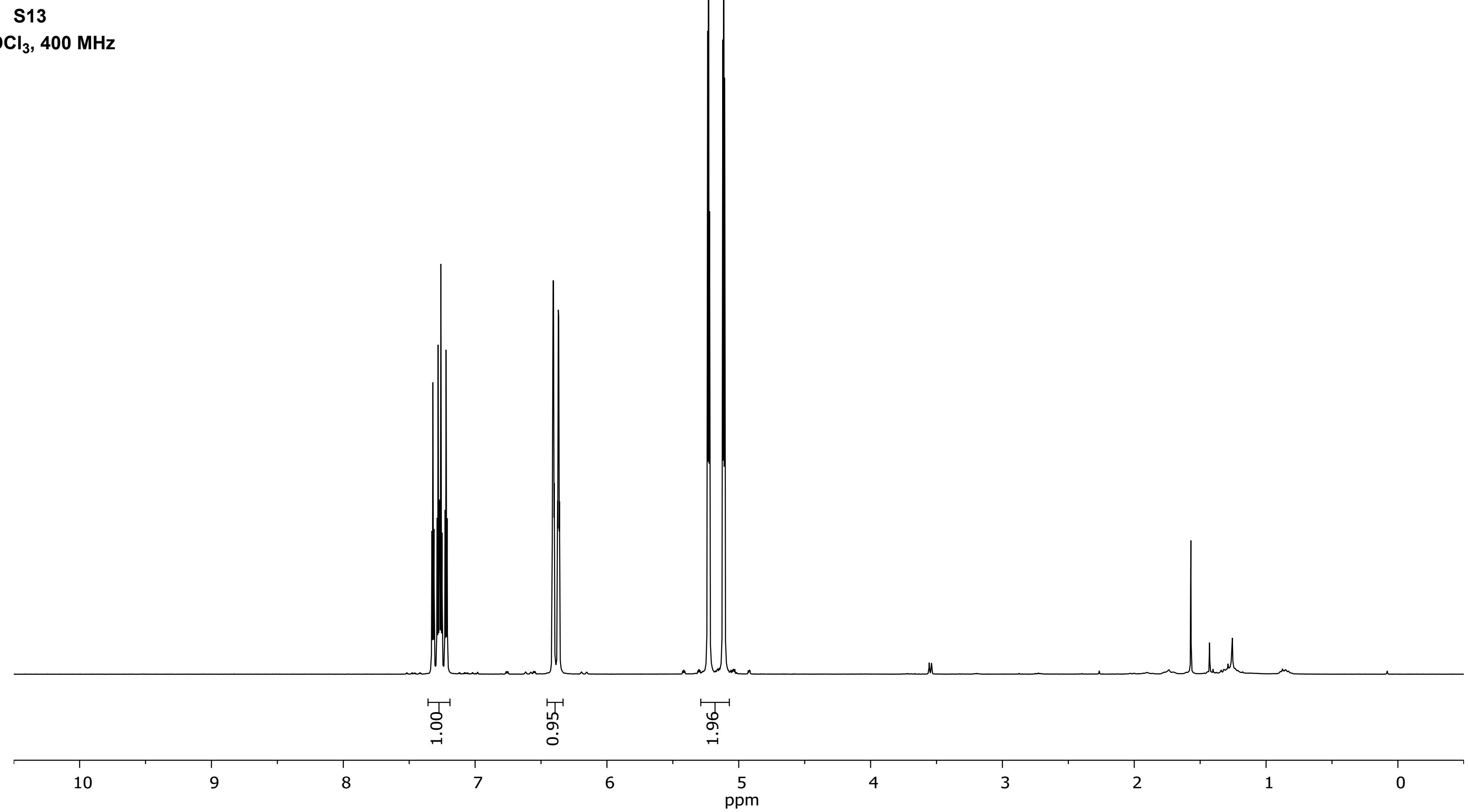

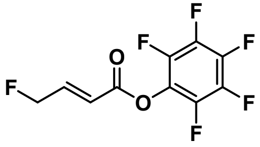

S13

$^{19}F\{^{1}H\}$, $CDCl_3$, 377 MHz

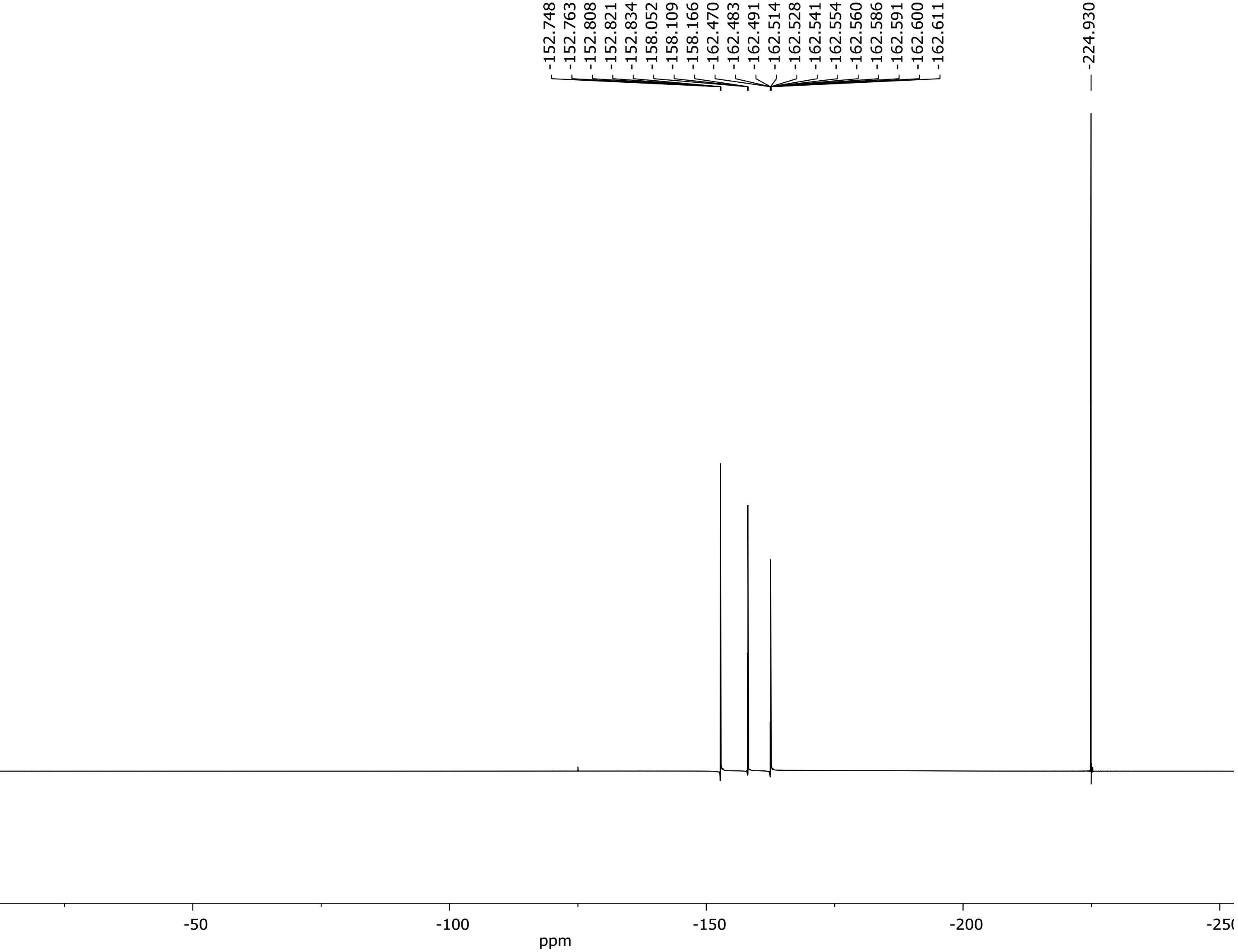

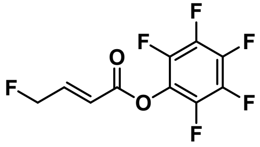

S13

$^{13}C\{^{1}H\}$, $CDCl_3$, 126 MHz

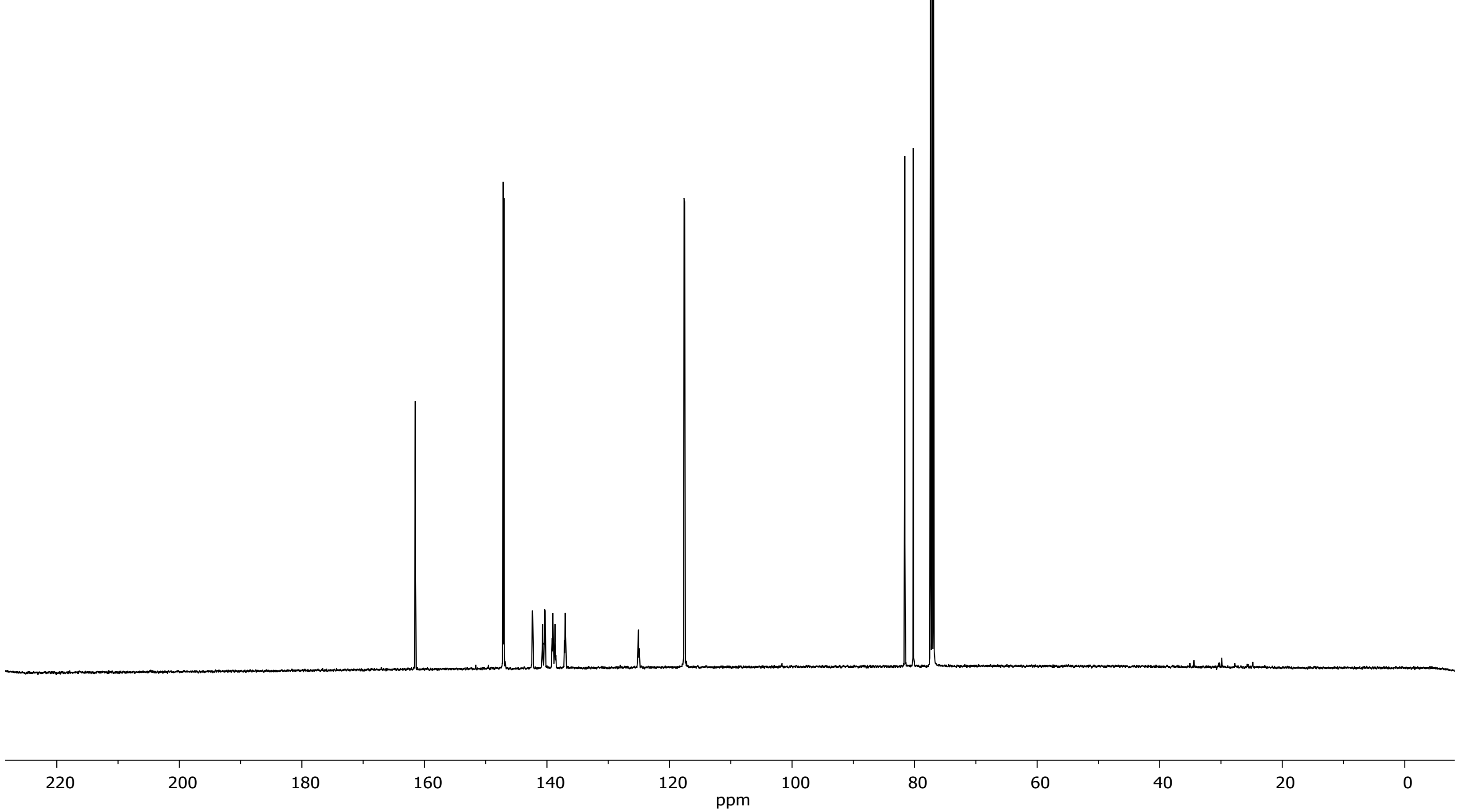

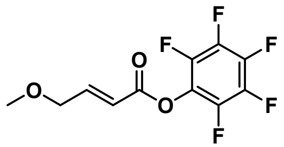

S14
^{1}H, CDCl$_3$, 400 MHz

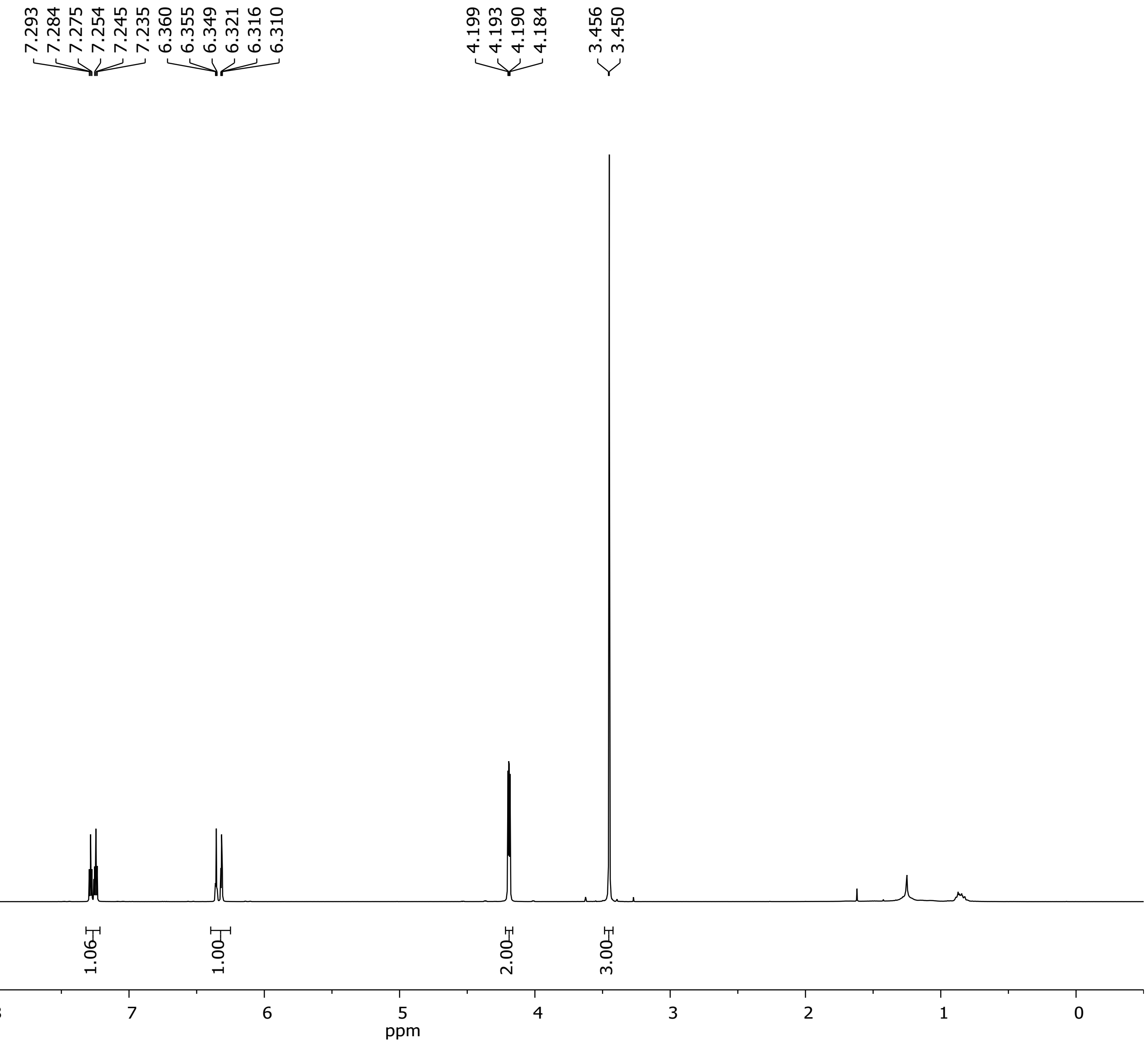

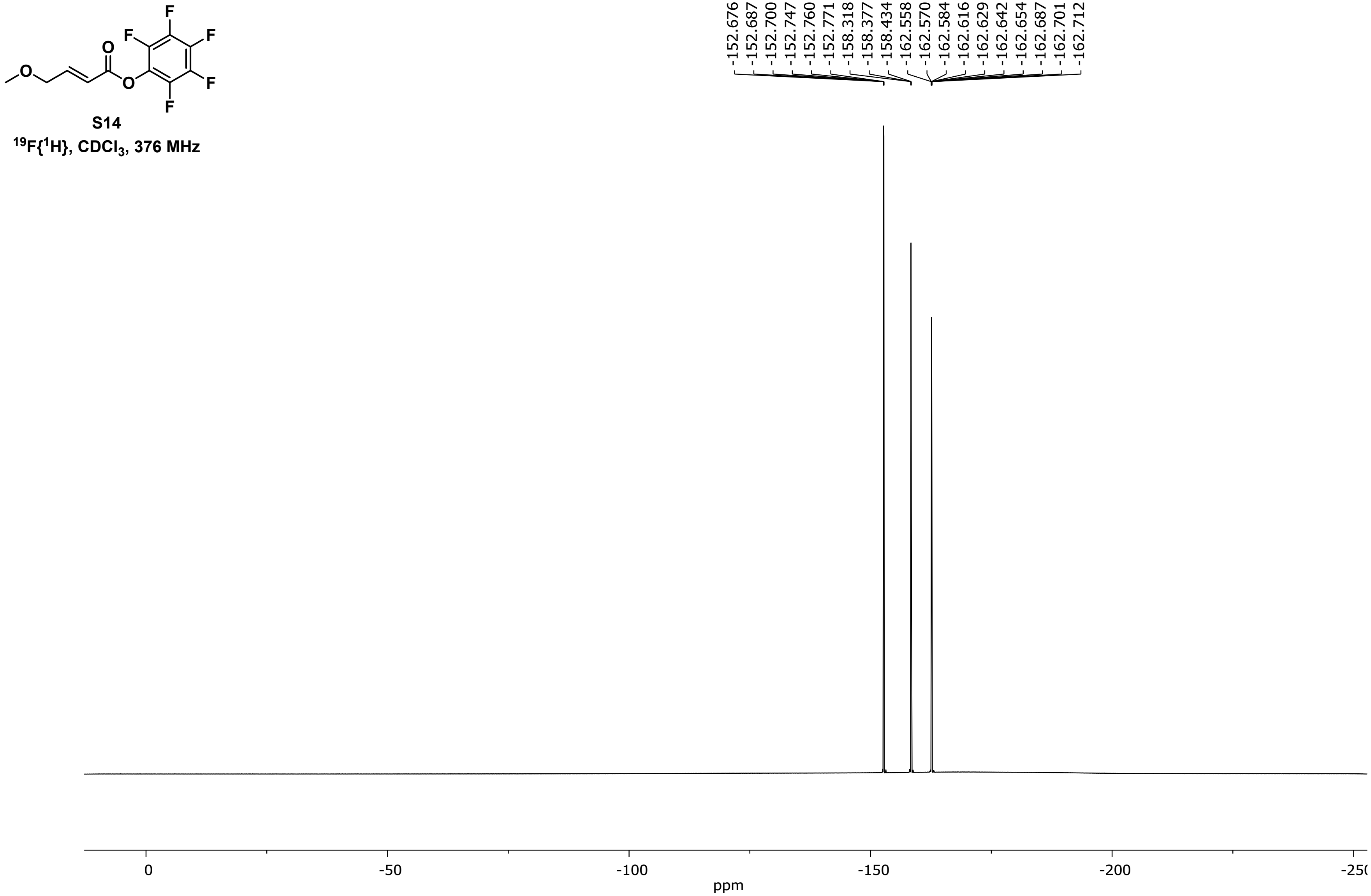

S14

$^{19}F\{^1H\}$, $CDCl_3$, 376 MHz

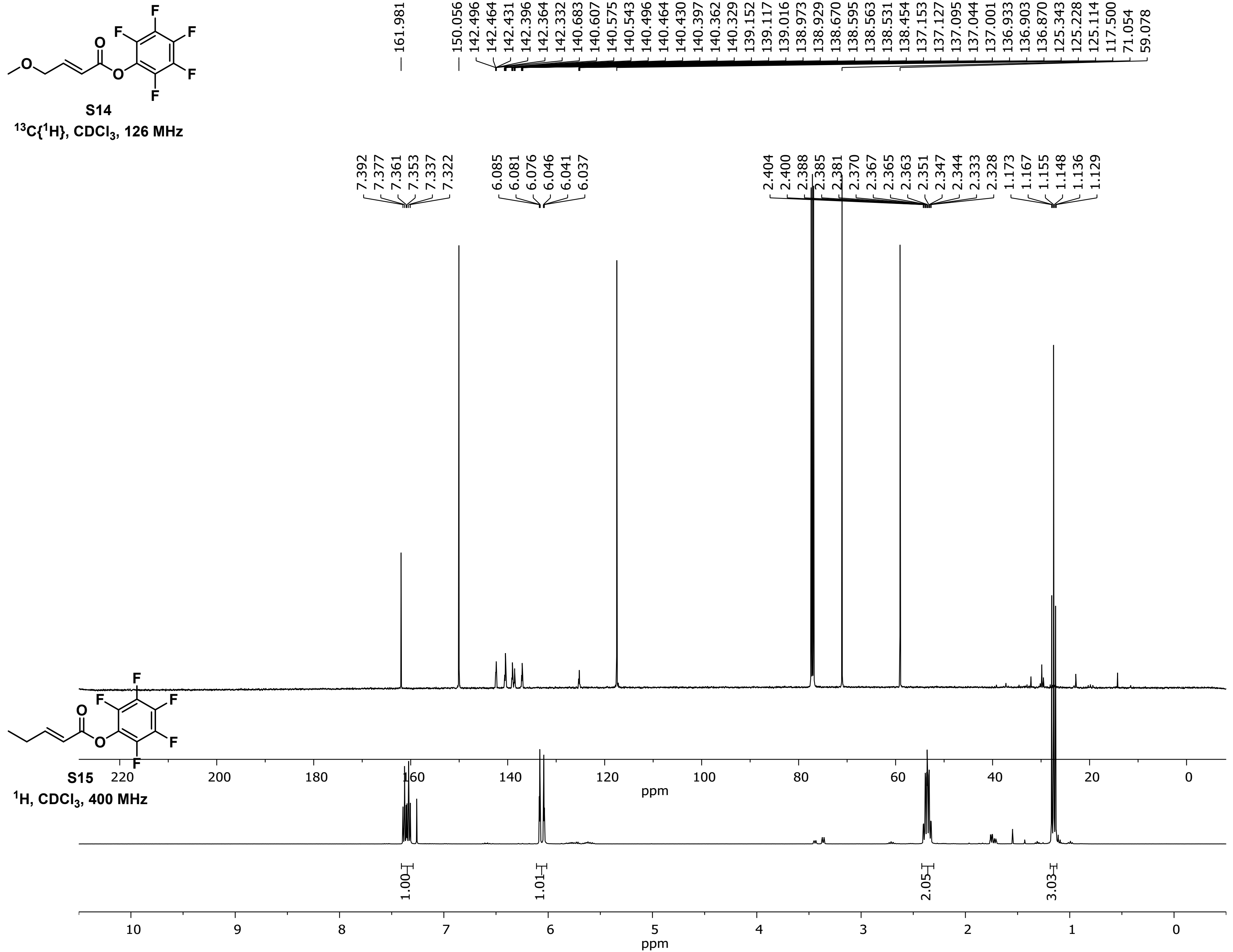
S14
$^{13}C\{^1H\}$, $CDCl_3$, 126 MHz
161.981
150.056 142.496 142.464 142.431 142.396 142.364 142.332 140.683 140.607 140.575 140.543 140.496 140.464 140.430 140.397 140.362 140.329 139.152 139.117 139.016 138.973 138.929 138.670 138.595 138.563 138.531 138.454 137.153 137.127 137.095 137.044 137.001 136.933 136.903 136.870 125.343 125.228 125.114 117.500 71.054 59.078
7.392 7.377 7.361 7.353 7.337 7.322
6.085 6.081 6.076 6.046 6.041 6.037
2.404 2.400 2.388 2.385 2.381 2.370 2.367 2.365 2.363 2.351 2.347 2.344 2.333 2.328
1.173 1.167 1.155 1.148 1.136 1.129
220 200 180 160 140 120 100 80 60 40 20 0
ppm
S15
^1H, $CDCl_3$, 400 MHz
1.00 1.01 2.05 3.03
10 9 8 7 6 5 4 3 2 1 0
ppm

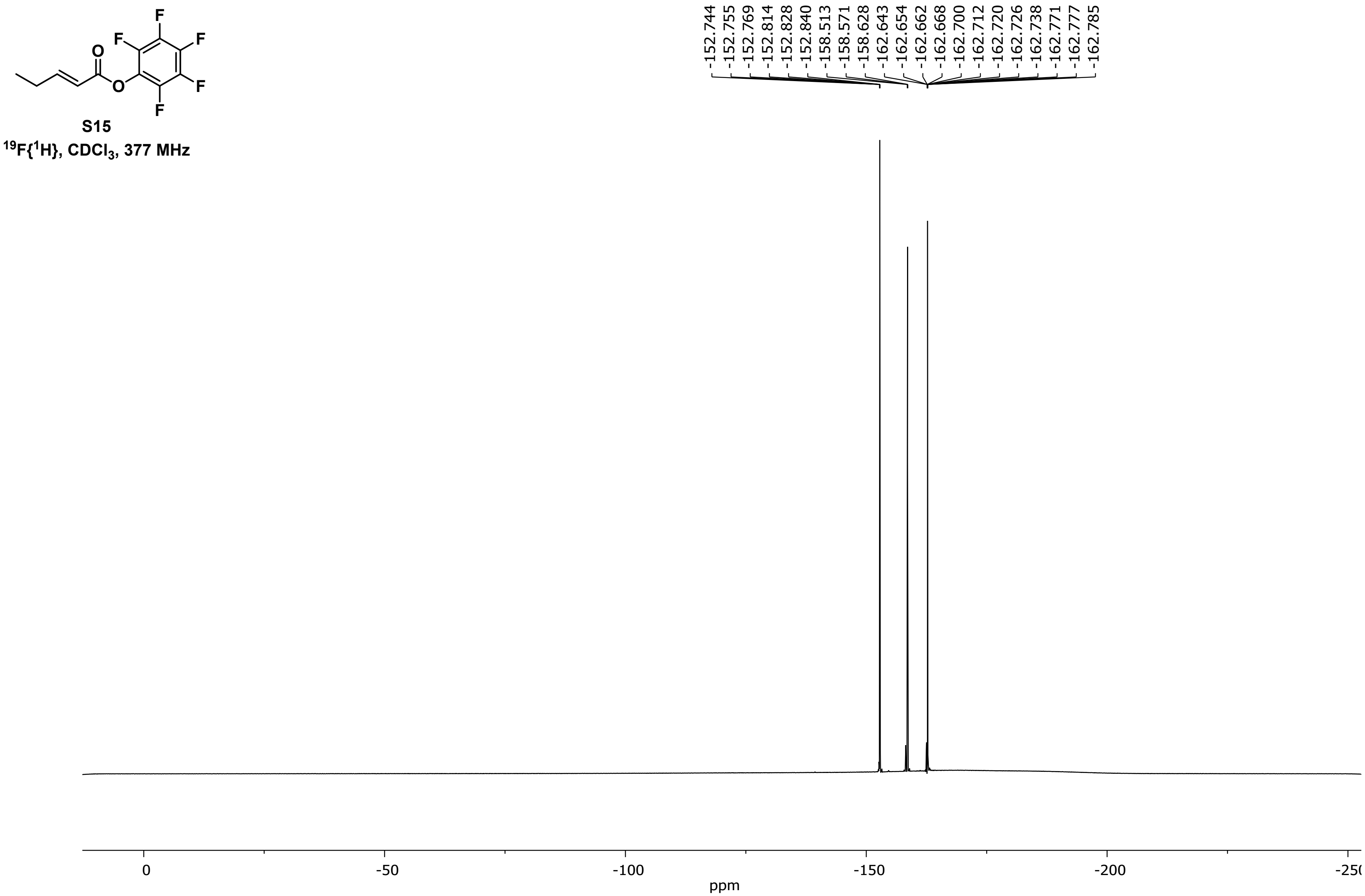
S15
$^{19}F\{^1H\}$, $CDCl_3$, 377 MHz
-152.744
-152.755
-152.769
-152.814
-152.828
-152.840
-158.513
-158.571
-158.628
-162.643
-162.654
-162.662
-162.668
-162.700
-162.712
-162.720
-162.726
-162.738
-162.771
-162.777
-162.785
0
-50
-100
-150
-200
ppm

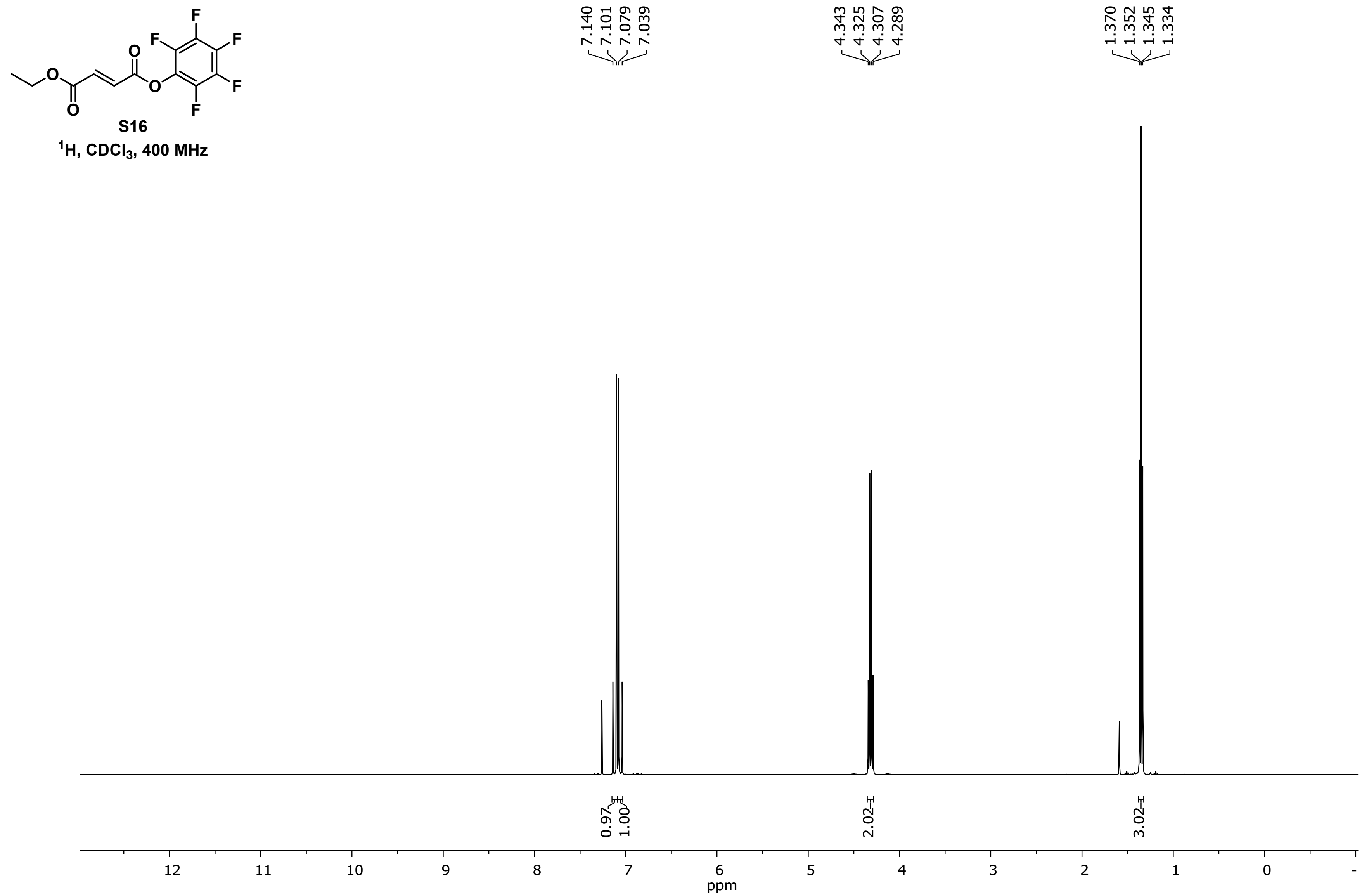
S16
^{1}H, CDCl$_3$, 400 MHz
7.140
7.101
7.079
7.039
4.343
4.325
4.307
4.289
1.370
1.352
1.345
1.334
0.97
1.00
2.02
3.02
12
11
10
9
8
7
6
5
4
3
2
1
0
ppm

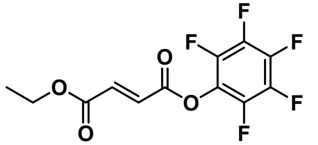

S16

$^{19}F\{^1H\}$, $CDCl_3$, 376 MHz

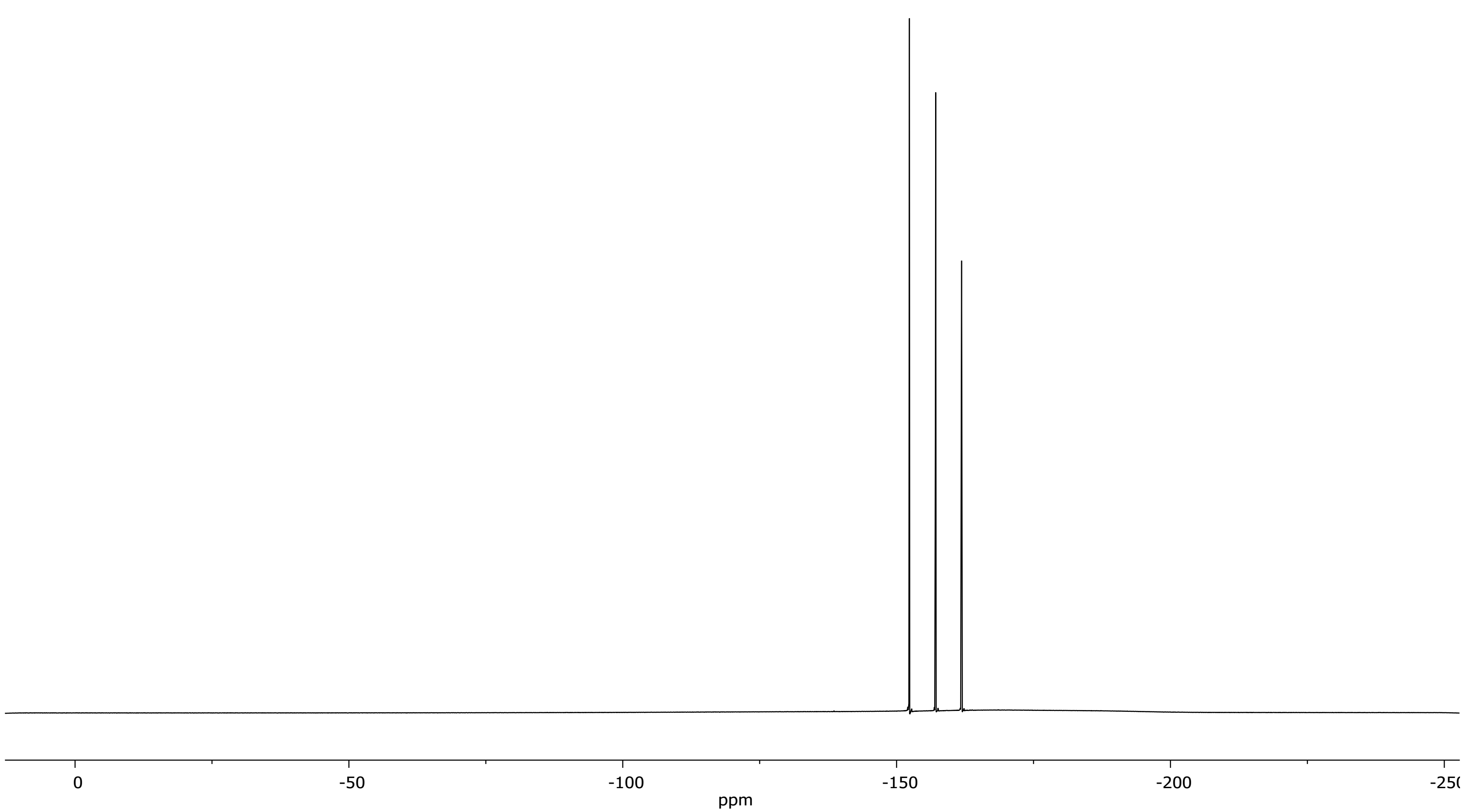

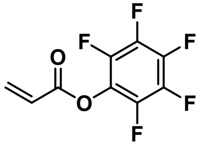

75

^{1}H, $CDCl_3$, 400 MHz

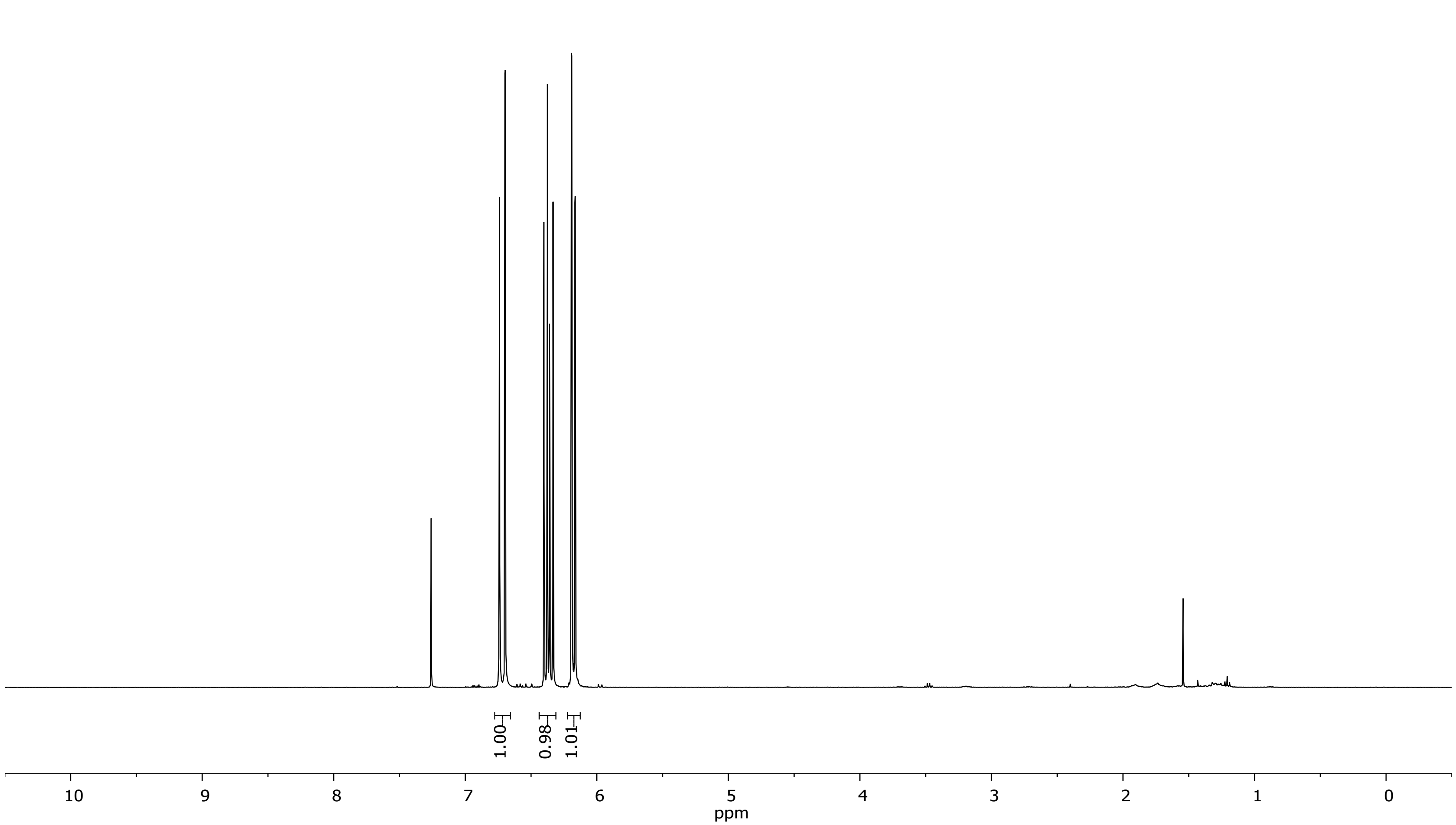

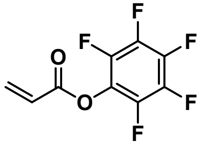

75

$^{19}F\{^{1}H\}$, $CDCl_3$, 377 MHz

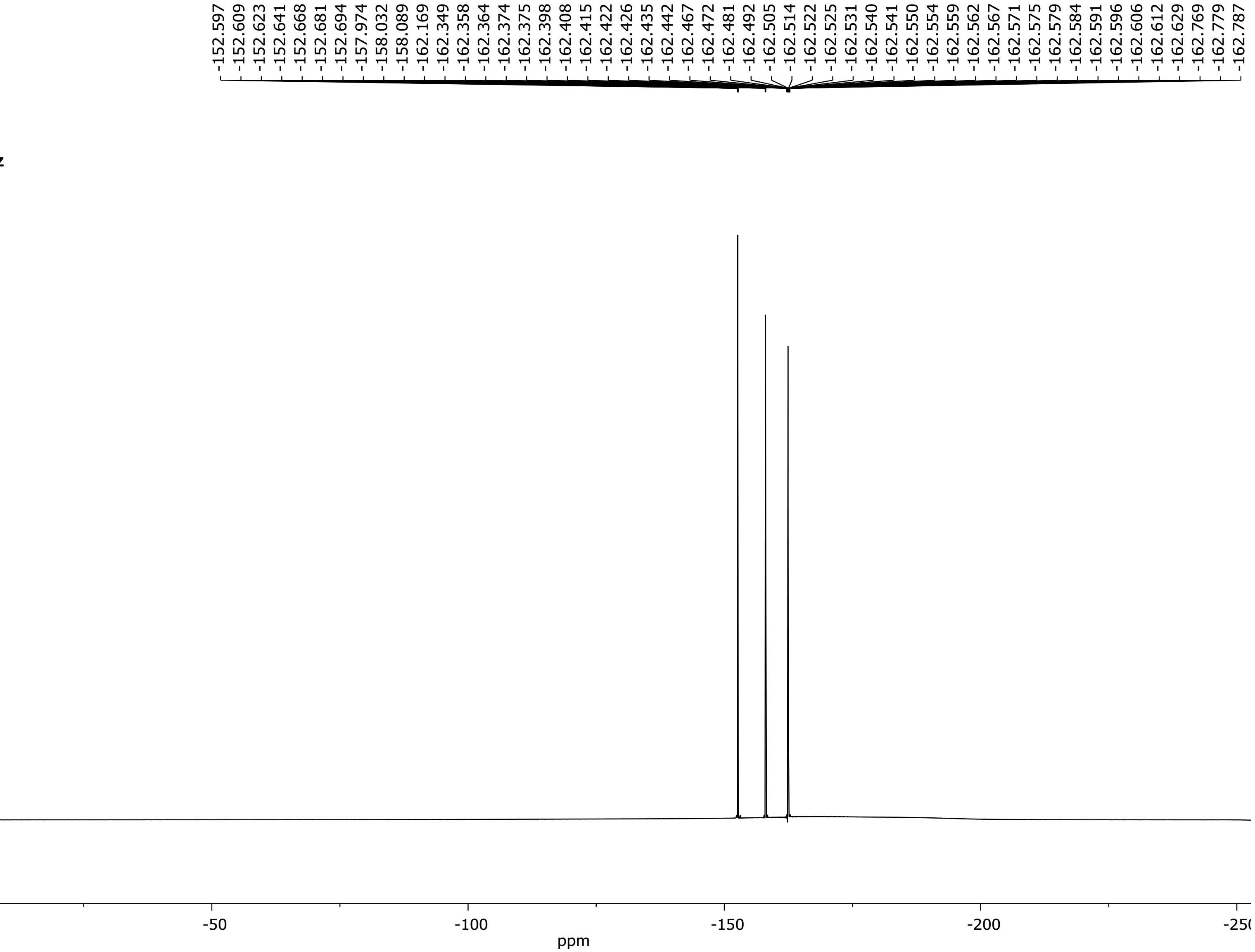

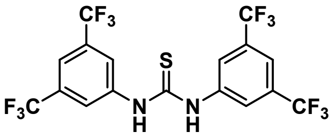

26
^{1}H, CDCl$_3$, 400 MHz

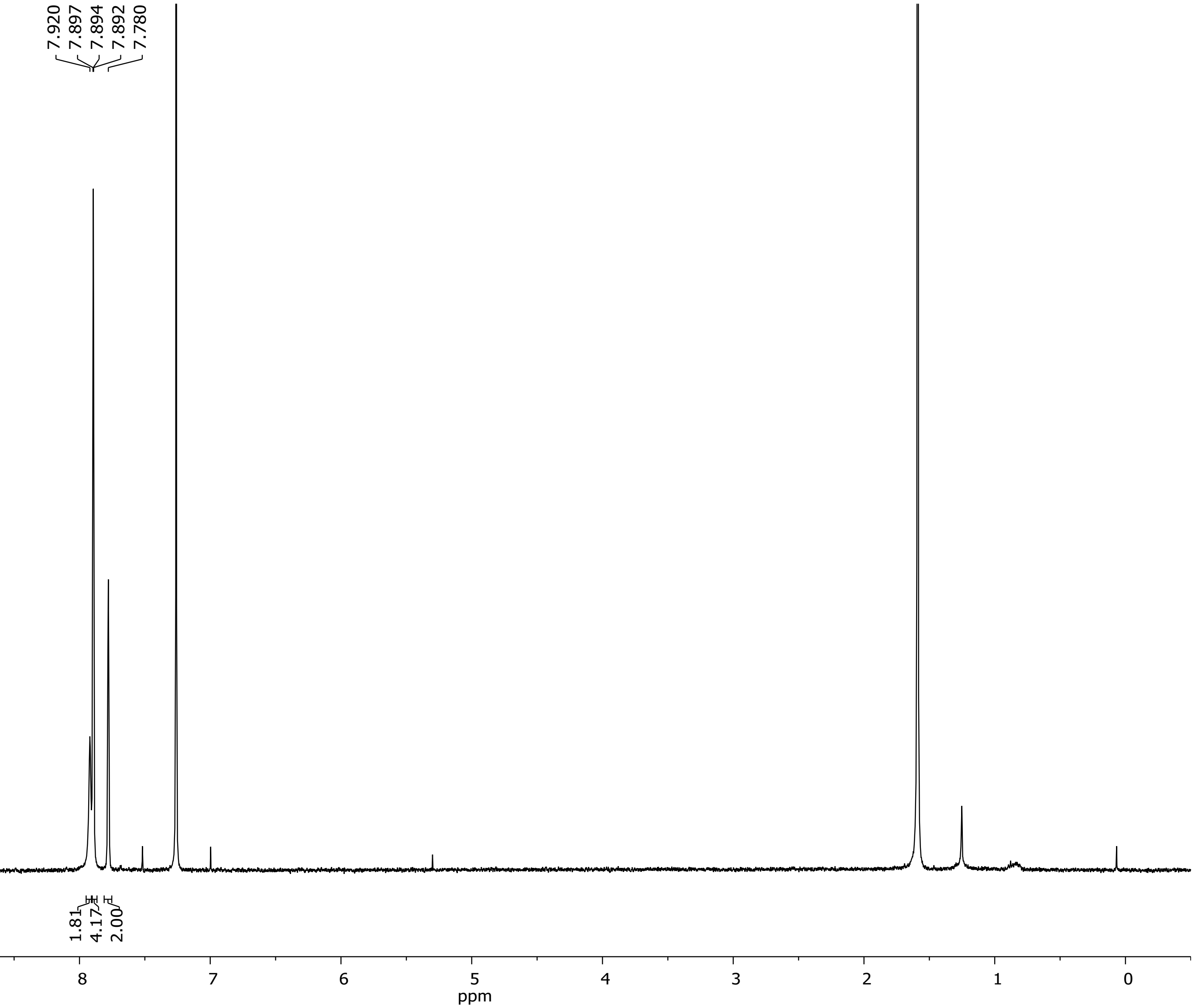

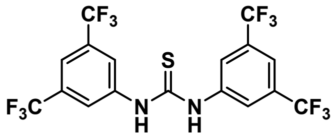

26

$^{19}F\{^1H\}$, $CDCl_3$, 377 MHz

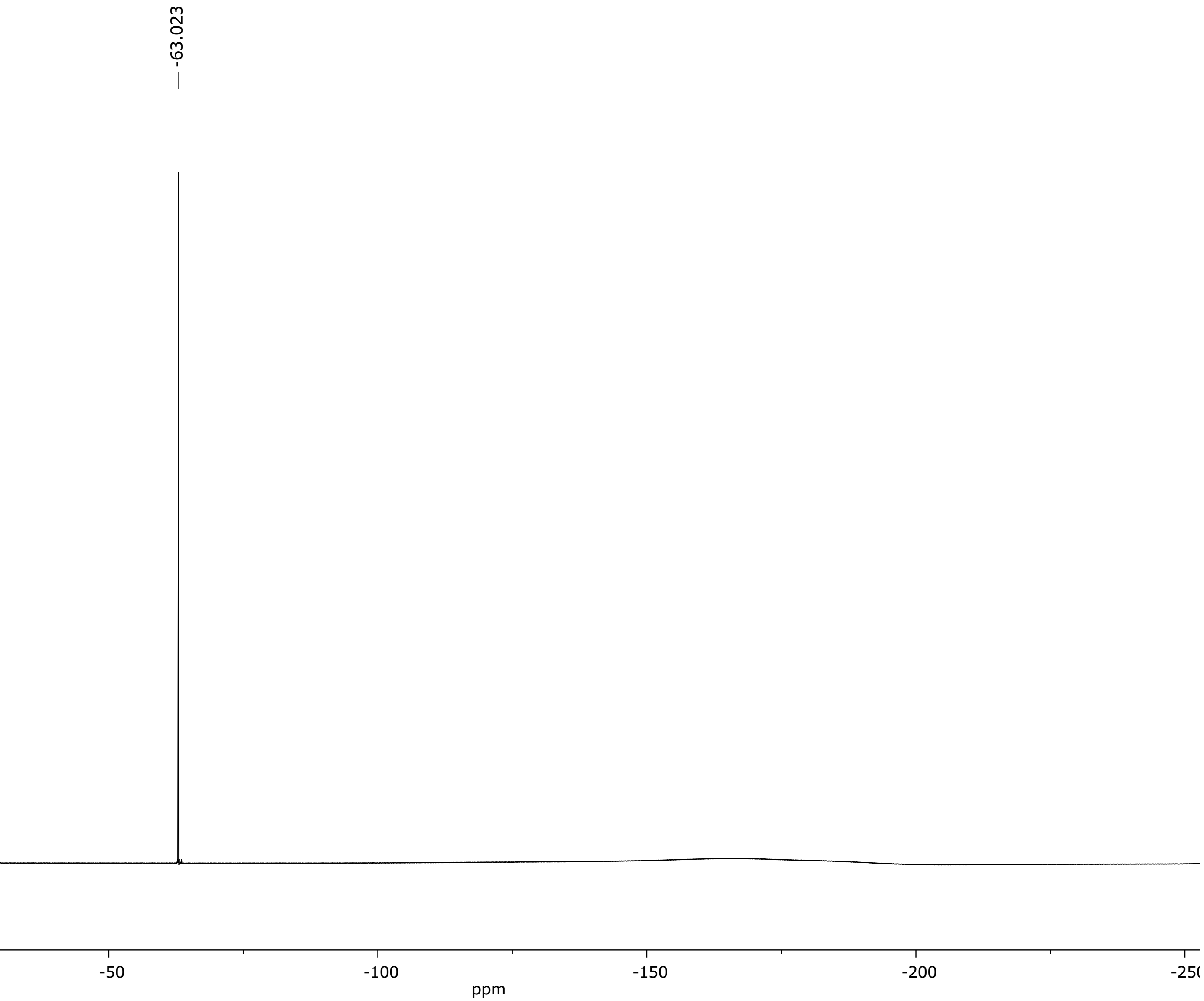

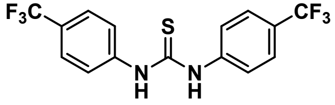

S17

^{1}H, $CDCl_3$, 400 MHz

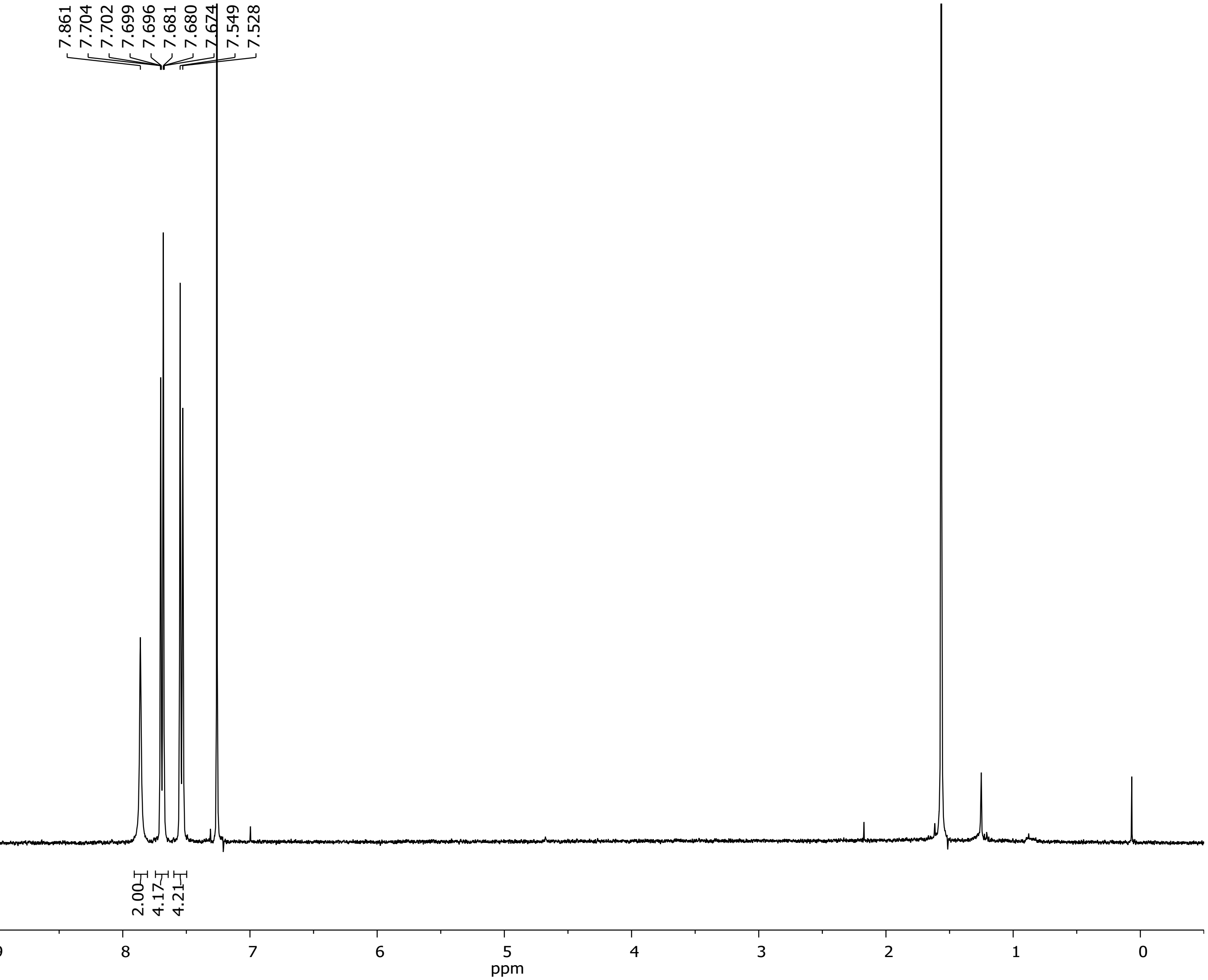

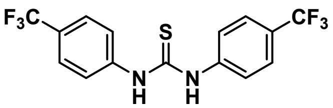

S17

$^{19}F\{^1H\}$, $CDCl_3$, 377 MHz

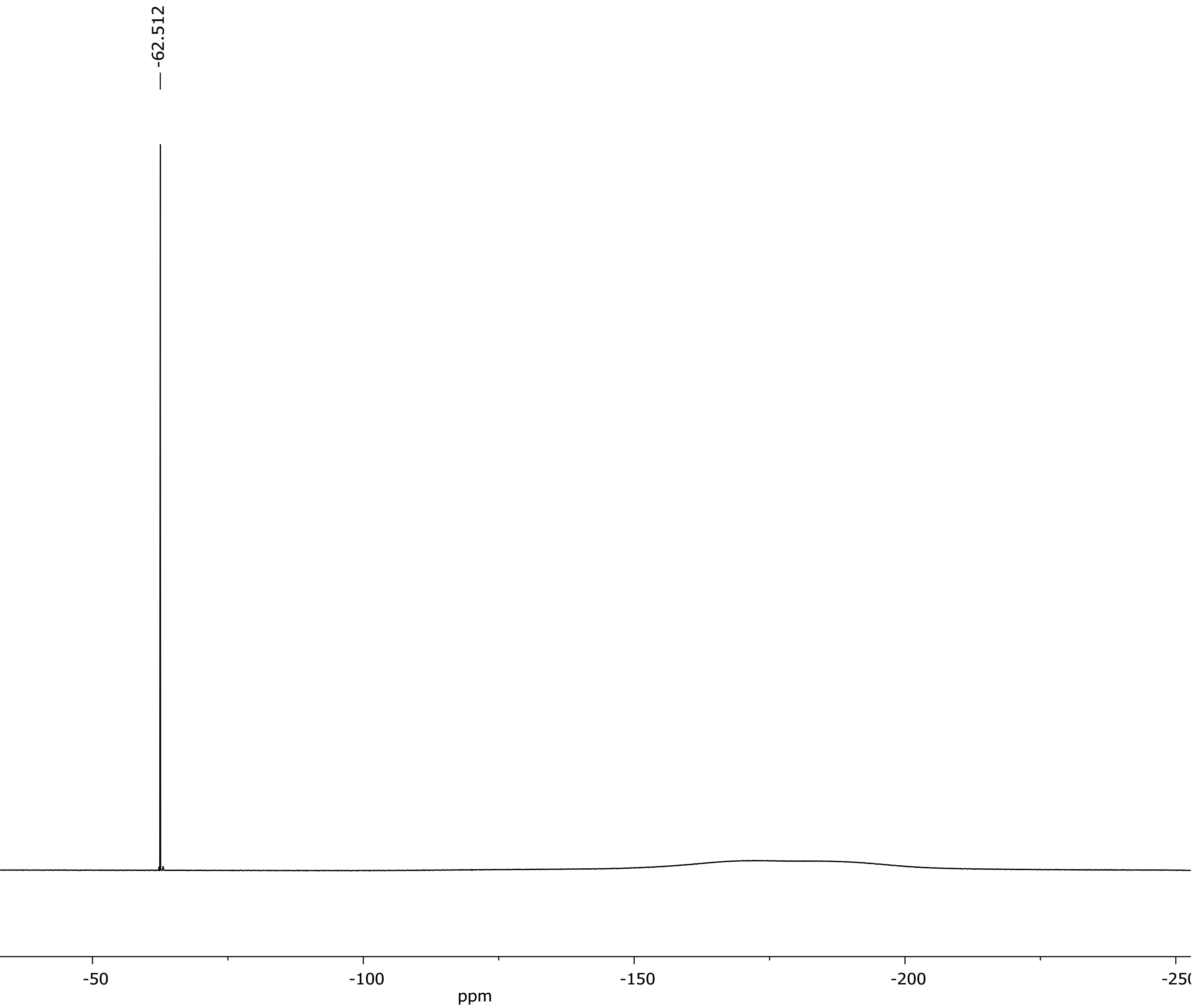

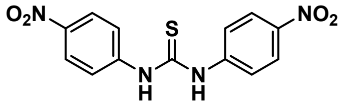

S18

^{1}H, $(CD_3)_2SO$, 400 MHz

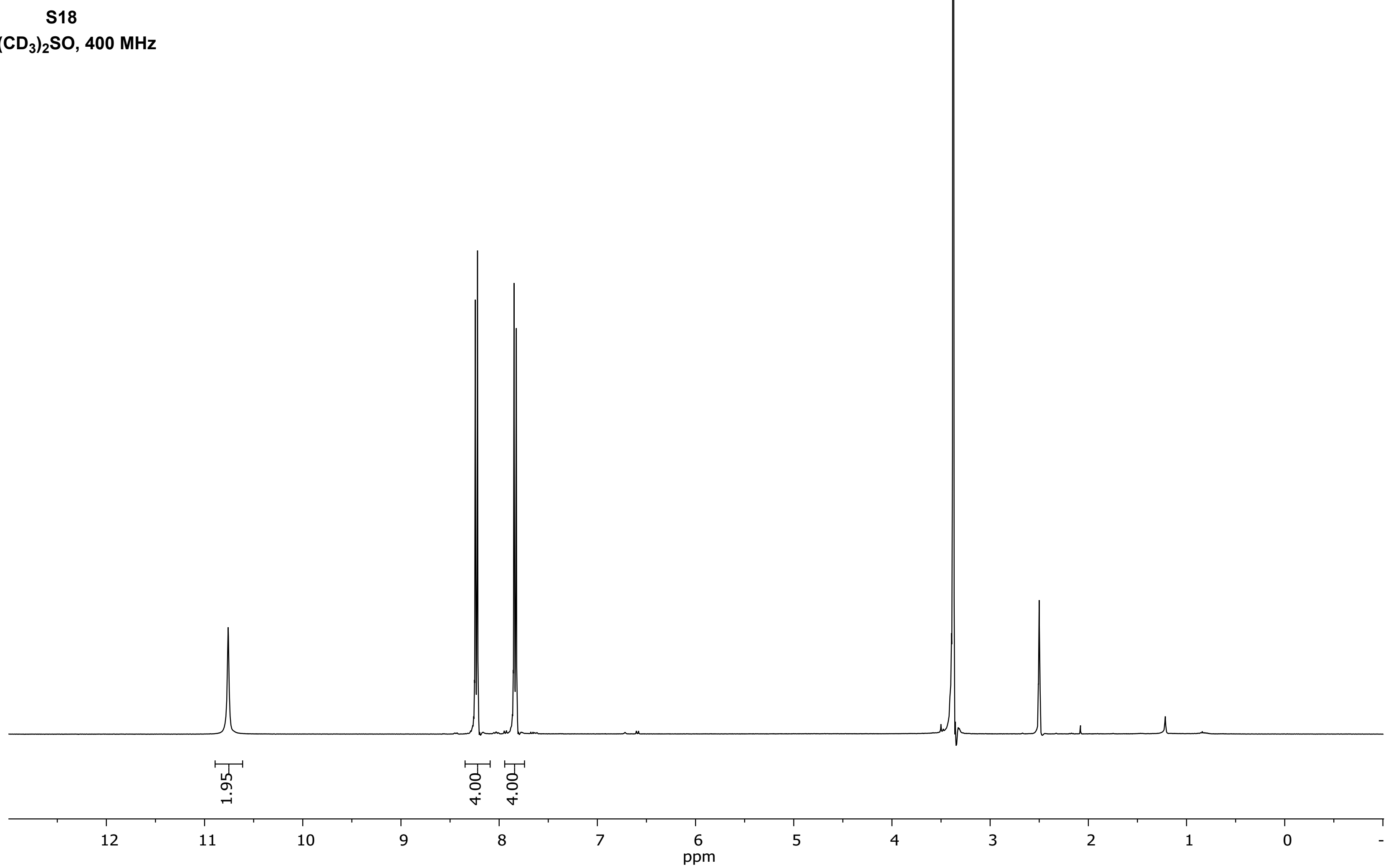

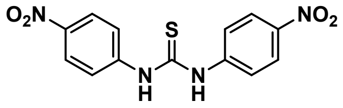

S18

$^{13}C\{^{1}H\}$, $(CD_3)_2SO$, 126 MHz

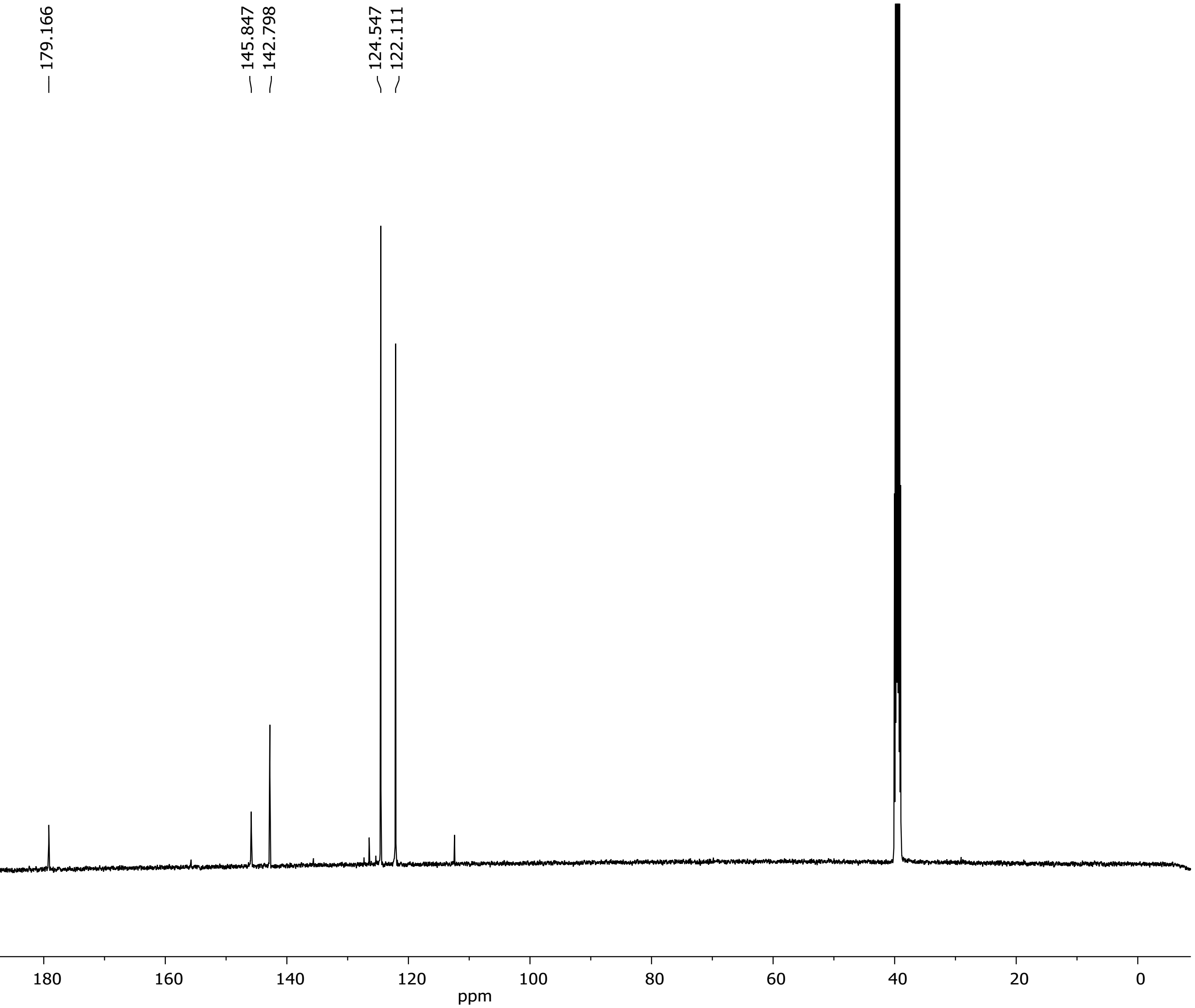

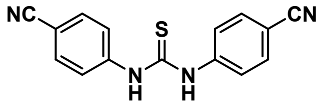

S19

^{1}H, $(CD_3)_2SO$, 400 MHz

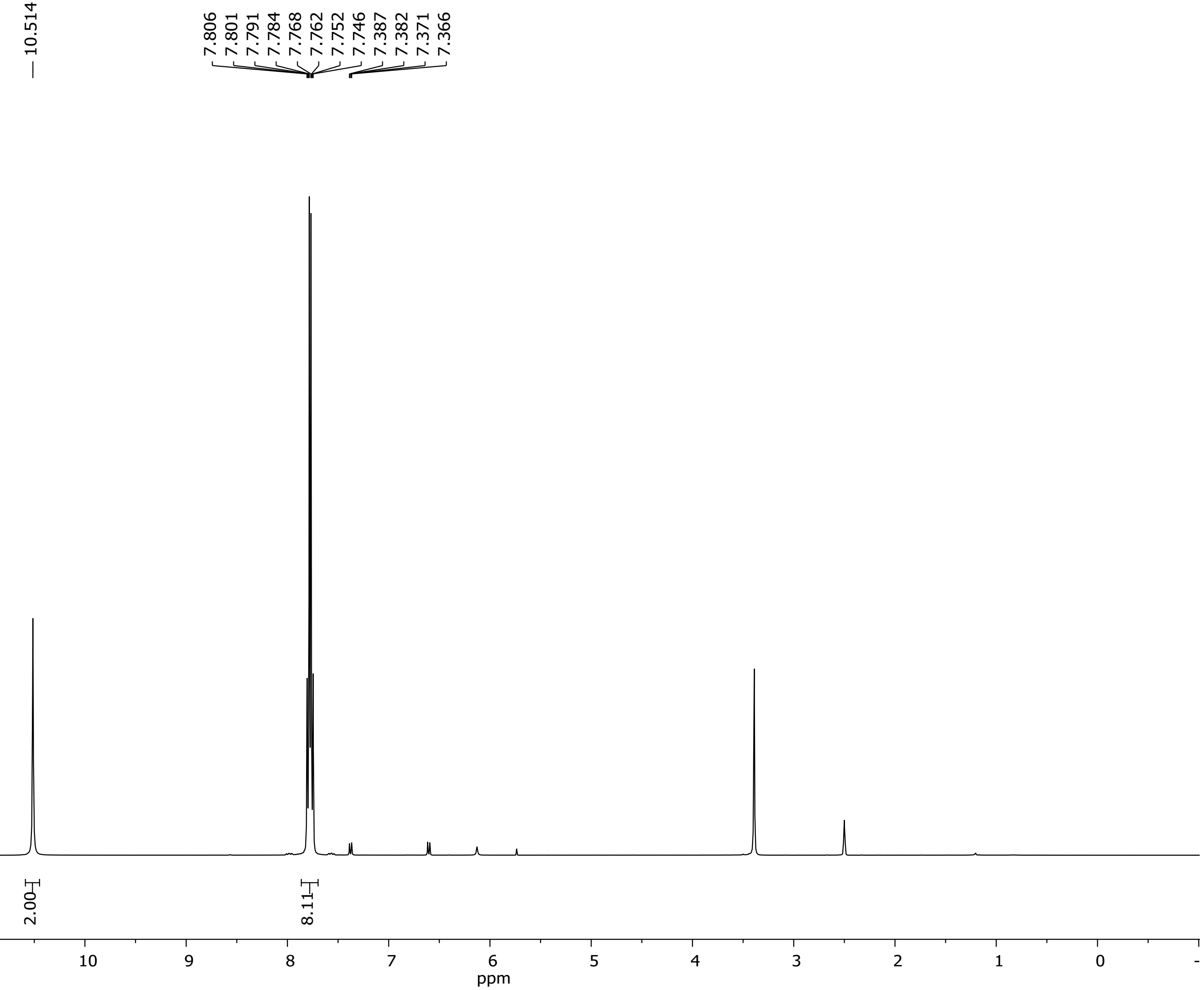

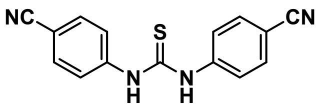

S19

$^{13}C\{^1H\}$, $(CD_3)_2SO$, 126 MHz

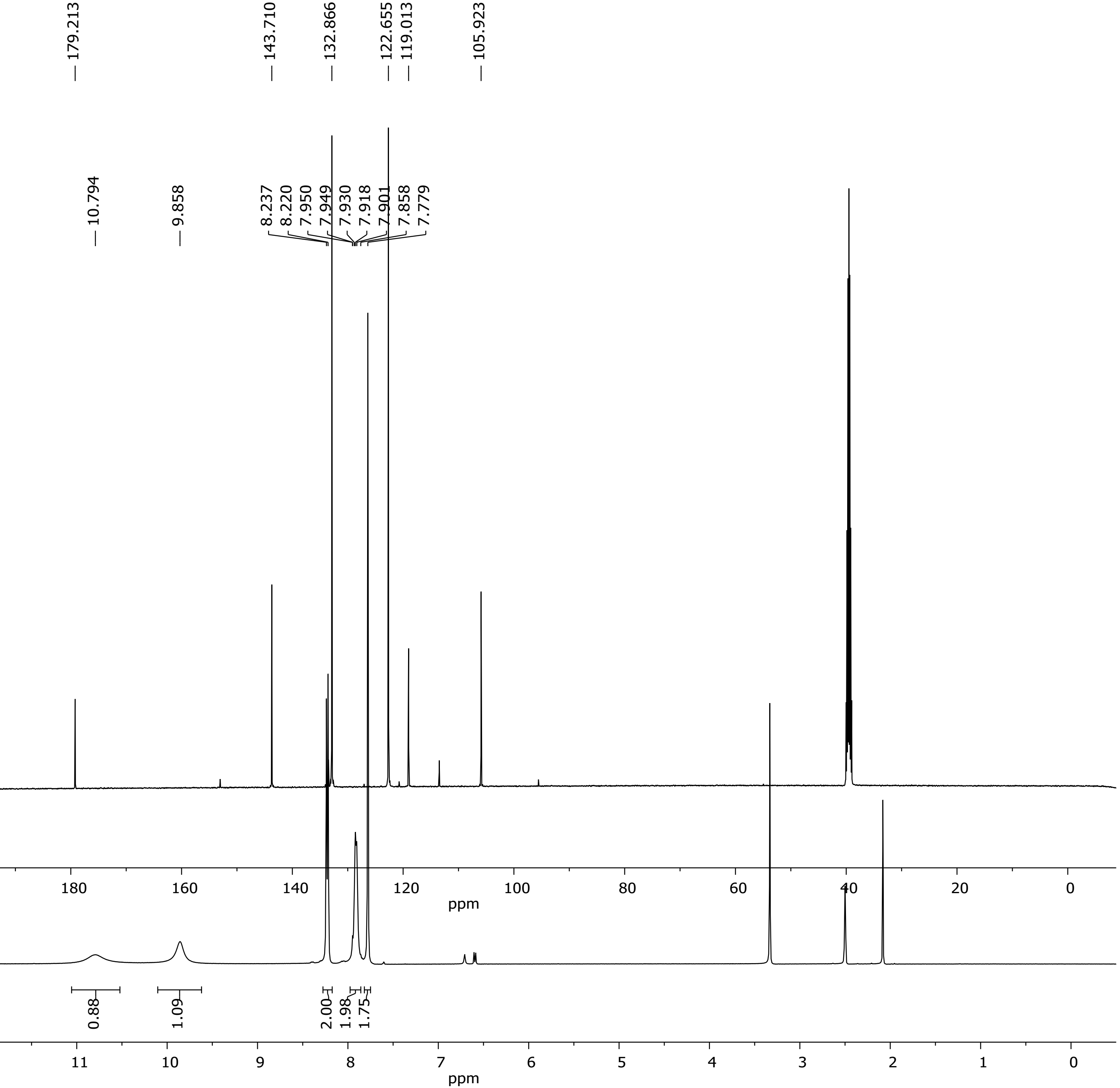

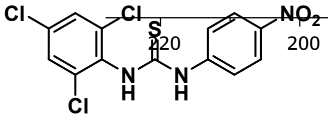

S20

^1H, $(CD_3)_2SO$, 500 MHz

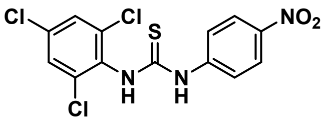

S20

$^{13}C\{^{1}H\}$, $(CD_3)_2SO$, 126 MHz

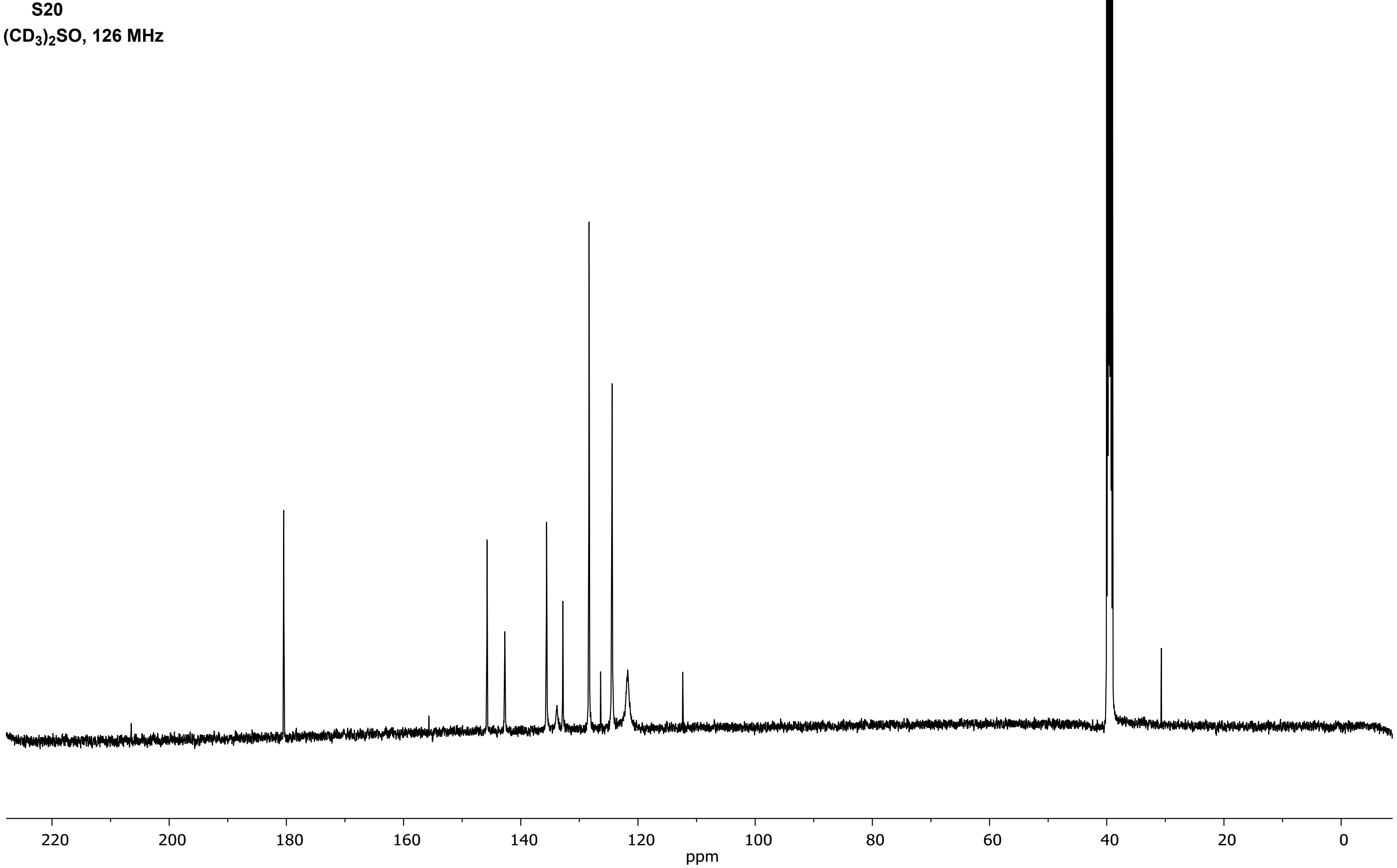

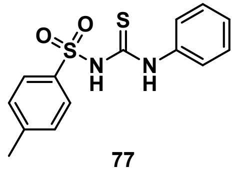

77

^{1}H, $(CD_3)_2SO$, 400 MHz

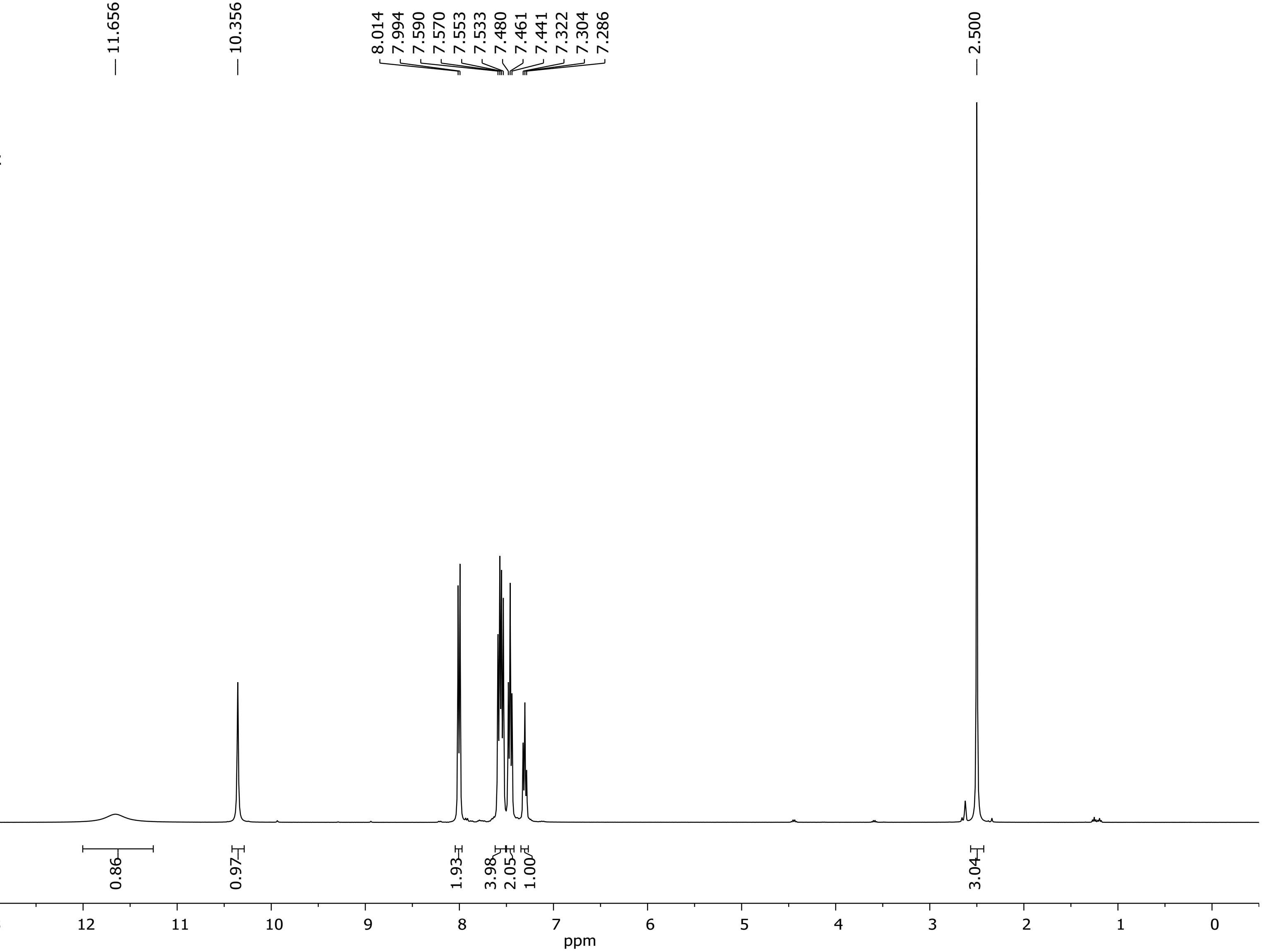

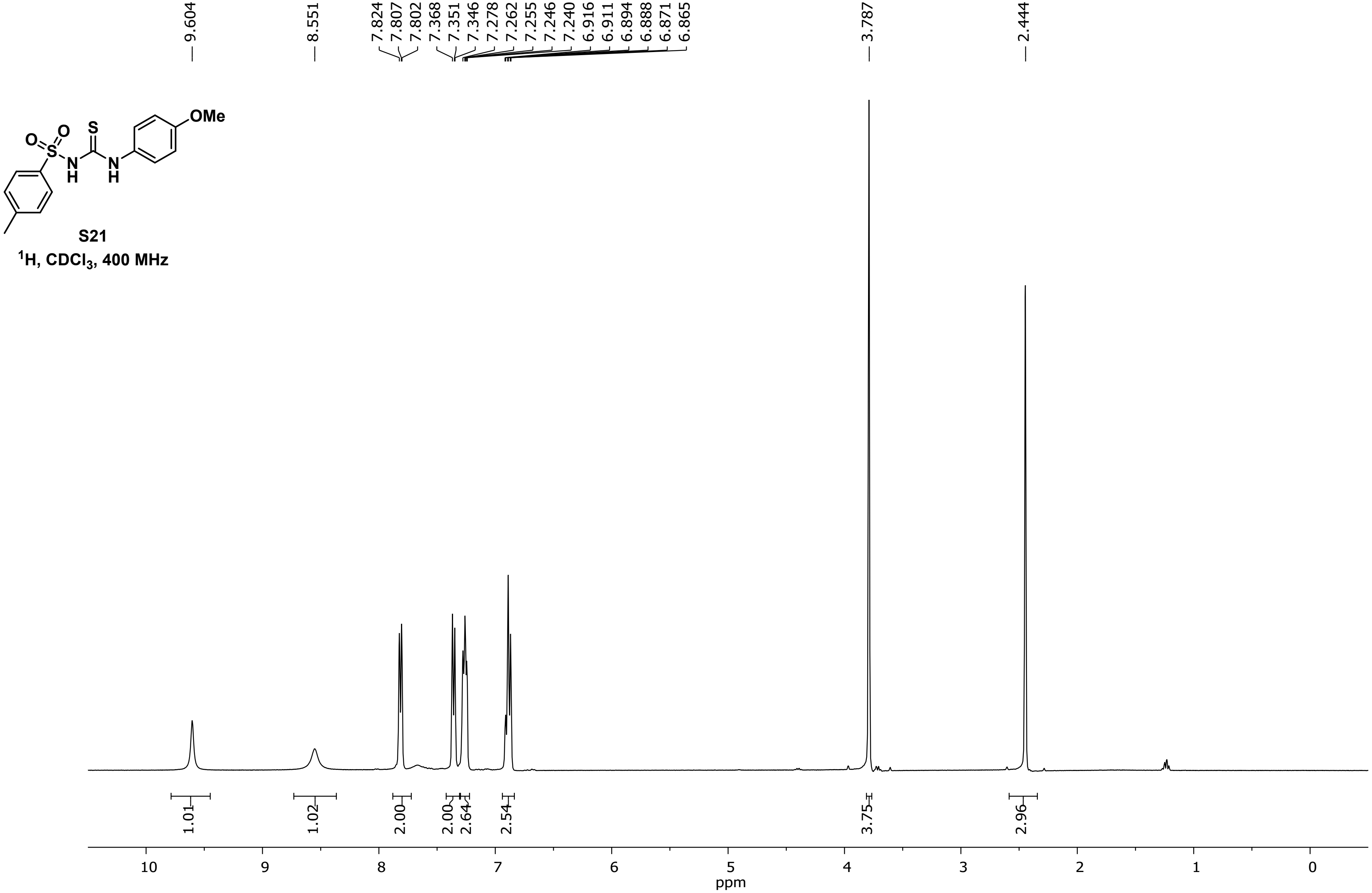
OMe
O
S
O
S
N
H
N
H
S21
1H, CDCl3, 400 MHz
9.604
8.551
7.824
7.807
7.802
7.368
7.351
7.346
7.278
7.262
7.255
7.246
7.240
6.916
6.911
6.894
6.888
6.871
6.865
3.787
2.444
1.01
1.02
2.00
2.00
2.64
2.54
3.75
2.96
10
9
8
7
6
5
4
3
2
1
0
ppm

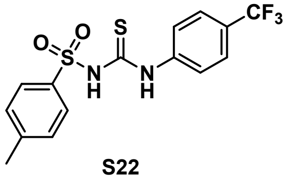

S22
^{1}H, $CDCl_3$, 400 MHz

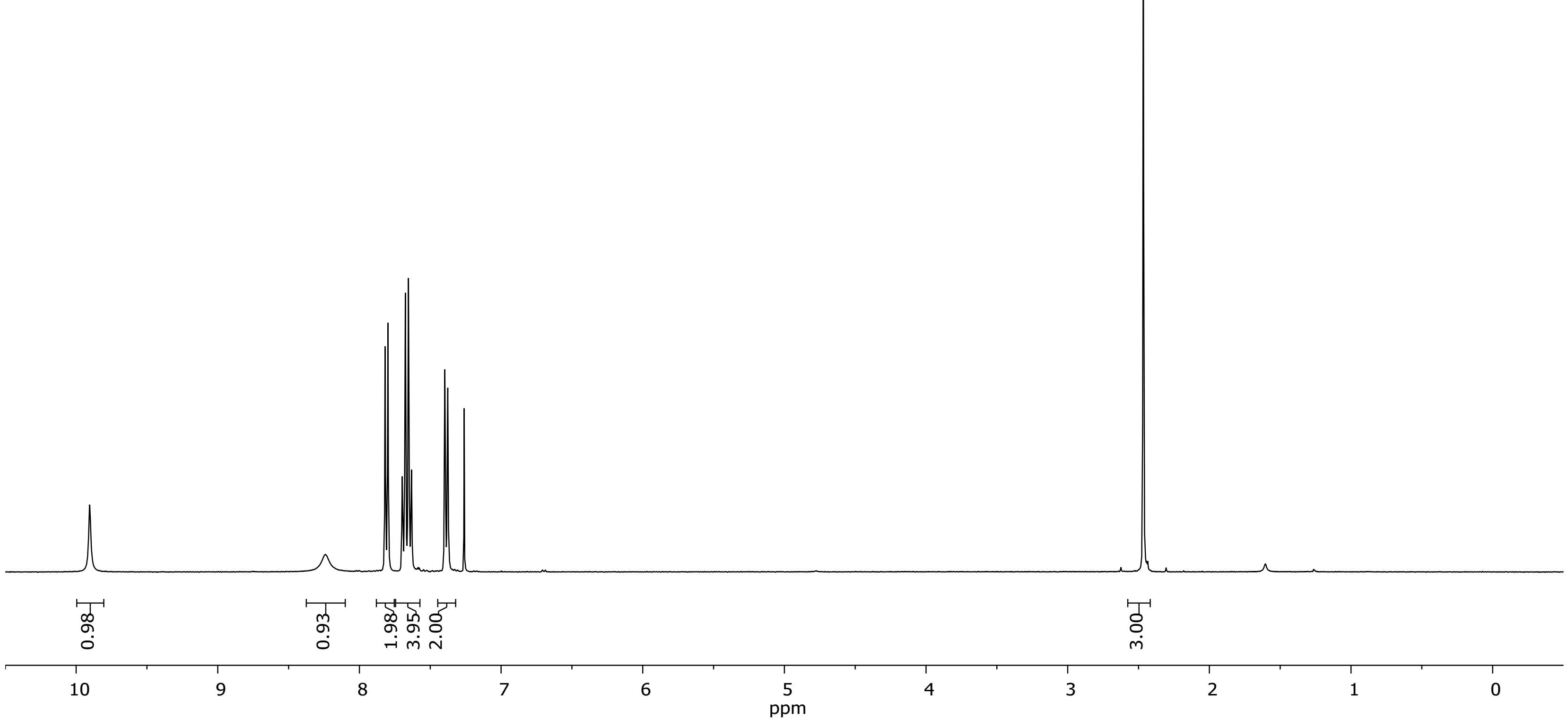

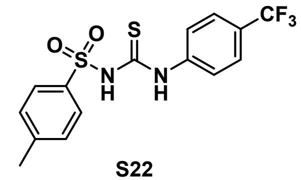

S22

$^{19}F\{^{1}H\}$, $CDCl_3$, 377 MHz

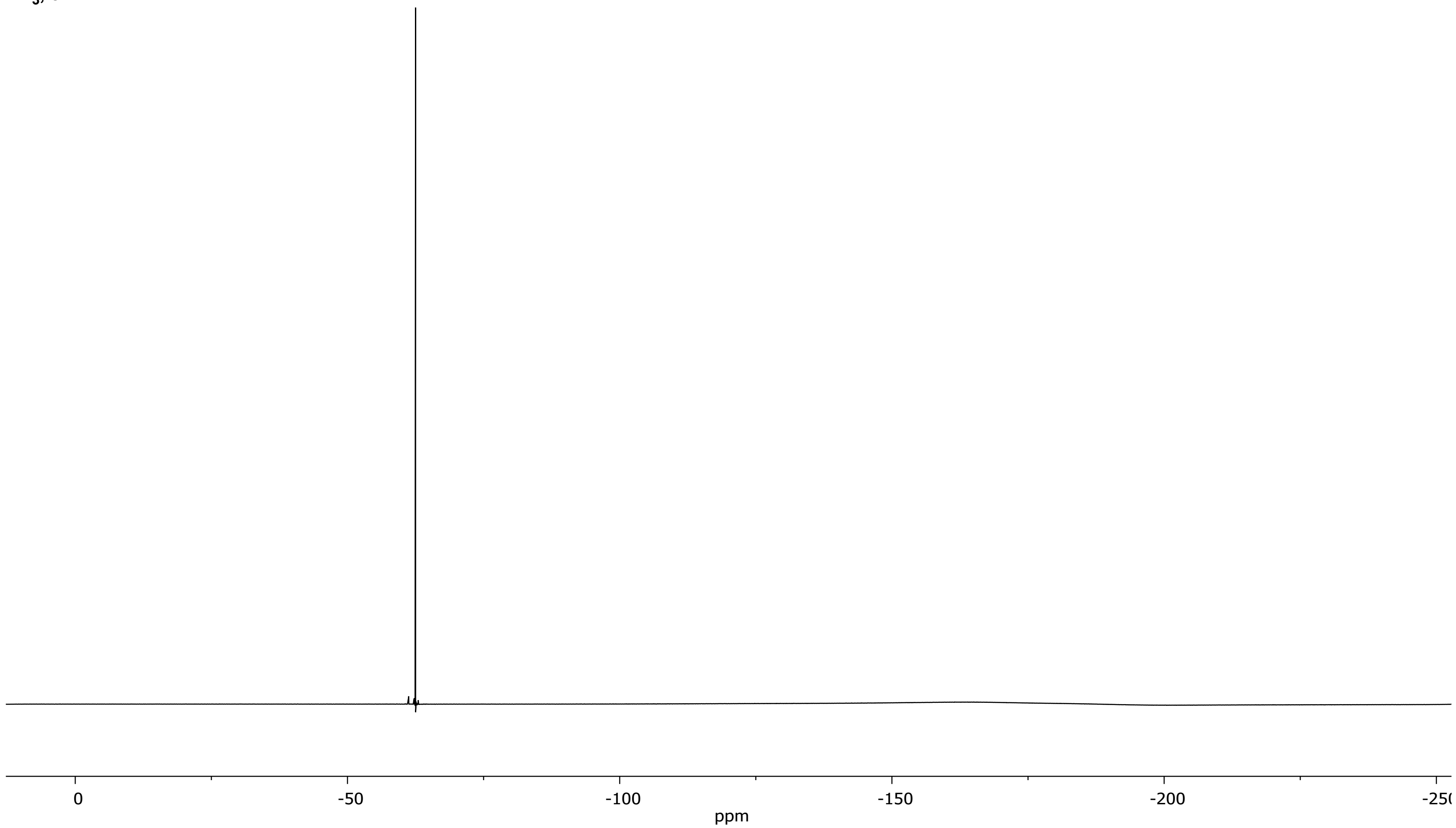

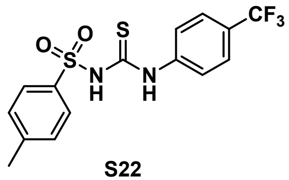

S22

$^{13}C\{^1H\}$, $CDCl_3$, 126 MHz

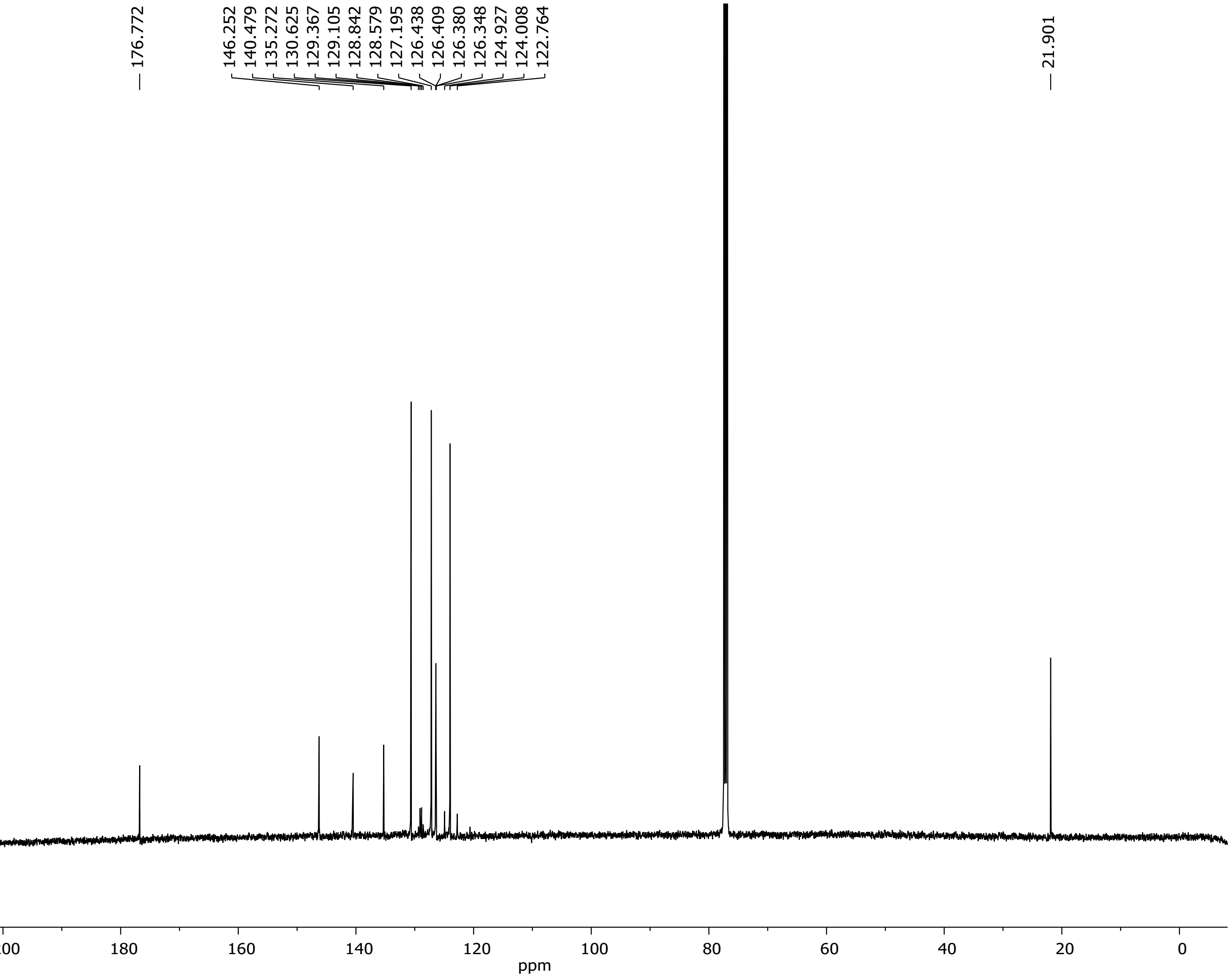

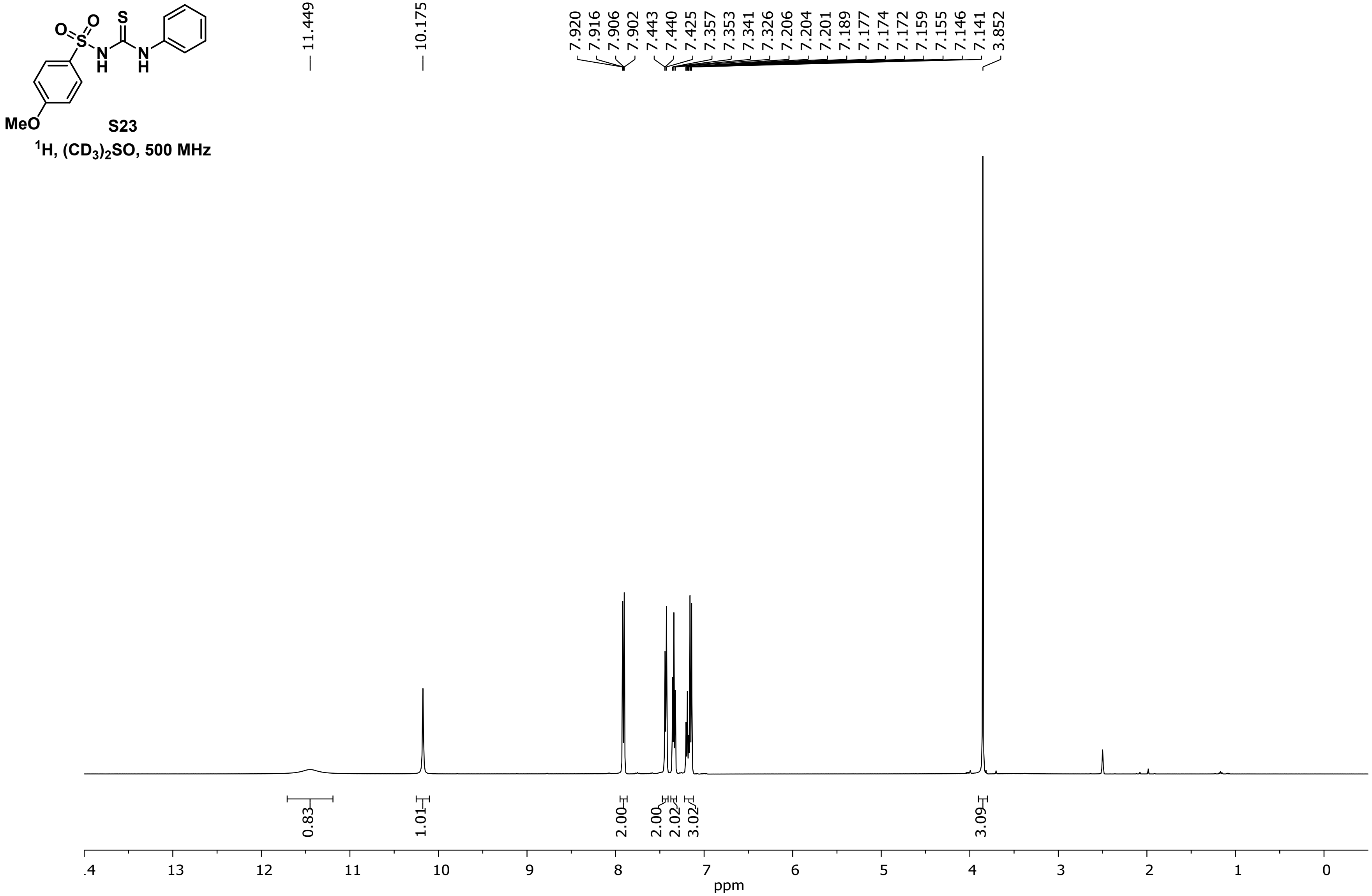

MeO
S23
1H, (CD3)2SO, 500 MHz
11.449
10.175
7.920
7.916
7.906
7.902
7.443
7.440
7.425
7.357
7.353
7.341
7.326
7.206
7.204
7.201
7.189
7.177
7.174
7.172
7.159
7.155
7.146
7.141
3.852
0.83
1.01
2.00
2.00
2.02
3.02
3.09
ppm

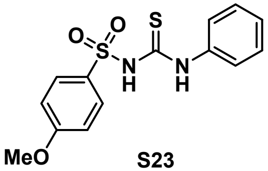

S23

$^{13}C\{^1H\}$, $(CD_3)_2SO$, 126 MHz

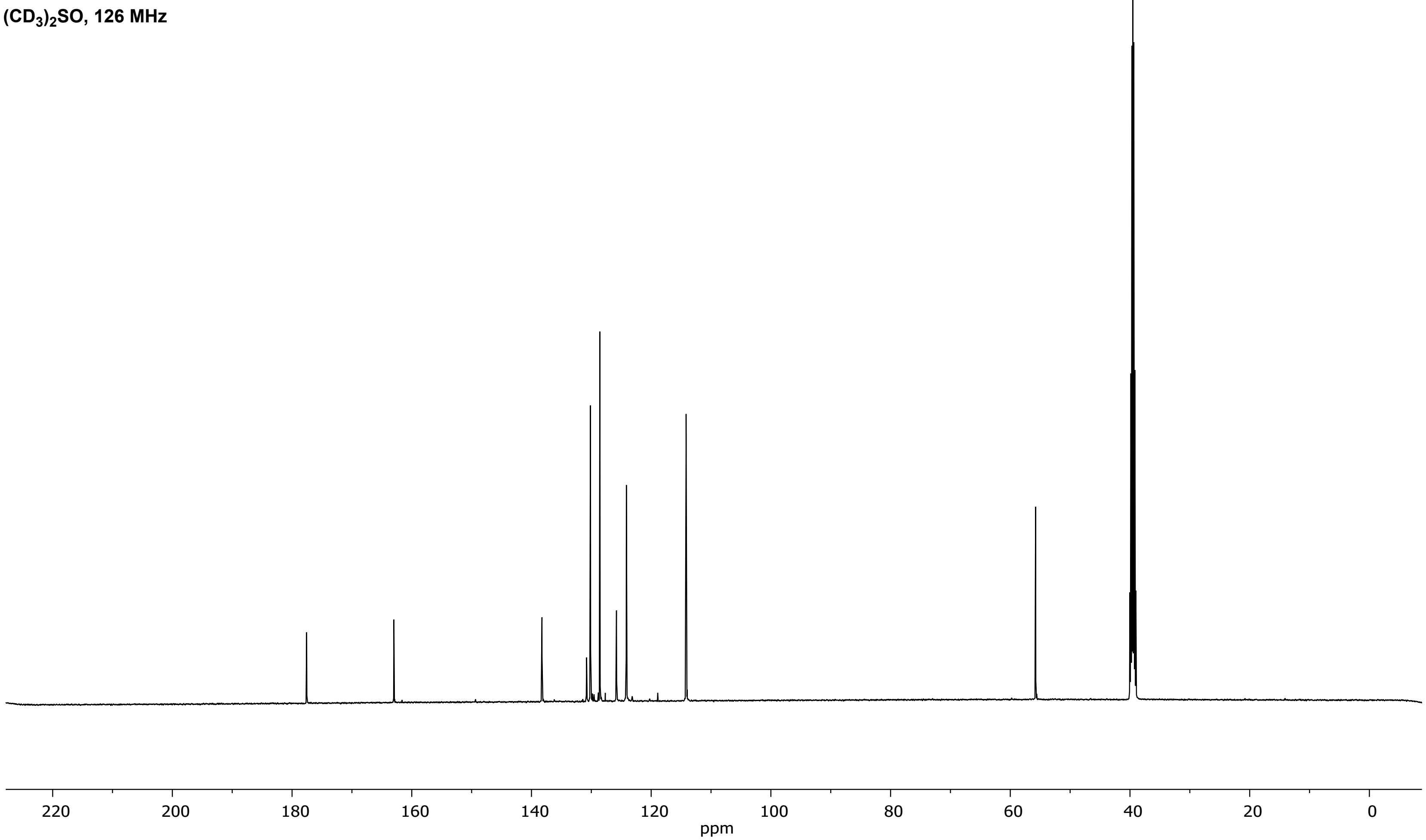

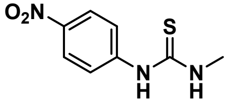

S24

^{1}H, $(CD_3)_2SO$, 400 MHz

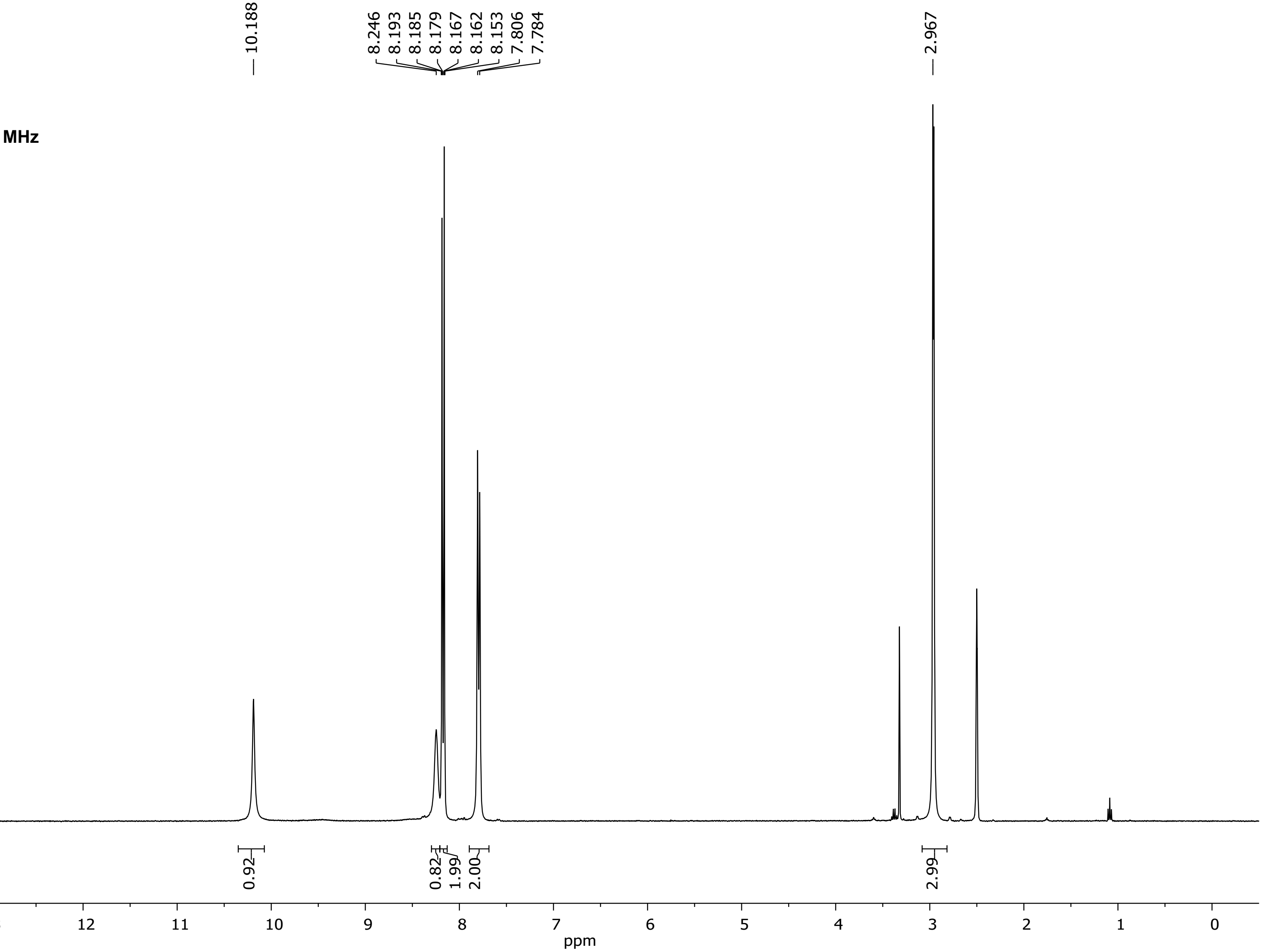

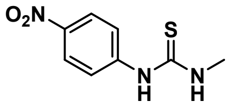

S24

$^{13}C\{^{1}H\}$, $(CD_3)_2SO$, 126 MHz

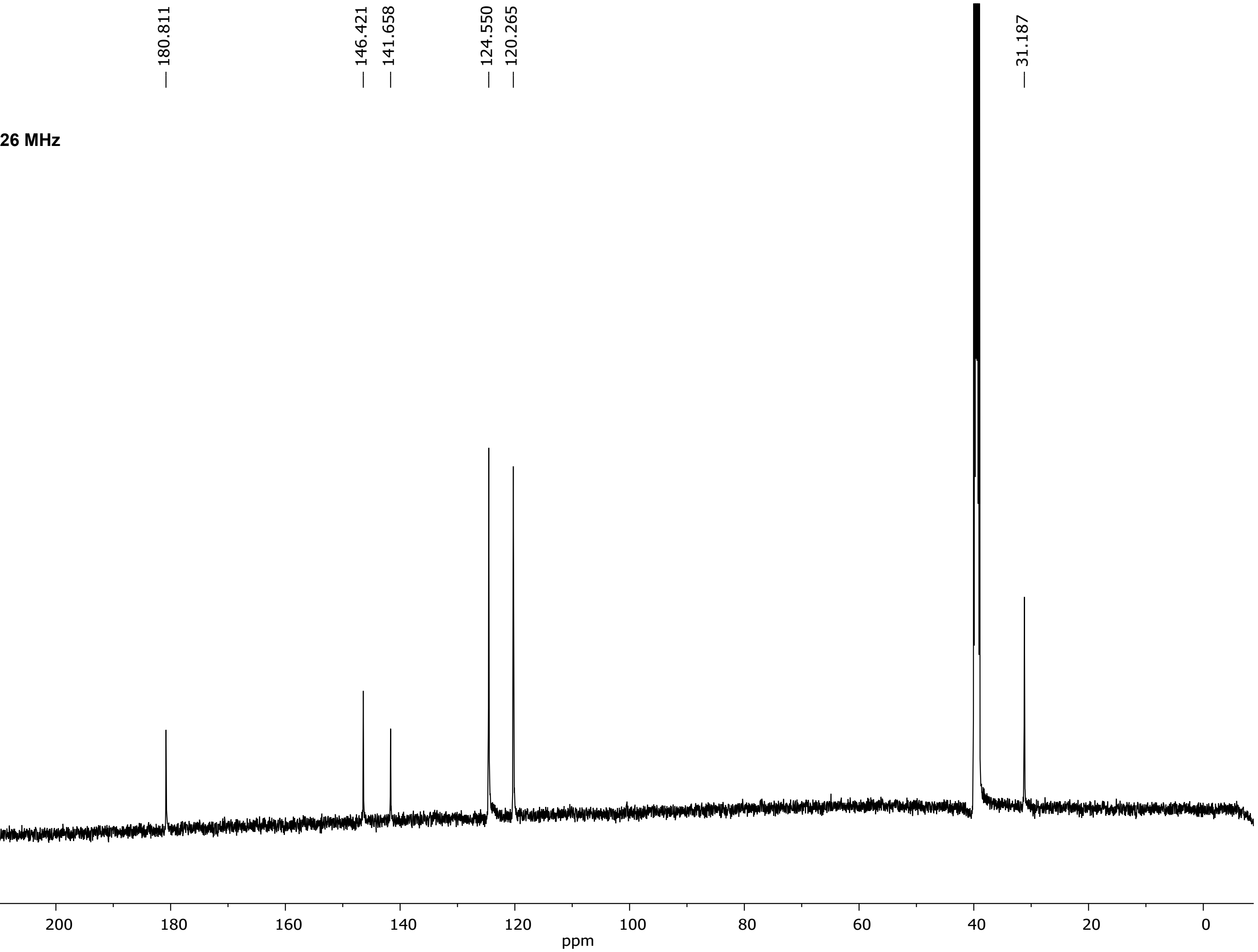

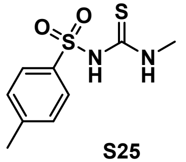

S25

^1H, $(CD_3)_2SO$, 400 MHz

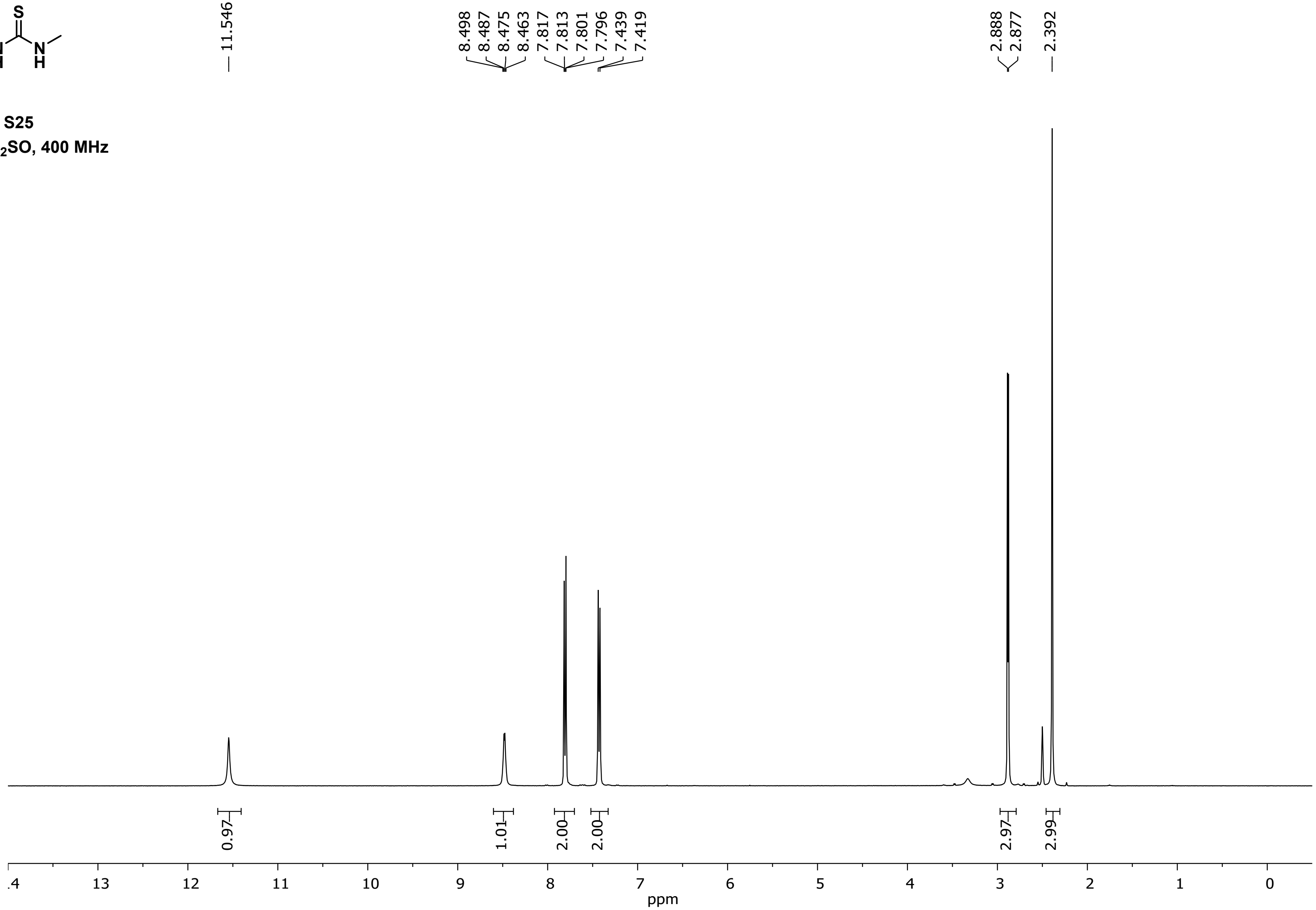

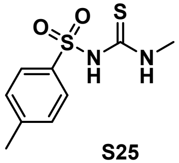

S25

$^{13}C\{^{1}H\}$, $(CD_3)_2SO$, 126 MHz

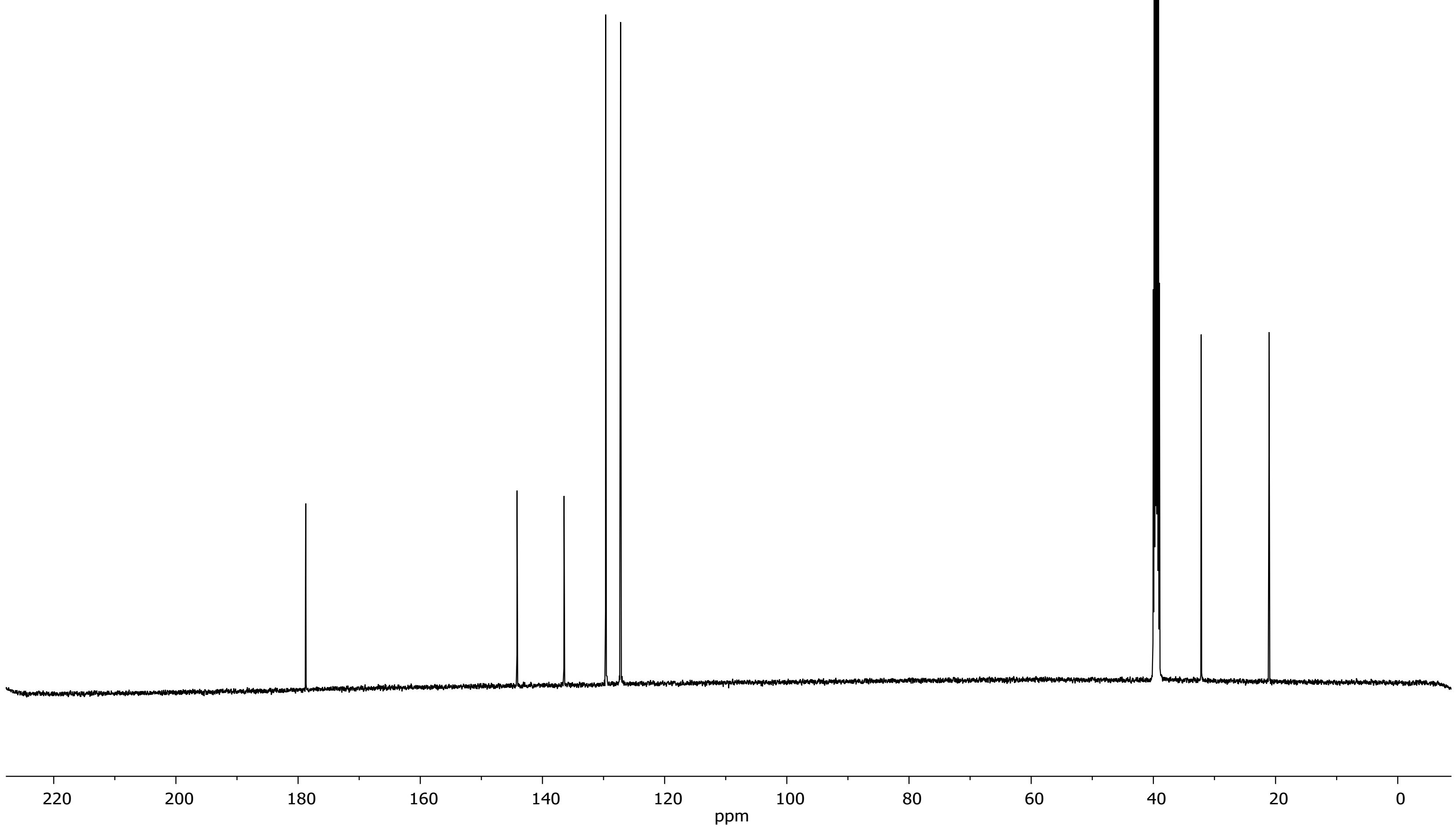

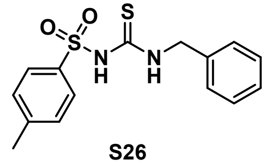

S26

^{1}H, $(CD_3)_2SO$, 400 MHz

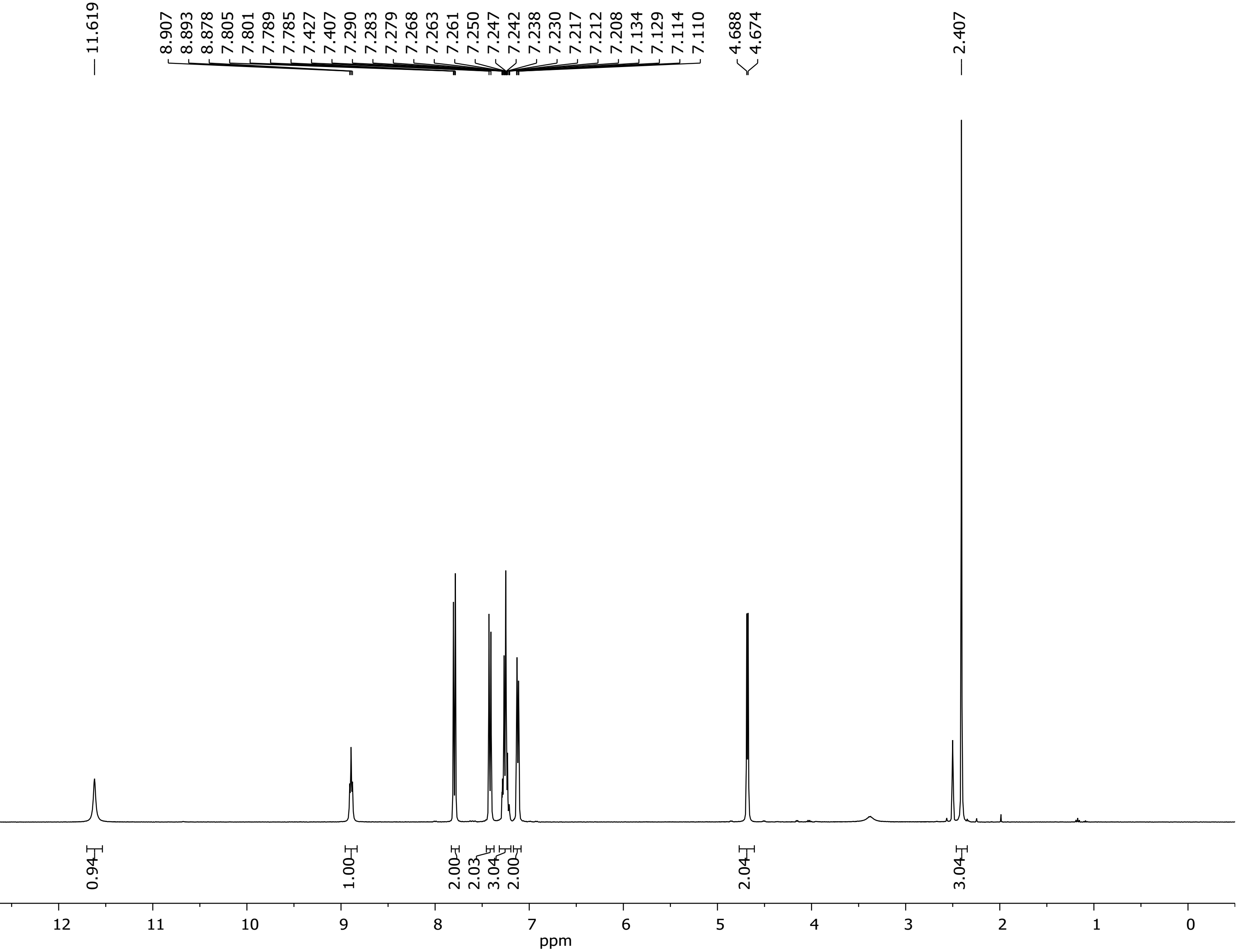

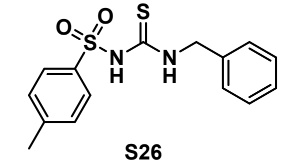

S26

$^{13}C\{^{1}H\}$, $(CD_3)_2SO$, 126 MHz

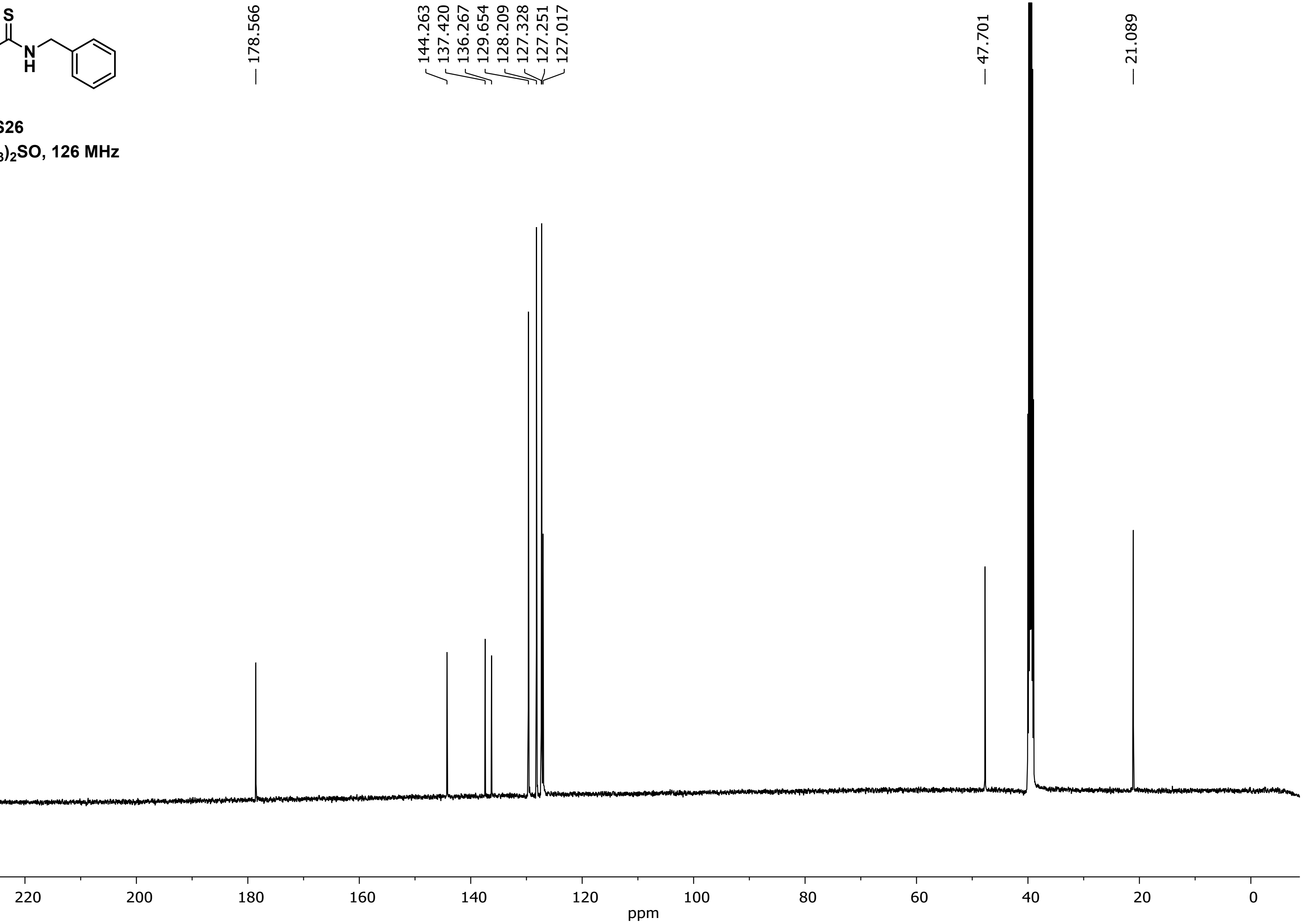

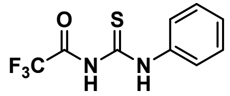

S27

^{1}H, $(CD_3)_2SO$, 400 MHz

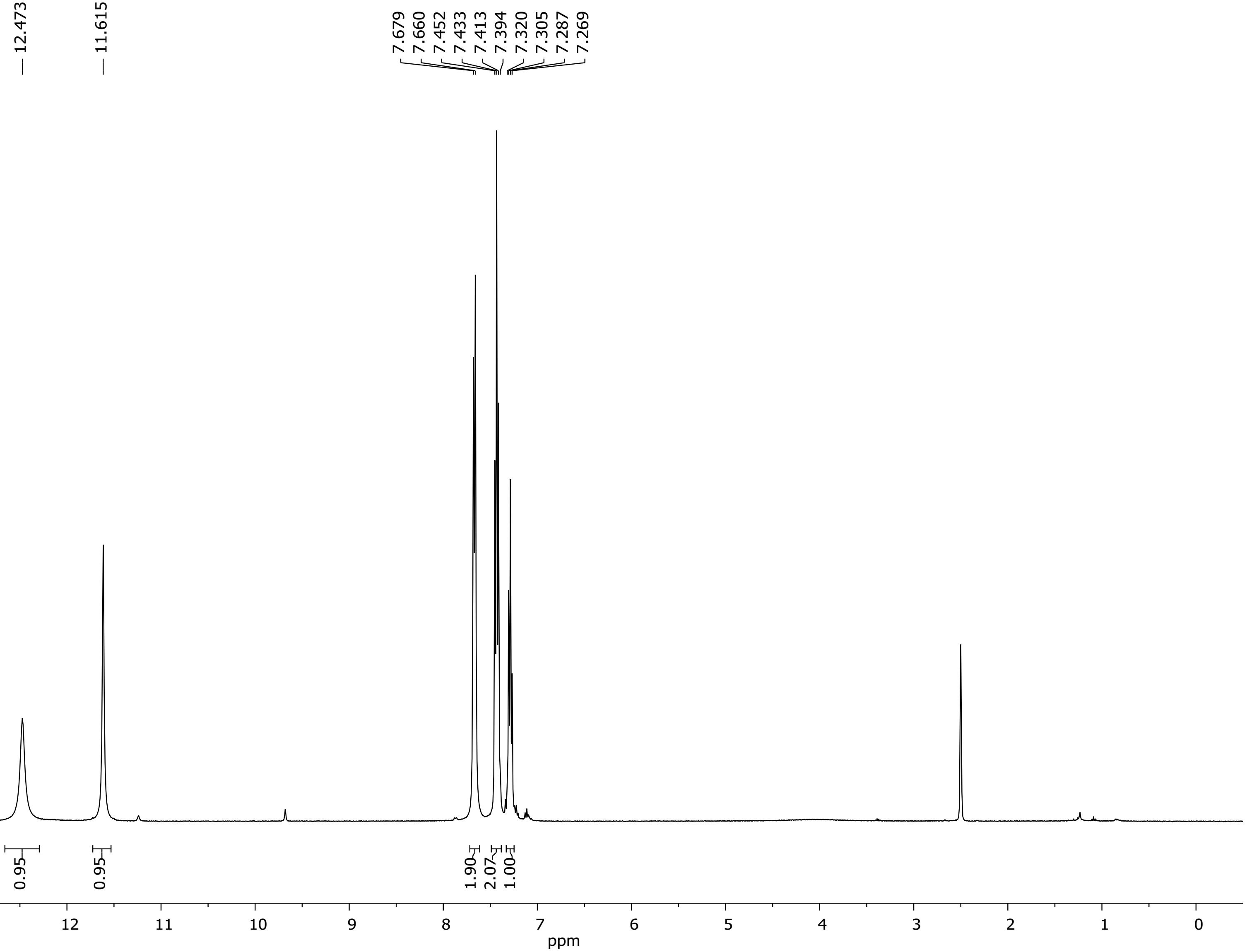

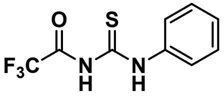

S27

$^{19}F\{^1H\}$, $(CD_3)_2SO$, 377 MHz

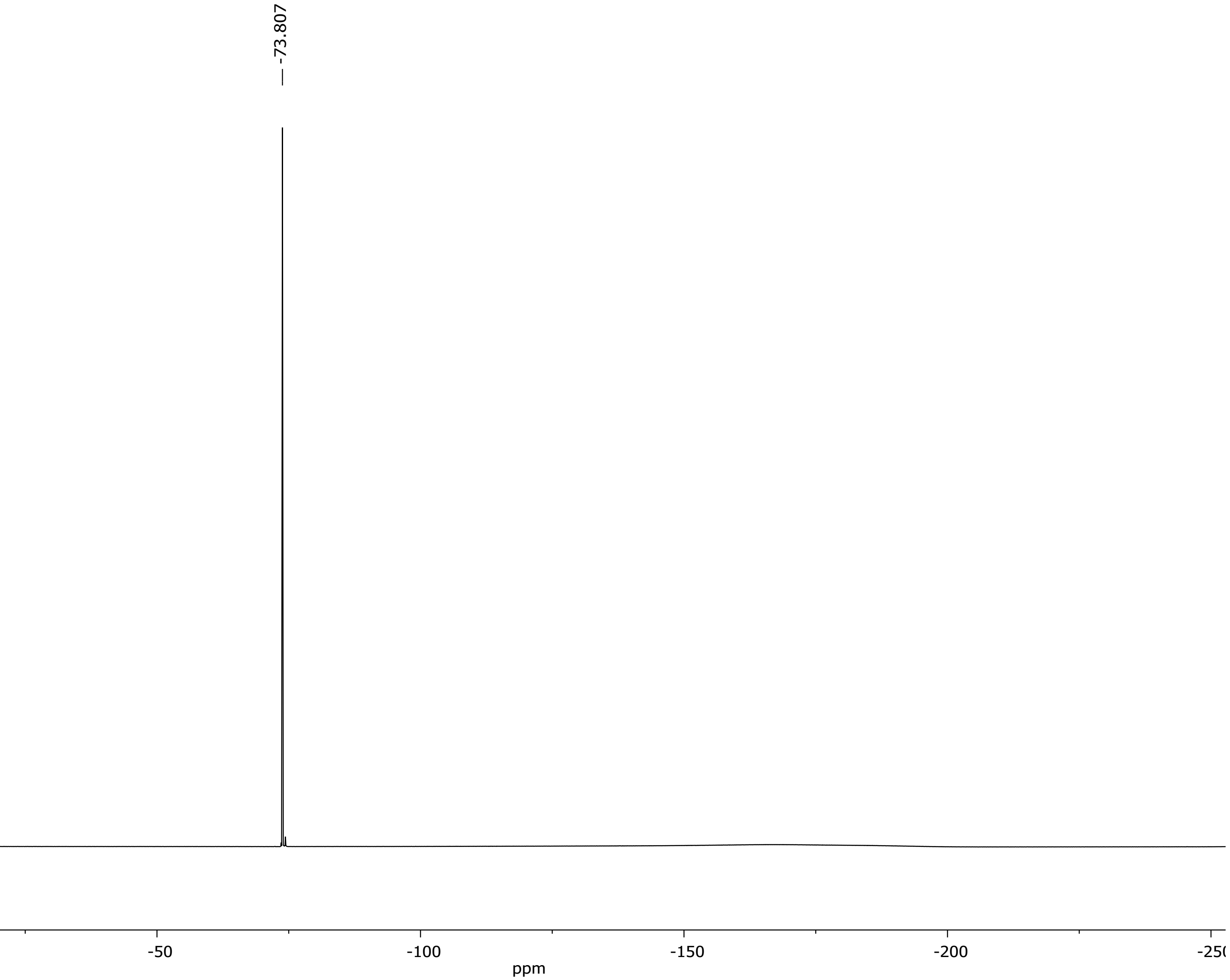

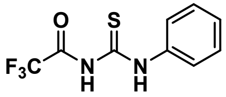

S27

$^{13}C\{^{1}H\}$, $(CD_3)_2SO$, 126 MHz

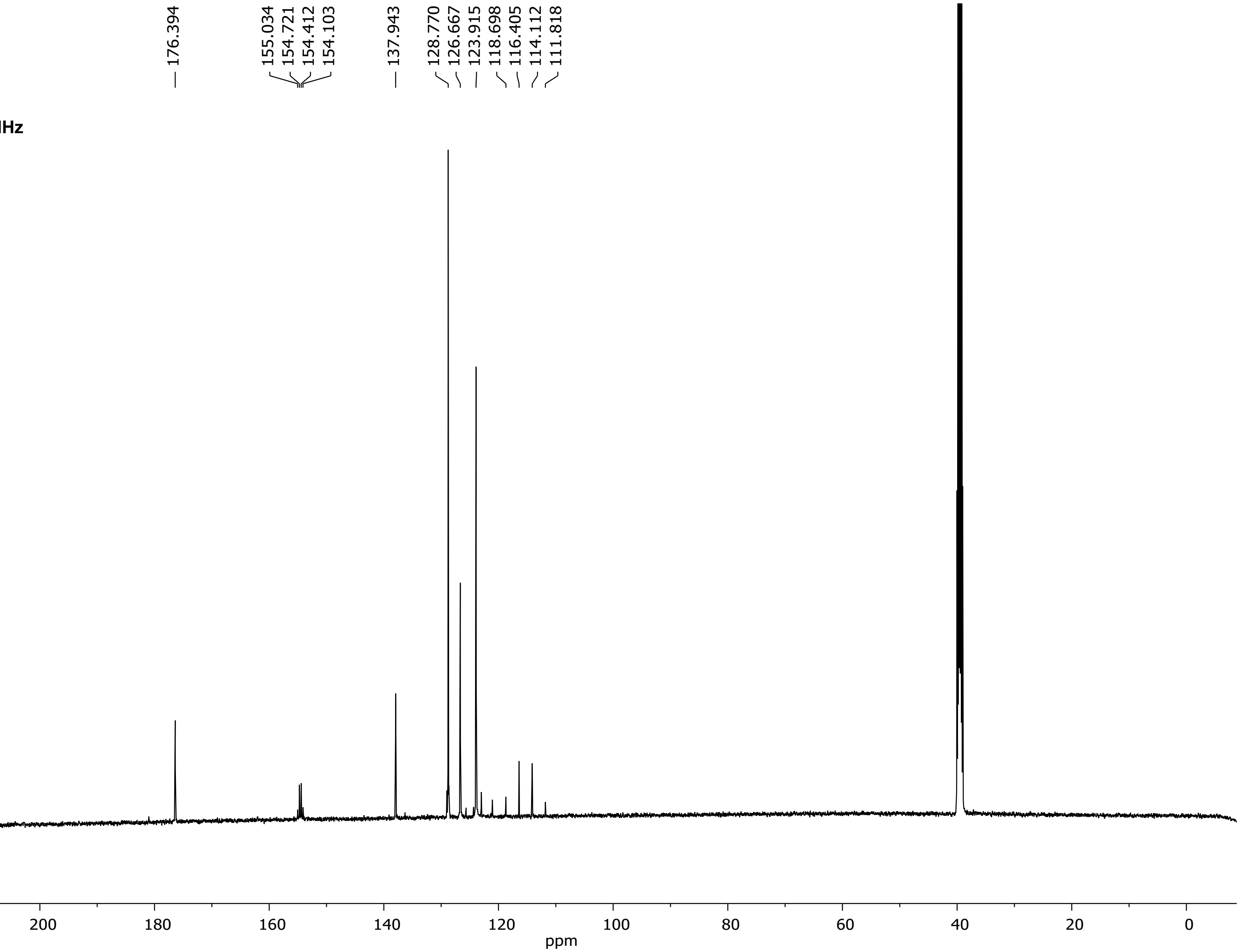

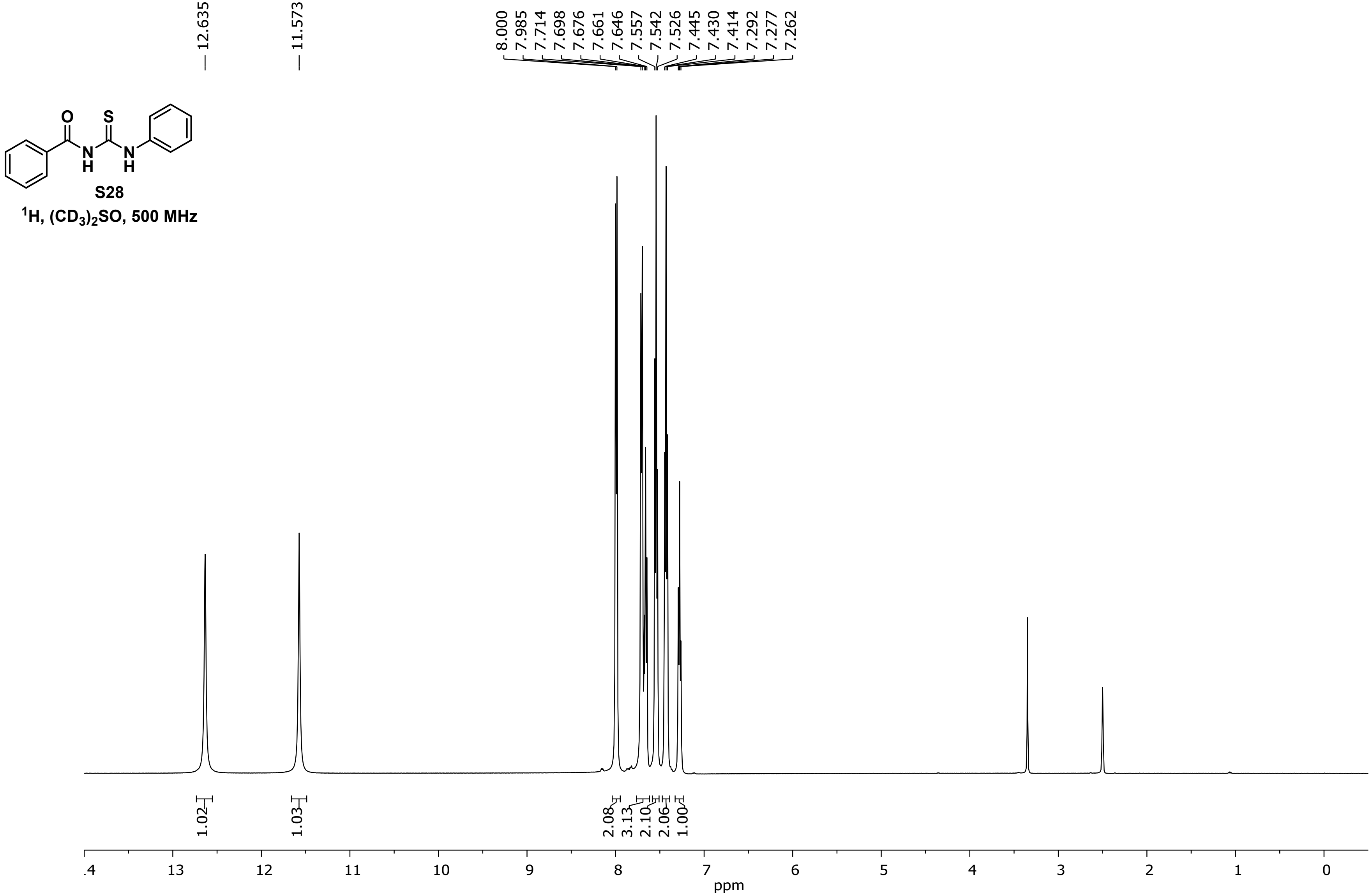

12.635
11.573
8.000
7.985
7.714
7.698
7.676
7.661
7.646
7.557
7.542
7.526
7.445
7.430
7.414
7.292
7.277
7.262
O
S
N
H
N
H
S28
^{1}H, $(CD_3)_2SO$, 500 MHz
1.02
1.03
2.08
3.13
2.10
2.06
1.00
ppm

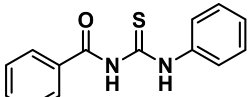

S28

$^{13}C\{^{1}H\}$, $(CD_3)_2SO$, 126 MHz

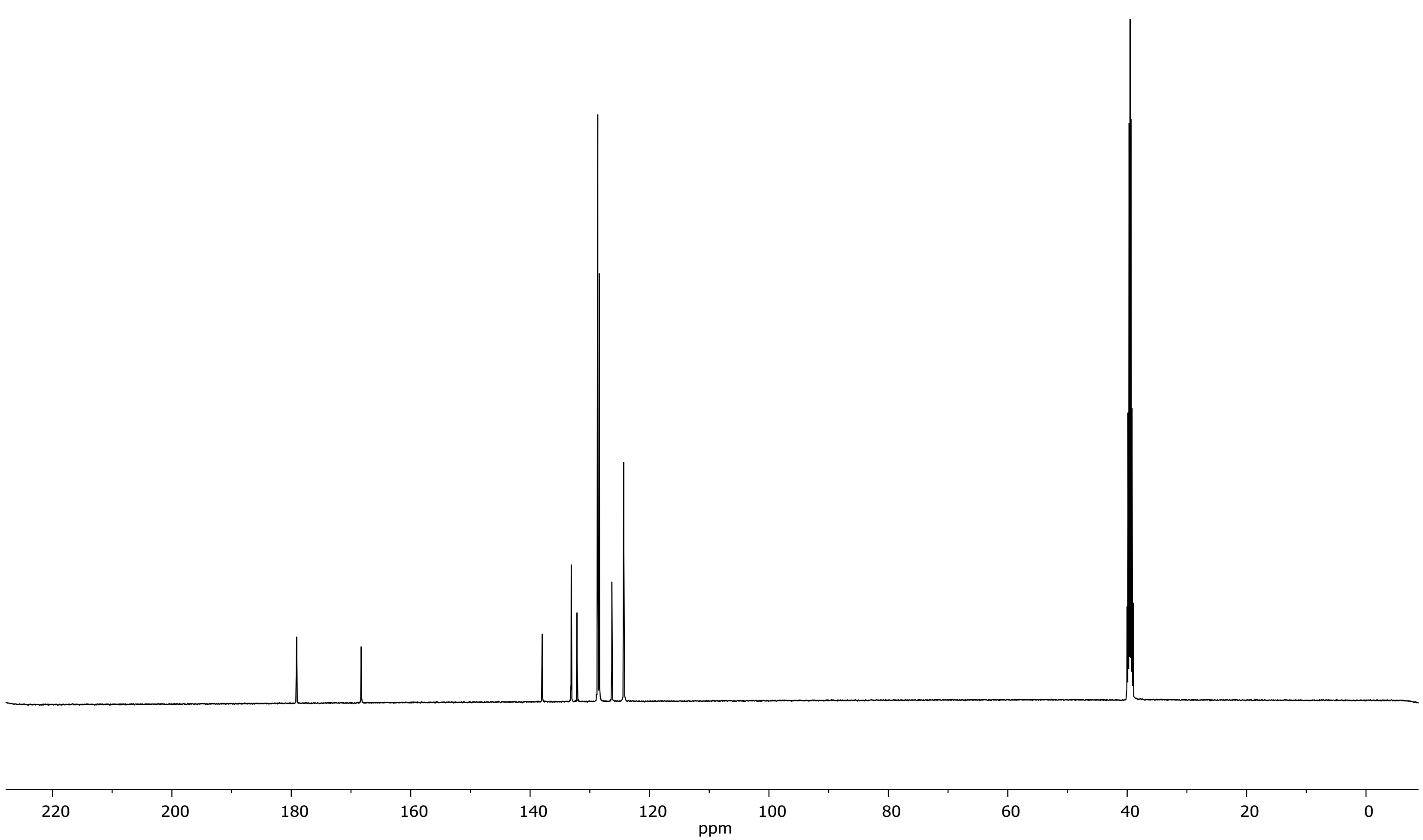

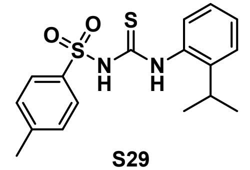

S29

^{1}H, $(CD_3)_2SO$, 400 MHz

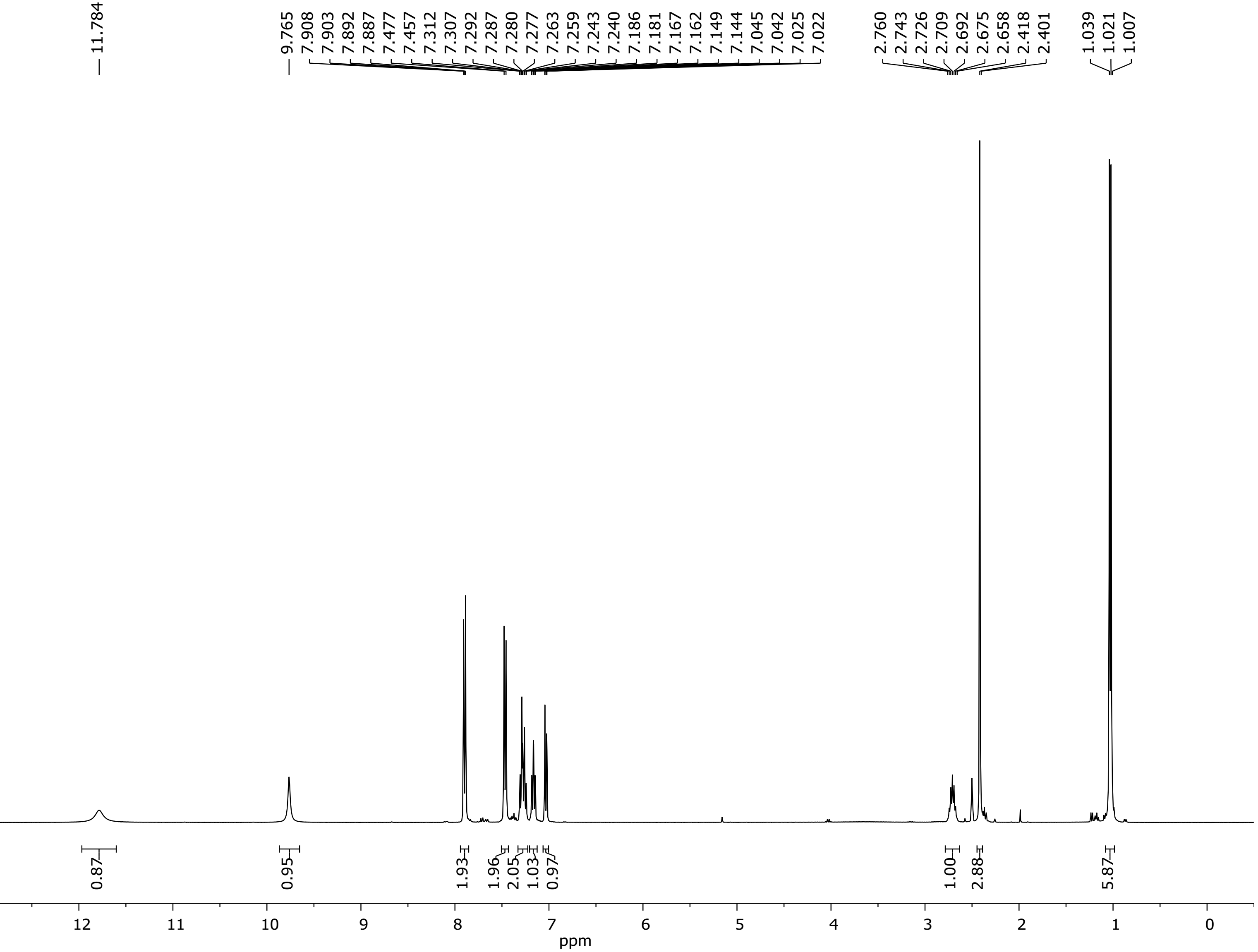

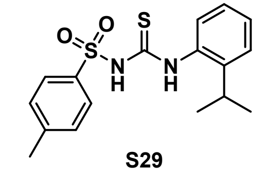

S29

$^{13}C\{^{1}H\}$, $(CD_3)_2SO$, 126 MHz

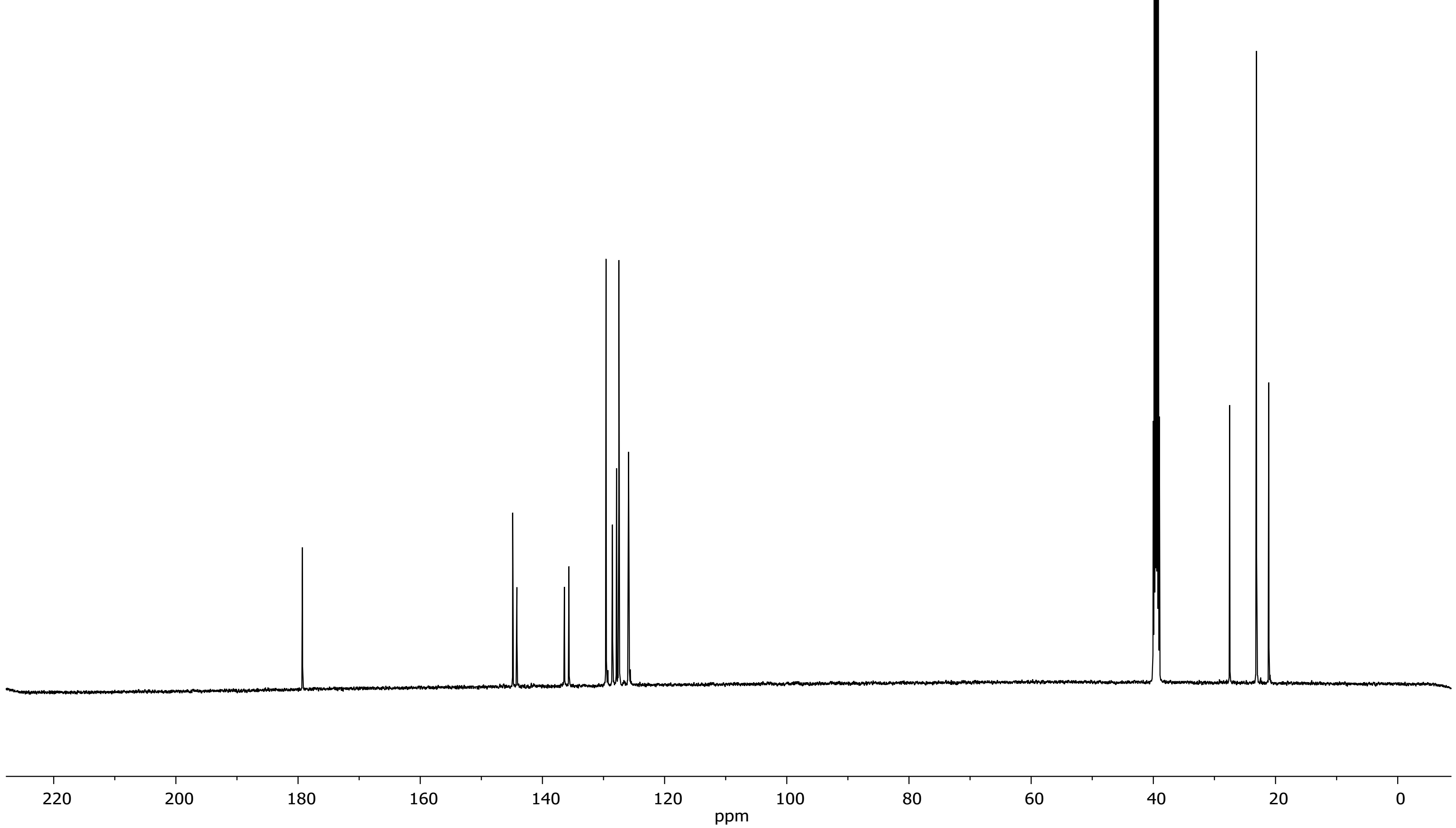

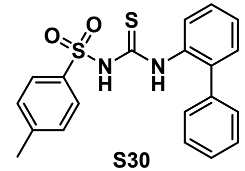

S30

^{1}H, $(CD_3)_2SO$, 500 MHz

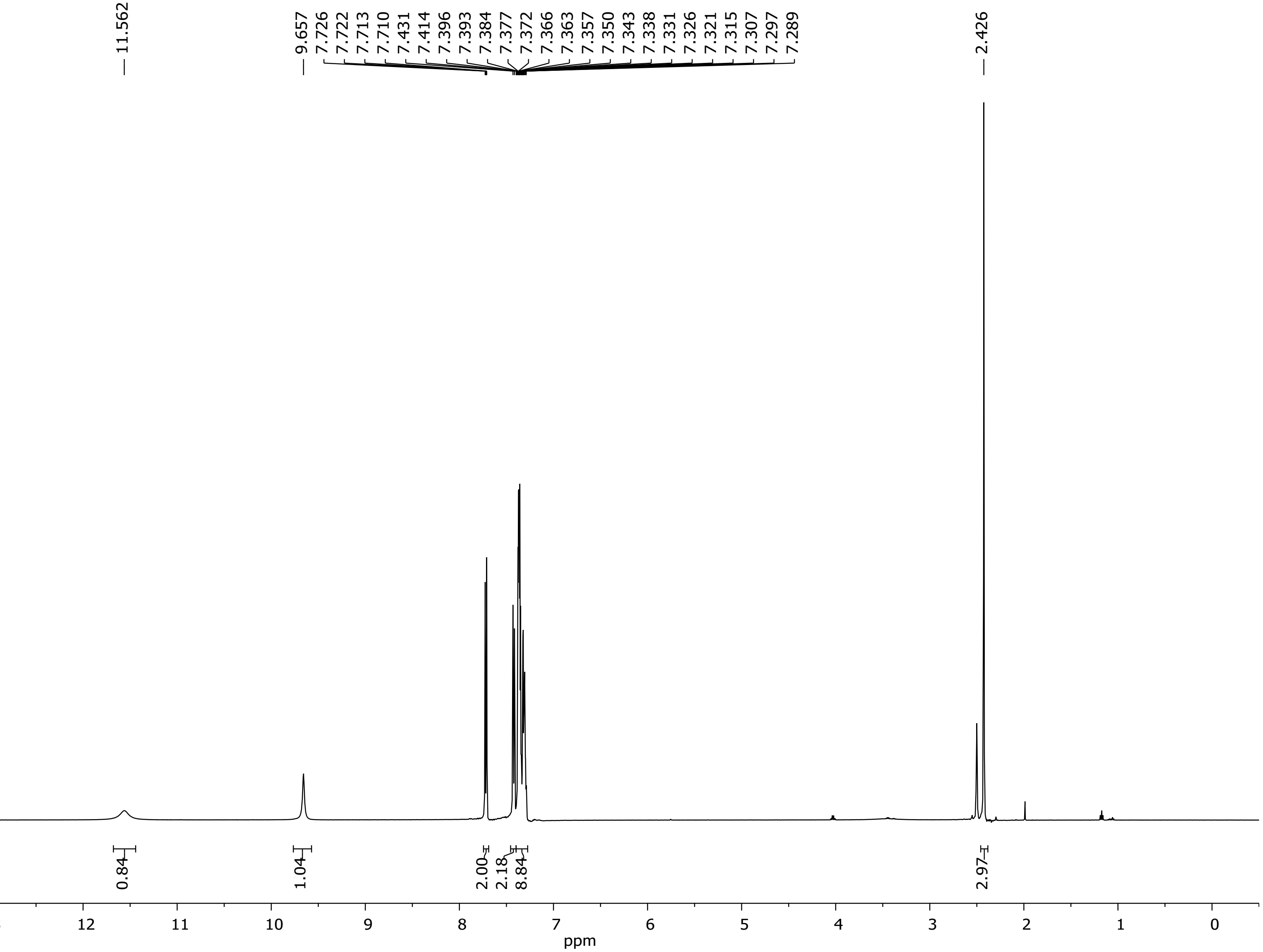

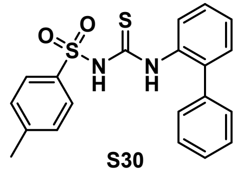

S30

$^{13}C\{^1H\}$, $(CD_3)_2SO$, 126 MHz

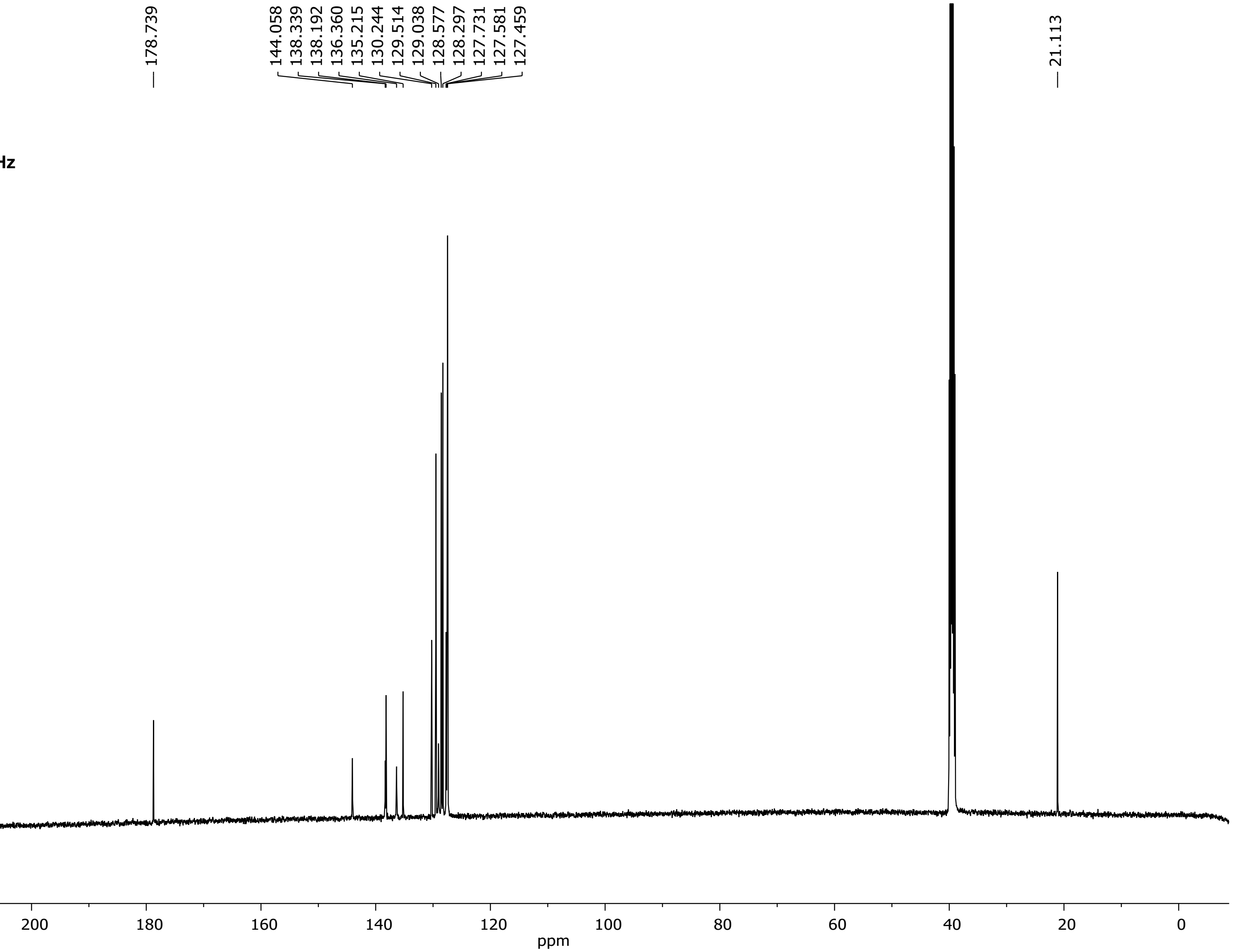

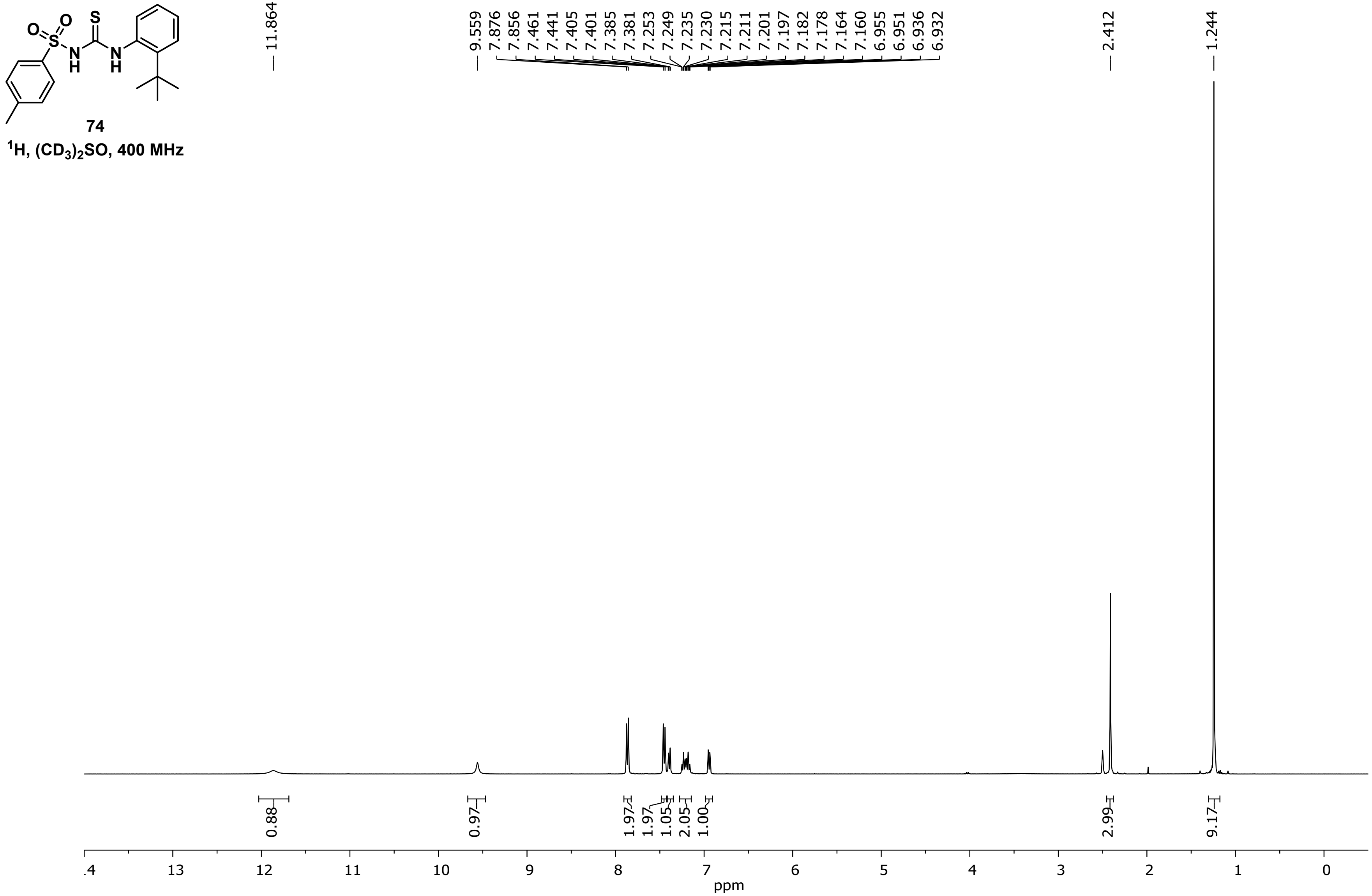
74
1H, (CD3)2SO, 400 MHz
11.864
9.559
7.876
7.856
7.461
7.441
7.405
7.401
7.385
7.381
7.253
7.249
7.235
7.230
7.215
7.211
7.201
7.197
7.182
7.178
7.164
7.160
6.955
6.951
6.936
6.932
2.412
1.244
0.88
0.97
1.97
1.97
1.05
2.05
1.00
2.99
9.17
ppm

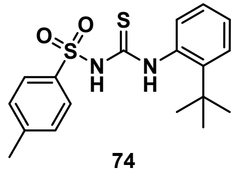

74

$^{13}C\{^{1}H\}$, $(CD_3)_2SO$, 126 MHz

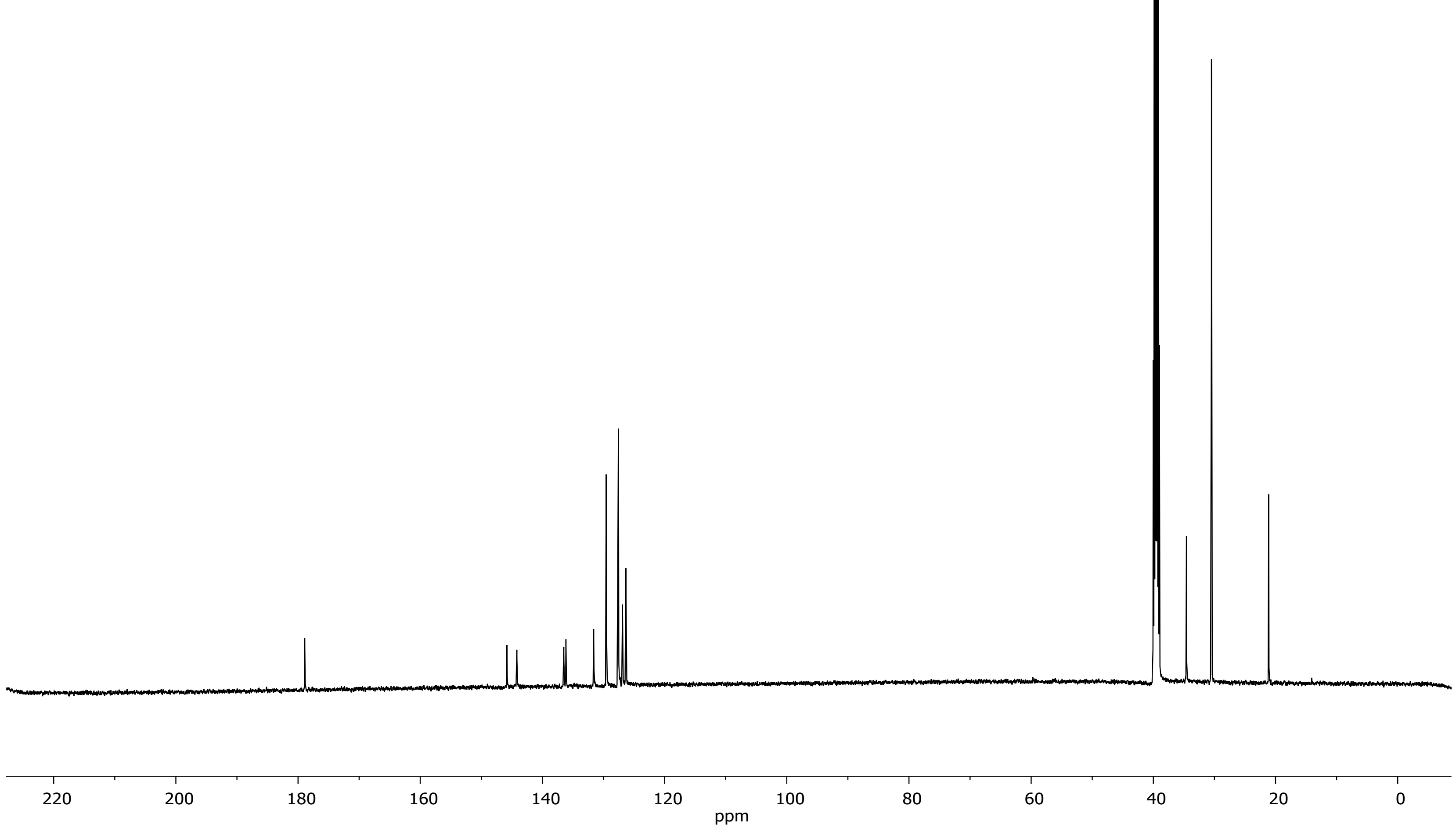

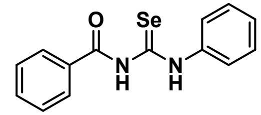

S31

^{1}H, $(CD_3)_2SO$, 400 MHz

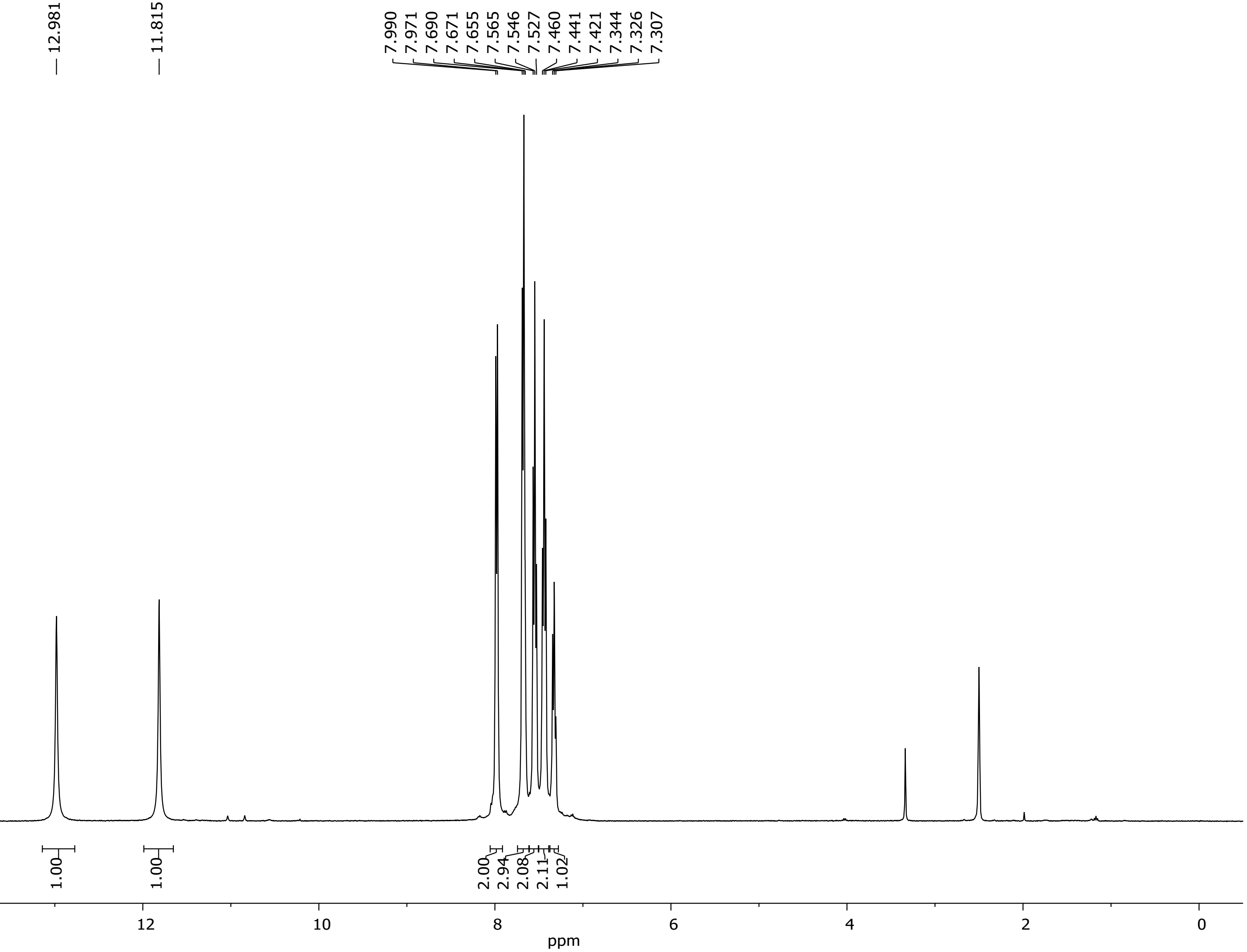

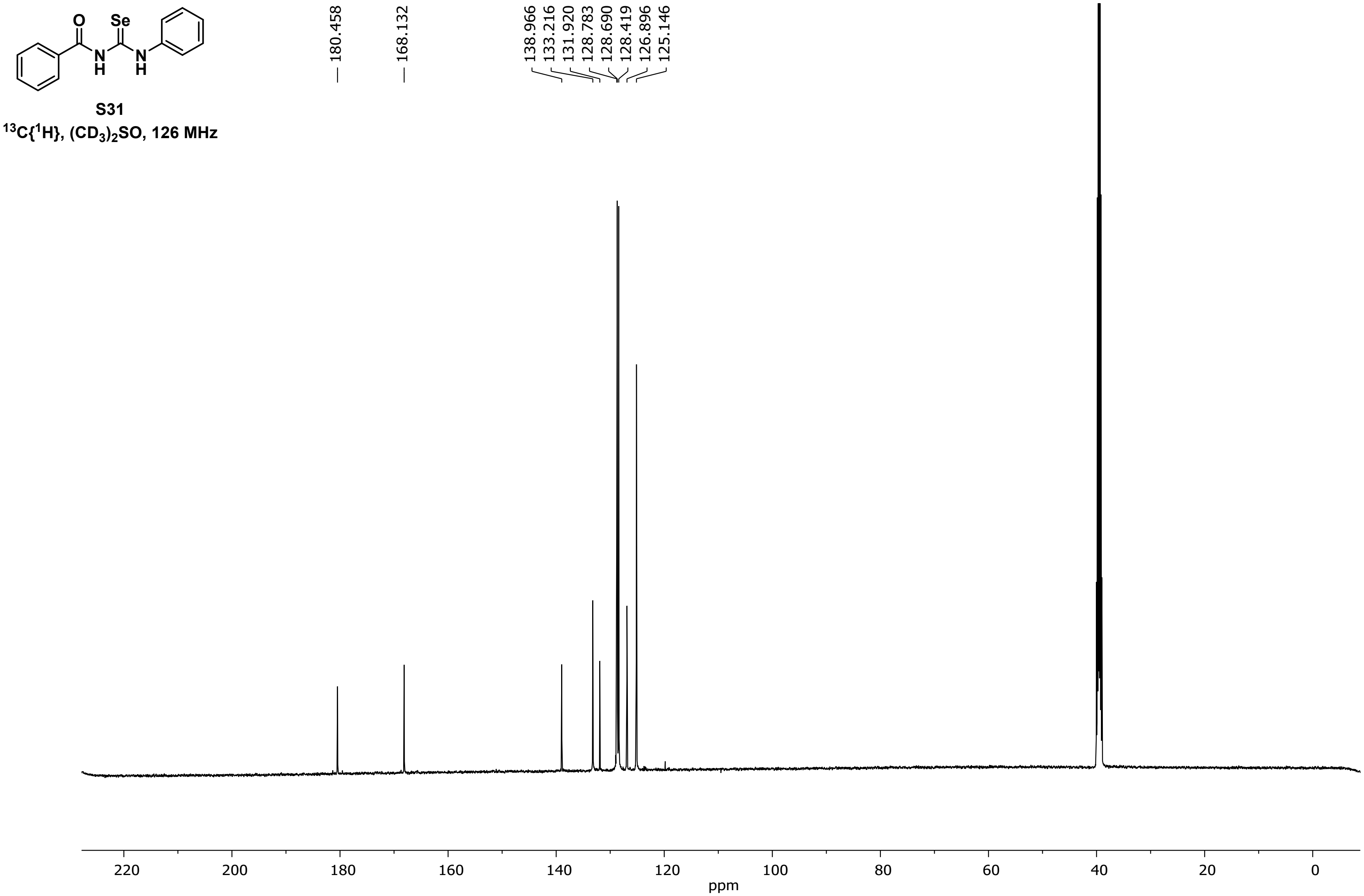
O
Se
N
H
N
H
S31
$^{13}C\{^1H\}$, $(CD_3)_2SO$, 126 MHz
180.458
168.132
138.966
133.216
131.920
128.783
128.690
128.419
126.896
125.146
220
200
180
160
140
120
100
80
60
40
20
0
ppm

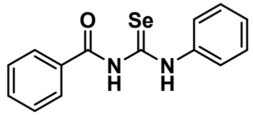

S31

$^{77}Se\{^1H\}$, $(CD_3)_2SO$, 95 MHz

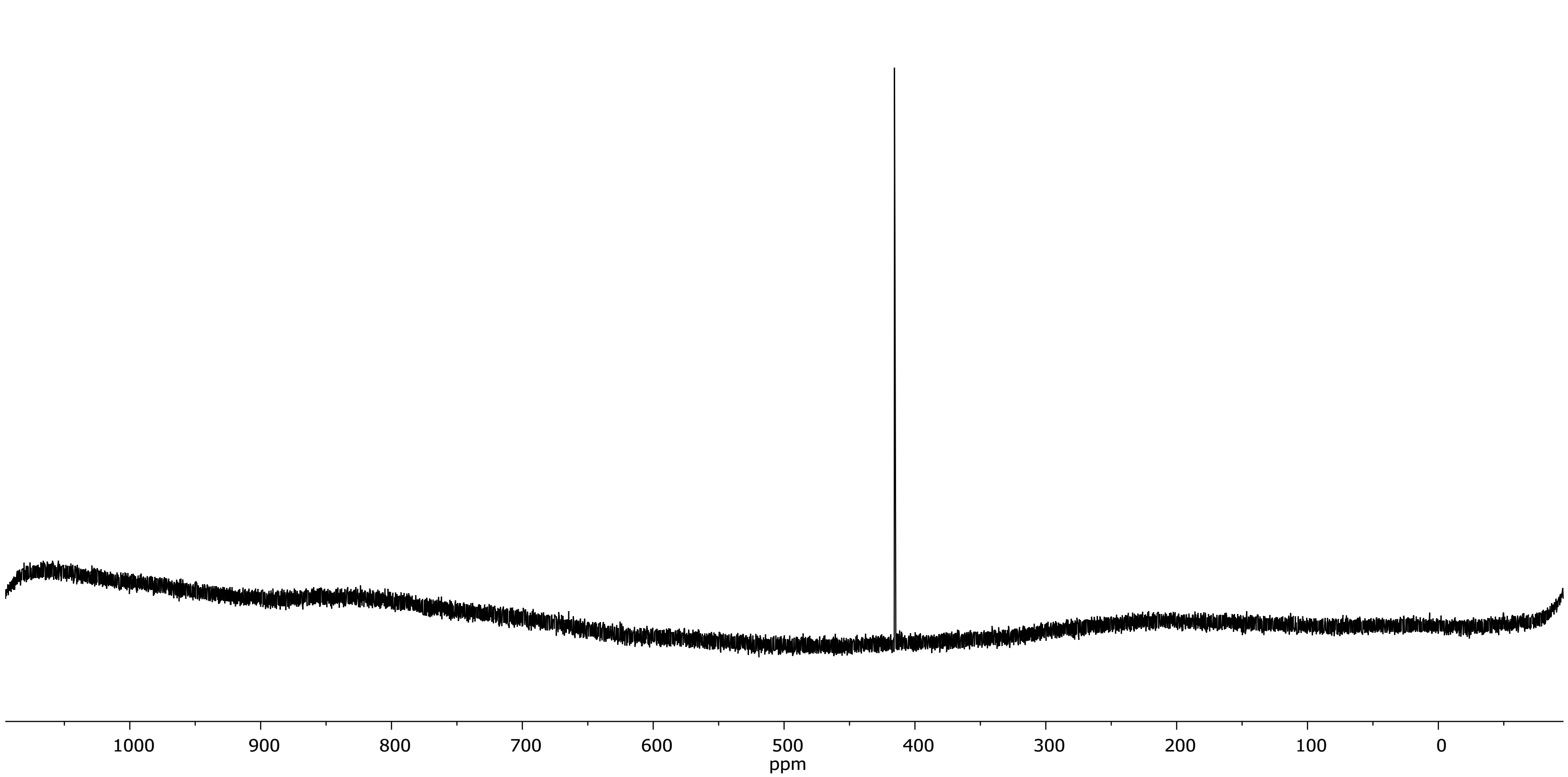

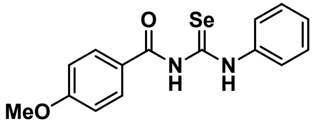

S32

^{1}H, $CDCl_3$, 400 MHz

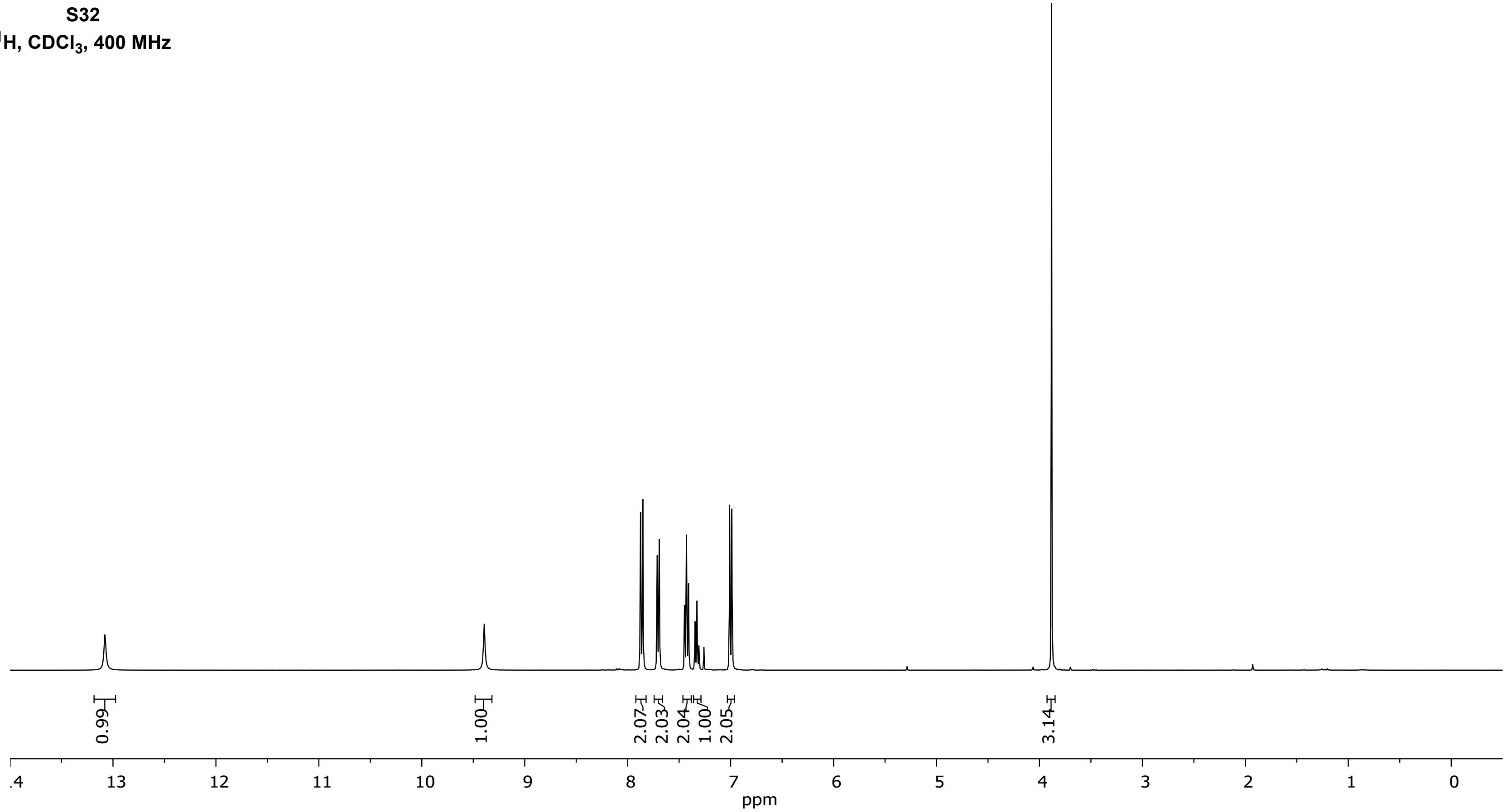

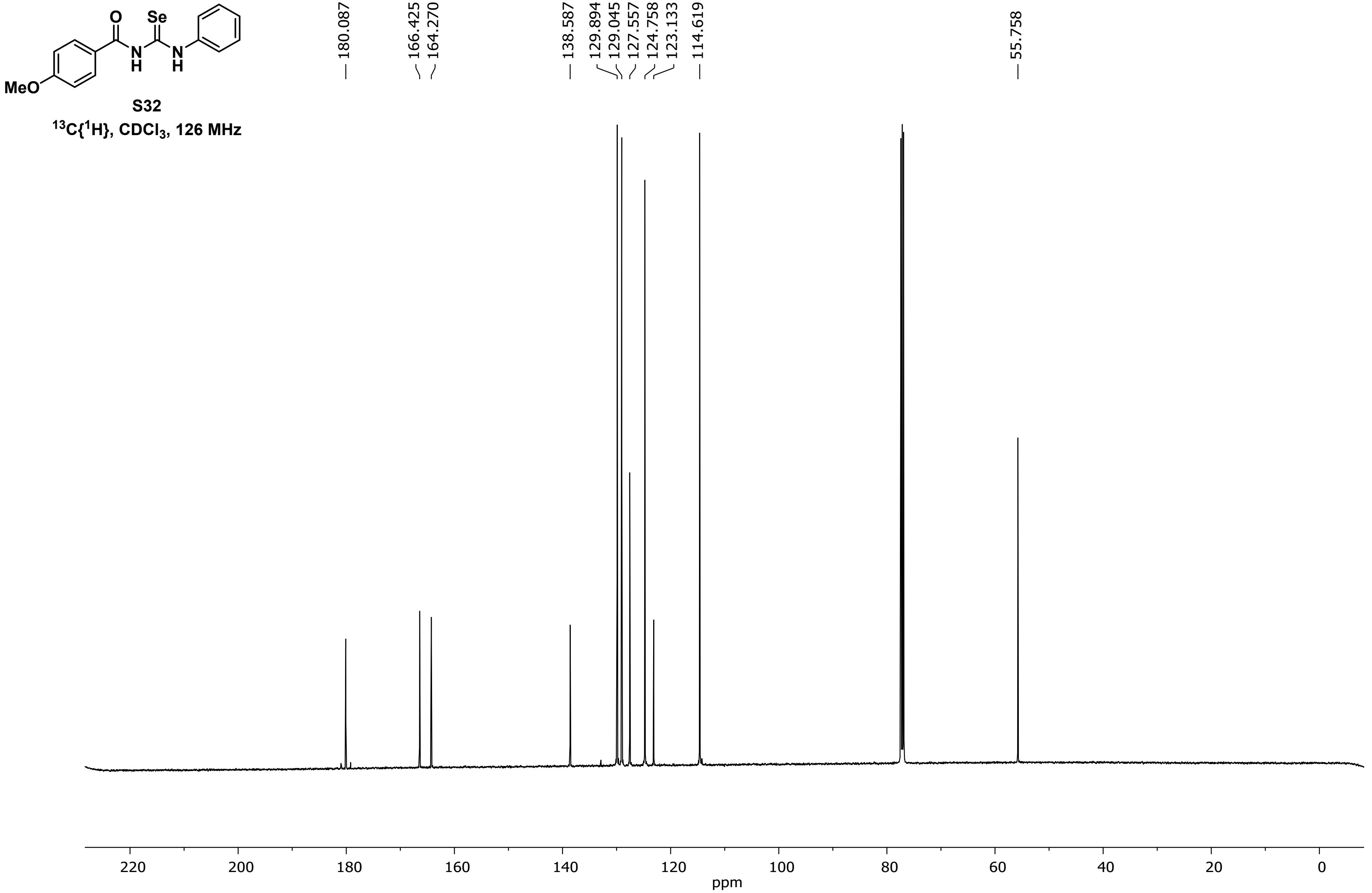

S32

$^{13}C\{^1H\}$, $CDCl_3$, 126 MHz

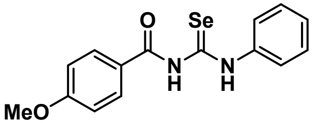

S32

$^{77}Se\{^1H\}$, $CDCl_3$, 95 MHz

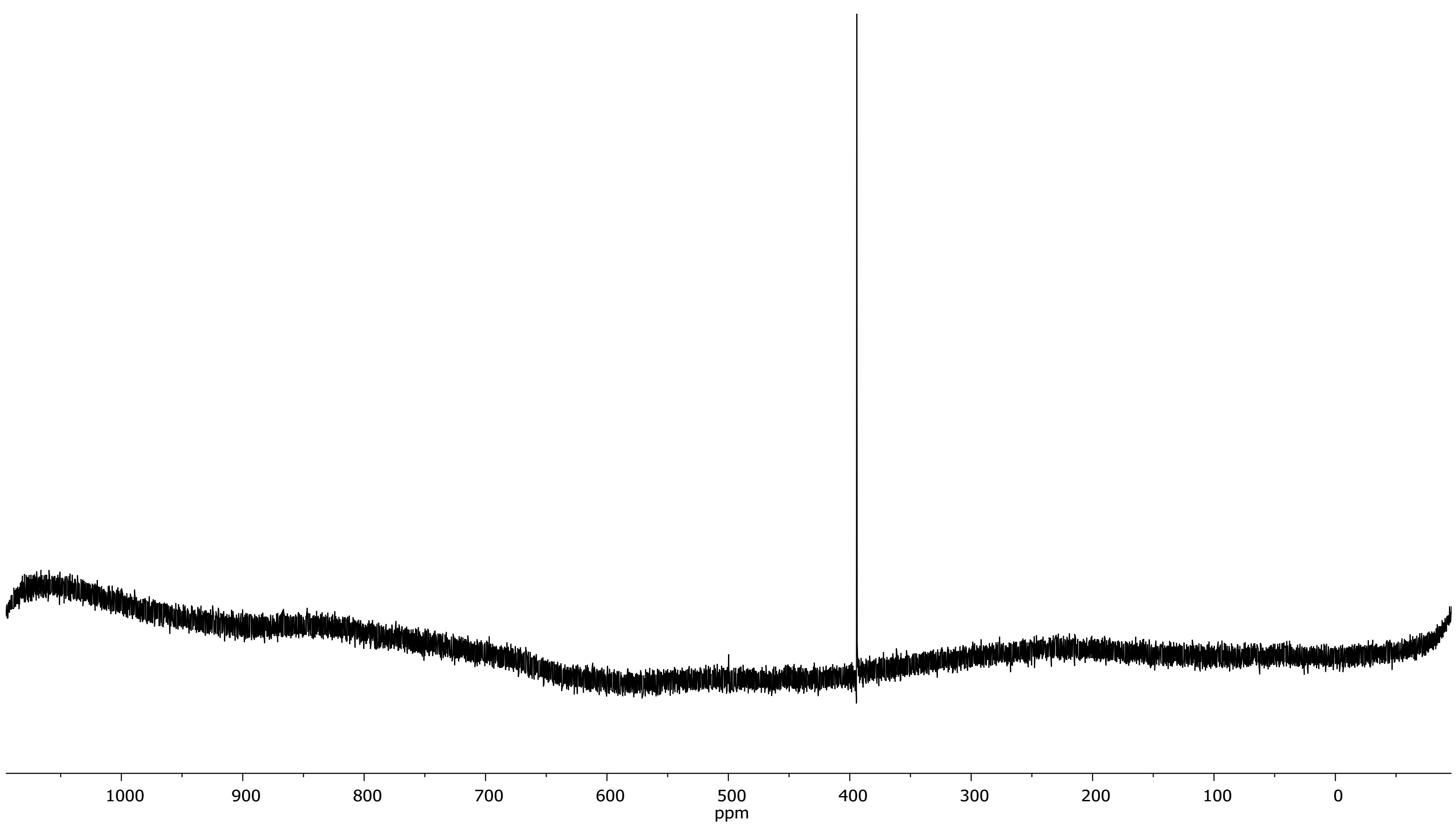

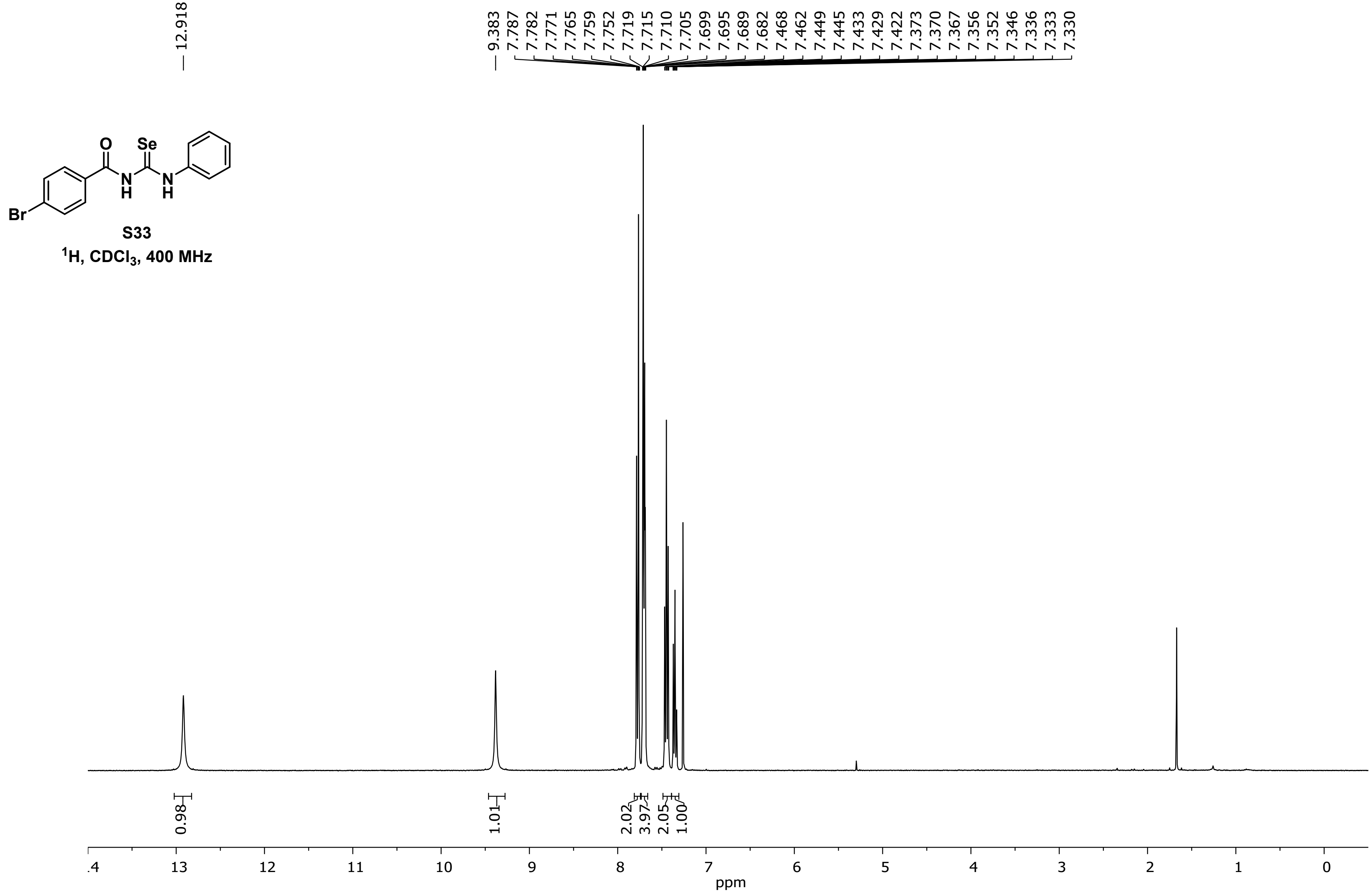

12.918
9.383
7.787 7.782 7.771 7.765 7.759 7.752 7.719 7.715 7.710 7.705 7.699 7.695 7.689 7.682 7.468 7.462 7.449 7.445 7.433 7.429 7.422 7.373 7.370 7.367 7.356 7.352 7.346 7.336 7.333 7.330
O
Se
N
H
N
H
Br
S33
^{1}H, $CDCl_3$, 400 MHz
0.98
1.01
2.02
3.97
2.05
1.00
.4
13
12
11
10
9
8
7
6
5
4
3
2
1
0
ppm

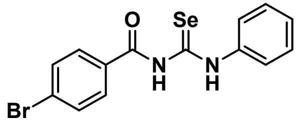

S33

$^{13}C\{^{1}H\}$, $CDCl_3$, 126 MHz

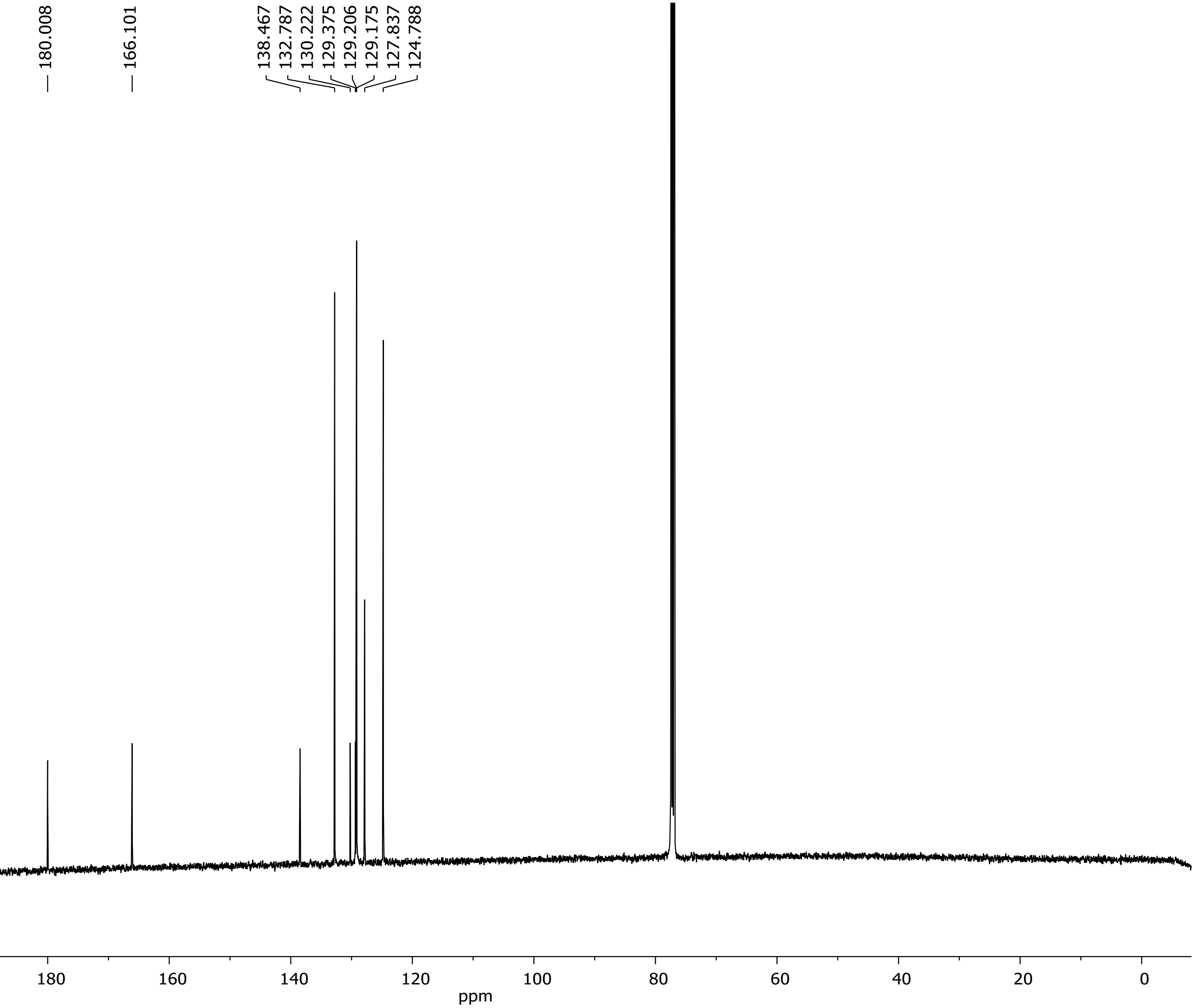

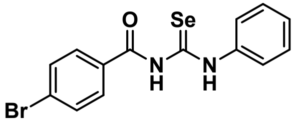

S33

$^{77}Se\{^{1}H\}$, $CDCl_3$, 95 MHz

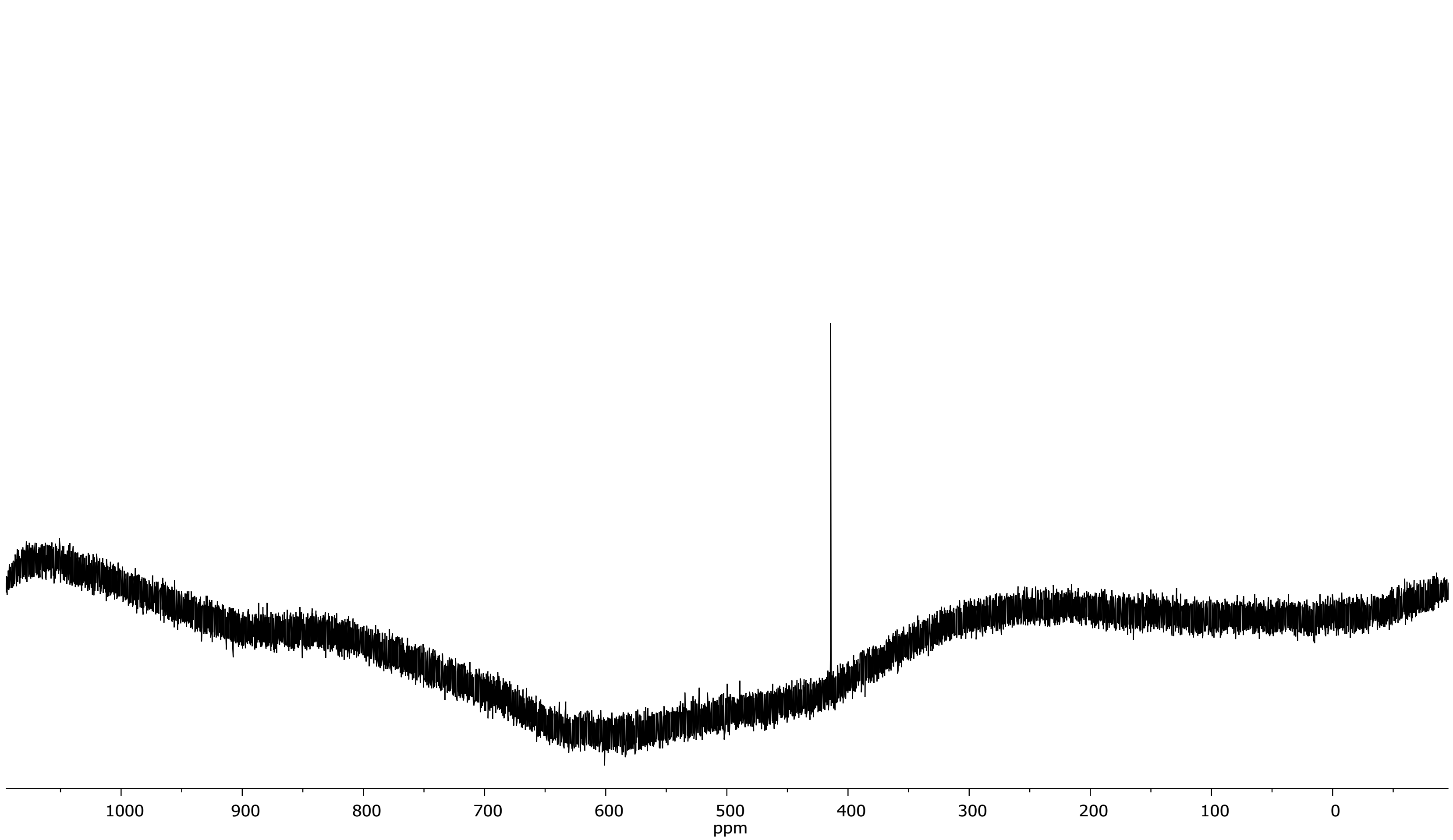

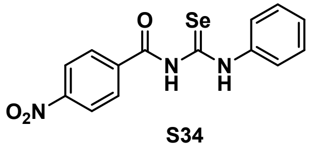

S34

^{1}H, $CDCl_3$, 400 MHz

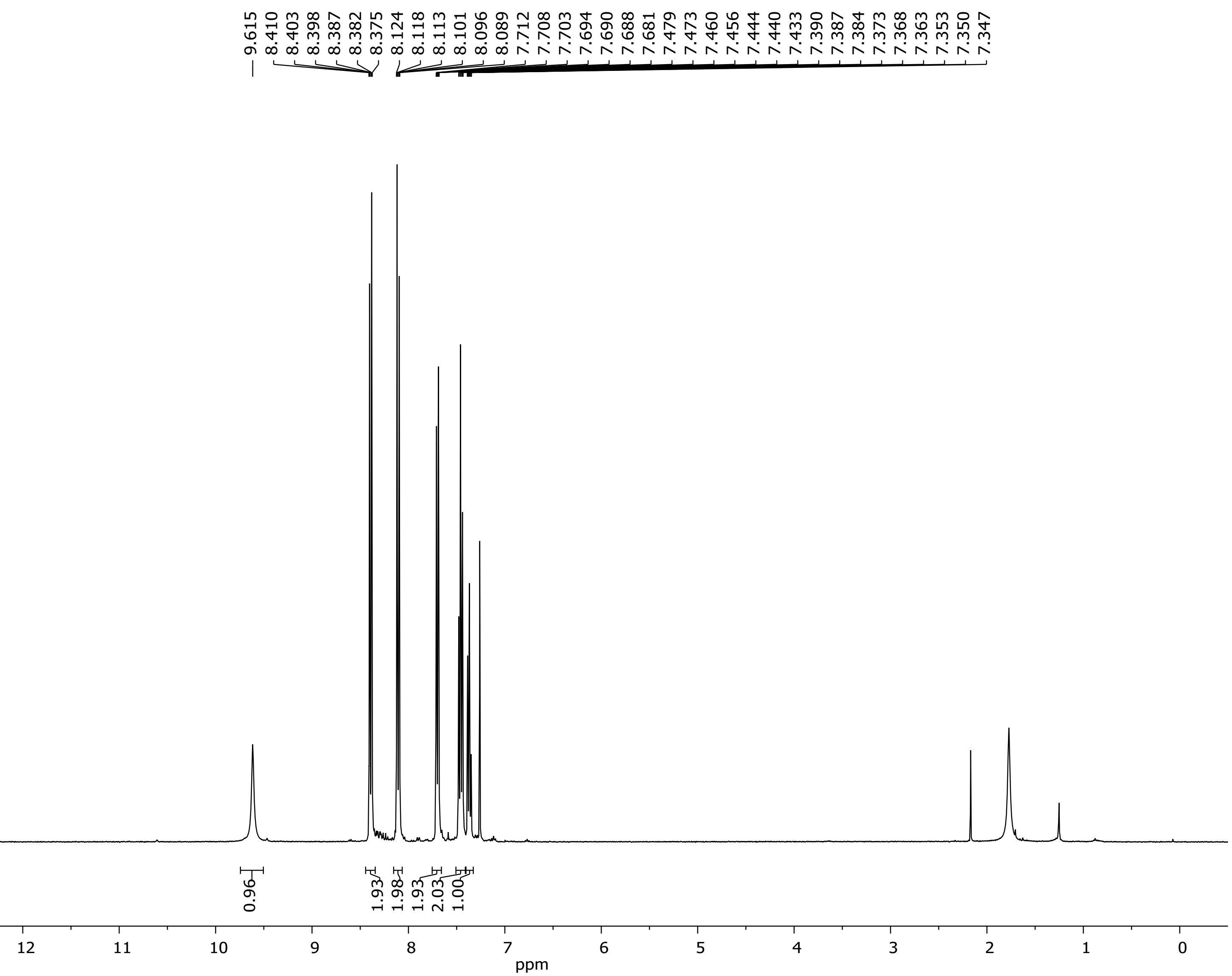

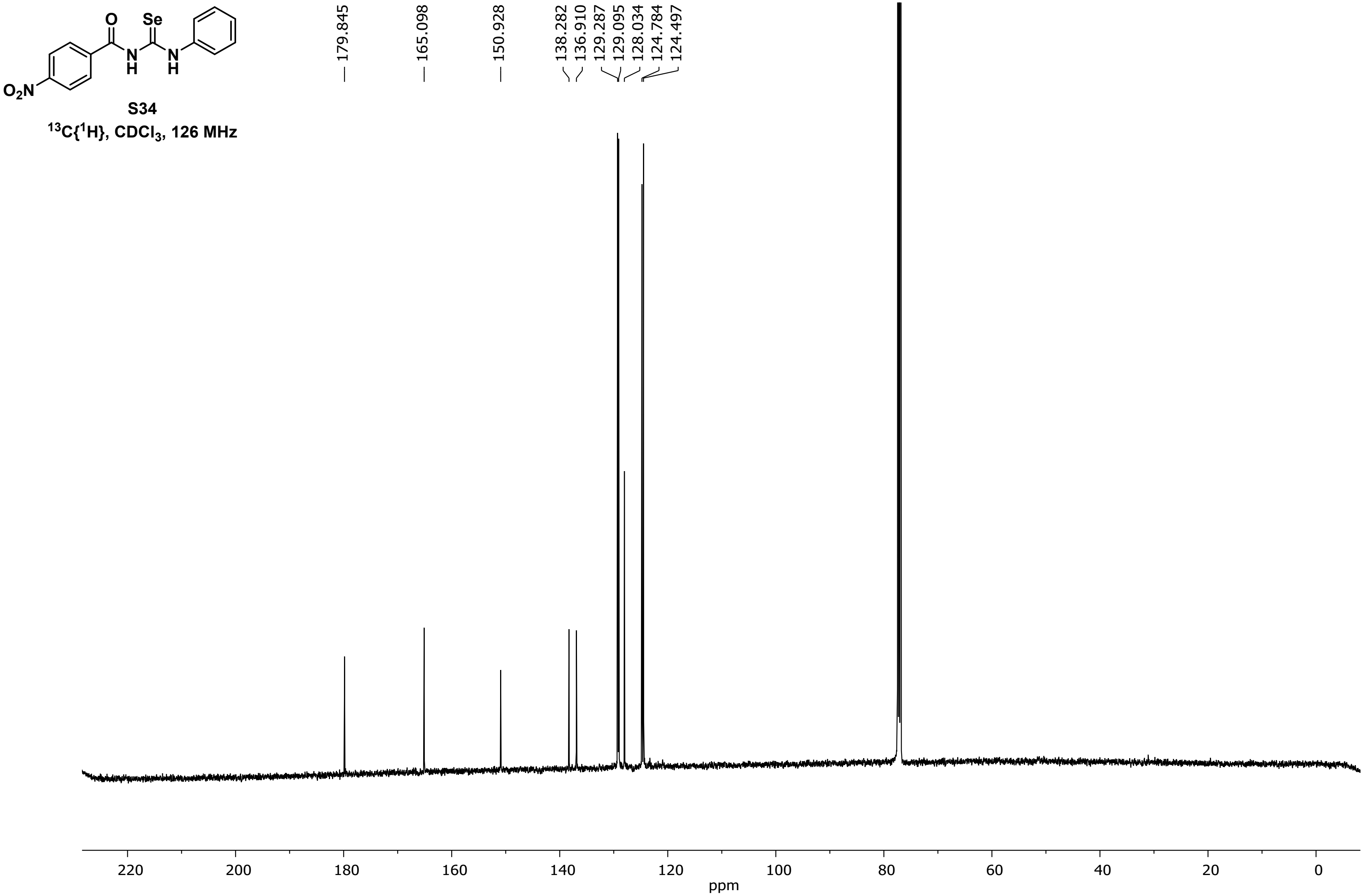

S34

$^{13}C\{^{1}H\}$, $CDCl_3$, 126 MHz

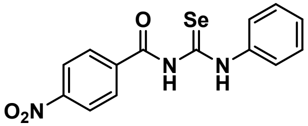

S34

$^{77}Se\{^1H\}$, $CDCl_3$, 95 MHz

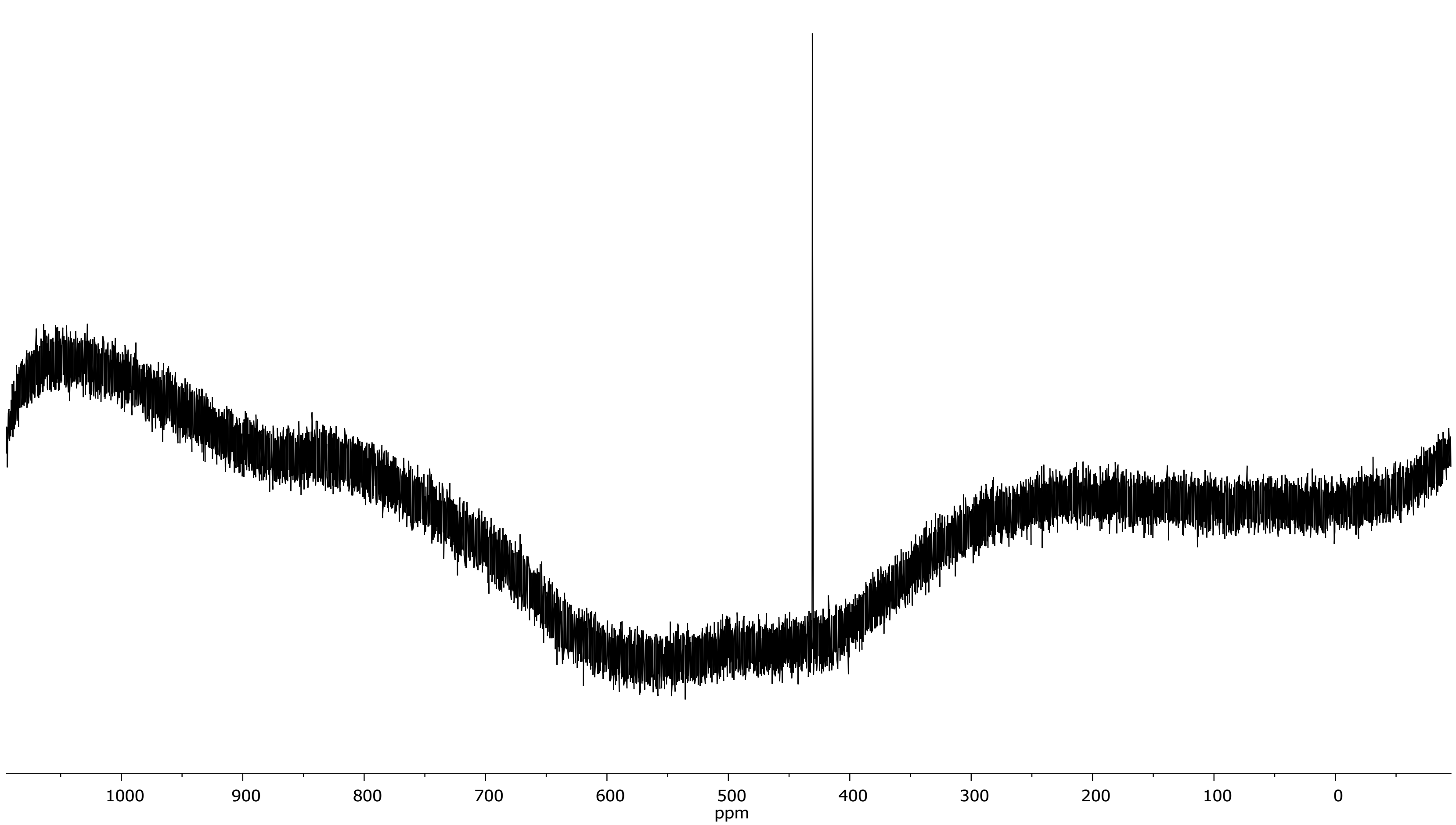

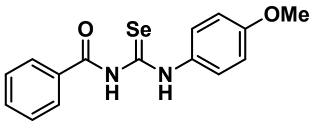

S35
^1H, $CDCl_3$, 500 MHz

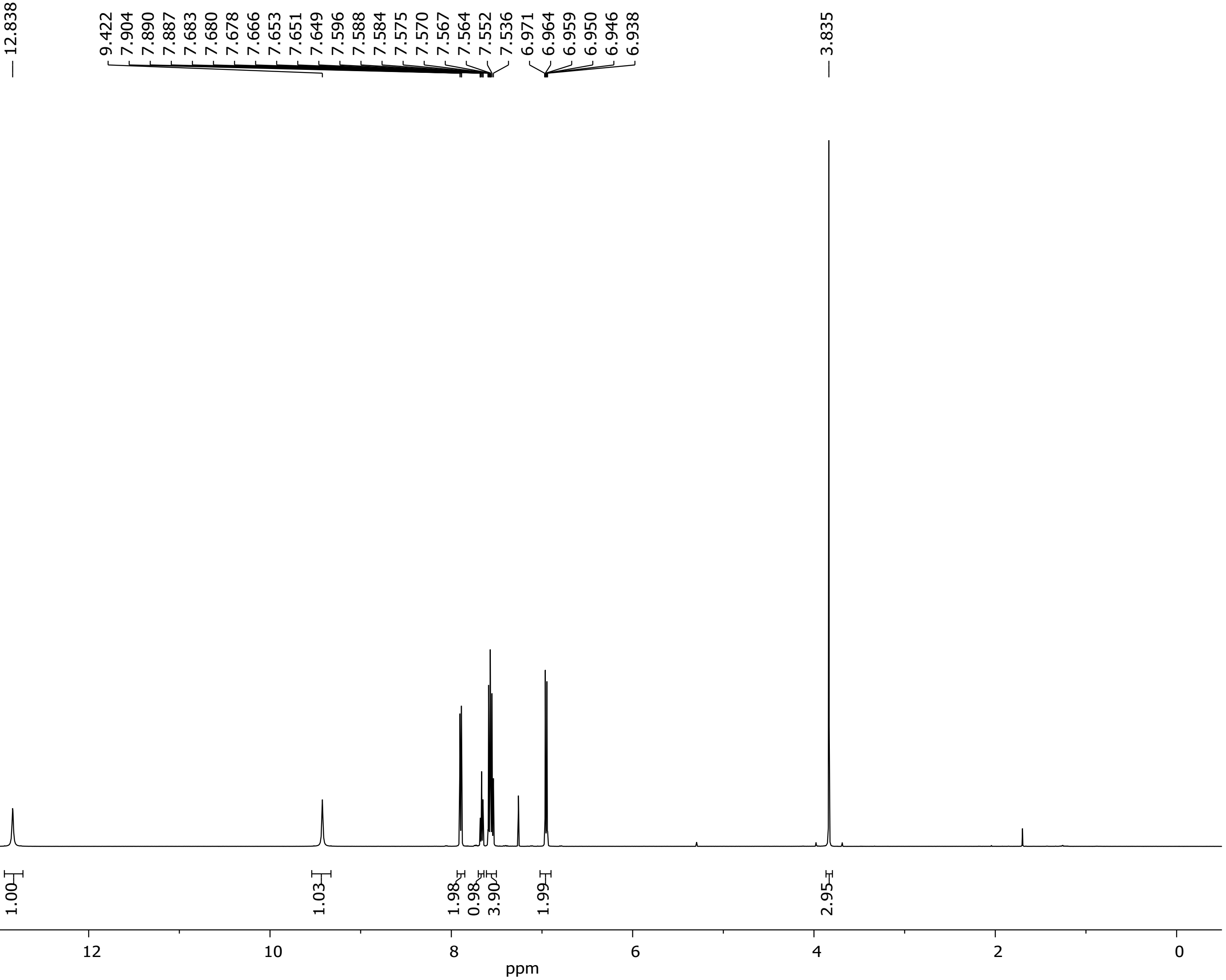

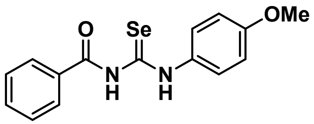

S35

$^{13}C\{^{1}H\}$, $CDCl_3$, 126 MHz

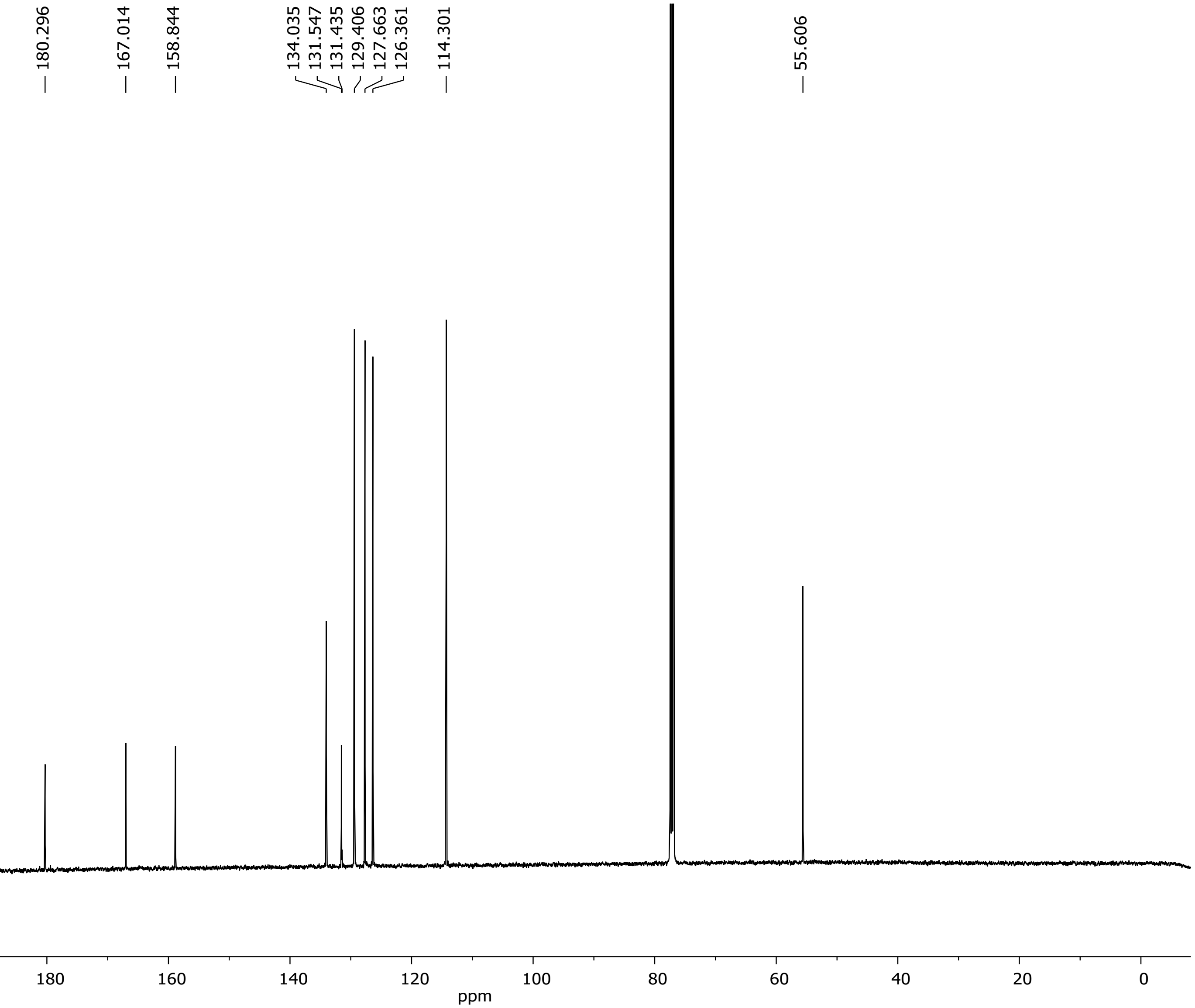

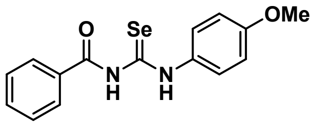

S35

$^{77}Se\{^{1}H\}$, $CDCl_3$, 95 MHz

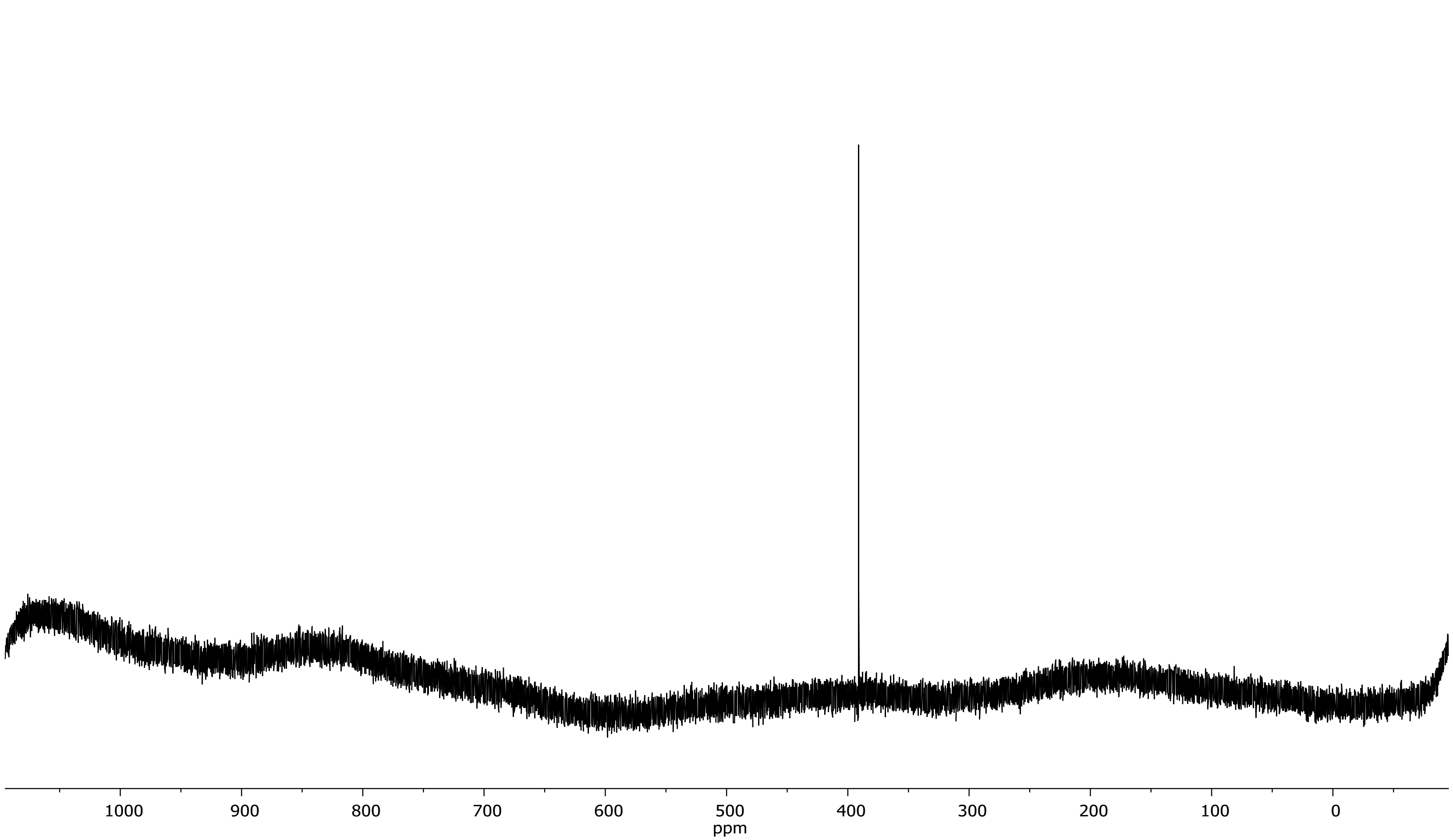

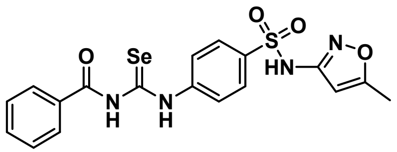

S36

^{1}H, $(CD_3)_2SO$, 400 MHz

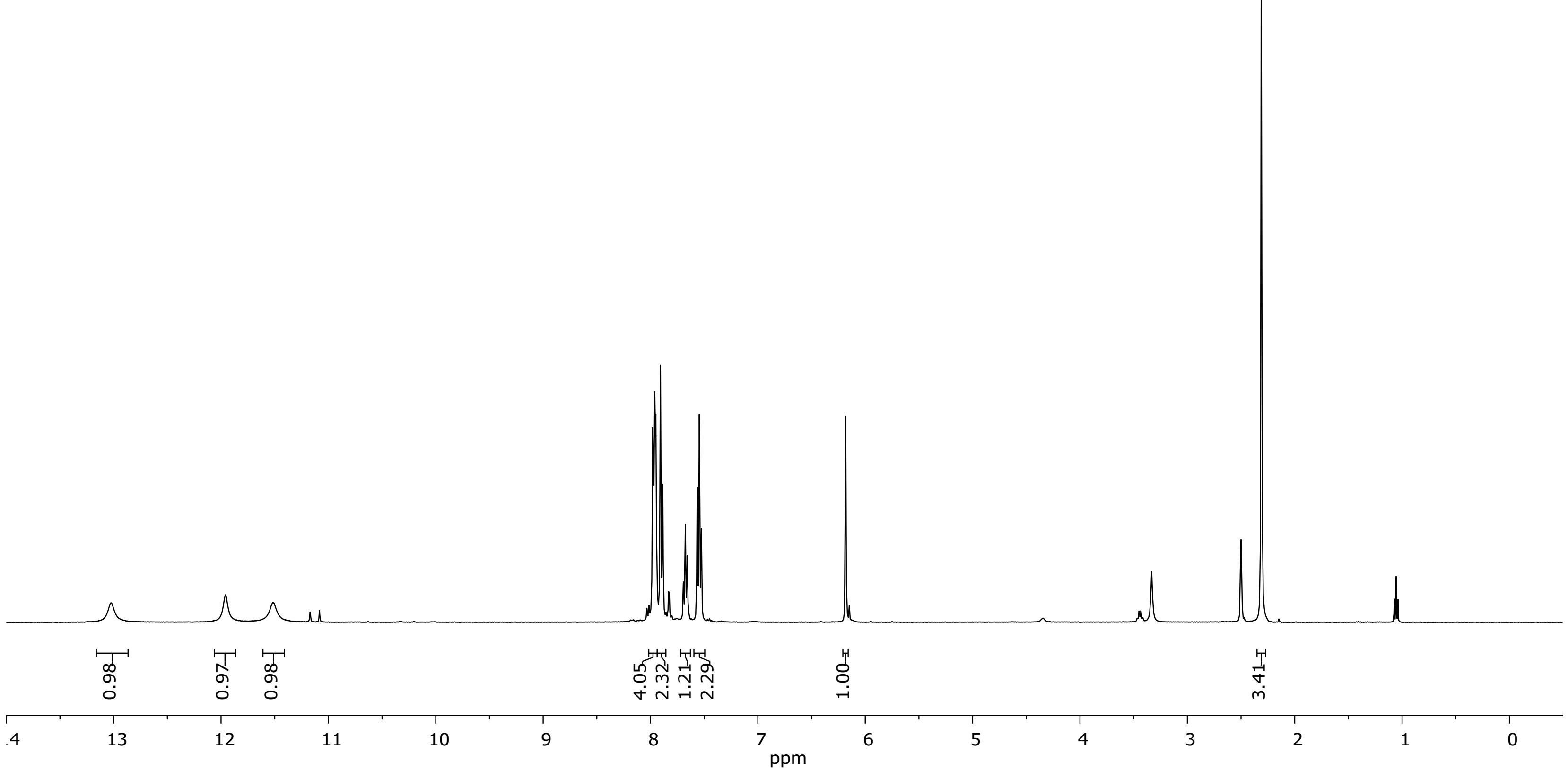

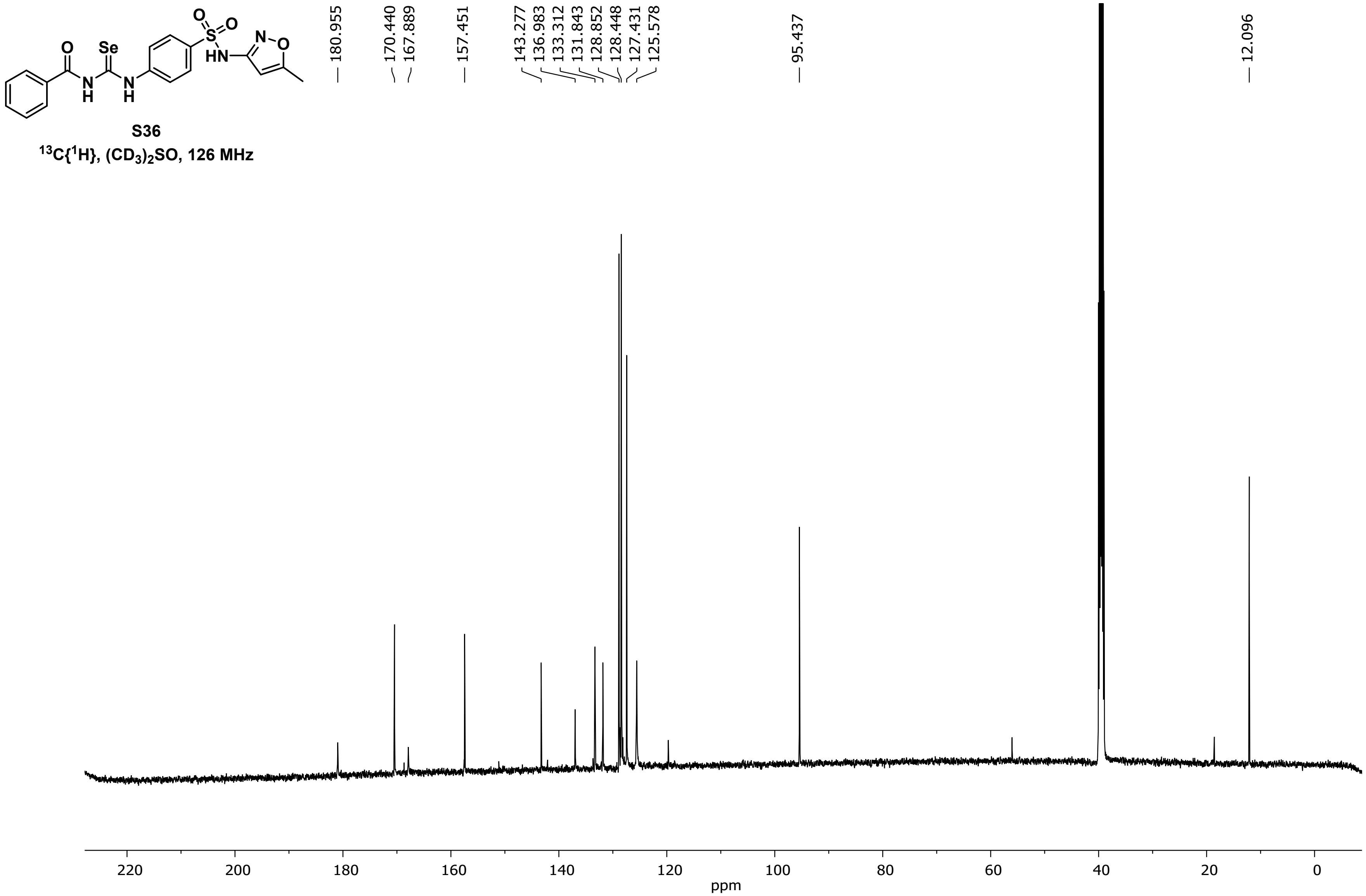

S36
$^{13}C\{^{1}H\}$, $(CD_3)_2SO$, 126 MHz
180.955
170.440
167.889
157.451
143.277
136.983
133.312
131.843
128.852
128.448
127.431
125.578
95.437
12.096
220
200
180
160
140
120
100
80
60
40
20
0
ppm

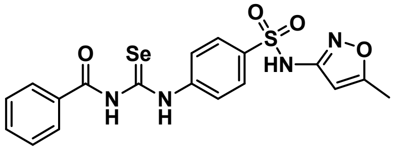

S36

$^{77}Se\{^1H\}$, $(CD_3)_2SO$, 95 MHz

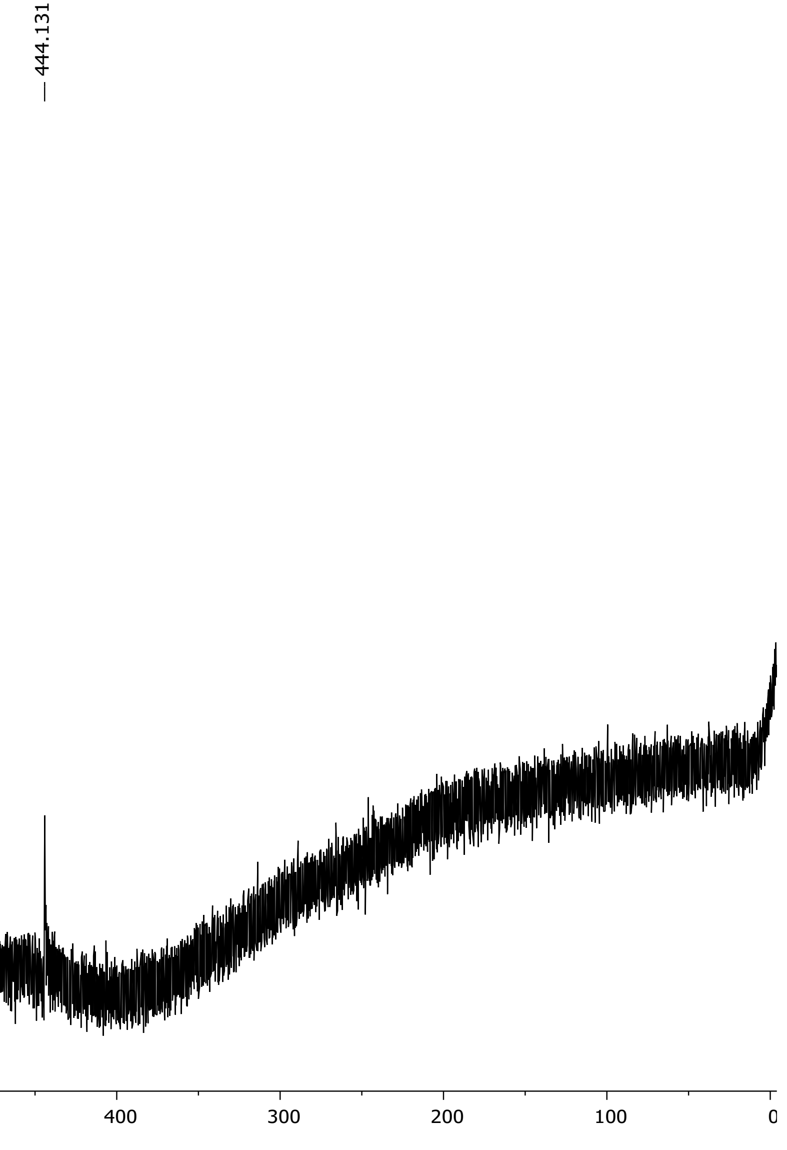

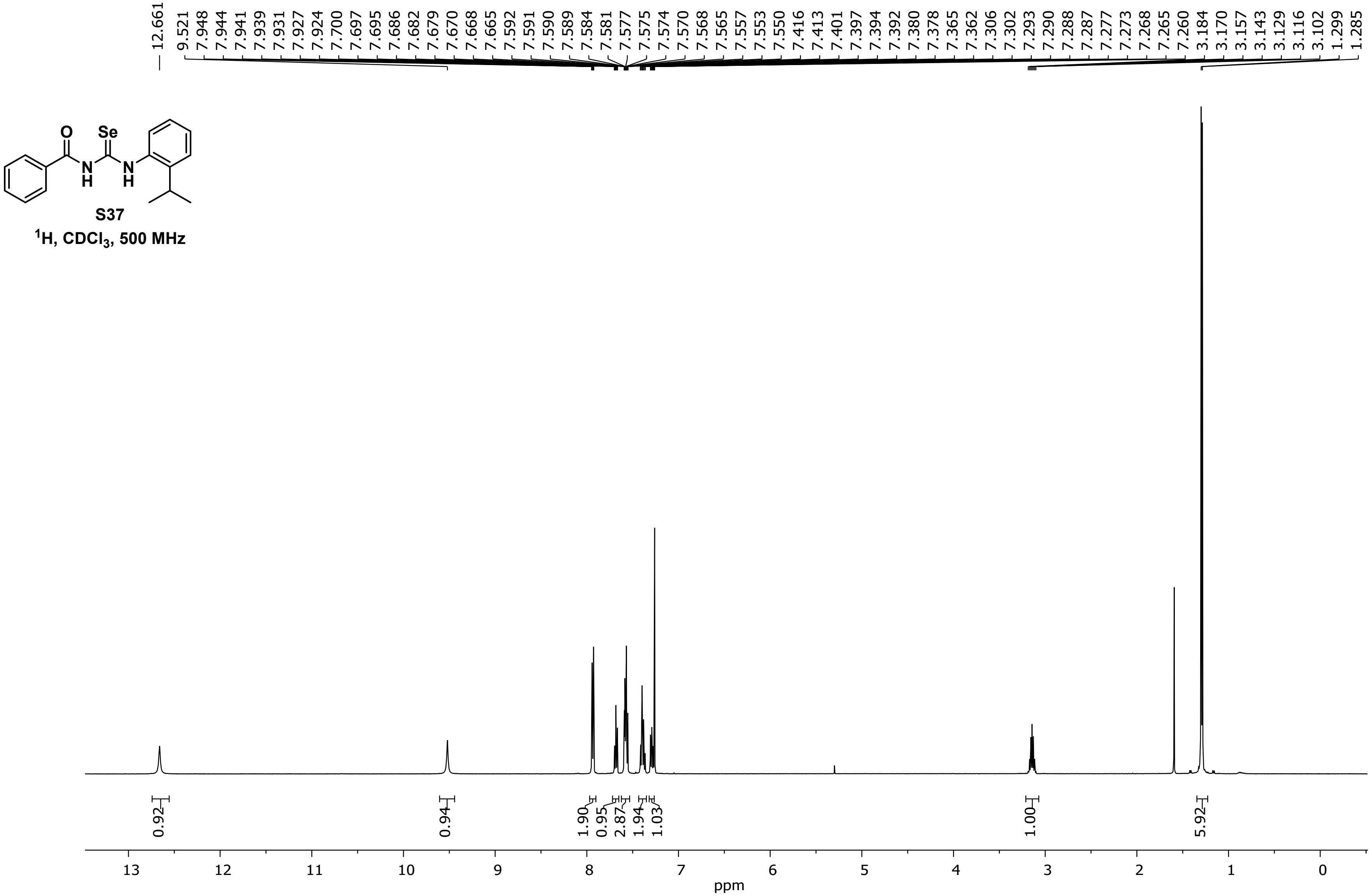

S37

^{1}H, $CDCl_3$, 500 MHz

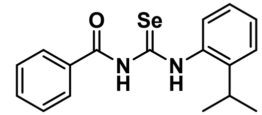

S37

$^{13}C\{^{1}H\}$, $CDCl_3$, 126 MHz

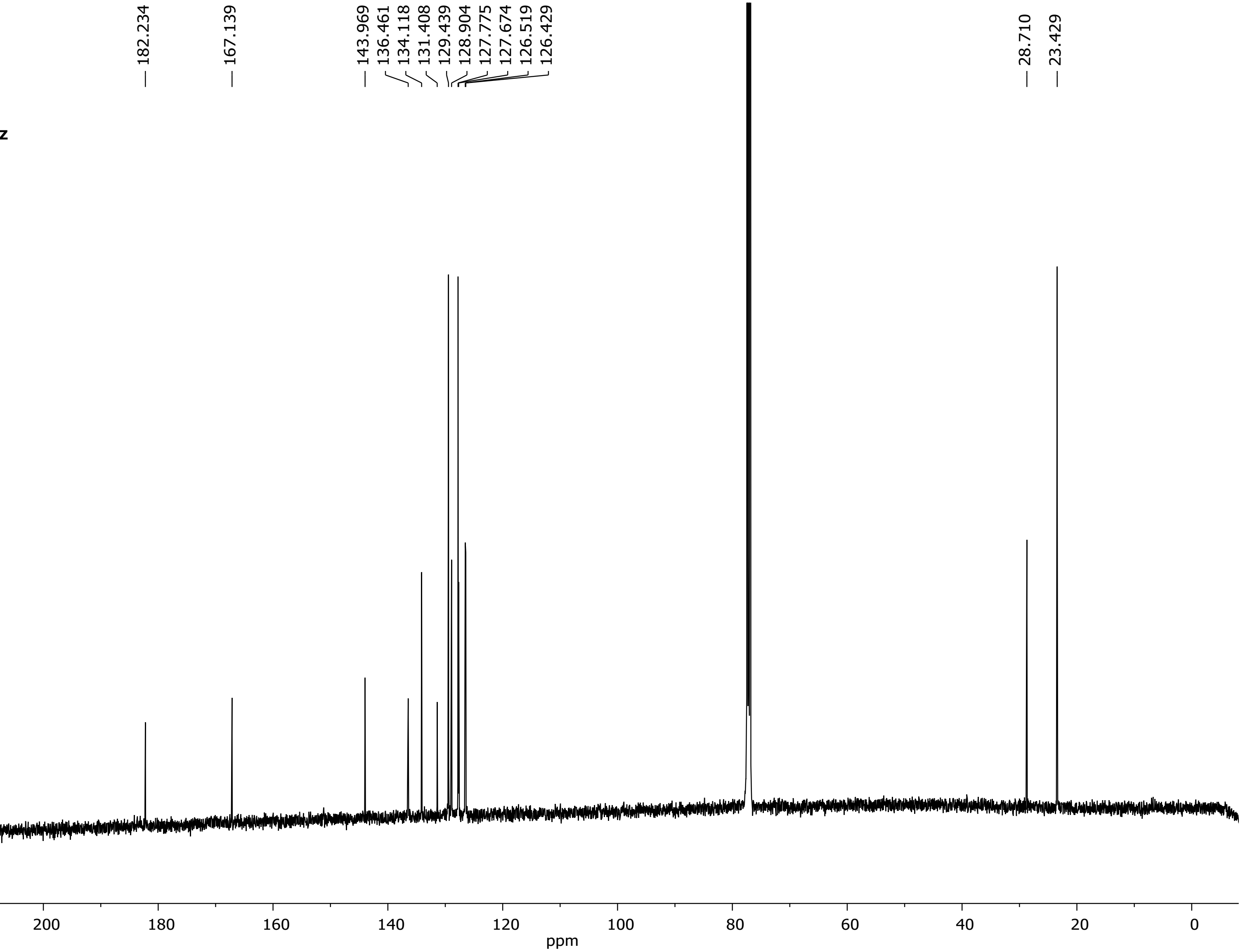

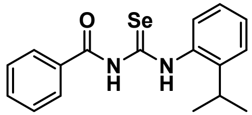

S37

$^{77}Se\{^1H\}$, $CDCl_3$, 95 MHz

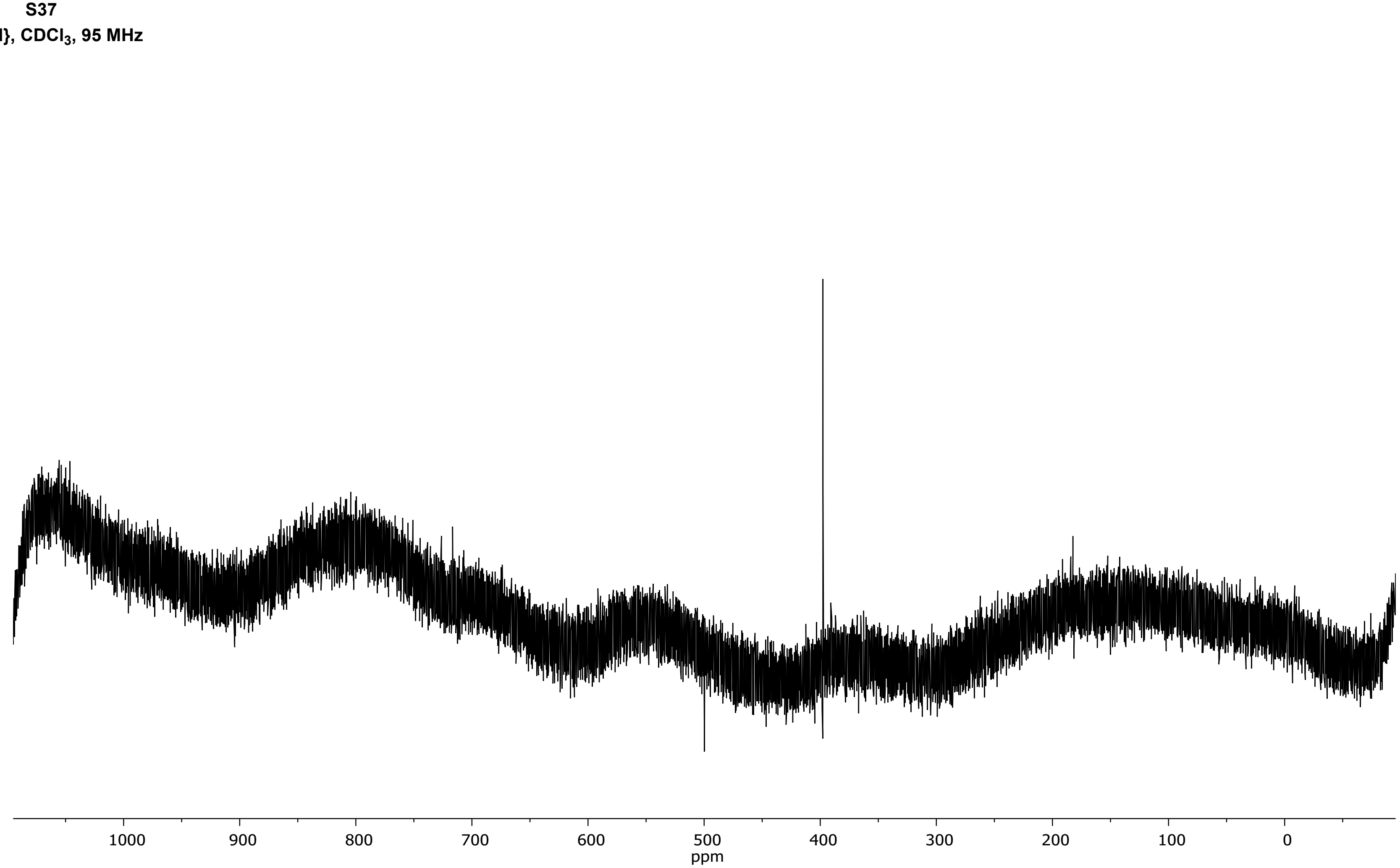

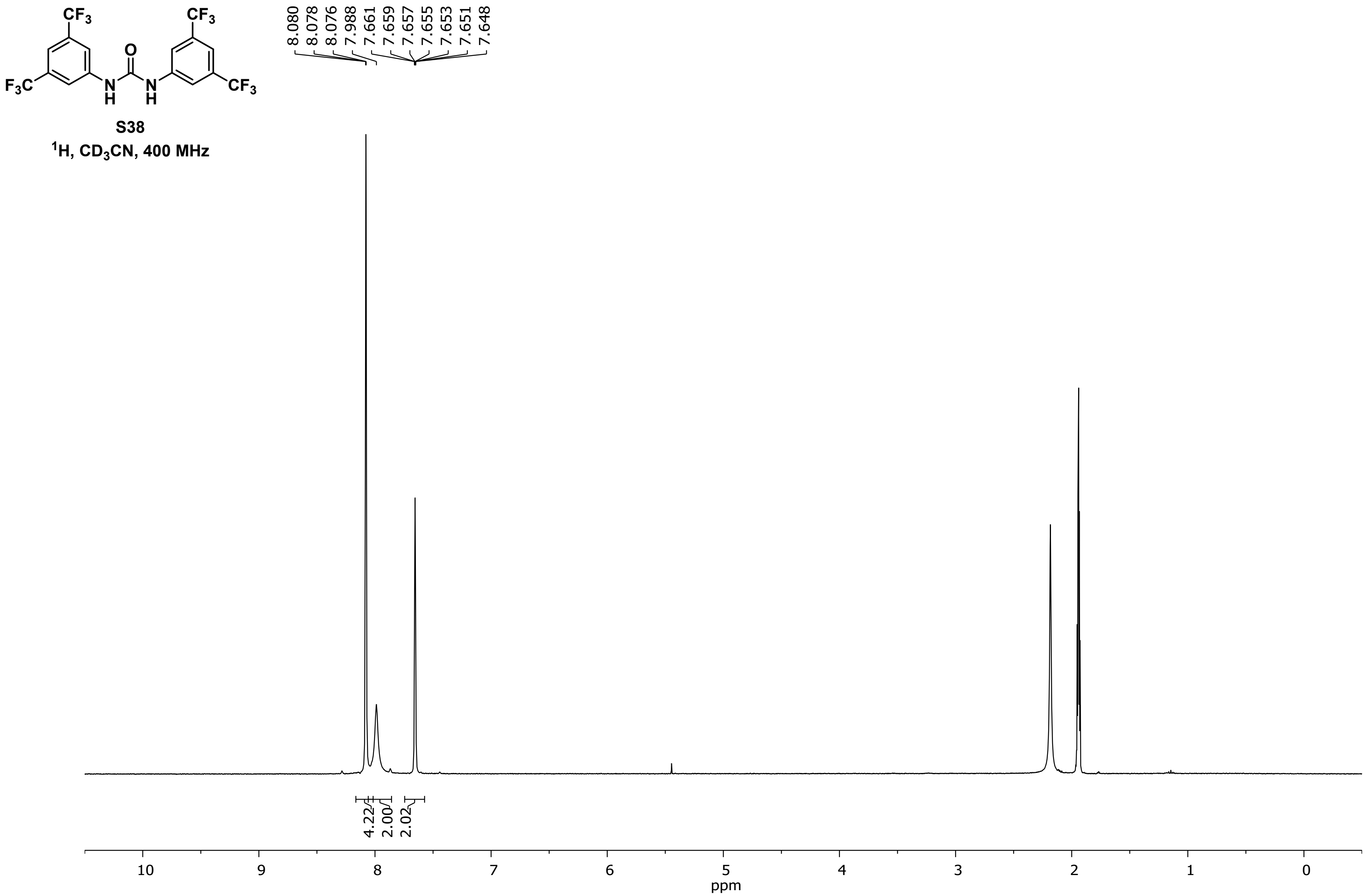
S38
1H, CD3CN, 400 MHz
8.080
8.078
8.076
7.988
7.661
7.659
7.657
7.655
7.653
7.651
7.648
4.22
2.00
2.02
ppm

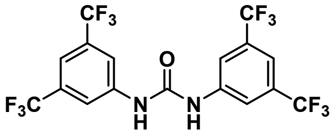

S38

$^{19}F\{^1H\}$, CD_3CN, 376 MHz

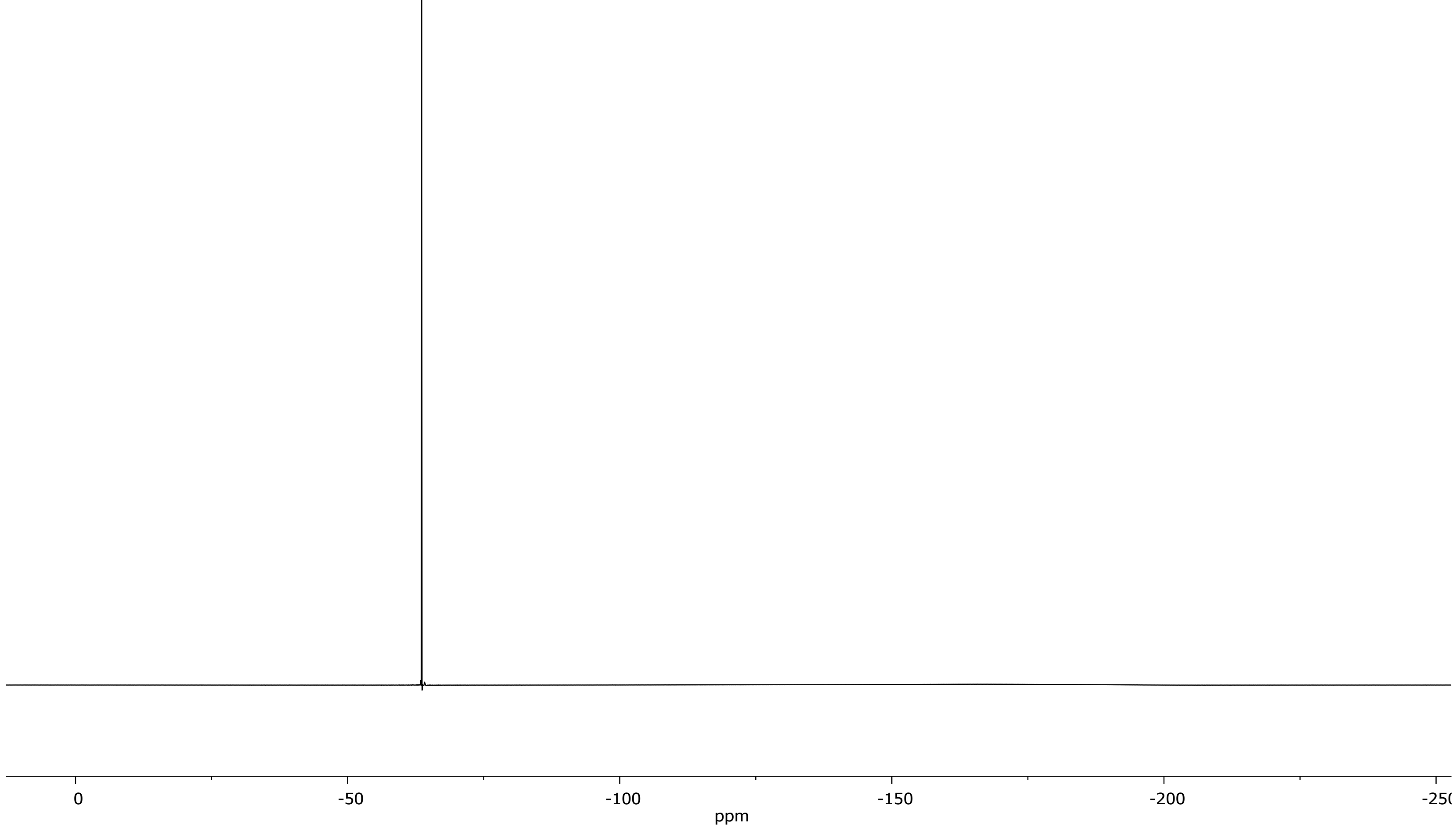

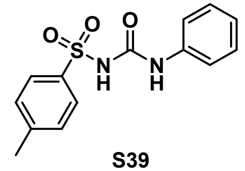

S39

^1H, $CDCl_3$, 400 MHz

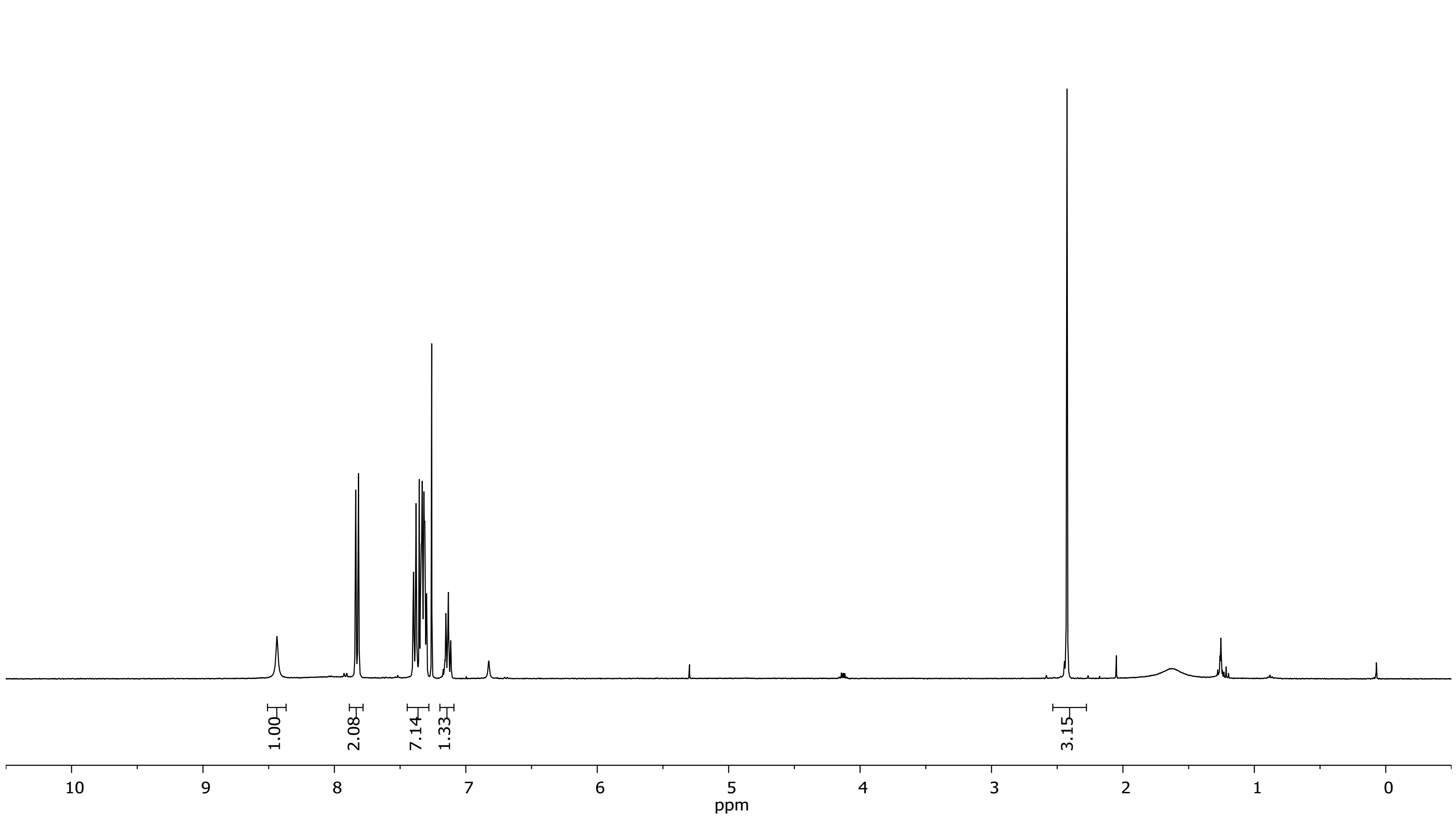

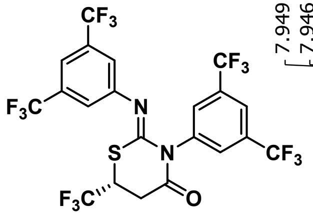

33

^{1}H, CDCl$_3$, 400 MHz

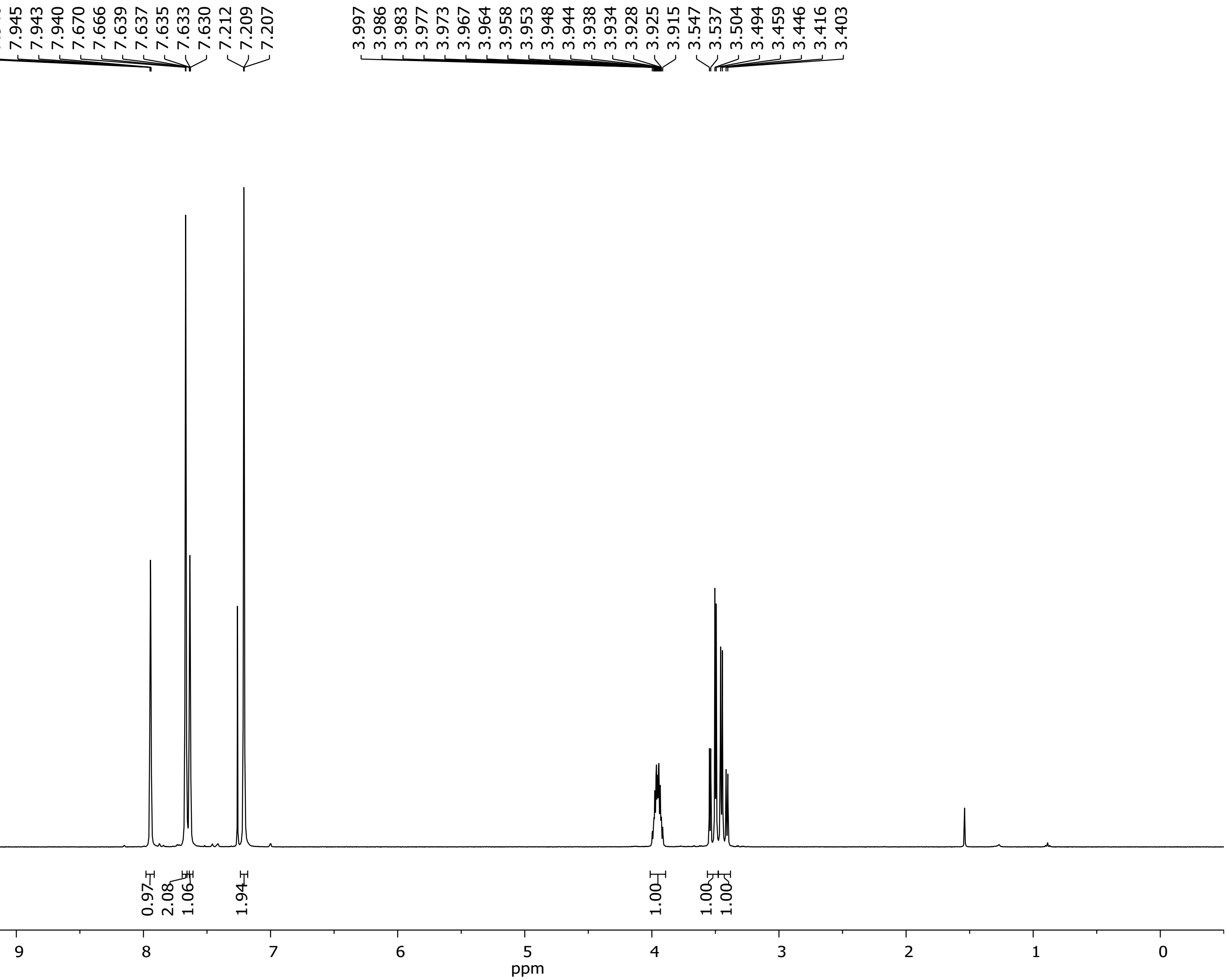

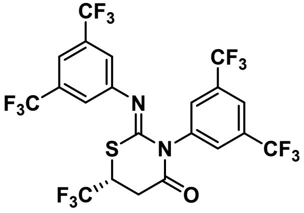

33

$^{19}F\{^{1}H\}$, $CDCl_3$, 376 MHz

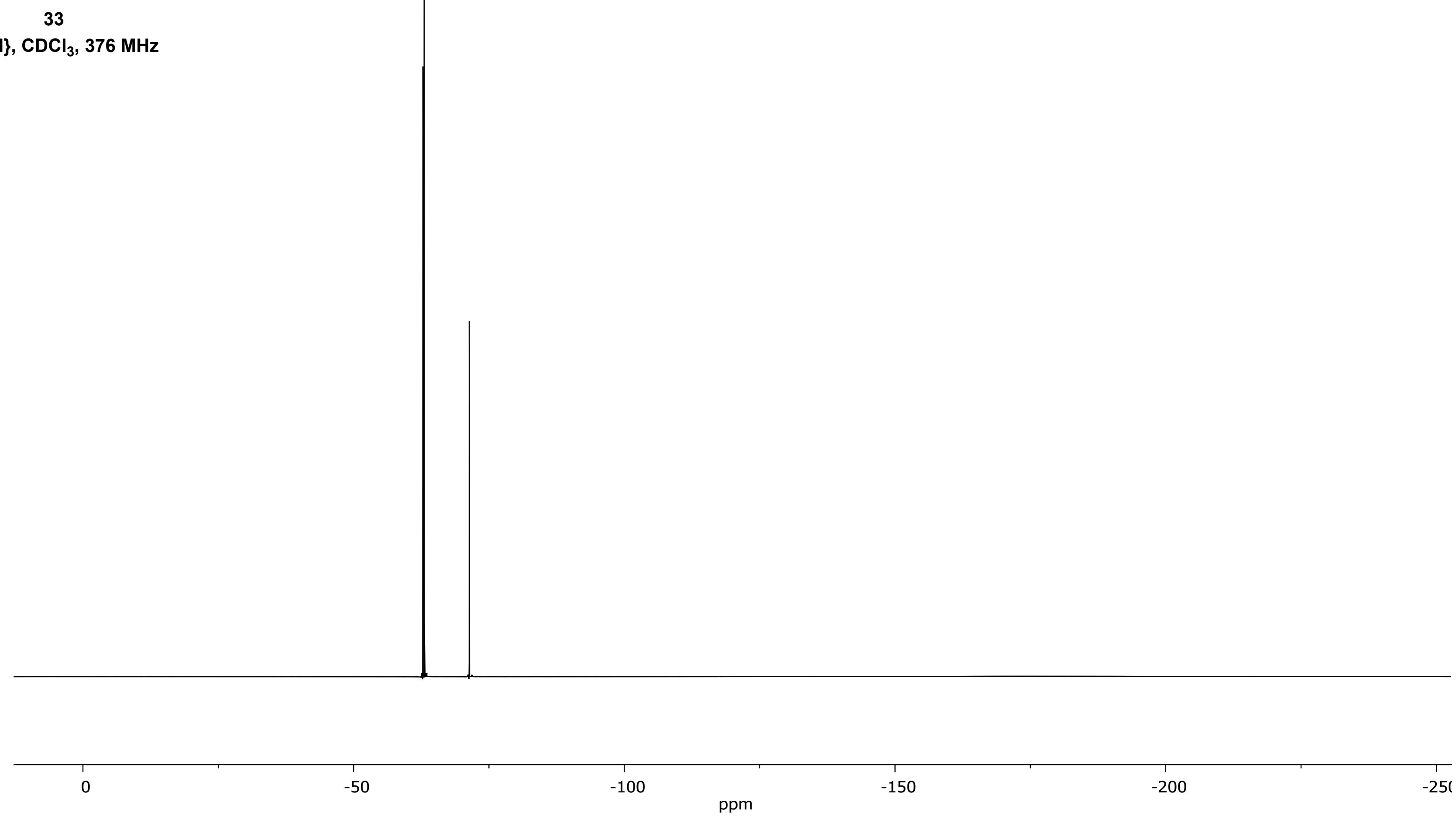

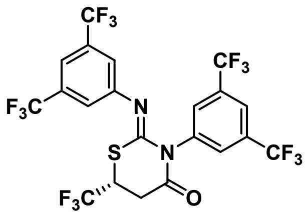

33

$^{13}C\{^1H\}$, $CDCl_3$, 126 MHz

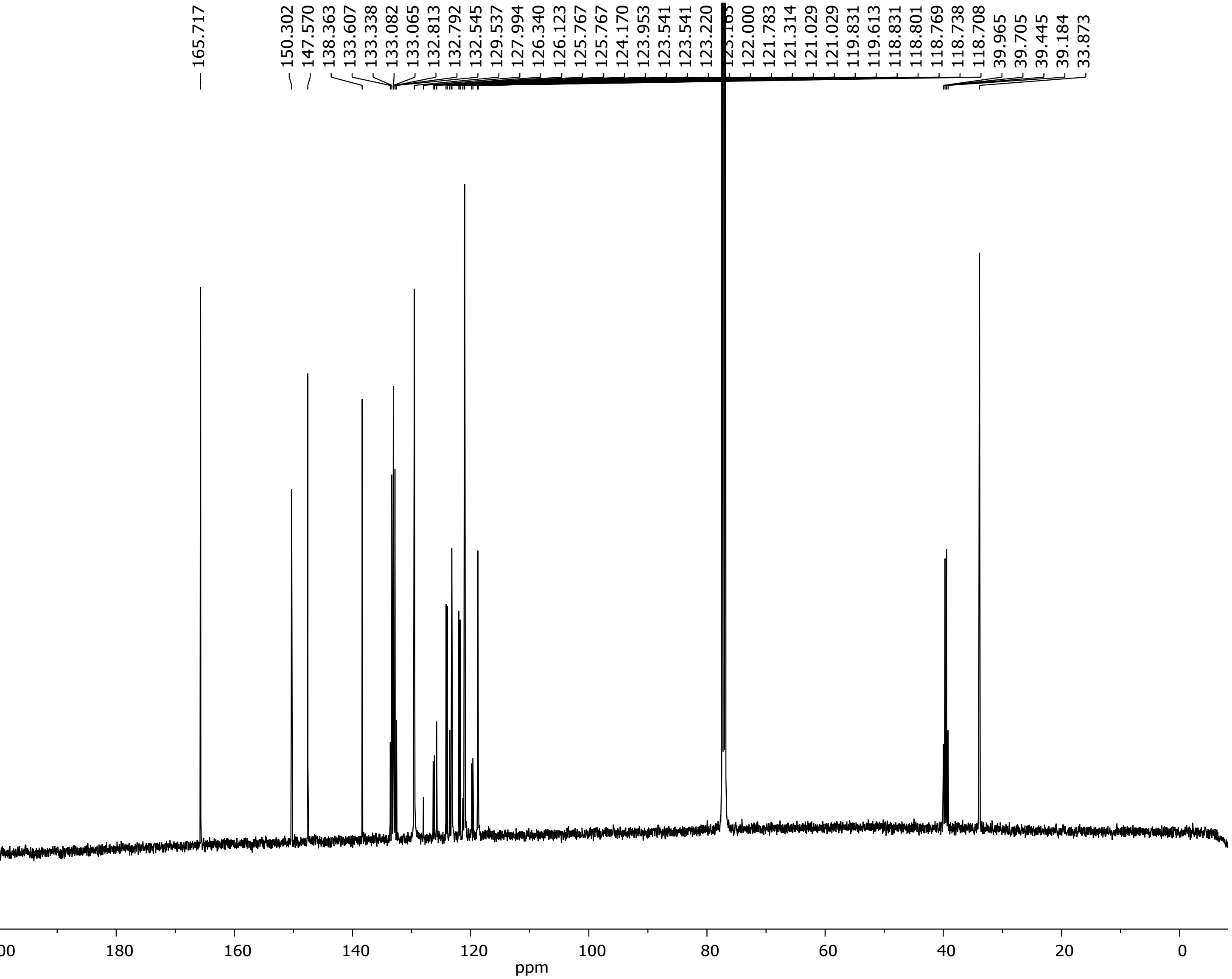

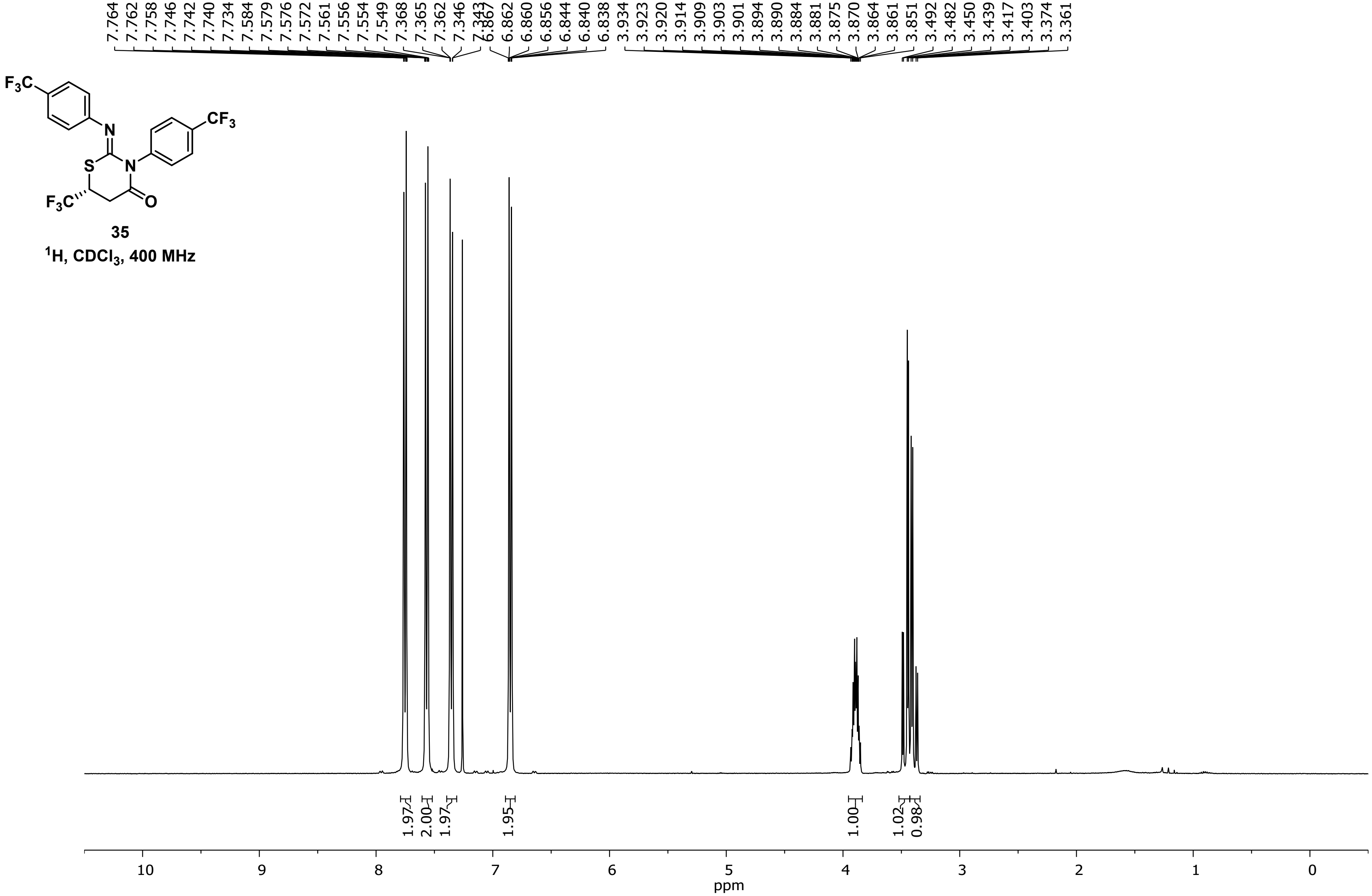

35

^{1}H, CDCl$_3$, 400 MHz

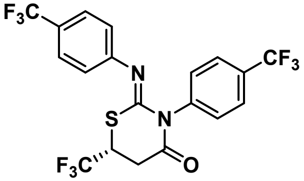

35

$^{19}F\{^{1}H\}$, $CDCl_3$, 376 MHz

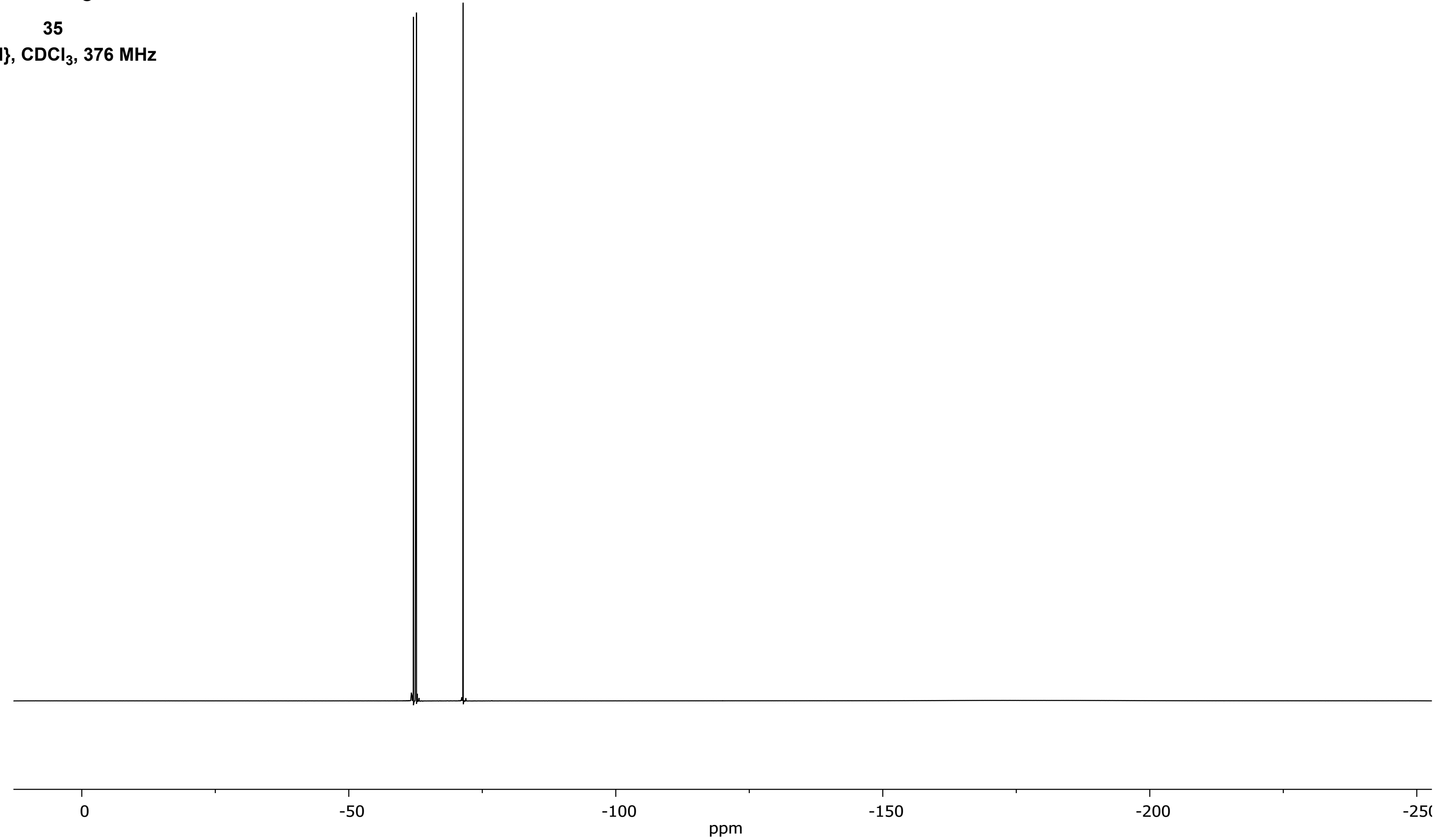

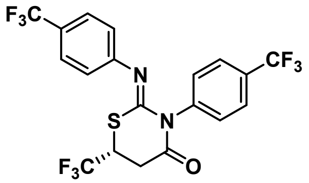

35

$^{13}C\{^1H\}$, $CDCl_3$, 126 MHz

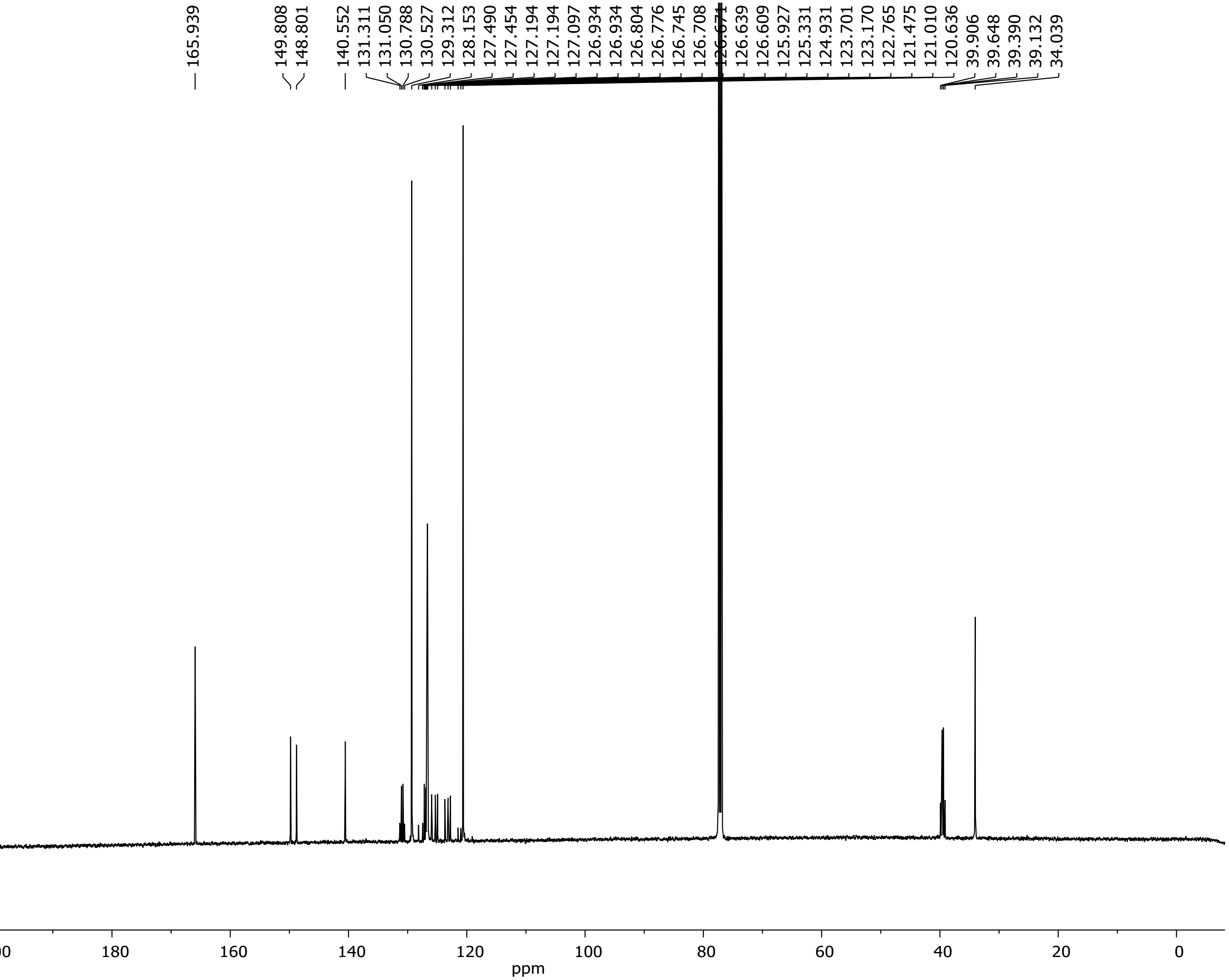

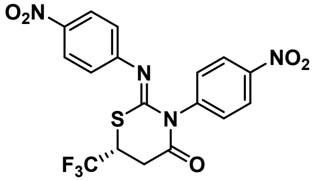

36

^{1}H, $CDCl_3$, 400 MHz

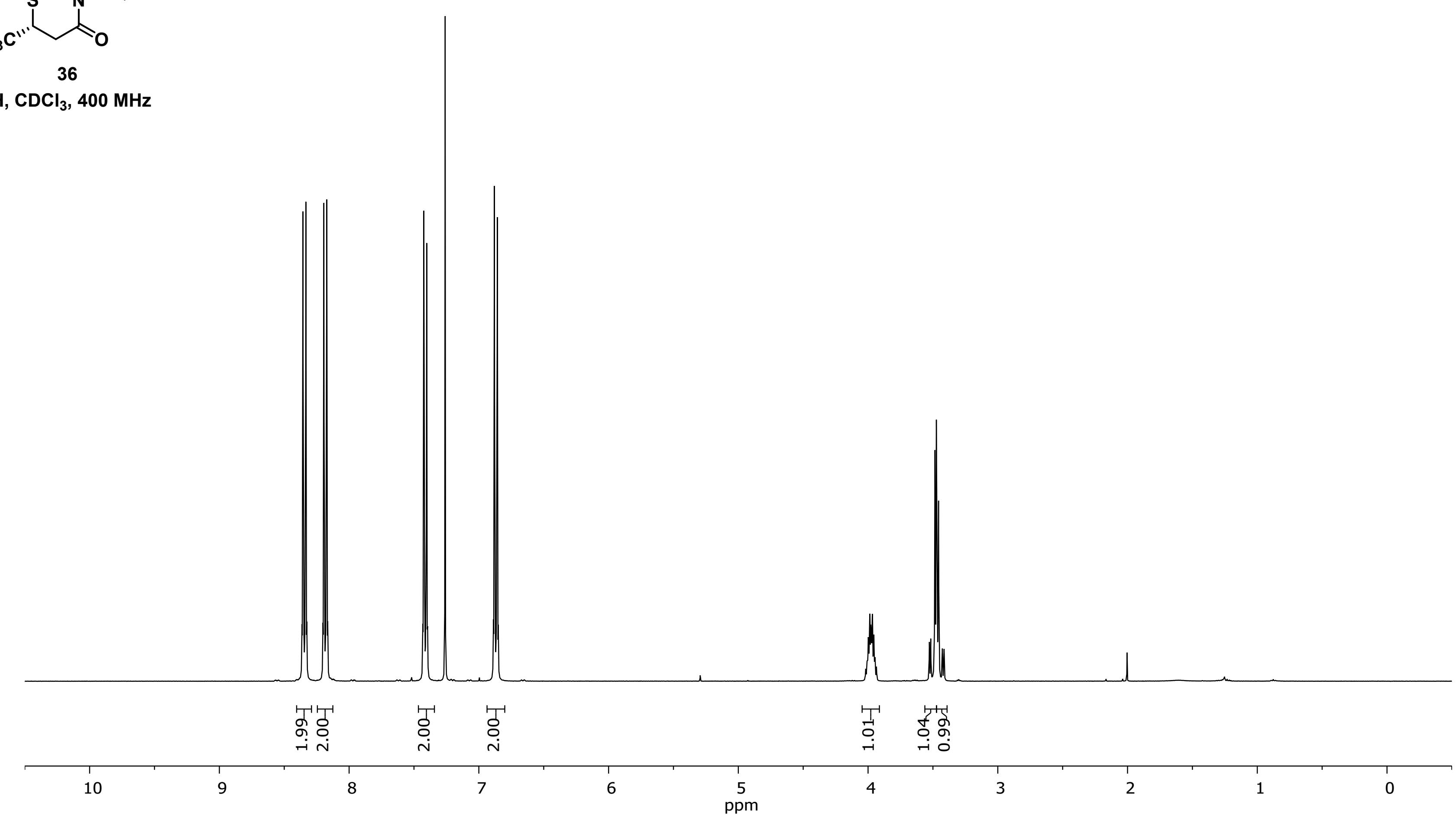

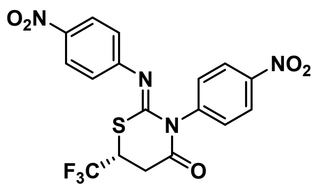

36

$^{19}F\{^{1}H\}$, $CDCl_3$, 377 MHz

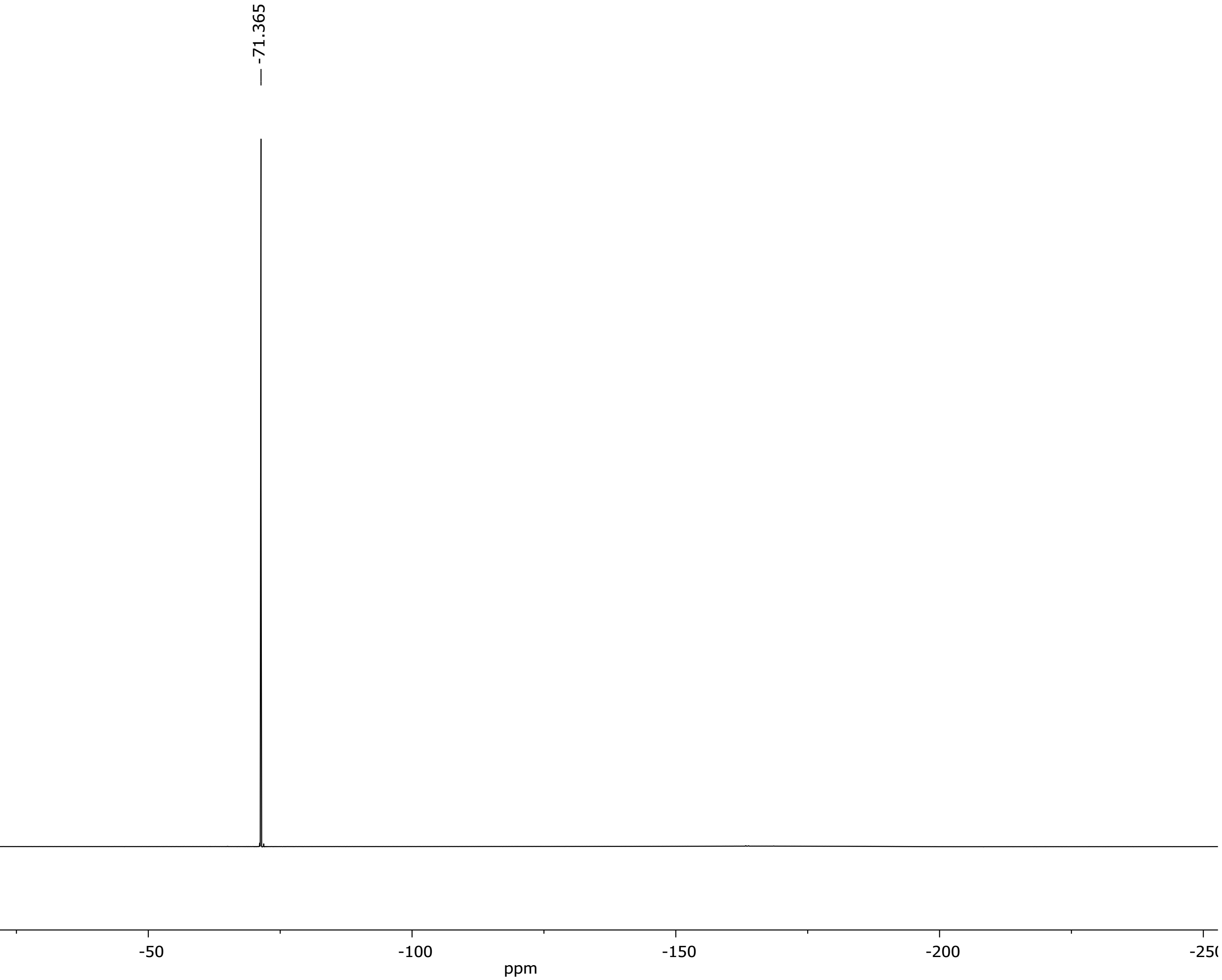

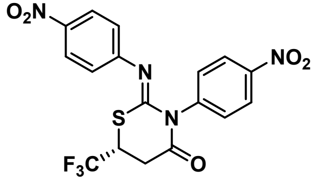

36

$^{13}C\{^{1}H\}$, $CDCl_3$, 126 MHz

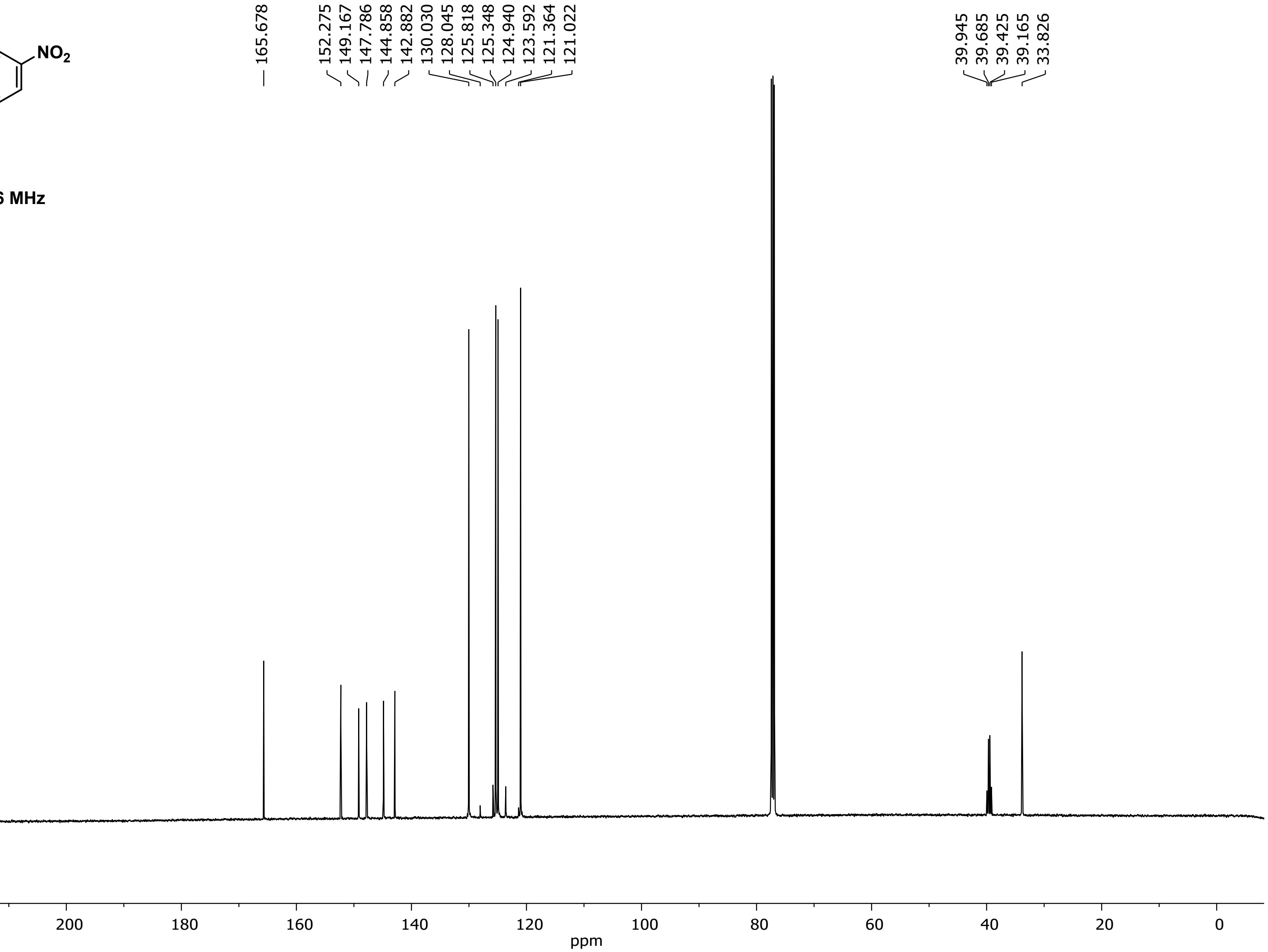

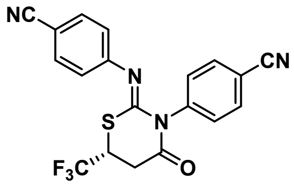

37

^{1}H, $CDCl_3$, 400 MHz

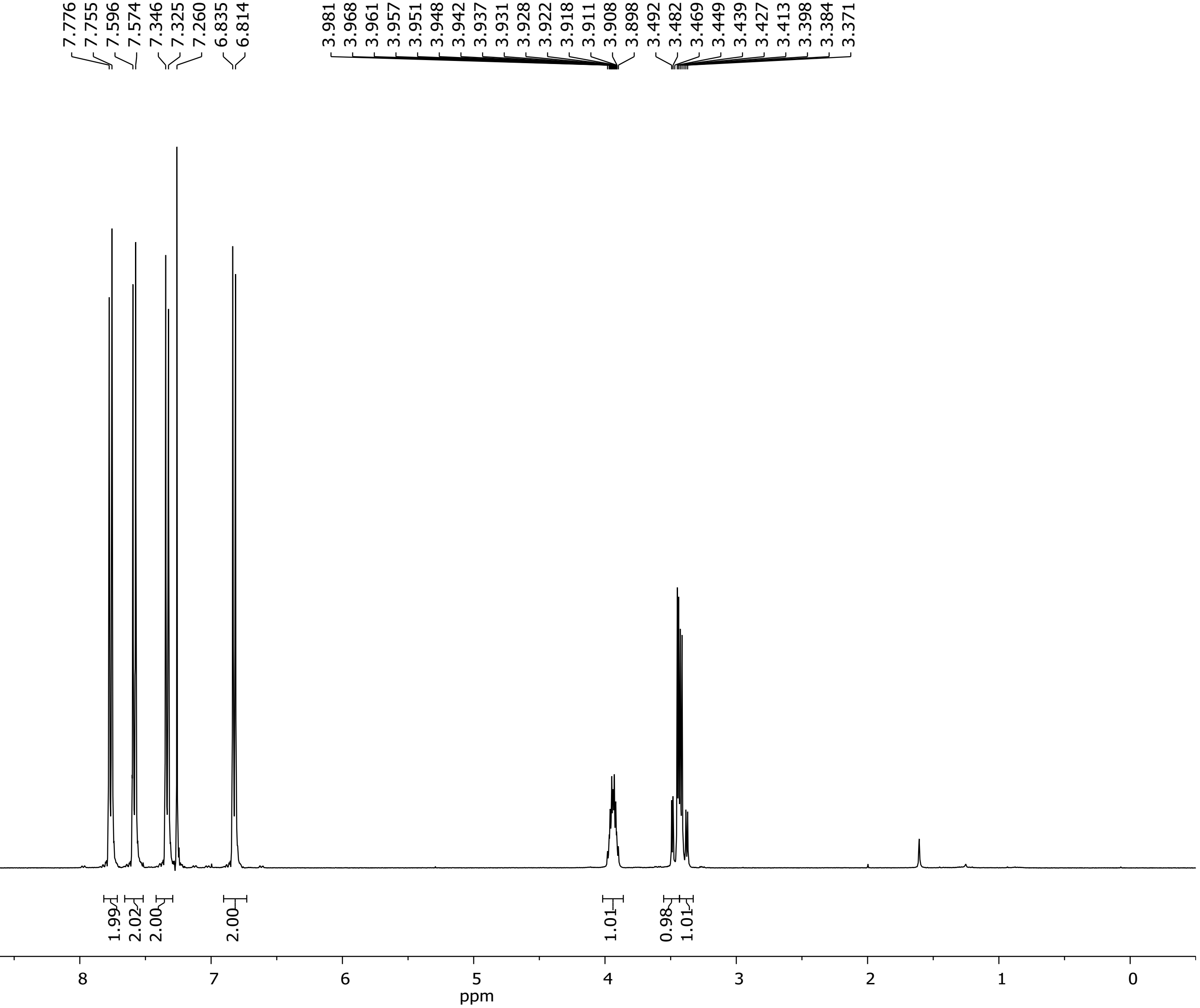

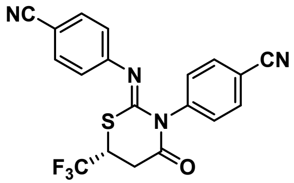

37

$^{19}F\{^{1}H\}$, $CDCl_3$, 376 MHz

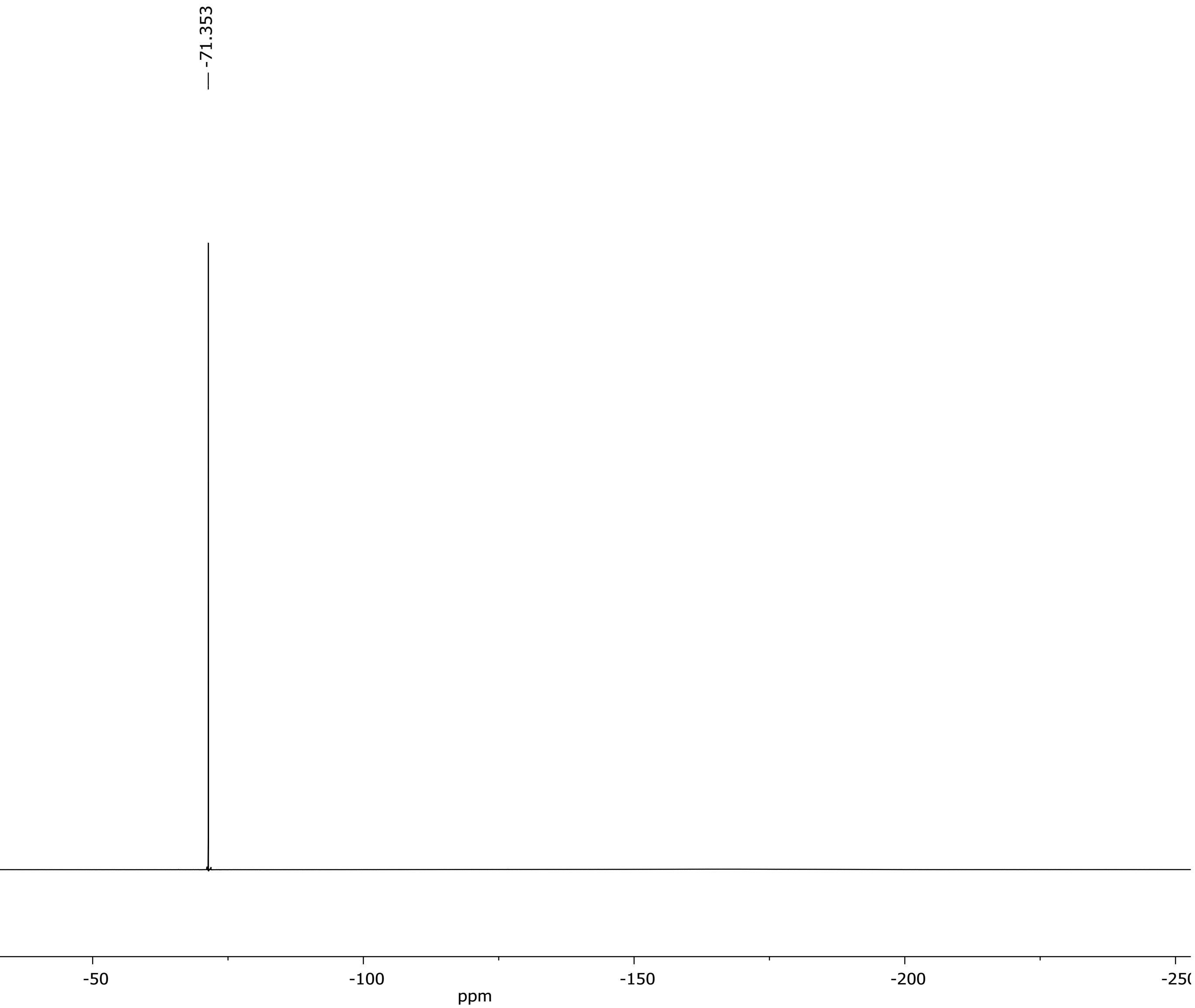

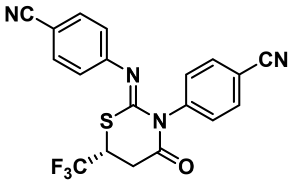

37

$^{13}C\{^1H\}$, $CDCl_3$, 126 MHz

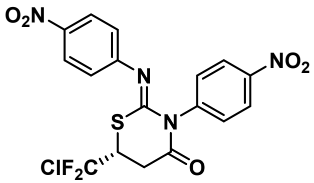

38

^1H, $CDCl_3$, 500 MHz

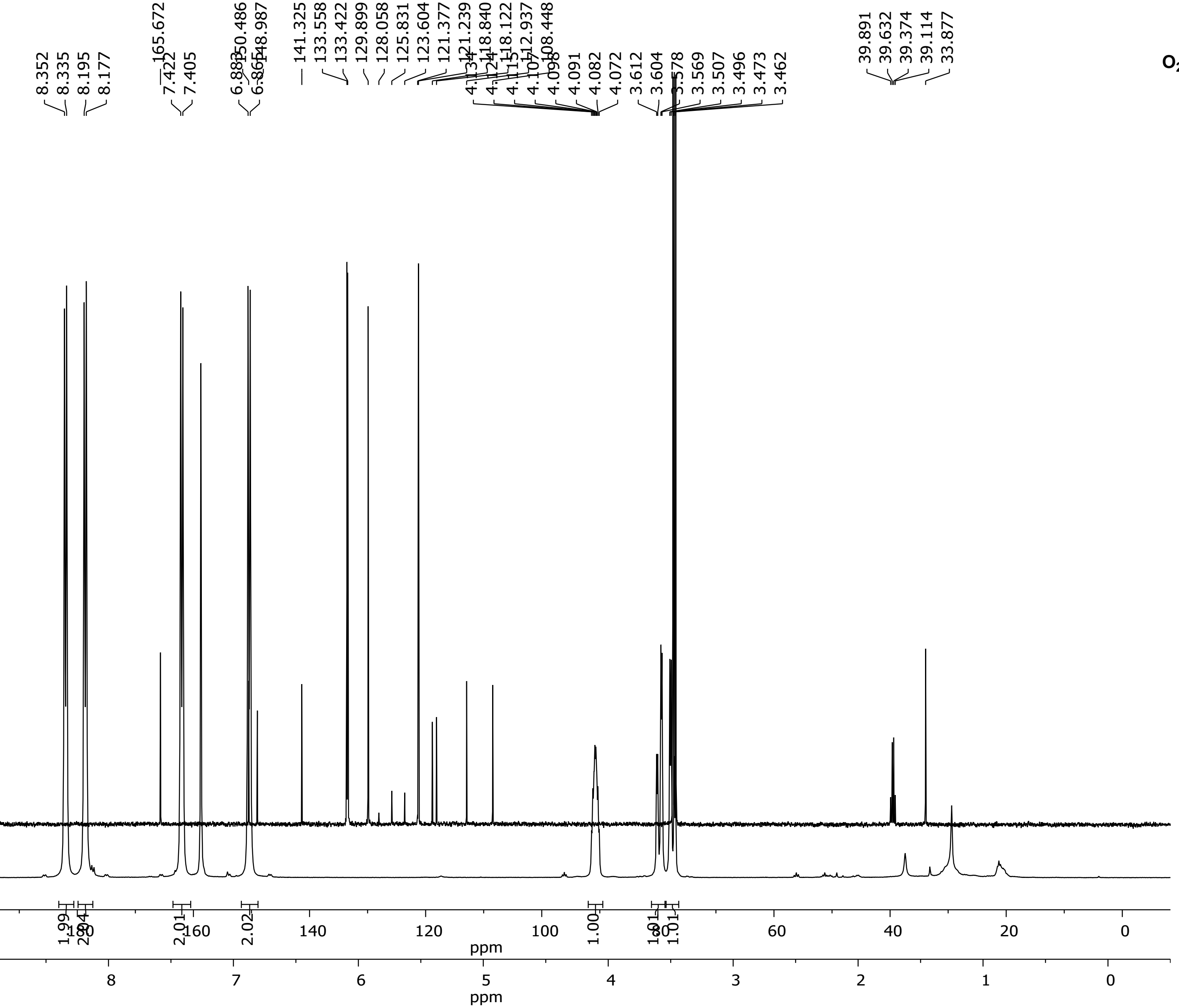

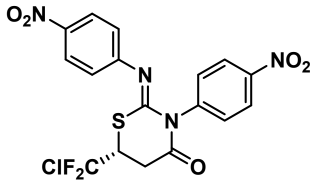

38

$^{19}F\{^1H\}$, $CDCl_3$, 377 MHz

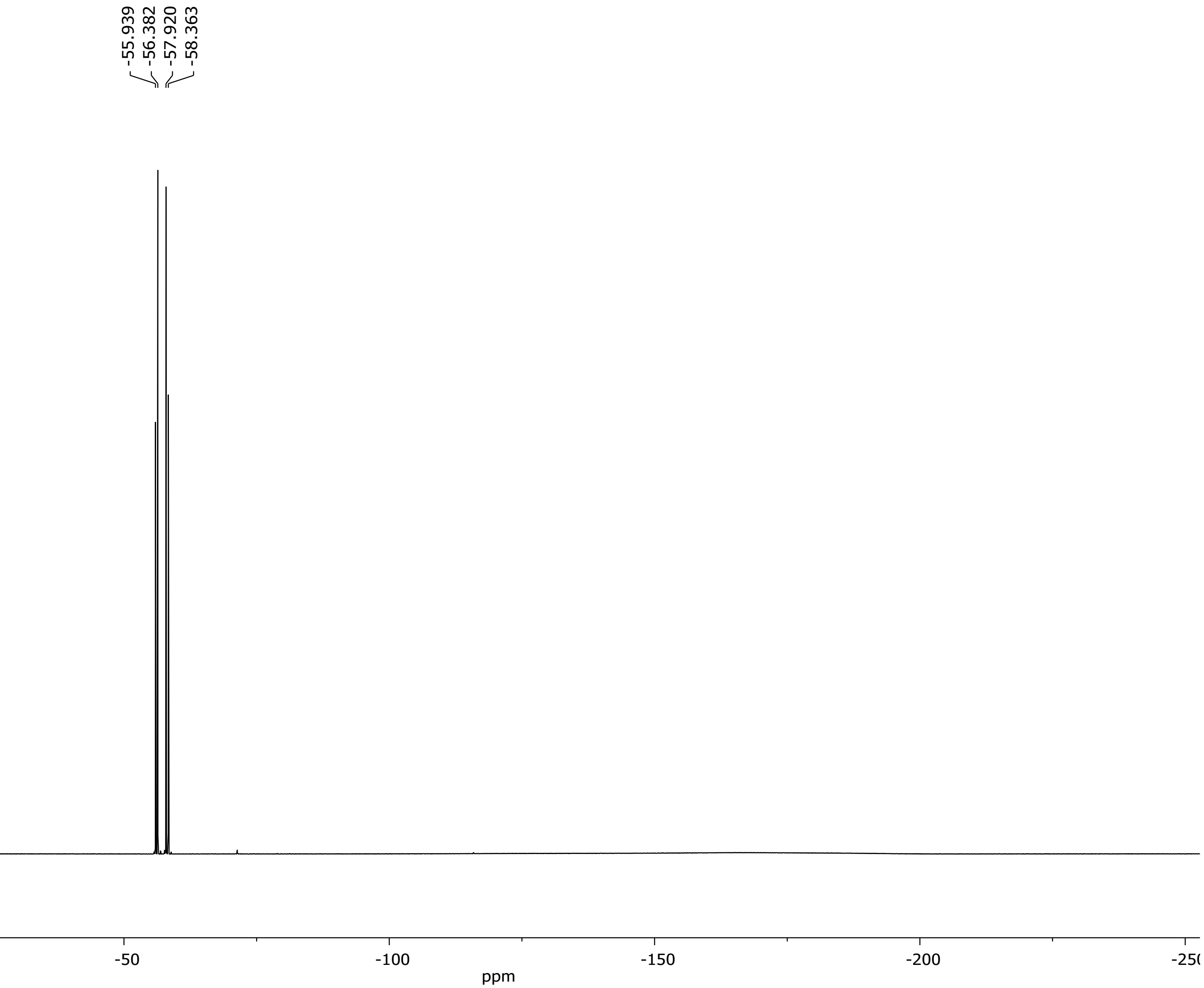

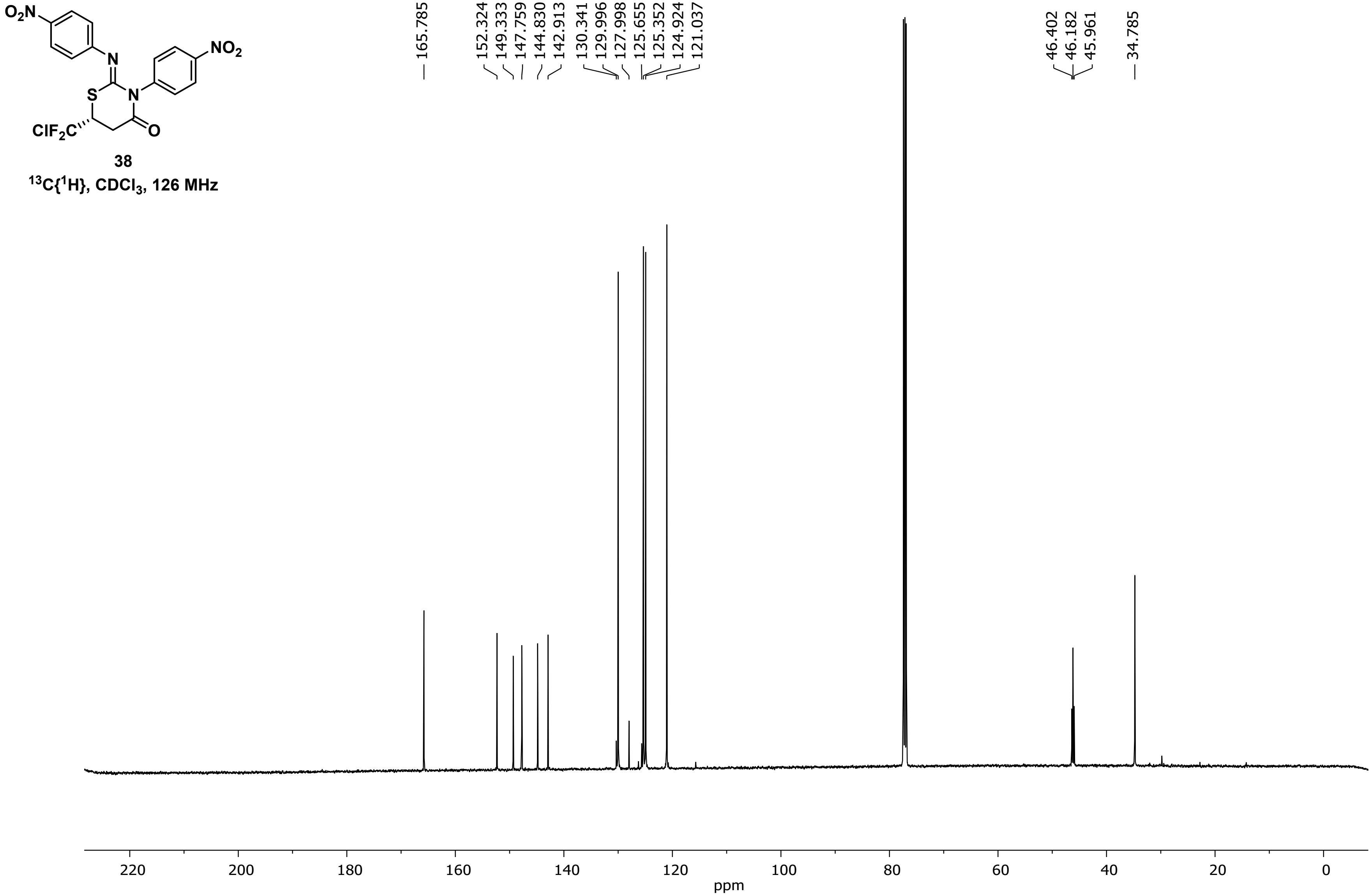

38

$^{13}C\{^1H\}$, $CDCl_3$, 126 MHz

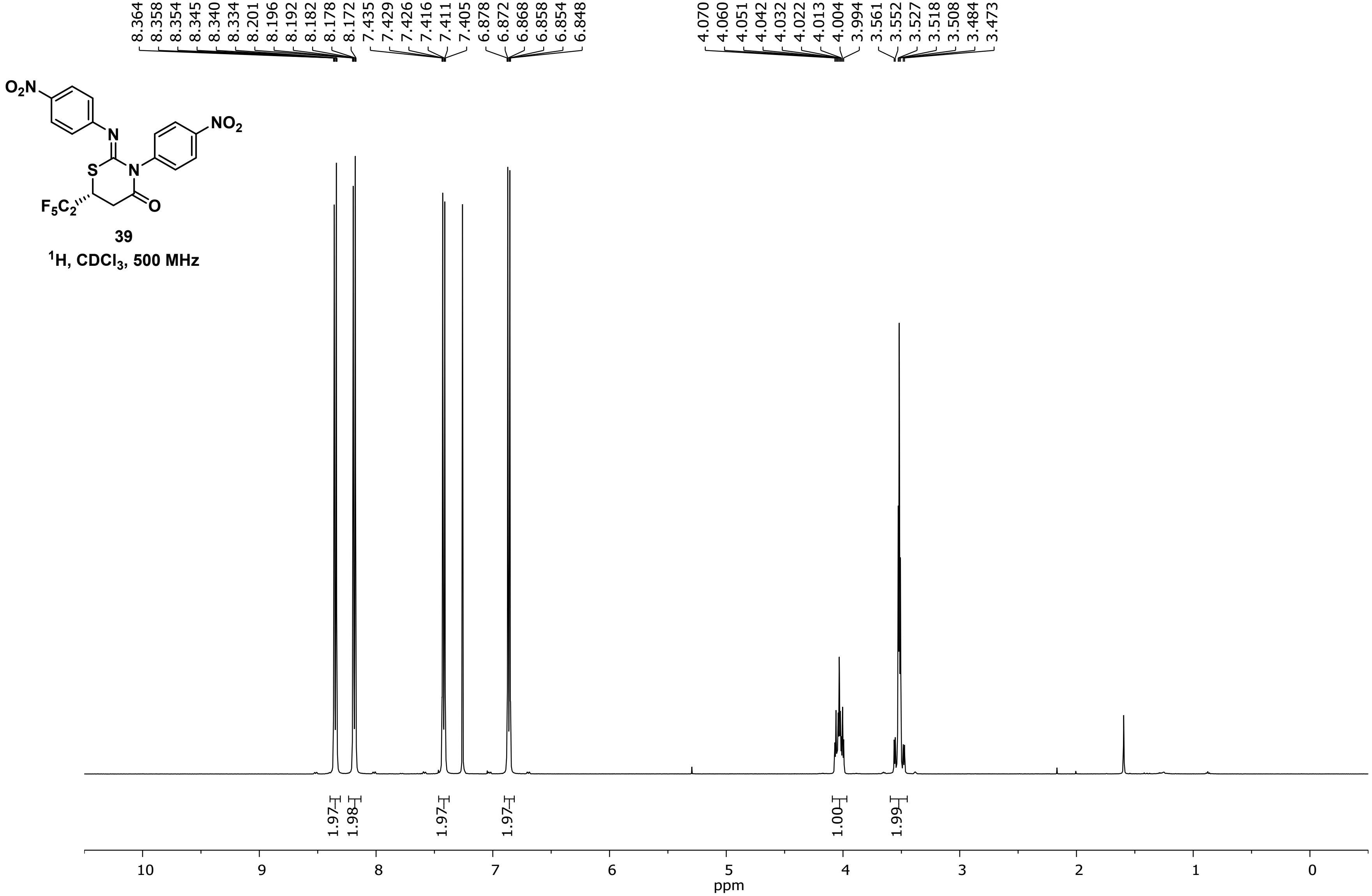
O2N
NO2
N
S
N
F5C2
O
39
1H, CDCl3, 500 MHz
8.364 8.358 8.354 8.345 8.340 8.334 8.201 8.196 8.192 8.182 8.178 8.172 7.435 7.429 7.426 7.416 7.411 7.405 6.878 6.872 6.868 6.858 6.854 6.848
4.070 4.060 4.051 4.042 4.032 4.022 4.013 4.004 3.994 3.561 3.552 3.527 3.518 3.508 3.484 3.473
1.97 1.98 1.97 1.97 1.00 1.99
10 9 8 7 6 5 4 3 2 1 0
ppm

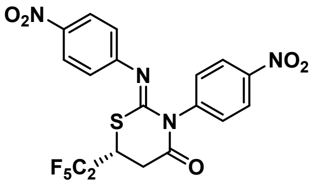

39

$^{19}F\{^1H\}$, $CDCl_3$, 377 MHz

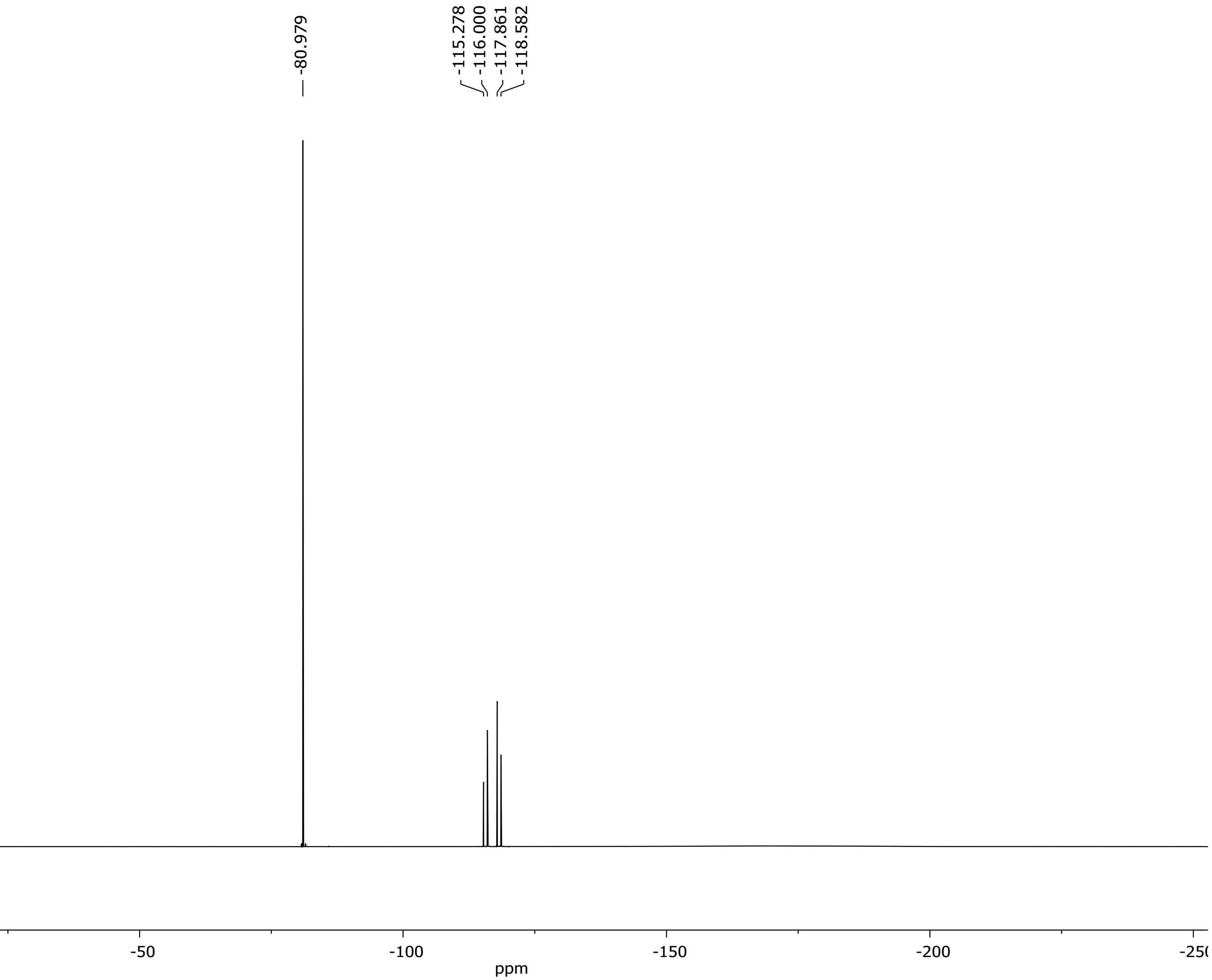

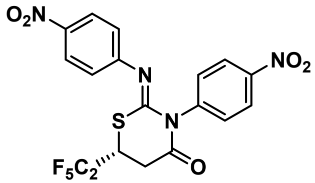

39

$^{13}C\{^1H\}$, $CDCl_3$, 126 MHz

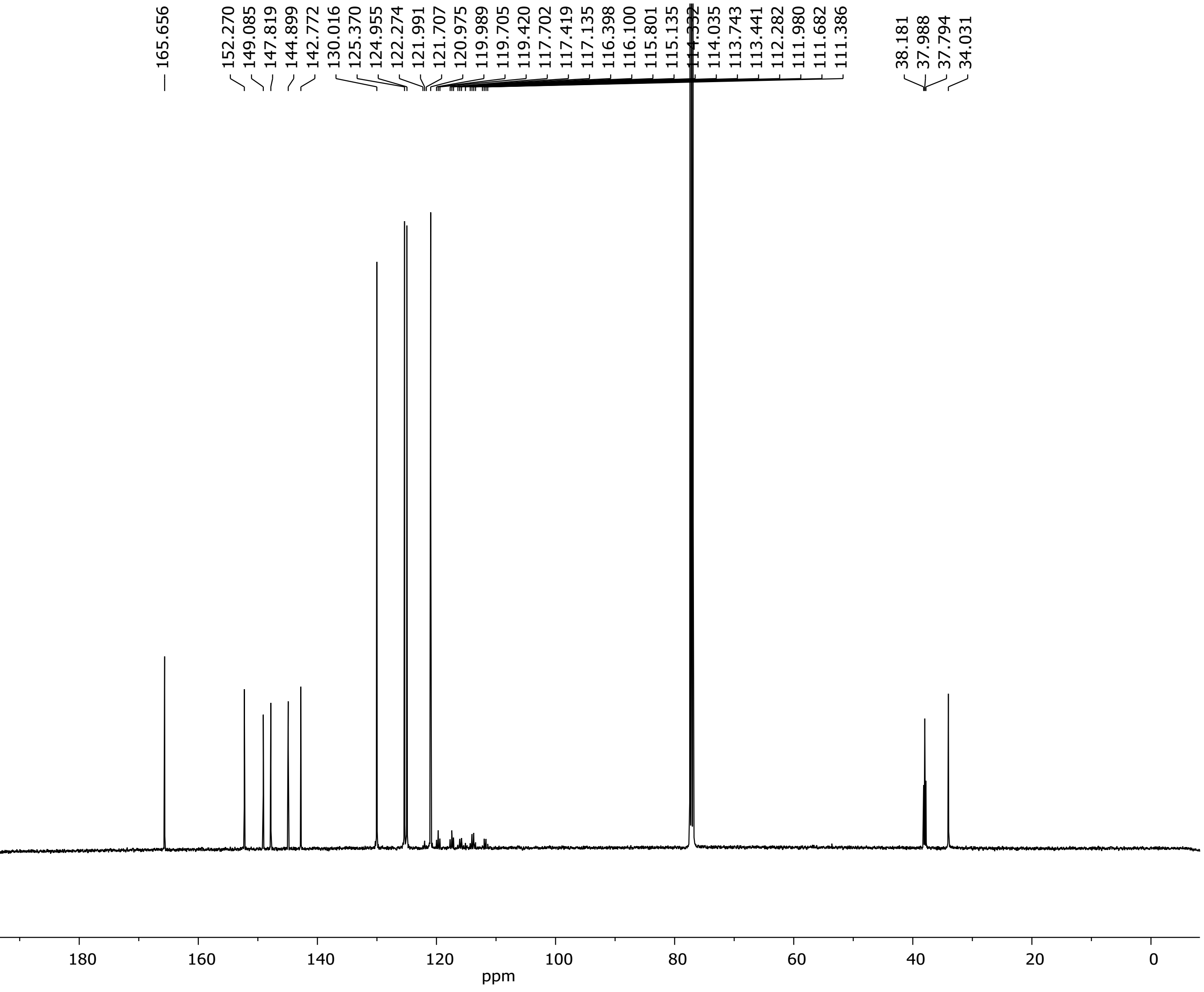

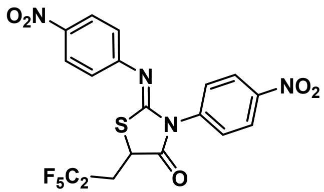

S40
^{1}H, $CDCl_3$, 500 MHz

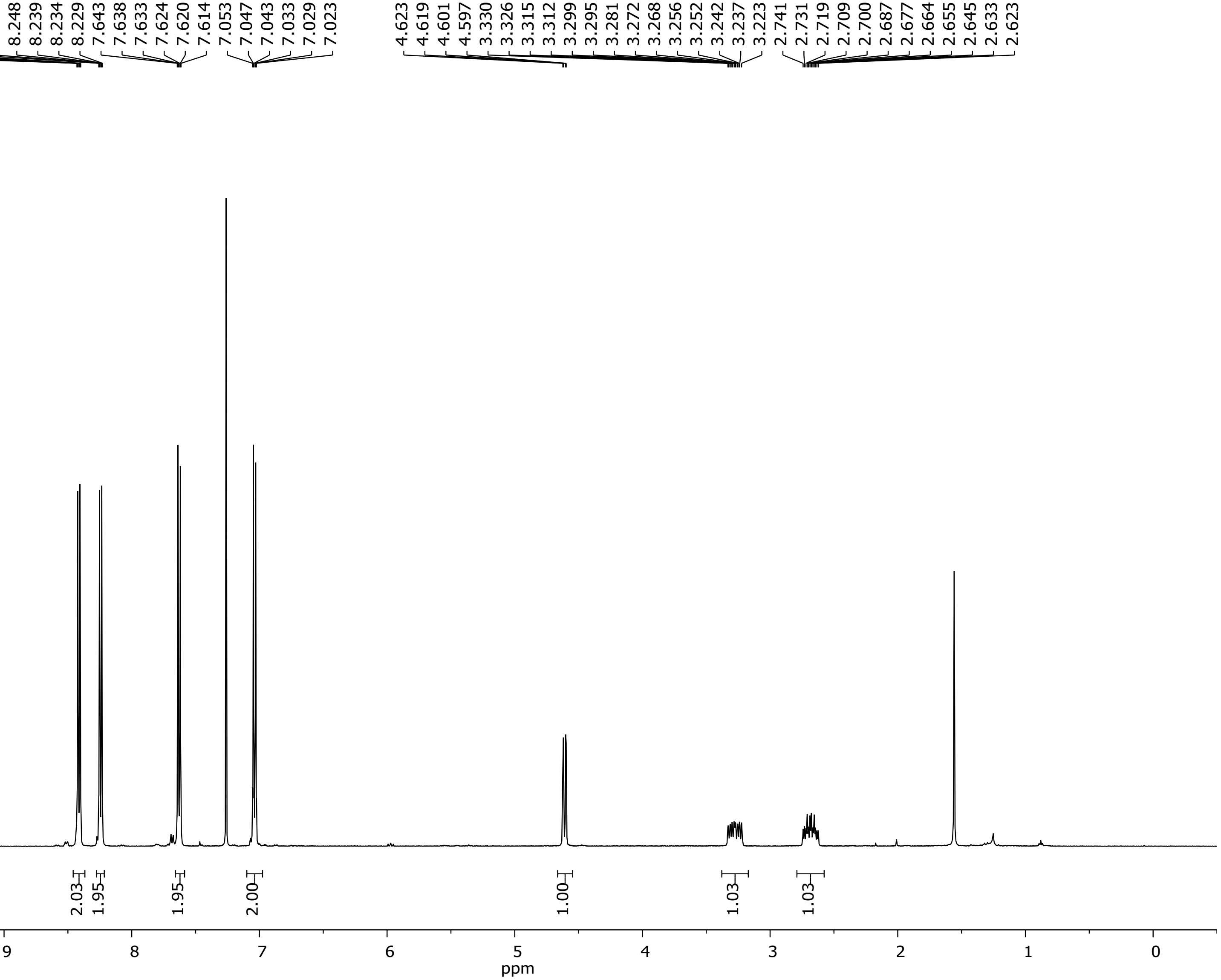

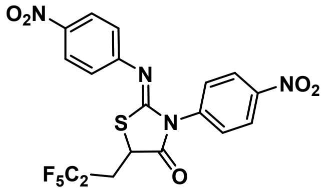

S40

$^{19}F\{^{1}H\}$, $CDCl_3$, 376 MHz

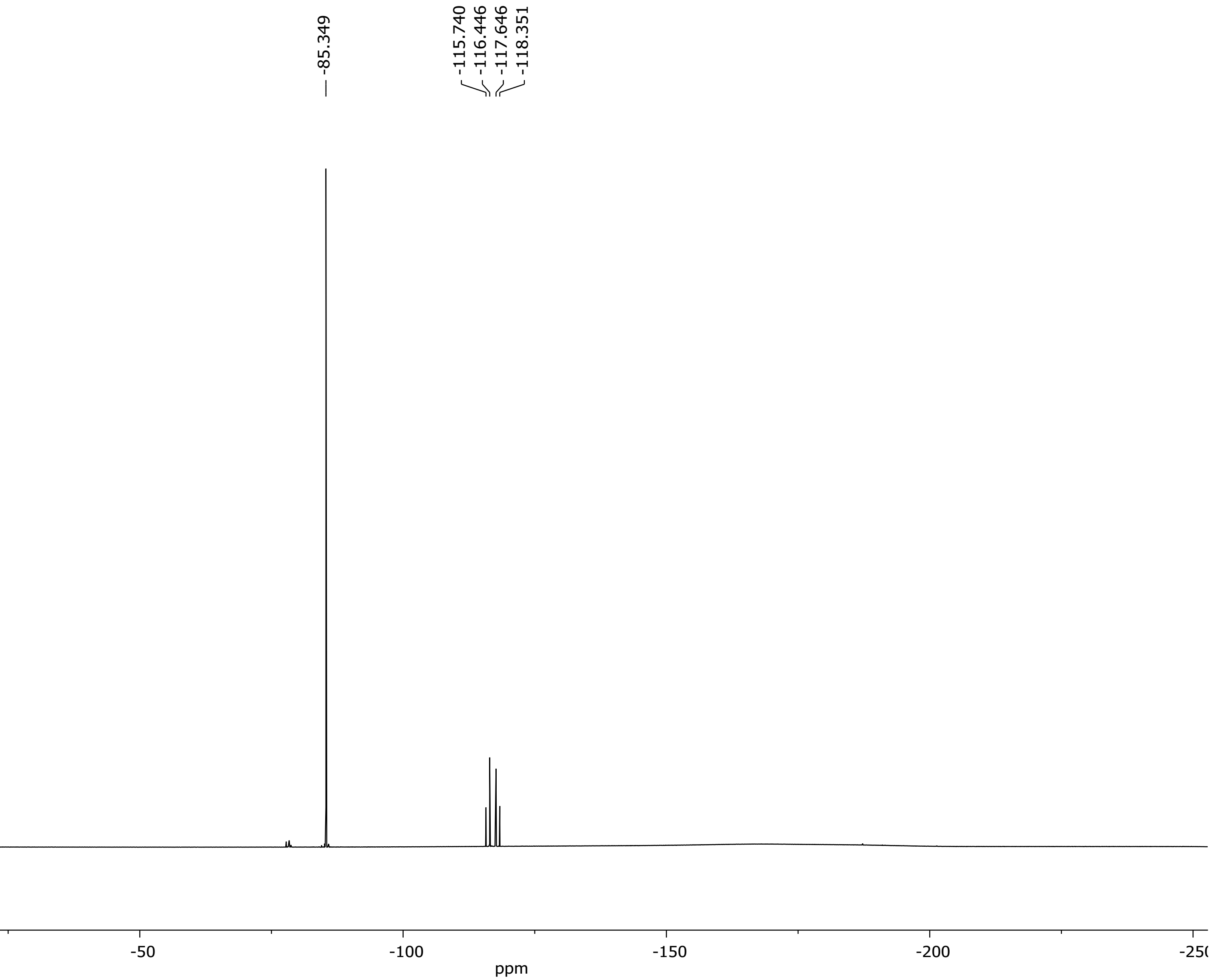

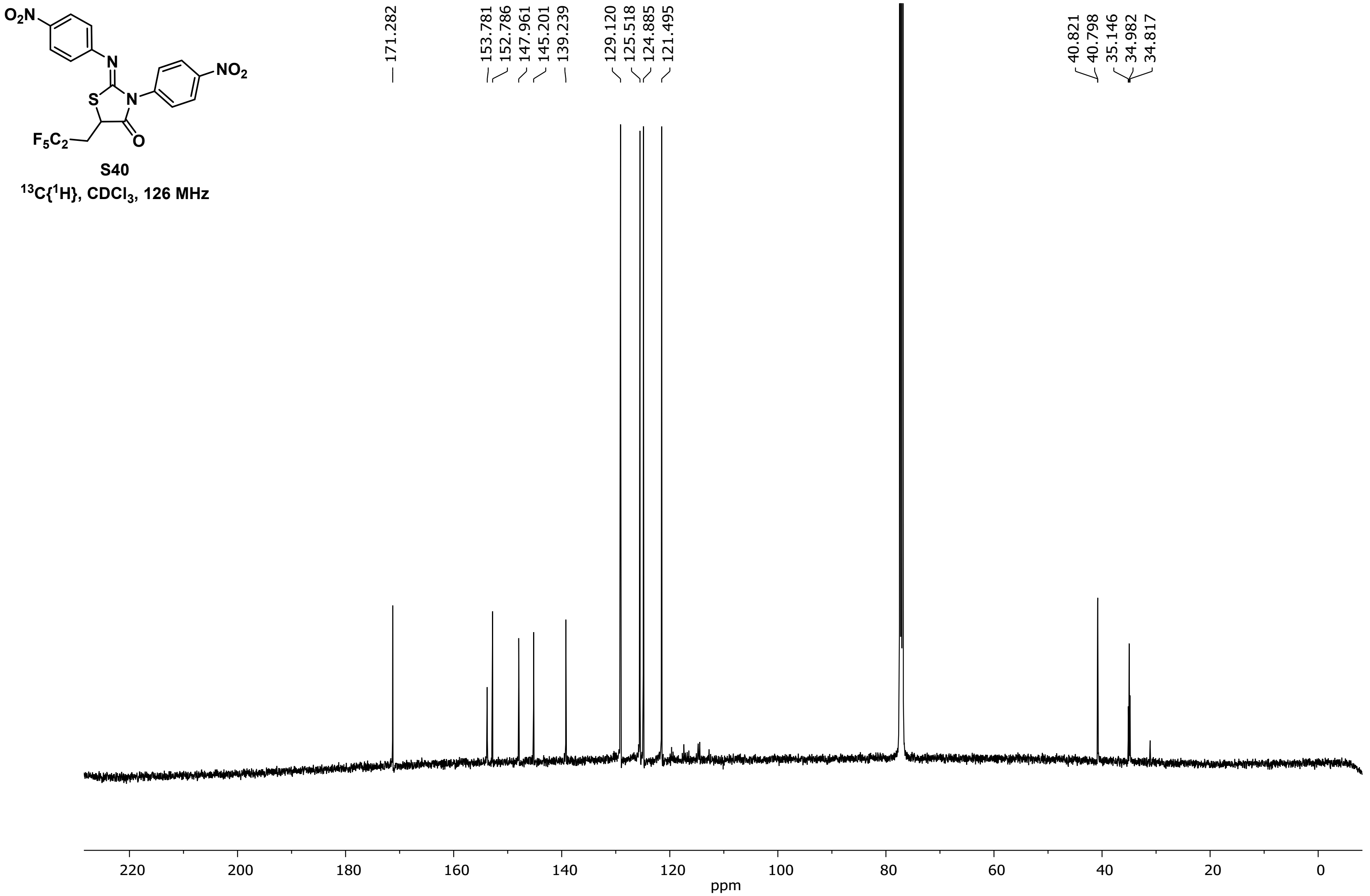

O2N
N
NO2
S
N
F5C2
O
S40
13C{1H}, CDCl3, 126 MHz
171.282
153.781
152.786
147.961
145.201
139.239
129.120
125.518
124.885
121.495
40.821
40.798
35.146
34.982
34.817
220
200
180
160
140
120
100
80
60
40
20
0
ppm

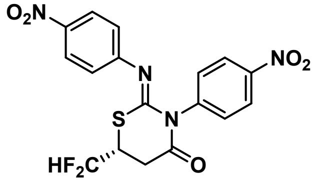

40

^{1}H, $CDCl_3$, 500 MHz

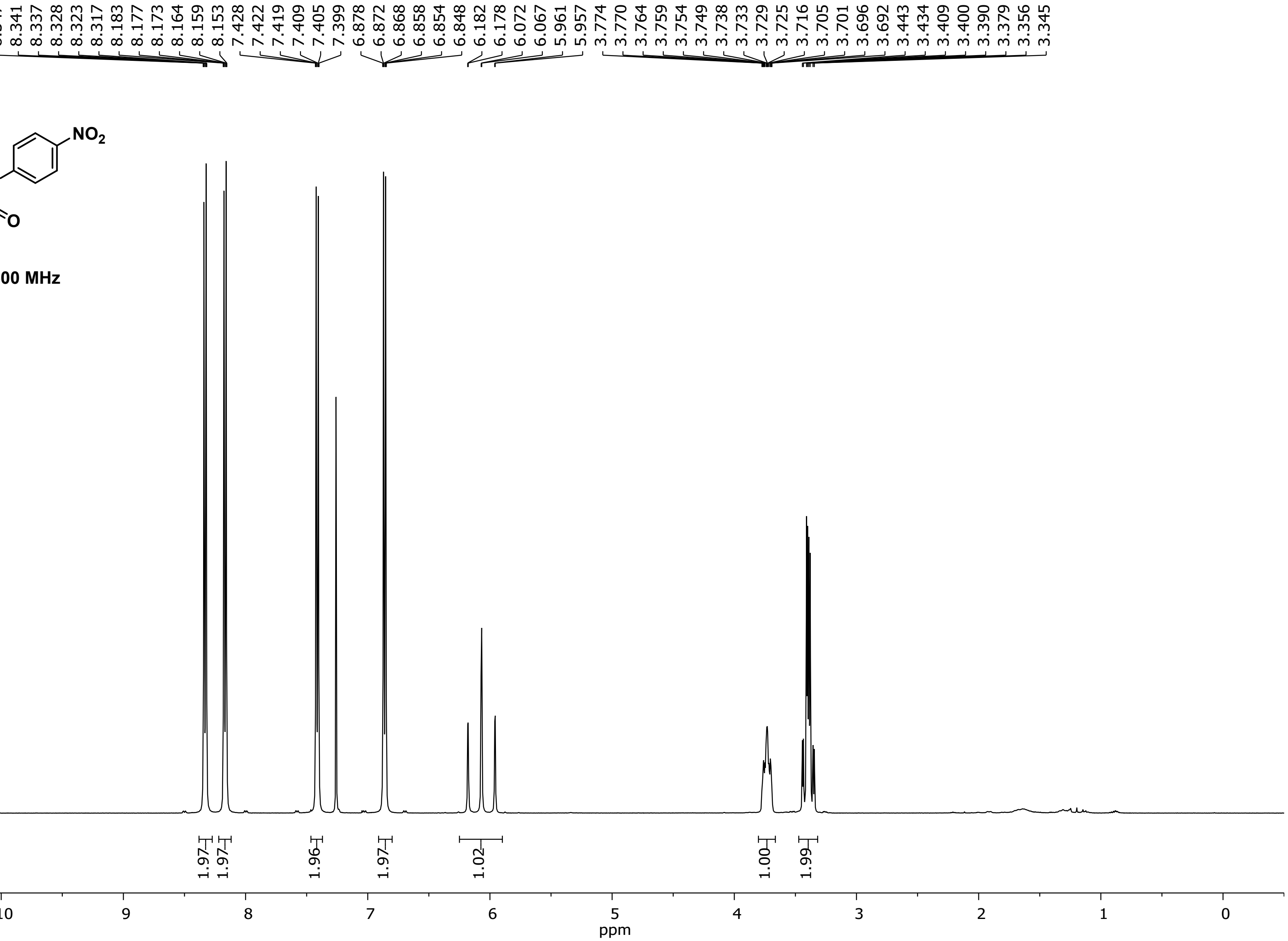

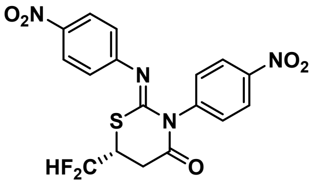

40
$^{19}F\{^1H\}$, $CDCl_3$, 376 MHz

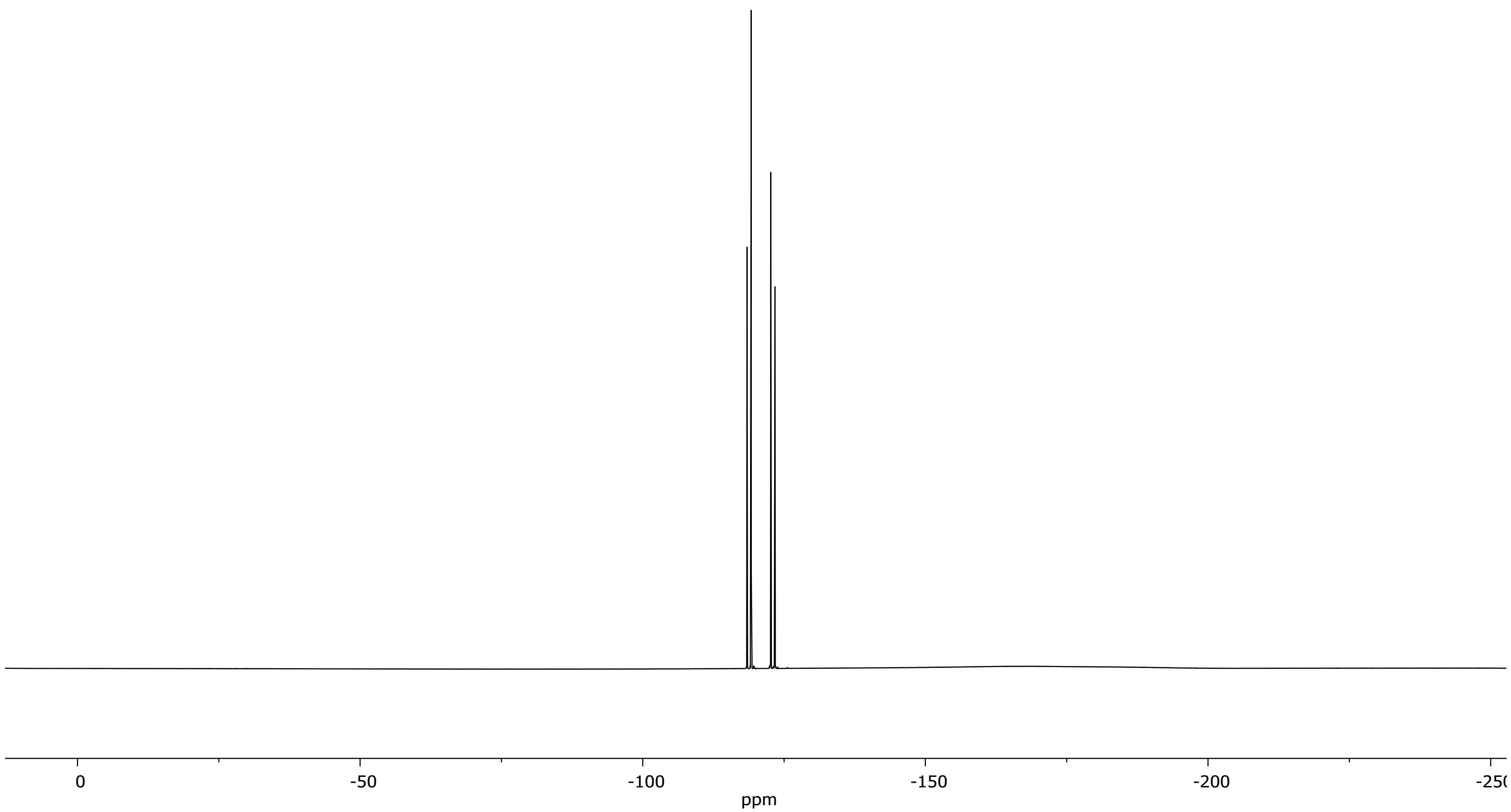

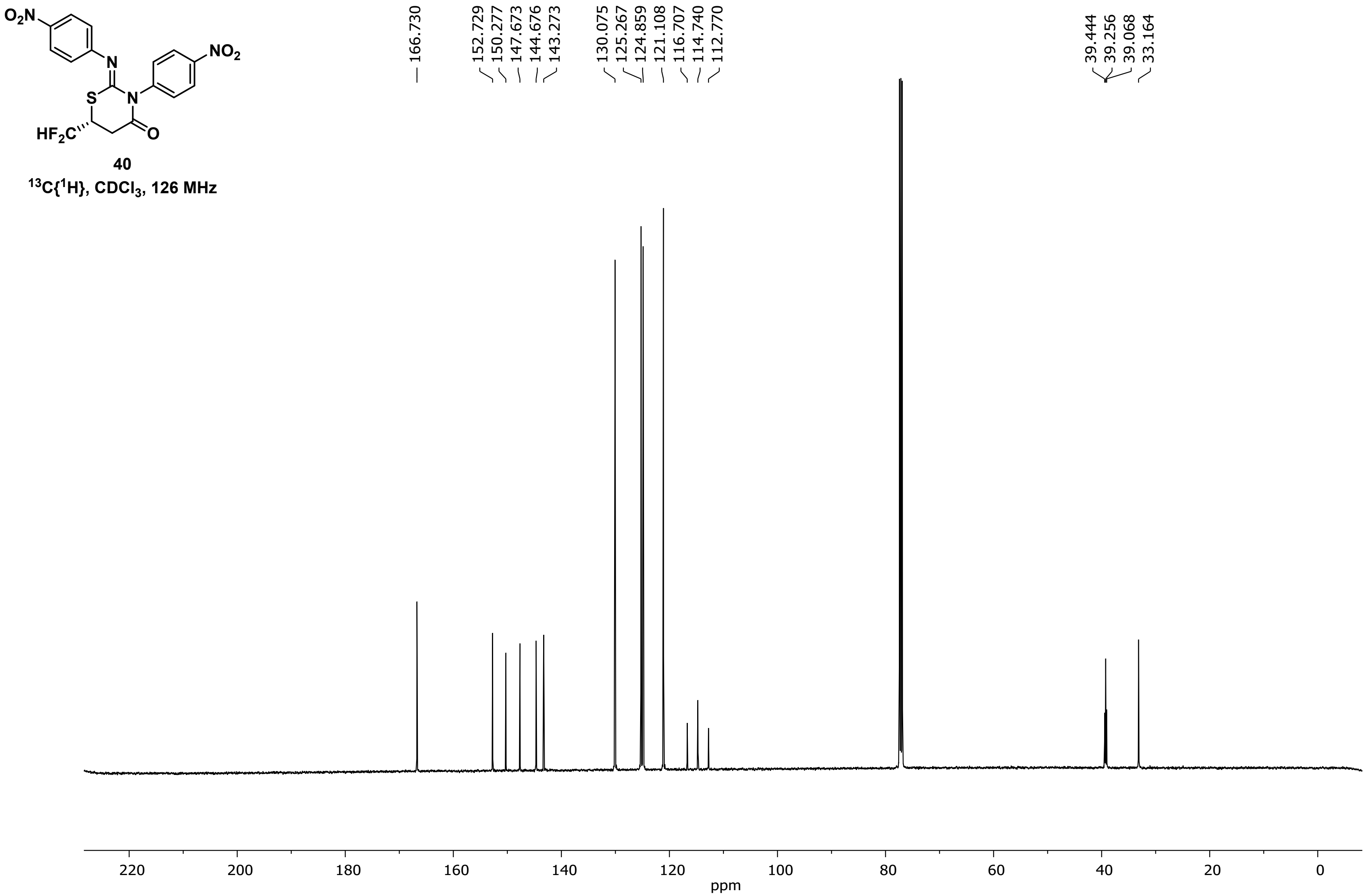

O2N
NO2
N
S
N
HF2C
O
40
13C{1H}, CDCl3, 126 MHz
166.730
152.729
150.277
147.673
144.676
143.273
130.075
125.267
124.859
121.108
116.707
114.740
112.770
39.444
39.256
39.068
33.164
220
200
180
160
140
120
100
80
60
40
20
0
ppm

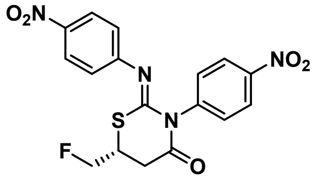

41

^{1}H, $(CD_3)_2CO$, 400 MHz

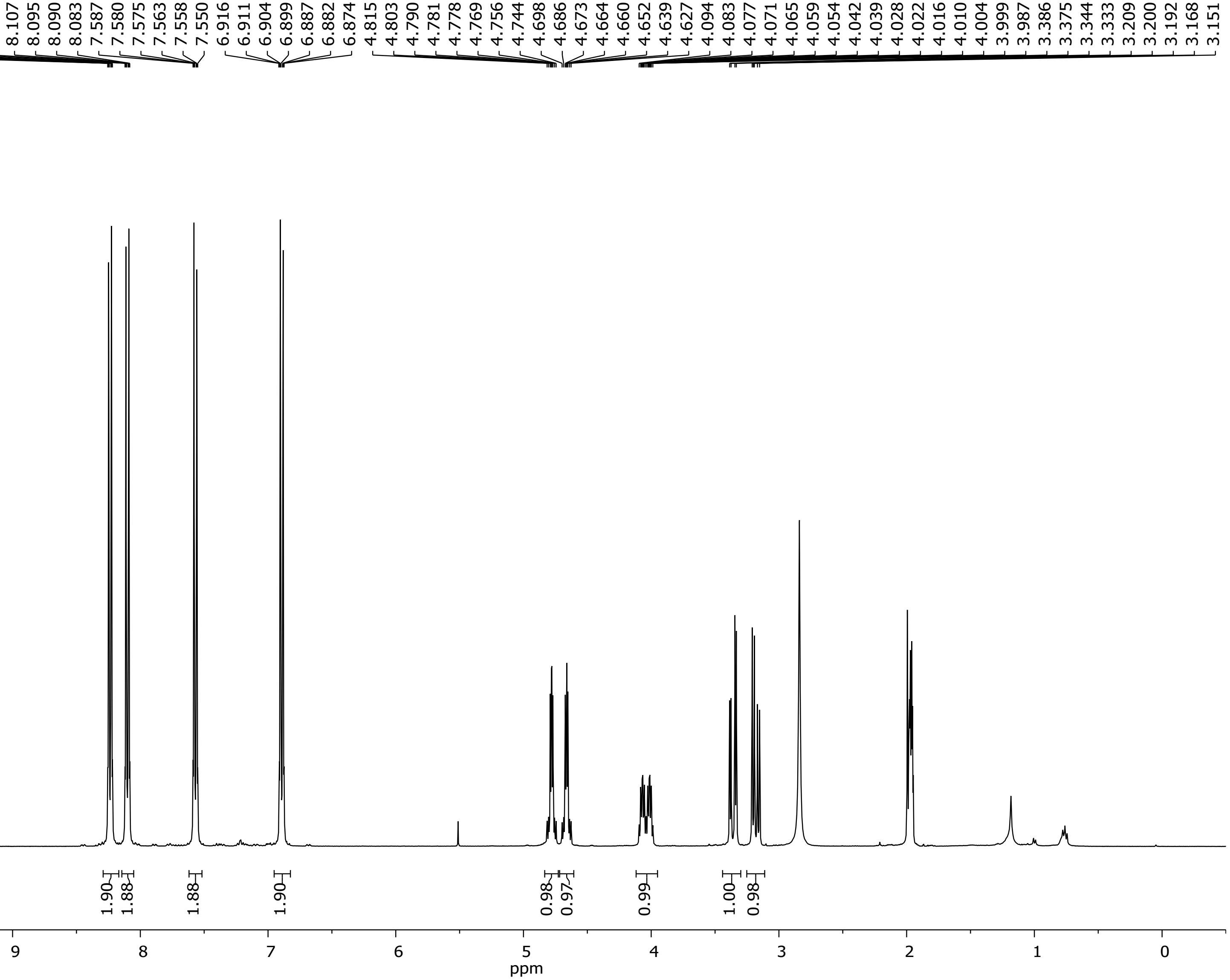

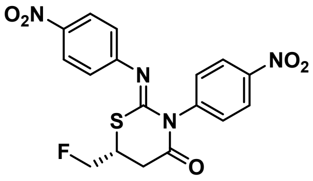

41

$^{19}F\{^1H\}$, $(CD_3)_2CO$, 376 MHz

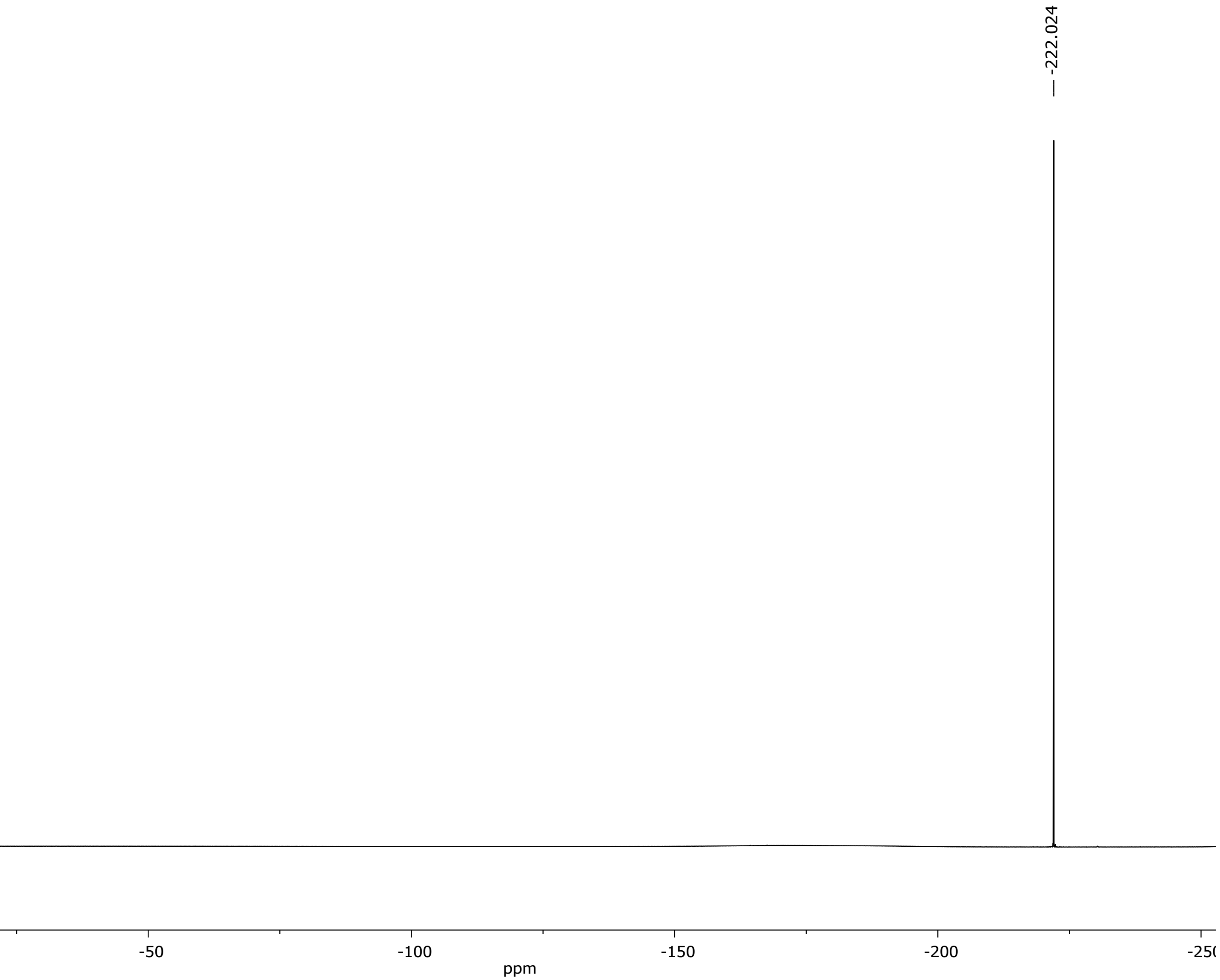

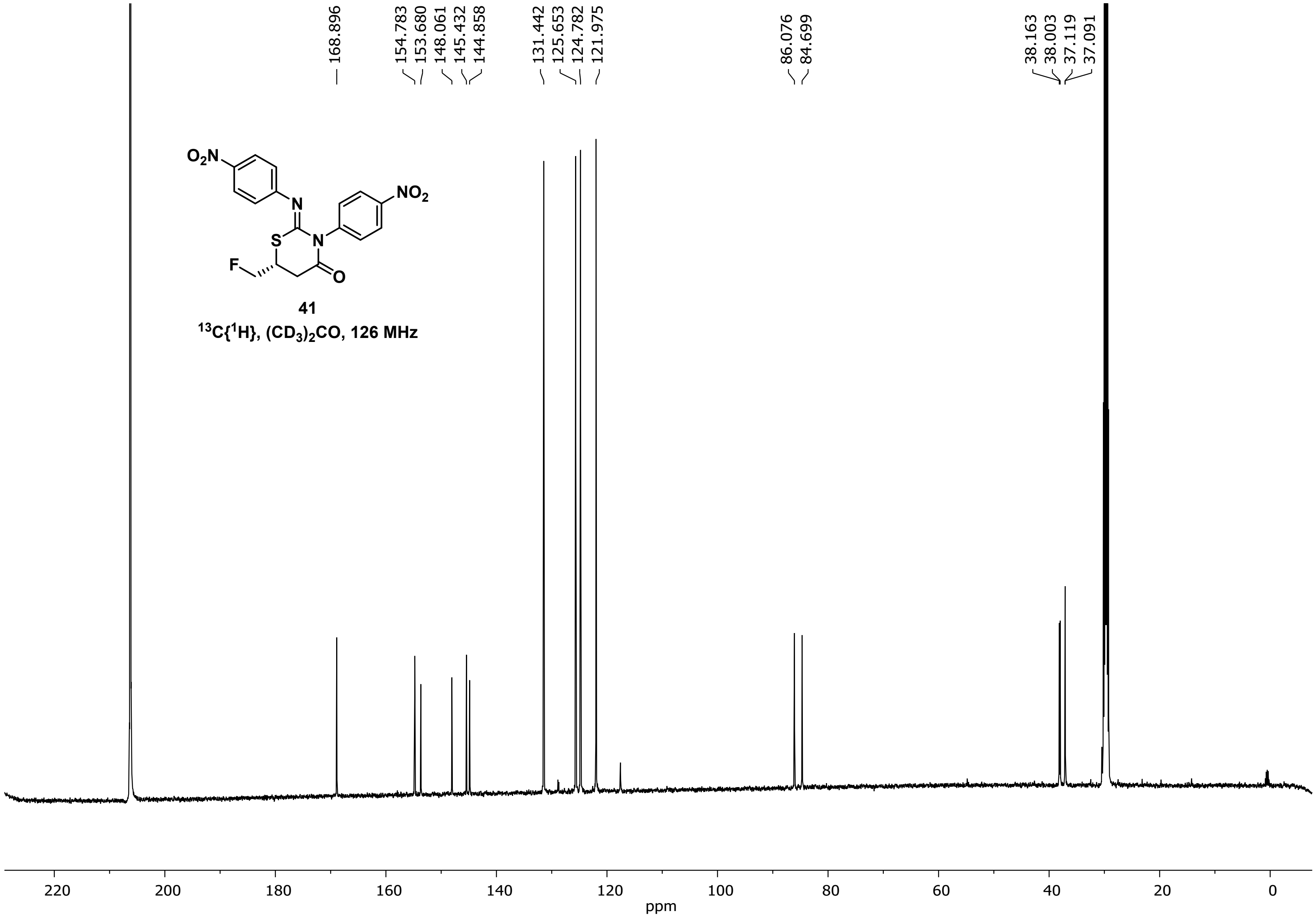

168.896
154.783
153.680
148.061
145.432
144.858
131.442
125.653
124.782
121.975
86.076
84.699
38.163
38.003
37.119
37.091
O2N
NO2
N
S
N
F
O
41
13C{1H}, (CD3)2CO, 126 MHz
220
200
180
160
140
120
100
80
60
40
20
0
ppm

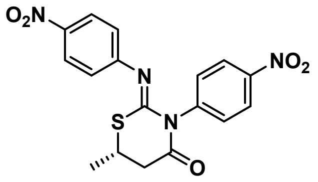

42

^{1}H, CD_3CN, 400 MHz

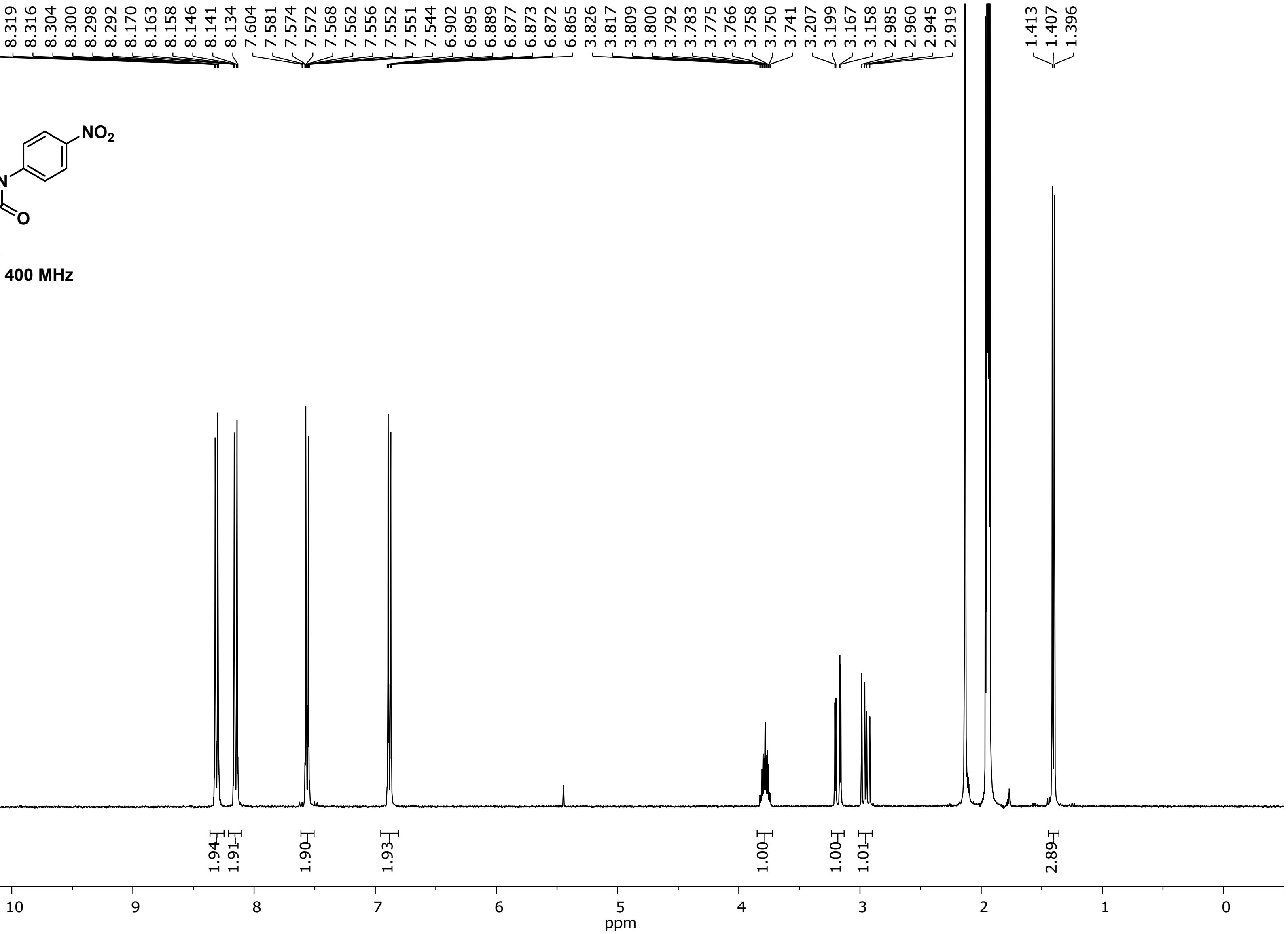

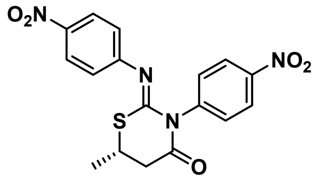

42

$^{13}C\{^1H\}$, CD_3CN, 126 MHz

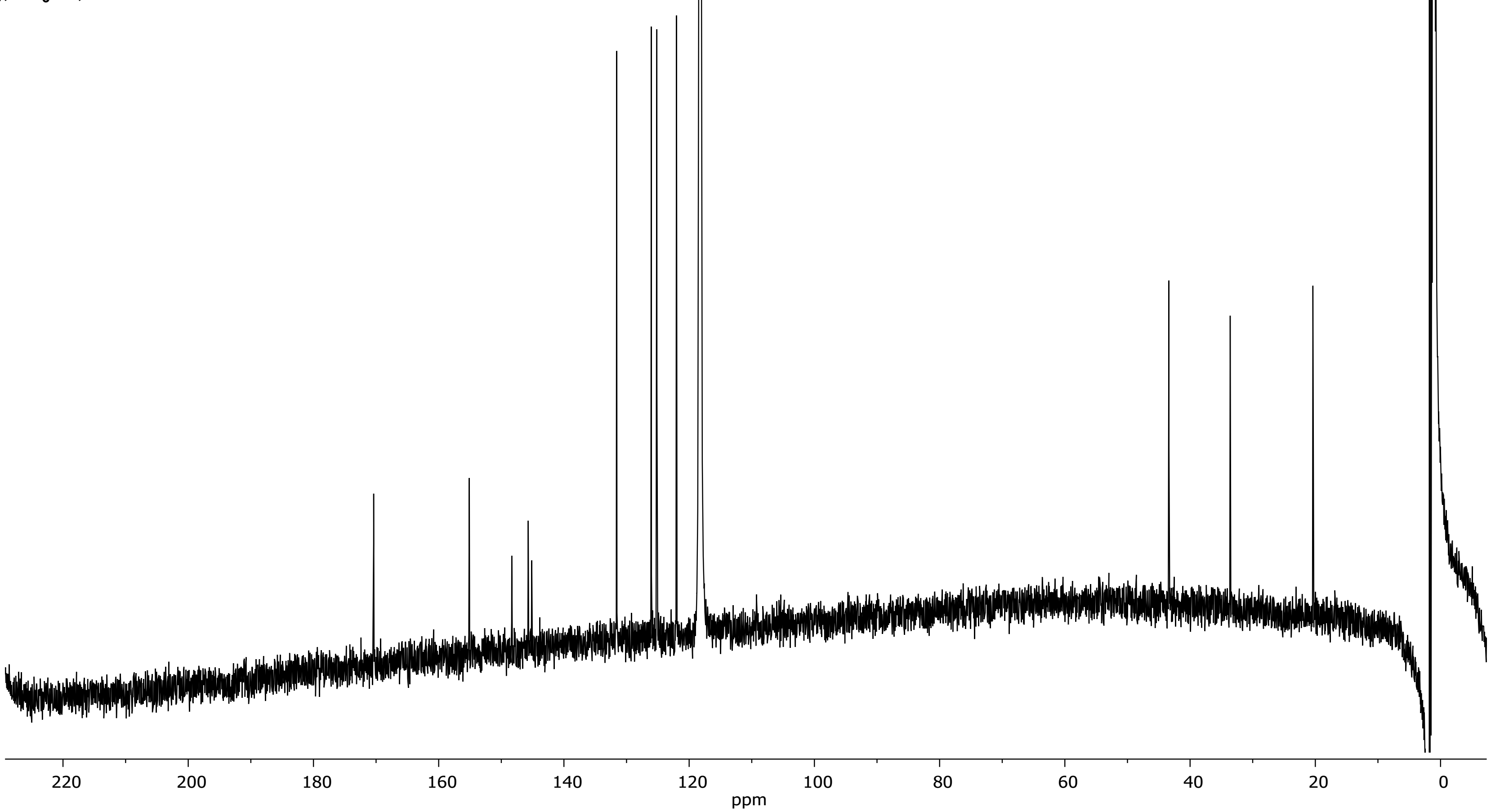

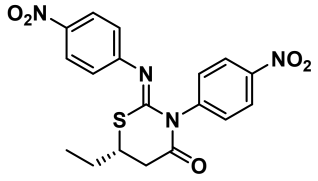

43

^{1}H, $CDCl_3$, 500 MHz

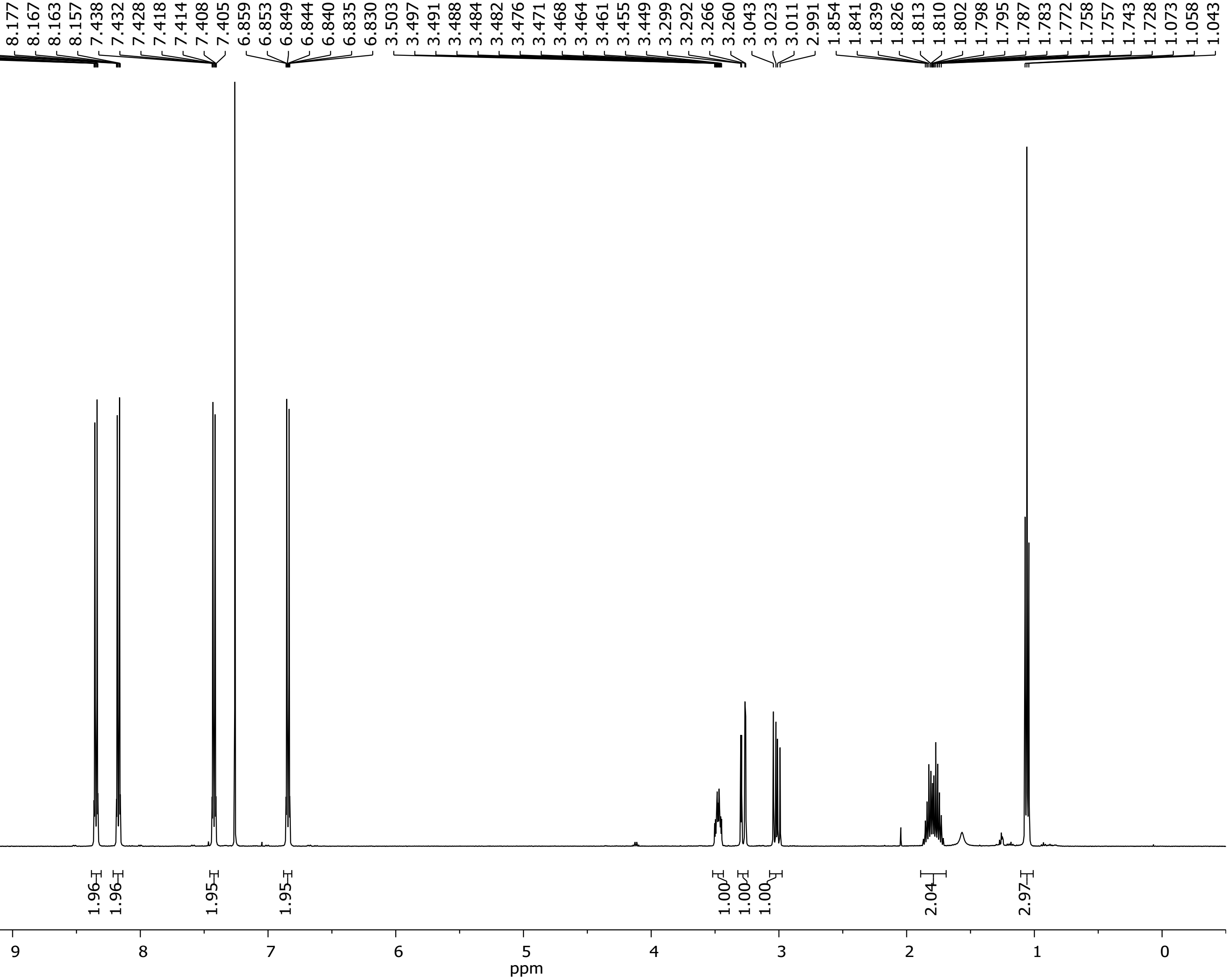

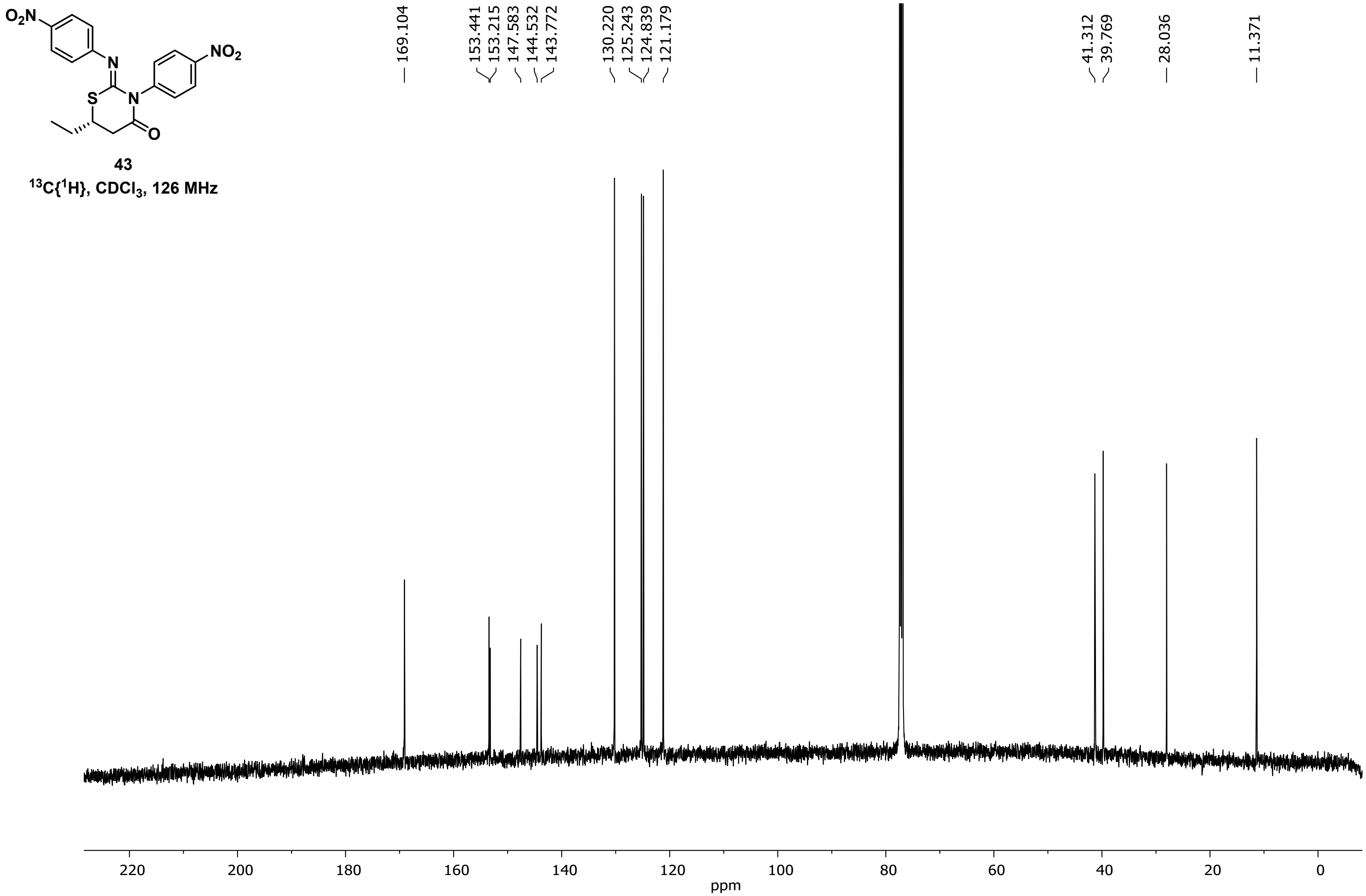
O2N
NO2
N
S
N
O
43
13C{1H}, CDCl3, 126 MHz
169.104
153.441
153.215
147.583
144.532
143.772
130.220
125.243
124.839
121.179
41.312
39.769
28.036
11.371
220
200
180
160
140
120
100
80
60
40
20
0
ppm

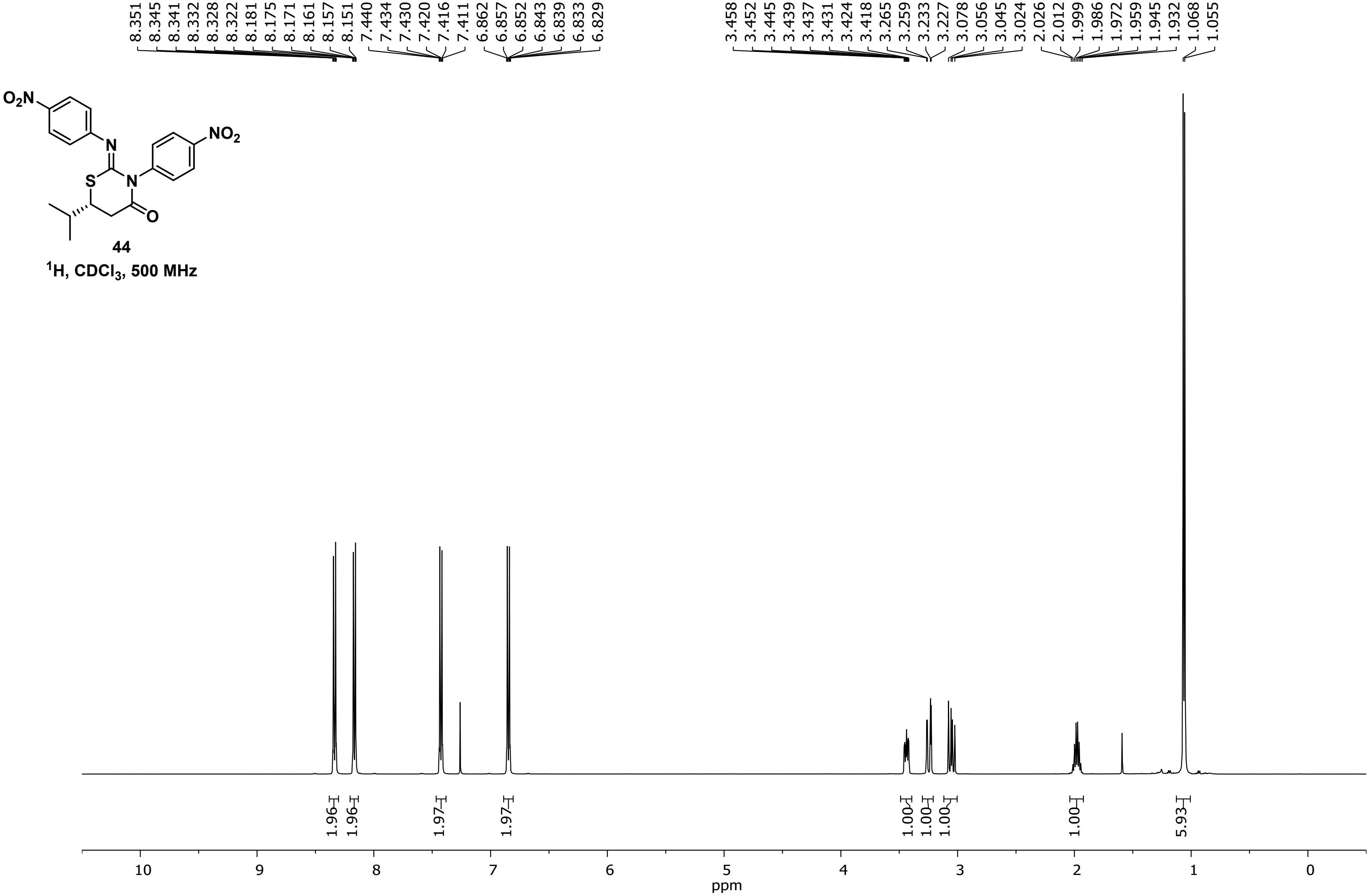

O_2N
NO_2
N
S
N
O
44
^1H, $CDCl_3$, 500 MHz
8.351 8.345 8.341 8.332 8.328 8.322 8.181 8.175 8.171 8.161 8.157 8.151 7.440 7.434 7.430 7.420 7.416 7.411 6.862 6.857 6.852 6.843 6.839 6.833 6.829
3.458 3.452 3.445 3.439 3.437 3.431 3.424 3.418 3.265 3.259 3.233 3.227 3.078 3.056 3.045 3.024 2.026 2.012 1.999 1.986 1.972 1.959 1.945 1.932 1.068 1.055
1.96 1.96 1.97 1.97 1.00 1.00 1.00 1.00 5.93
10 9 8 7 6 5 4 3 2 1 0
ppm

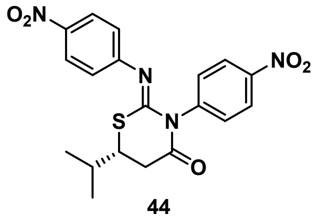

44

$^{13}C\{^{1}H\}$, $CDCl_3$, 126 MHz

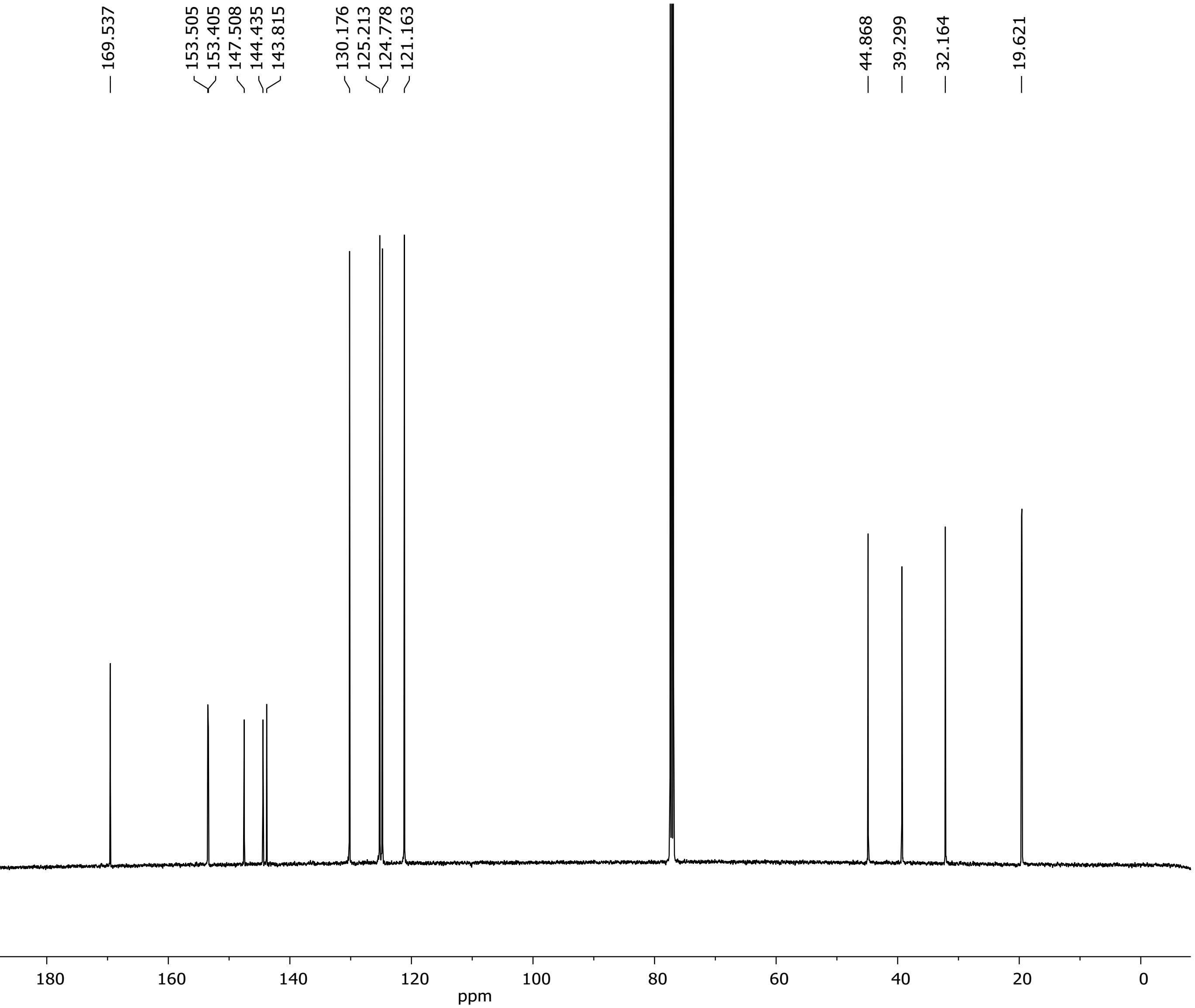

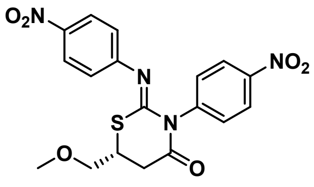

45

^{1}H, $CDCl_3$, 500 MHz

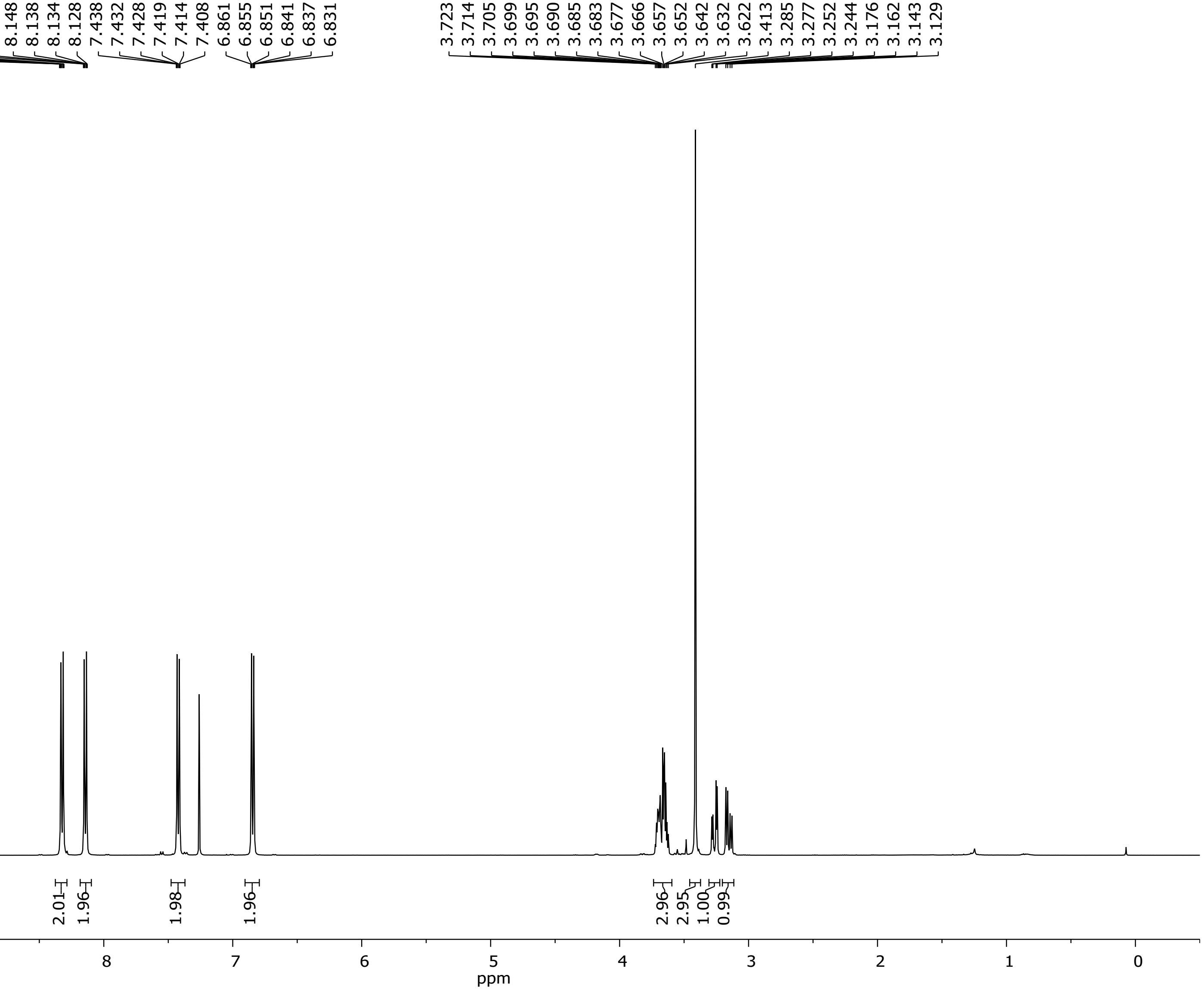

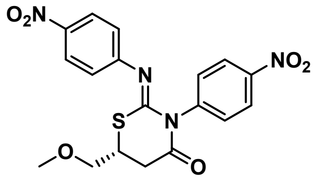

45

$^{13}C\{^1H\}$, $CDCl_3$, 126 MHz

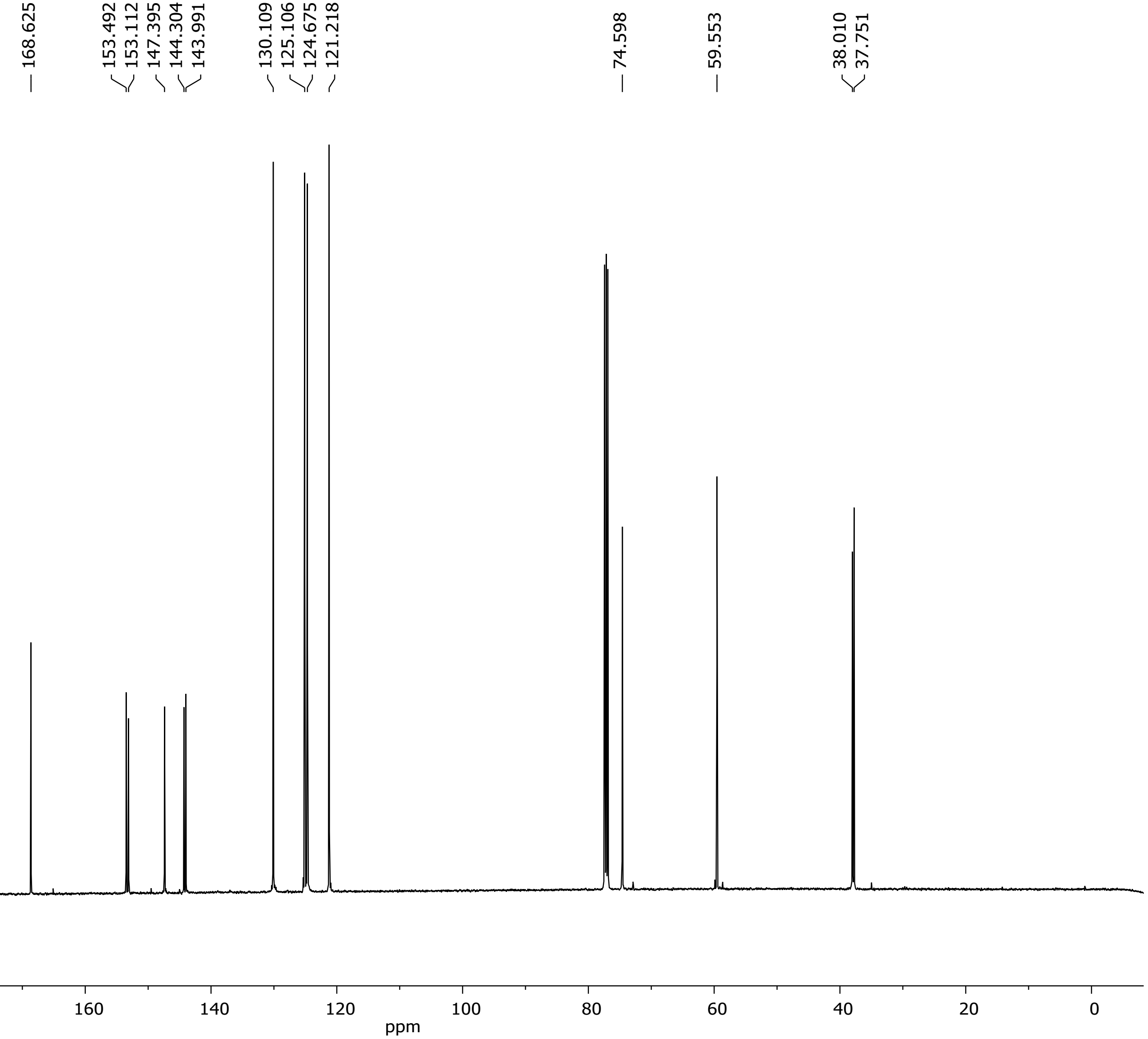

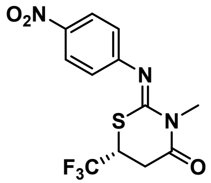

46

^{1}H, $CDCl_3$, 400 MHz

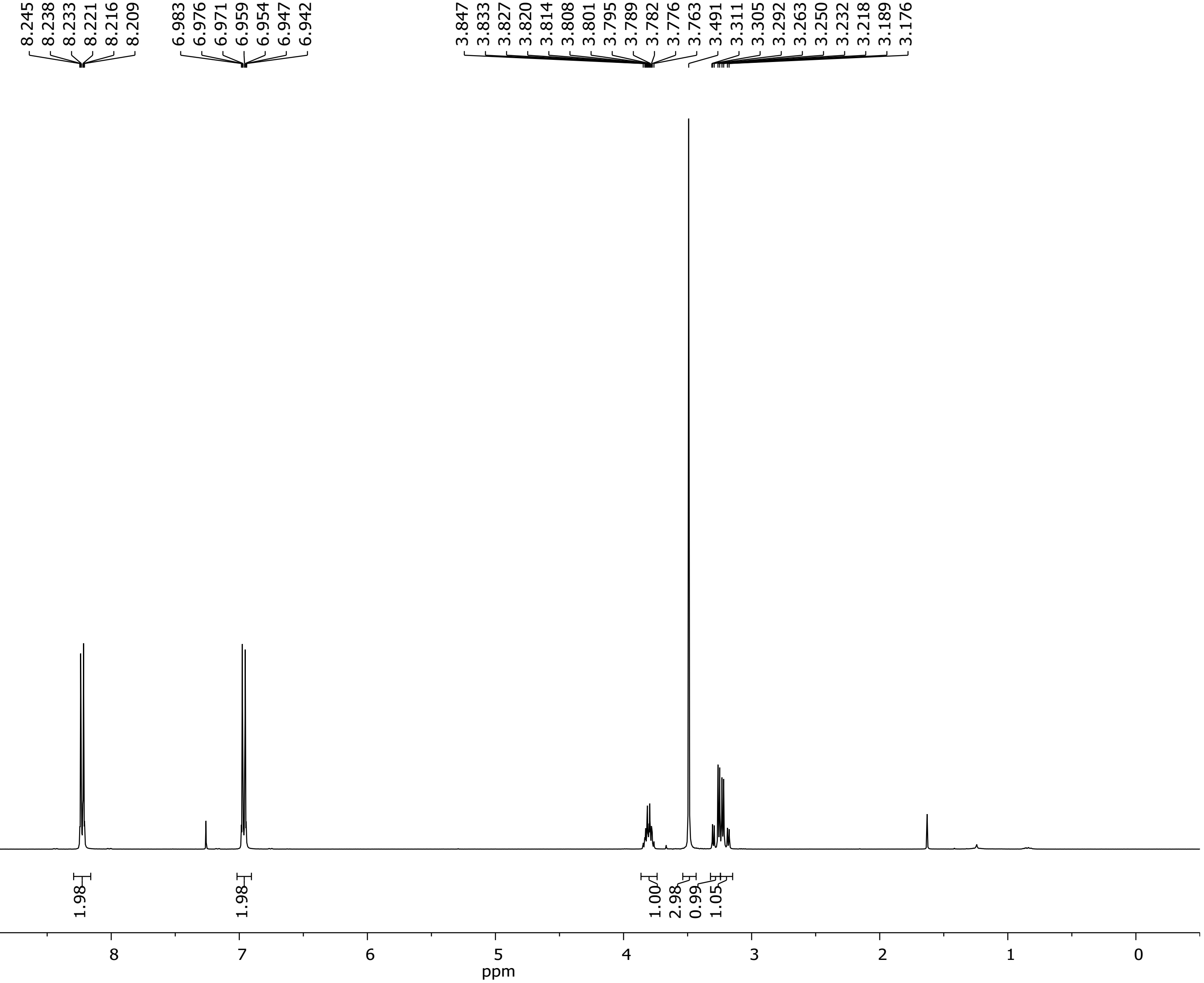

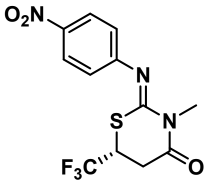

46

$^{19}F\{^1H\}$, $CDCl_3$, 377 MHz

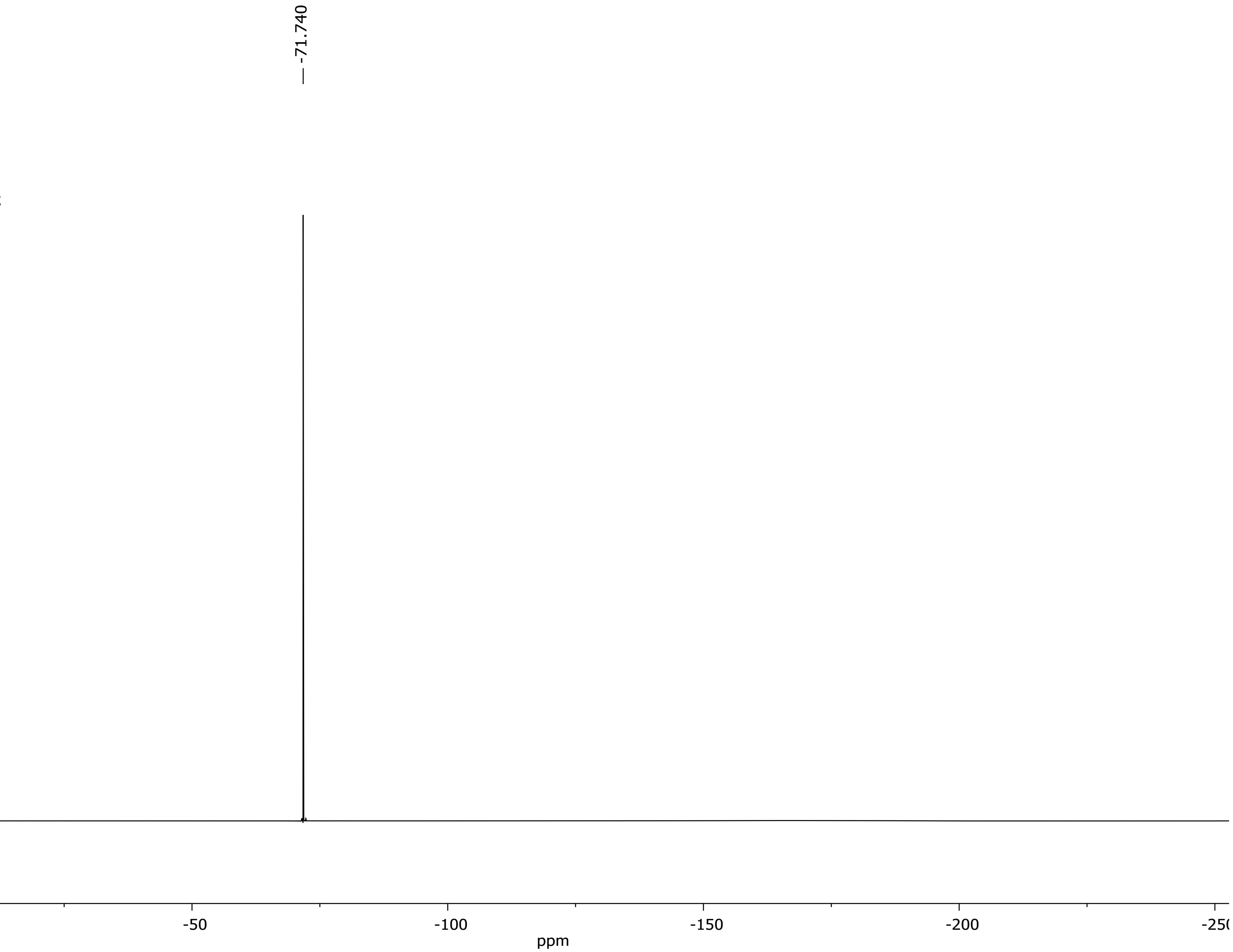

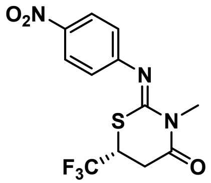

46

$^{13}C\{^1H\}$, $CDCl_3$, 126 MHz

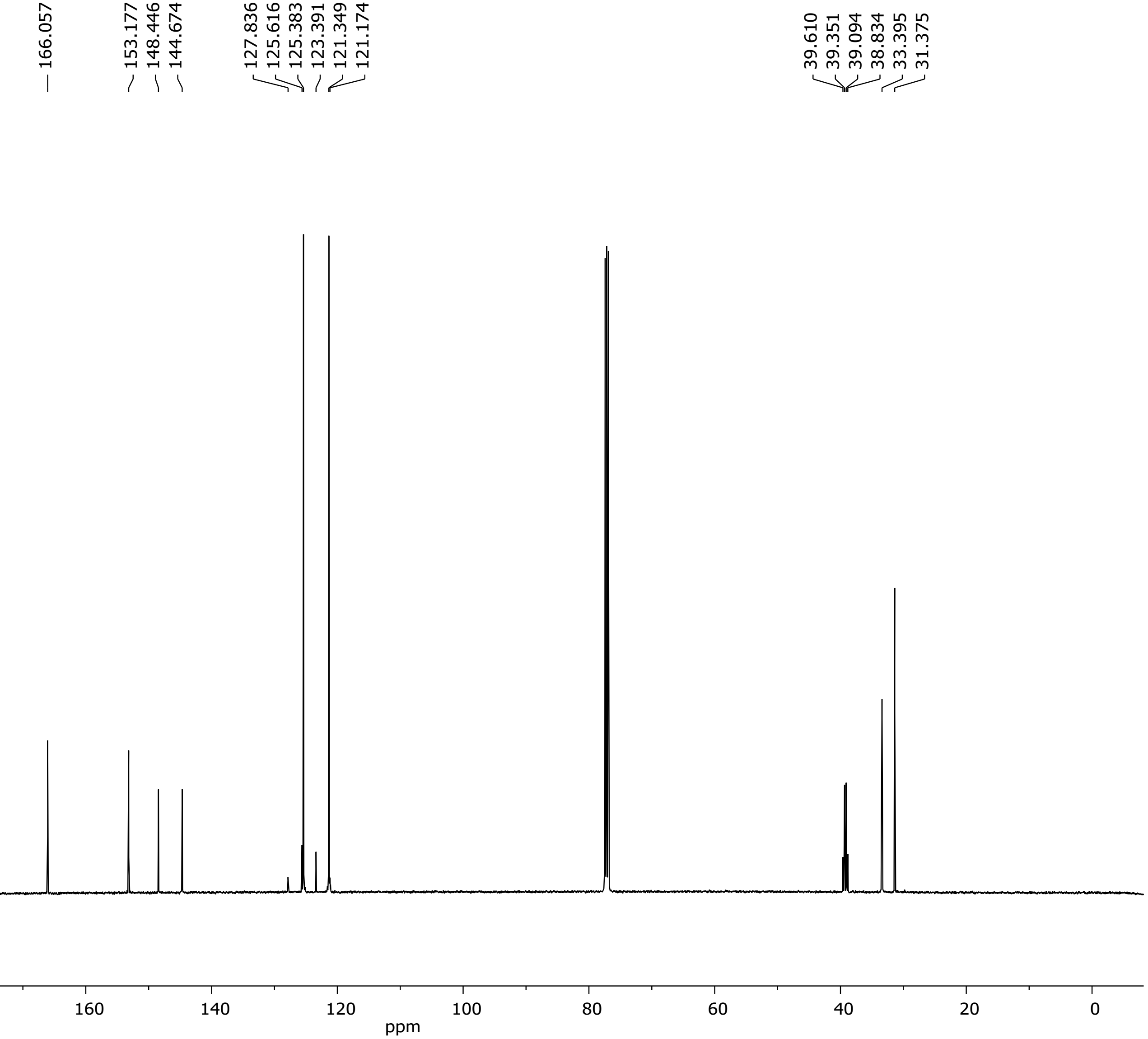

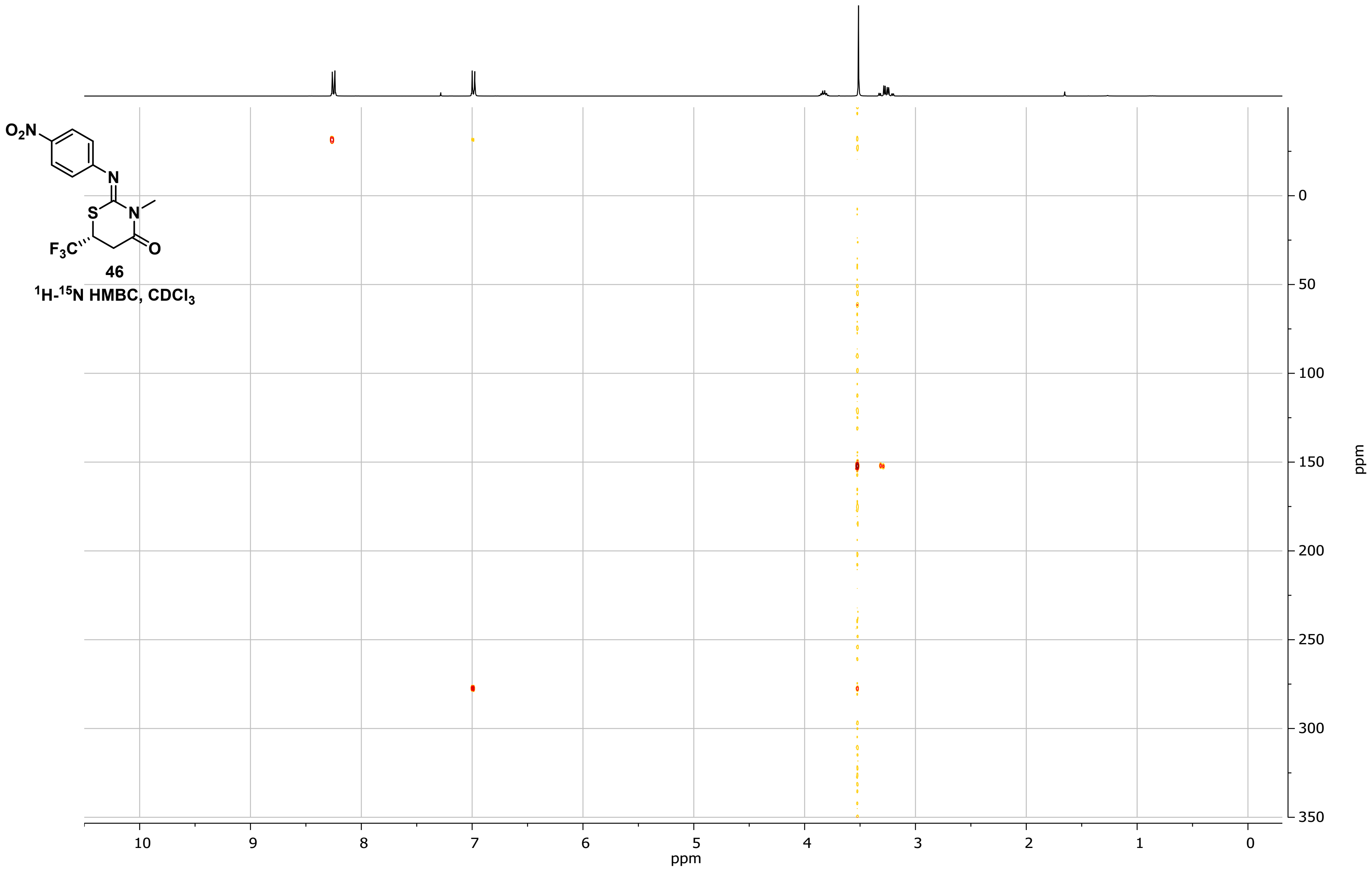

O2N
N
S
N
O
F3C
46
1H-15N HMBC, CDCl3
ppm
ppm

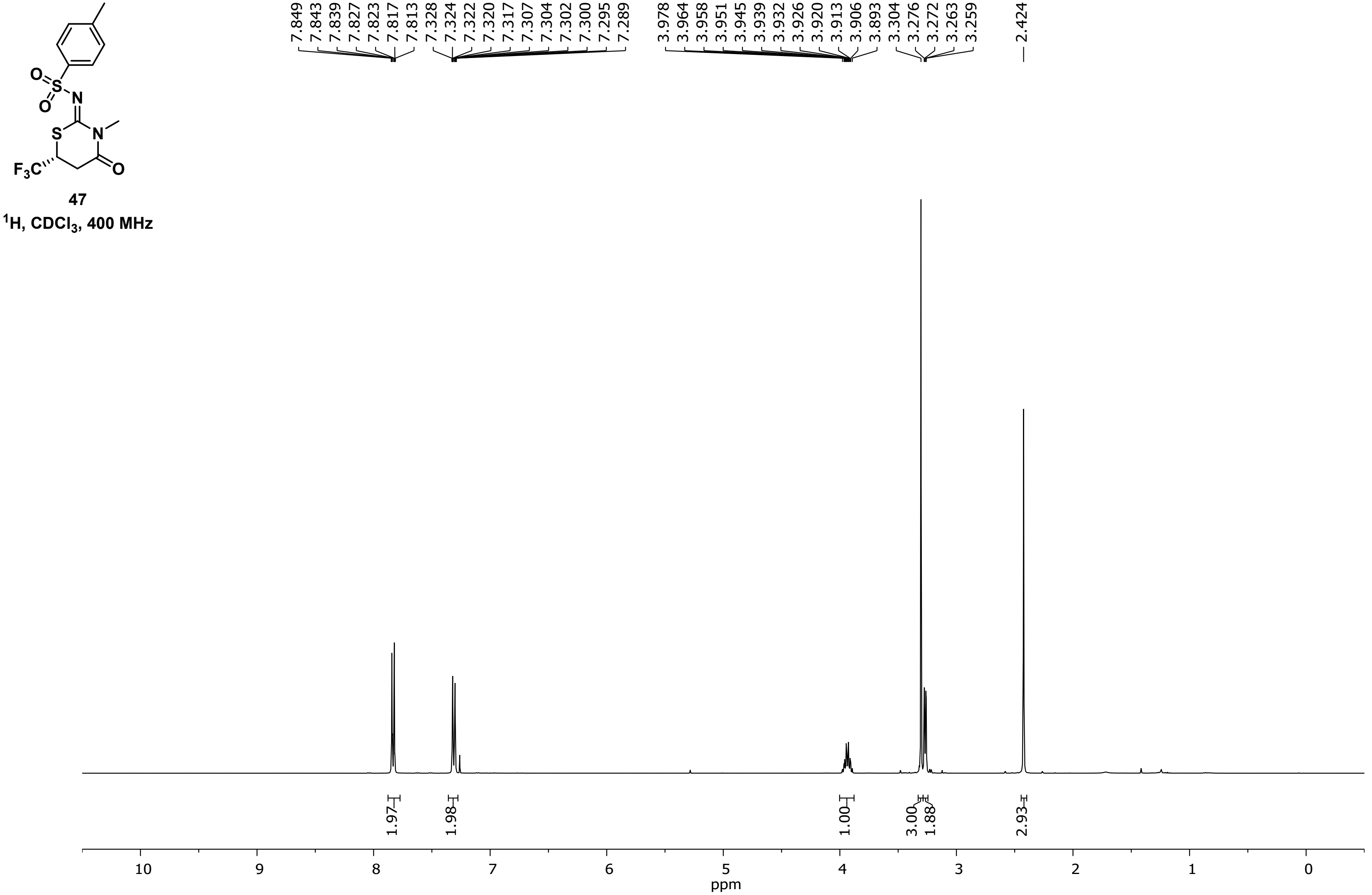

47

^{1}H, $CDCl_3$, 400 MHz

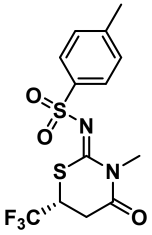

47

$^{19}F\{^{1}H\}$, $CDCl_3$, 377 MHz

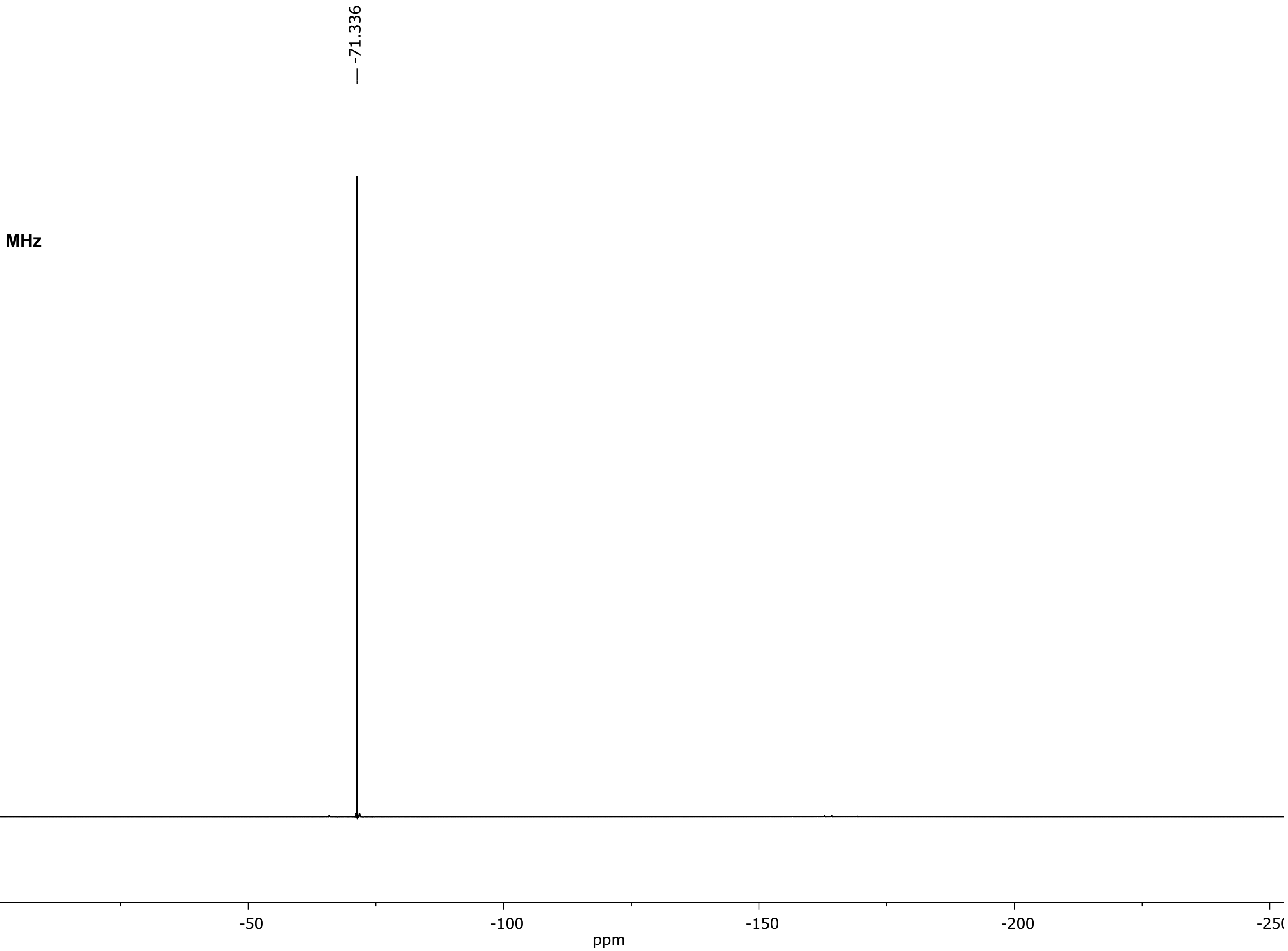

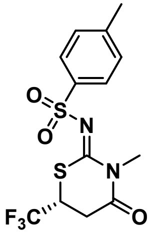

47

$^{13}C\{^1H\}$, $CDCl_3$, 126 MHz

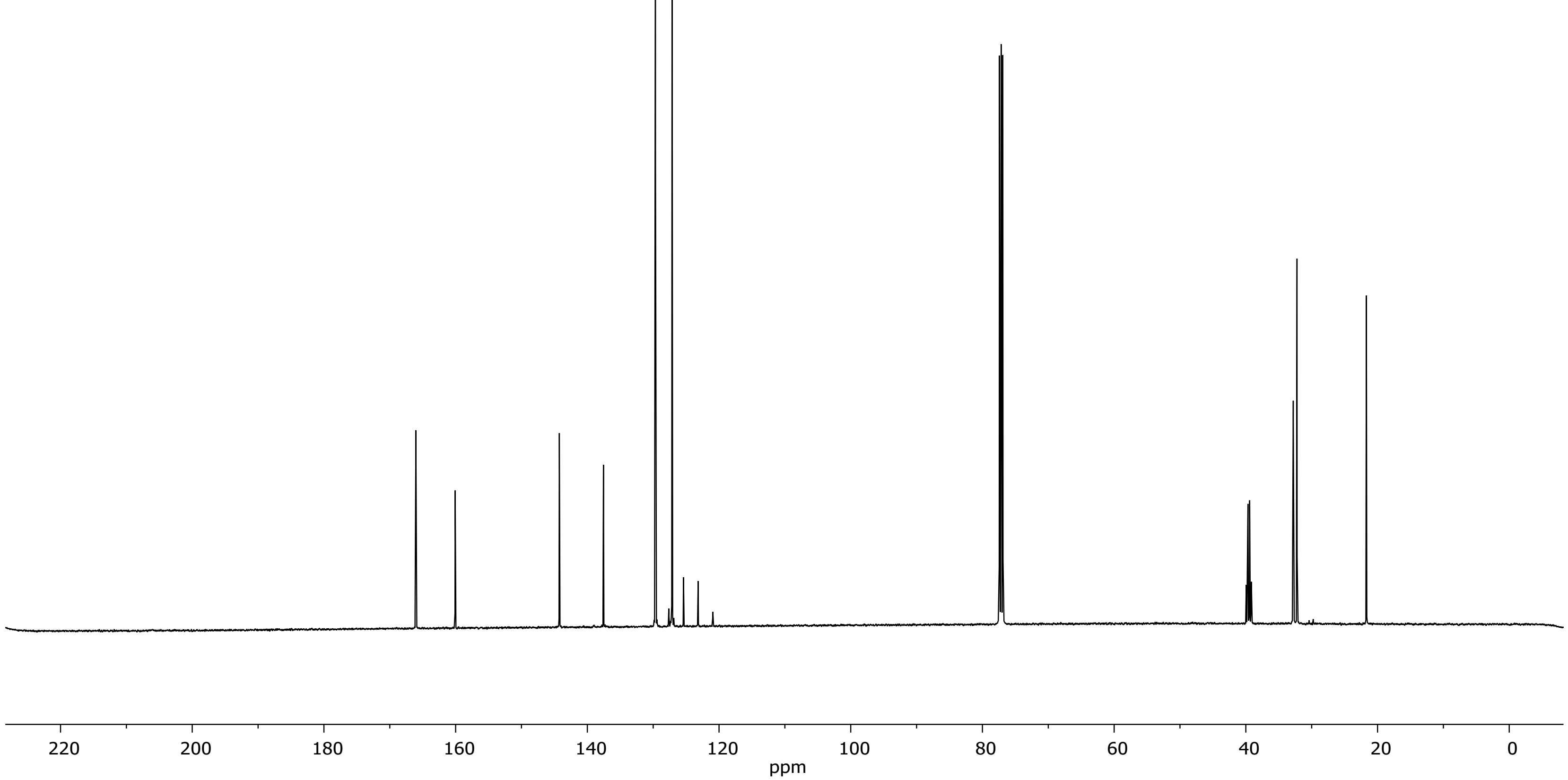

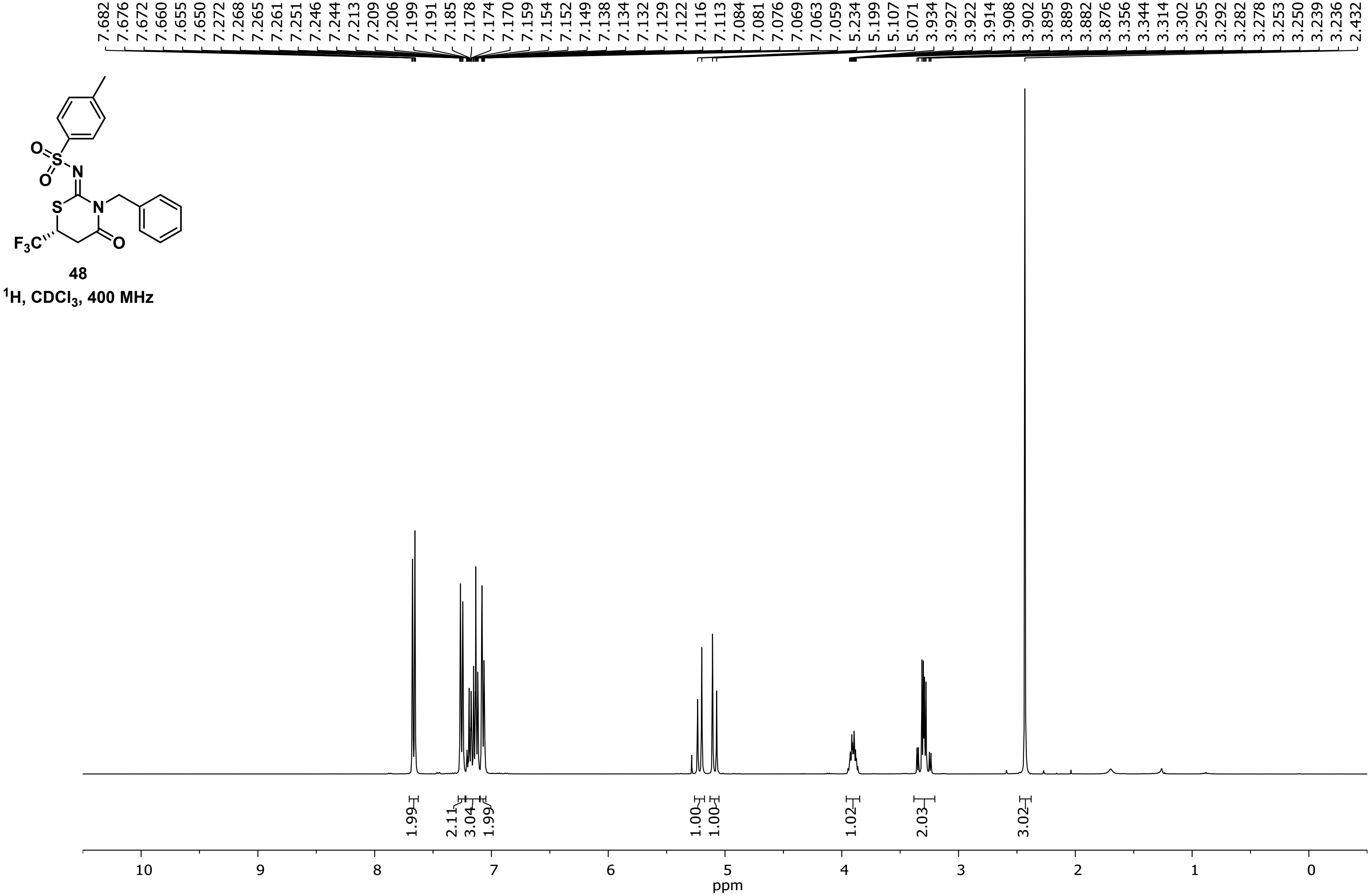

48
^1H, $CDCl_3$, 400 MHz

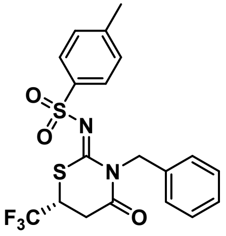

48

$^{19}F\{^{1}H\}$, $CDCl_3$, 377 MHz

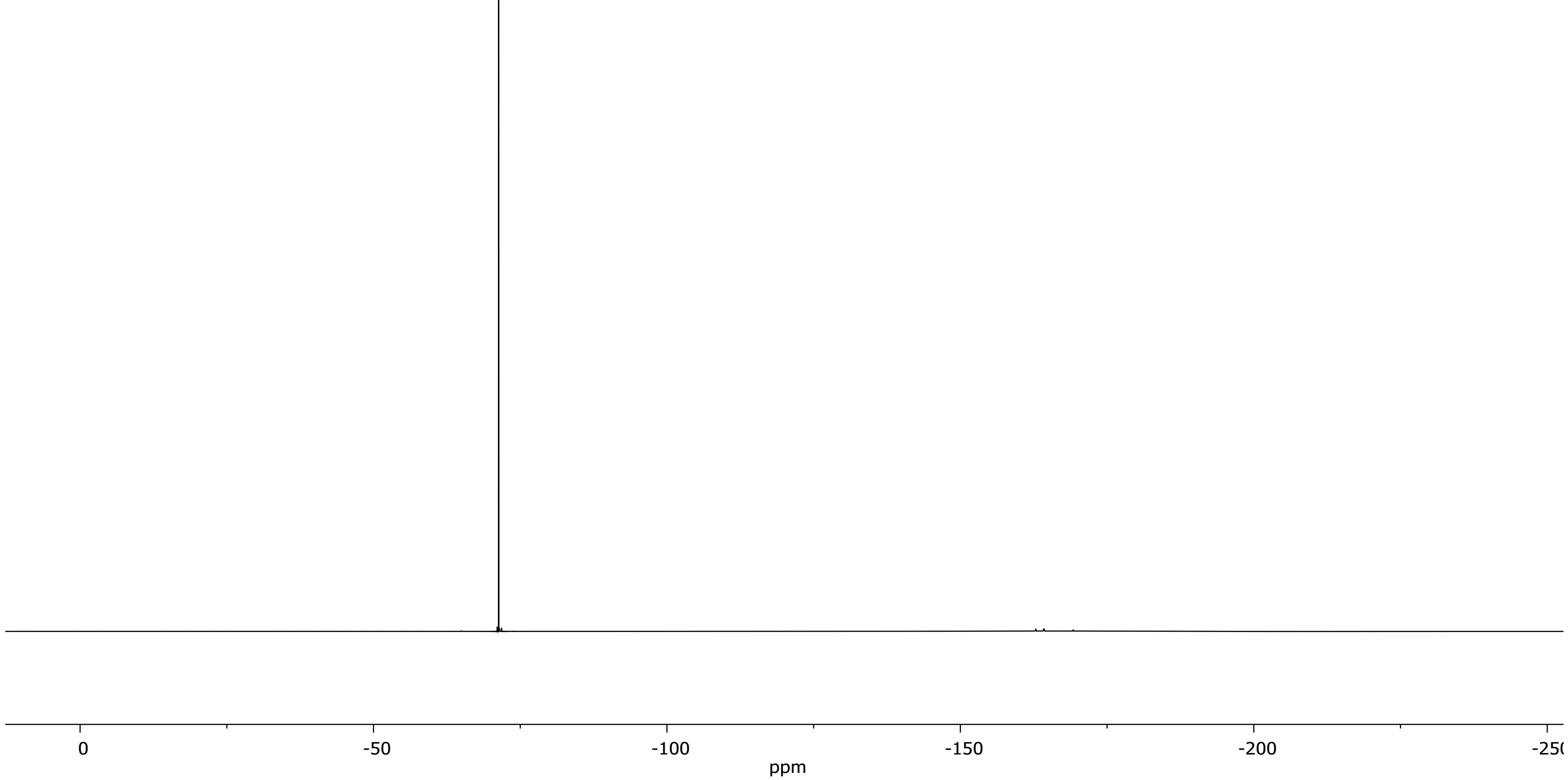

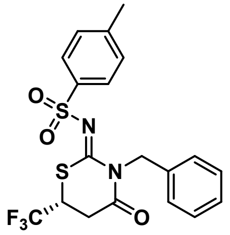

48

$^{13}C\{^{1}H\}$, $CDCl_3$, 126 MHz

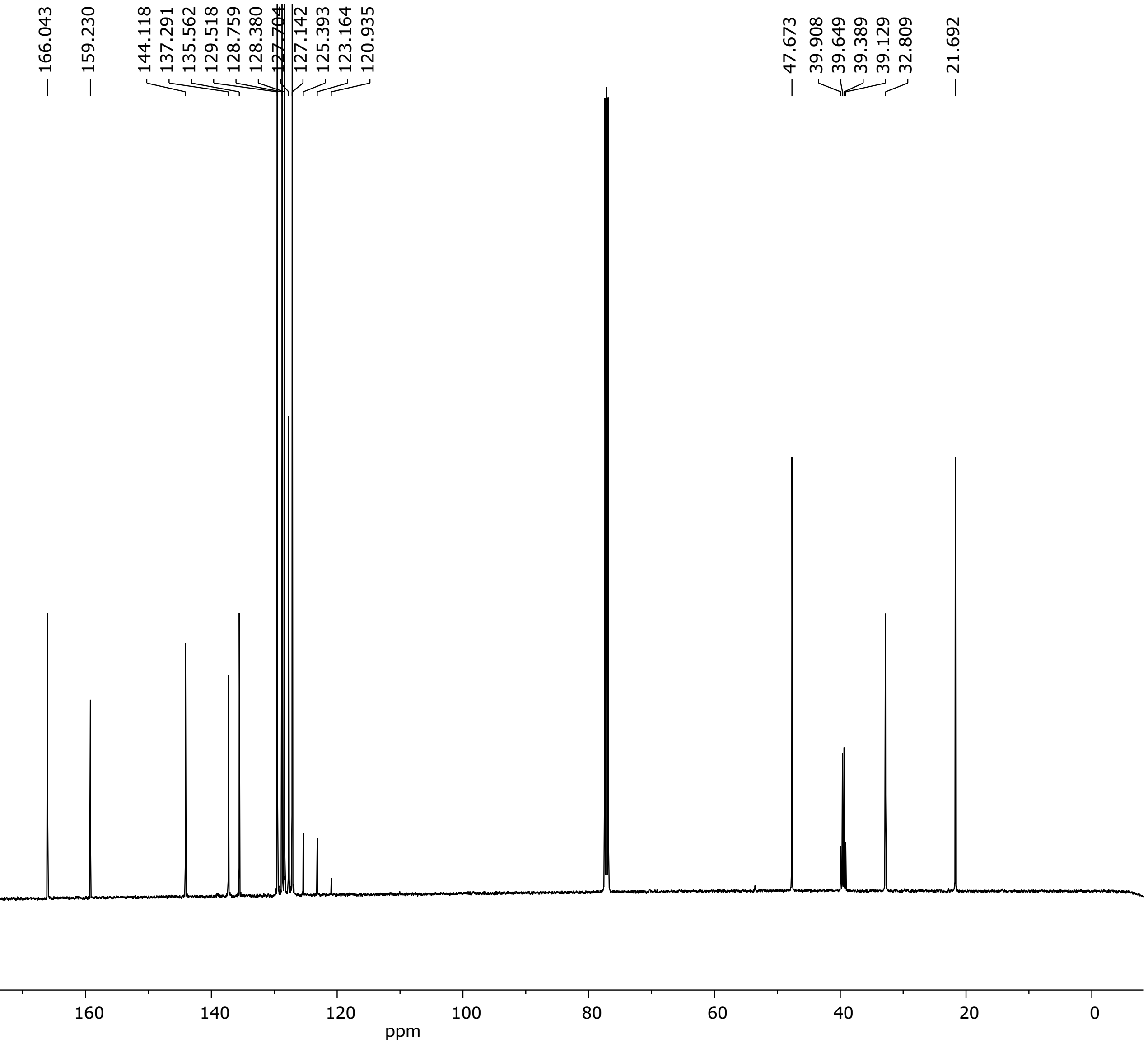

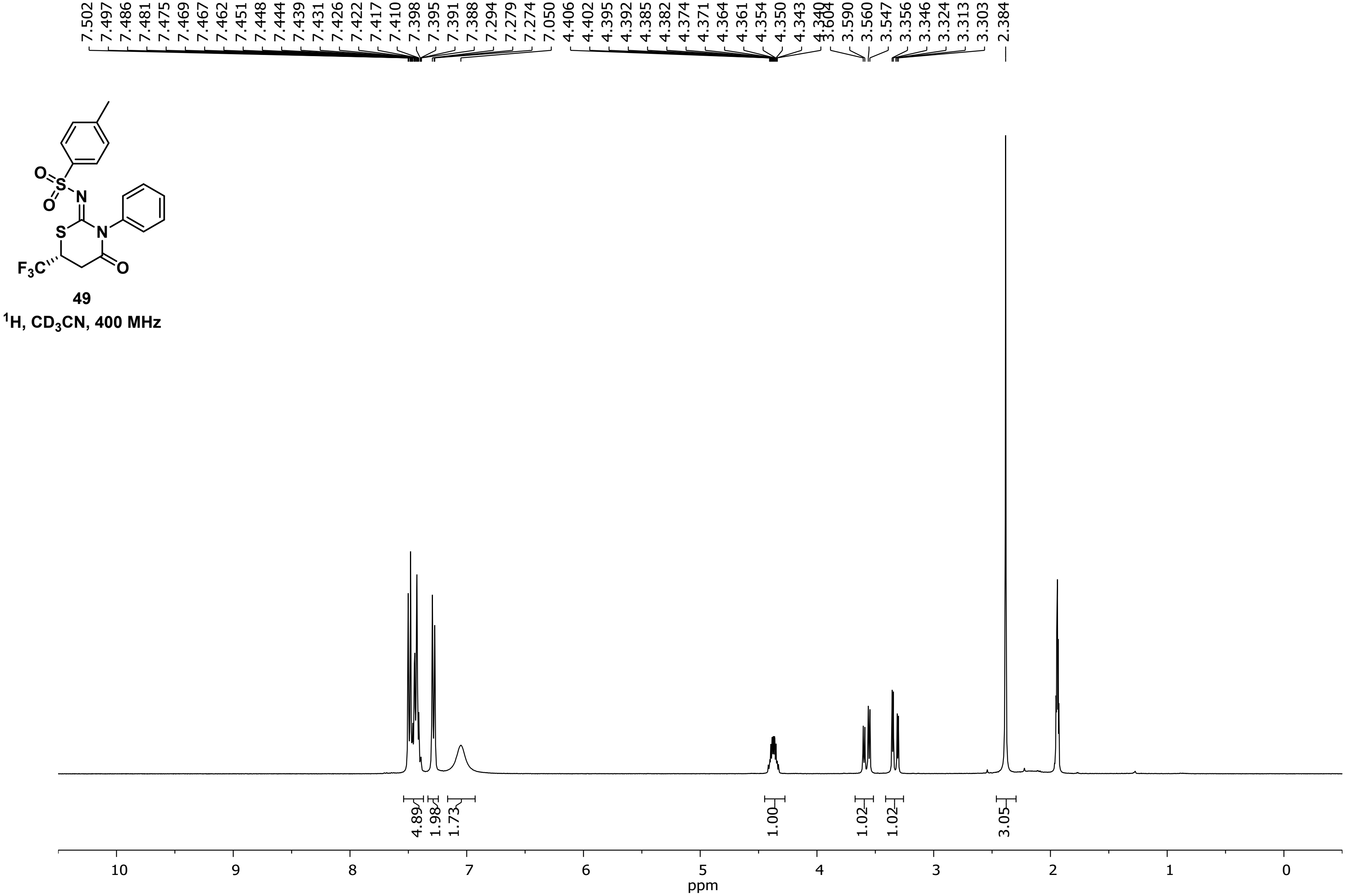

49

^{1}H, CD_3CN, 400 MHz

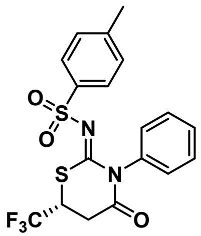

49

$^{19}F\{^{1}H\}$, CD_3CN, 377 MHz

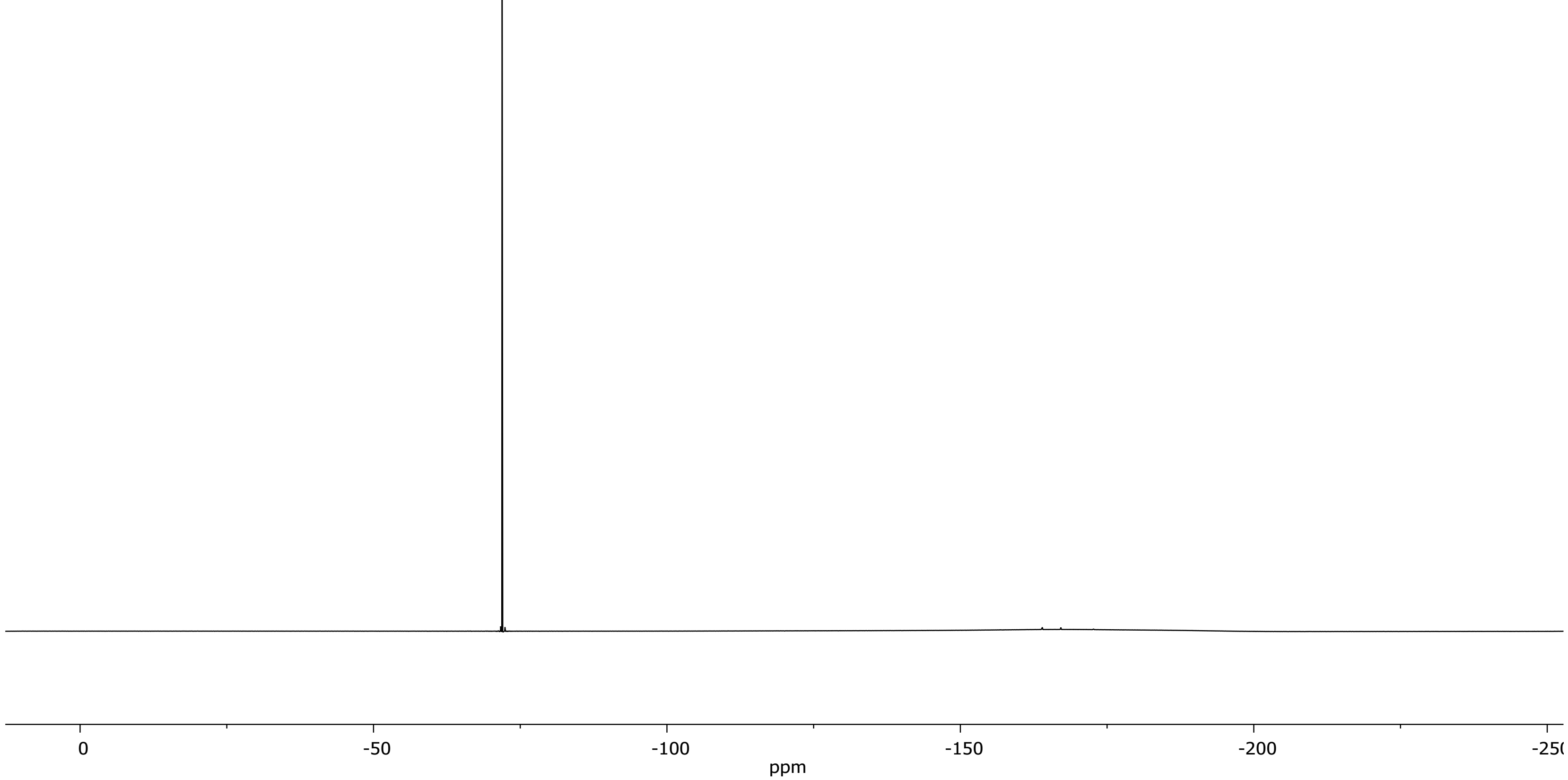

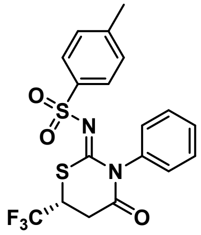

49

$^{13}C\{^{1}H\}$, CD_3CN, 126 MHz

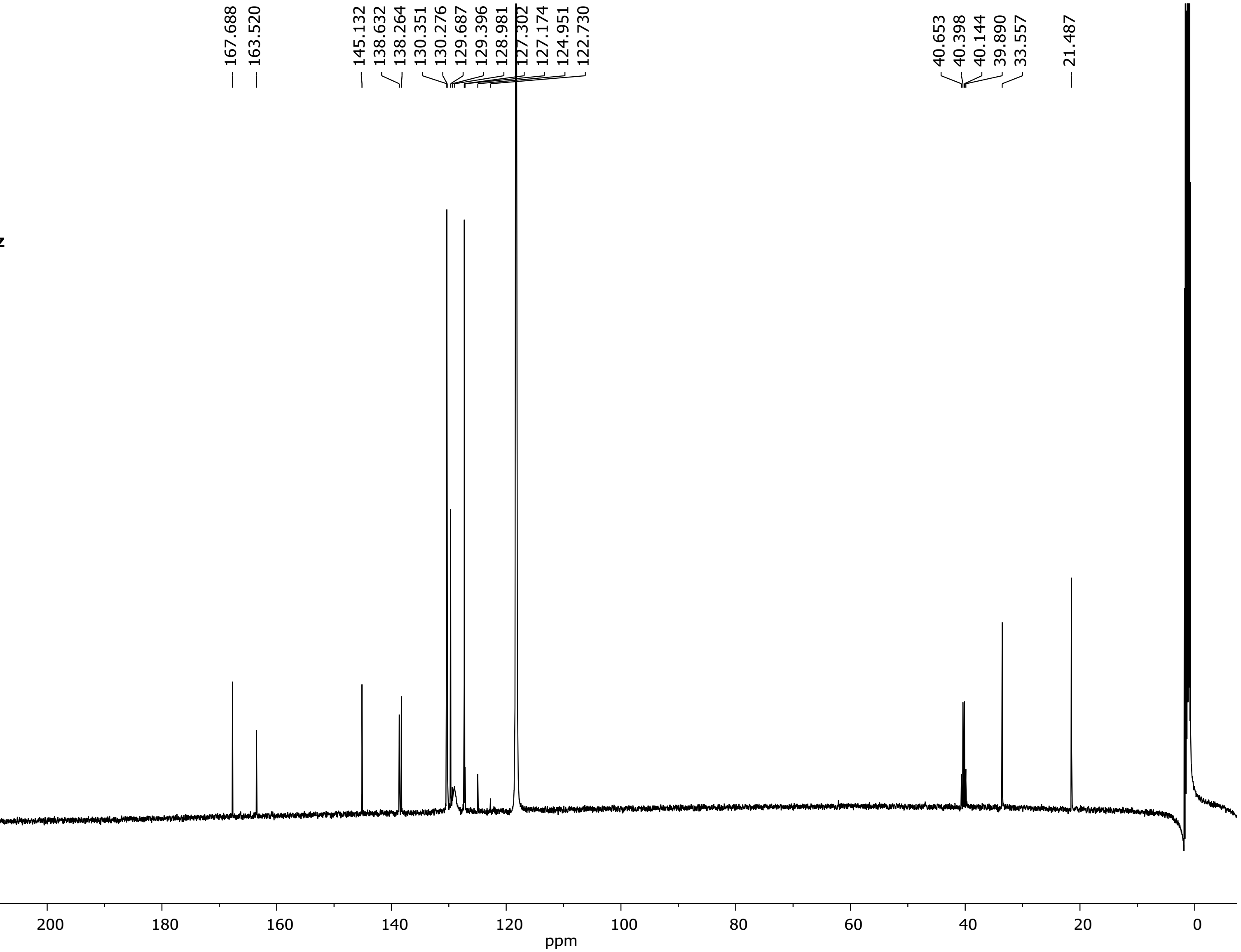

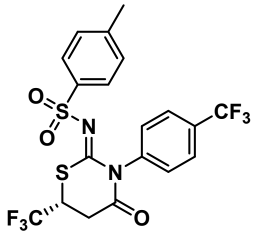

50

^{1}H, CD_3CN, 400 MHz

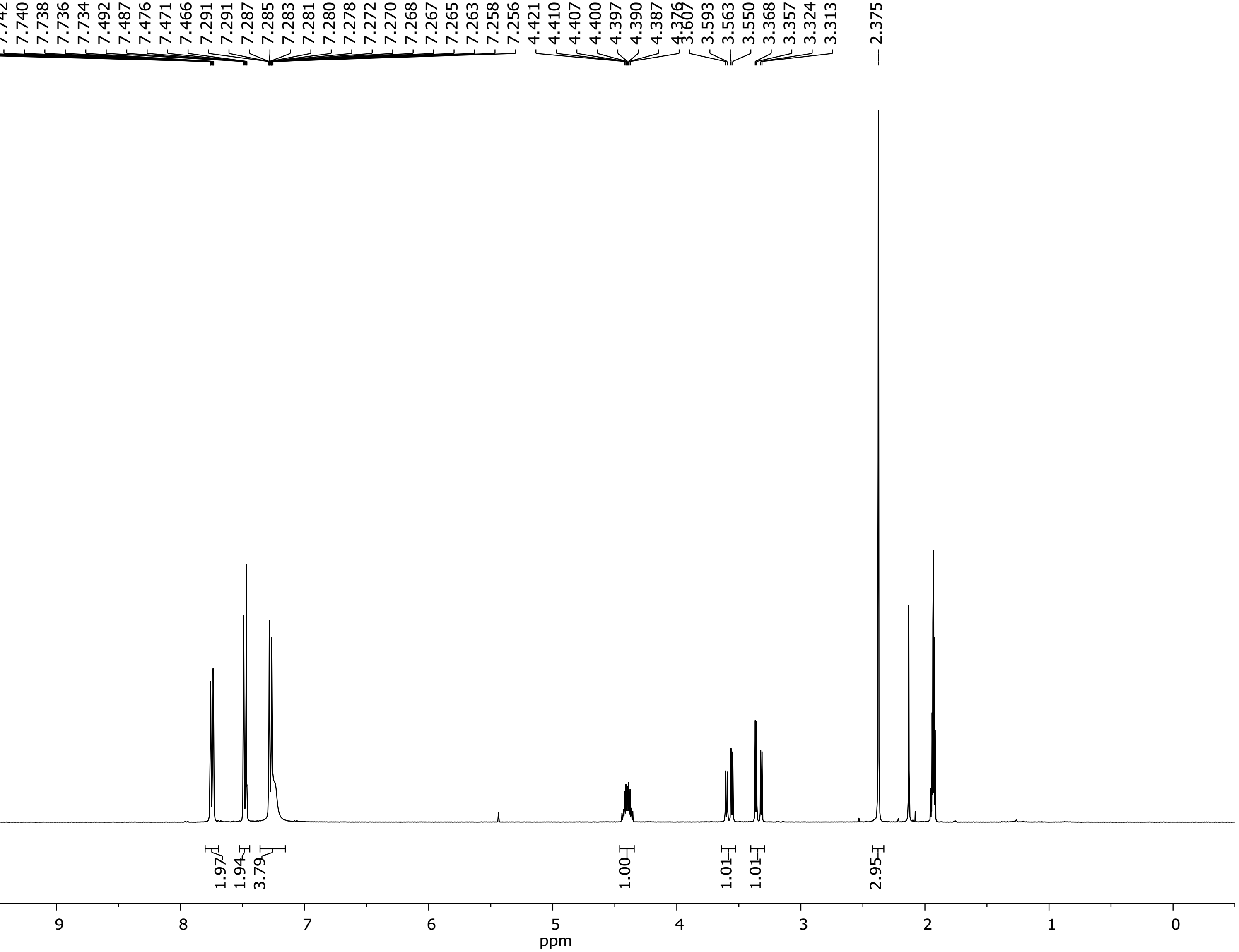

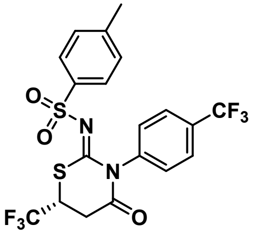

50

$^{19}F\{^{1}H\}$, CD_3CN, 377 MHz

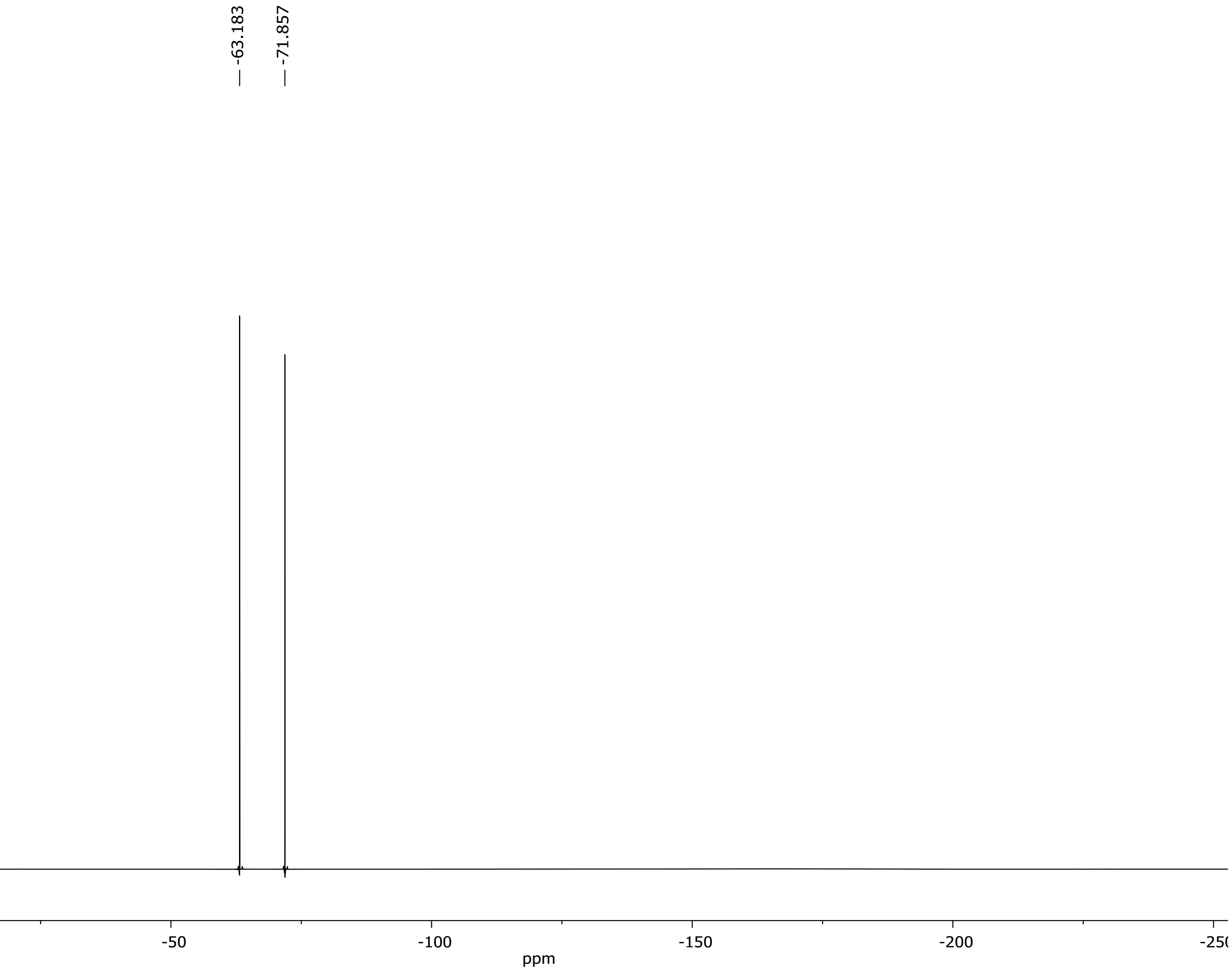

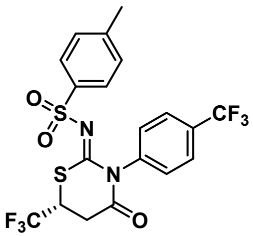

50

$^{13}C\{^1H\}$, CD_3CN, 377 MHz

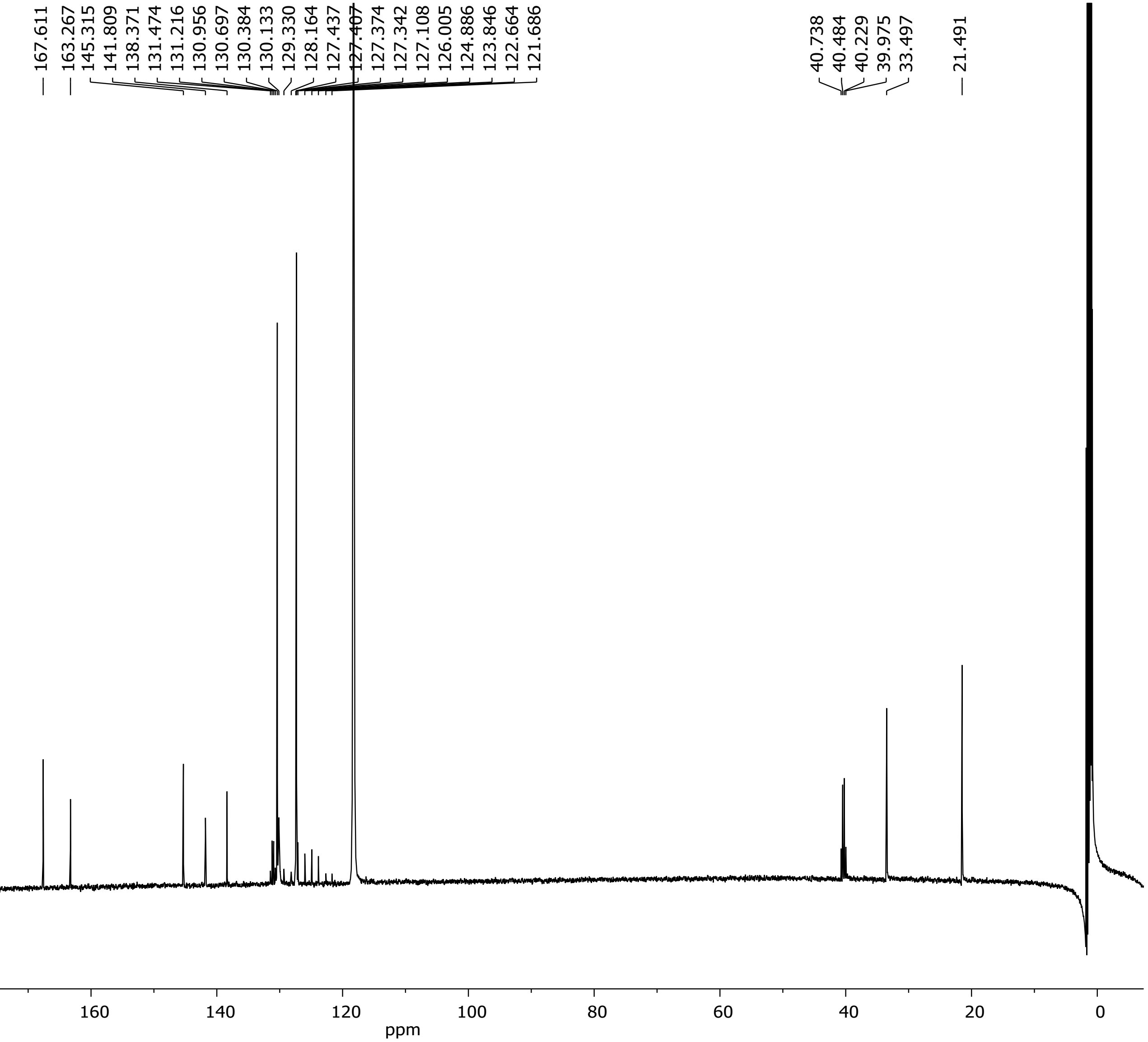

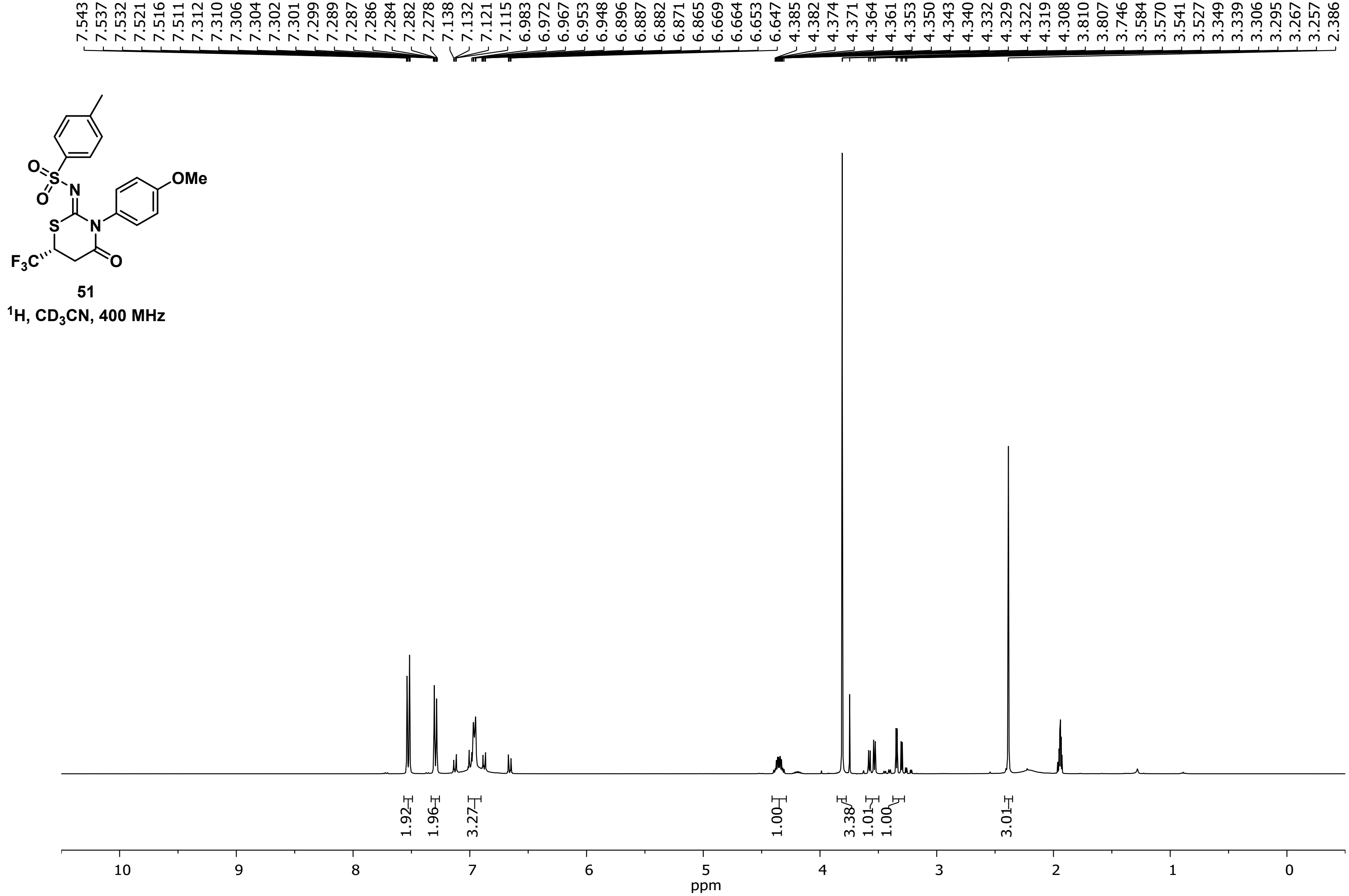

7.543
7.537
7.532
7.521
7.516
7.511
7.312
7.310
7.306
7.304
7.302
7.301
7.299
7.289
7.287
7.286
7.284
7.282
7.278
7.138
7.132
7.121
7.115
6.983
6.972
6.967
6.953
6.948
6.896
6.887
6.882
6.871
6.865
6.669
6.664
6.653
6.647
4.385
4.382
4.374
4.371
4.364
4.361
4.353
4.350
4.343
4.340
4.332
4.329
4.322
4.319
4.308
3.810
3.807
3.746
3.584
3.570
3.541
3.527
3.349
3.339
3.306
3.295
3.267
3.257
2.386
OMe
O
S
N
O
S
N
F_3C
O
51
^1H, CD_3CN, 400 MHz
1.92
1.96
3.27
1.00
3.38
1.01
1.00
3.01
10
9
8
7
6
5
4
3
2
1
0
ppm

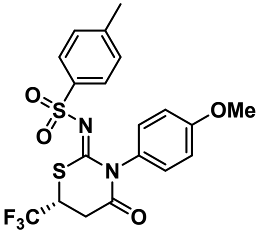

51

$^{19}F\{^1H\}$, CD_3CN, 377 MHz

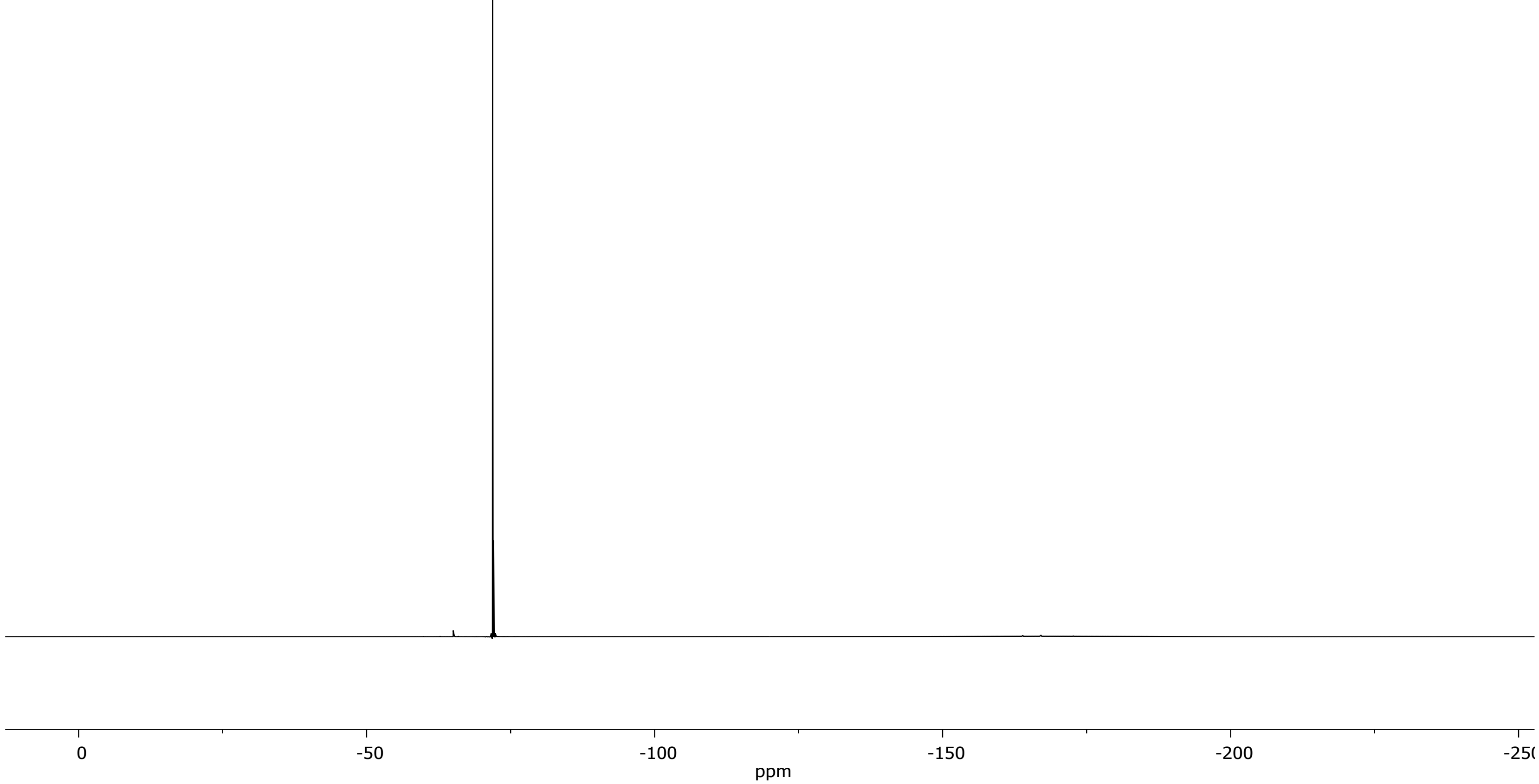

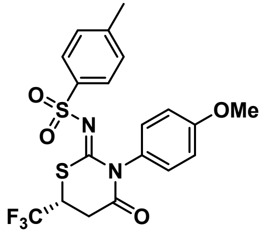

51

$^{13}C\{^{1}H\}$, CD_3CN, 126 MHz

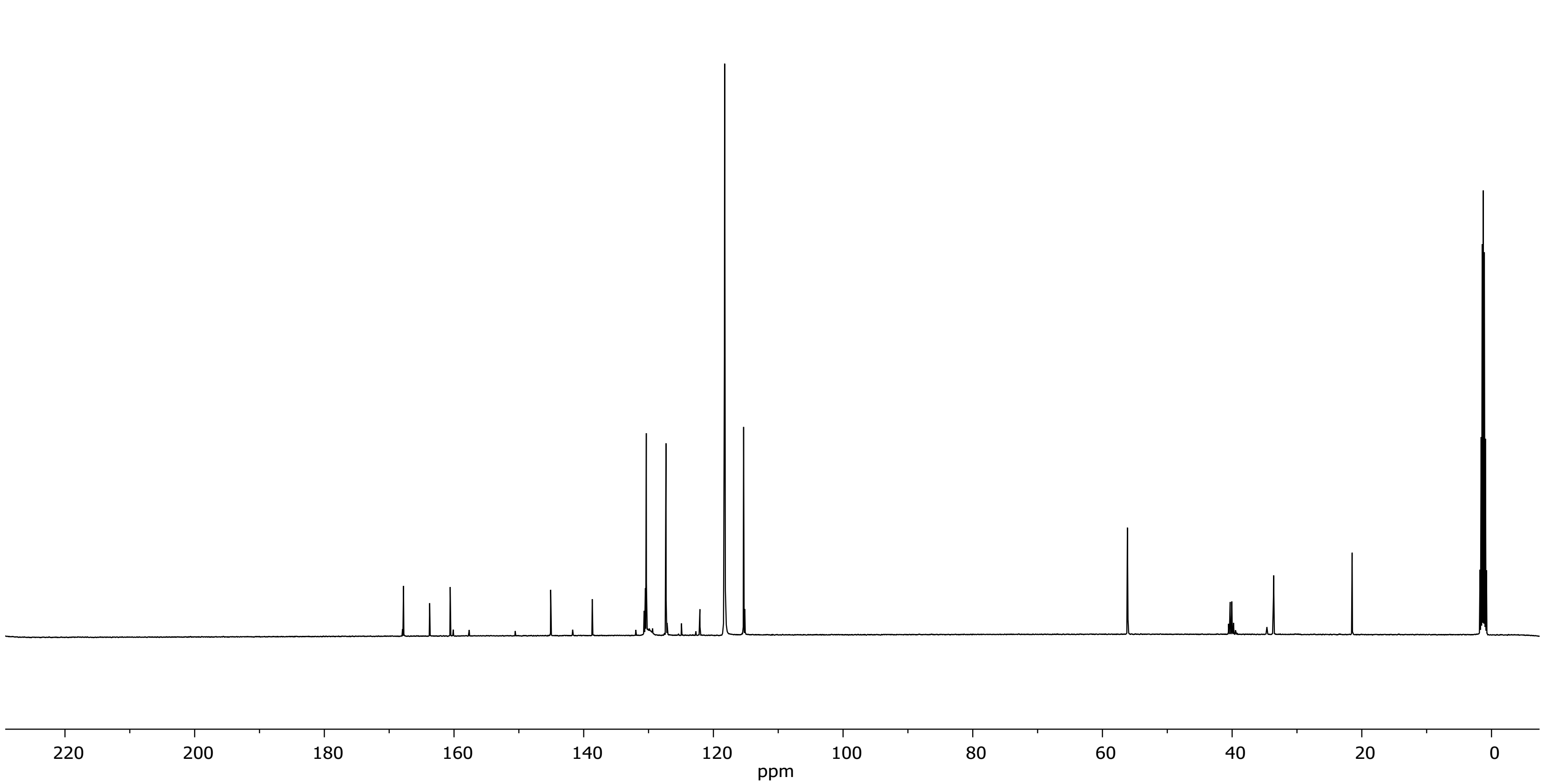

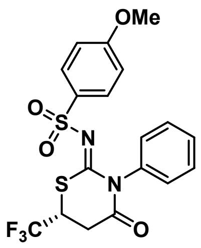

52

^{1}H, CD_3CN, 400 MHz

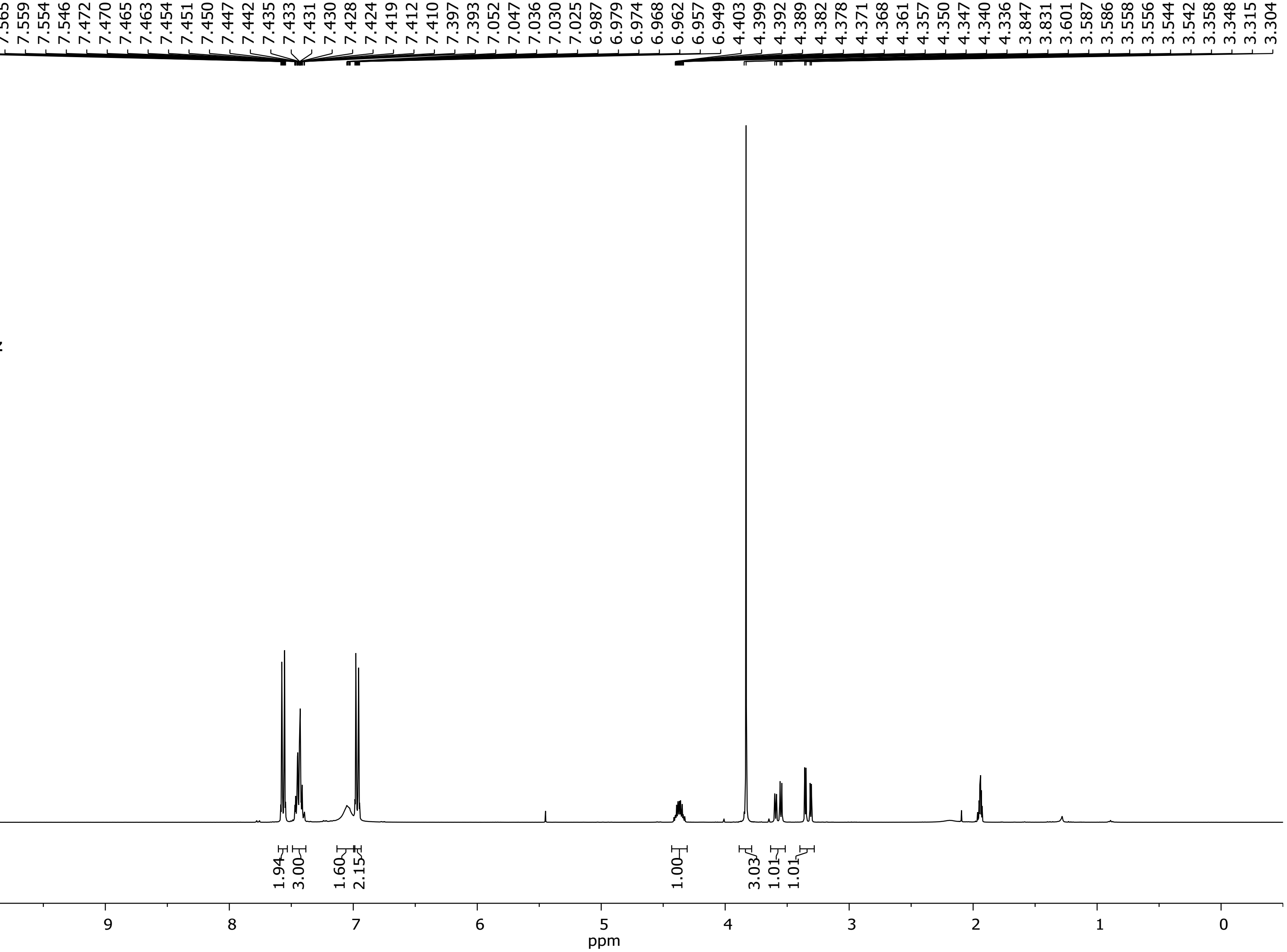

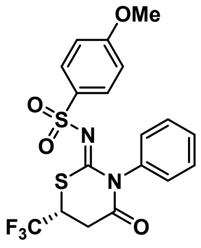

52

$^{19}F\{^{1}H\}$, CD_3CN, 377 MHz

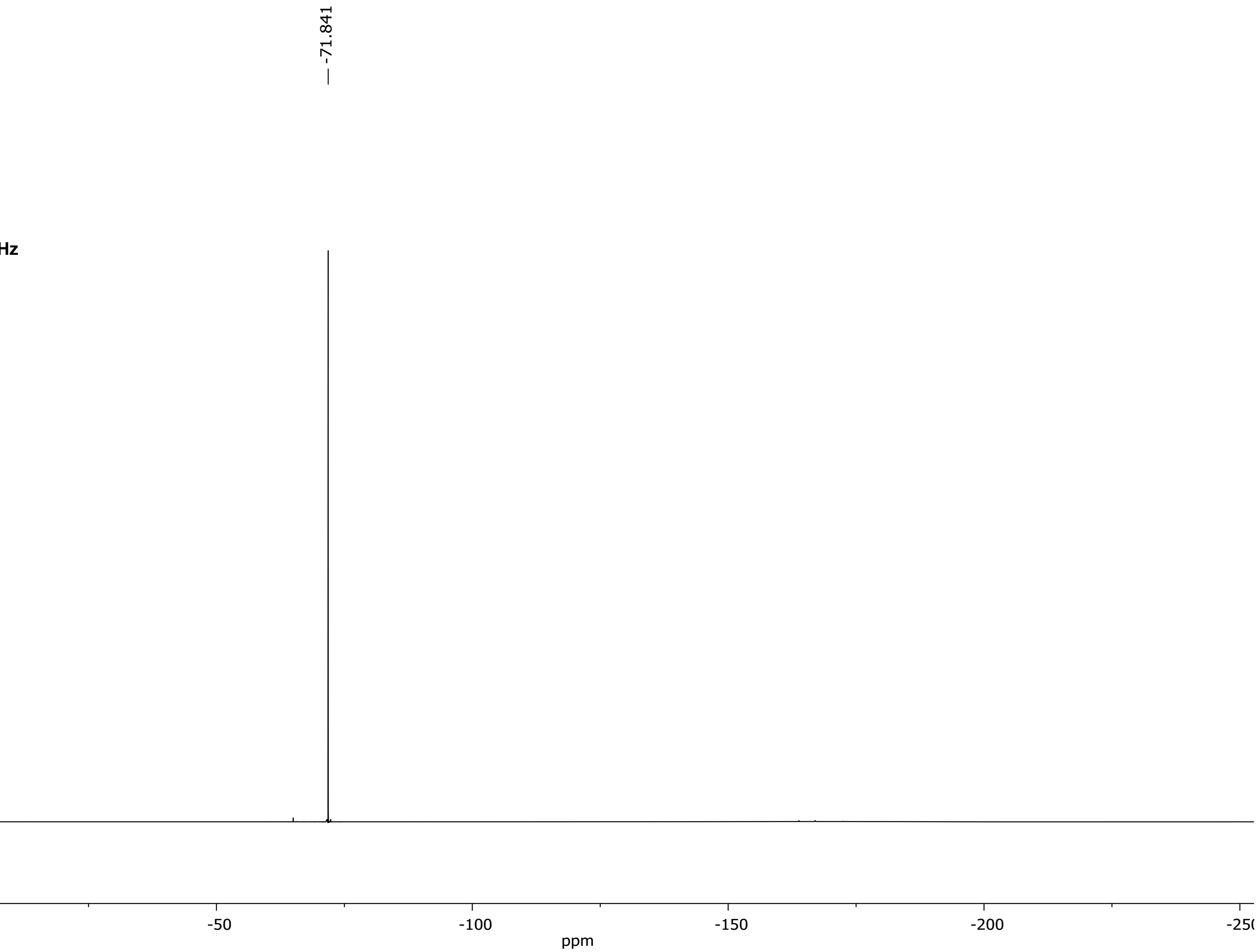

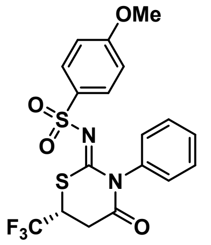

52

$^{13}C\{^{1}H\}$, CD_3CN, 126 MHz

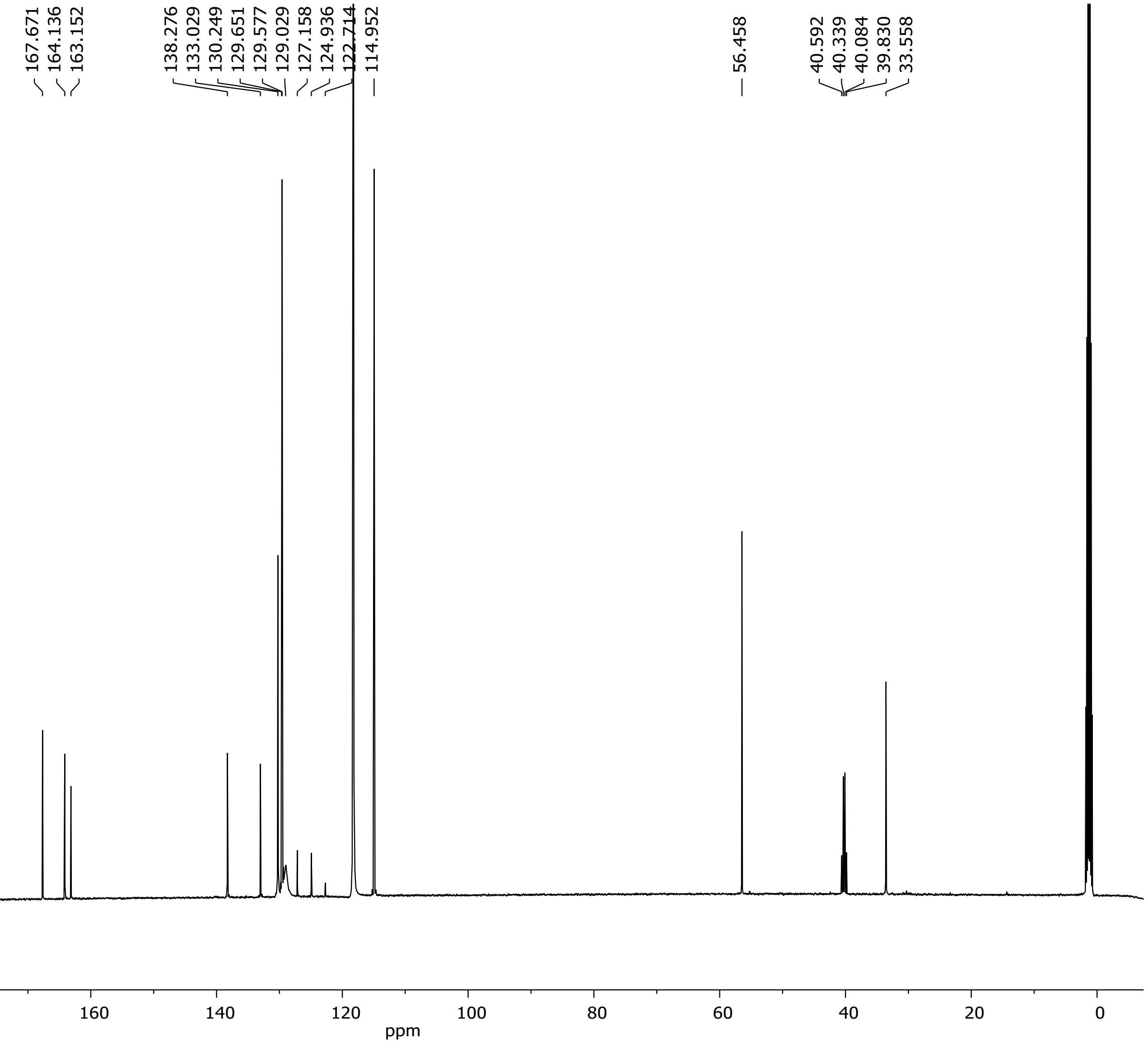

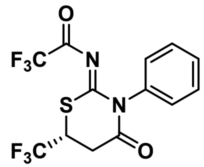

53

^1H, $CDCl_3$, 400 MHz

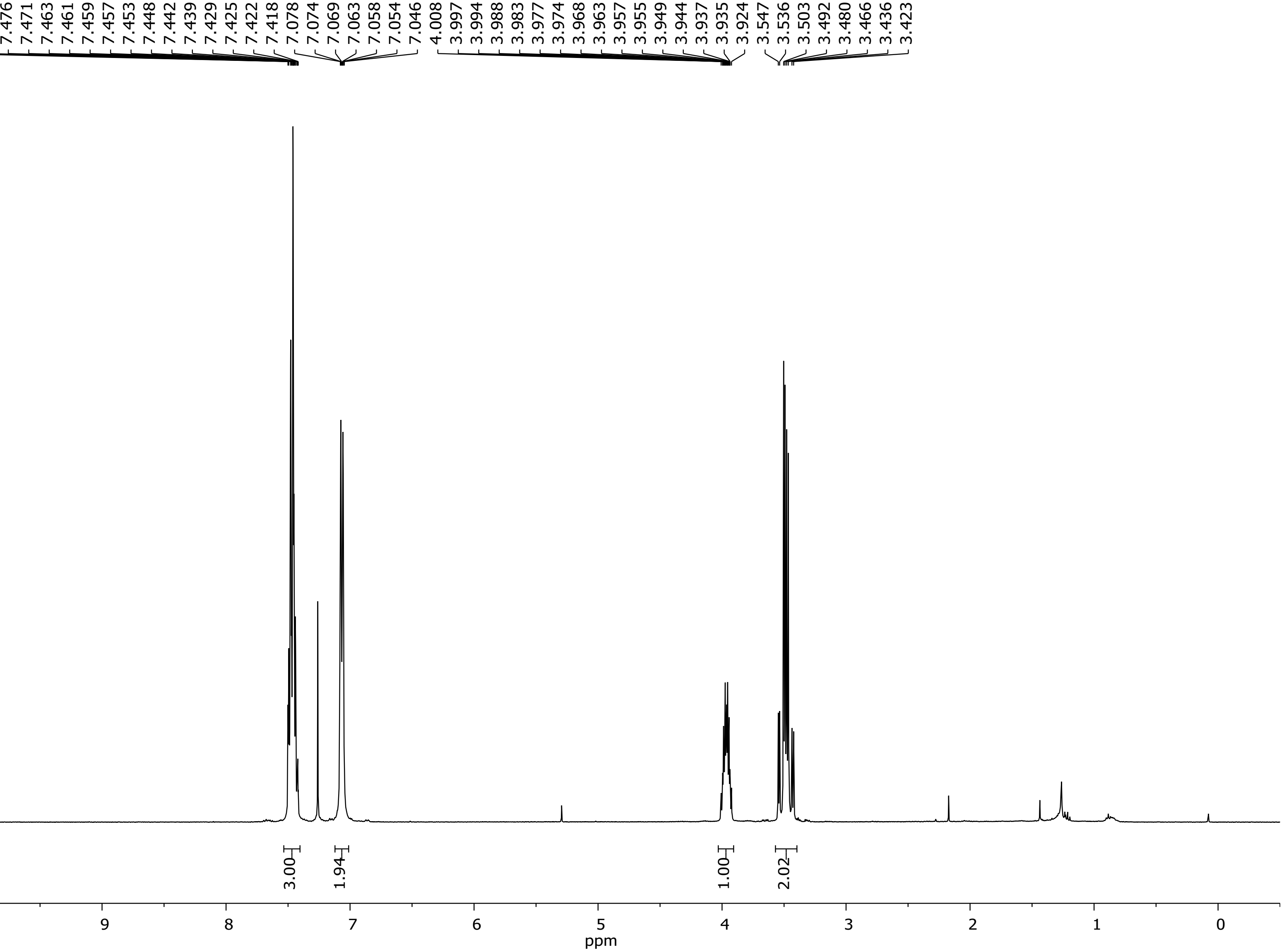

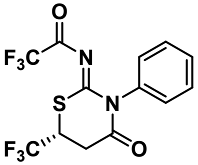

53

$^{19}F\{^{1}H\}$, $CDCl_3$, 377 MHz

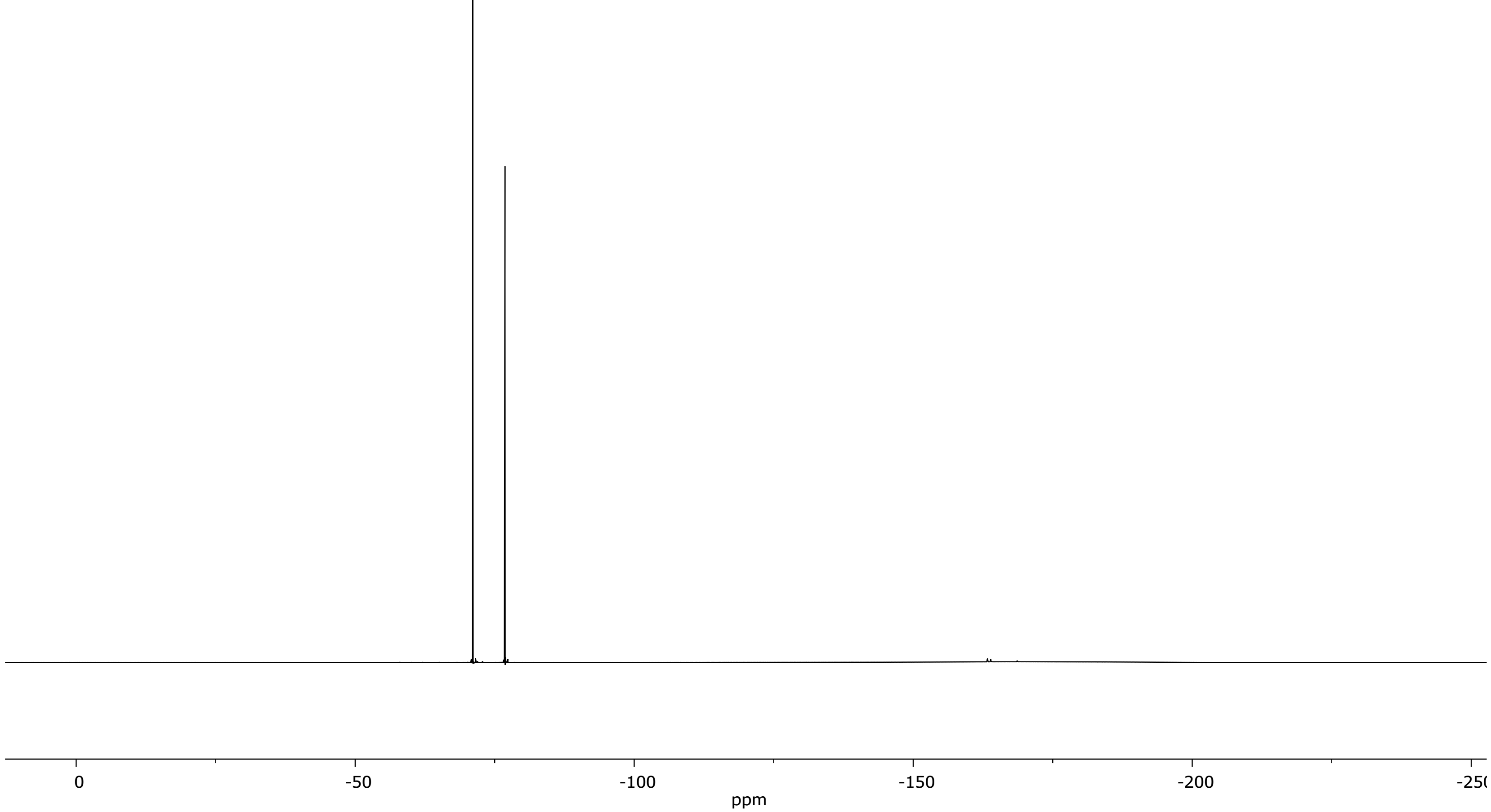

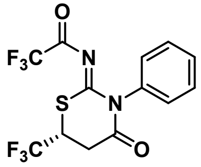

53

$^{13}C\{^{1}H\}$, $CDCl_3$, 126 MHz

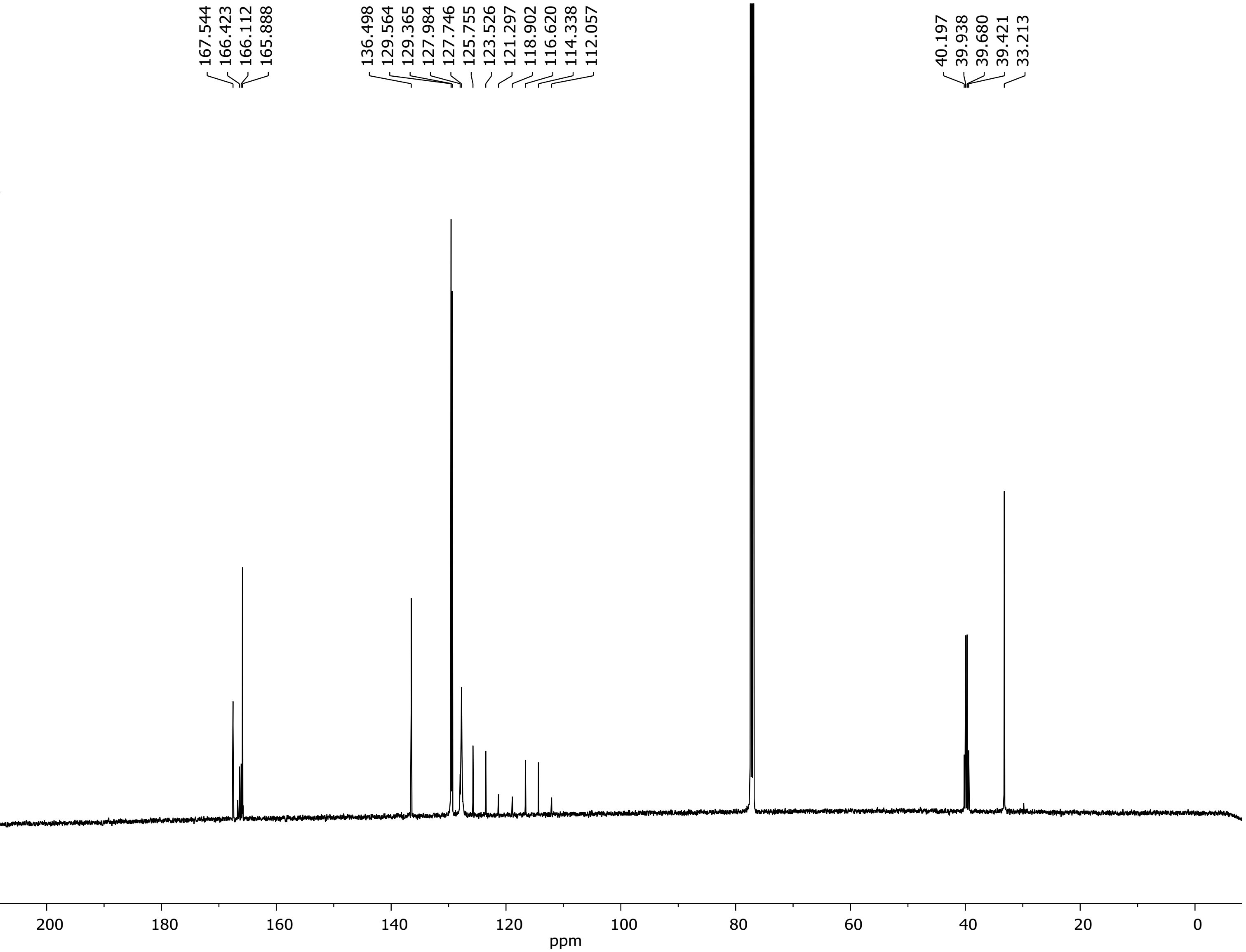

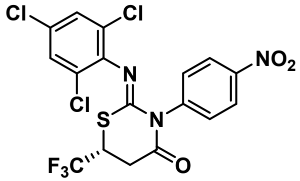

54

^{1}H, $CDCl_3$, 500 MHz

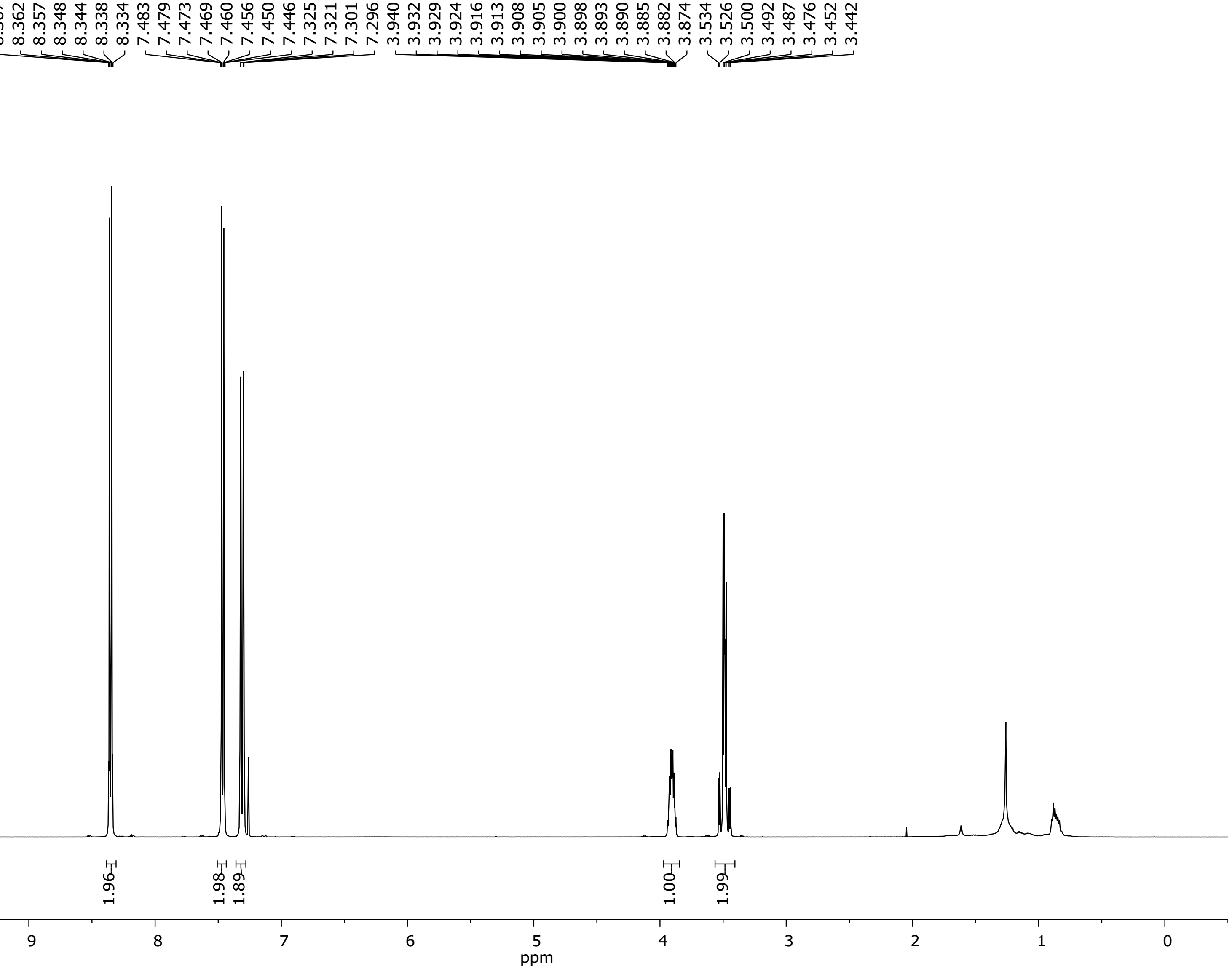

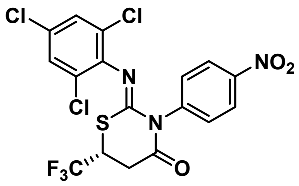

54

$^{19}F\{^1H\}$, $CDCl_3$, 376 MHz

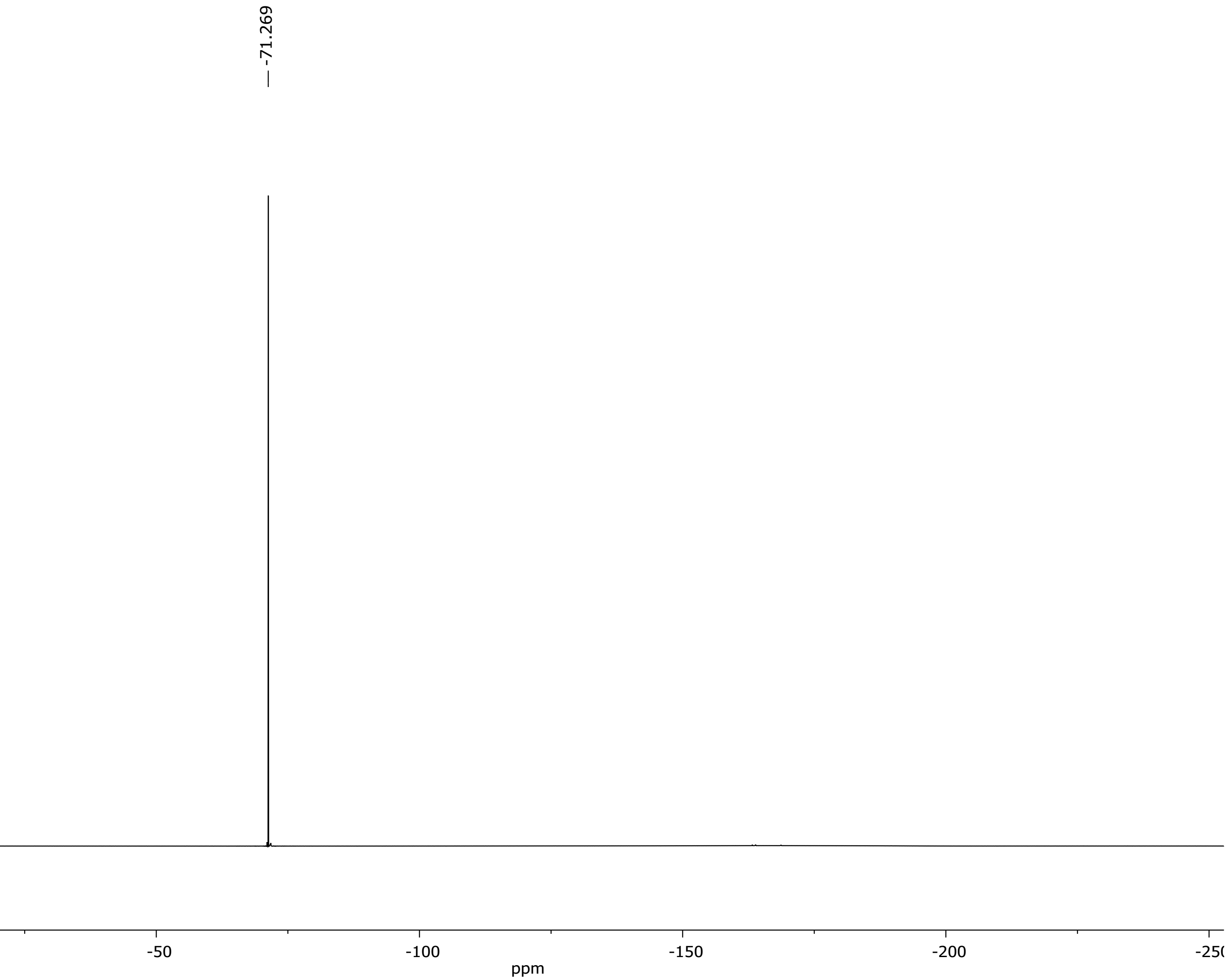

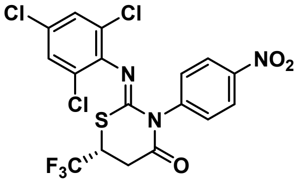

54

$^{13}C\{^1H\}$, $CDCl_3$, 126 MHz

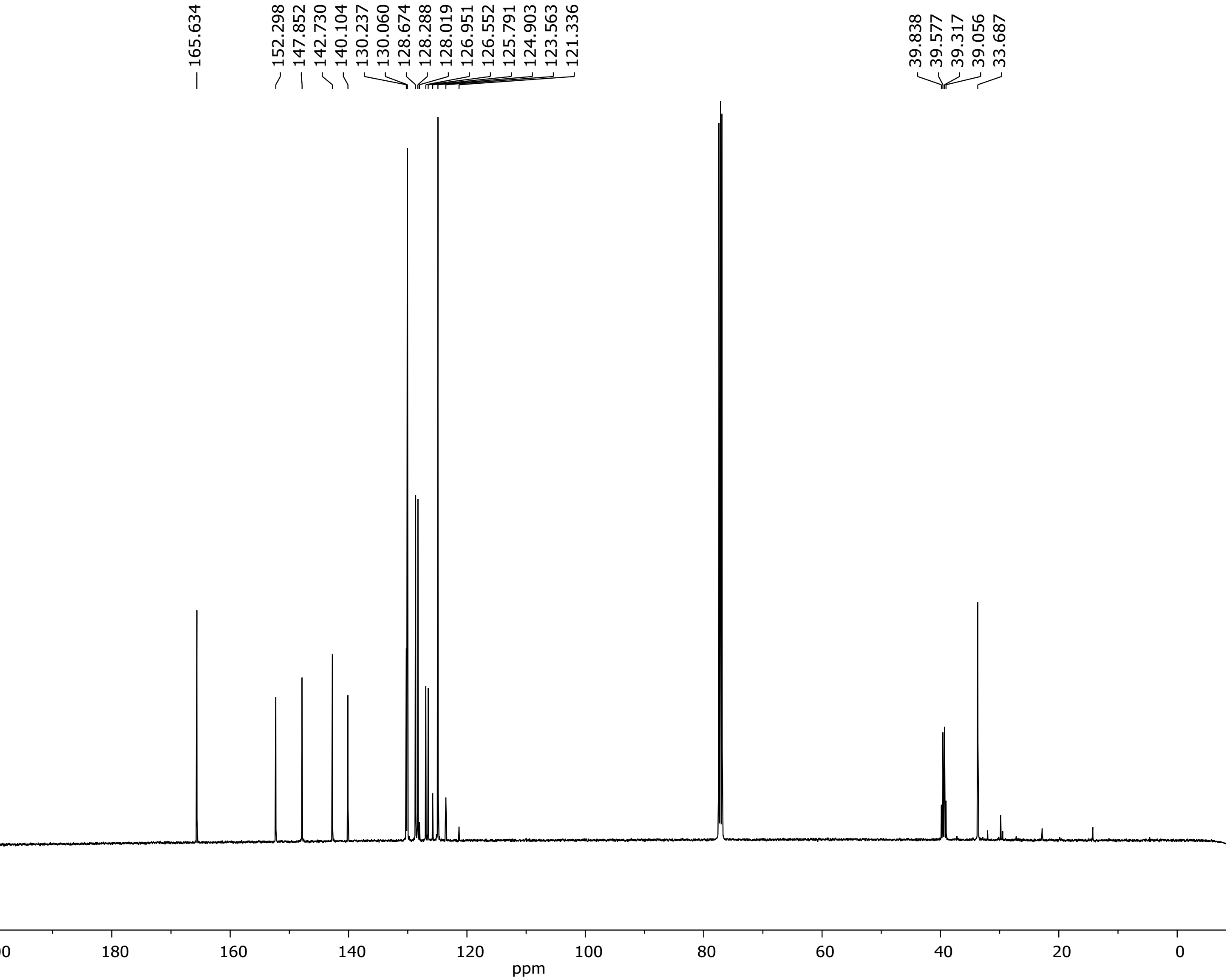

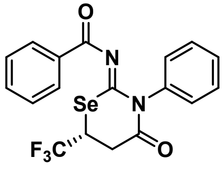

55

^{1}H, $CDCl_3$, 500 MHz

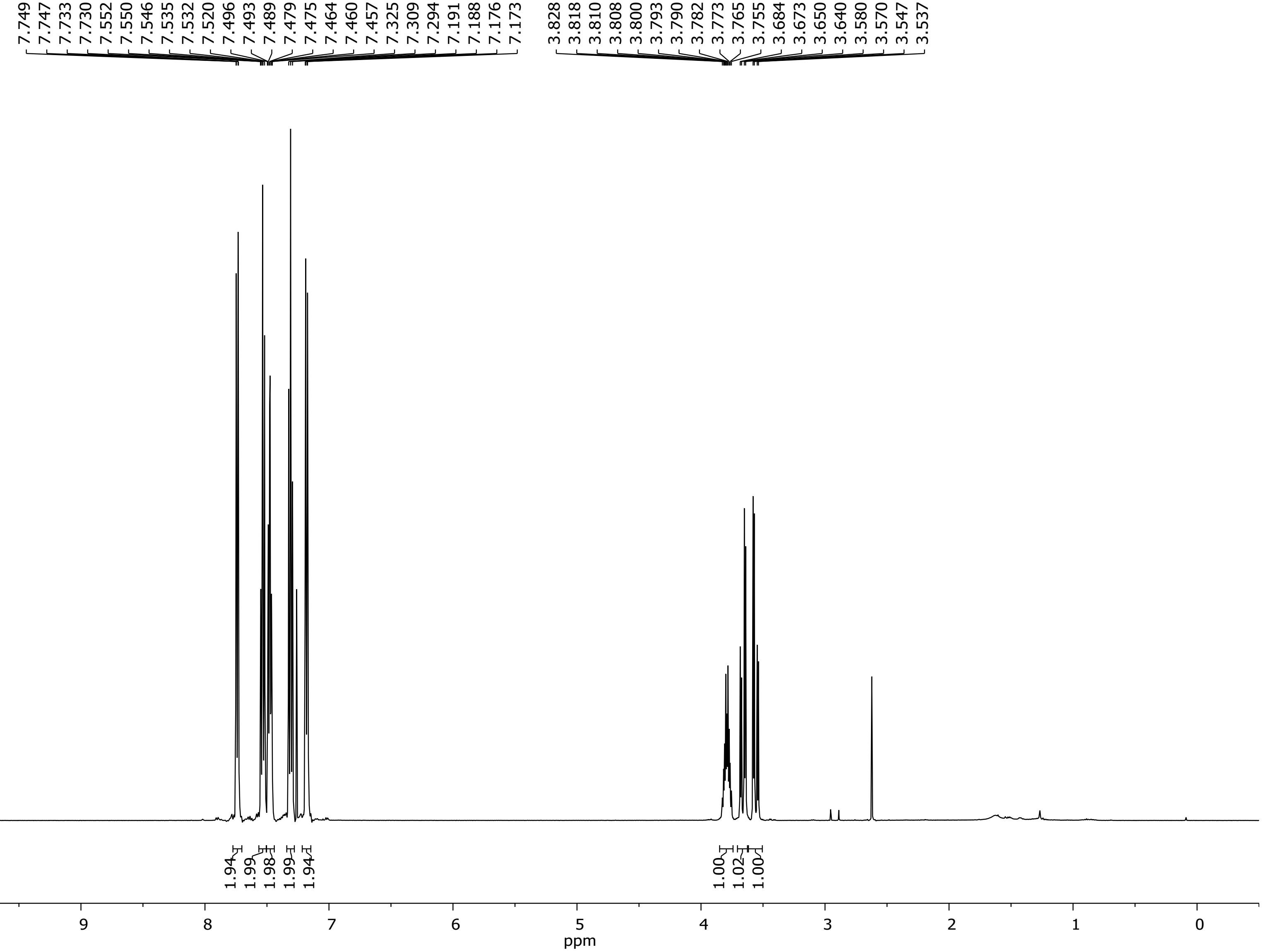

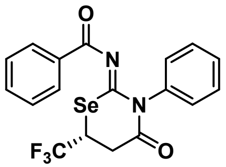

55

$^{19}F\{^1H\}$, $CDCl_3$, 470 MHz

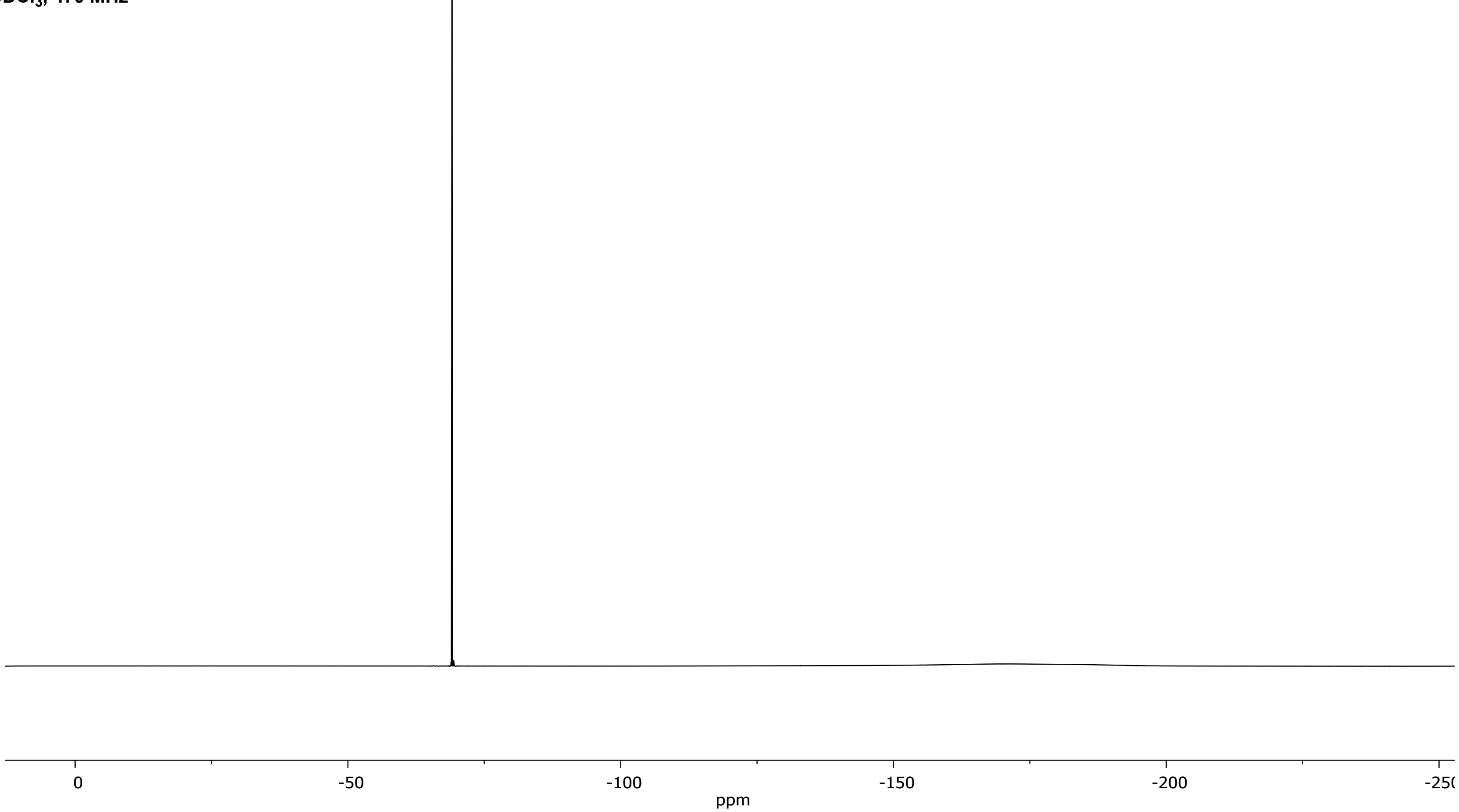

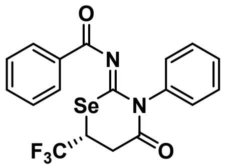

55

$^{13}C\{^{1}H\}$, $CDCl_3$, 126 MHz

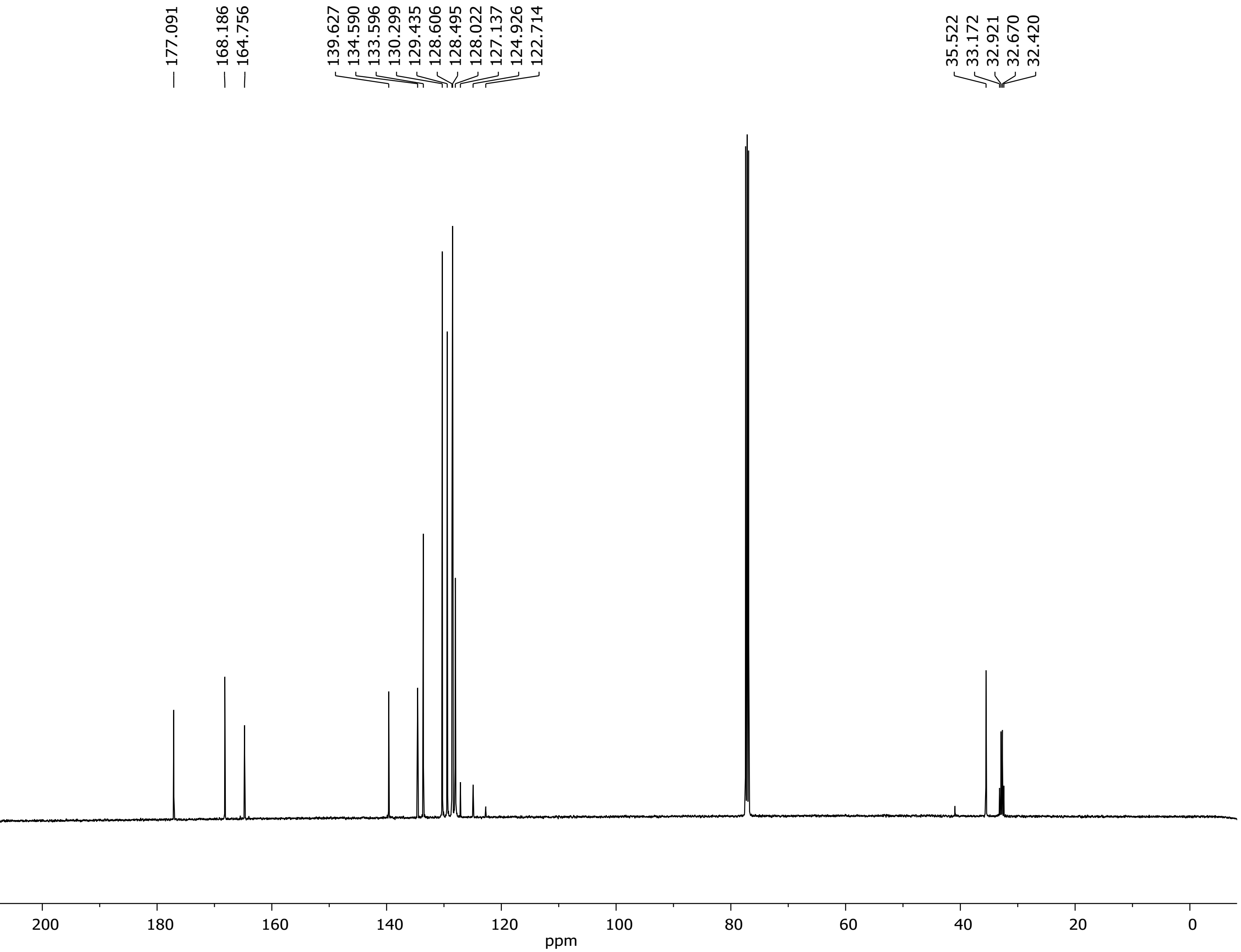

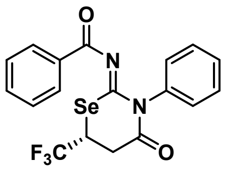

55

^{77}Se{^{1}H}, CDCl$_3$, 95 MHz

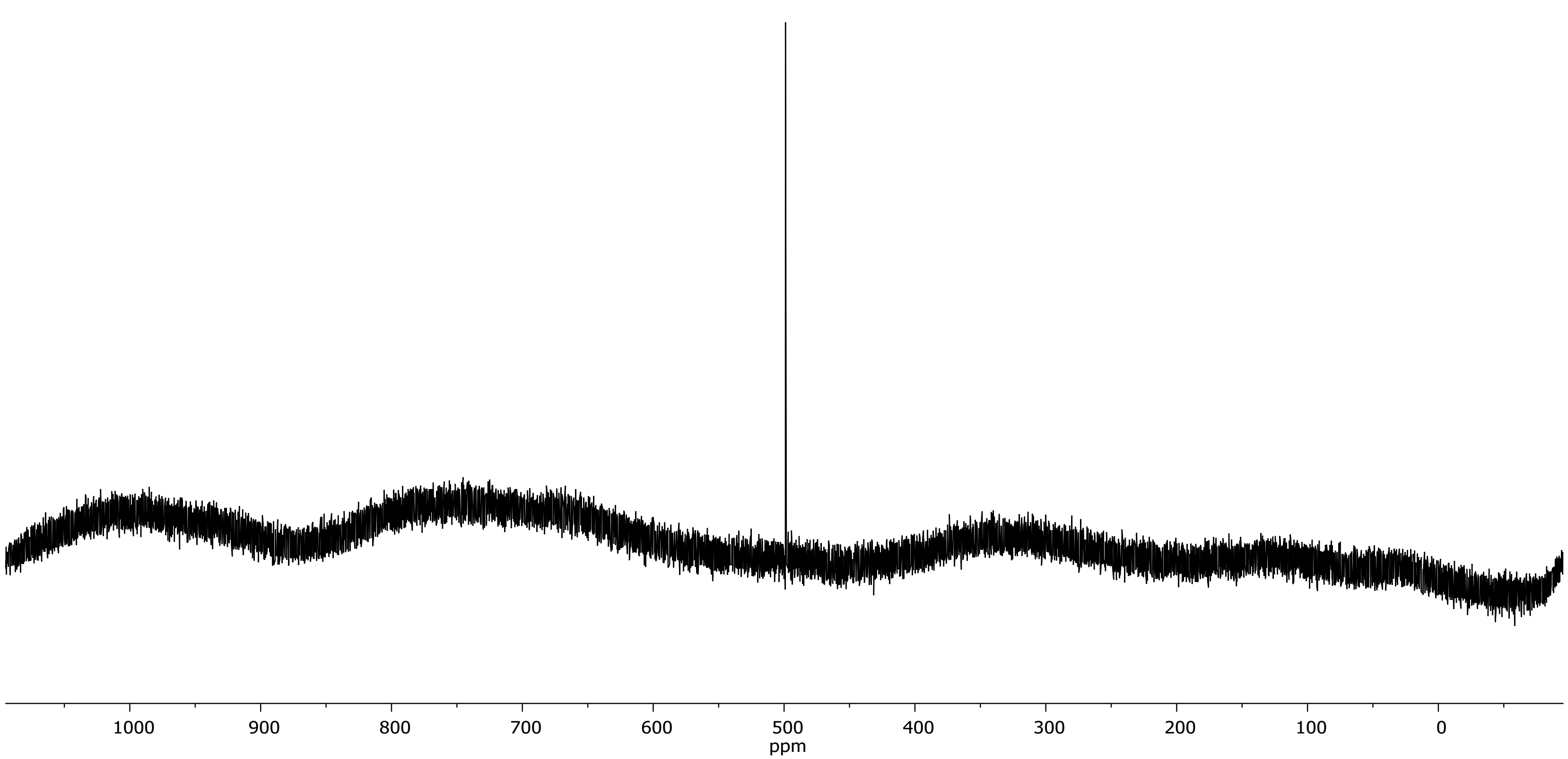

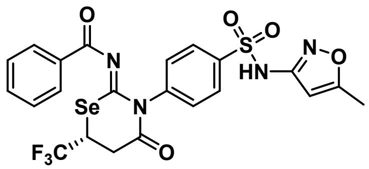

56

^{1}H, CD_3CN, 500 MHz

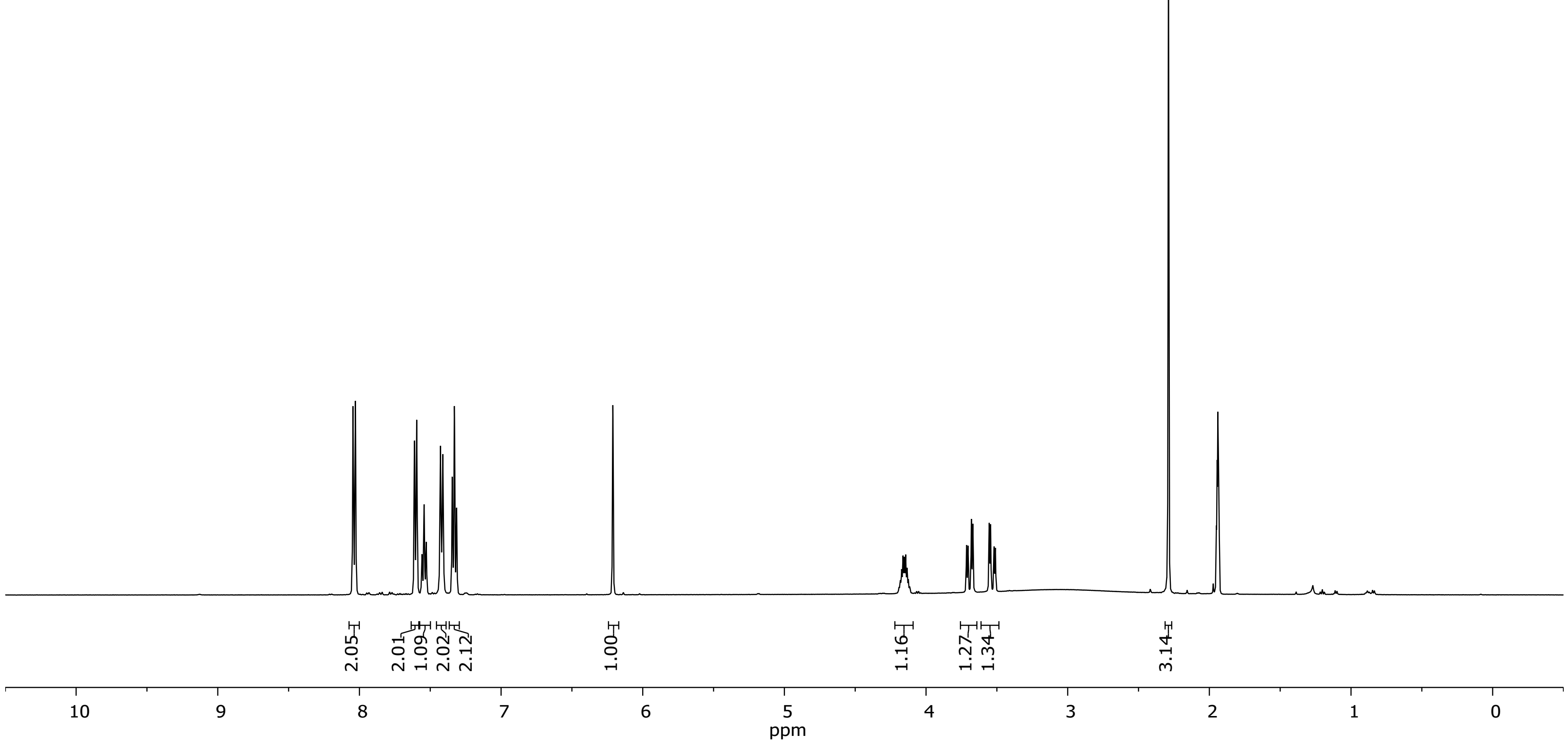

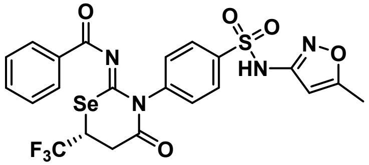

56

$^{19}F\{^1H\}$, CD_3CN, 470 MHz

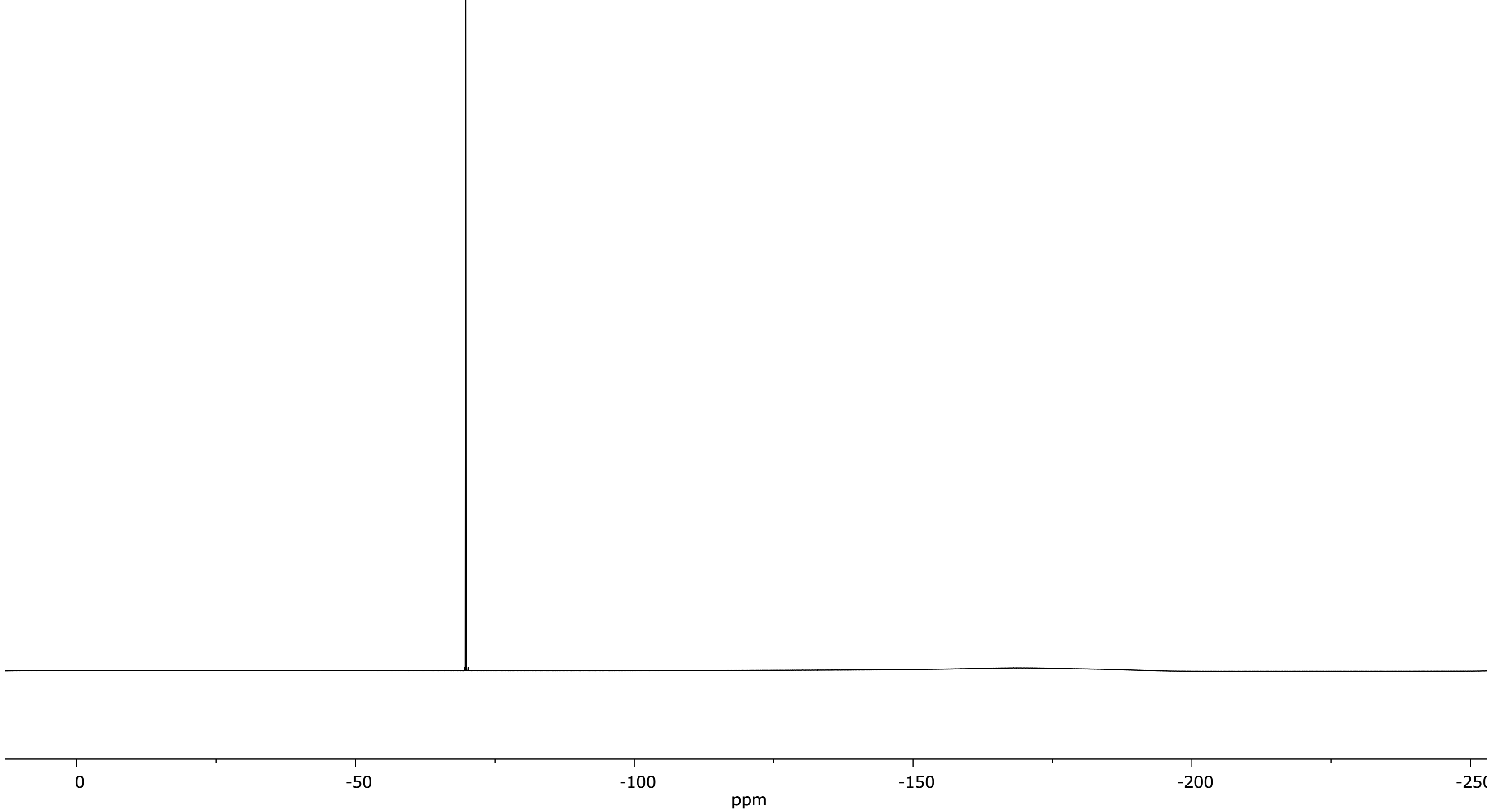

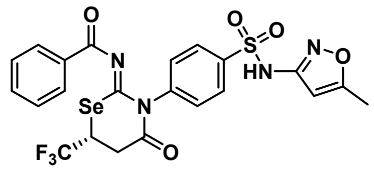

56

$^{13}C\{^{1}H\}$, CD_3CN, 126 MHz

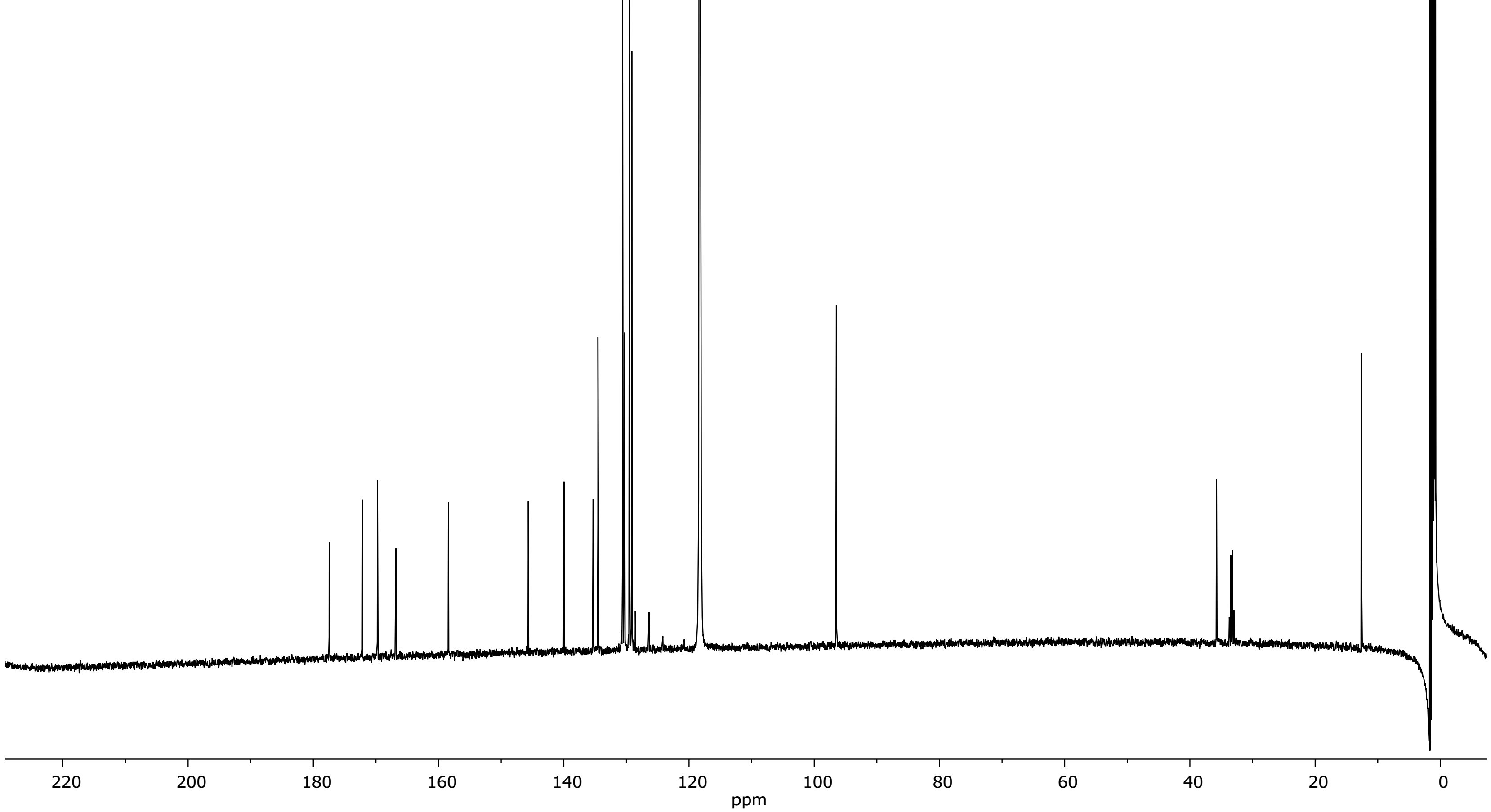

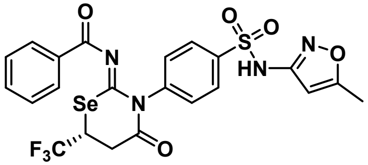

56

$^{77}Se\{^{1}H\}$, CD_3CN, 95 MHz

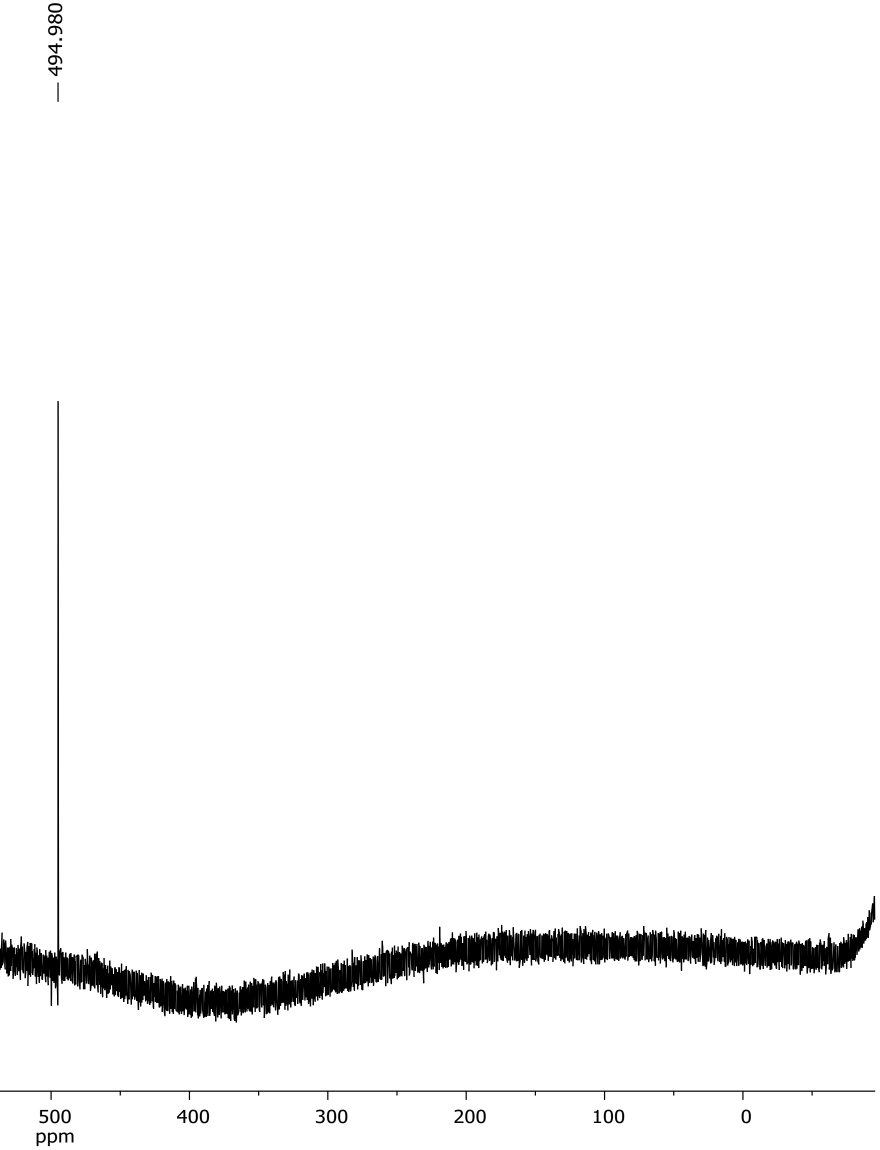

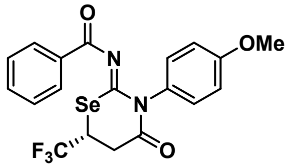

57
^{1}H, CDCl$_3$, 500 MHz

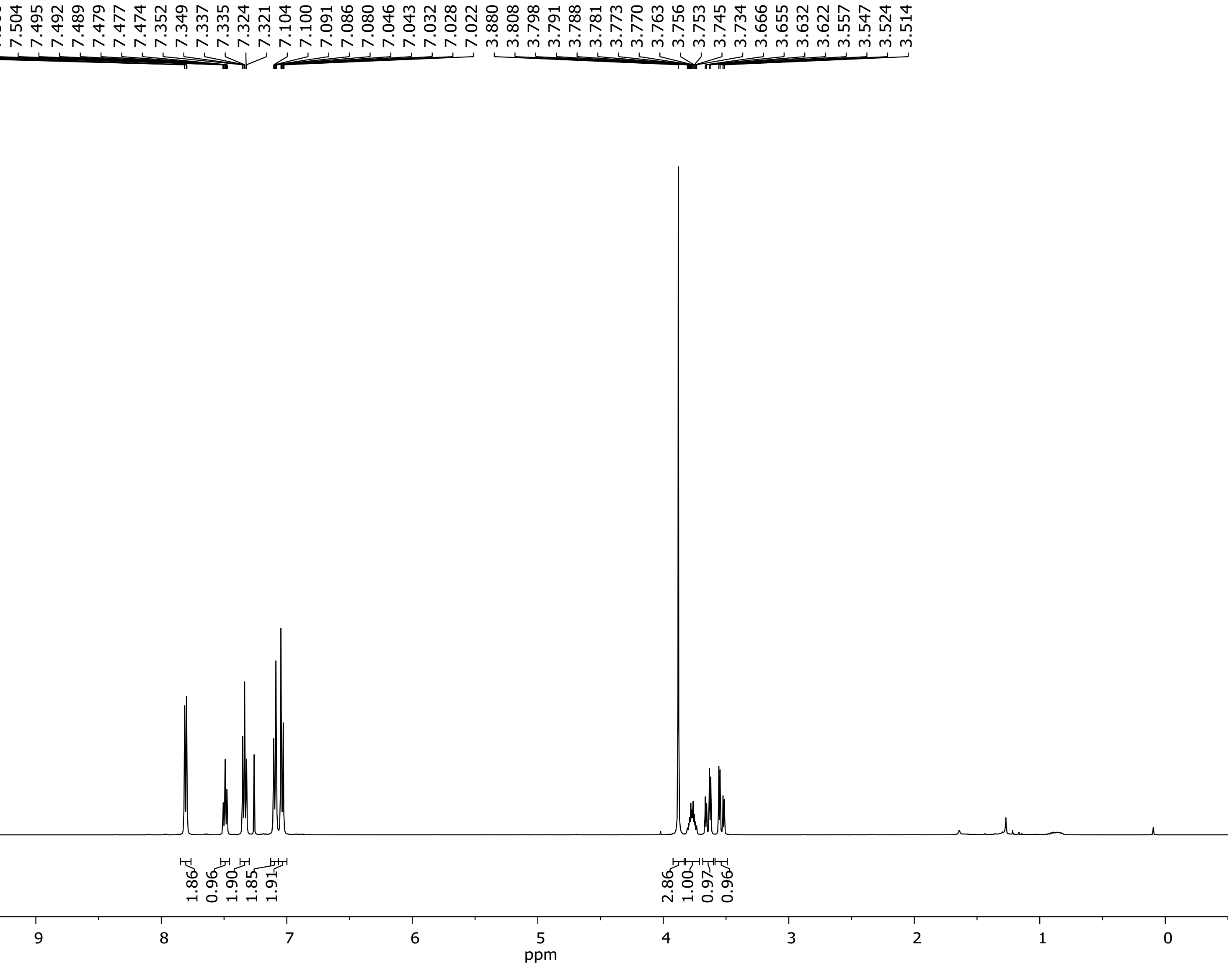

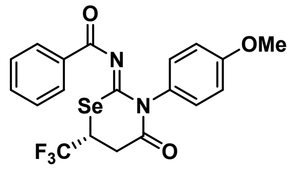

57

$^{19}F\{^{1}H\}$, $CDCl_3$, 376 MHz

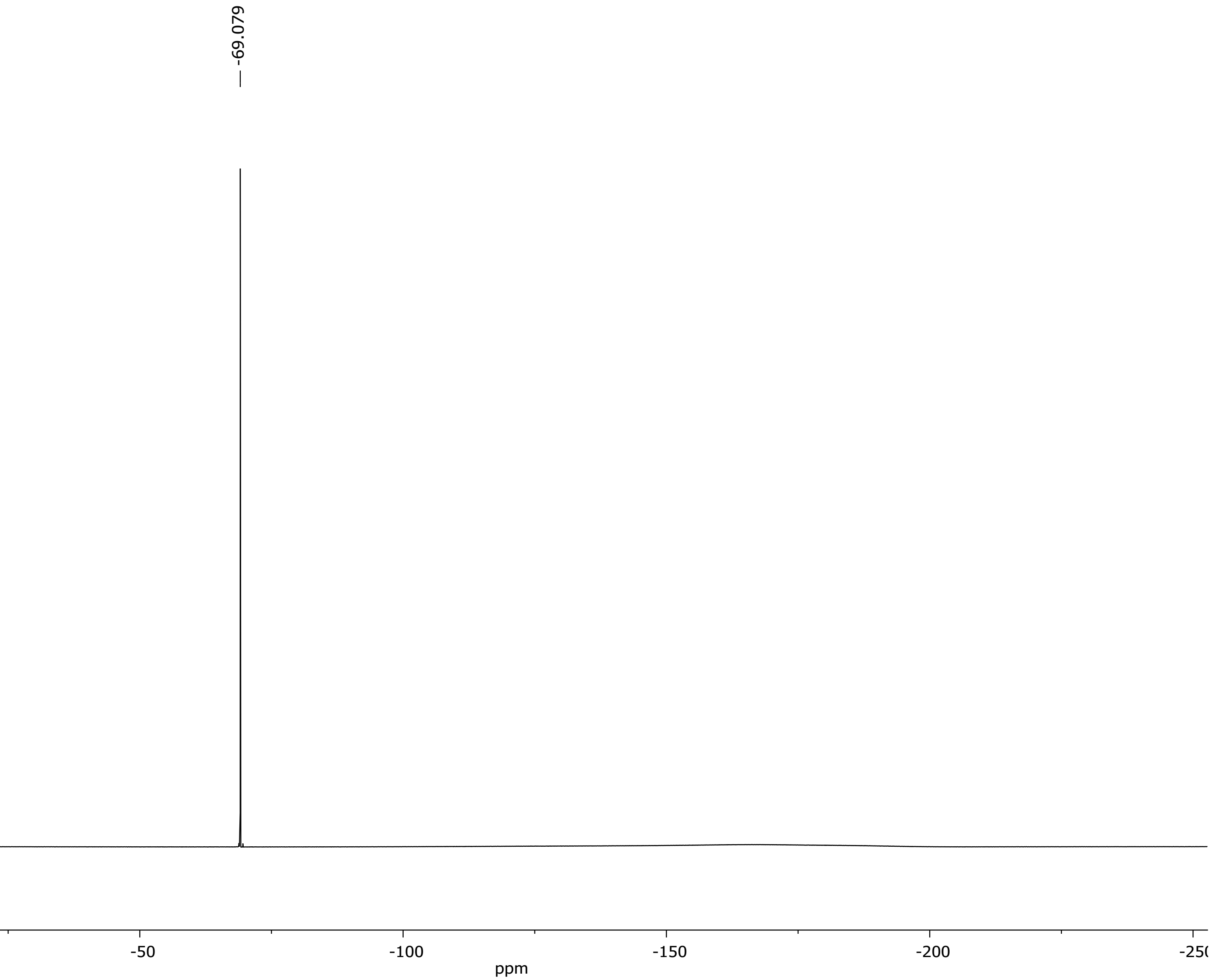

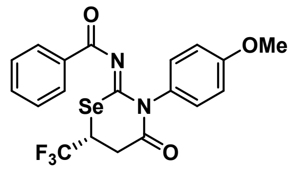

57

$^{13}C\{^1H\}$, $CDCl_3$, 126 MHz

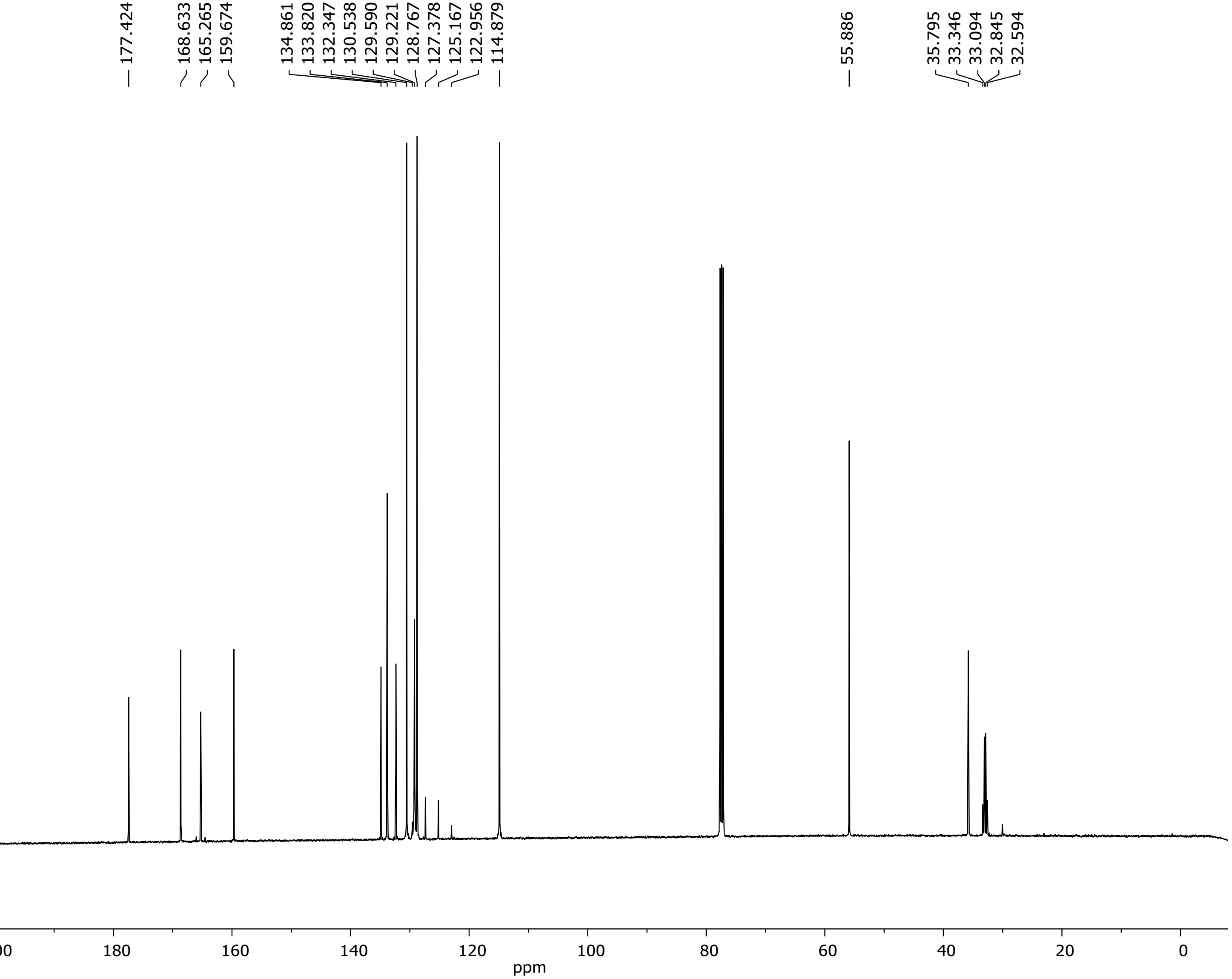

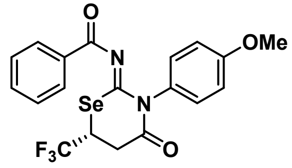

57

^{77}Se{^{1}H}, CDCl$_3$, 95 MHz

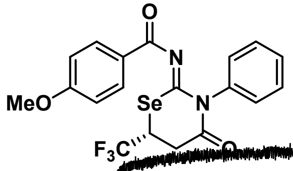

58

^{1}H, CDCl$_3$, 400 MHz

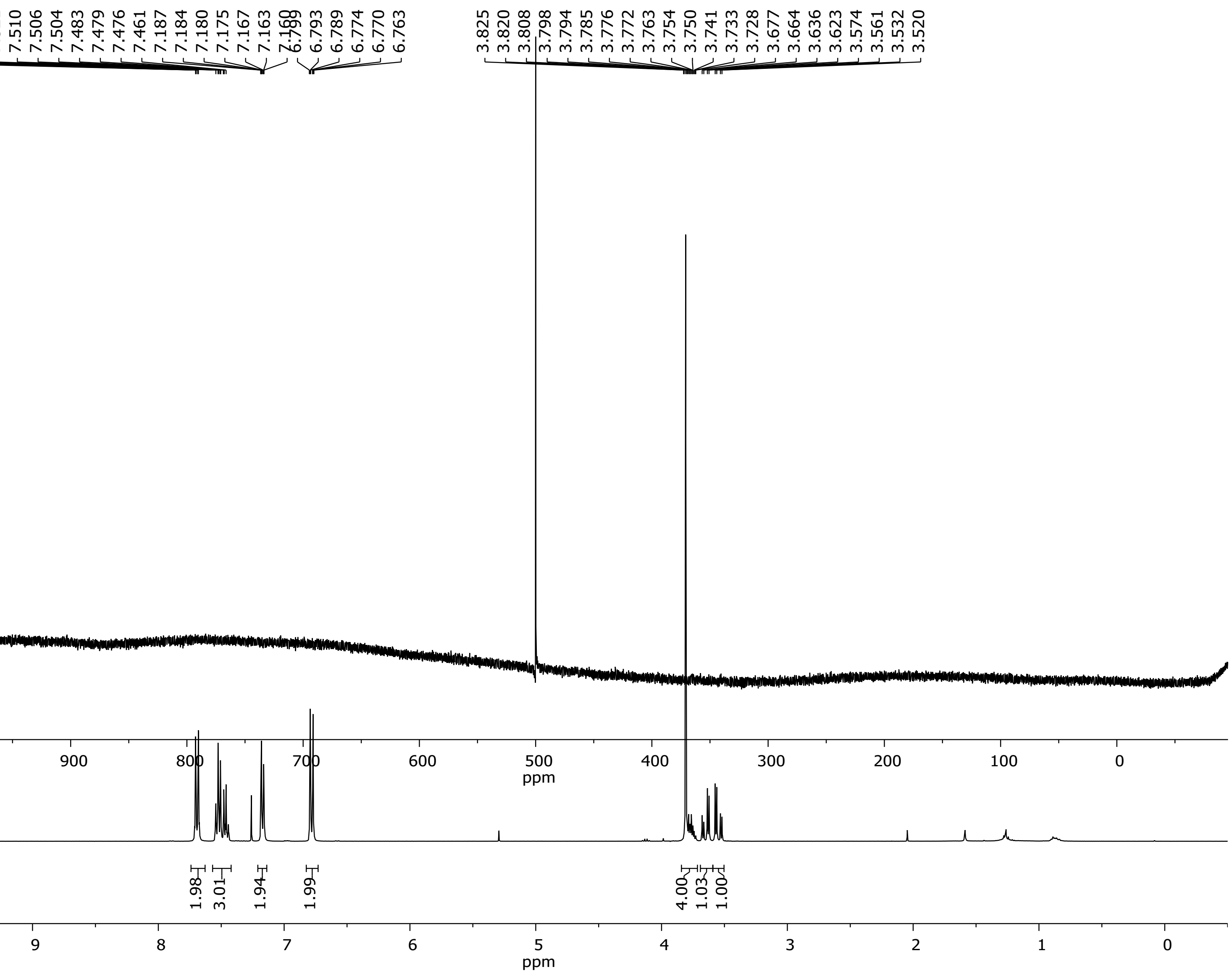

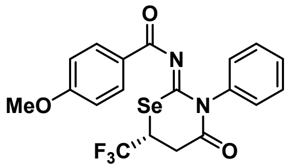

58

$^{19}F\{^{1}H\}$, $CDCl_3$, 377 MHz

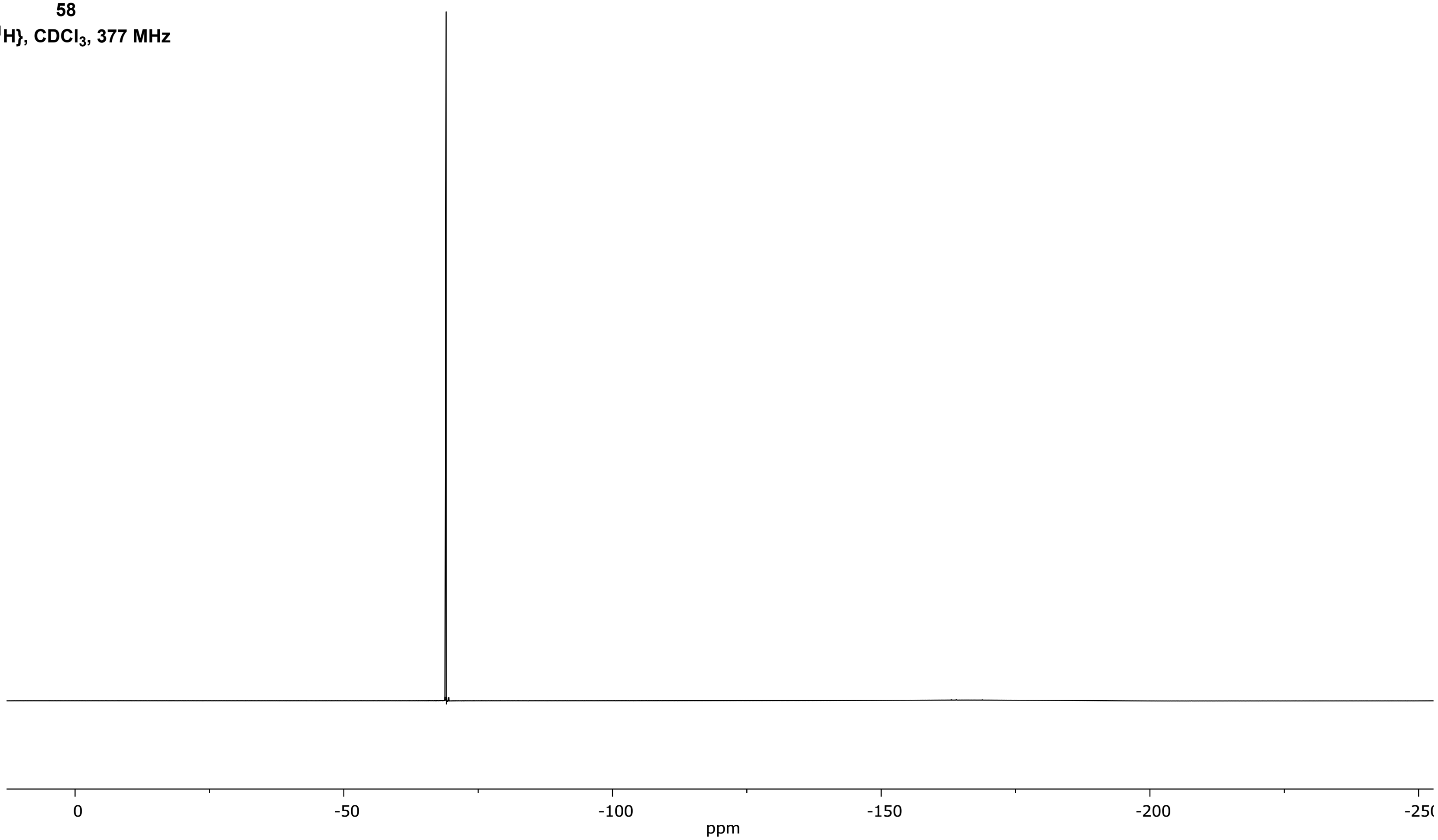

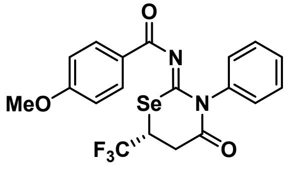

58

$^{13}C\{^{1}H\}$, $CDCl_3$, 126 MHz

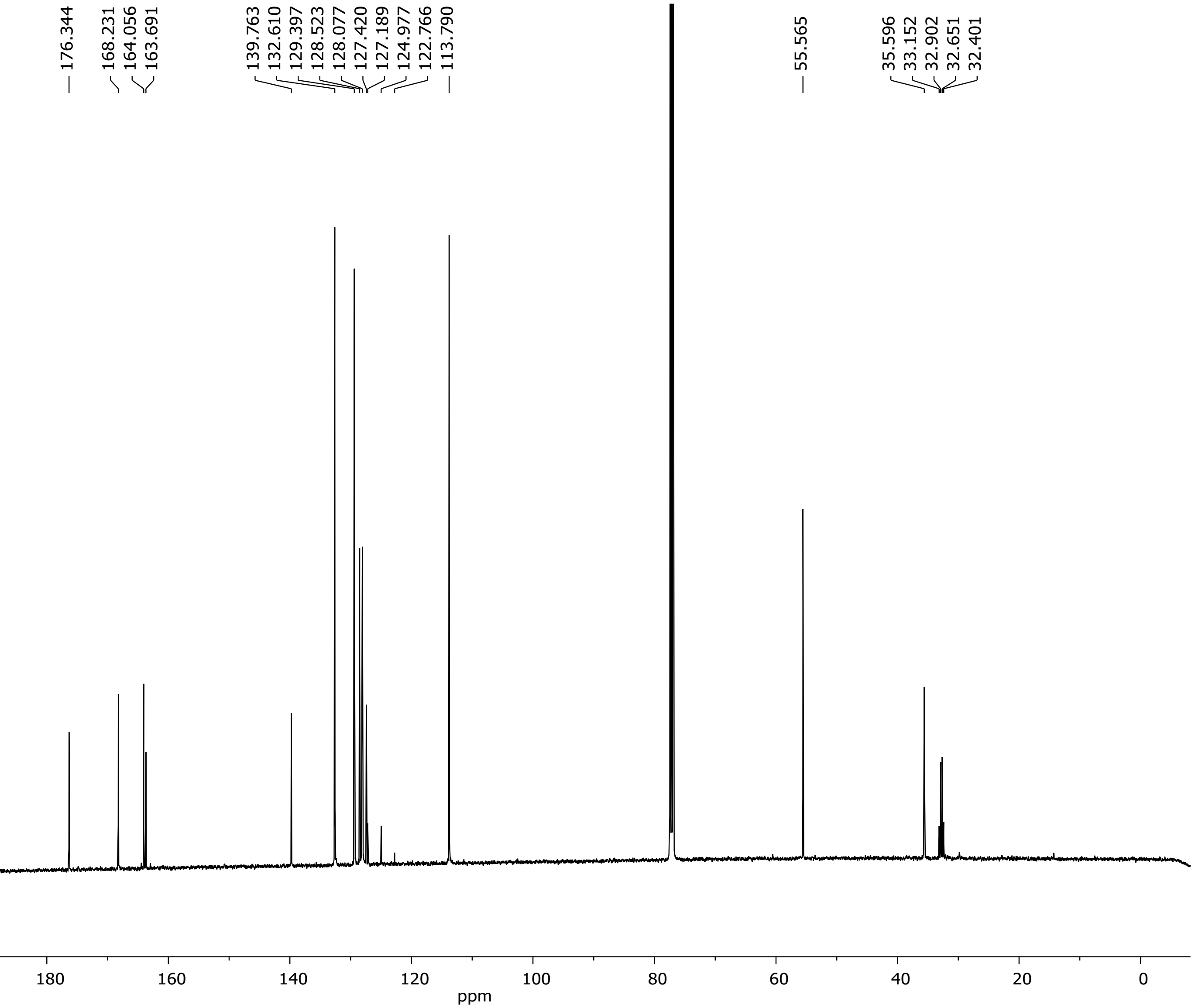

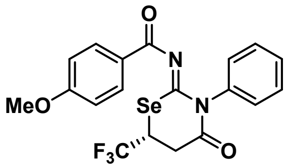

58

^{77}Se{^{1}H}, CDCl$_3$, 95 MHz

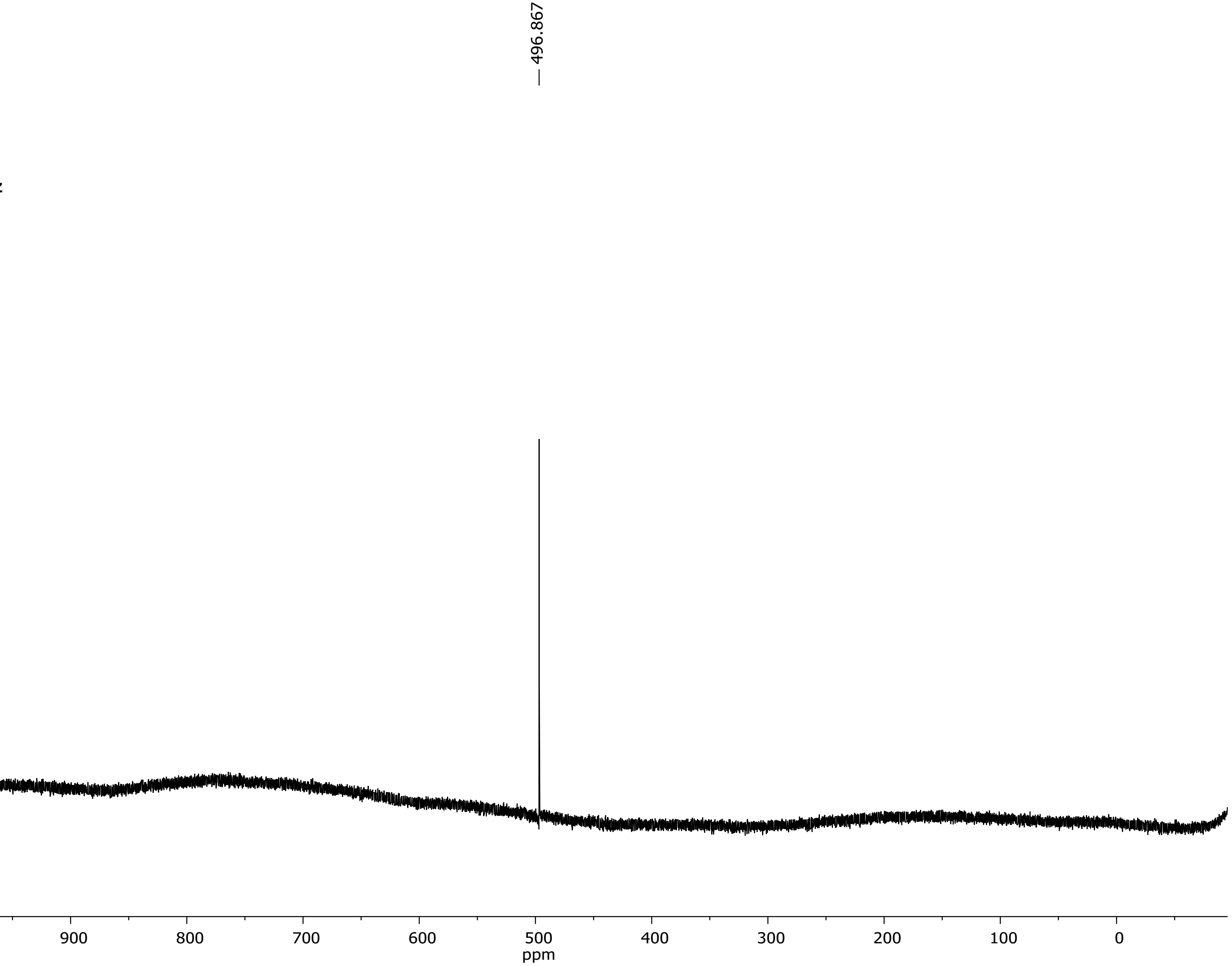

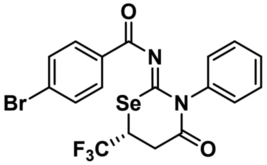

59

^1H, $CDCl_3$, 400 MHz

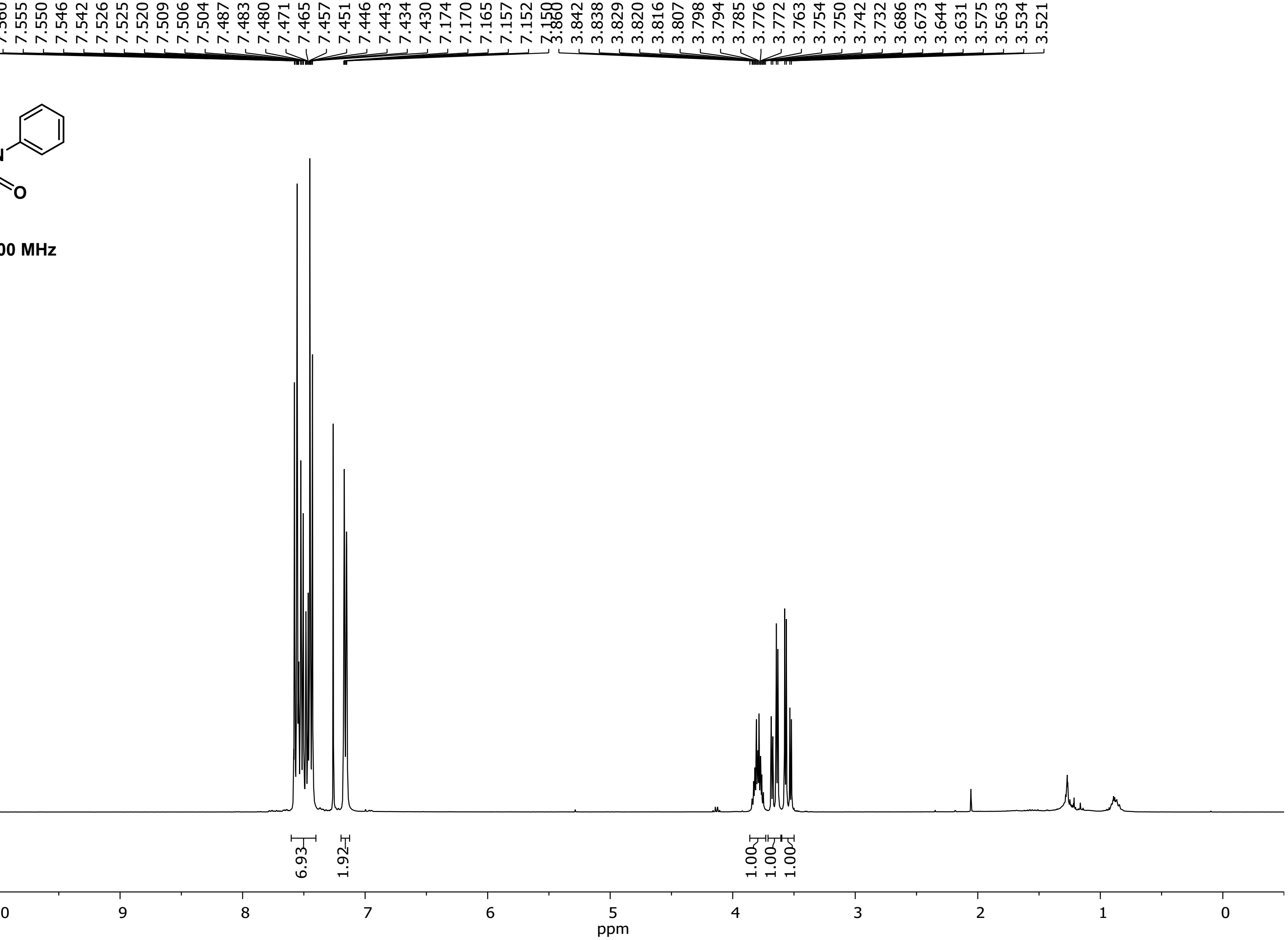

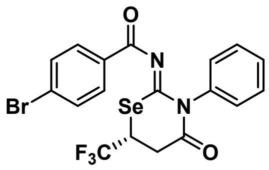

59

$^{19}F\{^1H\}$, $CDCl_3$, 377 MHz

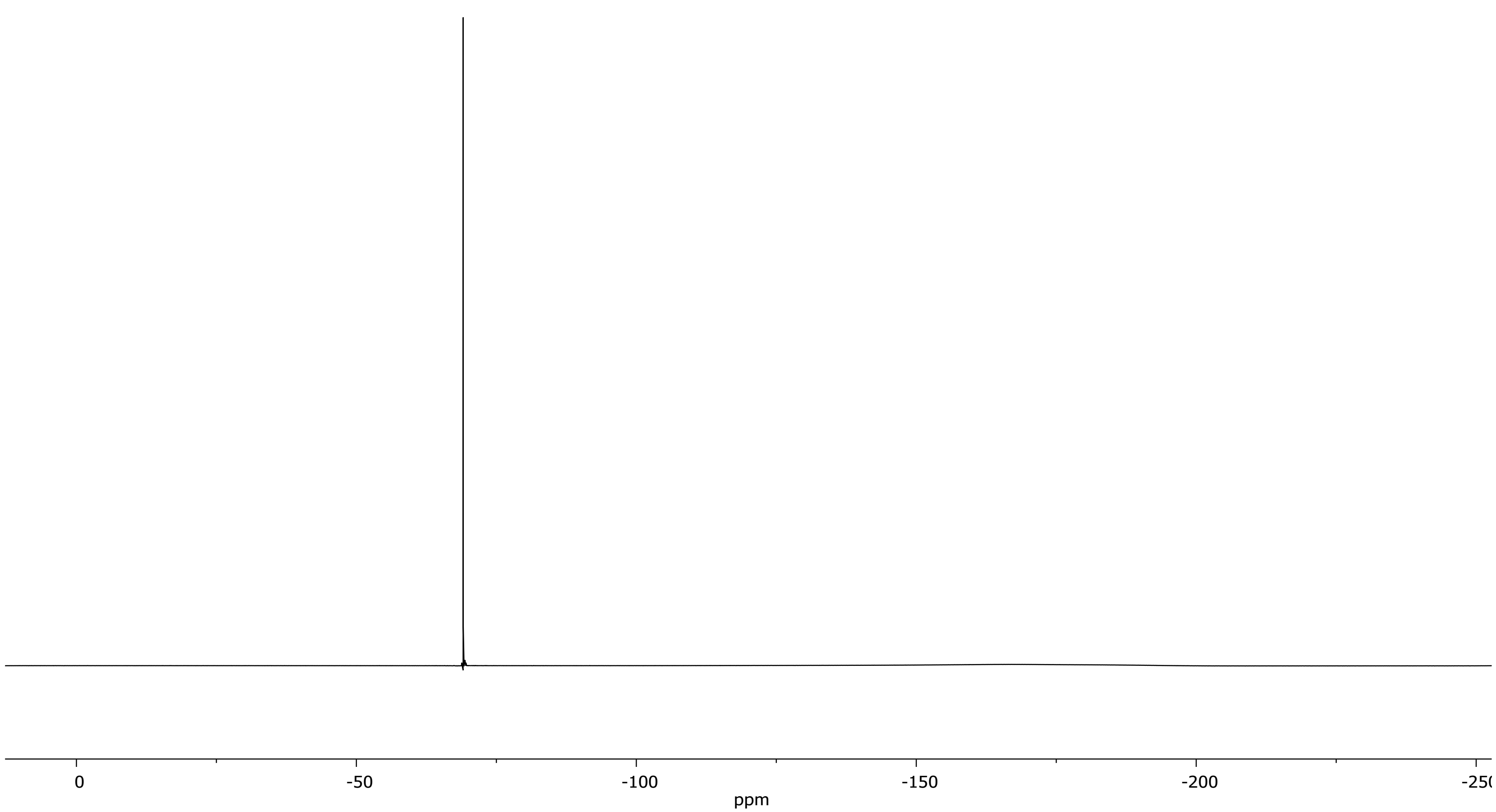

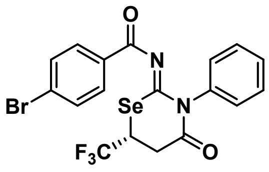

59

$^{13}C\{^{1}H\}$, $CDCl_3$, 126 MHz

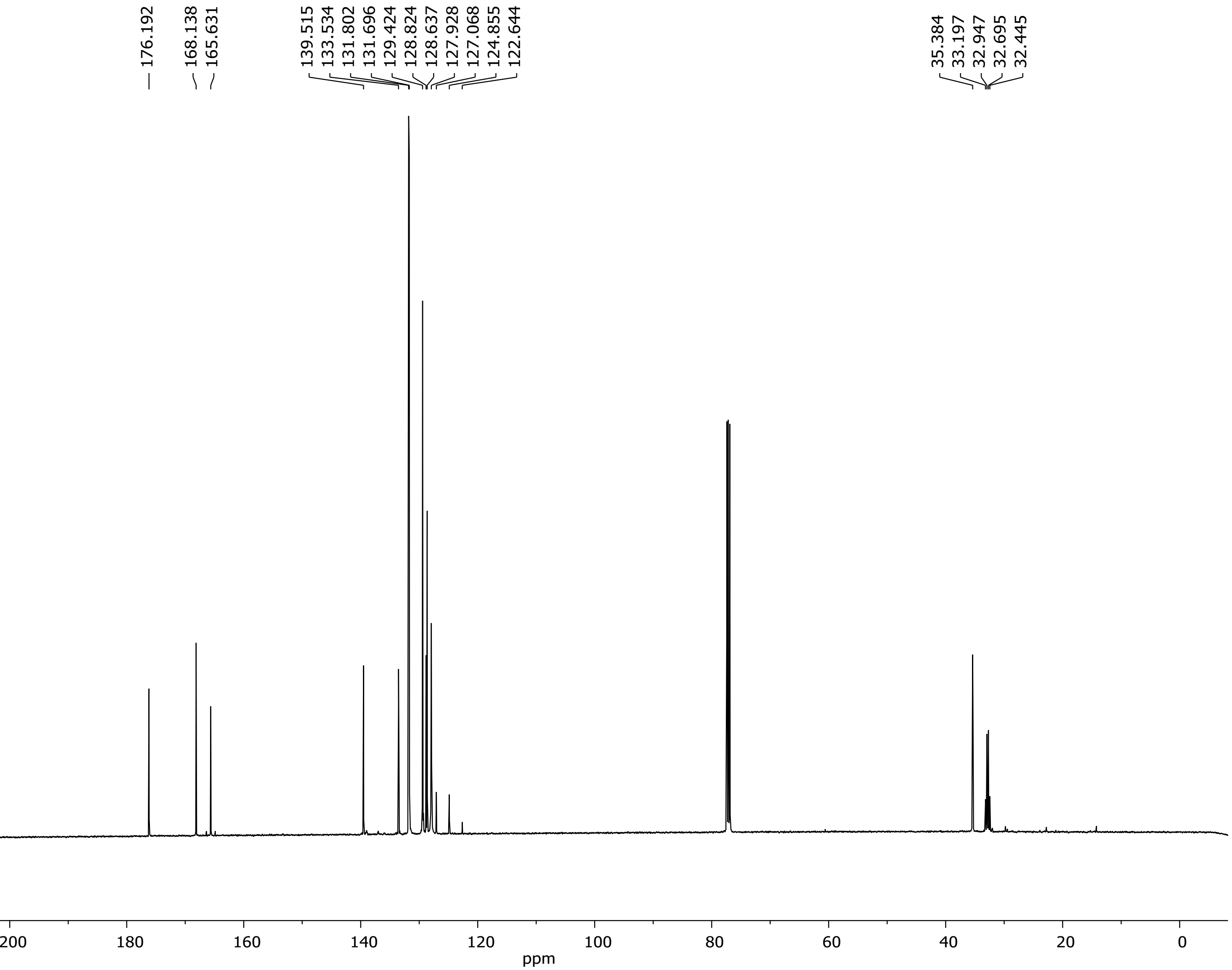

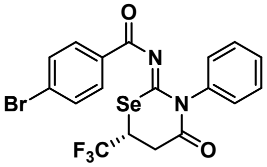

59

$^{77}Se\{^1H\}$, $CDCl_3$, 95 MHz

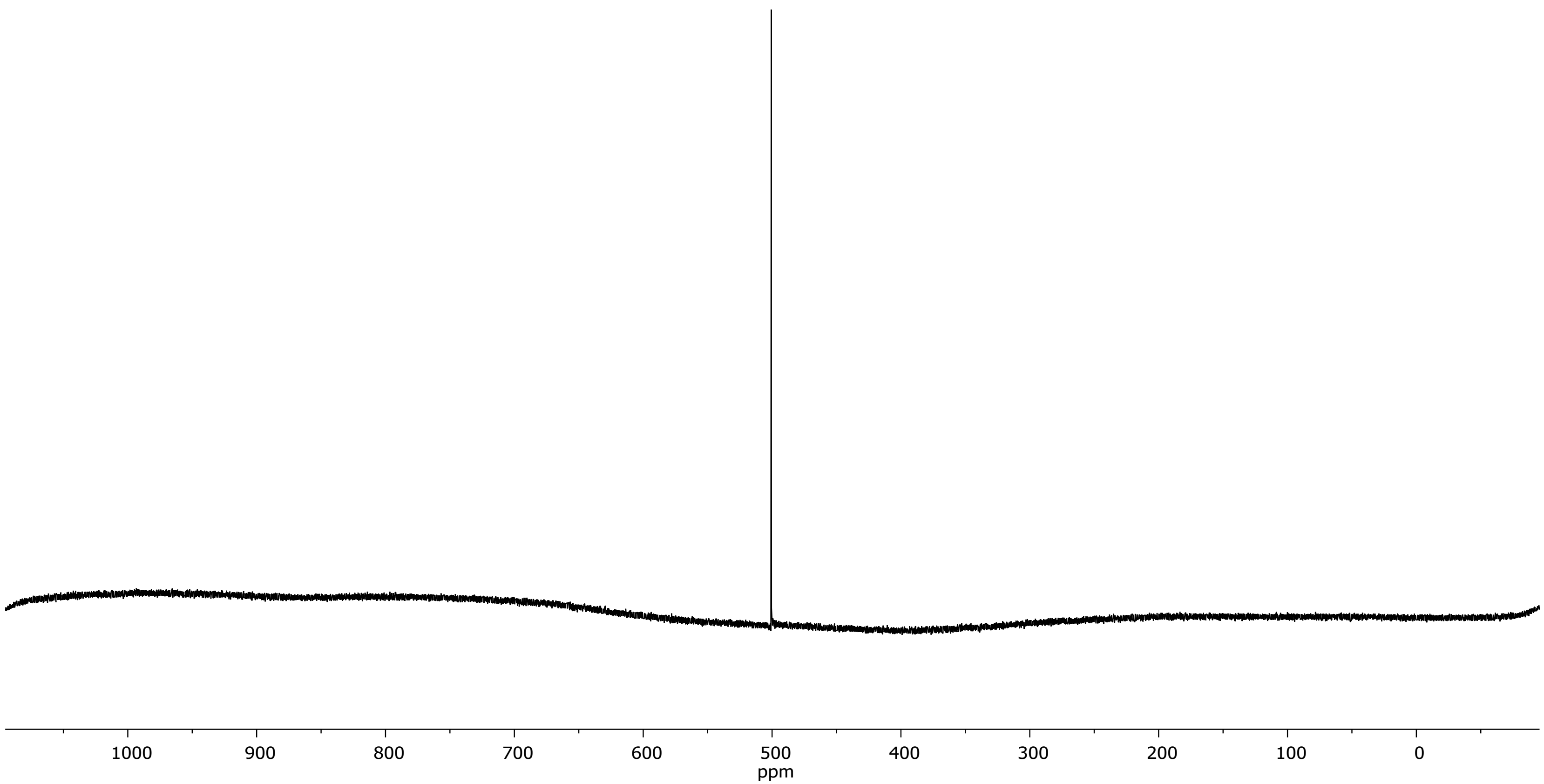

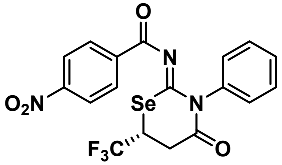

60

^{1}H, $CDCl_3$, 400 MHz

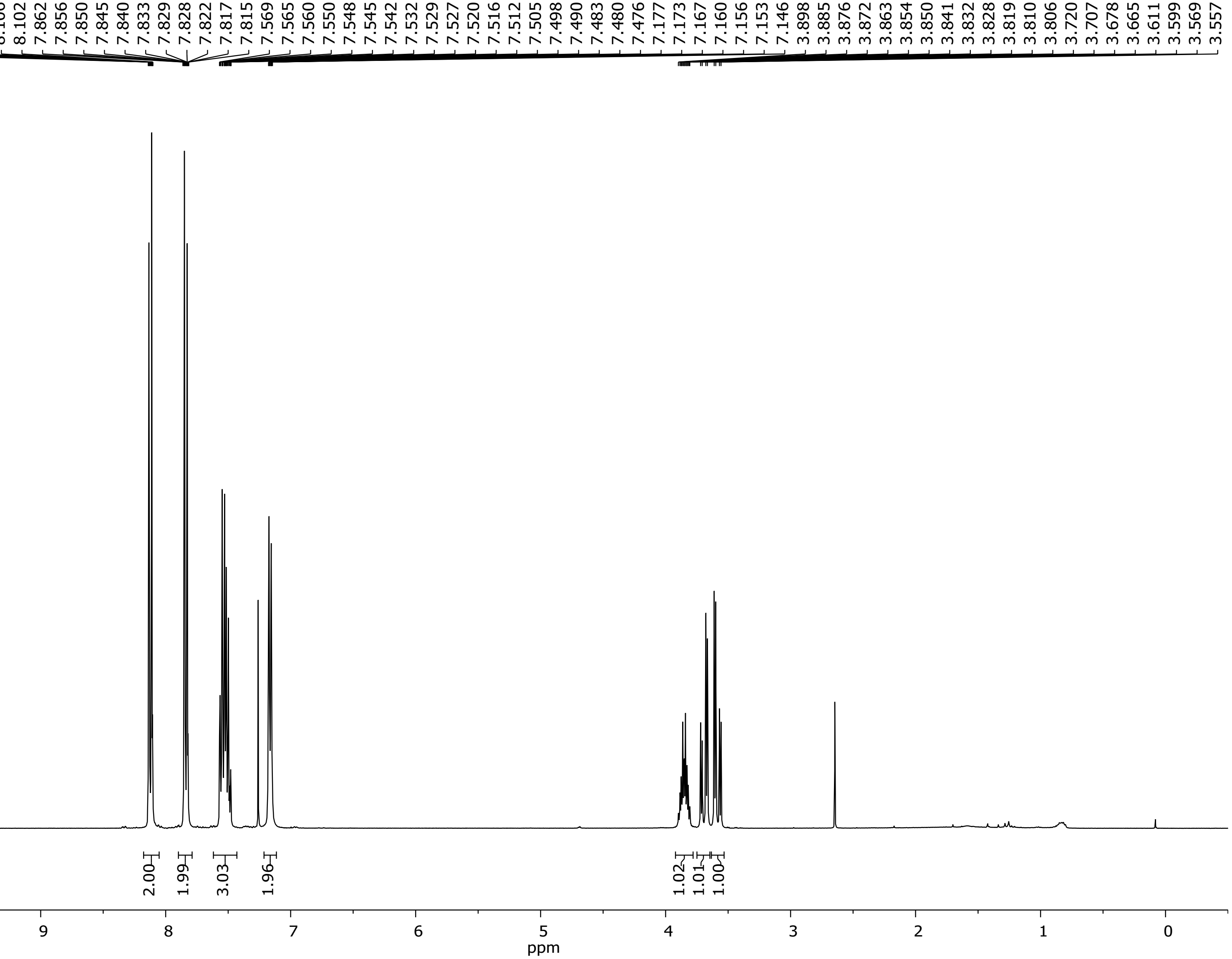

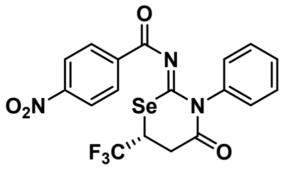

60

$^{19}F\{^1H\}$, $CDCl_3$, 377 MHz

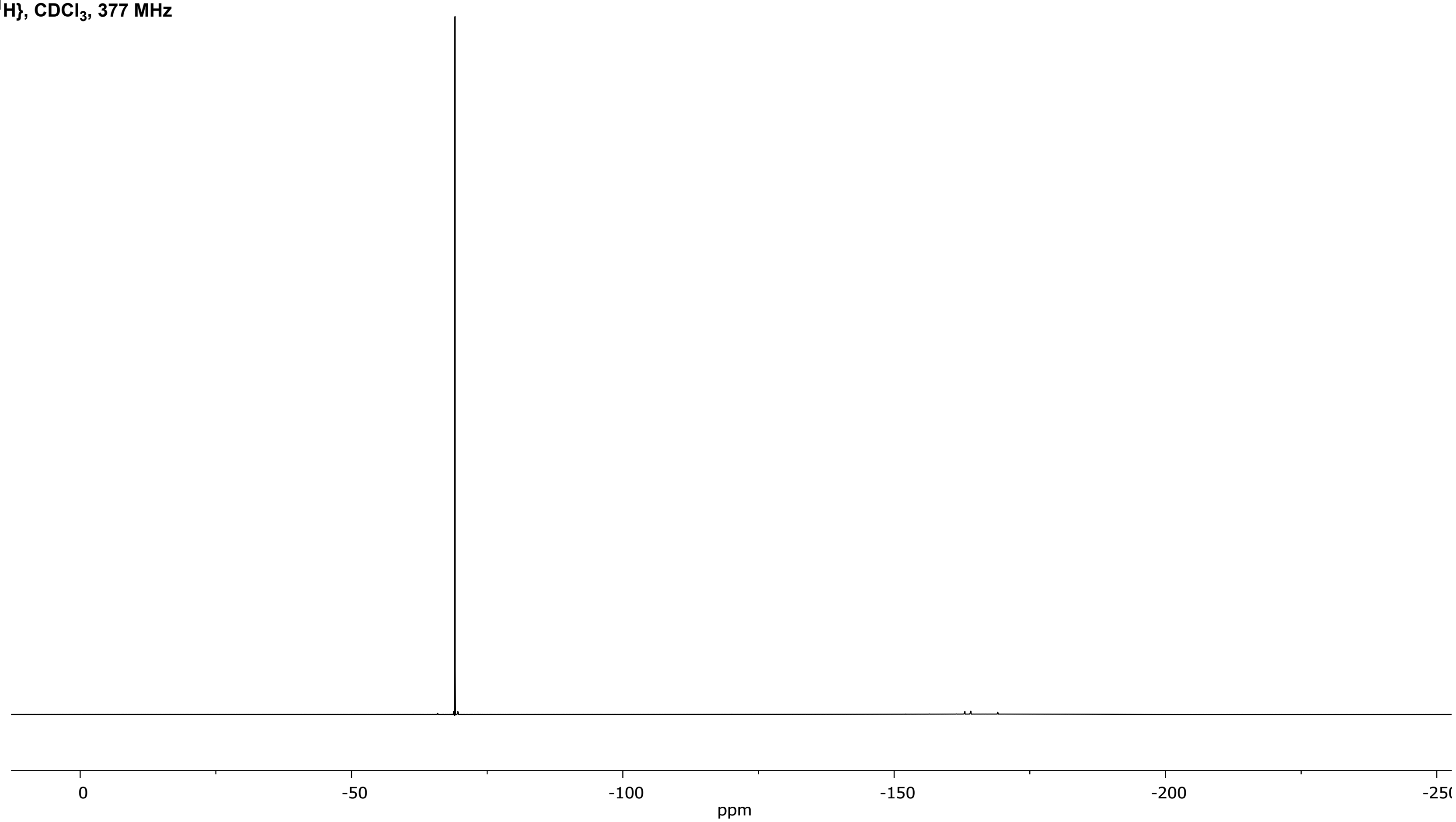

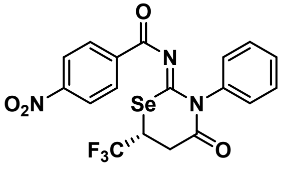

60

$^{13}C\{^1H\}$, $CDCl_3$, 126 MHz

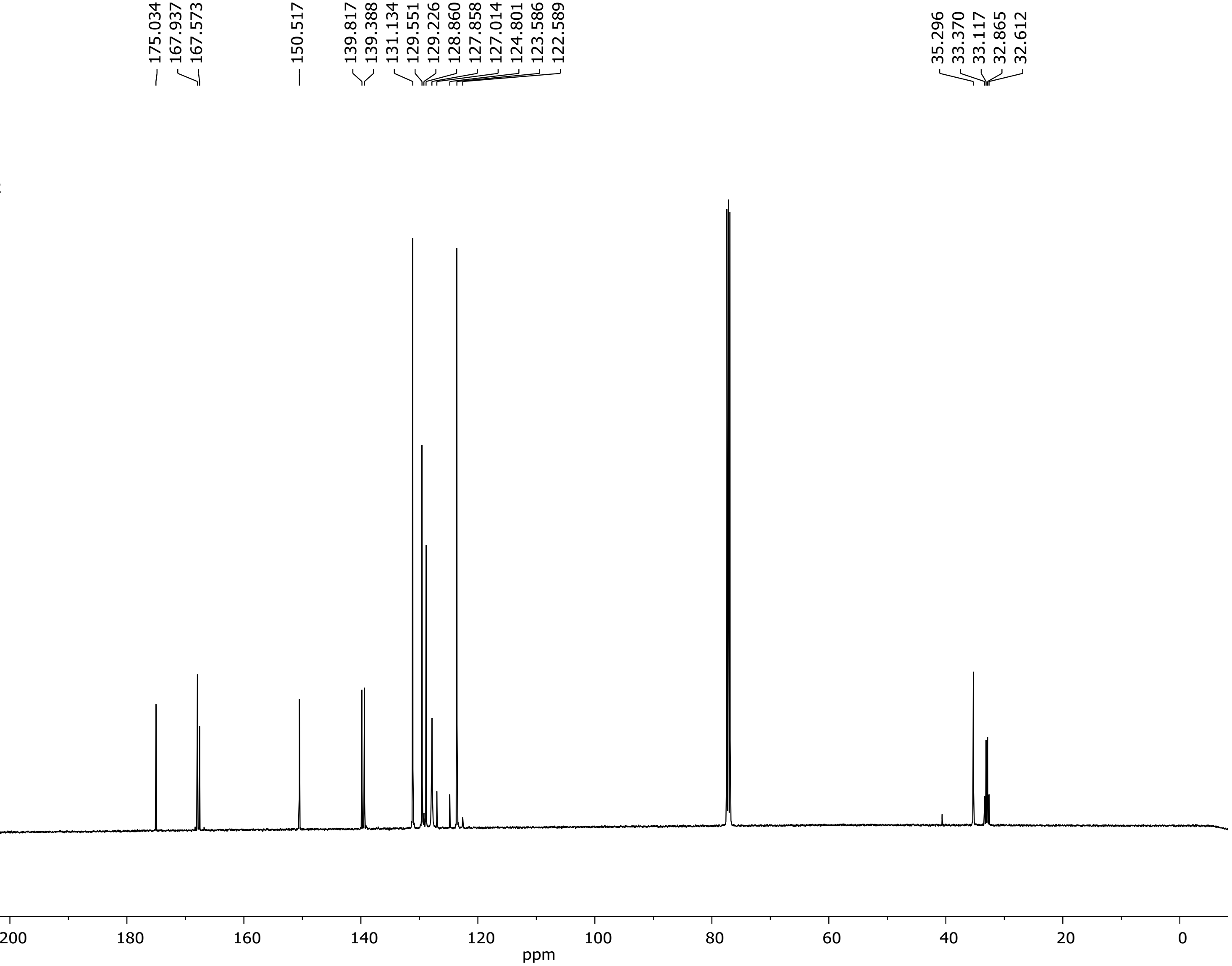

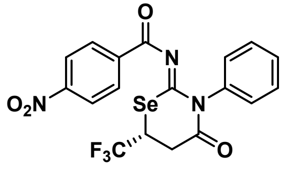

60

^{77}Se{^{1}H}, CDCl$_3$, 95 MHz

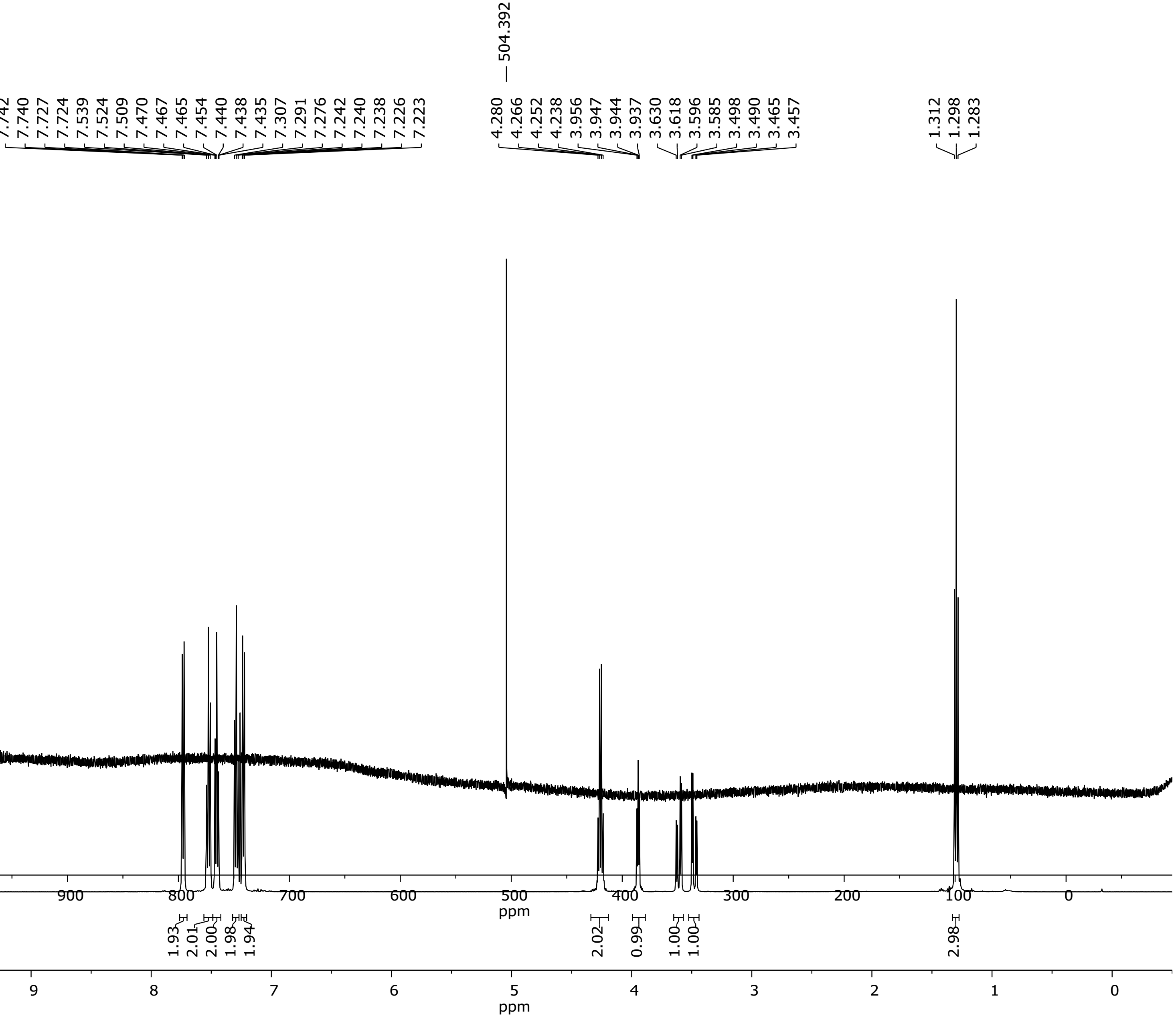

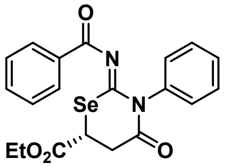

61

^{1}H, CDCl$_3$, 500 MHz

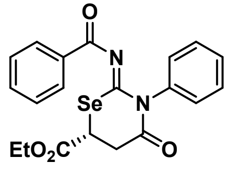

61

$^{13}C\{^{1}H\}$, $CDCl_3$, 126 MHz

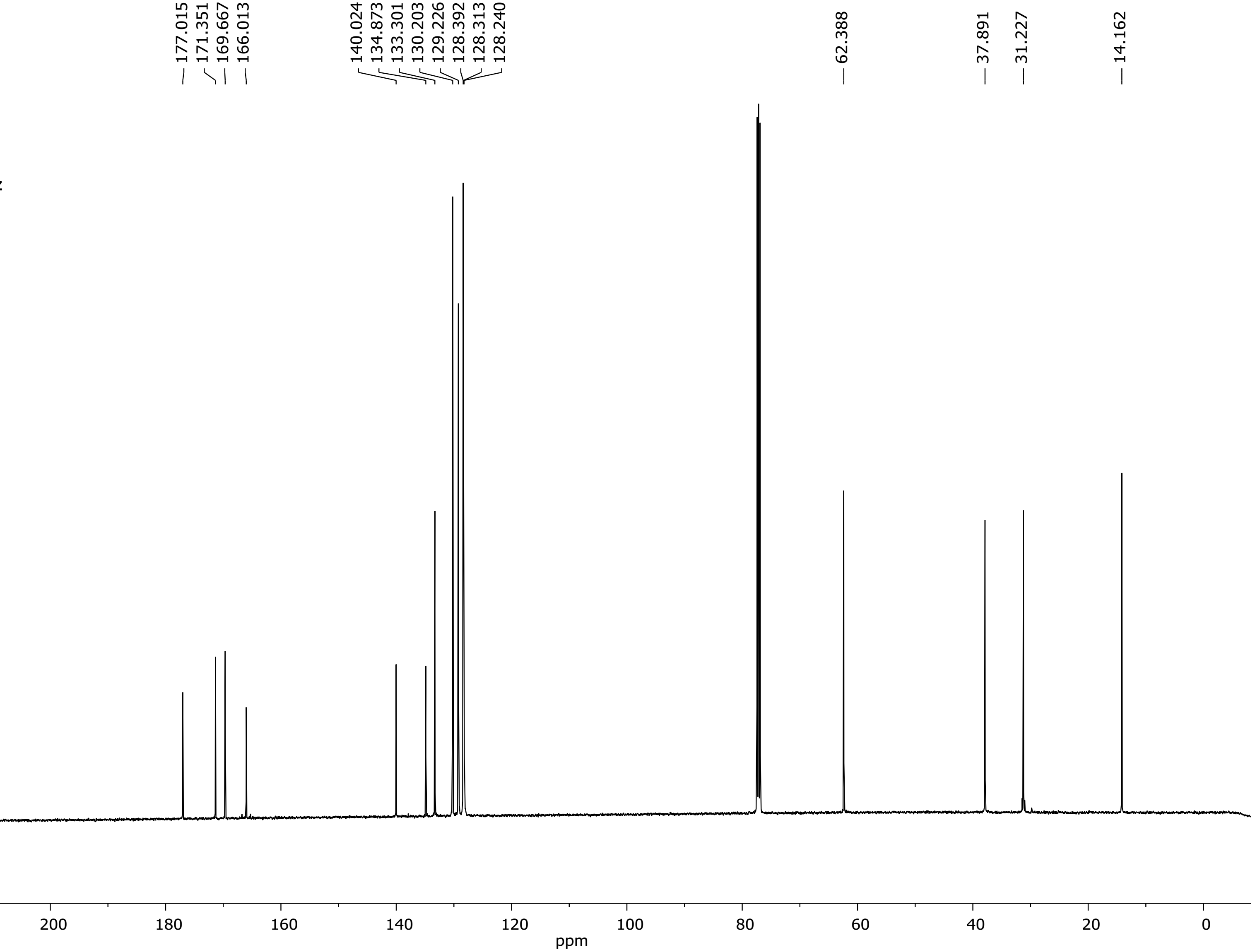

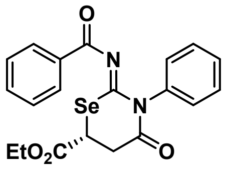

61

^{77}Se{^{1}H}, CDCl$_3$, 95 MHz

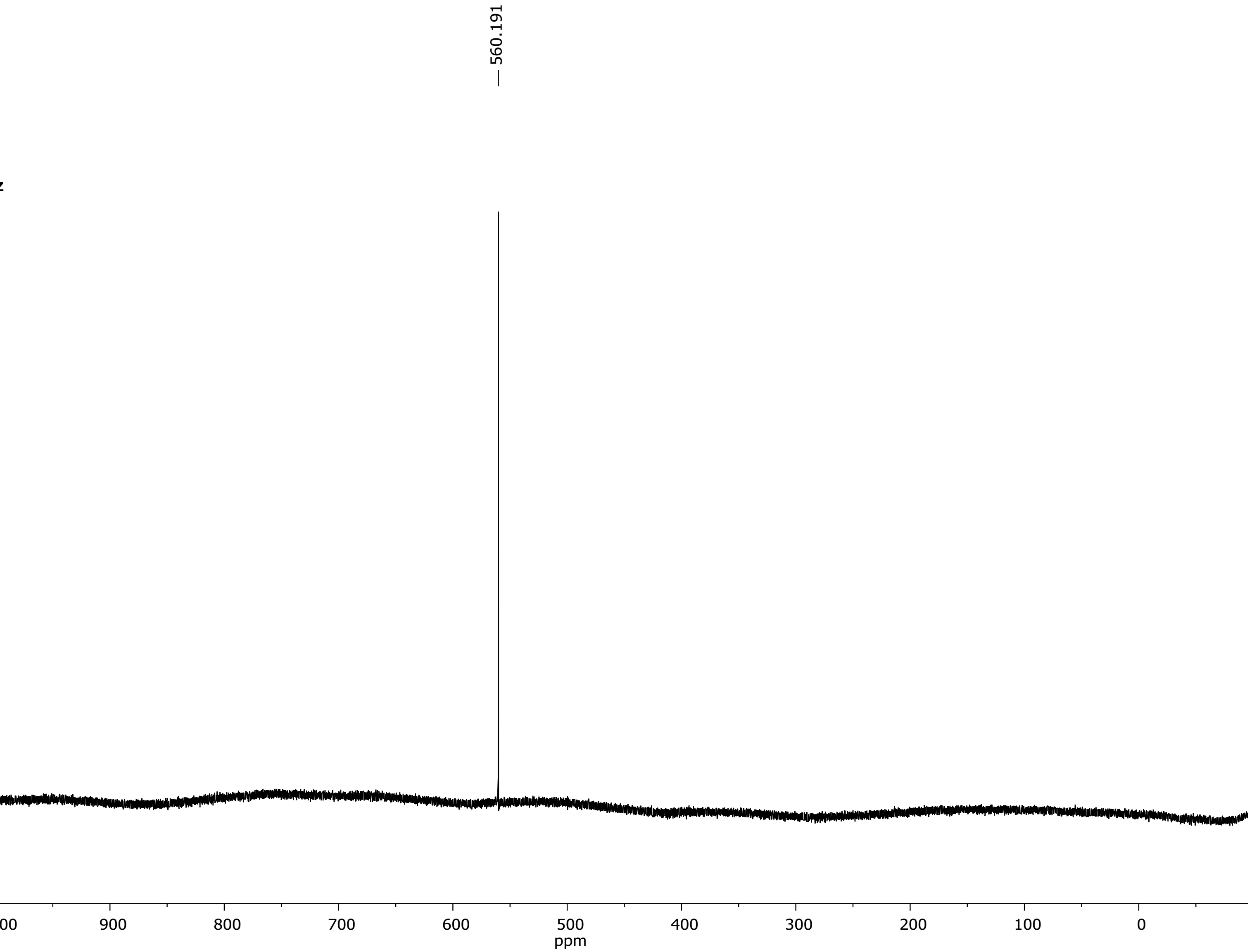

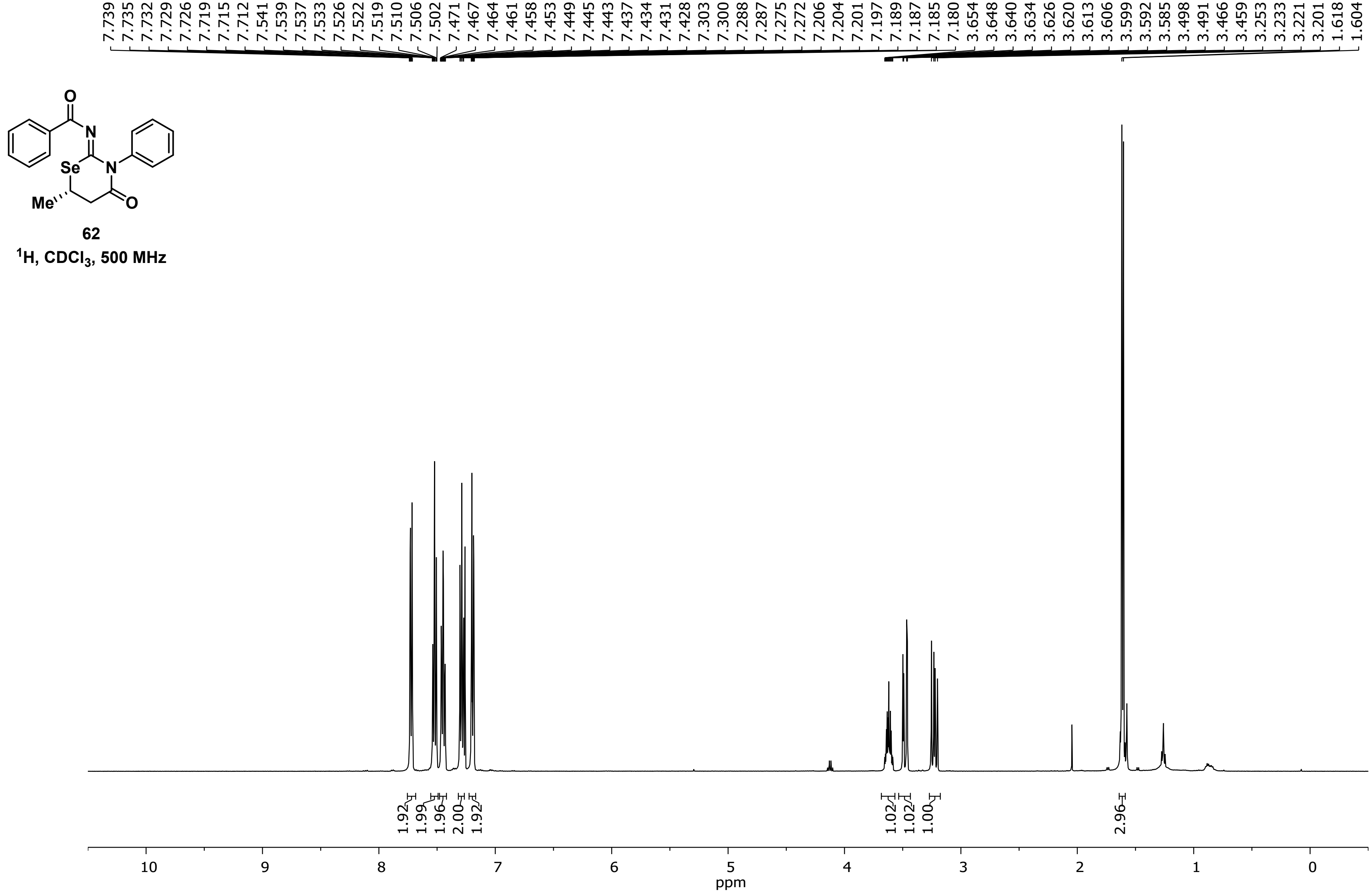

62

^{1}H, CDCl$_3$, 500 MHz

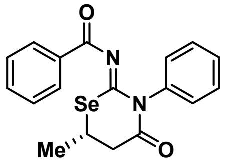

62

$^{13}C\{^1H\}$, $CDCl_3$, 126 MHz

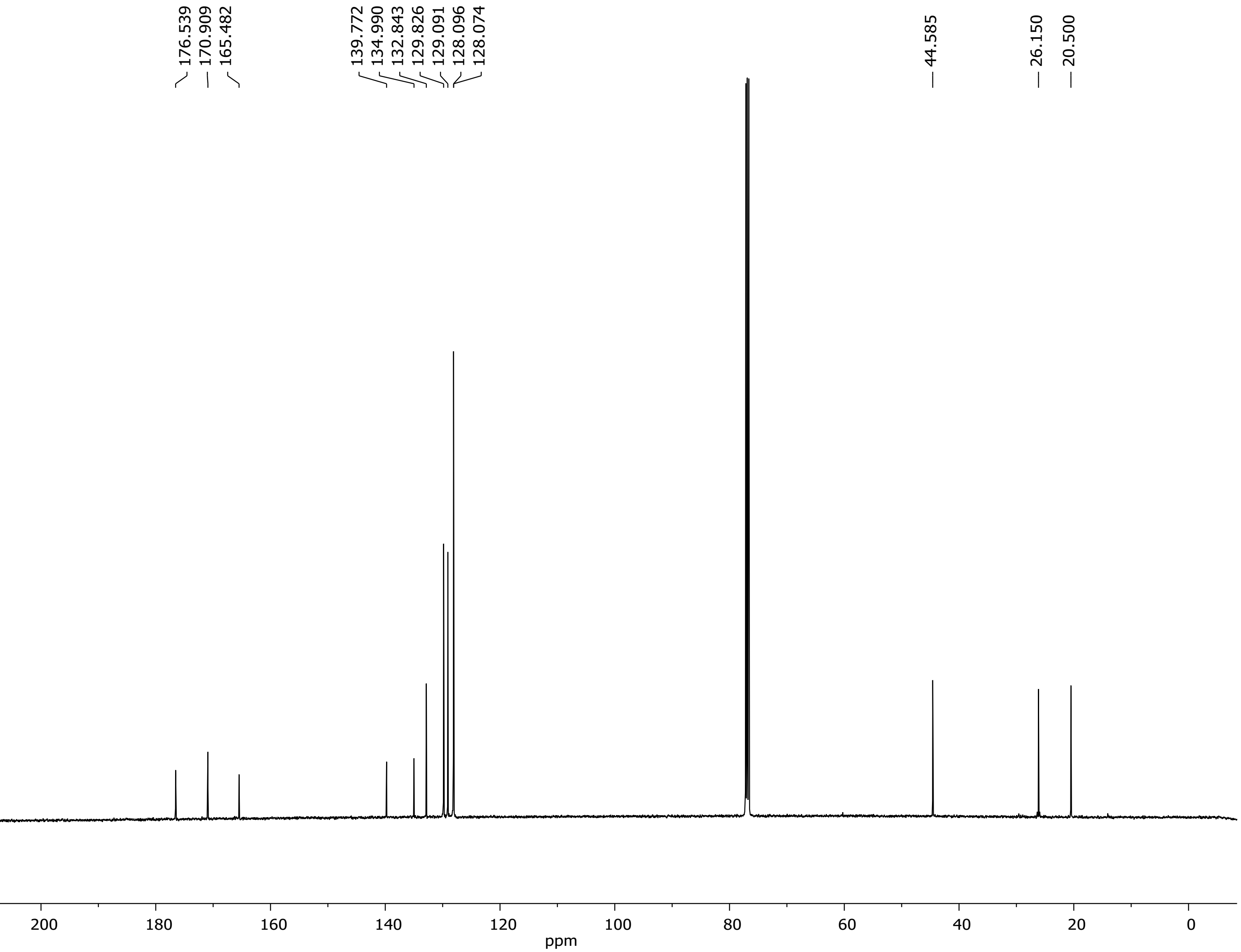

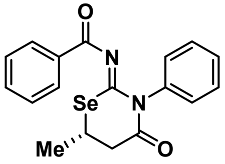

62

$^{77}Se\{^{1}H\}$, $CDCl_3$, 95 MHz

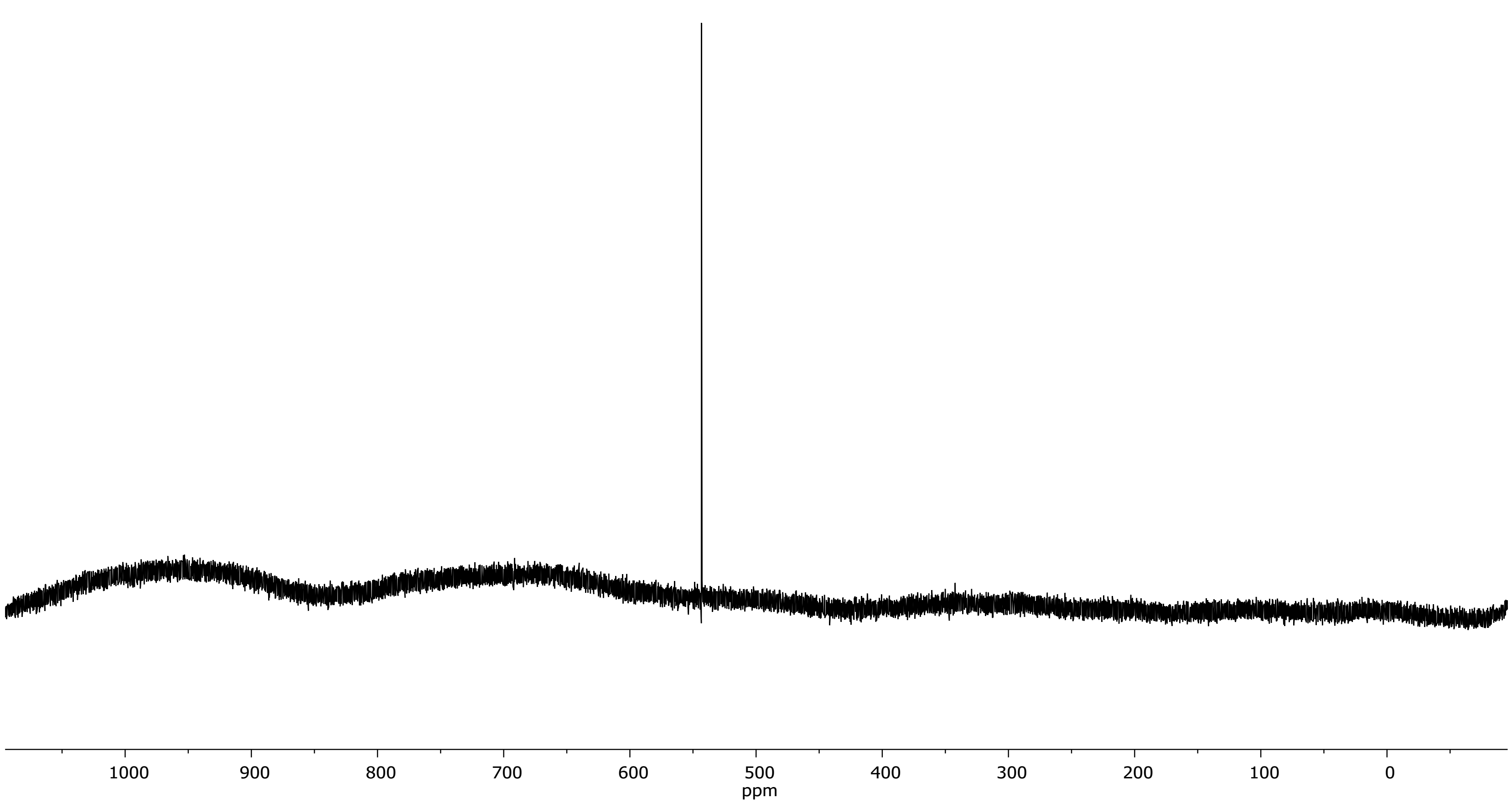

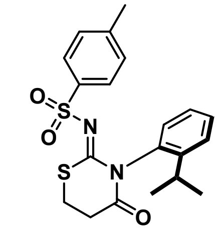

63
^{1}H, CDCl$_3$, 400 MHz

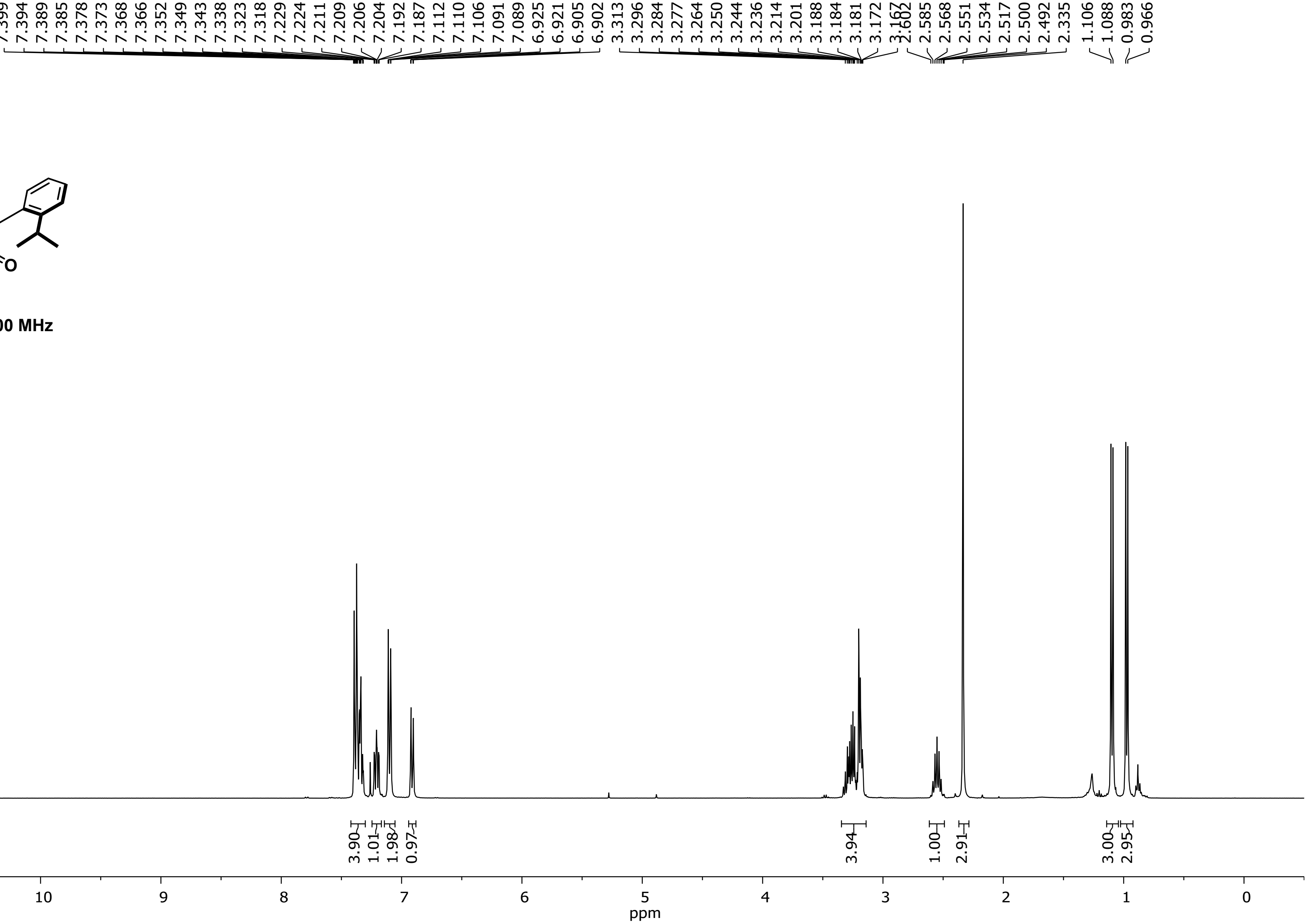

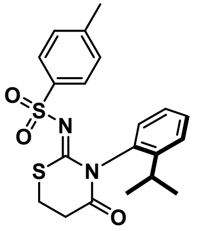

63

$^{13}C\{^{1}H\}$, $CDCl_3$, 126 MHz

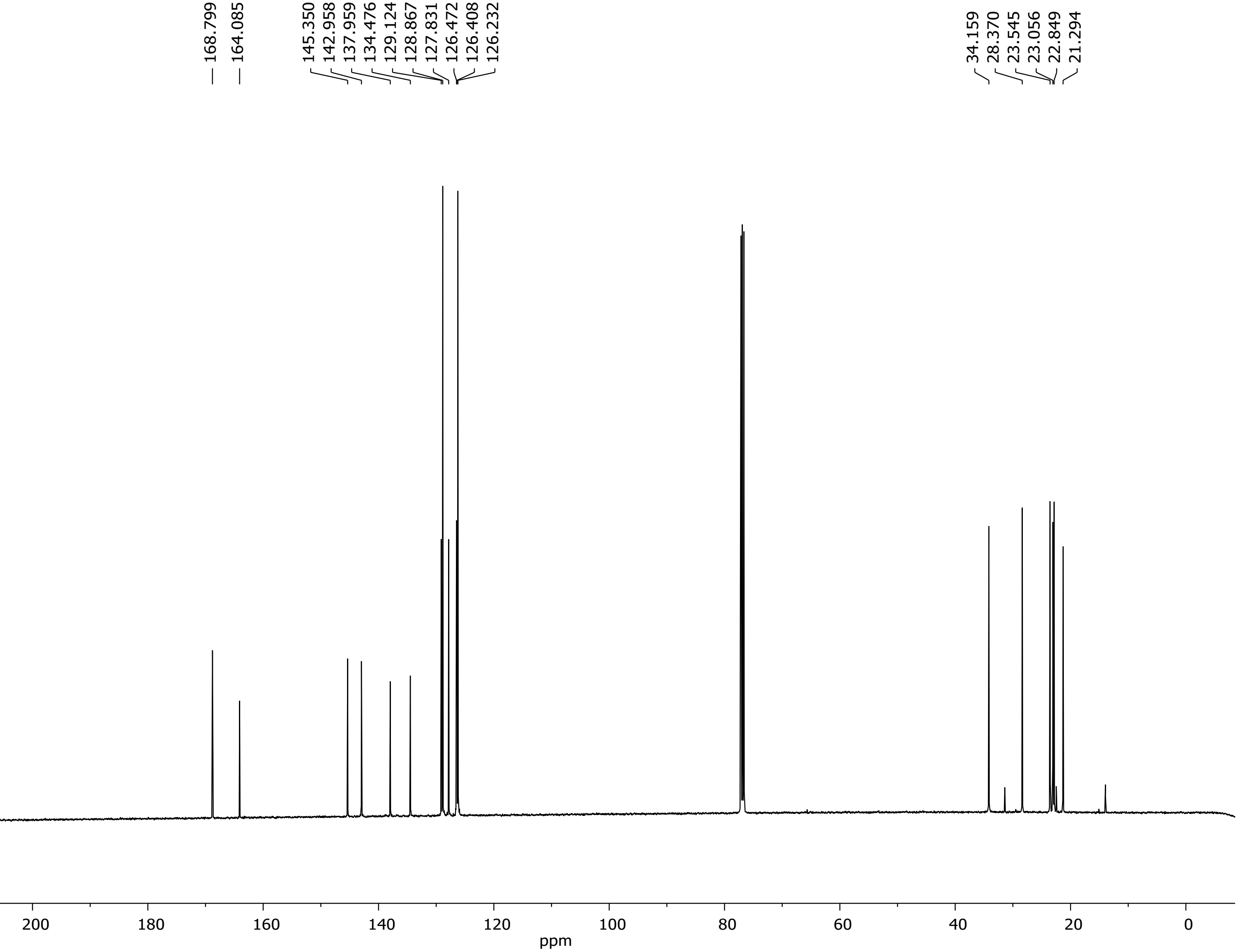

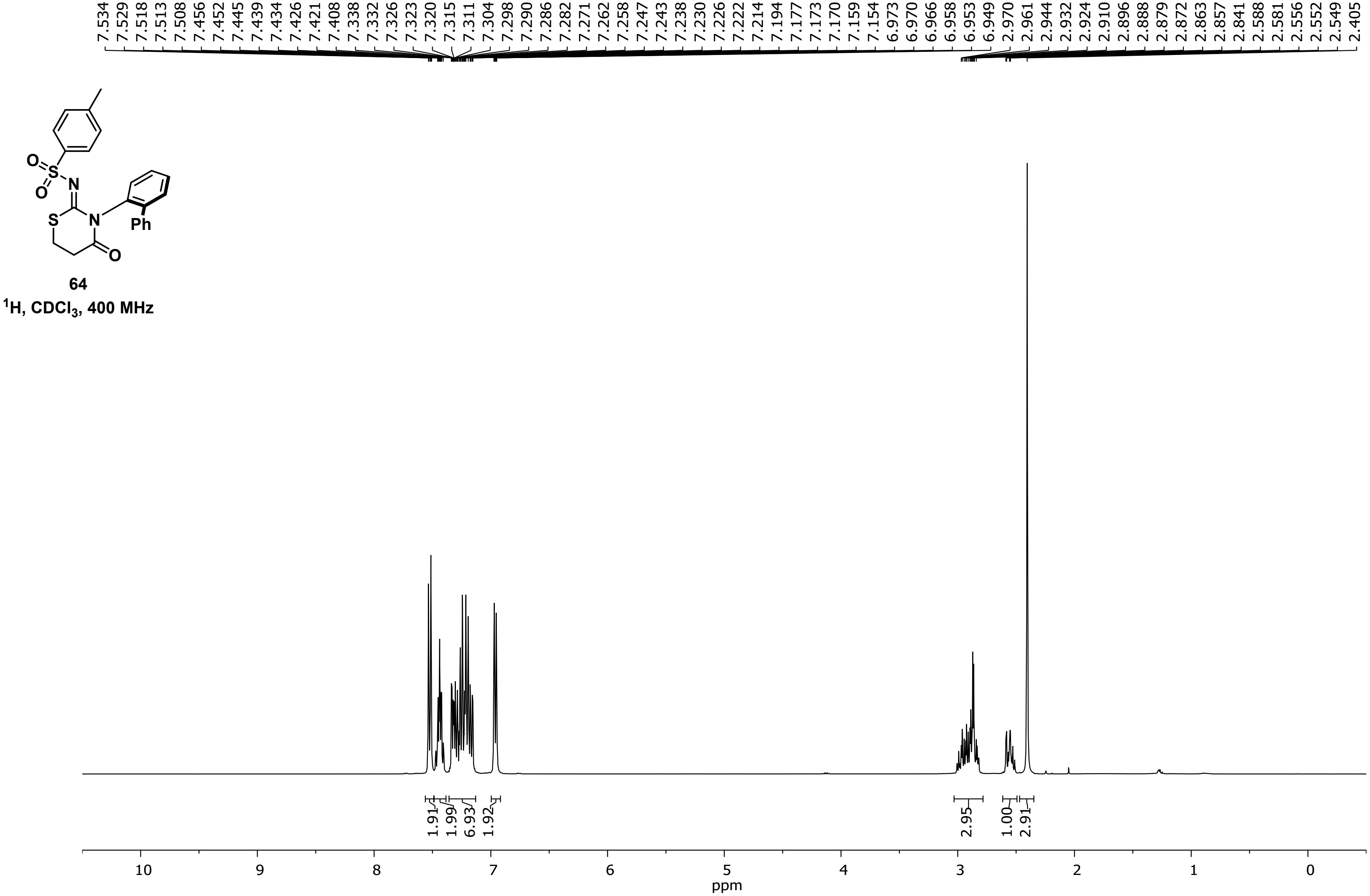

64

^{1}H, CDCl$_3$, 400 MHz

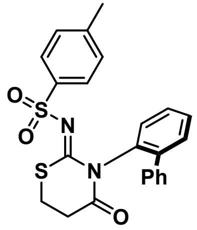

64

$^{13}C\{^{1}H\}$, $CDCl_3$, 126 MHz

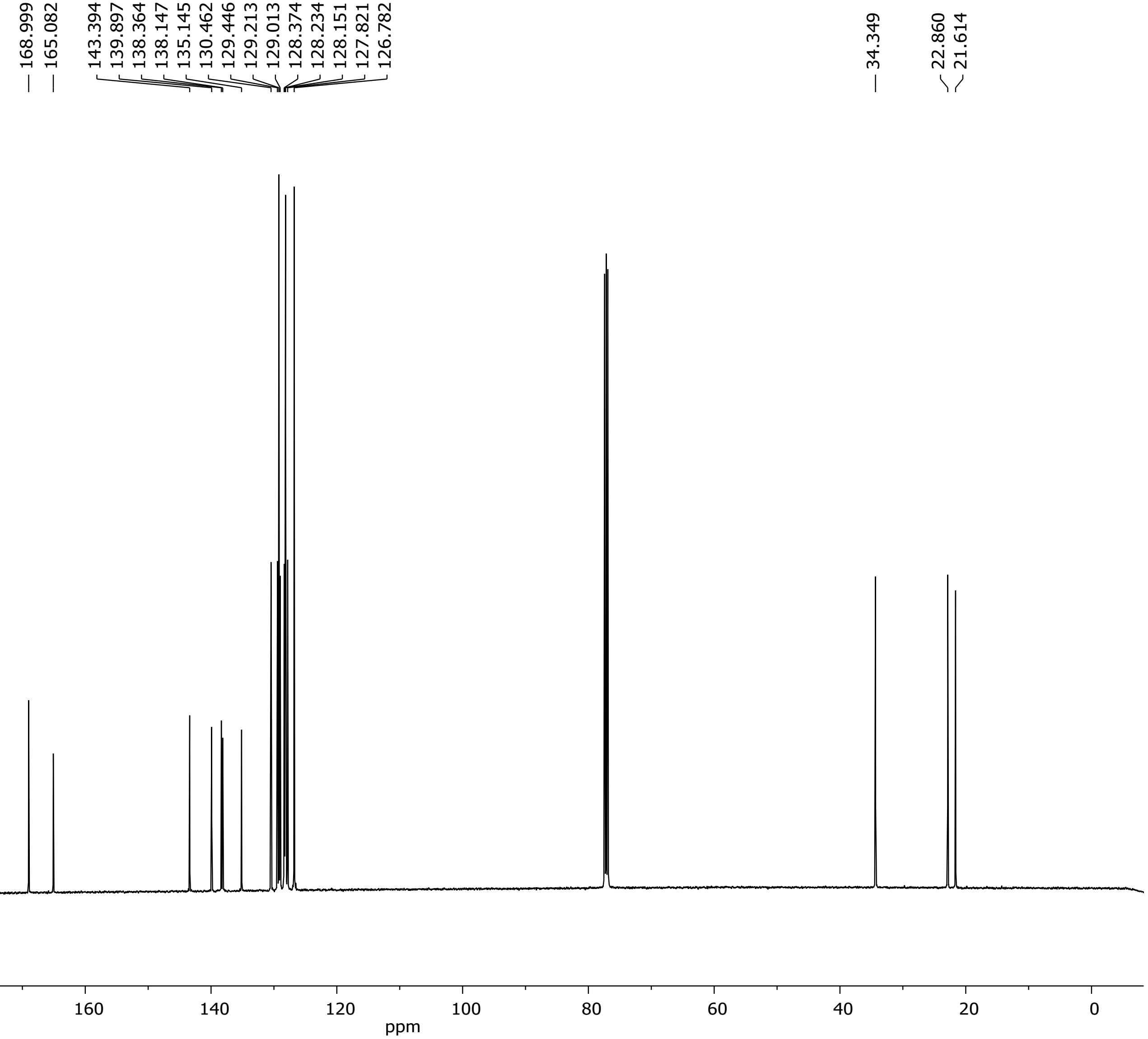

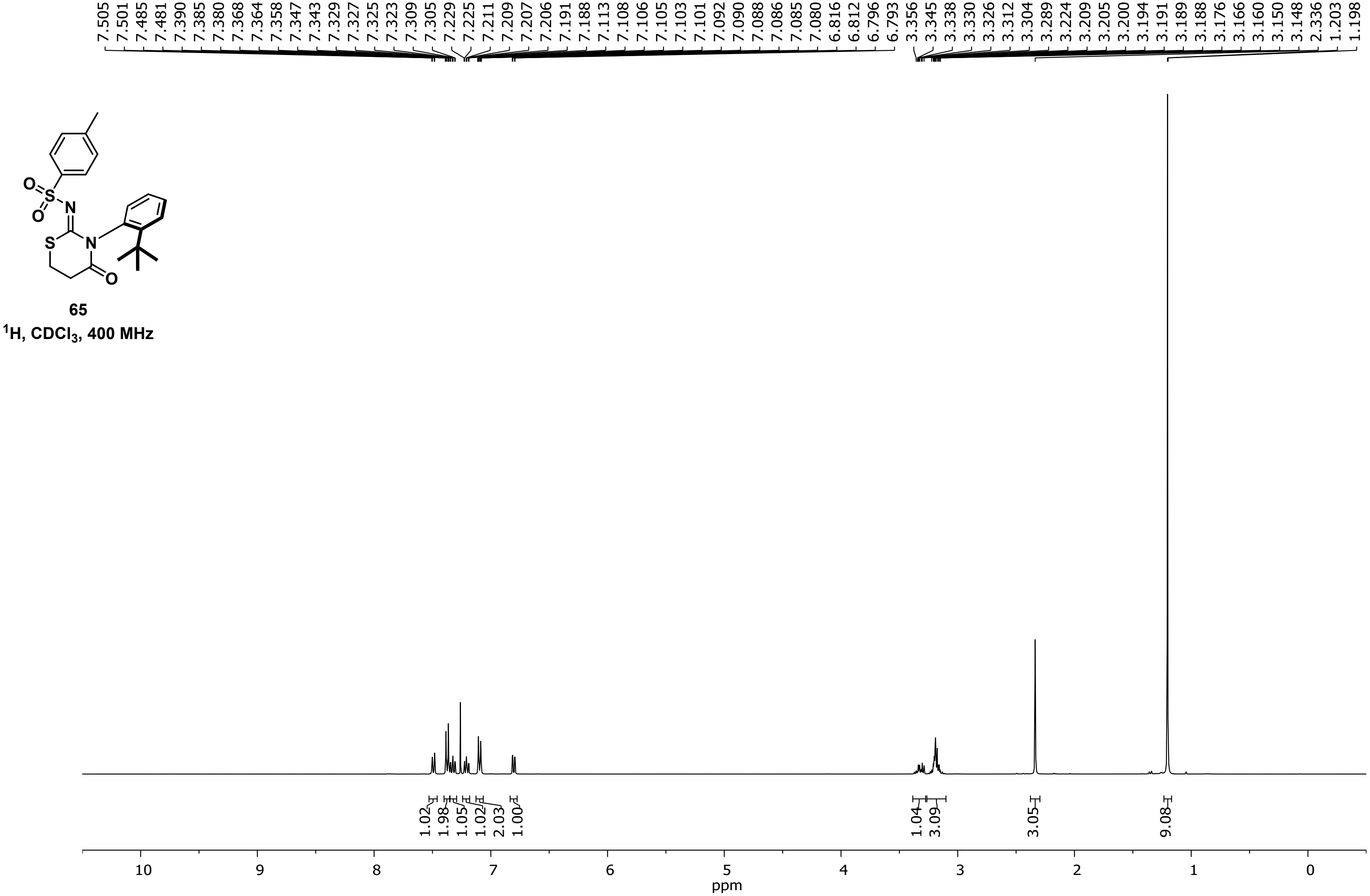

65

^{1}H, CDCl$_3$, 400 MHz

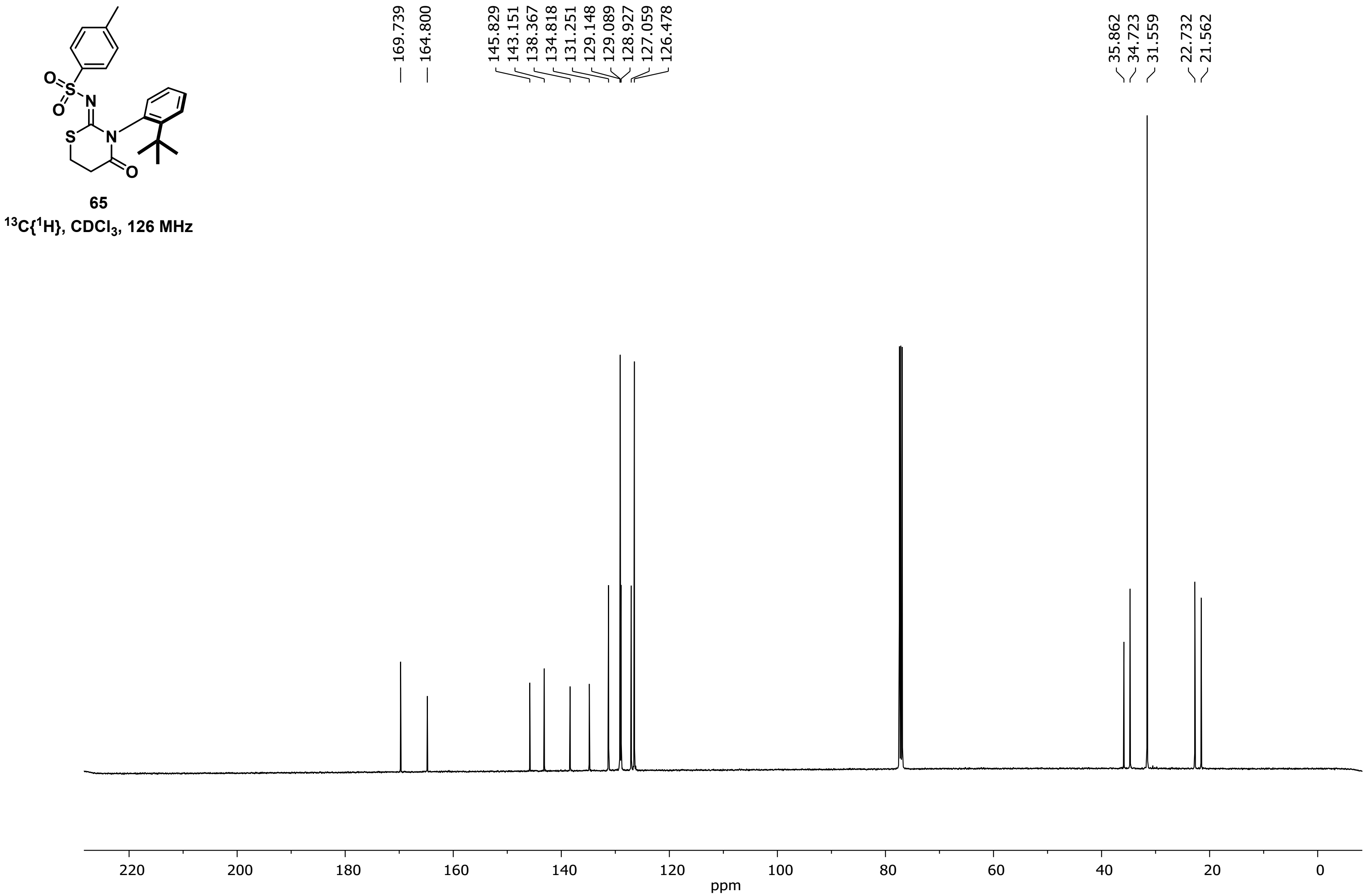
65
$^{13}C\{^1H\}$, $CDCl_3$, 126 MHz
169.739
164.800
145.829
143.151
138.367
134.818
131.251
129.148
129.089
128.927
127.059
126.478
35.862
34.723
31.559
22.732
21.562
220
200
180
160
140
120
100
80
60
40
20
0
ppm

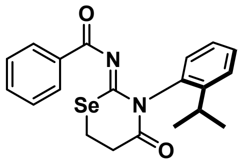

66

^{1}H, CDCl$_3$, 500 MHz

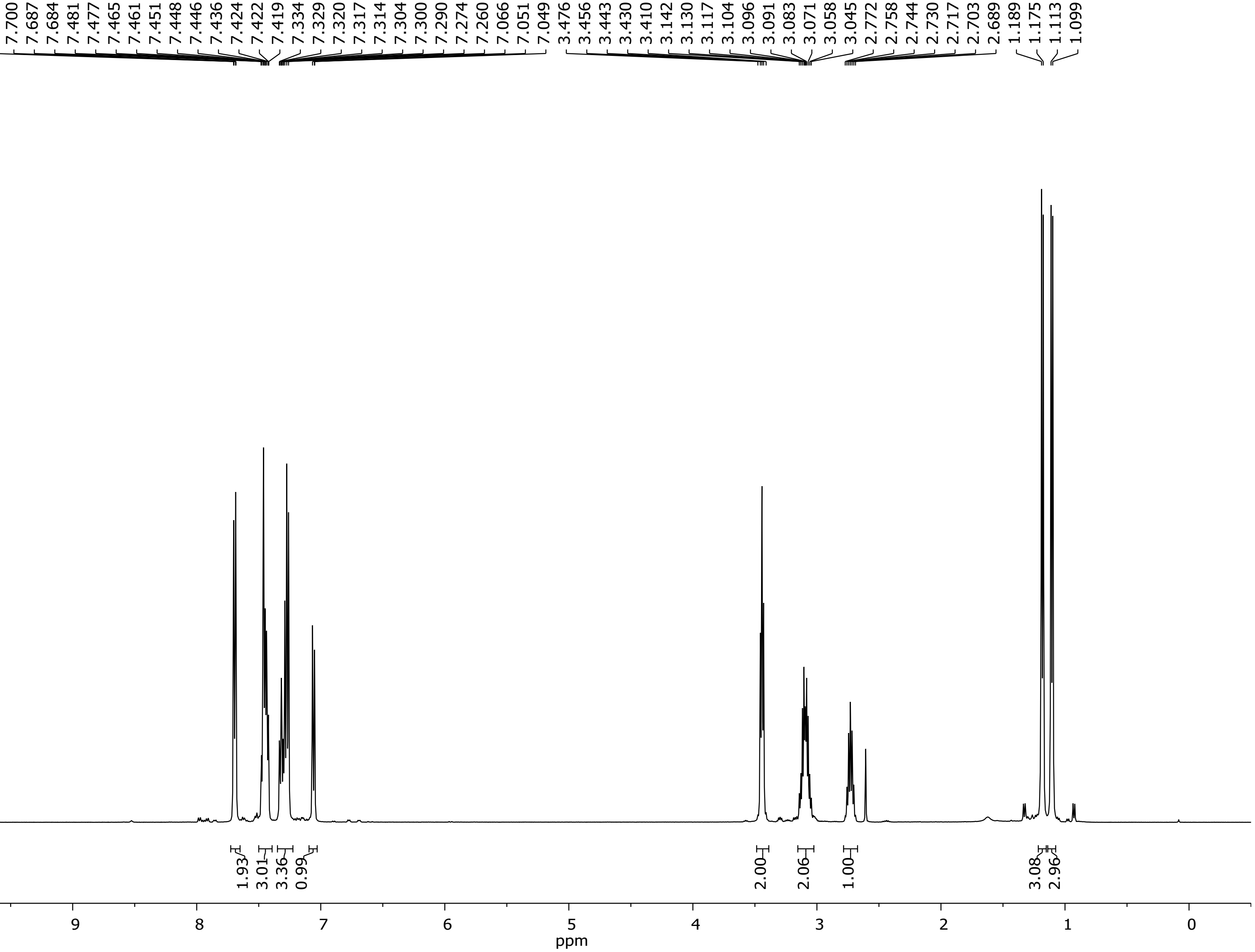

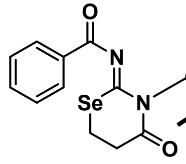

66

$^{13}C\{^1H\}$, $CDCl_3$, 126 MHz

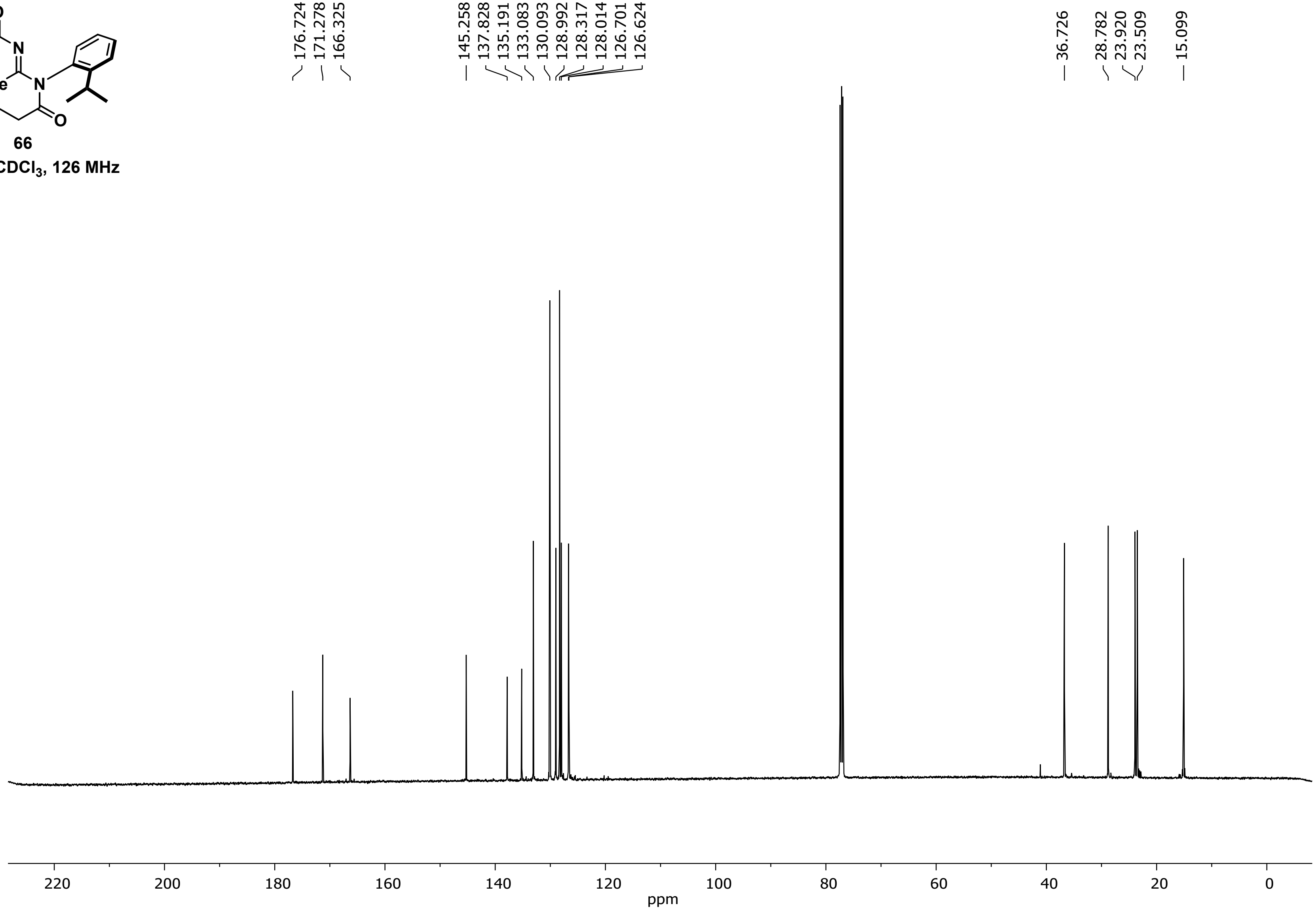

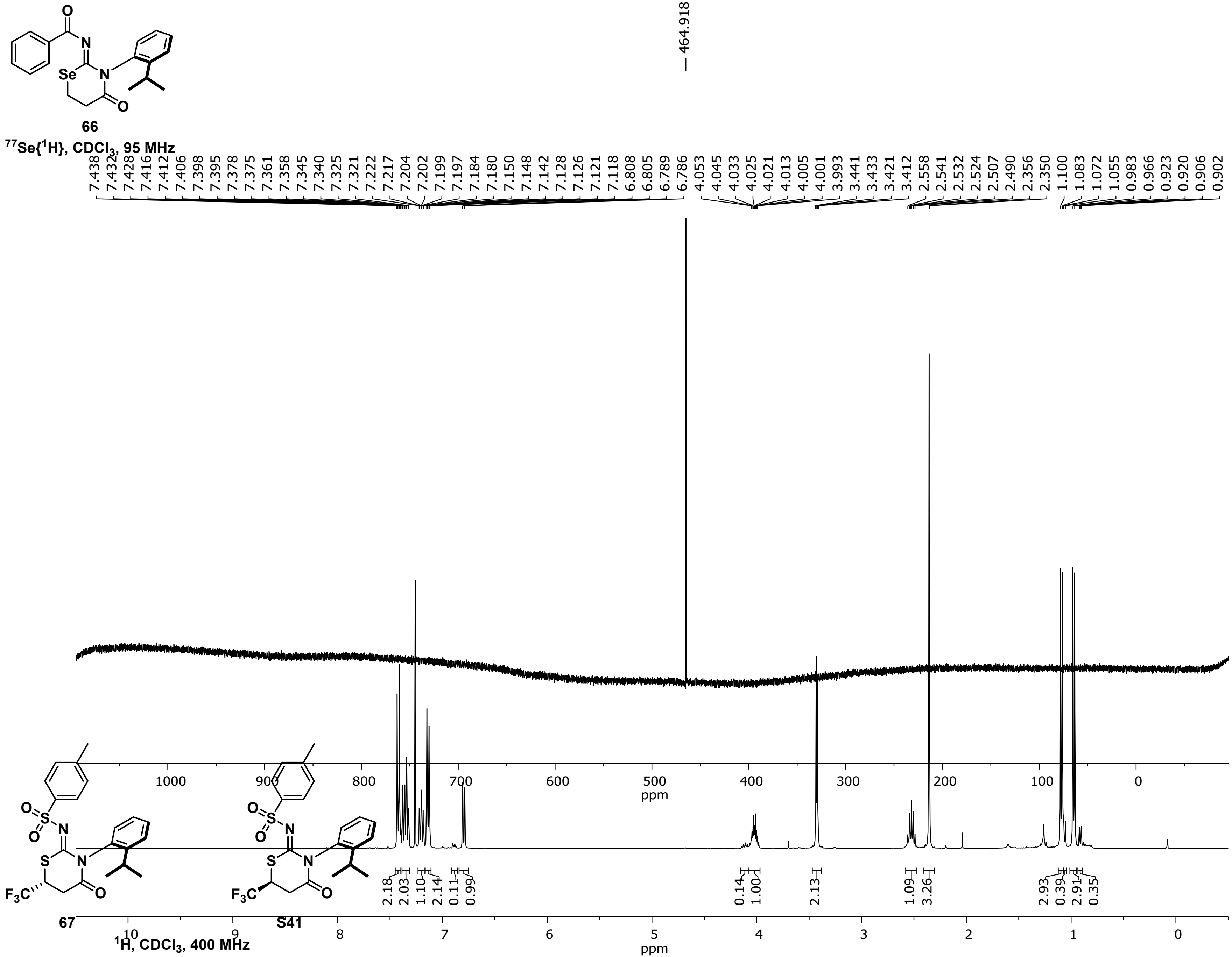

66

$^{77}Se\{^{1}H\}$, $CDCl_3$, 95 MHz

67

S41

^{1}H, $CDCl_3$, 400 MHz

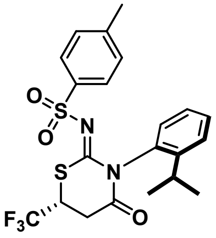

67

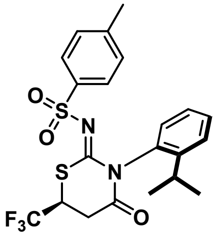

S41

$^{19}F\{^{1}H\}$, $CDCl_3$, 377 MHz

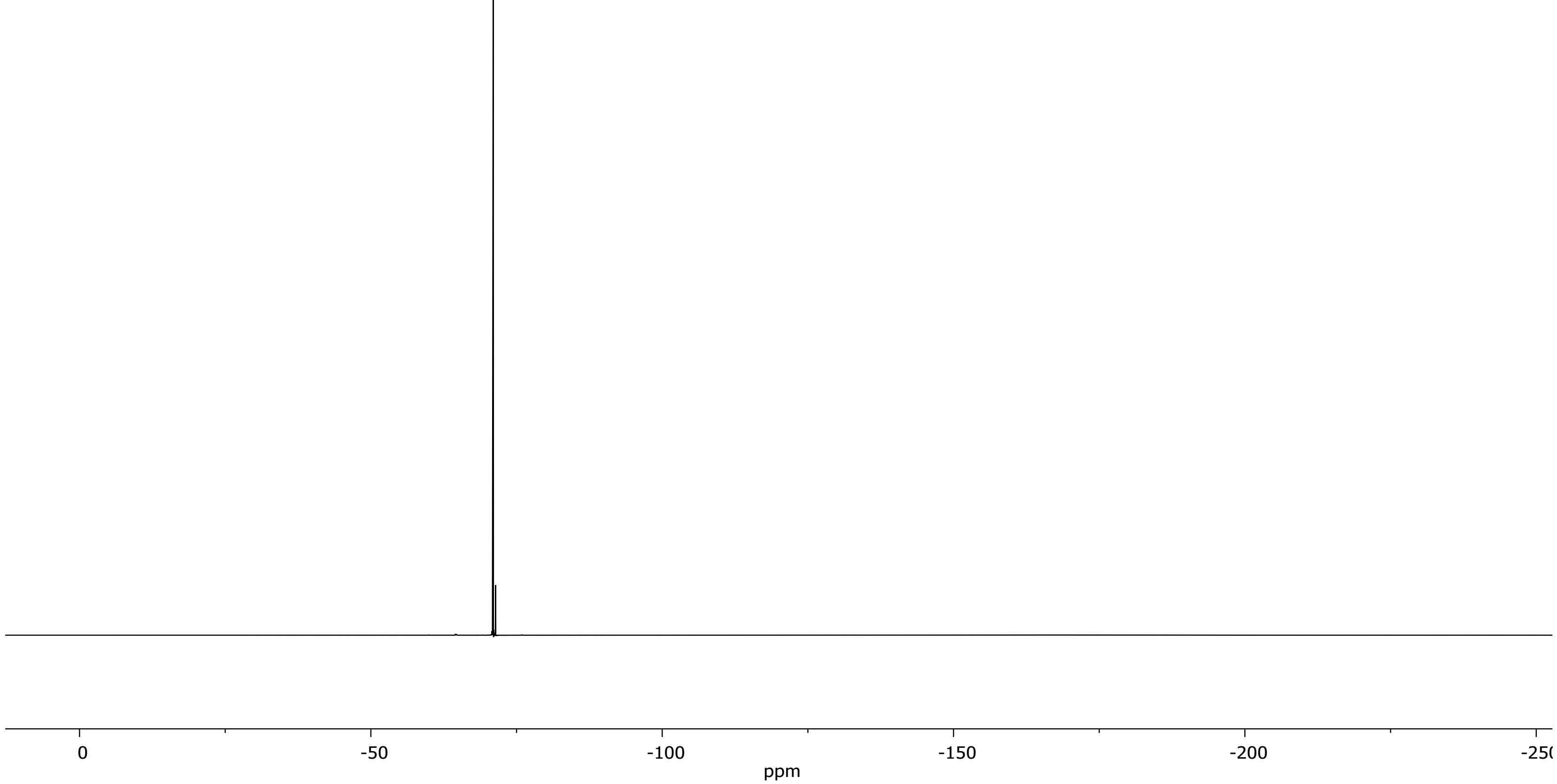

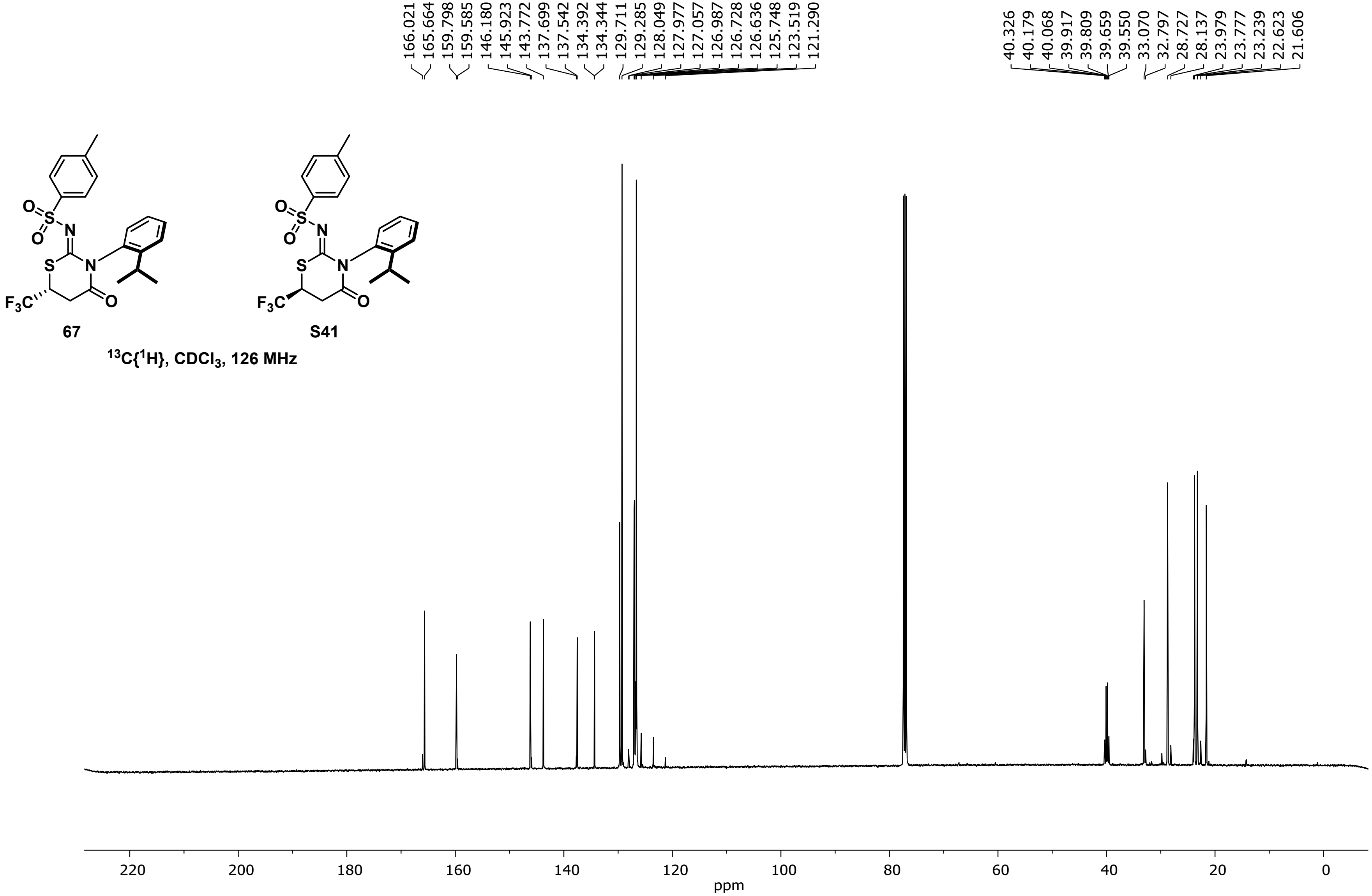
67
S41
13C{1H}, CDCl3, 126 MHz
166.021
165.664
159.798
159.585
146.180
145.923
143.772
137.699
137.542
134.392
134.344
129.711
129.285
128.049
127.977
127.057
126.987
126.728
126.636
125.748
123.519
121.290
40.326
40.179
40.068
39.917
39.809
39.659
39.550
33.070
32.797
28.727
28.137
23.979
23.777
23.239
22.623
21.606
220
200
180
160
140
120
100
80
60
40
20
0
ppm

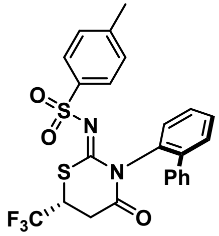

68

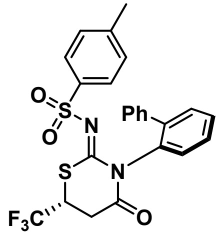

S42

^{1}H, CDCl$_3$, 400 MHz

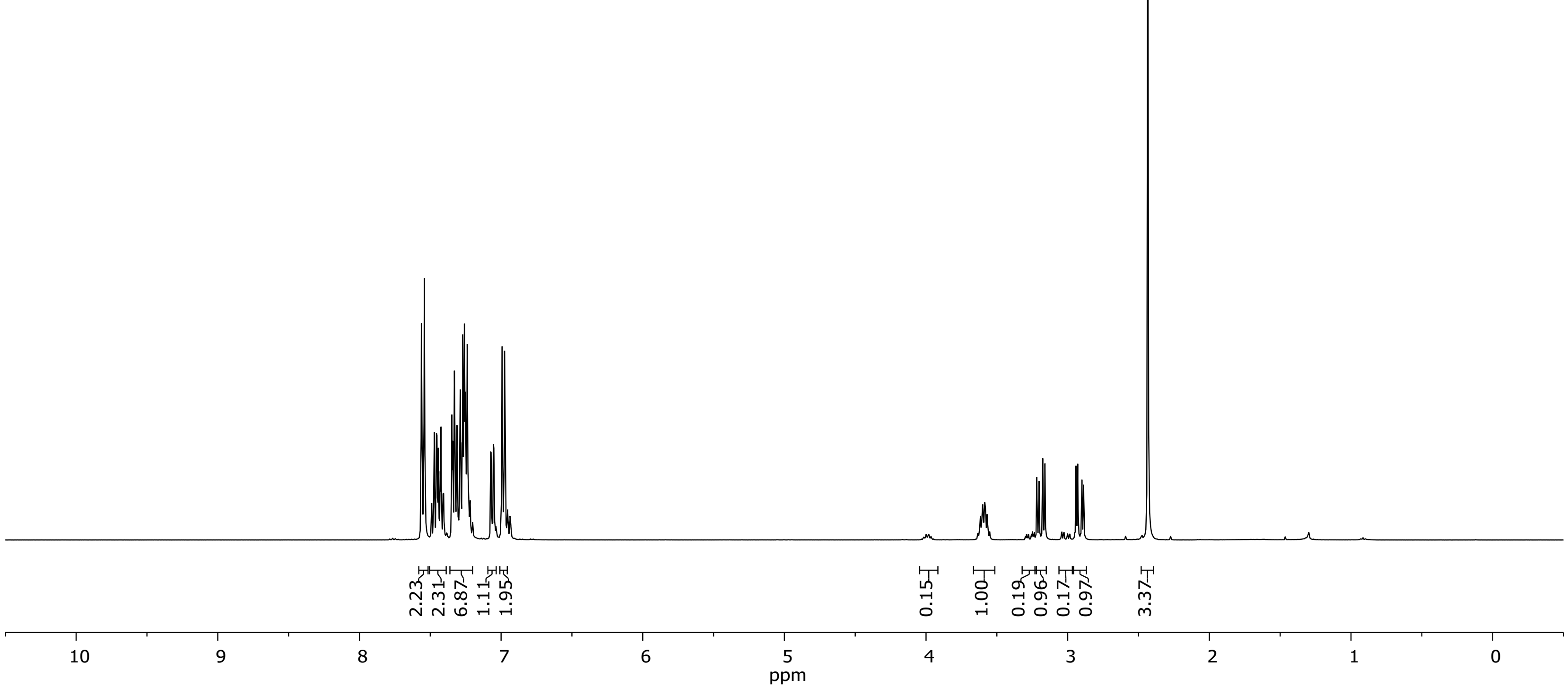

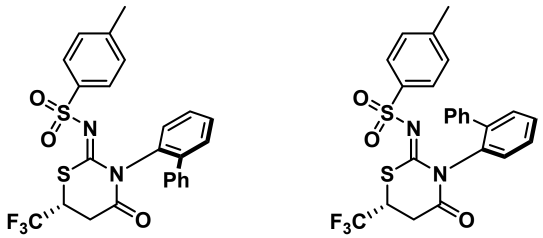

68 S42

$^{19}F\{^{1}H\}$, $CDCl_3$, 377 MHz

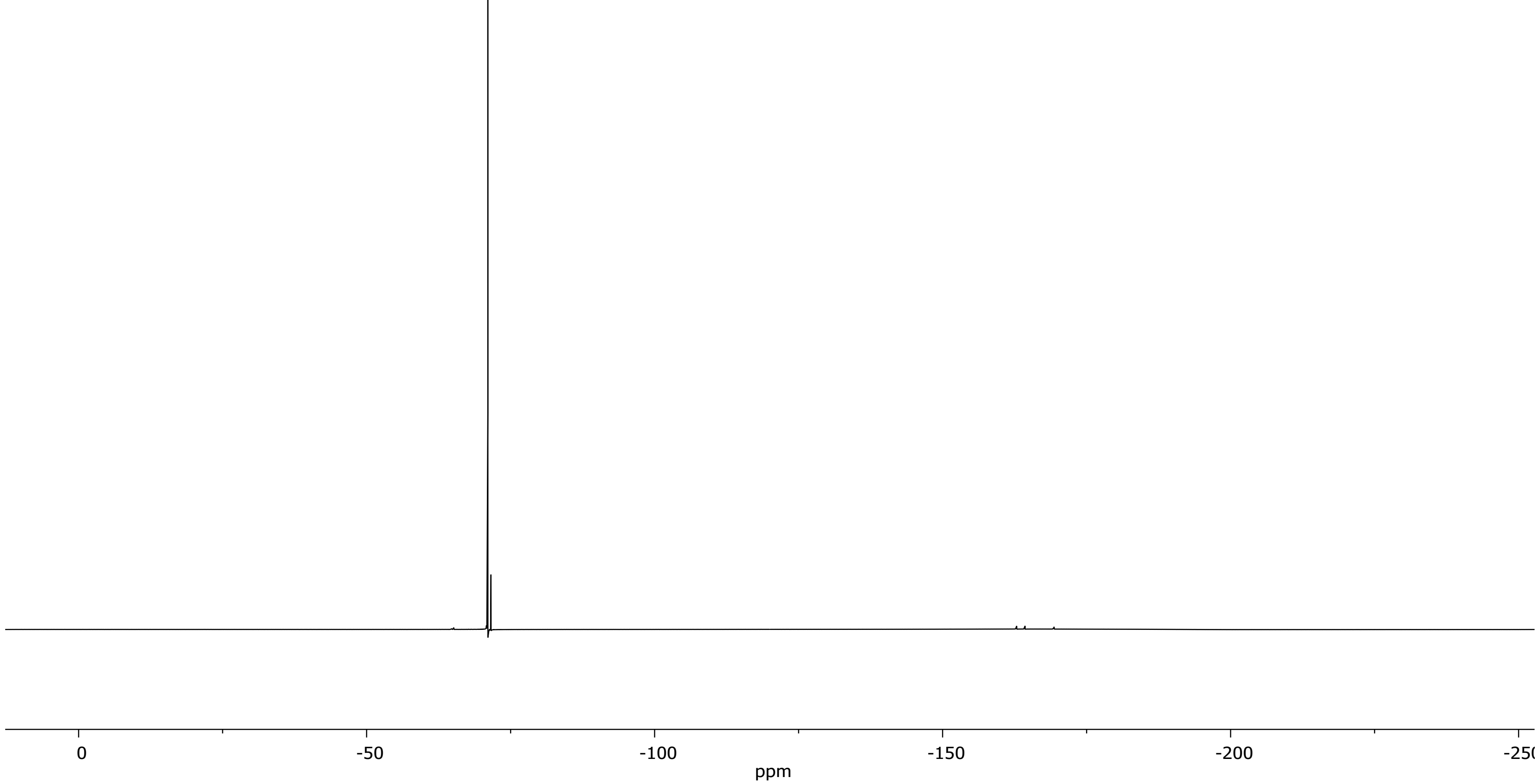

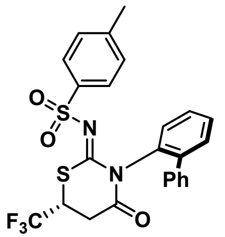

68

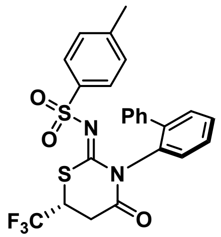

S42

$^{13}C\{^1H\}$, $CDCl_3$, 126 MHz

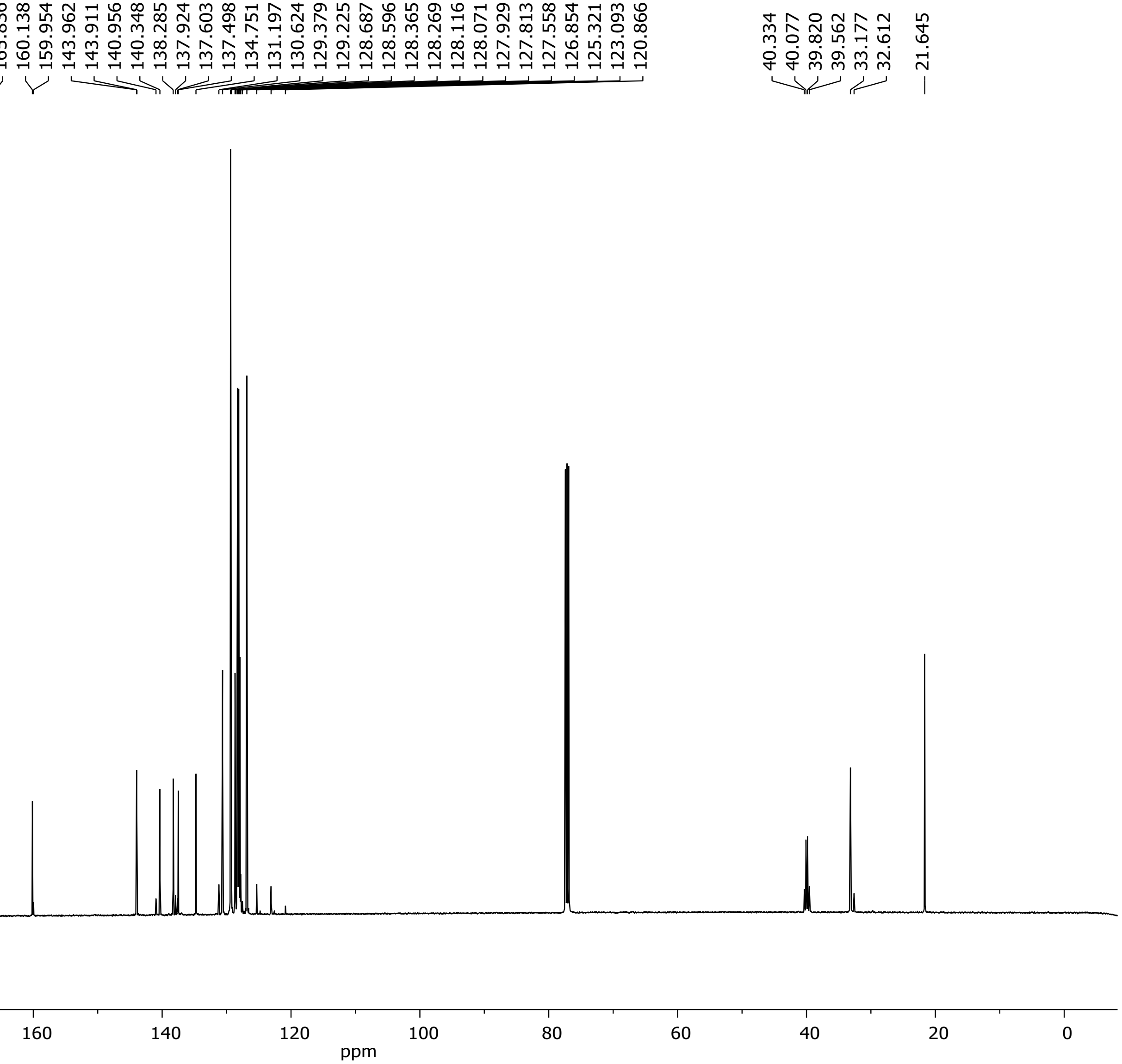

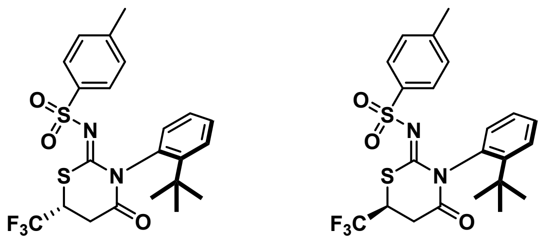

69 **S43**

^{1}H, CDCl$_3$, 500 MHz

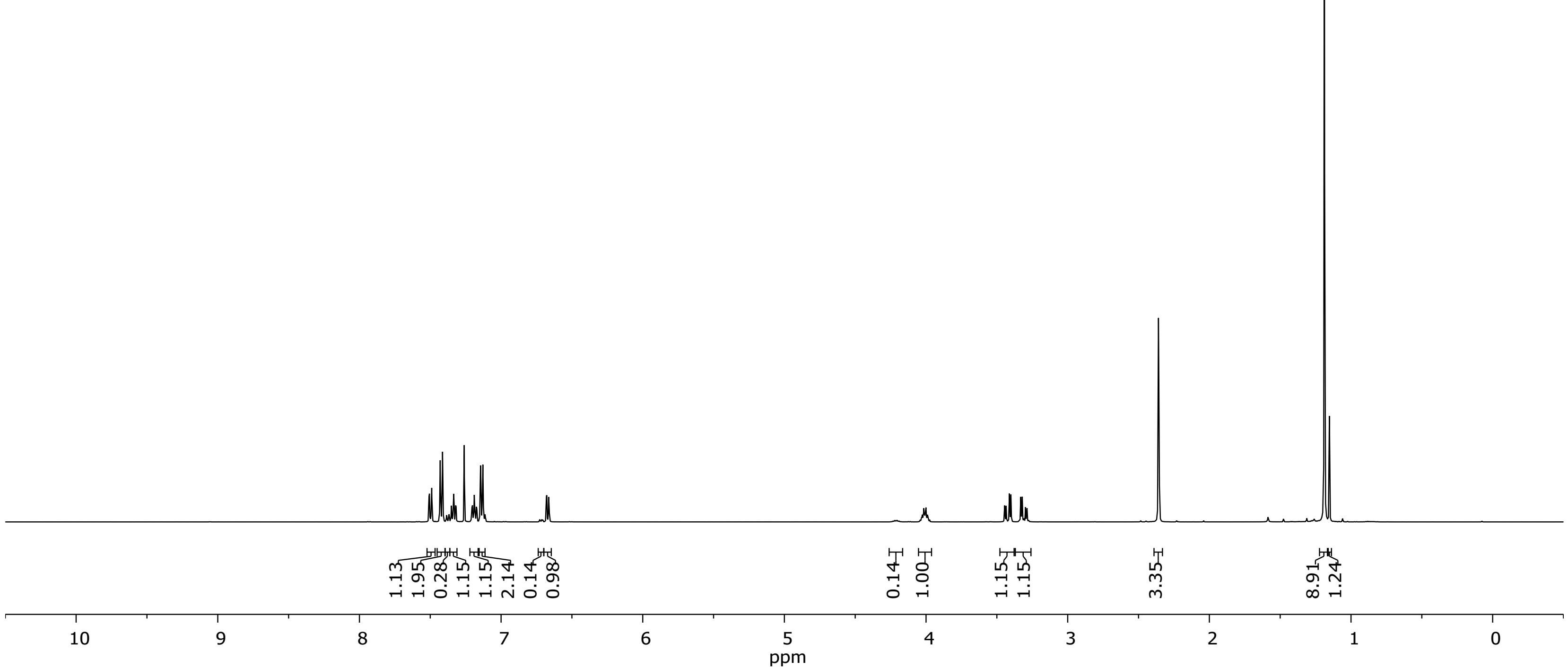

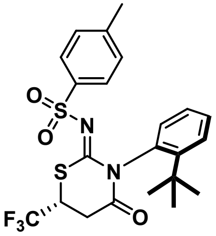

69

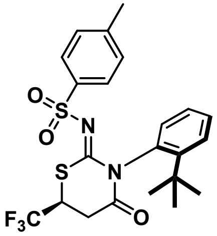

S43

$^{19}F\{^{1}H\}$, $CDCl_3$, 377 MHz

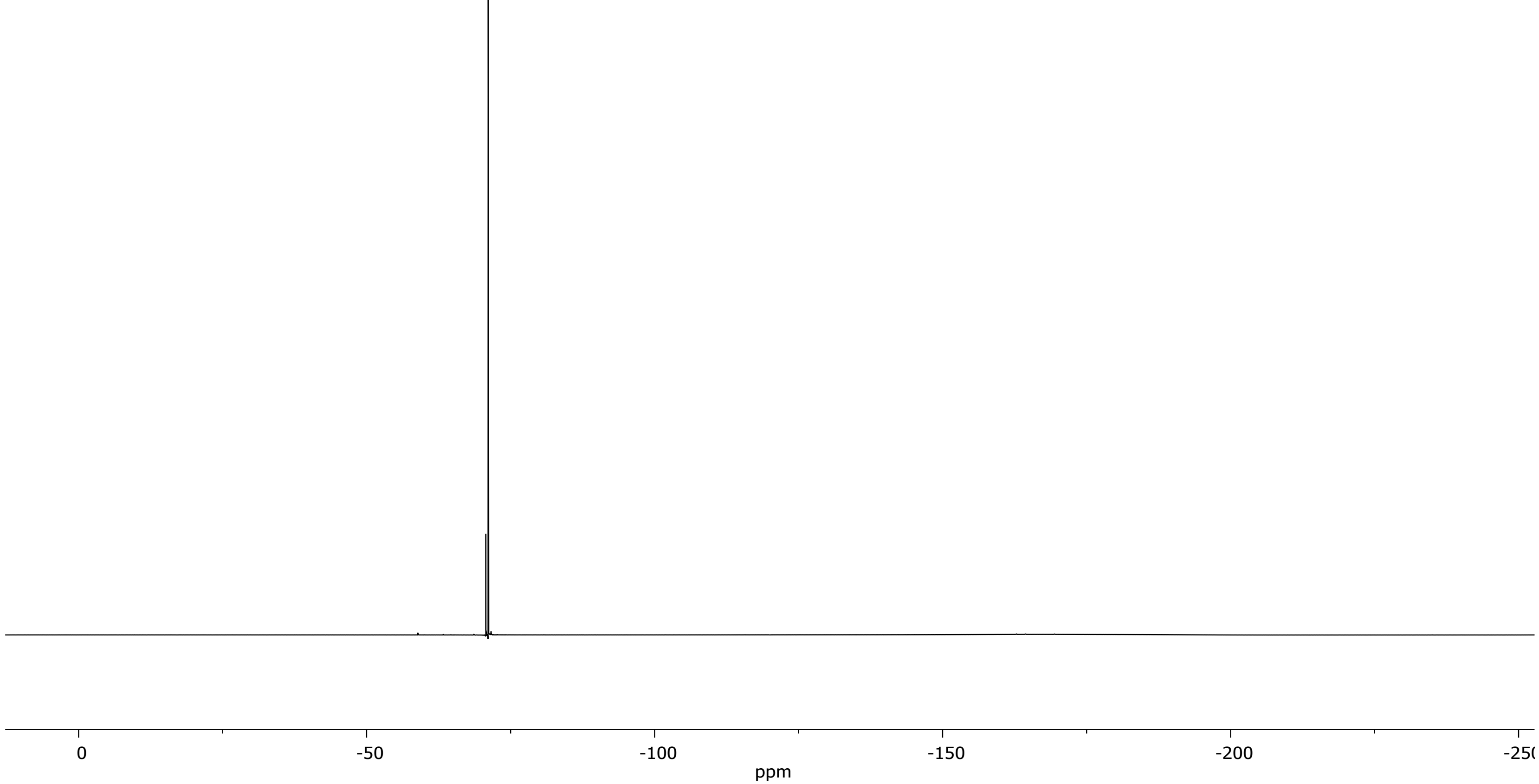

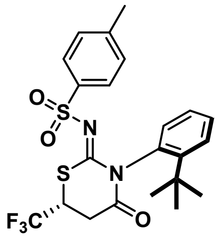

69

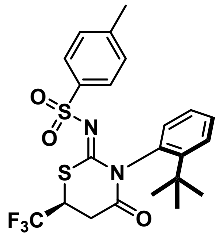

S43

$^{13}C\{^{1}H\}$, $CDCl_3$, 126 MHz

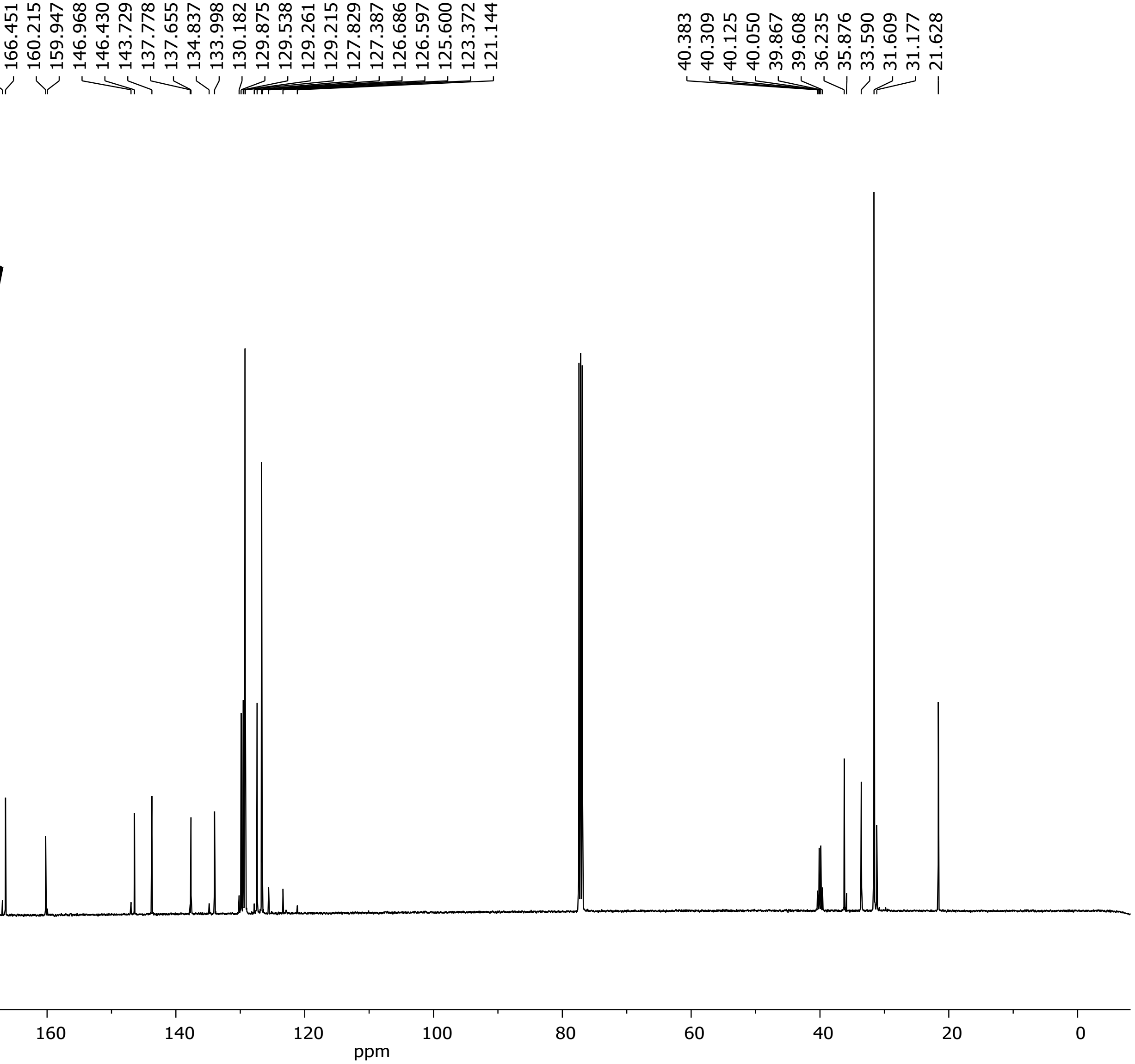

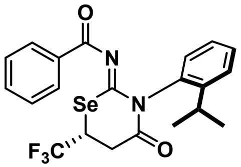

70

^1H, $CDCl_3$, 400 MHz

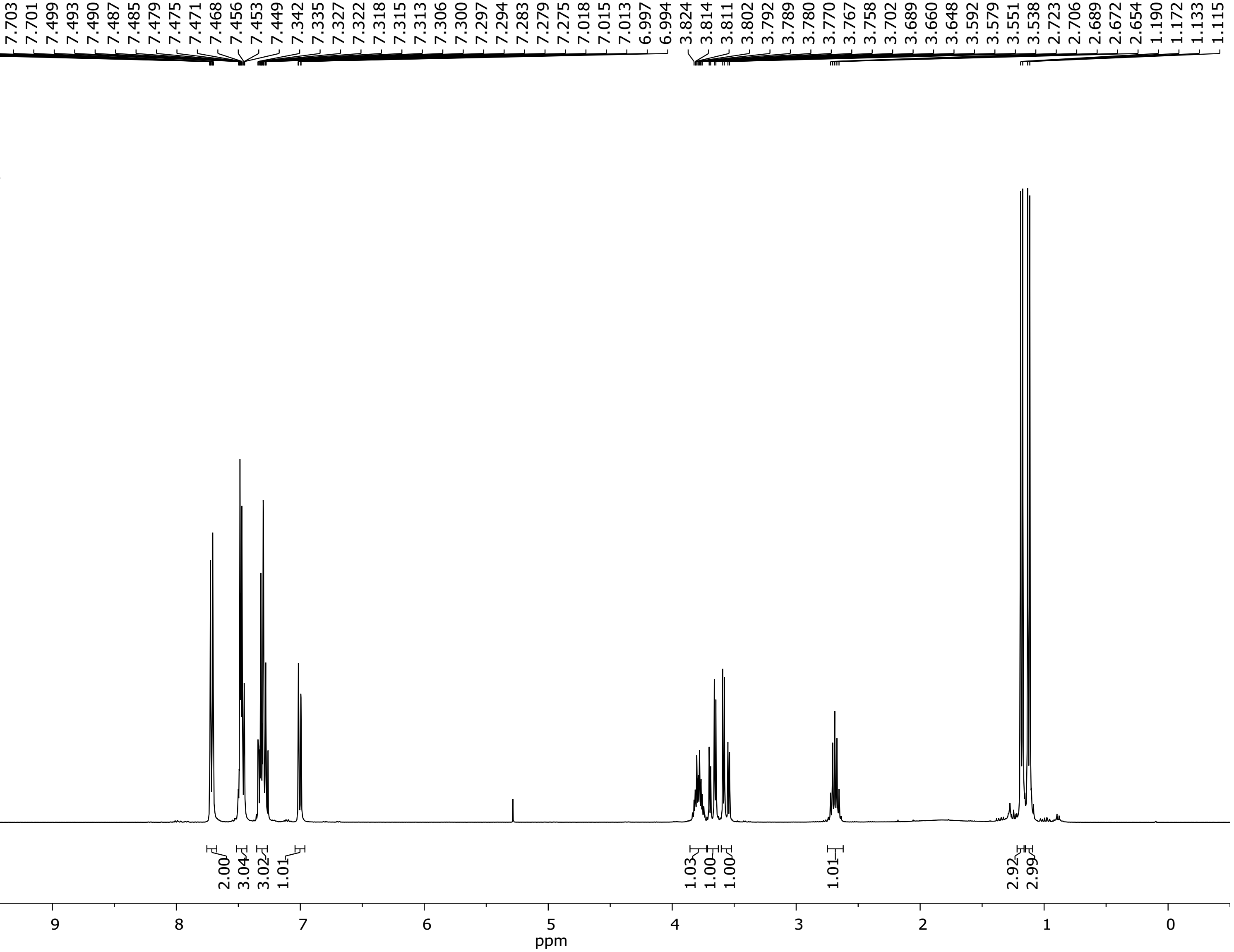

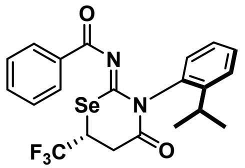

70

$^{19}F\{^1H\}$, $CDCl_3$, 377 MHz

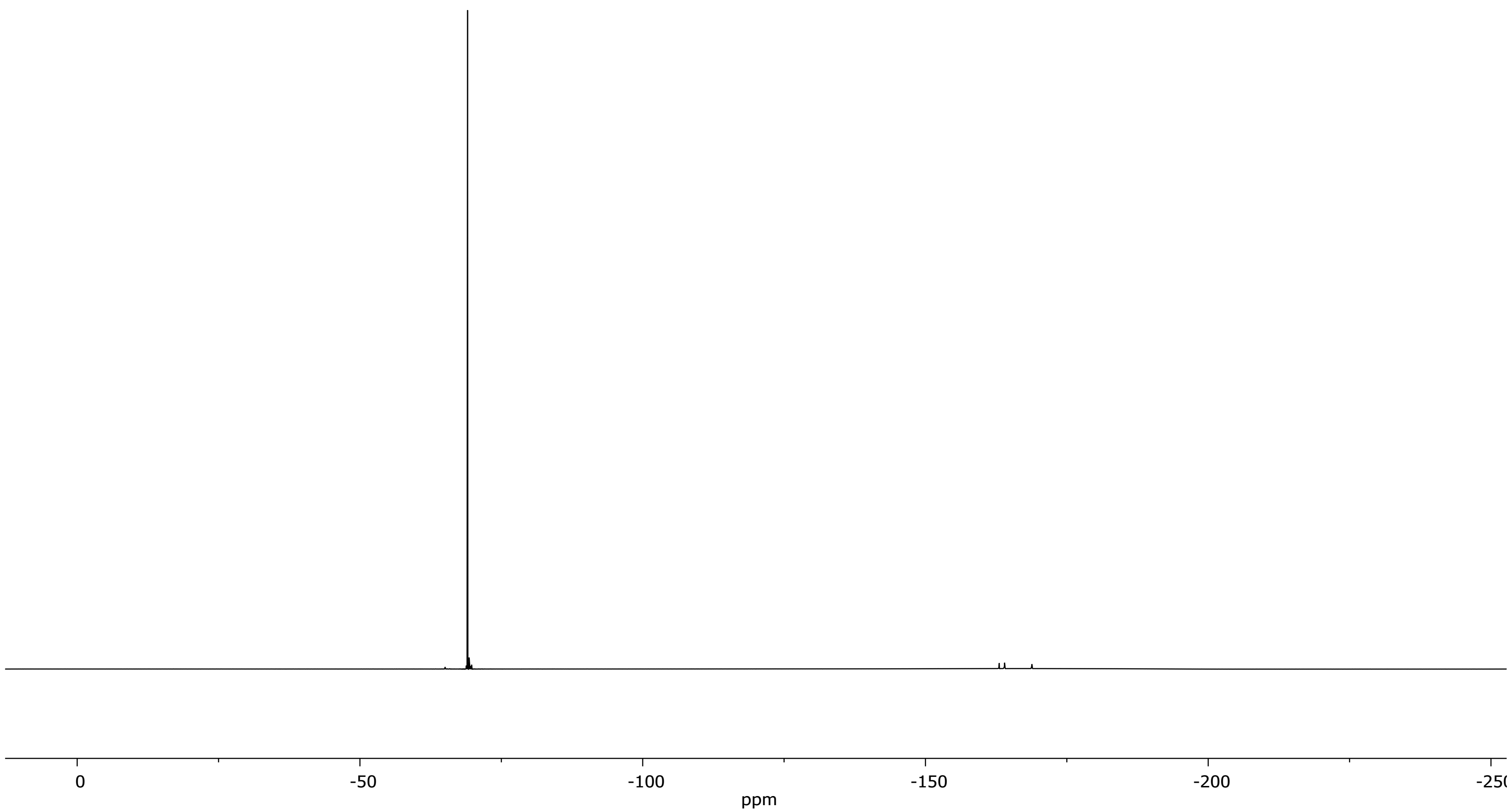

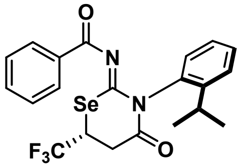

70

$^{13}C\{^{1}H\}$, $CDCl_3$, 126 MHz

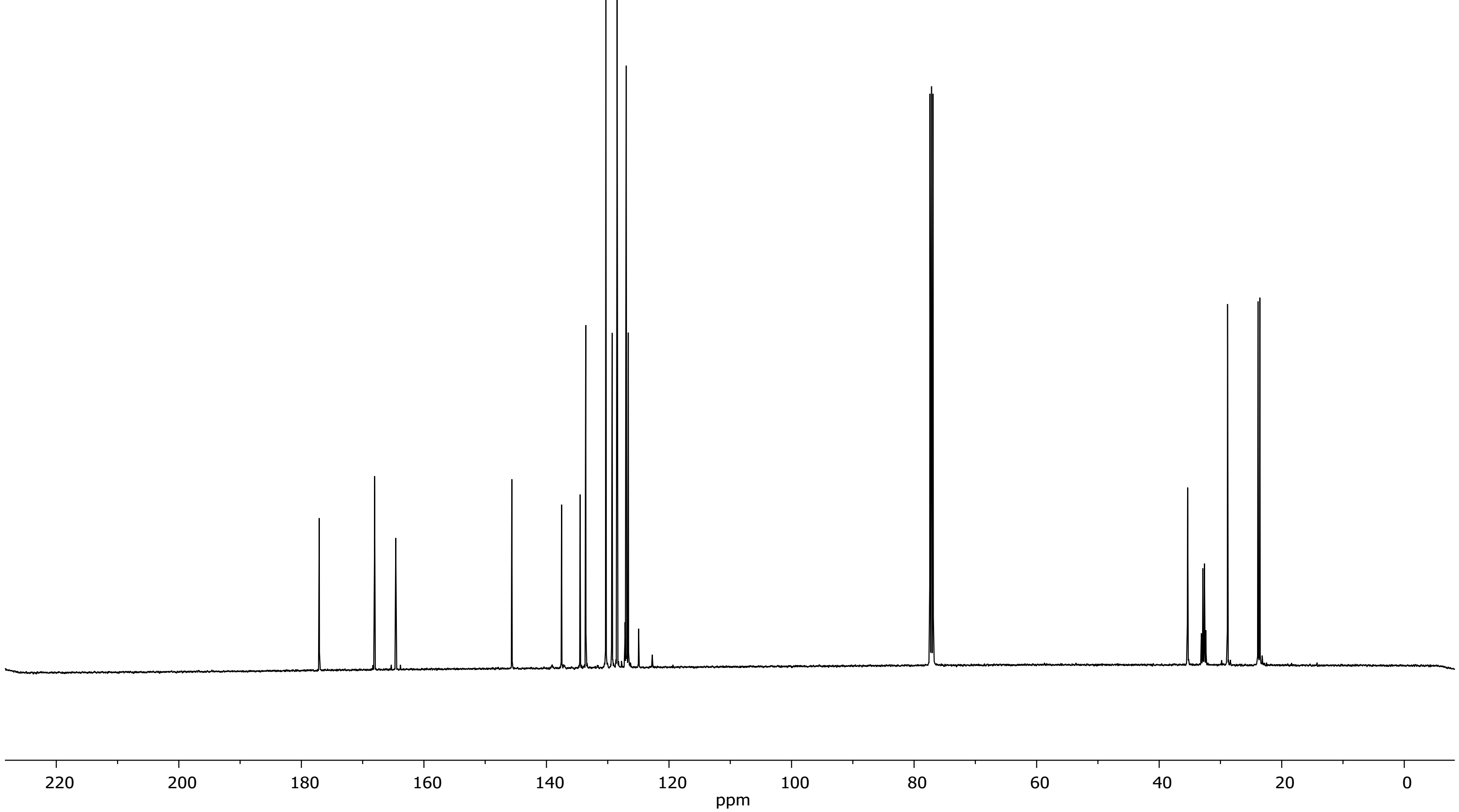

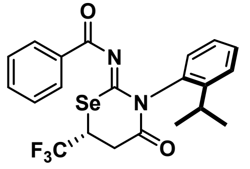

70

^{77}Se{^{1}H}, CDCl$_3$, 95 MHz

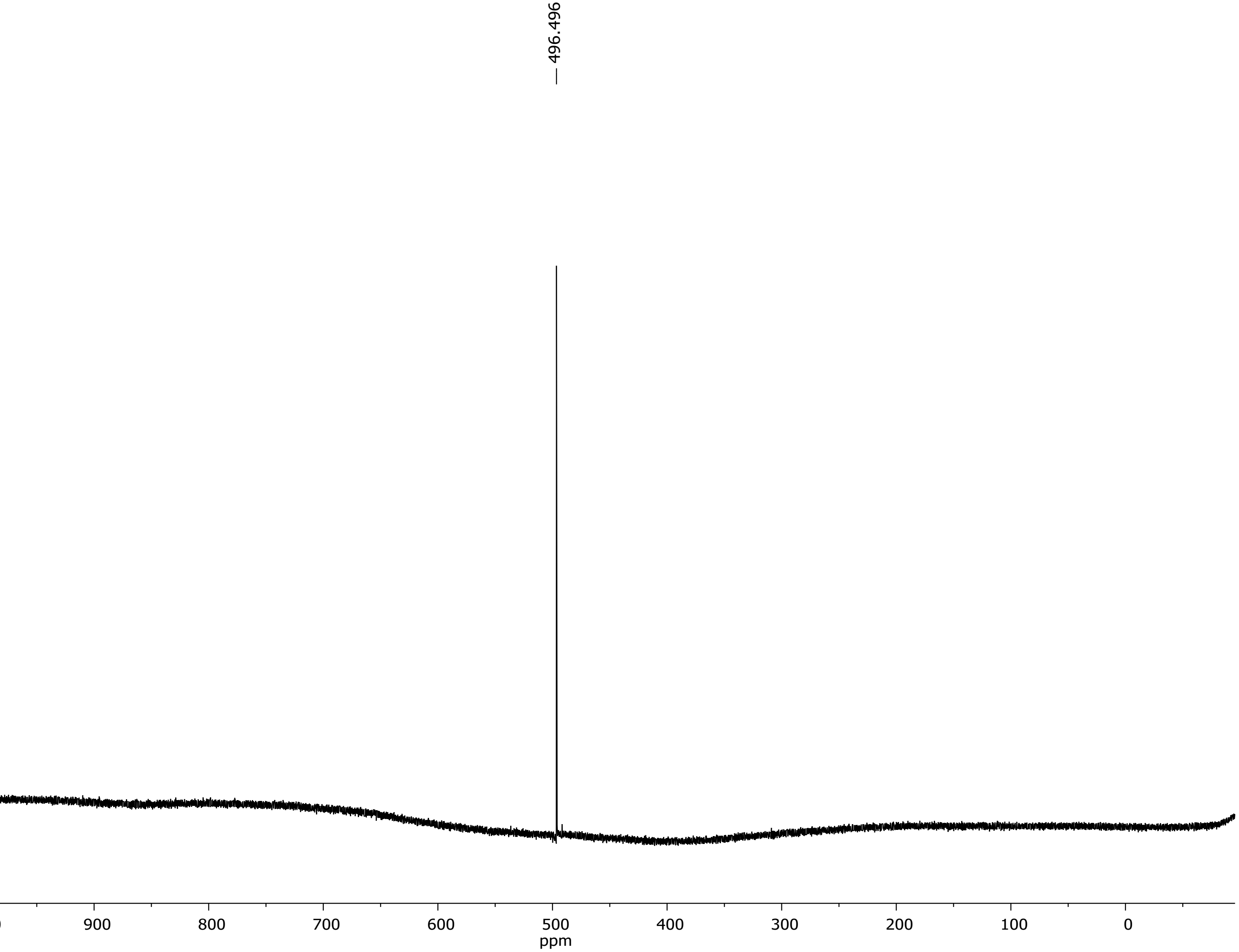

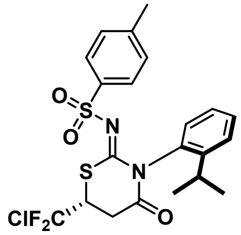

71

^{1}H, CDCl$_3$, 400 MHz

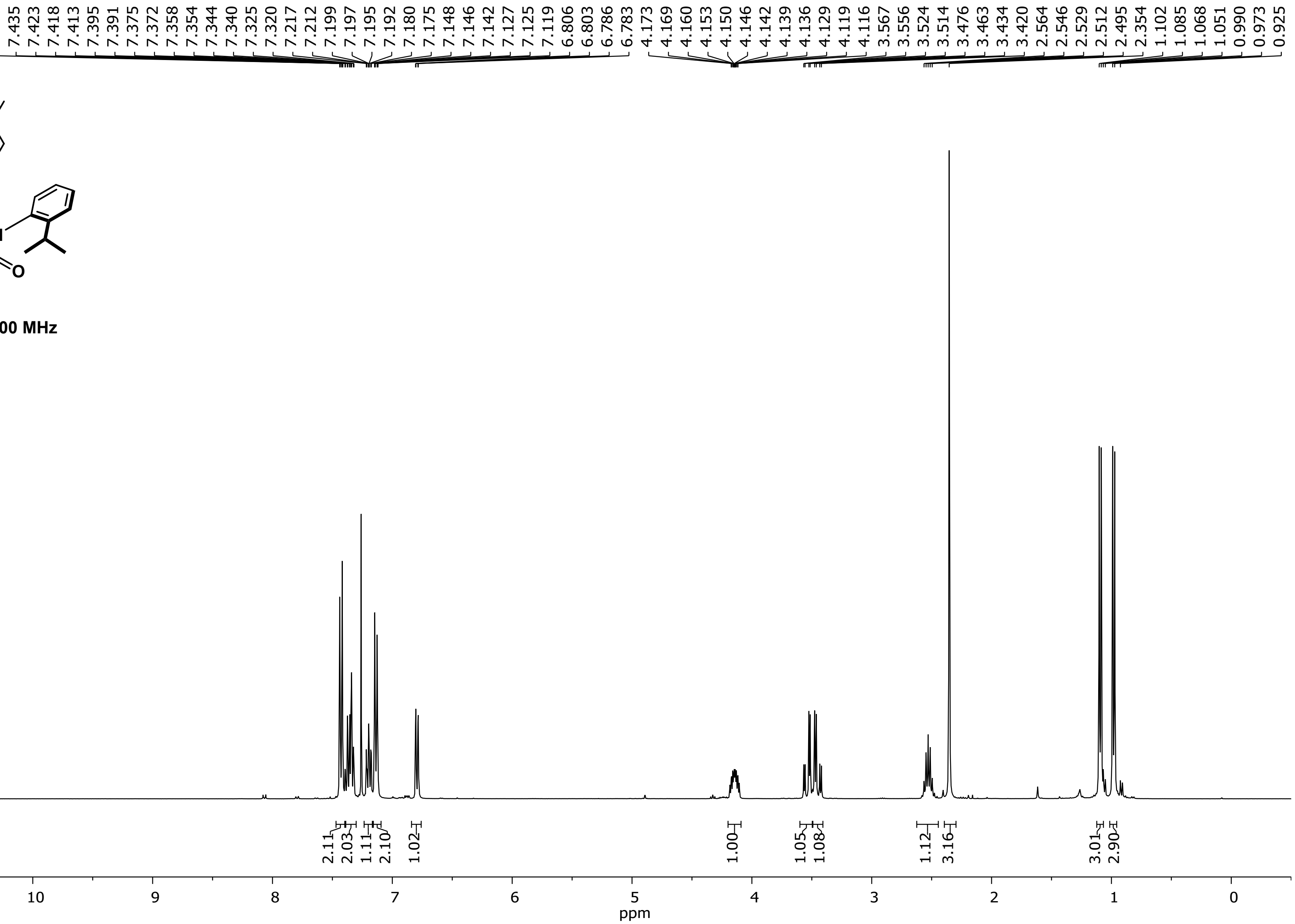

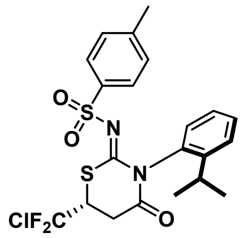

71

$^{19}F\{^1H\}$, $CDCl_3$, 377 MHz

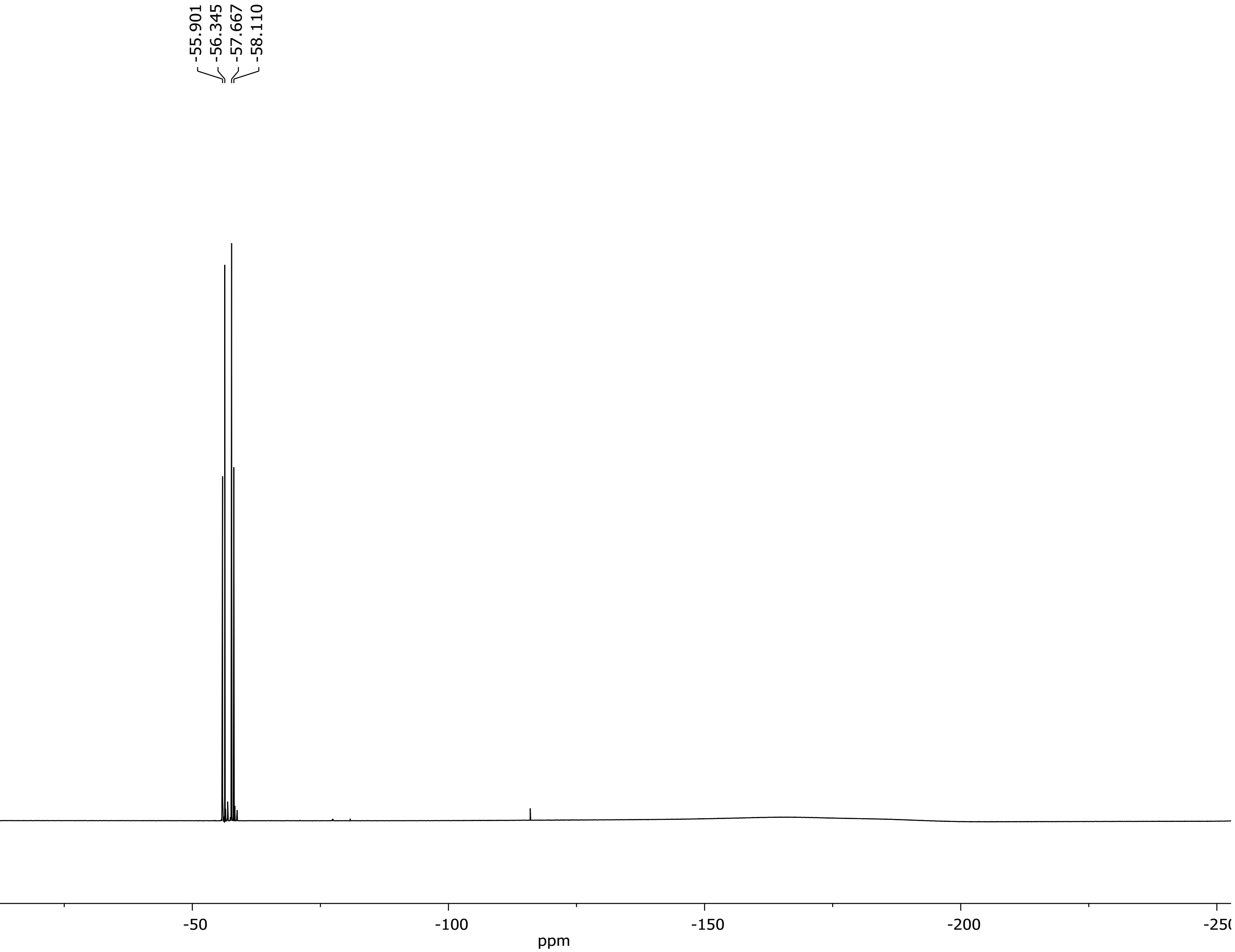

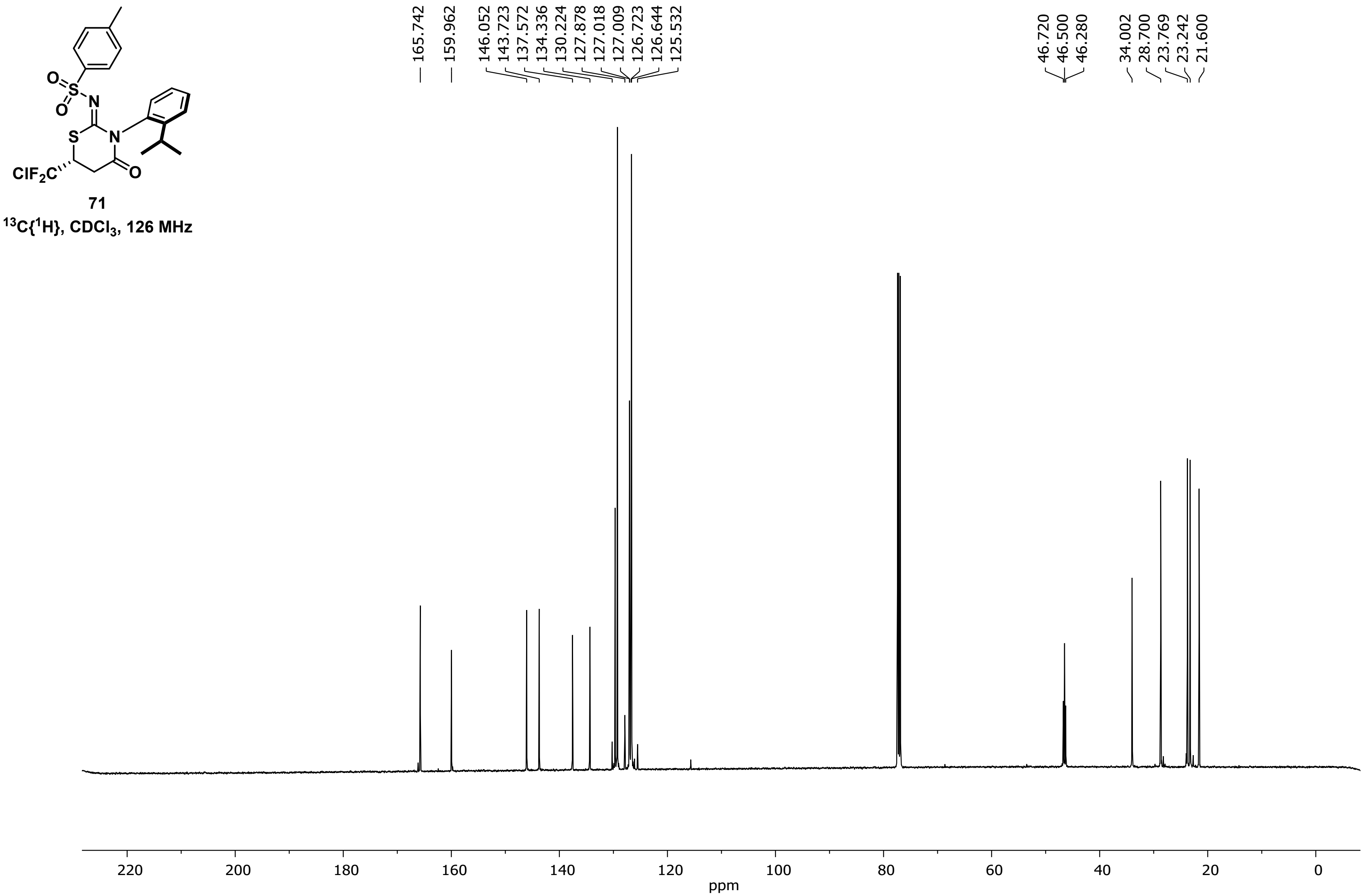
71
13C{1H}, CDCl3, 126 MHz
165.742
159.962
146.052
143.723
137.572
134.336
130.224
127.878
127.018
127.009
126.723
126.644
125.532
46.720
46.500
46.280
34.002
28.700
23.769
23.242
21.600
ppm

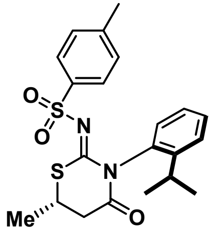

72

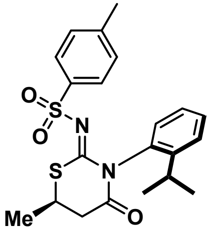

S44

^{1}H, $CDCl_3$, 400 MHz

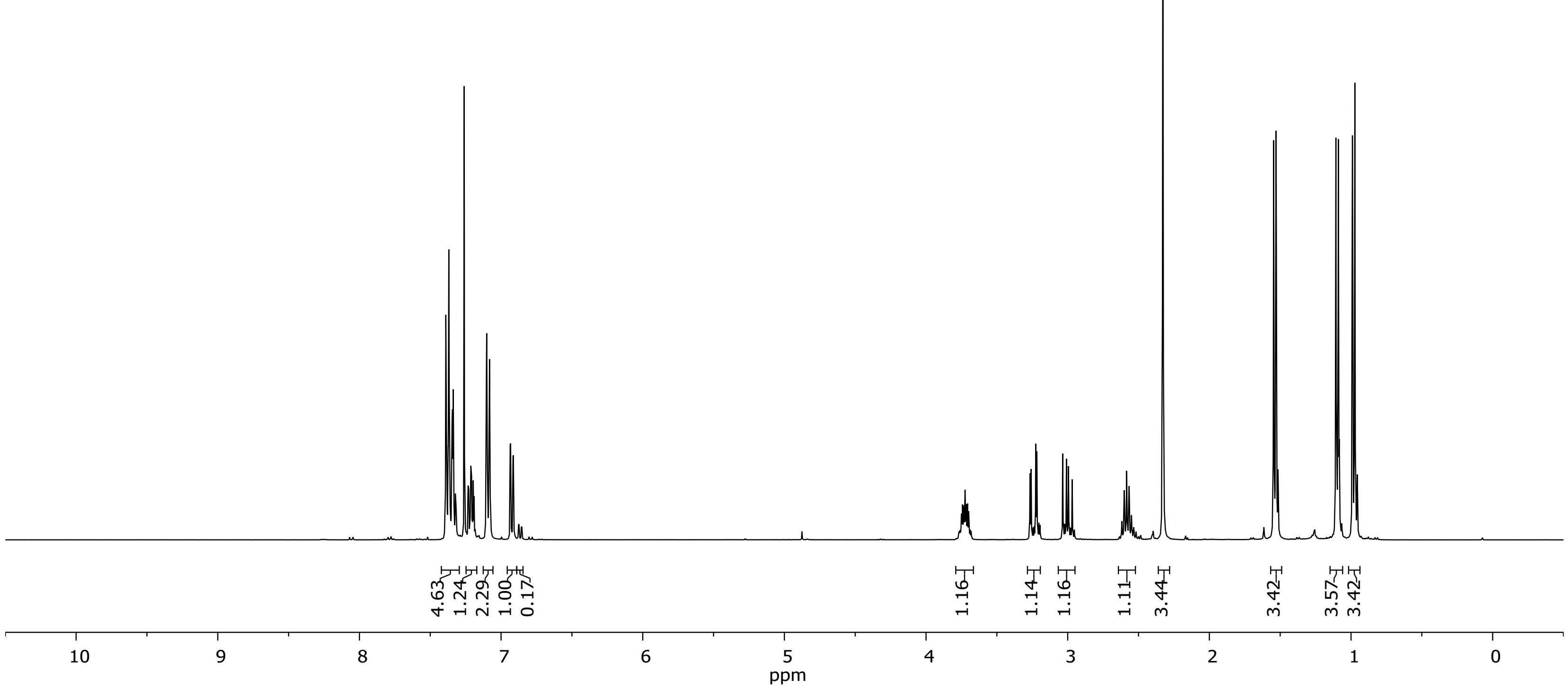

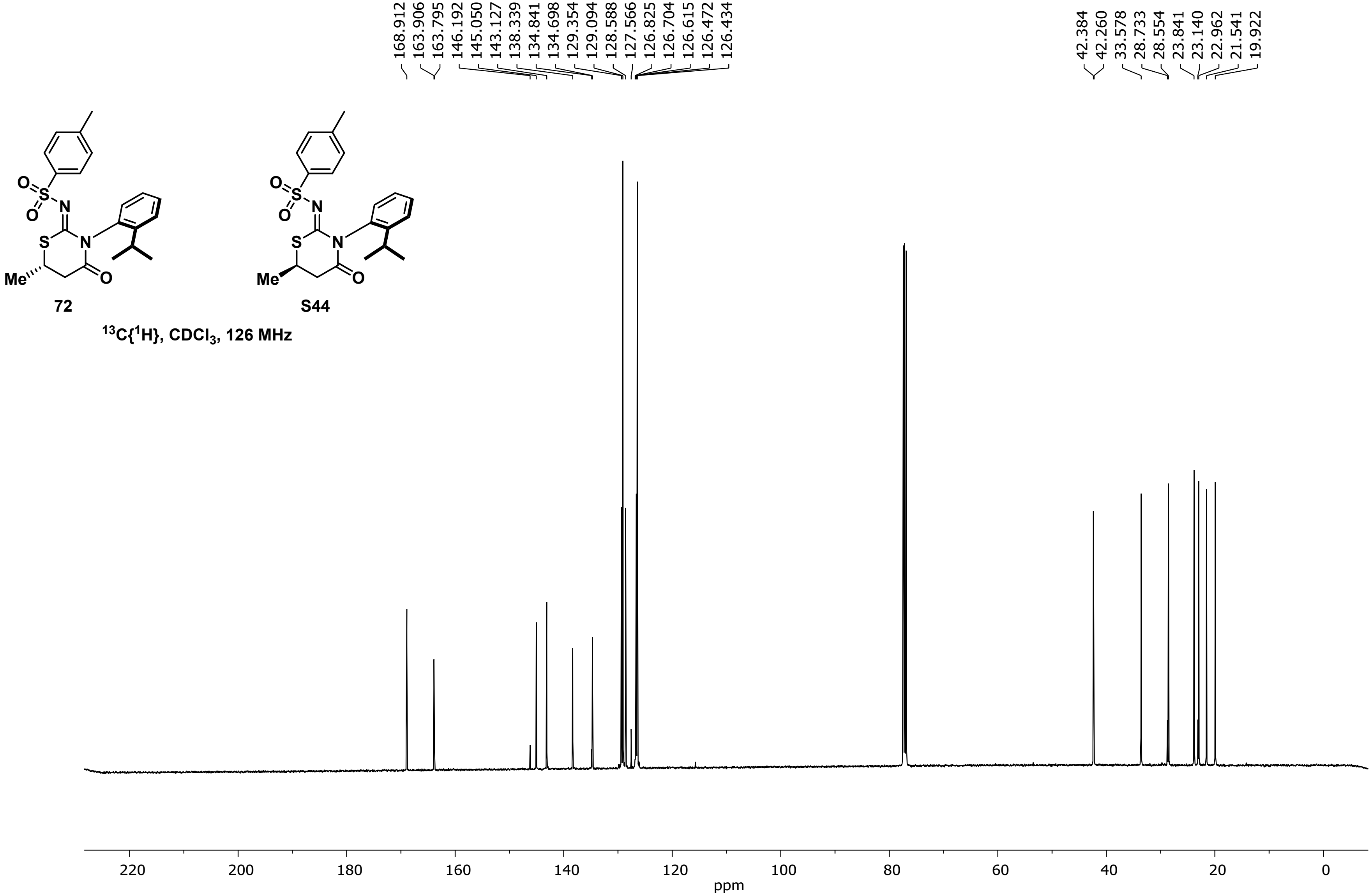

72 **S44**

$^{13}C\{^1H\}$, $CDCl_3$, 126 MHz

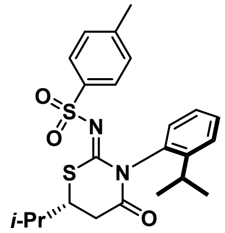

73

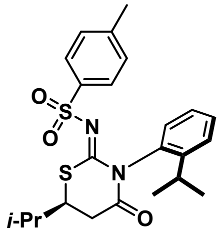

S45

^{1}H, CDCl$_3$, 400 MHz

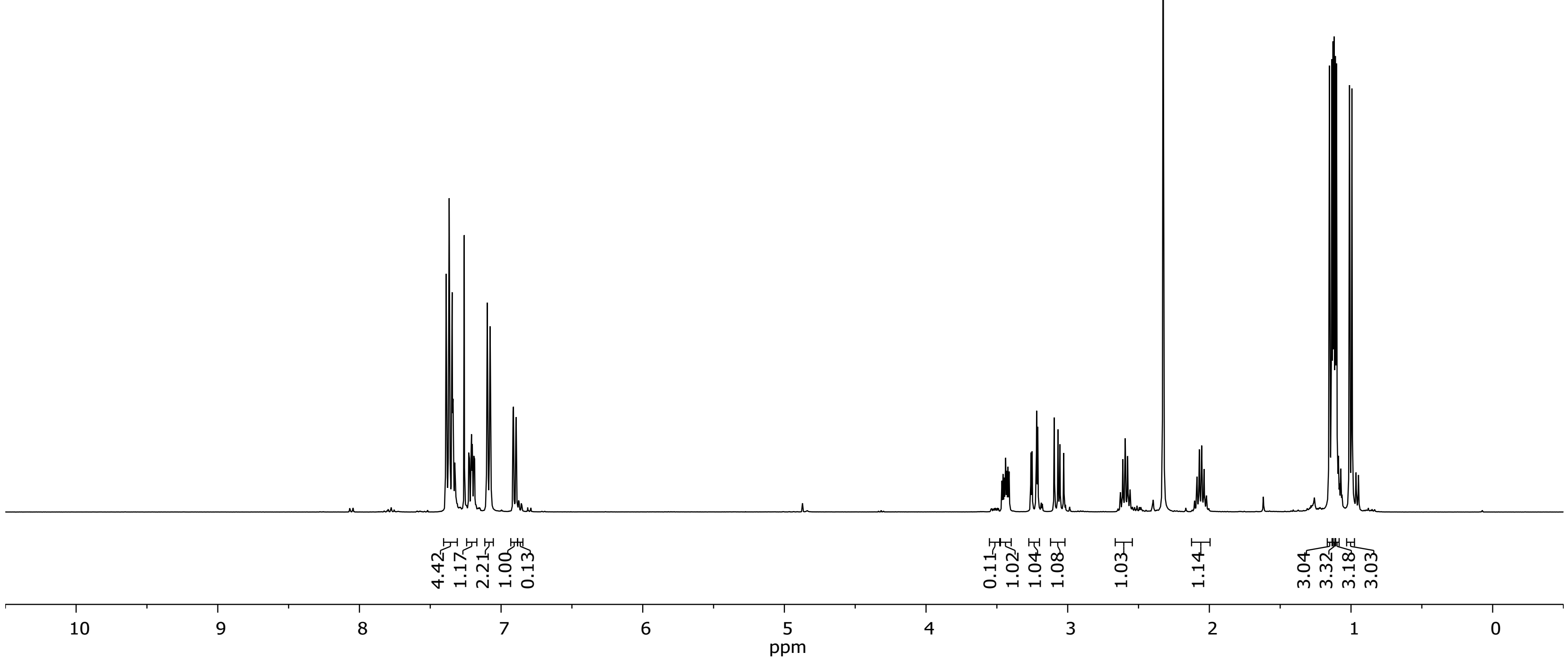

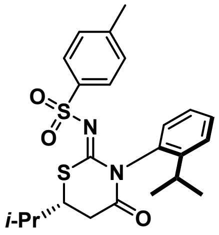

73

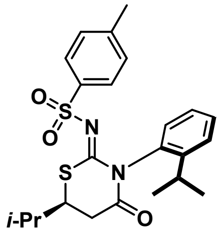

S45

$^{13}C\{^1H\}$, $CDCl_3$, 126 MHz

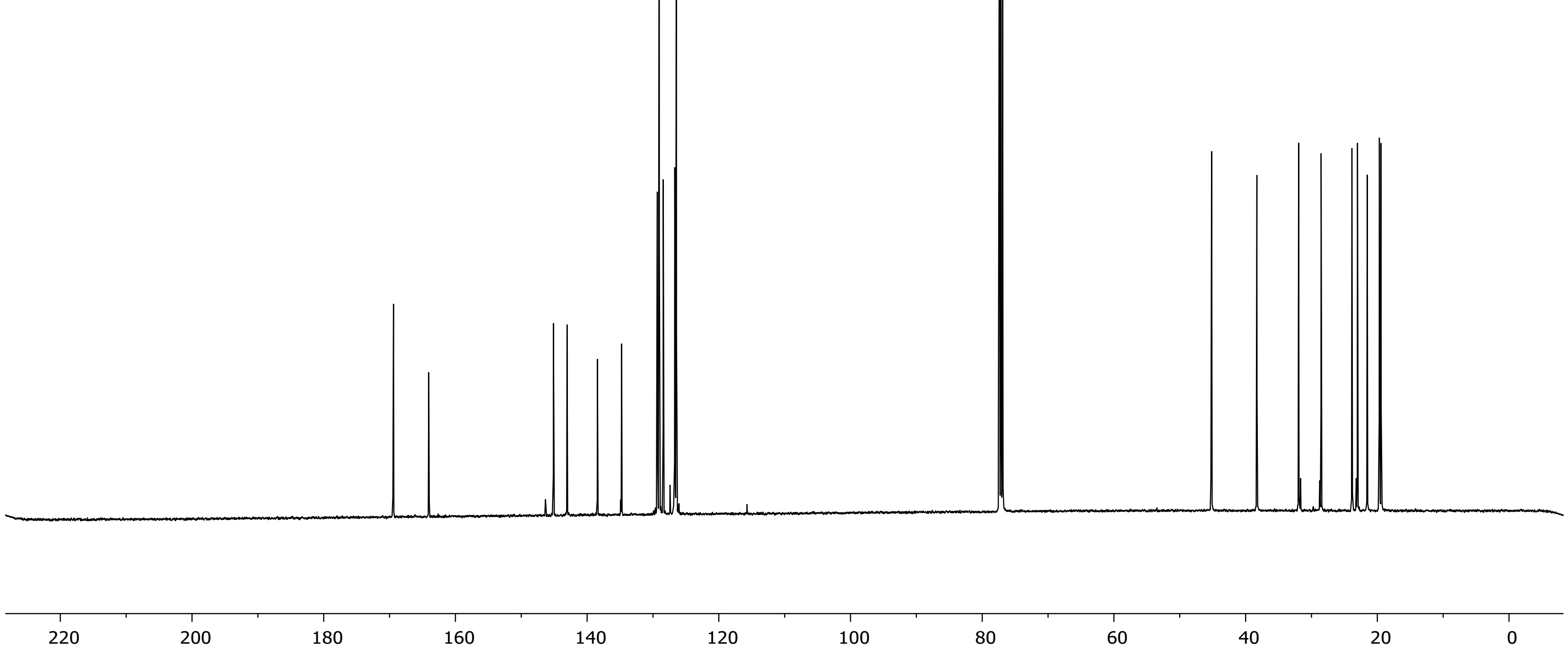

Appendix II: HPLC Traces of Novel Compounds

HPLC data for **33**: Chiralcel OD-H (90:10 *n*-hexane : IPA, flow rate 1.0 mLmin^{-1}, 254 nm, 30 °C) t_R (*S*) 4.7 min, t_R (*R*) 5.3 min, 95:5 er.

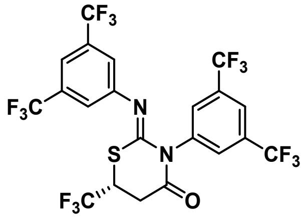

33

PDA Ch3 254nm

Peak#	Ret. Time	Area%
1	4.742	49.945
2	5.412	50.055
Total		100.000

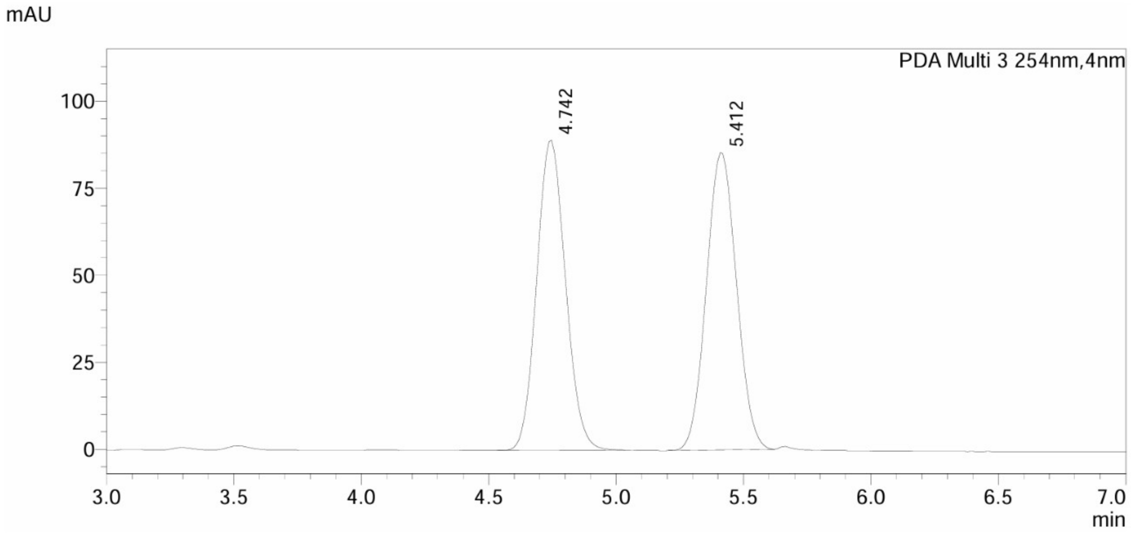

PDA Ch3 254nm

Peak#	Ret. Time	Area%
1	4.715	5.291
2	5.282	94.709
Total		100.000

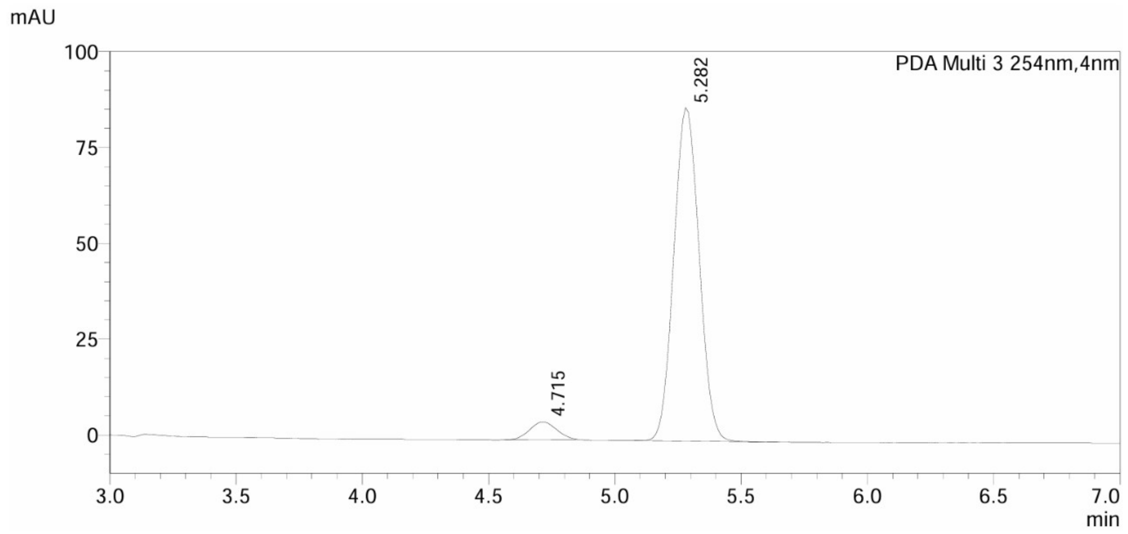

HPLC data for **35**: Chiralpak AD-H (97:3 *n*-hexane : IPA, flow rate 2.0 mLmin^{-1}, 220 nm, 40 °C) t_R (*R*) 9.1 min, t_R (*S*) 14.5 min, 95:5 er.

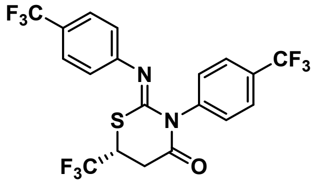

35

PDA Ch2 220nm

Peak#	Ret. Time	Area%
1	9.203	49.766
2	13.670	50.234
Total		100.000

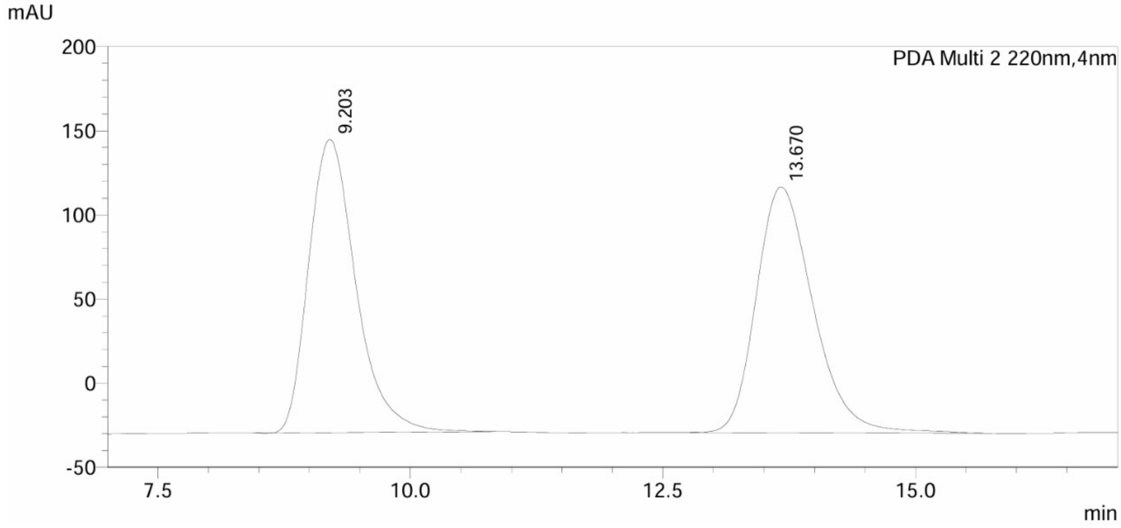

PDA Ch2 220nm

Peak#	Ret. Time	Area%
1	9.139	94.825
2	14.532	5.175
Total		100.000

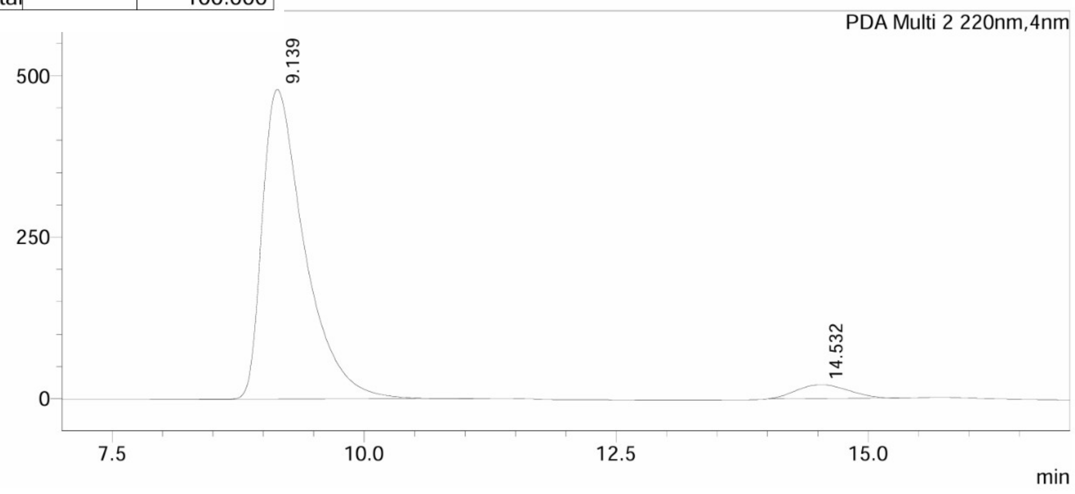

HPLC data for **36**: Chiralpak AD-H (70:30 *n*-hexane : IPA, flow rate 1.0 $mLmin^{-1}$, 211 nm, 30 °C) t_R (*R*) 13.1 min, t_R (*S*) 24.7 min, 98:2 er.

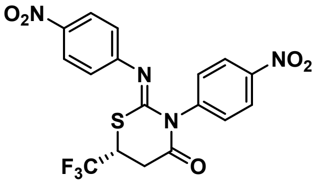

36

PDA Ch1 211nm

Peak#	Ret. Time	Area%
1	13.026	50.057
2	24.415	49.943
Total		100.000

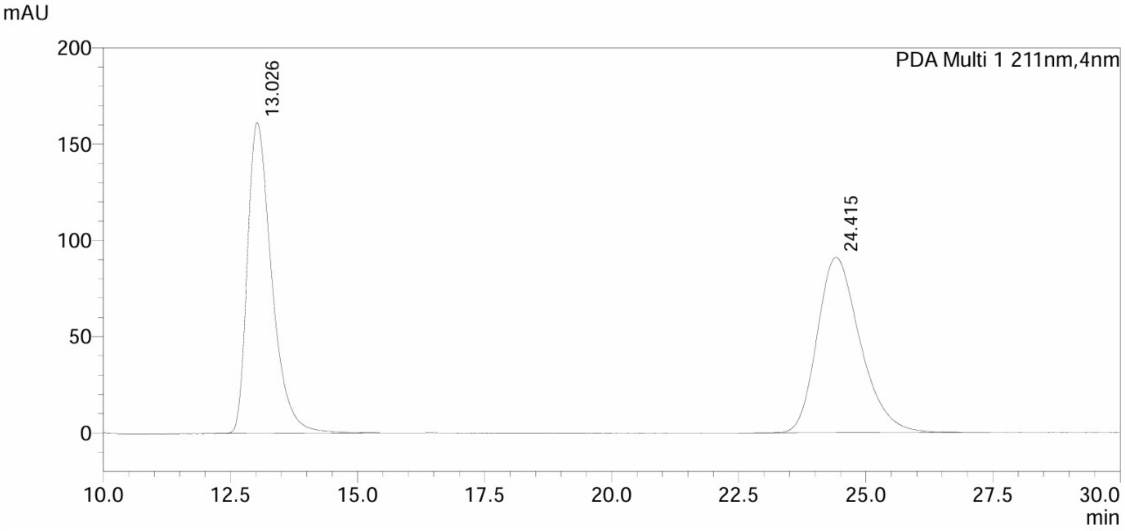

PDA Ch1 211nm

Peak#	Ret. Time	Area%
1	13.082	98.212
2	24.663	1.788
Total		100.000

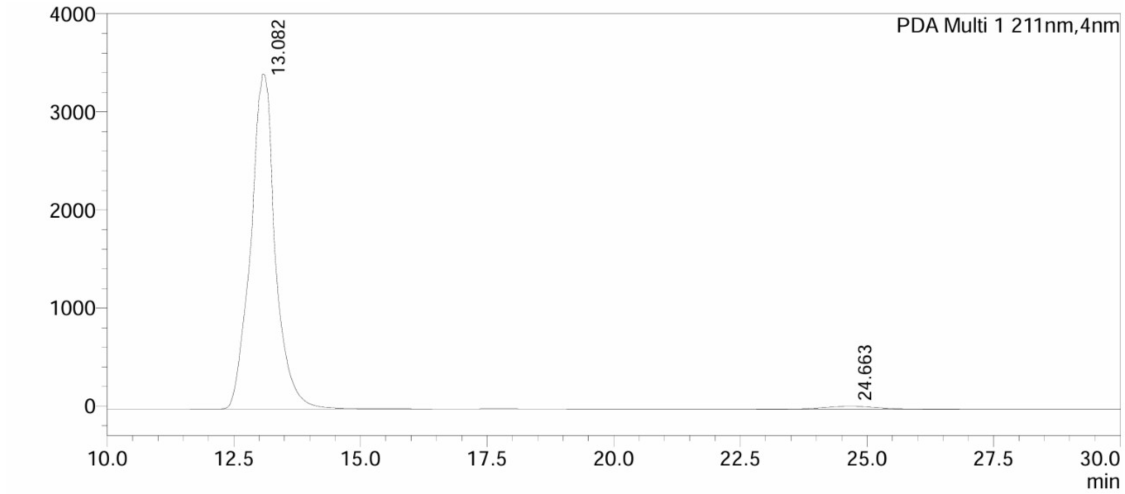

HPLC data for **37**: Chiralpak AD-H (70:30 *n*-hexane : IPA, flow rate 1.0 mLmin^{-1}, 211 nm, 30 °C) t_R (*R*) 9.8 min, t_R (*S*) 16.5 min, 97:3 er.

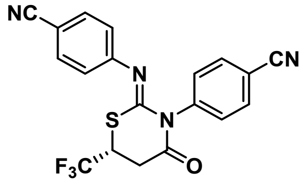

37

PDA Ch1 211nm

Peak#	Ret. Time	Area%
1	9.838	50.053
2	16.487	49.947
Total		100.000

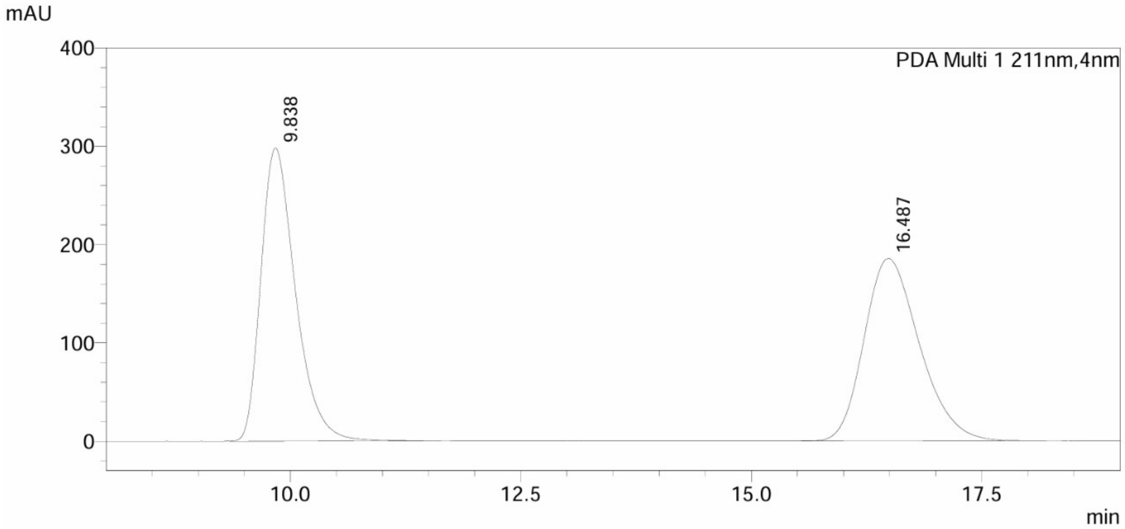

PDA Ch1 211nm

Peak#	Ret. Time	Area%
1	9.795	97.135
2	16.530	2.865
Total		100.000

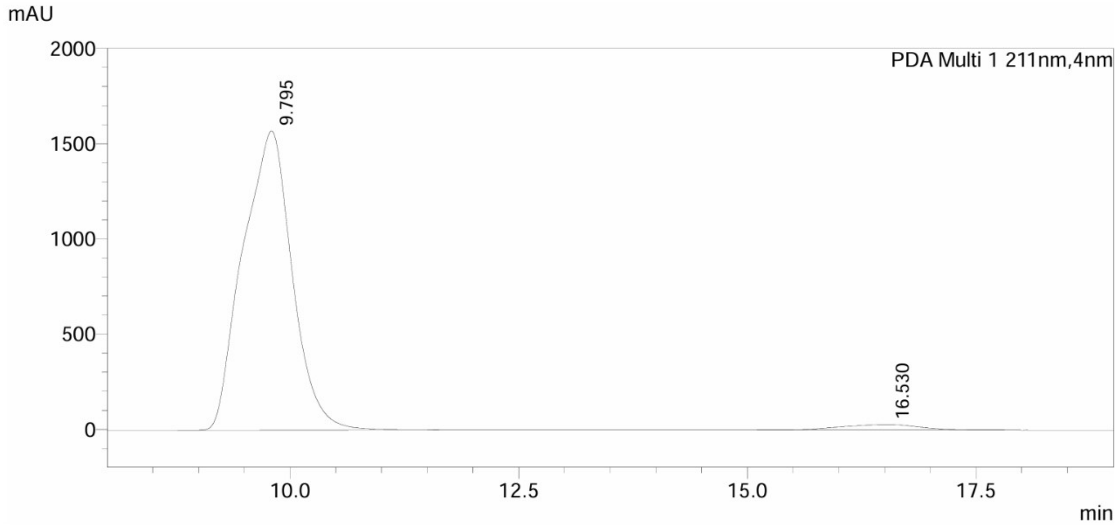

HPLC data for **38**: Chiralpak AD-H (70:30 *n*-hexane : IPA, flow rate 1.0 mLmin^{-1}, 211 nm, 30 °C) t_R (*R*) 15.3 min, t_R (*S*) 23.5 min, 98:2 er.

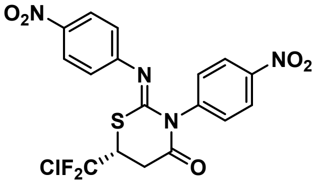

38

PDA Ch1 211nm

Peak#	Ret. Time	Area%
1	15.111	49.562
2	23.486	50.438
Total		100.000

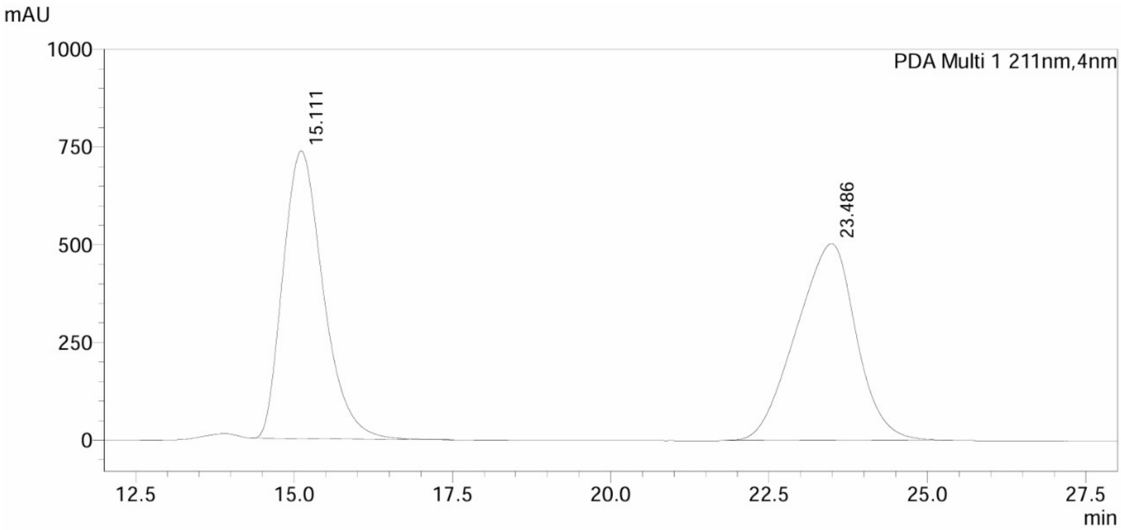

PDA Ch1 211nm

Peak#	Ret. Time	Area%
1	15.301	98.470
2	23.466	1.530
Total		100.000

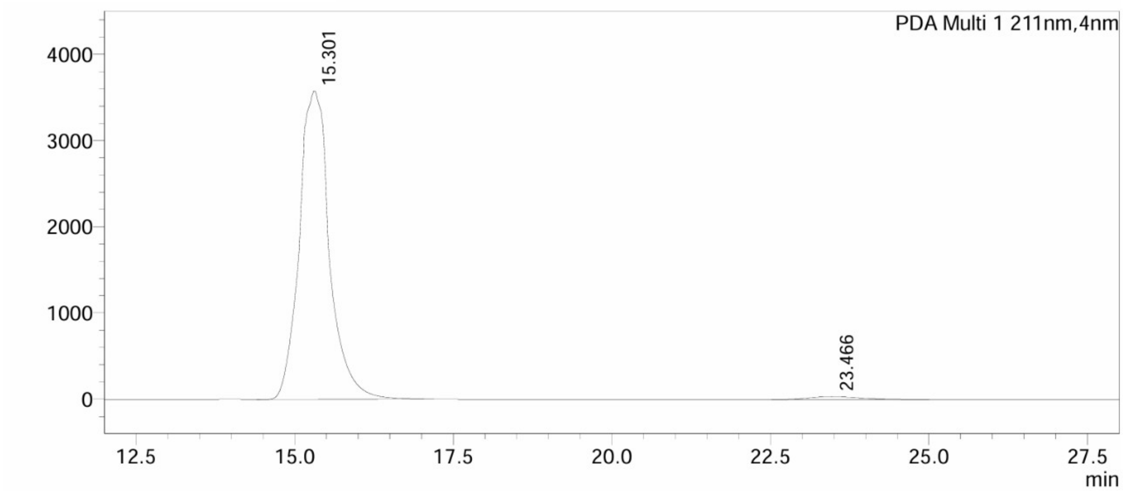

HPLC data for **39**: Chiralpak AD-H (80:20 *n*-hexane : IPA, flow rate 1.0 mLmin^{-1}, 211 nm, 30 °C) t_R (*R*) 16.9 min, t_R (*S*) 25.2 min, 98:2 er.

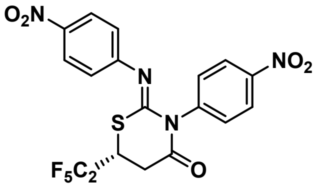

39

PDA Ch1 211nm

Peak#	Ret. Time	Area%
1	16.811	49.357
2	24.826	50.643
Total		100.000

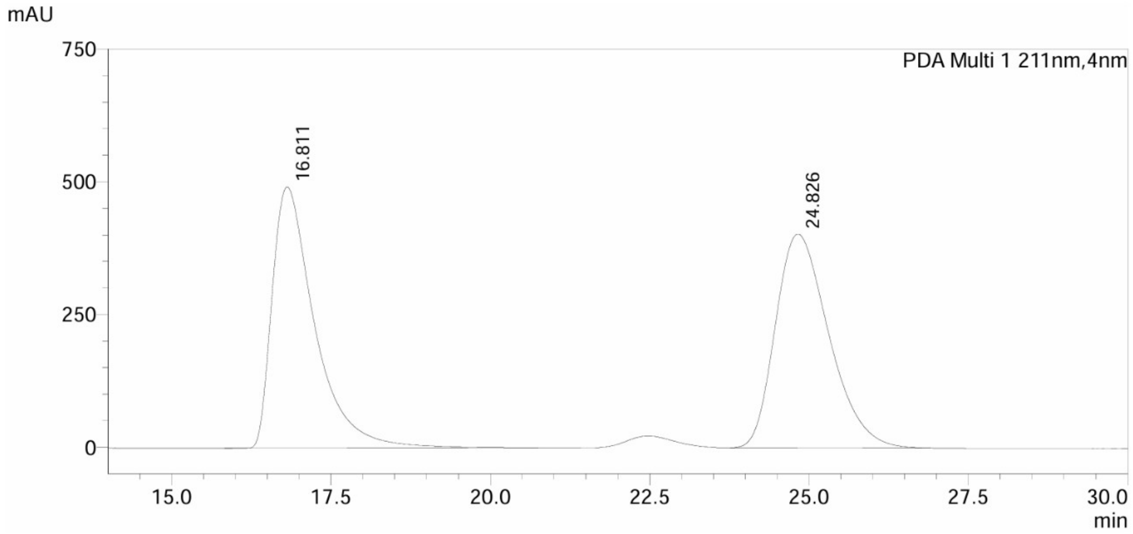

PDA Ch1 211nm

Peak#	Ret. Time	Area%
1	16.940	98.174
2	25.203	1.826
Total		100.000

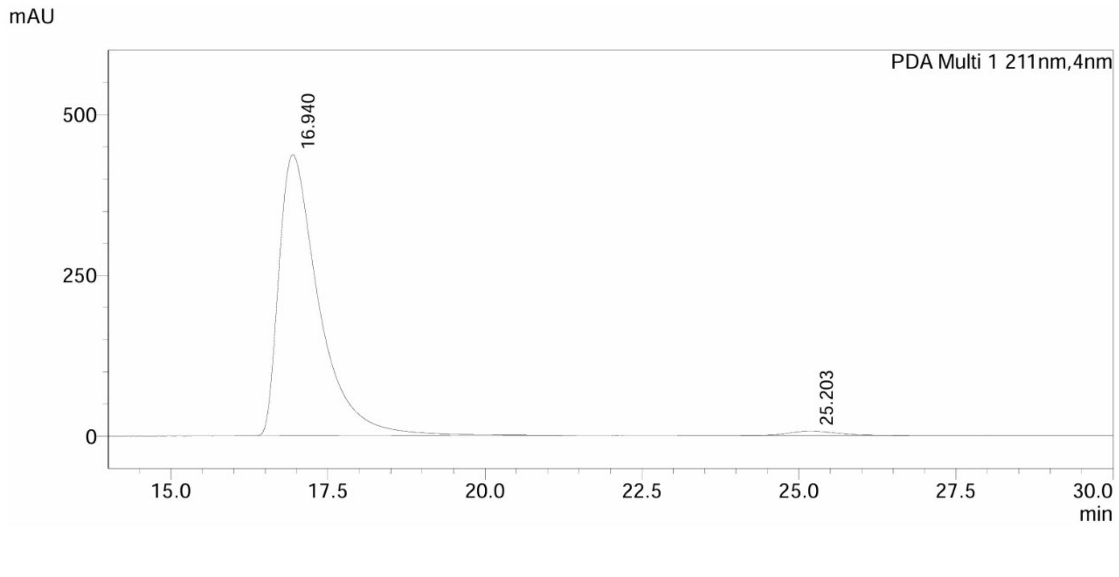

HPLC data for **S40**: Chiralpak AD-H (70:30 *n*-hexane : IPA, flow rate 1.0 mLmin^{-1}, 211 nm, 30 °C) t_R 10.2 min, t_R 16.2 min, 50:50 er.

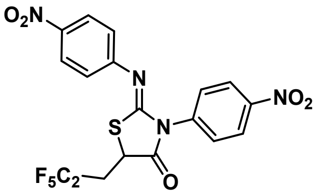

S40

PDA Ch1 211nm

Peak#	Ret. Time	Area%
1	10.167	49.986
2	16.180	50.014
Total		100.000

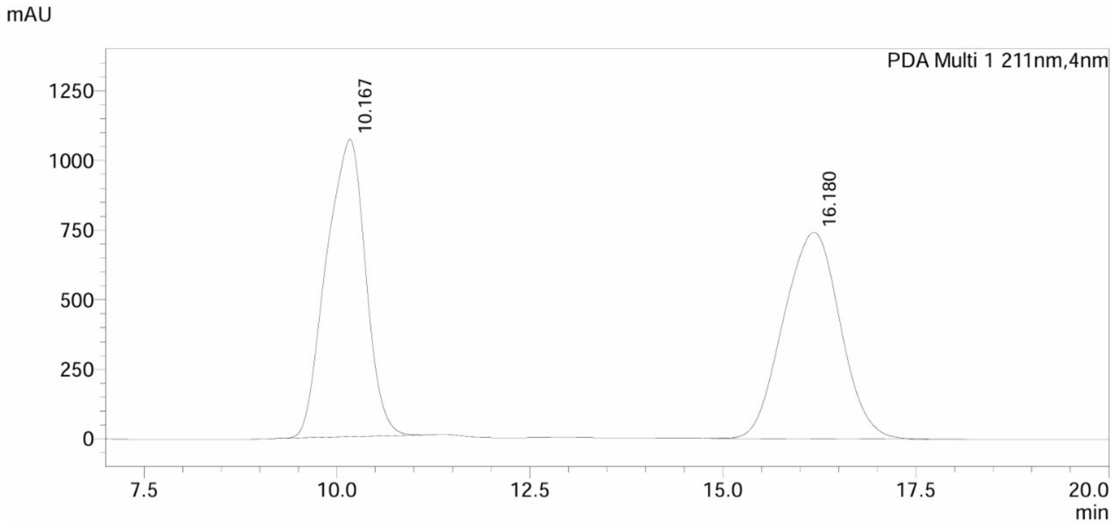

HPLC data for **40**: Chiralpak AD-H (70:30 *n*-hexane : IPA, flow rate 1.0 mLmin^{-1}, 211 nm, 30 °C) t_R (*R*) 24.4 min, t_R (*S*) 34.1 min, 80:20 er.

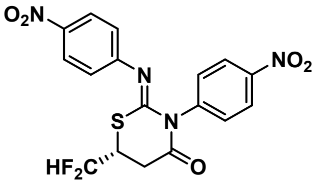

40

PDA Ch1 211nm

Peak#	Ret. Time	Area%
1	23.610	49.916
2	34.021	50.084
Total		100.000

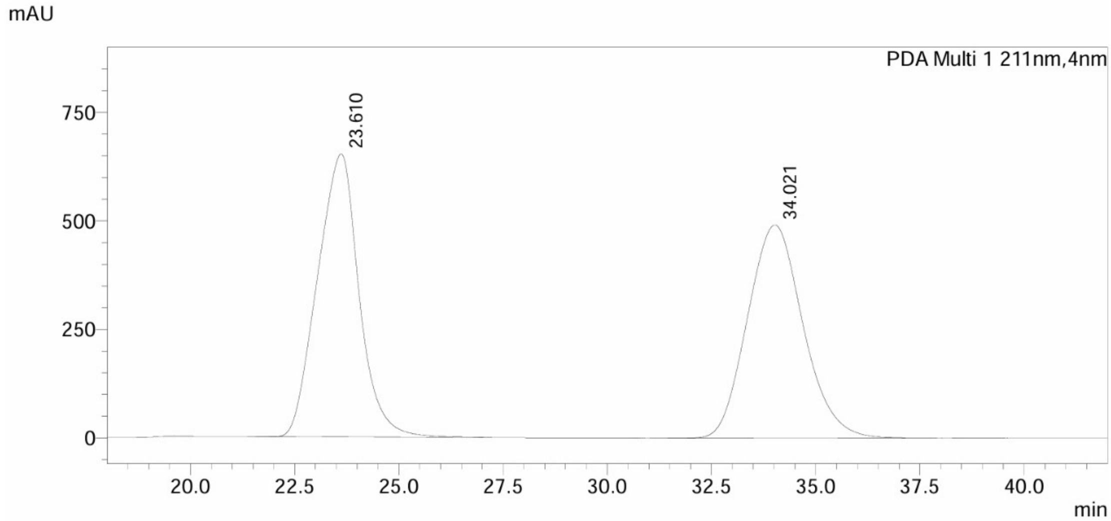

PDA Ch1 211nm

Peak#	Ret. Time	Area%
1	24.413	79.786
2	34.088	20.214
Total		100.000

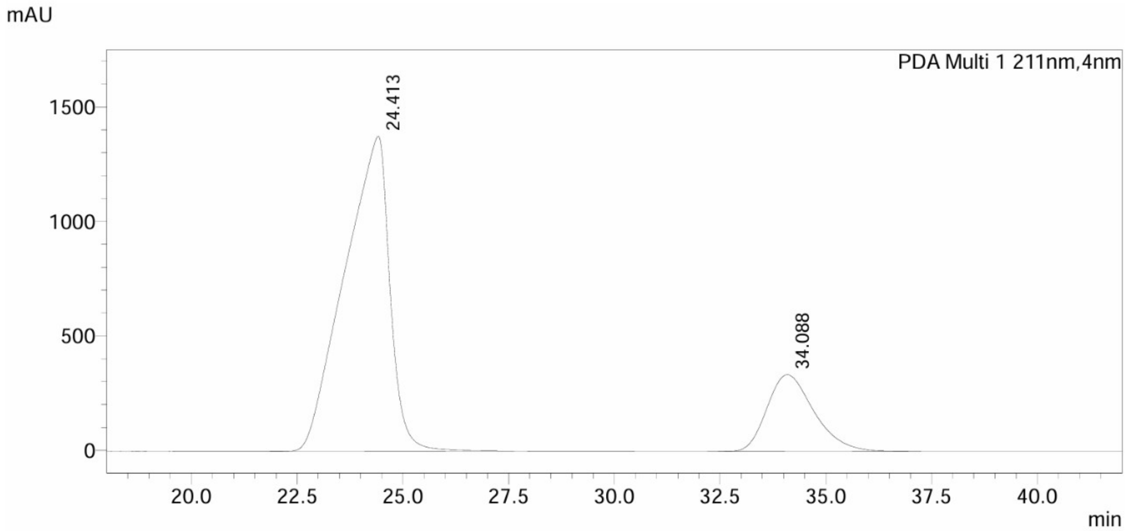

HPLC data for **41**: Chiralcel OJ-H (50:50 *n*-hexane : IPA, flow rate 0.7 mLmin^{-1}, 211 nm, 40 °C) t_R (*S*) 37.1 min, t_R (*R*) 44.5 min, 83:17 er.

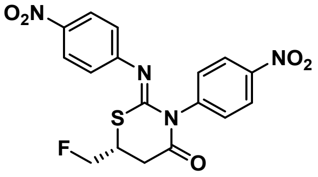

41

PDA Ch1 211nm

Peak#	Ret. Time	Area%
1	37.705	50.087
2	45.625	49.913
Total		100.000

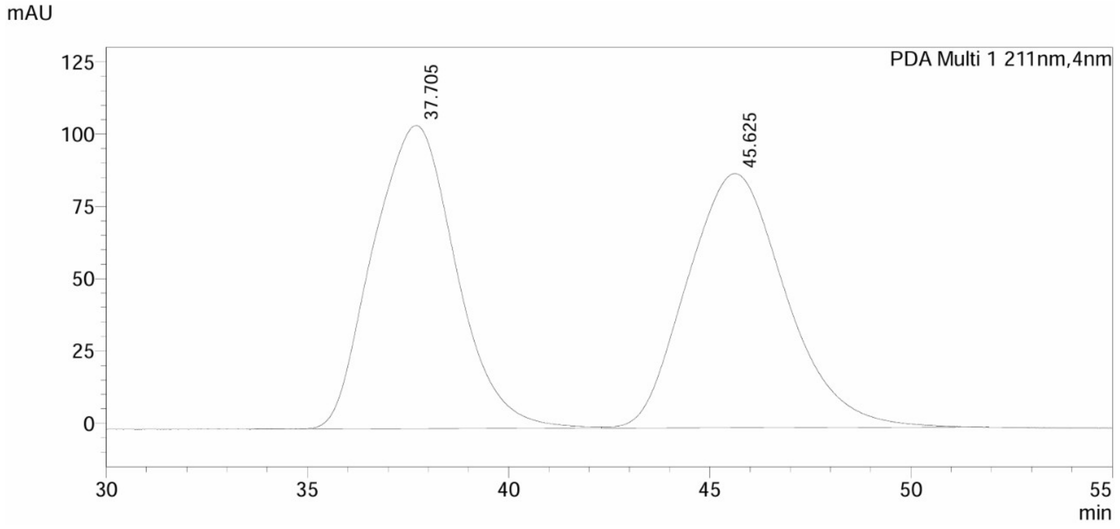

PDA Ch1 211nm

Peak#	Ret. Time	Area%
1	37.141	16.636
2	44.513	83.364
Total		100.000

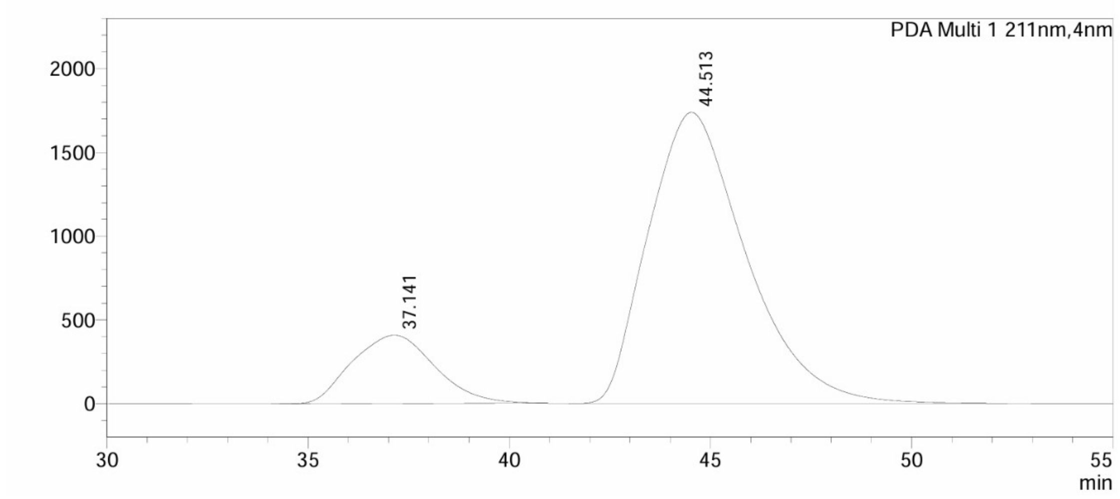

HPLC data for **42**: Chiralpak IB (80:20 *n*-hexane : IPA, flow rate 1.5 mLmin^{-1}, 211 nm, 30 °C) t_R (*R*) 19.3 min, t_R (*S*) 21.7 min, 86:14 er.

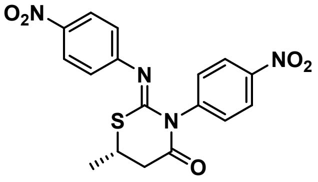

42

PDA Ch1 211nm

Peak#	Ret. Time	Area%
1	19.494	49.700
2	22.268	50.300
Total		100.000

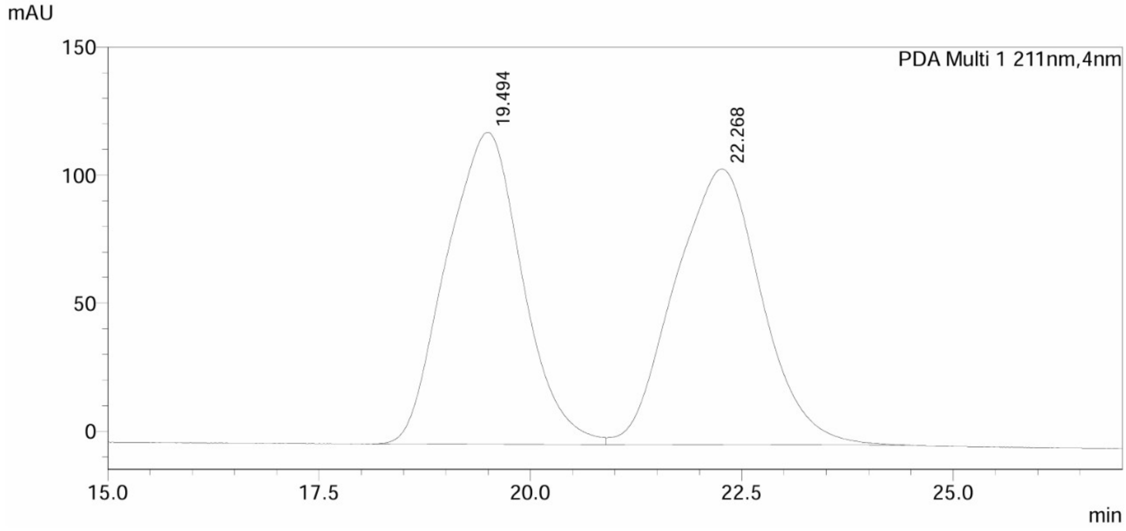

PDA Ch1 211nm

Peak#	Ret. Time	Area%
1	19.345	13.947
2	21.668	86.053
Total		100.000

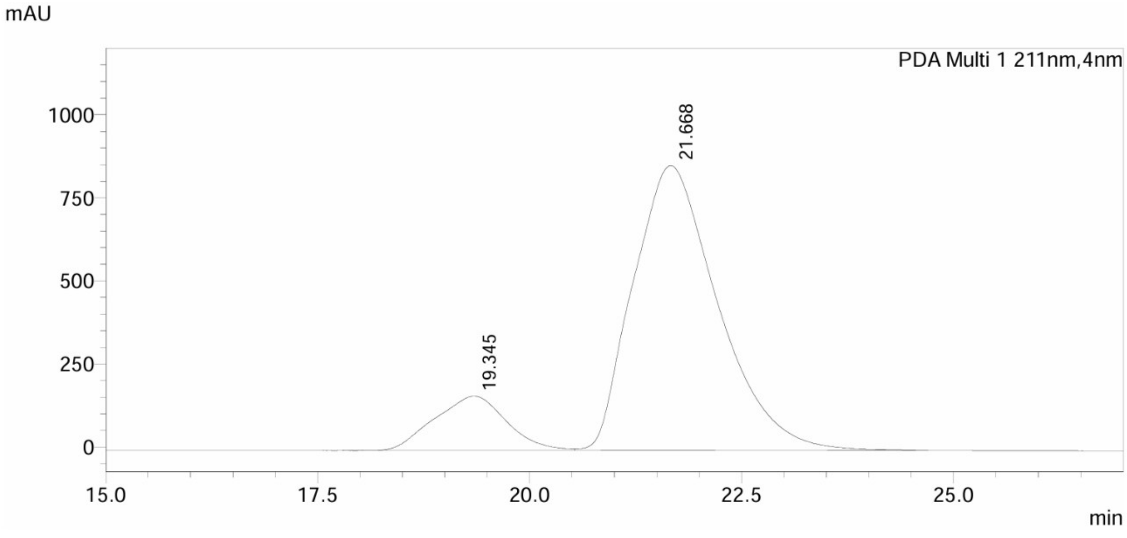

HPLC data for **43**: Chiralcel OD-H (70:30 *n*-hexane : IPA, flow rate 1.0 mLmin^{-1}, 211 nm, 30 °C) t_R (*R*) 18.1 min, t_R (*S*) 20.7 min, 89:11 er.

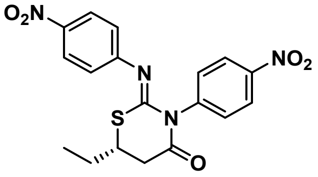

43

PDA Ch1 211nm

Peak#	Ret. Time	Area%
1	17.749	49.629
2	20.471	50.371
Total		100.000

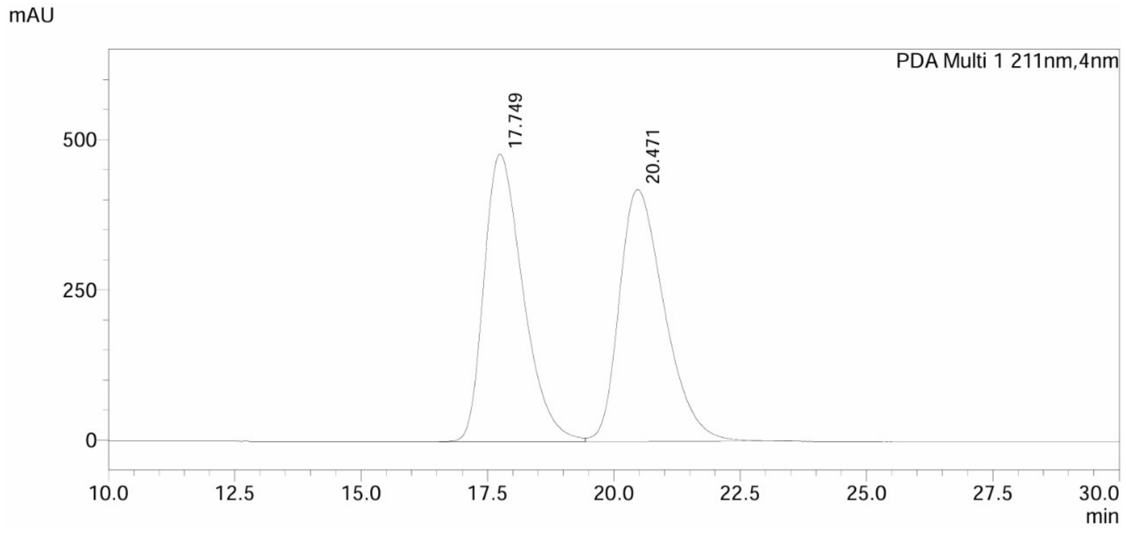

PDA Ch1 211nm

Peak#	Ret. Time	Area%
1	18.067	10.709
2	20.729	89.291
Total		100.000

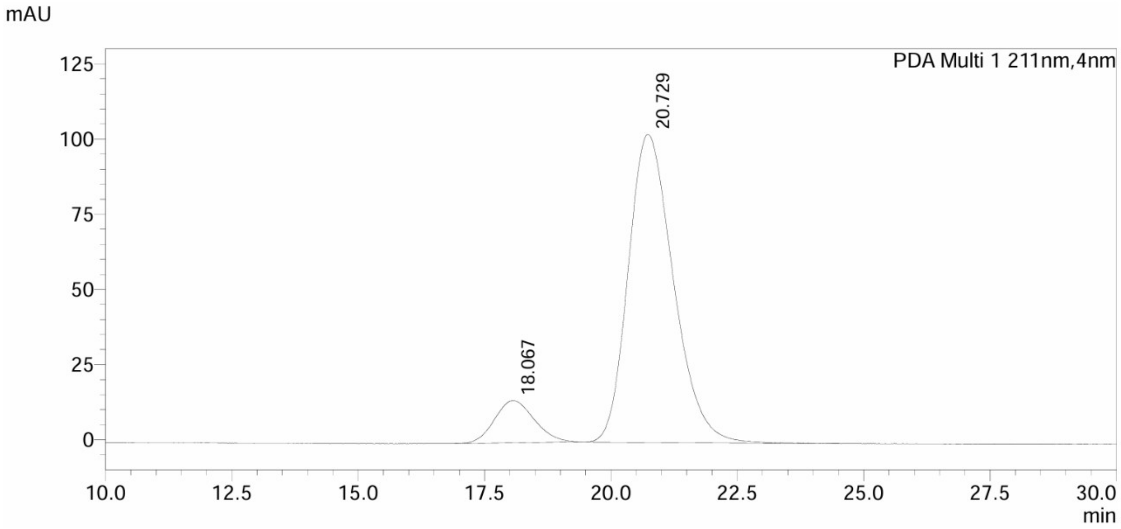

HPLC data for **44**: Chiralpak AD-H (60:40 *n*-hexane : IPA, flow rate 1.0 mLmin^{-1}, 211 nm, 30 °C) t_R (*R*) 18.3 min, t_R (*S*) 22.5 min, 94:6 er.

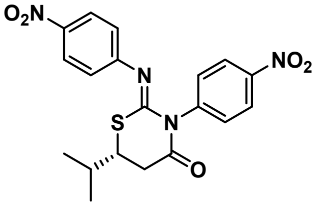

44

PDA Ch1 211nm

Peak#	Ret. Time	Area%
1	18.265	49.906
2	22.455	50.094
Total		100.000

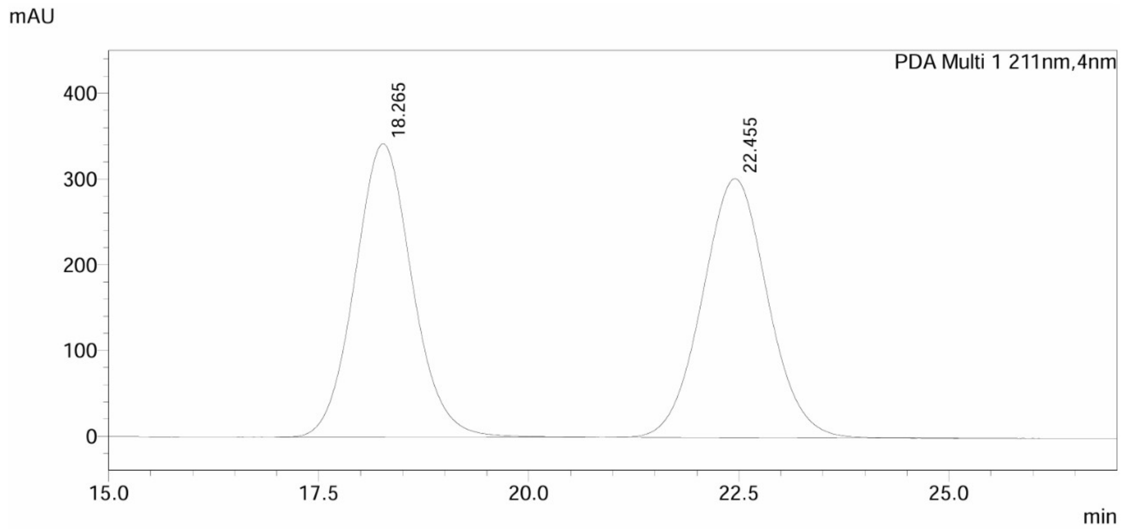

PDA Ch1 211nm

Peak#	Ret. Time	Area%
1	18.344	93.909
2	22.538	6.091
Total		100.000

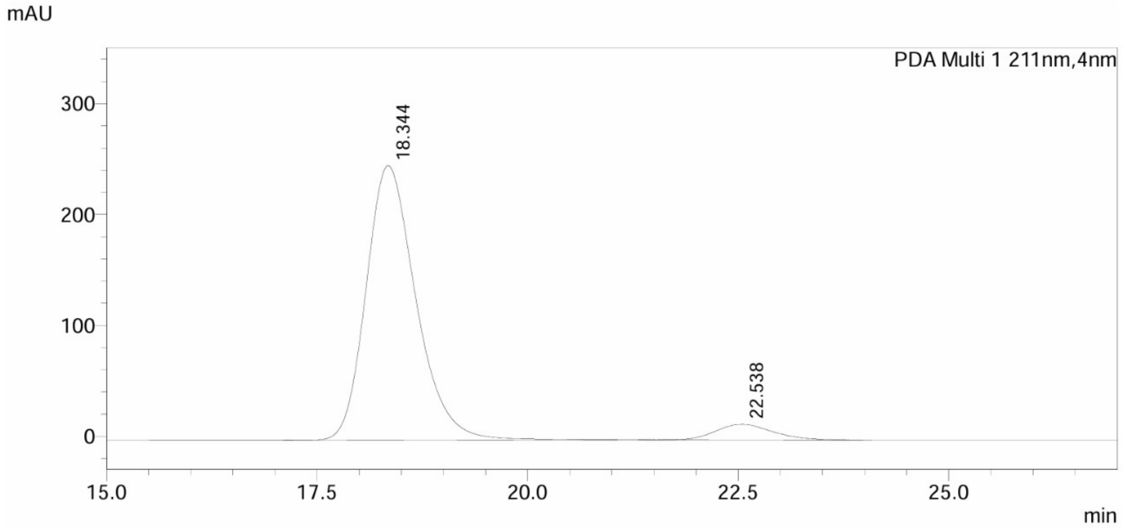

HPLC data for **45**: Chiralpak AD-H (70:30 *n*-hexane : IPA, flow rate 1.0 mLmin^{-1}, 211 nm, 30 °C) t_R (*S*) 28.8 min, t_R (*R*) 35.2 min, 86:14 er.

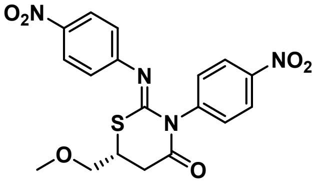

45

PDA Ch1 211nm

Peak#	Ret. Time	Area%
1	28.722	50.043
2	34.694	49.957
Total		100.000

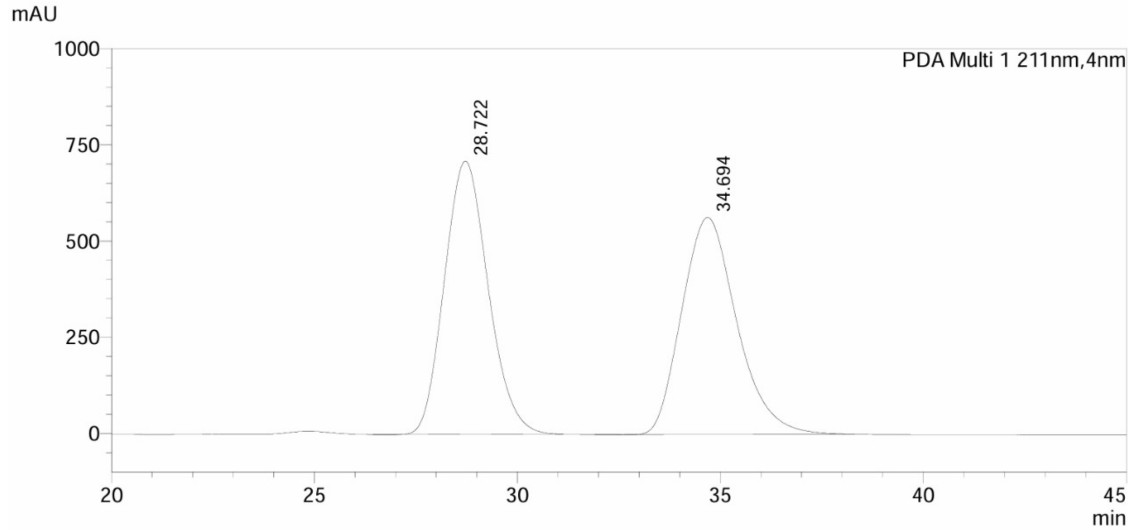

PDA Ch1 211nm

Peak#	Ret. Time	Area%
1	28.801	14.461
2	35.170	85.539
Total		100.000

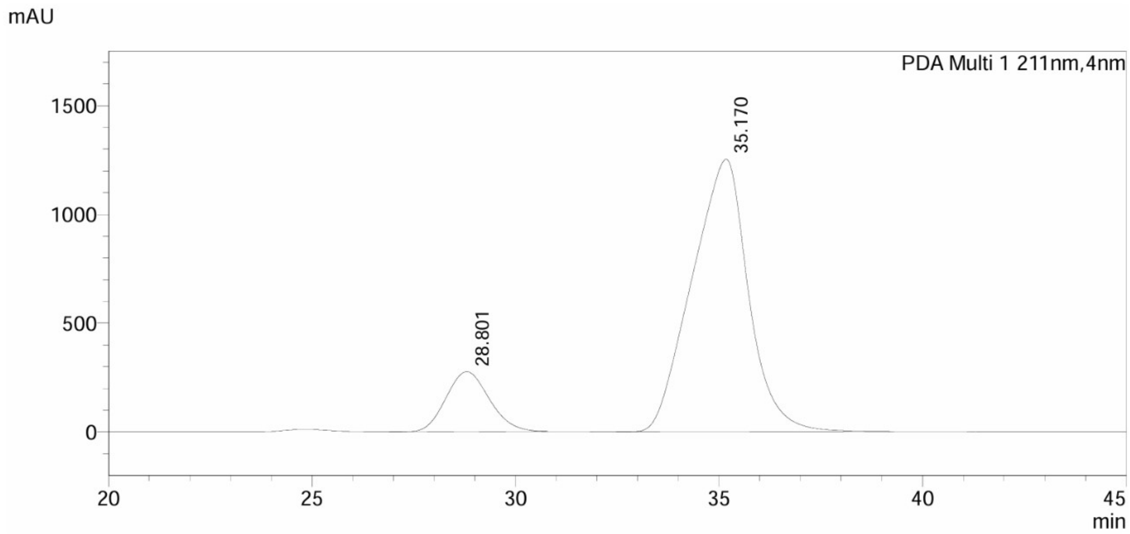

HPLC data for **46**: Chiralcel OD-H (90:10 *n*-hexane : IPA, flow rate 1.0 mLmin^{-1}, 211 nm, 30 °C) t_R 19.0 min, t_R 24.9 min, 50:50 er.

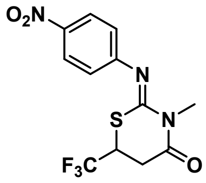

46

PDA Ch1 211nm

Peak#	Ret. Time	Area%
1	19.013	49.831
2	24.918	50.169
Total		100.000

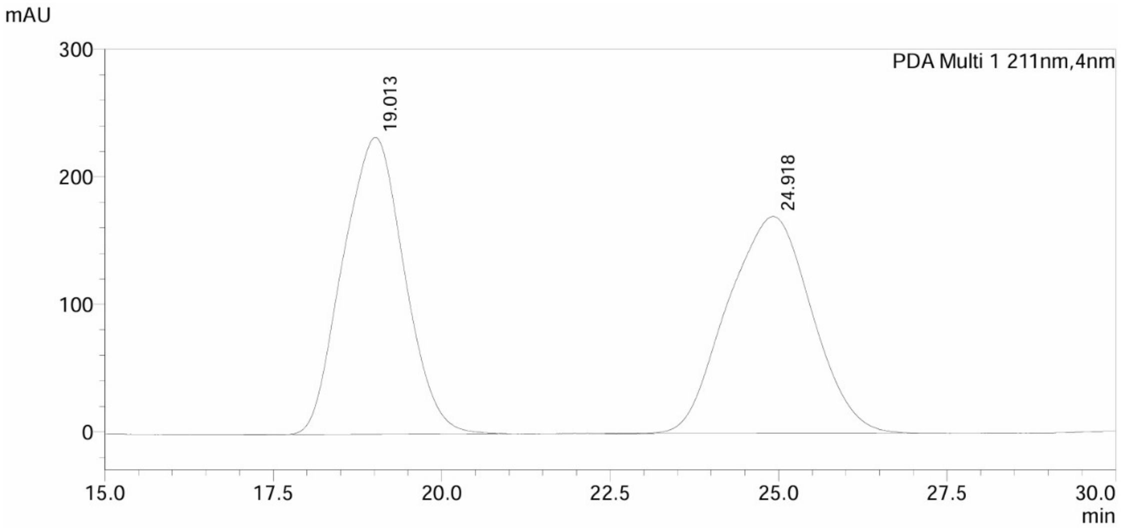

HPLC data for **47**: Chiralpak IB (80:20 *n*-hexane : IPA, flow rate 1.5 mLmin^{-1}, 254 nm, 30 °C) t_R (*S*) 11.6 min, t_R (*R*) 23.6 min, 67:33 er.

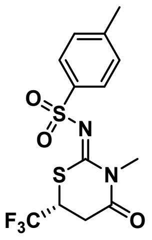

47

PDA Ch3 254nm

Peak#	Ret. Time	Area%
1	11.689	50.270
2	24.464	49.730
Total		100.000

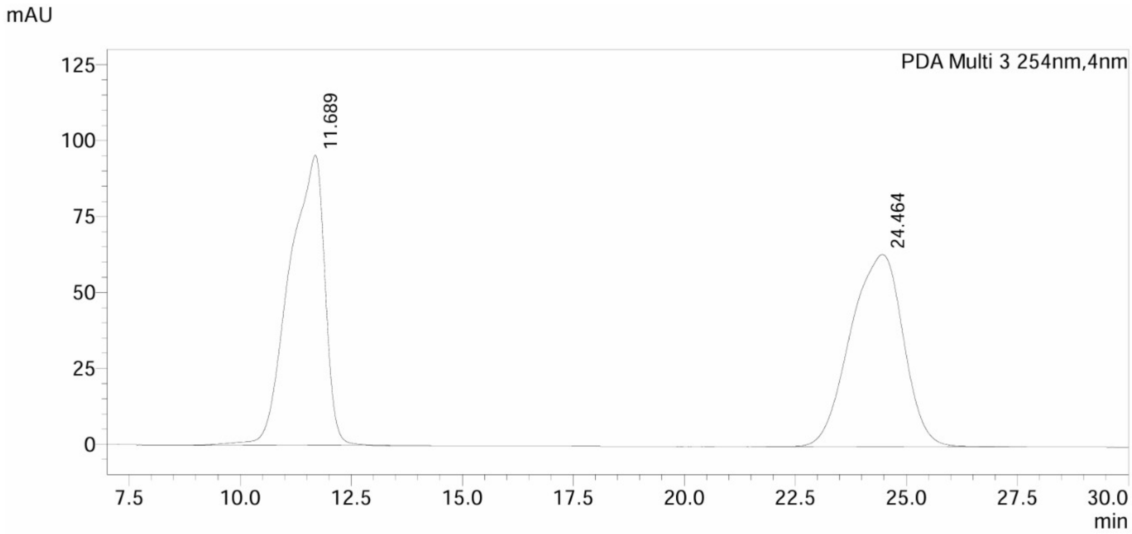

PDA Ch3 254nm

Peak#	Ret. Time	Area%
1	11.631	32.581
2	23.572	67.419
Total		100.000

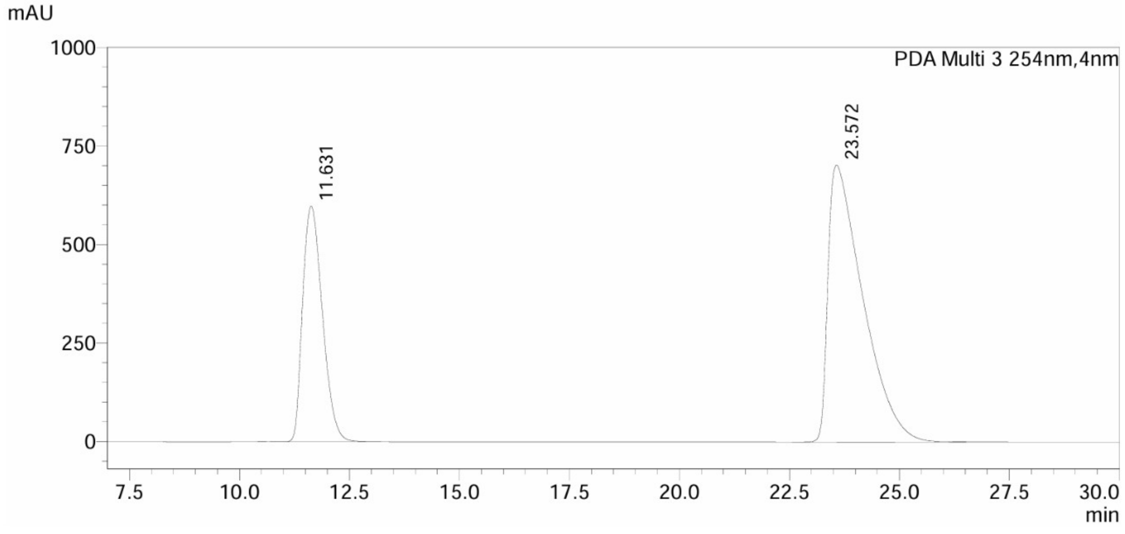

HPLC data for **48**: Chiralcel OD-H (70:30 *n*-hexane : IPA, flow rate 1.0 mLmin^{-1}, 211 nm, 30 °C) t_R (*S*) 13.4 min, t_R (*R*) 27.4 min, 78:22 er.

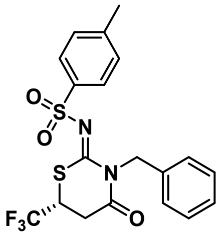

48

PDA Ch1 211nm

Peak#	Ret. Time	Area%
1	13.379	49.732
2	27.744	50.268
Total		100.000

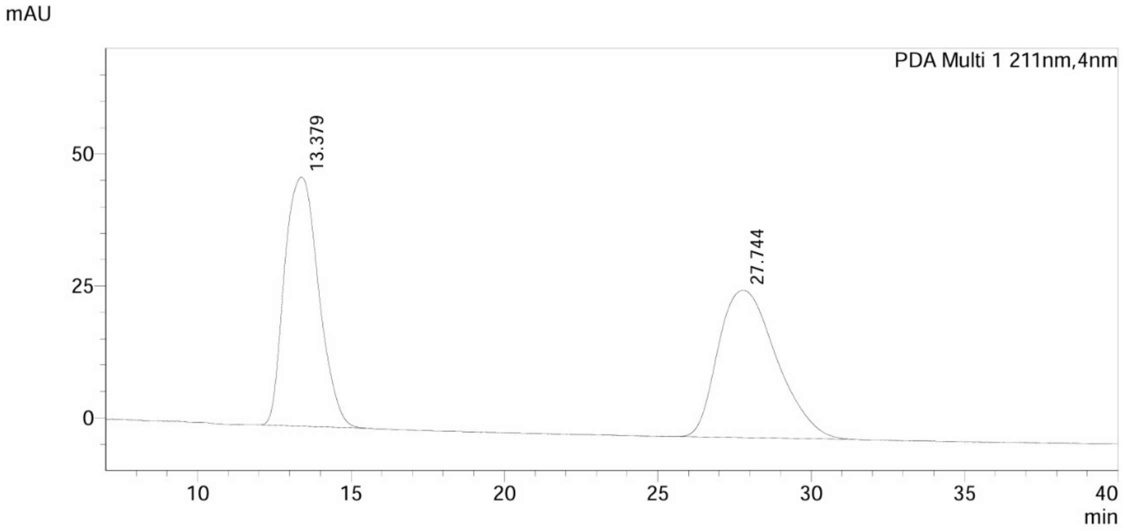

PDA Ch1 211nm

Peak#	Ret. Time	Area%
1	13.391	21.651
2	27.441	78.349
Total		100.000

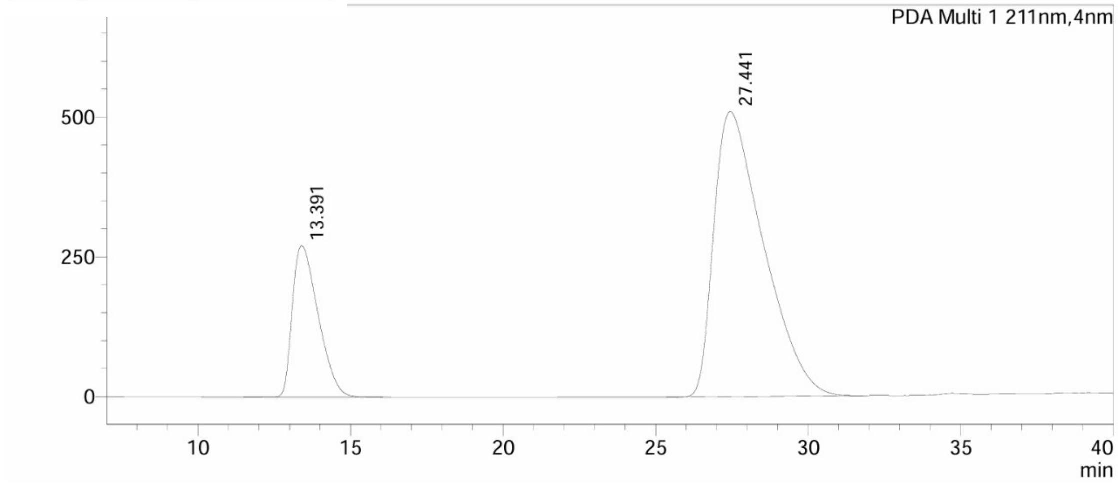

HPLC data for **49**: Chiralpak IA (80:20 *n*-hexane : IPA, flow rate 1.0 mLmin^{-1}, 211 nm, 30 °C) t_R (*S*) 13.7 min, t_R (*R*) 15.5 min, 91:9 er.

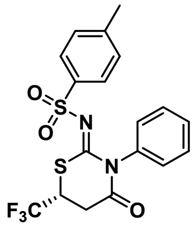

49

PDA Ch1 211nm

Peak#	Ret. Time	Area%
1	13.714	49.804
2	15.459	50.196
Total		100.000

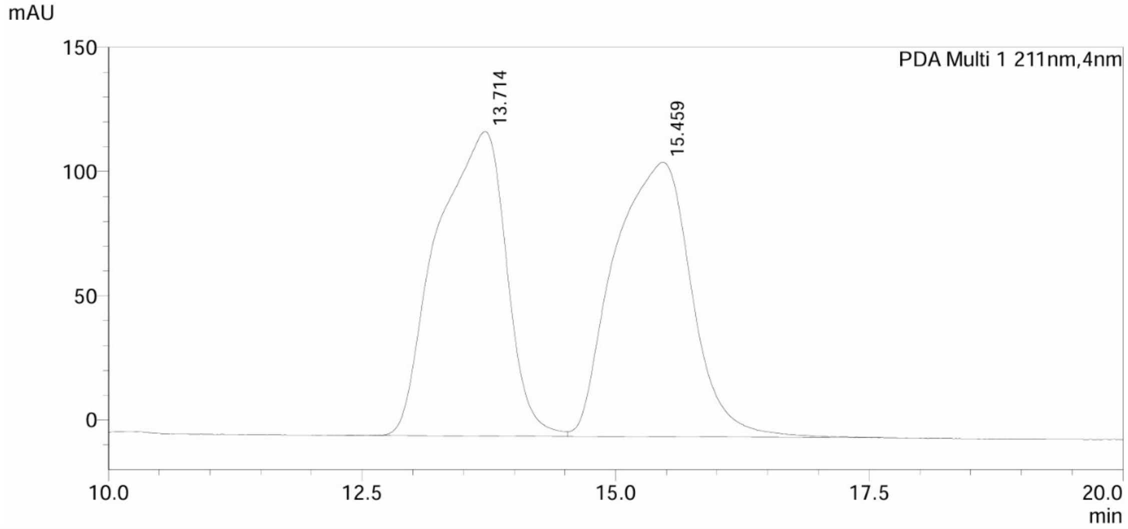

PDA Ch1 211nm

Peak#	Ret. Time	Area%
1	13.730	8.933
2	15.496	91.067
Total		100.000

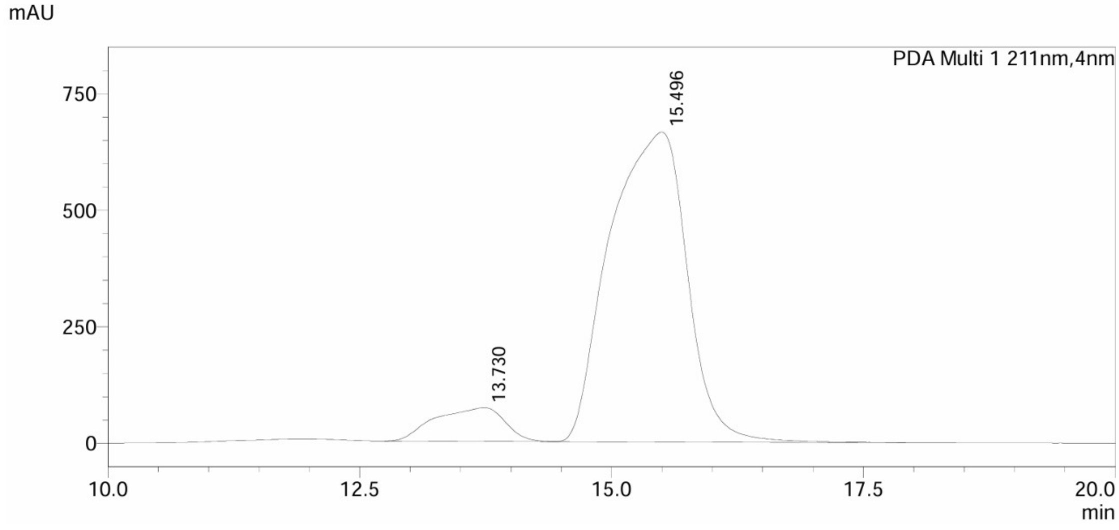

HPLC data for **50**: Chiralpak ID (93:7 *n*-hexane : IPA, flow rate 1.0 mLmin^{-1}, 254 nm, 40 °C) t_R (*S*) 22.9 min, t_R (*R*) 25.8 min, 88:12 er.

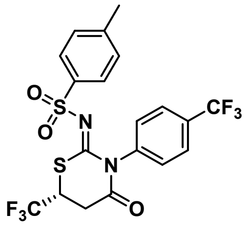

50

Detector A Channel 2 254nm

Peak#	Ret. Time	Area%
1	22.704	49.410
2	26.094	50.590
Total		100.000

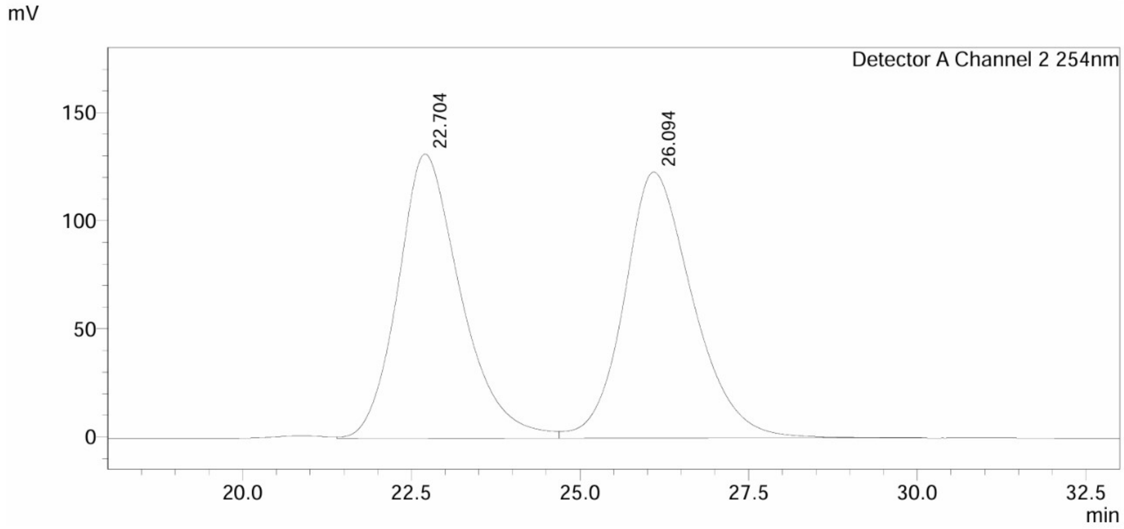

Detector A Channel 2 254nm

Peak#	Ret. Time	Area%
1	22.864	11.772
2	25.775	88.228
Total		100.000

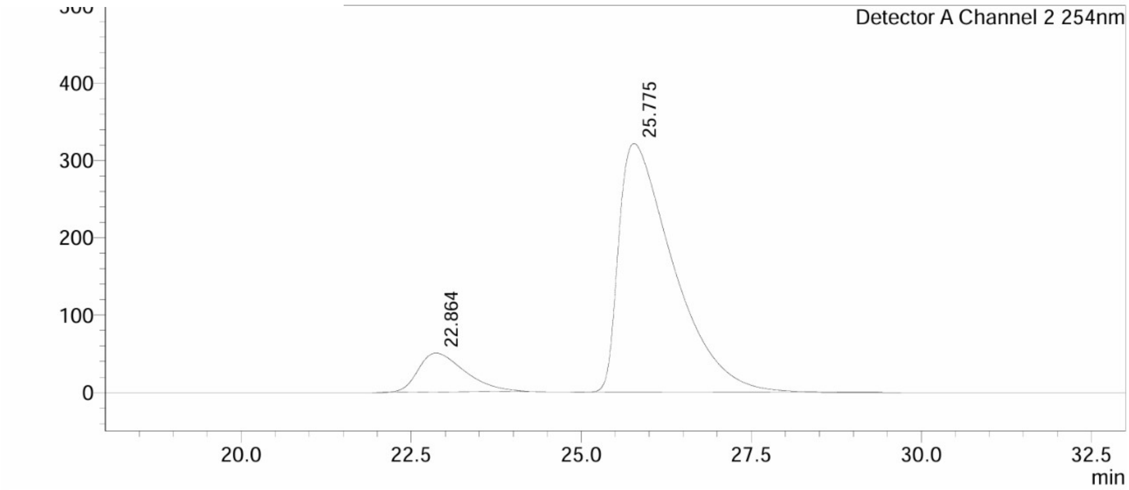

HPLC data for **51**: Chiralpak AD-H (80:20 *n*-hexane : IPA, flow rate 1.0 mLmin^{-1}, 254 nm, 30 °C) t_R (*S*) 18.0 min, t_R (*R*) 22.1 min, 90:10 er.

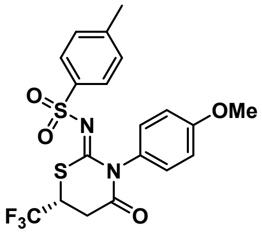

51

Detector A Channel 2 254nm

Peak#	Ret. Time	Area%
1	17.814	49.944
2	21.762	50.056
Total		100.000

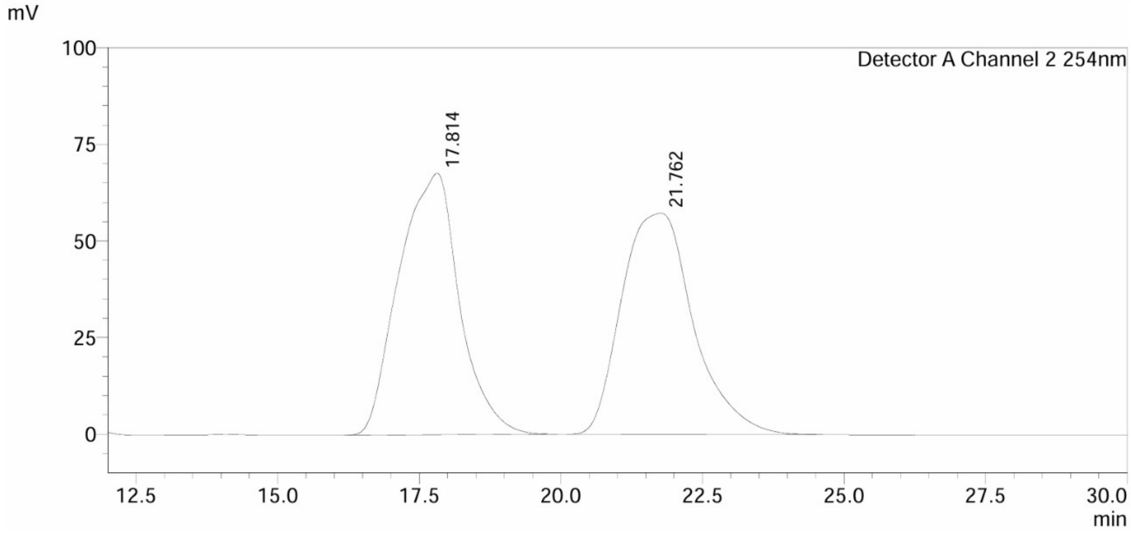

Detector A Channel 2 254nm

Peak#	Ret. Time	Area%
1	17.955	9.836
2	22.054	90.164
Total		100.000

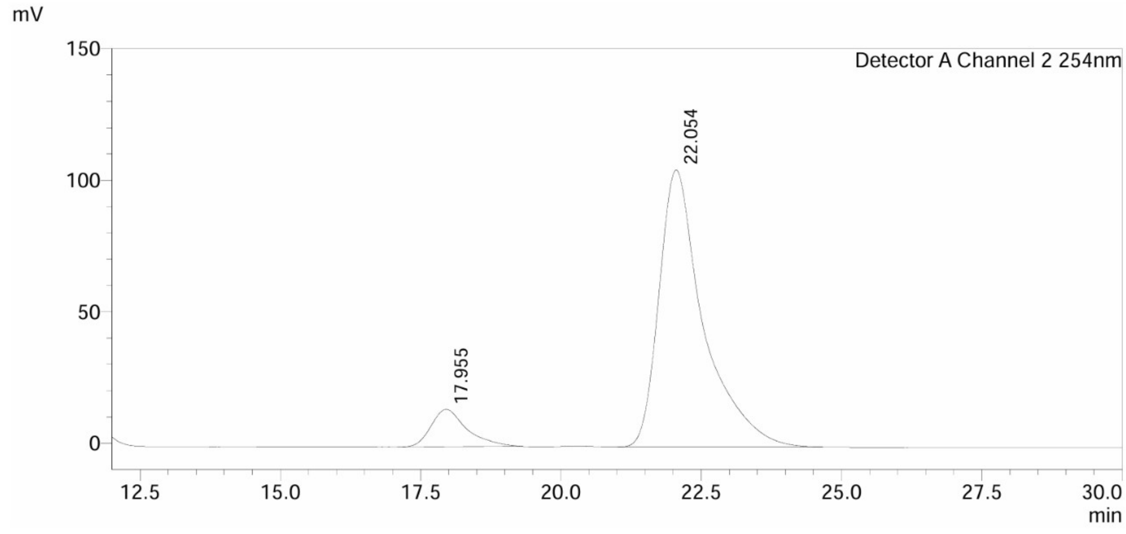

HPLC data for **52**: Chiralpak IA (80:20 *n*-hexane : IPA, flow rate 1.0 mLmin^{-1}, 211 nm, 30 °C) t_R (*S*) 17.2 min, t_R (*R*) 21.2 min, 91:9 er.

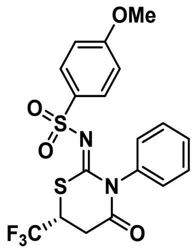

52

PDA Ch1 211nm

Peak#	Ret. Time	Area%
1	17.206	50.067
2	21.183	49.933
Total		100.000

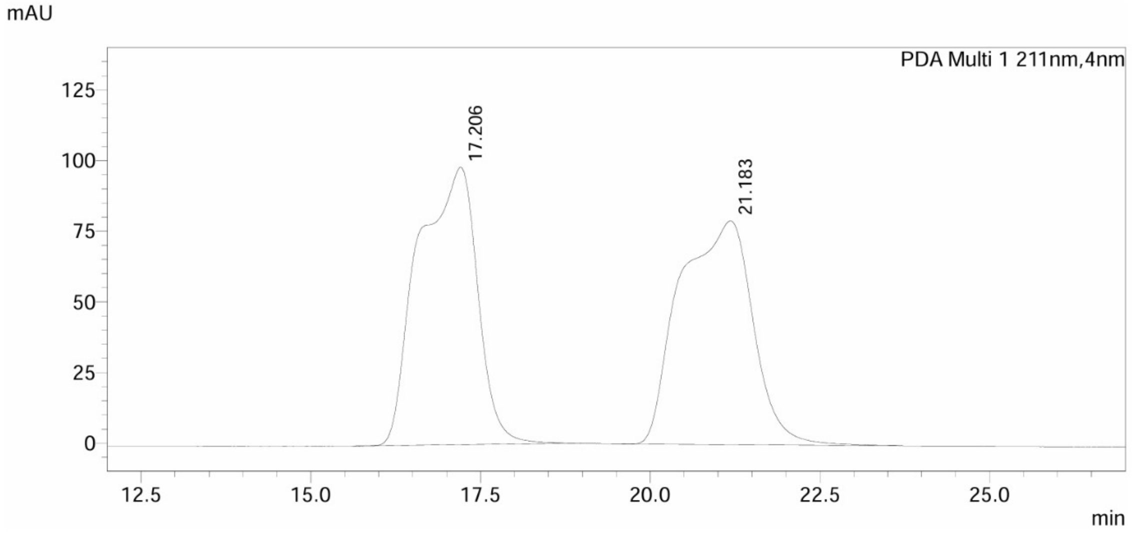

PDA Ch1 211nm

Peak#	Ret. Time	Area%
1	17.243	8.867
2	21.189	91.133
Total		100.000

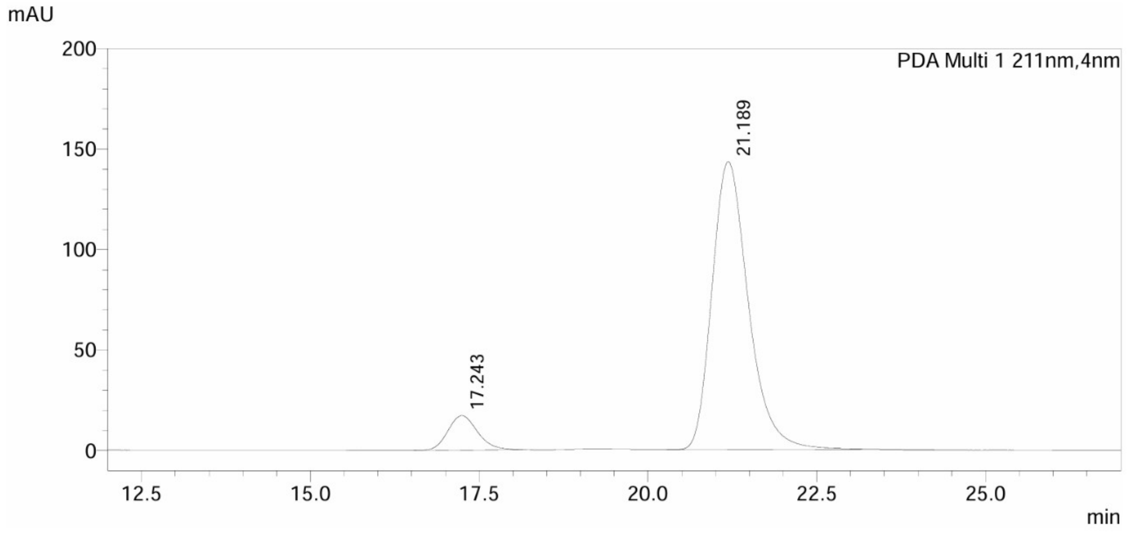

HPLC data for **53**: Chiralcel OD-H (90:10 *n*-hexane : IPA, flow rate 2.0 mLmin^{-1}, 211 nm, 40 °C) t_R (*S*) 7.0 min, t_R (*R*) 16.5 min, 96:4 er.

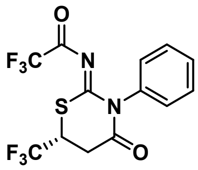

53

PDA Ch1 211nm

Peak#	Ret. Time	Area%
1	6.984	49.989
2	17.374	50.011
Total		100.000

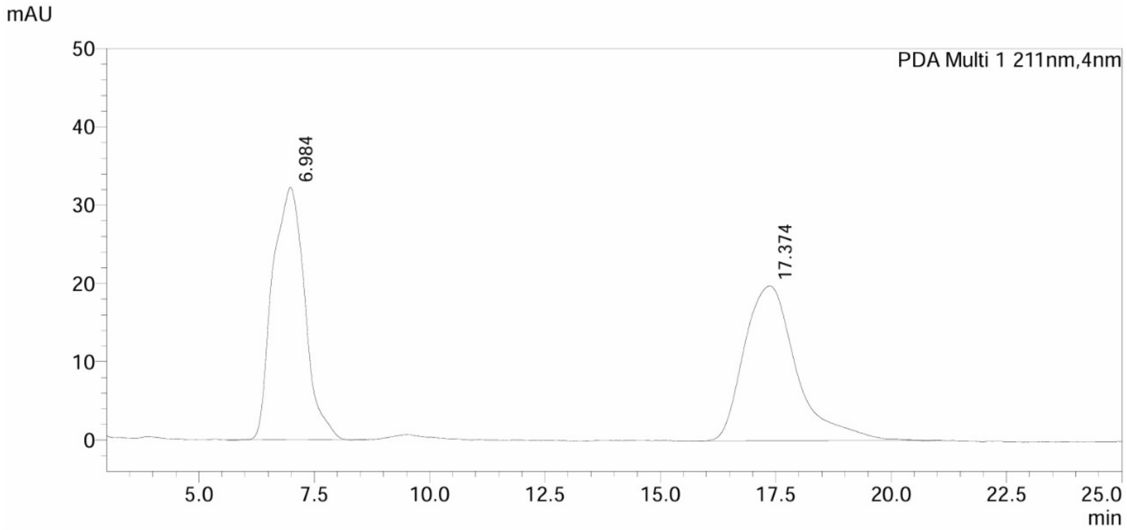

PDA Ch1 211nm

Peak#	Ret. Time	Area%
1	7.028	4.445
2	16.481	95.555
Total		100.000

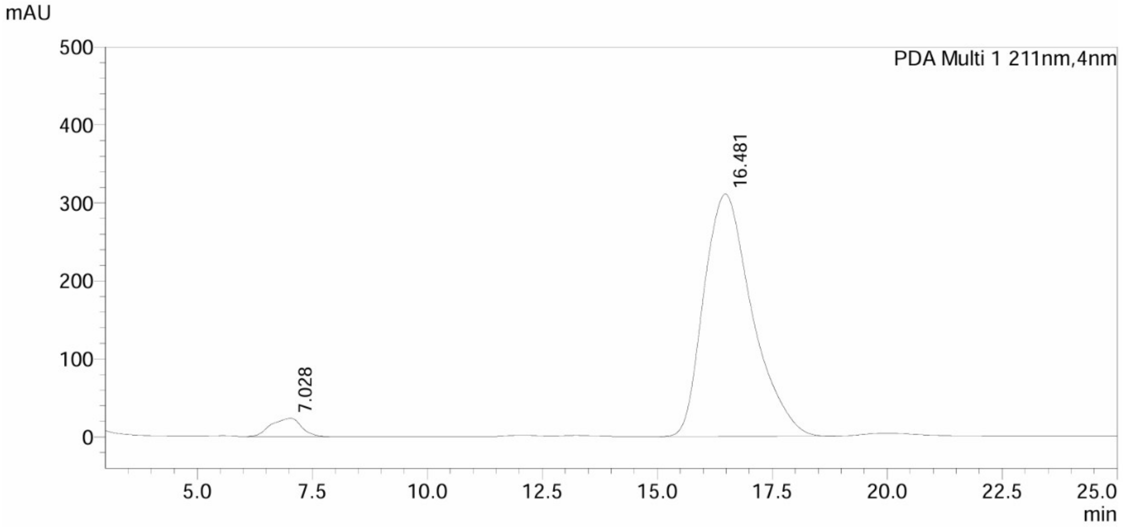

HPLC data for **54**: Chiralpak AD-H (90:10 *n*-hexane : IPA, flow rate 1.0 mLmin^{-1}, 211 nm, 30 °C) t_R (*R*) 14.0 min, t_R (*S*) 17.9 min, 98:2 er.

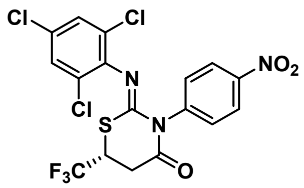

54

PDA Ch1 211nm

Peak#	Ret. Time	Area%
1	14.007	49.856
2	17.848	50.144
Total		100.000

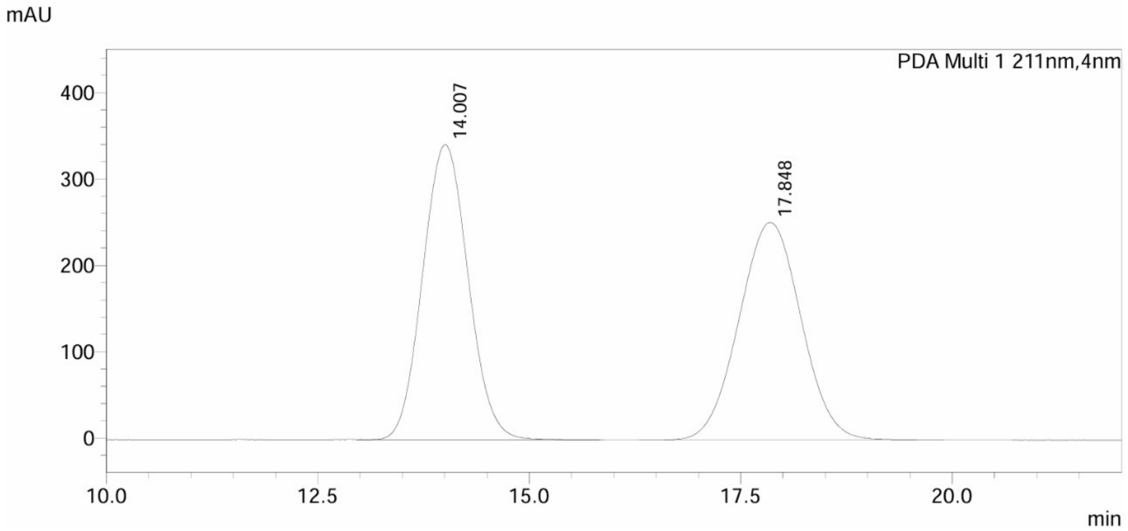

PDA Ch1 211nm

Peak#	Ret. Time	Area%
1	14.036	97.958
2	17.891	2.042
Total		100.000

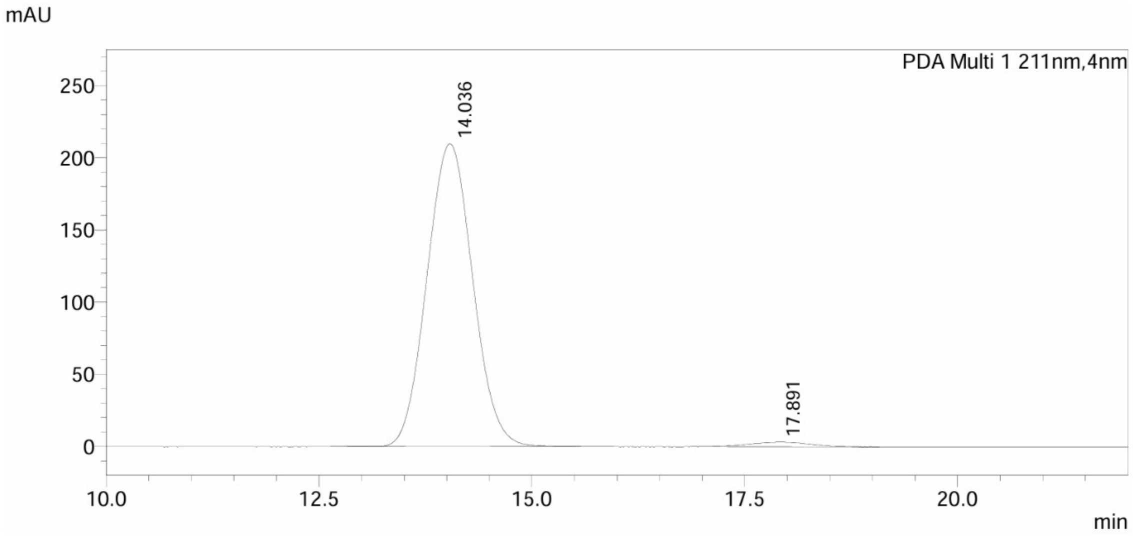

HPLC data for **55**: Chiralpak IA (80:20 *n*-hexane : IPA, flow rate 1.0 mLmin^{-1}, 254 nm, 30 °C) t_R (*S*) 8.0 min, t_R (*R*) 10.2 min, 99:1 er.

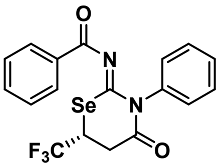

55

PDA Ch3 254nm

Peak#	Ret. Time	Area%
1	7.927	50.086
2	10.019	49.914
Total		100.000

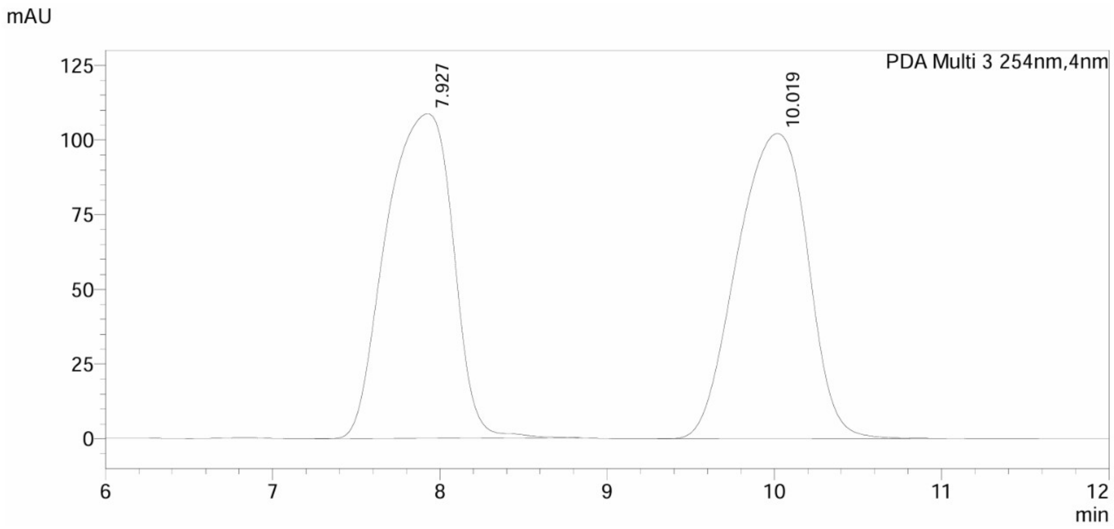

PDA Ch3 254nm

Peak#	Ret. Time	Area%
1	8.003	1.444
2	10.161	98.556
Total		100.000

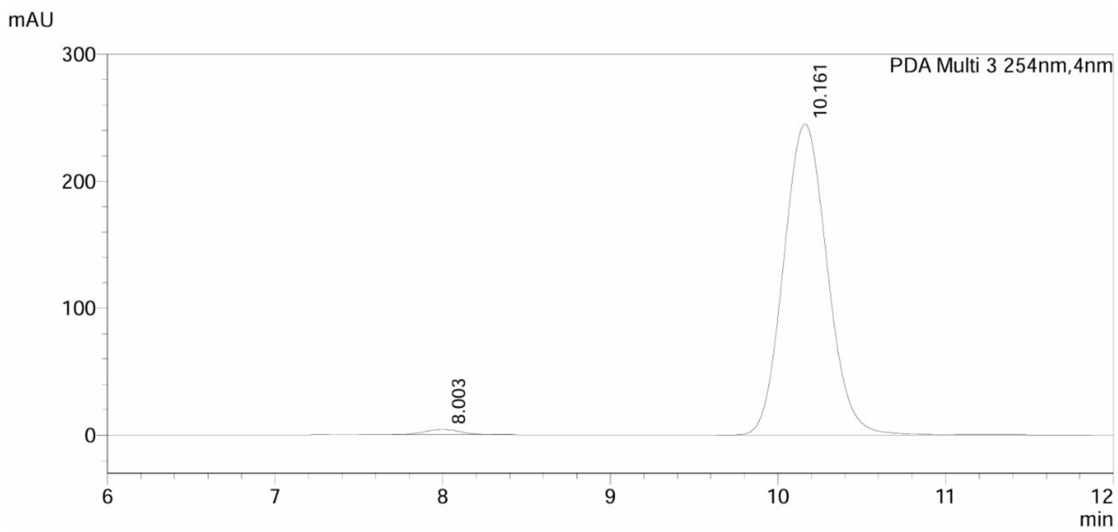

HPLC data for **56**: Chiralpak AD-H (60:40 *n*-hexane : IPA, flow rate 1.0 mLmin^{-1}, 254 nm, 30 °C) t_R (*S*) 8.8 min, t_R (*R*) 12.0 min, 98:2 er.

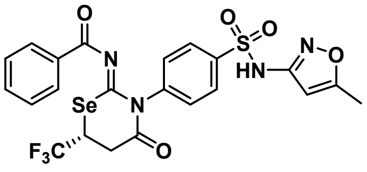

56

PDA Ch3 254nm

Peak#	Ret. Time	Area%
1	8.795	50.040
2	11.861	49.960
Total		100.000

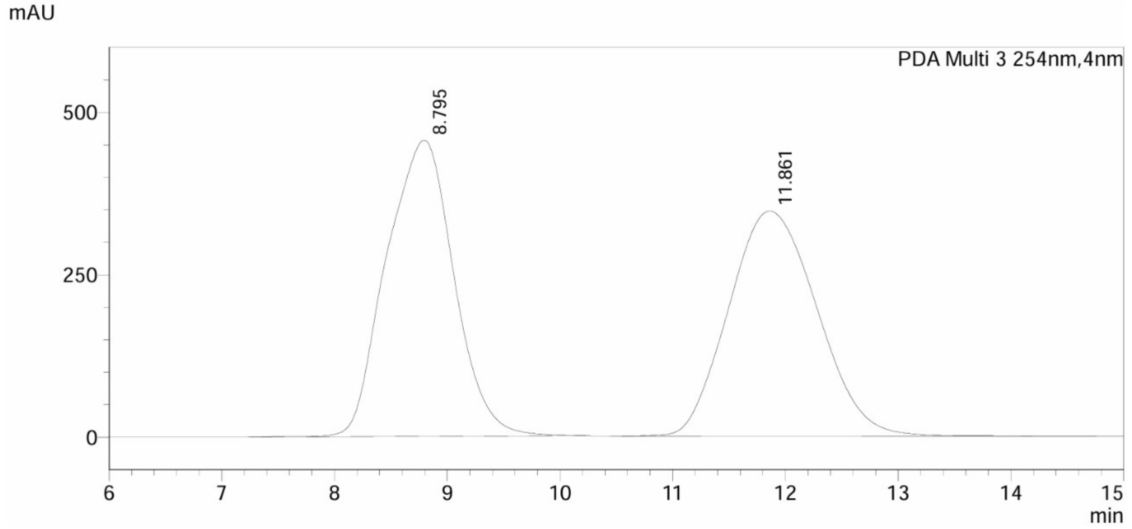

PDA Ch3 254nm

Peak#	Ret. Time	Area%
1	8.765	2.294
2	12.029	97.706
Total		100.000

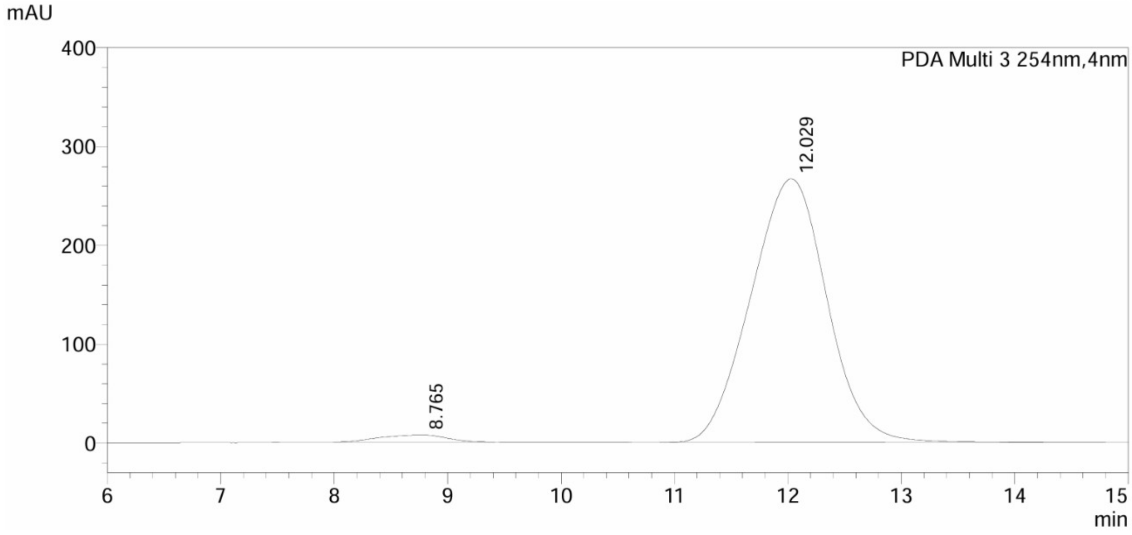

HPLC data for **57**: Chiralpak IA (80:20 *n*-hexane : IPA, flow rate 1.0 mLmin^{-1}, 220 nm, 30 °C) t_R (*S*) 10.8 min, t_R (*R*) 12.1 min, 98:2 er.

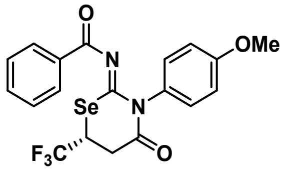

57

PDA Ch2 220nm

Peak#	Ret. Time	Area%
1	10.851	49.946
2	12.182	50.054
Total		100.000

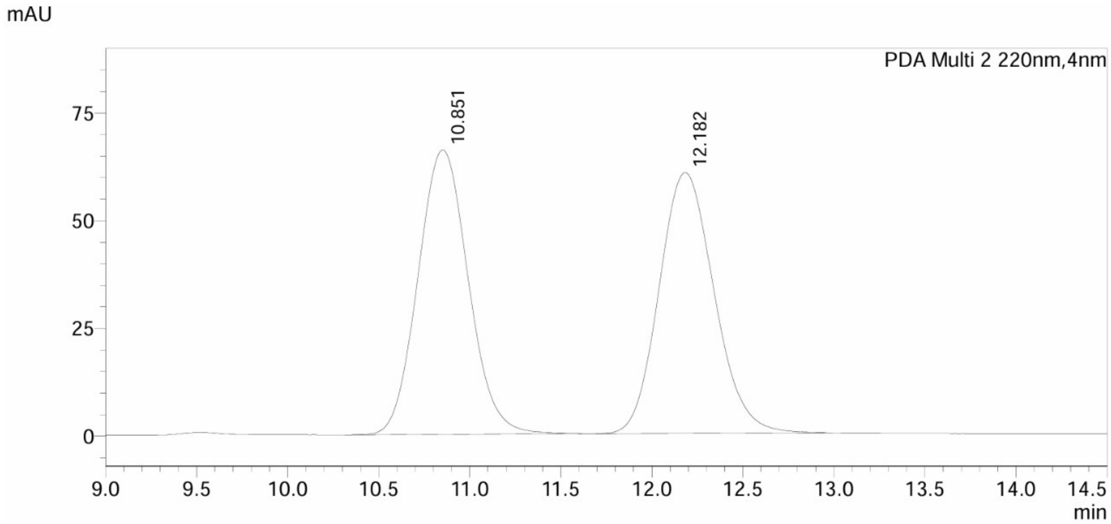

PDA Ch2 220nm

Peak#	Ret. Time	Area%
1	10.849	2.067
2	12.066	97.933
Total		100.000

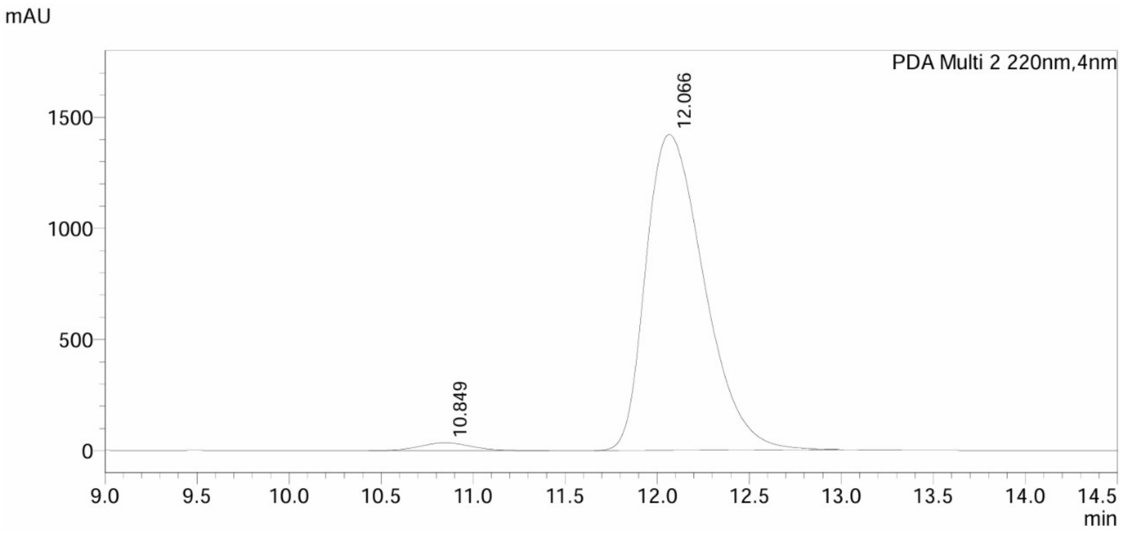

HPLC data for **58**: Chiralpak IB (90:10 *n*-hexane : IPA, flow rate 1.0 mLmin^{-1}, 211 nm, 30 °C) t_R (*S*) 30.6 min, t_R (*R*) 37.3 min, 99:1 er.

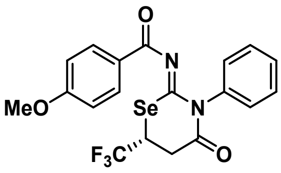

58

PDA Ch1 211nm

Peak#	Ret. Time	Area%
1	30.516	49.630
2	39.448	50.370
Total		100.000

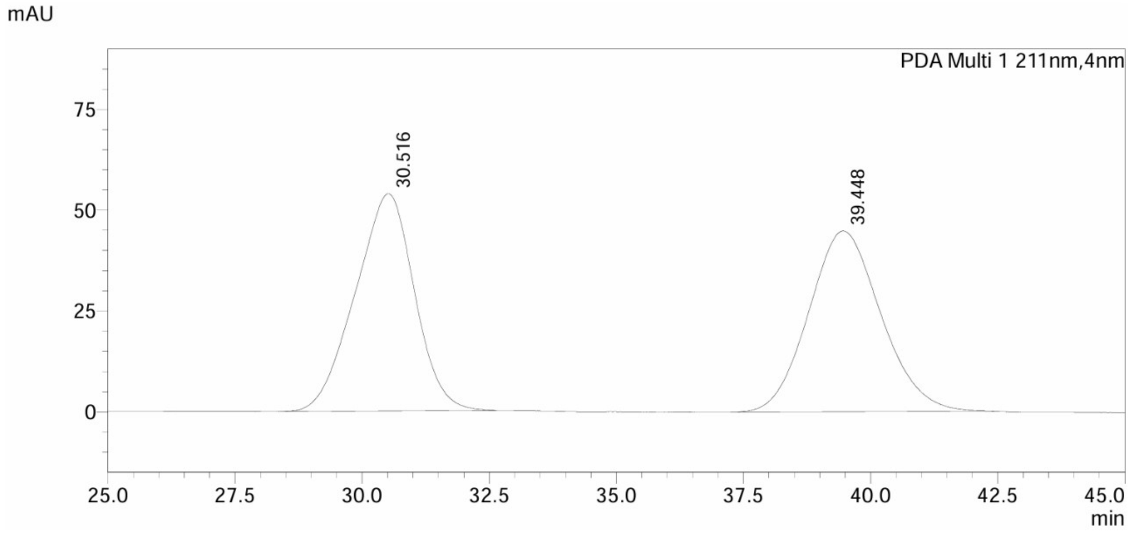

PDA Ch1 211nm

Peak#	Ret. Time	Area%
1	30.576	1.387
2	37.315	98.613
Total		100.000

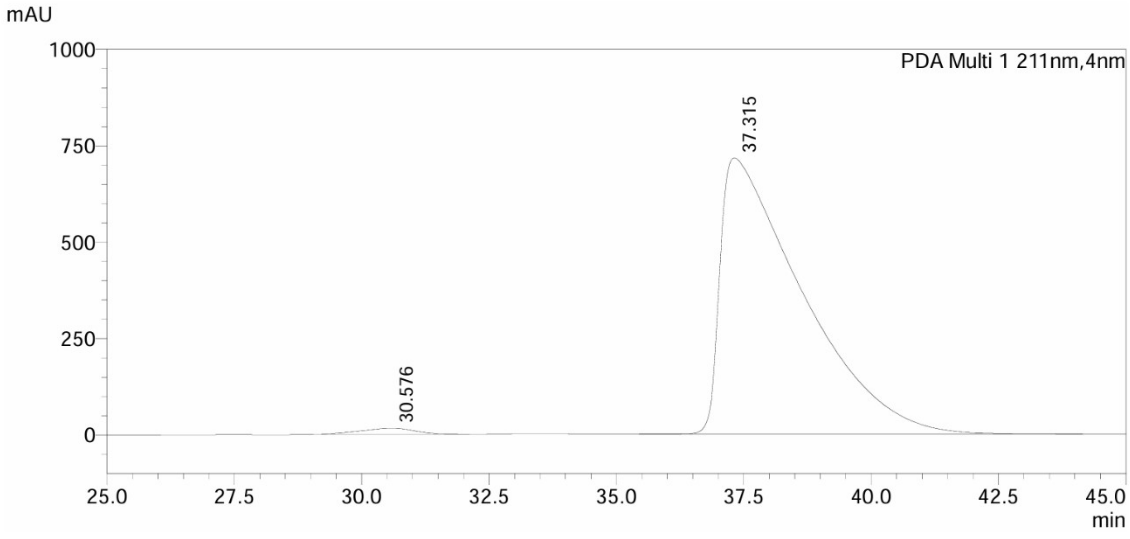

HPLC data for **59**: Chiralpak IA (80:20 *n*-hexane : IPA, flow rate 1.0 mLmin^{-1}, 254 nm, 30 °C) t_R (*S*) 11.1 min, t_R (*R*) 12.5 min, 98:2 er.

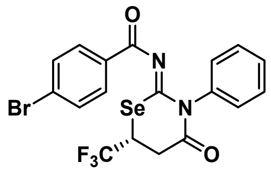

59

PDA Ch3 254nm

Peak#	Ret. Time	Area%
1	11.068	50.067
2	12.628	49.933
Total		100.000

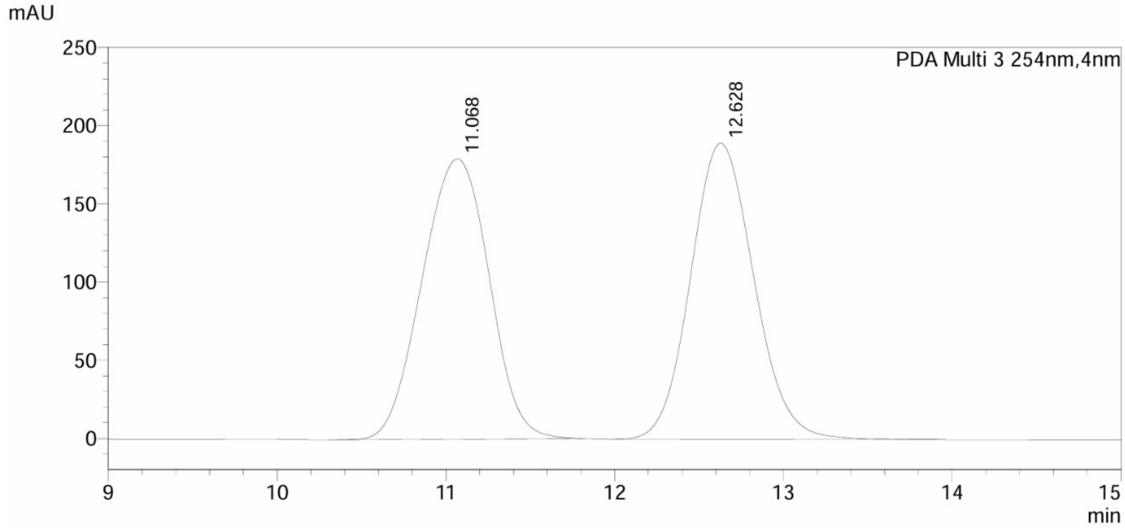

PDA Ch3 254nm

Peak#	Ret. Time	Area%
1	11.061	2.230
2	12.503	97.770
Total		100.000

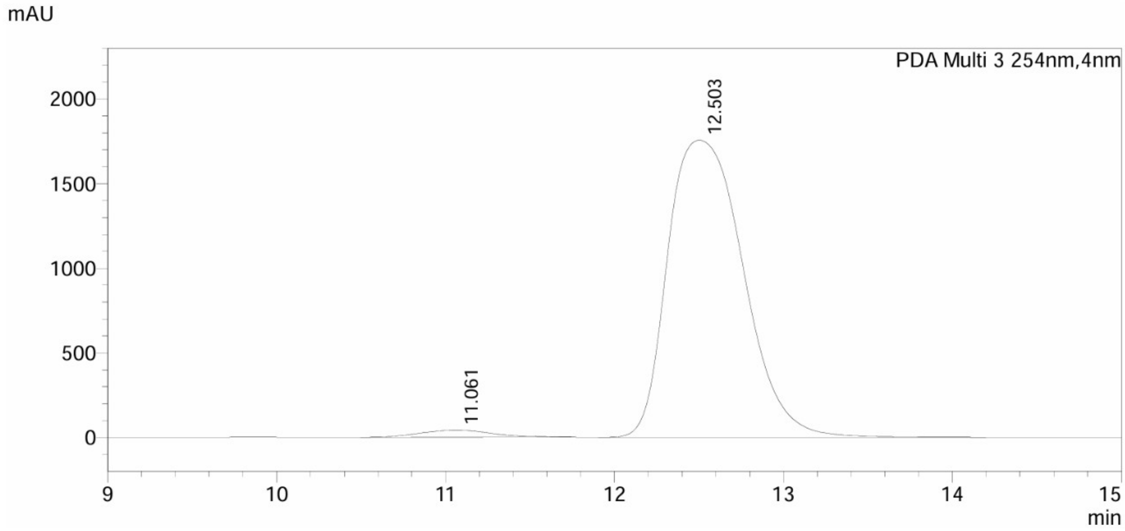

HPLC data for **60**: Chiralpak IA (80:20 *n*-hexane : IPA, flow rate 1.0 mLmin^{-1}, 211 nm, 30 °C) t_R (*S*) 13.8 min, t_R (*R*) 15.8 min, 97:3 er.

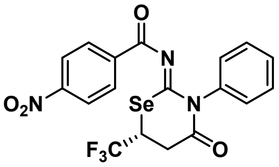

60

PDA Ch1 211nm

Peak#	Ret. Time	Area%
1	13.662	50.008
2	15.963	49.992
Total		100.000

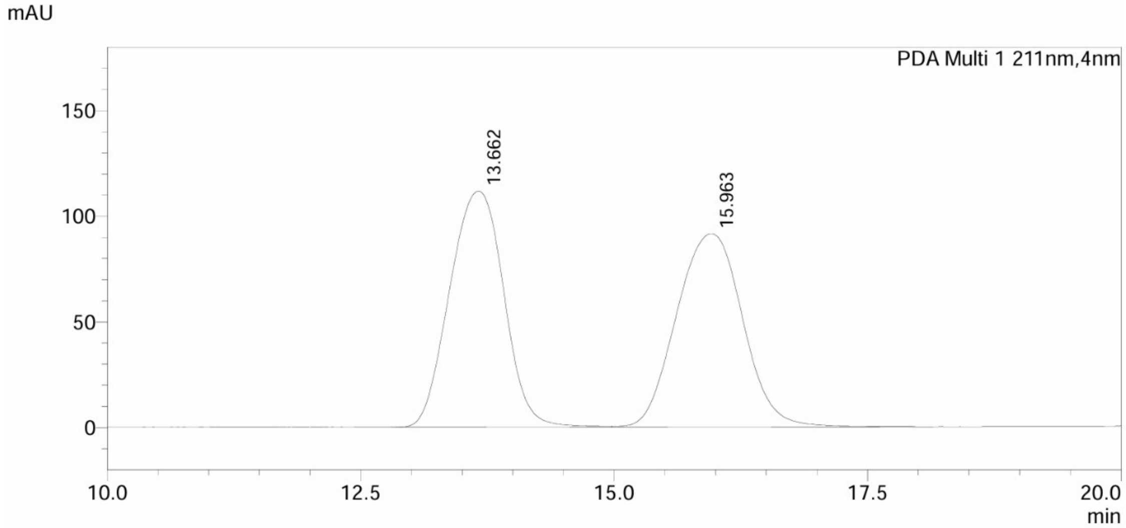

PDA Ch1 211nm

Peak#	Ret. Time	Area%
1	13.761	2.623
2	15.843	97.377
Total		100.000

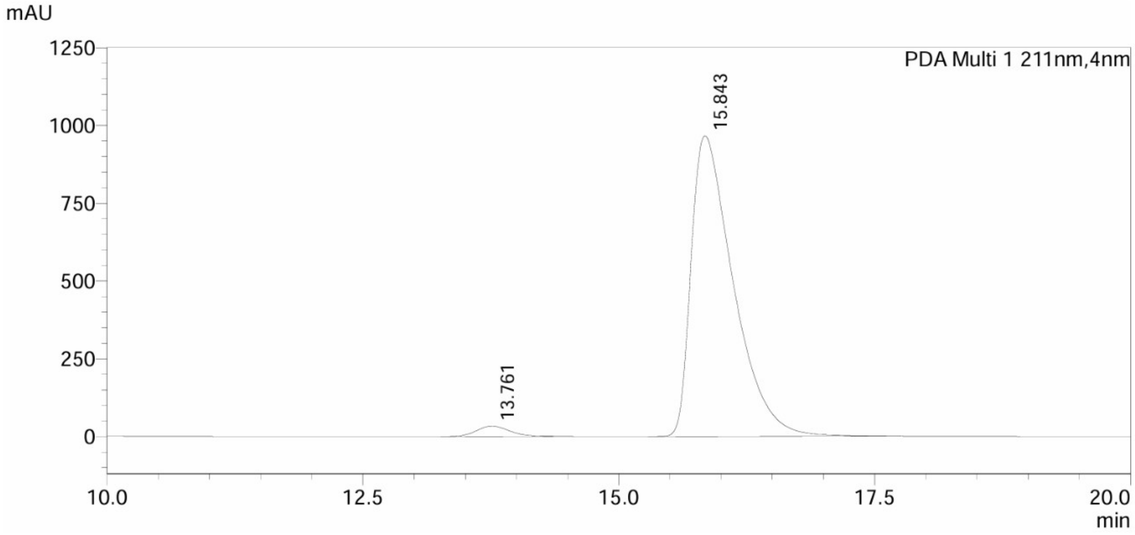

HPLC data for **61**: Chiralpak IA (80:20 *n*-hexane : IPA, flow rate 1.0 mLmin^{-1}, 211 nm, 30 °C) t_R (*S*) 12.1 min, t_R (*R*) 18.8 min, 93:7 er.

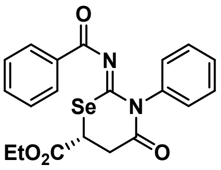

61

PDA Ch1 211nm

Peak#	Ret. Time	Area%
1	12.134	49.736
2	19.056	50.264
Total		100.000

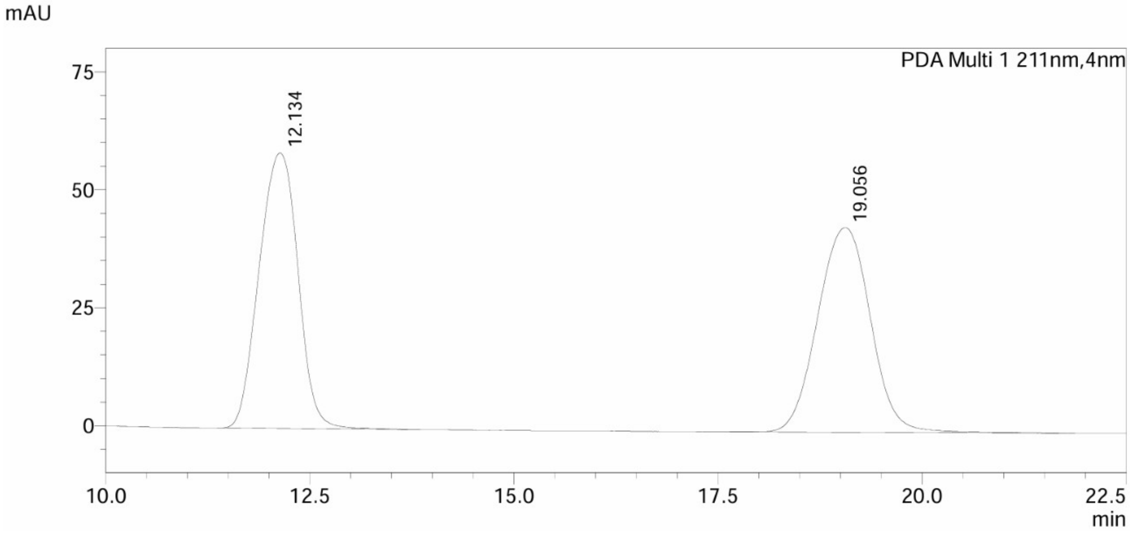

PDA Ch1 211nm

Peak#	Ret. Time	Area%
1	12.149	7.392
2	18.811	92.608
Total		100.000

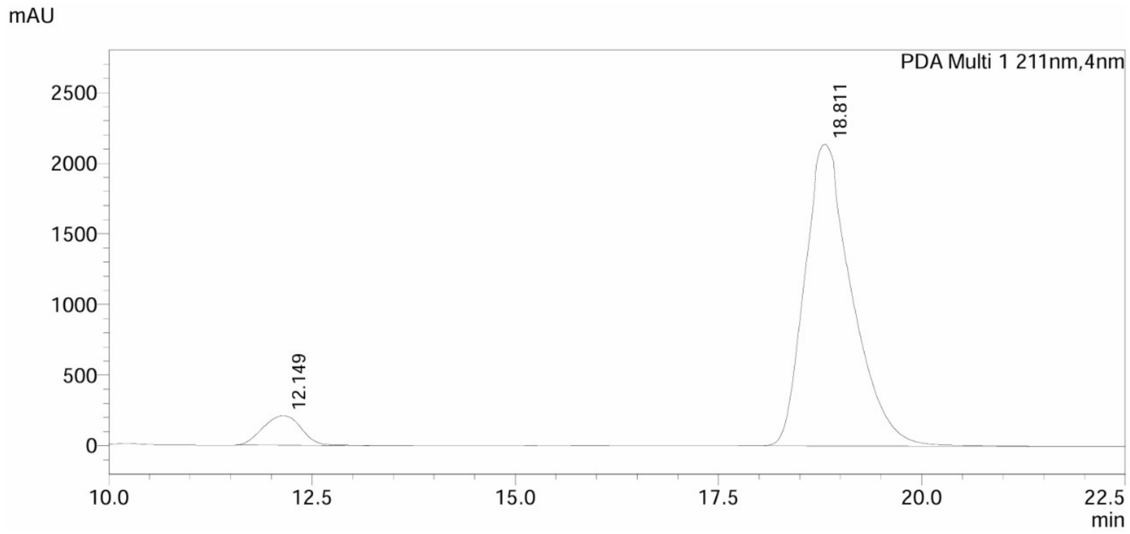

HPLC data for **62**: Chiralpak IA (90:10 *n*-hexane : IPA, flow rate 1.0 mLmin^{-1}, 211 nm, 30 °C) t_R (*R*) 16.0 min, t_R (*S*) 17.5 min, 87:13 er.

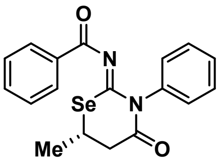

62

PDA Ch1 211nm

Peak#	Ret. Time	Area%
1	16.072	49.702
2	17.590	50.298
Total		100.000

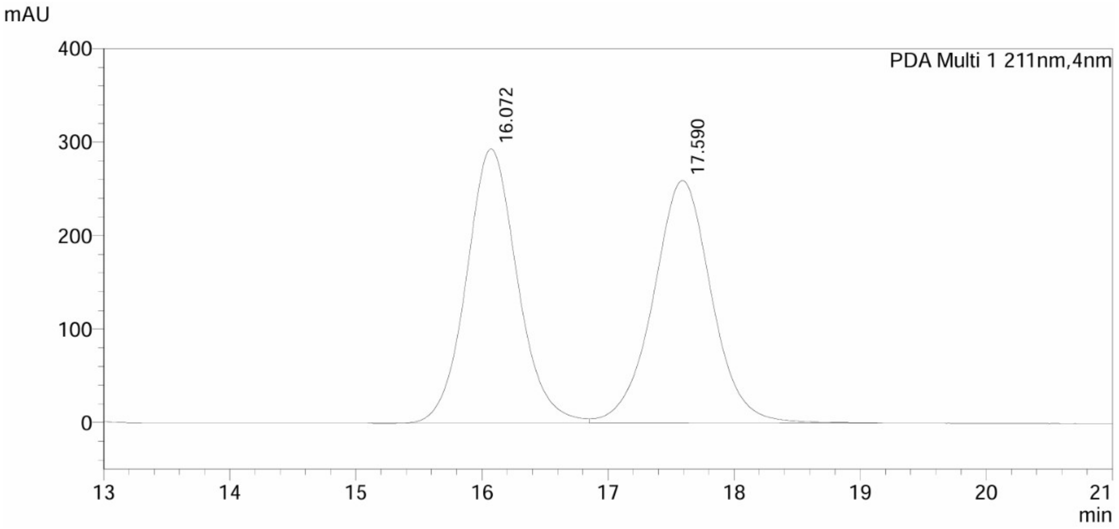

PDA Ch1 211nm

Peak#	Ret. Time	Area%
1	16.009	12.834
2	17.489	87.166
Total		100.000

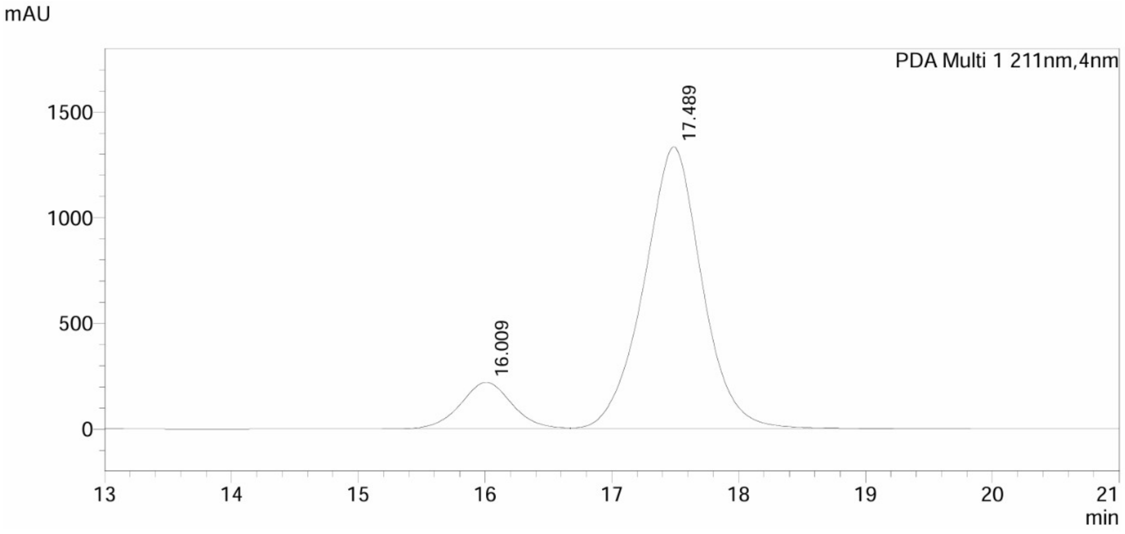

HPLC data for **63**: Chiralpak IB (80:20 *n*-hexane : IPA, flow rate 1.0 mLmin^{-1}, 211 nm, 30 °C) t_R (R_a) 26.0 min, t_R (S_a) 31.5 min, 87:13 er.

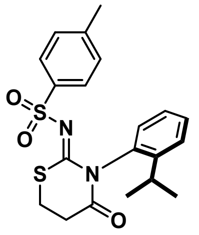

63

PDA Ch1 211nm

Peak#	Ret. Time	Area%
1	26.531	49.460
2	31.615	50.540
Total		100.000

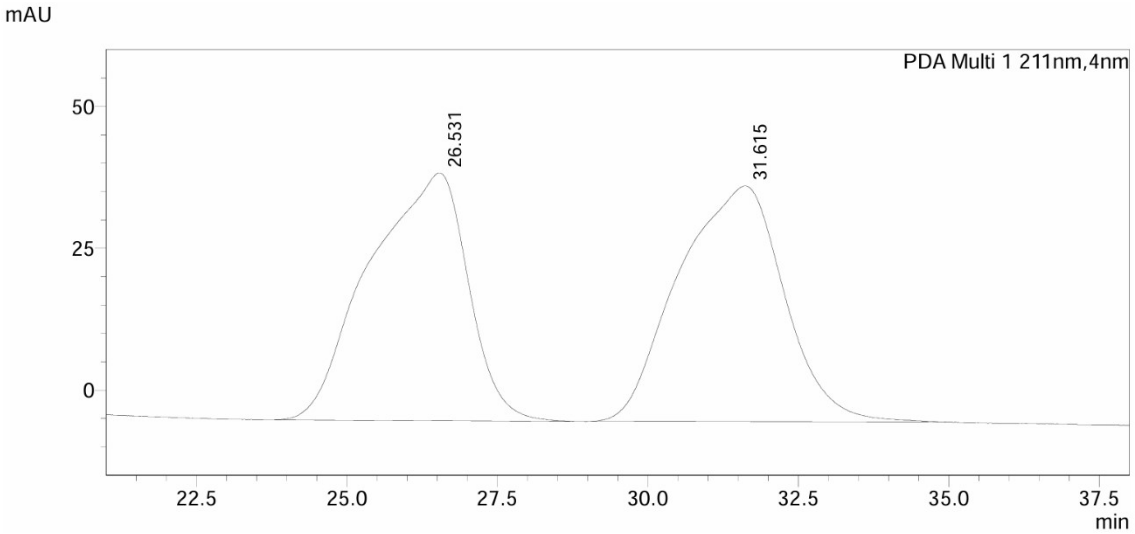

PDA Ch1 211nm

Peak#	Ret. Time	Area%
1	26.022	86.808
2	31.541	13.192
Total		100.000

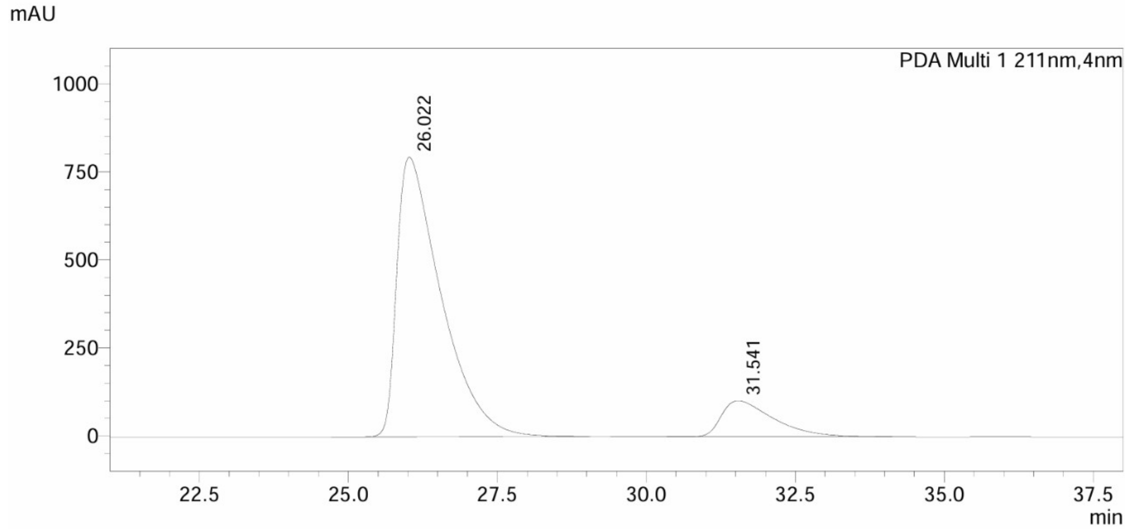

HPLC data for **64**: Chiralpak IB (80:20 *n*-hexane : IPA, flow rate 1.0 mLmin^{-1}, 211 nm, 30 °C) t_R (R_a) 31.9 min, t_R (S_a) 41.2 min, 73:27 er.

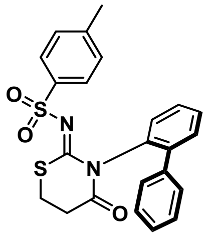

64

PDA Ch1 211nm

Peak#	Ret. Time	Area%
1	32.311	50.154
2	41.076	49.846
Total		100.000

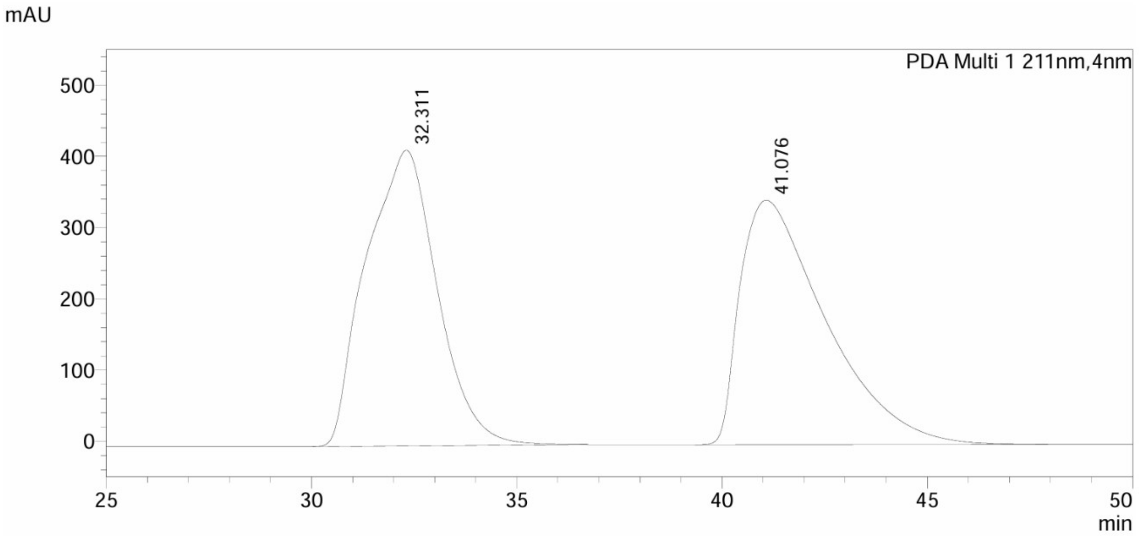

PDA Ch1 211nm

Peak#	Ret. Time	Area%
1	31.901	72.800
2	41.243	27.200
Total		100.000

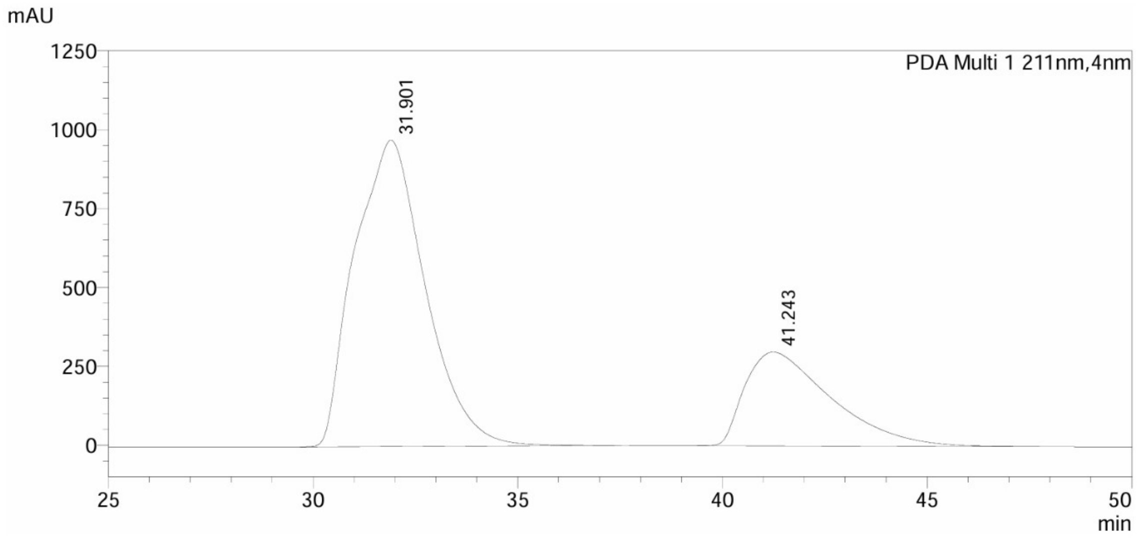

HPLC data for **65**: Chiralpak IB (60:40 *n*-hexane : IPA, flow rate 1.0 mLmin^{-1}, 211 nm, 30 °C) t_R (R_a) 12.1 min, t_R (S_a) 15.2 min, 80:20 er.

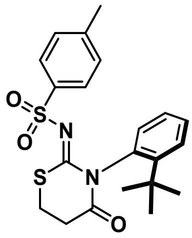

65

PDA Ch1 211nm

Peak#	Ret. Time	Area%
1	12.195	49.485
2	15.196	50.515
Total		100.000

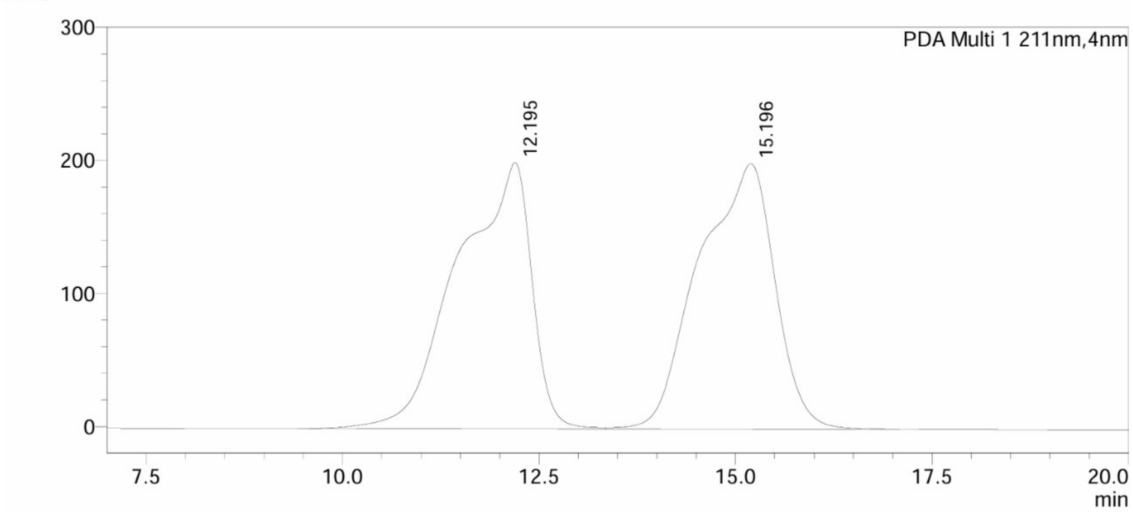

PDA Ch1 211nm

Peak#	Ret. Time	Area%
1	12.104	79.785
2	15.170	20.215
Total		100.000

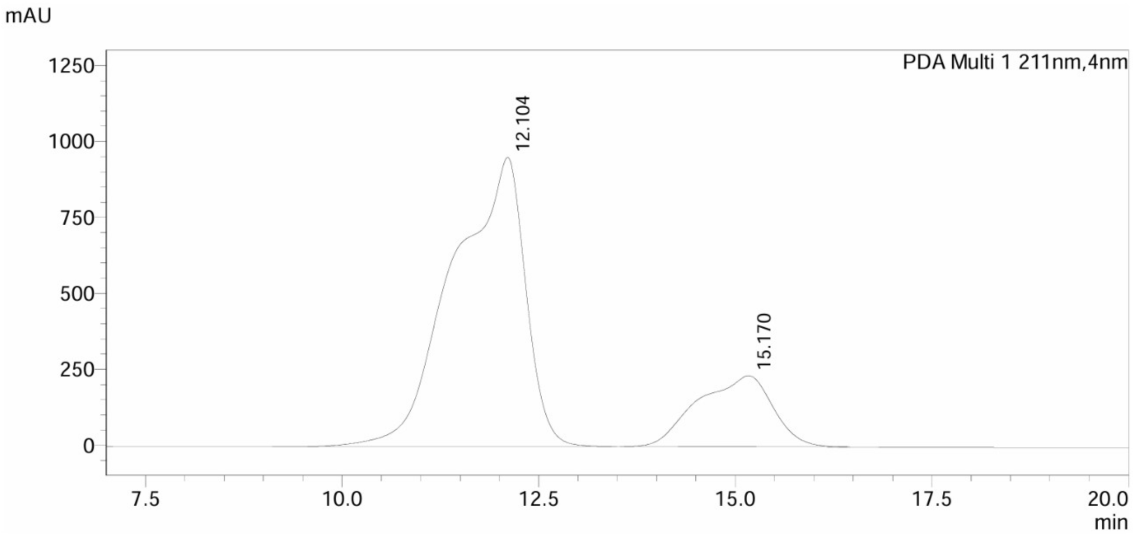

HPLC data for **66**: Chiralpak IB (80:20 *n*-hexane : IPA, flow rate 1.0 mLmin^{-1}, 254 nm, 30 °C) t_R (R_a) 8.3 min, t_R (S_a) 9.7 min, 77:23 er.

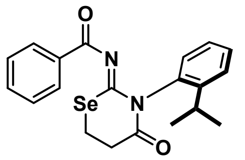

66

PDA Ch3 254nm

Peak#	Ret. Time	Area%
1	8.272	49.690
2	9.736	50.310
Total		100.000

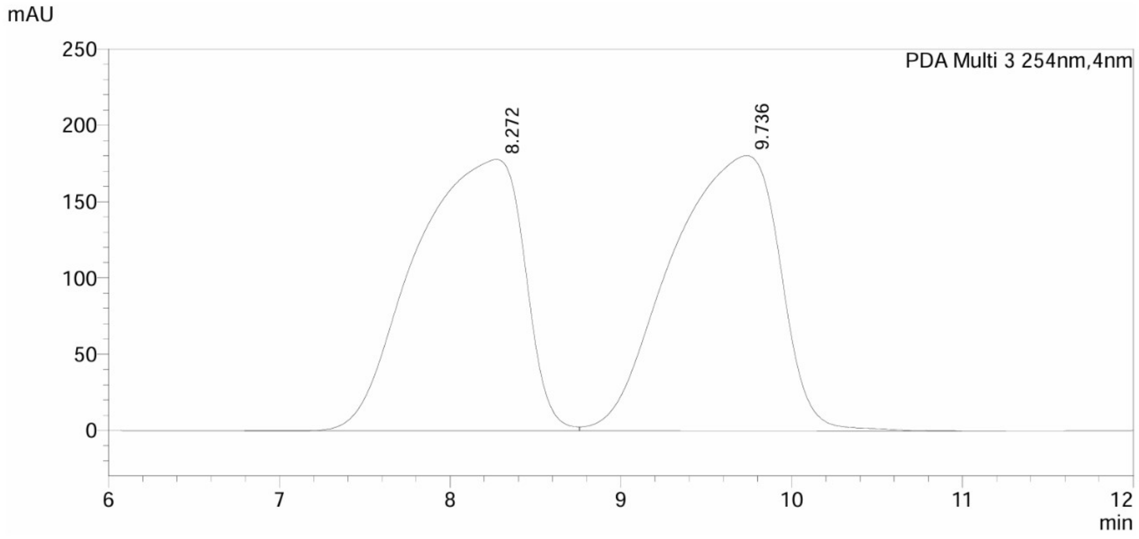

PDA Ch3 254nm

Peak#	Ret. Time	Area%
1	8.252	77.475
2	9.735	22.525
Total		100.000

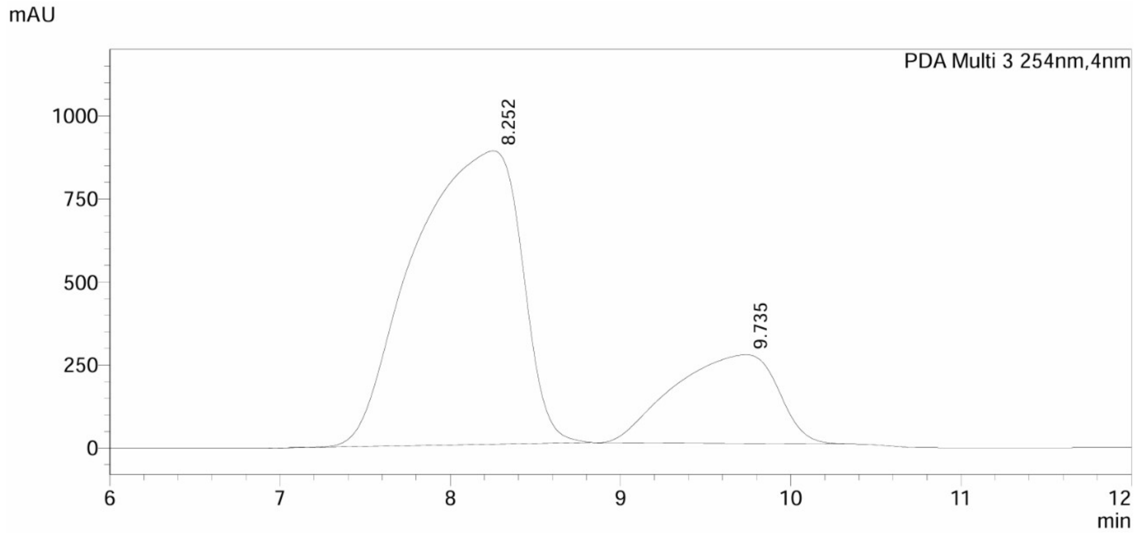

HPLC data for (R,R_a,Z)-**67**: Chiralpak AD-H (80:20 n-hexane : IPA, flow rate 1.0 mLmin^{-1}, 254 nm, 30 °C) t_R (R,R_a) 7.8 min, t_R (S,S_a) 17.5 min, 98:2 er.

HPLC data for (S,R_a,Z)-**S41**: er not determined.

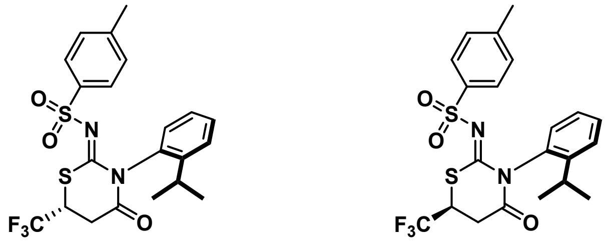

67 **S41**

Detector A Channel 2 254nm

Peak#	Ret. Time	Area%
1	7.835	49.958
2	17.492	50.042
Total		100.000

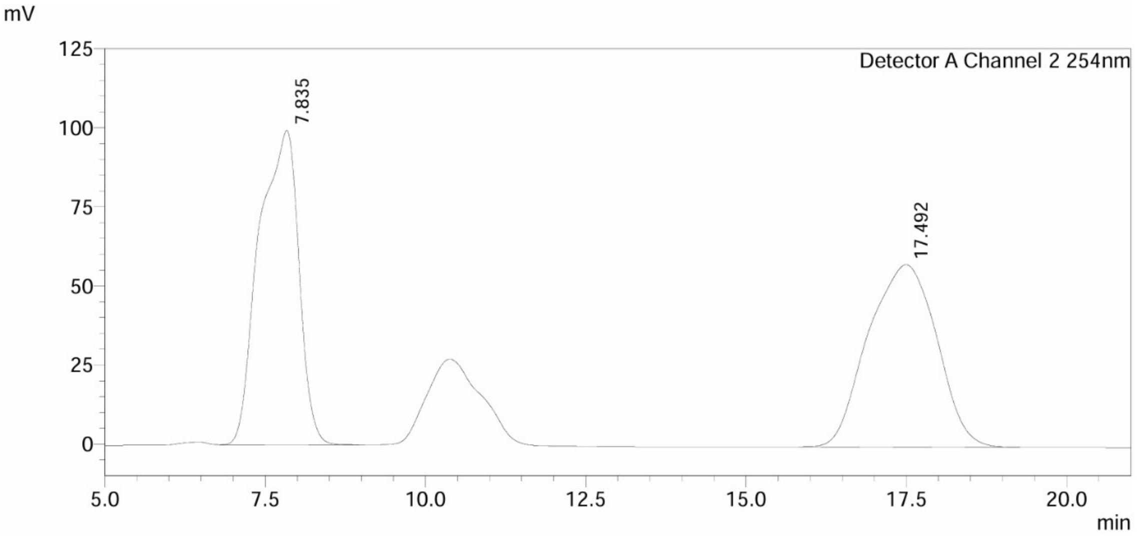

Detector A Channel 2 254nm

Peak#	Ret. Time	Area%
1	7.805	97.681
2	17.460	2.319
Total		100.000

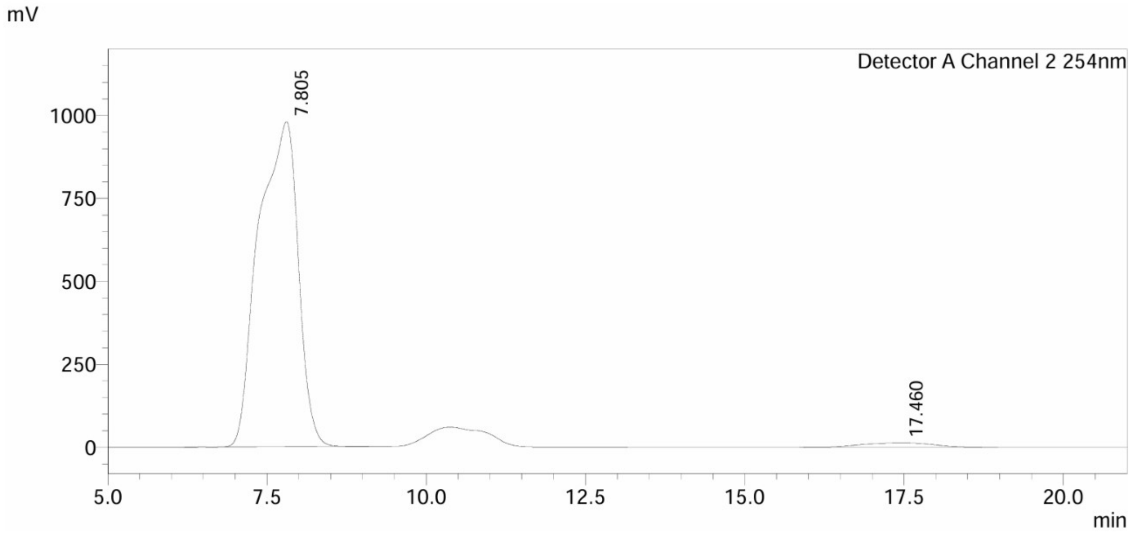

HPLC data for (R,R_a,Z)-**68**: Chiralcel OD-H (70:30 *n*-hexane : IPA, flow rate 1.0 mLmin^{-1}, 211 nm, 30 °C) t_R (S,S_a) 7.8 min, t_R (R,R_a) 18.5 min, 95:5 er.

HPLC data for (R,S_a,Z)-**S42**: Chiralcel OD-H (70:30 *n*-hexane : IPA, flow rate 1.0 mLmin^{-1}, 211 nm, 30 °C) t_R (S,R_a) 14.0 min, t_R (R,S_a) 33.2 min, 63:37 er.

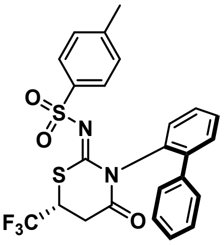

68

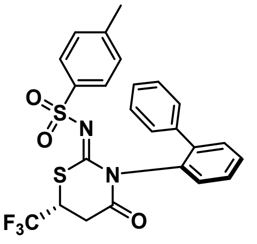

S42

PDA Ch1 211nm

Peak#	Ret. Time	Area%
1	7.807	32.812
2	13.996	16.845
3	19.274	33.073
4	33.525	17.270
Total		100.000

PDA Ch1 211nm

Peak#	Ret. Time	Area%
1	7.821	4.124
2	13.990	4.882
3	18.518	82.680
4	33.217	8.314
Total		100.000

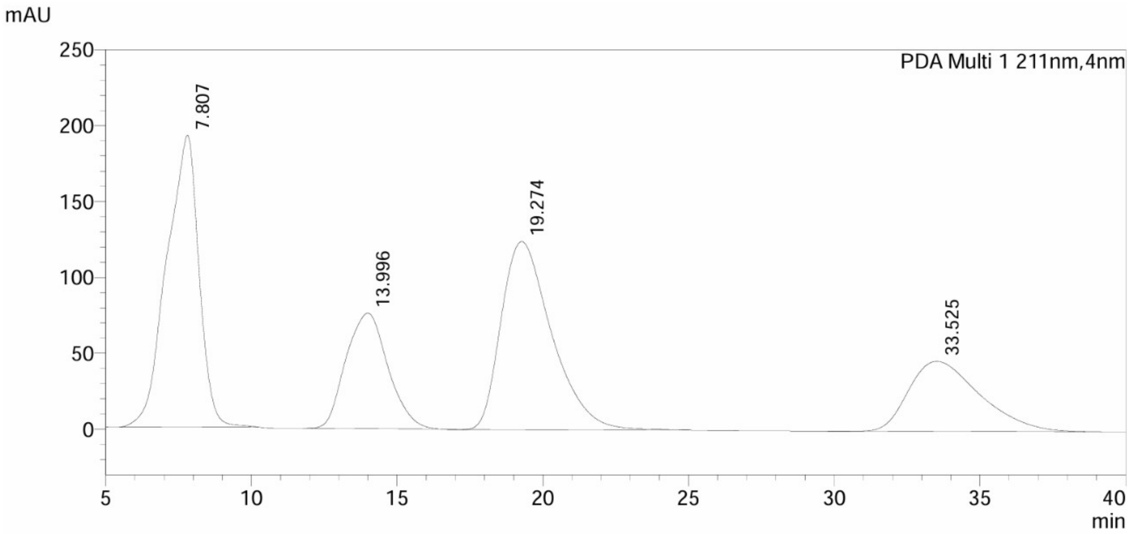

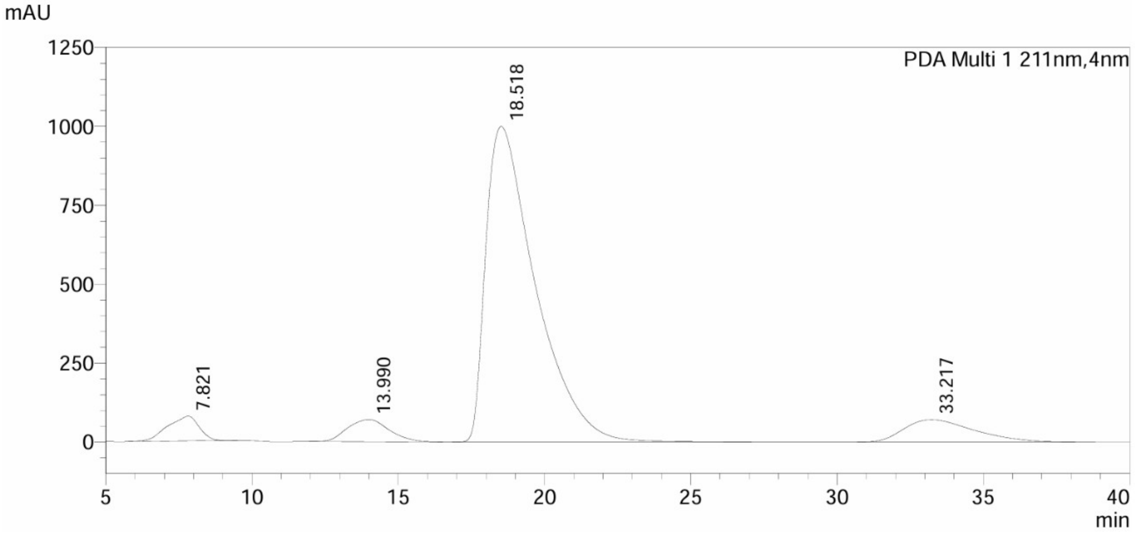

HPLC data for (R,R_a,Z)-**69**: Chiralpak IB (90:10 n-hexane : IPA, flow rate 1.0 mLmin^{-1}, 211 nm, 30 °C) t_R (R,R_a) 15.8 min, t_R (S,S_a) 20.3 min, 97:3 er.

HPLC data for (S,R_a,Z)-**S43**: Chiralpak IB (90:10 n-hexane : IPA, flow rate 1.0 mLmin^{-1}, 211 nm, 30 °C) t_R (S,R_a) 26.1 min, t_R (R,S_a) 40.6 min, 53:47 er.

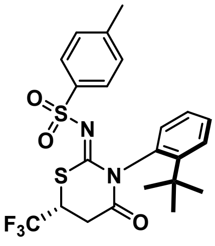

69

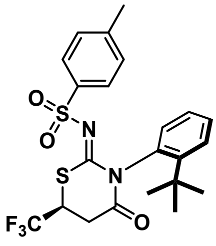

S43

PDA Ch1 211nm

Peak#	Ret. Time	Area%
1	16.366	43.158
2	20.503	44.296
3	26.925	6.000
4	42.109	6.547
Total		100.000

PDA Ch1 211nm

Peak#	Ret. Time	Area%
1	15.846	85.482
2	20.291	2.952
3	26.102	6.160
4	40.568	5.406
Total		100.000

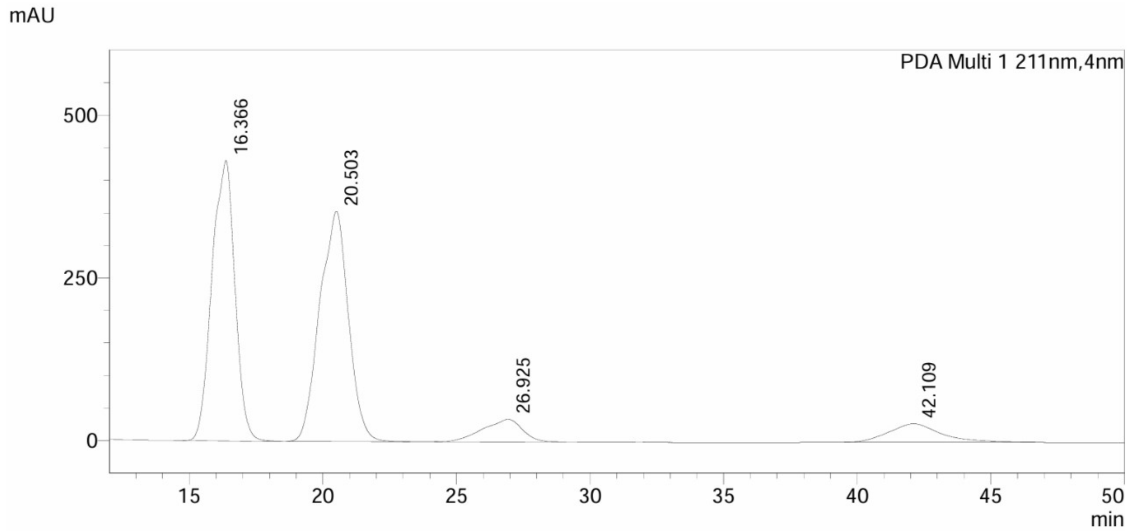

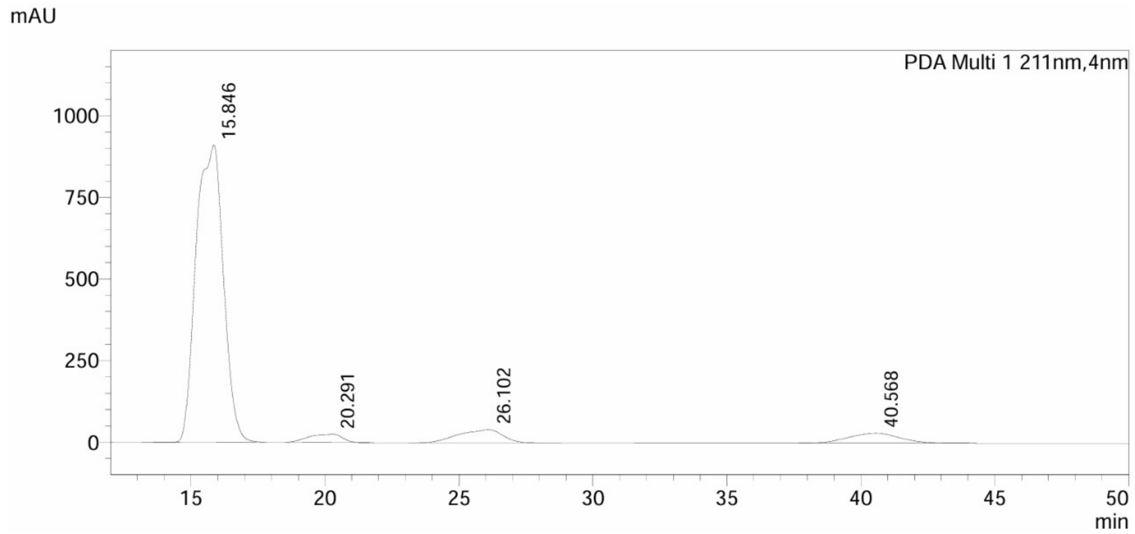

HPLC data for **70**: Chiralpak IB (98:2 *n*-hexane : IPA, flow rate 2.0 mLmin^{-1}, 220 nm, 30 °C) t_R (S,S_a) 8.6 min, t_R (R,R_a) 12.4 min, 98:2 er.

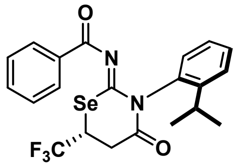

70

PDA Ch2 220nm

Peak#	Ret. Time	Area%
1	8.779	50.240
2	12.607	49.760
Total		100.000

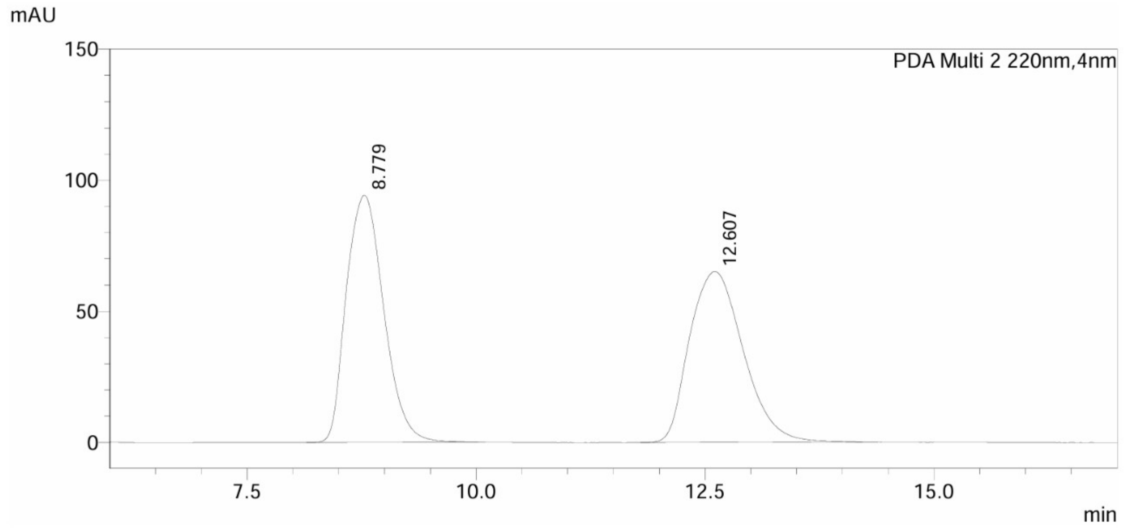

PDA Ch2 220nm

Peak#	Ret. Time	Area%
1	8.604	1.984
2	12.403	98.016
Total		100.000

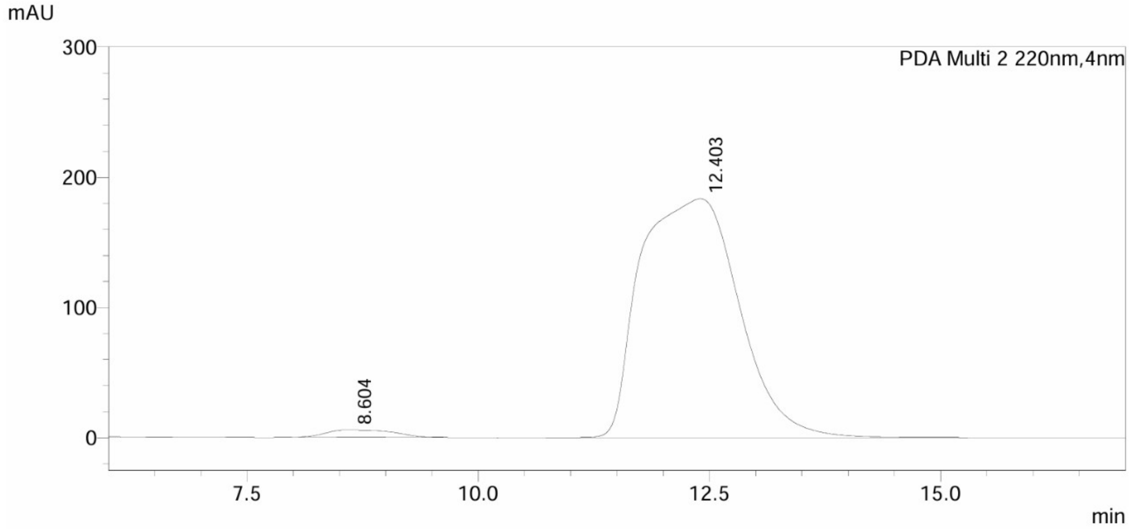

HPLC data for **71**: Chiralpak AD-H (80:20 *n*-hexane : IPA, flow rate 1.0 mLmin^{-1}, 211 nm, 30 °C) t_R (R,R_a) 8.7 min, t_R (S,S_a) 20.3 min, 98:2 er.

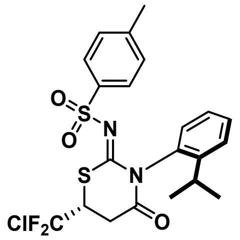

71

PDA Ch1 211nm

Peak#	Ret. Time	Area%
1	8.686	49.704
2	20.163	50.296
Total		100.000

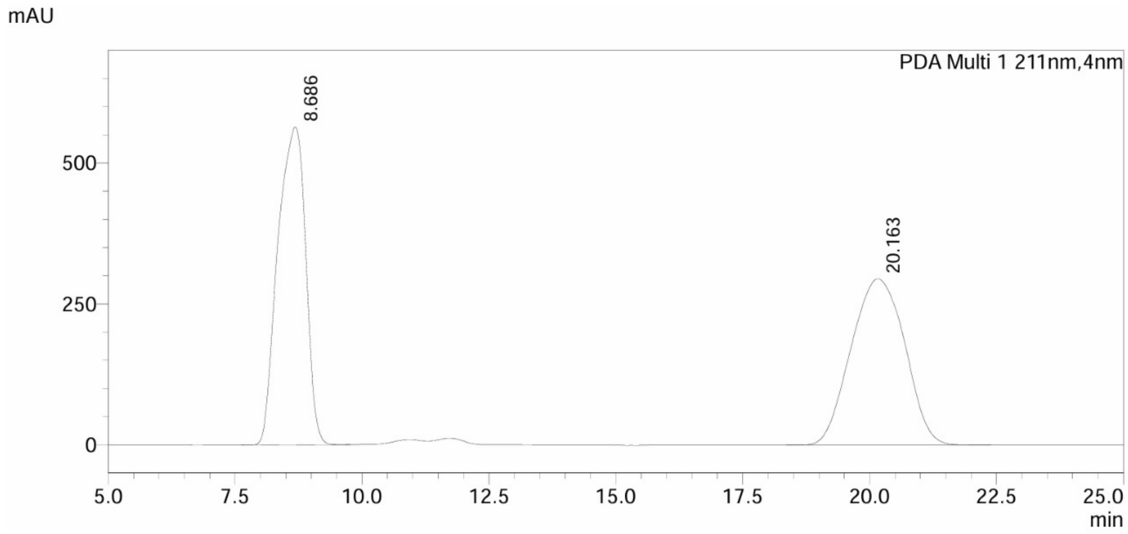

PDA Ch1 211nm

Peak#	Ret. Time	Area%
1	8.717	97.783
2	20.314	2.217
Total		100.000

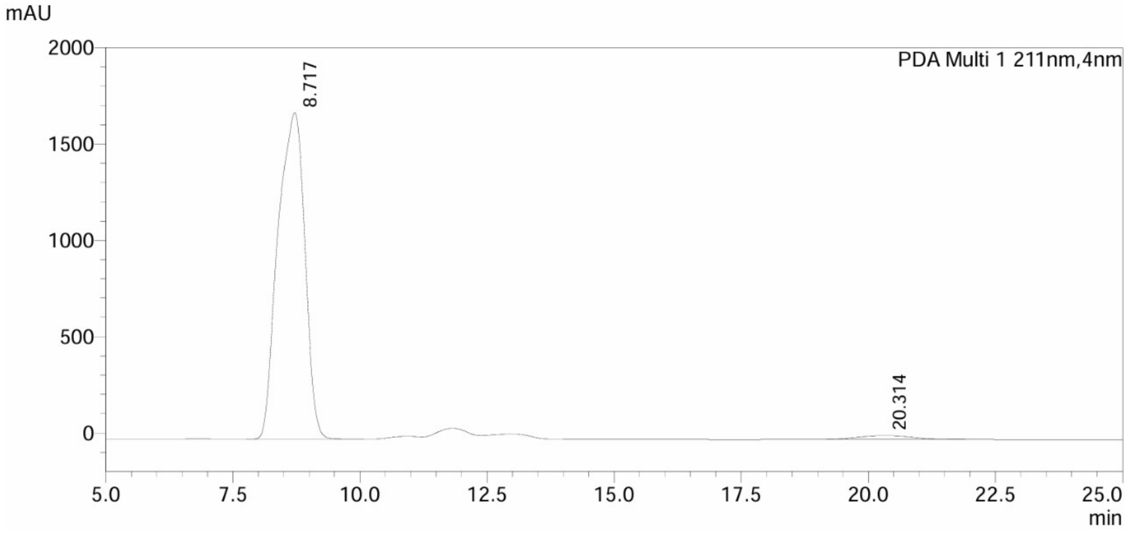

HPLC data for (S,R_a,Z)-**72**: Chiralpak IA (95:5 n-hexane : IPA, flow rate 1.0 mLmin^{-1}, 211 nm, 40 °C) t_R (S,R_a) 47.0 min, t_R (R,S_a) 58.5 min, 92:8 er.

HPLC data for (R,R_a,Z)-**S44**: Chiralpak IA (95:5 n-hexane : IPA, flow rate 1.0 mLmin^{-1}, 211 nm, 40 °C) t_R (S,S_a) 53.4 min, t_R (R,R_a) 76.0 min, 88:12 er.

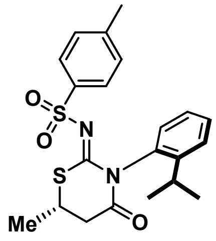

72

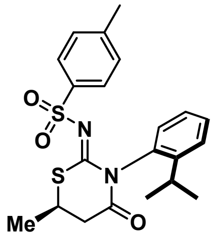

S44

PDA Ch1 211nm

Peak#	Ret. Time	Area%
1	46.947	44.970
2	53.369	4.919
3	58.390	45.037
4	76.266	5.074
Total		100.000

PDA Ch1 211nm

Peak#	Ret. Time	Area%
1	46.960	80.579
2	53.386	1.544
3	58.476	6.725
4	75.987	11.152
Total		100.000

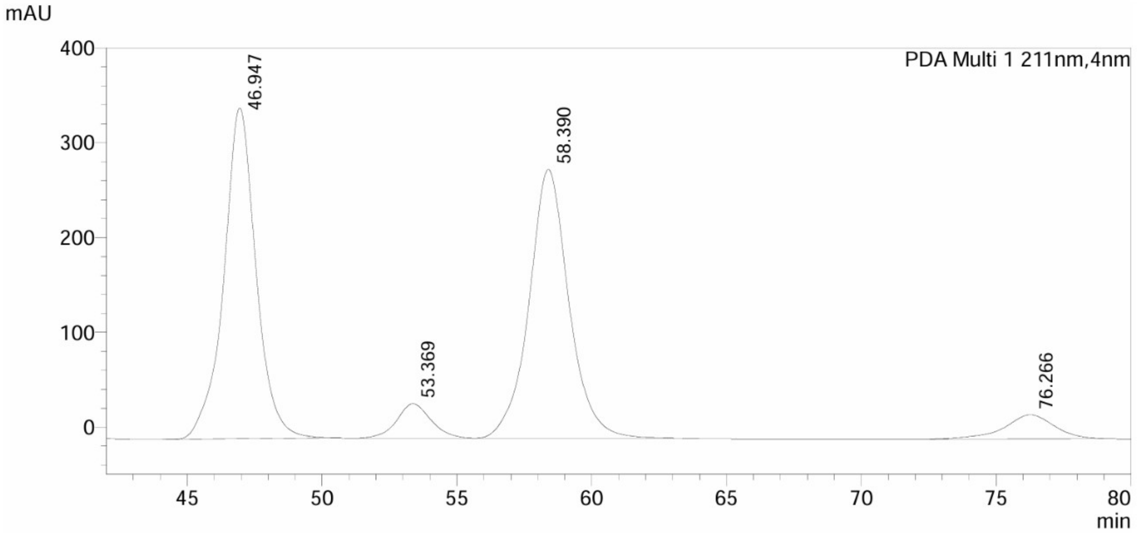

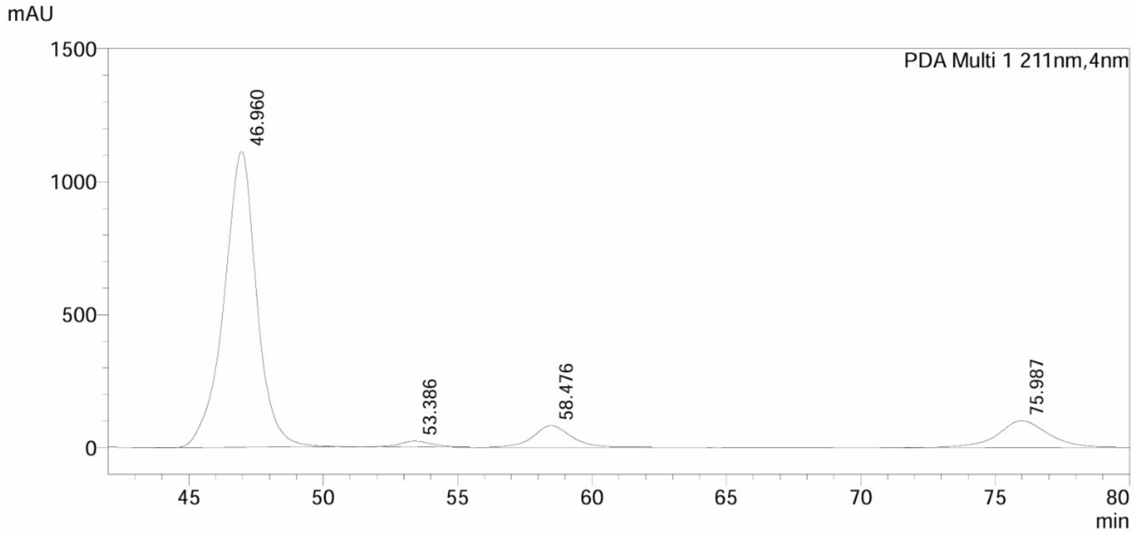

HPLC data for (R,R_a,Z)-**73**: Chiralpak AD-H (90:10 *n*-hexane : IPA, flow rate 1.0 mLmin^{-1}, 211 nm, 30 °C) t_R (R,R_a) 33.1 min, t_R (S,S_a) 47.1 min, 95:5 er.

HPLC data for (S,R_a,Z)-**S45**: Chiralpak AD-H (90:10 *n*-hexane : IPA, flow rate 1.0 mLmin^{-1}, 211 nm, 30 °C) t_R (R,S_a) 29.1 min, t_R (S,R_a) 54.7 min, 68:32 er.

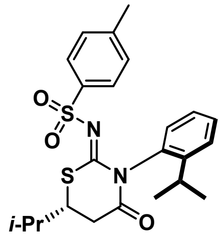

73

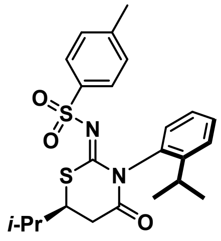

S45

RT [min]	Area%
28.475	5.0985
32.529	45.5746
45.988	44.1252
53.274	5.2018

RT [min]	Area%
29.136	2.4043
33.122	88.3377
47.125	4.2359
54.735	5.0221

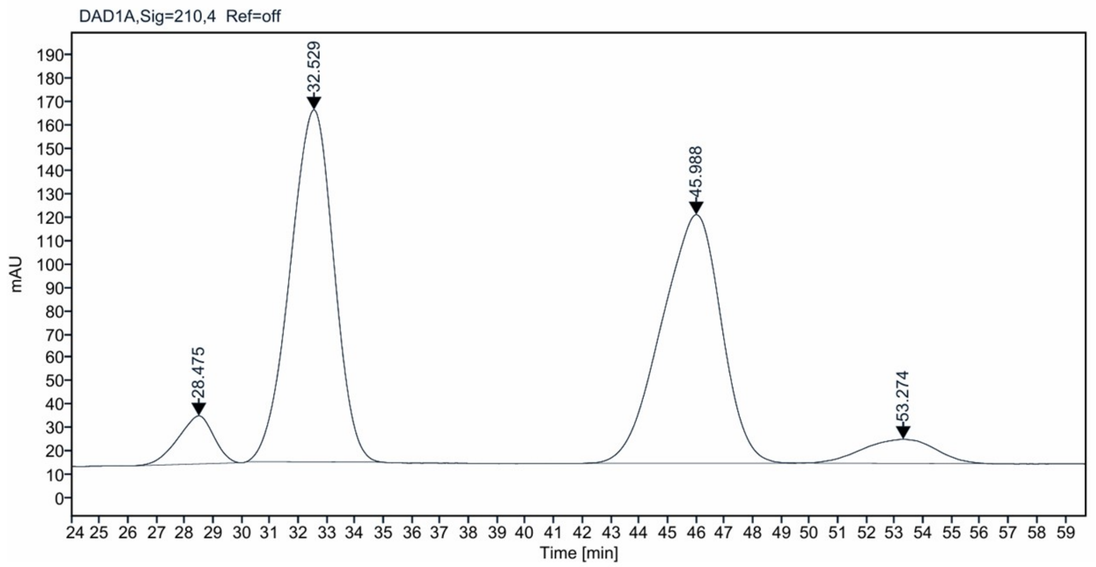
DAD1A,Sig=210,4 Ref=off
28.475
32.529
45.988
53.274
mAU
Time [min]

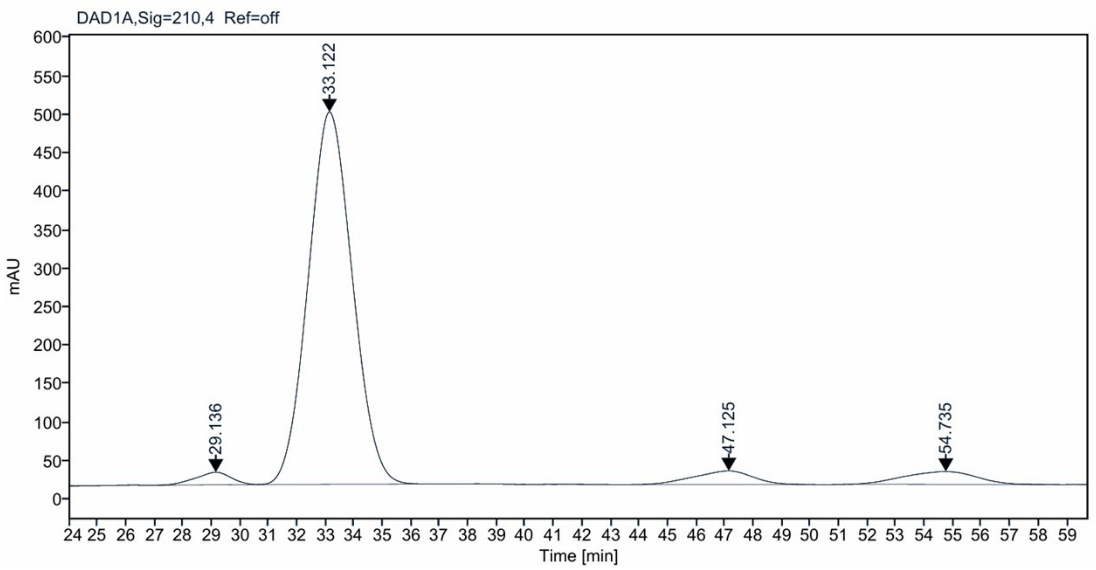
DAD1A,Sig=210,4 Ref=off
29.136
33.122
47.125
54.735
mAU
Time [min]

Appendix III: Computational Data

CF3_Michael_Acceptor/acyl_ammonium_ion_pair

Frequencies, energies and thermodynamic properties:

Lowest Vibrational Mode (1/cm) = 8.8127

2nd Lowest Vibrational Mode (1/cm) = 20.5513

E(RM062X) (a.u.) = -2572.63321020

Thermal correction to Enthalpy (a.u.) = 0.507813

Thermal correction to Gibbs Free Energy (a.u.) = 0.398690

Total Entropy (cal/Kmol) = 229.669

Esp(RM062X) (a.u.) = -2575.26059912

Optimised cartesian coordinates (Angstrom):

C	5.962400	-3.142995	-0.188524
C	5.388744	-4.049277	-1.092356
C	4.096110	-3.860571	-1.569888
C	3.392806	-2.742224	-1.122734
C	3.963289	-1.846183	-0.217057
C	5.261481	-2.030207	0.263490
H	6.979334	-3.310076	0.168376
H	5.962764	-4.913746	-1.427625
H	3.645507	-4.562373	-2.272984
H	5.714622	-1.326511	0.961372
C	1.913953	-0.854228	-0.534198
C	1.211367	1.259066	0.505206
C	2.120253	0.793209	1.652986
C	3.411125	0.219753	1.099938
H	3.989257	-0.269249	1.894416
S	1.759799	-2.265389	-1.546220
N	3.086642	-0.805374	0.104698
N	0.991963	0.116644	-0.417213
C	2.358843	1.868883	2.736006
C	3.559917	2.780976	2.471646
C	2.502589	1.201977	4.104443
H	1.451048	2.493803	2.762723
H	3.548835	3.228928	1.467834
H	3.568703	3.601559	3.203760
H	4.506821	2.230449	2.590911
H	1.601207	0.630068	4.367680
H	3.361110	0.511054	4.115272
H	2.671858	1.955065	4.887870
H	1.558019	-0.032292	2.119000
H	0.234841	1.436911	0.967372
C	1.679656	2.491262	-0.236501
C	1.175578	3.740093	0.144737
C	2.638088	2.425160	-1.253824
C	1.645626	4.906593	-0.456250
H	0.409818	3.795583	0.922840
C	3.107156	3.592190	-1.857340
H	3.024801	1.459285	-1.589722
C	2.617898	4.835052	-1.454613
H	1.246171	5.874167	-0.148073
H	3.855541	3.528662	-2.649052
H	2.985308	5.746967	-1.928114
C	-1.230644	1.078518	-0.999254
H	-0.960322	2.034821	-0.556506
C	-2.442316	0.886618	-1.521937
H	-2.735176	-0.059411	-1.983915
C	-3.474022	1.968012	-1.542540
F	-3.745758	2.344191	-2.799543
F	-4.629141	1.557074	-1.011354
F	-3.094011	3.060763	-0.880328

O -0.511192 -1.078013 -1.637743
C -0.278098 -0.058576 -1.032452
H 4.042490 0.984559 0.623134
O -1.046708 -0.310951 1.307385
C -2.178744 -0.848970 1.127617
C -2.346788 -2.219534 0.768356
C -3.399464 -0.111451 1.140164
C -3.588248 -2.805722 0.580875
C -4.646382 -0.688507 0.949732
C -4.751361 -2.039822 0.637173
F -3.336476 1.201078 1.405954
F -5.747892 0.058015 1.012326
F -3.678997 -4.106381 0.298834
F -1.257508 -2.993159 0.694765
F -5.947021 -2.605332 0.441127

CF3_Michael_Acceptor/C6F5OH
Frequencies, energies and thermodynamic properties:
Lowest Vibrational Mode (1/cm) = 111.0785
2nd Lowest Vibrational Mode (1/cm) = 141.1056
E(RM062X) (a.u.) = -802.732617403
Thermal correction to Enthalpy (a.u.) = 0.075937
Thermal correction to Gibbs Free Energy (a.u.) = 0.029109
Total Entropy (cal/Kmol) = 98.557
Esp(RM062X) (a.u.) = -803.686675322
Optimised cartesian coordinates (Angstrom):
C -0.234767 1.367630 -0.000143
C 1.071480 0.891834 0.000088
C 1.323220 -0.477089 -0.000141
C 0.255627 -1.367778 0.000104
C -1.046230 -0.882459 -0.000210
C -1.319165 0.486396 0.000091
O -2.565435 0.976289 -0.000016
F -2.074173 -1.730692 -0.000105
F 0.478209 -2.671996 0.000128
F 2.569392 -0.926193 -0.000068
F 2.082269 1.746196 0.000114
F -0.452829 2.675256 -0.000055
H -3.203310 0.245342 0.001266

CF3_Michael_Acceptor/CF3_ester
Frequencies, energies and thermodynamic properties:
Lowest Vibrational Mode (1/cm) = 20.0492
2nd Lowest Vibrational Mode (1/cm) = 25.5086
E(RM062X) (a.u.) = -1329.93763879
Thermal correction to Enthalpy (a.u.) = 0.132925
Thermal correction to Gibbs Free Energy (a.u.) = 0.066287
Total Entropy (cal/Kmol) = 140.252
Esp(RM062X) (a.u.) = -1331.50964551
Optimised cartesian coordinates (Angstrom):
C 2.439705 0.186295 -0.263914
H 2.562740 0.705547 -1.215776
C 3.469956 -0.210374 0.477319
H 3.334844 -0.731916 1.429016
O 0.150185 0.387174 -0.689292
C -1.172072 0.201388 -0.400663
C -1.986131 1.309892 -0.185503
C -1.721570 -1.077231 -0.336362
C -3.344355 1.143753 0.068010
C -3.074427 -1.248651 -0.069779
C -3.885756 -0.135611 0.134077
C 4.889532 0.027009 0.064287
F 4.985702 0.680750 -1.091690

F 5.537457 0.742225 0.986429

F 5.545170 -1.127737 -0.070479

O 0.750357 -0.617365 1.234228

C 1.060856 -0.080946 0.214767

F -1.474006 2.526606 -0.230879

F -0.950310 -2.131539 -0.536294

F -5.172609 -0.295806 0.383838

F -4.117529 2.199029 0.260061

F -3.593468 -2.463001 -0.010561

CF3_Michael_Acceptor/cycl-R_rot

Frequencies, energies and thermodynamic properties:

Lowest Vibrational Mode (1/cm) = 14.6628

2nd Lowest Vibrational Mode (1/cm) = 16.1673

E(RM062X) (a.u.) = -3187.94058066

Thermal correction to Enthalpy (a.u.) = 0.681301

Thermal correction to Gibbs Free Energy (a.u.) = 0.555298

Total Entropy (cal/Kmol) = 265.196

Esp(RM062X) (a.u.) = -3190.93033339

Optimised cartesian coordinates (Angstrom):

C -0.105535 0.585637 3.243174

C 1.363968 0.275974 3.442998

H 1.474872 -0.571169 4.135195

H -0.633735 -0.337186 2.962171

H -0.556069 0.957630 4.172777

N 0.431016 1.738794 1.076942

C 0.031255 2.709212 0.092562

C -0.620550 2.279951 -1.062796

C 0.292345 4.057738 0.323682

C -1.027639 3.215825 -2.005601

H -0.798272 1.213112 -1.207471

C -0.111907 5.003586 -0.612550

H 0.807520 4.359416 1.236588

C -0.764478 4.559994 -1.757774

H -1.543021 2.918181 -2.917209

H 0.072126 6.066240 -0.464384

N -1.195337 5.556662 -2.751794

O -0.946709 6.704341 -2.535034

O -1.770907 5.164372 -3.721750

C -0.430948 1.601530 2.174307

O -1.414902 2.291587 2.254357

C 2.134277 1.426064 4.072709

F 1.607231 1.744249 5.255898

F 2.102219 2.528407 3.315103

F 3.411453 1.114798 4.270683

S 2.137250 -0.275625 1.900648

C 1.522894 0.940157 0.747865

N 2.065105 1.071601 -0.389320

C 3.205866 0.389976 -0.801305

C 4.476094 0.741911 -0.310109

C 3.086452 -0.613776 -1.775131

C 5.608915 0.092638 -0.779328

H 4.562009 1.531294 0.437887

C 4.215516 -1.271500 -2.243238

H 2.094538 -0.870899 -2.150791

C 5.460850 -0.907068 -1.738337

H 6.601017 0.350957 -0.412067

H 4.139619 -2.059176 -2.992052

N 6.657180 -1.596581 -2.234157

O 6.506663 -2.464716 -3.063790

O 7.729973 -1.260983 -1.786106

N -1.463506 -0.725766 0.595754

C -2.693595 -0.606912 0.283636

C -0.912388 -2.076641 0.678589
S -3.504749 0.978763 0.165058
N -3.589729 -1.610369 -0.020025
C -2.006222 -3.114717 1.035617
H -0.190716 -2.080366 1.511846
C -0.129445 -2.448626 -0.567746
C -4.993087 0.208651 -0.372569
C -4.851828 -1.189726 -0.411844
C -3.176303 -3.002500 0.063070
C -1.504076 -4.566120 1.202699
H -2.385009 -2.786135 2.018697
C 1.049916 -3.194979 -0.448036
C -0.606841 -2.147494 -1.849520
C -6.191321 0.816742 -0.725948
C -5.914466 -1.994357 -0.822610
H -4.027819 -3.597477 0.425018
H -2.906094 -3.369333 -0.940744
C -1.533561 -5.401514 -0.080788
C -2.299884 -5.278433 2.297624
H -0.456336 -4.501920 1.541399
C 1.702048 -3.686503 -1.578266
H 1.450883 -3.407466 0.546120
C 0.049436 -2.630619 -2.983465
H -1.509556 -1.542506 -1.967751
C -7.259079 0.009608 -1.128974
H -6.294259 1.902479 -0.690451
C -7.117143 -1.378387 -1.176972
H -5.806587 -3.078194 -0.871758
H -1.049118 -4.900576 -0.930954
H -1.015403 -6.357800 0.084756
H -2.569576 -5.639426 -0.370185
H -2.198359 -4.767275 3.266385
H -3.371717 -5.311839 2.043552
H -1.955489 -6.315917 2.422127
C 1.196472 -3.415248 -2.850626
H 2.613386 -4.277095 -1.465498
H -0.340825 -2.396353 -3.975608
H -8.205962 0.472049 -1.410146
H -7.955500 -1.997660 -1.499643
H 1.703212 -3.800521 -3.737474

CF3_Michael_Acceptor/cycl-R

Frequencies, energies and thermodynamic properties:

Lowest Vibrational Mode (1/cm) = 10.7067
2nd Lowest Vibrational Mode (1/cm) = 15.0479
E(RM062X) (a.u.) = -3187.94659271
Thermal correction to Enthalpy (a.u.) = 0.681766
Thermal correction to Gibbs Free Energy (a.u.) = 0.554742
Total Entropy (cal/Kmol) = 267.344
Esp(RM062X) (a.u.) = -3190.91181533

Optimised cartesian coordinates (Angstrom):

C 1.590975 -2.599093 -1.140928
C 2.184908 0.110322 -0.399450
C -0.118050 -0.836384 -0.954335
C 0.341272 -2.198945 -0.396155
H 0.518927 -2.187984 0.687664
H -0.455017 -2.923310 -0.621591
N 0.824075 0.234466 -0.334257
C 0.307325 1.545028 -0.148593
C -0.471013 2.140828 -1.148206
C 0.522491 2.212106 1.066082
C -1.082938 3.369450 -0.916623
H -0.597363 1.616114 -2.095350

C	-0.070427	3.444075	1.301676
H	1.149563	1.750119	1.829167
C	-0.881291	3.992447	0.309935
H	-1.706515	3.843422	-1.673350
H	0.075836	3.973423	2.242099
N	-1.554064	5.271564	0.577252
O	-1.483307	5.720339	1.699218
O	-2.148044	5.799367	-0.333837
O	-0.224977	-0.766009	-2.215572
S	2.970430	-1.453378	-0.803598
N	2.952355	1.114338	-0.200851
C	4.335811	1.063933	-0.174023
C	5.028606	0.471878	0.901371
C	5.071329	1.678347	-1.205775
C	6.415140	0.481676	0.939356
H	4.462787	0.007295	1.710035
C	6.457760	1.688849	-1.176974
H	4.529728	2.144993	-2.029056
C	7.112074	1.088697	-0.103098
H	6.961322	0.026376	1.764259
H	7.036691	2.155016	-1.972987
N	8.576389	1.100624	-0.066421
O	9.162109	1.631990	-0.983199
O	9.124146	0.578255	0.878731
H	1.385187	-2.594554	-2.220057
C	2.043477	-3.995335	-0.769055
F	3.196327	-4.313521	-1.363774
F	1.134897	-4.895771	-1.149020
F	2.223263	-4.142796	0.544798
N	-1.480835	-0.534385	-0.245717
C	-2.552706	-0.314622	-0.981067
C	-1.582055	-0.510366	1.223238
S	-2.623333	-0.440983	-2.735837
N	-3.746506	0.015132	-0.447796
C	-2.598021	0.580825	1.620197
H	-0.597283	-0.189035	1.588722
C	-1.863445	-1.878604	1.817631
C	-4.353940	-0.105894	-2.676960
C	-4.792879	0.113296	-1.370564
C	-3.939936	0.277776	0.977662
C	-2.702863	0.854244	3.136203
H	-2.215673	1.503629	1.152001
C	-1.183314	-2.255486	2.980848
C	-2.803636	-2.756926	1.267958
C	-5.243056	-0.039568	-3.747340
C	-6.131146	0.388453	-1.090753
H	-4.611842	1.142258	1.068265
H	-4.432843	-0.593940	1.434978
C	-3.694729	-0.042308	3.883594
C	-3.063873	2.321949	3.375206
H	-1.696142	0.681014	3.554137
C	-1.456738	-3.472488	3.602762
H	-0.430582	-1.584479	3.402421
C	-3.076770	-3.976850	1.888008
H	-3.328360	-2.505962	0.342917
C	-6.581755	0.239792	-3.477470
H	-4.899197	-0.205290	-4.769699
C	-7.018264	0.448794	-2.163914
H	-6.476604	0.545305	-0.068984
H	-3.547179	-1.112735	3.684436
H	-3.590380	0.114836	4.967132
H	-4.732306	0.215523	3.618093
H	-2.329694	3.004395	2.921695

H -4.050817 2.555902 2.944375
H -3.113242 2.540288 4.452030
C -2.410089 -4.334603 3.059884
H -0.918512 -3.751212 4.510257
H -3.812161 -4.652885 1.448678
H -7.295764 0.292906 -4.300260
H -8.070774 0.661218 -1.971790
H -2.624454 -5.289535 3.542516

CF3_Michael_Acceptor/cycl-S_rot
Frequencies, energies and thermodynamic properties:
Lowest Vibrational Mode (1/cm) = 6.5622
2nd Lowest Vibrational Mode (1/cm) = 9.5325
E(RM062X) (a.u.) = -3187.94262625
Thermal correction to Enthalpy (a.u.) = 0.681392
Thermal correction to Gibbs Free Energy (a.u.) = 0.553543
Total Entropy (cal/Kmol) = 269.081
Esp(RM062X) (a.u.) = -3190.90451915
Optimised cartesian coordinates (Angstrom):
C 1.586170 -2.595895 -1.414898
C 2.111908 -0.205305 -0.057811
C -0.227384 -0.860514 -1.029544
C 0.177710 -2.335673 -0.915665
H 0.136000 -2.724411 0.108098
H -0.549477 -2.885657 -1.529440
N 0.773469 0.018434 -0.221779
C 0.350116 1.372147 -0.044475
C 0.062349 2.178341 -1.150715
C 0.209995 1.880097 1.253679
C -0.402187 3.477674 -0.960135
H 0.194321 1.766181 -2.148583
C -0.239393 3.177996 1.455365
H 0.463257 1.246846 2.105904
C -0.546818 3.950971 0.337913
H -0.640718 4.121801 -1.805159
H -0.356038 3.593209 2.455233
N -1.031659 5.326072 0.542923
O -1.143684 5.719582 1.681128
O -1.292646 5.984416 -0.436704
O -0.434041 -0.449512 -2.207497
S 2.799787 -1.847478 -0.297577
N 2.886359 0.719702 0.372776
C 4.265459 0.638888 0.446732
C 4.905767 0.802287 1.689761
C 5.053933 0.482428 -0.713248
C 6.290021 0.791207 1.781466
H 4.293258 0.939931 2.581379
C 6.437227 0.475389 -0.630371
H 4.559237 0.369113 -1.679057
C 7.038770 0.626463 0.618468
H 6.794863 0.910901 2.739026
H 7.055395 0.356311 -1.519057
N 8.499922 0.617358 0.709043
O 9.130946 0.466499 -0.313479
O 9.001756 0.760097 1.802065
H 1.786422 -3.675285 -1.357060
C 1.898063 -2.218420 -2.859934
F 0.921999 -2.592771 -3.683406
F 3.009062 -2.859775 -3.255923
F 2.132771 -0.921680 -3.044696
N -1.543450 -0.700991 -0.152168
C -2.606192 -0.135755 -0.687008
C -1.573028 -1.149752 1.247489

S -2.740787 0.387744 -2.363829
N -3.740974 0.094623 0.007980
C -2.475659 -0.201039 2.061942
H -0.543942 -1.027972 1.618363
C -1.960650 -2.611522 1.391449
C -4.411236 0.867458 -2.066564
C -4.789202 0.636673 -0.743051
C -3.861303 -0.163291 1.441838
C -2.499158 -0.482543 3.581611
H -2.039269 0.799782 1.921674
C -1.303076 -3.397385 2.344964
C -2.986816 -3.184224 0.632697
C -5.309857 1.415257 -2.979200
C -6.076929 0.932836 -0.295574
H -4.450729 0.653412 1.880317
H -4.411080 -1.105122 1.590756
C -3.596604 -1.448711 4.035895
C -2.618068 0.834910 4.349456
H -1.523991 -0.930570 3.835413
C -1.684058 -4.720656 2.560668
H -0.481062 -2.966422 2.922193
C -3.367874 -4.509795 0.846304
H -3.496242 -2.604620 -0.140986
C -6.596892 1.719476 -2.539064
H -5.011942 1.598548 -4.012924
C -6.973692 1.477924 -1.212888
H -6.379881 0.741576 0.733807
H -3.607543 -2.387310 3.464038
H -3.449939 -1.703230 5.095737
H -4.591072 -0.983093 3.948487
H -1.772899 1.504956 4.132196
H -3.546493 1.362964 4.078687
H -2.640529 0.655358 5.434296
C -2.723513 -5.278731 1.815228
H -1.163186 -5.319872 3.309444
H -4.170786 -4.943629 0.247925
H -7.316796 2.147516 -3.237719
H -7.986785 1.717328 -0.887032
H -3.022582 -6.315076 1.980579

CF3_Michael_Acceptor/cycl-S
Frequencies, energies and thermodynamic properties:
Lowest Vibrational Mode (1/cm) = 13.2539
2nd Lowest Vibrational Mode (1/cm) = 14.6724
E(RM062X) (a.u.) = -3187.93779164
Thermal correction to Enthalpy (a.u.) = 0.681519
Thermal correction to Gibbs Free Energy (a.u.) = 0.554348
Total Entropy (cal/Kmol) = 267.653
Esp(RM062X) (a.u.) = -3190.90224164
Optimised cartesian coordinates (Angstrom):
C 0.228732 -1.974809 -1.873457
C -1.150749 -1.966066 -2.488517
H -1.226517 -1.226439 -3.299900
H 0.224307 -2.655576 -1.012529
H 0.969372 -2.335159 -2.600179
N -0.443525 0.376042 -1.067615
C -0.108585 1.767258 -1.009578
C -0.147958 2.475009 0.198384
C 0.248273 2.431751 -2.192084
C 0.199005 3.821165 0.237404
H -0.468104 1.976308 1.109216
C 0.599621 3.776347 -2.164125
H 0.251658 1.879542 -3.127287

C	0.576359	4.446042	-0.945458
H	0.177153	4.381117	1.171004
H	0.880108	4.305884	-3.073166
N	0.948610	5.869145	-0.909047
O	1.257164	6.399111	-1.951503
O	0.927098	6.431130	0.161940
C	0.715017	-0.534683	-1.562722
O	1.391345	-0.005007	-2.484131
C	-1.504806	-3.309905	-3.092688
F	-2.745469	-3.326583	-3.579672
F	-1.416792	-4.297494	-2.197123
F	-0.675484	-3.605054	-4.093124
S	-2.364635	-1.571671	-1.190487
C	-1.709291	0.022319	-0.684511
N	-2.458706	0.792728	0.006549
C	-3.807517	0.593779	0.244996
C	-4.759409	0.685423	-0.791444
C	-4.251803	0.374401	1.562771
C	-6.112056	0.545924	-0.521483
H	-4.420648	0.875096	-1.810622
C	-5.603281	0.227988	1.839905
H	-3.514066	0.329018	2.364385
C	-6.516628	0.313911	0.792052
H	-6.856052	0.616279	-1.313801
H	-5.956998	0.053217	2.854953
N	-7.944771	0.163606	1.080264
O	-8.273083	-0.040594	2.227829
O	-8.721861	0.250176	0.155899
N	1.607657	-0.704441	-0.242278
C	2.911343	-0.578156	-0.335037
C	1.021184	-1.245081	0.989378
S	3.757367	0.217830	-1.658711
N	3.759341	-1.017669	0.620306
C	1.932208	-2.370821	1.516828
H	0.066371	-1.705947	0.702363
C	0.700501	-0.174146	2.012064
C	5.282846	-0.014558	-0.804718
C	5.101301	-0.694361	0.401970
C	3.325579	-1.814387	1.768574
C	1.356675	-3.142282	2.726057
H	2.008925	-3.083272	0.678220
C	-0.540972	-0.215410	2.655401
C	1.614162	0.827195	2.364110
C	6.554478	0.380400	-1.213206
C	6.177087	-0.986592	1.239540
H	4.048102	-2.632246	1.898900
H	3.357983	-1.180738	2.667891
C	1.707671	-2.549735	4.093404
C	1.794733	-4.606407	2.668821
H	0.259204	-3.116300	2.616116
C	-0.862332	0.717144	3.642137
H	-1.266694	-0.982612	2.371965
C	1.294660	1.759574	3.350401
H	2.577829	0.909040	1.857261
C	7.635937	0.091569	-0.381744
H	6.698605	0.906914	-2.158178
C	7.446914	-0.582958	0.829846
H	6.031383	-1.510045	2.184549
H	1.478447	-1.477456	4.169443
H	1.138622	-3.070636	4.877222
H	2.776296	-2.689155	4.321649
H	1.452045	-5.093687	1.744280
H	2.892857	-4.688237	2.709565

H 1.387477 -5.168850 3.521715
C 0.056710 1.706662 3.993099
H -1.834825 0.674036 4.135989
H 2.013213 2.538863 3.609934
H 8.639215 0.398105 -0.680228
H 8.305008 -0.798296 1.467932
H -0.193480 2.439399 4.762035

CF3_Michael_Acceptor/HyperBTM

Frequencies, energies and thermodynamic properties:

Lowest Vibrational Mode (1/cm) = 26.7604
2nd Lowest Vibrational Mode (1/cm) = 57.9421
E(RM062X) (a.u.) = -1242.68108630
Thermal correction to Enthalpy (a.u.) = 0.371678
Thermal correction to Gibbs Free Energy (a.u.) = 0.305623
Total Entropy (cal/Kmol) = 139.024
Esp(RM062X) (a.u.) = -1243.74354485

Optimised cartesian coordinates (Angstrom):

C -4.262530 0.973031 1.705921
C -5.081884 0.026598 1.089062
C -4.570448 -0.806552 0.088982
C -3.238265 -0.667538 -0.277562
C -2.412615 0.292849 0.336285
C -2.921403 1.115944 1.340791
H -4.669698 1.613207 2.490089
H -6.126268 -0.070457 1.387912
H -5.203340 -1.552803 -0.394046
H -2.284078 1.850300 1.833836
C -0.852054 -0.663172 -1.127167
C 1.406622 -0.103443 -1.317985
C 0.986323 1.302948 -0.824072
C -0.065141 1.190869 0.277299
H -0.509874 2.177247 0.477622
S -2.342062 -1.583659 -1.484368
N -1.120558 0.291010 -0.162611
N 0.251017 -0.908789 -1.701688
C 2.161531 2.231026 -0.444693
C 2.601261 2.139383 1.019395
C 1.826137 3.682431 -0.791325
H 3.015788 1.927239 -1.073553
H 2.788244 1.106938 1.347021
H 3.529866 2.711096 1.165556
H 1.842194 2.577462 1.687042
H 1.618759 3.801777 -1.864957
H 0.937651 4.022659 -0.235092
H 2.658630 4.352142 -0.528432
H 0.480326 1.757972 -1.692374
H 2.009511 0.051835 -2.226024
C 2.288128 -0.851872 -0.332650
C 3.679294 -0.826890 -0.482869
C 1.743460 -1.531774 0.763657
C 4.511225 -1.434125 0.457769
H 4.115252 -0.320870 -1.347869
C 2.572640 -2.139839 1.706383
H 0.659540 -1.591953 0.887188
C 3.959687 -2.086725 1.560541
H 5.594333 -1.401918 0.325517
H 2.131945 -2.662349 2.557484
H 4.607870 -2.563537 2.297932
H 0.371335 0.818121 1.218567

CF3_Michael_Acceptor/MA-product

Frequencies, energies and thermodynamic properties:

Lowest Vibrational Mode (1/cm) = 12.9010

2nd Lowest Vibrational Mode (1/cm) = 27.8348

E(RM062X) (a.u.) = -2748.01566672

Thermal correction to Enthalpy (a.u.) = 0.386975

Thermal correction to Gibbs Free Energy (a.u.) = 0.280216

Total Entropy (cal/Kmol) = 224.692

Esp(RM062X) (a.u.) = -2750.86693905

Optimised cartesian coordinates (Angstrom):

C	-0.917917	2.162890	-0.488778
H	-1.255076	1.216472	-0.935729
C	-1.770869	2.606724	0.692805
C	-2.960309	3.409949	0.184622
F	-2.547775	4.524841	-0.418976
F	-3.685298	2.723221	-0.699600
F	-3.768196	3.766660	1.180599
O	1.090522	2.340488	0.854832
C	0.545822	2.038522	-0.163601
H	-1.193708	3.288387	1.333634
S	-2.490650	1.278203	1.769744
C	-1.501665	-0.184245	1.339833
N	-1.982077	-1.241572	0.830636
N	-0.195394	-0.053209	1.721700
H	0.008692	0.772434	2.277579
C	-3.279725	-1.408839	0.354670
C	-4.025073	-2.493438	0.847713
C	-3.833630	-0.584472	-0.640207
C	-5.309223	-2.736315	0.382324
H	-3.578171	-3.137606	1.605368
C	-5.113317	-0.827789	-1.119736
H	-3.259310	0.248129	-1.043035
C	-5.834266	-1.897573	-0.597330
H	-5.901693	-3.566772	0.763220
H	-5.555016	-0.197252	-1.889969
N	-7.188604	-2.154786	-1.101555
O	-7.615847	-1.427749	-1.969151
O	-7.803303	-3.079870	-0.621831
C	0.906170	-0.872455	1.472366
C	0.854248	-2.074146	0.748841
C	2.140506	-0.410961	1.977838
C	2.026546	-2.791455	0.525652
H	-0.090872	-2.441076	0.360292
C	3.305215	-1.118246	1.747305
H	2.173587	0.521093	2.544105
C	3.230049	-2.303239	1.015846
H	2.010109	-3.718970	-0.045695
H	4.265237	-0.763012	2.120384
N	4.468977	-3.022042	0.713710
O	5.509450	-2.413277	0.838387
O	4.385259	-4.169369	0.344662
O	1.196724	1.519310	-1.238499
C	2.501497	1.127695	-1.121169
C	3.525915	1.971331	-0.694340
C	2.811011	-0.171415	-1.523192
C	4.827554	1.486432	-0.591852
C	4.114653	-0.645614	-1.444734
C	5.129952	0.184958	-0.981643
F	4.381310	-1.894282	-1.792761
F	1.853398	-0.971636	-1.956941
F	6.370211	-0.253670	-0.908305
F	5.793306	2.286474	-0.172168
F	3.286512	3.234580	-0.395435
H	-0.986231	2.916460	-1.291823

CF3_Michael_Acceptor/MA-R_pt_rot

Frequencies, energies and thermodynamic properties:

Lowest Vibrational Mode (1/cm) = 14.3681

2nd Lowest Vibrational Mode (1/cm) = 18.4051

E(RM062X) (a.u.) = -3187.94090470

Thermal correction to Enthalpy (a.u.) = 0.681771

Thermal correction to Gibbs Free Energy (a.u.) = 0.556928

Total Entropy (cal/Kmol) = 262.754

Esp(RM062X) (a.u.) = -3190.90298177

Optimised cartesian coordinates (Angstrom):

C	5.447228	-1.308211	-2.506161
C	5.679378	-2.213699	-1.457984
C	5.118730	-2.012853	-0.202107
C	4.304012	-0.892748	-0.027309
C	4.073513	0.004150	-1.071009
H	6.296141	-3.096062	-1.633089
H	5.284279	-2.721669	0.610479
C	2.673401	0.865456	0.529086
C	1.263271	2.865604	0.231911
C	2.446023	3.317487	-0.646001
C	2.893961	2.174703	-1.541264
H	3.822716	2.434710	-2.066148
S	3.393503	-0.450370	1.398131
N	3.184725	1.015333	-0.695269
N	1.675900	1.653510	0.996914
C	2.175938	4.621603	-1.432947
C	1.579397	4.408227	-2.827012
C	3.466184	5.435113	-1.540705
H	1.457505	5.209345	-0.838542
H	0.687521	3.766066	-2.822287
H	1.291174	5.378877	-3.255822
H	2.321233	3.961188	-3.507535
H	3.856270	5.705674	-0.548756
H	4.246408	4.864662	-2.069783
H	3.293038	6.363072	-2.104650
H	3.271963	3.520694	0.055385
H	1.113129	3.657636	0.977625
C	-0.019533	2.666262	-0.543037
C	-0.951241	3.707571	-0.602887
C	-0.253157	1.495647	-1.266549
C	-2.082724	3.593832	-1.409142
H	-0.784341	4.617362	-0.020175
C	-1.386866	1.376236	-2.068984
H	0.447543	0.662088	-1.193043
C	-2.296155	2.431949	-2.152535
H	-2.801238	4.413797	-1.455439
H	-1.564889	0.453504	-2.625543
H	-3.182766	2.339568	-2.782412
C	-0.207411	2.035126	2.616766
H	-0.550774	2.737585	1.855269
C	-1.439892	1.213084	3.019570
C	-1.244702	0.192812	4.136959
F	-2.429058	-0.081427	4.700507
F	-0.710789	-0.968625	3.795140
F	-0.474579	0.722325	5.099207
O	1.571255	0.488592	2.947686
C	1.042397	1.289275	2.222981
H	2.134852	1.897355	-2.286738
C	4.648216	-0.184638	-2.330108
H	4.455178	0.505803	-3.151284
H	5.893504	-1.494130	-3.483772
H	-2.088572	1.953622	3.513809
S	-2.574821	0.690999	1.697141

C -1.711349 -0.601830 0.731543

N -0.454671 -0.751219 1.043739

N -2.404182 -1.347835 -0.098851

C 0.314288 -1.741234 0.468316

C 1.048739 -2.593953 1.325132

C 0.483083 -1.902616 -0.927659

C 1.965351 -3.504057 0.826729

H 0.899548 -2.485871 2.399946

C 1.420486 -2.791203 -1.437056

H -0.126967 -1.311901 -1.610050

C 2.166129 -3.570601 -0.553492

H 2.546084 -4.144533 1.489933

H 1.584166 -2.886246 -2.509989

N 3.212544 -4.437923 -1.080795

O 3.350605 -4.504901 -2.283308

O 3.909644 -5.037280 -0.288269

C -3.692162 -1.212467 -0.508416

C -4.393240 -2.403325 -0.851743

C -4.383309 0.016932 -0.698425

C -5.694654 -2.380892 -1.311356

H -3.864833 -3.349740 -0.730189

C -5.688004 0.046619 -1.162960

H -3.874410 0.959735 -0.504988

C -6.341590 -1.149362 -1.461107

H -6.223375 -3.301033 -1.557349

H -6.208128 0.993086 -1.306995

N -7.710409 -1.113650 -1.945431

O -8.248836 -0.031706 -2.066305

O -8.253465 -2.167506 -2.207410

H 0.095575 2.626129 3.496572

CF3_Michael_Acceptor/MA-R_pt

Frequencies, energies and thermodynamic properties:

Lowest Vibrational Mode (1/cm) = 13.6087

2nd Lowest Vibrational Mode (1/cm) = 15.7017

E(RM062X) (a.u.) = -3187.95670984

Thermal correction to Enthalpy (a.u.) = 0.681533

Thermal correction to Gibbs Free Energy (a.u.) = 0.555523

Total Entropy (cal/Kmol) = 265.210

Esp(RM062X) (a.u.) = -3190.91949549

Optimised cartesian coordinates (Angstrom):

C 5.807566 -1.715154 1.593487

C 6.530882 -1.262845 0.480135

C 5.987246 -0.328248 -0.393896

C 4.701855 0.142104 -0.125707

C 3.978900 -0.319252 0.975998

H 7.528933 -1.658004 0.289059

H 6.540519 0.016596 -1.268417

C 2.398585 1.014147 -0.019449

C 0.189848 1.520864 0.932981

C 0.332919 0.172244 1.652752

C 1.758949 -0.053838 2.111723

H 1.900535 -1.099930 2.413260

S 3.742979 1.231142 -1.100493

N 2.686120 0.217026 1.009034

N 1.185694 1.602456 -0.163518

C -0.702815 -0.045202 2.778037

C -0.268208 0.457955 4.156461

C -1.076249 -1.526271 2.846912

H -1.605411 0.516317 2.481895

H 0.072399 1.503231 4.144615

H -1.112895 0.388610 4.857113

H 0.543819 -0.163617 4.565633

H	-1.471724	-1.880152	1.883098
H	-0.196102	-2.140012	3.100654
H	-1.839604	-1.702339	3.619075
H	0.126176	-0.577854	0.877274
H	-0.791823	1.482902	0.450387
C	0.245685	2.744991	1.820615
C	-0.957019	3.323098	2.241611
C	1.456992	3.276785	2.277102
C	-0.951970	4.395467	3.132632
H	-1.904813	2.924532	1.869544
C	1.461809	4.350785	3.166813
H	2.411746	2.862620	1.941945
C	0.258097	4.906393	3.602477
H	-1.896230	4.836095	3.456403
H	2.411392	4.756491	3.519355
H	0.264314	5.746144	4.299281
C	-0.522119	2.794975	-1.607006
H	-0.306741	3.685544	-2.213654
C	-1.464196	1.878473	-2.411117
C	-2.368521	2.729432	-3.289006
F	-3.143941	3.541382	-2.560553
F	-1.649328	3.505861	-4.105452
F	-3.166507	1.991504	-4.054096
O	1.674034	2.228395	-2.287182
C	0.850516	2.185729	-1.408768
H	2.041989	0.597108	2.953324
C	4.524912	-1.249947	1.861553
H	3.968091	-1.614348	2.724012
H	6.252227	-2.456051	2.258552
H	-0.886744	1.229607	-3.083690
S	-2.539592	0.802272	-1.404463
C	-1.498712	-0.717598	-1.246840
N	-2.056470	-1.782555	-0.755373
C	-3.340011	-1.899514	-0.281055
C	-4.133117	-2.976375	-0.741962
C	-3.885230	-1.050494	0.711186
C	-5.416739	-3.179746	-0.265868
H	-3.707699	-3.643507	-1.492303
C	-5.168519	-1.249394	1.197278
H	-3.279184	-0.236545	1.110551
C	-5.924255	-2.309544	0.700257
H	-6.031894	-4.001297	-0.630505
H	-5.589482	-0.597065	1.961124
N	-7.276880	-2.519205	1.208985
O	-7.694987	-1.751833	2.049179
O	-7.913789	-3.449924	0.765404
N	-0.273940	-0.554370	-1.700365
C	0.743239	-1.453919	-1.516856
C	0.795983	-2.480485	-0.531534
C	1.900575	-1.267033	-2.317670
C	1.965672	-3.191107	-0.302750
H	-0.086301	-2.693260	0.067423
C	3.058827	-1.993830	-2.113747
H	1.864895	-0.492654	-3.085749
C	3.093450	-2.936178	-1.083364
H	2.015024	-3.946763	0.481111
H	3.948649	-1.822719	-2.719347
N	4.338724	-3.633075	-0.796905
O	5.277508	-3.459266	-1.545931
O	4.380473	-4.351750	0.180579
H	-0.997901	3.135257	-0.680718

CF3_Michael_Acceptor/MA-R

Frequencies, energies and thermodynamic properties:

Lowest Vibrational Mode (1/cm) = 5.8319

2nd Lowest Vibrational Mode (1/cm) = 11.7034

E(RM062X) (a.u.) = -3187.94993819

Thermal correction to Enthalpy (a.u.) = 0.682306

Thermal correction to Gibbs Free Energy (a.u.) = 0.550292

Total Entropy (cal/Kmol) = 277.849

Esp(RM062X) (a.u.) = -3190.91703647

Optimised cartesian coordinates (Angstrom):

C	6.934837	2.976985	-1.928809
C	6.037967	4.052720	-1.898872
C	4.697315	3.853743	-1.579073
C	4.276106	2.556326	-1.293124
C	5.170476	1.485879	-1.332456
H	6.392293	5.059195	-2.124405
H	3.995971	4.689125	-1.549610
C	3.241158	0.379646	-0.710107
C	3.112684	-2.006822	-0.219214
C	4.271262	-2.127674	-1.224693
C	5.263751	-0.999247	-1.019218
H	5.986829	-0.974273	-1.844912
S	2.659788	2.027756	-0.845834
N	4.545288	0.274631	-1.017595
N	2.506663	-0.664393	-0.328212
C	4.941613	-3.520776	-1.242737
C	6.103708	-3.679790	-0.258212
C	5.413716	-3.853642	-2.658709
H	4.160517	-4.249732	-0.970609
H	5.844186	-3.380914	0.767074
H	6.421136	-4.732333	-0.229992
H	6.975191	-3.086758	-0.578169
H	6.152062	-3.115116	-3.010485
H	5.894659	-4.842306	-2.685018
H	4.575804	-3.862431	-3.370848
H	3.807739	-1.972820	-2.212831
H	2.343181	-2.709173	-0.560631
C	3.474552	-2.343198	1.213590
C	4.092207	-1.413255	2.056584
C	3.237610	-3.638130	1.686649
C	4.493746	-1.783493	3.340360
H	4.261215	-0.386057	1.722948
C	3.640659	-4.009729	2.968030
H	2.731783	-4.361835	1.042299
C	4.276644	-3.083976	3.795935
H	4.974627	-1.049348	3.988949
H	3.450807	-5.023792	3.323739
H	4.590609	-3.371701	4.800667
C	0.394919	-1.538519	0.538488
H	0.779978	-2.544895	0.668083
C	-1.017786	-1.250947	0.879591
C	-1.591282	-2.266094	1.847003
F	-0.874397	-2.306237	2.973521
F	-2.847800	-1.982003	2.194200
F	-1.589954	-3.502546	1.337137
O	0.668656	0.691287	-0.140454
C	1.108007	-0.479159	0.042636
H	5.817121	-1.093602	-0.072735
C	6.516700	1.679377	-1.645977
H	7.221141	0.848184	-1.664333
H	7.982357	3.154974	-2.175133
H	-1.122483	-0.269853	1.365307
S	-2.149686	-1.196149	-0.618692
C	-2.907747	0.433942	-0.403621

N -1.989005 1.412857 -0.163328
H -0.992984 1.118586 -0.213031
C -2.183996 2.742228 0.185204
C -1.020986 3.451004 0.561489
C -3.425635 3.404535 0.183400
C -1.088767 4.783743 0.921268
H -0.067361 2.919394 0.572674
C -3.492597 4.743470 0.551514
H -4.325628 2.873317 -0.110042
C -2.331824 5.418010 0.913186
H -0.196724 5.335254 1.214210
H -4.445677 5.270574 0.553288
N -2.413625 6.828644 1.293037
O -3.504414 7.354382 1.295864
O -1.384328 7.396930 1.584483
N -4.162660 0.645295 -0.480896
C -5.112614 -0.332473 -0.730827
C -6.125111 -0.554741 0.220576
C -5.131453 -1.056806 -1.937302
C -7.118412 -1.493466 -0.012464
H -6.109660 0.018523 1.147884
C -6.127244 -1.992293 -2.182204
H -4.364247 -0.867316 -2.689686
C -7.106127 -2.199480 -1.213969
H -7.901026 -1.683170 0.720567
H -6.152659 -2.559826 -3.111305
N -8.159636 -3.186354 -1.468503
O -9.005361 -3.344169 -0.616861
O -8.128372 -3.791013 -2.516811

CF3_Michael_Acceptor/MA-S_pt_rot
Frequencies, energies and thermodynamic properties:
Lowest Vibrational Mode (1/cm) = 11.7212
2nd Lowest Vibrational Mode (1/cm) = 18.0102
E(RM062X) (a.u.) = -3187.95694997
Thermal correction to Enthalpy (a.u.) = 0.682217
Thermal correction to Gibbs Free Energy (a.u.) = 0.555784
Total Entropy (cal/Kmol) = 266.101
Esp(RM062X) (a.u.) = -3190.92099530
Optimised cartesian coordinates (Angstrom):
C -3.769213 -4.339921 0.754915
C -2.864528 -4.756088 1.744323
C -1.966144 -3.861582 2.314739
C -1.984257 -2.543191 1.858903
C -2.869196 -2.136892 0.859989
H -2.865209 -5.796505 2.070303
H -1.261439 -4.180066 3.083996
C -1.718199 -0.183264 1.206340
C -2.207296 1.938804 0.073136
C -2.693532 1.050204 -1.077409
C -3.455486 -0.147441 -0.546771
H -3.600197 -0.889922 -1.342841
S -0.956159 -1.223274 2.369328
N -2.678913 -0.794131 0.514662
N -1.386031 1.112681 0.993691
C -3.459377 1.816876 -2.176848
C -4.965079 1.941916 -1.933100
C -3.195351 1.166577 -3.535552
H -3.030771 2.833056 -2.202106
H -5.466461 0.967917 -2.047793
H -5.206327 2.336782 -0.935686
H -5.403722 2.621850 -2.677623
H -2.125200 1.188850 -3.789092

H	-3.527057	0.115302	-3.541041
H	-3.744199	1.691218	-4.331024
H	-1.762716	0.668383	-1.525357
H	-1.535538	2.661826	-0.393186
C	-3.266429	2.722507	0.817204
C	-3.452930	4.067358	0.478889
C	-4.091965	2.138985	1.785405
C	-4.470854	4.811454	1.074237
H	-2.796098	4.532445	-0.261367
C	-5.107555	2.884715	2.382677
H	-3.950424	1.098911	2.090507
C	-5.304186	4.218734	2.022630
H	-4.608835	5.858582	0.800181
H	-5.747064	2.421013	3.135417
H	-6.100658	4.799065	2.491162
C	0.342411	2.898607	1.027137
H	1.108874	3.260097	1.721896
C	0.974308	2.545302	-0.334595
C	1.106320	3.782386	-1.196874
F	1.873785	4.716396	-0.632937
F	1.607930	3.512246	-2.396192
F	-0.103910	4.338795	-1.394048
O	0.285949	1.043458	2.531625
C	-0.229680	1.641104	1.624661
H	-4.438195	0.124692	-0.132841
C	-3.786296	-3.027914	0.297391
H	-4.483385	-2.715601	-0.479584
H	-4.460270	-5.062863	0.319974
H	0.356085	1.830036	-0.904554
S	2.569067	1.708263	-0.149790
C	2.116756	-0.059432	-0.479267
N	3.053438	-0.926404	-0.246706
N	0.881329	-0.263494	-0.886272
C	4.385838	-0.666200	-0.062748
C	5.152561	0.091556	-0.982034
C	5.055148	-1.244182	1.041956
C	6.512021	0.281346	-0.793311
H	4.663157	0.518977	-1.857997
C	6.412403	-1.056780	1.238858
H	4.472615	-1.841030	1.744763
C	7.129706	-0.290774	0.318695
H	7.103337	0.861506	-1.500567
H	6.925583	-1.493166	2.094771
N	8.561103	-0.092012	0.519234
O	9.077735	-0.604801	1.488983
O	9.163618	0.576750	-0.293063
C	0.356215	-1.523284	-1.051711
C	-0.671454	-1.690504	-2.019120
C	0.710275	-2.662465	-0.281302
C	-1.345297	-2.888843	-2.172910
H	-0.912813	-0.845931	-2.666112
C	0.041176	-3.867171	-0.432915
H	1.510926	-2.580103	0.449273
C	-0.991876	-3.971615	-1.363822
H	-2.136310	-3.002642	-2.913928
H	0.300409	-4.731423	0.178131
N	-1.740769	-5.212091	-1.465953
O	-2.723544	-5.228043	-2.179725
O	-1.361432	-6.168865	-0.823480
H	-0.428320	3.676829	0.932259

CF3_Michael_Acceptor/MA-S_pt

Frequencies, energies and thermodynamic properties:

Lowest Vibrational Mode (1/cm) = 15.7742

2nd Lowest Vibrational Mode (1/cm) = 17.2012

E(RM062X) (a.u.) = -3187.94879907

Thermal correction to Enthalpy (a.u.) = 0.681318

Thermal correction to Gibbs Free Energy (a.u.) = 0.555805

Total Entropy (cal/Kmol) = 264.163

Esp(RM062X) (a.u.) = -3190.91377171

Optimised cartesian coordinates (Angstrom):

C	5.658991	1.568418	2.061192
C	5.895732	2.201915	0.829958
C	5.272775	1.761821	-0.331554
C	4.389580	0.685781	-0.225641
C	4.156229	0.058040	0.998478
H	6.568409	3.059252	0.783660
H	5.442950	2.259791	-1.287565
C	2.669501	-1.069602	-0.334035
C	1.109070	-2.800269	0.472115
C	2.275582	-3.135067	1.421060
C	2.864996	-1.856416	1.990511
H	3.793386	-2.069714	2.536986
S	3.406507	-0.011943	-1.491241
N	3.207776	-0.962484	0.882065
N	1.617165	-1.887794	-0.589410
C	1.914787	-4.161190	2.520802
C	1.390706	-3.541240	3.819497
C	3.130126	-5.039150	2.821421
H	1.125602	-4.808900	2.104955
H	0.566383	-2.832190	3.659790
H	1.024118	-4.336781	4.484200
H	2.196308	-3.015850	4.356518
H	3.454779	-5.596883	1.930999
H	3.977736	-4.426040	3.167702
H	2.898299	-5.764862	3.614276
H	3.048876	-3.593515	0.782573
H	0.841947	-3.735951	-0.036282
C	-0.109572	-2.245267	1.174528
C	-1.133861	-3.117671	1.556377
C	-0.193977	-0.895065	1.522284
C	-2.204175	-2.654951	2.319744
H	-1.086245	-4.169418	1.262239
C	-1.265925	-0.429234	2.282553
H	0.574283	-0.192151	1.191378
C	-2.263568	-1.312484	2.697025
H	-2.994949	-3.344256	2.620132
H	-1.325015	0.628332	2.548647
H	-3.100357	-0.947631	3.295179
C	-0.326921	-2.510269	-2.072297
H	-0.747603	-2.961367	-1.171005
C	-1.346597	-1.535299	-2.688607
C	-2.170555	-2.242352	-3.748047
F	-1.380796	-2.773865	-4.688375
F	-2.890459	-3.246892	-3.233137
F	-3.013791	-1.419990	-4.364123
O	1.568346	-1.262964	-2.769147
C	1.004792	-1.825606	-1.867059
H	2.170307	-1.334845	2.664895
C	4.792966	0.486584	2.165944
H	4.600463	0.009384	3.126785
H	6.157291	1.937767	2.958202
H	-0.827650	-0.704752	-3.186250
S	-2.496446	-0.819326	-1.468480
C	-1.607367	0.708104	-0.976326
N	-0.391875	0.781221	-1.461243

N -2.221175 1.628537 -0.277459
C 0.472361 1.798046 -1.140582
C 1.291191 2.328767 -2.167584
C 0.674338 2.281453 0.177237
C 2.304616 3.229810 -1.893817
H 1.125643 1.977375 -3.186938
C 1.703102 3.165855 0.464805
H 0.018008 1.935297 0.974469
C 2.520713 3.621793 -0.570189
H 2.944289 3.619896 -2.684931
H 1.886035 3.504478 1.484139
N 3.650450 4.483308 -0.256909
O 3.794861 4.848052 0.890656
O 4.409102 4.781282 -1.157822
C -3.483879 1.650256 0.231727
C -4.079447 2.932760 0.393294
C -4.238197 0.530940 0.677971
C -5.343138 3.094071 0.925187
H -3.499675 3.798794 0.071913
C -5.506046 0.685854 1.217528
H -3.813787 -0.469646 0.634355
C -6.055173 1.961818 1.333449
H -5.790938 4.081222 1.032165
H -6.075165 -0.177767 1.560161
N -7.385369 2.119230 1.900149
O -7.983246 1.121062 2.246061
O -7.834429 3.242177 2.001201
H -0.107708 -3.318759 -2.788754

CF3_Michael_Acceptor/MA-S

Frequencies, energies and thermodynamic properties:

Lowest Vibrational Mode (1/cm) = 11.3528
2nd Lowest Vibrational Mode (1/cm) = 20.5196
E(RM062X) (a.u.) = -3187.95028278
Thermal correction to Enthalpy (a.u.) = 0.682002
Thermal correction to Gibbs Free Energy (a.u.) = 0.555777
Total Entropy (cal/Kmol) = 265.663
Esp(RM062X) (a.u.) = -3190.91353033

Optimised cartesian coordinates (Angstrom):

C -4.936250 -3.683418 -1.715028
C -3.845238 -4.559349 -1.654702
C -2.546498 -4.073436 -1.523544
C -2.370008 -2.694429 -1.428306
C -3.460669 -1.824333 -1.493549
H -4.012047 -5.635784 -1.706920
H -1.693539 -4.752370 -1.473594
C -1.752096 -0.320819 -1.129802
C -2.072958 2.069689 -0.815546
C -3.193372 1.888575 -1.857653
C -3.991880 0.630629 -1.555636
H -4.641338 0.376297 -2.404923
S -0.858378 -1.819241 -1.228618
N -3.066005 -0.487890 -1.364338
N -1.223990 0.858959 -0.816892
C -4.078755 3.141860 -2.047637
C -5.293723 3.218064 -1.119172
C -4.534440 3.239348 -3.504303
H -3.435097 4.012689 -1.838267
H -5.034329 3.098009 -0.057933
H -5.783530 4.195811 -1.235461
H -6.038879 2.449749 -1.380135
H -3.677760 3.304833 -4.190666
H -5.131141 2.357273 -3.787515

H	-5.162975	4.128834	-3.656564
H	-2.666772	1.714803	-2.810357
H	-1.424254	2.876201	-1.179409
C	-2.571484	2.448867	0.564201
C	-2.478595	3.782635	0.976181
C	-3.197821	1.522983	1.406342
C	-3.017407	4.189187	2.196481
H	-1.978608	4.509924	0.331154
C	-3.733361	1.926074	2.629639
H	-3.268814	0.472764	1.115482
C	-3.650011	3.261873	3.024695
H	-2.938244	5.233516	2.502922
H	-4.218274	1.192560	3.276679
H	-4.071186	3.577526	3.980579
C	0.685692	2.099602	0.055065
H	0.030918	2.846649	0.496165
C	2.143686	2.288179	0.108973
C	2.493677	3.764059	0.123089
F	2.107919	4.351240	-1.011955
F	1.895325	4.415856	1.125857
F	3.806680	3.981343	0.248088
O	0.899603	-0.014363	-0.958433
C	0.226913	0.958879	-0.573323
H	-4.622236	0.736498	-0.660809
C	-4.762045	-2.303436	-1.639857
H	-5.615135	-1.625671	-1.678827
H	-5.944984	-4.086074	-1.815109
H	2.635036	1.848929	-0.769956
S	3.122296	1.559011	1.582785
C	2.470814	-0.123704	1.649419
N	3.158154	-1.159060	1.370209
N	1.203321	-0.189411	2.157934
H	0.792686	0.716010	2.366549
C	4.319548	-1.120114	0.615642
C	4.282185	-0.643509	-0.710024
C	5.519981	-1.630162	1.137907
C	5.428998	-0.655517	-1.489358
H	3.324985	-0.295571	-1.107007
C	6.672795	-1.636559	0.365228
H	5.534085	-2.011407	2.159350
C	6.611390	-1.147377	-0.937919
H	5.419817	-0.292825	-2.516206
H	7.615013	-2.016209	0.757690
N	7.825146	-1.159725	-1.758209
O	8.841402	-1.592706	-1.262112
O	7.748984	-0.736010	-2.890002
C	0.295941	-1.238243	2.078627
C	0.642016	-2.578832	1.833796
C	-1.059349	-0.893548	2.250430
C	-0.359873	-3.535407	1.706836
H	1.684958	-2.860816	1.727873
C	-2.056596	-1.842080	2.114033
H	-1.313571	0.144295	2.474483
C	-1.692919	-3.157434	1.826513
H	-0.110001	-4.575223	1.498610
H	-3.109106	-1.578451	2.218168
N	-2.745984	-4.148989	1.603236
O	-3.894411	-3.765522	1.612577
O	-2.413316	-5.297024	1.409142

CF3_Michael_Acceptor/pNO2_thiourea

Frequencies, energies and thermodynamic properties:

Lowest Vibrational Mode (1/cm) = 18.6815

2nd Lowest Vibrational Mode (1/cm) = 21.7203

E(RM062X) (a.u.) = -1418.04990065

Thermal correction to Enthalpy (a.u.) = 0.251465

Thermal correction to Gibbs Free Energy (a.u.) = 0.181252

Total Entropy (cal/Kmol) = 147.775

Esp(RM062X) (a.u.) = -1419.34300615

Optimised cartesian coordinates (Angstrom):

C	-0.010122	1.964661	0.081432
S	-0.923571	3.348093	-0.105177
N	1.353766	2.059251	0.123383
H	1.684195	3.016258	0.223140
N	-0.500866	0.708768	0.270880
H	0.140222	0.042540	0.699144
C	-1.817630	0.241178	0.178794
C	-2.757629	0.760615	-0.724929
C	-2.164941	-0.844466	0.999652
C	-4.030956	0.210625	-0.786694
H	-2.492443	1.585810	-1.380248
C	-3.433891	-1.399768	0.937124
H	-1.428459	-1.246755	1.697331
C	-4.353857	-0.856707	0.045689
H	-4.771858	0.598601	-1.484094
H	-3.715341	-2.237635	1.572957
C	2.349450	1.077612	0.045142
C	3.582038	1.360050	0.659970
C	2.187958	-0.124495	-0.666159
C	4.629343	0.455633	0.587476
H	3.706231	2.300119	1.199910
C	3.231832	-1.038536	-0.730380
H	1.261120	-0.339243	-1.196526
C	4.434327	-0.739726	-0.099629
H	5.586923	0.662590	1.063028
H	3.123355	-1.971243	-1.281951
N	5.535029	-1.708228	-0.170714
O	5.333037	-2.748914	-0.753086
O	6.581297	-1.410962	0.358233
N	-5.701693	-1.433283	-0.024638
O	-5.948306	-2.378536	0.689216
O	-6.490527	-0.932354	-0.792758

CF3_Michael_Acceptor/product

Frequencies, energies and thermodynamic properties:

Lowest Vibrational Mode (1/cm) = 19.1238

2nd Lowest Vibrational Mode (1/cm) = 22.6778

E(RM062X) (a.u.) = -1945.26747655

Thermal correction to Enthalpy (a.u.) = 0.307288

Thermal correction to Gibbs Free Energy (a.u.) = 0.220747

Total Entropy (cal/Kmol) = 182.142

Esp(RM062X) (a.u.) = -1947.17713409

Optimised cartesian coordinates (Angstrom):

C	0.198428	3.406049	-0.599559
C	0.078420	0.622002	-0.641485
C	-1.932075	2.076681	-1.057062
C	-1.073336	3.267513	-1.414322
H	-0.804175	3.182847	-2.478106
H	-1.708753	4.155132	-1.299960
N	-1.292894	0.877215	-0.709850
C	-2.159753	-0.227233	-0.383884
C	-2.488182	-0.452750	0.951322
C	-2.656782	-1.037356	-1.402710
C	-3.330073	-1.509419	1.280485
H	-2.084649	0.201107	1.725613
C	-3.500397	-2.095675	-1.085103

H -2.380965 -0.836602 -2.438578
C -3.817493 -2.308855 0.252375
H -3.608614 -1.714148 2.312750
H -3.907679 -2.749571 -1.854366
N -4.710612 -3.431994 0.594680
O -5.116450 -4.122134 -0.310283
O -4.984132 -3.596707 1.759952
O -3.133976 2.129106 -1.090590
S 1.261860 1.950377 -0.777346
N 0.472533 -0.573612 -0.499774
C 1.807153 -0.932470 -0.316536
C 2.498783 -1.581335 -1.352021
C 2.443272 -0.710294 0.917641
C 3.812721 -1.989474 -1.166277
H 1.990207 -1.761397 -2.299545
C 3.754327 -1.121234 1.110937
H 1.895795 -0.217474 1.722100
C 4.420037 -1.753082 0.063651
H 4.366917 -2.489583 -1.959133
H 4.263097 -0.958330 2.059918
N 5.807331 -2.188962 0.265267
O 6.314690 -1.976934 1.342908
O 6.367227 -2.735663 -0.657431
H 0.797963 4.243408 -0.984051
C -0.070275 3.717824 0.864223
F -0.776095 4.842281 0.978173
F 1.056552 3.873572 1.550032
F -0.772784 2.745631 1.459810

CF3_Michael_Acceptor/TS-acyl

Frequencies, energies and thermodynamic properties:

Lowest Vibrational Mode (1/cm) = -121.9738

2nd Lowest Vibrational Mode (1/cm) = 10.1528

E(RM062X) (a.u.) = -2572.61729427

Thermal correction to Enthalpy (a.u.) = 0.505164

Thermal correction to Gibbs Free Energy (a.u.) = 0.396543

Total Entropy (cal/Kmol) = 228.612

Esp(RM062X) (a.u.) = -2575.24147968

Optimised cartesian coordinates (Angstrom):

C 6.671002 -1.747644 0.267353
C 6.324437 -2.999860 -0.246781
C 4.983837 -3.345754 -0.428728
C 4.007431 -2.416839 -0.086080
C 4.355512 -1.164910 0.438062
C 5.693530 -0.814013 0.615240
H 7.722674 -1.489020 0.398820
H 7.104436 -3.714268 -0.512543
H 4.707149 -4.320805 -0.833020
H 5.970014 0.164510 1.008306
C 2.035838 -0.945700 0.405474
C 0.831255 1.026580 0.959631
C 1.988461 1.314763 1.941465
C 3.319295 0.958535 1.289643
H 4.123398 0.965454 2.039387
S 2.261298 -2.599300 -0.218009
N 3.240783 -0.383063 0.722211
N 0.897138 -0.361531 0.512450
C 1.976932 2.735196 2.549439
C 2.761892 3.777749 1.748513
C 2.491010 2.695628 3.989270
H 0.921575 3.054321 2.580039
H 2.486652 3.797089 0.684510
H 2.576318 4.780310 2.161463

H 3.845804 3.593317 1.818154
H 1.870135 2.042528 4.619898
H 3.525824 2.318115 4.025464
H 2.488721 3.701854 4.434023
H 1.835133 0.602251 2.768710
H -0.110457 1.124587 1.519091
C 0.765908 1.989858 -0.215705
C -0.077103 3.104828 -0.145326
C 1.539661 1.801750 -1.367394
C -0.128070 4.026521 -1.191226
H -0.705075 3.248143 0.736876
C 1.490053 2.722326 -2.414305
H 2.179517 0.921170 -1.465131
C 0.659378 3.840076 -2.327445
H -0.792405 4.889711 -1.120939
H 2.094396 2.557135 -3.307978
H 0.615913 4.556521 -3.149563
C -1.065066 -0.438798 -1.136102
H -1.376071 0.586532 -0.922510
C -0.893625 -0.864402 -2.382700
H -0.562563 -1.881448 -2.608310
C -1.079847 0.044390 -3.551608
F 0.072450 0.219924 -4.213024
F -1.952666 -0.456407 -4.433895
F -1.520272 1.255024 -3.204115
O -0.469124 -2.509642 -0.096755
C -0.745108 -1.341388 0.023931
H 3.586142 1.671441 0.493229
O -1.407306 -0.906091 1.232194
C -2.750906 -0.805923 1.134082
C -3.581394 -1.929947 1.182865
C -3.349139 0.449692 0.998270
C -4.965089 -1.795877 1.119299
C -4.729632 0.593740 0.936349
C -5.539632 -0.536173 0.985638
F -2.580770 1.530575 0.933094
F -5.273951 1.794013 0.815077
F -5.739641 -2.867674 1.179639
F -3.060253 -3.135911 1.320359
F -6.854558 -0.411662 0.919231

CF3_Michael_Acceptor/TS-cycl-R_rot
Frequencies, energies and thermodynamic properties:
Lowest Vibrational Mode (1/cm) = -107.2018
2nd Lowest Vibrational Mode (1/cm) = 12.1212
E(RM062X) (a.u.) = -3187.93331614
Thermal correction to Enthalpy (a.u.) = 0.680738
Thermal correction to Gibbs Free Energy (a.u.) = 0.555516
Total Entropy (cal/Kmol) = 263.552
Esp(RM062X) (a.u.) = -3190.89449964
Optimised cartesian coordinates (Angstrom):
C -0.167217 -1.461371 2.611883
C 1.337506 -1.394643 2.821634
H 1.618699 -2.308799 3.367011
H -0.386767 -2.432652 2.153195
H -0.653785 -1.464511 3.598063
N 0.619360 0.469257 0.727786
C 0.205944 1.696456 0.197015
C 0.190209 1.946068 -1.189862
C -0.232927 2.707592 1.073700
C -0.292181 3.146557 -1.689952
H 0.571078 1.187161 -1.870362
C -0.722495 3.911596 0.581693

H	-0.169019	2.537882	2.145361
C	-0.755134	4.109598	-0.795166
H	-0.314704	3.343242	-2.761048
H	-1.062904	4.699689	1.251747
N	-1.272974	5.376645	-1.319826
O	-1.684389	6.194325	-0.526974
O	-1.266287	5.539376	-2.519970
C	-0.874298	-0.321788	1.873031
O	-1.345259	0.607552	2.501502
C	1.808319	-0.280027	3.747109
F	1.116731	-0.316822	4.892443
F	1.683870	0.948330	3.253386
F	3.096866	-0.444347	4.061121
S	2.314908	-1.510662	1.301099
C	1.812455	-0.006385	0.411036
N	2.617969	0.448864	-0.497460
C	3.966895	0.225759	-0.578363
C	4.836495	0.358673	0.533565
C	4.540027	-0.057180	-1.840127
C	6.203525	0.191565	0.392962
H	4.416944	0.606644	1.509527
C	5.906023	-0.229461	-1.987255
H	3.876163	-0.134667	-2.701988
C	6.727431	-0.107694	-0.865983
H	6.873997	0.296604	1.244992
H	6.346754	-0.454478	-2.957530
N	8.167016	-0.284929	-1.014807
O	8.599759	-0.539739	-2.118804
O	8.861487	-0.170632	-0.027315
N	-1.690484	-0.808382	0.729171
C	-2.981882	-0.489120	0.678159
C	-1.169294	-1.800778	-0.227853
S	-3.713426	0.878029	1.484916
N	-3.863320	-1.191843	-0.048903
C	-2.180461	-2.961735	-0.300837
H	-0.242413	-2.188380	0.210291
C	-0.803326	-1.214762	-1.578517
C	-5.258770	0.474571	0.751000
C	-5.158786	-0.663959	-0.051830
C	-3.529897	-2.429976	-0.761278
C	-1.693559	-4.176395	-1.125324
H	-2.296047	-3.300611	0.742294
C	0.417177	-1.595180	-2.147280
C	-1.661815	-0.388387	-2.315182
C	-6.474086	1.139341	0.903049
C	-6.264011	-1.166477	-0.738955
H	-4.326743	-3.155304	-0.547613
H	-3.541694	-2.211778	-1.839215
C	-2.021019	-4.118458	-2.619715
C	-2.245244	-5.470535	-0.524951
H	-0.595462	-4.197601	-1.020270
C	0.767160	-1.179615	-3.431636
H	1.106809	-2.218077	-1.571338
C	-1.316086	0.023231	-3.602043
H	-2.605394	-0.035025	-1.895623
C	-7.580743	0.643138	0.218930
H	-6.552276	2.025181	1.534808
C	-7.474518	-0.494709	-0.591786
H	-6.185178	-2.051952	-1.369620
H	-1.715008	-3.174464	-3.092051
H	-1.501610	-4.936325	-3.139872
H	-3.099630	-4.257531	-2.794895
H	-1.922741	-5.600849	0.518441

H -3.347072 -5.471579 -0.543166
H -1.902123 -6.343240 -1.099371
C -0.103799 -0.374498 -4.166442
H 1.723479 -1.486605 -3.858410
H -1.995191 0.669023 -4.161358
H -8.541369 1.150223 0.315625
H -8.354646 -0.864183 -1.119446
H 0.165794 -0.049766 -5.172767

CF3_Michael_Acceptor/TS-cycl-R

Frequencies, energies and thermodynamic properties:

Lowest Vibrational Mode (1/cm) = -158.3451

2nd Lowest Vibrational Mode (1/cm) = 9.8201

E(RM062X) (a.u.) = -3187.93917543

Thermal correction to Enthalpy (a.u.) = 0.680413

Thermal correction to Gibbs Free Energy (a.u.) = 0.554712

Total Entropy (cal/Kmol) = 264.560

Esp(RM062X) (a.u.) = -3190.90456649

Optimised cartesian coordinates (Angstrom):

C 1.707617 -2.526199 -1.147645
C 2.243743 0.203164 -0.329952
C -0.305280 -1.093178 -1.077964
C 0.399807 -2.270189 -0.428837
H 0.567778 -2.147790 0.645961
H -0.268186 -3.135431 -0.578371
N 0.915592 0.307736 -0.432702
C 0.316150 1.546344 -0.199038
C -0.578447 2.058402 -1.156724
C 0.505867 2.259349 1.003014
C -1.311005 3.212935 -0.908054
H -0.687182 1.527262 -2.102828
C -0.205410 3.422236 1.253741
H 1.215405 1.885395 1.739941
C -1.121507 3.873014 0.302959
H -2.015771 3.606514 -1.639771
H -0.070812 3.977620 2.181056
N -1.907800 5.074722 0.591911
O -1.792095 5.576428 1.688413
O -2.639455 5.496686 -0.275280
O -0.378831 -1.011402 -2.297945
S 3.045543 -1.390616 -0.646440
N 3.033617 1.167575 0.008338
C 4.408889 1.155420 -0.040094
C 5.148499 1.361139 1.144693
C 5.115051 1.029048 -1.258935
C 6.532948 1.420881 1.123215
H 4.606018 1.472578 2.084211
C 6.498963 1.090254 -1.289692
H 4.554435 0.890235 -2.184202
C 7.194571 1.282238 -0.096056
H 7.107041 1.572829 2.036197
H 7.047156 0.995304 -2.226097
N 8.654157 1.347378 -0.124948
O 9.208557 1.219699 -1.194862
O 9.236843 1.525155 0.922744
H 1.552300 -2.458637 -2.233548
C 2.191646 -3.936003 -0.865573
F 3.390022 -4.166002 -1.404226
F 1.345616 -4.832825 -1.378769
F 2.287641 -4.187773 0.441475
N -1.518877 -0.683244 -0.331148
C -2.615350 -0.335434 -1.007776
C -1.532777 -0.695779 1.148290

S -2.778415 -0.367773 -2.752269
N -3.746827 0.051158 -0.400766
C -2.487106 0.408290 1.645938
H -0.517676 -0.406971 1.454594
C -1.829305 -2.075352 1.704230
C -4.461742 0.110701 -2.600958
C -4.820835 0.293336 -1.265377
C -3.866603 0.207479 1.048826
C -2.526033 0.579713 3.181089
H -2.086523 1.344565 1.225218
C -1.114475 -2.518918 2.821958
C -2.822736 -2.896933 1.161737
C -5.383894 0.310438 -3.626852
C -6.117166 0.667465 -0.908751
H -4.493709 1.088894 1.236043
H -4.376600 -0.679015 1.455965
C -3.530914 -0.323958 3.902970
C -2.818240 2.039621 3.533393
H -1.514297 0.338041 3.547928
C -1.408229 -3.747691 3.409758
H -0.318650 -1.892950 3.233450
C -3.116414 -4.128811 1.748426
H -3.374356 -2.591067 0.268982
C -6.677425 0.688959 -3.278080
H -5.099004 0.171627 -4.670844
C -7.037296 0.862405 -1.935026
H -6.405710 0.798038 0.134002
H -3.444081 -1.383495 3.626278
H -3.378798 -0.248058 4.989512
H -4.563847 -0.000241 3.698416
H -2.075566 2.723363 3.098161
H -3.810906 2.339910 3.160516
H -2.818687 2.182824 4.623768
C -2.415908 -4.553201 2.877369
H -0.843745 -4.080300 4.282371
H -3.894081 -4.761012 1.316783
H -7.418833 0.849269 -4.061590
H -8.057721 1.154413 -1.684069
H -2.646087 -5.517014 3.334306

CF3_Michael_Acceptor/TS-cycl-S_rot

Frequencies, energies and thermodynamic properties:

Lowest Vibrational Mode (1/cm) = -156.7463
2nd Lowest Vibrational Mode (1/cm) = 8.1342
E(RM062X) (a.u.) = -3187.93750850
Thermal correction to Enthalpy (a.u.) = 0.680296
Thermal correction to Gibbs Free Energy (a.u.) = 0.553889
Total Entropy (cal/Kmol) = 266.046
Esp(RM062X) (a.u.) = -3190.89948963

Optimised cartesian coordinates (Angstrom):

C 1.652237 -2.328958 -1.753183
C 2.128018 -0.164125 -0.016573
C -0.452022 -0.928146 -1.318490
C 0.188999 -2.305513 -1.335471
H 0.133958 -2.825313 -0.375108
H -0.408222 -2.881925 -2.059465
N 0.849102 0.074099 -0.296316
C 0.363312 1.370375 -0.050457
C 0.029146 2.207057 -1.126289
C 0.157863 1.824479 1.265126
C -0.546932 3.453459 -0.896838
H 0.223884 1.861852 -2.140000
C -0.408601 3.067102 1.506705

H	0.455389	1.185523	2.098078
C	-0.765898	3.857778	0.414761
H	-0.819891	4.111019	-1.721053
H	-0.578080	3.429170	2.519822
N	-1.380331	5.168970	0.661637
O	-1.571779	5.495642	1.811270
O	-1.666228	5.850054	-0.296446
O	-0.638142	-0.336664	-2.375873
S	2.777340	-1.800964	-0.433491
N	2.911099	0.657555	0.608965
C	4.281700	0.603845	0.647852
C	4.942778	0.777888	1.884952
C	5.073283	0.476817	-0.520384
C	6.325476	0.800432	1.963702
H	4.335961	0.895477	2.783502
C	6.455786	0.501256	-0.449848
H	4.578502	0.369424	-1.486590
C	7.070776	0.657838	0.793311
H	6.836142	0.927576	2.917357
H	7.067185	0.407640	-1.346412
N	8.527530	0.683871	0.868937
O	9.154722	0.550536	-0.160054
O	9.039569	0.836350	1.957469
H	1.925520	-3.382037	-1.914138
C	2.003162	-1.648064	-3.070601
F	1.129679	-1.969811	-4.025313
F	3.207373	-2.063345	-3.487807
F	2.069516	-0.320054	-2.999795
N	-1.573120	-0.816667	-0.334137
C	-2.649107	-0.097204	-0.650466
C	-1.483796	-1.473033	0.985350
S	-2.932266	0.675269	-2.198684
N	-3.675929	0.077662	0.197594
C	-2.239414	-0.623238	2.023855
H	-0.418832	-1.439210	1.255581
C	-1.936161	-2.920768	0.944312
C	-4.520973	1.186607	-1.648920
C	-4.761465	0.780580	-0.336051
C	-3.673153	-0.416145	1.574718
C	-2.130555	-1.146064	3.474247
H	-1.754013	0.363022	1.993523
C	-1.228530	-3.870705	1.690046
C	-3.066529	-3.324774	0.226392
C	-5.475773	1.910318	-2.361302
C	-5.966839	1.071211	0.303869
H	-4.163185	0.342345	2.199187
H	-4.266585	-1.341866	1.618435
C	-3.240926	-2.116248	3.886449
C	-2.087295	0.035705	4.443944
H	-1.167046	-1.676794	3.548866
C	-1.661340	-5.194126	1.746079
H	-0.326401	-3.567745	2.228016
C	-3.499378	-4.650687	0.280344
H	-3.620904	-2.611821	-0.389184
C	-6.679374	2.207885	-1.727421
H	-5.284474	2.231167	-3.386377
C	-6.920167	1.790741	-0.411746
H	-6.160986	0.744454	1.325338
H	-3.366172	-2.951057	3.182439
H	-3.011416	-2.542294	4.873977
H	-4.206795	-1.594061	3.974683
H	-1.228299	0.692387	4.240707
H	-3.004180	0.641529	4.362869

H -2.010134 -0.314474 5.483594

C -2.803528 -5.585793 1.046056

H -1.101145 -5.923776 2.333156

H -4.383805 -4.953004 -0.282885

H -7.443494 2.771070 -2.264415

H -7.871462 2.031118 0.064524

H -3.143573 -6.621818 1.087126

CF3_Michael_Acceptor/TS-cycl-S

Frequencies, energies and thermodynamic properties:

Lowest Vibrational Mode (1/cm) = -147.8870

2nd Lowest Vibrational Mode (1/cm) = 11.6807

E(RM062X) (a.u.) = -3187.93294578

Thermal correction to Enthalpy (a.u.) = 0.680546

Thermal correction to Gibbs Free Energy (a.u.) = 0.555204

Total Entropy (cal/Kmol) = 263.805

Esp(RM062X) (a.u.) = -3190.89656748

Optimised cartesian coordinates (Angstrom):

C 0.106479 -2.100773 -2.001132

C -1.338517 -1.890280 -2.404411

H -1.424812 -1.064198 -3.125643

H 0.156583 -2.838408 -1.190319

H 0.668925 -2.505225 -2.854976

N -0.539842 0.417566 -1.117527

C -0.029909 1.724556 -1.046786

C -0.005876 2.460745 0.153375

C 0.506133 2.305439 -2.211269

C 0.571333 3.722068 0.199692

H -0.455099 2.038203 1.049212

C 1.089170 3.566381 -2.174534

H 0.449359 1.755917 -3.147446

C 1.121281 4.252657 -0.964930

H 0.599525 4.295166 1.125420

H 1.504519 4.023778 -3.071183

N 1.737083 5.583502 -0.917849

O 2.199614 6.031396 -1.943040

O 1.753850 6.163144 0.144985

C 0.855306 -0.797423 -1.680733

O 1.469265 -0.239165 -2.580519

C -1.896586 -3.122644 -3.089812

F -3.161813 -2.954318 -3.468592

F -1.857903 -4.194316 -2.290895

F -1.189580 -3.418118 -4.182165

S -2.382533 -1.509452 -0.960894

C -1.761887 0.163995 -0.639602

N -2.501192 0.975160 0.038872

C -3.849122 0.846244 0.278629

C -4.794479 0.644981 -0.754825

C -4.326202 1.012938 1.597420

C -6.150692 0.589261 -0.477648

H -4.446779 0.543060 -1.783022

C -5.680601 0.953050 1.883738

H -3.600599 1.195336 2.390613

C -6.580648 0.737347 0.841025

H -6.881892 0.437755 -1.270539

H -6.050219 1.074580 2.901018

N -8.010124 0.677186 1.135169

O -8.360262 0.812744 2.287617

O -8.774111 0.493380 0.212548

N 1.596928 -0.894350 -0.379741

C 2.910784 -0.692710 -0.376687

C 0.959436 -1.424472 0.837507

S 3.794686 0.205794 -1.590425

N 3.702651 -1.139635 0.611551
C 1.854066 -2.552510 1.387974
H 0.012416 -1.874244 0.518013
C 0.619053 -0.357775 1.860067
C 5.271891 -0.007082 -0.660366
C 5.041439 -0.749709 0.499740
C 3.232120 -1.992730 1.707372
C 1.232866 -3.339456 2.565478
H 1.968479 -3.256296 0.546862
C -0.633799 -0.416413 2.480216
C 1.531324 0.622660 2.271604
C 6.548739 0.457110 -0.970476
C 6.073099 -1.037658 1.393674
H 3.960372 -2.806301 1.829158
H 3.229189 -1.390551 2.627918
C 1.530721 -2.766924 3.953874
C 1.671186 -4.803374 2.504333
H 0.140762 -3.309486 2.413132
C -0.960797 0.466769 3.509382
H -1.362835 -1.162062 2.150928
C 1.204164 1.508456 3.297378
H 2.504614 0.726726 1.788829
C 7.583146 0.172594 -0.082362
H 6.730070 1.031669 -1.879949
C 7.345689 -0.564434 1.085052
H 5.890652 -1.610120 2.303088
H 1.304265 -1.694403 4.035657
H 0.926190 -3.294952 4.705565
H 2.587951 -2.916840 4.224915
H 1.364448 -5.276791 1.560195
H 2.766670 -4.888095 2.587719
H 1.229338 -5.376656 3.332311
C -0.040213 1.429585 3.923722
H -1.938918 0.401648 3.988990
H 1.924434 2.269288 3.602877
H 8.589675 0.531915 -0.299490
H 8.170249 -0.773422 1.767666
H -0.294270 2.121507 4.728360

CF3_Michael_Acceptor/TS-MA-R
Frequencies, energies and thermodynamic properties:
Lowest Vibrational Mode (1/cm) = -184.9680
2nd Lowest Vibrational Mode (1/cm) = 4.5828
E(RM062X) (a.u.) = -3187.94763983
Thermal correction to Enthalpy (a.u.) = 0.680837
Thermal correction to Gibbs Free Energy (a.u.) = 0.554671
Total Entropy (cal/Kmol) = 265.539
Esp(RM062X) (a.u.) = -3190.90852411
Optimised cartesian coordinates (Angstrom):
C -6.259958 1.915444 -0.150895
C -6.694052 0.747207 0.489989
C -5.781696 -0.132062 1.064980
C -4.425367 0.175181 0.962355
C -3.995925 1.327500 0.301765
H -7.760193 0.524287 0.543591
H -6.113839 -1.040997 1.568994
C -1.966709 0.412469 0.899772
C 0.209002 1.406217 0.400107
C -0.515613 2.041055 -0.797441
C -1.897744 2.514267 -0.391653
H -2.482579 2.794087 -1.277621
S -3.070497 -0.756480 1.574161
N -2.599442 1.417523 0.276649

N	-0.630780	0.338110	0.978540
C	0.315167	3.131977	-1.511390
C	0.108548	4.545724	-0.961797
H	-0.897355	4.921055	-1.208127
H	0.240130	4.605902	0.127793
H	0.833194	5.230749	-1.425252
H	-0.658629	1.212220	-1.512052
H	1.081225	0.901532	-0.026003
C	0.683971	2.377974	1.459038
C	2.006643	2.831314	1.415814
C	-0.176793	2.875070	2.443409
C	2.454274	3.793715	2.319269
H	2.690086	2.423882	0.665386
C	0.271184	3.836604	3.349268
H	-1.205487	2.512325	2.516432
C	1.584266	4.303791	3.283586
H	3.487850	4.140666	2.274429
H	-0.408771	4.219284	4.112202
H	1.933485	5.055694	3.993160
C	1.403948	-0.856679	1.672245
H	2.040631	-0.076448	1.266219
C	1.990754	-2.037930	2.132823
C	3.416755	-1.937475	2.605087
F	4.198728	-1.282290	1.744380
F	3.469957	-1.267056	3.764736
F	3.976600	-3.128184	2.831970
O	-0.777320	-1.666884	2.057337
C	-0.014651	-0.811661	1.619472
H	-1.861312	3.376375	0.291565
C	-4.907781	2.226003	-0.253125
H	-4.579527	3.136407	-0.753888
H	-6.994104	2.594722	-0.585819
H	1.383007	-2.691506	2.762137
S	2.307693	-3.596230	0.518241
C	1.209625	-2.789346	-0.602726
N	-0.078098	-3.286796	-0.517411
H	-0.159465	-4.073435	0.119142
C	-1.286154	-2.778861	-0.960085
C	-1.438251	-1.591835	-1.706007
C	-2.443195	-3.503698	-0.593065
C	-2.710110	-1.121894	-2.013109
H	-0.556919	-1.049027	-2.031300
C	-3.706722	-3.047140	-0.916528
H	-2.330823	-4.430550	-0.027400
C	-3.829699	-1.842569	-1.610223
H	-2.838213	-0.193544	-2.570594
H	-4.597794	-3.598850	-0.619957
N	-5.162083	-1.311993	-1.892294
O	-5.240297	-0.225726	-2.422683
O	-6.118954	-1.985178	-1.577568
N	1.438368	-1.779225	-1.365739
C	2.646658	-1.148208	-1.610850
C	2.554349	0.191891	-2.052471
C	3.929732	-1.733629	-1.539260
C	3.678887	0.945979	-2.344084
H	1.560269	0.623403	-2.180155
C	5.063242	-0.990030	-1.841013
H	4.037458	-2.774650	-1.248041
C	4.929288	0.342426	-2.222386
H	3.600119	1.981081	-2.675214
H	6.054753	-1.437248	-1.783496
N	6.128911	1.123463	-2.529444
O	5.982159	2.280302	-2.858002

O 7.203561 0.573766 -2.438562
H 1.375875 2.863099 -1.368574
C 0.027465 3.107229 -3.012564
H -1.039425 3.298199 -3.211459
H 0.606548 3.884003 -3.532976
H 0.285765 2.134485 -3.457445

CF3_Michael_Acceptor/TS-MA-S

Frequencies, energies and thermodynamic properties:

Lowest Vibrational Mode (1/cm) = -133.2872
2nd Lowest Vibrational Mode (1/cm) = 19.7386
E(RM062X) (a.u.) = -3187.94750675
Thermal correction to Enthalpy (a.u.) = 0.680593
Thermal correction to Gibbs Free Energy (a.u.) = 0.557801
Total Entropy (cal/Kmol) = 258.438
Esp(RM062X) (a.u.) = -3190.90928200

Optimised cartesian coordinates (Angstrom):

C 6.602875 0.840039 -0.451944
C 6.748102 -0.468436 0.029498
C 5.696515 -1.114920 0.671372
C 4.490800 -0.426113 0.800889
C 4.354067 0.880704 0.329827
H 7.694900 -0.991964 -0.106558
H 5.803413 -2.135871 1.041456
C 2.222061 0.498806 1.105459
C 0.388565 2.120461 0.975261
C 1.524990 3.141148 1.187851
C 2.708756 2.777673 0.308486
H 3.584389 3.388668 0.565647
S 3.000891 -1.002060 1.524151
N 3.066715 1.379811 0.556614
N 0.914245 0.764109 1.270226
C 1.091347 4.616840 1.021692
C 1.262249 5.173617 -0.394759
C 1.854092 5.492835 2.016306
H 0.021904 4.666059 1.283123
H 0.801406 6.170138 -0.456463
H 2.328601 5.293264 -0.643754
H 0.798574 4.543126 -1.166510
H 1.563536 6.547805 1.907017
H 1.655695 5.189339 3.054467
H 2.940375 5.426324 1.843143
H 1.843874 3.006500 2.234469
H -0.369733 2.317740 1.743886
C -0.256736 2.232169 -0.388371
C -1.392789 3.036930 -0.533202
C 0.313134 1.646371 -1.523342
C -1.922978 3.290353 -1.797715
H -1.858218 3.476020 0.352693
C -0.227035 1.885417 -2.787974
H 1.185626 0.995926 -1.426720
C -1.337901 2.718435 -2.928843
H -2.801648 3.929809 -1.898740
H 0.223575 1.418863 -3.665802
H -1.756483 2.908921 -3.918396
C -1.356685 -0.050597 1.781327
H -1.801748 0.684825 1.111894
C -2.141588 -0.914120 2.521826
C -3.576111 -0.530052 2.756669
F -3.631701 0.533985 3.578478
F -4.209354 -0.177453 1.638019
F -4.286496 -1.491123 3.345930
O 0.608850 -1.219648 2.351265

C 0.060544 -0.253458 1.845478
H 2.493561 2.908837 -0.761947
C 5.407840 1.537206 -0.306123
H 5.295731 2.550668 -0.691020
H 7.439715 1.321972 -0.958758
H -1.683118 -1.453268 3.351376
S -2.590511 -3.047323 1.494740
C -1.562590 -2.661532 0.131031
N -1.825583 -1.838590 -0.823519
N -0.311247 -3.256281 0.188421
H -0.211964 -3.901275 0.965739
C -3.074879 -1.293128 -1.054507
C -4.261331 -2.056901 -1.083936
C -3.149317 0.077610 -1.365476
C -5.478166 -1.459688 -1.373191
H -4.216863 -3.124038 -0.872726
C -4.362145 0.687605 -1.643396
H -2.224523 0.652422 -1.370407
C -5.517827 -0.090832 -1.638377
H -6.399323 -2.040379 -1.394588
H -4.422936 1.751825 -1.867985
N -6.802790 0.541432 -1.930226
O -6.811969 1.730891 -2.164183
O -7.794769 -0.154242 -1.923745
C 0.857061 -2.970481 -0.487754
C 1.032085 -1.866796 -1.349762
C 1.958602 -3.822266 -0.241583
C 2.286249 -1.595490 -1.881543
H 0.186014 -1.224799 -1.576093
C 3.200758 -3.564668 -0.791422
H 1.821998 -4.687409 0.410079
C 3.359139 -2.434601 -1.594655
H 2.442238 -0.726586 -2.521901
H 4.050919 -4.215927 -0.591978
N 4.684532 -2.098291 -2.104319
O 5.578197 -2.899787 -1.936305
O 4.827289 -1.028746 -2.655743

CF3_Michael_Acceptor/TS-turn-R_rot
Frequencies, energies and thermodynamic properties:
Lowest Vibrational Mode (1/cm) = -158.3844
2nd Lowest Vibrational Mode (1/cm) = 11.5905
E(RM062X) (a.u.) = -3187.93994850
Thermal correction to Enthalpy (a.u.) = 0.680072
Thermal correction to Gibbs Free Energy (a.u.) = 0.554449
Total Entropy (cal/Kmol) = 264.396
Esp(RM062X) (a.u.) = -3190.90144270
Optimised cartesian coordinates (Angstrom):
C -0.417053 -0.778291 2.647146
C 0.993656 -0.779774 3.201651
H 1.103253 -1.611794 3.913025
H -0.598578 -1.783791 2.249605
H -1.143636 -0.597317 3.450089
N 0.456555 0.680928 0.725989
C 0.297818 1.887907 -0.033388
C 0.321577 1.862293 -1.431349
C 0.148908 3.105356 0.643239
C 0.161502 3.037931 -2.156971
H 0.488145 0.923514 -1.953442
C -0.012757 4.286352 -0.071798
H 0.157107 3.113124 1.729870
C -0.011625 4.228953 -1.461551
H 0.174270 3.034454 -3.245746

H	-0.131898	5.242270	0.435394
N	-0.184688	5.476298	-2.223730
O	-0.323813	6.503673	-1.601146
O	-0.179414	5.405105	-3.431180
C	-0.702533	0.344138	1.626626
O	-1.346101	1.319338	2.043783
C	1.388922	0.462692	3.985442
F	0.474454	0.738580	4.915964
F	1.528712	1.550906	3.225223
F	2.552890	0.275853	4.611302
S	2.171886	-1.144554	1.878023
C	1.676362	0.056958	0.635416
N	2.487679	0.308901	-0.316550
C	3.807041	-0.108382	-0.373462
C	4.769552	0.384383	0.531296
C	4.214177	-0.978399	-1.401768
C	6.098522	0.005543	0.420521
H	4.456817	1.072336	1.318164
C	5.541485	-1.366482	-1.514914
H	3.468907	-1.337622	-2.112257
C	6.466441	-0.869835	-0.599965
H	6.851775	0.379973	1.112323
H	5.868045	-2.043253	-2.303061
N	7.869439	-1.274446	-0.718403
O	8.165823	-2.036636	-1.611400
O	8.658103	-0.826766	0.083580
N	-1.733936	-0.546425	0.485600
C	-3.018808	-0.368411	0.527619
C	-1.182929	-1.676876	-0.257236
S	-3.822823	1.001578	1.314134
N	-3.920366	-1.206105	-0.050251
C	-2.145832	-2.877826	-0.155104
H	-0.251410	-1.963579	0.255150
C	-0.802535	-1.303589	-1.675897
C	-5.381768	0.399790	0.756108
C	-5.249815	-0.792033	0.036330
C	-3.521735	-2.470676	-0.664444
C	-1.618980	-4.179177	-0.800361
H	-2.243296	-3.065111	0.927908
C	0.430092	-1.729875	-2.181840
C	-1.658260	-0.566575	-2.504007
C	-6.633374	0.970792	0.966377
C	-6.364539	-1.434920	-0.500613
H	-4.268497	-3.228560	-0.388961
H	-3.535611	-2.358290	-1.759538
C	-1.967853	-4.348338	-2.281438
C	-2.112074	-5.393509	-0.011897
H	-0.519747	-4.147112	-0.712657
C	0.799245	-1.436338	-3.495046
H	1.111925	-2.288568	-1.534526
C	-1.291763	-0.274518	-3.817380
H	-2.611230	-0.189988	-2.125696
C	-7.752979	0.331090	0.433198
H	-6.734254	1.898861	1.531383
C	-7.616790	-0.856785	-0.291840
H	-6.262936	-2.358869	-1.070003
H	-1.696682	-3.473870	-2.889507
H	-1.433440	-5.218913	-2.689105
H	-3.044918	-4.537727	-2.414295
H	-1.775718	-5.358430	1.034684
H	-3.213141	-5.440072	-0.013804
H	-1.739423	-6.326934	-0.458655
C	-0.062570	-0.708747	-4.316732

H 1.765272 -1.771505 -3.877290
H -1.964897 0.306387 -4.450249
H -8.741959 0.765952 0.582718
H -8.501680 -1.342616 -0.705276
H 0.225996 -0.474087 -5.342653

CF3_Michael_Acceptor/TS-turn-R

Frequencies, energies and thermodynamic properties:

Lowest Vibrational Mode (1/cm) = -113.4847

2nd Lowest Vibrational Mode (1/cm) = 10.1204

E(RM062X) (a.u.) = -3187.94123551

Thermal correction to Enthalpy (a.u.) = 0.680211

Thermal correction to Gibbs Free Energy (a.u.) = 0.552895

Total Entropy (cal/Kmol) = 267.959

Esp(RM062X) (a.u.) = -3190.90569267

Optimised cartesian coordinates (Angstrom):

C 1.698931 -2.592390 -1.436692
C 2.194302 0.157439 -0.702815
C 0.008278 -0.815068 -1.387137
C 0.386218 -2.167403 -0.811602
H 0.476025 -2.137625 0.281811
H -0.401879 -2.877146 -1.096434
N 0.822239 0.278471 -0.783732
C 0.232232 1.561411 -0.600508
C -0.680029 2.065085 -1.536663
C 0.507482 2.290354 0.567844
C -1.340786 3.265170 -1.291682
H -0.878989 1.500722 -2.445770
C -0.136393 3.494197 0.812150
H 1.219898 1.902517 1.293850
C -1.063544 3.956171 -0.118284
H -2.062829 3.663946 -2.002769
H 0.061597 4.065112 1.718137
N -1.781345 5.207102 0.160147
O -1.630361 5.711714 1.250399
O -2.488503 5.659176 -0.709841
O -0.357909 -0.703008 -2.545539
S 3.028801 -1.393048 -1.073776
N 2.930651 1.155271 -0.418183
C 4.310606 1.114245 -0.278389
C 4.906916 0.560406 0.870644
C 5.125633 1.696411 -1.266154
C 6.285477 0.578476 1.025177
H 4.274015 0.119530 1.642042
C 6.505164 1.713830 -1.120205
H 4.655091 2.133674 -2.147210
C 7.065919 1.154076 0.025395
H 6.761036 0.153854 1.908065
H 7.148311 2.155907 -1.879781
N 8.522945 1.175857 0.187415
O 9.183051 1.678845 -0.693585
O 8.987720 0.689702 1.193875
H 1.595382 -2.688440 -2.526911
C 2.140612 -3.948014 -0.917507
F 3.340815 -4.285392 -1.393065
F 1.277099 -4.891120 -1.293762
F 2.215426 -3.981261 0.412858
N -1.580266 -0.568596 -0.244131
C -2.739018 -0.447745 -0.793775
C -1.456110 -0.397185 1.201336
S -3.060012 -0.694555 -2.527219
N -3.890704 -0.126895 -0.133524
C -2.474750 0.662770 1.677370

H -0.452396 0.014491 1.383272
C -1.527119 -1.722532 1.939986
C -4.778797 -0.431112 -2.253498
C -5.045578 -0.138565 -0.910627
C -3.880541 0.233454 1.280468
C -2.360798 1.066784 3.161809
H -2.231929 1.558280 1.078626
C -0.635838 -1.968696 2.990846
C -2.476050 -2.699405 1.616914
C -5.803628 -0.479739 -3.192566
C -6.349427 0.097815 -0.476683
H -4.586507 1.063816 1.424224
H -4.232263 -0.623109 1.877377
C -3.147850 0.177453 4.128869
C -2.792445 2.523630 3.343741
H -1.290097 0.996078 3.421841
C -0.707945 -3.152531 3.724177
H 0.123427 -1.221862 3.237027
C -2.550246 -3.884267 2.349839
H -3.164830 -2.545357 0.782779
C -7.109880 -0.238784 -2.763018
H -5.590748 -0.703371 -4.239054
C -7.375911 0.044673 -1.420480
H -6.562588 0.312938 0.570507
H -2.944046 -0.893815 3.993457
H -2.892494 0.439413 5.166259
H -4.231696 0.336307 4.012458
H -2.199526 3.209504 2.720181
H -3.851838 2.653155 3.069161
H -2.682889 2.835994 4.392812
C -1.670779 -4.111795 3.408793
H -0.005769 -3.328674 4.540936
H -3.296548 -4.636168 2.087525
H -7.927600 -0.275653 -3.483748
H -8.402489 0.226263 -1.099190
H -1.727989 -5.040307 3.979281

CF3_Michael_Acceptor/TS-turn-S_rot
Frequencies, energies and thermodynamic properties:
Lowest Vibrational Mode (1/cm) = -166.8112
2nd Lowest Vibrational Mode (1/cm) = 7.5736
E(RM062X) (a.u.) = -3187.94087403
Thermal correction to Enthalpy (a.u.) = 0.680112
Thermal correction to Gibbs Free Energy (a.u.) = 0.553433
Total Entropy (cal/Kmol) = 266.618
Esp(RM062X) (a.u.) = -3190.90329525
Optimised cartesian coordinates (Angstrom):
C 1.623409 -1.897201 -2.404160
C 2.001606 0.067820 -0.447135
C -0.265490 -0.449607 -1.575146
C 0.217651 -1.873448 -1.830060
H 0.235812 -2.493972 -0.928520
H -0.500182 -2.302762 -2.541166
N 0.690294 0.350450 -0.737521
C 0.188957 1.619521 -0.311203
C -0.167026 2.583877 -1.258333
C 0.030585 1.877800 1.055733
C -0.712543 3.795491 -0.841228
H -0.021967 2.369417 -2.314205
C -0.507720 3.083894 1.483142
H 0.341018 1.125630 1.781399
C -0.876836 4.019532 0.519657
H -1.002426 4.561402 -1.558672

H	-0.644970	3.304174	2.540725
N	-1.455380	5.299140	0.963038
O	-1.578576	5.478810	2.152627
O	-1.776192	6.097501	0.114026
O	-0.816434	0.151326	-2.503910
S	2.810241	-1.378469	-1.137420
N	2.677121	0.817359	0.335906
C	4.039298	0.695647	0.563422
C	4.501763	0.280995	1.825903
C	4.975070	1.052413	-0.428271
C	5.861829	0.207888	2.091226
H	3.773708	0.018627	2.594179
C	6.335540	0.985837	-0.169205
H	4.615625	1.386417	-1.402588
C	6.761007	0.561308	1.088203
H	6.232489	-0.116528	3.062520
H	7.068619	1.259273	-0.926728
N	8.198138	0.488544	1.364870
O	8.962219	0.794269	0.477098
O	8.545348	0.125476	2.466513
H	1.900919	-2.933569	-2.644827
C	1.829623	-1.112368	-3.692703
F	0.879868	-1.398900	-4.581462
F	3.003183	-1.434667	-4.247300
F	1.841049	0.208157	-3.512799
N	-1.537867	-0.740219	-0.295145
C	-2.728485	-0.254866	-0.466439
C	-1.189550	-1.397546	0.963728
S	-3.283192	0.588266	-1.929084
N	-3.724336	-0.327639	0.459572
C	-2.017090	-0.794227	2.119724
H	-0.132129	-1.149175	1.153735
C	-1.300263	-2.910627	0.883212
C	-4.889829	0.747655	-1.227226
C	-4.950456	0.201352	0.058165
C	-3.498325	-0.888230	1.787874
C	-1.668896	-1.348218	3.519385
H	-1.767452	0.278365	2.119357
C	-0.319987	-3.704207	1.490246
C	-2.381773	-3.534346	0.251565
C	-6.011188	1.331906	-1.808680
C	-6.138110	0.216852	0.789015
H	-4.084918	-0.300591	2.507506
H	-3.864489	-1.926232	1.813828
C	-2.501958	-2.558497	3.949571
C	-1.790030	-0.237385	4.563784
H	-0.611169	-1.658956	3.483397
C	-0.434222	-5.093788	1.497073
H	0.544580	-3.225960	1.958082
C	-2.496665	-4.924992	0.256493
H	-3.142762	-2.937425	-0.257095
C	-7.200960	1.355415	-1.080149
H	-5.959229	1.759451	-2.811377
C	-7.260478	0.802312	0.202715
H	-6.192622	-0.219178	1.786416
H	-2.501803	-3.366154	3.203996
H	-2.103012	-2.968938	4.888834
H	-3.547461	-2.268291	4.139819
H	-1.113965	0.601465	4.339516
H	-2.818362	0.157068	4.597792
H	-1.544177	-0.613805	5.567654
C	-1.528082	-5.707751	0.885652
H	0.337184	-5.699279	1.976096

H -3.346259 -5.398975 -0.238125
H -8.091055 1.807751 -1.519078
H -8.198972 0.824149 0.758370
H -1.619178 -6.795200 0.888110

CF3_Michael_Acceptor/TS-turn-S
Frequencies, energies and thermodynamic properties:
Lowest Vibrational Mode (1/cm) = -180.4842
2nd Lowest Vibrational Mode (1/cm) = 9.2011
E(RM062X) (a.u.) = -3187.93662572
Thermal correction to Enthalpy (a.u.) = 0.680137
Thermal correction to Gibbs Free Energy (a.u.) = 0.553740
Total Entropy (cal/Kmol) = 266.025
Esp(RM062X) (a.u.) = -3190.90111371
Optimised cartesian coordinates (Angstrom):
C 0.330726 -1.784923 -2.028965
C -1.064152 -1.884935 -2.607621
H -1.223727 -1.163438 -3.423080
H 0.404250 -2.467007 -1.173204
H 1.079239 -2.078643 -2.777269
N -0.454176 0.481155 -1.147783
C -0.199441 1.888932 -1.038252
C -0.208383 2.527111 0.206274
C 0.046756 2.627828 -2.202167
C 0.055967 3.889595 0.296883
H -0.442851 1.961437 1.104190
C 0.313450 3.989572 -2.123772
H 0.032728 2.125137 -3.165860
C 0.320181 4.594486 -0.871441
H 0.055726 4.400730 1.258318
H 0.508976 4.580329 -3.017043
N 0.605670 6.035938 -0.781479
O 0.837219 6.630201 -1.808869
O 0.594293 6.545790 0.315073
C 0.687304 -0.321964 -1.706687
O 1.402822 0.275940 -2.518440
C -1.327312 -3.258533 -3.192795
F -2.577446 -3.376613 -3.639396
F -1.131547 -4.228407 -2.296215
F -0.508719 -3.490878 -4.217826
S -2.271121 -1.576448 -1.280000
C -1.693101 0.036822 -0.746881
N -2.461569 0.751440 -0.022455
C -3.795465 0.478543 0.237472
C -4.766620 0.539701 -0.782433
C -4.204356 0.215257 1.557651
C -6.105597 0.324718 -0.493955
H -4.455477 0.766286 -1.802895
C -5.541765 -0.007556 1.852873
H -3.453033 0.198185 2.347142
C -6.474961 0.047888 0.820998
H -6.865294 0.370634 -1.273004
H -5.869031 -0.217984 2.870015
N -7.888946 -0.182823 1.128575
O -8.186576 -0.424623 2.276936
O -8.684172 -0.120598 0.218021
N 1.661511 -0.581241 -0.205216
C 2.953740 -0.503486 -0.279491
C 1.045723 -1.184729 0.973688
S 3.843659 0.298486 -1.587747
N 3.803060 -1.003641 0.658386
C 1.934383 -2.336796 1.487638
H 0.095887 -1.632163 0.641738

C 0.698839 -0.156527 2.031736
C 5.359711 -0.026246 -0.751791
C 5.152839 -0.731287 0.438438
C 3.330163 -1.805541 1.784484
C 1.327071 -3.140076 2.659955
H 2.022987 -3.024227 0.629281
C -0.547597 -0.224610 2.662896
C 1.601022 0.842639 2.417471
C 6.643206 0.328162 -1.156517
C 6.222921 -1.086516 1.258723
H 4.033524 -2.638599 1.924650
H 3.346658 -1.187680 2.695624
C 1.669971 -2.602098 4.051792
C 1.737075 -4.609885 2.557803
H 0.231773 -3.089661 2.537644
C -0.884916 0.680694 3.669836
H -1.263964 -0.990690 2.353422
C 1.265613 1.748166 3.423383
H 2.568466 0.939822 1.920116
C 7.718387 -0.027022 -0.340917
H 6.803553 0.874241 -2.087606
C 7.507424 -0.724770 0.852212
H 6.062149 -1.625550 2.192398
H 1.458809 -1.528925 4.161566
H 1.081570 -3.138892 4.810337
H 2.732959 -2.768171 4.288448
H 1.399459 -5.056775 1.611182
H 2.832630 -4.715552 2.611759
H 1.305605 -5.194451 3.383631
C 0.022048 1.669483 4.052885
H -1.861200 0.617679 4.154374
H 1.975609 2.525994 3.709653
H 8.731358 0.246724 -0.638390
H 8.358465 -0.991526 1.480371
H -0.241671 2.381150 4.836965

CF3_Michael_Acceptor_TS-MA-conf/TS-MA-R-iii

Frequencies, energies and thermodynamic properties:

Lowest Vibrational Mode (1/cm) = -50.7313

2nd Lowest Vibrational Mode (1/cm) = 10.3495

E(RM062X) (a.u.) = -3187.92983433

Thermal correction to Enthalpy (a.u.) = 0.681389

Thermal correction to Gibbs Free Energy (a.u.) = 0.549992

Total Entropy (cal/Kmol) = 276.550

Esp(RM062X) (a.u.) = -3190.89913858

Optimised cartesian coordinates (Angstrom):

C 7.257155 2.834692 -1.877399
C 6.371814 3.919596 -1.969072
C 5.013956 3.756677 -1.721569
C 4.563991 2.479975 -1.382794
C 5.444298 1.400423 -1.301472
H 6.753220 4.905954 -2.235087
H 4.322231 4.597473 -1.787406
C 3.472483 0.358948 -0.745675
C 3.272805 -2.019288 -0.127926
C 4.497132 -2.191191 -1.041429
C 5.484054 -1.062948 -0.820425
H 6.272070 -1.088695 -1.583636
S 2.926016 1.992470 -1.003247
N 4.782284 0.217106 -0.953858
N 2.685955 -0.669880 -0.347602
C 5.154352 -3.587266 -0.939708
C 6.238528 -3.698658 0.135771

C	5.726904	-3.988673	-2.299550
H	4.349393	-4.298512	-0.691752
H	5.904569	-3.351327	1.123713
H	6.548888	-4.748601	0.237122
H	7.133271	-3.121612	-0.146980
H	6.491961	-3.268399	-2.631383
H	6.204314	-4.977443	-2.241620
H	4.943075	-4.031928	-3.069487
H	4.108370	-2.083639	-2.067212
H	2.533626	-2.738373	-0.497714
C	3.517114	-2.272835	1.344362
C	4.064882	-1.292887	2.179267
C	3.230428	-3.537207	1.869613
C	4.351570	-1.586008	3.512515
H	4.267825	-0.286944	1.801868
C	3.518498	-3.830422	3.201197
H	2.778390	-4.297660	1.227684
C	4.086290	-2.856613	4.023497
H	4.779121	-0.815231	4.155668
H	3.291305	-4.820381	3.599824
H	4.309097	-3.083326	5.067332
C	0.514254	-1.519833	0.487621
H	0.872144	-2.545404	0.533251
C	-0.747692	-1.220719	0.848331
C	-1.636865	-2.259324	1.472460
F	-1.263764	-2.475018	2.744388
F	-2.910946	-1.885511	1.507054
F	-1.561425	-3.434126	0.848038
O	0.864895	0.681768	-0.278286
C	1.316452	-0.427050	-0.057028
H	5.952626	-1.098528	0.174404
C	6.811076	1.561731	-1.542838
H	7.507282	0.727019	-1.468948
H	8.319170	2.990237	-2.070558
H	-1.097918	-0.190847	0.919019
S	-2.318412	-1.011839	-1.513746
C	-3.042837	0.404608	-0.816010
N	-2.132693	1.439862	-0.591799
H	-1.177259	1.185802	-0.833343
C	-2.287821	2.694688	-0.040544
C	-1.099193	3.427151	0.197177
C	-3.526046	3.289687	0.283316
C	-1.137418	4.698943	0.734367
H	-0.140524	2.963676	-0.041945
C	-3.561983	4.567705	0.824245
H	-4.445260	2.740549	0.108211
C	-2.376138	5.261827	1.047125
H	-0.222734	5.259989	0.919834
H	-4.513654	5.035032	1.074396
N	-2.425758	6.603041	1.618464
O	-3.511896	7.072008	1.881135
O	-1.376042	7.181924	1.802082
N	-4.264374	0.573989	-0.419962
C	-5.273999	-0.334502	-0.636142
C	-6.110377	-0.676324	0.449197
C	-5.570547	-0.878134	-1.907332
C	-7.184870	-1.536040	0.286223
H	-5.884610	-0.251726	1.428250
C	-6.650949	-1.726987	-2.082649
H	-4.936286	-0.618823	-2.754133
C	-7.446169	-2.052682	-0.982508
H	-7.824588	-1.809535	1.124158
H	-6.888008	-2.143517	-3.060803

N -8.581018 -2.951572 -1.164998
O -9.262950 -3.214270 -0.197217
O -8.787646 -3.392388 -2.275608

CF3_Michael_Acceptor_TS-MA-conf/TS-MA-R-ii
Frequencies, energies and thermodynamic properties:
Lowest Vibrational Mode (1/cm) = -104.5920
2nd Lowest Vibrational Mode (1/cm) = 10.1083
E(RM062X) (a.u.) = -3187.94059886
Thermal correction to Enthalpy (a.u.) = 0.680773
Thermal correction to Gibbs Free Energy (a.u.) = 0.553113
Total Entropy (cal/Kmol) = 268.684
Esp(RM062X) (a.u.) = -3190.90402630
Optimised cartesian coordinates (Angstrom):
C 5.058498 -3.918405 -1.580405
C 3.936663 -4.594483 -2.081697
C 2.694216 -3.972695 -2.138131
C 2.598001 -2.667590 -1.654844
C 3.711507 -2.006371 -1.133791
H 4.038781 -5.620613 -2.436343
H 1.818852 -4.491460 -2.531703
C 2.086376 -0.410087 -0.802627
C 2.431912 1.731338 0.366084
C 3.639147 1.013134 0.993622
C 4.350834 0.164311 -0.040609
H 5.119521 -0.455707 0.436697
S 1.168369 -1.657417 -1.595544
N 3.375630 -0.733679 -0.658648
N 1.573050 0.749977 -0.342351
C 4.595971 1.953941 1.760223
C 5.711521 2.555818 0.901464
C 5.200376 1.212471 2.953005
H 3.982034 2.780291 2.154388
H 6.441495 1.784896 0.607403
H 5.338211 3.040781 -0.011527
H 6.256798 3.312727 1.483637
H 4.422415 0.869177 3.650654
H 5.770825 0.331180 2.618099
H 5.891403 1.864556 3.506469
H 3.207863 0.314340 1.728318
H 1.844604 2.095815 1.217282
C 2.768401 2.905074 -0.530719
C 2.784917 4.193655 0.013593
C 3.109602 2.727931 -1.875758
C 3.171961 5.284253 -0.762900
H 2.492679 4.343362 1.055993
C 3.495782 3.819538 -2.653694
H 3.070060 1.735932 -2.333174
C 3.535768 5.097841 -2.097099
H 3.181606 6.284402 -0.326703
H 3.760828 3.669584 -3.701512
H 3.836082 5.951251 -2.707208
C -0.364364 2.238915 0.010916
C -1.690619 2.507625 -0.179570
C -2.156186 3.916829 0.070589
F -3.478210 4.052915 0.083299
F -1.683815 4.410551 1.218323
F -1.701962 4.718361 -0.906608
O -0.466529 0.221251 -1.205382
C 0.176654 1.027131 -0.559140
H 4.823413 0.768643 -0.829695
C 4.967138 -2.615607 -1.104289
H 5.844477 -2.098276 -0.716708

H 6.024031 -4.424852 -1.556662
H -2.277393 1.939955 -0.903097
S -3.225325 1.555298 1.631508
C -2.667669 -0.083999 1.375698
N -3.300109 -1.053069 0.807538
N -1.366371 -0.297241 1.817184
C -4.607858 -0.981226 0.378067
C -5.673024 -0.601292 1.223990
C -4.906047 -1.389584 -0.938744
C -6.981882 -0.623468 0.769345
H -5.455189 -0.292942 2.246213
C -6.211071 -1.401841 -1.406516
H -4.081914 -1.689835 -1.587071
C -7.237020 -1.019349 -0.543849
H -7.809225 -0.336836 1.417139
H -6.445467 -1.705539 -2.425801
N -8.616230 -1.038638 -1.026166
O -8.814643 -1.384828 -2.170688
O -9.493155 -0.708027 -0.257772
C -0.525669 -1.382423 1.671697
C -0.904954 -2.634677 1.143190
C 0.816000 -1.199050 2.082006
C 0.040468 -3.640199 0.991198
H -1.934278 -2.797909 0.842516
C 1.758886 -2.198641 1.924086
H 1.105070 -0.239223 2.519432
C 1.362918 -3.412615 1.360125
H -0.242288 -4.603078 0.567096
H 2.797047 -2.050505 2.222801
N 2.365025 -4.447992 1.120717
O 3.520978 -4.188848 1.378974
O 1.991269 -5.510060 0.674267
H 0.237886 2.927575 0.596829
H -0.956290 0.521133 2.255513

CF3_Michael_Acceptor_TS-MA-conf/TS-MA-R-i

Frequencies, energies and thermodynamic properties:

Lowest Vibrational Mode (1/cm) = -184.9680

2nd Lowest Vibrational Mode (1/cm) = 4.5828

E(RM062X) (a.u.) = -3187.94763983

Thermal correction to Enthalpy (a.u.) = 0.680837

Thermal correction to Gibbs Free Energy (a.u.) = 0.554671

Total Entropy (cal/Kmol) = 265.539

Esp(RM062X) (a.u.) = -3190.90852411

Optimised cartesian coordinates (Angstrom):

C -6.259958 1.915444 -0.150895
C -6.694052 0.747207 0.489989
C -5.781696 -0.132062 1.064980
C -4.425367 0.175181 0.962355
C -3.995925 1.327500 0.301765
H -7.760193 0.524287 0.543591
H -6.113839 -1.040997 1.568994
C -1.966709 0.412469 0.899772
C 0.209002 1.406217 0.400107
C -0.515613 2.041055 -0.797441
C -1.897744 2.514267 -0.391653
H -2.482579 2.794087 -1.277621
S -3.070497 -0.756480 1.574161
N -2.599442 1.417523 0.276649
N -0.630780 0.338110 0.978540
C 0.315167 3.131977 -1.511390
C 0.108548 4.545724 -0.961797
H -0.897355 4.921055 -1.208127

H	0.240130	4.605902	0.127793
H	0.833194	5.230749	-1.425252
H	-0.658629	1.212220	-1.512052
H	1.081225	0.901532	-0.026003
C	0.683971	2.377974	1.459038
C	2.006643	2.831314	1.415814
C	-0.176793	2.875070	2.443409
C	2.454274	3.793715	2.319269
H	2.690086	2.423882	0.665386
C	0.271184	3.836604	3.349268
H	-1.205487	2.512325	2.516432
C	1.584266	4.303791	3.283586
H	3.487850	4.140666	2.274429
H	-0.408771	4.219284	4.112202
H	1.933485	5.055694	3.993160
C	1.403948	-0.856679	1.672245
H	2.040631	-0.076448	1.266219
C	1.990754	-2.037930	2.132823
C	3.416755	-1.937475	2.605087
F	4.198728	-1.282290	1.744380
F	3.469957	-1.267056	3.764736
F	3.976600	-3.128184	2.831970
O	-0.777320	-1.666884	2.057337
C	-0.014651	-0.811661	1.619472
H	-1.861312	3.376375	0.291565
C	-4.907781	2.226003	-0.253125
H	-4.579527	3.136407	-0.753888
H	-6.994104	2.594722	-0.585819
H	1.383007	-2.691506	2.762137
S	2.307693	-3.596230	0.518241
C	1.209625	-2.789346	-0.602726
N	-0.078098	-3.286796	-0.517411
H	-0.159465	-4.073435	0.119142
C	-1.286154	-2.778861	-0.960085
C	-1.438251	-1.591835	-1.706007
C	-2.443195	-3.503698	-0.593065
C	-2.710110	-1.121894	-2.013109
H	-0.556919	-1.049027	-2.031300
C	-3.706722	-3.047140	-0.916528
H	-2.330823	-4.430550	-0.027400
C	-3.829699	-1.842569	-1.610223
H	-2.838213	-0.193544	-2.570594
H	-4.597794	-3.598850	-0.619957
N	-5.162083	-1.311993	-1.892294
O	-5.240297	-0.225726	-2.422683
O	-6.118954	-1.985178	-1.577568
N	1.438368	-1.779225	-1.365739
C	2.646658	-1.148208	-1.610850
C	2.554349	0.191891	-2.052471
C	3.929732	-1.733629	-1.539260
C	3.678887	0.945979	-2.344084
H	1.560269	0.623403	-2.180155
C	5.063242	-0.990030	-1.841013
H	4.037458	-2.774650	-1.248041
C	4.929288	0.342426	-2.222386
H	3.600119	1.981081	-2.675214
H	6.054753	-1.437248	-1.783496
N	6.128911	1.123463	-2.529444
O	5.982159	2.280302	-2.858002
O	7.203561	0.573766	-2.438562
H	1.375875	2.863099	-1.368574
C	0.027465	3.107229	-3.012564
H	-1.039425	3.298199	-3.211459

H 0.606548 3.884003 -3.532976
H 0.285765 2.134485 -3.457445

CF3_Michael_Acceptor_TS-MA-conf/TS-MA-R-iv
Frequencies, energies and thermodynamic properties:
Lowest Vibrational Mode (1/cm) = -87.8545
2nd Lowest Vibrational Mode (1/cm) = 12.3779
E(RM062X) (a.u.) = -3187.94336914
Thermal correction to Enthalpy (a.u.) = 0.681649
Thermal correction to Gibbs Free Energy (a.u.) = 0.556540
Total Entropy (cal/Kmol) = 263.314
Esp(RM062X) (a.u.) = -3190.90668472
Optimised cartesian coordinates (Angstrom):
C -7.219874 0.787081 -0.494897
C -7.262103 0.482659 -1.863967
C -6.142308 -0.014792 -2.520885
C -4.979745 -0.202347 -1.772294
C -4.939231 0.107494 -0.412786
H -8.185847 0.637885 -2.422391
H -6.170246 -0.252667 -3.585076
C -2.784157 -0.634270 -0.721416
C -1.132481 -0.898623 1.078128
C -1.893959 0.257082 1.747160
C -3.389131 0.106815 1.551684
H -3.906173 1.035734 1.828856
S -3.428450 -0.791988 -2.334798
N -3.677900 -0.144028 0.138800
N -1.532650 -0.978978 -0.349865
C -1.518949 0.490065 3.228245
C -2.333792 -0.332626 4.230591
C -1.642339 1.978900 3.557315
H -0.458234 0.207678 3.333160
H -3.371456 0.032550 4.289688
H -2.360412 -1.405045 3.991044
H -1.896558 -0.224708 5.233726
H -1.422634 2.161395 4.619297
H -0.945071 2.581128 2.958139
H -2.664128 2.345189 3.362901
H -1.580894 1.150884 1.186046
H -0.080516 -0.590555 1.077267
C -1.254393 -2.242018 1.764326
C -0.247771 -2.645168 2.647964
C -2.374389 -3.060956 1.584296
C -0.371529 -3.834427 3.364780
H 0.639462 -2.019755 2.776604
C -2.497540 -4.251514 2.300201
H -3.162909 -2.781388 0.881018
C -1.500346 -4.636771 3.196723
H 0.419918 -4.138003 4.051811
H -3.375707 -4.882263 2.153259
H -1.597708 -5.569142 3.755184
C 0.678809 -1.924080 -0.905164
H 0.944649 -2.005284 0.146304
C 1.566342 -2.299465 -1.868278
C 2.730628 -3.170417 -1.488176
F 3.651289 -3.270388 -2.440656
F 3.334933 -2.765161 -0.366957
F 2.292804 -4.418259 -1.252249
O -0.937564 -1.327507 -2.515913
C -0.601033 -1.412250 -1.345995
H -3.811023 -0.722802 2.139063
C -6.061628 0.605666 0.251649
H -6.036436 0.844677 1.314195

H -8.111961 1.176200 -0.002913
H 1.255179 -2.372094 -2.910331
S 3.132549 -0.356504 -2.662180
C 2.423623 0.900216 -1.684176
N 1.071269 1.106549 -1.945512
H 0.721927 0.532541 -2.708228
C 0.137574 1.920101 -1.336674
C -1.181792 1.847922 -1.845392
C 0.395594 2.782271 -0.248020
C -2.228478 2.497669 -1.219383
H -1.372496 1.231478 -2.725850
C -0.650265 3.473200 0.350638
H 1.409825 2.889859 0.121674
C -1.953391 3.290381 -0.103929
H -3.254614 2.392792 -1.577360
H -0.463376 4.128448 1.200934
N -3.059595 3.850937 0.659097
O -2.815504 4.703171 1.483104
O -4.171598 3.408522 0.445047
N 2.999241 1.652070 -0.805864
C 4.297598 1.489734 -0.366762
C 5.121779 2.631565 -0.286171
C 4.809509 0.255611 0.090397
C 6.413669 2.550180 0.210550
H 4.721507 3.586158 -0.629898
C 6.094443 0.167604 0.601718
H 4.182282 -0.632345 0.036175
C 6.885893 1.315383 0.651764
H 7.056759 3.427459 0.264474
H 6.494088 -0.780107 0.960135
N 8.242459 1.222071 1.187252
O 8.627987 0.140958 1.576185
O 8.913827 2.230686 1.216771

CF3_Michael_Acceptor_TS-MA-conf/TS-MA-R-ix

Frequencies, energies and thermodynamic properties:

Lowest Vibrational Mode (1/cm) = -184.9621

2nd Lowest Vibrational Mode (1/cm) = 10.8039

E(RM062X) (a.u.) = -3187.93523408

Thermal correction to Enthalpy (a.u.) = 0.680928

Thermal correction to Gibbs Free Energy (a.u.) = 0.554318

Total Entropy (cal/Kmol) = 266.473

Esp(RM062X) (a.u.) = -3190.89624583

Optimised cartesian coordinates (Angstrom):

C -3.499284 -4.852767 1.323671
C -2.177897 -5.267480 1.547710
C -1.128303 -4.357862 1.499372
C -1.432328 -3.024853 1.218217
C -2.746426 -2.616775 0.990254
H -1.970299 -6.316911 1.759787
H -0.097424 -4.672329 1.669599
C -1.635868 -0.623399 0.679506
C -2.718793 1.513369 0.164802
C -3.840802 0.650014 -0.437295
C -4.089419 -0.583731 0.407102
H -4.722884 -1.294033 -0.139106
S -0.312116 -1.685202 1.080046
N -2.817470 -1.252693 0.683550
N -1.512508 0.683056 0.378702
C -5.135068 1.436345 -0.746414
C -6.132913 1.502490 0.412739
C -5.811258 0.847355 -1.985253
H -4.826081 2.466395 -0.990422

H	-6.948102	2.194893	0.157118
H	-6.590026	0.517195	0.596109
H	-5.679862	1.850966	1.351654
H	-6.072249	-0.211074	-1.824150
H	-6.740227	1.388588	-2.216425
H	-5.154818	0.902768	-2.865917
H	-3.442322	0.300272	-1.401344
H	-2.464371	2.236997	-0.615012
C	-3.085495	2.275666	1.420897
C	-3.100795	1.662819	2.679180
C	-3.470911	3.615347	1.310304
C	-3.523615	2.372929	3.802431
H	-2.779010	0.625061	2.799261
C	-3.896338	4.324924	2.432529
H	-3.438828	4.105231	0.334073
C	-3.929399	3.702450	3.680415
H	-3.532399	1.885236	4.778518
H	-4.195851	5.369494	2.332623
H	-4.259158	4.256497	4.560814
C	-0.029484	2.563689	-0.260341
H	-0.855446	3.180315	-0.602525
C	1.276588	2.917081	-0.564245
C	1.573847	4.354799	-0.895405
F	1.299932	5.140351	0.155618
F	2.855773	4.561688	-1.197527
F	0.837298	4.804925	-1.914326
O	0.743300	0.529333	0.610104
C	-0.188417	1.251420	0.281702
H	-4.568223	-0.344415	1.369062
C	-3.805223	-3.526240	1.043646
H	-4.834554	-3.217008	0.866630
H	-4.306595	-5.584992	1.363839
H	2.088973	2.416046	-0.032302
S	1.936436	1.972054	-2.616242
C	2.767040	0.602909	-1.882477
N	3.950025	0.990365	-1.275685
H	4.242514	1.935633	-1.507610
C	4.809173	0.290581	-0.441814
C	4.491087	-0.930109	0.187475
C	6.065870	0.883145	-0.189546
C	5.410837	-1.532574	1.034496
H	3.525384	-1.393114	0.011891
C	6.982640	0.281883	0.653841
H	6.313985	1.829841	-0.673760
C	6.644968	-0.928854	1.257021
H	5.173399	-2.472295	1.531241
H	7.953786	0.735662	0.845792
N	7.607309	-1.575189	2.147606
O	8.664778	-1.016791	2.341104
O	7.297505	-2.634752	2.646259
N	2.459432	-0.651949	-1.860635
C	1.245598	-1.252348	-2.096384
C	0.003750	-0.607869	-2.317271
C	1.235242	-2.668514	-1.996672
C	-1.176571	-1.332587	-2.342379
H	-0.032070	0.471190	-2.440772
C	0.058401	-3.396916	-2.003743
H	2.192372	-3.173155	-1.856696
C	-1.150324	-2.710859	-2.143403
H	-2.131346	-0.832942	-2.496013
H	0.058276	-4.478316	-1.870184
N	-2.412508	-3.427070	-2.011162
O	-3.441604	-2.777032	-2.038534

O -2.378142 -4.626822 -1.858327

CF3_Michael_Acceptor_TS-MA-conf/TS-MA-R-viii

Frequencies, energies and thermodynamic properties:

Lowest Vibrational Mode (1/cm) = -166.6097

2nd Lowest Vibrational Mode (1/cm) = 9.2326

E(RM062X) (a.u.) = -3187.93706911

Thermal correction to Enthalpy (a.u.) = 0.680759

Thermal correction to Gibbs Free Energy (a.u.) = 0.553743

Total Entropy (cal/Kmol) = 267.328

Esp(RM062X) (a.u.) = -3190.89903908

Optimised cartesian coordinates (Angstrom):

C 6.017677 -3.958980 -1.264156
C 5.200573 -4.509882 -2.261195
C 3.980928 -3.925899 -2.589495
C 3.602972 -2.774985 -1.901149
C 4.414793 -2.234254 -0.903233
H 5.522769 -5.409095 -2.787500
H 3.339536 -4.354434 -3.360653
C 2.653923 -0.752600 -0.844370
C 2.612649 1.292544 0.497203
C 3.463931 0.482199 1.487861
C 4.494154 -0.351750 0.751057
H 4.943339 -1.085740 1.432951
S 2.109899 -1.872110 -2.066905
N 3.835089 -1.102207 -0.320182
N 2.004850 0.364650 -0.483458
C 4.081366 1.326408 2.624712
C 5.461720 1.907995 2.307520
C 4.146521 0.493331 3.905592
H 3.389336 2.165875 2.804261
H 6.225791 1.115107 2.275676
H 5.490886 2.451081 1.351995
H 5.757245 2.610021 3.100783
H 4.592397 1.074880 4.725751
H 3.145489 0.166330 4.222941
H 4.763923 -0.407051 3.757913
H 2.760983 -0.225550 1.952042
H 1.774557 1.699733 1.076455
C 3.339159 2.435827 -0.180480
C 3.221949 3.723912 0.353534
C 4.168273 2.230914 -1.288447
C 3.945853 4.784221 -0.189218
H 2.556421 3.895557 1.203411
C 4.891798 3.292036 -1.833102
H 4.256171 1.241332 -1.743738
C 4.788343 4.568603 -1.280289
H 3.845996 5.783606 0.237437
H 5.536197 3.119454 -2.696649
H 5.354234 5.397716 -1.708164
C 0.131106 1.921571 -0.782642
H 0.750725 2.748225 -0.441926
C -1.238050 2.079654 -0.886830
C -1.781317 3.446320 -1.212251
F -3.113255 3.507198 -1.193111
F -1.314625 4.391286 -0.392826
F -1.409590 3.796192 -2.453542
O 0.129537 -0.298870 -1.598572
C 0.674399 0.617877 -1.005594
H 5.293883 0.258339 0.304338
C 5.638198 -2.815263 -0.568994
H 6.276961 -2.390828 0.205108
H 6.969354 -4.434820 -1.024715

H -1.820770 1.264789 -1.326266
S -2.229029 1.954380 1.256998
C -3.307676 0.594592 0.950819
N -4.612821 0.992327 0.720380
H -4.730313 2.000825 0.721751
C -5.756071 0.263308 0.442574
C -6.912768 1.010805 0.123757
C -5.840588 -1.143524 0.471509
C -8.112382 0.385817 -0.159363
H -6.849098 2.100233 0.098415
C -7.046398 -1.770711 0.185505
H -4.962515 -1.731302 0.716694
C -8.166739 -1.007882 -0.126767
H -9.003227 0.961091 -0.407003
H -7.123153 -2.856976 0.206807
N -9.430090 -1.679527 -0.424768
O -9.449959 -2.890432 -0.401235
O -10.392614 -0.989232 -0.679577
N -3.004729 -0.655457 0.870648
C -1.756645 -1.198034 1.089558
C -1.352288 -2.238536 0.227506
C -0.896915 -0.833719 2.152539
C -0.111981 -2.838715 0.356208
H -2.030732 -2.540008 -0.570871
C 0.345017 -1.432990 2.293117
H -1.214686 -0.074679 2.864107
C 0.738571 -2.410995 1.375482
H 0.211645 -3.621136 -0.328143
H 1.007963 -1.160283 3.113989
N 2.072942 -2.989988 1.470299
O 2.828239 -2.564345 2.324587
O 2.378667 -3.856443 0.679214

CF3_Michael_Acceptor_TS-MA-conf/TS-MA-R-vii

Frequencies, energies and thermodynamic properties:

Lowest Vibrational Mode (1/cm) = -157.2619

2nd Lowest Vibrational Mode (1/cm) = 5.8305

E(RM062X) (a.u.) = -3187.93673395

Thermal correction to Enthalpy (a.u.) = 0.680632

Thermal correction to Gibbs Free Energy (a.u.) = 0.551772

Total Entropy (cal/Kmol) = 271.210

Esp(RM062X) (a.u.) = -3190.89848612

Optimised cartesian coordinates (Angstrom):

C 6.402645 -3.770134 -1.023730
C 5.583199 -4.440652 -1.943348
C 4.320973 -3.951921 -2.263300
C 3.900515 -2.776299 -1.642936
C 4.713118 -2.117079 -0.720127
H 5.938895 -5.357170 -2.415312
H 3.678612 -4.468671 -2.977737
C 2.878800 -0.731098 -0.707771
C 2.699999 1.323825 0.604992
C 3.591164 0.563418 1.601635
C 4.692611 -0.173086 0.862156
H 5.206079 -0.868191 1.539614
S 2.363060 -1.957662 -1.837026
N 4.096047 -0.970949 -0.210260
N 2.161772 0.353916 -0.375839
C 4.124301 1.436279 2.759499
C 5.470402 2.109073 2.477609
C 4.215958 0.599990 4.036448
H 3.375200 2.227146 2.929244
H 6.283826 1.366690 2.452474

H	5.483707	2.664385	1.528890
H	5.704329	2.818920	3.284288
H	3.232331	0.204484	4.329285
H	4.897219	-0.254769	3.896539
H	4.603604	1.203169	4.870348
H	2.936087	-0.209326	2.035019
H	1.831644	1.681993	1.172841
C	3.364373	2.507653	-0.067869
C	3.179876	3.785595	0.471769
C	4.196280	2.352873	-1.181770
C	3.840020	4.886144	-0.071893
H	2.510595	3.918482	1.325536
C	4.856131	3.454439	-1.727297
H	4.333133	1.371491	-1.642605
C	4.685731	4.721398	-1.169363
H	3.687438	5.876983	0.359085
H	5.502606	3.320955	-2.596183
H	5.201135	5.582199	-1.598454
C	0.231551	1.819154	-0.767976
H	0.812038	2.682089	-0.447944
C	-1.126512	1.930935	-0.974804
C	-1.682697	3.260695	-1.412941
F	-1.303113	4.264224	-0.619178
F	-1.228148	3.552393	-2.642470
F	-3.012454	3.280527	-1.496530
O	0.336658	-0.428343	-1.497133
C	0.830978	0.526961	-0.923442
H	5.437032	0.507572	0.422653
C	5.982362	-2.600978	-0.398645
H	6.625514	-2.080913	0.310619
H	7.390210	-4.170497	-0.791467
H	-1.661728	1.071055	-1.388701
S	-2.317262	1.930895	1.112599
C	-3.423019	0.599128	0.788825
N	-4.732838	1.025257	0.647941
H	-4.830450	2.034770	0.693989
C	-5.908534	0.327565	0.435680
C	-7.077671	1.107146	0.277561
C	-6.014807	-1.077356	0.380465
C	-8.308586	0.515670	0.067004
H	-6.999676	2.195068	0.323191
C	-7.252719	-1.670590	0.169278
H	-5.126989	-1.688781	0.500995
C	-8.384088	-0.876475	0.013415
H	-9.208700	1.115919	-0.056548
H	-7.345523	-2.754974	0.125475
N	-9.680869	-1.511955	-0.209533
O	-9.722402	-2.721958	-0.249741
O	-10.648273	-0.794644	-0.342724
N	-3.151831	-0.652438	0.633818
C	-1.920317	-1.250005	0.804238
C	-0.977244	-0.897209	1.798005
C	-1.627554	-2.348297	-0.032994
C	0.233870	-1.565140	1.891289
H	-1.203391	-0.092823	2.493508
C	-0.427026	-3.030228	0.062001
H	-2.367494	-2.636963	-0.780321
C	0.503992	-2.615663	1.012796
H	0.960274	-1.297265	2.658726
H	-0.193108	-3.861377	-0.602286
N	1.800776	-3.274444	1.068902
O	1.941890	-4.322462	0.480584
O	2.692924	-2.726063	1.692478

CF3_Michael_Acceptor_TS-MA-conf/TS-MA-R-vi

Frequencies, energies and thermodynamic properties:

Lowest Vibrational Mode (1/cm) = -95.3079

2nd Lowest Vibrational Mode (1/cm) = 6.2238

E(RM062X) (a.u.) = -3187.92682929

Thermal correction to Enthalpy (a.u.) = 0.680852

Thermal correction to Gibbs Free Energy (a.u.) = 0.547975

Total Entropy (cal/Kmol) = 279.664

Esp(RM062X) (a.u.) = -3190.89573391

Optimised cartesian coordinates (Angstrom):

C	-8.632458	1.931228	0.368980
C	-8.175919	3.145884	-0.163178
C	-6.833732	3.327561	-0.478918
C	-5.959732	2.265264	-0.247913
C	-6.415052	1.061480	0.291319
H	-8.881883	3.959034	-0.335300
H	-6.474122	4.269060	-0.896030
C	-4.183385	0.570051	0.023210
C	-3.171786	-1.608181	0.522827
C	-4.268649	-1.689387	1.597678
C	-5.584751	-1.185921	1.038066
H	-6.325232	-1.077123	1.840868
S	-4.233594	2.215458	-0.554320
N	-5.375638	0.137614	0.446582
N	-3.088566	-0.213444	0.025192
C	-4.393764	-3.080966	2.258909
C	-5.361452	-4.034334	1.552429
C	-4.800154	-2.921019	3.724267
H	-3.387901	-3.531721	2.236681
H	-6.404677	-3.707409	1.687445
H	-5.168102	-4.125950	0.474402
H	-5.276989	-5.037983	1.993715
H	-4.901762	-3.903121	4.208344
H	-4.056544	-2.337783	4.286489
H	-5.771179	-2.406613	3.807139
H	-3.955644	-0.978977	2.380143
H	-2.225773	-1.799244	1.043010
C	-3.317681	-2.597893	-0.614287
C	-2.626603	-3.812382	-0.547065
C	-4.163801	-2.353195	-1.701077
C	-2.802377	-4.781679	-1.532849
H	-1.945161	-3.999517	0.286917
C	-4.339184	-3.322851	-2.688664
H	-4.690140	-1.399534	-1.793733
C	-3.665315	-4.541116	-2.602703
H	-2.258153	-5.725268	-1.468841
H	-5.001932	-3.121388	-3.531768
H	-3.801981	-5.297548	-3.377201
C	-0.744185	-0.604420	-0.631272
H	-0.840626	-1.669090	-0.434195
C	0.470378	-0.079888	-0.975073
C	1.536448	-0.997228	-1.504220
F	2.716044	-0.393991	-1.654414
F	1.722844	-2.077819	-0.739551
F	1.186510	-1.447255	-2.718419
O	-1.805965	1.500993	-0.743802
C	-1.845285	0.314779	-0.474998
H	-5.995634	-1.853168	0.265761
C	-7.762786	0.871813	0.604313
H	-8.127122	-0.071335	1.010151
H	-9.690935	1.810191	0.602362
H	0.543424	0.955690	-1.311276

S 1.654351 0.556469 1.146727
C 3.396957 0.433008 0.956335
N 3.858369 -0.872747 1.026474
H 3.117454 -1.542583 1.204376
C 5.098423 -1.418374 0.762421
C 5.178207 -2.830676 0.769726
C 6.264545 -0.672821 0.491299
C 6.370486 -3.481000 0.516767
H 4.275656 -3.410163 0.973335
C 7.461803 -1.327859 0.234987
H 6.219412 0.411366 0.487687
C 7.508465 -2.718509 0.249731
H 6.431591 -4.568203 0.520484
H 8.367793 -0.761027 0.024152
N 8.773570 -3.396431 -0.018688
O 9.748020 -2.714144 -0.248544
O 8.783973 -4.608293 0.002572
N 4.238601 1.394812 0.791948
C 3.867650 2.697446 0.536767
C 4.402774 3.732887 1.329101
C 3.036953 3.038847 -0.553441
C 4.101882 5.060683 1.065521
H 5.055813 3.468381 2.161557
C 2.740708 4.363024 -0.831490
H 2.644538 2.240317 -1.183467
C 3.271470 5.361068 -0.012869
H 4.502482 5.865275 1.680601
H 2.104740 4.635579 -1.672601
N 2.953954 6.758373 -0.298014
O 2.227502 6.997135 -1.238459
O 3.431625 7.609487 0.420665

CF3_Michael_Acceptor_TS-MA-conf/TS-MA-R-v

Frequencies, energies and thermodynamic properties:

Lowest Vibrational Mode (1/cm) = -193.4111

2nd Lowest Vibrational Mode (1/cm) = 12.8907

E(RM062X) (a.u.) = -3187.94497830

Thermal correction to Enthalpy (a.u.) = 0.680676

Thermal correction to Gibbs Free Energy (a.u.) = 0.556152

Total Entropy (cal/Kmol) = 262.083

Esp(RM062X) (a.u.) = -3190.90463753

Optimised cartesian coordinates (Angstrom):

C -5.146113 -3.581970 -0.789316
C -6.002275 -2.478170 -0.899672
C -5.491165 -1.194895 -1.069269
C -4.105887 -1.042760 -1.119530
C -3.255062 -2.142223 -0.988935
H -7.081895 -2.623889 -0.851040
H -6.153123 -0.330956 -1.153033
C -1.710133 -0.441319 -1.169893
C 0.723611 -0.716389 -0.977854
C 0.350391 -1.991318 -0.203251
C -0.804873 -2.705806 -0.876685
H -1.158088 -3.537683 -0.254357
S -3.204379 0.450036 -1.310234
N -1.910320 -1.759424 -1.023885
N -0.487194 0.105498 -1.194815
C 1.542475 -2.920169 0.104437
C 1.896064 -3.907082 -1.012122
C 1.277867 -3.697350 1.396122
H 2.409481 -2.258539 0.280067
H 2.820715 -4.442274 -0.749460
H 1.105561 -4.665807 -1.125828

H	2.052308	-3.427885	-1.987796
H	0.485687	-4.448361	1.244833
H	2.183968	-4.228502	1.724239
H	0.960884	-3.032800	2.212412
H	-0.022905	-1.620516	0.765180
H	1.352951	-0.139620	-0.287540
C	1.487219	-0.929745	-2.267892
C	0.837526	-1.218709	-3.471804
C	2.885692	-0.885952	-2.241426
C	1.576421	-1.495103	-4.622671
H	-0.254271	-1.224253	-3.521516
C	3.624682	-1.162293	-3.390008
H	3.396744	-0.638889	-1.306632
C	2.970573	-1.475407	-4.582529
H	1.058670	-1.722305	-5.556074
H	4.714882	-1.128620	-3.355353
H	3.547168	-1.691355	-5.483509
C	0.928452	2.121510	-1.282566
H	1.839499	1.534926	-1.212847
C	0.965173	3.500578	-1.018091
C	2.269364	4.211018	-1.260478
F	2.571626	4.210839	-2.562975
F	2.250796	5.483527	-0.869949
F	3.300464	3.617551	-0.631307
O	-1.416901	2.180500	-1.391736
C	-0.366189	1.549845	-1.316138
H	-0.533082	-3.097986	-1.869001
C	-3.762591	-3.433238	-0.832444
H	-3.106046	-4.299001	-0.750584
H	-5.567666	-4.580148	-0.664550
H	0.109309	4.090361	-1.354594
S	0.608526	3.944343	1.126704
C	0.869690	2.266967	1.623048
N	0.027722	1.326350	1.869918
C	-1.358257	1.320422	1.879577
C	-2.232426	2.404886	1.643474
C	-1.934323	0.053913	2.146637
C	-3.608901	2.217340	1.654669
H	-1.830746	3.392774	1.438268
C	-3.305323	-0.143811	2.156314
H	-1.261151	-0.778329	2.358171
C	-4.129428	0.950166	1.900457
H	-4.284662	3.048209	1.456065
H	-3.740724	-1.122888	2.352883
N	-5.578499	0.747951	1.845486
O	-6.007766	-0.354614	2.105234
O	-6.272481	1.691028	1.534703
N	2.213658	1.945219	1.613615
C	2.835226	0.726281	1.856822
C	2.332976	-0.243563	2.745728
C	4.060371	0.490923	1.198746
C	3.046929	-1.413975	2.967315
H	1.401123	-0.062359	3.273843
C	4.763939	-0.684384	1.405479
H	4.445985	1.244544	0.509114
C	4.240584	-1.630356	2.285380
H	2.684200	-2.163554	3.668863
H	5.701986	-0.878773	0.887245
N	4.956712	-2.890720	2.485823
O	4.443462	-3.728766	3.195315
O	6.021970	-3.031619	1.929089
H	2.816430	2.673617	1.243475

CF3_Michael_Acceptor_TS-MA-conf/TS-MA-R-xiii

Frequencies, energies and thermodynamic properties:

Lowest Vibrational Mode (1/cm) = -134.8135

2nd Lowest Vibrational Mode (1/cm) = 13.8271

E(RM062X) (a.u.) = -3187.93706226

Thermal correction to Enthalpy (a.u.) = 0.681021

Thermal correction to Gibbs Free Energy (a.u.) = 0.555827

Total Entropy (cal/Kmol) = 263.494

Esp(RM062X) (a.u.) = -3190.89848931

Optimised cartesian coordinates (Angstrom):

C	-7.356309	1.833453	-1.309335
C	-6.842210	2.730698	-2.256411
C	-5.488537	2.733397	-2.575524
C	-4.663031	1.819386	-1.922637
C	-5.176486	0.930517	-0.978233
H	-7.511153	3.437042	-2.749299
H	-5.082681	3.430625	-3.309769
C	-2.946979	0.353699	-0.913927
C	-1.992349	-1.422027	0.490383
C	-3.232664	-1.195327	1.369154
C	-4.464343	-0.949870	0.523336
H	-5.295536	-0.614115	1.155923
S	-2.935704	1.610583	-2.123898
N	-4.174571	0.119470	-0.433500
N	-1.860092	-0.317597	-0.489473
C	-3.443300	-2.283803	2.444727
C	-4.306188	-3.462945	1.989153
C	-4.037929	-1.651409	3.703811
H	-2.444214	-2.671238	2.704571
H	-5.354892	-3.154062	1.853906
H	-3.956513	-3.910773	1.048136
H	-4.295936	-4.248356	2.758787
H	-3.370390	-0.881844	4.119309
H	-5.007267	-1.175227	3.485921
H	-4.205922	-2.412323	4.479840
H	-3.041117	-0.252161	1.897621
H	-1.137956	-1.332706	1.171597
C	-1.929885	-2.767908	-0.201803
C	-1.197619	-3.804454	0.385802
C	-2.629614	-3.013698	-1.388187
C	-1.193676	-5.077592	-0.181632
H	-0.626516	-3.611064	1.297448
C	-2.624322	-4.287074	-1.957584
H	-3.180473	-2.211633	-1.886627
C	-1.913745	-5.323193	-1.351182
H	-0.619742	-5.878542	0.287183
H	-3.174285	-4.468168	-2.882513
H	-1.909584	-6.318653	-1.798143
C	0.575259	-0.730332	-0.607419
H	0.562113	-1.339789	0.292345
C	1.758463	-0.520311	-1.279078
C	2.877443	-1.495822	-1.018617
F	3.100413	-1.668421	0.289622
F	2.544809	-2.701693	-1.509143
F	4.031488	-1.166507	-1.587548
O	-0.562101	0.775029	-2.010090
C	-0.580827	-0.038088	-1.101820
H	-4.781118	-1.841833	-0.039038
C	-6.534861	0.920189	-0.657359
H	-6.943821	0.225413	0.075114
H	-8.421511	1.849345	-1.075856
H	1.729281	-0.124203	-2.295253
S	2.950994	1.519634	-0.691869

C 3.117111 1.190357 1.013492
N 1.992634 1.169339 1.838254
H 2.213511 0.749360 2.739036
C 0.693431 1.609319 1.714185
C -0.206222 1.173401 2.718373
C 0.210178 2.476492 0.709934
C -1.545084 1.508149 2.675683
H 0.169116 0.545655 3.529029
C -1.134663 2.810759 0.665944
H 0.884248 2.856647 -0.050685
C -2.010796 2.298386 1.622380
H -2.235786 1.153156 3.440210
H -1.513796 3.460560 -0.122165
N -3.440367 2.524577 1.481190
O -3.819204 3.279283 0.611260
O -4.192312 1.918136 2.221949
N 4.197595 0.836507 1.626105
C 5.434289 0.743668 1.020271
C 6.038782 1.824105 0.343490
C 6.155131 -0.461019 1.149923
C 7.313093 1.703420 -0.188403
H 5.494159 2.763197 0.248294
C 7.424809 -0.595851 0.609554
H 5.687428 -1.291780 1.679173
C 7.990322 0.491698 -0.053618
H 7.789155 2.534005 -0.707747
H 7.982467 -1.527207 0.696650
N 9.333095 0.360641 -0.617497
O 9.907081 -0.697833 -0.480908
O 9.804739 1.317137 -1.192663

CF3_Michael_Acceptor_TS-MA-conf/TS-MA-R-xii

Frequencies, energies and thermodynamic properties:

Lowest Vibrational Mode (1/cm) = -100.8931
2nd Lowest Vibrational Mode (1/cm) = 9.5552
E(RM062X) (a.u.) = -3187.92481392
Thermal correction to Enthalpy (a.u.) = 0.680856
Thermal correction to Gibbs Free Energy (a.u.) = 0.549230
Total Entropy (cal/Kmol) = 277.031
Esp(RM062X) (a.u.) = -3190.89242434

Optimised cartesian coordinates (Angstrom):

C 7.657144 0.033729 -2.523798
C 7.370915 1.395322 -2.701969
C 6.140132 1.921570 -2.325308
C 5.202351 1.051585 -1.768880
C 5.486589 -0.304131 -1.600676
H 8.123696 2.052717 -3.138290
H 5.914118 2.980587 -2.457015
C 3.353897 -0.225809 -0.741764
C 2.169390 -2.146942 0.230844
C 3.026206 -2.964671 -0.750745
C 4.446023 -2.433759 -0.775531
H 5.016841 -2.898476 -1.589790
S 3.590093 1.440490 -1.200593
N 4.409442 -0.992043 -1.031356
N 2.252582 -0.710786 -0.134158
C 2.963910 -4.490986 -0.512789
C 4.014011 -5.023350 0.466032
C 3.078516 -5.227656 -1.847711
H 1.966113 -4.706086 -0.096809
H 3.807520 -6.080375 0.687738
H 5.022657 -4.975346 0.025823
H 4.030860 -4.478526 1.420350

H	4.034445	-4.994200	-2.343587
H	3.041237	-6.315787	-1.693738
H	2.263135	-4.951041	-2.531544
H	2.589787	-2.767834	-1.743582
H	1.132662	-2.446941	0.039757
C	2.483009	-2.363094	1.697106
C	3.529578	-1.688375	2.334665
C	1.734821	-3.299649	2.418911
C	3.842709	-1.971352	3.664434
H	4.108901	-0.927577	1.804556
C	2.048583	-3.583561	3.746643
H	0.898444	-3.811063	1.935435
C	3.108534	-2.924343	4.370277
H	4.661303	-1.439336	4.151721
H	1.458063	-4.316908	4.298150
H	3.353354	-3.142864	5.410954
C	0.075452	-0.328494	0.970191
H	-0.001924	-1.371220	1.266409
C	-0.937704	0.541008	1.243984
C	-1.984070	0.159818	2.252382
F	-1.473089	0.255106	3.489922
F	-3.053135	0.954831	2.226818
F	-2.406086	-1.098763	2.109586
O	1.266011	1.336980	-0.198227
C	1.181456	0.187693	0.194561
H	4.977061	-2.606604	0.172635
C	6.723629	-0.836140	-1.971185
H	6.958368	-1.890305	-1.827323
H	8.632130	-0.354189	-2.821037
H	-0.803012	1.613150	1.095066
S	-2.512251	0.468844	-0.769975
C	-3.860325	1.548387	-0.563825
N	-5.112125	1.253474	-0.734127
C	-5.604501	-0.018936	-0.908381
C	-6.536716	-0.247029	-1.943196
C	-5.294911	-1.088447	-0.035576
C	-7.123429	-1.490350	-2.121132
H	-6.786629	0.582556	-2.605651
C	-5.885893	-2.330070	-0.198554
H	-4.589336	-0.921018	0.777374
C	-6.789772	-2.522148	-1.245292
H	-7.836368	-1.671361	-2.924340
H	-5.658266	-3.155258	0.474946
N	-7.406759	-3.833109	-1.421567
O	-7.091312	-4.719608	-0.656804
O	-8.203858	-3.972122	-2.324546
N	-3.672096	2.871034	-0.169972
C	-2.577652	3.701320	-0.040299
C	-1.289160	3.444842	-0.555268
C	-2.797131	4.921798	0.642984
C	-0.262940	4.358124	-0.360783
H	-1.089671	2.523092	-1.093651
C	-1.778979	5.838609	0.824579
H	-3.792336	5.134945	1.038066
C	-0.510044	5.543732	0.325111
H	0.734808	4.149727	-0.743687
H	-1.952973	6.773862	1.354660
N	0.575318	6.498908	0.521203
O	1.670755	6.213965	0.087545
O	0.326398	7.530180	1.107657
H	-4.559900	3.304789	0.073138

CF3_Michael_Acceptor_TS-MA-conf/TS-MA-R-xi

Frequencies, energies and thermodynamic properties:

Lowest Vibrational Mode (1/cm) = -127.6379

2nd Lowest Vibrational Mode (1/cm) = 11.7363

E(RM062X) (a.u.) = -3187.93273106

Thermal correction to Enthalpy (a.u.) = 0.680933

Thermal correction to Gibbs Free Energy (a.u.) = 0.554393

Total Entropy (cal/Kmol) = 266.324

Esp(RM062X) (a.u.) = -3190.89730460

Optimised cartesian coordinates (Angstrom):

C	3.680690	6.299501	-0.401985
C	2.416160	6.876891	-0.219468
C	1.298441	6.083919	0.018899
C	1.475251	4.701229	0.064383
C	2.733497	4.128657	-0.126804
H	2.305888	7.961029	-0.260429
H	0.313196	6.528137	0.167645
C	1.470728	2.232913	0.223438
C	2.344426	-0.069282	0.196372
C	3.412303	0.530014	-0.738227
C	3.859781	1.882863	-0.218278
H	4.519471	2.372522	-0.944776
S	0.251768	3.477975	0.360078
N	2.682732	2.733088	-0.043165
N	1.248550	0.917388	0.382506
C	4.605874	-0.405484	-1.035657
C	5.741255	-0.343076	-0.008313
C	5.174475	-0.106941	-2.423797
H	4.205952	-1.432823	-1.053650
H	6.284228	0.612288	-0.086428
H	5.401348	-0.458817	1.029387
H	6.465091	-1.144635	-0.215372
H	4.410829	-0.193350	-3.209485
H	5.586927	0.914346	-2.466072
H	5.988183	-0.806141	-2.662810
H	2.890592	0.705762	-1.694672
H	1.895242	-0.907886	-0.351405
C	2.874163	-0.597509	1.513201
C	3.229389	-1.949510	1.591763
C	3.062976	0.224245	2.627413
C	3.795812	-2.464618	2.755308
H	3.068724	-2.601107	0.727283
C	3.629046	-0.291995	3.794136
H	2.762032	1.274660	2.598678
C	4.002769	-1.633923	3.858056
H	4.071237	-3.519343	2.803077
H	3.772646	0.358255	4.658618
H	4.443326	-2.036505	4.771581
C	-0.229317	-0.892265	1.146723
H	0.580841	-1.608743	1.066527
C	-1.472089	-1.315240	1.585619
C	-1.491535	-2.583691	2.398118
F	-0.775948	-3.560446	1.829093
F	-0.950395	-2.361442	3.603398
F	-2.716659	-3.060459	2.615552
O	-0.958758	1.322802	0.806489
C	-0.076715	0.477141	0.783237
H	4.387615	1.806120	0.744385
C	3.860610	4.920823	-0.357084
H	4.850504	4.484778	-0.487990
H	4.544768	6.940737	-0.580021
H	-2.197154	-0.567510	1.911841
S	-2.995612	-2.084328	-0.054043
C	-2.611587	-0.891491	-1.275126

N -3.390112 0.059115 -1.668769
C -4.675057 0.262387 -1.208821
C -5.003832 1.526486 -0.678868
C -5.689218 -0.711894 -1.318030
C -6.292775 1.808441 -0.252977
H -4.216040 2.276735 -0.601699
C -6.984103 -0.434621 -0.907138
H -5.446760 -1.687973 -1.738532
C -7.269937 0.822083 -0.374868
H -6.551551 2.778711 0.168443
H -7.774735 -1.178983 -0.991249
N -8.634360 1.115840 0.063059
O -9.469047 0.245613 -0.053472
O -8.860622 2.215031 0.519971
N -1.364910 -0.861698 -1.895053
C -0.267436 -1.693146 -1.959444
C -0.109240 -2.917444 -1.275256
C 0.790660 -1.242639 -2.785414
C 1.089429 -3.609144 -1.351177
H -0.912274 -3.300394 -0.653696
C 1.977936 -1.944983 -2.880525
H 0.663197 -0.315077 -3.347860
C 2.131439 -3.112303 -2.133322
H 1.224504 -4.535725 -0.793907
H 2.790341 -1.595542 -3.515713
N 3.409947 -3.810750 -2.154730
O 4.295863 -3.355197 -2.848288
O 3.529235 -4.803271 -1.469444
H -1.301555 -0.049839 -2.506137

CF3_Michael_Acceptor_TS-MA-conf/TS-MA-R-xiv

Frequencies, energies and thermodynamic properties:

Lowest Vibrational Mode (1/cm) = -193.8158
2nd Lowest Vibrational Mode (1/cm) = 17.5306
E(RM062X) (a.u.) = -3187.93578565
Thermal correction to Enthalpy (a.u.) = 0.681103
Thermal correction to Gibbs Free Energy (a.u.) = 0.556572
Total Entropy (cal/Kmol) = 262.098
Esp(RM062X) (a.u.) = -3190.89704171

Optimised cartesian coordinates (Angstrom):

C -6.679174 -1.564870 0.685009
C -7.012027 -0.478262 -0.135007
C -6.031048 0.196731 -0.855360
C -4.712109 -0.238874 -0.732461
C -4.380791 -1.309336 0.100423
H -8.050813 -0.154977 -0.209887
H -6.283401 1.045654 -1.493480
C -2.288684 -0.711106 -0.659458
C -0.188738 -1.769214 0.029548
C -0.976787 -2.146288 1.294190
C -2.381989 -2.587068 0.932638
H -2.990743 -2.708247 1.837369
S -3.291085 0.444436 -1.499977
N -3.001590 -1.545236 0.113289
N -0.953611 -0.775325 -0.751550
C -0.239308 -3.144824 2.212972
C -0.527594 -4.616482 1.906011
C -0.564157 -2.839408 3.675374
H 0.839584 -2.968958 2.067269
H -1.562643 -4.881036 2.174750
H -0.371961 -4.872067 0.848242
H 0.136139 -5.255627 2.506229
H -0.247717 -1.822508 3.951685

H	-1.646756	-2.924028	3.864235
H	-0.053883	-3.547378	4.344414
H	-1.079627	-1.201392	1.852858
H	0.704488	-1.243407	0.391330
C	0.244246	-2.934167	-0.835486
C	1.529278	-3.460860	-0.671447
C	-0.623393	-3.529812	-1.757493
C	1.932425	-4.584757	-1.390626
H	2.218430	-2.981937	0.028081
C	-0.220778	-4.653620	-2.479041
H	-1.621942	-3.118152	-1.925619
C	1.054400	-5.187909	-2.291667
H	2.937972	-4.985445	-1.252760
H	-0.906130	-5.110480	-3.194841
H	1.368165	-6.066264	-2.858105
C	1.161820	0.220969	-1.543457
H	1.764559	-0.558656	-1.086147
C	1.753171	1.448827	-1.897466
C	3.194515	1.433727	-2.334275
F	3.983422	0.740923	-1.511464
F	3.292274	0.858136	-3.541345
F	3.713713	2.656726	-2.454596
O	-0.961121	1.073650	-2.082554
C	-0.253124	0.233408	-1.532663
H	-2.393976	-3.534894	0.373465
C	-5.362285	-1.997218	0.816187
H	-5.114072	-2.844738	1.454445
H	-7.464605	-2.084140	1.235533
H	1.156196	2.096697	-2.544220
S	1.785499	2.866767	-0.221933
C	1.353832	1.677218	1.002520
N	0.041877	1.522683	1.422695
H	0.002704	0.801146	2.141106
C	-1.198253	2.074531	1.135144
C	-2.282752	1.515256	1.854476
C	-1.463832	3.101338	0.209354
C	-3.582352	1.943041	1.652956
H	-2.084055	0.734615	2.592210
C	-2.768184	3.532161	0.006772
H	-0.655022	3.548929	-0.358727
C	-3.813070	2.950305	0.718147
H	-4.414157	1.502929	2.202054
H	-2.980909	4.315167	-0.719879
N	-5.187274	3.368178	0.451668
O	-5.375572	4.170110	-0.436611
O	-6.068730	2.884072	1.128050
N	2.150007	0.804923	1.522051
C	3.524922	0.764420	1.384760
C	4.357731	1.898170	1.485131
C	4.122840	-0.500497	1.216734
C	5.733635	1.773783	1.368325
H	3.915830	2.878349	1.655129
C	5.495722	-0.633538	1.085086
H	3.478795	-1.380169	1.178123
C	6.285751	0.511774	1.156015
H	6.385304	2.643470	1.439952
H	5.960726	-1.606532	0.934056
N	7.737467	0.383827	1.020765
O	8.195487	-0.723384	0.842538
O	8.405801	1.391067	1.094167

CF3_Michael_Acceptor_TS-MA-conf/TS-MA-R-xix

Frequencies, energies and thermodynamic properties:

Lowest Vibrational Mode (1/cm) = -126.3993

2nd Lowest Vibrational Mode (1/cm) = 16.1802

E(RM062X) (a.u.) = -3187.93115188

Thermal correction to Enthalpy (a.u.) = 0.680938

Thermal correction to Gibbs Free Energy (a.u.) = 0.556200

Total Entropy (cal/Kmol) = 262.533

Esp(RM062X) (a.u.) = -3190.89369923

Optimised cartesian coordinates (Angstrom):

C	2.551322	5.282020	1.137323
C	1.177194	5.500274	1.321785
C	0.277451	4.441857	1.296546
C	0.786422	3.160890	1.074842
C	2.151761	2.947954	0.884343
H	0.809619	6.513658	1.486874
H	-0.792288	4.602492	1.439662
C	1.362578	0.801117	0.638487
C	2.758891	-1.165418	0.145806
C	3.731089	-0.134012	-0.452035
C	3.794885	1.115081	0.402522
H	4.336505	1.906642	-0.129999
S	-0.112684	1.663823	0.971717
N	2.433520	1.600165	0.635550
N	1.443480	-0.525602	0.395352
C	5.126214	-0.710987	-0.780284
C	6.132844	-0.626953	0.370044
C	5.689423	-0.015551	-2.020299
H	4.977840	-1.774746	-1.029307
H	7.043777	-1.182621	0.104088
H	6.432056	0.416888	0.555403
H	5.747738	-1.045730	1.310693
H	5.784943	1.069200	-1.852409
H	6.688290	-0.405233	-2.264641
H	5.039918	-0.165493	-2.895147
H	3.275584	0.172673	-1.404492
H	2.594168	-1.898633	-0.651659
C	3.258027	-1.887449	1.379690
C	3.173252	-1.312713	2.652597
C	3.871213	-3.136219	1.233702
C	3.723610	-1.963854	3.756445
H	2.670149	-0.353075	2.798176
C	4.423203	-3.786102	2.336376
H	3.918279	-3.601371	0.245758
C	4.356674	-3.197147	3.599368
H	3.652678	-1.506628	4.744651
H	4.900567	-4.759082	2.209303
H	4.786255	-3.705387	4.464175
C	0.304255	-2.697032	0.013519
H	1.237565	-3.217552	-0.185723
C	-0.893843	-3.304372	-0.226997
C	-0.920484	-4.783009	-0.499663
F	-0.546833	-5.461877	0.597383
F	-2.126161	-5.229474	-0.831140
F	-0.070656	-5.132667	-1.469462
O	-0.811187	-0.711195	0.574154
C	0.237448	-1.296391	0.360371
H	4.272595	0.940586	1.378672
C	3.060603	4.008310	0.917587
H	4.128436	3.850910	0.771267
H	3.237320	6.129545	1.160892
H	-1.837350	-2.871618	0.113869
S	-1.744868	-2.552147	-2.522845
C	-2.780911	-1.367002	-1.804787
N	-3.770571	-1.728468	-1.047191

C -4.527761 -0.855682 -0.289802
C -3.952439 0.104229 0.578043
C -5.934446 -0.975768 -0.319526
C -4.751852 0.916788 1.366800
H -2.865740 0.170987 0.637870
C -6.739477 -0.160264 0.459830
H -6.375767 -1.727589 -0.974724
C -6.138184 0.781821 1.294145
H -4.315509 1.650112 2.043911
H -7.825047 -0.244104 0.432724
N -6.981259 1.644089 2.119036
O -8.182043 1.495238 2.055674
O -6.436821 2.466619 2.823603
N -2.614511 0.000313 -2.098423
C -1.490326 0.782168 -2.256680
C -0.189003 0.294164 -2.503981
C -1.666870 2.183081 -2.128620
C 0.888287 1.164200 -2.523882
H -0.037761 -0.768641 -2.670592
C -0.589435 3.049732 -2.137442
H -2.673437 2.576528 -1.970700
C 0.694795 2.525130 -2.296918
H 1.890727 0.789529 -2.723685
H -0.727297 4.120315 -1.986992
N 1.854891 3.392222 -2.162841
O 2.952978 2.866383 -2.112694
O 1.674782 4.585280 -2.078249
H -3.440110 0.551827 -1.876724

CF3_Michael_Acceptor_TS-MA-conf/TS-MA-R-x
Frequencies, energies and thermodynamic properties:
Lowest Vibrational Mode (1/cm) = -193.2876
2nd Lowest Vibrational Mode (1/cm) = 9.6698
E(RM062X) (a.u.) = -3187.93221907
Thermal correction to Enthalpy (a.u.) = 0.680603
Thermal correction to Gibbs Free Energy (a.u.) = 0.552822
Total Entropy (cal/Kmol) = 268.936
Esp(RM062X) (a.u.) = -3190.89592756
Optimised cartesian coordinates (Angstrom):
C -0.183871 7.032676 -1.058549
C -1.540101 7.044240 -0.705485
C -2.164643 5.893206 -0.233545
C -1.400476 4.732480 -0.123198
C -0.052504 4.722213 -0.485428
H -2.114537 7.966687 -0.797072
H -3.219183 5.899048 0.045926
C -0.300588 2.540946 0.212305
C 1.525168 0.938336 0.358677
C 2.078305 1.680232 -0.868137
C 1.914433 3.176981 -0.681153
H 2.116586 3.710884 -1.619199
S -1.925553 3.150498 0.434183
N 0.525871 3.461098 -0.305929
N 0.107737 1.313737 0.546451
C 3.514947 1.260935 -1.252120
C 4.619887 2.051856 -0.546549
C 3.700925 1.357124 -2.766921
H 3.610692 0.195757 -0.972411
H 4.663783 3.085453 -0.925522
H 4.494830 2.092962 0.544307
H 5.595561 1.589042 -0.754605
H 2.967091 0.737813 -3.302604
H 3.586319 2.397937 -3.111242

H	4.707787	1.020631	-3.055676
H	1.419299	1.373059	-1.698319
H	1.514750	-0.120637	0.084405
C	2.322400	1.102419	1.636174
C	3.270681	0.129261	1.971207
C	2.168501	2.217036	2.467132
C	4.073951	0.281624	3.100088
H	3.380179	-0.753900	1.336144
C	2.971750	2.369936	3.597506
H	1.414079	2.976496	2.247280
C	3.930433	1.406896	3.912492
H	4.810225	-0.484473	3.348465
H	2.842857	3.244271	4.237580
H	4.556583	1.527076	4.798068
C	-0.329914	-0.888173	1.512726
H	0.734013	-1.009249	1.695562
C	-1.172911	-2.006279	1.628673
C	-0.786295	-3.019203	2.676121
F	0.518290	-3.317276	2.633601
F	-1.034007	-2.535066	3.897872
F	-1.457567	-4.167782	2.575704
O	-2.038080	0.674336	0.997724
C	-0.860273	0.335958	1.041432
H	2.577718	3.575031	0.101960
C	0.580435	5.874226	-0.954504
H	1.636652	5.873839	-1.222648
H	0.286618	7.947870	-1.419896
H	-2.251788	-1.819815	1.617702
S	-1.065438	-3.363645	-0.136288
C	-0.867702	-1.981753	-1.207093
N	-1.958100	-1.167504	-1.435712
H	-1.682847	-0.276957	-1.844973
C	-3.320146	-1.295860	-1.189260
C	-4.056767	-0.097725	-1.091120
C	-4.000918	-2.524814	-1.104621
C	-5.426654	-0.117147	-0.896763
H	-3.526466	0.853255	-1.144654
C	-5.373610	-2.547539	-0.896348
H	-3.461026	-3.460304	-1.216031
C	-6.071214	-1.348217	-0.791856
H	-5.997270	0.806384	-0.813146
H	-5.909701	-3.493443	-0.833869
N	-7.519096	-1.379216	-0.582336
O	-8.052659	-2.459716	-0.464403
O	-8.107920	-0.321602	-0.539068
N	0.228132	-1.556670	-1.737630
C	1.497710	-2.103136	-1.632082
C	2.078472	-2.611061	-0.449340
C	2.297454	-2.033577	-2.793214
C	3.403136	-3.029702	-0.436057
H	1.490351	-2.673406	0.462911
C	3.617122	-2.457100	-2.791780
H	1.848475	-1.629764	-3.701433
C	4.156618	-2.947454	-1.604314
H	3.857918	-3.414996	0.475868
H	4.231754	-2.404739	-3.689347
N	5.555866	-3.380910	-1.583086
O	6.190187	-3.296007	-2.611012
O	6.004575	-3.798653	-0.539154

CF3_Michael_Acceptor_TS-MA-conf/TS-MA-R-xviii

Frequencies, energies and thermodynamic properties:

Lowest Vibrational Mode (1/cm) = -148.6875

2nd Lowest Vibrational Mode (1/cm) = 6.2819

E(RM062X) (a.u.) = -3187.93009767

Thermal correction to Enthalpy (a.u.) = 0.680889

Thermal correction to Gibbs Free Energy (a.u.) = 0.552791

Total Entropy (cal/Kmol) = 269.604

Esp(RM062X) (a.u.) = -3190.89200629

Optimised cartesian coordinates (Angstrom):

C	-6.954814	-4.161836	-0.695958
C	-6.112165	-5.239794	-1.002791
C	-4.729668	-5.085778	-1.007449
C	-4.211177	-3.827081	-0.703939
C	-5.052827	-2.754306	-0.408053
H	-6.544252	-6.213202	-1.237495
H	-4.069769	-5.922725	-1.239867
C	-3.000211	-1.732985	-0.188210
C	-2.663860	0.619024	0.439652
C	-4.059580	0.823872	-0.180395
C	-4.977958	-0.321544	0.198349
H	-5.913515	-0.266156	-0.371718
S	-2.522269	-3.360270	-0.608418
N	-4.329154	-1.588681	-0.136518
N	-2.151745	-0.726049	0.078832
C	-4.694984	2.201670	0.121121
C	-5.457401	2.281874	1.448093
C	-5.629606	2.604721	-1.021768
H	-3.866524	2.929959	0.146740
H	-6.393611	1.703384	1.397248
H	-4.880910	1.923047	2.311201
H	-5.736387	3.327429	1.644259
H	-6.068623	3.593540	-0.823584
H	-5.096386	2.656605	-1.979661
H	-6.460395	1.886886	-1.118551
H	-3.900636	0.770381	-1.271020
H	-2.002549	1.337691	-0.061847
C	-2.583196	0.852462	1.933606
C	-2.161629	2.101173	2.402140
C	-2.964841	-0.129675	2.852501
C	-2.154885	2.377554	3.767763
H	-1.834882	2.861703	1.687417
C	-2.957482	0.146047	4.220082
H	-3.268127	-1.122842	2.510260
C	-2.560565	1.401819	4.679612
H	-1.824910	3.355497	4.121934
H	-3.258873	-0.626586	4.929236
H	-2.553531	1.615593	5.749653
C	0.140736	0.032242	0.593068
H	-0.170221	1.068490	0.694628
C	1.452091	-0.327964	0.843241
C	2.253142	0.589142	1.732094
F	3.565749	0.364483	1.716936
F	2.063783	1.878057	1.436390
F	1.857687	0.424403	3.003907
O	-0.375135	-2.136358	-0.154368
C	-0.732234	-1.007176	0.144972
H	-5.220905	-0.325748	1.272018
C	-6.440899	-2.905354	-0.392749
H	-7.102416	-2.075435	-0.146663
H	-8.035536	-4.308255	-0.691351
H	1.677096	-1.384229	1.004065
S	2.843335	-0.502072	-1.070882
C	3.570355	1.089869	-1.172180
N	4.926016	1.237309	-0.926945
H	5.176451	2.215174	-0.794437

C 5.934594 0.330547 -0.640183
C 6.989305 0.786805 0.177095
C 5.996114 -0.975068 -1.164804
C 8.060035 -0.034362 0.487408
H 6.947885 1.801138 0.577558
C 7.062239 -1.804264 -0.848293
H 5.215639 -1.332460 -1.830472
C 8.079084 -1.330497 -0.022832
H 8.870828 0.313132 1.125792
H 7.119944 -2.814045 -1.252160
N 9.200780 -2.209259 0.306391
O 9.187631 -3.336857 -0.135407
O 10.084874 -1.763265 1.004200
N 2.957312 2.214929 -1.358079
C 1.619242 2.353053 -1.641139
C 0.914788 3.382589 -0.976704
C 0.934014 1.589697 -2.615684
C -0.423465 3.623385 -1.238564
H 1.453531 3.984385 -0.244371
C -0.405460 1.819133 -2.880757
H 1.468836 0.813939 -3.159942
C -1.073563 2.829161 -2.184814
H -0.966229 4.419902 -0.730411
H -0.937057 1.237563 -3.633045
N -2.475750 3.085288 -2.479039
O -3.059202 2.309180 -3.210952
O -2.995394 4.063243 -1.984146

CF3_Michael_Acceptor_TS-MA-conf/TS-MA-R-xvii

Frequencies, energies and thermodynamic properties:

Lowest Vibrational Mode (1/cm) = -127.1767
2nd Lowest Vibrational Mode (1/cm) = 7.8233
E(RM062X) (a.u.) = -3187.92885559
Thermal correction to Enthalpy (a.u.) = 0.680578
Thermal correction to Gibbs Free Energy (a.u.) = 0.552275
Total Entropy (cal/Kmol) = 270.035
Esp(RM062X) (a.u.) = -3190.89178255

Optimised cartesian coordinates (Angstrom):

C 7.334890 1.487493 -1.941947
C 6.856328 2.769959 -1.634140
C 5.577137 2.950553 -1.119115
C 4.789651 1.815311 -0.924964
C 5.266428 0.539682 -1.231978
H 7.495614 3.638095 -1.798503
H 5.186468 3.938429 -0.870005
C 3.170607 0.016157 -0.440437
C 2.206123 -2.250427 -0.367741
C 3.213957 -2.534923 -1.495864
C 4.545601 -1.873135 -1.193843
H 5.223785 -1.960261 -2.051477
S 3.165301 1.751441 -0.272039
N 4.314746 -0.448211 -0.952305
N 2.138031 -0.789936 -0.120968
C 3.353904 -4.035066 -1.841459
C 4.422258 -4.773717 -1.030320
C 3.629685 -4.198688 -3.336444
H 2.378351 -4.501774 -1.628556
H 5.433439 -4.445945 -1.319808
H 4.314053 -4.633720 0.054419
H 4.360518 -5.852160 -1.236314
H 2.814287 -3.780042 -3.943975
H 4.563752 -3.688866 -3.622233
H 3.738459 -5.261602 -3.596487

H	2.801416	-2.029558	-2.384361
H	1.227795	-2.536515	-0.771222
C	2.441270	-3.018245	0.917567
C	1.760660	-4.224850	1.115515
C	3.354764	-2.585265	1.884780
C	2.014726	-5.005313	2.241627
H	1.025122	-4.555924	0.377991
C	3.608004	-3.365493	3.013462
H	3.875042	-1.630505	1.772005
C	2.945760	-4.580245	3.190082
H	1.478292	-5.945068	2.382431
H	4.323633	-3.017975	3.760321
H	3.144857	-5.188494	4.073907
C	-0.154904	-1.058155	0.785939
H	-0.175323	-2.123249	0.571113
C	-1.270173	-0.424138	1.264145
C	-2.353360	-1.247152	1.902450
F	-2.763517	-2.259483	1.136927
F	-1.900031	-1.789732	3.043811
F	-3.425969	-0.525494	2.236647
O	0.995519	0.987828	0.718322
C	0.971542	-0.208376	0.492071
H	5.037454	-2.304588	-0.309002
C	6.552219	0.354588	-1.745338
H	6.935395	-0.638129	-1.980213
H	8.343274	1.371471	-2.341067
H	-1.194101	0.599164	1.636211
S	-2.698323	0.441241	-0.614352
C	-3.574210	1.667751	0.265527
N	-4.854330	1.829145	0.337012
C	-5.798132	0.981972	-0.207523
C	-6.858224	1.564761	-0.934303
C	-5.810449	-0.416653	-0.007819
C	-7.878999	0.791677	-1.464890
H	-6.855535	2.646731	-1.071535
C	-6.834671	-1.195811	-0.522951
H	-5.009725	-0.880971	0.563086
C	-7.854704	-0.585669	-1.252825
H	-8.692763	1.239135	-2.033765
H	-6.853744	-2.273583	-0.367018
N	-8.928905	-1.409882	-1.802039
O	-8.882835	-2.606153	-1.612795
O	-9.812592	-0.856452	-2.420096
N	-2.840991	2.553240	1.058310
C	-1.577646	3.090776	0.871353
C	-0.941396	3.158899	-0.383786
C	-0.929091	3.639419	1.997232
C	0.318265	3.723136	-0.497133
H	-1.439980	2.763846	-1.265258
C	0.327819	4.206975	1.887790
H	-1.425741	3.595346	2.968091
C	0.946671	4.233335	0.638073
H	0.817743	3.780154	-1.463499
H	0.838716	4.615604	2.758540
N	2.288887	4.783750	0.525152
O	2.784791	5.302688	1.498618
O	2.860768	4.681213	-0.547107
H	-3.397679	2.983941	1.793539

CF3_Michael_Acceptor_TS-MA-conf/TS-MA-R-xvi

Frequencies, energies and thermodynamic properties:

Lowest Vibrational Mode (1/cm) = -149.4170

2nd Lowest Vibrational Mode (1/cm) = 13.0627

E(RM062X) (a.u.) = -3187.93072656

Thermal correction to Enthalpy (a.u.) = 0.680763

Thermal correction to Gibbs Free Energy (a.u.) = 0.553534

Total Entropy (cal/Kmol) = 267.774

Esp(RM062X) (a.u.) = -3190.89294073

Optimised cartesian coordinates (Angstrom):

C	7.579373	-1.241955	1.373229
C	7.123592	-2.544656	1.120759
C	5.799762	-2.782491	0.765708
C	4.945190	-1.683574	0.677287
C	5.398542	-0.388700	0.933206
H	7.816815	-3.383010	1.198467
H	5.424182	-3.785860	0.557644
C	3.205263	0.043436	0.391135
C	2.164612	2.267782	0.388931
C	3.240720	2.592412	1.440752
C	4.572682	1.983444	1.041908
H	5.298198	2.073126	1.861055
S	3.252984	-1.690689	0.222980
N	4.377947	0.556755	0.779150
N	2.116740	0.803890	0.173030
C	3.345020	4.097648	1.777316
C	4.325814	4.877135	0.896768
C	3.714852	4.274840	3.250151
H	2.339337	4.524705	1.630892
H	5.366935	4.597363	1.123532
H	4.155828	4.723769	-0.178255
H	4.228356	5.953323	1.100768
H	2.959689	3.826162	3.911680
H	4.685008	3.801221	3.470231
H	3.800626	5.341550	3.503244
H	2.912995	2.071512	2.355474
H	1.208510	2.538703	0.852139
C	2.300803	3.012053	-0.923854
C	1.584227	4.198887	-1.110345
C	3.162606	2.569751	-1.933147
C	1.751940	4.951618	-2.271186
H	0.889361	4.536110	-0.337067
C	3.329669	3.322045	-3.096066
H	3.707635	1.628182	-1.826075
C	2.631305	4.517820	-3.263949
H	1.188138	5.876375	-2.404143
H	4.005143	2.967750	-3.876394
H	2.761730	5.104175	-4.174941
C	-0.236486	0.991887	-0.575502
H	-0.276067	2.056297	-0.359114
C	-1.367396	0.318455	-0.973716
C	-2.520290	1.120405	-1.513664
F	-2.869021	2.127223	-0.707644
F	-2.185841	1.669667	-2.690610
F	-3.611089	0.385958	-1.739778
O	0.955435	-1.029307	-0.510739
C	0.919471	0.175606	-0.332107
H	4.997729	2.452975	0.142016
C	6.728365	-0.145090	1.281816
H	7.092395	0.865533	1.465513
H	8.624080	-1.080870	1.642083
H	-1.275234	-0.688283	-1.388917
S	-2.527003	-0.644665	0.933266
C	-3.366576	-1.886325	0.039840
N	-4.750986	-1.871637	-0.070493
H	-5.084075	-2.724747	-0.514923
C	-5.756545	-0.994817	0.292195

C -7.071810 -1.513660 0.236957
C -5.583091 0.354975 0.664755
C -8.169465 -0.733943 0.549905
H -7.216400 -2.555581 -0.055496
C -6.684399 1.136585 0.983958
H -4.589954 0.790085 0.697234
C -7.964043 0.591992 0.928926
H -9.178786 -1.140607 0.509116
H -6.554938 2.180242 1.267365
N -9.114284 1.425925 1.264824
O -8.908186 2.571074 1.603158
O -10.217250 0.929480 1.188086
N -2.819809 -2.868270 -0.607382
C -1.503889 -3.263553 -0.526278
C -0.808696 -3.428381 0.693082
C -0.847172 -3.618184 -1.726047
C 0.493791 -3.897864 0.707316
H -1.308595 -3.180875 1.628399
C 0.457068 -4.079612 -1.722172
H -1.393713 -3.511127 -2.664049
C 1.117838 -4.214496 -0.500599
H 1.035715 -4.021552 1.644281
H 0.970203 -4.335190 -2.648286
N 2.501813 -4.658855 -0.493041
O 3.010367 -4.992110 -1.539879
O 3.101036 -4.652253 0.569705

CF3_Michael_Acceptor_TS-MA-conf/TS-MA-R-xv

Frequencies, energies and thermodynamic properties:

Lowest Vibrational Mode (1/cm) = -182.1758
2nd Lowest Vibrational Mode (1/cm) = 11.7370
E(RM062X) (a.u.) = -3187.93347488
Thermal correction to Enthalpy (a.u.) = 0.680278
Thermal correction to Gibbs Free Energy (a.u.) = 0.553472
Total Entropy (cal/Kmol) = 266.887
Esp(RM062X) (a.u.) = -3190.89406859

Optimised cartesian coordinates (Angstrom):

C -6.751729 -1.334829 0.742888
C -7.056106 -0.263364 -0.107057
C -6.058667 0.361655 -0.850120
C -4.752565 -0.108412 -0.720162
C -4.449368 -1.163047 0.143170
H -8.085243 0.088255 -0.187401
H -6.288643 1.200156 -1.510352
C -2.343592 -0.655022 -0.646007
C -0.287512 -1.792423 0.026422
C -1.054192 -2.087747 1.324395
C -2.490623 -2.465513 1.020057
H -3.079976 -2.502606 1.945195
S -3.314267 0.508991 -1.513057
N -3.078215 -1.439475 0.159090
N -1.016304 -0.774467 -0.755402
C -0.344672 -3.095169 2.254914
C -0.701997 -4.560759 1.994705
C -0.628246 -2.738038 3.714521
H 0.738602 -2.972688 2.084220
H -1.740839 -4.772369 2.293985
H -0.583048 -4.850837 0.941133
H -0.052732 -5.212246 2.597593
H -0.273816 -1.725025 3.956842
H -1.709295 -2.778925 3.924822
H -0.131258 -3.445998 4.393668
H -1.077892 -1.118649 1.848353

H	0.641859	-1.310066	0.348942
C	0.058381	-2.998628	-0.820563
C	1.327270	-3.574676	-0.701880
C	-0.876966	-3.583007	-1.681764
C	1.647402	-4.735925	-1.403717
H	2.071480	-3.106553	-0.052464
C	-0.557305	-4.743547	-2.386144
H	-1.864109	-3.132883	-1.815188
C	0.702107	-5.326874	-2.242423
H	2.640921	-5.175497	-1.301406
H	-1.294877	-5.191107	-3.054359
H	0.951199	-6.234177	-2.795244
C	1.125102	0.123134	-1.575870
H	1.694904	-0.663703	-1.091788
C	1.787742	1.311882	-1.971313
C	3.231805	1.161449	-2.379052
F	3.929751	0.394100	-1.535780
F	3.301910	0.575431	-3.581878
F	3.876199	2.324437	-2.484168
O	-0.960891	1.064851	-2.106928
C	-0.280987	0.201328	-1.554094
H	-2.569800	-3.440534	0.515643
C	-5.446906	-1.801001	0.882212
H	-5.219350	-2.636280	1.544225
H	-7.549456	-1.814790	1.311112
H	1.249729	1.932897	-2.693575
S	1.808788	2.802789	-0.438218
C	1.321857	1.731524	0.878155
N	0.144909	1.516324	1.361676
C	-1.087982	2.086277	1.084967
C	-2.157944	1.548855	1.843153
C	-1.387551	3.113291	0.161749
C	-3.463744	1.983076	1.685759
H	-1.924456	0.780504	2.581435
C	-2.693329	3.559313	0.000787
H	-0.598523	3.555017	-0.438559
C	-3.714506	2.987413	0.753439
H	-4.281986	1.558708	2.266668
H	-2.928134	4.340226	-0.721312
N	-5.096056	3.419959	0.533849
O	-5.307108	4.219385	-0.351393
O	-5.957191	2.949485	1.244043
N	2.284187	0.879940	1.390184
C	3.671766	0.836353	1.304186
C	4.490395	1.958576	1.082005
C	4.273209	-0.421409	1.505689
C	5.867475	1.808798	1.006485
H	4.052449	2.945732	0.967581
C	5.647824	-0.573443	1.441391
H	3.638849	-1.288725	1.699311
C	6.431101	0.547485	1.177322
H	6.511366	2.669186	0.830002
H	6.116303	-1.546147	1.582794
N	7.884563	0.397071	1.094104
O	8.350395	-0.706513	1.272513
O	8.543689	1.383229	0.852344
H	1.868283	0.150523	1.966577

CF3_Michael_Acceptor_TS-MA-conf/TS-MA-R-xxiii

Frequencies, energies and thermodynamic properties:

Lowest Vibrational Mode (1/cm) =	-106.1505
2nd Lowest Vibrational Mode (1/cm) =	9.1193
E(RM062X) (a.u.) =	-3187.91489894

Thermal correction to Enthalpy (a.u.) = 0.680869

Thermal correction to Gibbs Free Energy (a.u.) = 0.549440

Total Entropy (cal/Kmol) = 276.615

Esp(RM062X) (a.u.) = -3190.88530730

Optimised cartesian coordinates (Angstrom):

C	-6.669691	3.760229	-1.102498
C	-5.721976	4.483462	-1.841916
C	-4.430699	3.998185	-2.016036
C	-4.111719	2.773157	-1.430412
C	-5.051346	2.062386	-0.682816
H	-6.000216	5.438464	-2.288821
H	-3.688694	4.555278	-2.589358
C	-3.247964	0.642940	-0.524729
C	-3.270341	-1.515771	0.648410
C	-4.331669	-0.839787	1.531964
C	-5.289800	-0.033545	0.677637
H	-5.941678	0.582493	1.310088
S	-2.580361	1.925723	-1.498708
N	-4.516754	0.872691	-0.175176
N	-2.591095	-0.481169	-0.171032
C	-5.057542	-1.814063	2.488019
C	-6.319988	-2.452277	1.902318
C	-5.394664	-1.097137	3.795717
H	-4.342847	-2.620111	2.721374
H	-6.690976	-3.228935	2.586618
H	-7.123406	-1.706757	1.791245
H	-6.150704	-2.922326	0.923204
H	-6.048833	-0.230217	3.609132
H	-5.924087	-1.772332	4.483434
H	-4.487667	-0.735908	4.301609
H	-3.771412	-0.122545	2.153803
H	-2.511548	-1.900279	1.339378
C	-3.775521	-2.656327	-0.210230
C	-4.401685	-2.432950	-1.441125
C	-3.652673	-3.966133	0.266037
C	-4.927103	-3.502037	-2.167161
H	-4.480794	-1.422958	-1.852334
C	-4.178616	-5.034058	-0.458645
H	-3.139996	-4.148884	1.214016
C	-4.823788	-4.802742	-1.674032
H	-5.414739	-3.316663	-3.125581
H	-4.077323	-6.050964	-0.076227
H	-5.233684	-5.637860	-2.244335
C	-0.602937	-1.937599	-0.311953
H	-1.130819	-2.755612	0.171165
C	0.726509	-2.054913	-0.583241
C	1.365321	-3.415250	-0.563297
F	0.990076	-4.095476	-1.662665
F	2.690631	-3.373670	-0.582207
F	0.981399	-4.147582	0.482707
O	-0.698390	0.248219	-1.197628
C	-1.237557	-0.670838	-0.606251
H	-5.917852	-0.668063	0.034479
C	-6.351990	2.541973	-0.512259
H	-7.096675	1.984713	0.055339
H	-7.678349	4.158276	-0.986008
H	1.244500	-1.308694	-1.187227
S	1.922292	-1.175869	1.519130
C	3.504332	-0.567421	1.146012
N	4.510626	-1.358696	0.940599
C	5.733493	-0.950969	0.457420
C	5.878298	-0.114694	-0.677585
C	6.905384	-1.463005	1.060634

C 7.132317 0.201890 -1.174208
H 4.987222 0.269114 -1.176130
C 8.162514 -1.141937 0.576399
H 6.794727 -2.122633 1.921980
C 8.264048 -0.308850 -0.538124
H 7.246833 0.837129 -2.051578
H 9.065610 -1.530048 1.045512
N 9.585874 0.031008 -1.056291
O 10.554683 -0.430053 -0.492240
O 9.648649 0.758989 -2.023669
N 3.718884 0.817592 1.181881
C 2.837428 1.874432 1.052292
C 1.623075 1.778420 0.343290
C 3.207990 3.121151 1.598207
C 0.795897 2.880747 0.219506
H 1.327426 0.841340 -0.117370
C 2.386346 4.229843 1.467726
H 4.153067 3.205700 2.137985
C 1.177763 4.095525 0.786782
H -0.140569 2.804252 -0.330354
H 2.666918 5.191365 1.895308
N 0.296119 5.250998 0.651750
O -0.758027 5.097371 0.072007
O 0.660906 6.304870 1.125797
H 4.679889 1.092209 1.375021

CF3_Michael_Acceptor_TS-MA-conf/TS-MA-R-xxii

Frequencies, energies and thermodynamic properties:

Lowest Vibrational Mode (1/cm) = -135.3890

2nd Lowest Vibrational Mode (1/cm) = 13.6187

E(RM062X) (a.u.) = -3187.92987472

Thermal correction to Enthalpy (a.u.) = 0.680795

Thermal correction to Gibbs Free Energy (a.u.) = 0.554807

Total Entropy (cal/Kmol) = 265.163

Esp(RM062X) (a.u.) = -3190.89123648

Optimised cartesian coordinates (Angstrom):

C 6.612791 4.759427 -0.606570
C 5.666309 5.786808 -0.730305
C 4.303760 5.510192 -0.688478
C 3.911106 4.181580 -0.526068
C 4.855295 3.160678 -0.413382
H 6.000995 6.817040 -0.856565
H 3.563407 6.306489 -0.777835
C 2.911157 1.938320 -0.223628
C 2.802131 -0.509185 0.054080
C 4.185144 -0.484844 -0.629942
C 5.009242 0.674427 -0.103907
H 5.934471 0.778118 -0.683792
S 2.275657 3.555768 -0.412424
N 4.247132 1.910973 -0.258641
N 2.168285 0.832077 -0.039079
C 4.967166 -1.818865 -0.555431
C 5.828337 -1.985739 0.701013
C 5.842928 -1.976817 -1.799007
H 4.227754 -2.634017 -0.574182
H 6.698339 -1.310223 0.676636
H 5.279883 -1.802655 1.635295
H 6.218929 -3.013036 0.739730
H 6.413111 -2.916077 -1.746631
H 5.231421 -2.000756 -2.710241
H 6.570014 -1.152419 -1.879960
H 3.980825 -0.285032 -1.694629
H 2.173729 -1.173411 -0.554671

C	2.816291	-1.033076	1.474223
C	2.591203	-2.398032	1.683057
C	3.098997	-0.210384	2.567569
C	2.677077	-2.939752	2.963582
H	2.350145	-3.041362	0.831501
C	3.183035	-0.752288	3.850831
H	3.250404	0.863204	2.427903
C	2.979298	-2.117545	4.050416
H	2.501226	-4.006093	3.114198
H	3.404278	-0.102176	4.698904
H	3.043551	-2.539062	5.054867
C	-0.024492	-0.142064	0.582618
H	0.369129	-1.155597	0.600725
C	-1.323454	0.118632	0.985088
C	-1.946854	-0.858300	1.949441
F	-3.275029	-0.778695	2.046536
F	-1.647437	-2.128794	1.668315
F	-1.469052	-0.615491	3.179748
O	0.277206	2.104589	-0.033345
C	0.737440	0.986917	0.145676
H	5.270462	0.554521	0.958396
C	6.224655	3.433591	-0.445109
H	6.966884	2.642720	-0.339276
H	7.676019	5.000868	-0.634020
H	-1.584710	1.153841	1.212723
S	-2.982915	0.327959	-0.731150
C	-3.849892	-1.192892	-0.816130
N	-3.128208	-2.381440	-0.858960
H	-3.670862	-3.191265	-0.567092
C	-1.819184	-2.622446	-1.225852
C	-1.182737	-3.758559	-0.685445
C	-1.114134	-1.809469	-2.136584
C	0.143646	-4.030583	-0.969278
H	-1.737196	-4.402067	-0.000757
C	0.213775	-2.073887	-2.420386
H	-1.607393	-0.961627	-2.603089
C	0.838406	-3.164715	-1.814284
H	0.649006	-4.889448	-0.529318
H	0.770339	-1.444409	-3.113632
N	2.252670	-3.392561	-2.061359
O	2.841359	-2.597903	-2.767010
O	2.784940	-4.346352	-1.532063
N	-5.129999	-1.344432	-0.811818
C	-6.047695	-0.339803	-0.573958
C	-7.124385	-0.194563	-1.471771
C	-6.007053	0.481864	0.573568
C	-8.117574	0.747816	-1.252059
H	-7.159845	-0.842394	-2.348365
C	-7.003369	1.416087	0.808503
H	-5.183565	0.368196	1.276975
C	-8.045257	1.543472	-0.110590
H	-8.946970	0.870535	-1.947087
H	-6.984166	2.049421	1.694370
N	-9.093668	2.532724	0.134020
O	-9.016273	3.208089	1.137078
O	-9.987477	2.627570	-0.678841

CF3_Michael_Acceptor_TS-MA-conf/TS-MA-R-xxi

Frequencies, energies and thermodynamic properties:

Lowest Vibrational Mode (1/cm) =	-127.8148
2nd Lowest Vibrational Mode (1/cm) =	11.9306
E(RM062X) (a.u.) =	-3187.92890664
Thermal correction to Enthalpy (a.u.) =	0.680916

Thermal correction to Gibbs Free Energy (a.u.) = 0.554696

Total Entropy (cal/Kmol) = 265.653

Esp(RM062X) (a.u.) = -3190.89295423

Optimised cartesian coordinates (Angstrom):

C	7.188267	-2.228404	0.076349
C	7.083906	-2.591961	-1.274046
C	5.984598	-2.206699	-2.033766
C	4.992135	-1.454008	-1.408217
C	5.095454	-1.098221	-0.063274
H	7.874651	-3.183708	-1.736488
H	5.898314	-2.485023	-3.084906
C	3.066120	-0.142705	-0.586311
C	1.653003	1.129980	0.979068
C	2.424639	0.368152	2.069421
C	3.887047	0.209596	1.707223
H	4.372191	-0.488705	2.400197
S	3.520335	-0.830469	-2.122617
N	3.985177	-0.359966	0.361815
N	1.921861	0.528642	-0.350669
C	2.210224	0.931759	3.491778
C	3.194558	2.033006	3.893813
C	2.253670	-0.209526	4.508777
H	1.193634	1.358440	3.511237
H	4.208285	1.624565	4.032015
H	3.250179	2.848698	3.159130
H	2.887452	2.468788	4.855666
H	1.470106	-0.955306	4.308763
H	3.225633	-0.727300	4.481299
H	2.108355	0.173744	5.529216
H	2.004262	-0.646808	2.062638
H	0.596265	0.934087	1.195448
C	1.869324	2.629624	0.945582
C	0.957128	3.460263	1.604756
C	2.976812	3.202241	0.310455
C	1.167042	4.836884	1.662501
H	0.073780	3.023114	2.077141
C	3.186369	4.580082	0.365879
H	3.686214	2.577395	-0.239461
C	2.286847	5.399025	1.048661
H	0.448493	5.473838	2.180882
H	4.054513	5.015400	-0.131656
H	2.451166	6.477002	1.089814
C	-0.244950	1.406712	-1.160316
C	-1.022896	1.765596	-2.226497
C	-2.207975	2.651293	-1.944955
F	-3.040642	2.792752	-2.967210
F	-2.917558	2.220190	-0.893468
F	-1.780661	3.886353	-1.629157
O	1.374958	0.438714	-2.562030
C	1.019896	0.778241	-1.449363
H	4.437915	1.162699	1.718497
C	6.200021	-1.476402	0.702142
H	6.292423	-1.195429	1.750797
H	8.061324	-2.539448	0.651171
H	-0.583117	1.872257	-3.217992
S	-2.277714	-0.148786	-3.302267
C	-3.241333	-0.484601	-1.901673
N	-2.621893	-0.913967	-0.719498
N	-4.507963	-0.225202	-1.885457
C	-1.407637	-1.505351	-0.445295
C	-0.542011	-2.071677	-1.406803
C	-1.045170	-1.588311	0.921754
C	0.672058	-2.614830	-1.016615

H -0.815485 -2.054030 -2.456874
C 0.159746 -2.140317 1.311299
H -1.731008 -1.199962 1.678268
C 1.032292 -2.618885 0.331102
H 1.350464 -3.035667 -1.757909
H 0.435120 -2.195959 2.364081
N 2.360047 -3.071020 0.715873
O 2.684597 -2.973162 1.884635
O 3.094439 -3.495441 -0.151195
C -5.351127 -0.547770 -0.843341
C -5.498962 -1.869807 -0.360817
C -6.155790 0.467575 -0.280133
C -6.408513 -2.161881 0.643826
H -4.899132 -2.668308 -0.800410
C -7.060089 0.184289 0.730786
H -6.044559 1.483711 -0.660289
C -7.176419 -1.130233 1.182793
H -6.533124 -3.178672 1.013990
H -7.677716 0.964942 1.172930
N -8.129493 -1.435488 2.248036
O -8.794597 -0.526104 2.693879
O -8.204100 -2.582088 2.633034
H -0.626369 1.517764 -0.148462
H -3.188663 -0.760402 0.111393

CF3_Michael_Acceptor_TS-MA-conf/TS-MA-R-xxiv
Frequencies, energies and thermodynamic properties:
Lowest Vibrational Mode (1/cm) = -124.2677
2nd Lowest Vibrational Mode (1/cm) = 14.8989
E(RM062X) (a.u.) = -3187.93316292
Thermal correction to Enthalpy (a.u.) = 0.680557
Thermal correction to Gibbs Free Energy (a.u.) = 0.555180
Total Entropy (cal/Kmol) = 263.879
Esp(RM062X) (a.u.) = -3190.89754500
Optimised cartesian coordinates (Angstrom):
C -0.967582 5.951171 -1.001928
C -0.286409 5.694273 -2.199836
C -0.176298 4.398724 -2.693292
C -0.746793 3.366074 -1.949098
C -1.407639 3.623166 -0.746634
H 0.162774 6.519257 -2.753732
H 0.353228 4.191900 -3.624391
C -1.553568 1.323016 -0.815736
C -2.583539 -0.077973 0.921728
C -2.335222 1.136887 1.834292
C -2.626695 2.429249 1.098790
H -2.310918 3.291262 1.699410
S -0.723373 1.651747 -2.313749
N -1.857358 2.443048 -0.144910
N -1.872369 0.097986 -0.367065
C -3.044666 1.049947 3.203911
C -4.457475 1.639296 3.222394
C -2.184610 1.725015 4.273144
H -3.114384 -0.020263 3.457620
H -4.427123 2.735394 3.116685
H -5.102434 1.236628 2.428667
H -4.936914 1.419952 4.187533
H -1.200580 1.241345 4.362257
H -2.021316 2.788165 4.033185
H -2.677067 1.678353 5.255323
H -1.253345 1.130415 2.027664
H -2.091004 -0.922879 1.418766
C -4.044565 -0.420422 0.706127

C -4.644159 -1.378638 1.530958
C -4.823777 0.232951 -0.254178
C -6.006469 -1.652294 1.425507
H -4.034822 -1.915728 2.262437
C -6.187444 -0.041731 -0.362143
H -4.372141 0.960079 -0.933459
C -6.783464 -0.977820 0.482810
H -6.461127 -2.400805 2.076523
H -6.784842 0.475791 -1.114587
H -7.849947 -1.192415 0.396757
C -1.851884 -2.344831 -0.645110
C -1.333873 -3.469011 -1.263515
C -2.096299 -4.753115 -1.075716
F -1.430292 -5.833401 -1.489538
F -2.441670 -4.960774 0.197176
F -3.233697 -4.717350 -1.784883
O -0.837639 -0.852914 -2.166524
C -1.468590 -1.065208 -1.140227
H -3.693570 2.544683 0.853706
C -1.540813 4.924546 -0.259637
H -2.064954 5.134778 0.672065
H -1.045486 6.975996 -0.636928
H -0.898315 -3.364632 -2.259406
S 0.668379 -4.274748 -0.335850
C 1.530910 -2.781620 -0.118357
N 2.861545 -2.860450 -0.523686
N 0.984756 -1.718761 0.380946
C 3.970373 -2.209607 -0.003148
C 3.962720 -1.566094 1.249567
C 5.158623 -2.230319 -0.758576
C 5.108670 -0.943296 1.719451
H 3.063642 -1.570449 1.863401
C 6.306123 -1.612218 -0.289884
H 5.165588 -2.734369 -1.726531
C 6.265255 -0.965605 0.943538
H 5.116077 -0.446498 2.688504
H 7.226859 -1.617872 -0.871303
N 7.469856 -0.301195 1.440206
O 8.471346 -0.362493 0.761976
O 7.402461 0.274703 2.503437
C 1.430579 -0.430368 0.169929
C 2.056448 0.007359 -1.021270
C 1.146453 0.533450 1.162143
C 2.349228 1.346540 -1.218297
H 2.280775 -0.711657 -1.808547
C 1.407344 1.880263 0.961440
H 0.692745 0.187765 2.092518
C 1.999470 2.275455 -0.238002
H 2.807308 1.689850 -2.145327
H 1.155917 2.626400 1.715364
N 2.203187 3.698061 -0.498808
O 1.752331 4.492300 0.298097
O 2.806976 4.012201 -1.501845
H -2.445666 -2.467709 0.254957
H 3.058588 -3.615489 -1.175123

CF3_Michael_Acceptor_TS-MA-conf/TS-MA-R-xx

Frequencies, energies and thermodynamic properties:

Lowest Vibrational Mode (1/cm) = -146.6720

2nd Lowest Vibrational Mode (1/cm) = 12.1869

E(RM062X) (a.u.) = -3187.93246125

Thermal correction to Enthalpy (a.u.) = 0.680836

Thermal correction to Gibbs Free Energy (a.u.) = 0.555957

Total Entropy (cal/Kmol) = 262.830

Esp(RM062X) (a.u.) = -3190.89586022

Optimised cartesian coordinates (Angstrom):

C	-5.182923	3.260798	-1.936029
C	-4.131548	4.151854	-2.192222
C	-2.807252	3.753084	-2.046020
C	-2.564301	2.451035	-1.609213
C	-3.613358	1.572666	-1.333502
H	-4.352260	5.169881	-2.514780
H	-1.983994	4.439314	-2.249282
C	-1.810303	0.241988	-0.793527
C	-1.966055	-1.985576	0.244998
C	-3.374300	-1.522311	0.656384
C	-4.033487	-0.752213	-0.471015
H	-4.974192	-0.302817	-0.130310
S	-1.008080	1.687982	-1.350153
N	-3.142483	0.337002	-0.872143
N	-1.188205	-0.842016	-0.292715
C	-4.263885	-2.655113	1.217127
C	-5.089265	-3.392402	0.159662
C	-5.184351	-2.096510	2.302768
H	-3.588024	-3.383393	1.694316
H	-5.590840	-4.256061	0.619835
H	-5.876603	-2.740016	-0.250398
H	-4.481955	-3.764726	-0.677338
H	-5.839700	-1.309229	1.896532
H	-5.830096	-2.887962	2.709956
H	-4.608700	-1.664285	3.134296
H	-3.216424	-0.801702	1.473535
H	-1.473347	-2.273717	1.181148
C	-1.931395	-3.161581	-0.709620
C	-2.097202	-2.992826	-2.088489
C	-1.784214	-4.453241	-0.193047
C	-2.150497	-4.102296	-2.932314
H	-2.180730	-1.991586	-2.519157
C	-1.839731	-5.562161	-1.035543
H	-1.623861	-4.590146	0.879453
C	-2.030697	-5.388916	-2.406876
H	-2.281629	-3.959031	-4.006124
H	-1.724881	-6.564750	-0.620408
H	-2.071045	-6.256040	-3.068151
C	0.900065	-1.987651	0.352102
H	0.339290	-2.779884	0.838230
C	2.278435	-2.022902	0.401701
C	2.922106	-3.363075	0.634363
F	2.810078	-4.115848	-0.471607
F	4.222945	-3.291514	0.908536
F	2.337448	-4.045150	1.622213
O	0.830850	0.052487	-0.836451
C	0.256439	-0.877486	-0.282471
H	-4.242151	-1.381340	-1.349203
C	-4.943164	1.960015	-1.506770
H	-5.769009	1.276412	-1.312184
H	-6.213183	3.593284	-2.068222
H	2.860808	-1.350799	-0.234375
S	3.142280	-1.005839	2.438695
C	2.167411	0.365804	2.009282
N	0.958749	0.455561	2.476409
C	0.052352	1.460618	2.197736
C	0.348152	2.821511	1.939427
C	-1.310566	1.080754	2.236356
C	-0.663511	3.721140	1.628130
H	1.377312	3.176907	1.978584

C -2.324939 1.966775 1.921126
H -1.529888 0.046807 2.508616
C -1.983362 3.278457 1.587672
H -0.436551 4.762381 1.401725
H -3.372326 1.662593 1.920837
N -3.036409 4.198384 1.161109
O -4.175590 3.786230 1.144290
O -2.714854 5.320609 0.837484
N 2.622363 1.229435 1.017990
C 3.847855 1.273948 0.365947
C 5.077693 1.114504 1.032537
C 3.844365 1.559175 -1.013406
C 6.268282 1.219838 0.330385
H 5.092466 0.915400 2.101184
C 5.033346 1.671444 -1.717576
H 2.889110 1.670674 -1.528941
C 6.234450 1.492988 -1.036216
H 7.226126 1.104391 0.835439
H 5.039158 1.882508 -2.785785
N 7.493591 1.602636 -1.773085
O 8.525126 1.425395 -1.163906
O 7.438611 1.865667 -2.954305
H 1.865421 1.632294 0.469054

CF3_Michael_Acceptor_TS-MA-conf/TS-MA-R-xxvii
Frequencies, energies and thermodynamic properties:
Lowest Vibrational Mode (1/cm) = -57.6559
2nd Lowest Vibrational Mode (1/cm) = 16.9159
E(RM062X) (a.u.) = -3187.92165898
Thermal correction to Enthalpy (a.u.) = 0.681381
Thermal correction to Gibbs Free Energy (a.u.) = 0.554798
Total Entropy (cal/Kmol) = 266.416
Esp(RM062X) (a.u.) = -3190.89108182
Optimised cartesian coordinates (Angstrom):
C -0.977195 6.464947 -1.031389
C -0.648447 6.290565 -2.384377
C -0.568486 5.020058 -2.942247
C -0.822262 3.928776 -2.110770
C -1.139513 4.103517 -0.763387
H -0.454648 7.163171 -3.008990
H -0.317826 4.878428 -3.994281
C -1.225078 1.810519 -0.905043
C -1.890802 0.325414 0.942799
C -1.321119 1.437543 1.837296
C -1.709602 2.800300 1.302132
H -1.160948 3.590542 1.829380
S -0.814549 2.233144 -2.545381
N -1.348629 2.879643 -0.116818
N -1.442928 0.550251 -0.459838
C -1.659764 1.263921 3.336031
C -2.990070 1.892684 3.761502
C -0.526306 1.825855 4.195611
H -1.715889 0.178280 3.521602
H -2.925485 2.992447 3.752316
H -3.835356 1.593475 3.126173
H -3.223570 1.591814 4.792909
H 0.425228 1.301662 4.024797
H -0.367238 2.895811 3.984863
H -0.773447 1.733553 5.263207
H -0.227042 1.352393 1.735428
H -1.398141 -0.594853 1.277259
C -3.391729 0.133978 1.010154
C -3.909341 -0.863244 1.843471

C -4.272894 0.954110 0.297001
C -5.286490 -1.018994 1.988329
H -3.225123 -1.523021 2.383233
C -5.651456 0.797268 0.440986
H -3.897561 1.722267 -0.384376
C -6.160567 -0.183862 1.291727
H -5.677614 -1.800413 2.641696
H -6.329902 1.442714 -0.119072
H -7.239266 -0.306577 1.401333
C -1.799180 -1.850911 -0.901111
C -2.005218 -2.834957 -1.802479
C -2.728499 -4.078195 -1.361355
F -2.655299 -5.071605 -2.239557
F -2.311014 -4.525871 -0.179324
F -4.036784 -3.794700 -1.220945
O -1.043162 -0.310538 -2.528331
C -1.389783 -0.536551 -1.384486
H -2.788515 2.998484 1.391924
C -1.228489 5.379098 -0.200984
H -1.488865 5.524511 0.846680
H -1.039072 7.472685 -0.619193
H -1.957212 -2.657913 -2.877232
S 0.279716 -4.363088 -2.248141
C 1.184362 -3.278991 -1.258496
N 1.441797 -2.039568 -1.860890
N 1.567540 -3.578620 -0.052191
C 2.519890 -1.185874 -1.714133
C 3.745147 -1.579782 -1.140248
C 2.380357 0.132740 -2.191865
C 4.778823 -0.665794 -1.007671
H 3.889094 -2.607626 -0.811260
C 3.410940 1.048096 -2.063332
H 1.438453 0.425051 -2.658727
C 4.598902 0.641274 -1.457661
H 5.729543 -0.959646 -0.564819
H 3.303416 2.071785 -2.419467
N 5.686446 1.605200 -1.305698
O 5.506339 2.732231 -1.713349
O 6.710249 1.229065 -0.779483
C 1.982671 -2.717678 0.927189
C 1.479467 -1.403340 1.117044
C 2.946870 -3.187946 1.852791
C 1.947897 -0.600321 2.141919
H 0.734872 -1.013888 0.422650
C 3.417654 -2.391398 2.881658
H 3.321391 -4.204201 1.724108
C 2.914753 -1.096263 3.017315
H 1.589099 0.420480 2.268702
H 4.168368 -2.756562 3.581468
N 3.405992 -0.243353 4.089335
O 4.255218 -0.687834 4.830319
O 2.940950 0.875021 4.190167
H -1.960900 -2.053009 0.154719
H 0.828416 -1.840938 -2.647650

CF3_Michael_Acceptor_TS-MA-conf/TS-MA-R-xxvi

Frequencies, energies and thermodynamic properties:

Lowest Vibrational Mode (1/cm) = -80.0818

2nd Lowest Vibrational Mode (1/cm) = 13.6227

E(RM062X) (a.u.) = -3187.93068206

Thermal correction to Enthalpy (a.u.) = 0.680787

Thermal correction to Gibbs Free Energy (a.u.) = 0.554840

Total Entropy (cal/Kmol) = 265.080

Esp(RM062X) (a.u.) = -3190.89540926

Optimised cartesian coordinates (Angstrom):

C	-1.993114	5.787293	-1.727463
C	-1.184397	5.559564	-2.850324
C	-0.732611	4.280762	-3.156909
C	-1.105361	3.238550	-2.308654
C	-1.904016	3.469299	-1.188363
H	-0.905360	6.395356	-3.492852
H	-0.104892	4.096573	-4.029872
C	-1.535369	1.206344	-0.970961
C	-2.465046	-0.210923	0.811882
C	-2.627284	1.102350	1.592669
C	-3.038293	2.241525	0.684851
H	-2.955882	3.194278	1.222337
S	-0.686711	1.543479	-2.456143
N	-2.126854	2.293498	-0.461303
N	-1.599619	-0.002465	-0.377971
C	-3.524554	0.973305	2.844215
C	-5.013528	1.218903	2.588365
C	-3.017747	1.915871	3.936866
H	-3.407583	-0.060006	3.211453
H	-5.203988	2.278639	2.355924
H	-5.415319	0.609387	1.766649
H	-5.587327	0.978461	3.495183
H	-1.983668	1.677653	4.225876
H	-3.036810	2.962730	3.594883
H	-3.649301	1.844627	4.834327
H	-1.620207	1.354468	1.949288
H	-1.907230	-0.870052	1.486651
C	-3.755303	-0.898117	0.413243
C	-4.245736	-1.938667	1.208044
C	-4.488580	-0.490843	-0.706733
C	-5.466292	-2.542159	0.909812
H	-3.664273	-2.279378	2.068426
C	-5.709231	-1.095226	-1.006589
H	-4.113658	0.298590	-1.363819
C	-6.203834	-2.116732	-0.195353
H	-5.837959	-3.353417	1.537838
H	-6.273142	-0.769427	-1.882069
H	-7.157582	-2.590774	-0.432749
C	-1.049228	-2.410847	-0.347709
C	-0.714487	-3.539036	-1.040704
C	-1.131014	-4.852921	-0.440118
F	-0.640641	-5.911181	-1.078583
F	-0.790386	-4.952956	0.848840
F	-2.469266	-4.969045	-0.486000
O	-0.471959	-0.962364	-2.114107
C	-0.988661	-1.136987	-1.027523
H	-4.064015	2.135211	0.299249
C	-2.366533	4.749633	-0.880932
H	-2.994245	4.939806	-0.011457
H	-2.336034	6.799120	-1.508510
H	-0.615035	-3.517886	-2.125949
S	1.763866	-4.244955	-1.083948
C	2.529306	-2.787356	-0.571687
N	3.601614	-2.325395	-1.130865
N	1.902326	-2.152222	0.513345
C	4.480386	-1.441399	-0.549726
C	4.838486	-1.483017	0.819905
C	5.119689	-0.489023	-1.376241
C	5.767897	-0.597450	1.338888
H	4.377654	-2.224697	1.472773
C	6.041730	0.407022	-0.862172

H 4.863804 -0.473480 -2.436639
C 6.354723 0.347388 0.495870
H 6.047565 -0.627535 2.391218
H 6.525461 1.148210 -1.496954
N 7.325580 1.287103 1.045949
O 7.823704 2.097597 0.294332
O 7.585438 1.212211 2.227958
C 1.684878 -0.804476 0.698710
C 1.962891 0.163122 -0.289062
C 1.103330 -0.391052 1.918928
C 1.616195 1.488243 -0.081472
H 2.391897 -0.132990 -1.243593
C 0.744958 0.928967 2.119425
H 0.929381 -1.131332 2.702135
C 0.976142 1.856330 1.100046
H 1.797407 2.235592 -0.853018
H 0.286999 1.249933 3.054517
N 0.442159 3.205440 1.220662
O -0.318838 3.434815 2.142272
O 0.747584 4.021571 0.379333
H -1.344804 -2.496496 0.695499
H 1.320080 -2.770688 1.071243

CF3_Michael_Acceptor_TS-MA-conf/TS-MA-R-xxv

Frequencies, energies and thermodynamic properties:

Lowest Vibrational Mode (1/cm) = -94.6578

2nd Lowest Vibrational Mode (1/cm) = 4.3796

E(RM062X) (a.u.) = -3187.91930276

Thermal correction to Enthalpy (a.u.) = 0.680468

Thermal correction to Gibbs Free Energy (a.u.) = 0.547318

Total Entropy (cal/Kmol) = 280.238

Esp(RM062X) (a.u.) = -3190.88995171

Optimised cartesian coordinates (Angstrom):

C 7.166796 4.718867 0.899953
C 6.095270 5.458703 1.421657
C 4.808958 4.931006 1.448533
C 4.620895 3.645613 0.939924
C 5.687289 2.915353 0.414140
H 6.271388 6.460736 1.814252
H 3.972757 5.501478 1.855050
C 3.979110 1.401003 0.132182
C 4.258368 -0.859061 -0.790220
C 5.416042 -0.216898 -1.573925
C 6.203636 0.711764 -0.670491
H 6.929445 1.293845 -1.252342
S 3.122802 2.739199 0.851930
N 5.278202 1.658671 -0.044606
N 3.425798 0.215283 -0.192204
C 6.314564 -1.236135 -2.311536
C 7.490487 -1.760748 -1.482881
C 6.830318 -0.620423 -3.612970
H 5.671231 -2.091186 -2.576577
H 7.982831 -2.582944 -2.022027
H 8.246787 -0.974285 -1.331563
H 7.189218 -2.140478 -0.496577
H 6.003190 -0.347829 -4.284291
H 7.418342 0.289354 -3.409778
H 7.483724 -1.327332 -4.144441
H 4.931839 0.410547 -2.340214
H 3.626256 -1.351624 -1.538514
C 4.676557 -1.881096 0.246859
C 4.688223 -3.235028 -0.106029
C 5.096652 -1.506219 1.527359

C 5.145289 -4.196622 0.792954
H 4.335396 -3.536909 -1.095579
C 5.553484 -2.468962 2.427940
H 5.065130 -0.459953 1.842101
C 5.586371 -3.813935 2.060294
H 5.149980 -5.249115 0.505231
H 5.879488 -2.165042 3.423946
H 5.942301 -4.565478 2.766797
C 1.502680 -1.336437 -0.131506
C 0.164883 -1.512312 0.016448
C -0.369867 -2.917004 0.077815
F -1.698246 -2.986745 0.058115
F 0.097238 -3.689245 -0.906500
F 0.018281 -3.494467 1.228454
O 1.373381 0.946340 0.458142
C 2.028364 0.000252 0.071973
H 6.740606 0.170956 0.123305
C 6.981632 3.438893 0.389232
H 7.819772 2.868066 -0.009199
H 8.167623 5.152123 0.894776
H -0.489279 -0.733309 0.414322
S -1.211758 -0.910384 -2.185604
C -2.648608 -0.495115 -1.327117
N -2.642991 -0.122894 -0.080424
N -3.822413 -0.609094 -2.078526
C -3.695525 -0.247581 0.789489
C -4.587946 -1.348847 0.775962
C -3.858161 0.727276 1.801780
C -5.609361 -1.448069 1.704877
H -4.465304 -2.126622 0.021538
C -4.882604 0.638746 2.728169
H -3.157247 1.562929 1.829161
C -5.755630 -0.448545 2.668629
H -6.297796 -2.292228 1.696017
H -5.016580 1.396851 3.498720
N -6.835422 -0.549598 3.642000
O -6.942432 0.330827 4.469463
O -7.574913 -1.508874 3.578092
C -4.998809 0.114515 -1.983650
C -5.095557 1.334625 -1.284878
C -6.135007 -0.383010 -2.653183
C -6.301146 2.016786 -1.235723
H -4.218846 1.755087 -0.795828
C -7.338854 0.299503 -2.611061
H -6.059033 -1.323571 -3.201240
C -7.412200 1.491357 -1.892103
H -6.385537 2.961095 -0.699783
H -8.221913 -0.084310 -3.119794
N -8.681449 2.213328 -1.836316
O -9.628400 1.743057 -2.428015
O -8.721103 3.245225 -1.203049
H 2.137194 -2.168252 -0.426272
H -3.729021 -1.195294 -2.902944

CF3_Michael_Acceptor_TS-MA-conf/TS-MA-S-iii
Frequencies, energies and thermodynamic properties:
Lowest Vibrational Mode (1/cm) = -140.1437
2nd Lowest Vibrational Mode (1/cm) = 15.4918
E(RM062X) (a.u.) = -3187.94160125
Thermal correction to Enthalpy (a.u.) = 0.680667
Thermal correction to Gibbs Free Energy (a.u.) = 0.556302
Total Entropy (cal/Kmol) = 261.750
Esp(RM062X) (a.u.) = -3190.90181159

Optimised cartesian coordinates (Angstrom):

C	5.930896	2.657108	-0.954982
C	6.437236	1.361950	-1.132636
C	5.740951	0.251190	-0.665410
C	4.523782	0.469464	-0.019704
C	4.021655	1.761202	0.153599
H	7.387262	1.218843	-1.648559
H	6.130756	-0.759097	-0.806972
C	2.316143	0.528069	1.079422
C	0.209260	1.474044	1.892523
C	1.094406	2.670171	2.295649
C	2.088783	2.982181	1.190436
H	2.848510	3.690376	1.547487
S	3.442470	-0.732256	0.661283
N	2.785046	1.752208	0.807655
N	1.088020	0.323531	1.583092
C	0.295992	3.915187	2.748354
C	-0.050068	4.894158	1.622859
C	1.058544	4.645582	3.854511
H	-0.648159	3.544111	3.180000
H	-0.736600	5.664172	2.003667
H	0.852071	5.413420	1.262213
H	-0.534157	4.410365	0.762583
H	0.507231	5.538997	4.181837
H	1.213876	3.999405	4.730628
H	2.046555	4.976317	3.495804
H	1.674368	2.316925	3.164046
H	-0.355176	1.187737	2.788692
C	-0.763151	1.785484	0.775584
C	-2.073388	2.152850	1.098077
C	-0.350025	1.835254	-0.560225
C	-2.939571	2.623202	0.110223
H	-2.411774	2.088989	2.135493
C	-1.212559	2.309451	-1.546948
H	0.657199	1.516426	-0.840069
C	-2.502412	2.723592	-1.210450
H	-3.956570	2.918680	0.374998
H	-0.876458	2.356002	-2.584731
H	-3.173466	3.101137	-1.984293
C	-0.717905	-1.238072	2.196102
H	-1.480047	-0.472621	2.058877
C	-1.055219	-2.534027	2.522019
C	-2.375425	-2.793427	3.190739
F	-2.374732	-2.270756	4.426804
F	-3.405426	-2.225854	2.545559
F	-2.649959	-4.086843	3.328465
O	1.496971	-1.908091	1.766511
C	0.657321	-1.026582	1.849650
H	1.607225	3.407666	0.297882
C	4.717365	2.878586	-0.310477
H	4.322455	3.886826	-0.185615
H	6.493924	3.510931	-1.333849
H	-0.262329	-3.215532	2.832571
S	-1.357726	-3.901050	0.562518
C	-1.645308	-2.502096	-0.448774
N	-0.838843	-1.797450	-1.172592
N	-2.954874	-2.057904	-0.352361
C	0.488930	-2.000666	-1.487188
C	1.308450	-3.102663	-1.143331
C	1.066403	-0.971349	-2.274547
C	2.637413	-3.145030	-1.541115
H	0.899415	-3.914167	-0.548465
C	2.388695	-1.008221	-2.680565

H 0.424885 -0.137077 -2.560787
C 3.166575 -2.099256 -2.294528
H 3.276681 -3.979444 -1.254290
H 2.827073 -0.206914 -3.274490
N 4.588830 -2.096127 -2.614273
O 4.993716 -1.287319 -3.420183
O 5.304180 -2.887047 -2.032250
C -3.621729 -1.054862 -1.032429
C -3.223756 -0.547169 -2.285425
C -4.808671 -0.569271 -0.440569
C -3.989226 0.423861 -2.913388
H -2.319374 -0.919532 -2.756455
C -5.574436 0.396410 -1.068692
H -5.114136 -0.963088 0.531001
C -5.147852 0.892320 -2.300060
H -3.689959 0.826848 -3.880467
H -6.485505 0.780634 -0.612015
N -5.913354 1.956358 -2.940853
O -7.001536 2.229558 -2.484081
O -5.416557 2.519400 -3.893964
H -3.497197 -2.531435 0.362233

CF3_Michael_Acceptor_TS-MA-conf/TS-MA-S-ii
Frequencies, energies and thermodynamic properties:
Lowest Vibrational Mode (1/cm) = -120.4338
2nd Lowest Vibrational Mode (1/cm) = 11.9005
E(RM062X) (a.u.) = -3187.93569739
Thermal correction to Enthalpy (a.u.) = 0.681112
Thermal correction to Gibbs Free Energy (a.u.) = 0.553134
Total Entropy (cal/Kmol) = 269.353
Esp(RM062X) (a.u.) = -3190.89979260
Optimised cartesian coordinates (Angstrom):
C -7.017676 3.237141 -0.727025
C -6.083932 4.283081 -0.771775
C -4.735574 4.050131 -0.522079
C -4.346818 2.743271 -0.225616
C -5.279892 1.706988 -0.164397
H -6.417528 5.294296 -1.007470
H -3.993745 4.849389 -0.550749
C -3.355843 0.554439 0.341738
C -3.236792 -1.869940 0.718371
C -4.680336 -1.699765 1.224405
C -5.444595 -0.751687 0.319742
H -6.413954 -0.493624 0.765175
S -2.728998 2.166281 0.124699
N -4.680167 0.487999 0.172273
N -2.605052 -0.533434 0.603860
C -5.422563 -3.037315 1.449208
C -6.179057 -3.557076 0.223450
C -6.381197 -2.905631 2.633313
H -4.655413 -3.781153 1.719683
H -6.548597 -4.573361 0.423067
H -7.058087 -2.929302 0.007320
H -5.557438 -3.599087 -0.681894
H -6.921417 -3.848502 2.801823
H -5.845013 -2.648478 3.558323
H -7.131220 -2.120592 2.444548
H -4.585537 -1.205638 2.205144
H -2.696429 -2.391011 1.517854
C -3.101057 -2.665939 -0.563791
C -2.831191 -4.036733 -0.483320
C -3.288548 -2.079921 -1.819878
C -2.783564 -4.817155 -1.636771

H -2.657136 -4.494221 0.494208
C -3.239796 -2.861006 -2.974887
H -3.467297 -1.005753 -1.913908
C -2.995681 -4.231248 -2.885371
H -2.572725 -5.885082 -1.560847
H -3.387618 -2.393278 -3.949575
H -2.956214 -4.839983 -3.790114
C -0.379899 -1.582946 0.834677
H -0.800093 -2.576416 0.698384
C 0.970968 -1.415224 0.919144
C 1.821399 -2.606538 1.264550
F 1.627071 -2.938742 2.553130
F 1.512199 -3.685689 0.545258
F 3.127785 -2.383868 1.132487
O -0.727456 0.744855 0.749875
C -1.179717 -0.382747 0.740369
H -5.618350 -1.176497 -0.680299
C -6.632958 1.935697 -0.422519
H -7.362720 1.127191 -0.392244
H -8.068330 3.444618 -0.933657
H 1.395444 -0.440781 1.172081
S 1.983383 -1.063024 -1.396354
C 3.360559 -0.150190 -0.823182
N 3.364274 1.006950 -0.244736
N 4.561481 -0.833419 -0.948000
H 4.443969 -1.776741 -1.303491
C 2.296077 1.870767 -0.173516
C 1.449251 2.165509 -1.266631
C 2.100041 2.574311 1.036481
C 0.435638 3.099535 -1.143135
H 1.600630 1.646846 -2.211801
C 1.086256 3.505817 1.170362
H 2.767271 2.359050 1.872322
C 0.259461 3.759039 0.075080
H -0.222601 3.327576 -1.980724
H 0.924643 4.037325 2.107308
N -0.829405 4.708719 0.217067
O -0.914008 5.349045 1.240365
O -1.628713 4.804207 -0.701167
C 5.856039 -0.481824 -0.621875
C 6.256132 0.789032 -0.157457
C 6.839368 -1.485417 -0.788215
C 7.592930 1.034660 0.126015
H 5.514675 1.569181 -0.021922
C 8.169116 -1.239891 -0.505349
H 6.533038 -2.470646 -1.144827
C 8.536068 0.026278 -0.047359
H 7.911226 2.013519 0.482392
H 8.924409 -2.014078 -0.632072
N 9.938673 0.297035 0.254254
O 10.738695 -0.597892 0.086372
O 10.232004 1.401765 0.656430

CF3_Michael_Acceptor_TS-MA-conf/TS-MA-S-i

Frequencies, energies and thermodynamic properties:

Lowest Vibrational Mode (1/cm) = -133.2872

2nd Lowest Vibrational Mode (1/cm) = 19.7386

E(RM062X) (a.u.) = -3187.94750675

Thermal correction to Enthalpy (a.u.) = 0.680593

Thermal correction to Gibbs Free Energy (a.u.) = 0.557801

Total Entropy (cal/Kmol) = 258.438

Esp(RM062X) (a.u.) = -3190.90928200

Optimised cartesian coordinates (Angstrom):

C	6.602875	0.840039	-0.451944
C	6.748102	-0.468436	0.029498
C	5.696515	-1.114920	0.671372
C	4.490800	-0.426113	0.800889
C	4.354067	0.880704	0.329827
H	7.694900	-0.991964	-0.106558
H	5.803413	-2.135871	1.041456
C	2.222061	0.498806	1.105459
C	0.388565	2.120461	0.975261
C	1.524990	3.141148	1.187851
C	2.708756	2.777673	0.308486
H	3.584389	3.388668	0.565647
S	3.000891	-1.002060	1.524151
N	3.066715	1.379811	0.556614
N	0.914245	0.764109	1.270226
C	1.091347	4.616840	1.021692
C	1.262249	5.173617	-0.394759
C	1.854092	5.492835	2.016306
H	0.021904	4.666059	1.283123
H	0.801406	6.170138	-0.456463
H	2.328601	5.293264	-0.643754
H	0.798574	4.543126	-1.166510
H	1.563536	6.547805	1.907017
H	1.655695	5.189339	3.054467
H	2.940375	5.426324	1.843143
H	1.843874	3.006500	2.234469
H	-0.369733	2.317740	1.743886
C	-0.256736	2.232169	-0.388371
C	-1.392789	3.036930	-0.533202
C	0.313134	1.646371	-1.523342
C	-1.922978	3.290353	-1.797715
H	-1.858218	3.476020	0.352693
C	-0.227035	1.885417	-2.787974
H	1.185626	0.995926	-1.426720
C	-1.337901	2.718435	-2.928843
H	-2.801648	3.929809	-1.898740
H	0.223575	1.418863	-3.665802
H	-1.756483	2.908921	-3.918396
C	-1.356685	-0.050597	1.781327
H	-1.801748	0.684825	1.111894
C	-2.141588	-0.914120	2.521826
C	-3.576111	-0.530052	2.756669
F	-3.631701	0.533985	3.578478
F	-4.209354	-0.177453	1.638019
F	-4.286496	-1.491123	3.345930
O	0.608850	-1.219648	2.351265
C	0.060544	-0.253458	1.845478
H	2.493561	2.908837	-0.761947
C	5.407840	1.537206	-0.306123
H	5.295731	2.550668	-0.691020
H	7.439715	1.321972	-0.958758
H	-1.683118	-1.453268	3.351376
S	-2.590511	-3.047323	1.494740
C	-1.562590	-2.661532	0.131031
N	-1.825583	-1.838590	-0.823519
N	-0.311247	-3.256281	0.188421
H	-0.211964	-3.901275	0.965739
C	-3.074879	-1.293128	-1.054507
C	-4.261331	-2.056901	-1.083936
C	-3.149317	0.077610	-1.365476
C	-5.478166	-1.459688	-1.373191
H	-4.216863	-3.124038	-0.872726
C	-4.362145	0.687605	-1.643396

H -2.224523 0.652422 -1.370407
C -5.517827 -0.090832 -1.638377
H -6.399323 -2.040379 -1.394588
H -4.422936 1.751825 -1.867985
N -6.802790 0.541432 -1.930226
O -6.811969 1.730891 -2.164183
O -7.794769 -0.154242 -1.923745
C 0.857061 -2.970481 -0.487754
C 1.032085 -1.866796 -1.349762
C 1.958602 -3.822266 -0.241583
C 2.286249 -1.595490 -1.881543
H 0.186014 -1.224799 -1.576093
C 3.200758 -3.564668 -0.791422
H 1.821998 -4.687409 0.410079
C 3.359139 -2.434601 -1.594655
H 2.442238 -0.726586 -2.521901
H 4.050919 -4.215927 -0.591978
N 4.684532 -2.098291 -2.104319
O 5.578197 -2.899787 -1.936305
O 4.827289 -1.028746 -2.655743

CF3_Michael_Acceptor_TS-MA-conf/TS-MA-S-iv

Frequencies, energies and thermodynamic properties:

Lowest Vibrational Mode (1/cm) = -114.6001

2nd Lowest Vibrational Mode (1/cm) = 8.0403

E(RM062X) (a.u.) = -3187.92742926

Thermal correction to Enthalpy (a.u.) = 0.681189

Thermal correction to Gibbs Free Energy (a.u.) = 0.551827

Total Entropy (cal/Kmol) = 272.267

Esp(RM062X) (a.u.) = -3190.89499663

Optimised cartesian coordinates (Angstrom):

C 7.898594 -3.743939 -0.764131
C 7.338657 -4.827986 -0.073134
C 6.128901 -4.695925 0.600386
C 5.498140 -3.452355 0.572366
C 6.062162 -2.372429 -0.108039
H 7.854476 -5.788743 -0.066357
H 5.684372 -5.537524 1.133275
C 4.138929 -1.390421 0.692561
C 3.473493 0.931890 0.266640
C 4.982372 1.182058 0.120280
C 5.638372 0.044203 -0.636326
H 6.730627 0.134873 -0.589166
S 3.960289 -3.018984 1.296442
N 5.269224 -1.224095 -0.004496
N 3.248901 -0.400795 0.878549
C 5.324185 2.569744 -0.469102
C 5.399002 2.606724 -1.997772
C 6.636348 3.078915 0.128257
H 4.521952 3.254728 -0.148510
H 5.501851 3.647924 -2.336089
H 6.282062 2.058106 -2.362299
H 4.508008 2.183733 -2.482675
H 6.893472 4.065005 -0.284830
H 6.571204 3.172916 1.221975
H 7.466223 2.392836 -0.106512
H 5.378949 1.154328 1.148624
H 3.129692 1.669420 0.999978
C 2.650199 1.077574 -0.998161
C 1.977340 2.279514 -1.240787
C 2.556579 0.042780 -1.935685
C 1.244298 2.455767 -2.412825
H 2.020673 3.081825 -0.499713

C 1.818759 0.216990 -3.106343
H 3.044140 -0.919417 -1.758150
C 1.164655 1.424613 -3.349494
H 0.725385 3.399129 -2.589963
H 1.748468 -0.599002 -3.827348
H 0.584219 1.558296 -4.263850
C 1.042401 0.377064 1.642505
H 1.269595 1.393824 1.339564
C -0.257738 0.059907 1.960005
C -1.162601 1.151974 2.456005
F -0.834122 1.461466 3.722756
F -1.059629 2.277576 1.750672
F -2.444636 0.790385 2.480769
O 1.799726 -1.845127 1.884427
C 1.983702 -0.697338 1.516404
H 5.331511 0.008333 -1.692615
C 7.271202 -2.502943 -0.795126
H 7.710780 -1.670769 -1.344049
H 8.844133 -3.872958 -1.292130
H -0.495495 -0.929688 2.353423
S -1.276895 -0.328320 -0.174369
C -3.042551 -0.432778 -0.198823
N -3.888698 0.533089 -0.115455
N -3.502715 -1.737515 -0.282828
C -3.552436 1.872016 -0.116620
C -2.703160 2.456166 -1.080459
C -4.149852 2.705635 0.851240
C -2.438360 3.816454 -1.055768
H -2.250437 1.826172 -1.845063
C -3.880250 4.063879 0.891794
H -4.816722 2.251805 1.585032
C -3.020864 4.604575 -0.063571
H -1.786000 4.278484 -1.795423
H -4.324832 4.708782 1.648414
N -2.729145 6.035756 -0.029631
O -3.267129 6.706949 0.824047
O -1.962178 6.481365 -0.856147
C -4.781473 -2.252470 -0.383945
C -5.958122 -1.477836 -0.450752
C -4.889777 -3.662094 -0.430130
C -7.193609 -2.102414 -0.561027
H -5.891699 -0.395687 -0.412979
C -6.119613 -4.282060 -0.539913
H -3.981119 -4.264893 -0.377782
C -7.267253 -3.490958 -0.604728
H -8.107879 -1.512771 -0.614506
H -6.202748 -5.367227 -0.575653
N -8.572481 -4.136273 -0.721640
O -8.605015 -5.346928 -0.766191
O -9.555000 -3.429115 -0.768321
H -2.756293 -2.425117 -0.274281

CF3_Michael_Acceptor_TS-MA-conf/TS-MA-S-ix

Frequencies, energies and thermodynamic properties:

Lowest Vibrational Mode (1/cm) = -117.2044

2nd Lowest Vibrational Mode (1/cm) = 10.6628

E(RM062X) (a.u.) = -3187.92969015

Thermal correction to Enthalpy (a.u.) = 0.681013

Thermal correction to Gibbs Free Energy (a.u.) = 0.554315

Total Entropy (cal/Kmol) = 266.660

Esp(RM062X) (a.u.) = -3190.89371566

Optimised cartesian coordinates (Angstrom):

C 3.244744 5.058515 -0.817826

C	1.919962	5.456379	-0.590296
C	0.882580	4.529195	-0.603866
C	1.206791	3.192732	-0.835325
C	2.526574	2.801715	-1.066031
H	1.698036	6.505622	-0.393018
H	-0.149862	4.832689	-0.422218
C	1.481528	0.752763	-1.091344
C	2.658971	-1.383975	-1.286181
C	3.621732	-0.591143	-2.186761
C	3.903285	0.768921	-1.574398
H	4.424696	1.416341	-2.292140
S	0.122443	1.815326	-0.839377
N	2.633042	1.418139	-1.242987
N	1.423106	-0.590050	-1.091944
C	4.910107	-1.365674	-2.550231
C	6.072043	-1.162693	-1.573862
C	5.352625	-0.998977	-3.967050
H	4.644875	-2.436002	-2.547611
H	5.792426	-1.346302	-0.526818
H	6.889591	-1.852901	-1.827696
H	6.475855	-0.140488	-1.648149
H	4.578080	-1.246593	-4.707358
H	5.564558	0.079591	-4.043530
H	6.271072	-1.538879	-4.239519
H	3.064957	-0.418210	-3.122339
H	2.358147	-2.271715	-1.856176
C	3.256646	-1.824884	0.034670
C	3.748732	-3.127647	0.162631
C	3.379331	-0.942228	1.112777
C	4.372055	-3.536299	1.340734
H	3.639045	-3.829688	-0.668029
C	3.999211	-1.350726	2.293629
H	2.991793	0.077152	1.042272
C	4.502001	-2.647083	2.407870
H	4.750358	-4.556099	1.427763
H	4.089263	-0.651876	3.127304
H	4.986118	-2.966812	3.331999
C	0.181598	-2.650132	-0.567496
H	1.101903	-3.216111	-0.448322
C	-1.030191	-3.205938	-0.199533
C	-1.115722	-4.700711	-0.057685
F	-0.906316	-5.290455	-1.246380
F	-0.190853	-5.185522	0.775373
F	-2.305150	-5.115598	0.366945
O	-0.826704	-0.533867	-0.744842
C	0.169635	-1.244842	-0.809689
H	4.511420	0.701078	-0.660397
C	3.569314	3.728176	-1.062921
H	4.602551	3.421018	-1.223778
H	4.041004	5.803467	-0.794227
H	-1.970321	-2.734713	-0.499673
S	-1.547505	-2.574911	2.060157
C	-2.707225	-1.395554	1.526478
N	-3.708695	-1.760426	0.791472
N	-2.605404	-0.065698	1.945439
H	-3.469944	0.450789	1.800772
C	-4.541388	-0.866269	0.141392
C	-5.938487	-1.023545	0.254397
C	-4.039102	0.156418	-0.697946
C	-6.807862	-0.181072	-0.421678
H	-6.322003	-1.823625	0.888472
C	-4.903540	0.996964	-1.382155
H	-2.958267	0.252146	-0.815876

C -6.279290 0.823359 -1.231044
H -7.887666 -0.291862 -0.332322
H -4.526762 1.782241 -2.036311
N -7.191552 1.715100 -1.945578
O -6.709900 2.588574 -2.633714
O -8.382281 1.537026 -1.811493
C -1.528832 0.776538 2.161380
C -1.824115 2.160164 2.162644
C -0.190013 0.375899 2.354400
C -0.829587 3.112500 2.295671
H -2.859785 2.479470 2.027740
C 0.807684 1.331849 2.481702
H 0.062352 -0.680238 2.393122
C 0.490634 2.686205 2.428159
H -1.061841 4.176722 2.273655
H 1.842898 1.029062 2.632186
O 2.695933 3.284814 2.264748
N 1.561940 3.674318 2.450596
O 1.263662 4.833348 2.638302

CF3_Michael_Acceptor_TS-MA-conf/TS-MA-S-viii
Frequencies, energies and thermodynamic properties:
Lowest Vibrational Mode (1/cm) = -117.2204
2nd Lowest Vibrational Mode (1/cm) = 10.6675
E(RM062X) (a.u.) = -3187.92969022
Thermal correction to Enthalpy (a.u.) = 0.681014
Thermal correction to Gibbs Free Energy (a.u.) = 0.554320
Total Entropy (cal/Kmol) = 266.649
Esp(RM062X) (a.u.) = -3190.89371057
Optimised cartesian coordinates (Angstrom):
C -3.506258 4.917444 0.818063
C -2.188389 5.391318 0.739745
C -1.108840 4.518348 0.816290
C -1.381795 3.158040 0.964528
C -2.694079 2.691839 1.053585
H -2.005618 6.458023 0.606781
H -0.081917 4.880716 0.746749
C -1.546113 0.701608 1.142599
C -2.610313 -1.511651 1.303603
C -3.698240 -0.744253 2.072966
C -3.996167 0.571752 1.378917
H -4.644517 1.198131 2.005691
S -0.232333 1.838391 1.020020
N -2.740250 1.299611 1.182929
N -1.413255 -0.642598 1.165027
C -4.972208 -1.577653 2.345059
C -6.039411 -1.479032 1.251236
C -5.573236 -1.177519 3.692701
H -4.651326 -2.629700 2.419638
H -5.646468 -1.678218 0.244443
H -6.837896 -2.208620 1.449378
H -6.505704 -0.481154 1.246050
H -4.864606 -1.347523 4.516229
H -5.850565 -0.111187 3.695530
H -6.483741 -1.758404 3.899335
H -3.247003 -0.500749 3.048919
H -2.305990 -2.343537 1.951272
C -3.050190 -2.062356 -0.037602
C -3.515498 -3.378546 -0.124837
C -3.031817 -1.266951 -1.185843
C -3.980073 -3.881596 -1.338635
H -3.509210 -4.014344 0.764479
C -3.493275 -1.769726 -2.402503

H -2.642804 -0.247630 -1.136542
C -3.974150 -3.076589 -2.479016
H -4.339191 -4.910304 -1.396818
H -3.470593 -1.139542 -3.293472
H -4.331803 -3.472749 -3.430665
C -0.049551 -2.651688 0.728864
H -0.936974 -3.276698 0.677948
C 1.148215 -3.138016 0.311574
C 1.297759 -4.613007 0.067729
F 1.271230 -5.278444 1.235497
F 0.303123 -5.107235 -0.673078
F 2.444903 -4.926849 -0.522480
O 0.837592 -0.473958 0.892638
C -0.121626 -1.222159 0.947754
H -4.478699 0.432274 0.400250
C -3.780669 3.564522 0.980207
H -4.806851 3.201101 1.024664
H -4.335289 5.621976 0.742774
H 2.083006 -2.586861 0.437032
S 1.444710 -2.335400 -2.148657
C 2.612024 -1.192431 -1.601424
N 3.676437 -1.587727 -0.962095
N 2.473888 0.172268 -1.884847
H 3.334610 0.690432 -1.726154
C 4.528809 -0.745511 -0.281347
C 5.923777 -0.915658 -0.433423
C 4.069222 0.232904 0.636276
C 6.823206 -0.133914 0.272976
H 6.277930 -1.680451 -1.125550
C 4.964299 1.013407 1.350465
H 2.994785 0.340947 0.792479
C 6.333603 0.828101 1.156883
H 7.898449 -0.257678 0.150712
H 4.616694 1.760600 2.062886
N 7.276487 1.656466 1.902231
O 6.827564 2.502545 2.645747
O 8.461203 1.459150 1.739153
C 1.391055 1.009263 -2.057342
C 1.681068 2.395059 -2.014408
C 0.048809 0.610908 -2.242330
C 0.685780 3.347908 -2.118354
H 2.717384 2.712067 -1.878917
C -0.950935 1.570217 -2.340638
H -0.196589 -0.446506 -2.303233
C -0.634501 2.923832 -2.265512
H 0.916892 4.411429 -2.071713
H -1.991231 1.277762 -2.483835
O -2.846549 3.522207 -2.208048
N -1.702522 3.914973 -2.299257
O -1.392775 5.081443 -2.409282

CF3_Michael_Acceptor_TS-MA-conf/TS-MA-S-vii

Frequencies, energies and thermodynamic properties:

Lowest Vibrational Mode (1/cm) = -99.1496
2nd Lowest Vibrational Mode (1/cm) = 7.3018
E(RM062X) (a.u.) = -3187.92478746
Thermal correction to Enthalpy (a.u.) = 0.680730
Thermal correction to Gibbs Free Energy (a.u.) = 0.548046
Total Entropy (cal/Kmol) = 279.257
Esp(RM062X) (a.u.) = -3190.89247641

Optimised cartesian coordinates (Angstrom):

C -7.376249 -0.360297 -2.878083
C -6.951026 0.859931 -3.423978

C	-5.763955	1.453496	-3.009014
C	-5.014258	0.797214	-2.032669
C	-5.444657	-0.411857	-1.484488
H	-7.556273	1.349619	-4.187578
H	-5.426802	2.399705	-3.434324
C	-3.467171	-0.129427	-0.345197
C	-2.544063	-1.744656	1.260572
C	-4.021753	-2.074264	1.532529
C	-4.792530	-2.126887	0.227181
H	-5.870952	-2.187893	0.421306
S	-3.489739	1.307182	-1.332834
N	-4.549405	-0.891928	-0.522327
N	-2.467696	-0.477845	0.490148
C	-4.229915	-3.338445	2.398260
C	-4.350129	-4.640164	1.600827
C	-5.461177	-3.158033	3.286861
H	-3.349408	-3.418753	3.056626
H	-4.343499	-5.496251	2.290845
H	-5.302399	-4.675841	1.048194
H	-3.531153	-4.783140	0.881756
H	-5.629292	-4.052853	3.903399
H	-5.349008	-2.295215	3.959358
H	-6.364766	-2.999365	2.676280
H	-4.409515	-1.215224	2.104169
H	-2.094522	-1.527605	2.237223
C	-1.757497	-2.853678	0.592288
C	-1.029179	-3.742157	1.391234
C	-1.781427	-3.044035	-0.792963
C	-0.361294	-4.822923	0.819028
H	-0.985584	-3.582655	2.471722
C	-1.111686	-4.125331	-1.366220
H	-2.314959	-2.346010	-1.443157
C	-0.407308	-5.020492	-0.561510
H	0.204126	-5.508164	1.452511
H	-1.135777	-4.263728	-2.448225
H	0.118763	-5.863494	-1.012231
C	-0.195696	-0.107398	1.379416
H	-0.114062	-1.144591	1.696889
C	0.851126	0.746842	1.536618
C	1.964804	0.388884	2.479215
F	1.551987	0.555141	3.747068
F	2.348656	-0.884115	2.368299
F	3.043178	1.156924	2.333634
O	-1.351716	1.451798	0.039072
C	-1.322011	0.376762	0.607844
H	-4.497785	-2.979671	-0.402677
C	-6.632666	-1.015398	-1.902715
H	-6.964845	-1.968140	-1.491413
H	-8.307370	-0.809335	-3.225597
H	0.739745	1.811269	1.326481
S	2.296147	0.532234	-0.603694
C	3.660615	1.605221	-0.543792
N	3.509937	2.952774	-0.219472
N	4.898993	1.289867	-0.773902
C	2.435369	3.804488	-0.072195
C	1.107440	3.524708	-0.459656
C	2.716432	5.072207	0.493207
C	0.105705	4.462113	-0.253459
H	0.856832	2.565644	-0.903152
C	1.721522	6.011823	0.685918
H	3.742238	5.303496	0.786654
C	0.413964	5.694266	0.316228
H	-0.921089	4.234456	-0.535847

H 1.943022 6.983012 1.126136
N -0.646860 6.672365 0.528998
O -0.346369 7.740909 1.016452
O -1.775644 6.368110 0.208237
C 5.372548 0.005724 -0.906626
C 5.101000 -1.017088 0.032584
C 6.249996 -0.282168 -1.974397
C 5.675291 -2.270261 -0.097957
H 4.437815 -0.804848 0.870204
C 6.818551 -1.537902 -2.120099
H 6.471222 0.511536 -2.688974
C 6.523559 -2.522256 -1.178149
H 5.475987 -3.059146 0.626061
H 7.488208 -1.764756 -2.948533
N 7.121766 -3.845710 -1.320155
O 7.861425 -4.040239 -2.261184
O 6.849436 -4.687155 -0.490553
H 4.416347 3.390285 -0.069741

CF3_Michael_Acceptor_TS-MA-conf/TS-MA-S-vi

Frequencies, energies and thermodynamic properties:

Lowest Vibrational Mode (1/cm) = -138.9866

2nd Lowest Vibrational Mode (1/cm) = 11.7384

E(RM062X) (a.u.) = -3187.93203755

Thermal correction to Enthalpy (a.u.) = 0.680944

Thermal correction to Gibbs Free Energy (a.u.) = 0.553373

Total Entropy (cal/Kmol) = 268.497

Esp(RM062X) (a.u.) = -3190.89576817

Optimised cartesian coordinates (Angstrom):

C -2.352822 -6.163568 -1.918722
C -3.492823 -5.423582 -2.263092
C -3.651383 -4.111648 -1.827194
C -2.641842 -3.557939 -1.040690
C -1.513643 -4.301355 -0.691149
H -4.266020 -5.879854 -2.882176
H -4.536980 -3.532216 -2.092293
C -1.021971 -2.306504 0.345307
C 1.068881 -1.836691 1.532167
C 1.013959 -3.338322 1.874803
C 0.601355 -4.132739 0.649788
H 0.393391 -5.176696 0.918936
S -2.585006 -1.945057 -0.348491
N -0.633921 -3.563957 0.108292
N -0.255133 -1.427188 1.007373
C 2.301498 -3.888429 2.530550
C 3.331202 -4.438674 1.539624
C 1.938440 -4.966609 3.552444
H 2.763258 -3.050836 3.077522
H 4.268321 -4.664401 2.068899
H 2.977902 -5.379878 1.089340
H 3.566607 -3.737156 0.726959
H 2.844103 -5.378990 4.020450
H 1.294546 -4.565954 4.348802
H 1.403909 -5.800281 3.068703
H 0.201158 -3.434133 2.613400
H 1.191304 -1.305763 2.484277
C 2.204279 -1.455255 0.604740
C 3.427231 -1.062259 1.162782
C 2.095413 -1.572783 -0.784018
C 4.539236 -0.847887 0.351173
H 3.508468 -0.934461 2.245228
C 3.208251 -1.350793 -1.597791
H 1.140363 -1.831012 -1.248936

C	4.435254	-1.005899	-1.031894
H	5.487768	-0.541172	0.796175
H	3.112547	-1.446252	-2.680777
H	5.303859	-0.833694	-1.670933
C	0.175387	0.899569	1.675794
H	1.235255	0.682291	1.784545
C	-0.308862	2.180475	1.881834
C	0.464024	3.078631	2.808644
F	0.294462	2.671460	4.075324
F	1.779948	3.050685	2.571835
F	0.073482	4.350994	2.766130
O	-1.936613	0.107400	0.980225
C	-0.753410	-0.083718	1.213779
H	1.369563	-4.117403	-0.137804
C	-1.346982	-5.616276	-1.128555
H	-0.460238	-6.193712	-0.868232
H	-2.248262	-7.188514	-2.276526
H	-1.391112	2.315585	1.949176
S	-0.148518	3.622857	-0.013848
C	-0.339491	2.288426	-1.125062
N	-1.599363	1.803507	-1.451968
N	0.606408	1.648383	-1.733637
C	-2.897080	2.046166	-1.051831
C	-3.325276	3.034032	-0.140495
C	-3.875167	1.206250	-1.636433
C	-4.669884	3.151698	0.181286
H	-2.605449	3.711875	0.306798
C	-5.214002	1.322179	-1.317025
H	-3.556349	0.449734	-2.356164
C	-5.603170	2.297801	-0.399377
H	-5.002071	3.912278	0.886722
H	-5.959844	0.669907	-1.768860
N	-7.013038	2.428162	-0.049407
O	-7.800667	1.668270	-0.571416
O	-7.326164	3.287609	0.745442
C	1.961852	1.916804	-1.675687
C	2.686396	2.173771	-0.493684
C	2.672586	1.840490	-2.893887
C	4.063490	2.334957	-0.528734
H	2.164713	2.233633	0.455743
C	4.046801	2.010327	-2.940757
H	2.110091	1.630937	-3.804599
C	4.729062	2.247963	-1.748685
H	4.628751	2.509976	0.385681
H	4.596135	1.944219	-3.878810
N	6.187551	2.338560	-1.768430
O	6.750018	2.233197	-2.836947
O	6.762777	2.502332	-0.714263
H	-1.513405	1.034493	-2.113033

CF3_Michael_Acceptor_TS-MA-conf/TS-MA-S-v

Frequencies, energies and thermodynamic properties:

Lowest Vibrational Mode (1/cm) = -167.7310

2nd Lowest Vibrational Mode (1/cm) = 13.2661

E(RM062X) (a.u.) = -3187.93151947

Thermal correction to Enthalpy (a.u.) = 0.680602

Thermal correction to Gibbs Free Energy (a.u.) = 0.553802

Total Entropy (cal/Kmol) = 266.872

Esp(RM062X) (a.u.) = -3190.89262992

Optimised cartesian coordinates (Angstrom):

C	7.678746	0.441137	0.754706
C	7.469220	1.762423	0.333614
C	6.268693	2.143265	-0.256622

C	5.287706	1.165807	-0.422844
C	5.494752	-0.146436	0.004189
H	8.254732	2.505156	0.477443
H	6.081450	3.174114	-0.558850
C	3.336469	-0.308734	-0.781054
C	1.950510	-2.325470	-0.650359
C	3.293791	-3.072626	-0.689712
C	4.322276	-2.358371	0.165569
H	5.319930	-2.786092	0.004379
S	3.681543	1.371636	-1.095019
N	4.372856	-0.949931	-0.227401
N	2.161452	-0.910190	-1.034000
C	3.178971	-4.577081	-0.354310
C	3.303322	-4.903844	1.136411
C	4.221850	-5.365694	-1.147288
H	2.182166	-4.901830	-0.695827
H	3.059590	-5.963070	1.303355
H	4.336011	-4.749646	1.487379
H	2.633759	-4.302503	1.767233
H	4.159361	-6.438118	-0.912193
H	4.079667	-5.244361	-2.230981
H	5.240839	-5.028617	-0.897346
H	3.634108	-2.993402	-1.735308
H	1.343792	-2.774846	-1.444397
C	1.186238	-2.409395	0.656522
C	0.181014	-3.371527	0.801142
C	1.473494	-1.558123	1.729268
C	-0.507962	-3.500582	2.005544
H	-0.067426	-4.020798	-0.042401
C	0.783061	-1.685974	2.934703
H	2.229728	-0.774223	1.634894
C	-0.205171	-2.659776	3.077352
H	-1.292372	-4.252812	2.103669
H	1.014080	-1.013972	3.763028
H	-0.748720	-2.754471	4.018745
C	-0.219827	-0.764499	-1.656559
H	-0.333190	-1.844233	-1.635033
C	-1.344443	0.038398	-1.663362
C	-2.598535	-0.486547	-2.308639
F	-2.417183	-0.584717	-3.634643
F	-2.934553	-1.704900	-1.878025
F	-3.652584	0.313200	-2.134835
O	1.226858	1.093012	-1.590323
C	1.033427	-0.104500	-1.454843
H	4.084608	-2.410234	1.239094
C	6.696180	-0.531695	0.602639
H	6.859639	-1.552340	0.947663
H	8.626681	0.168809	1.220454
H	-1.212225	1.118432	-1.780159
S	-2.198286	0.209364	0.521441
C	-2.889216	1.809716	0.372647
N	-4.262782	2.001290	0.335381
N	-2.218975	2.909660	0.230656
C	-5.381617	1.189439	0.378541
C	-5.396196	-0.219984	0.315558
C	-6.619091	1.869511	0.476573
C	-6.600972	-0.907196	0.361485
H	-4.467481	-0.773144	0.224467
C	-7.819204	1.185603	0.518845
H	-6.618383	2.960275	0.522324
C	-7.799400	-0.207409	0.464131
H	-6.615112	-1.995079	0.310516
H	-8.766883	1.716315	0.596444

N -9.059302 -0.943220 0.510468
O -10.086488 -0.305402 0.593827
O -9.014073 -2.153219 0.463935
C -0.857199 3.051068 0.385778
C -0.173497 3.876150 -0.532300
C -0.126483 2.496224 1.462678
C 1.185647 4.112859 -0.414514
H -0.742664 4.318765 -1.350755
C 1.228650 2.745691 1.600756
H -0.639305 1.873613 2.193904
C 1.873479 3.547014 0.657388
H 1.717651 4.728583 -1.138289
H 1.795209 2.326430 2.431640
N 3.308949 3.756271 0.770884
O 3.882942 3.311001 1.740630
O 3.874406 4.344567 -0.132199
H -4.479118 2.995162 0.289030

CF3_Michael_Acceptor_TS-MA-conf/TS-MA-S-xiii
Frequencies, energies and thermodynamic properties:
Lowest Vibrational Mode (1/cm) = -64.6455
2nd Lowest Vibrational Mode (1/cm) = 9.4812
E(RM062X) (a.u.) = -3187.91769616
Thermal correction to Enthalpy (a.u.) = 0.681201
Thermal correction to Gibbs Free Energy (a.u.) = 0.550663
Total Entropy (cal/Kmol) = 274.741
Esp(RM062X) (a.u.) = -3190.88959644
Optimised cartesian coordinates (Angstrom):
C 4.759820 5.680671 0.148532
C 3.446811 6.125832 -0.067123
C 2.427774 5.227913 -0.363846
C 2.755930 3.874280 -0.443004
C 4.064076 3.434875 -0.236007
H 3.219672 7.190165 0.002303
H 1.403932 5.566915 -0.526941
C 3.016614 1.426667 -0.630591
C 4.129318 -0.769959 -0.562441
C 5.371149 0.033815 -0.983294
C 5.439176 1.334565 -0.207382
H 6.232433 1.979441 -0.605639
S 1.679995 2.534425 -0.783723
N 4.169071 2.045990 -0.365840
N 2.923926 0.082485 -0.734944
C 6.690552 -0.768992 -0.915778
C 7.407581 -0.690733 0.434679
C 7.630568 -0.309671 -2.030970
H 6.432029 -1.822850 -1.108392
H 8.248523 -1.399050 0.447329
H 7.826892 0.314594 0.598365
H 6.753581 -0.931878 1.284454
H 8.580905 -0.861190 -1.988869
H 7.185736 -0.469962 -3.023807
H 7.862414 0.762916 -1.930484
H 5.198401 0.296570 -2.039885
H 4.037184 -1.577977 -1.298791
C 4.184016 -1.370045 0.828222
C 4.654218 -2.679308 0.982494
C 3.808935 -0.641735 1.961536
C 4.781522 -3.239779 2.251414
H 4.920824 -3.264603 0.098629
C 3.933851 -1.204297 3.232305
H 3.401823 0.368323 1.867856
C 4.427187 -2.499857 3.380401

H 5.148944 -4.261624 2.358426
H 3.634433 -0.628165 4.109083
H 4.520003 -2.939150 4.374879
C 1.537928 -1.963856 -0.836711
H 2.402345 -2.595230 -0.647713
C 0.296317 -2.492300 -0.823456
C 0.115467 -3.982287 -0.867061
F 0.420200 -4.442970 -2.091510
F 0.905592 -4.624336 -0.010751
F -1.143901 -4.360867 -0.632598
O 0.688671 0.220361 -1.118799
C 1.636666 -0.510720 -0.925414
H 5.617636 1.175320 0.866830
C 5.089914 4.332569 0.069240
H 6.110445 3.994396 0.247102
H 5.540393 6.404064 0.386595
H -0.583023 -1.890503 -1.058008
S -0.623669 -2.285161 1.763737
C -2.033465 -1.384392 1.371133
N -2.780125 -1.913875 0.303303
N -2.387554 -0.298991 1.996162
C -3.501777 -1.253453 -0.671874
C -3.378768 0.130872 -0.912571
C -4.362592 -2.016248 -1.489034
C -4.115035 0.731456 -1.921245
H -2.688156 0.727329 -0.319235
C -5.093981 -1.418666 -2.500706
H -4.452072 -3.089254 -1.309924
C -4.969805 -0.044584 -2.702189
H -4.024088 1.799043 -2.116713
H -5.765267 -2.001510 -3.129596
N -5.741692 0.592462 -3.765180
O -6.455484 -0.109387 -4.448346
O -5.627568 1.789854 -3.912001
C -3.660473 0.202981 2.045823
C -4.822980 -0.608180 2.109785
C -3.831631 1.607531 2.109699
C -6.083177 -0.043648 2.198326
H -4.719137 -1.693434 2.087985
C -5.089235 2.178915 2.189290
H -2.938914 2.234281 2.085157
C -6.209578 1.346789 2.227577
H -6.976272 -0.665536 2.246022
H -5.219300 3.259785 2.224096
N -7.536371 1.940428 2.312222
O -7.620193 3.150416 2.346514
O -8.494731 1.197154 2.343985
H -2.624262 -2.904696 0.142960

CF3_Michael_Acceptor_TS-MA-conf/TS-MA-S-xii

Frequencies, energies and thermodynamic properties:

Lowest Vibrational Mode (1/cm) = -43.1607

2nd Lowest Vibrational Mode (1/cm) = 9.9637

E(RM062X) (a.u.) = -3187.91958643

Thermal correction to Enthalpy (a.u.) = 0.680543

Thermal correction to Gibbs Free Energy (a.u.) = 0.549410

Total Entropy (cal/Kmol) = 275.993

Esp(RM062X) (a.u.) = -3190.89060599

Optimised cartesian coordinates (Angstrom):

C 3.353560 5.813001 1.271547
C 1.958510 5.957374 1.305436
C 1.122054 4.893711 0.985721
C 1.715601 3.682415 0.629644

C	3.103614	3.544711	0.586871
H	1.521694	6.915572	1.588666
H	0.036835	5.000676	1.013515
C	2.464843	1.421509	-0.016724
C	4.020713	-0.427720	-0.478527
C	4.961256	0.699043	-0.938752
C	4.888095	1.866935	0.027047
H	5.424239	2.735421	-0.377315
S	0.912142	2.188795	0.186417
N	3.484897	2.255356	0.198963
N	2.649303	0.122526	-0.333119
C	6.411029	0.231260	-1.203550
C	7.333248	0.300488	0.016733
C	7.010446	1.038064	-2.355688
H	6.348946	-0.820709	-1.527262
H	8.293006	-0.180965	-0.220101
H	7.552353	1.345926	0.285942
H	6.916912	-0.201210	0.901736
H	8.046386	0.725144	-2.550618
H	6.433525	0.906234	-3.282455
H	7.026738	2.113112	-2.114583
H	4.545982	1.046584	-1.898968
H	3.969672	-1.140761	-1.310204
C	4.464505	-1.159833	0.770786
C	5.181608	-2.353615	0.635505
C	4.223257	-0.649337	2.050577
C	5.679220	-3.010718	1.759235
H	5.350585	-2.770915	-0.360575
C	4.720186	-1.307697	3.175574
H	3.640711	0.265649	2.185860
C	5.455074	-2.484503	3.031875
H	6.237097	-3.940918	1.640677
H	4.527095	-0.900447	4.169285
H	5.841079	-2.999132	3.913234
C	1.730158	-2.137966	-0.746174
H	2.683648	-2.607856	-0.509619
C	0.668530	-2.871608	-1.144256
C	0.865653	-4.322622	-1.483929
F	1.672509	-4.439074	-2.554957
F	1.452779	-5.006389	-0.499334
F	-0.267605	-4.939729	-1.796371
O	0.408662	-0.197964	-0.512058
C	1.505108	-0.713670	-0.533097
H	5.305617	1.624446	1.015667
C	3.947648	4.608311	0.913046
H	5.031442	4.499478	0.895450
H	3.988229	6.660562	1.532791
H	-0.278685	-2.417096	-1.450275
S	-1.048634	-3.457479	0.927697
C	-2.155891	-2.240943	0.432330
N	-2.311560	-1.868006	-0.808302
N	-2.893067	-1.671634	1.482055
C	-2.785821	-0.647552	-1.212479
C	-2.546357	0.562072	-0.509673
C	-3.493781	-0.570360	-2.435734
C	-3.012290	1.771023	-0.992532
H	-1.974180	0.531704	0.416758
C	-3.970358	0.635538	-2.921076
H	-3.664039	-1.494868	-2.989626
C	-3.728189	1.800823	-2.192368
H	-2.828153	2.700968	-0.455523
H	-4.526012	0.689366	-3.856379
N	-4.219029	3.074374	-2.697090

O -4.838657 3.076995 -3.740166

O -3.983784 4.074922 -2.051123

C -4.194586 -1.206733 1.484257

C -5.108159 -1.460814 0.440205

C -4.630219 -0.473778 2.608083

C -6.402985 -0.972050 0.510519

H -4.805580 -2.059290 -0.416739

C -5.924556 0.010697 2.683051

H -3.928601 -0.284515 3.422378

C -6.798686 -0.235104 1.625118

H -7.115440 -1.166405 -0.289995

H -6.262860 0.582788 3.545756

N -8.163032 0.280472 1.693004

O -8.487526 0.900698 2.682474

O -8.901294 0.061319 0.757698

H -2.477509 -1.814368 2.397843

CF3_Michael_Acceptor_TS-MA-conf/TS-MA-S-xi

Frequencies, energies and thermodynamic properties:

Lowest Vibrational Mode (1/cm) = -110.5018

2nd Lowest Vibrational Mode (1/cm) = 10.3080

E(RM062X) (a.u.) = -3187.92199084

Thermal correction to Enthalpy (a.u.) = 0.680431

Thermal correction to Gibbs Free Energy (a.u.) = 0.551262

Total Entropy (cal/Kmol) = 271.858

Esp(RM062X) (a.u.) = -3190.88812167

Optimised cartesian coordinates (Angstrom):

C -6.587421 3.856023 -0.553634

C -5.533404 4.777521 -0.642183

C -4.214795 4.376740 -0.455844

C -3.979338 3.029285 -0.179860

C -5.030474 2.116024 -0.077513

H -5.748628 5.823886 -0.861631

H -3.381851 5.078363 -0.517949

C -3.243301 0.730218 0.333998

C -3.408452 -1.697698 0.685538

C -4.788566 -1.352047 1.272348

C -5.475505 -0.308099 0.411735

H -6.377951 0.066488 0.911388

S -2.432379 2.254971 0.102333

N -4.571106 0.828681 0.226044

N -2.620795 -0.447582 0.547718

C -5.681265 -2.586373 1.539113

C -6.572715 -2.988880 0.361067

C -6.539522 -2.345186 2.781451

H -5.002888 -3.427077 1.758129

H -7.060988 -3.949436 0.580320

H -7.371629 -2.247479 0.201268

H -6.017456 -3.103041 -0.580614

H -7.185147 -3.212498 2.981687

H -5.918582 -2.168384 3.671655

H -7.192545 -1.468639 2.641732

H -4.577155 -0.880562 2.246142

H -2.894472 -2.294133 1.449491

C -3.446936 -2.485794 -0.607410

C -3.353850 -3.881023 -0.550931

C -3.624765 -1.863227 -1.847189

C -3.469045 -4.644931 -1.710691

H -3.189400 -4.371477 0.412011

C -3.739224 -2.627976 -3.008378

H -3.668202 -0.773895 -1.924355

C -3.669192 -4.019311 -2.941778

H -3.394084 -5.731998 -1.653678

H -3.877147 -2.131728 -3.970288
H -3.755438 -4.615382 -3.851654
C -0.535424 -1.767780 0.663940
H -1.078793 -2.690806 0.473835
C 0.815845 -1.778117 0.791920
C 1.508513 -3.084026 1.068570
F 1.204742 -3.493479 2.315260
F 1.120816 -4.058885 0.248027
F 2.834206 -2.998421 1.020785
O -0.600729 0.588854 0.692367
C -1.188338 -0.471768 0.643900
H -5.755573 -0.697984 -0.578325
C -6.355428 2.514572 -0.269166
H -7.181491 1.806710 -0.206109
H -7.612392 4.194682 -0.709590
H 1.360576 -0.875903 1.076195
S 2.034983 -1.470581 -1.554157
C 3.489862 -0.807665 -0.894656
N 3.405331 0.335807 -0.082854
N 4.632412 -1.408165 -1.016909
C 2.490832 1.372044 -0.066901
C 1.687517 1.717451 -1.172759
C 2.409376 2.151401 1.106812
C 0.804260 2.779088 -1.085701
H 1.756860 1.140462 -2.090844
C 1.534687 3.218940 1.194038
H 3.038910 1.892279 1.960287
C 0.727876 3.516707 0.096204
H 0.176826 3.050135 -1.933978
H 1.459301 3.812664 2.104012
N -0.232073 4.602064 0.197680
O -0.206859 5.310340 1.177807
O -1.037078 4.737972 -0.709617
C 5.840872 -0.857074 -0.657115
C 6.759712 -1.654502 0.064005
C 6.248678 0.442525 -1.048505
C 8.015937 -1.178483 0.399985
H 6.450719 -2.660108 0.351765
C 7.507877 0.921519 -0.725012
H 5.565705 1.064896 -1.628473
C 8.378280 0.109107 0.002162
H 8.720721 -1.789424 0.962582
H 7.828012 1.916534 -1.031495
N 9.702015 0.617069 0.349297
O 9.991112 1.741114 -0.001019
O 10.445865 -0.109540 0.972337
H 4.156667 0.429421 0.597850

CF3_Michael_Acceptor_TS-MA-conf/TS-MA-S-xiv

Frequencies, energies and thermodynamic properties:

Lowest Vibrational Mode (1/cm) = -82.8939

2nd Lowest Vibrational Mode (1/cm) = 13.2831

E(RM062X) (a.u.) = -3187.92204605

Thermal correction to Enthalpy (a.u.) = 0.680551

Thermal correction to Gibbs Free Energy (a.u.) = 0.552231

Total Entropy (cal/Kmol) = 270.073

Esp(RM062X) (a.u.) = -3190.89068423

Optimised cartesian coordinates (Angstrom):

C 0.065352 5.942300 -1.378623
C -1.164813 5.486291 -1.874155
C -1.399587 4.128875 -2.068039
C -0.373664 3.239742 -1.748739
C 0.851526 3.696858 -1.260496

H	-1.952118	6.203536	-2.108033
H	-2.356864	3.770872	-2.449987
C	1.228491	1.428585	-1.235033
C	3.240489	0.381990	-0.314708
C	3.945799	1.639364	-0.853358
C	3.113248	2.872251	-0.554952
H	3.495508	3.741721	-1.106255
S	-0.380466	1.493320	-1.904481
N	1.736904	2.643167	-1.005484
N	1.887642	0.297837	-0.924200
C	5.413694	1.776984	-0.386430
C	5.596393	2.526900	0.935923
C	6.244365	2.446622	-1.482015
H	5.797257	0.751663	-0.253815
H	6.646360	2.456507	1.254864
H	5.364640	3.597358	0.817553
H	4.972587	2.128149	1.748269
H	7.292423	2.549308	-1.165496
H	6.224623	1.864501	-2.414757
H	5.862486	3.456503	-1.702044
H	3.959662	1.512505	-1.948284
H	3.795216	-0.476625	-0.710831
C	3.201059	0.277689	1.195321
C	4.126212	-0.553223	1.835917
C	2.316322	1.035951	1.970716
C	4.184317	-0.608604	3.227621
H	4.807450	-1.162762	1.236232
C	2.368637	0.976043	3.362814
H	1.567175	1.677234	1.498368
C	3.308033	0.159996	3.993746
H	4.911077	-1.261013	3.714157
H	1.668374	1.566689	3.955928
H	3.347792	0.113980	5.083188
C	1.979363	-2.150728	-0.755374
H	2.643156	-2.078531	0.104622
C	1.732412	-3.360636	-1.335580
C	2.718409	-4.467088	-1.082281
F	3.844904	-4.229470	-1.780453
F	3.079116	-4.553142	0.197564
F	2.290958	-5.666586	-1.470746
O	0.335477	-0.981861	-1.997838
C	1.312061	-0.981974	-1.272131
H	3.092509	3.118010	0.516835
C	1.091947	5.057732	-1.065065
H	2.043312	5.415884	-0.672831
H	0.222693	7.011363	-1.231017
H	1.197172	-3.421960	-2.283791
S	-0.177614	-4.685291	-0.317912
C	-1.127438	-3.312587	0.138170
N	-2.459951	-3.363036	-0.295818
N	-0.633047	-2.335990	0.827929
C	-3.313948	-2.323945	-0.624352
C	-2.835851	-1.050246	-0.987676
C	-4.702159	-2.562134	-0.621385
C	-3.724148	-0.032511	-1.291367
H	-1.762123	-0.881288	-1.056513
C	-5.593247	-1.548712	-0.936272
H	-5.072504	-3.552223	-0.350615
C	-5.094118	-0.286729	-1.254030
H	-3.369965	0.958999	-1.570797
H	-6.668600	-1.720564	-0.924953
N	-6.026083	0.792178	-1.572552
O	-7.211419	0.544323	-1.551410

O -5.565167 1.880383 -1.842660
C -1.347837 -1.382756 1.503492
C -2.499297 -1.671713 2.276448
C -0.878999 -0.050875 1.485640
C -3.164440 -0.667791 2.959653
H -2.868357 -2.696907 2.325097
C -1.541174 0.961041 2.157483
H 0.029281 0.154907 0.921080
C -2.687610 0.642909 2.886845
H -4.053544 -0.884229 3.550684
H -1.186401 1.991293 2.129148
N -3.392139 1.699859 3.601461
O -2.945225 2.826085 3.538973
O -4.389338 1.402616 4.223443
H -2.782487 -4.296259 -0.534782

CF3_Michael_Acceptor_TS-MA-conf/TS-MA-S-x
Frequencies, energies and thermodynamic properties:
Lowest Vibrational Mode (1/cm) = -89.8842
2nd Lowest Vibrational Mode (1/cm) = 13.0595
E(RM062X) (a.u.) = -3187.92330888
Thermal correction to Enthalpy (a.u.) = 0.680228
Thermal correction to Gibbs Free Energy (a.u.) = 0.552868
Total Entropy (cal/Kmol) = 268.053
Esp(RM062X) (a.u.) = -3190.89001089
Optimised cartesian coordinates (Angstrom):
C 8.013971 -1.688748 -1.985323
C 7.530727 -2.710757 -2.815716
C 6.209207 -3.136324 -2.731235
C 5.382153 -2.512270 -1.797362
C 5.868796 -1.501798 -0.967169
H 8.198454 -3.177627 -3.540559
H 5.828736 -3.929601 -3.376156
C 3.681501 -1.585740 -0.263985
C 2.704383 -0.052747 1.375926
C 4.111421 -0.080101 2.002666
C 5.164937 -0.003685 0.913955
H 6.160805 -0.218922 1.323154
S 3.692825 -2.848239 -1.467201
N 4.881445 -1.020397 -0.101595
N 2.593399 -1.178597 0.417608
C 4.323224 0.975627 3.112844
C 4.876196 2.314656 2.617490
C 5.234246 0.406052 4.200830
H 3.335182 1.160804 3.565041
H 4.299456 2.735894 1.781633
H 4.858787 3.046103 3.438341
H 5.924636 2.212624 2.295362
H 4.799985 -0.495058 4.657808
H 6.219063 0.136907 3.786129
H 5.399122 1.146982 4.996494
H 4.197081 -1.074497 2.470494
H 2.003702 -0.278056 2.187723
C 2.333523 1.276740 0.755577
C 1.612060 2.197832 1.523002
C 2.787432 1.653143 -0.513994
C 1.395221 3.492981 1.054040
H 1.229611 1.898927 2.502476
C 2.573408 2.949222 -0.981267
H 3.313256 0.940677 -1.154476
C 1.891713 3.875903 -0.192032
H 0.830779 4.204931 1.659531
H 2.934981 3.234229 -1.970782

H 1.719184 4.887916 -0.564746
C 0.190632 -1.401688 0.963327
H 0.113185 -0.361387 1.278241
C -0.837581 -2.277204 1.125662
C -1.954218 -1.928652 2.068050
F -1.488802 -1.832403 3.325356
F -2.525609 -0.749824 1.793536
F -2.916938 -2.849559 2.097901
O 1.379235 -2.918965 -0.397112
C 1.360674 -1.904784 0.268862
H 5.191040 0.980553 0.424029
C 7.193526 -1.069118 -1.048873
H 7.570803 -0.270012 -0.411286
H 9.052900 -1.369218 -2.074625
H -0.687622 -3.341968 0.950306
S -2.376112 -2.416310 -0.959658
C -2.481249 -0.701895 -1.109964
N -1.418759 0.028885 -1.282376
N -3.732496 -0.072481 -1.120534
H -3.717548 0.852350 -1.544669
C -1.424964 1.401466 -1.167497
C -0.828149 2.185076 -2.180924
C -1.950998 2.065467 -0.030244
C -0.764402 3.564659 -2.074967
H -0.413716 1.675185 -3.051479
C -1.884523 3.443518 0.084641
H -2.402127 1.472333 0.766101
C -1.290352 4.180922 -0.939892
H -0.297182 4.169893 -2.850961
H -2.271513 3.955270 0.965044
N -1.138904 5.620727 -0.778553
O -1.624825 6.140930 0.203058
O -0.521725 6.229516 -1.628844
C -5.008751 -0.506681 -0.812435
C -6.080402 0.215045 -1.383884
C -5.302783 -1.565777 0.071328
C -7.396089 -0.109206 -1.104203
H -5.859953 1.039997 -2.064061
C -6.621028 -1.896305 0.347728
H -4.497817 -2.122387 0.539533
C -7.655446 -1.172876 -0.241789
H -8.220189 0.444829 -1.551027
H -6.854808 -2.710229 1.032629
O -9.239669 -2.463524 0.801054
N -9.039668 -1.527690 0.057697
O -9.918862 -0.867132 -0.452081

CF3_Michael_Acceptor_TS-MA-conf/TS-MA-S-xvi

Frequencies, energies and thermodynamic properties:

Lowest Vibrational Mode (1/cm) = -115.9437
2nd Lowest Vibrational Mode (1/cm) = 5.6041
E(RM062X) (a.u.) = -3187.91917952
Thermal correction to Enthalpy (a.u.) = 0.680569
Thermal correction to Gibbs Free Energy (a.u.) = 0.551373
Total Entropy (cal/Kmol) = 271.916
Esp(RM062X) (a.u.) = -3190.88588653

Optimised cartesian coordinates (Angstrom):

C 8.237596 -1.972564 -2.025653
C 7.691356 -3.088342 -2.676241
C 6.367546 -3.460746 -2.466344
C 5.603661 -2.688726 -1.591366
C 6.152712 -1.583774 -0.939207
H 8.310436 -3.671969 -3.358414

H	5.937527	-4.326592	-2.971887
C	4.001982	-1.508330	-0.126414
C	3.138770	0.252328	1.342120
C	4.581115	0.290253	1.879829
C	5.567460	0.194439	0.730811
H	6.585541	0.031397	1.108133
S	3.925586	-2.928070	-1.138113
N	5.220190	-0.957239	-0.105386
N	2.961249	-0.983414	0.542618
C	4.868101	1.486647	2.816770
C	5.388240	2.739280	2.106529
C	5.850279	1.063490	3.909760
H	3.912894	1.741733	3.304430
H	5.425781	3.576828	2.818015
H	6.413039	2.583499	1.733875
H	4.758557	3.047739	1.259850
H	6.072360	1.906578	4.579951
H	5.445959	0.241130	4.517664
H	6.803335	0.725962	3.471453
H	4.687824	-0.629709	2.477831
H	2.488779	0.138572	2.217531
C	2.726149	1.495069	0.582343
C	2.041170	2.505469	1.265702
C	3.095243	1.702754	-0.751643
C	1.783707	3.728865	0.648098
H	1.720134	2.335867	2.296761
C	2.837990	2.926129	-1.370045
H	3.593609	0.914115	-1.321297
C	2.203682	3.948873	-0.664137
H	1.251678	4.513216	1.189303
H	3.134720	3.082270	-2.408518
H	2.004055	4.906228	-1.147893
C	0.571839	-1.002427	1.133306
H	0.577872	0.065107	1.345637
C	-0.569229	-1.743592	1.310002
C	-1.614030	-1.196320	2.242694
F	-1.197764	-1.349665	3.511969
F	-1.821424	0.111779	2.068504
F	-2.792343	-1.808721	2.162566
O	1.645216	-2.758693	-0.013807
C	1.689570	-1.663811	0.517970
H	5.567974	1.095340	0.099414
C	7.479773	-1.203791	-1.148537
H	7.908208	-0.334766	-0.649880
H	9.276781	-1.697745	-2.210102
H	-0.517846	-2.832595	1.284223
S	-1.865124	-2.064819	-0.792644
C	-3.444614	-1.385487	-0.519990
N	-3.562161	-0.015099	-0.268289
N	-4.488601	-2.142037	-0.432417
C	-2.751926	1.048993	-0.616421
C	-1.863912	1.029262	-1.711502
C	-2.884372	2.238487	0.131759
C	-1.143453	2.165171	-2.045591
H	-1.751628	0.124065	-2.301461
C	-2.161677	3.372287	-0.195574
H	-3.567317	2.255182	0.983228
C	-1.298387	3.325532	-1.289271
H	-0.469971	2.164178	-2.901682
H	-2.262023	4.290914	0.380800
N	-0.556652	4.520814	-1.661422
O	-0.558003	5.458440	-0.891120
O	0.035671	4.518979	-2.719080

C -5.781186 -1.662460 -0.364685
C -6.308532 -0.742171 -1.301512
C -6.642090 -2.174513 0.630801
C -7.635646 -0.347492 -1.239636
H -5.663080 -0.353838 -2.090660
C -7.967119 -1.776087 0.705858
H -6.236676 -2.893980 1.343067
C -8.450210 -0.864360 -0.232259
H -8.049659 0.353966 -1.962846
H -8.632087 -2.163199 1.476781
N -9.847512 -0.442193 -0.161243
O -10.539769 -0.911494 0.715607
O -10.242470 0.357521 -0.981577
H -4.370490 0.242314 0.294326

CF3_Michael_Acceptor_TS-MA-conf/TS-MA-S-xv
Frequencies, energies and thermodynamic properties:
Lowest Vibrational Mode (1/cm) = -82.0437
2nd Lowest Vibrational Mode (1/cm) = 5.3567
E(RM062X) (a.u.) = -3187.92592216
Thermal correction to Enthalpy (a.u.) = 0.680024
Thermal correction to Gibbs Free Energy (a.u.) = 0.551067
Total Entropy (cal/Kmol) = 271.413
Esp(RM062X) (a.u.) = -3190.89293419
Optimised cartesian coordinates (Angstrom):
C -5.849265 -3.300040 -2.718221
C -5.240471 -3.076770 -3.961376
C -4.273463 -2.088335 -4.117840
C -3.925137 -1.335164 -2.997759
C -4.539241 -1.554341 -1.762816
H -5.525835 -3.686271 -4.819492
H -3.797159 -1.910642 -5.082995
C -3.083201 0.130707 -1.197844
C -2.695958 0.885688 1.089018
C -4.212001 0.650887 1.254767
C -4.616068 -0.645903 0.575128
H -5.708680 -0.704305 0.476079
S -2.774194 -0.014218 -2.907706
N -4.055404 -0.684824 -0.779059
N -2.396945 0.925838 -0.362897
C -4.708523 0.739212 2.716947
C -4.681116 -0.584435 3.486270
C -6.120495 1.325782 2.748081
H -4.038661 1.447806 3.231771
H -4.907398 -0.396685 4.545862
H -5.450891 -1.276081 3.108523
H -3.708404 -1.094253 3.437410
H -6.490673 1.392645 3.781383
H -6.147764 2.333845 2.309433
H -6.820676 0.689081 2.183495
H -4.687697 1.473968 0.696876
H -2.493607 1.897444 1.459381
C -1.846193 -0.095109 1.868512
C -1.345635 0.303823 3.113073
C -1.672774 -1.424196 1.462142
C -0.743905 -0.620921 3.966529
H -1.450098 1.345897 3.425572
C -1.087432 -2.351950 2.323101
H -2.007678 -1.757367 0.476677
C -0.635910 -1.957821 3.583828
H -0.366969 -0.296346 4.937913
H -0.971970 -3.388828 1.999892
H -0.176247 -2.687199 4.252789

C -0.650601 2.618971 0.003751
H -0.331547 2.104853 0.912269
C -0.110141 3.811178 -0.376629
C 0.717110 4.564094 0.624825
F -0.046170 4.974917 1.654115
F 1.681960 3.804961 1.160842
F 1.286839 5.656326 0.124344
O -1.686644 2.242708 -2.086890
C -1.558442 1.972688 -0.914352
H -4.267593 -1.534304 1.120960
C -5.510652 -2.543026 -1.601106
H -5.982607 -2.720471 -0.634695
H -6.602403 -4.082864 -2.620833
H -0.575777 4.406828 -1.160246
S 1.779040 3.520368 -2.053756
C 2.187778 1.984014 -1.392983
N 1.346916 0.995155 -1.357295
N 3.480462 1.916040 -0.846473
C 1.478614 -0.089783 -0.519320
C 1.865012 0.032839 0.837236
C 1.171412 -1.375434 -1.016605
C 2.018279 -1.087882 1.633429
H 2.067225 1.024387 1.245001
C 1.320794 -2.502514 -0.224533
H 0.840367 -1.462292 -2.052758
C 1.759622 -2.347206 1.089426
H 2.323597 -1.003098 2.675593
H 1.100786 -3.498271 -0.607556
N 1.855874 -3.517323 1.949588
O 1.483696 -4.585220 1.507743
O 2.288627 -3.368012 3.072810
C 4.364611 0.856391 -0.818007
C 4.207910 -0.293987 -1.618319
C 5.488490 0.950874 0.029322
C 5.125467 -1.329507 -1.541577
H 3.373328 -0.364839 -2.313676
C 6.410631 -0.078604 0.101988
H 5.617722 1.846104 0.640173
C 6.212894 -1.218093 -0.676990
H 5.012170 -2.221992 -2.155454
H 7.274554 -0.015546 0.761953
N 7.174805 -2.313219 -0.595428
O 8.126825 -2.179593 0.142504
O 6.972815 -3.299435 -1.269982
H 3.864879 2.822551 -0.596712

Me_Michael_Acceptor/acyl_ammonium_ion_pair

Frequencies, energies and thermodynamic properties:

Lowest Vibrational Mode (1/cm) = 17.2774

2nd Lowest Vibrational Mode (1/cm) = 23.2636

E(RM062X) (a.u.) = -2275.21374105

Thermal correction to Enthalpy (a.u.) = 0.527841

Thermal correction to Gibbs Free Energy (a.u.) = 0.423224

Total Entropy (cal/Kmol) = 220.185

Esp(RM062X) (a.u.) = -2277.48416201

Optimised cartesian coordinates (Angstrom):

C -5.157437 3.666703 0.426376
C -4.540885 4.584538 -0.436178
C -3.331258 4.282575 -1.053210
C -2.755431 3.040167 -0.788730
C -3.368146 2.131269 0.075701
C -4.582849 2.429161 0.696333
H -6.107075 3.924398 0.896789

H	-5.014833	5.547862	-0.627808
H	-2.847085	4.993498	-1.724061
H	-5.069472	1.719376	1.364466
C	-1.507142	0.923472	-0.530577
C	-1.014569	-1.355786	0.233865
C	-1.749850	-0.897641	1.501758
C	-2.996768	-0.117985	1.130652
H	-3.439290	0.353123	2.018096
S	-1.241019	2.408176	-1.407565
N	-2.621452	0.956909	0.208371
N	-0.722650	-0.163045	-0.599398
C	-2.030675	-2.037419	2.505291
C	-3.371052	-2.747860	2.299583
C	-1.934734	-1.501541	3.934100
H	-1.225472	-2.778499	2.372787
H	-3.521819	-3.089284	1.265323
H	-3.427760	-3.629536	2.954597
H	-4.212283	-2.089265	2.569032
H	-0.935334	-1.090983	4.138147
H	-2.673470	-0.701156	4.102833
H	-2.134652	-2.299281	4.664496
H	-1.040122	-0.199992	1.974183
H	-0.035057	-1.698214	0.583941
C	-1.707292	-2.439108	-0.562334
C	-1.347070	-3.773547	-0.341035
C	-2.729740	-2.152884	-1.473554
C	-2.018903	-4.806218	-0.993147
H	-0.532651	-4.001120	0.351480
C	-3.401134	-3.185814	-2.128575
H	-3.007348	-1.117568	-1.686992
C	-3.052529	-4.514261	-1.884129
H	-1.729743	-5.842412	-0.809857
H	-4.197810	-2.950135	-2.836156
H	-3.577929	-5.321199	-2.397583
C	1.305892	-1.315291	-1.450180
H	0.887383	-2.280305	-1.167936
C	2.563821	-1.219732	-1.906192
H	2.940104	-0.224208	-2.166893
C	3.496143	-2.370165	-2.060266
O	0.835653	0.978866	-1.795881
C	0.517592	-0.080465	-1.305244
H	-3.760456	-0.746559	0.648032
O	1.313422	-0.054906	1.239489
C	2.500680	0.285086	0.984724
C	2.895231	1.629447	0.700504
C	3.570581	-0.651342	0.838335
C	4.198915	1.998605	0.410078
C	4.874091	-0.290772	0.537254
C	5.203728	1.039433	0.296002
F	3.296368	-1.953918	1.009625
F	5.813318	-1.234383	0.411403
F	4.498385	3.282223	0.197572
F	1.963501	2.589045	0.776061
F	6.461086	1.395176	0.004057
H	3.021129	-3.321866	-1.788554
H	4.382918	-2.223420	-1.423409
H	3.861378	-2.431854	-3.097272

Me_Michael_Acceptor/C6F5OH

Frequencies, energies and thermodynamic properties:

Lowest Vibrational Mode (1/cm) = 111.0785

2nd Lowest Vibrational Mode (1/cm) = 141.1056

E(RM062X) (a.u.) = -802.732617403

Thermal correction to Enthalpy (a.u.) = 0.075937

Thermal correction to Gibbs Free Energy (a.u.) = 0.029109

Total Entropy (cal/Kmol) = 98.557

Esp(RM062X) (a.u.) = -803.686675322

Optimised cartesian coordinates (Angstrom):

C -0.234767 1.367630 -0.000143
C 1.071480 0.891834 0.000088
C 1.323220 -0.477089 -0.000141
C 0.255627 -1.367778 0.000104
C -1.046230 -0.882459 -0.000210
C -1.319165 0.486396 0.000091
O -2.565435 0.976289 -0.000016
F -2.074173 -1.730692 -0.000105
F 0.478209 -2.671996 0.000128
F 2.569392 -0.926193 -0.000068
F 2.082269 1.746196 0.000114
F -0.452829 2.675256 -0.000055
H -3.203310 0.245342 0.001266

Me_Michael_Acceptor/cycl-R_rot

Frequencies, energies and thermodynamic properties:

Lowest Vibrational Mode (1/cm) = 14.6168

2nd Lowest Vibrational Mode (1/cm) = 18.9956

E(RM062X) (a.u.) = -2890.51631942

Thermal correction to Enthalpy (a.u.) = 0.701812

Thermal correction to Gibbs Free Energy (a.u.) = 0.581546

Total Entropy (cal/Kmol) = 253.121

Esp(RM062X) (a.u.) = -2893.12138078

Optimised cartesian coordinates (Angstrom):

C 0.307192 -1.149736 -2.851552
C -1.069551 -1.018909 -3.474428
H -1.251361 -1.891297 -4.118839
H 0.373470 -2.127154 -2.356401
H 1.080851 -1.114365 -3.631567
N -0.520046 0.582444 -1.129076
C -0.274693 1.826265 -0.464566
C -0.400805 1.941307 0.926232
C 0.076170 2.954445 -1.221584
C -0.145951 3.151524 1.561355
H -0.718545 1.084021 1.513192
C 0.336312 4.168896 -0.596917
H 0.150320 2.864703 -2.301276
C 0.228395 4.243943 0.787452
H -0.236462 3.250206 2.641987
H 0.610400 5.052246 -1.171368
N 0.503510 5.525672 1.454091
O 0.832287 6.462878 0.763535
O 0.387748 5.573093 2.657463
C 0.683813 0.038736 -1.932409
O 1.358005 0.944980 -2.489699
C -1.301791 0.257305 -4.270520
S -2.291397 -1.245114 -2.134638
C -1.776125 0.042891 -1.007496
N -2.595230 0.443329 -0.106938
C -3.933343 0.105098 -0.043504
C -4.856123 0.558353 -1.010255
C -4.409181 -0.638993 1.053787
C -6.205647 0.266554 -0.891240
H -4.494683 1.150067 -1.852194
C -5.757629 -0.940219 1.177155
H -3.696126 -0.969173 1.809887
C -6.640189 -0.484342 0.200640
H -6.925710 0.613673 -1.630978

H -6.133568 -1.519086 2.019512
N -8.064250 -0.795953 0.328337
O -8.419457 -1.447168 1.285964
O -8.814070 -0.387767 -0.530737
N 1.575492 -0.658058 -0.752165
C 2.867314 -0.453125 -0.727213
C 0.991766 -1.696109 0.101783
S 3.697009 0.859156 -1.565231
N 3.723174 -1.218640 -0.010280
C 1.955292 -2.898699 0.151447
H 0.073693 -2.030730 -0.401508
C 0.579444 -1.177015 1.464436
C 5.216463 0.350037 -0.827926
C 5.049222 -0.778635 -0.021276
C 3.308339 -2.441385 0.677399
C 1.397173 -4.132593 0.896576
H 2.094958 -3.187545 -0.904072
C -0.673080 -1.542450 1.968608
C 1.421171 -0.380146 2.250577
C 6.471817 0.931685 -0.985020
C 6.124689 -1.346263 0.661068
H 4.072578 -3.208014 0.486429
H 3.283028 -2.245910 1.760118
C 1.680728 -4.158936 2.400959
C 1.919388 -5.416348 0.249661
H 0.302799 -4.108754 0.758427
C -1.075184 -1.132809 3.240388
H -1.344139 -2.145709 1.350986
C 1.021273 0.029011 3.522236
H 2.389695 -0.047625 1.870859
C 7.553016 0.368693 -0.307334
H 6.603502 1.809664 -1.619684
C 7.378454 -0.755968 0.506372
H 5.991892 -2.220266 1.298748
H 1.390189 -3.228897 2.909617
H 1.122375 -4.984136 2.866663
H 2.749413 -4.338571 2.598589
H 1.625354 -5.482974 -0.808076
H 3.019297 -5.460575 0.299914
H 1.526875 -6.302864 0.769059
C -0.226694 -0.347313 4.021563
H -2.056121 -1.423371 3.621246
H 1.684126 0.655431 4.121696
H 8.543303 0.813541 -0.412399
H 8.234607 -1.181287 1.031726
H -0.540292 -0.022572 5.015090
H -2.299301 0.249977 -4.730841
H -0.548234 0.334338 -5.068692
H -1.211305 1.150152 -3.636671

Me_Michael_Acceptor/cycl-R
Frequencies, energies and thermodynamic properties:
Lowest Vibrational Mode (1/cm) = 9.9210
2nd Lowest Vibrational Mode (1/cm) = 15.8179
E(RM062X) (a.u.) = -2890.52310822
Thermal correction to Enthalpy (a.u.) = 0.701846
Thermal correction to Gibbs Free Energy (a.u.) = 0.580234
Total Entropy (cal/Kmol) = 255.953
Esp(RM062X) (a.u.) = -2893.13007809
Optimised cartesian coordinates (Angstrom):
C 1.512266 -2.842279 -1.678695
C 2.298332 -0.283019 -0.609000
C -0.086601 -0.995988 -1.217775

C	0.290826	-2.449764	-0.882997
H	0.459590	-2.616017	0.190968
H	-0.560712	-3.069185	-1.205546
N	0.946702	-0.066050	-0.523238
C	0.521380	1.244940	-0.182665
C	-0.272592	1.983622	-1.069162
C	0.834464	1.771636	1.080363
C	-0.799937	3.211283	-0.678793
H	-0.478808	1.566960	-2.054738
C	0.326914	3.000739	1.473569
H	1.470343	1.200707	1.757246
C	-0.500851	3.692345	0.590900
H	-1.433519	3.792868	-1.347102
H	0.550885	3.419275	2.453732
N	-1.084750	4.968065	1.025356
O	-0.937151	5.289079	2.183448
O	-1.688523	5.622453	0.207250
O	-0.269841	-0.738643	-2.445216
S	2.953771	-1.872569	-1.096827
N	3.125564	0.658704	-0.333963
C	4.498218	0.519501	-0.277758
C	5.124161	-0.253953	0.722876
C	5.304046	1.228648	-1.191048
C	6.506506	-0.327778	0.800573
H	4.507932	-0.793287	1.443652
C	6.686882	1.156613	-1.122866
H	4.816852	1.835290	-1.954960
C	7.272054	0.377212	-0.126397
H	6.998655	-0.922127	1.569110
H	7.316949	1.695159	-1.829376
N	8.731244	0.300527	-0.047679
O	9.377387	0.928686	-0.856865
O	9.217608	-0.387723	0.822497
H	1.341339	-2.567333	-2.727752
C	1.872366	-4.314167	-1.558780
N	-1.400003	-0.706218	-0.379039
C	-2.497438	-0.353054	-1.012210
C	-1.411407	-0.831901	1.086873
S	-2.678215	-0.280287	-2.763987
N	-3.639611	-0.023983	-0.371557
C	-2.334822	0.263859	1.659420
H	-0.388774	-0.606137	1.416814
C	-1.739696	-2.236681	1.559451
C	-4.382371	0.127303	-2.557383
C	-4.730761	0.226636	-1.209378
C	-3.729647	0.097872	1.082310
C	-2.329312	0.384412	3.198470
H	-1.929033	1.208647	1.259892
C	-1.019363	-2.766488	2.636128
C	-2.759953	-3.002335	0.985047
C	-5.327500	0.352495	-3.555392
C	-6.032482	0.539016	-0.817742
H	-4.343451	0.981160	1.305738
H	-4.241970	-0.790494	1.483099
C	-3.328290	-0.528061	3.916693
C	-2.585010	1.837004	3.606616
H	-1.311644	0.112995	3.528425
C	-1.330106	-4.023965	3.150656
H	-0.204900	-2.184286	3.075093
C	-3.070695	-4.262747	1.497592
H	-3.318914	-2.628502	0.123687
C	-6.630501	0.668325	-3.173148
H	-5.053640	0.280185	-4.609408

C -6.976270 0.758370 -1.819865
H -6.308733 0.604577 0.234650
H -3.261952 -1.578299 3.600686
H -3.148720 -0.492021 5.001217
H -4.361428 -0.184779 3.748507
H -1.840965 2.523049 3.175213
H -3.580820 2.165906 3.267894
H -2.555788 1.945528 4.700750
C -2.362212 -4.773724 2.585101
H -0.760397 -4.422260 3.991949
H -3.869144 -4.849332 1.040049
H -7.387284 0.844249 -3.938574
H -8.001712 1.002781 -1.539560
H -2.606615 -5.759715 2.983736
H 2.018782 -4.600820 -0.506948
H 1.055344 -4.921253 -1.975720
H 2.789913 -4.551496 -2.116983

Me_Michael_Acceptor/cycl-S_rot

Frequencies, energies and thermodynamic properties:

Lowest Vibrational Mode (1/cm) = 10.9628

2nd Lowest Vibrational Mode (1/cm) = 14.6435

E(RM062X) (a.u.) = -2890.52022205

Thermal correction to Enthalpy (a.u.) = 0.701973

Thermal correction to Gibbs Free Energy (a.u.) = 0.580378

Total Entropy (cal/Kmol) = 255.919

Esp(RM062X) (a.u.) = -2893.12557609

Optimised cartesian coordinates (Angstrom):

C 1.534535 -3.005647 -1.488856
C 2.302094 -0.371386 -0.565671
C -0.083181 -1.064129 -1.189929
C 0.258456 -2.516341 -0.825959
H 0.343995 -2.650537 0.260764
H -0.586069 -3.125037 -1.184223
N 0.950898 -0.135242 -0.508426
C 0.540813 1.193633 -0.216465
C -0.242087 1.915231 -1.126466
C 0.860710 1.756992 1.029279
C -0.749640 3.163427 -0.776276
H -0.456473 1.470323 -2.097382
C 0.373442 3.006105 1.381776
H 1.487248 1.199341 1.725700
C -0.442716 3.681998 0.476380
H -1.374169 3.732190 -1.463887
H 0.604114 3.452527 2.347926
N -1.004489 4.981316 0.867623
O -0.849549 5.339319 2.013875
O -1.598561 5.617376 0.028228
O -0.285132 -0.807370 -2.413090
S 2.954266 -1.997780 -0.911077
N 3.134615 0.579106 -0.346288
C 4.505802 0.434281 -0.265421
C 5.115586 -0.231022 0.818916
C 5.324969 1.031329 -1.244296
C 6.496565 -0.307142 0.917029
H 4.487782 -0.683455 1.587922
C 6.706512 0.954552 -1.156549
H 4.849934 1.555019 -2.074331
C 7.276075 0.285036 -0.074941
H 6.976922 -0.817605 1.750544
H 7.347032 1.406780 -1.912437
N 8.733997 0.205896 0.025751
O 9.392471 0.715150 -0.853908

O 9.206756 -0.365152 0.983756
H 1.767365 -3.996360 -1.072385
C 1.506285 -3.103964 -3.008224
N -1.405590 -0.745533 -0.332895
C -2.503362 -0.398202 -0.965942
C -1.406759 -0.820081 1.136431
S -2.702738 -0.379275 -2.718753
N -3.636828 -0.033687 -0.326852
C -2.309125 0.307626 1.680782
H -0.377928 -0.598961 1.450256
C -1.752657 -2.201757 1.662051
C -4.400531 0.050554 -2.509115
C -4.733320 0.201003 -1.161863
C -3.711298 0.141279 1.122103
C -2.288106 0.479359 3.215007
H -1.894197 1.232909 1.246411
C -1.027654 -2.704289 2.748588
C -2.792828 -2.970552 1.128615
C -5.353110 0.252669 -3.504880
C -6.026959 0.543860 -0.768722
H -4.310401 1.040828 1.319198
H -4.232536 -0.724234 1.559579
C -3.292106 -0.394850 3.972845
C -2.522189 1.947802 3.576705
H -1.270918 0.206162 3.545280
C -1.353063 -3.936224 3.313482
H -0.197933 -2.120545 3.155628
C -3.118233 -4.205483 1.691613
H -3.356942 -2.619247 0.261193
C -6.648301 0.597686 -3.121035
H -5.091070 0.140668 -4.558416
C -6.978574 0.739672 -1.768471
H -6.291745 0.650405 0.283291
H -3.240676 -1.456545 3.694842
H -3.103634 -0.321744 5.053990
H -4.322411 -0.045456 3.799977
H -1.774514 2.609783 3.115346
H -3.517328 2.277219 3.236659
H -2.480870 2.092460 4.666260
C -2.405034 -4.688117 2.788991
H -0.779490 -4.312886 4.162083
H -3.932330 -4.794604 1.265991
H -7.410865 0.755944 -3.884599
H -7.997871 1.006904 -1.486543
H -2.661338 -5.654050 3.227271
H 1.345214 -2.120975 -3.463746
H 0.679453 -3.764010 -3.314172
H 2.446034 -3.537348 -3.379081

Me_Michael_Acceptor/cycl-S

Frequencies, energies and thermodynamic properties:

Lowest Vibrational Mode (1/cm) = 7.9531

2nd Lowest Vibrational Mode (1/cm) = 18.1811

E(RM062X) (a.u.) = -2890.51608262

Thermal correction to Enthalpy (a.u.) = 0.701853

Thermal correction to Gibbs Free Energy (a.u.) = 0.579743

Total Entropy (cal/Kmol) = 257.002

Esp(RM062X) (a.u.) = -2893.12243592

Optimised cartesian coordinates (Angstrom):

C -0.273153 -1.363709 2.731307
C 1.089343 -1.235253 3.373373
H 1.191577 -0.251845 3.856404
H -0.299686 -2.298019 2.152776

H	-1.050920	-1.421191	3.506350
N	0.523311	0.538139	1.186017
C	0.251615	1.831328	0.637275
C	0.363056	2.075375	-0.737886
C	-0.116160	2.876856	1.497533
C	0.075810	3.332842	-1.257434
H	0.694521	1.282588	-1.402736
C	-0.409166	4.136908	0.988867
H	-0.173387	2.685957	2.565009
C	-0.316153	4.340567	-0.383786
H	0.154247	3.532039	-2.325126
H	-0.697129	4.957706	1.643676
N	-0.627920	5.671041	-0.927645
O	-0.970186	6.533007	-0.150997
O	-0.527311	5.831072	-2.122622
C	-0.676770	-0.103326	1.927395
O	-1.361385	0.735256	2.571739
C	1.386617	-2.341917	4.372867
S	2.331366	-1.321679	2.037614
C	1.785135	0.030893	1.005961
N	2.587680	0.510780	0.129316
C	3.931242	0.203482	0.031482
C	4.851493	0.583877	1.031716
C	4.414252	-0.430313	-1.130204
C	6.204890	0.325321	0.883312
H	4.485359	1.092330	1.924355
C	5.766595	-0.698415	-1.283433
H	3.703902	-0.701048	-1.912036
C	6.646144	-0.318640	-0.272227
H	6.922724	0.616632	1.648886
H	6.148149	-1.193248	-2.175363
N	8.073997	-0.597058	-0.430662
O	8.433675	-1.163281	-1.439326
O	8.822238	-0.248485	0.455595
N	-1.555834	-0.694035	0.693732
C	-2.850227	-0.496550	0.685594
C	-0.964985	-1.645980	-0.251864
S	-3.690852	0.729137	1.634907
N	-3.698406	-1.201033	-0.098691
C	-1.918342	-2.847477	-0.408917
H	-0.042810	-2.016404	0.217612
C	-0.565282	-1.002311	-1.564047
C	-5.204617	0.279502	0.848329
C	-5.027457	-0.772288	-0.054211
C	-3.274243	-2.354117	-0.892319
C	-1.353221	-4.004031	-1.264915
H	-2.054501	-3.232417	0.615869
C	0.686853	-1.308117	-2.107195
C	-1.420504	-0.147425	-2.270587
C	-6.464197	0.838018	1.050040
C	-6.096352	-1.283977	-0.789241
H	-4.033219	-3.139638	-0.771515
H	-3.248516	-2.061694	-1.953021
C	-1.639782	-3.895137	-2.765126
C	-1.867611	-5.344762	-0.738148
H	-0.258671	-3.986060	-1.126881
C	1.075522	-0.781538	-3.339452
H	1.368493	-1.957800	-1.551399
C	-1.034447	0.378016	-3.502967
H	-2.389183	0.140411	-1.856963
C	-7.538756	0.331562	0.319349
H	-6.603964	1.655654	1.759160
C	-7.354024	-0.715970	-0.589638

H -5.955633 -2.097076 -1.501579
H -1.356317 -2.921089 -3.188207
H -1.077167 -4.671238 -3.304364
H -2.707720 -4.063556 -2.976366
H -1.574285 -5.505750 0.309548
H -2.967289 -5.390578 -0.794155
H -1.469291 -6.177757 -1.335783
C 0.213719 0.061589 -4.041829
H 2.056704 -1.026178 -3.750633
H -1.708347 1.048308 -4.039338
H -8.532011 0.760453 0.457626
H -8.205234 -1.097164 -1.155364
H 0.517043 0.477406 -5.004094
H 0.662619 -2.288163 5.198565
H 1.297695 -3.332080 3.901117
H 2.396975 -2.243514 4.794194

Me_Michael_Acceptor/HyperBTM

Frequencies, energies and thermodynamic properties:

Lowest Vibrational Mode (1/cm) = 26.7604

2nd Lowest Vibrational Mode (1/cm) = 57.9421

E(RM062X) (a.u.) = -1242.68108630

Thermal correction to Enthalpy (a.u.) = 0.371678

Thermal correction to Gibbs Free Energy (a.u.) = 0.305623

Total Entropy (cal/Kmol) = 139.024

Esp(RM062X) (a.u.) = -1243.74354485

Optimised cartesian coordinates (Angstrom):

C -4.262530 0.973031 1.705921
C -5.081884 0.026598 1.089062
C -4.570448 -0.806552 0.088982
C -3.238265 -0.667538 -0.277562
C -2.412615 0.292849 0.336285
C -2.921403 1.115944 1.340791
H -4.669698 1.613207 2.490089
H -6.126268 -0.070457 1.387912
H -5.203340 -1.552803 -0.394046
H -2.284078 1.850300 1.833836
C -0.852054 -0.663172 -1.127167
C 1.406622 -0.103443 -1.317985
C 0.986323 1.302948 -0.824072
C -0.065141 1.190869 0.277299
H -0.509874 2.177247 0.477622
S -2.342062 -1.583659 -1.484368
N -1.120558 0.291010 -0.162611
N 0.251017 -0.908789 -1.701688
C 2.161531 2.231026 -0.444693
C 2.601261 2.139383 1.019395
C 1.826137 3.682431 -0.791325
H 3.015788 1.927239 -1.073553
H 2.788244 1.106938 1.347021
H 3.529866 2.711096 1.165556
H 1.842194 2.577462 1.687042
H 1.618759 3.801777 -1.864957
H 0.937651 4.022659 -0.235092
H 2.658630 4.352142 -0.528432
H 0.480326 1.757972 -1.692374
H 2.009511 0.051835 -2.226024
C 2.288128 -0.851872 -0.332650
C 3.679294 -0.826890 -0.482869
C 1.743460 -1.531774 0.763657
C 4.511225 -1.434125 0.457769
H 4.115252 -0.320870 -1.347869
C 2.572640 -2.139839 1.706383

H 0.659540 -1.591953 0.887188
C 3.959687 -2.086725 1.560541
H 5.594333 -1.401918 0.325517
H 2.131945 -2.662349 2.557484
H 4.607870 -2.563537 2.297932
H 0.371335 0.818121 1.218567

Me_Michael_Acceptor/MA-R_pt_rot

Frequencies, energies and thermodynamic properties:

Lowest Vibrational Mode (1/cm) = 12.3327
2nd Lowest Vibrational Mode (1/cm) = 17.2725
E(RM062X) (a.u.) = -2890.52043429
Thermal correction to Enthalpy (a.u.) = 0.701818
Thermal correction to Gibbs Free Energy (a.u.) = 0.581463
Total Entropy (cal/Kmol) = 253.307
Esp(RM062X) (a.u.) = -2893.12629120

Optimised cartesian coordinates (Angstrom):

C 5.062028 -1.448523 -2.428759
C 5.366933 -2.298905 -1.353776
C 4.907980 -2.022167 -0.071151
C 4.122628 -0.881646 0.106210
C 3.825346 -0.036311 -0.963292
H 5.956120 -3.200222 -1.527651
H 5.129476 -2.689406 0.763168
C 2.581150 0.946406 0.694956
C 1.167941 2.938778 0.389149
C 2.282283 3.330655 -0.600526
C 2.661734 2.135888 -1.459252
H 3.559344 2.357172 -2.052403
S 3.341607 -0.340215 1.574896
N 2.992546 1.016665 -0.574166
N 1.640747 1.779570 1.196278
C 1.959873 4.590147 -1.438754
C 1.246750 4.306562 -2.763841
C 3.245025 5.376917 -1.700262
H 1.299414 5.221955 -0.822274
H 0.349455 3.682878 -2.647525
H 0.937649 5.255732 -3.225041
H 1.923338 3.806421 -3.474601
H 3.719484 5.697617 -0.761408
H 3.972251 4.764787 -2.257765
H 3.035197 6.273551 -2.301218
H 3.159072 3.568263 0.024019
H 1.070616 3.776508 1.091913
C -0.166148 2.690256 -0.274681
C -1.111644 3.718977 -0.315512
C -0.435341 1.485033 -0.926054
C -2.293782 3.559329 -1.036995
H -0.916523 4.655954 0.212593
C -1.617619 1.321116 -1.645106
H 0.275207 0.658790 -0.859039
C -2.542020 2.364704 -1.713959
H -3.023753 4.369860 -1.070015
H -1.822801 0.372908 -2.146811
H -3.467294 2.236691 -2.278719
C -0.109127 2.242644 2.956852
H -0.488225 2.952457 2.218042
C -1.279751 1.347967 3.416493
C -0.930263 0.246674 4.407469
O 1.751089 0.784640 3.232403
C 1.133953 1.520776 2.506702
H 1.856132 1.830008 -2.141826
C 4.294469 -0.303326 -2.251599

H 4.048707 0.347326 -3.090694
H 5.426270 -1.694329 -3.426917
H -1.951710 2.051583 3.932137
S -2.421789 0.836955 2.077132
C -1.657504 -0.558427 1.207742
N -0.392997 -0.768744 1.492654
N -2.388474 -1.335254 0.433460
C 0.316342 -1.774201 0.876416
C 1.119625 -2.622780 1.677697
C 0.373475 -1.956770 -0.528225
C 1.985611 -3.545190 1.118231
H 1.063916 -2.510672 2.761537
C 1.256840 -2.860781 -1.100885
H -0.283229 -1.367075 -1.166529
C 2.067798 -3.637221 -0.273271
H 2.614339 -4.181490 1.740312
H 1.330762 -2.969490 -2.182561
N 3.060609 -4.516522 -0.870268
O 3.091756 -4.612899 -2.078957
O 3.828990 -5.096524 -0.129602
C -3.684418 -1.223605 0.048290
C -4.346134 -2.436300 -0.306131
C -4.433669 -0.021731 -0.106247
C -5.653444 -2.462474 -0.746250
H -3.776051 -3.361297 -0.210244
C -5.744371 -0.040746 -0.554297
H -3.969157 0.939569 0.100319
C -6.353633 -1.256630 -0.866473
H -6.146164 -3.399920 -1.001479
H -6.305560 0.885697 -0.672700
N -7.727797 -1.269269 -1.332491
O -8.311015 -0.208207 -1.433030
O -8.232749 -2.340705 -1.601490
H 0.218434 2.817478 3.838941
H -0.359782 0.670552 5.247897
H -0.327600 -0.535664 3.935213
H -1.854933 -0.198164 4.800559

Me_Michael_Acceptor/MA-R_pt

Frequencies, energies and thermodynamic properties:

Lowest Vibrational Mode (1/cm) = 10.4402
2nd Lowest Vibrational Mode (1/cm) = 19.1989
E(RM062X) (a.u.) = -2890.53315300
Thermal correction to Enthalpy (a.u.) = 0.702243
Thermal correction to Gibbs Free Energy (a.u.) = 0.581687
Total Entropy (cal/Kmol) = 253.731
Esp(RM062X) (a.u.) = -2893.13820633

Optimised cartesian coordinates (Angstrom):

C -5.415150 -1.251116 -2.256225
C -6.257495 -1.156030 -1.139106
C -5.840646 -0.506763 0.017911
C -4.560026 0.044992 0.028984
C -3.719098 -0.062500 -1.080373
H -7.249612 -1.607092 -1.171887
H -6.487736 -0.441135 0.893597
C -2.301754 0.964101 0.405969
C -0.061696 1.801534 -0.148479
C -0.058903 0.716584 -1.235208
C -1.427608 0.569330 -1.868726
H -1.476902 -0.359063 -2.452939
S -3.746490 0.825831 1.366426
N -2.456914 0.488011 -0.829372
N -1.143944 1.523801 0.825649

C	1.070688	0.881224	-2.276671
C	0.712824	1.749891	-3.485131
C	1.544421	-0.497285	-2.738844
H	1.910132	1.364660	-1.748317
H	0.299846	2.729439	-3.204543
H	1.613581	1.927183	-4.090536
H	-0.018105	1.241470	-4.133905
H	1.883557	-1.105863	-1.887612
H	0.729993	-1.041787	-3.244756
H	2.377372	-0.406504	-3.451580
H	0.144208	-0.215145	-0.690843
H	0.879732	1.662497	0.394587
C	-0.128668	3.226920	-0.652013
C	1.062097	3.950837	-0.778640
C	-1.333567	3.818342	-1.048034
C	1.055227	5.234242	-1.323394
H	2.002147	3.499147	-0.450230
C	-1.340744	5.103184	-1.590760
H	-2.281730	3.286079	-0.933890
C	-0.146360	5.809994	-1.736774
H	1.990758	5.787510	-1.420354
H	-2.285123	5.554959	-1.898574
H	-0.154438	6.814800	-2.162257
C	0.349496	2.353244	2.700594
H	0.010852	3.026886	3.500740
C	1.334154	1.353845	3.343578
C	2.176525	2.068390	4.398256
O	-1.841125	1.492100	2.981901
C	-0.945271	1.742657	2.214915
H	-1.683727	1.415694	-2.524959
C	-4.136513	-0.703811	-2.247695
H	-3.485457	-0.790275	-3.116974
H	-5.761810	-1.774320	-3.147941
H	0.770504	0.529679	3.797785
S	2.515879	0.591881	2.169216
C	1.598795	-0.829401	1.469213
N	2.251043	-1.690558	0.737939
C	3.554308	-1.608773	0.322992
C	4.368808	-2.761320	0.442517
C	4.112468	-0.470556	-0.310744
C	5.674677	-2.774135	-0.014618
H	3.937015	-3.646145	0.911555
C	5.417549	-0.476342	-0.776623
H	3.497299	0.419000	-0.448485
C	6.190383	-1.626602	-0.620208
H	6.301815	-3.658776	0.088116
H	5.844474	0.397635	-1.266985
N	7.563654	-1.634301	-1.110251
O	7.990928	-0.624110	-1.627955
O	8.211469	-2.650427	-0.975991
N	0.333515	-0.875207	1.843388
C	-0.598580	-1.727033	1.317060
C	-0.506964	-2.436673	0.083765
C	-1.828760	-1.823478	2.022398
C	-1.608545	-3.089117	-0.449958
H	0.434261	-2.441716	-0.459958
C	-2.919919	-2.498656	1.509017
H	-1.904603	-1.300740	2.977277
C	-2.813105	-3.104025	0.254253
H	-1.545891	-3.590592	-1.415559
H	-3.866225	-2.538319	2.048265
N	-3.986539	-3.722472	-0.341310
O	-4.995743	-3.801015	0.328680

O -3.903463 -4.128090 -1.483005
H 0.850401 2.974110 1.947752
H 1.520220 2.488733 5.173973
H 2.880114 1.372792 4.874401
H 2.752341 2.895072 3.954208

Me_Michael_Acceptor/MA-R

Frequencies, energies and thermodynamic properties:

Lowest Vibrational Mode (1/cm) = 8.8863
2nd Lowest Vibrational Mode (1/cm) = 12.1737
E(RM062X) (a.u.) = -2890.52259154
Thermal correction to Enthalpy (a.u.) = 0.702426
Thermal correction to Gibbs Free Energy (a.u.) = 0.576570
Total Entropy (cal/Kmol) = 264.886
Esp(RM062X) (a.u.) = -2893.13184347

Optimised cartesian coordinates (Angstrom):

C 6.577701 2.997548 -2.120738
C 5.637128 4.034692 -2.081844
C 4.325800 3.791619 -1.680447
C 3.978398 2.490242 -1.323161
C 4.915241 1.457122 -1.374527
H 5.933553 5.045420 -2.364692
H 3.589784 4.596438 -1.643659
C 3.070762 0.295438 -0.607290
C 3.071954 -2.073112 -0.034224
C 4.172948 -2.181164 -1.103936
C 5.129327 -1.009540 -0.989465
H 5.802759 -0.983442 -1.856100
S 2.412356 1.913523 -0.767721
N 4.360729 0.233736 -0.984660
N 2.405004 -0.760861 -0.147232
C 4.897147 -3.546816 -1.123641
C 6.124781 -3.628069 -0.211787
C 5.292866 -3.905843 -2.556542
H 4.165716 -4.296899 -0.780513
H 5.919913 -3.302792 0.817850
H 6.484826 -4.666383 -0.169752
H 6.949991 -3.014807 -0.607429
H 5.977043 -3.150149 -2.975125
H 5.811470 -4.875242 -2.585240
H 4.413246 -3.969073 -3.213488
H 3.646243 -2.073808 -2.066308
H 2.311697 -2.815835 -0.303659
C 3.535578 -2.348470 1.382369
C 4.155028 -1.365049 2.160619
C 3.393673 -3.639101 1.903885
C 4.650602 -1.676507 3.427121
H 4.252517 -0.341388 1.789913
C 3.890397 -3.951797 3.167994
H 2.889075 -4.405994 1.310736
C 4.526814 -2.971536 3.930452
H 5.132494 -0.900593 4.024312
H 3.774539 -4.963087 3.561473
H 4.914841 -3.213359 4.921308
C 0.387227 -1.674102 0.894832
H 0.854775 -2.640177 1.063559
C -1.011118 -1.480421 1.358770
C -1.439629 -2.521580 2.383310
O 0.523853 0.527359 0.076950
C 1.017582 -0.617965 0.298397
H 5.738503 -1.053777 -0.073858
C 6.233156 1.695099 -1.767629
H 6.973870 0.895801 -1.791669

H 7.601867 3.209698 -2.430149
H -1.151731 -0.468803 1.765303
S -2.261270 -1.558979 -0.073707
C -3.014726 0.074181 -0.063376
N -2.125248 1.089266 0.136627
H -1.115989 0.821391 0.110636
C -2.354481 2.439601 0.349226
C -1.212362 3.206430 0.678511
C -3.607579 3.077374 0.264078
C -1.311456 4.564775 0.911781
H -0.249540 2.697345 0.753104
C -3.705889 4.442618 0.505718
H -4.491064 2.503849 0.003095
C -2.565285 5.171801 0.823750
H -0.435523 5.158870 1.167756
H -4.668364 4.948543 0.441740
N -2.679852 6.608273 1.070098
O -3.779497 7.111607 1.002920
O -1.667279 7.221434 1.328923
N -4.268081 0.277362 -0.230678
C -5.212518 -0.705120 -0.461046
C -6.353668 -0.749943 0.364370
C -5.114002 -1.610732 -1.536412
C -7.351413 -1.686975 0.146834
H -6.433560 -0.036315 1.185036
C -6.112522 -2.546200 -1.767213
H -4.250411 -1.562686 -2.201421
C -7.216742 -2.575772 -0.918658
H -8.231202 -1.736697 0.786780
H -6.045569 -3.250123 -2.595531
N -8.270128 -3.564602 -1.156874
O -9.224258 -3.567538 -0.411125
O -8.131997 -4.328448 -2.086375
H -0.759040 -2.480915 3.247468
H -1.385985 -3.535307 1.958229
H -2.465999 -2.346299 2.731348

Me_Michael_Acceptor/MA-S_pt_rot

Frequencies, energies and thermodynamic properties:

Lowest Vibrational Mode (1/cm) = 6.8077
2nd Lowest Vibrational Mode (1/cm) = 14.0310
E(RM062X) (a.u.) = -2890.51922515
Thermal correction to Enthalpy (a.u.) = 0.702591
Thermal correction to Gibbs Free Energy (a.u.) = 0.579968
Total Entropy (cal/Kmol) = 258.082
Esp(RM062X) (a.u.) = -2893.13771810

Optimised cartesian coordinates (Angstrom):

C -3.377495 4.150335 -1.178237
C -2.415560 4.415104 -2.165073
C -1.549423 3.421388 -2.605833
C -1.657543 2.158974 -2.021874
C -2.600910 1.904249 -1.026811
H -2.344796 5.415682 -2.592327
H -0.801341 3.625430 -3.373214
C -1.541818 -0.131510 -1.121872
C -2.207963 -2.097245 0.178415
C -2.679531 -1.076964 1.222252
C -3.347130 0.105014 0.546067
H -3.467068 0.933458 1.257903
S -0.678984 0.744977 -2.346680
N -2.497450 0.595566 -0.540507
N -1.299365 -1.415904 -0.773192
C -3.534854 -1.688682 2.353917

C	-5.036721	-1.733427	2.061833
C	-3.272995	-0.943712	3.663640
H	-3.185061	-2.726418	2.489256
H	-5.476887	-0.723921	2.085130
H	-5.273032	-2.189751	1.089842
H	-5.545872	-2.322535	2.837950
H	-2.219074	-1.020584	3.969404
H	-3.527369	0.125182	3.573717
H	-3.888455	-1.357843	4.475121
H	-1.741103	-0.706420	1.664455
H	-1.598202	-2.812875	0.735258
C	-3.293825	-2.869707	-0.539473
C	-3.618389	-4.151359	-0.082325
C	-4.019051	-2.321845	-1.604430
C	-4.667741	-4.865216	-0.659033
H	-3.050780	-4.597418	0.739200
C	-5.067135	-3.036338	-2.182887
H	-3.775039	-1.333639	-2.003010
C	-5.397725	-4.305637	-1.706989
H	-4.911208	-5.863050	-0.290985
H	-5.626034	-2.599735	-3.011907
H	-6.218768	-4.862436	-2.161423
C	0.311389	-3.307717	-0.576377
H	1.063969	-3.777440	-1.221762
C	0.939779	-2.951529	0.789800
C	1.159735	-4.208259	1.623486
O	0.448044	-1.571313	-2.213333
C	-0.143967	-2.067759	-1.292153
H	-4.334228	-0.145364	0.126576
C	-3.486911	2.894138	-0.594303
H	-4.232105	2.704742	0.178488
H	-4.040326	4.949598	-0.844414
H	0.316185	-2.226397	1.334832
S	2.527361	-2.085031	0.560650
C	2.114213	-0.305363	0.713994
N	3.071059	0.526677	0.432355
N	0.880910	-0.019394	1.105043
C	4.391007	0.224008	0.238637
C	5.175009	-0.440139	1.214634
C	5.036960	0.663670	-0.941412
C	6.525867	-0.668979	1.013479
H	4.701599	-0.765902	2.141524
C	6.385595	0.435025	-1.152418
H	4.441423	1.185342	-1.691400
C	7.120226	-0.233176	-0.171489
H	7.129983	-1.177303	1.764007
H	6.879551	0.763993	-2.065839
N	8.541457	-0.475596	-0.386961
O	9.037947	-0.079756	-1.420294
O	9.158243	-1.062018	0.477092
C	0.413366	1.267133	1.149933
C	-0.618208	1.565189	2.086028
C	0.820695	2.326352	0.290696
C	-1.254798	2.792201	2.116088
H	-0.884454	0.796795	2.813314
C	0.191803	3.560484	0.321273
H	1.634809	2.151917	-0.407763
C	-0.855737	3.784066	1.215504
H	-2.048885	3.003091	2.832483
H	0.490549	4.358252	-0.358625
N	-1.572392	5.043414	1.178890
O	-2.583646	5.143916	1.845854
O	-1.144986	5.930929	0.469576

H -0.514245 -4.025366 -0.455590
H 1.603087 -3.954317 2.595110
H 1.830428 -4.913341 1.109561
H 0.203066 -4.724559 1.800916

Me_Michael_Acceptor/MA-S_pt

Frequencies, energies and thermodynamic properties:

Lowest Vibrational Mode (1/cm) = 14.3743

2nd Lowest Vibrational Mode (1/cm) = 19.5632

E(RM062X) (a.u.) = -2890.52375835

Thermal correction to Enthalpy (a.u.) = 0.701771

Thermal correction to Gibbs Free Energy (a.u.) = 0.581304

Total Entropy (cal/Kmol) = 253.542

Esp(RM062X) (a.u.) = -2893.13094811

Optimised cartesian coordinates (Angstrom):

C 5.270854 -1.163463 -2.469429
C 5.574754 -2.044310 -1.418401
C 5.059158 -1.840712 -0.143969
C 4.214193 -0.746139 0.047141
C 3.913424 0.126953 -0.998739
H 6.213429 -2.908558 -1.604811
H 5.279636 -2.530948 0.671801
C 2.576712 0.987232 0.655561
C 1.045678 2.893333 0.359660
C 2.152317 3.385532 -0.592573
C 2.633668 2.238433 -1.463729
H 3.526051 2.531648 -2.032465
S 3.360680 -0.303034 1.507289
N 3.018015 1.122506 -0.597445
N 1.583519 1.758881 1.159093
C 1.758007 4.633236 -1.417725
C 1.130504 4.318508 -2.778504
C 2.979010 5.534738 -1.603969
H 1.017824 5.189673 -0.820089
H 0.287771 3.615893 -2.713019
H 0.757790 5.246718 -3.235238
H 1.877852 3.894993 -3.467814
H 3.378188 5.876446 -0.637911
H 3.782976 4.999403 -2.134340
H 2.719700 6.421647 -2.200033
H 2.993002 3.672538 0.060077
H 0.863900 3.707761 1.072711
C -0.246510 2.548482 -0.344900
C -1.252136 3.515733 -0.439619
C -0.419000 1.316459 -0.979566
C -2.393678 3.271685 -1.201302
H -1.134359 4.470580 0.079259
C -1.562878 1.067457 -1.736580
H 0.337168 0.534754 -0.876040
C -2.543885 2.051845 -1.862891
H -3.169778 4.035133 -1.276882
H -1.691105 0.099433 -2.225620
H -3.437632 1.857227 -2.458234
C -0.201388 2.120622 2.911774
H -0.658872 2.774086 2.164172
C -1.246323 1.112656 3.432319
C -2.033143 1.712248 4.594630
O 1.690776 0.707778 3.166279
C 1.070094 1.452347 2.452676
H 1.864282 1.893086 -2.169306
C 4.442494 -0.063838 -2.277788
H 4.196068 0.607813 -3.100235
H 5.684568 -1.349361 -3.461251

H -0.740222 0.190282 3.742623
S -2.469501 0.644396 2.152662
C -1.677112 -0.724394 1.257388
N -0.432397 -0.943265 1.614871
N -2.367809 -1.470699 0.422508
C 0.351982 -1.893810 1.015191
C 1.202723 -2.675063 1.837607
C 0.448748 -2.079472 -0.388456
C 2.148029 -3.529974 1.300672
H 1.117483 -2.555244 2.918525
C 1.409509 -2.915466 -0.936520
H -0.237075 -1.541269 -1.040699
C 2.263095 -3.623696 -0.089146
H 2.812230 -4.112387 1.938593
H 1.513404 -3.023378 -2.015816
N 3.323053 -4.434415 -0.665161
O 3.370726 -4.546987 -1.872184
O 4.126069 -4.947101 0.089355
C -3.654852 -1.349049 0.004899
C -4.298443 -2.548636 -0.417407
C -4.405650 -0.145456 -0.117574
C -5.595008 -2.562961 -0.889708
H -3.725110 -3.473841 -0.346290
C -5.705682 -0.152067 -0.597049
H -3.951693 0.809038 0.137780
C -6.299057 -1.356752 -0.974679
H -6.075814 -3.491144 -1.196238
H -6.269510 0.775657 -0.690104
N -7.662595 -1.356606 -1.473725
O -8.248650 -0.295030 -1.544565
O -8.154562 -2.418374 -1.798172
H 0.121163 2.750690 3.757599
H -1.346355 1.962572 5.416546
H -2.552468 2.634887 4.291958
H -2.781986 1.001836 4.969097

Me_Michael_Acceptor/MA-S

Frequencies, energies and thermodynamic properties:

Lowest Vibrational Mode (1/cm) = 14.8782

2nd Lowest Vibrational Mode (1/cm) = 22.6976

E(RM062X) (a.u.) = -2890.52409555

Thermal correction to Enthalpy (a.u.) = 0.702364

Thermal correction to Gibbs Free Energy (a.u.) = 0.581886

Total Entropy (cal/Kmol) = 253.566

Esp(RM062X) (a.u.) = -2893.12972629

Optimised cartesian coordinates (Angstrom):

C -4.365833 3.834896 1.759681
C -3.188680 4.591451 1.709615
C -1.948884 3.972944 1.564291
C -1.918211 2.584783 1.447421
C -3.094561 1.834051 1.504073
H -3.240308 5.678530 1.779567
H -1.029550 4.559331 1.520053
C -1.553943 0.162274 1.112431
C -2.120790 -2.179324 0.795822
C -3.217043 -1.884024 1.838034
C -3.880875 -0.550591 1.534914
H -4.508226 -0.231937 2.379171
S -0.506795 1.558773 1.224604
N -2.843452 0.465807 1.354582
N -1.153344 -1.062188 0.789862
C -4.227767 -3.038882 2.026399
C -5.449239 -2.978697 1.105236

C	-4.681594	-3.099580	3.485699
H	-3.681898	-3.971475	1.806166
H	-5.184099	-2.880790	0.043113
H	-6.040666	-3.898925	1.218789
H	-6.105794	-2.136047	1.374471
H	-3.832114	-3.264597	4.164292
H	-5.175854	-2.160462	3.782281
H	-5.402355	-3.916625	3.635628
H	-2.676422	-1.764116	2.791317
H	-1.557210	-3.046802	1.160501
C	-2.660146	-2.510213	-0.580709
C	-2.722447	-3.848305	-0.984394
C	-3.177820	-1.523824	-1.427212
C	-3.305787	-4.196835	-2.202167
H	-2.309279	-4.624531	-0.334988
C	-3.756631	-1.868812	-2.648645
H	-3.125928	-0.471746	-1.139137
C	-3.827504	-3.207472	-3.036187
H	-3.348623	-5.245036	-2.502665
H	-4.154457	-1.087668	-3.299376
H	-4.282825	-3.477931	-3.990289
C	0.628822	-2.494376	-0.083176
H	-0.107439	-3.190927	-0.479353
C	2.061421	-2.841815	-0.154905
C	2.314034	-4.338940	-0.266507
O	1.046990	-0.394890	0.905517
C	0.285513	-1.309284	0.531746
H	-4.511791	-0.589367	0.635085
C	-4.337915	2.445423	1.661389
H	-5.257335	1.860475	1.691499
H	-5.326254	4.340105	1.869651
H	2.597020	-2.421690	0.706904
S	3.067556	-2.123883	-1.625684
C	2.603618	-0.386446	-1.676274
N	3.391045	0.580070	-1.399596
N	1.344487	-0.183829	-2.177186
H	0.842009	-1.043392	-2.380322
C	4.536362	0.422509	-0.639902
C	4.448920	-0.087555	0.672239
C	5.780596	0.849514	-1.135864
C	5.584859	-0.190253	1.460031
H	3.461682	-0.366662	1.049910
C	6.922397	0.740649	-0.354778
H	5.835758	1.258398	-2.145238
C	6.809626	0.219713	0.932815
H	5.536076	-0.579422	2.476120
H	7.895818	1.055295	-0.728306
N	8.011617	0.108989	1.760879
O	9.065311	0.470582	1.285155
O	7.891359	-0.339575	2.879621
C	0.549270	0.948877	-2.082001
C	1.030310	2.245846	-1.826959
C	-0.835952	0.747880	-2.248217
C	0.131871	3.297220	-1.681034
H	2.097276	2.418360	-1.726059
C	-1.731003	1.790742	-2.094238
H	-1.196027	-0.256318	-2.479184
C	-1.233938	3.059264	-1.794644
H	0.488212	4.303469	-1.463351
H	-2.805756	1.635992	-2.192875
N	-2.178043	4.150029	-1.553675
O	-3.360445	3.888972	-1.571090
O	-1.728704	5.254079	-1.338410

H 1.909099 -4.838690 0.625909

H 1.809772 -4.753859 -1.152048

H 3.386741 -4.571534 -0.334495

Me_Michael_Acceptor/Me_ester

Frequencies, energies and thermodynamic properties:

Lowest Vibrational Mode (1/cm) = 33.1391

2nd Lowest Vibrational Mode (1/cm) = 48.3949

E(RM062X) (a.u.) = -1032.52305687

Thermal correction to Enthalpy (a.u.) = 0.153117

Thermal correction to Gibbs Free Energy (a.u.) = 0.092635

Total Entropy (cal/Kmol) = 127.296

Esp(RM062X) (a.u.) = -1033.73601298

Optimised cartesian coordinates (Angstrom):

C 3.451144 0.252262 -0.305519

H 3.550568 0.741158 -1.276484

C 4.508333 -0.100783 0.437082

H 4.311778 -0.587517 1.399266

O 1.157257 0.426042 -0.727929

C -0.153968 0.219346 -0.424956

C -0.987937 1.310966 -0.197347

C -0.682790 -1.068107 -0.351227

C -2.340485 1.121444 0.070130

C -2.029442 -1.264306 -0.070885

C -2.858777 -0.166763 0.143136

C 5.929578 0.115958 0.055906

O 1.767910 -0.531330 1.215664

C 2.090639 -0.016150 0.185567

F -0.500241 2.538600 -0.247101

F 0.103196 -2.110724 -0.559152

F -4.140739 -0.349972 0.407075

F -3.130201 2.163788 0.271313

F -2.525724 -2.488774 -0.005615

H 6.023148 0.611255 -0.918925

H 6.436372 0.725175 0.820483

H 6.462415 -0.847228 0.023762

Me_Michael_Acceptor/pNO2_thiourea

Frequencies, energies and thermodynamic properties:

Lowest Vibrational Mode (1/cm) = 18.6815

2nd Lowest Vibrational Mode (1/cm) = 21.7203

E(RM062X) (a.u.) = -1418.04990065

Thermal correction to Enthalpy (a.u.) = 0.251465

Thermal correction to Gibbs Free Energy (a.u.) = 0.181252

Total Entropy (cal/Kmol) = 147.775

Esp(RM062X) (a.u.) = -1419.34300615

Optimised cartesian coordinates (Angstrom):

C -0.010122 1.964661 0.081432

S -0.923571 3.348093 -0.105177

N 1.353766 2.059251 0.123383

H 1.684195 3.016258 0.223140

N -0.500866 0.708768 0.270880

H 0.140222 0.042540 0.699144

C -1.817630 0.241178 0.178794

C -2.757629 0.760615 -0.724929

C -2.164941 -0.844466 0.999652

C -4.030956 0.210625 -0.786694

H -2.492443 1.585810 -1.380248

C -3.433891 -1.399768 0.937124

H -1.428459 -1.246755 1.697331

C -4.353857 -0.856707 0.045689

H -4.771858 0.598601 -1.484094

H -3.715341 -2.237635 1.572957

C 2.349450 1.077612 0.045142
C 3.582038 1.360050 0.659970
C 2.187958 -0.124495 -0.666159
C 4.629343 0.455633 0.587476
H 3.706231 2.300119 1.199910
C 3.231832 -1.038536 -0.730380
H 1.261120 -0.339243 -1.196526
C 4.434327 -0.739726 -0.099629
H 5.586923 0.662590 1.063028
H 3.123355 -1.971243 -1.281951
N 5.535029 -1.708228 -0.170714
O 5.333037 -2.748914 -0.753086
O 6.581297 -1.410962 0.358233
N -5.701693 -1.433283 -0.024638
O -5.948306 -2.378536 0.689216
O -6.490527 -0.932354 -0.792758

Me_Michael_Acceptor/product

Frequencies, energies and thermodynamic properties:

Lowest Vibrational Mode (1/cm) = 17.2658

2nd Lowest Vibrational Mode (1/cm) = 22.7400

E(RM062X) (a.u.) = -1647.84535300

Thermal correction to Enthalpy (a.u.) = 0.327796

Thermal correction to Gibbs Free Energy (a.u.) = 0.246591

Total Entropy (cal/Kmol) = 170.911

Esp(RM062X) (a.u.) = -1649.39867118

Optimised cartesian coordinates (Angstrom):

C 0.173755 3.883215 0.248719
C 0.085389 1.140167 -0.238252
C -1.897136 2.662081 -0.523514
C -0.993586 3.852644 -0.723723
H -0.628686 3.835811 -1.762868
H -1.626452 4.741025 -0.598939
N -1.287107 1.421115 -0.267592
C -2.170658 0.291130 -0.142496
C -2.471276 -0.200732 1.126487
C -2.712219 -0.288754 -1.289117
C -3.326179 -1.289800 1.258045
H -2.034459 0.269932 2.008213
C -3.570836 -1.375072 -1.170334
H -2.458862 0.113037 -2.270785
C -3.857994 -1.853829 0.103879
H -3.581711 -1.697817 2.234512
H -4.011176 -1.850539 -2.045103
N -4.764881 -3.009181 0.236059
O -5.229521 -3.477027 -0.776555
O -4.990466 -3.421615 1.349294
O -3.098192 2.736596 -0.584606
S 1.265038 2.457104 -0.096666
N 0.455406 -0.072846 -0.312706
C 1.787479 -0.466071 -0.209144
C 2.501155 -0.816639 -1.367459
C 2.406086 -0.574864 1.049095
C 3.815072 -1.254859 -1.275762
H 2.008234 -0.741235 -2.337169
C 3.717686 -1.016259 1.148083
H 1.842917 -0.313497 1.945892
C 4.404114 -1.347490 -0.017645
H 4.383675 -1.526303 -2.164099
H 4.212359 -1.106487 2.114018
N 5.792310 -1.811657 0.084369
O 6.280590 -1.895792 1.188419
O 6.374056 -2.083998 -0.941089

H 0.813674 4.742600 0.005577

C -0.248402 3.947330 1.708810

H 0.628689 3.989734 2.367787

H -0.850008 3.070110 1.993239

H -0.855338 4.849449 1.877713

Me_Michael_Acceptor/TS-acyl

Frequencies, energies and thermodynamic properties:

Lowest Vibrational Mode (1/cm) = -159.1750

2nd Lowest Vibrational Mode (1/cm) = 11.2941

E(RM062X) (a.u.) = -2275.19404571

Thermal correction to Enthalpy (a.u.) = 0.525426

Thermal correction to Gibbs Free Energy (a.u.) = 0.421019

Total Entropy (cal/Kmol) = 219.744

Esp(RM062X) (a.u.) = -2277.46016117

Optimised cartesian coordinates (Angstrom):

C -6.549765 -1.764538 -0.222148

C -6.203621 -3.037891 0.237824

C -4.864833 -3.380013 0.439112

C -3.888678 -2.426129 0.171478

C -4.236959 -1.153916 -0.299577

C -5.573026 -0.805763 -0.495620

H -7.600204 -1.509598 -0.369712

H -6.982908 -3.772259 0.444897

H -4.589291 -4.372246 0.800411

H -5.848373 0.188749 -0.847341

C -1.922000 -0.915738 -0.192626

C -0.730971 1.102054 -0.607976

C -1.858240 1.419013 -1.615633

C -3.204407 1.013553 -1.028497

H -3.981268 1.038681 -1.806196

S -2.144200 -2.601291 0.338116

N -3.122729 -0.349514 -0.515978

N -0.789318 -0.308370 -0.233431

C -1.849633 2.866368 -2.157168

C -2.673136 3.861442 -1.334458

C -2.321387 2.886596 -3.611998

H -0.798481 3.200165 -2.141639

H -2.431359 3.836183 -0.262536

H -2.488857 4.883936 -1.695955

H -3.751811 3.667233 -1.446379

H -1.675537 2.268628 -4.252818

H -3.350487 2.500820 -3.694752

H -2.317798 3.911860 -4.010887

H -1.664238 0.748751 -2.469139

H 0.227522 1.239023 -1.127468

C -0.724911 2.010806 0.610450

C 0.102248 3.139549 0.619493

C -1.544026 1.765985 1.719113

C 0.095874 4.019238 1.701971

H 0.762838 3.327119 -0.229932

C -1.550995 2.643654 2.803403

H -2.175825 0.874813 1.751576

C -0.734228 3.775397 2.796490

H 0.747570 4.894821 1.693112

H -2.194024 2.437962 3.661089

H -0.737909 4.459753 3.646662

C 1.122266 -0.507889 1.498387

H 1.386414 0.549155 1.399555

C 1.051282 -1.101271 2.691410

H 0.768955 -2.160175 2.710675

C 1.308393 -0.419694 3.995058

O 0.525076 -2.448506 0.228605

C 0.780318 -1.262600 0.246820

H -3.513007 1.687158 -0.213143

O 1.514629 -0.716230 -0.907157

C 2.849126 -0.659624 -0.752881

C 3.662344 -1.793171 -0.861955

C 3.470869 0.564829 -0.486910

C 5.044766 -1.697520 -0.730130

C 4.850012 0.671372 -0.358514

C 5.639888 -0.468403 -0.468406

F 2.724956 1.656875 -0.361609

F 5.412917 1.845554 -0.113747

F 5.799395 -2.779911 -0.851007

F 3.129484 -2.973299 -1.127465

F 6.954755 -0.380101 -0.340830

H 1.575411 0.636799 3.852698

H 2.119158 -0.919967 4.547492

H 0.414425 -0.469857 4.637002

Me_Michael_Acceptor/TS-cycl-R_rot

Frequencies, energies and thermodynamic properties:

Lowest Vibrational Mode (1/cm) = -131.1259

2nd Lowest Vibrational Mode (1/cm) = 12.8276

E(RM062X) (a.u.) = -2890.50993623

Thermal correction to Enthalpy (a.u.) = 0.700989

Thermal correction to Gibbs Free Energy (a.u.) = 0.581795

Total Entropy (cal/Kmol) = 250.865

Esp(RM062X) (a.u.) = -2893.11486570

Optimised cartesian coordinates (Angstrom):

C -0.176948 -1.345251 2.950155

C 1.277308 -1.129128 3.343037

H 1.573527 -1.996932 3.951270

H -0.276909 -2.323497 2.463760

H -0.783376 -1.381893 3.868238

N 0.664898 0.605208 1.146849

C 0.240409 1.786509 0.528409

C 0.345779 2.000252 -0.862028

C -0.344081 2.795949 1.320864

C -0.148343 3.158321 -1.444264

H 0.826696 1.247110 -1.481781

C -0.845636 3.957008 0.746164

H -0.390561 2.659202 2.398144

C -0.748685 4.118727 -0.632505

H -0.076064 3.322942 -2.518572

H -1.295036 4.740329 1.354688

N -1.276160 5.340281 -1.245645

O -1.793212 6.162314 -0.521726

O -1.171700 5.464498 -2.446025

C -0.852248 -0.236307 2.138513

O -1.436426 0.668067 2.713978

C 1.534378 0.138803 4.146299

S 2.377022 -1.300482 1.893405

C 1.885651 0.125098 0.901592

N 2.709594 0.565582 0.002264

C 4.053513 0.302920 -0.063457

C 4.933069 0.530678 1.023927

C 4.611505 -0.123991 -1.290480

C 6.295644 0.322024 0.895539

H 4.523553 0.886491 1.969890

C 5.972940 -0.340584 -1.425486

H 3.939551 -0.278849 -2.135396

C 6.804310 -0.118643 -0.327667

H 6.974285 0.500288 1.728729

H 6.401822 -0.677056 -2.368417

N 8.239099 -0.341797 -0.464051
O 8.658177 -0.722223 -1.536604
O 8.942890 -0.137754 0.502082
N -1.559325 -0.777144 0.930115
C -2.858045 -0.537799 0.789717
C -0.919941 -1.758113 0.036382
S -3.723074 0.789343 1.533498
N -3.648295 -1.295292 0.011347
C -1.861767 -2.972503 -0.084447
H -0.006453 -2.085404 0.546503
C -0.494092 -1.181502 -1.300261
C -5.187852 0.293762 0.696134
C -4.967936 -0.842303 -0.086320
C -3.195415 -2.518227 -0.659055
C -1.253664 -4.175808 -0.841991
H -2.040232 -3.295416 0.954820
C 0.767713 -1.532533 -1.792657
C -1.334254 -0.394217 -2.098353
C -6.446575 0.888907 0.755171
C -5.992320 -1.410455 -0.843962
H -3.968526 -3.283267 -0.503749
H -3.129193 -2.309557 -1.737079
C -1.489893 -4.172410 -2.354511
C -1.766980 -5.485122 -0.240845
H -0.165190 -4.130087 -0.668711
C 1.174643 -1.129189 -3.064384
H 1.443417 -2.123036 -1.168141
C -0.928834 0.009770 -3.370041
H -2.310582 -0.065250 -1.737700
C -7.473268 0.326850 0.000263
H -6.620550 1.773324 1.369825
C -7.246426 -0.807742 -0.789363
H -5.819024 -2.292332 -1.460513
H -1.204257 -3.224821 -2.832672
H -0.898491 -4.974073 -2.820216
H -2.547590 -4.371240 -2.589565
H -1.504753 -5.569847 0.823912
H -2.863395 -5.552753 -0.327619
H -1.337688 -6.350011 -0.767327
C 0.323870 -0.360792 -3.859607
H 2.160523 -1.415743 -3.434604
H -1.593769 0.626761 -3.976912
H -8.465153 0.779550 0.023745
H -8.064593 -1.229751 -1.374248
H 0.639707 -0.044122 -4.855020
H 0.895934 0.141519 5.042960
H 1.303543 1.037821 3.560295
H 2.583975 0.183007 4.468359

Me_Michael_Acceptor/TS-cycl-R

Frequencies, energies and thermodynamic properties:

Lowest Vibrational Mode (1/cm) = -161.0763
2nd Lowest Vibrational Mode (1/cm) = 7.7267
E(RM062X) (a.u.) = -2890.51587789
Thermal correction to Enthalpy (a.u.) = 0.701037
Thermal correction to Gibbs Free Energy (a.u.) = 0.580622
Total Entropy (cal/Kmol) = 253.435
Esp(RM062X) (a.u.) = -2893.12312690

Optimised cartesian coordinates (Angstrom):

C 1.658466 -2.813996 -1.546840
C 2.393819 -0.250062 -0.391208
C -0.245518 -1.228057 -1.285921
C 0.351950 -2.545018 -0.827198

H	0.491275	-2.608177	0.258503
H	-0.384539	-3.313622	-1.120525
N	1.076044	-0.025869	-0.478956
C	0.573400	1.221118	-0.108108
C	-0.284090	1.905463	-0.989684
C	0.816403	1.775060	1.166959
C	-0.932919	3.070655	-0.598375
H	-0.434671	1.497729	-1.989493
C	0.189544	2.946003	1.560849
H	1.500151	1.268608	1.846762
C	-0.696550	3.568034	0.680227
H	-1.610722	3.595486	-1.270864
H	0.365520	3.376804	2.545722
N	-1.401190	4.773484	1.119648
O	-1.268064	5.117959	2.273850
O	-2.089329	5.356308	0.311817
O	-0.325501	-0.964492	-2.480587
S	3.038596	-1.882677	-0.789978
N	3.246885	0.629393	0.033959
C	4.614254	0.548507	-0.050324
C	5.391246	0.724586	1.117244
C	5.292171	0.390397	-1.283913
C	6.775718	0.722692	1.066825
H	4.874411	0.863088	2.067642
C	6.675511	0.391038	-1.343551
H	4.708293	0.276056	-2.197728
C	7.406273	0.552439	-0.165358
H	7.374981	0.851271	1.967200
H	7.199048	0.273091	-2.291476
N	8.864119	0.551170	-0.225311
O	9.390466	0.391507	-1.305796
O	9.477836	0.708365	0.808576
H	1.562034	-2.486668	-2.590633
C	2.050906	-4.284646	-1.495938
N	-1.431063	-0.825343	-0.471966
C	-2.502640	-0.328038	-1.087484
C	-1.439083	-1.007428	0.995313
S	-2.674335	-0.137443	-2.822744
N	-3.605124	0.057830	-0.426224
C	-2.309293	0.098775	1.626963
H	-0.403838	-0.830393	1.317876
C	-1.834409	-2.415979	1.395807
C	-4.326238	0.417794	-2.595342
C	-4.666714	0.463207	-1.243151
C	-3.704136	0.058210	1.032771
C	-2.325649	0.109598	3.171872
H	-1.847849	1.045464	1.302327
C	-1.154730	-3.035698	2.450294
C	-2.885469	-3.095479	0.771906
C	-5.240202	0.791538	-3.578911
C	-5.936840	0.867550	-0.829833
H	-4.270011	0.952308	1.325525
H	-4.270400	-0.831988	1.346534
C	-3.378979	-0.802126	3.810391
C	-2.526916	1.539821	3.678185
H	-1.327440	-0.228468	3.497929
C	-1.537962	-4.299542	2.895028
H	-0.316068	-2.520976	2.926224
C	-3.269022	-4.362105	1.215137
H	-3.412726	-2.647595	-0.074401
C	-6.507178	1.202561	-3.172967
H	-4.969633	0.759957	-4.635394
C	-6.849072	1.236836	-1.814841

H -6.213463 0.890504 0.223932
H -3.359816 -1.830502 3.424807
H -3.214595 -0.848641 4.896850
H -4.391527 -0.396974 3.654757
H -1.750218 2.222054 3.304324
H -3.503986 1.934456 3.355104
H -2.507634 1.567898 4.777455
C -2.601395 -4.963669 2.281588
H -1.000377 -4.770468 3.719683
H -4.091329 -4.881544 0.720386
H -7.241229 1.498700 -3.923137
H -7.848829 1.556685 -1.518496
H -2.901668 -5.954392 2.626707
H 2.108010 -4.640567 -0.456532
H 3.024614 -4.456492 -1.976933
H 1.297151 -4.882861 -2.028477

Me_Michael_Acceptor/TS-cycl-S_rot

Frequencies, energies and thermodynamic properties:

Lowest Vibrational Mode (1/cm) = -178.4552
2nd Lowest Vibrational Mode (1/cm) = 8.9078
E(RM062X) (a.u.) = -2890.51286395
Thermal correction to Enthalpy (a.u.) = 0.700937
Thermal correction to Gibbs Free Energy (a.u.) = 0.580229
Total Entropy (cal/Kmol) = 254.052
Esp(RM062X) (a.u.) = -2893.11865574

Optimised cartesian coordinates (Angstrom):

C -1.612026 1.791975 -2.821236
C -2.152635 -0.011340 -0.726095
C 0.489245 0.685808 -1.874690
C -0.227610 1.983810 -2.213886
H -0.319814 2.662250 -1.361522
H 0.434331 2.467284 -2.950692
N -0.877143 -0.319378 -1.002238
C -0.341845 -1.513038 -0.494625
C 0.264259 -2.422921 -1.378057
C -0.336564 -1.799298 0.885407
C 0.895042 -3.566552 -0.898616
H 0.238024 -2.215813 -2.445654
C 0.286479 -2.937503 1.374480
H -0.834713 -1.114530 1.570632
C 0.904645 -3.801769 0.471240
H 1.370739 -4.275241 -1.575089
H 0.302961 -3.162333 2.440105
N 1.572401 -5.003896 0.983111
O 1.573128 -5.184403 2.180291
O 2.090693 -5.749161 0.182705
O 0.952313 -0.005603 -2.779409
S -2.826635 1.465169 -1.495230
N -2.906325 -0.683456 0.087267
C -4.271675 -0.582009 0.191371
C -4.853663 -0.382152 1.463642
C -5.135644 -0.763472 -0.915989
C -6.228865 -0.341745 1.626941
H -4.192529 -0.258751 2.322261
C -6.511136 -0.729571 -0.760568
H -4.703373 -0.942160 -1.901082
C -7.046300 -0.513343 0.510234
H -6.678110 -0.179976 2.605890
H -7.178504 -0.873259 -1.609346
N -8.495669 -0.474545 0.675321
O -9.185944 -0.627417 -0.309537
O -8.938252 -0.290005 1.788953

H -1.940600 2.777033 -3.184114
C -1.709697 0.787542 -3.961365
N 1.406121 0.825905 -0.681373
C 2.575762 0.196078 -0.679242
C 1.000512 1.628389 0.488188
S 3.236600 -0.716353 -2.025283
N 3.417095 0.230642 0.369753
C 1.578413 0.987627 1.763989
H -0.093203 1.533212 0.545146
C 1.354814 3.096651 0.337934
C 4.714538 -0.985076 -1.111684
C 4.644399 -0.405951 0.156002
C 3.086051 0.868948 1.643743
C 1.128383 1.663503 3.078784
H 1.181647 -0.037789 1.771378
C 0.441954 4.061694 0.779547
C 2.584872 3.512180 -0.181681
C 5.852010 -1.679335 -1.519575
C 5.712782 -0.487746 1.049710
H 3.481970 0.232534 2.446830
H 3.592692 1.844808 1.690895
C 2.042777 2.792412 3.562438
C 0.984747 0.607212 4.175516
H 0.128932 2.089104 2.889563
C 0.765097 5.416386 0.735594
H -0.534341 3.743845 1.155101
C 2.908485 4.869243 -0.228021
H 3.303468 2.782209 -0.562903
C 6.920046 -1.770344 -0.630214
H 5.903666 -2.136013 -2.509099
C 6.849457 -1.178844 0.637395
H 5.667115 -0.024671 2.035145
H 2.246077 3.544881 2.787352
H 1.575174 3.306992 4.414528
H 3.007551 2.393613 3.914047
H 0.243167 -0.158366 3.902946
H 1.945822 0.099422 4.356638
H 0.667205 1.067394 5.122577
C 2.004027 5.823127 0.237744
H 0.044253 6.157404 1.085070
H 3.872131 5.180968 -0.634312
H 7.821598 -2.307355 -0.927351
H 7.698538 -1.257179 1.317542
H 2.258747 6.883557 0.200552
H -0.971849 1.032318 -4.739930
H -2.714262 0.817969 -4.405286
H -1.501641 -0.232073 -3.612639

Me_Michael_Acceptor/TS-cycl-S

Frequencies, energies and thermodynamic properties:

Lowest Vibrational Mode (1/cm) = -139.0151

2nd Lowest Vibrational Mode (1/cm) = 10.9724

E(RM062X) (a.u.) = -2890.51047329

Thermal correction to Enthalpy (a.u.) = 0.700886

Thermal correction to Gibbs Free Energy (a.u.) = 0.580752

Total Entropy (cal/Kmol) = 252.843

Esp(RM062X) (a.u.) = -2893.11617929

Optimised cartesian coordinates (Angstrom):

C -0.183796 -1.471817 2.879129
C 1.262504 -1.245735 3.277043
H 1.375928 -0.231916 3.687399
H -0.273685 -2.434336 2.356535
H -0.789735 -1.545120 3.795204

N	0.640069	0.600422	1.247837
C	0.198438	1.821039	0.721916
C	0.267739	2.126930	-0.652439
C	-0.372479	2.766344	1.596621
C	-0.252032	3.316452	-1.142489
H	0.743815	1.423674	-1.331786
C	-0.897856	3.958908	1.115328
H	-0.387394	2.552920	2.662401
C	-0.838996	4.212772	-0.251575
H	-0.208462	3.554735	-2.204458
H	-1.338218	4.695041	1.786186
N	-1.394547	5.467391	-0.766920
O	-1.901888	6.231429	0.023998
O	-1.321357	5.674241	-1.958016
C	-0.860832	-0.327129	2.115857
O	-1.482339	0.522742	2.737613
C	1.732214	-2.278722	4.292197
S	2.361167	-1.348672	1.820355
C	1.858303	0.144994	0.942043
N	2.662828	0.665834	0.069496
C	4.007804	0.425548	-0.045022
C	4.904737	0.516776	1.048186
C	4.548694	0.171792	-1.326853
C	6.266193	0.336806	0.871307
H	4.512450	0.743568	2.039632
C	5.908678	-0.014396	-1.511243
H	3.864572	0.130374	-2.175091
C	6.756933	0.064805	-0.406809
H	6.957705	0.410140	1.709750
H	6.323694	-0.216727	-2.497715
N	8.190400	-0.128106	-0.594101
O	8.594590	-0.348928	-1.715942
O	8.907810	-0.060203	0.381115
N	-1.542470	-0.809857	0.866252
C	-2.840599	-0.575208	0.715701
C	-0.878272	-1.733252	-0.068598
S	-3.734428	0.698106	1.518437
N	-3.608109	-1.292601	-0.121876
C	-1.805722	-2.947508	-0.273313
H	0.028637	-2.082127	0.439302
C	-0.436547	-1.077197	-1.362938
C	-5.175490	0.243213	0.618402
C	-4.928755	-0.843560	-0.223664
C	-3.132312	-2.473905	-0.848064
C	-1.172498	-4.102460	-1.083646
H	-2.000522	-3.326581	0.743906
C	0.835912	-1.387778	-1.854553
C	-1.271961	-0.252183	-2.127912
C	-6.440166	0.825003	0.682754
C	-5.930583	-1.372937	-1.037487
H	-3.900863	-3.252845	-0.748590
H	-3.047615	-2.207028	-1.911898
C	-1.379550	-4.016289	-2.598083
C	-1.685379	-5.447550	-0.566840
H	-0.087984	-4.058318	-0.886759
C	1.257310	-0.910101	-3.095601
H	1.507809	-2.007149	-1.254390
C	-0.850860	0.228609	-3.367179
H	-2.256386	0.048287	-1.764700
C	-7.444724	0.301494	-0.127706
H	-6.635519	1.670126	1.344459
C	-7.190510	-0.782935	-0.977131
H	-5.736230	-2.215554	-1.700976

H -1.094134 -3.040673 -3.016617
H -0.771498 -4.785309 -3.096279
H -2.430566 -4.210959 -2.864486
H -1.445409 -5.588784 0.497140
H -2.778890 -5.520798 -0.681730
H -1.235755 -6.277941 -1.130582
C 0.412128 -0.103782 -3.858211
H 2.250359 -1.169230 -3.466823
H -1.512335 0.873875 -3.947866
H -8.440603 0.745172 -0.100777
H -7.991544 -1.175053 -1.605005
H 0.739697 0.271299 -4.829237
H 2.779404 -2.108543 4.577397
H 1.642210 -3.298728 3.888412
H 1.111515 -2.214516 5.197632

Me_Michael_Acceptor/TS-MA-R
Frequencies, energies and thermodynamic properties:
Lowest Vibrational Mode (1/cm) = -195.9971
2nd Lowest Vibrational Mode (1/cm) = 5.0630
E(RM062X) (a.u.) = -2890.51804246
Thermal correction to Enthalpy (a.u.) = 0.700697
Thermal correction to Gibbs Free Energy (a.u.) = 0.577658
Total Entropy (cal/Kmol) = 258.957
Esp(RM062X) (a.u.) = -2893.12342069
Optimised cartesian coordinates (Angstrom):
C 4.582473 -3.920101 -1.676015
C 3.404609 -4.502772 -2.162725
C 2.210232 -3.789019 -2.179250
C 2.218014 -2.489335 -1.673487
C 3.388206 -1.921704 -1.167093
H 3.422563 -5.526953 -2.536779
H 1.291065 -4.235950 -2.561234
C 1.891262 -0.212762 -0.768149
C 2.426715 1.873307 0.398531
C 3.584035 1.062005 1.006576
C 4.212315 0.167114 -0.043008
H 4.931377 -0.519780 0.421299
S 0.863664 -1.378602 -1.562277
N 3.159560 -0.636766 -0.661704
N 1.477736 0.965605 -0.281144
C 4.620697 1.918978 1.766927
C 5.768782 2.441213 0.898716
C 5.182139 1.122132 2.945117
H 4.077782 2.787163 2.175763
H 6.434580 1.619378 0.590977
H 5.423058 2.959648 -0.006633
H 6.376912 3.150159 1.479209
H 4.389413 0.833782 3.650987
H 5.677484 0.202352 2.594113
H 5.929772 1.712676 3.494296
H 3.108890 0.393329 1.742591
H 1.889332 2.294640 1.255691
C 2.847165 3.009052 -0.511763
C 2.969453 4.297610 0.018428
C 3.169398 2.792102 -1.855718
C 3.438914 5.346673 -0.770137
H 2.695417 4.479323 1.060724
C 3.637397 3.841937 -2.646352
H 3.051246 1.801442 -2.302277
C 3.780864 5.118991 -2.103655
H 3.531471 6.346950 -0.343813
H 3.886293 3.660555 -3.693231

H 4.146352 5.939321 -2.723597
C -0.365432 2.547037 0.164028
C -1.701234 2.931679 -0.016364
C -2.086341 4.367863 0.206761
O -0.619429 0.562969 -1.084534
C 0.073504 1.342087 -0.437369
H 4.729504 0.739637 -0.827952
C 4.594805 -2.622014 -1.176225
H 5.515021 -2.177276 -0.799424
H 5.509258 -4.495034 -1.681702
H -2.241990 2.403888 -0.805281
S -3.168542 2.046796 1.578471
C -2.771470 0.342030 1.397907
N -3.500500 -0.578846 0.873382
N -1.504592 0.030542 1.866853
C -4.788094 -0.385540 0.418500
C -5.819911 0.112682 1.242738
C -5.105525 -0.782593 -0.896593
C -7.118070 0.215962 0.768037
H -5.586139 0.411311 2.264583
C -6.398651 -0.671213 -1.383981
H -4.306207 -1.172852 -1.527735
C -7.392586 -0.173493 -0.542873
H -7.921120 0.595504 1.398414
H -6.648230 -0.965859 -2.402334
N -8.759953 -0.060702 -1.047194
O -8.976366 -0.407748 -2.187977
O -9.608706 0.374198 -0.299562
C -0.740429 -1.108315 1.702737
C -1.216226 -2.331896 1.187616
C 0.619834 -1.013824 2.077238
C -0.341712 -3.394909 1.003555
H -2.262672 -2.428356 0.918333
C 1.491792 -2.070900 1.890508
H 0.984933 -0.074771 2.500836
C 1.002543 -3.253841 1.334335
H -0.697055 -4.336095 0.585618
H 2.545973 -1.989229 2.156776
N 1.928934 -4.350043 1.062300
O 3.103116 -4.173873 1.306674
O 1.477665 -5.377203 0.605600
H 0.288596 3.187343 0.750071
H -1.014557 0.833834 2.247982
H -1.671298 4.748099 1.150986
H -1.669176 4.973194 -0.614753
H -3.175134 4.504900 0.214339

Me_Michael_Acceptor/TS-MA-S
Frequencies, energies and thermodynamic properties:
Lowest Vibrational Mode (1/cm) = -224.1195
2nd Lowest Vibrational Mode (1/cm) = 10.0597
E(RM062X) (a.u.) = -2890.52036486
Thermal correction to Enthalpy (a.u.) = 0.700564
Thermal correction to Gibbs Free Energy (a.u.) = 0.580812
Total Entropy (cal/Kmol) = 252.040
Esp(RM062X) (a.u.) = -2893.12581797
Optimised cartesian coordinates (Angstrom):
C 6.274168 0.857810 -0.724597
C 6.461744 -0.428342 -0.202061
C 5.459724 -1.051640 0.537058
C 4.260695 -0.365455 0.723249
C 4.082511 0.920039 0.209003
H 7.400401 -0.953572 -0.381536

H	5.599775	-2.055660	0.941290
C	2.006415	0.561251	1.145596
C	0.163165	2.155638	1.066615
C	1.296387	3.198484	1.153751
C	2.426440	2.805146	0.218069
H	3.309606	3.433983	0.394924
S	2.827864	-0.919190	1.575567
N	2.810843	1.423084	0.502414
N	0.719809	0.822239	1.387526
C	0.834706	4.660336	0.950139
C	0.895663	5.150468	-0.499609
C	1.655757	5.589952	1.844843
H	-0.213277	4.713186	1.287274
H	0.413125	6.135671	-0.576810
H	1.939730	5.273789	-0.828778
H	0.392022	4.475131	-1.205585
H	1.349576	6.636746	1.703369
H	1.530752	5.338191	2.908045
H	2.728531	5.520534	1.602973
H	1.683738	3.116555	2.182614
H	-0.546395	2.379524	1.873148
C	-0.576966	2.195365	-0.253369
C	-1.731629	2.979566	-0.360160
C	-0.083522	1.553264	-1.393437
C	-2.355933	3.156741	-1.594415
H	-2.138437	3.462778	0.531780
C	-0.716774	1.716208	-2.626762
H	0.802857	0.918112	-1.324679
C	-1.846720	2.528430	-2.732353
H	-3.248438	3.780979	-1.665921
H	-0.323860	1.206330	-3.508301
H	-2.338159	2.659686	-3.697739
C	-1.492047	0.000126	2.074978
H	-1.968852	0.724290	1.415546
C	-2.289610	-0.857273	2.877590
C	-3.715843	-0.441227	3.137265
O	0.523753	-1.116178	2.593242
C	-0.099602	-0.190211	2.075061
H	2.143617	2.892171	-0.841227
C	5.085199	1.553779	-0.524382
H	4.939693	2.550796	-0.940210
H	7.070194	1.323312	-1.307019
H	-1.760608	-1.283268	3.734198
S	-2.604924	-2.898360	2.037460
C	-1.705793	-2.646915	0.544100
N	-2.084315	-1.941251	-0.458705
N	-0.450375	-3.224644	0.552524
H	-0.266456	-3.766784	1.390447
C	-3.359545	-1.421498	-0.592321
C	-4.519876	-2.221058	-0.519414
C	-3.498138	-0.055948	-0.901474
C	-5.773910	-1.666390	-0.723933
H	-4.422761	-3.283502	-0.299024
C	-4.748662	0.511726	-1.094459
H	-2.594991	0.547476	-0.978687
C	-5.875276	-0.302865	-0.999951
H	-6.675281	-2.275369	-0.670727
H	-4.859782	1.571306	-1.321894
N	-7.197949	0.286085	-1.206163
O	-7.261203	1.473134	-1.442155
O	-8.163361	-0.441647	-1.129873
C	0.657194	-2.974528	-0.234567
C	0.744350	-1.922923	-1.169311

C 1.785620 -3.798114 -0.024422
C 1.951422 -1.662519 -1.805497
H -0.127717 -1.305970 -1.365649
C 2.979127 -3.554970 -0.678902
H 1.712152 -4.624130 0.685887
C 3.056823 -2.469526 -1.551898
H 2.044854 -0.827753 -2.500729
H 3.853789 -4.181471 -0.508557
N 4.336187 -2.146464 -2.176826
O 5.252075 -2.926650 -2.030184
O 4.420289 -1.110248 -2.798721
H -4.286060 -1.221200 3.658187
H -4.229886 -0.203200 2.194137
H -3.715903 0.463955 3.766806

Me_Michael_Acceptor/TS-turn-R_rot

Frequencies, energies and thermodynamic properties:

Lowest Vibrational Mode (1/cm) = -183.4658

2nd Lowest Vibrational Mode (1/cm) = 10.5148

E(RM062X) (a.u.) = -2890.51652502

Thermal correction to Enthalpy (a.u.) = 0.700641

Thermal correction to Gibbs Free Energy (a.u.) = 0.580451

Total Entropy (cal/Kmol) = 252.961

Esp(RM062X) (a.u.) = -2893.12190628

Optimised cartesian coordinates (Angstrom):

C 0.347980 -0.991013 -2.896015
C -1.029327 -0.939626 -3.534996
H -1.162584 -1.833559 -4.161157
H 0.455486 -1.945940 -2.368482
H 1.128911 -0.937662 -3.667341
N -0.539030 0.695705 -1.190781
C -0.330311 1.928629 -0.489658
C -0.388996 1.977304 0.907448
C -0.075578 3.097723 -1.218155
C -0.166043 3.174475 1.578589
H -0.629999 1.079495 1.470320
C 0.149495 4.300431 -0.558290
H -0.046511 3.054570 -2.303948
C 0.107105 4.314617 0.831625
H -0.205119 3.226691 2.665504
H 0.350777 5.218257 -1.108149
N 0.348082 5.583887 1.536303
O 0.582572 6.564671 0.868631
O 0.299832 5.576405 2.744821
C 0.623452 0.233886 -2.010819
O 1.335044 1.128301 -2.477159
C -1.309146 0.308789 -4.358841
S -2.254275 -1.191767 -2.202672
C -1.773504 0.094924 -1.061241
N -2.586255 0.453053 -0.141080
C -3.912784 0.066591 -0.062949
C -4.858586 0.489049 -1.020514
C -4.350472 -0.693651 1.037937
C -6.195759 0.148347 -0.890419
H -4.526697 1.094974 -1.864467
C -5.686182 -1.044231 1.171900
H -3.620342 -0.996291 1.788745
C -6.592442 -0.619970 0.203285
H -6.934406 0.470520 -1.623059
H -6.033675 -1.636718 2.016981
N -8.003485 -0.985097 0.341724
O -8.325253 -1.653903 1.298727
O -8.774861 -0.600894 -0.508960

N 1.609331 -0.588517 -0.678830
C 2.891862 -0.430785 -0.661808
C 1.013980 -1.658480 0.116462
S 3.752839 0.893941 -1.474593
N 3.761697 -1.240394 0.007608
C 1.957745 -2.879914 0.134806
H 0.093800 -1.966066 -0.405011
C 0.592564 -1.184113 1.493241
C 5.273419 0.321366 -0.795428
C 5.093839 -0.832762 -0.024311
C 3.320601 -2.463352 0.672881
C 1.381098 -4.127110 0.841325
H 2.094579 -3.139268 -0.929049
C -0.665715 -1.550833 1.981944
C 1.434096 -0.409255 2.301530
C 6.537699 0.877695 -0.962486
C 6.173854 -1.447180 0.608557
H 4.064844 -3.246982 0.470320
H 3.294978 -2.291094 1.760150
C 1.657394 -4.198883 2.345664
C 1.890661 -5.399296 0.162260
H 0.287650 -4.086662 0.699475
C -1.073584 -1.163828 3.259208
H -1.336440 -2.137983 1.348415
C 1.028059 -0.021268 3.577821
H 2.407637 -0.078380 1.932899
C 7.622689 0.265002 -0.333324
H 6.675067 1.774842 -1.568433
C 7.439279 -0.882877 0.443094
H 6.035281 -2.340035 1.218329
H 1.375948 -3.279707 2.878616
H 1.087462 -5.029939 2.786533
H 2.723120 -4.396198 2.542478
H 1.603043 -5.432774 -0.898855
H 2.989594 -5.459080 0.218193
H 1.483671 -6.295154 0.653952
C -0.226355 -0.398208 4.060980
H -2.058805 -1.455999 3.628034
H 1.690997 0.588350 4.194360
H 8.620551 0.689983 -0.448256
H 8.296455 -1.348462 0.931516
H -0.545224 -0.089957 5.058096
H -0.549831 0.405477 -5.149581
H -1.271734 1.217329 -3.740865
H -2.299088 0.248382 -4.830945

TS-turn-R

Frequencies, energies and thermodynamic properties:

Lowest Vibrational Mode (1/cm) = -136.0175

2nd Lowest Vibrational Mode (1/cm) = 9.5511

E(RM062X) (a.u.) = -2890.51995560

Thermal correction to Enthalpy (a.u.) = 0.700836

Thermal correction to Gibbs Free Energy (a.u.) = 0.578777

Total Entropy (cal/Kmol) = 256.895

Esp(RM062X) (a.u.) = -2893.12619392

Optimised cartesian coordinates (Angstrom):

C 1.617310 -2.792974 -1.896581
C 2.319574 -0.215232 -0.790204
C 0.035236 -0.937508 -1.534120
C 0.339668 -2.385960 -1.192962
H 0.429394 -2.549533 -0.109753
H -0.497833 -2.983963 -1.580952
N 0.955807 0.006177 -0.814101

C	0.471066	1.296902	-0.465834
C	-0.410735	1.984461	-1.310277
C	0.814055	1.850431	0.779471
C	-0.976780	3.188346	-0.900625
H	-0.660300	1.555199	-2.278669
C	0.266081	3.056123	1.189313
H	1.505229	1.321068	1.433625
C	-0.635968	3.700557	0.345544
H	-1.675004	3.726944	-1.539850
H	0.517773	3.490155	2.155908
N	-1.255699	4.952476	0.797055
O	-1.055123	5.300278	1.939680
O	-1.938528	5.563187	0.007961
O	-0.317979	-0.631840	-2.669146
S	3.011661	-1.806151	-1.229394
N	3.121432	0.720102	-0.451980
C	4.492930	0.579604	-0.328917
C	5.062405	-0.133283	0.745301
C	5.341488	1.221768	-1.250802
C	6.439732	-0.212658	0.888615
H	4.407797	-0.620917	1.469178
C	6.719607	1.142905	-1.116929
H	4.894744	1.781925	-2.072707
C	7.251171	0.425310	-0.047255
H	6.891885	-0.760668	1.714046
H	7.385521	1.630050	-1.827797
N	8.706035	0.342732	0.101012
O	9.392798	0.903059	-0.723768
O	9.145532	-0.282056	1.040631
H	1.531434	-2.551682	-2.965234
C	1.965451	-4.261295	-1.715237
N	-1.473826	-0.724207	-0.383918
C	-2.622818	-0.451251	-0.904331
C	-1.336712	-0.754323	1.070796
S	-2.966594	-0.446827	-2.650590
N	-3.744435	-0.140868	-0.192085
C	-2.271982	0.309036	1.689177
H	-0.304216	-0.448896	1.292579
C	-1.508015	-2.151978	1.638999
C	-4.662436	-0.107805	-2.319447
C	-4.902478	0.026350	-0.947297
C	-3.705202	0.036059	1.255931
C	-2.128568	0.509369	3.212200
H	-1.964208	1.254078	1.208376
C	-0.635758	-2.585707	2.644282
C	-2.528349	-3.010253	1.213412
C	-5.692675	0.036154	-3.243068
C	-6.184670	0.292605	-0.467825
H	-4.349624	0.889771	1.509493
H	-4.116851	-0.862098	1.743163
C	-2.983209	-0.433521	4.064496
C	-2.448132	1.959469	3.582396
H	-1.066731	0.324911	3.451897
C	-0.795725	-3.838089	3.235890
H	0.177535	-1.930121	2.966479
C	-2.689488	-4.264470	1.803614
H	-3.205582	-2.709456	0.410355
C	-6.976997	0.307782	-2.768416
H	-5.499513	-0.063349	-4.312441
C	-7.216551	0.432254	-1.396826
H	-6.379176	0.384514	0.600929
H	-2.863980	-1.491033	3.790680
H	-2.708944	-0.327846	5.124503

H -4.051140 -0.177093 3.979273
H -1.803901 2.673267 3.047503
H -3.494010 2.202542 3.334725
H -2.317400 2.125382 4.661986
C -1.828546 -4.679316 2.820207
H -0.108486 -4.160308 4.020155
H -3.490808 -4.922670 1.463155
H -7.798624 0.420845 -3.476699
H -8.226602 0.640300 -1.040981
H -1.955754 -5.661030 3.279576
H 2.922073 -4.510336 -2.197163
H 2.030775 -4.520016 -0.648309
H 1.180814 -4.877368 -2.178157

Me_Michael_Acceptor/TS-turn-S_rot

Frequencies, energies and thermodynamic properties:

Lowest Vibrational Mode (1/cm) = -141.4351
2nd Lowest Vibrational Mode (1/cm) = 9.5114
E(RM062X) (a.u.) = -2890.51856759
Thermal correction to Enthalpy (a.u.) = 0.700674
Thermal correction to Gibbs Free Energy (a.u.) = 0.579274
Total Entropy (cal/Kmol) = 255.508
Esp(RM062X) (a.u.) = -2893.12343797

Optimised cartesian coordinates (Angstrom):

C 1.587880 -2.948724 -1.713243
C 2.312075 -0.329523 -0.741546
C -0.004596 -1.027609 -1.457505
C 0.283004 -2.475533 -1.091682
H 0.323537 -2.612393 -0.003333
H -0.551689 -3.067575 -1.494390
N 0.952900 -0.085037 -0.772669
C 0.501845 1.230106 -0.470497
C -0.359851 1.914940 -1.337219
C 0.857855 1.812983 0.758044
C -0.894996 3.145104 -0.964695
H -0.618712 1.462527 -2.292365
C 0.341481 3.043910 1.130612
H 1.535864 1.286483 1.428711
C -0.543066 3.685536 0.265968
H -1.578118 3.682156 -1.621317
H 0.603852 3.500902 2.083636
N -1.130263 4.966224 0.678488
O -0.928357 5.338862 1.813010
O -1.789513 5.574518 -0.132274
O -0.353704 -0.735794 -2.603627
S 2.972688 -1.968634 -1.016131
N 3.130169 0.620253 -0.490467
C 4.496636 0.474929 -0.335773
C 5.044386 -0.182685 0.784504
C 5.365357 1.062591 -1.275602
C 6.418436 -0.261863 0.955315
H 4.375278 -0.627038 1.522918
C 6.740300 0.982550 -1.114753
H 4.935850 1.581298 -2.133100
C 7.249672 0.320179 0.000536
H 6.852961 -0.766887 1.816863
H 7.421091 1.427059 -1.839367
N 8.700962 0.237613 0.177929
O 9.405459 0.748784 -0.663754
O 9.120543 -0.338181 1.157213
H 1.799181 -3.958837 -1.334149
C 1.623066 -2.965137 -3.234753
N -1.468058 -0.754165 -0.337381

C -2.614190 -0.472170 -0.866884
C -1.332553 -0.754414 1.118857
S -2.963653 -0.509476 -2.610108
N -3.725018 -0.123382 -0.158808
C -2.241485 0.347312 1.708573
H -0.292774 -0.470464 1.333894
C -1.543584 -2.131900 1.722802
C -4.652825 -0.136108 -2.281287
C -4.884629 0.041412 -0.913382
C -3.680264 0.095089 1.283586
C -2.093676 0.584789 3.226182
H -1.913010 1.272054 1.202867
C -0.689322 -2.559618 2.745925
C -2.582567 -2.975406 1.313334
C -5.685206 -0.001760 -3.204308
C -6.159476 0.342581 -0.435193
H -4.304300 0.970662 1.511926
H -4.113030 -0.778606 1.796069
C -2.971355 -0.313056 4.103571
C -2.379398 2.051637 3.556088
H -1.036837 0.381842 3.472544
C -0.885618 -3.790359 3.370853
H 0.138902 -1.916819 3.055434
C -2.779706 -4.208179 1.936741
H -3.246229 -2.680903 0.496772
C -6.962334 0.303905 -2.731762
H -5.498980 -0.134816 -4.271266
C -7.193342 0.471867 -1.363147
H -6.346779 0.469004 0.631296
H -2.879934 -1.380328 3.858734
H -2.693062 -0.185841 5.160133
H -4.032375 -0.031366 4.012271
H -1.716161 2.734914 3.004960
H -3.418230 2.312894 3.297302
H -2.248585 2.243080 4.631403
C -1.936895 -4.616146 2.971014
H -0.211957 -4.108062 4.168606
H -3.595102 -4.855076 1.608327
H -7.785599 0.409791 -3.439244
H -8.198166 0.706607 -1.009197
H -2.092313 -5.581058 3.456549
H 0.812169 -3.607312 -3.611956
H 2.579522 -3.371893 -3.591271
H 1.482531 -1.959673 -3.650255

Me_Michael_Acceptor/TS-turn-S

Frequencies, energies and thermodynamic properties:

Lowest Vibrational Mode (1/cm) = -160.5560
2nd Lowest Vibrational Mode (1/cm) = 9.9181
E(RM062X) (a.u.) = -2890.51570127
Thermal correction to Enthalpy (a.u.) = 0.700510
Thermal correction to Gibbs Free Energy (a.u.) = 0.578922
Total Entropy (cal/Kmol) = 255.903
Esp(RM062X) (a.u.) = -2893.12208291

Optimised cartesian coordinates (Angstrom):

C -0.320730 -1.265136 2.765807
C 1.043855 -1.208276 3.418149
H 1.185311 -0.245543 3.931903
H -0.380504 -2.182520 2.164536
H -1.106413 -1.306983 3.533710
N 0.529745 0.606548 1.235429
C 0.293647 1.900088 0.666829
C 0.377750 2.107973 -0.715092

C	-0.013559	2.973685	1.513965
C	0.125795	3.364871	-1.254108
H	0.660335	1.288158	-1.370039
C	-0.268857	4.234152	0.986421
H	-0.055303	2.807706	2.586901
C	-0.202109	4.404972	-0.392175
H	0.183432	3.538988	-2.327447
H	-0.510121	5.078427	1.630087
N	-0.474665	5.735929	-0.956975
O	-0.748662	6.629823	-0.189641
O	-0.411318	5.863402	-2.158219
C	-0.648671	0.022884	1.987155
O	-1.357424	0.861050	2.570145
C	1.289869	-2.355825	4.384856
S	2.289277	-1.308816	2.086306
C	1.777154	0.054415	1.052911
N	2.583469	0.509892	0.169445
C	3.917743	0.163458	0.058364
C	4.854981	0.509134	1.054735
C	4.372840	-0.473106	-1.112452
C	6.199296	0.212533	0.894141
H	4.510831	1.021274	1.953940
C	5.715775	-0.779409	-1.277686
H	3.650105	-0.714244	-1.892456
C	6.612873	-0.434365	-0.269658
H	6.930652	0.476828	1.656642
H	6.076207	-1.276813	-2.176945
N	8.031399	-0.752554	-0.441156
O	8.367064	-1.318986	-1.457742
O	8.795295	-0.434376	0.442990
N	-1.584653	-0.636954	0.642216
C	-2.873030	-0.477552	0.638542
C	-0.979574	-1.603092	-0.272235
S	-3.743058	0.742181	1.585233
N	-3.725136	-1.211186	-0.126152
C	-1.913471	-2.822861	-0.416242
H	-0.053376	-1.953362	0.208334
C	-0.577988	-0.977344	-1.593042
C	-5.253548	0.245101	0.826137
C	-5.060693	-0.813223	-0.067625
C	-3.276329	-2.358657	-0.912032
C	-1.325799	-3.984317	-1.249398
H	-2.050136	-3.193033	0.614116
C	0.675773	-1.282169	-2.133044
C	-1.433511	-0.126656	-2.304207
C	-6.522554	0.775751	1.038405
C	-6.128257	-1.355297	-0.782314
H	-4.019616	-3.159764	-0.792886
H	-3.247817	-2.073943	-1.975092
C	-1.616572	-3.909565	-2.750776
C	-1.809724	-5.325417	-0.695453
H	-0.231594	-3.940979	-1.114224
C	1.066456	-0.757029	-3.365301
H	1.357419	-1.929005	-1.573901
C	-1.045267	0.398076	-3.536296
H	-2.404309	0.157109	-1.892384
C	-7.595649	0.235843	0.328392
H	-6.671802	1.597724	1.740505
C	-7.397411	-0.815863	-0.571605
H	-5.977684	-2.171486	-1.489078
H	-1.351896	-2.938049	-3.191571
H	-1.040593	-4.684671	-3.277280
H	-2.681659	-4.100954	-2.956811

H -1.509648 -5.459538 0.354173
H -2.908294 -5.396683 -0.746152
H -1.395314 -6.161442 -1.277867
C 0.205427 0.084115 -4.070982
H 2.049057 -1.000697 -3.773780
H -1.719408 1.065402 -4.076114
H -8.596750 0.642647 0.476323
H -8.246175 -1.223778 -1.122233
H 0.510898 0.499704 -5.032679
H 0.566153 -2.294929 5.210229
H 1.160144 -3.326585 3.883308
H 2.302038 -2.313173 4.811182

----

atropisomerism/CF3_S_Ts_iPr_B

Frequencies, energies and thermodynamic properties:

Lowest Vibrational Mode (1/cm) = 14.5004

2nd Lowest Vibrational Mode (1/cm) = 28.5553

E(RM062X) (a.u.) = -2202.77184649

Thermal correction to Enthalpy (a.u.) = 0.398532

Thermal correction to Gibbs Free Energy (a.u.) = 0.310903

Total Entropy (cal/Kmol) = 184.432

Esp(RM062X) (a.u.) = -2204.69678720

Optimised cartesian coordinates (Angstrom):

N 0.788958 -0.815802 -0.245034
C 0.104007 0.173525 0.201473
S 0.517234 1.856735 -0.185593
C -0.755331 2.798089 0.703630
C -1.166524 2.129635 1.998751
N -1.011003 -0.134419 0.966627
C -1.459587 -1.511750 0.938296
C -2.064008 -2.023864 -0.215915
C -2.494406 -3.357240 -0.170802
C -2.329165 -4.140601 0.968015
C -1.715793 -3.606166 2.102171
C -1.276386 -2.287305 2.082406
C -1.722838 0.739678 1.813699
O -2.703687 0.359276 2.393076
H -2.969691 -3.788742 -1.054871
H -1.577257 -4.214148 2.997201
H -0.791364 -1.844792 2.954034
H -1.939650 2.717723 2.508490
H -0.290362 2.067407 2.661854
H -0.285926 3.769234 0.917063
C -1.939900 3.091549 -0.204524
F -2.588800 1.972009 -0.549339
F -2.815140 3.882753 0.413786
F -1.562110 3.698126 -1.323799
H -2.677342 -5.174858 0.969702
C -2.244259 -1.207287 -1.482472
H -1.825108 -0.206473 -1.313799
C -3.728117 -1.016379 -1.806818
H -4.215789 -1.981305 -2.015619
H -4.258008 -0.540240 -0.968397
H -3.845737 -0.378519 -2.695695
C -1.484554 -1.836544 -2.653193
H -1.551927 -1.191967 -3.542338
H -0.421451 -1.977567 -2.408361
H -1.906459 -2.819138 -2.915836
S 2.061814 -0.719644 -1.315920
O 2.250382 -2.076093 -1.796056
O 1.852838 0.355219 -2.278755
C 3.429658 -0.296134 -0.266477
C 3.904624 -1.259811 0.624721

C 3.999138 0.970834 -0.365597

C 4.975037 -0.930137 1.450893

H 3.443915 -2.248498 0.663756

C 5.075728 1.283297 0.464859

H 3.609558 1.689841 -1.087887

C 5.557354 0.337929 1.370056

H 5.362098 -1.667176 2.155838

H 5.540565 2.267992 0.399678

H 6.399600 0.588194 2.017246

----

atropisomerism/CF3_S_Ts_iPr_F

Frequencies, energies and thermodynamic properties:

Lowest Vibrational Mode (1/cm) = 25.1148

2nd Lowest Vibrational Mode (1/cm) = 26.1756

E(RM062X) (a.u.) = -2202.77047290

Thermal correction to Enthalpy (a.u.) = 0.398604

Thermal correction to Gibbs Free Energy (a.u.) = 0.311726

Total Entropy (cal/Kmol) = 182.849

Esp(RM062X) (a.u.) = -2204.69687963

Optimised cartesian coordinates (Angstrom):

N 0.616682 -0.527506 -0.889683

C -0.122289 0.332815 -0.291855

S 0.296739 2.056504 -0.211080

C -1.011897 2.715234 0.861711

C -1.419641 1.722757 1.932224

N -1.299225 -0.134849 0.277422

C -1.847217 -1.372910 -0.241354

C -1.395436 -2.615748 0.214101

C -2.009974 -3.752864 -0.328370

C -3.022341 -3.654120 -1.279367

C -3.449765 -2.401288 -1.719867

C -2.857348 -1.256931 -1.196182

C -1.965786 0.427387 1.381015

O -2.907809 -0.134328 1.872778

H -1.682606 -4.740697 0.004077

H -4.240021 -2.313996 -2.466731

H -3.171271 -0.262255 -1.516882

H -2.196502 2.146266 2.580885

H -0.543396 1.493079 2.557540

H -0.564636 3.604510 1.328799

C -2.189194 3.218600 0.040584

F -2.808798 2.226101 -0.606745

F -3.090516 3.800787 0.830882

F -1.805526 4.111417 -0.865571

H -3.478159 -4.560637 -1.681260

C -0.289989 -2.761972 1.241632

H 0.079323 -1.754722 1.486701

C 0.881921 -3.561028 0.664901

H 0.584395 -4.599705 0.452977

H 1.238531 -3.107285 -0.272086

H 1.714126 -3.595883 1.384787

C -0.821054 -3.395698 2.530310

H -0.024517 -3.454402 3.287323

H -1.652943 -2.809140 2.946796

H -1.184979 -4.418124 2.344536

S 2.044336 -0.189002 -1.683899

O 2.413255 -1.430890 -2.335410

O 1.911955 1.030450 -2.471929

C 3.194420 0.115464 -0.359639

C 3.574158 -0.951619 0.455857

C 3.708782 1.398249 -0.187668

C 4.478673 -0.713883 1.487212

H 3.171401 -1.950515 0.281038

C 4.621062 1.619149 0.844314
H 3.402308 2.202809 -0.857510
C 4.999136 0.568604 1.680083
H 4.786301 -1.534309 2.137094
H 5.038594 2.616096 0.991936
H 5.711678 0.747562 2.487014

----

atropisomerism/CF3_S_Ts_iPr-TS_O_rot

Frequencies, energies and thermodynamic properties:

Lowest Vibrational Mode (1/cm) = -24.4501
2nd Lowest Vibrational Mode (1/cm) = 14.0916
E(RM062X) (a.u.) = -2202.72742026
Thermal correction to Enthalpy (a.u.) = 0.397352
Thermal correction to Gibbs Free Energy (a.u.) = 0.312409
Total Entropy (cal/Kmol) = 178.777
Esp(RM062X) (a.u.) = -2204.65205304

Optimised cartesian coordinates (Angstrom):

N 1.071035 -1.213602 -0.200145
C 0.414243 -0.123547 -0.206545
S 0.969833 1.394352 -0.952538
C -0.147572 2.542540 -0.090581
C -0.383045 2.112849 1.346313
N -0.872329 -0.130381 0.394543
C -1.850186 -1.042616 -0.167907
C -3.080329 -1.446396 0.415817
C -3.874711 -2.339329 -0.326220
C -3.545239 -2.802105 -1.592189
C -2.377253 -2.335920 -2.183707
C -1.557792 -1.466910 -1.480569
C -0.925434 0.708698 1.543152
O -1.318108 0.358414 2.618908
H -4.809830 -2.679966 0.115186
H -2.094062 -2.635712 -3.193573
H -0.664328 -1.112295 -1.989525
H -1.098364 2.786533 1.840860
H 0.561961 2.196483 1.903777
H 0.401760 3.494800 -0.084819
C -1.403932 2.831507 -0.901106
F -2.137130 1.752228 -1.168227
F -2.186133 3.690109 -0.242090
F -1.084014 3.389025 -2.068069
H -4.204834 -3.496728 -2.113918
C -3.661761 -0.908290 1.715899
H -2.864692 -0.899464 2.461714
C -4.797824 -1.743905 2.305035
H -5.714562 -1.697463 1.698429
H -4.512585 -2.799609 2.422577
H -5.048058 -1.350110 3.301060
C -4.145438 0.533400 1.503902
H -4.421781 0.996667 2.463326
H -3.391576 1.173136 1.021453
H -5.031130 0.539105 0.848852
S 2.547495 -1.412486 -0.996131
O 2.752590 -2.843696 -1.056363
O 2.542824 -0.623661 -2.223420
C 3.699007 -0.709246 0.152646
C 3.964169 -1.407213 1.332326
C 4.319642 0.499278 -0.156071
C 4.869054 -0.859340 2.236711
H 3.472978 -2.361066 1.532535
C 5.230047 1.029575 0.757683
H 4.099519 1.003773 -1.097754
C 5.498283 0.354902 1.948654

H 5.090577 -1.385269 3.166295
H 5.733503 1.971144 0.534394
H 6.210792 0.775750 2.660047
----
atropisomerism/CF3_S_Ts_iPr-TS
Frequencies, energies and thermodynamic properties:
Lowest Vibrational Mode (1/cm) = -31.4408
2nd Lowest Vibrational Mode (1/cm) = 20.9720
E(RM062X) (a.u.) = -2202.73182653
Thermal correction to Enthalpy (a.u.) = 0.397246
Thermal correction to Gibbs Free Energy (a.u.) = 0.313818
Total Entropy (cal/Kmol) = 175.587
Esp(RM062X) (a.u.) = -2204.65424962
Optimised cartesian coordinates (Angstrom):
N 0.616048 -1.339076 0.617215
C -0.014400 -0.370224 0.106810
S 0.410181 0.527505 -1.389612
C 0.564933 2.130978 -0.491592
C 0.643879 1.959171 1.029235
N -1.068166 0.215370 0.862803
C -2.462803 -0.098013 0.654281
C -2.923594 -1.265607 -0.010560
C -4.312640 -1.418860 -0.145836
C -5.235468 -0.493520 0.324867
C -4.766396 0.656621 0.947654
C -3.400190 0.850516 1.100532
C -0.610404 1.314872 1.613792
O -1.165274 1.746102 2.587572
H -4.686832 -2.310827 -0.646428
H -5.455557 1.418298 1.315496
H -3.063147 1.759894 1.585473
H 0.758309 2.937572 1.512589
H 1.536840 1.360428 1.271044
H 1.510471 2.561097 -0.851314
C -0.523915 3.100400 -0.914252
F -1.731548 2.724442 -0.485881
F -0.284581 4.313453 -0.413991
F -0.584778 3.213636 -2.238530
H -6.304344 -0.669256 0.196184
C -2.041230 -2.394354 -0.539707
H -1.145199 -1.962308 -0.998110
C -2.675692 -3.214283 -1.665344
H -3.499853 -3.848066 -1.307628
H -3.054368 -2.572863 -2.474485
H -1.912739 -3.883379 -2.089155
C -1.617773 -3.320531 0.607325
H -0.831251 -4.017064 0.277231
H -1.234415 -2.761197 1.470245
H -2.486209 -3.910280 0.941064
S 1.927869 -2.093051 -0.181089
O 2.181056 -3.285881 0.598118
O 1.625189 -2.195072 -1.601634
C 3.221005 -0.918085 0.099140
C 3.699595 -0.772851 1.403032
C 3.712392 -0.168959 -0.968096
C 4.690743 0.175568 1.641425
H 3.304104 -1.391133 2.210978
C 4.712087 0.768996 -0.712166
H 3.318453 -0.325790 -1.972664
C 5.191950 0.943233 0.586824
H 5.078918 0.310230 2.651765
H 5.118719 1.362535 -1.532048
H 5.972069 1.681417 0.779835

----

atropisomerism/H_Se_Bz_iPr_B

Frequencies, energies and thermodynamic properties:

Lowest Vibrational Mode (1/cm) = 16.2527

2nd Lowest Vibrational Mode (1/cm) = 29.5052

E(RM062X) (a.u.) = -3434.27237340

Thermal correction to Enthalpy (a.u.) = 0.386962

Thermal correction to Gibbs Free Energy (a.u.) = 0.308404

Total Entropy (cal/Kmol) = 165.339

Esp(RM062X) (a.u.) = -3435.71101244

Optimised cartesian coordinates (Angstrom):

N	-0.534700	-0.044986	0.805845
C	0.127546	0.919703	0.314362
C	0.854250	3.499358	-0.781044
C	1.504675	2.534115	-1.749830
N	1.466498	0.716651	-0.032915
C	2.136521	-0.400915	0.594535
C	2.081163	-1.681437	0.030417
C	2.778519	-2.702085	0.689974
C	3.491021	-2.460225	1.862561
C	3.522437	-1.177997	2.410482
C	2.841716	-0.146640	1.770329
C	2.169114	1.365477	-1.061987
O	3.266832	0.975701	-1.376283
H	2.760904	-3.712141	0.274268
H	4.075019	-0.980042	3.330043
H	2.851012	0.868092	2.172135
H	2.298086	3.031806	-2.322137
H	0.760401	2.146459	-2.464268
H	0.435916	4.368138	-1.303474
H	4.022256	-3.278332	2.352093
C	1.317725	-1.970588	-1.247540
H	0.736063	-1.069832	-1.498550
C	0.329410	-3.123769	-1.058835
H	0.856053	-4.075121	-0.886862
H	-0.334453	-2.939252	-0.201078
H	-0.292300	-3.248366	-1.958764
C	2.284462	-2.245883	-2.403335
H	1.731197	-2.406041	-3.341225
H	2.976753	-1.403529	-2.543801
H	2.880613	-3.150114	-2.203333
C	-2.890407	-0.612125	0.516124
C	-2.638342	-1.124186	-0.761565
C	-4.176876	-0.695640	1.061026
C	-3.669020	-1.718256	-1.488107
H	-1.633904	-1.058333	-1.183122
C	-5.202562	-1.295461	0.336311
H	-4.353807	-0.284801	2.056109
C	-4.948720	-1.806803	-0.938841
H	-3.472275	-2.116256	-2.484946
H	-6.204089	-1.365174	0.763810
H	-5.753631	-2.276650	-1.506991
H	1.556741	3.850553	-0.014566
Se	-0.666041	2.655092	0.091618
C	-1.822553	0.066584	1.320467
O	-2.056109	0.622399	2.369549

----

atropisomerism/H_Se_Bz_iPr_F

Frequencies, energies and thermodynamic properties:

Lowest Vibrational Mode (1/cm) = 19.6295

2nd Lowest Vibrational Mode (1/cm) = 25.5982

E(RM062X) (a.u.) = -3434.27774264

Thermal correction to Enthalpy (a.u.) = 0.386817

Thermal correction to Gibbs Free Energy (a.u.) = 0.308216

Total Entropy (cal/Kmol) = 165.430

Esp(RM062X) (a.u.) = -3435.71439007

Optimised cartesian coordinates (Angstrom):

N -0.551923 0.560023 -0.472564
C 0.691875 0.873116 -0.357999
C 3.170192 2.153009 0.367343
C 3.628679 1.309754 -0.801343
N 1.606476 -0.154321 -0.575094
C 1.052961 -1.490105 -0.664960
C 0.715366 -2.182805 0.503162
C 0.168716 -3.465466 0.352042
C -0.027168 -4.032255 -0.904424
C 0.321611 -3.320478 -2.053955
C 0.860815 -2.044830 -1.929748
C 2.998883 -0.058225 -0.794831
O 3.637556 -1.062798 -0.980089
H -0.110000 -4.030780 1.244666
H 0.171173 -3.755967 -3.042867
H 1.134801 -1.462442 -2.811081
H 4.712628 1.140487 -0.773329
H 3.382480 1.806087 -1.754200
H 3.688337 3.119814 0.388966
H -0.453780 -5.033328 -0.987937
C 0.894323 -1.586712 1.887635
H 1.371737 -0.601858 1.775273
C 1.820578 -2.450294 2.747607
H 1.383594 -3.444734 2.926778
H 2.797072 -2.589690 2.260530
H 1.986943 -1.976300 3.726560
C -0.461284 -1.373369 2.567545
H -0.329477 -0.886529 3.545554
H -1.117919 -0.740552 1.951588
H -0.972360 -2.334499 2.734717
C -2.937607 0.854689 -0.213945
C -3.129291 -0.519972 -0.403513
C -4.038460 1.686558 0.024891
C -4.415748 -1.054329 -0.351200
H -2.265389 -1.158956 -0.593084
C -5.321359 1.149424 0.075692
H -3.868913 2.754584 0.169363
C -5.510308 -0.222098 -0.112265
H -4.564625 -2.125133 -0.499402
H -6.178055 1.799015 0.262479
H -6.516627 -0.643301 -0.072702
H 3.333892 1.647614 1.328766
Se 1.286457 2.608135 0.181639
C -1.572995 1.468417 -0.253967
O -1.408255 2.667272 -0.110579

----

atropisomerism/H_Se_Bz_iPr-TS

Frequencies, energies and thermodynamic properties:

Lowest Vibrational Mode (1/cm) = -32.7217

2nd Lowest Vibrational Mode (1/cm) = 7.1759

E(RM062X) (a.u.) = -3434.23733501

Thermal correction to Enthalpy (a.u.) = 0.386033

Thermal correction to Gibbs Free Energy (a.u.) = 0.309916

Total Entropy (cal/Kmol) = 160.200

Esp(RM062X) (a.u.) = -3435.67374676

Optimised cartesian coordinates (Angstrom):

N 0.821989 0.322419 -0.395054
C -0.057741 -0.383827 0.170299
C -0.528458 -2.858273 1.285818

C -0.155436 -3.029298 -0.184138
N -1.187976 -0.829479 -0.574703
C -2.428325 -0.100854 -0.653507
C -2.601930 1.249593 -0.243788
C -3.885670 1.803567 -0.387108
C -4.980161 1.102251 -0.873672
C -4.806655 -0.232126 -1.222172
C -3.554329 -0.818031 -1.105982
C -1.017983 -2.142151 -1.068980
O -1.557949 -2.549983 -2.064267
H -4.029367 2.843285 -0.094176
H -5.642340 -0.832699 -1.585092
H -3.452986 -1.858859 -1.392275
H -0.345077 -4.058554 -0.515418
H 0.914452 -2.818568 -0.341478
H -0.007705 -3.593196 1.911265
H -5.951696 1.589698 -0.965030
C -1.521138 2.185083 0.298020
H -0.791029 1.607603 0.870660
C -2.043510 3.225509 1.293845
H -2.632644 4.017510 0.809155
H -2.660928 2.763899 2.078532
H -1.185390 3.713142 1.779492
C -0.796495 2.884360 -0.857551
H 0.039157 3.493542 -0.479191
H -0.398856 2.164879 -1.585521
H -1.496197 3.552852 -1.384464
C 3.238149 0.640303 -0.296968
C 3.375856 0.013563 -1.541526
C 4.371291 1.072236 0.403645
C 4.646414 -0.182746 -2.079017
H 2.487914 -0.308436 -2.087484
C 5.637323 0.874903 -0.137796
H 4.241079 1.558006 1.371848
C 5.774399 0.246956 -1.378652
H 4.756794 -0.668570 -3.049580
H 6.521712 1.209635 0.406414
H 6.768354 0.093468 -1.802863
H -1.610204 -2.958774 1.442943
Se 0.066230 -1.104872 1.956743
C 1.902586 0.848106 0.326794
O 1.729937 1.451293 1.362508
----
atropisomerism/H_Se_Ts_iPr_B
Frequencies, energies and thermodynamic properties:
Lowest Vibrational Mode (1/cm) = 20.2580
2nd Lowest Vibrational Mode (1/cm) = 28.6182
E(RM062X) (a.u.) = -3869.31272382
Thermal correction to Enthalpy (a.u.) = 0.388449
Thermal correction to Gibbs Free Energy (a.u.) = 0.307775
Total Entropy (cal/Kmol) = 169.793
Esp(RM062X) (a.u.) = -3870.99459938
Optimised cartesian coordinates (Angstrom):
N 0.118997 -0.305623 -0.912689
C -0.111628 0.820472 -0.338715
C 0.087572 3.412339 0.913444
C -0.590937 2.580370 1.979822
N -1.373748 0.976564 0.232723
C -2.446897 0.158862 -0.295544
C -2.677351 -1.135715 0.182761
C -3.760655 -1.835393 -0.364513
C -4.572419 -1.273730 -1.347540
C -4.316222 0.015415 -1.813791

C -3.248381 0.732318 -1.282057
C -1.672117 1.700615 1.406367
O -2.758966 1.589784 1.913642
H -3.970696 -2.847866 -0.011997
H -4.944202 0.462344 -2.585752
H -3.025352 1.745112 -1.622379
H -1.083918 3.219612 2.723242
H 0.143653 1.947957 2.504143
H 0.824495 4.098698 1.348113
H -5.407617 -1.847262 -1.753302
C -1.807938 -1.776724 1.246362
H -0.973737 -1.090648 1.456874
C -1.208493 -3.090720 0.739108
H -1.991556 -3.847702 0.577668
H -0.680989 -2.933379 -0.213847
H -0.499795 -3.499596 1.475637
C -2.594430 -1.978622 2.544334
H -1.946014 -2.400464 3.327190
H -3.003603 -1.024491 2.906720
H -3.433625 -2.674681 2.389439
S 1.541782 -0.695553 -1.682815
O 1.269297 -1.932379 -2.389693
O 2.064402 0.453665 -2.417676
C 2.651654 -1.049118 -0.336166
C 2.420701 -2.186676 0.438878
C 3.733710 -0.203610 -0.103818
C 3.288889 -2.465822 1.490771
H 1.578071 -2.843196 0.216709
C 4.600224 -0.500632 0.948658
H 3.893936 0.664714 -0.744155
C 4.374582 -1.623199 1.744644
H 3.123152 -3.349031 2.109328
H 5.455374 0.147907 1.143942
H 5.054274 -1.849411 2.567873
H -0.633018 3.993426 0.323462
Se 1.121732 2.284385 -0.288892
----
atropisomerism/H_Se_Ts_iPr_F
Frequencies, energies and thermodynamic properties:
Lowest Vibrational Mode (1/cm) = 18.0506
2nd Lowest Vibrational Mode (1/cm) = 32.4274
E(RM062X) (a.u.) = -3869.31341149
Thermal correction to Enthalpy (a.u.) = 0.388678
Thermal correction to Gibbs Free Energy (a.u.) = 0.307761
Total Entropy (cal/Kmol) = 170.306
Esp(RM062X) (a.u.) = -3870.99411460
Optimised cartesian coordinates (Angstrom):
N 0.286715 -0.624536 -0.235902
C -0.104390 0.565326 0.056860
C -0.369517 3.448036 0.354223
C -0.727826 2.932093 1.729990
N -1.245692 0.652186 0.848881
C -2.059742 -0.541884 0.938389
C -2.827985 -0.945004 -0.160919
C -3.591817 -2.111602 -0.013640
C -3.593835 -2.839022 1.172960
C -2.817901 -2.415754 2.253476
C -2.045584 -1.266541 2.130040
C -1.647460 1.741776 1.655109
O -2.682371 1.680489 2.266358
H -4.196642 -2.457095 -0.855432
H -2.809436 -2.981962 3.185894
H -1.423760 -0.918436 2.956485

H -1.265898 3.696181 2.304638
H 0.180497 2.649702 2.285746
H 0.258166 4.345652 0.414451
H -4.200284 -3.742733 1.254055
C -2.832976 -0.193358 -1.480093
H -2.211229 0.706939 -1.365531
C -4.243314 0.274368 -1.848951
H -4.916953 -0.581307 -2.009381
H -4.674003 0.902985 -1.055530
H -4.219883 0.860848 -2.779633
C -2.221656 -1.048331 -2.594761
H -2.142890 -0.466804 -3.525592
H -1.217944 -1.405794 -2.322449
H -2.849999 -1.929504 -2.798922
S 1.540103 -1.001333 -1.257360
O 1.294562 -2.367419 -1.683577
O 1.731466 0.032715 -2.271909
C 2.938499 -0.988454 -0.163620
C 3.065479 -2.022995 0.764772
C 3.867411 0.045205 -0.256849
C 4.153783 -2.007641 1.632337
H 2.325249 -2.824254 0.800142
C 4.955008 0.043613 0.617107
H 3.742629 0.825991 -1.008427
C 5.094124 -0.976285 1.558107
H 4.272832 -2.806395 2.365731
H 5.697119 0.841007 0.557737
H 5.947017 -0.972229 2.238922
H -1.261852 3.680480 -0.241270
Se 0.716100 2.161087 -0.621439

----

atropisomerism/H_Se_Ts_iPr-TS

Frequencies, energies and thermodynamic properties:

Lowest Vibrational Mode (1/cm) = -32.1300

2nd Lowest Vibrational Mode (1/cm) = 22.7746

E(RM062X) (a.u.) = -3869.27506226

Thermal correction to Enthalpy (a.u.) = 0.387447

Thermal correction to Gibbs Free Energy (a.u.) = 0.309747

Total Entropy (cal/Kmol) = 163.532

Esp(RM062X) (a.u.) = -3870.95334510

Optimised cartesian coordinates (Angstrom):

N 0.420884 0.640289 -0.809963
C -0.143930 -0.185044 -0.029953
C 0.093068 -2.573276 1.312460
C 0.358298 -2.819760 -0.170789
N -1.204408 -0.968198 -0.572507
C -2.592994 -0.598818 -0.459532
C -3.061731 0.698699 -0.115051
C -4.453445 0.878443 -0.044437
C -5.375272 -0.135358 -0.268606
C -4.900383 -1.408803 -0.558985
C -3.533235 -1.631637 -0.643140
C -0.772827 -2.244538 -1.006998
O -1.305852 -2.850565 -1.897616
H -4.829631 1.870124 0.204228
H -5.585862 -2.242671 -0.718399
H -3.196447 -2.636582 -0.874667
H 0.382926 -3.895144 -0.390188
H 1.329783 -2.393852 -0.471107
H 0.822645 -3.103312 1.936812
H -6.444920 0.068291 -0.203916
C -2.199261 1.934298 0.142753
H -1.281366 1.630134 0.653435

C -2.834887 2.951296 1.095123
H -3.674294 3.492541 0.635171
H -3.192187 2.475888 2.020475
H -2.077934 3.701210 1.367954
C -1.830737 2.616803 -1.179890
H -1.090498 3.414485 -1.013314
H -1.407499 1.908496 -1.903562
H -2.732315 3.066562 -1.625791
S 1.641679 1.688622 -0.262453
O 1.716257 2.726845 -1.270014
O 1.381474 2.044315 1.129599
C 3.083995 0.668204 -0.368154
C 3.528195 0.292178 -1.637677
C 3.735325 0.275895 0.799118
C 4.652659 -0.522651 -1.730748
H 3.002928 0.632438 -2.531920
C 4.867665 -0.530541 0.686226
H 3.362325 0.603575 1.769937
C 5.318314 -0.931163 -0.571664
H 5.016016 -0.833600 -2.711038
H 5.398566 -0.845112 1.585660
H 6.202525 -1.565609 -0.652098
H -0.918862 -2.884514 1.602602
Se 0.357764 -0.675178 1.764711
----
atropisomerism/H_S_Ts_biPh-B
Frequencies, energies and thermodynamic properties:
Lowest Vibrational Mode (1/cm) = 13.5691
2nd Lowest Vibrational Mode (1/cm) = 26.1489
E(RM062X) (a.u.) = -1979.08573915
Thermal correction to Enthalpy (a.u.) = 0.386982
Thermal correction to Gibbs Free Energy (a.u.) = 0.304869
Total Entropy (cal/Kmol) = 172.821
Esp(RM062X) (a.u.) = -1980.72922013
Optimised cartesian coordinates (Angstrom):
N 0.764693 -0.807187 -0.358412
C 0.258224 0.063893 0.444647
S 0.908375 1.683748 0.654345
C -0.369892 2.431190 1.699492
C -0.741244 1.496199 2.828956
N -0.890173 -0.318307 1.126611
C -1.642821 -1.424221 0.579809
C -2.670680 -1.135759 -0.328430
C -3.447831 -2.201107 -0.799457
C -3.191176 -3.507877 -0.389110
C -2.155803 -3.772427 0.508091
C -1.381658 -2.724342 0.999216
C -1.423479 0.262378 2.300884
O -2.389187 -0.230155 2.821242
H -4.256613 -1.994561 -1.503163
H -1.953259 -4.794976 0.829559
H -0.574667 -2.902533 1.711434
H -1.449220 1.969389 3.520570
H 0.153429 1.200129 3.399547
H 0.079154 3.360091 2.073094
H -3.803037 -4.325425 -0.773752
S 1.950061 -0.499866 -1.480709
O 2.112650 -1.745672 -2.205454
O 1.638245 0.720515 -2.218620
C 3.426486 -0.227468 -0.524815
C 3.945463 -1.293169 0.211862
C 4.044800 1.019168 -0.561157
C 5.109085 -1.089564 0.948470

H 3.445988 -2.263812 0.205044
C 5.215416 1.205637 0.174018
H 3.610549 1.823569 -1.156945
C 5.741577 0.156459 0.927667
H 5.529224 -1.907956 1.534818
H 5.717240 2.174069 0.155782
H 6.656472 0.308299 1.502830
H -1.238119 2.687083 1.073469
C -2.947518 0.269071 -0.745595
C -2.007465 1.002051 -1.485005
C -4.152212 0.882156 -0.378038
C -2.265069 2.326140 -1.837703
H -1.071768 0.533734 -1.797947
C -4.408803 2.206672 -0.734044
H -4.884430 0.317582 0.203443
C -3.464500 2.932883 -1.460455
H -1.522114 2.882512 -2.412418
H -5.349559 2.674169 -0.437696
H -3.664928 3.969695 -1.736415

----

atropisomerism/H_S_Ts_biPh-F

Frequencies, energies and thermodynamic properties:

Lowest Vibrational Mode (1/cm) = 17.7158

2nd Lowest Vibrational Mode (1/cm) = 20.3934

E(RM062X) (a.u.) = -1979.08504145

Thermal correction to Enthalpy (a.u.) = 0.387013

Thermal correction to Gibbs Free Energy (a.u.) = 0.305436

Total Entropy (cal/Kmol) = 171.693

Esp(RM062X) (a.u.) = -1980.72976950

Optimised cartesian coordinates (Angstrom):

N -0.085482 -0.555458 -1.051727
C 0.253533 -1.278723 -0.044133
S -0.798028 -2.506088 0.658116
C 0.271158 -3.221619 1.934587
C 1.032216 -2.141846 2.667820
N 1.527169 -1.059005 0.479395
C 2.452924 -0.307630 -0.342434
C 2.411443 1.093906 -0.389445
C 3.318283 1.740631 -1.240442
C 4.240040 1.023680 -1.999825
C 4.268958 -0.368007 -1.930521
C 3.366911 -1.031806 -1.103135
C 1.993434 -1.424815 1.759631
O 3.111443 -1.127198 2.094008
H 3.300592 2.830990 -1.292763
H 4.986460 -0.938104 -2.522159
H 3.357987 -2.121420 -1.042920
H 1.628706 -2.563152 3.486213
H 0.337894 -1.397415 3.091428
H -0.414380 -3.754520 2.605610
H 4.938927 1.556117 -2.646847
S -1.506428 -0.700144 -1.905967
O -1.365612 0.217199 -3.021190
O -1.817099 -2.100598 -2.166791
C -2.737188 -0.044039 -0.800056
C -2.721543 1.324590 -0.532752
C -3.689603 -0.895932 -0.246510
C -3.675604 1.846860 0.336292
H -1.973750 1.965045 -1.002375
C -4.647076 -0.357346 0.613007
H -3.677719 -1.958703 -0.492808
C -4.635035 1.006420 0.907013
H -3.675660 2.914382 0.562973

H -5.404645 -1.007034 1.053603
H -5.384214 1.421041 1.583642
H 0.944683 -3.955625 1.471263
C 1.429700 1.899151 0.386139
C 1.300077 1.766319 1.775700
C 0.634553 2.842492 -0.279806
C 0.383182 2.547480 2.479129
H 1.951169 1.077028 2.316085
C -0.272742 3.630955 0.426469
H 0.719516 2.941479 -1.364447
C -0.407040 3.480300 1.807169
H 0.298057 2.436268 3.561634
H -0.886216 4.359690 -0.107510
H -1.121243 4.093541 2.359713

----

atropisomerism/H_S_Ts_biPh-TS

Frequencies, energies and thermodynamic properties:

Lowest Vibrational Mode (1/cm) = -22.5131

2nd Lowest Vibrational Mode (1/cm) = 31.5509

E(RM062X) (a.u.) = -1979.05058148

Thermal correction to Enthalpy (a.u.) = 0.385365

Thermal correction to Gibbs Free Energy (a.u.) = 0.308268

Total Entropy (cal/Kmol) = 162.265

Esp(RM062X) (a.u.) = -1980.68994854

Optimised cartesian coordinates (Angstrom):

N 0.354791 0.497795 -0.565687
C -0.069605 -0.332640 0.292833
S 0.493578 -0.532111 1.968592
C 0.648192 -2.357312 1.847963
C 0.933029 -2.779120 0.409687
N -0.974508 -1.353127 -0.153137
C -2.398273 -1.206262 -0.240110
C -3.064444 0.039545 -0.219069
C -4.465291 0.059323 -0.313337
C -5.222732 -1.100144 -0.405743
C -4.562881 -2.326365 -0.380288
C -3.178283 -2.378069 -0.292242
C -0.307832 -2.564100 -0.440029
O -0.723234 -3.380464 -1.219368
H -4.957949 1.033689 -0.302322
H -5.124420 -3.260917 -0.425115
H -2.698950 -3.351698 -0.277991
H 1.168444 -3.849167 0.352465
H 1.792946 -2.220129 0.001660
H 1.490364 -2.609510 2.505336
H -6.309450 -1.047091 -0.481563
S 1.643547 1.572716 -0.244714
O 1.672986 2.467054 -1.383894
O 1.585789 2.107765 1.106591
C 2.996746 0.429693 -0.369017
C 3.307030 -0.091286 -1.626545
C 3.687198 0.059718 0.783103
C 4.332619 -1.027990 -1.722089
H 2.752595 0.231455 -2.509562
C 4.717936 -0.873626 0.668348
H 3.418019 0.500795 1.743053
C 5.033001 -1.418008 -0.577048
H 4.590225 -1.451579 -2.693721
H 5.276761 -1.173778 1.555896
H 5.837552 -2.150755 -0.659546
H -0.259483 -2.839405 2.236257
C -2.400351 1.367560 -0.062134
C -2.124830 2.157463 -1.182382

C -2.071174 1.842313 1.212493
C -1.481422 3.384104 -1.033719
H -2.383233 1.787808 -2.176775
C -1.426875 3.070245 1.359187
H -2.299932 1.233362 2.090043
C -1.122468 3.837972 0.236192
H -1.242300 3.981692 -1.914856
H -1.149126 3.421586 2.354284
H -0.598104 4.788367 0.349235
----
atropisomerism/H_S_Ts_iPr_alkene_B
Frequencies, energies and thermodynamic properties:
Lowest Vibrational Mode (1/cm) = 14.6793
2nd Lowest Vibrational Mode (1/cm) = 30.1239
E(RM062X) (a.u.) = -1864.86136113
Thermal correction to Enthalpy (a.u.) = 0.365591
Thermal correction to Gibbs Free Energy (a.u.) = 0.286735
Total Entropy (cal/Kmol) = 165.967
Esp(RM062X) (a.u.) = -1866.38702476
Optimised cartesian coordinates (Angstrom):
N -0.320206 -0.247210 0.522570
C 0.075162 0.908951 0.120685
S -0.796396 2.408259 0.515402
C 0.111111 3.602847 -0.355958
C 1.237324 3.393174 -1.042680
N 1.223508 0.951796 -0.640132
C 1.855175 -0.323501 -0.921273
C 2.651201 -0.946950 0.045068
C 3.230210 -2.174561 -0.305184
C 3.024263 -2.750116 -1.556022
C 2.221901 -2.106652 -2.499311
C 1.633422 -0.888824 -2.176173
C 1.881097 2.085191 -1.188395
O 2.930087 1.941337 -1.765873
H 3.854884 -2.691674 0.426790
H 2.053428 -2.551178 -3.481143
H 0.998728 -0.363277 -2.891824
H 3.490545 -3.707360 -1.795367
C 2.883454 -0.358425 1.424133
H 2.359489 0.607493 1.476495
C 4.371214 -0.086840 1.663740
H 4.950965 -1.022689 1.656253
H 4.784734 0.576659 0.890010
H 4.519260 0.391111 2.643663
C 2.301244 -1.267674 2.510622
H 2.419480 -0.802552 3.500963
H 1.231321 -1.456691 2.340181
H 2.824033 -2.236831 2.530489
S -1.661263 -0.545318 1.451152
O -1.452220 -1.865498 2.017313
O -1.944516 0.575245 2.343224
C -2.949572 -0.644632 0.233105
C -2.965164 -1.745182 -0.625706
C -3.901094 0.369531 0.160169
C -3.962947 -1.819023 -1.592880
H -2.208568 -2.526503 -0.533443
C -4.897795 0.277999 -0.812198
H -3.864790 1.205209 0.860547
C -4.925171 -0.809244 -1.685020
H -3.993725 -2.669710 -2.275104
H -5.656342 1.058768 -0.883835
H -5.706764 -0.874902 -2.443824
H 1.751634 4.218638 -1.534849

H -0.325888 4.601252 -0.265557

----

atropisomerism/H_S_Ts_iPr_alkene_F

Frequencies, energies and thermodynamic properties:

Lowest Vibrational Mode (1/cm) = 12.5367

2nd Lowest Vibrational Mode (1/cm) = 24.2657

E(RM062X) (a.u.) = -1864.86113145

Thermal correction to Enthalpy (a.u.) = 0.365329

Thermal correction to Gibbs Free Energy (a.u.) = 0.285883

Total Entropy (cal/Kmol) = 167.208

Esp(RM062X) (a.u.) = -1866.38793284

Optimised cartesian coordinates (Angstrom):

N 0.302318 -0.757978 0.859083

C -0.670709 -1.332824 0.248372

S -0.561990 -2.981820 -0.425347

C -2.132545 -3.190701 -1.131853

C -3.128730 -2.301076 -1.146428

N -1.840615 -0.610296 0.117856

C -1.840462 0.721935 0.690581

C -1.426035 1.812185 -0.081193

C -1.462808 3.073889 0.528236

C -1.889936 3.236218 1.843617

C -2.296318 2.129391 2.590325

C -2.270013 0.866530 2.008246

C -3.039878 -0.967017 -0.551292

O -3.943139 -0.168597 -0.612816

H -1.149750 3.949044 -0.046052

H -2.631764 2.248240 3.621482

H -2.579306 -0.018616 2.566744

H -1.906325 4.232561 2.288713

C -0.964988 1.665572 -1.519206

H -0.964241 0.593746 -1.769364

C 0.464817 2.182178 -1.697763

H 0.521321 3.266048 -1.511598

H 1.154924 1.680335 -1.003415

H 0.813058 1.999540 -2.725755

C -1.938128 2.363784 -2.473246

H -1.629480 2.210630 -3.518308

H -2.958363 1.972440 -2.350506

H -1.961900 3.448139 -2.283163

S 1.821893 -1.358223 1.128023

O 2.101298 -1.169513 2.539230

O 1.989330 -2.685117 0.539608

C 2.788023 -0.202711 0.191004

C 3.197679 0.980131 0.802931

C 3.076057 -0.493474 -1.141154

C 3.914194 1.905115 0.045824

H 2.956139 1.165700 1.850506

C 3.794240 0.441405 -1.884544

H 2.746434 -1.436619 -1.580279

C 4.207931 1.636608 -1.292711

H 4.245511 2.838316 0.503461

H 4.033211 0.234902 -2.928786

H 4.768785 2.365506 -1.880147

H -4.079925 -2.533314 -1.625423

H -2.242931 -4.175000 -1.596152

----

atropisomerism/H_S_Ts_iPr_alkene-TS

Frequencies, energies and thermodynamic properties:

Lowest Vibrational Mode (1/cm) = -36.1523

2nd Lowest Vibrational Mode (1/cm) = 16.8551

E(RM062X) (a.u.) = -1864.81305094

Thermal correction to Enthalpy (a.u.) = 0.364085

Thermal correction to Gibbs Free Energy (a.u.) = 0.287828

Total Entropy (cal/Kmol) = 160.498

Esp(RM062X) (a.u.) = -1866.33485304

Optimised cartesian coordinates (Angstrom):

N 0.451056 0.521159 -0.812184
C -0.139958 -0.203474 0.032562
S 0.382529 -0.513850 1.737918
C 0.730553 -2.194699 1.353592
C 0.329799 -2.850303 0.251370
N -1.190853 -1.045959 -0.399019
C -2.585535 -0.731558 -0.215981
C -3.069805 0.573541 0.067570
C -4.455693 0.724109 0.227721
C -5.356441 -0.329909 0.133875
C -4.863618 -1.604720 -0.116252
C -3.498286 -1.799219 -0.281606
C -0.733609 -2.370700 -0.660348
O -1.240198 -3.079005 -1.491941
H -4.845262 1.719611 0.437823
H -5.533731 -2.463348 -0.181612
H -3.146420 -2.806002 -0.482214
H -6.425048 -0.153497 0.263067
C -2.207016 1.829301 0.162096
H -1.275361 1.582669 0.685058
C -2.816277 2.950144 1.006923
H -3.680523 3.419906 0.515721
H -3.130997 2.590089 1.997303
H -2.059475 3.734582 1.153233
C -1.865018 2.355245 -1.237291
H -1.088524 3.134523 -1.183009
H -1.503345 1.560900 -1.902853
H -2.765860 2.796015 -1.692680
S 1.692230 1.616433 -0.360962
O 1.860754 2.454009 -1.528544
O 1.345620 2.211626 0.922369
C 3.088745 0.544894 -0.191285
C 3.590732 -0.066708 -1.341329
C 3.647573 0.347703 1.069330
C 4.681750 -0.921356 -1.210986
H 3.136691 0.122397 -2.315842
C 4.746808 -0.502371 1.178699
H 3.226579 0.852786 1.939416
C 5.255691 -1.136453 0.044428
H 5.089587 -1.415982 -2.093332
H 5.206900 -0.668082 2.153710
H 6.113514 -1.804447 0.137806
H 0.672089 -3.869455 0.069826
H 1.379851 -2.674247 2.092108

----

atropisomerism/H_S_Ts_iPr_B

Frequencies, energies and thermodynamic properties:

Lowest Vibrational Mode (1/cm) = 16.6340

2nd Lowest Vibrational Mode (1/cm) = 25.9087

E(RM062X) (a.u.) = -1866.08181850

Thermal correction to Enthalpy (a.u.) = 0.389342

Thermal correction to Gibbs Free Energy (a.u.) = 0.309368

Total Entropy (cal/Kmol) = 168.319

Esp(RM062X) (a.u.) = -1867.60936744

Optimised cartesian coordinates (Angstrom):

N 0.192360 0.226040 0.912359
C -0.064659 -0.942100 0.439005
S 1.085578 -2.275690 0.454098
C 0.123881 -3.566309 -0.381894

C -0.615645 -2.996976 -1.571902
N -1.339070 -1.133341 -0.090598
C -2.378974 -0.215129 0.326190
C -2.548741 1.023056 -0.303434
C -3.602832 1.828688 0.147225
C -4.444921 1.421834 1.179455
C -4.250125 0.185553 1.794974
C -3.211874 -0.634100 1.363060
C -1.672718 -2.012961 -1.143322
O -2.759237 -1.949248 -1.656345
H -3.764453 2.801290 -0.323421
H -4.902414 -0.140416 2.606291
H -3.036472 -1.608404 1.822693
H -1.132168 -3.785610 -2.133060
H 0.083540 -2.490907 -2.257319
H 0.866922 -4.313101 -0.689178
H -5.255782 2.075061 1.506443
C -1.644628 1.500592 -1.423061
H -0.862652 0.740934 -1.572297
C -0.951350 2.810661 -1.039123
H -1.682403 3.626496 -0.927635
H -0.410666 2.700279 -0.087066
H -0.237860 3.110936 -1.822011
C -2.423338 1.638511 -2.734092
H -1.750440 1.935716 -3.552610
H -2.905255 0.688729 -3.007842
H -3.207138 2.407025 -2.646696
S 1.618749 0.664536 1.646101
O 1.407343 2.030849 2.088214
O 2.036245 -0.345594 2.611033
C 2.800065 0.692768 0.311948
C 2.654681 1.653563 -0.689702
C 3.853378 -0.217789 0.315225
C 3.583680 1.683577 -1.726364
H 1.829440 2.366919 -0.654460
C 4.782466 -0.169555 -0.724238
H 3.938970 -0.946745 1.122383
C 4.644478 0.774063 -1.741994
H 3.485182 2.425160 -2.520442
H 5.616924 -0.872205 -0.736441
H 5.372519 0.805696 -2.554366
H -0.559812 -4.033016 0.340596

----

atropisomerism/H_S_Ts_iPr_F

Frequencies, energies and thermodynamic properties:

Lowest Vibrational Mode (1/cm) = 14.3572
2nd Lowest Vibrational Mode (1/cm) = 31.0530
E(RM062X) (a.u.) = -1866.08203469
Thermal correction to Enthalpy (a.u.) = 0.389514
Thermal correction to Gibbs Free Energy (a.u.) = 0.309311
Total Entropy (cal/Kmol) = 168.800
Esp(RM062X) (a.u.) = -1867.60824851

Optimised cartesian coordinates (Angstrom):

N -0.320262 -0.551453 0.059812
C 0.030411 0.683970 0.152465
S -0.855654 1.870907 1.107077
C 0.151479 3.359337 0.864953
C 0.628602 3.466389 -0.565441
N 1.199897 1.044361 -0.511244
C 2.061694 -0.036560 -0.939765
C 2.793441 -0.764225 0.007437
C 3.617757 -1.791173 -0.474251
C 3.709820 -2.078612 -1.832942

C 2.964912 -1.341871 -2.755242
C 2.135659 -0.322144 -2.303064
C 1.578222 2.348517 -0.904959
O 2.613271 2.523428 -1.491677
H 4.198117 -2.381693 0.238580
H 3.025956 -1.563514 -3.821626
H 1.539089 0.265752 -3.002377
H 1.176172 4.402436 -0.729269
H -0.224884 3.435386 -1.261579
H -0.513935 4.191015 1.129516
H 4.362060 -2.884474 -2.173824
C 2.696635 -0.506448 1.500630
H 2.029770 0.353490 1.661307
C 4.059400 -0.137604 2.092826
H 4.774197 -0.968462 1.991170
H 4.487212 0.742975 1.591116
H 3.960642 0.089602 3.164873
C 2.087695 -1.714073 2.220839
H 1.915627 -1.481588 3.282440
H 1.128955 -2.009412 1.771408
H 2.766018 -2.580159 2.170092
S -1.562297 -1.264207 0.904076
O -1.367710 -2.691893 0.721341
O -1.657475 -0.731149 2.258220
C -3.006108 -0.773649 -0.008699
C -3.201233 -1.311110 -1.282102
C -3.906264 0.119992 0.565727
C -4.327980 -0.924292 -2.002005
H -2.482672 -2.021125 -1.695457
C -5.034337 0.492927 -0.165547
H -3.724782 0.505325 1.570133
C -5.240603 -0.024587 -1.444129
H -4.498961 -1.330917 -2.999752
H -5.754434 1.188316 0.268019
H -6.124145 0.271305 -2.012242
H 0.989862 3.346411 1.575584

----

atropisomerism/H_S_Ts_iPr-TS

Frequencies, energies and thermodynamic properties:

Lowest Vibrational Mode (1/cm) = -34.9357

2nd Lowest Vibrational Mode (1/cm) = 19.6019

E(RM062X) (a.u.) = -1866.04239348

Thermal correction to Enthalpy (a.u.) = 0.388298

Thermal correction to Gibbs Free Energy (a.u.) = 0.311606

Total Entropy (cal/Kmol) = 161.412

Esp(RM062X) (a.u.) = -1867.56611539

Optimised cartesian coordinates (Angstrom):

N 0.444993 0.584961 -0.688493
C -0.100479 -0.227709 0.120093
S 0.403058 -0.601791 1.782934
C 0.181482 -2.413094 1.576710
C 0.423129 -2.829357 0.128061
N -1.162487 -1.036478 -0.382371
C -2.555806 -0.682409 -0.261193
C -3.039283 0.628241 0.000760
C -4.431496 0.789155 0.097038
C -5.338777 -0.254388 -0.030835
C -4.847952 -1.536906 -0.244315
C -3.479088 -1.741906 -0.347513
C -0.721004 -2.332882 -0.741171
O -1.250773 -3.000730 -1.587352
H -4.820398 1.789239 0.284710
H -5.521684 -2.391215 -0.326861

H -3.128601 -2.755183 -0.514910
H 0.439914 -3.922169 0.030089
H 1.391476 -2.444904 -0.232692
H 0.928499 -2.859910 2.245123
H -6.410137 -0.065211 0.048016
C -2.186844 1.888364 0.141133
H -1.265243 1.637711 0.674525
C -2.825700 2.985597 0.996608
H -3.675336 3.470493 0.494527
H -3.168853 2.599218 1.967625
H -2.074734 3.766221 1.187444
C -1.828362 2.446925 -1.241704
H -1.070953 3.241425 -1.156535
H -1.429345 1.671280 -1.907459
H -2.729716 2.873366 -1.710245
S 1.680057 1.641657 -0.193764
O 1.800422 2.598108 -1.275160
O 1.397060 2.105212 1.159909
C 3.101128 0.584883 -0.190367
C 3.582710 0.128767 -1.419275
C 3.696091 0.239843 1.021018
C 4.687212 -0.718471 -1.423958
H 3.101432 0.432675 -2.350640
C 4.809323 -0.599712 0.996616
H 3.292718 0.627546 1.956904
C 5.296657 -1.079841 -0.219315
H 5.079066 -1.091343 -2.371088
H 5.296854 -0.878215 1.931825
H 6.165553 -1.740007 -0.230714
H -0.819203 -2.709578 1.920164
----
atropisomerism/H_S_Ts_tBu-B
Frequencies, energies and thermodynamic properties:
Lowest Vibrational Mode (1/cm) = 19.7510
2nd Lowest Vibrational Mode (1/cm) = 24.2685
E(RM062X) (a.u.) = -1905.33910729
Thermal correction to Enthalpy (a.u.) = 0.419017
Thermal correction to Gibbs Free Energy (a.u.) = 0.337456
Total Entropy (cal/Kmol) = 171.659
Esp(RM062X) (a.u.) = -1906.90908701
Optimised cartesian coordinates (Angstrom):
N -0.466582 -0.572225 0.232313
C -0.096274 0.643689 0.024989
S -0.907034 2.039088 0.737895
C 0.029664 3.420552 0.023039
C 0.420872 3.137677 -1.409501
N 1.041534 0.821123 -0.753628
C 1.852260 -0.354419 -1.000029
C 2.830701 -0.799406 -0.093127
C 3.503709 -1.978610 -0.465441
C 3.244221 -2.656933 -1.651856
C 2.281623 -2.175853 -2.536714
C 1.587482 -1.023798 -2.197229
C 1.375121 1.976107 -1.492610
O 2.376010 1.993552 -2.160147
H 4.263890 -2.389436 0.196323
H 2.068640 -2.693417 -3.472954
H 0.814843 -0.622417 -2.855858
H 0.933200 3.999615 -1.853680
H -0.470107 2.910144 -2.016561
H -0.651930 4.278217 0.088271
H 3.802217 -3.565720 -1.883692
C 3.171463 -0.144243 1.262902

C 4.643778 -0.409107 1.627686
H 4.847509 -1.468848 1.829467
H 5.322615 -0.067874 0.830952
H 4.889501 0.143057 2.546146
C 2.289085 -0.772279 2.355965
H 2.560083 -0.354574 3.338516
H 1.221383 -0.575596 2.186386
H 2.429567 -1.863061 2.393967
S -1.702212 -1.038068 1.241647
O -1.550298 -2.472636 1.392233
O -1.748110 -0.197762 2.434467
C -3.156182 -0.729826 0.266652
C -3.395544 -1.550188 -0.837189
C -4.020776 0.300563 0.626678
C -4.529879 -1.314642 -1.608523
H -2.704786 -2.359395 -1.080771
C -5.157502 0.519803 -0.152264
H -3.806354 0.909055 1.506404
C -5.407142 -0.281920 -1.265828
H -4.735423 -1.942981 -2.476295
H -5.850368 1.318762 0.115344
H -6.297230 -0.104704 -1.871849
H 0.907222 3.627444 0.651280
C 2.984646 1.384380 1.281119
H 3.410802 1.857339 0.383086
H 1.932027 1.676916 1.379647
H 3.507212 1.794355 2.157664

----

atropisomerism/H_S_Ts_tBu-F

Frequencies, energies and thermodynamic properties:

Lowest Vibrational Mode (1/cm) = 15.3711
2nd Lowest Vibrational Mode (1/cm) = 33.2207
E(RM062X) (a.u.) = -1905.33867110
Thermal correction to Enthalpy (a.u.) = 0.418772
Thermal correction to Gibbs Free Energy (a.u.) = 0.338466
Total Entropy (cal/Kmol) = 169.018
Esp(RM062X) (a.u.) = -1906.91039091

Optimised cartesian coordinates (Angstrom):

N -0.263877 0.021854 -1.009601
C -0.017671 -1.071748 -0.375462
S -1.193469 -2.370353 -0.195399
C -0.240833 -3.587398 0.752163
C 0.559458 -2.911653 1.841813
N 1.248689 -1.188398 0.185808
C 2.290021 -0.331936 -0.349730
C 2.555596 0.966104 0.113979
C 3.603310 1.639671 -0.543324
C 4.346512 1.071967 -1.571370
C 4.065448 -0.224304 -1.998647
C 3.032773 -0.915177 -1.381565
C 1.621531 -2.013937 1.266617
O 2.741382 -1.957549 1.703856
H 3.842226 2.660109 -0.242526
H 4.637129 -0.689219 -2.802814
H 2.777154 -1.930326 -1.691007
H 1.075347 -3.647852 2.470330
H -0.098788 -2.305658 2.486106
H -0.994698 -4.267334 1.168954
H 5.145938 1.648041 -2.040347
C 1.804490 1.754778 1.205867
C 1.064193 2.910349 0.507565
H 1.768750 3.609764 0.033919
H 0.397027 2.519090 -0.275398

H 0.466738 3.475554 1.240595
C 2.825078 2.323007 2.209208
H 2.296440 2.893749 2.987642
H 3.381454 1.508963 2.699048
H 3.550353 3.000513 1.739609
S -1.694449 0.387926 -1.772186
O -1.450224 1.654945 -2.436974
O -2.182075 -0.746443 -2.547771
C -2.827708 0.679321 -0.427548
C -2.652647 1.817926 0.360695
C -3.872409 -0.215377 -0.209633
C -3.538067 2.047084 1.410587
H -1.839020 2.514328 0.151473
C -4.757778 0.031773 0.839808
H -3.987021 -1.084386 -0.858946
C -4.586920 1.154973 1.648756
H -3.415527 2.930188 2.039263
H -5.584377 -0.656236 1.022825
H -5.281076 1.342146 2.469755
H 0.400109 -4.157485 0.065504
C 0.783025 0.964514 2.036387
H -0.073691 0.618848 1.444861
H 1.247554 0.116191 2.559677
H 0.383918 1.638406 2.809501
----
atropisomerism/H_S_Ts_tBu-TS
Frequencies, energies and thermodynamic properties:
Lowest Vibrational Mode (1/cm) = -44.1563
2nd Lowest Vibrational Mode (1/cm) = 18.9238
E(RM062X) (a.u.) = -1905.28604529
Thermal correction to Enthalpy (a.u.) = 0.417610
Thermal correction to Gibbs Free Energy (a.u.) = 0.339841
Total Entropy (cal/Kmol) = 163.678
Esp(RM062X) (a.u.) = -1906.85245391
Optimised cartesian coordinates (Angstrom):
N -0.415334 -0.626310 -0.282984
C -0.042855 0.298537 0.505446
S -0.830124 0.736656 2.038956
C -0.391898 2.502890 1.976065
C -0.562260 3.013130 0.558129
N 1.013449 1.155853 0.091607
C 2.395022 0.834708 -0.210056
C 3.061529 -0.427529 -0.280832
C 4.396126 -0.385478 -0.738458
C 5.110090 0.770055 -1.013881
C 4.496954 1.992705 -0.771325
C 3.173952 2.004015 -0.368816
C 0.448499 2.389317 -0.386206
O 0.771222 2.896539 -1.421931
H 4.920360 -1.328369 -0.867462
H 5.032761 2.935736 -0.888307
H 2.720931 2.972666 -0.176143
H -0.396810 4.098272 0.499013
H -1.581546 2.819034 0.185723
H -1.088823 2.992226 2.668520
H 6.140536 0.707861 -1.366056
C 2.549808 -1.830671 0.140197
C 3.717654 -2.796135 0.446026
H 4.287442 -3.087843 -0.445902
H 4.410750 -2.379215 1.192284
H 3.285880 -3.718402 0.861799
C 1.787823 -2.490959 -1.026786
H 1.350992 -3.447488 -0.699770

H	0.983557	-1.870297	-1.427144
H	2.498154	-2.702069	-1.841169
S	-1.702768	-1.669139	0.055119
O	-1.563431	-2.758859	-0.890410
O	-1.769222	-1.956725	1.483987
C	-3.091019	-0.670548	-0.410624
C	-3.295134	-0.417603	-1.768404
C	-3.935919	-0.170662	0.577832
C	-4.375383	0.377667	-2.139225
H	-2.621366	-0.838691	-2.516544
C	-5.018967	0.616982	0.187737
H	-3.746677	-0.403108	1.626195
C	-5.232815	0.892454	-1.162963
H	-4.553336	0.591267	-3.193970
H	-5.698330	1.014061	0.943185
H	-6.080788	1.511353	-1.461003
H	0.632827	2.649788	2.342919
C	1.751956	-1.756100	1.472179
H	2.296369	-2.280689	2.269663
H	1.618796	-0.730704	1.839021
H	0.760264	-2.220760	1.400218
